# In situ profiling reveals metabolic alterations in the tumor microenvironment of ovarian cancer after chemotherapy

**DOI:** 10.1038/s41698-023-00454-0

**Published:** 2023-11-03

**Authors:** Sara Corvigno, Sunil Badal, Meredith L. Spradlin, Michael Keating, Igor Pereira, Elaine Stur, Emine Bayraktar, Katherine I. Foster, Nicholas W. Bateman, Waleed Barakat, Kathleen M. Darcy, Thomas P. Conrads, G. Larry Maxwell, Philip L. Lorenzi, Susan K. Lutgendorf, Yunfei Wen, Li Zhao, Premal H. Thaker, Michael J. Goodheart, Jinsong Liu, Nicole Fleming, Sanghoon Lee, Livia S. Eberlin, Anil K. Sood

**Affiliations:** 1 Department of Gynecologic Oncology and Reproductive Medicine, The University of Texas MD Anderson Cancer Center, Houston, TX USA; 2 Department of Chemistry, The University of Texas at Austin, Austin, TX USA; 3 Gynecologic Cancer Center of Excellence, Department of Gynecologic Surgery and Obstetrics, Uniformed Services University of the Health Sciences, Walter Reed National Military Medical Center, Bethesda, MD USA; 4 The Henry M. Jackson Foundation for the Advancement of Military Medicine, Inc., Bethesda, MD USA; 5 Women’s Health Integrated Research Center, Women’s Service Line, Inova Health System, Falls Church, VA USA; 6 Department of Bioinformatics and Computational Biology, The University of Texas MD Anderson Cancer Center, Houston, TX USA; 7 Departments of Psychological and Brain Sciences, Obstetrics and Gynecology, and Urology, University of Iowa, Iowa City, IA USA; 8 Department of Genomic Medicine, The University of Texas MD Anderson Cancer Center, Houston, TX USA; 9 Department of Obstetrics and Gynecology, Division of Gynecologic Oncology, Washington University, St. Louis, MO USA; 10 Department of Obstetrics and Gynecology, Division of Gynecologic Oncology, University of Iowa, Iowa City, IA USA; 11 Department of Anatomic Pathology, The University of Texas MD Anderson Cancer Center, Houston, TX USA; 12 Department of Surgery, Baylor College of Medicine, Houston, TX USA; 13 Center for RNA Interference and Non-Coding RNA, The University of Texas MD Anderson Cancer Center, Houston, TX USA

**Keywords:** Cancer metabolism, Cancer, Oncology

## Abstract

In this study, we investigated the metabolic alterations associated with clinical response to chemotherapy in patients with ovarian cancer. Pre- and post-neoadjuvant chemotherapy (NACT) tissues from patients with high-grade serous ovarian cancer (HGSC) who had poor response (PR) or excellent response (ER) to NACT were examined. Desorption electrospray ionization mass spectrometry (DESI-MS) was performed on sections of HGSC tissues collected according to a rigorous laparoscopic triage algorithm. Quantitative MS-based proteomics and phosphoproteomics were performed on a subgroup of pre-NACT samples. Highly abundant metabolites in the pre-NACT PR tumors were related to *pyrimidine metabolism* in the epithelial regions and *oxygen-dependent proline hydroxylation of hypoxia-inducible factor alpha* in the stromal regions. Metabolites more abundant in the epithelial regions of post-NACT PR tumors were involved in the *metabolism of nucleotides*, and metabolites more abundant in the stromal regions of post-NACT PR tumors were related to *aspartate and asparagine metabolism*, *phenylalanine and tyrosine metabolism*, *nucleotide biosynthesis*, and the *urea cycle*. A predictive model built on ions with differential abundances allowed the classification of patients’ tumor responses as ER or PR with 75% accuracy (10-fold cross-validation ridge regression model). These findings offer new insights related to differential responses to chemotherapy and could lead to novel actionable targets.

## Introduction

The standard first-line chemotherapy approach in high-grade serous ovarian cancer (HGSC) has been a combination of taxanes and platinum for over two decades^1^. High overall mortality^2^ from HGSC is related to an advanced stage at diagnosis and the rapid emergence of chemotherapy resistance. Mechanisms of resistance, including metabolic changes and adaptation, are not fully understood. Here, we used an innovative strategy to characterize spatially resolved metabolic changes in a highly clinically annotated set of HGSC samples collected before and after neoadjuvant chemotherapy (NACT) from patients treated consistently according to a surgical algorithm^3^.

Mass spectrometry (MS) is a powerful technology to spatially characterize the molecular composition of tissues within the tumor microenvironment (TME)^4–8^. We employed desorption electrospray ionization (DESI)-MS, which allows the simultaneous detection of diverse metabolites and lipid species directly from native tissues under ambient conditions^9,10^. The use of histologically compatible spray solvents allows for the same tissue sections to be stained with hematoxylin and eosin (H&E) to visualize tissue morphology^11^. To corroborate metabolic findings with molecular data, we performed global proteomics and phosphoproteomics using laser capture (LC)-MS. These results provide new insights into the metabolic alterations in the tumor and stromal compartments based on response to NACT.

## Results

### Metabolic profiling of pre-chemotherapy tumor tissues using DESI-MS

**Fig. 1:**
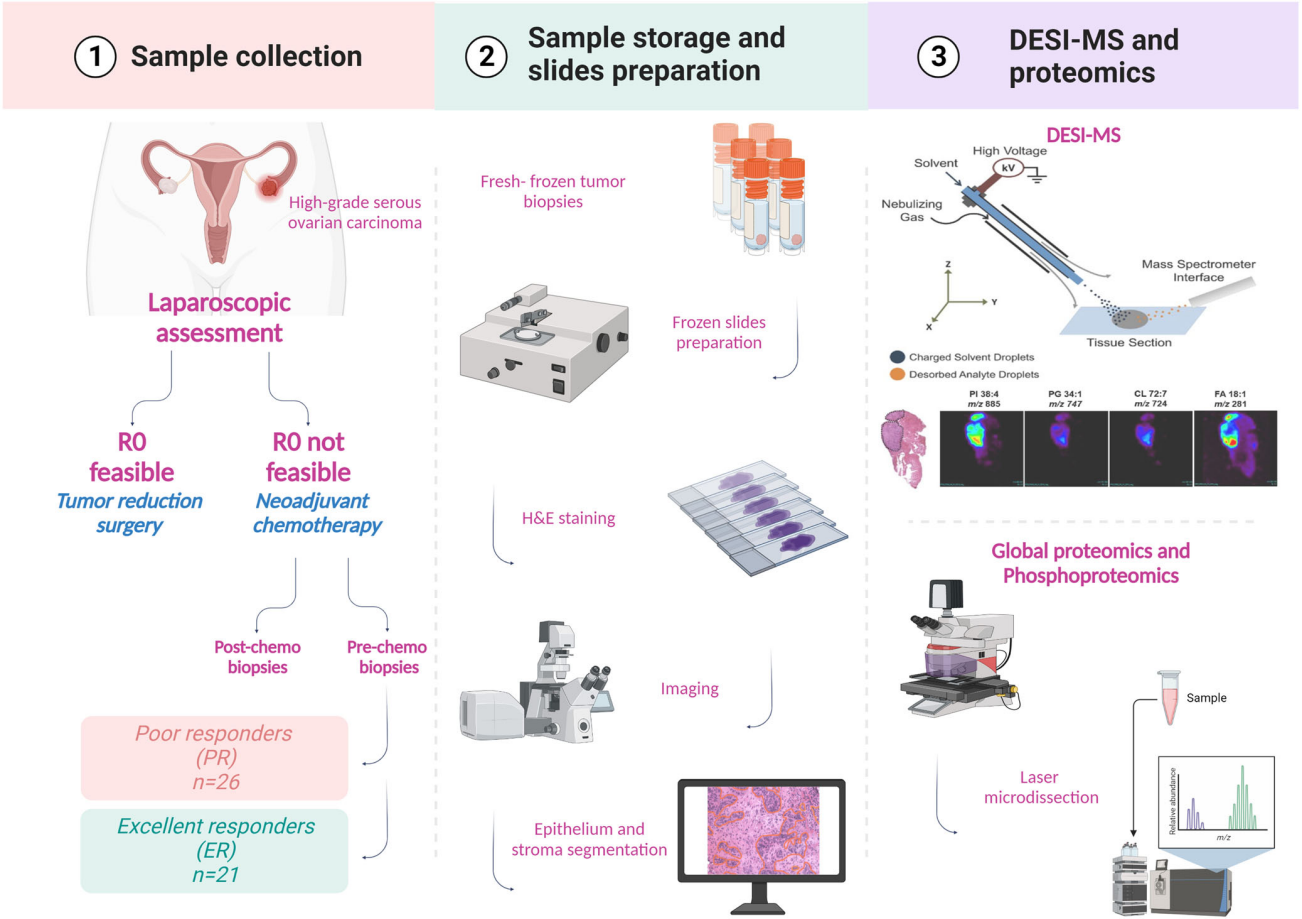
Graphical abstract. R0 absence of macroscopic residual at surgery, ER excellent responders, PR poor responders.

**Fig. 2:**
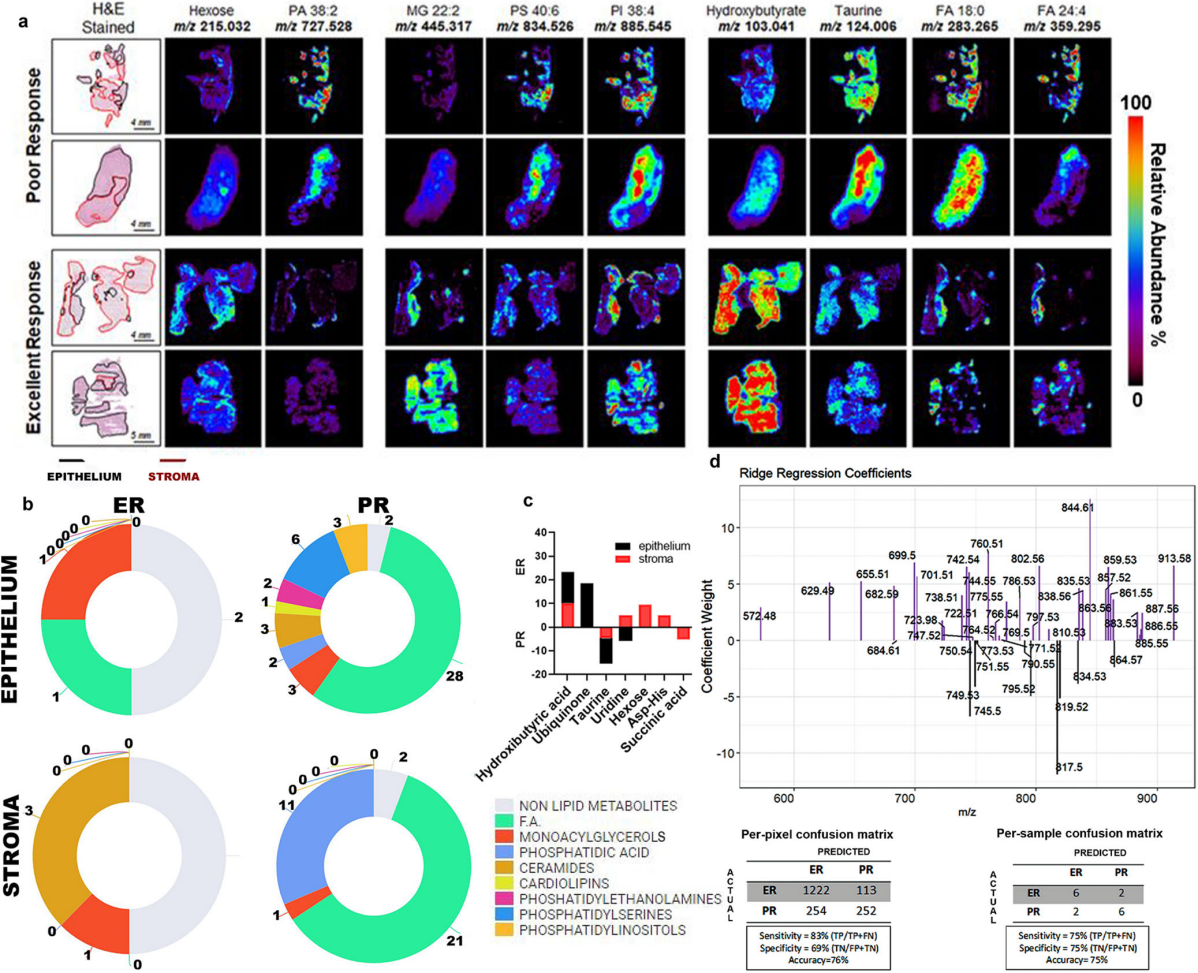
Analysis of high-grade serous ovarian cancer (HGSC) samples obtained prior to neoadjuvant chemotherapy (NACT) based on excellent (ER) or poor (PR) response. a DESI-MS imaging of tumor tissue sections obtained from 52 patients (30 ER and 22 PR) was performed. Negative ion mode DESI-MS ion images of two ER and two PR tumors showing the spatial distribution and relative abundances of nine metabolites and lipid species for each sample are represented. Within each column, the ion images were normalized to the same ion intensity (100% relative abundance, red) for ease of comparison among individual samples and response groups. Optical images of the H&E-stained tissue sections are shown for each sample, with regions of tumor epithelium outlined in black and regions of stroma outlined in red. b Distribution of lipid classes representing higher relative abundances in the stroma and epithelium of ER and PR pre-chemotherapy tissues, from DESI-MS. c Histograms representing relative abundances of small metabolites in the epithelium and stroma of ER and PR tumors. d Plots of ridge regression coefficients for the predictive model. The analysis was restricted to the primary tumor sites (adnexa and ovaries, N = 16), and samples from metastatic sites (omentum or abdominal organs) were excluded (N = 36). A ridge regression model was used to estimate the probability of every mass spectrum belonging to either the ER or PR group. ER excellent responders, PR poor responders.

We first used DESI-MS on pre-NACT tissues from patients stratified as having excellent response (ER) or poor response (PR) to NACT (Supplementary Table 1). MS imaging data were extracted from the epithelial and stromal regions following manual segmentation (Fig. 1). A preliminary analysis on reproducibility was performed where two sections from the same tumor (one mouse xenograft and two human ovarian cancer samples) were analyzed with DESI-MS (Supplementary Fig. 1A) and a cosine similarity test was performed, yielding a score of 0.981 for mouse xenograft (Supplementary Fig. 1B) and 0.998 (Supplementary Fig. 1C) and 0.968 (not shown) for human tumors. In addition, four sections from 4 mouse xenografts were analyzed with an average cosine score of 0.972 (Supplementary Fig. 1D). Manual segmentation allowed us to distinguish between epithelial and stromal areas; the resolution of the technique does not allow for single cell-level segmentation. Stromal regions were mostly characterized by fibroblast-like cells and extracellular matrix (e.g., elastic fibers) that can be identified with H&E stain. Necrotic areas were excluded. Representative DESI-MS images of nine metabolites for two PR and ER tissues are shown in Fig. 2a. We employed negative ion mode DESI-MS imaging to detect small molecules, such as sugars, nucleotides, and amino acids, and a vast range of lipid classes. Multiple lipids, such as fatty acids, monoacylglycerols, ceramides, cardiolipins, and phospholipids, showed different relative abundances between ER and PR tissues. Significance analysis of microarrays (SAM) revealed that the epithelial regions of PR samples, as compared to those of ER samples, had significantly higher relative abundances of fatty acids, phosphatidic acids, ceramides, cardiolipins, and monoacylglycerols; the stromal regions of PR samples had higher relative abundances of fatty acids and phosphatidic acids (Table 1 and Fig. 2b) as compared to those of ER samples. A number of small molecules were also detected in both the ER and PR samples (Fig. 2c); SAM revealed that several molecules had significantly higher relative abundances in the epithelial PR samples than in the epithelial ER samples. In particular, hydroxybutyric acid and ubiquinone were detected at significantly higher relative abundances in the ER samples, while taurine and uridine were detected at significantly higher relative abundances in the PR samples. The stromal regions of the ER samples had significantly higher relative abundances of hydroxybutyric acid, hexose, and uridine, while metabolites with significantly higher abundances in the PR stroma samples included succinic acid and taurine (Table 1).

**Table 1:** Attribution of compounds for pre-chemotherapy tumors (DESI-MS imaging acquired in the negative ion mode; attributions were assigned based on high mass accuracy and MS/MS measurements).

A. Compounds identified by SAM as relatively more abundant in ER (excellent responders) compared to PR (poor responders) samples. Data were extracted from epithelial regions.					
Tentative attribution	Molecular formula	Detected *m/z*	Mass error (ppm)	SAM score	KEGG ID
Metabolites					
Hydroxybutyric acid	C_4_H_7_O_3_	103.0404	−3.0	13.254	C05984
Ubiquinone	C_29_H_41_O_4_	453.3030	−2.3	18.495	C11378
Fatty acids (FA)					
FA 21:1; O	C_21_H_39_O_5_	371.2807	−1.0	7.452	
Monoacylglycerols (MG)					
MG 22:2	C_25_H_46_O_4_Cl	445.3174	−4.2	10.518	

B. Compounds identified by SAM as relatively less abundant in ER compared to PR samples. Data were extracted from epithelial regions.				
Tentative attribution	Molecular formula	Detected *m/z*	Mass error (ppm)	SAM score
Metabolites				
Taurine	C_2_H_6_NO_3_S	124.0078	−1.7	−10.640
Uridine	C_9_H_12_N_2_O_6_Cl	279.0385	1.4	−5.833
Fatty acids (FA)				
FA 10:0	C_10_H_19_O_2_	171.1387	2.3	−5.678
FA 12:2	C_12_H_19_O_2_	195.1394	1.5	−5.146
FA 14:0	C_14_H_27_O_2_	227.2019	−0.9	−6.750
FA 15:4	C_15_H_21_O_2_	233.1550	1.3	−6.450
FA 15:0	C_15_H_29_O_2_	241.2176	1.2	−9.989
FA 16:1	C_16_H_29_O_2_	253.2179	2.4	−4.239
FA 16:0	C_16_H_31_O_2_	255.2332	0.8	−9.877
FA 17:1	C_17_H_31_O_2_	267.2331	0.4	−5.638
FA 17:0	C_17_H_33_O_2_	269.2488	0.7	−7.161
FA 18:3	C_18_H_29_O_2_	277.2174	0.4	−7.261
FA 18:2	C_18_H_31_O_2_	279.2336	2.3	−6.531
FA 18:1	C_18_H_32_O_2_	281.2493	2.5	−5.851
FA 18:0	C_18_H_35_O_2_	283.2648	1.9	−9.898
FA 20:3	C_20_H_33_O_2_	305.2469	−5.6	−4.224
FA 20:2	C_20_H_35_O_2_	307.2628	−4.9	−7.848
FA 20:1	C_20_H_37_O_2_	309.2795	2.3	−6.736
FA 20:0	C_20_H_39_O_2_	311.2954	−0.6	−8.123
FA 18:1	C_18_H_34_O_2_Cl	317.2256	0.9	−5.735
FA 22:6	C_22_H_31_O_2_	327.2326	1.2	−4.395
FA 22:3	C_22_H_37_O_2_	333.2794	1.5	−6.475
FA 22:2	C_22_H_39_O_2_	335.2957	0.3	−9.145
FA 22:1	C_22_H_41_O_2_	337.3117	1.5	−6.872
FA 24:2	C_24_H_43_O_2_	363.3267	−0.6	−9.104
FA 24:1	C_24_H_45_O_2_	365.3427	0.5	−6.978
FA 24:0	C_24_H_47_O_2_	367.3586	1.2	−5.353
FA 26:2	C_26_H_47_O_2_	391.3579	−0.8	−11.433
FA 26:1	C_26_H_49_O_2_	393.3738	0.0	−10.034
FA 26:0	C_26_H_51_O_2_	395.3896	0.3	−5.498
Monoacylglycerols (MG)				
MG 18:2	C_21_H_38_O_4_Cl	389.2468	1.0	−9.014
MG 18:1	C_21_H_40_O_4_Cl	391.2623	0.5	−4.708
MG 20:4	C_23_H_38_O_4_Cl	413.2467	0.7	−7.177
Ceramides (Cer)				
Cer d34:1	C_34_H_67_NO_3_Cl	572.4797	3.1	−10.403
Cer d42:2	C_42_H_81_NO_3_Cl	682.5891	2.9	−9.818
Cer d42:1	C_42_H_81_NO_3_Cl	684.6070	0.4	−4.100
Phosphatidic acids (PA)				
PA 34:1	C_37_H_70_O_8_P	673.4795	−2.8	−5.278
PA 41:6	C_44_H_74_O_8_P	761.5131	0.5	−4.560
Cardiolipins				
CL 72:7	C_81_H_142_O_17_P_2_	724.4836	−4.3	−7.918
Phosphatidylethanolamines				
PE 34:4	C_39_H_69_NO_8_P	710.4752	−2.0	−4.842
PE 38:4	C_43_H_77_NO_8_P	766.5383	−12.0	−10.030
Phosphatidylserines (PS)				
PS 36:2	C_42_H_77_NO_10_P	786.5284	−0.9	−8.830
PS 36:1	C_42_H_79_NO_10_P	788.5443	−0.5	−7.352
PS 38:4	C_44_H_77_NO_10_P	810.5272	−2.3	−10.043
PS 38:3	C_44_H_79_NO_10_P	812.5430	−2.1	−6.212
PS 40:6	C_46_H_77_NO_10_P	834.5261	−3.6	−6.371
PS 40:4	C_46_H_81_NO_10_P	838.5593	−1.3	−7.859
Phosphatidylinositols				
PI 36:4	C_45_H_78_O_13_P	857.5170	−1.9	−6.163
PI 38:5	C_47_H_80_O_13_P	883.5321	−2.4	−4.348
PI 38:4	C_47_H_82_O_13_P	885.5491	−0.9	−8.881

C. Compounds identified by SAM as relatively more abundant in ER compared to PR samples. Data were extracted from stromal regions.				
Metabolites				
Hydroxybutyric acid	C_4_H_7_O_3_	103.0404	−3.0	18.209
Hexose	C_6_H_12_O_6_Cl	215.0327	−0.4	9.5241
Asp-His	C_10_H_13_N_4_O_5_	269.0883	1.8	5.1653
Uridine	C_9_H_12_N_2_O_6_Cl	279.0385	1.4	5.157
Monoacylglycerols				
MG 20:0	C_23_H_46_O_4_Cl	421.3103	3.1	6.5347
Ceramides				
Cer d34:1	C_34_H_67_NO_3_Cl	572.4797	3.1	10.64
Cer d42:2	C_42_H_81_NO_3_Cl	682.5891	2.9	8.031
Cer d42:1	C_42_H_83_NO_3_Cl	684.6081	2.1	8.0668

D. Compounds identified by SAM as relatively less abundant in ER compared to PR samples. Data were extracted from stromal regions.				
Metabolites				
Succinic acid	C_4_H_5_O_4_	117.0195	−1.4	−5.0948
Taurine	C_2_H_6_NO_3_S	124.0078	0.7	−4.6595
Fatty Acids (FA)				
FA 9:1;O	C_9_H_15_O_3_	171.1029	1.4	−5.5852
FA 11:1;O	C_11_H_19_O_3_	199.1344	2.2	−4.4793
FA 16:1	C_16_H_29_O_2_	253.2179	2.4	−6.796
FA 18:3	C_18_H_29_O_2_	277.2165	2.9	−5.2488
FA 18:2	C_18_H_31_O_2_	279.2336	2.3	−7.3715
FA 18:1	C_18_H_32_O_2_	281.2493	2.5	−7.7215
FA 18:0	C_18_H_35_O_2_	283.2648	1.9	−4.8951
FA 20:4	C_20_H_31_O_2_	303.2333	1.1	−7.0052
FA 20:3	C_20_H_33_O_2_	305.2476	3.3	−9.9855
FA 20:2	C_20_H_35_O_2_	307.2638	−1.6	−9.4183
FA 20:1	C_20_H_37_O_2_	309.2795	2.3	−9.1167
FA 22:6	C_22_H_31_O_2_	327.2326	1.2	−5.0138
FA 22:5	C_22_H_33_O_2_	329.2477	2.7	−5.781
FA 22:4	C_22_H_35_O_2_	331.2649	2.0	−10.495
FA 22:3	C_22_H_37_O_2_	333.2794	1.5	−7.3874
FA 22:2	C_22_H_39_O_2_	335.2952	1.2	−5.069
FA 22:1	C_22_H_41_O_2_	337.3117	1.5	−7.3867
FA 24:4	C_24_H_39_O_2_	359.2947	2.5	−8.9018
FA 24:2	C_24_H_43_O_2_	363.3262	1.9	−5.7095
FA 24:1	C_24_H_45_O_2_	365.3427	0.5	−8.6896
FA 24:0	C_24_H_47_O_2_	367.3586	1.2	−6.4545
Monoacylglycerols (MG)				
MG 16:0	C_19_H_38_O_4_Cl	365.2458	1.6	−4.9891
Phosphatidic acids (PA)				
PA 35:2	C_38_H_70_O_8_P	685.4814	0	−7.2447
PA 35:1	C_38_H_72_O_8_P	687.497	0	−6.1941
PA 36:2	C_39_H_72_O_8_P	699.4971	0.1	−7.4984
PA 36:1	C_39_H_74_O_8_P	701.5126	0.1	−6.2007
PA 37:4	C_40_H_70_O_8_P	709.4815	0.2	−7.8465
PA 37:3	C_40_H_70_O_8_P	711.4971	0.1	−9.3891
PA 37:2	C_40_H_74_O_8_P	713.5129	0.3	−8.9105
PA 38:3	C_41_H_74_O_8_P	725.5126	−0.1	−7.6253
PA 38:2	C_41_H_76_O_8_P	727.5278	−0.7	−7.9704
PA 39:5	C_42_H_72_O_8_P	735.4974	0.5	−7.9133
PA 39:4	C_42_H_74_O_8_P	737.5126	−0.1	−9.1543

To investigate if the small metabolites whose relative abundances significantly differed between ER and PR tumors were associated with specific metabolic pathways, we used two publicly available software programs. These programs analyzed the metabolites with higher or lower relative abundances in the different cohorts and tissues and provided the metabolic pathways in which such metabolites are particularly enriched. Taurine and uridine (Table 1B), which were detected at higher relative abundances in the epithelium of PR samples, mapped mainly to the “recycle of bile acids and salts” pathway (FDR-adjusted *p* < 0.05) and the “pyrimidine salvage and catabolism” pathway (FDR-adjusted *p* < 0.05) (Supplementary Table 2A, B). In contrast, the metabolites hydroxybutyric acid and ubiquinone (Table 1A), which had higher relative abundance in the epithelial regions of ER samples, showed a correlation with the “respiratory electron transport” and “metabolism of amino acids and derivatives” pathways (FDR-adjusted *p* < 0.05) (Supplementary Table 3A).

In the stromal regions of the pre-chemotherapy tissues, hydroxybutyric acid, hexose, asp-his, and uridine, which were detected at higher relative abundances in ER samples (Table 1C), were associated with several pathways, including the “pyrimidine salvage” pathway (Supplementary Table 4A, B, Table 2A). The metabolites succinic acid and taurine (Table 1D), which had higher abundances in PR tumors, were related to the “transport of bile salts and organic acids metal ions and amine compounds” and ”oxygen-dependent proline hydroxylation of hypoxia-inducible factor alpha” pathways (FDR-adjusted *p* < 0.05 for both) (Supplementary Table 5A, B, Table 2A). Table 2A summarizes the pathways related to the detected metabolites that may be altered in pre-chemotherapy ER and PR tissue samples.

**Table 2:** Summary of deregulated pathways.

A. Metabolic pathways upregulated in pre-chemotherapy tissues from ER and PR tumors.			
Upregulated pathways in chemo-naïve tissues of ER versus PR			
ER		PR	
Stroma	Epithelium	Stroma	Epithelium
Pyrimidine salvage (entities ratio 0.01)	Respiratory electron transport^a^ (entities ratio 0.008)	Transport of bile salts and organic acids metal ions and amine compounds^a^ (entities ratio 0.04)	Recycle of bile acids and salts (entities ratio 0.01)
	Metabolism of amino acids and derivatives (entities ratio 0.15)	Oxygen-dependent proline hydroxylation of Hypoxia-inducible Factor Alpha^a^ (entities ratio 0.003)	Pyrimidine salvage (entities ratio 0.01)
			Pyrimidine catabolism (entities ratio 0.02)

B. Metabolic pathways upregulated and downregulated in post- versus pre-chemotherapy tissues from ER and PR tumors.				
Deregulated pathways in post-chemo versus pre-chemo tissues of ER and PR				
ER		PR		
Stroma	Epithelium	Stroma	Epithelium	
Upregulated	Urea cycle (entities ratio 0.12)	TP53 Regulates Metabolic Genes (entities ratio 0.01)	Aspartate and asparagine metabolism (entities ratio 0.01)	TP53 Regulates Metabolic Gene (entities ratio 0.01)
	Citric acids cycle (entities ratio 0.02)	Metabolism of nucleotides (entities ratio 0.08)	Phenylalanine and tyrosine metabolism (entities ratio 0.02)	Metabolism of nucleotides (entities ratio 0.08)
			Nucleotide biosynthesis (entities ratio 0.03)	
			Urea cycle (entities ratio 0.01)	
Downregulated	Metabolism of nucleotides^b^ (entities ratio 0.08)	Urea cycle^b^ (entities ratio 0.01)	GABA degradation and synthesis (entities ratio 0.01)	2-Hydroxyglutarate
	Nucleotide salvage^b^ (entities ratio 0.03)	Metabolism of nucleotides^b^ (entities ratio 0.08)		
	Purine catabolism^b^ (entities ratio 0.03)	Phenylalanine and tyrosine metabolism^b^ (entities ratio 0.02)		

^a^*p* < 0.05 probability score corrected for false-discovery rate (FDR) using Benjamini-Hochberg method.

^b^*p* < 0.01 probability score corrected for false-discovery rate (FDR) using Benjamini-Hochberg method.

Next, we built a predictive model based on the DESI-MS data extracted from the pre-NACT samples from the ER and PR groups acquired from tumor primary sites (*N* = 16). This model predicted response to chemotherapy using cross-validation with a per-pixel sensitivity of 83%, specificity of 69%, and total accuracy of 76%, with a positive predictive value of 92% (Fig. 2d). When the predictive performance per patient was analyzed, sensitivity, specificity, and accuracy values of 75% were achieved.

### Metabolic profiling of post-chemotherapy tumor tissues

Next, we analyzed the matched post-chemotherapy tissues from ER and PR tumors and examined the metabolic changes occurring in response to chemotherapy. Discriminant analysis using the sparse partial least squares algorithm was used to identify and plot the most discriminative features^12^. When the epithelial areas of matched pre- and post-NACT tissues were analyzed, the number of metabolic species with lower relative abundances after chemotherapy (as compared to pre-chemotherapy) was higher in ER tumors than in PR tumors. Specifically, 113 metabolites (small molecules and lipids) had lower relative abundances in the epithelial areas of post-NACT tissues of ER tumors, while 65 metabolic species had higher relative abundances in the epithelial areas of post-NACT tissues of ER tumors (SAM, FDR *p* < 0.01) (Table 3A, B). In the epithelial areas of PR tumors, 60 metabolic species showed lower relative abundances in post-NACT tissues, while 45 metabolic species showed higher relative abundances in post-NACT tissues compared to pre-chemotherapy ones (Table 3C, D). Small metabolites (but not lipids) with lower relative abundances in the epithelial regions of post-NACT ER tissues included glycerophosphoethanolamine, citrate, glutamic acid, hypoxanthine, aspartate, pyroglutamate, fumarate, and uracil. Conversely, hydroxyglutaric acid was the only small metabolite with a lower relative abundance in the epithelial areas of post-NACT PR tissues.

**Table 3:** Attribution of compounds for post- versus pre-chemotherapy tumors (the m/z data was acquired using DESI-MS imaging in the negative ion mode; attributions were assigned based on high mass accuracy and MS/MS measurements).

A. Compounds identified by SAM as relatively less abundant in post- compared to pre-chemotherapy samples. Data were extracted from epithelial regions of ER samples.				
Tentative attribution	Molecular formula	Detected *m/z*	Mass error (ppm)	SAM score
Metabolites				
Uracil	C_4_H_3_O_2_N_2_	111.0199	−0.9	−20.440
Fumarate	C_47_H_84_O_13_P	115.0039	1.7	−4.562
Pyroglutamate	C_5_H_6_NO_3_	128.0355	1.6	−3.348
Aspartate	C_4_H_6_NO_4_	132.0305	2.3	−4.681
Hypoxanthine	C_5_H_3_ON_4_	135.0317	3.5	−22.383
Glutamic acid	C_5_H_8_NO_4_	146.0449	−6.7	−5.777
Citrate	C_6_H_7_O_7_	191.0193	−2.1	−4.788
Glycerophosphoethanolamine	C_5_H_13_O_6_NP	214.0481	−2.3	−10.391
Galactosylglycerol or Glucosylglycerol	C_9_H_17_O_8_	253.0931	0.8	−16.093
Fatty acids (FA)				
FA 9:0	C_9_H_17_O_2_	157.1235	0.6	−10.222
FA 14:3	C_14_H_21_O_2_	221.1547	0.0	−15.669
FA 14:1	C_14_H_25_O_2_	225.1862	0.9	−9.174
FA 14:0	C_14_H_27_O_2_	227.2014	−1.1	−7.506
FA 16:2	C_16_H_27_O_2_	251.2008	3.6	−6.347
FA 16:1	C_16_H_29_O_2_	253.2177	1.6	−8.238
FA 18:1	C_18_H_32_O_2_	281.2492	2.1	−26.771
FA 18:0	C_18_H_35_O_2_	283.2648	1.9	−13.642
FA 20:3	C_20_H_33_O_2_	305.2483	−1.0	−11.617
FA 20:2	C_20_H_35_O_2_	307.2638	−1.6	−29.185
FA 20:1	C_20_H_37_O_2_	309.2795	−2.3	−31.634
FA 20:0	C_20_H_39_O_2_	311.2952	−1.3	−9.691
FA 18:0	C_18_H_36_O_2_Cl	319.2407	−0.6	−4.592
FA 22:4	C_22_H_35_O_2_	331.2649	2.0	−24.749
FA 22:3	C_22_H_37_O_2_	333.2794	−1.5	−17.342
FA 22:2	C_22_H_39_O_2_	335.2952	−1.2	−19.512
FA 22:1	C_22_H_41_O_2_	337.3102	−3.0	−32.050
FA 22:0	C_22_H_43_O_2_	339.3269	−0.1	−17.807
FA 24:5	C_24_H_37_O_2_	357.2807	2.2	−6.846
FA 24:4	C_24_H_39_O_2_	359.2947	−2.5	−9.482
FA 24:3	C_24_H_41_O_2_	361.3106	−1.7	−9.997
FA 24:2	C_24_H_43_O_2_	363.3262	−1.9	−15.153
FA 24:1	C_24_H_45_O_2_	365.3427	0.5	−32.048
FA 24:0	C_24_H_47_O_2_	367.3586	1.2	−22.423
FA 26:5	C_26_H_41_O_2_	385.3105	−1.8	−4.939
FA 26:2	C_26_H_47_O_2_	391.3587	1.4	−7.404
FA 26:1	C_26_H_49_O_5_	393.3734	−1.0	−15.907
FA 11:1;O	C_11_H_19_O_3_	199.1344	2.2	−14.381
Diacylglycerols (DG)				
DG 34:2	C_37_H_68_O_5_Cl	627.4758	−0.4	−7.127
DG 34:1	C_37_H_70_O_5_Cl	629.4913	−0.6	−11.224
DG 36:3	C_39_H_70_O_5_Cl	653.4928	1.6	−10.206
DG 36:2	C_39_H_72_O_5_Cl	655.5080	1.0	−19.553
DG 38:4	C_41_H_72_O_5_Cl	679.5089	2.2	−6.385
Ceramides (Cer)				
Cer d34:0	C_34_H_69_NO_3_Cl	574.4962	−1.6	−9.628
Cer d38:1	C_38_H_75_NO_3_Cl	628.5462	3.3	−6.822
Glycerophosphoethanolamines (PE)				
PE O-34:2 or PE P-34:1	C_39_H_75_NO_7_P	700.5272	−2.1	−8.500
PE 34:2	C_39_H_73_NO_8_P	714.5052	−3.8	−13.740
PE 34:1	C_39_H_75_NO_8_P	716.5248	1.7	−20.240
PE O-36:3 or P-36:2	C_41_H_77_NO_7_P	726.5459	2.2	−12.602
PE O-36:2 or PE P-36:1	C_41_H_79_NO_7_P	728.5631	4.3	−14.666
PE 36:3	C_41_H_75_NO_8_P	740.5232	−0.5	−24.207
PE 36:2	C_41_H_77_NO_8_P	742.5407	2.0	−23.970
PE 36:1	C_41_H_79_NO_8_P	744.5533	−2.1	−12.773
PE O-38:6 or PE P-38:5	C_43_H_75_NO_7_P	748.5255	−4.2	−8.770
PE O-38:5 or PE P-38:4	C_43_H_77_NO_7_P	750.5443	0.0	−6.960
PE O-38:4 or PE P-38:3	C_43_H_79_NO_7_P	752.5554	−6.1	−11.648
PE 38:5	C_43_H_75_NO_8_P	764.5244	1.1	−15.555
PE 38:3	C_43_H_79_NO_8_P	768.5546	−0.4	−15.319
PE 39:6	C_44_H_75_NO_8_P	776.5258	2.9	−4.261
PE 40:5	C_45_H_79_NO_8_P	792.5545	−1.6	−12.876
PE 37:1	C_42_H_82_NO_8_PCl	794.5485	0.8	−8.316
PE O-40:8 or PE P-40:7	C_45_H_76_NO_8_PCl	808.5068	1.8	−7.000
PE 39:2	C_44_H_84_NO_8_PCl	820.5603	3.1	−6.123
PE 39:1	C_44_H_86_NO_8_PCl	822.5730	−6.7	−6.590
Cardiolipins (CL)				
CL 70:7	C_79_H_138_O_17_P_2_	710.4697	−1.8	−10.258
CL 70:6	C_79_H_140_O_17_P_2_	711.4767	−3.0	−5.128
CL 72:8	C_81_H_140_O_17_P_2_	723.4766	−3.0	−9.789
CL 72:7	C_81_H_142_O_17_P_2_	724.4855	−1.6	−4.359
CL 72:6	C_81_H_144_O_17_P_2_	725.4940	−0.7	−9.349
CL 74:9	C_83_H_142_O_17_P_2_	736.4847	−2.7	−11.996
CL 74:8	C_83_H_144_O_17_P_2_	737.4921	−3.3	−10.454
CL 74:7	C_83_H_146_O_17_P_2_	738.5015	−1.1	−16.994
CL 74:6	C_83_H_148_O_17_P_2_	739.5074	−3.7	−5.309
Phosphatidic Acids (PA)				
PA 36:2	C_39_H_72_O_8_P	699.4948	−3.1	−6.786
PA 36:1	C_39_H_74_O_8_P	701.5120	−1.0	−5.129
Glycerophosphoinositols (PI)				
LysoPI 18:0	C_27_H_52_O_12_P	599.3215	2.2	−7.078
PI 32:1	C_41_H_76_O_13_P	807.5016	−1.6	−12.308
PI 34:2	C_43_H_78_O_13_P	833.5166	−2.4	−21.550
PI 34:1	C_43_H_80_O_13_P	835.5342	0.0	−32.764
PI 36:4	C_45_H_78_O_13_P	857.5163	−2.7	−15.171
PI 36:3	C_45_H_80_O_13_P	859.5347	0.6	−16.601
PI 36:2	C_45_H_82_O_13_P	861.5486	−1.5	−33.085
PI 36:1	C_45_H_84_O_13_P	863.5643	−1.4	−28.121
PI 38:5	C_47_H_80_O_13_P	883.5332	−1.1	−17.153
PI 38:3	C_47_H_84_O_13_P	887.5629	−2.9	−18.851
PI 38:2	C_47_H_86_O_13_P	889.5752	−6.7	−20.727
PI 40:5	C_49_H_84_O_13_P	911.5638	−1.9	−14.072
PI 40:4	C_49_H_86_O_13_P	913.5793	−2.1	−19.153
Glycerophosphoglycerols (PG)				
LysoPG 18:1	C_24_H_46_O_9_P	509.2881	−2.9	−8.881
PG 32:0	C_38_H_75_O_10_P	721.5026	0.1	−14.381
PG 34:2	C_40_H_74_O_10_P	745.5015	−1.5	−19.098
PG 36:2	C_42_H_78_O_10_P	773.5358	2.6	−22.063
PG 36:1	C_42_H_80_O_10_P	775.5507	1.6	−28.345
PG 38:5	C_44_H_76_O_10_P	795.5153	−3.6	−8.290
PG 38:4	C_44_H_78_O_10_P	797.5313	−3.1	−21.018
PG 38:3	C_44_H_80_O_10_P	799.5467	−3.5	−20.588
PG 40:7	C_46_H_76_O_10_P	819.5160	−2.7	−9.366
PG 40:6	C_46_H_78_O_10_P	821.5309	−3.5	−8.422
PG 40:5	C_46_H_80_O_10_P	823.5496	0.2	−6.882
PG 38:1	C_44_H_85_O_10_PCl	839.5527	−5.6	−22.818
PG 40:2	C_46_H_87_O_10_PCl	865.5725	−0.7	−28.840
Glycerophosphoserines (PS)				
PS 35:2	C_41_H_75_NO_10_P	772.5187	−6.8	−8.281
PS 36:2	C_42_H_77_NO_10_P	786.5270	−2.7	−9.734
PS 37:1	C_43_H_81_NO_10_P	802.5659	6.9	−3.325
PS 38:4	C_44_H_77_NO_10_P	810.5296	0.7	−7.364
PS 38:3	C_44_H_79_NO_10_P	812.5437	−1.2	−14.019
PS 38:2	C_44_H_81_NO_10_P	814.5577	−3.3	−8.148
PS 38:1	C_44_H_83_O_10_NP	816.5745	−1.8	−3.511
PS 39:4	C_45_H_79_NO_10_P	824.5454	0.8	−7.984
PS 40:6	C_46_H_77_NO_10_P	834.5271	−2.4	−15.284
PS 40:5	C_46_H_79_NO_10_P	836.5406	−4.9	−27.667
PS 40:4	C_46_H_81_NO_10_P	838.5644	4.8	−26.875
PS 40:3	C_46_H_83_NO_10_P	840.5746	−1.7	−13.881
PS 40:1	C_46_H_87_O_10_NP	844.6080	0.8	−6.663

B. Compounds identified by SAM as relatively more abundant in post- compared to pre-chemotherapy samples. Data were extracted from epithelial regions of ER samples.				
Metabolites				
Hydroxyvaleric acid	C_5_H_9_O_3_	117.0559	1.7	7.770
Taurine	C_2_H_6_NO_3_S	124.0064	−8.0	21.448
Leucinic acid or Leucic acid	C_6_H_11_O_3_	131.0721	1.6	5.152
Hydroxynicotinic acid	C_6_H_4_NO_3_	138.0198	0.7	7.478
Glutamine	C_5_H_9_N_2_O_3_	145.0621	1.4	16.405
Xanthine	C_5_H_3_O_2_N_4_	151.0260	−0.7	8.581
Aconitic acid	C_6_H_5_O_6_	173.0096	2.5	8.680
Ascorbic acid	C_6_H_7_O_6_	175.0252	2.3	55.475
Hexose	C_6_H_11_O_6_	179.0562	0.5	19.654
Methylaconitate	C_7_H_7_O_6_	187.0252	2.1	13.749
Ribitol or Xylitol	C_5_H_12_O_9_Cl	187.0363	−4.6	36.741
Galactonic or Gluconic acid	C_6_H_11_O_7_	195.0511	0.1	5.483
Hexose	C_6_H_12_O_6_Cl	215.0327	−0.4	28.667
Methyluric acid	C_6_H_6_N_4_O_3_Cl	217.0121	3.4	12.073
Inosine	C_10_H_11_N_4_O_5_	267.0735	0.0	21.397
Asp-His	C_10_H_13_N_4_O_5_	269.0883	−1.8	37.237
Glutathione	C_10_H_16_N_3_O_6_S	306.0773	2.5	36.893
Dehydrocholesterol	C_27_H_44_OCl	419.3021	−5.9	27.631
Fatty acids (FA)				
FA 15:0	C_15_H_29_O_2_	241.2172	−0.4	15.165
FA 19:0	C_19_H_37_O_2_	297.2792	−2.4	7.445
FA 20:5	C_20_H_29_O_2_	301.2174	0.3	22.496
FA 20:4	C_20_H_31_O_2_	303.2333	1.1	15.808
FA 22:6	C_22_H_31_O_2_	327.2326	1.2	22.369
FA 20:4	C_20_H_32_O_2_Cl	339.2088	2.4	25.097
FA 22:6	C_22_H_32_O_2_Cl	363.2094	−0.6	17.398
FA 22:3	C_22_H_38_O_2_Cl	369.2554	−2.7	12.146
FA 24:5	C_24_H_38_O_2_Cl	393.2582	4.1	55.254
FA 36:3	C_36_H_63_O_4_	559.4735	0.6	7.954
Monoacylglycerols (MG) and Diacylglycerols (DG)				
MG 20:4	C_23_H_38_O_4_Cl	413.2465	0.2	39.965
MG 20:3	C_23_H_40_O_4_Cl	415.2631	2.5	12.391
MG 22:6	C_25_H_38_O_4_Cl	437.2459	−1.1	17.631
DG 40:10	C_43_H_63_O_5_	659.4680	−0.1	19.690
Ceramides (Cer)				
Cer d34:1	C_34_H_67_NO_3_Cl	572.4818	0.5	13.771
Cer d38:2	C_38_H_73_NO_3_Cl	626.5350	10.0	32.563
PI-Cer d27:2	C_33_H_61_NO_11_P	678.3983	−0.7	20.687
Cer d42:1	C_42_H_83_NO_3_Cl	684.6072	0.7	15.105
PE-Cer d36:1	C_38_H_76_N_2_O_6_P	687.5449	0.4	46.883
Cer d46:2	C_46_H_89_NO_3_Cl	738.6601	8.7	21.894
Glycerophosphoethanolamines (PE)				
PE O-36:5 or PE P-36:4	C_41_H_73_NO_7_Cl	722.5116	−1.9	17.341
PE 38:6	C_43_H_73_NO_8_P	762.5082	0.4	14.431
PE O-38:3 or PE P-38:2	C_43_H_82_NO_7_PCl	790.5535	1.5	18.181
PE 39:4	C_44_H_80_NO_8_PCl	816.5310	−0.7	12.594
Cardiolipins (CL)				
CL 70:5	C_79_H_142_O_17_P_2_	712.4837	−4.2	20.813
CL 74:10	C_83_H_140_O_17_P_2_	735.4814	3.5	44.982
Phosphatidic Acids (PA)				
PA 24:2	C_37_H_68_O_8_P	671.4676	2.8	25.761
PA 24:1	C_37_H_70_O_8_P	673.4814	1.6	10.164
PA 35:2	C_38_H_70_O_8_P	685.4819	0.8	19.362
PA 36:4	C_39_H_68_O_8_P	695.4690	4.7	40.482
PA 36:3	C_39_H_70_O_8_P	697.4815	0.2	11.986
PA 37:5	C_40_H_68_O_8_P	707.4674	2.4	17.812
PA 37:2	C_40_H_74_O_8_P	713.5129	0.3	22.091
PA 39:6	C_42_H_70_O_8_P	733.4810	−0.5	27.702
PA 39:3	C_42_H_76_O_8_P	739.5256	−3.7	14.616
PA 41:6	C_44_H_74_O_8_P	761.5147	2.7	16.314
PA 41:5	C_44_H_76_O_8_P	763.5256	−3.6	18.852
Glycerophosphoinositols (PI)				
LysoPI 15:0	C_24_H_46_O_12_P	557.2729	−0.5	10.733
PI 32:0	C_41_H_78_O_13_P	809.5158	−3.5	8.976
PI 38:6	C_47_H_78_O_13_P	881.5196	1.1	9.944
PI 39:5	C_48_H_83_O_13_PCl	933.5302	3.9	9.518
Glycerophosphoglycerols (PG)				
PG 40:8	C_46_H_74_O_10_P	817.5011	1.7	8.858
PG 44:12	C_50_H_74_O_10_P	865.4996	3.4	7.085
Glycerophosphoserines (PS)				
PS 36:1	C_42_H_79_NO_10_P	788.5466	2.4	13.818
PS 41:6	C_46_H_81_NO_10_P	848.5439	−0.9	7.492
PS 39:8	C_45_H_72_NO_10_PCl	852.4524	−7.5	26.773
PS 42:1	C_48_H_91_O_10_NP	872.6408	2.5	22.164

C. Compounds identified by SAM as relatively less abundant in post- compared to pre-chemotherapy samples. Data were extracted from epithelial regions of PR samples.				
Metabolites				
Hydroxyglutaric acid	C_5_H_7_O_5_	147.0305	4.1	−7.003
Fatty acids (FA)				
FA 14:3	C_14_H_21_O_2_	221.1550	1.3	−6.927
FA 14:1	C_14_H_25_O_2_	225.1866	2.7	−6.208
FA 16:1	C_16_H_29_O_2_	253.2179	2.4	−8.600
FA 17:1	C_17_H_31_O_2_	267.2336	2.4	−12.007
FA 18:2	C_18_H_31_O_2_	279.2336	2.3	−6.317
FA 18:1	C_18_H_32_O_2_	281.2493	2.5	−17.734
FA 18:0	C_18_H_35_O_2_	283.2648	1.9	−5.779
FA 19:1	C_19_H_35_O_2_	295.2650	2.5	−9.818
FA 20:2	C_20_H_35_O_2_	307.2638	−1.6	−18.639
FA 20:1	C_20_H_37_O_2_	309.2795	2.3	−21.688
FA 20:0	C_20_H_39_O_2_	311.2952	−1.3	−12.279
FA 22:4	C_22_H_35_O_2_	331.2649	2.0	−13.417
FA 22:3	C_22_H_37_O_2_	333.2794	−1.5	−8.500
FA 22:2	C_22_H_39_O_2_	335.2952	−1.2	−13.027
FA 22:1	C_22_H_41_O_2_	337.3117	1.5	−19.667
FA 23:0	C_23_H_45_O_2_	353.3420	−1.4	−5.843
FA 24:4	C_24_H_39_O_2_	359.2947	−2.5	−9.861
FA 24:3	C_24_H_41_O_2_	361.3106	−1.7	−7.446
FA 24:2	C_24_H_43_O_2_	363.3262	−1.9	−11.294
FA 24:1	C_24_H_45_O_2_	365.3427	0.5	−18.486
FA 24:0	C_24_H_47_O_2_	367.3586	1.2	−7.162
FA 26:2	C_26_H_47_O_2_	391.3590	2.2	−12.245
FA 26:1	C_26_H_49_O_5_	393.3734	−1.0	−11.849
Glycerophosphoethanolamines (PE)				
PE O-34:3 or PE P-34:2	C_39_H_73_NO_7_P	698.5133	0.4	−8.520
PE O-34:2 or PE P-34:1	C_39_H_75_NO_7_P	700.5272	−2.1	−7.230
PE 34:2	C_39_H_73_NO_8_P	714.5052	−3.8	−7.365
PE 34:1	C_39_H_75_NO_8_P	716.5248	1.7	−9.922
PE 35:3	C_40_H_74_NO_8_P	726.5027	−7.2	−9.643
PE O-36:3 or PE P-36:2	C_41_H_77_NO_7_P	726.5459	2.2	−8.766
PE 36:3	C_41_H_75_NO_8_P	740.5232	−0.5	−9.819
PE 36:1	C_41_H_79_NO_8_P	744.5533	−2.1	−7.509
Cardiolipins (CL)				
CL 70:7	C_79_H_138_O_17_P_2_	710.4697	−1.8	−7.162
CL 70:6	C_79_H_140_O_17_P_2_	711.4767	−3.0	−9.924
CL 70:4	C_79_H_144_O_17_P_2_	713.4935	−1.4	−9.373
CL 72:7	C_81_H_142_O_17_P_2_	724.4855	−1.6	−6.559
CL 72:6	C_81_H_144_O_17_P_2_	725.4940	−0.7	−11.899
CL 72:4	C_81_H_148_O_17_P_2_	727.5054	−2.0	−7.283
CL 74:8	C_83_H_144_O_17_P_2_	737.4921	−3.3	−5.934
CL 74:7	C_83_H_146_O_17_P_2_	738.5015	−1.1	−9.658
CL 74:6	C_83_H_148_O_17_P_2_	739.5074	−3.7	−6.328
Glycerophosphoinositols (PI)				
PI 34:1	C_43_H_80_O_13_P	835.5342	0.0	−8.203
PI 36:2	C_45_H_82_O_13_P	861.5486	−1.5	−9.421
PI 36:1	C_45_H_84_O_13_P	863.5643	−1.4	−12.320
PI 40:4	C_49_H_86_O_13_P	913.5811	−0.1	−8.275
Glycerophosphoglycerols (PG)				
PG 34:1	C_40_H_76_O_10_P	747.5160	−2.9	−11.758
PG 36:3	C_42_H_76_O_10_P	771.5152	−3.9	−9.188
PG 36:2	C_42_H_78_O_10_P	773.5358	2.6	−7.498
PG 36:1	C_42_H_80_O_10_P	775.5507	1.6	−6.307
PG 38:4	C_44_H_78_O_10_P	797.5313	−3.1	−7.565
PG 38:3	C_44_H_80_O_10_P	799.5467	−3.5	−7.821
PG 38:2	C_44_H_82_O_10_P	801.5639	−1.5	−6.257
PG 40:6	C_46_H_78_O_10_P	821.5309	−3.5	−6.754
PG 40:5	C_46_H_80_O_10_P	823.5496	0.2	−5.715
PG 38:1	C_44_H_85_O_10_PCl	839.5527	−5.6	−6.945
PG 40:2	C_46_H_87_O_10_PCl	865.5725	−0.7	−5.844
Glycerophosphoserines (PS)				
PS 36:2	C_42_H_77_NO_10_P	786.5270	−2.7	−6.506
PS 40:6	C_46_H_77_NO_10_P	834.5271	2.4	−6.066
PS 40:4	C_46_H_81_NO_10_P	838.5644	−4.8	−8.518
PS 40:3	C_46_H_83_NO_10_P	840.5746	−1.7	−8.019

D. Compounds identified by SAM as relatively more abundant in post- compared to pre-chemotherapy samples. Data were extracted from epithelial regions of PR samples.				
Metabolites				
Taurine	C_2_H_6_NO_3_S	124.0064	−8.0	21.448
Glutamine	C_5_H_9_N_2_O_3_	145.0621	1.4	15.322
Xanthine	C_5_H_3_O_2_N_4_	151.0260	−0.7	12.537
Aconitic acid	C_6_H_5_O_6_	173.0096	2.5	10.117
Ascorbic acid	C_6_H_7_O_6_	175.0252	2.3	27.156
Hexose	C_6_H_12_O_6_Cl	215.0327	−0.4	18.663
Asp-His	C_10_H_13_N_4_O_5_	269.0883	1.8	20.157
Inosine	C_10_H_11_N_4_O_5_	267.0735	0.0	11.295
Glutathione	C_10_H_16_N_3_O_6_S	306.0773	2.5	13.103
Fatty acids (FA)				
FA 16:0	C_16_H_31_O_2_	255.2324	−2.4	9.961
FA 20:5	C_20_H_29_O_2_	301.2174	0.3	12.822
FA 22:6	C_22_H_31_O_2_	327.2326	−1.2	13.521
FA 20:4	C_20_H_32_O_2_Cl	339.2088	−2.4	11.230
FA 24:6	C_24_H_35_O_2_	355.2634	−2.5	9.375
FA 22:3	C_22_H_38_O_2_Cl	369.2554	−2.7	14.855
FA 24:5	C_24_H_38_O_2_Cl	393.2582	−4.1	12.955
Monoacylglycerols (MG) and Diacylglycerols (DG)				
MG 18:0	C_21_H_40_O_4_Cl	391.2620	−0.2	10.376
MG 20:4	C_23_H_38_O_4_Cl	413.2465	0.2	17.338
MG 20:3	C_23_H_40_O_4_Cl	415.2631	2.5	20.052
MG 22:6	C_25_H_38_O_4_Cl	437.2459	−1.1	15.148
DG 36:4	C_39_H_68_O_5_Cl	651.4748	−2.0	9.042
DG 36:1	C_39_H_74_O_5_Cl	657.5229	−0.2	9.097
Ceramides (Cer)				
Cer d46:2	C_46_H_89_NO_3_Cl	738.6601	8.7	10.092
Glycerophosphoethanolamines (PE)				
PE O-38:3 or PE P38:2	C_43_H_82_NO_7_PCl	790.5535	1.5	9.961
PE P-36:4 or PE O-36:5	C_41_H_73_NO_7_Cl	722.5116	−1.9	11.627
PE 38:5	C_43_H_75_NO_8_P	764.5244	1.1	10.616
PE 39:5	C_44_H_77_NO_8_P	778.5378	−1.0	11.813
PE 40:5	C_45_H_79_NO_8_P	792.5545	−1.6	−12.876
PE 39:4	C_44_H_80_NO_8_PCl	816.5310	−0.7	11.928
PE 41:4	C_46_H_84_NO_8_PCl	844.562	−1.4	9.197
Cardiolipins (CL)				
CL 74:10	C_83_H_140_O_17_P_2_	735.4814	3.5	10.836
CL 76:9	C_85_H_146_O_17_P_2_	750.5045	2.9	9.173
CL 80:8	C_89_H_156_O_17_P_2_	779.5440	3.3	9.578
Phosphatidic acids (PA)				
LysoPA 19:0	C_22_H_45_O_7_P	451.2859	7.0	10.894
PA 24:2	C_37_H_68_O_8_P	671.4676	2.8	10.733
PA 36:4	C_39_H_68_O_8_P	695.4690	4.7	13.224
PA 37:5	C_40_H_68_O_8_P	707.4674	2.4	9.715
PA 37:2	C_40_H_74_O_8_P	713.5129	0.3	9.836
PA 39:6	C_42_H_70_O_8_P	733.4810	−0.5	12.086
Glycerophosphoinositols (PI)				
LysoPI 15:0	C_24_H_46_O_12_P	557.2729	−0.5	10.667
LysoPI 32:0	C_41_H_80_O_12_P	795.5396	0.4	9.086
PI O-33:2 or PI P-33:1	C_42_H_79_O_12_PCl	841.5011	−1.0	9.257
PI P-35:2	C_44_H_81_O_12_PCl	867.5158	−0.2	9.154
Glycerophosphoglycerols (PG)				
PG 40:8	C_46_H_74_O_10_P	817.5011	−1.7	16.007
PG 44:12	C_50_H_74_O_10_P	865.4996	−3.4	15.160
Glycerophosphoserines (PS)				
PS 36:1	C_42_H_79_NO_10_P	788.5466	2.4	11.903
PS 39:8	C_45_H_72_NO_10_PCl	852.4524	−7.5	17.193

E. Compounds identified by SAM as relatively less abundant in post- compared to pre-chemotherapy samples. Data were extracted from stromal regions of ER samples.				
Metabolites				
Uracil	C_4_H_3_O_2_N_2_	111.0199	−0.9	−11.953
Hypoxanthine	C_5_H_3_ON_4_	135.0317	3.7	−22.577
Glutamic acid	C_5_H_8_NO_4_	146.0449	−6.7	−10.498
Xanthine	C_5_H_3_O_2_N_4_	151.0260	−0.7	−17.540
Inosine	C_10_H_2_N_4_O_5_	267.0735	0.0	−16.967
Fatty acids (FA)				
FA 12:0	C_12_H_23_O_2_	199.1699	−2.5	−5.449
FA 18:1	C_18_H_32_O_2_	281.2478	−2.8	−8.885
FA 20:4	C_20_H_31_O_2_	303.2333	1.1	−8.974
FA 20:2	C_20_H_35_O_2_	307.2638	−1.6	−7.230
FA 20:1	C_20_H_37_O_2_	309.2795	−2.3	−13.741
FA 20:0	C_20_H_39_O_2_	311.2952	−1.3	−9.172
FA 18:1	C_18_H_34_O_2_Cl	317.2245	−2.5	−8.859
FA 22:5	C_22_H_33_O_2_	329.2477	−2.7	−6.274
FA 22:4	C_22_H_35_O_2_	331.2649	2.0	−11.732
FA 22:3	C_22_H_37_O_2_	333.2794	−1.5	−6.074
FA 22:1	C_22_H_41_O_2_	337.3102	−3.0	−9.161
FA 20:4	C_20_H_32_O_2_Cl	339.2088	−2.4	−20.489
FA 22:0	C_22_H_43_O_2_	339.3269	0.0	−6.886
FA 24:5	C_24_H_37_O_2_	357.2807	2.2	−3.911
FA 24:4	C_24_H_39_O_2_	359.2947	−2.5	−7.490
FA 24:1	C_24_H_45_O_2_	365.3427	0.5	−23.955
FA 24:0	C_24_H_47_O_2_	367.3586	1.2	−19.080
FA 24:5	C_24_H_38_O_2_Cl	393.2582	4.1	−9.357
FA 11:1;O	C_11_H_19_O_3_	199.1337	−1.4	−5.492
Monoacylglycerols (MG) and Diacylglycerols (DG)				
MG 18:2	C_21_H_38_O_4_Cl	389.2478	3.3	−9.341
MG 18:0	C_21_H_40_O_4_Cl	391.2620	−0.2	−12.385
MG 20:0	C_23_H_46_O_4_Cl	421.3103	3.1	−23.749
DG 34:2	C_37_H_68_O_5_Cl	627.4758	−0.4	−5.296
DG 34:1	C_37_H_70_O_5_Cl	629.4913	−0.6	−7.707
DG 36:3	C_39_H_70_O_5_Cl	653.4928	1.6	−6.659
DG 36:2	C_39_H_72_O_5_Cl	655.5080	1.0	−11.045
DG 38:4	C_41_H_72_O_5_Cl	679.5089	2.2	−4.582
Ceramides (Cer)				
Cer d32:1	C_32_H_63_NO_3_Cl	544.4519	0.9	−19.867
Cer d34:2	C_34_H_65_NO_3_Cl	570.4655	−1.8	−19.259
Cer d34:0	C_34_H_69_NO_3_Cl	574.4962	−1.6	−19.485
Cer d38:1	C_38_H_75_NO_3_Cl	628.5462	3.3	−6.822
Cer d40:1	C_40_H_79_NO_3_Cl	656.5752	−0.1	−18.481
PI-Cer d27:2	C_33_H_61_NO_11_P	678.3983	−0.7	−4.402
Cer d40:1	C_42_H_79_NO_3_Cl	680.5770	2.4	−11.772
PE-Cer d37:1	C_39_H_79_N_2_O_6_PCl	737.5359	−1.5	−14.636
Cer d46:2	C_46_H_89_NO_3_Cl	738.6601	8.7	−13.640
Glycerophosphoethanolamines (PE)				
PE O-34:2 or PE P-34:1	C_39_H_75_NO_7_P	700.5272	−2.1	−5.874
PE O-36:5 or PE P-36:4	C_41_H_73_NO_7_Cl	722.5116	−1.9	−8.954
PE O-38:6 or PE P-38:5	C_43_H_75_NO_7_P	748.5255	−4.2	−7.729
PE O-38:5 or PE P-36:4	C_43_H_77_NO_7_P	750.5443	0.0	−11.090
PE O-38:3 or PE P-38:2	C_43_H_82_NO_7_PCl	790.5535	1.5	−4.531
PE 37:1	C_42_H_82_NO_8_PCl	794.5485	−0.8	−4.623
Cardiolipins (CL)				
CL 72:7	C_81_H_142_O_17_P_2_	724.4855	−1.6	−6.514
CL 72:6	C_81_H_144_O_17_P_2_	725.4940	0.7	−6.330
Glycerophosphoinositols (PI)				
PI 34:2	C_43_H_78_O_13_P	833.5166	−2.4	−4.807
PI 34:1	C_43_H_80_O_13_P	835.5342	0.0	−12.021
PI 36:4	C_45_H_78_O_13_P	857.5163	−2.7	−6.558
PI 36:2	C_45_H_82_O_13_P	861.5486	−1.5	−6.045
PI 36:1	C_45_H_84_O_13_P	863.5643	−1.4	−14.574
PI 38:4	C_47_H_82_O_13_P	885.5483	−1.8	−9.892
PI 38:3	C_47_H_84_O_13_P	887.5629	−2.9	−9.073
PI 40:5	C_49_H_84_O_13_P	911.5638	−1.9	−6.344
PI 40:4	C_49_H_86_O_13_P	913.5793	−2.1	−7.552
Glycerophosphoglycerols (PG)				
PG 34:1	C_40_H_76_O_10_P	747.5160	−2.9	−12.887
PG 36:1	C_42_H_80_O_10_P	775.5507	1.6	−15.975
Glycerophosphoserines (PS)				
PS O-36:2 or PS P-36:1	C_42_H_79_NO_9_P	772.5490	−1.0	−6.308
PS 38:4	C_44_H_77_NO_10_P	810.5296	0.7	−9.858
PS 40:6	C_46_H_77_NO_10_P	834.5271	−2.4	−14.218
PS 40:5	C_46_H_79_NO_10_P	836.5406	−4.9	−16.751
PS 40:4	C_46_H_81_NO_10_P	838.5644	4.8	−12.498
PS 42:6	C_48_H_82_NO_10_P	862.5556	−5.5	−3.969

F. Compounds identified by SAM as relatively more abundant in post- compared to pre-chemotherapy samples. Data were extracted from stromal regions of ER samples.				
Metabolites				
Valeric acid	C_5_H_9_O_2_	101.061	1.9	5.427
Fumaric acid	C_4_H_3_O_4_	115.0035	−1.6	13.889
Taurine	C_2_H_6_NO_3_S	124.0073	−0.7	13.426
Glutarate semialdehyde	C_5_H_7_O_3_	115.0399	−1.5	5.951
Succinic acid	C_4_H_5_O_4_	117.0195	1.4	8.33
Pyroglutamic acid	C_5_H_6_NO_3_	128.0354	0.6	1.355
Aspartic acid	C_4_H_6_NO_4_	132.0304	1.3	3.233
Malic acid	C_4_H_5_O_5_	133.0141	−1.1	12.019
Hydroxyglutaric acid	C_5_H_7_O_5_	147.0297	−1.3	15.204
Gluconic acid or Galactonic acid	C_6_H_11_O_7_	195.051	0.0	8.924
Hexose	C_6_H_12_O_6_Cl	215.0324	−1.8	25.277
Asp-His	C_10_H_13_N_4_O_5_	269.0883	−1.8	17.671
Fatty acids (FA)				
FA 9:0	C_9_H_17_O_2_	157.1232	−1.3	9.331
FA 11:7	C_11_H_7_O_2_	171.0454	1.2	10.622
FA 10:0	C_10_H_19_O_2_	171.1387	−2.3	5.978
FA 11:0	C_11_H_21_O_2_	185.1548	0.5	0.723
FA 13:3	C_13_H_19_O_2_	207.1383	−3.9	2.271
FA 14:0	C_14_H_27_O_2_	227.201	−3.1	3.873
FA 15:0	C_15_H_29_O_2_	241.2167	−2.5	8.34
FA 16:1	C_16_H_29_O_2_	253.2166	−2.8	2.816
FA 16:0	C_16_H_31_O_2_	255.2322	−3.1	5.791
FA 17:1	C_17_H_31_O_2_	267.2324	−2.2	3.69
FA 17:0	C_17_H_33_O_2_	269.2478	−3.0	3.318
FA 16:0;O	C_16_H_31_O_3_	271.2285	2.2	1.324
FA 18:3	C_18_H_29_O_2_	277.2165	−2.9	7.989
FA 18:2	C_18_H_31_O_2_	279.2322	−2.9	1.013
FA 18:0	C_18_H_35_O_2_	283.2634	−3.2	2.611
FA 18:1;O	C_18_H_33_O_3_	297.2428	−2.4	5.597
FA 20:5	C_20_H_29_O_2_	301.2168	−1.7	4.08
Diacylglycerols (DG)				
DG 43:6	C_46_H_78_O_5_Cl	745.5558	2.0	0.224
Glycerophosphoethanolamines (PE)				
PE 34:1	C_39_H_75_NO_8_P	716.519	−6.4	1.184
PE 38:5	C_43_H_75_NO_8_P	764.5216	−2.6	1.99
Phosphatidic acids (PA)				
PA 36:1	C_39_H_74_O_8_P	701.5102	−3.5	6.809
PA 38:0	C_41_H_81_O_8_PCl	767.5396	4.3	1.719
PA 43:7	C_46_H_76_O_8_P	787.5278	−0.6	4.834
PA 45:7	C_48_H_80_O_8_P	815.5575	−2.6	0.352
Glycerophosphoglycerols				
PG 34:2	C_40_H_74_O_10_P	745.4978	−6.3	1.953
PG 36:4	C_42_H_74_O_10_P	769.4995	−3.9	5.211
PG 38:4	C_44_H_78_O_10_P	797.5315	−2.9	3.107
PG 40:8	C_46_H_74_O_10_P	817.5	−3.1	7.942
PG 40:6	C_46_H_78_O_10_P	821.5299	−4.7	5.911
PG 40:5	C_46_H_81_O_10_PCl	859.5275	1.6	3.737
Glycerophosphoserines (PS)				
PS 34:1	C_40_H_75_NO_10_P	760.5111	−3.02	8.04
PS 36:2	C_42_H_77_NO_10_P	786.5262	−3.69	3.504
PS 40:2	C_46_H_85_NO_10_P	842.5904	−1.54	2.065

G. Compounds identified by SAM as relatively more abundant in post- compared to pre-chemotherapy samples. Data were extracted from stromal regions of PR samples.				
Metabolites				
Fumaric acid	C_4_H_3_O_4_	115.0035	−1.6	1.322
Taurine	C_2_H_6_NO_3_S	124.0073	−0.7	21.157
Pyroglutamic acid	C_5_H_6_NO_3_	128.0354	0.6	3.641
Aspartic acid	C_4_H_6_NO_4_	132.0304	1.3	7.146
Aconitic acid	C_6_H_5_O_6_	173.0091	−0.4	3.392
Gluconic acid or Galactonic acid	C_6_H_11_O_7_	195.051	−5.3	12.54
Hexose	C_6_H_12_O_6_Cl	215.0324	−1.8	19.122
Asp-His	C_10_H_13_N_4_O_5_	269.0883	−1.8	20.157
Uridine	C_9_H_12_N_2_O_6_Cl	279.0385	−1.4	1.824
Fatty acids (FA)				
FA 9:0	C_9_H_17_O_2_	157.1235	0.6	12.071
FA 10:0	C_10_H_19_O_2_	171.1387	−2.3	11.921
FA 11:0	C_11_H_21_O_2_	185.1548	0.5	2.680
FA 9:2	C_9_H_14_O_2_Cl	189.0684	−2.1	1.391
FA 12:2	C_12_H_19_O_2_	195.1387	−2.1	1.59
FA 13:3	C_13_H_19_O_2_	207.1383	−3.9	3.376
FA(14:0)	C14H27O2	227.201	−3.1	7.26
FA 15:4	C_15_H_21_O_2_	233.1547	0.0	2.254
FA 14:4;O	C_14_H_19_O_3_	235.1338	−0.9	2.254
FA 15:0	C_20_H_37_O_2_	241.2167	−2.3	5.397
FA 16:0	C_20_H_39_O_2_	255.2322	−1.3	9.617
FA 19:0	C_19_H_37_O_2_	297.2792	−2.4	0.853
FA 20:0	C_20_H_39_O_2_	311.2948	−2.6	FA 20:0
FA 24:4	C_24_H_39_O_2_	359.2945	−3.1	1.802
Ceramides (Cer)				
Cer d34:1	C_34_H_67_NO_3_Cl	572.4797	−3.1	5.171
Cer d42:2	C_42_H_81_NO_3_Cl	682.5891	−2.9	4.551
Glycerophosphoethanolamines (PE)				
PE 34:2	C_39_H_73_NO_8_P	714.5125	6.4	3.216
PE O-36:5 or PE P-36:4	C_41_H_73_NO_7_P	722.5104	−3.6	11.536
PE 36:2	C_41_H_77_NO_8_P	742.5368	−3.2	2.06
PE 36:1	C_41_H_79_NO_8_P	744.5519	−4.0	4.241
PE 37:5	C_42_H_73_NO_8_P	750.5069	−1.3	5.344
PE 37:4	C_42_H_75_NO_8_P	752.5268	4.3	5.244
PE 38:5	C_43_H_75_NO_8_P	764.5216	−2.6	2.862
PE 38:4	C_43_H_77_NO_8_P	766.5366	−3.4	0.425
PE 39:5	C_44_H_77_NO_8_P	778.5363	−3.7	0.689
Cardiolipins (CL)				
CL 72:8	C_81_H_141_O_17_P_2_	723.4766	3.0	13.016
CL 72:7	C_81_H_142_O_17_P_2_	724.4855	1.6	6.596
Phosphatidic acids (PA)				
PA 34:1	C_37_H_70_O_8_P	673.4776	−5.6	0.232
PA 36:2	C_39_H_72_O_8_P	699.4942	−4.0	7.63
PA 36:1	C_39_H_74_O_8_P	701.5102	−3.6	6.263
PA 35:0	C_38_H_75_O_8_PCl	725.4895	0.1	1.202
PA 38:3	C_41_H_74_O_8_P	725.5129	0.3	6.552
PA 41:6	C_44_H_74_O_8_P	761.5141	1.8	2.944
PA 38:0	C_41_H_81_O_8_PCl	767.5396	4.3	2.237
PA 43:7	C_46_H_76_O_8_P	787.5278	−0.6	13.418
PA 43:6	C_46_H_78_O_8_P	789.5452	1.5	14.853
PA 45:7	C_48_H_80_O_8_P	815.5575	−2.6	7.342
PA 45:6	C_48_H_82_O_8_P	817.5785	3.9	6.619
Glycerophosphoinositols (PI)				
PI 34:1	C_43_H_80_O_13_P	835.5303	−4.7	1.149
PI 36:4	C_45_H_78_O_13_P	857.5155	−3.6	10.541
PI 36:2	C_45_H_82_O_13_P	861.5486	−1.5	0.449
PI 38:5	C_47_H_80_O_13_P	883.5314	−3.2	7.812
PI 38:4	C_47_H_82_O_13_P	885.5465	−3.8	2.522
Glycerophosphoglycerols (PG)				
PG 34:2	C_40_H_74_O_10_P	745.4978	−6.3	10.578
PG 34:1	C_40_H_76_O_10_P	747.5155	−3.6	9.167
PG 36:4	C_42_H_74_O_10_P	769.4995	−3.9	11.536
PG 36:3	C_42_H_76_O_10_P	771.5142	−5.2	9.769
PG 36:2	C_42_H_78_O_10_P	773.5301	−4.8	17.46
PG 38:6	C_44_H_74_O_10_P	793.5004	−2.6	1.432
PG 38:4	C_44_H_78_O_10_P	797.5315	−2.9	5.684
PG 40:8	C_46_H_74_O_10_P	817.5	−3.1	0.755
PG 40:6	C_46_H_78_O_10_P	821.5299	−4.7	6.352
PG 40:5	C_46_H_81_O_10_PCl	859.5275	1.6	9.626
PG 42:5	C_48_H_85_O_10_PCl	887.5562	−1.4	0.826
Glycerophosphoserines (PS)				
PS 34:1	C_40_H_75_NO_10_P	760.5111	−3.0	15.483
PS 35:3	C_41_H_73_NO_10_P	770.5023	5.8	8.446
PS O-36:2 or PS P-36:1	C_42_H_79_NO_9_P	772.5479	−2.5	5.33
PS 36:2	C_42_H_77_NO_10_P	786.5262	−3.7	10.854
PS 36:1	C_42_H_79_NO_10_P	788.5419	−3.6	21.855
PS O-38:5 or PS P-38:4	C_44_H_77_NO_9_P	794.5341	−0.1	2.789
PS 37:4	C_43_H_75_NO_10_P	796.5157	2.9	2.768
PS 38:3	C_44_H_79_NO_10_P	812.5409	−4.7	9.888
PS 38:1	C_44_H_83_NO_10_P	816.5726	−4.2	4.956
PS 40:2	C_46_H_85_NO_10_P	842.5904	−1.5	8.10

H. Compounds identified by SAM as relatively less abundant in post- compared to pre-chemotherapy samples. Data were extracted from stromal regions of PR samples.				
Metabolites				
Valeric acid	C_5_H_9_O_2_	101.061	1.9	−2.427
Glutarate semialdehyde	C_5_H_7_O_3_	115.0399	−1.5	−4.447
Succinic acid	C_4_H_5_O_4_	117.0195	1.4	−12.384
Hydroxyvaleric acid	C_5_H_9_O_3_	117.0559	1.7	−7.855
Malic acid	C_4_H_5_O_5_	133.0141	−1.1	−8.58
Glutamic acid	C_5_H_8_NO_4_	146.0457	−1.2	−4.628
Hydroxyglutaric acid	C_5_H_7_O_5_	147.0297	−1.3	−12.647
Fatty acids (FA)				
FA 9:1; O	C_9_H_15_O_4_	171.1023	−1.8	−20.144
FA 11:1;O	C_11_H_19_O_3_	199.1337	−1.4	−13.216
FA 12:0	C_12_H_22_O_2_	199.1699	−2.3	−10.301
FA 16:1	C_16_H_29_O_2_	253.2166	−2.8	−12.592
FA 17:1	C_17_H_31_O_2_	267.2324	−2.2	−8.513
FA 16:0;O	C_16_H_31_O_3_	271.2285	2.2	−8.934
FA 18:3	C_18_H_29_O_2_	277.2165	−2.9	−7.175
FA 18:2	C_18_H_31_O_2_	279.2322	−2.9	−13.189
FA 18:1	C_18_H_33_O_2_	281.2478	−2.8	−11.404
FA 18:0	C_18_H_35_O_2_	283.2634	−3.2	−3.155
FA 20:5	C_20_H_29_O_2_	301.2168	−1.7	−12.692
FA 20:4	C_20_H_31_O_2_	303.232	−3.3	−5.464
FA 20:3	C_20_H_33_O_2_	305.2476	−3.3	−22.548
FA 20:2	C_20_H_35_O_2_	307.2633	−3.3	−20.444
FA 20:1	C_20_H_37_O_2_	309.2789	−3.2	−15.464
FA 22:6	C_22_H_31_O_2_	327.2321	−2.8	−9.732
FA 22:5	C_22_H_33_O_2_	329.2477	−2.7	−9.96
FA 22:4	C_22_H_35_O_2_	331.2632	−3.3	−19.011
FA 22:3	C_22_H_37_O_2_	333.2787	−3.6	−16.218
FA 22:1	C_22_H_41_O_2_	337.3104	−2.4	−16.04
FA 20:4	C_20_H_32_O_2_Cl	339.2086	−2.9	−3.325
FA 22:0	C_22_H_43_O_2_	339.3267	−0.6	−12.848
FA 24:5	C_24_H_37_O_2_	357.2785	−3.9	−6.204
FA 24:2	C_24_H_43_O_2_	363.3257	−3.3	−17.31
FA 24:1	C_24_H_45_O_2_	365.3417	−2.2	−23.635
FA 24:0	C_24_H_47_O_2_	367.3574	−2.2	−22.636
FA 24:6	C_24_H_36_O_2_Cl	391.2439	7.7	−2.463
FA 26:2	C_26_H_47_O_2_	391.3564	−4.6	−12.891
FA 24:5	C_24_H_38_O_2_Cl	393.261	11.2	−4.285
FA 26:1	C_26_H_49_O_2_	393.3721	−4.3	−15.643
FA 26:0	C_26_H_51_O_2_	395.3881	−3.5	−10.224
Monoacylglycerols (MG) and Diacylglycerols (DG)				
MG 16:0	C_19_H_38_O_4_Cl	365.2458	−1.6	−18.923
MG 18:0	C_21_H_40_O_4_Cl	391.2620	−0.2	−12.385
MG 20:0	C_23_H_46_O_4_Cl	421.3103	3.1	−23.749
MG 29:0	C_32_H_63_O_4_	511.47	−6.3	−3.033
DG P-31:1	C_34_H_63_O_4_	535.4717	−2.8	−11.621
DG O-31:1 or DG P-31:0	C_34_H_65_O_4_	537.4878	−1.9	−14.271
DG P-33:2	C_36_H_65_O_4_	561.4877	−2.0	−14.806
DG O-33:2 or DG P-33:1	C_36_H_67_O_4_	563.5018	−4.8	−7.763
DG O-33:1 or DG P-33:0	C_36_H_69_O_4_	565.5182	−3.4	−16.294
Glycerophosphoethanolamines (PE)				
PE 39:6	C_44_H_75_NO_8_P	776.5211	−3.2	−3.52
PE O-42:6 or PE P-42:5	C_47_H_84_NO_7_PCl	840.5686	0.7	−4.849
Phosphatidic acids (PA)				
PA 37:1	C_40_H_76_O_8_P	715.5262	−2.9	−2.123
PA 38:2	C_41_H_76_O_8_P	727.526	−3.2	−14.876
Glycerophosphoinositols (PI)				
PI 40:4	C_49_H_86_O_13_P	913.5793	−2.1	−7.552
PI 39:5	C_48_H_83_O_13_PCl	933.5323	6.2	−2.112
Glycerophosphoserines (PS)				
PS 38:4	C_44_H_77_NO_10_P	810.5291	−1.0	−2.127
PS 40:6	C_46_H_77_NO_10_P	834.5291	0.1	−12.505
PS 40:4	C_46_H_81_NO_10_P	838.5604	0.7	−12.192

**Fig. 3:**
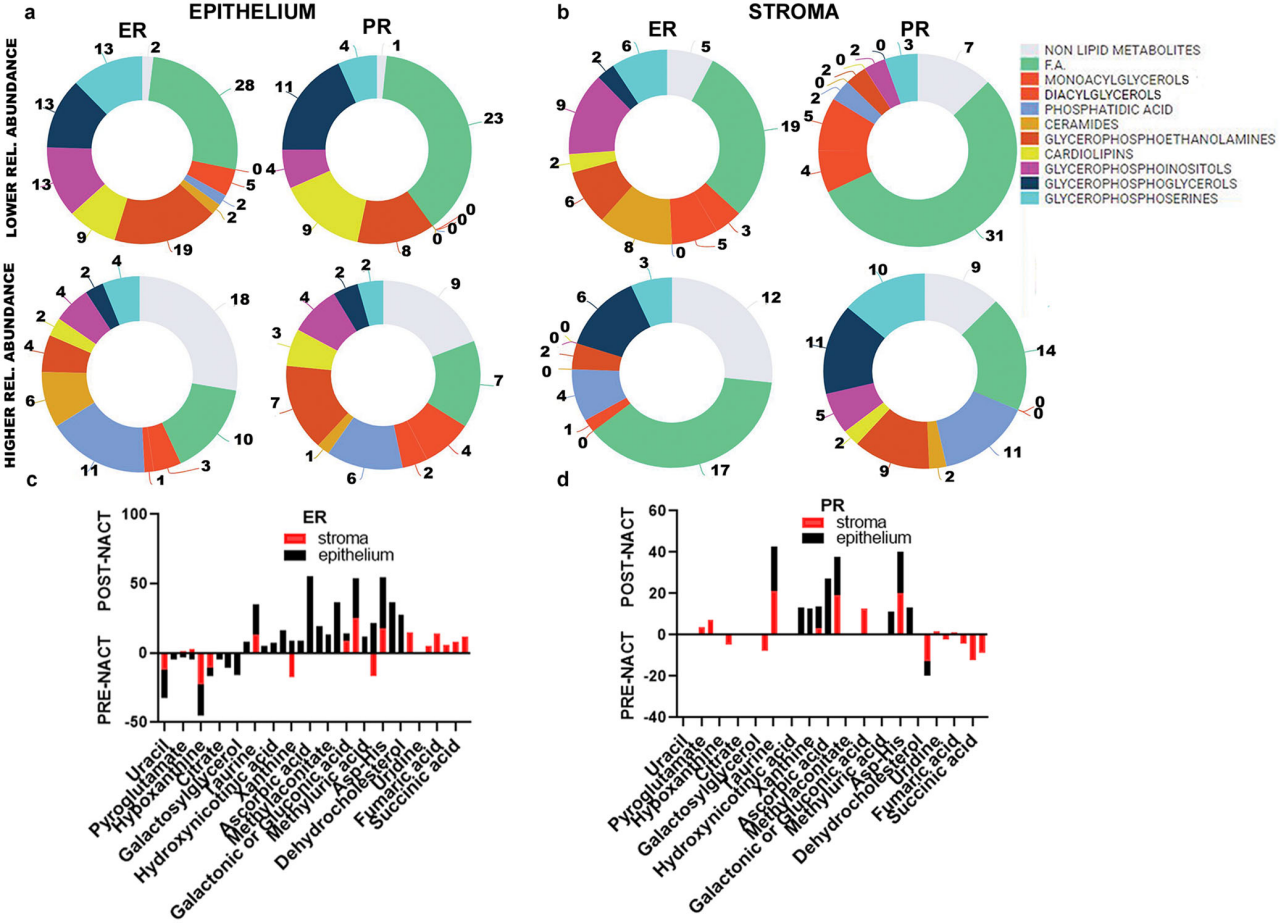
Comparative analysis of high-grade serous ovarian cancer (HGSC) samples obtained post neoadjuvant chemotherapy (NACT) versus samples obtained prior neoadjuvant chemotherapy (NACT), based on excellent (ER) or poor (PR) response: lipids and small metabolites. **a**, **b** Pie charts summarize the number of lipids in each lipid class, with higher and lower relative abundance in the epithelium (**a**) and stroma (**b**) of ER and PR post- versus pre-chemotherapy tissues identified using DESI-MS analysis. **c**, **d** Histograms representing the relative abundances of small metabolites in the epithelium and stroma of ER (**c**) and PR (**d**) tumors of post- versus pre-NACT tissues identified using DESI-MS. The data shown in the pie charts were obtained from DESI-MS analysis of tumor tissue sections from pre-chemotherapy samples from 52 patients (30 ER and 22 PR) and post-chemotherapy samples from 37 patients (20 ER and 17 PR). ER excellent responders, PR poor responders.

**Fig. 4:**
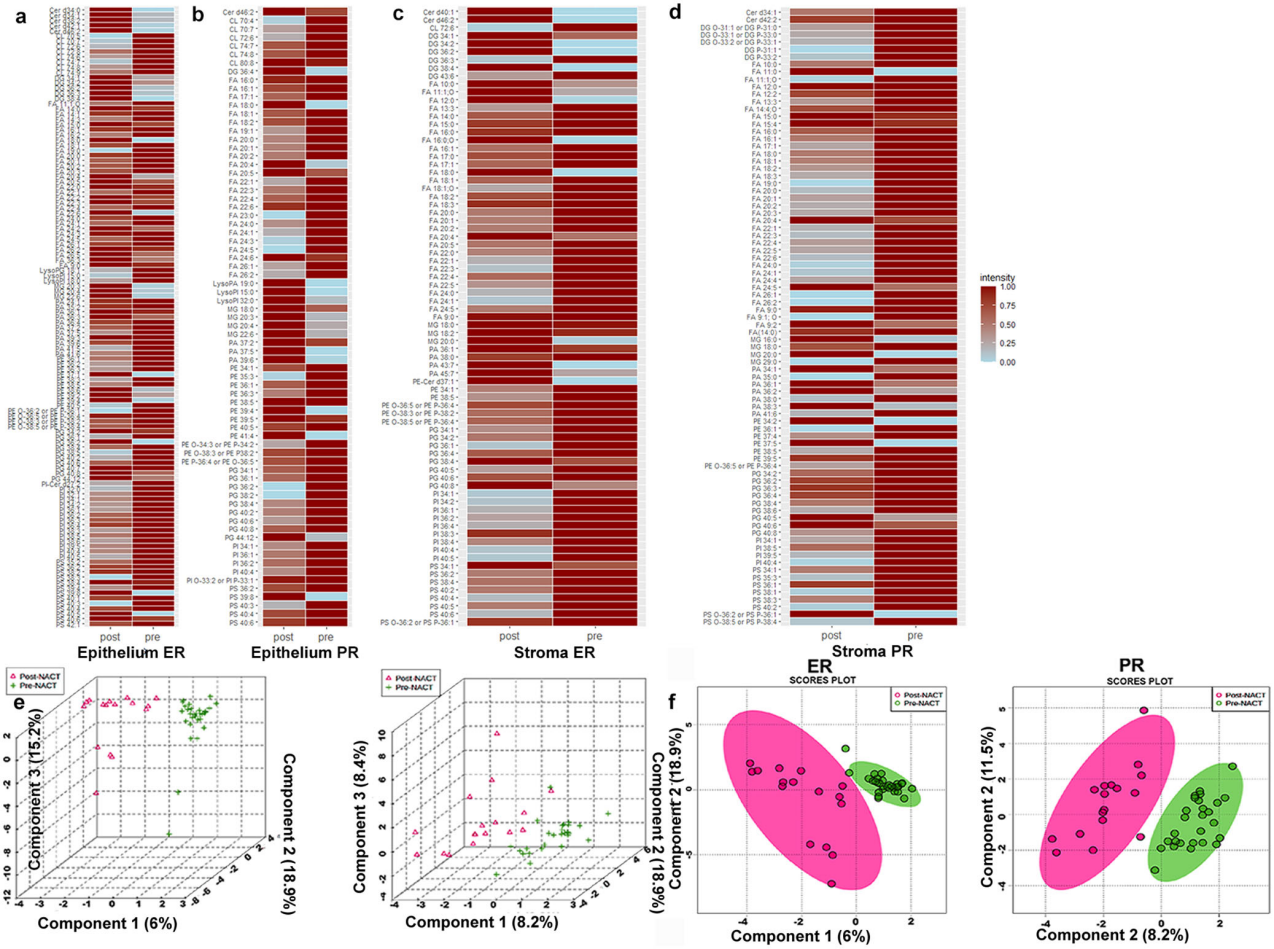
Comparative analysis of high-grade serous ovarian cancer (HGSC) samples obtained post neoadjuvant chemotherapy (NACT) versus samples obtained prior neoadjuvant chemotherapy (NACT), based on excellent (ER) or poor (PR) response: lipids heatmaps and sPLS-DA plots. **a**, **b** Heatmaps representing the relative abundances of lipids in the epithelial areas of ER tumors (**a**); epithelial areas of PR tumors (**b**); stromal areas of ER tumors (**c**); and stromal areas of PR tumors (**d**). **e**, **f** Plots for sparse partial least squares discriminant analysis (sPLS-DA) in tri-dimensional (**e**) and bi-dimensional (**f**) settings. post post-NACT; pre pre-NACT; ER excellent responders, PR poor responders. For heatmap abbreviations see Tables 1 and 3. The data shown in the heatmaps were obtained from DESI-MS analysis of tumor tissue sections from pre-chemotherapy samples from 52 patients (30 ER and 22 PR) and post-chemotherapy samples from 37 patients (20 ER and 17 PR).

In the epithelial regions, fatty acid species had a lower relative abundance in post-NACT tissues for both ER and PR tumors, while the relative abundance of phosphatidic acids was higher in the epithelial areas of post-NACT tissues for both ER and PR tumors. Indeed, phosphatidic acids were detected at higher relative abundances after chemotherapy in both ER and PR tumors (Fig. 3a). Interestingly, ceramides were highly abundant in the epithelial areas of post-NACT ER tumors. In the stromal areas, glycerophosphoinositol species were highly abundant in post-NACT tissues in both ER and PR tumors, while glycerophosphoserine and glycerophosphoethanolamine species were particularly abundant in post-NACT PR tumors. Heatmaps show distinct distributions of the normalized ion intensities of lipid species with different abundances in the epithelial areas (Fig. 4a, b) and stromal areas (Fig. 4c, d) in both pre- and post-NACT tissues from ER and PR tumors.

To investigate if the metabolites that had higher or lower relative abundances in post- versus pre-NACT tissues of ER and PR tumors were related to specific metabolic pathways, we analyzed the non-lipid metabolites identified with DESI-MS. The histograms in Fig. 3c, d represent the higher and lower relative abundances of metabolites in ER and PR tumors of post- versus pre-NACT samples. The epithelial regions of post-NACT samples from ER tumors showed lower relative abundances of uracil, fumarate, pyroglutamate, aspartate, hypoxanthine, glutamic acid, citrate, and galactosylglycerol (Table 3A), which are involved in the “urea cycle,” “phenylalanine and tyrosine metabolism,” and “nucleotide metabolism” pathways (FDR-adjusted *p* < 0.01) (Supplementary Table 6A, B), whereas several metabolites, including hydroxy valeric acid, taurine, leucinic acid, hydroxyniconitic acid, and glutamine (Table 3B), which had higher relative abundances in the epithelial regions of ER tumors, are involved in the “TP53-regulated metabolic genes” and “metabolism of nucleotides” pathways (FDR-adjusted *p* < 0.05) (Supplementary Table 7A, B). Metabolites with lower abundances in the post-NACT stromal regions of ER tumors, including uracil, hypoxanthine, glutamic acid, xanthine, and inosine (Table 3E), were associated with the pathways “metabolism of nucleotides” (FDR-adjusted < 0.01), “nucleotide salvage” (FDR-adjusted *p* < 0.01), and “purine catabolism” (FDR-adjusted *p* < 0.01) (Supplementary Table 8A, B), while metabolites such as valeric acid, fumaric acid, taurine, glutarate semialdeyde, and succininc acid (Table 3F), which had high relative abundances in the stromal regions, were mostly involved in the “urea cycle” and “citric acid cycle” pathways (FDR-adjusted *p* < 0.05) (Supplementary Table 9A, B).

In the epithelial regions of post-NACT PR tumors, taurine, glutamine, xanthine, aconitic acid, ascorbic acid, hexose, asp-his, inosine, and glutathione (Table 3D) had higher relative abundances and were mostly associated with the “metabolism of nucleotides” and “TP53 regulated-metabolic genes” pathways (FDR-adjusted *p* < 0.05) (Supplementary Table 10A, B, Table 2B). In the stromal regions of post-NACT PR tumors, the metabolites with higher abundances included fumaric acid, taurine, pyroglutamic acid, aspartic acid, and aconitic acid (Table 3G), which were related to the “aspartate and asparagine metabolism,” “phenylalanine and tyrosine metabolism,” and “nucleotide biosynthesis” and “urea cycle” pathways (Supplementary Table 11A, B, Table 2B), whereas less abundant metabolites, such as valeric acid, glutarate semialdehyde, succinic acid, hydroxyvaleric acid, malic acid, glutamic acid, and hydroxyglutaric acid, were associated with the “GABA degradation and synthesis” pathway (Supplementary Table 12A, B, Table 2B). Table 2B summarizes the deregulated pathways in post-NACT ER and PR tissues compared with pre-treatment tissues.

Sparse partial least squares discriminant analysis (sPLS-DA) of the data acquired from pre- and post-NACT samples in both ER and PR tumors showed a clear separation of the two tissue groups in the tri- or bi-dimensional score plots (Fig. 4e, f). These results indicate that different adaptive metabolic changes occur in tissues based on response to NACT.

### Quantitative proteomic and phosphoproteomic analyses of pre-chemotherapy samples from ER and PR tumors

**Fig. 5:**
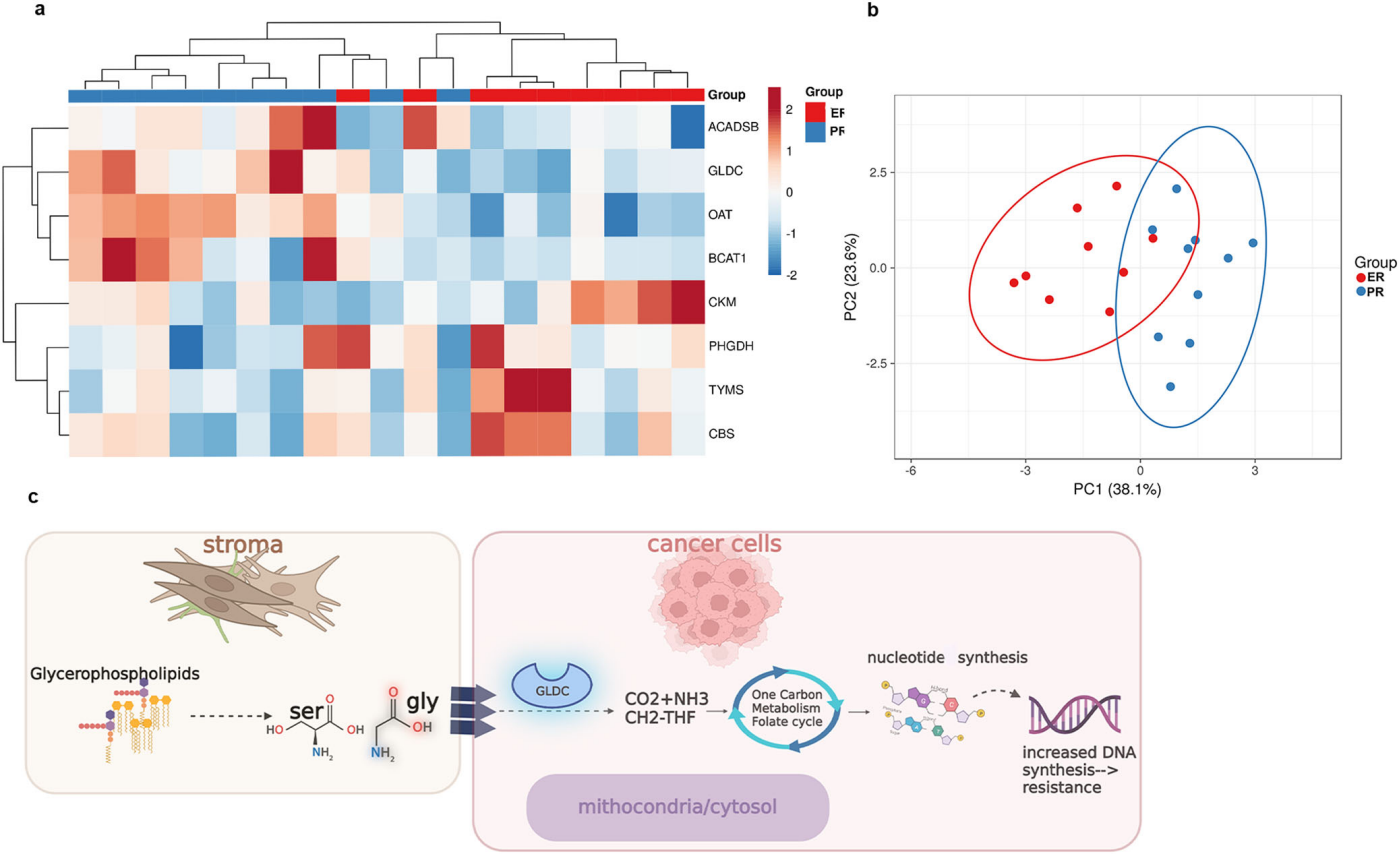
Proteomic analysis of high-grade serous ovarian cancer (HGSC) samples obtained prior to neoadjuvant chemotherapy (NACT) based on excellent (ER) or poor (PR) response. **a** Unsupervised clustering of protein expression in ER and PR tissues. **b** PCA plot of the same features by case. **c** Proposed mechanism for the metabolic interactions between stroma and cancer cells. PCA principal component analysis, Ser serine, gly glycine, GLDC glycine decarboxylase. The results from the proteomics analyses shown in this figure were obtained from analysis performed on tumor tissue sections from pre-chemotherapy samples from 15 patients (7 ER and 8 PR). Differential analyses of global proteome or transcriptome matrixes were performed using LIMMA.

To identify differentially expressed enzymes and phosphoproteins in PR versus ER tumors, we generated global proteomic and phosphoproteomic data for whole-tumor equivalent collections of pre-chemotherapy samples, as described previously^13^. A total of 7148 proteins and more than 1075 phosphosites were co-quantified across cases (Supplemental Tables 13–19). We selected proteins and phosphosites with significantly different expressions based on clinical response and metabolic pathways previously identified by DESI-MS. Pathways with the highest number of proteins quantified included the “metabolism of amino acids and derivatives,” “metabolism of nucleotides,” and “respiratory electron transport and related” pathways. Differential analysis revealed that most proteins and phosphosites that differed significantly (LIMMA *p* < 0.05, ±1.5-fold change) between PR and ER cases mapped to the “metabolism of amino acids and derivatives” and “metabolism of nucleotides” pathways (*z*-score = 0.728 *p*-value 1.69E-13, derived from Ingenuity Pathway Analysis) (Fig. 5a). Principal component analysis of these proteins by case revealed a distinct separation of the pre-NACT PR and ER tumors (Fig. 5b).

The quantitative proteomic analysis confirmed that phosphosites, which are related to the metabolism of nucleotides and particular pyrimidines, were significantly elevated in the PR tumors, which was concordant with the DESI-MS data (Supplementary Tables 14, 16, and 18). Interestingly, uridine (which DESI-MS revealed to have a high relative abundance in the tumor epithelium of pre-NACT PR samples), which serves as a substrate for cytidine 5-prime triphosphate synthetase (CTPS1), was highly abundant in pre-NACT PR samples (logFC 0.97, LIMMA *p* = 0.008) (Supplementary Table 18). CTPS1 catalyzes the conversion of uridine triphosphate into cytidine triphosphate and regulates intracellular rates of RNA, DNA, and phospholipid synthesis^14,15^; its phosphorylation is inhibitory, which could explain the higher abundance of uridine. Lastly, the increase of ornithine aminotransferase in PR tumors, measured by proteomic analysis, correlated well with the high relative abundances of molecules detected by DESI-MS that are associated with aspartate and asparagine pathways in the stroma (e.g., fumaric acid, taurine, aspartic acid, and glucose)^16^.

For the four enzymes that were upregulated in the pre-NACT PR samples (Supplementary Tables 13, 15, and 17), we analyzed the correlation between mRNA expression and progression-free survival (PFS) in patients with HGSC from publicly available databases (Supplementary Fig. 2). Interestingly, higher expression of glycine decarboxylase (GLDC) was positively correlated with worse prognosis (HR 1.16, CI 1.00–1.35, *p* = 0.046) (KMplotter.com). We also investigated the expression of GLDC mRNA in several organs (gTEX.org) and the expression of its protein (Protein Atlas) and mRNA (TCGA) in several cancer types (Supplementary Fig. 3). GLDC mRNA expression is low in normal ovarian and fallopian tube samples, but it is detected at high protein levels in ovarian cancer. A possible mechanism explaining the metabolic interaction between stroma and cancer cells involving GLDC activity is proposed in Fig. 5c. We further explored the possible impact of GLDC expression on the response of ovarian cancer cells to chemotherapy. After consulting the public database DepMap Public 22Q4 at Cancer Cell Line Encyclopedia (CCLE), we identified the ovarian cancer cell lines with higher GLDC expression based on average [log2(TPM + 1)] expression (Supplementary Fig. 4A). IGROV1 cells are among the top two ovarian cancer cell lines listed in CCLE with the highest GLDC expression. We then measured GLDC mRNA expression in IGROV1 and other ovarian cancer cell lines and in the HIO 180 non-transformed epithelial ovarian cancer cells to confirm that IGROV1 had the highest GLDC expression among them (Supplementary Fig. 4B). Next, the level of GLDC mRNA in IGROV1 was evaluated after transient transfection with three silencing RNAs (siRNA29, siRNA35, siRNA03). SiRNA35 resulted in the lowest mRNA levels of GLDC (Supplementary Fig. 4C). Cell viability was then evaluated in IGROV1 cells transfected with siRNA35 or siRNA control and treated for 72 h with carboplatin. The IC50 level of carboplatin in IGROV1 cells transfected with siRNA35 was 3.3 times lower than the IC50 in IGROV1 cells transfected with siRNA control, 3.7 µM versus 12.3 µM (*P* = 0.0003, hypothesis test, alpha 0.05) (Supplementary Fig. 4D).

## Discussion

Relapse represents a major challenge in the treatment of patients with ovarian cancer, and studying the molecular changes related to therapy response is essential to identifying novel actionable targets. Nutrient availability inside the TME and paracrine communication influence the metabolic reprogramming of cancer cells, generating a complex metabolic profile^17^. In particular, reprogramming of nucleotide metabolism towards increased levels of nucleotide precursors and nucleotides has been found in recurrent tumor cells, including several cancer models^14,18^. Also, the metabolic dependency of ovarian cancer cells on neighboring stroma cells plays an important role in fueling tumor cell growth^15^. Many studies have investigated the metabolic interactions between the TME and cancer cells in inducing a permissive environment for tumor growth. The increased use of glucose and glutamine by cancer cells results in lactate accumulation, which decreases the activation of dendritic and T cells while stimulating the polarization of macrophages towards an M2-like phenotype^16,19^. Moreover, lactate stimulates angiogenesis^20^ and promotes acidification of the TME. This stimulates the proteolytic activity of metalloproteinases^21^, which in turn enhances extracellular matrix degradation and tumor invasion. Therefore, while much is known about how the metabolic interactions between stroma and cancer cells induce tumor cell proliferation and invasion, less is known about how these interactions promote resistance to chemotherapy. A few recent studies identified some important metabolic vulnerabilities in ovarian cancers that can be exploited to increase treatment response; these vulnerabilities include glutamine and serine metabolism^22,23^. To thoroughly study the metabolic heterogeneity of the HGSC TME in relation to therapy response, we performed a comparative analysis of metabolic species (nucleotides, proteins, sugars, and lipids) present in pre- and post-NACT tissues. We used DESI-MS imaging to obtain spatially resolved metabolomic information about the epithelial and stromal regions, and we used global proteomics and phosphoproteomics to corroborate the metabolic findings.

The use of highly clinically annotated samples, as presented here, is important for obtaining reliable results. When comparing the relative abundances of metabolites detected within the stromal regions of post- versus pre-NACT tissues, we found that PR tumors had higher abundances of fumaric acid, taurine, and aspartic acid, which are related to aspartate and asparagine metabolism. Interestingly, proliferating cells with impending glutamine depletion often adapt by utilizing asparagine, which is structurally similar to glutamine and can be used to fuel the TCA cycle, as an energy source^24,25^. It is plausible that an elevated demand for glutathione to counteract chemotherapy-induced cell damage may result in the depletion of glutamine in proliferating cells; in this scenario, stromal cells may support cancer cells by fueling them with asparagine to sustain their proliferation in tissues that respond less to chemotherapy. Moreover, the stroma of post-chemotherapy PR tissues showed elevated abundances of glycerophosphoserines (PS 36:1, PS 34:1, PS 36:2), which might be substrates for serine synthesis^26^. We believe that the increased nucleotide metabolism in PR cancer cells might be sustained by an increased influx of glycine and serine from the tumor stroma, leading to the increased activity of GLDC. GLDC fuels one-carbon metabolism via glycine breakdown to form CO_2_, NH_3_, and 5,10-methylene-tetrahydrofolate (CH2-THF)^27^; in particular, CH2-THF has been shown to be crucial for nucleotide synthesis^28,29^. As shown in other cancer types, GLDC may sustain nucleotide synthesis during cell proliferation in HGSC tumorigenesis^30,31^. However, an increase in nucleotide synthesis does not necessarily translate into a higher sensitivity to carboplatin-based chemotherapy (the main type of neoadjuvant chemotherapy used for ovarian cancer in our cohort and in general), since carboplatin does not show cell-cycle specificity^32^.

Our findings suggest several strategies to overcome chemotherapy resistance in HGSC, including targeting glycerophosphoserine and interfering with the metabolism of aspartate and glycine. While the antibody-based targeting of phosphatidylserine has been shown in pre-clinical studies to overcome resistance to radiation and chemotherapy^33,34^, the targeting of glycerophosphoserine has yet to be investigated. Interfering with aspartate and glycine metabolism could be done by blocking GLDC. GLDC, a mitochondrial enzyme, is part of a complex that oxidatively decarboxylates glycine^35^; high GLDC activity is strongly correlated with high rates of glutaminolysis and the synthesis of acetyl-CoA and fatty acids^36^. Notably, patients with HGSC with elevated GLDC levels have significantly worse PFS^37^, a finding similar to that in patients with non-small cell lung cancer^30^, in whom GLDC inhibition has been investigated in vitro and in vivo^38^. Combined treatment with GLDC inhibitors and platinum-based compounds, a completely novel strategy, might enhance sensitivity to chemotherapy in ovarian cancer. As mentioned above, and based on prior publications, glutaminolysis, and glutamine metabolism are part of the basis of reprogramming ovarian cancer cells towards increased proliferation and invasiveness^39^. Our study provides additional data on how the heterogeneous metabolism inside ovarian cancers might affect response to chemotherapy by promoting glycine-dependent nucleotide synthesis in cancer cells. However, additional work is needed to further test the biological importance of GLDC in ovarian and other cancers.

This study provides evidence that tumors with low sensitivity to NACT are characterized by different metabolic profiles, a finding that can be leveraged to stratify patients for treatment purposes. Additional research is needed to examine the therapeutic efficacy of targeting these differences. The availability of highly annotated tissues from patients undergoing standardized treatment and follow-up makes our results particularly relevant and translatable to the clinic. Future work will focus on the analysis of metabolomic and proteomics/phosphoproteomics profiles within an expanded cohort of tissues from patients with HGSC.

Our subgroup analysis was limited by the small sample size; therefore, a larger cohort and additional validation studies are needed. Tissue segmentation in stroma and epithelium was based on the morphological analysis of H&E-stained slides; additional subclassification of the TME with the identification of vessels, immune cell clusters, and different fibroblast subtypes is needed to further elucidate the metabolic changes in the stroma. Although MS imaging data provide spatially resolved molecular information, they are not quantitative; thus, the pathway analyses based on these data were exploratory in nature. Moreover, it should be noted that the predictive model was built from data extracted from tumor regions annotated by pathologic evaluation within the primary tissue types. As such, the model is limited to these tissue types and needs to be further expanded and validated for use in tissues in which higher degrees of cellular heterogeneity may influence the metabolic profiles.

## Methods

### Patients

A total of 112 frozen samples from 59 patients were collected and analyzed with DESI-MS; these included pre-chemotherapy samples from 52 patients (30 ER and 22 PR) and post-chemotherapy samples from 37 patients (20 ER and 17 PR). Among these, frozen tumor sections were retrieved from 48 patients from the MD Anderson Department of Gynecologic Oncology, 7 patients from the Gynecologic Cancer Translational Research Center of Excellence (GYN-COE) Program, and 4 patients from Washington University, St. Louis, as part of a collaborative study with the University of Iowa and MD Anderson Cancer Center. When available, two pre- or post-chemotherapy samples (one from adnexa and the other from a metastatic site such as the omentum, uterus, or abdominal organs) for each patient were collected and analyzed.

The collection of tissues from patients diagnosed and treated at the MD Anderson Cancer Center followed a specific algorithm: patients with suspected advanced primary ovarian cancer underwent surgical laparoscopy, during which their metastatic burden was assigned a modified Fagotti score^40,41^ and their tissues obtained and stored. Following laparoscopy, patients with a predictive index value < 8 underwent primary reductive surgery, and patients with a predictive index value ≥ 8 underwent NACT followed by interval reduction surgery. After three to four cycles of carboplatin-based NACT (generally a paclitaxel- and carboplatin-based regimen), patients were considered “excellent responders” (ER) if there was a complete response or only microscopic disease left at time of interval surgery, or they were considered “poor responders” (PR) if they presented stable or progressive disease on radiologic evaluation and/or suboptimal interval cytoreduction after NACT, according to Response Evaluation Criteria in Solid Tumors version 1.1. At interval surgery, post-chemotherapy tissues were collected and stored. The study was approved by the Institutional Review Board of The University of Texas MD Anderson Cancer Center, and all samples were collected after obtaining written informed consent from patients.

For the collection of tissues from GYN-COE, frozen tumors and clinical data were collected before and after neoadjuvant paclitaxel and carboplatin chemotherapy from patients with histologically confirmed advanced-stage, high-grade serous ovarian or tubal carcinoma and banked at the Women’s Health Integrated Research Center in Annandale, VA. These patients provided broad consent for their tissues to be used in future research under WCG IRB Protocol #20110222, Tissue and Data Acquisition Activity for the Study of Gynecologic Disease. The paired tumor specimens and clinical data were collaboratively evaluated under WCG IRB Protocol #14-1679, an Integrated Molecular Analysis of Endometrial Cancer, Ovarian Cancer, and Other Medical Conditions to Identify and Validate Clinically Informative Biomarkers and Factors, and the fully executed Material Transfer Agreement #205-20.

For the collection from Washington University, frozen tumors and clinical data were collected before neoadjuvant paclitaxel and carboplatin chemotherapy from patients with histologically confirmed advanced-stage, high-grade serous ovarian or tubal carcinoma and banked at the University of Iowa as part of a collaborative study; these patients gave informed consent as part of our Washington University Tumor Tissue Banking IRB 201105400 or our collaborative R01 with Iowa: IRB 201104242 and 20511102. The study was approved by the Institutional Review Board of the University of Iowa (protocol #201507805).

Unidentified frozen blocks from HGSC of two different patients were obtained from the Cooperative Human Tissue Network (CHTN) and used to test the reproducibility of DESI-MS on multiple sections (Supplementary Fig. 1).

### In vivo models of ovarian cancer

Animal protocols were approved by the MD Anderson Institutional Animal Care and Use Committee and experiments were performed with 6- to 8-week-old female athymic nude mice (NCr-nude) obtained from Taconic Biosciences. Luciferase-labeled SKOV3ip1 ovarian cancer cells were used to establish xenograft models for all mouse experiments as described before^42^. Cells were cultured to 70–90% confluence and then trypsinized, washed twice with phosphate-buffered saline, and resuspended in ice-cold Hank’s Balanced Salt Solution (cat. #21-021-CV; Cellgro). The mice were then inoculated with 1 × 10^6^ SKOV3ip1 cells via intraperitoneal injection to the right side of the abdomen. Tumor establishment was subsequently confirmed after injection of 200 μL of 14.3 mg/mL luciferin (cat. #LUCK-1G; GoldBio) using a Xenogen IVIS in vivo imaging system. Following tumor establishments, mice were randomly assigned to treatment groups as described in {Glassman, 2023 #119}. For the purpose of this study, tumors from mice treated with vehicle control were considered. Investigators sacrificed the mice via carbon dioxide euthanasia followed by cervical dislocation once the mice were moribund. At the time of gross necropsy, mouse tumor weights, nodule numbers, distribution of metastasis, and presence of ascites were recorded. All tumor tissues were dissected, and samples were snap-frozen, fixed in formalin for paraffin embedding, or snap-frozen in optimal cutting temperature compound (Mercedes Scientific) for frozen slide preparation.

### DESI-MS

A 2-dimensional Omni Spray (Prosolia Inc, Indianapolis, IN) was used for tissue imaging with an LTQ-Orbitrap Elite mass spectrometer (Thermo Scientific, San Juan, CA). DESI-MS imaging was performed in the negative ion mode from *m/z* 100 to 1500 with a hybrid mass spectrometer, which allows tandem MS experiments to be performed with high mass accuracy (<5 ppm mass error) and high mass resolution (60,000 resolving power). Imaging was performed using a spatial resolution of 200 µm. Ion images were assembled using Biomap (Novartis) software. For negative ion mode analyses, the histologically compatible spray solvent dimethylformamide:acetonitrile (DMF:ACN) 1:1 (v/v) was used to perform the imaging analyses at a flow rate of 1.2 µL/min^38^. DESI-MS data were deposited at https://data.mendeley.com/datasets/zzr5rk7vj5/1. For many cases we analyzed multiple sections of the same tumor; prior studies have evaluated the reproducibility of DESI-MS imaging on serial tissue sections^43^.

### Histopathology and light microscopy

The same tissue sections analyzed by DESI-MS were then subjected to standard H&E staining. Pathologic diagnosis was made by Dr. Jinsong Liu using light microscopy. Light microscopy images were obtained and subjected to manual tissue segmentation into the two regions of interest, epithelium, and stroma, based on morphologic assessment.

### DESI-MS reproducibility

In order to test the reproducibility of DESI-MS on several sections from the same tumor block, we used sections from frozen tumor blocks derived from two patients with ovarian cancer and 5 total ovarian cancer xenograft mice. Two sections from each human tumor and four sections from four of the five xenografts were used for the analysis. For one of the five xenografts two sections were analyzed. After DESI-MS, mass spectra were identified, and a Cosine Similarity analysis was performed. (Supplementary Fig. 1).

### Global proteomics and phosphoproteomics analysis

Global proteomics and phosphoproteomics analysis of pre-chemotherapy samples from 7 ER and 8 PR patients, including tumors from primary and metastatic disease sites for a subset of cases, was performed as described previously^13^. Briefly, laser microdissection was used to collect whole tumor samples (cancer and stromal cells combined), which underwent pressure-assisted digestion employing a barocycler (2320EXT Pressure BioSciences, Inc.) and a heat-stable form of trypsin (SMART Trypsin, ThermoFisher Scientific, Inc.). Peptide digestion was labeled per tandem mass tag channel (TMT-11, ThermoFisher Scientific, Inc.). Sample multiplexes were separated offline using basic reversed-phase liquid chromatographic fractionation on a 1260 Infinity II liquid chromatograph (Agilent) into 96 fractions using a linear gradient of acetonitrile (0.69% min) followed by concatenation (36 total fractions for global proteomics and 12 fractions for phosphopeptides serially enriched by TiO2 and Fe-IMAC). Each pooled fraction was resuspended in 100 mM NH4HCO3 and analyzed by LC-MS/MS employing a nanoflow LC system (EASY-nLC 1200, ThermoFisher Scientific) coupled online with an Orbitrap Fusion Lumos Tribrid mass spectrometer (ThermoFisher Scientific). In brief, each fraction was loaded onto a nanoflow HPLC system fitted with a reversed-phase trap column (Acclaim PepMap100 C18, 20 mm, nanoViper, Thermo Scientific) and a heated (50 °C) reversed-phase analytical column (Acclaim PepMap RSLC C18, 2 µm, 100 Å, 75 µm × 500 mm, nanoViper, Thermo Fisher Scientific) coupled online with the mass spectrometer. Peptides were eluted using a linear gradient of 2% mobile phase B (95% acetonitrile, 0.1% formic acid) to 32% mobile phase B over 120 min at a constant flow rate of 250 nL/min. High-resolution (R = 60,000 at m/z 200) broadband (m/z 400-1600) mass spectra were acquired, followed by the selection of the 12 most intense molecular ions in each MS scan for high-energy collisional dissociation. Global protein-level and phosphosite identifications were generated by searching .raw data files with a publicly available, non-redundant human proteome database (Swiss-Prot, Homo sapiens [http://www.uniprot.org/]) using Mascot (Matrix Science), Proteome Discoverer (Thermo Fisher Scientific), and in-house tools using identical parameters as described previously^34^. Differential analyses of global proteome or transcriptome matrixes were performed using the LIMMA package (version 3.8) in R (version 3.5.2), and candidates mapping to metabolomic pathways of interest identified from the Reactome databases were prioritized for downstream analysis. A more detailed description of this method can be found in^13^. A total of 7148 proteins and >1075 phosphosites were co-quantified across cases (Supplemental Tables 13–18). Protein and phosphosite mapping to the Reactome pathways altered between ER and PR cases observed in the metabolomic analysis were selected for further analysis. Pathways with the highest number of proteins quantified included the metabolism of amino acids and derivatives, metabolism of nucleotides, and respiratory electron transport and related pathways. Differential analysis revealed that most proteins and phosphosites were significantly altered (LIMMA *p* < 0.05, ±1.5-fold change) between the PR and ER cases mapped to the metabolism of amino acids and derivatives and metabolism of nucleotide pathways. A heatmap for the eight proteins significantly altered in PR versus ER pre-chemotherapy tumors, mostly mapping to the metabolism of amino acids and derivatives pathway (*z*-score = 0.728 *p*-value 1.69E-13, derived from Ingenuity Pathway Analysis), is shown in Fig. 5a. A principal component analysis plot of these proteins by case (Fig. 5b) illustrates a distinct separation of the pre-chemotherapy PR and ER tumors. For the phosphoproteome analysis, the comparison reflects the relative abundance of a given phosphosite, i.e., phosphorylated residue, of interest in one condition versus the other. The mass spectrometry proteomics data are available at the ProteomeXchange Consortium via the PRIDE (10.1093/nar/gky1106) partner repository with the dataset identifier PXD014980.

### Quantification

MS data were extracted from the regions of interest using MSiReader software. Significance analysis of microarrays was used to identify ions with significantly different abundances in ER samples compared with PR samples and in pre-chemotherapy samples compared with post-chemotherapy samples. Features below 10% FDR were selected. Selective analyses were carried out separately for data extracted from epithelial and stromal regions. The identified metabolites were divided for analytical purposes into non-lipid (such as uracil, fumarate, hypoxanthine, glutamic acid, and citrate) and lipid (such as fatty acids, glycolipids, ceramides, and cardiolipins) categories.

### Pathway analysis

To study the non-lipid metabolic species, we carried out pathway analyses using REACTOME (https://reactome.org/) and confirmed the findings in Pathway Studio (https://www.pathwaystudio.com/). Enriched REACTOME pathways were ordered according to a probability score corrected for FDR using the Benjamini-Hochberg method. We selected the 10 most relevant pathways in REACTOME sorted by p-value, and pathways with a common hit in Pathway Studio were considered. Pathway analysis was performed only when at least two metabolites (small molecules) were recognized by the software. In cases in which one software was not able to recognize more than two metabolites, the results from the other software were considered.

### Prediction model

The glmnet package^44^ in R version 3.6.3 was used to create a ridge regression model for the classification of treatment response. Prior to analysis, data were pre-processed by removing m/z values that were present in less than 10% of all spectra. Intensities were median-normalized by dividing the intensity of each individual ion in a spectrum by the median intensity for the same spectrum. Ten-fold cross-validation was used to create a ridge regression model for the classification of ER and PR samples from the epithelial regions of pre-chemotherapy tumor tissues. The analysis was restricted to the primary sites (adnexa and ovaries), and samples from metastatic sites (omentum or abdominal organs) were excluded. The ridge regression model used 78 features for class distinction, represented by a variety of small molecules and lipid species between 100 and 1000 m/z. The ridge regression model was used to estimate the probability of every mass spectrum belonging to either the ER or PR group. If more than 50% of the spectra were correctly predicted for a single sample, we considered the sample to be correctly classified in our per-sample prediction results. Pixel-based and sample-based accuracies, sensitivities, and specificities were calculated.

### Sparse partial least squares discriminant analysis

Metaboanalyst 5.0 was used for sparse partial least squares discriminant analysis (sPLS-DA). Prior to sPLS-DA, data were TIC-normalized and mean-centered. sPLS-DA plots were used to visualize the distinct separation between pre- and post-chemotherapy samples in both the ER samples and PR samples (Fig. 3b).

### Reporting summary

Further information on research design is available in the Nature Research Reporting Summary linked to this article.

### Supplementary information

supplementary material

Reporting Summary

## Data Availability

This study did not generate new unique reagents. Further information and requests for resources and reagents should be directed to and will be fulfilled by the lead contacts, Anil K. Sood (asood@mdanderson.org) and Livia S. Eberlin (Livia.Eberlin@bcm.edu). The MS proteomics data are available at the ProteomeXchange Consortium via the PRIDE (10.1093/nar/gky1106) partner repository with the dataset identifier PXD014980. DESI-MS data were deposited at https://data.mendeley.com/datasets/zzr5rk7vj5/1.

## Code availability

The glmnet package in R^44^ was used to create a ridge regression model for the classification of treatment response. Metaboanalyst 5.0 was used for sparse partial least squares discriminant analysis (sPLS-DA).
