# Supplementary material for: In situ profiling reveals metabolic alterations in the tumor microenvironment of ovarian cancer after chemotherapy
Source: NPJ Precis Oncol. 2023 Nov 3;7:115. doi: 10.1038/s41698-023-00454-0 (PMC10624842; doi:10.1038/s41698-023-00454-0)
Supplement: Supplementary file 1 — supplementary material [file 41698_2023_454_MOESM1_ESM.pdf]

Supplementary Materials and Methods

GLDC expression in CCLE

Data on GLDC mRNA expression in ovarian cancer cell lines were downloaded from the DepMap Public 22Q4 Primary Files at Cancer Cell Line Encyclopedia (CCLE, https://depmap.org/portal/download/all/) and plotted using GraphPad Prism version 9.0.0, for cell lines with GLDC expression above 3.

Cell culture

All ovarian cancer cell lines were obtained from the American Type Culture Collection and the MD Anderson Characterized Cell Line Core Facility, which supplies authenticated cell lines. OVCAR8ip2 was derived from 2 consecutive intraperitoneal injections in nude mice after first injection and tumor formation, while SKOV3ip1 was derived from 1 consecutive intraperitoneal injection in nude mice after first injection and tumor formation. The immortalized non-transformed human ovarian surface epithelial cell line HIO-180 was a kind gift from Dr. Andrew Godwin at the Fox Chase Cancer Center (Philadelphia, PA). Cells were maintained and propagated *in vitro* by serial passage in either Roswell Park Memorial Institute Medium (RPMI) or Dulbecco's Modified Eagle Medium (DMEM) supplemented with 10% fetal bovine serum (FBS) and 0.1% gentamicin sulfate. Cells were maintained at 37 °C in a 5% CO2 incubator with 95% humidity. All of the experiments were performed with 70–80% confluent cultures.

RNA isolation and quantitative PCR

Total RNA was isolated using Direct-zol RNA miniprep kit (cat# R2050 Zymo Research, Irvine CA) according to the manufacturer’s instructions. Complementary DNA was synthesized from 200 ng of the total RNA using Verso cDNA Synthesis Kit (cat# AB1453A, ThermoFisher, Waltham MA). The resulting cDNA was then used to amplify GLDC; a quantitative PCR was performed in triplicate using Power Syber Green (cat# 4367659, ThermoFisher) and specific primers for GLDC

(forward: GCTTGGTGAGAATGATGCCTGG; reverse: CAGATGTTGCTGGTAGCCTTGTC) and for 18S (forward: CGCCGCTAGAGGTGAAATTC; reverse: TTGGCAAATGCTTTCGCTC) (Sigma-Aldrich, Saint Louis, MO). Expression levels are shown as log2 fold change of GLDC expression after normalization to the housekeeping gene and to the HIO 180 cells.

Transient transfection and GLDC silencing

IGROV1 cells were seeded at the density of $1x10^5$ per well in a six-wells plate. The day after they were treated with 3 μL RNAiMAX (cat# 137778-150, Invitrogen, Waltham, MA) and 2 uL siRNA per ml of Opti-MEM (cat# 31985070 ThermoFisher), for four hours. Four types of siRNAs were used, three active siRNAs, SASI_Hs01_00155729 (siRNA29), SASI_Hs02_00302003 (siRNA03), and SASI_Hs01_00155735 (siRNA35) and control siRNA (Control UUCUCCGAACGUGUCACGU[dT][dT]; Control_as ACGUGACACGUUCGGAGAA[dT][dT]). All siRNA sequences were purchased from Sigma. After that, cells were washed with phosphate-buffered saline (PBS) solution and regular medium was added. After 48 hours cells were trypsinized and RNA was isolated (as above). GLDC mRNA expression levels were measured via quantitative PCR as described above (numbers were normalized to the housekeeping gene 18S and to the siRNA control transfected cells)

MTT and cell viability assay

Cells were seeded at the density of $1x10^4$ per well in a 96-well plate. The day after, cells were transfected as above with siRNA control and siRNA35 (ratios between RNAiMAX and siRNAs were maintained as above). Six replicates per condition were performed. The day after, cells were treated with 11 increasing concentrations of carboplatin (cat# C177500, Toronto Research Chemicals, Canada), for 72 hours. After 48h from the first transfection, cells were transfected again (this time using 1.5 μL of RNAiMAX and 1 μL of siRNA per ml of medium). After 72 h of carboplatin treatment, 3-(4,5-dimethylthiazol-2-yl)-2,5-diphenyltetrazolium bromide) tetrazolium (MTT) was added to the medium and absorbance at 570nm was read according to the

manufacturer instructions. Absorbance values were normalized, IC50 was calculated using Graph Pad Prism version 9.0.0 and dose-response curves were plotted. Hypothesis test with alpha 0.05 was performed.

Supplementary Figures Legends

Supplementary Figures 1: Comparison of ion images between two sections from one mouse xenograft and two human HGSC tissues and (A and B). Mass spectra are plotted and average per tissue cosine similarity scores are shown for one xenograft and one human tissue (B and C). In D, four additional mouse xenografts, with four sections each are analyzed and average cosine similarity scores resulting from paired comparisons between sections are shown.

OvCa = ovarian cancer; H&E= hematoxylin and eosin; m/z = mass-to-charge ratio

Supplementary Figures 2: Kaplan-Meier plots for mRNA expression of the four enzymes of interest identified by the proteomic analysis. Plots were obtained from KMplot.com. Serous ovarian cancers grade 2+3 (N=1029) were selected; patients were split by median cut-off for expression of each gene. A) Kaplan-Meier plot for PFS according to high and low ACADSB (205355_at). B) Kaplan-Meier plot for PFS according to high and low BCAT1 (214452_at) C) Kaplan-Meier plot for PFS according to high and low GLDC (204836_at). D) Kaplan-Meier plot for PFS according to high and low ornithine aminotransferase (201599_at).

Supplementary Figures 3: GLCD expression in normal and tumor tissues. A) Bulk tissue gene expression for GLDC (gtexportal.com). B) Protein expression for GLDC (HPA002318 antibody) in 21 cancer types (N: maximum 12 patients each tumor type), according to Protein Atlas (proteinatlas.org). C) RNA-seq data from TCGA for the GLDC mRNA levels in 17 cancer types.

Supplementary Figures 4: Biological effects of GLDC silencing. A) CCLE data for mRNA expression of GLDC in ovarian cancer cell lines (Log2 expression data above 3 were plotted). B) mRNA expression of GLDC measured with qPCR in ten cell lines, Log2 fold change values were

normalized on normal ovarian cell lines; **=T test p value <0.01. C) mRNA expression of GLDC measured with qRT-PCR in IGROV1 cells after transient transfection with siRNAs 29, 35 and 03. Log2 fold change values were normalized on cells treated with siRNA control. D) Cell viability assay measured with MTT of IGROV1 transfected with siRNA35 and siRNA control, after treatment with carboplatin at increasing doses for 72h. Each dot represents mean viability and whiskers represent SEM (standard error of the mean)

Supplementary Table Legends

Supplementary Table 1: Patients' demographic and clinical characteristics.

Supplementary Table 2: A) Reactome pathway analysis of upregulated metabolites in the epithelial regions of pre-chemotherapy PR tissues. B): Pathway Studio pathway analysis of upregulated metabolites in epithelial regions of pre-chemotherapy PR tissues.

Supplementary Table 3: A) Reactome pathway analysis of upregulated metabolites in the epithelial regions of pre-chemotherapy ER tissues. B) Pathway Studio pathway analysis of upregulated metabolites in the epithelial regions of pre-chemotherapy ER tissues.

Supplementary Table 4: A) Reactome pathway analysis of upregulated metabolites in the stromal regions of pre-chemotherapy ER tissues. B) Pathway Studio pathway analysis of upregulated metabolites in the stromal regions of pre-chemotherapy ER tissues.

Supplementary Table 5: A) Reactome pathway analysis of upregulated metabolites in the stromal regions of pre-chemotherapy PR tissues. B) Pathway Studio pathway analysis of upregulated metabolites in the stromal regions of pre-chemotherapy PR tissues.

Supplementary Table 6: A) Reactome pathway analysis of downregulated metabolites in post-chemotherapy versus pre-chemotherapy ER samples. B) Pathway Studio pathway analysis of downregulated metabolites in post-chemotherapy versus pre-chemotherapy ER samples.

Supplementary Table 7: A) Reactome pathway analysis of upregulated metabolites in post-chemotherapy versus pre-chemotherapy ER samples. B) Pathway Studio pathway analysis of upregulated metabolites in post-chemotherapy versus pre-chemotherapy ER samples.

Supplementary Table 8: A) Reactome pathway analysis of downregulated metabolites in the stromal regions of post-chemotherapy versus pre-chemotherapy ER samples. B) Pathway Studio pathway analysis of downregulated metabolites in the stromal regions of post-chemotherapy versus pre-chemotherapy ER samples.

Supplementary Table 9: A) Reactome pathway analysis of upregulated metabolites in the stromal regions of post-chemotherapy versus pre-chemotherapy ER samples. B) Pathway Studio pathway analysis of upregulated metabolites in the stromal regions of post-chemotherapy versus pre-chemotherapy ER samples.

Supplementary Table 10: A) Reactome pathway analysis of upregulated metabolites in the epithelial regions of post-chemotherapy versus pre-chemotherapy PR samples. B) Pathway Studio pathway analysis of upregulated metabolites in the epithelial regions of post-chemotherapy versus pre-chemotherapy PR samples.

Supplementary Table 11: A) Reactome pathway analysis of upregulated metabolites in the stromal regions of post-chemotherapy versus pre-chemotherapy PR samples. B) Pathway Studio pathway analysis of upregulated metabolites in the stromal regions of post-chemotherapy versus pre-chemotherapy PR samples.

Supplementary Table 12: A) Reactome pathway analysis of downregulated metabolites in the stromal regions of post-chemotherapy versus pre-chemotherapy PR samples. B) Pathway studio pathway analysis of downregulated metabolites in the stromal regions of post-chemotherapy versus pre-chemotherapy PR samples.

Supplementary Table 13: Global proteomic data for NACT-ER and NACT-PR tumors.

Supplementary Table 14: Phosphoproteomic data for NACT-ER and NACT-PR tumors.

Supplementary Table 15: Global proteomics mapping to Reactome pathways prioritized from metabolomic analysis of NACT-ER and NACT-PR tumors.

Supplementary Table 16: Phosphoproteomic data mapping to Reactome pathways prioritized from metabolomic analysis for NACT-ER and NACT-PR tumors.

Supplementary Table 17: Differential analysis of proteins mapping to Reactome pathways in NACT-PR versus NACT-ER tumors (LIMMA $p<0.05$, ± 1.5-fold-change).

Supplementary Table 18: Differential analysis of phosphosites mapping to Reactome pathways in NACT-PR versus NACT-ER tumors (LIMMA $p<0.05$, ± 1.5-fold-change).

Supplementary Table 19: Nomenclature of the samples undergone proteomic and phosphoproteomic analysis.

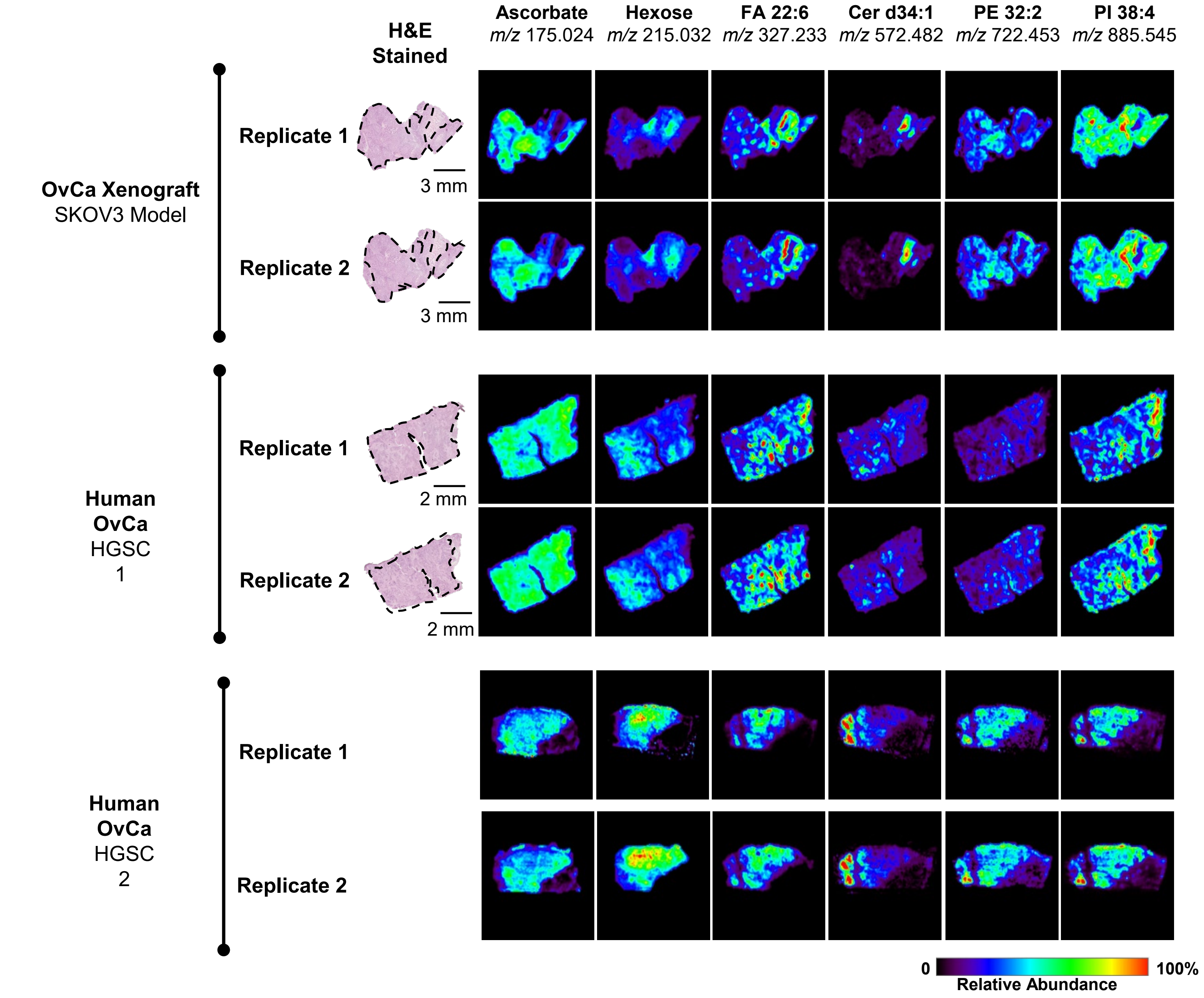

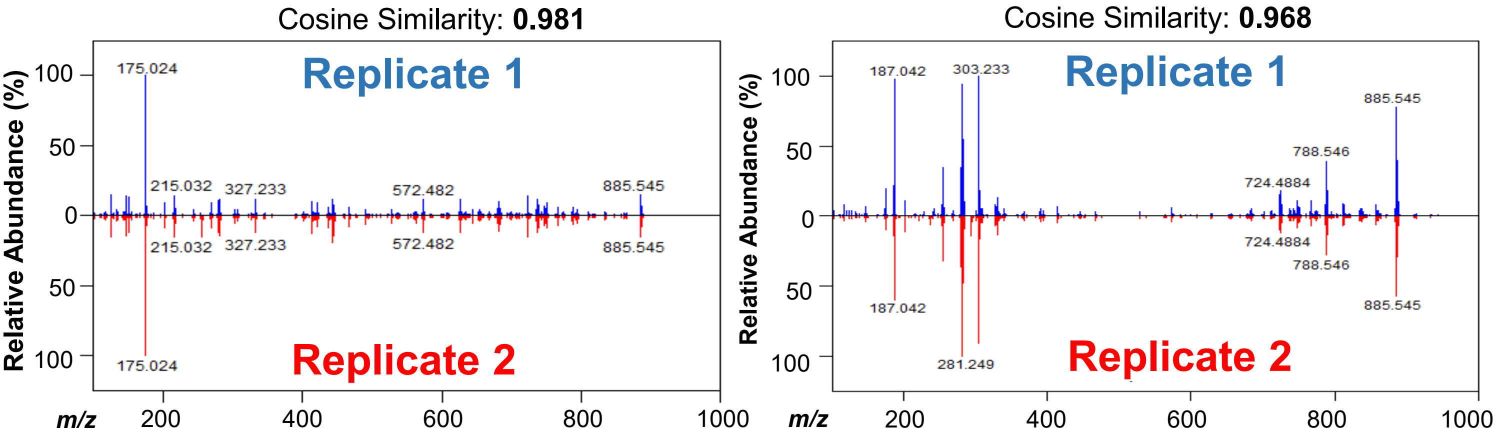

m/z 100-1000	Replicate	1	2	3	4
	1	1.00	0.972	0.959	0.962
	2	0.972	1.00	0.971	0.988
	3	0.959	0.971	1.00	0.978
	4	0.962	0.988	0.978	1.00

Average Cosine Score: **0.972**

Supplementary Figure 1

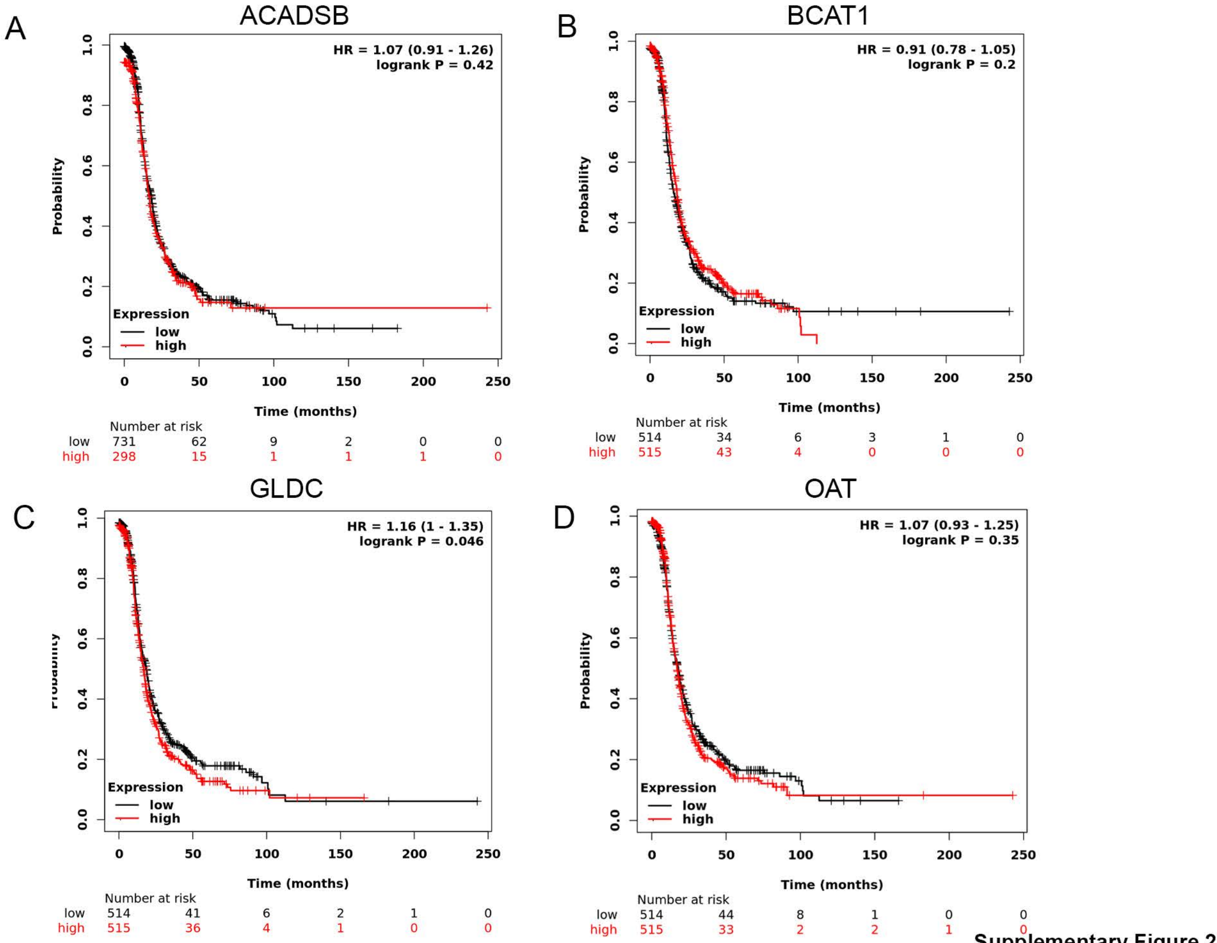

Supplementary Figure 2

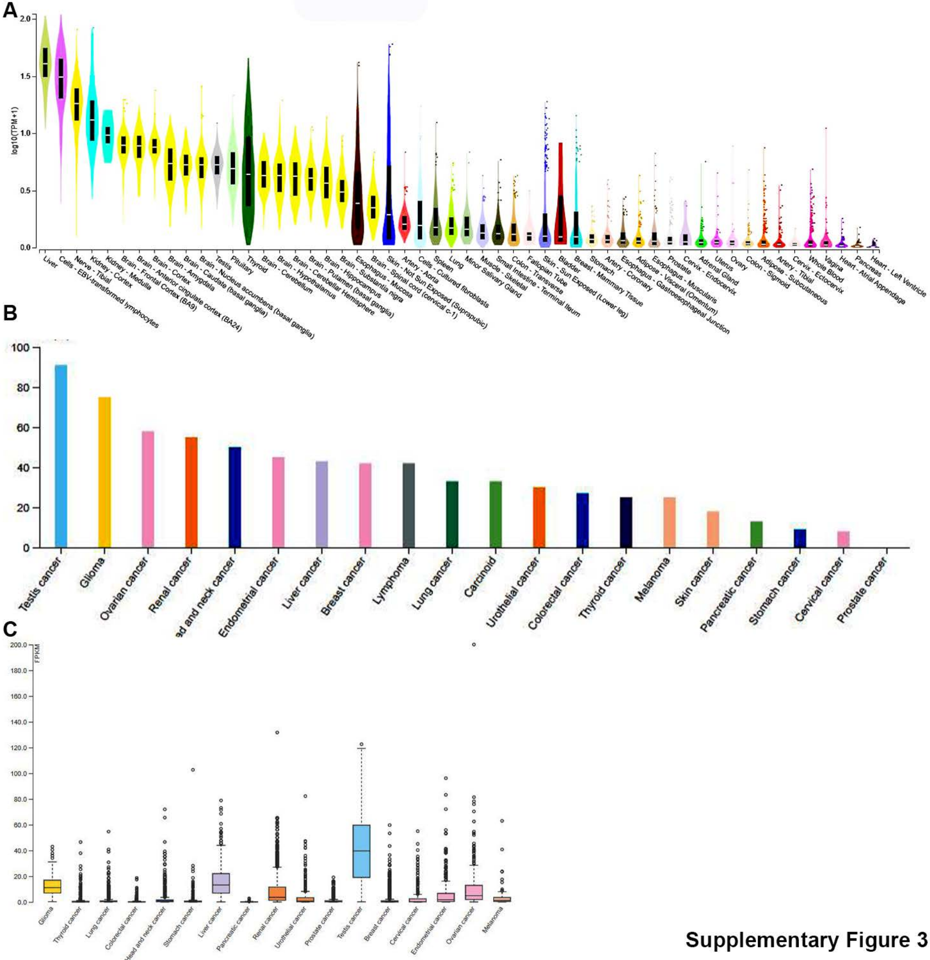

Supplementary Figure 3

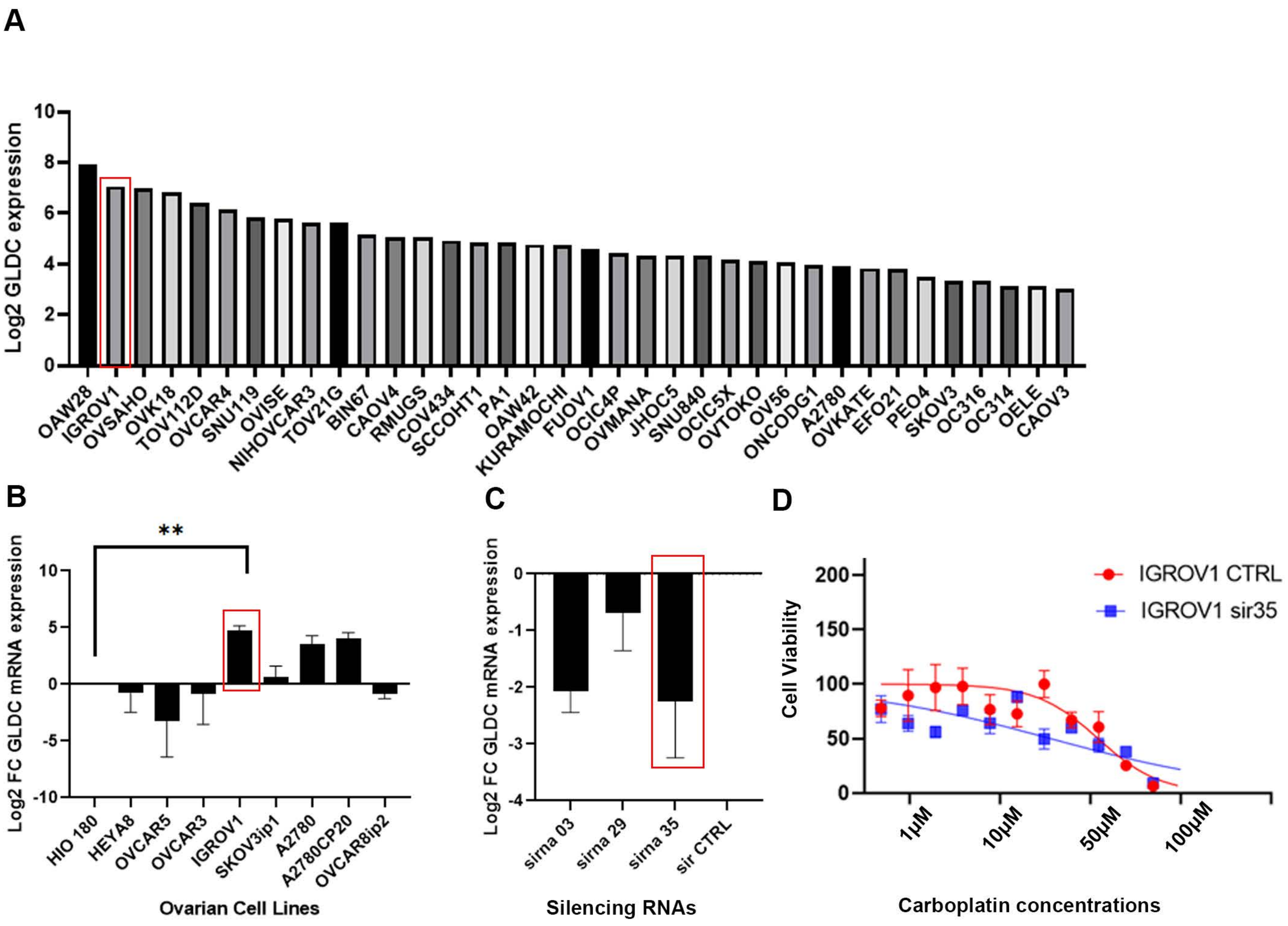

Supplementary Figure 4

Table S1. Patients' demographic and clinical characteristics

Patients' demographic and clinical characteristics.

Group	Patient ID	Age (yr)	BMI (kg/m^2)	CA-125 at diagnosis (units/ml)	Stage	BRCA status
NACT-ER	NACT-ER-1	49	28.9	26.6	IIIC	Patient declined
	NACT-ER-2	53	29.8	1934	IVA	No mutation
	NACT-ER-3	77	22.6	465.5	IIIC	No mutation
	NACT-ER-4	45	34.3	6201	IIIC	BRCA1
	NACT-ER-6	71	21.4	740.1	IIIC	No mutation
	NACT-ER-7	78	23.9	365.3	IIIC	No mutation
	NACT-ER-8	58	30.4	87.6	IIIC	Unknown
	NACT-ER-9	67	21.7	1133	IIIC	No mutation
	NACT-ER-10	65	41	1105	IIIC	BRCA1
	NACT-ER-11	58	28.9	1618	IIIC	No mutation
	NACT-ER-12	69	29.2	3520	IVA	Patient Declined
	NACT-ER-13	47	21.5	625.8	IIIC	BRCA2
	NACT-ER-14	66	33.6	105	IIIC	No mutation
	NACT-ER-15	56	32.2	78.6	IIIC	No mutation
	NACT-ER-16	44	41.4	255.8	IIIC	No mutation
	NACT-ER-17	66	31.1	1649	IIIC	Unknown
	NACT-ER-18	57	25.9	521	IIIC	Unknown
	NACT-ER-19	64	33.2	2680	IIIC	No mutation
	NACT-ER-20	62	20.4	770.2	IIIC	No mutation
	NACT-ER-21	70	22.8	1202	IIIC	No mutation
	NACT-ER-22	74	24	1649	IVB	No mutation
	NACT-ER-23	57	43.3	820.6	IIIC	No mutation
	NACT-ER-24	58	29.8	1255	IIIC	No mutation
	NACT-ER-25	61	31.9	186.5	IVB	No mutation
	NACT-ER-26	62	18	5523	IIIC	BRCA2
	NACT-ER-27	58	28.1	4333.8	IVB	No mutation
	NACT-ER-28	55	23.9	10000	IIIA2	Unknown
	NACT-ER-29	62	26.9	1337	IIIC	Unknown
	INOVA-ER-1	69	24.9392	628	IVB	No mutation
	INOVA- ER-2	55	23.2199	10000	IIIA2	Unknown
	INOVA-ER-3	62	26.8481	1337	IIIC	Unknown
	INOVA-ER-4	59	22.4943	632.9	IIIC	No mutation
	IOWA-ER-206	44	24.32	2421	III	Unknown
	IOWA-ER-253	65	27.87	893.2	IV	Unknown
	NACT-PR-1	62	28.2	1116	IIIC	No mutation
	NACT-PR-2	57	32.1	494.6	IIIC	No mutation
	NACT-PR-3	73	18.9	335.3	IVB	No mutation
	NACT-PR-5	62	29.4	1467	IIIC	Patient declined
	NACT-PR-6	73	30.8	335.6	IIIC	No mutation
	NACT-PR-7	67	27.7	3452	IVA	BRCA2
	NACT-PR-8	60	27.6	387.8	IIIC	No mutation
	NACT-PR-10	59	19.9	551.3	IVA	Unknown

NACT-PR	NACT-PR-11	72	31.1	1381	IVB	No mutation
	NACT-PR-12	66	20.6	162.8	IIIC	No mutation
	NACT-PR-13	57	25.9	521	IIIC	Unknown
	NACT-PR-14	68	30.4	79.4	IIIC	No mutation
	NACT-PR-15	49	23.8	726.1	IIIC	VUS
	NACT-PR-16	59	27.7	367.7	IIIC	No mutation
	NACT-PR-17	54	31.7	493.5	IIIC	Unknown
	NACT-PR-18	67	32.2	3721	IVB	No mutation
	NACT-PR-19	80	21.8	61.4	IIIC	Unknown
	NACT-PR-20	69	30.3	1012	IIIC	No mutation
	NACT-PR-21	63	29	303	IVB	No mutation
	NACT-PR-22	47	41.9	5605	IIIC	BRCA1
	INOVA-PR-1	58	28.1	4333.8	IIIC	Unknown
	INOVA-PR-2	69	34.3	1593	IIIC	No mutation
	INOVA-PR-3	64	26.5	8037	IVB	No mutation
	IOWA-PR-2	60	23.86	340.7	IV	Unknown
	IOWA-PR-154	87	23.26	318.7	IV	Unknown
		NS	NS	NS	NS	

NACT-ER, excellent response (ER) to neoadjuvant chemotherapy (NACT);
NACT-PR, poor response to NACT; BMI, body mass index; VUS, variant of unknown significance;
NS: non statistically significant difference between ER and PR

Pathway identifier	Pathway name	Entities ratio	Entities pValue	Entities FDR	Reactions ratio	Species identi	Submitted entities found	Mapped entities
R-HSA-5619063	Defective SLC29A3 causes histiocytosis-lymphadenopathy plus syndrome (HLAS)	0.003721425	0.006687347	0.067712736	1.34E-04	9606	C00299	16704
R-HSA-425407	SLC-mediated transmembrane transport	0.087719298	0.00809139	0.067712736	0.025610083	9606	C00299;C00245	15891;16704
R-HSA-382551	Transport of small molecules	0.116427432	0.01535702	0.067712736	0.054035935	9606	C00299;C00245	15891;16704
R-HSA-83936	Transport of nucleosides and free purine and pyrimidine bases across the plasma membrane	0.011164274	0.019994735	0.067712736	0.002145347	9606	C00299	16704
R-HSA-159418	Recycling of bile acids and salts	0.011164274	0.019994735	0.067712736	0.002279431	9606	C00245	15891
R-HSA-73614	Pyrimidine salvage	0.012227539	0.021888464	0.067712736	0.001340842	9606	C00299	16704
R-HSA-8963693	Aspartate and asparagine metabolism	0.013290803	0.024725624	0.067712736	0.001474926	9606	C00245	15891
R-HSA-442660	Na+/Cl- dependent neurotransmitter transporters	0.017012228	0.030387583	0.067712736	0.002011263	9606	C00245	15891
R-HSA-352230	Amino acid transport across the plasma membrane	0.018607124	0.033212381	0.067712736	0.004827031	9606	C00245	15891
R-HSA-73621	Pyrimidine catabolism	0.020733652	0.036972368	0.067712736	0.002279431	9606	C00299	16704
R-HSA-1614558	Degradation of cysteine and homocysteine	0.02020202	0.040725029	0.067712736	0.002949853	9606	C00245	15891
R-HSA-8956321	Nucleotide salvage	0.024455077	0.043534718	0.067712736	0.002949853	9606	C00299	16704
R-HSA-193368	Synthesis of bile acids and bile salts via 7alpha-hydroxycholesterol	0.028708134	0.053801653	0.067712736	0.005229284	9606	C00245	15891
R-HSA-425397	Transport of vitamins, nucleosides, and related molecules	0.034556087	0.061233717	0.067712736	0.005229284	9606	C00299	16704
R-HSA-425393	Transport of inorganic cations/anions and amino acids/oligopeptides	0.030834662	0.067712736	0.067712736	0.010056315	9606	C00245	15891
R-HSA-1614635	Sulfur amino acid metabolism	0.034556087	0.068636478	0.068636478	0.004827031	9606	C00245	15891
R-HSA-192105	Synthesis of bile acids and bile salts	0.041998937	0.079685676	0.079685676	0.011128989	9606	C00245	15891
R-HSA-194068	Bile acid and bile salt metabolism	0.042530569	0.080603466	0.080603466	0.013274336	9606	C00245	15891
R-HSA-425366	Transport of bile salts and organic acids, metal ions and amine compounds	0.040935673	0.080603466	0.080603466	0.008983642	9606	C00245	15891
R-HSA-5619102	SLC transporter disorders	0.042530569	0.087015178	0.087015178	0.008313221	9606	C00299	16704
R-HSA-8956319	Nucleotide catabolism	0.056353004	0.100679014	0.100679014	0.008447305	9606	C00299	16704
R-HSA-5619115	Disorders of transmembrane transporters	0.051568315	0.104305304	0.104305304	0.010726736	9606	C00299	16704
R-HSA-15869	Metabolism of nucleotides	0.082402977	0.144596278	0.144596278	0.018771789	9606	C00299	16704
R-HSA-8957322	Metabolism of steroids	0.080808081	0.145481102	0.145481102	0.026682757	9606	C00245	15891
R-HSA-71291	Metabolism of amino acids and derivatives	0.151515152	0.26401662	0.26401662	0.037543577	9606	C00245	15891
R-HSA-1643685	Disease	0.18394471	0.411733019	0.411733019	0.116519174	9606	C00299	16704
R-HSA-1430728	Metabolism	0.758639022	0.580221149	0.580221149	0.288549209	9606	C00299;C00245	15891;16704
R-HSA-556833	Metabolism of lipids	0.337054758	0.604124219	0.604124219	0.11893269	9606	C00245	15891

Supplementary Table 2a: Reactome pathways analysis of upregulated metabolites in epithelial regions of pre-chemo PR tissues

PR= poor response; ER= excellent response

Name	Parent Folder	Percent Overl	Overlapping E	p-value	Jaccard similarity
Bile Acid Metabolism (Alternative Pathway)	Steroids Metabolism; Steroids Metabolism (Metabolic Pathway); generic; generic	2	taurine	0.01667072	0.02040816
Bile acids alternative biosynthesis template	Animal	2	taurine	0.01667072	0.02040816
Cys degradation template	Aminoacid degradation	1	taurine	0.01805362	0.01886792
Pantothenate-CoA biosynthesis template	anabolism	1	taurine	0.0187447	0.01818182
Bile Acids Metabolism	Steroids Metabolism; Steroids Metabolism (Metabolic Pathway); generic; generic	1	taurine	0.02564218	0.01333333
Bile acids synthesis template	Animal	1	taurine	0.02564218	0.01333333
Pyrimidine Metabolism	Nucleotides Metabolism; Nucleotides Metabolism (Metabolic Pathway); generic; generic	0	uridine	0.03970595	0.00862069
Pyrimidine metabolism template	metabolic cycles	0	uridine	0.04243818	0.00806452
Ser/Gly/Thr/Cys Metabolism	Amino Acids Metabolism; Amino Acids Metabolism (Metabolic Pathway); generic; generic	0	taurine	0.05027166	0.00680272

Supplementary table 2b: Pathway Studio pathway analysis of upregulated metabolites in epithelial regions of pre-chemo PR tissues

PR= poor response; ER= excellent response

Pathway identifier	Pathway name	Entities ratio	Entities pValue	Entities FDR	Reactions ratio	Submitted entities found	Mapped entities
R-HSA-611105	Respiratory electron transport	0.007974482	0.017150706	0.044470365	0.002145347	C11378	46245
R-HSA-1614517	Sulfide oxidation to sulfate	0.008506114	0.019047183	0.044470365	8.05E-04	C11378	46245
R-HSA-500753	Pyrimidine biosynthesis	0.011164274	0.019994735	0.044470365	8.05E-04	C11378	46245
R-HSA-163200	Respiratory electron transport, ATP synthesis by chemiosmotic coupling, and heat production by uncoupling proteins.	0.013822435	0.027558664	0.044470365	0.003754358	C11378	46245
R-HSA-1614558	Degradation of cysteine and homocysteine	0.02020202	0.040725029	0.044470365	0.002949853	C11378	46245
R-HSA-8956320	Nucleotide biosynthesis	0.024986709	0.044470365	0.044470365	0.002949853	C11378	46245
R-HSA-1428517	The citric acid (TCA) cycle and respiratory electron transport	0.031897927	0.068636478	0.068636478	0.008447305	C11378	46245
R-HSA-1614635	Sulfur amino acid metabolism	0.034556087	0.068636478	0.068636478	0.004827031	C11378	46245
R-HSA-15869	Metabolism of nucleotides	0.082402977	0.144596278	0.144596278	0.018771789	C11378	46245
R-HSA-71291	Metabolism of amino acids and derivatives	0.151515152	0.26401662	0.26401662	0.037543577	C11378	46245
R-HSA-1430728	Metabolism	0.758639022	0.943223827	0.943223827	0.288549209	C11378	46245

Supplementary Table 3a: Reactome pathway analysis of upregulated metabolites in the epithelial regions of pre-chemo ER tissues

PR= poor response; ER= excellent response

Name	Parent Folder	Percent Overlap	Overlapping Entities	p-value	Jaccard similarity
Coenzymes (Co)	Fatty Acyl & Other Lipids	10	coenzyme Q10	3.16592E-05	0.090909091
Unsaturated hydroxy fatty acids	Fatty Acyl & Other Lipids	0	alpha-hydroxybutyrate	0.002085268	0.001515152
Ubiquinoine Biosynthesis in Humans	Electron Transport Chain; Electron Transport Chain (Metabolic Pathway); generic; generic	2	coenzyme Q10	0.012169567	0.027777778
Ubiquinone-Q10 biosynthesis (eukaryotic) template	Eukaryots	2	coenzyme Q10	0.017016533	0.02
Leu/Ile/Val biosynthesis template	Aminoacid biosynthesis	1	alpha-hydroxybutyrate	0.019780875	0.017241379
Methionine metabolism template	Aminoacid metabolism and interconvertion	0	alpha-hydroxybutyrate	0.036285191	0.009433962

Suplementary Table 3b : Pathway Studio pathway analysis of upregulated metabolites in the epithelial regions of pre-chemo ER tissues

PR= poor response; ER= excellent response

Pathway identifier	Pathway name	Entities ratio	Entities pValue	Entities FDR	Reactions ratio	Submitted entities found	Mapped entities
R-HSA-5619063	Defective SLC29A3 causes histiocytosis-lymphadenopathy plus syndrome (HLAS)	0.003721425	0.010014231	0.064586107	1.34E-04	C00299	16704
R-HSA-9694548	Maturation of spike protein	0.003189793	0.01286311	0.064586107	4.02E-04	C00221	15903
R-HSA-9683686	Maturation of spike protein	0.003721425	0.014285497	0.064586107	5.36E-04	C00221	15903
R-HSA-9683701	Translation of Structural Proteins	0.00744285	0.024203968	0.064586107	0.001474926	C00221	15903
R-HSA-83936	Transport of nucleosides and free purine and pyrimidine bases across the plasma membrane	0.011164274	0.029841677	0.064586107	0.002145347	C00299	16704
R-HSA-73614	Pyrimidine salvage	0.012227539	0.032652371	0.064586107	0.001340842	C00299	16704
R-HSA-9694635	Translation of Structural Proteins	0.009569378	0.035457631	0.064586107	0.001877179	C00221	15903
R-HSA-9678108	SARS-CoV-1 Infection	0.012759171	0.043840859	0.064586107	0.005363368	C00221	15903
R-HSA-9694516	SARS-CoV-2 Infection	0.014354067	0.048014195	0.064586107	0.00670421	C00221	15903
R-HSA-9679506	SARS-CoV Infections	0.015417331	0.050789662	0.064586107	0.012201663	C00221	15903
R-HSA-73621	Pyrimidine catabolism	0.020733652	0.05494274	0.064586107	0.002279431	C00299	16704
R-HSA-8956321	Nucleotide salvage	0.024455077	0.064586107	0.064586107	0.002949853	C00299	16704
R-HSA-425397	Transport of vitamins, nucleosides, and related molecules	0.034556087	0.090429798	0.090429798	0.005229284	C00299	16704
R-HSA-5619102	SLC transporter disorders	0.042530569	0.127640819	0.127640819	0.008313221	C00299	16704
R-HSA-1643685	Disease	0.18394471	0.137583718	0.137583718	0.116519174	C00299;C00221	15903;16704
R-HSA-8956319	Nucleotide catabolism	0.056353004	0.147151103	0.147151103	0.008447305	C00299	16704
R-HSA-5619115	Disorders of transmembrane transporters	0.051568315	0.152304255	0.152304255	0.010726736	C00299	16704
R-HSA-15869	Metabolism of nucleotides	0.082402977	0.208853878	0.208853878	0.018771789	C00299	16704
R-HSA-425407	SLC-mediated transmembrane transport	0.087719298	0.246310128	0.246310128	0.025610083	C00299	16704
R-HSA-382551	Transport of small molecules	0.116427432	0.327602369	0.327602369	0.054035935	C00299	16704
R-HSA-5663205	Infectious disease	0.066985646	0.328703457	0.328703457	0.043979619	C00221	15903
R-HSA-1430728	Metabolism	0.758639022	0.986471515	0.986471515	0.288549209	C00299	16704

Supplementary Table 4a: Reactome pathway analysis of upregulated metabolites in the stromal regions of pre-chemo ER tissues

PR= poor response; ER= excellent response

Name	Parent Folder	Percent Overlap	Overlapping Entities	Jaccard similarity	p-value
Unsaturated hydroxy fatty acids	Fatty Acyl & Other Lipids	0	alpha-hydroxybutyrate	0.001510574	0.001043177
Cellulose degradation template	catabolilsm	16	beta-D-glucose	0.111111111	0.003135886
Sucrose import (cell wall) template	catabolilsm	6	beta-D-glucose	0.055555556	0.007827413
IAA storage via glucoside conjugation template	Plant hormone storage and degradation	6	beta-D-glucose	0.052631579	0.008347783
Polysaccharide Degradation	Carbohydrates Metabolism (Metabolic Pathway); generic; generic	5	beta-D-glucose	0.043478261	0.010427444
Fructan biosynthesis template	Plants	4	beta-D-glucose	0.041666667	0.010946905
Monolignol storage and Gymnosperm transport template	Lignin and Lignan biosynthesis	4	beta-D-glucose	0.04	0.011466184
Maltose metabolism template	catabolilsm	3	beta-D-glucose	0.029411765	0.016131517
Glycogen Metabolism	Carbohydrates Metabolism (Metabolic Pathway); generic; generic	2	beta-D-glucose	0.025	0.01923357
Starch, Glycogen metabolism template	metabolic cycles	2	beta-D-glucose	0.020833333	0.023359488
Pentose-Phosphate Shunt	Carbohydrates Metabolism (Metabolic Pathway); generic; generic	2	beta-D-glucose	0.020408163	0.023874413
scopolin and esculin biosynthesis template	Defense response	2	beta-D-glucose	0.019607843	0.024903719
Amino sugars synthesis (bacteria) template	Bacteria	1	beta-D-glucose	0.018518519	0.026446321
Pentose phosphate shunt template	catabolilsm	1	beta-D-glucose	0.017241379	0.028500593
Leu/Ile/Val biosynthesis template	Aminoacid biosynthesis	1	alpha-hydroxybutyrate	0.016666667	0.029526644
Glycolisys template	catabolilsm	1	beta-D-glucose	0.015873016	0.031064365
Gluconeogenesis template	anabolism	1	beta-D-glucose	0.014925373	0.033112133
Glucose Metabolism	Carbohydrates Metabolism (Metabolic Pathway); generic; generic	1	beta-D-glucose	0.014084507	0.035157013
Amino sugars synthesis (Eukaryots) template	Eukaryots	1	beta-D-glucose	0.013888889	0.035667782
Methionine metabolism template	Aminoacid metabolism and interconvertion	0	alpha-hydroxybutyrate	0.009259259	0.053935645
Pyrimidine Metabolism	Nucleotides Metabolism; Nucleotides Metabolism (Metabolic Pathway); generic; generic	0	uridine	0.008474576	0.058968784
Pyrimidine metabolism template	metabolic cycles	0	uridine	0.007936508	0.062982419

Supplementary table 4b: Pathway Studio pathway analysis of upregulated metabolites in the stromal regions of pre-chemo ER tissues

PR= poor response; ER= excellent response

Pathway identifier	Pathway name	Entities ratio	Entities pValue	Entities FDR	Reactions ratio	Submitted entities found	Mapped entities
R-HSA-425366	bile salts and organic acids,	0.040935673	0.001693185	0.033212381	0.008983642	C00042;C00245	30031;15891
R-HSA-433137	coupled sulphate, di-	0.002126528	0.003824088	0.033212381	6.70E-04	C00042	30031
R-HSA-3214842	demethylate histones	0.002658161	0.005733385	0.033212381	0.002279431	C00042	30031
R-HSA-1234174	response to hypoxia	0.003189793	0.006687347	0.033212381	0.001340842	C00042	30031
R-HSA-1234176	dependent proline	0.003189793	0.006687347	0.033212381	0.001206758	C00042	30031
R-HSA-112122	mediated reversal of	0.003721425	0.007640851	0.033212381	8.05E-04	C00042	30031
R-HSA-112126	mediated reversal of	0.003721425	0.007640851	0.033212381	8.05E-04	C00042	30031
R-HSA-425407	transmembrane transport	0.087719298	0.00809139	0.033212381	0.025610083	C00042;C00245	30031;15891
R-HSA-5221030	TDG demethylate	0.004784689	0.009546485	0.033212381	6.70E-04	C00042	30031
R-HSA-73943	alkylation damage by	0.004784689	0.009546485	0.033212381	0.001743095	C00042	30031
R-HSA-77108	Utilization of Ketone Bodies	0.005316321	0.009546485	0.033212381	4.02E-04	C00042	30031
R-HSA-1650814	biosynthesis and modifying	0.004784689	0.009546485	0.033212381	0.00160901	C00042	30031
R-HSA-73942	DNA Damage Reversal	0.005316321	0.010498615	0.033212381	0.002011263	C00042	30031
R-HSA-916853	Degradation of GABA	0.005316321	0.010498615	0.033212381	4.02E-04	C00042	30031
R-HSA-5625886	PKN1 stimulates	0.005847953	0.011450287	0.033212381	0.001474926	C00042	30031
R-HSA-2559580	Stress Induced Senescence	0.004253057	0.012401502	0.033212381	0.002413516	C00042	30031
R-HSA-2299718	of Prophase Chromosomes	0.005316321	0.014302557	0.033212381	6.70E-04	C00042	30031
R-HSA-1474290	Collagen formation	0.00744285	0.014302557	0.033212381	0.002011263	C00042	30031
R-HSA-5625740	RHO GTPases activate PKNs	0.007974482	0.015252398	0.033212381	0.002279431	C00042	30031
R-HSA-382551	small molecules	0.116427432	0.01535702	0.033212381	0.054035935	C00042;C00245	30031;15891
R-HSA-71262	Carnitine synthesis	0.008506114	0.016201781	0.033212381	8.05E-04	C00042	30031
R-HSA-6782861	wybutosine at G37 of	0.00744285	0.018099174	0.033212381	0.001072674	C00042	30031
R-HSA-74182	Ketone body metabolism	0.01010101	0.018099174	0.033212381	0.001474926	C00042	30031
R-HSA-2559583	Cellular Senescence	0.00744285	0.018099174	0.033212381	0.004290695	C00042	30031
R-HSA-3247509	Chromatin modifying enzymes	0.008506114	0.019994735	0.033212381	0.009654063	C00042	30031
R-HSA-4839726	Chromatin organization	0.008506114	0.019994735	0.033212381	0.009654063	C00042	30031
R-HSA-159418	Recycling of bile acids and salts	0.011164274	0.019994735	0.033212381	0.002279431	C00245	15891

R-HSA-71291	Metabolism of amino acids and derivatives	0.151515152	0.020193906	0.033212381	0.037543577	C00042;C00245	30031;15891
R-HSA-212165	Epigenetic regulation of gene expression	0.009037746	0.020941828	0.033212381	0.002279431	C00042	30031
R-HSA-389599	Alpha-oxidation of phytanate	0.013290803	0.024725624	0.033212381	0.001072674	C00042	30031
R-HSA-8963693	Aspartate and asparagine metabolism	0.013290803	0.024725624	0.033212381	0.001474926	C00245	15891
R-HSA-888590	GABA synthesis, release, reuptake and degradation	0.009569378	0.026614775	0.033212381	0.001340842	C00042	30031
R-HSA-71403	Citric acid cycle (TCA cycle)	0.014885699	0.027558664	0.033212381	0.002279431	C00042	30031
R-HSA-68875	Mitotic Prophase	0.008506114	0.028502095	0.033212381	0.003620274	C00042	30031
R-HSA-442660	Na+/Cl-dependent neurotransmitter transporters	0.017012228	0.030387583	0.033212381	0.002011263	C00245	15891
R-HSA-6782315	tRNA modification in the nucleus and cytosol	0.014354067	0.030387583	0.033212381	0.004424779	C00042	30031
R-HSA-352230	Amino acid transport across the plasma membrane	0.018607124	0.033212381	0.033212381	0.004827031	C00245	15891
R-HSA-72306	tRNA processing	0.016480595	0.034153064	0.034153064	0.005765621	C00042	30031
R-HSA-68886	M Phase	0.012227539	0.03509329	0.03509329	0.007776884	C00042	30031
R-HSA-1614558	Degradation of cysteine and homocysteine	0.02020202	0.040725029	0.040725029	0.002949853	C00245	15891
R-HSA-69278	Cell Cycle, Mitotic	0.018607124	0.046340285	0.046340285	0.017296862	C00042	30031
R-HSA-1640170	Cell Cycle	0.019670388	0.048208374	0.048208374	0.023330652	C00042	30031
R-HSA-8953854	Metabolism of RNA	0.023391813	0.051939058	0.051939058	0.012469831	C00042	30031
R-HSA-71406	Pyruvate metabolism and Citric Acid (TCA) cycle	0.023923445	0.053801653	0.053801653	0.004692947	C00042	30031

R-HSA-193368	Synthesis of bile acids and bile salts via 7alpha-hydroxycholesterol	0.028708134	0.053801653	0.053801653	0.005229284	C00245	15891
R-HSA-112310	Neurotransmitter release cycle	0.026049973	0.055662416	0.055662416	0.004827031	C00042	30031
R-HSA-390918	Peroxisomal lipid metabolism	0.032429559	0.058450127	0.058450127	0.004692947	C00042	30031
R-HSA-73894	DNA Repair	0.029239766	0.061233717	0.061233717	0.020783052	C00042	30031
R-HSA-425393	Transport of inorganic cations/anions and amino acids/oligopeptides	0.030834662	0.067712736	0.067712736	0.010056315	C00245	15891
R-HSA-1428517	The citric acid (TCA) cycle and respiratory electron transport	0.031897927	0.068636478	0.068636478	0.008447305	C00042	30031
R-HSA-1614635	Sulfur amino acid metabolism	0.034556087	0.068636478	0.068636478	0.004827031	C00245	15891
R-HSA-192105	Synthesis of bile acids and bile salts	0.041998937	0.079685676	0.079685676	0.011128989	C00245	15891
R-HSA-373076	Class A/1 (Rhodopsin-like receptors)	0.041998937	0.079685676	0.079685676	0.010458568	C00042	30031
R-HSA-194068	Bile acid and bile salt metabolism	0.042530569	0.080603466	0.080603466	0.013274336	C00245	15891
R-HSA-2262752	Cellular responses to stress	0.04040404	0.08335409	0.08335409	0.022660231	C00042	30031
R-HSA-8953897	Cellular responses to stimuli	0.041467305	0.087929306	0.087929306	0.026816841	C00042	30031
R-HSA-112315	Transmission across Chemical Synapses	0.037214248	0.091581237	0.091581237	0.017967283	C00042	30031
R-HSA-112316	Neuronal System	0.037214248	0.091581237	0.091581237	0.02105122	C00042	30031
R-HSA-1474244	Extracellular matrix organization	0.014885699	0.100679014	0.100679014	0.005229284	C00042	30031
R-HSA-195258	RHO GTPase Effectors	0.014885699	0.119635311	0.119635311	0.013140252	C00042	30031
R-HSA-194315	Signaling by Rho GTPases	0.014885699	0.119635311	0.119635311	0.025207831	C00042	30031

R-HSA-9716542	Signaling by Rho GTPases, Miro GTPases and RHOBTB3	0.014885699	0.119635311	0.119635311	0.026414588	C00042	30031
R-HSA-74160	Gene expression (Transcription)	0.035087719	0.128591149	0.128591149	0.025878252	C00042	30031
R-HSA-556833	Metabolism of lipids	0.337054758	0.137502575	0.137502575	0.11893269	C00042;C00245	30031;15891
R-HSA-500792	GPCR ligand binding	0.072833599	0.140165289	0.140165289	0.011665326	C00042	30031
R-HSA-8957322	Metabolism of steroids	0.080808081	0.145481102	0.145481102	0.026682757	C00245	15891
R-HSA-418594	G alpha (i) signalling events	0.0579479	0.180498615	0.180498615	0.009117726	C00042	30031
R-HSA-8978868	Fatty acid metabolism	0.133971292	0.23583961	0.23583961	0.028828104	C00042	30031
R-HSA-388396	GPCR downstream signalling	0.082402977	0.238347107	0.238347107	0.020380799	C00042	30031
R-HSA-372790	Signaling by GPCR	0.084529506	0.246675671	0.246675671	0.032046125	C00042	30031
R-HSA-162582	Signal Transduction	0.129186603	0.343047091	0.343047091	0.174711719	C00042	30031
R-HSA-1430728	Metabolism	0.758639022	0.580221149	0.580221149	0.288549209	C00042;C00245	30031;15891

Supplementary Table 5a: Reactome pathway analysis of the upregulated metabolites in the stromal regions of pre-chemo PR tissues

PR= poor response; ER= excellent response

Name	Parent Folder	Percent Overlap	Overlapping Entities	Jaccard similarity	p-value
phytosiderophore biosynthesis template	Plants	5	succinate	0.052631579	0.00626794
Ketonogenesis	Carboxylic Acids Metabolism; Carboxylic Acids Metabolism (Metabolic Pathway); generic; generic	5	succinate	0.047619048	0.006963161
Respiratory Chain and Oxidative Phosphorylation	Electron Transport Chain; Electron Transport Chain (Metabolic Pathway); generic; generic	5	succinate	0.047619048	0.006963161
alpha Oxidation of Phytanic Acid	Lipids Metabolism; Lipids Metabolism (Metabolic Pathway); generic; generic	4	succinate	0.043478261	0.007658139
Clavulanate biosynthesis template	Secondary metabolism	4	succinate	0.043478261	0.007658139
Formalin and formate degradation template	catabolilsm	3	succinate	0.038461538	0.00870015
Fatty acid alpha-oxidation (peroxisomes) template	Eukaryots; Fatty acid biosynthesis & recycling	3	succinate	0.034482759	0.009741614
Respiratory chain and oxidative phosphorylation template	anabolism	3	succinate	0.029411765	0.01147617
Gibberellin biosynthesis template	Plant hormone biosynthesis	2	succinate	0.028571429	0.011822899
UMP pyrimidine precursor biosynthesis template	anabolism	2	succinate	0.023255814	0.01459454
Reductive TCA cycle template	metabolic cycles	2	succinate	0.021276596	0.015978901
Proline metabolism template	Aminoacid metabolism and interconvertion	2	succinate	0.020833333	0.016324839

Bile Acid Metabolism (Alternative Pathway)	Steroids Metabolism; Steroids Metabolism (Metabolic Pathway); generic; generic	2	taurine	0.020408163	0.016670716
Bile acids alternative biosynthesis template	Animal	2	taurine	0.020408163	0.016670716
scopolin and esculin biosynthesis template	Defense response	2	succinate	0.020408163	0.016670716
Ubiquinone-Q10 biosynthesis (eukaryotic) template	Eukaryots	2	succinate	0.02	0.017016533
Ubiquinone-8 biosynthesis template	anabolism	1	succinate	0.019607843	0.017362288
Ubiquinone-6 biosynthesis (eukaryotic) template	Eukaryots	1	succinate	0.019607843	0.017362288
Ubiquinone-9 biosynthesis (eukaryotic) template	Eukaryots	1	succinate	0.019230769	0.017707983
Cys degradation template	Aminoacid degradation	1	taurine	0.018867925	0.018053617
Ubiquinone-7 biosynthesis template	anabolism	1	succinate	0.018867925	0.018053617
Mutations in Krebs Cycle Enzymes in Cancer	Hallmarks of Cancer (7): Deregulated Metabolism; Hallmarks of Cancer (7): Deregulated Metabolism (Hallmarks of Cancer); mitochondria; oncogenic; oncogenic	1	succinate	0.017857143	0.018266357
Selenoamino acid metabolism template	Aminoacid biosynthesis	1	succinate	0.018518519	0.018399191
HIF1A as Master Regulator of Angiogenesis in Cancer	Hallmarks of Cancer (5): Inducing Angiogenesis; Hallmarks of Cancer (5): Inducing Angiogenesis (Hallmarks of Cancer); cardiovascular system	1	succinate	0.01754386	0.018598472
Pantothenate-CoA biosynthesis template	anabolism	1	taurine	0.018181818	0.018744703

Supplementary Table 5b: Pathway studio pathway analysis of the upregulated metabolites in the stromal regions of pre-chemo PR tissues

PR= poor response; ER= excellent response

Pathway identifier	Pathway name	Entities ratio	Entities pValue	Entities FDR	Reactions ratio	Submitted entities found	Mapped entities
R-HSA-70635	Urea cycle	0.011695906	9.34E-05	0.00769738	0.001072674	C00122;C00049;C00025	29991;29985;29806
R-HSA-15869	Metabolism of nucleotides	0.082402977	2.33E-04	0.00769738	0.018771789	C00122;C00262;C00106;C00049;C00025	29991;17368;29985;29806;17568
R-HSA-5619067	Defective SLC1A1 is implicated in schizophrenia 18 (SCZD18) and dicarboxylic aminoaciduria (DCBXA)	0.003189793	2.93E-04	0.00769738	1.34E-04	C00049;C00025	29991;29985
R-HSA-5619062	Defective SLC1A3 causes episodic ataxia 6 (EA6)	0.003189793	2.93E-04	0.00769738	1.34E-04	C00049;C00025	29991;29985
R-HSA-8963691	Phenylalanine and tyrosine metabolism	0.017012228	3.08E-04	0.00769738	0.00160901	C00122;C00049;C00025	29991;29985;29806
R-HSA-70263	Gluconeogenesis	0.017012228	3.08E-04	0.00769738	0.003486189	C00049;C00158;C00025	29991;29985;30769
R-HSA-73817	Purine ribonucleoside monophosphate biosynthesis	0.019670388	4.30E-04	0.00946493	0.002145347	C00122;C00049;C00025	29991;29985;29806
R-HSA-425407	SLC-mediated transmembrane transport	0.087719298	5.44E-04	0.010335172	0.025610083	C00262;C00106;C00049;C00158;C00025	29991;17368;29985;30769;17568
R-HSA-8956320	Nucleotide biosynthesis	0.024986709	8.63E-04	0.014668564	0.002949853	C00122;C00049;C00025	29991;29985;29806
R-HSA-70326	Glucose metabolism	0.022328549	0.001358819	0.020382288	0.006436042	C00049;C00158;C00025	29991;29985;30769
R-HSA-8963684	Tyrosine catabolism	0.007974482	0.002035751	0.024429017	8.05E-04	C00122;C00025	29985;29806
R-HSA-73929	Base-Excision Repair, AP Site Formation	0.008506114	0.002035751	0.024429017	0.003888442	C00262;C00106	17368;17568
R-HSA-382551	Transport of small molecules	0.116427432	0.002386642	0.026253062	0.054035935	C00262;C00106;C00049;C00158;C00025	29991;17368;29985;30769;17568
R-HSA-500753	Pyrimidine biosynthesis	0.011164274	0.003467911	0.034679108	8.05E-04	C00049;C00025	29991;29985
R-HSA-83936	Transport of nucleosides and free purine and pyrimidine bases across the plasma membrane	0.011164274	0.003467911	0.034679108	0.002145347	C00262;C00106	17368;17568
R-HSA-8963693	Aspartate and asparagine metabolism	0.013290803	0.005256768	0.042485707	0.001474926	C00049;C00025	29991;29985
R-HSA-8964539	Glutamate and glutamine metabolism	0.014354067	0.006069387	0.042485707	0.001877179	C00049;C00025	29991;29985
R-HSA-379724	tRNA Aminoacylation	0.013822435	0.006069387	0.042485707	0.005631537	C00049;C00025	29991;29985
R-HSA-379716	Cytosolic tRNA aminoacylation	0.013822435	0.006069387	0.042485707	0.002815768	C00049;C00025	29991;29985
R-HSA-379726	Mitochondrial tRNA aminoacylation	0.013822435	0.006069387	0.042485707	0.002815768	C00049;C00025	29991;29985
R-HSA-71403	Citric acid cycle (TCA cycle)	0.014885699	0.006496104	0.042897737	0.002279431	C00122;C00158	18012;30769
R-HSA-73884	Base Excision Repair	0.013290803	0.006936296	0.042897737	0.005363368	C00262;C00106	17368;17568

R-HSA-8849932	Synaptic adhesion-like molecules	0.001063264	0.008579547	0.042897737	4.02E-04	C00025	29985
R-HSA-6794362	Protein-protein interactions at synapses	0.001063264	0.008579547	0.042897737	4.02E-04	C00025	29985
R-HSA-500657	Presynaptic function of Kainate receptors	0.001063264	0.008579547	0.042897737	2.68E-04	C00025	29985
R-HSA-5619076	Defective SLC17A8 causes autosomal dominant deafness 25 (DFNA25)	0.001063264	0.008579547	0.042897737	1.34E-04	C00025	29985
R-HSA-72766	Translation	0.018607124	0.012640319	0.063201595	0.011128989	C00049;C00025	29991;29985
R-HSA-451307	Activation of Na-permeable kainate receptors	0.001594896	0.012844734	0.06422367	2.68E-04	C00025	29985
R-HSA-5578999	Defective GCLC causes HAGGSD	0.001594896	0.012844734	0.06422367	1.34E-04	C00025	29985
R-HSA-8956321	Nucleotide salvage	0.024455077	0.015734109	0.06837441	0.002949853	C00262;C00106	17368;17568
R-HSA-888568	GABA synthesis	0.002126528	0.017093602	0.06837441	2.68E-04	C00025	29985
R-HSA-433137	Sodium-coupled sulphate, di- and tri-carboxylate transporters	0.002126528	0.017093602	0.06837441	6.70E-04	C00158	30769
R-HSA-9729555	Sensory perception of sour taste	0.002126528	0.017093602	0.06837441	4.02E-04	C00158	30769
R-HSA-8964540	Alanine metabolism	0.002126528	0.021326207	0.076190156	5.36E-04	C00025	29985
R-HSA-71406	Pyruvate metabolism and Citric Acid (TCA) cycle	0.023923445	0.023570587	0.076190156	0.004692947	C00122;C00158	18012;30769
R-HSA-442982	Ras activation upon Ca2+ influx through NMDA receptor	0.003189793	0.025542603	0.076190156	2.68E-04	C00025	29985
R-HSA-210455	Astrocytic Glutamate-Glutamine Uptake And Metabolism	0.003189793	0.025542603	0.076190156	4.02E-04	C00025	29985
R-HSA-112313	Neurotransmitter uptake and metabolism In glial cells	0.003189793	0.025542603	0.076190156	4.02E-04	C00025	29985
R-HSA-9617324	Negative regulation of NMDA receptor-mediated neuronal transmission	0.003189793	0.025542603	0.076190156	5.36E-04	C00025	29985
R-HSA-9664535	LTC4-CYSLTR mediated IL4 production	0.003721425	0.029742844	0.076190156	4.02E-04	C00025	29985
R-HSA-110331	Cleavage of the damaged purine	0.003721425	0.029742844	0.076190156	0.001206758	C00262	17368
R-HSA-73927	Depurination	0.003721425	0.029742844	0.076190156	0.001206758	C00262	17368

R-HSA-425397	Transport of vitamins, nucleosides, and related molecules	0.034556087	0.030106268	0.076190156	0.005229284	C00262;C00106	17368;17568
R-HSA-73894	DNA Repair	0.029239766	0.030106268	0.076190156	0.020783052	C00262;C00106	17368;17568
R-HSA-9717189	Sensory perception of taste	0.049441786	0.03111956	0.076190156	0.003352105	C00049;C00158;C00025	29991;29985;30769
R-HSA-9026762	Biosynthesis of maresin conjugates in tissue regeneration (MCTR)	0.004253057	0.033926984	0.076190156	5.36E-04	C00025	29985
R-HSA-71387	Metabolism of carbohydrates	0.081871345	0.035088259	0.076190156	0.030705283	C00049;C00158;C00025	29991;29985;30769
R-HSA-425393	Transport of inorganic cations/anions and amino acids/oligopeptides	0.030834662	0.036364335	0.076190156	0.010056315	C00049;C00025	29991;29985
R-HSA-1428517	The citric acid (TCA) cycle and respiratory electron transport	0.031897927	0.037297691	0.076190156	0.008447305	C00122;C00158	18012;30769
R-HSA-8955332	Carboxyterminal post-translational modifications of tubulin	0.004784689	0.038095078	0.076190156	8.05E-04	C00025	29985
R-HSA-110329	Cleavage of the damaged pyrimidine	0.004784689	0.038095078	0.076190156	0.002681684	C00106	17568
R-HSA-73928	Depyrimidination	0.004784689	0.038095078	0.076190156	0.002681684	C00106	17568
R-HSA-442742	CREB1 phosphorylation through NMDA receptor-mediated activation of RAS signaling	0.005316321	0.042247179	0.084494359	8.05E-04	C00025	29985
R-HSA-916853	Degradation of GABA	0.005316321	0.046383342	0.090844365	4.02E-04	C00025	29985
R-HSA-399710	Activation of AMPA receptors	0.001594896	0.050503619	0.090844365	6.70E-04	C00025	29985
R-HSA-451308	Activation of Ca-permeable Kainate Receptor	0.001594896	0.050503619	0.090844365	2.68E-04	C00025	29985
R-HSA-451326	Activation of kainate receptors upon glutamate binding	0.002126528	0.054608063	0.090844365	8.05E-04	C00025	29985
R-HSA-451306	Ionotropic activity of kainate receptors	0.002126528	0.054608063	0.090844365	5.36E-04	C00025	29985
R-HSA-70688	Proline catabolism	0.005847953	0.054608063	0.090844365	5.36E-04	C00025	29985
R-HSA-5619102	SLC transporter disorders	0.042530569	0.057876602	0.090844365	0.008313221	C00049;C00025	29991;29985
R-HSA-9620244	Long-term potentiation	0.003189793	0.06276967	0.090844365	8.05E-04	C00025	29985

R-HSA-9662360	Sensory processing of sound by inner hair cells of the cochlea	0.003189793	0.06276967	0.090844365	9.39E-04	C00025	29985
R-HSA-428643	Organic anion transporters	0.007974482	0.066826938	0.090844365	0.001206758	C00025	29985
R-HSA-438066	Unblocking of NMDA receptors, glutamate binding and activation	0.003189793	0.070868586	0.090844365	6.70E-04	C00025	29985
R-HSA-210500	Glutamate Neurotransmitter Release Cycle	0.004253057	0.070868586	0.090844365	0.001072674	C00025	29985
R-HSA-399721	Glutamate binding, activation of AMPA receptors and synaptic plasticity	0.004253057	0.070868586	0.090844365	0.001206758	C00025	29985
R-HSA-5676934	Protein repair	0.006911217	0.070868586	0.090844365	9.39E-04	C00049	29991
R-HSA-200425	Carnitine metabolism	0.00744285	0.070868586	0.090844365	0.001072674	C00158	30769
R-HSA-9026766	Biosynthesis of protectin and resolvin conjugates in tissue regeneration (PCTR and RCTR)	0.009037746	0.070868586	0.090844365	0.001072674	C00025	29985
R-HSA-174403	Glutathione synthesis and recycling	0.00744285	0.074894667	0.090844365	0.001206758	C00025	29985
R-HSA-9659379	Sensory processing of sound	0.004784689	0.074894667	0.090844365	0.001743095	C00025	29985
R-HSA-8956319	Nucleotide catabolism	0.056353004	0.07546551	0.090844365	0.008447305	C00262;C00106	17368;17568
R-HSA-977347	Serine biosynthesis	0.008506114	0.078905233	0.090844365	9.39E-04	C00025	29985
R-HSA-5619115	Disorders of transmembrane transporters	0.051568315	0.080432685	0.090844365	0.010726736	C00049;C00025	29991;29985
R-HSA-9026395	Biosynthesis of DHA-derived sulfido conjugates	0.011164274	0.08688003	0.090844365	0.001743095	C00025	29985
R-HSA-8964208	Phenylalanine metabolism	0.011164274	0.08688003	0.090844365	8.05E-04	C00049	29991
R-HSA-446210	Synthesis of UDP-N-acetyl-glucosamine	0.011164274	0.08688003	0.090844365	9.39E-04	C00025	29985
R-HSA-2024096	HS-GAG degradation	0.011695906	0.090844365	0.090844365	0.001743095	C00049	29991
R-HSA-917937	Iron uptake and transport	0.011164274	0.090844365	0.090844365	0.003218021	C00158	30769
R-HSA-73614	Pyrimidine salvage	0.012227539	0.094793394	0.094793394	0.001340842	C00106	17568
R-HSA-70921	Histidine catabolism	0.011695906	0.10654916	0.10654916	0.001072674	C00025	29985
R-HSA-5628897	TP53 Regulates Metabolic Genes	0.013822435	0.10654916	0.10654916	0.001877179	C00025	29985

R-HSA-888590	GABA synthesis, release, reuptake and degradation	0.009569378	0.11431075	0.11431075	0.001340842	C00025	29985
R-HSA-9709957	Sensory Perception	0.101010101	0.115797662	0.115797662	0.018369536	C00049;C00158;C00025	29991;29985;30769
R-HSA-196757	Metabolism of folate and pterines	0.015417331	0.118169022	0.118169022	0.004022526	C00025	29985
R-HSA-5423646	Aflatoxin activation and detoxification	0.015417331	0.118169022	0.118169022	0.002413516	C00025	29985
R-HSA-71291	Metabolism of amino acids and derivatives	0.151515152	0.12439913	0.12439913	0.037543577	C00122;C00049;C00025	29991;29985;29806
R-HSA-2142691	Synthesis of Leukotrienes (LT) and Eoxins (EX)	0.016480595	0.125840776	0.125840776	0.0025476	C00025	29985
R-HSA-74217	Purine salvage	0.017012228	0.129654358	0.129654358	0.00160901	C00262	17368
R-HSA-5673001	RAF/MAP kinase cascade	0.013290803	0.129654358	0.129654358	0.006838294	C00025	29985
R-HSA-5684996	MAPK1/MAPK3 signaling	0.013290803	0.129654358	0.129654358	0.007240547	C00025	29985
R-HSA-5683057	MAPK family signaling cascades	0.013290803	0.129654358	0.129654358	0.009117726	C00025	29985
R-HSA-438064	Post NMDA receptor activation events	0.006379585	0.133453145	0.133453145	0.004692947	C00025	29985
R-HSA-1638091	Heparan sulfate/heparin (HS-GAG) metabolism	0.01754386	0.133453145	0.133453145	0.003888442	C00049	29991
R-HSA-71064	Lysine catabolism	0.017012228	0.141006533	0.141006533	0.001743095	C00025	29985
R-HSA-156590	Glutathione conjugation	0.016480595	0.141006533	0.141006533	0.001877179	C00025	29985
R-HSA-71240	Tryptophan catabolism	0.018075492	0.141006533	0.141006533	0.002145347	C00025	29985
R-HSA-352230	Amino acid transport across the plasma membrane	0.018607124	0.141006533	0.141006533	0.004827031	C00025	29985
R-HSA-442755	Activation of NMDA receptors and postsynaptic events	0.008506114	0.155937962	0.155937962	0.006167873	C00025	29985
R-HSA-73621	Pyrimidine catabolism	0.020733652	0.155937962	0.155937962	0.002279431	C00106	17568
R-HSA-9717207	Sensory perception of sweet, bitter, and umami (glutamate) taste	0.047315258	0.156478755	0.156478755	0.002815768	C00049;C00025	29991;29985
R-HSA-70895	Branched-chain amino acid catabolism	0.019670388	0.159634578	0.159634578	0.003083937	C00025	29985
R-HSA-1614558	Degradation of cysteine and homocysteine	0.02020202	0.170638237	0.170638237	0.002949853	C00025	29985
R-HSA-70171	Glycolysis	0.017012228	0.174277556	0.174277556	0.002949853	C00158	30769

R-HSA-389661	Glyoxylate metabolism and glycine degradation	0.021796917	0.177902673	0.177902673	0.003083937	C00049	29991
R-HSA-112314	Neurotransmitter receptors and postsynaptic signal transmission	0.012759171	0.185110495	0.185110495	0.011128989	C00025	29985
R-HSA-75105	Fatty acyl-CoA biosynthesis	0.023391813	0.185110495	0.185110495	0.003083937	C00158	30769
R-HSA-499943	Interconversion of nucleotide di- and triphosphates	0.026581606	0.195816919	0.195816919	0.004424779	C00025	29985
R-HSA-1630316	Glycosaminoglycan metabolism	0.030834662	0.223758679	0.223758679	0.010458568	C00049	29991
R-HSA-112310	Neurotransmitter release cycle	0.026049973	0.227189996	0.227189996	0.004827031	C00025	29985
R-HSA-196807	Nicotinate metabolism	0.028176502	0.227189996	0.227189996	0.003888442	C00025	29985
R-HSA-74259	Purine catabolism	0.031897927	0.237403196	0.237403196	0.004424779	C00262	17368
R-HSA-5579029	Metabolic disorders of biological oxidation enzymes	0.028176502	0.237403196	0.237403196	0.0050952	C00025	29985
R-HSA-420499	Class C/3 (Metabotropic glutamate/pherom one receptors)	0.03030303	0.250834105	0.250834105	9.39E-04	C00025	29985
R-HSA-446219	Synthesis of substrates in N-glycan biosythesis	0.036150984	0.260768871	0.260768871	0.006570126	C00025	29985
R-HSA-9018677	Biosynthesis of DHA-derived SPMs	0.038277512	0.270586357	0.270586357	0.007776884	C00025	29985
R-HSA-1614635	Sulfur amino acid metabolism	0.034556087	0.273833	0.273833	0.004827031	C00025	29985
R-HSA-416476	G alpha (q) signalling events	0.034556087	0.277066791	0.277066791	0.00415661	C00025	29985
R-HSA-446193	Biosynthesis of the N-glycan precursor (dolichol lipid-linked oligosaccharide, LLO) and transfer to a nascent protein	0.043062201	0.302479781	0.302479781	0.008447305	C00025	29985
R-HSA-3928662	EPHB-mediated forward signaling	0.005316321	0.308707599	0.308707599	0.002949853	C00025	29985
R-HSA-2682334	EPH-Ephrin signaling	0.005316321	0.308707599	0.308707599	0.0050952	C00025	29985
R-HSA-392499	Metabolism of proteins	0.125465178	0.310668384	0.310668384	0.061410566	C00049;C00025	29991;29985
R-HSA-425366	Transport of bile salts and organic acids, metal ions and amine compounds	0.040935673	0.314885941	0.314885941	0.008983642	C00158	30769
R-HSA-8978868	Fatty acid metabolism	0.133971292	0.315741454	0.315741454	0.028828104	C00158;C00025	29985;30769
R-HSA-422475	Axon guidance	0.009569378	0.333127544	0.333127544	0.017833199	C00025	29985

R-HSA-9675108	Nervous system development	0.009569378	0.333127544	0.333127544	0.017833199	C00025	29985
R-HSA-112315	Transmission across Chemical Synapses	0.037214248	0.350936135	0.350936135	0.017967283	C00025	29985
R-HSA-112316	Neuronal System	0.037214248	0.350936135	0.350936135	0.02105122	C00025	29985
R-HSA-1266738	Developmental Biology	0.019138756	0.385290199	0.385290199	0.024805578	C00025	29985
R-HSA-2142753	Arachidonic acid metabolism	0.0579479	0.385290199	0.385290199	0.010324484	C00025	29985
R-HSA-3700989	Transcriptional Regulation by TP53	0.02020202	0.385290199	0.385290199	0.009519979	C00025	29985
R-HSA-446203	Asparagine N-linked glycosylation	0.061669325	0.404574826	0.404574826	0.017699115	C00025	29985
R-HSA-212436	Generic Transcription Pathway	0.029239766	0.431191443	0.431191443	0.017430947	C00025	29985
R-HSA-73857	RNA Polymerase II Transcription	0.030834662	0.438967439	0.438967439	0.021319389	C00025	29985
R-HSA-74160	Gene expression (Transcription)	0.035087719	0.461731643	0.461731643	0.025878252	C00025	29985
R-HSA-9018678	Biosynthesis of specialized proresolving mediators (SPMs)	0.074428495	0.464209601	0.464209601	0.016626441	C00025	29985
R-HSA-196849	Metabolism of water-soluble vitamins and cofactors	0.069112174	0.466677413	0.466677413	0.019308126	C00025	29985
R-HSA-9662851	Anti-inflammatory response favouring Leishmania parasite infection	0.018607124	0.486059026	0.486059026	0.003486189	C00025	29985
R-HSA-9664433	Leishmania parasite growth and survival	0.018607124	0.486059026	0.486059026	0.003486189	C00025	29985
R-HSA-500792	GPCR ligand binding	0.072833599	0.493164088	0.493164088	0.011665326	C00025	29985
R-HSA-156580	Phase II - Conjugation of compounds	0.080808081	0.518474981	0.518474981	0.009654063	C00025	29985
R-HSA-5668914	Diseases of metabolism	0.085061138	0.533990808	0.533990808	0.024671494	C00025	29985
R-HSA-9658195	Leishmania infection	0.03030303	0.536170681	0.536170681	0.008447305	C00025	29985
R-HSA-597592	Post-translational protein modification	0.092503987	0.55747605	0.55747605	0.038079914	C00025	29985
R-HSA-418594	G alpha (i) signalling events	0.0579479	0.591705421	0.591705421	0.009117726	C00025	29985
R-HSA-196854	Metabolism of vitamins and cofactors	0.099415205	0.593643525	0.593643525	0.027353178	C00025	29985
R-HSA-1430728	Metabolism	0.758639022	0.633504919	0.633504919	0.288549209	C00122;C00262;C00106;C00049;C00158;C00025	29991;17368;29985;18012;30769;29806;17568
R-HSA-1643685	Disease	0.18394471	0.657004584	0.657004584	0.116519174	C00049;C00025	29991;29985
R-HSA-5663205	Infectious disease	0.066985646	0.697487564	0.697487564	0.043979619	C00025	29985

R-HSA-388396	GPCR downstream signalling	0.082402977	0.706297982	0.706297982	0.020380799	C00025	29985
R-HSA-372790	Signaling by GPCR	0.084529506	0.720476086	0.720476086	0.032046125	C00025	29985
R-HSA-211859	Biological oxidations	0.173312068	0.795595552	0.795595552	0.025073746	C00025	29985
R-HSA-162582	Signal Transduction	0.129186603	0.849025333	0.849025333	0.174711719	C00025	29985
R-HSA-556833	Metabolism of lipids	0.337054758	0.90258093	0.90258093	0.11893269	C00158;C00025	29985;30769

Supplementary table 6a: Reactome pathway analysis of downregulated metabolites in the epithelial regions post-chemo versus pre-chemo ER tissue

PR= poor response; ER= excellent response

Name	Parent Folder	Percent Overlap	Overlapping Entities	Jaccard similarity	Hit type	p-value
Brain Dysfunction in Phenylketonuria	Phenylketonuria; Phenylketonuria (Endocrine and Metabolic Diseases); Phenylketonuria (Endocrine and Metabolic Diseases); mitochondria; nervous system	9	citrate;glutamate;aspartate	0.078947368	Disease	1.28595E-06
Metabolic Reprogramming in Cancer: Overview	Hallmarks of Cancer (7): Deregulated Metabolism; Hallmarks of Cancer (7): Deregulated Metabolism (Hallmarks of Cancer); oncogenic; oncogenic	3	citrate;aspartate;fumarate;glutamate	0.033333333	Pathological Process	2.02266E-06
Hyperammonemia Type I	Urea Cycle Disorders; Urea Cycle Disorders (Urology/Nephrology Diseases); mitochondria	7	fumarate;glutamate;aspartate	0.0625	Disease	2.96904E-06
Hyperammonemia Type II	Urea Cycle Disorders; Urea Cycle Disorders (Urology/Nephrology Diseases); generic; mitochondria	6	fumarate;glutamate;aspartate	0.06	Disease	3.42357E-06
Citrullinemia Type I	Urea Cycle Disorders; Urea Cycle Disorders (Urology/Nephrology Diseases); mitochondria	6	fumarate;glutamate;aspartate	0.058823529	Disease	3.66721E-06
Argininosuccinic Aciduria in Urea Cycle Disorders	Urea Cycle Disorders; Urea Cycle Disorders (Urology/Nephrology Diseases)	6	glutamate;aspartate;fumarate	0.056603774	Disease	4.18848E-06
Citrullinemia Type II	Urea Cycle Disorders; Urea Cycle Disorders (Urology/Nephrology Diseases); mitochondria	5	glutamate;aspartate;fumarate	0.053571429	Disease	5.05912E-06
Argininemia	Urea Cycle Disorders; Urea Cycle Disorders (Urology/Nephrology Diseases)	5	glutamate;aspartate;fumarate	0.049180328	Disease	6.7634E-06
Purine metabolism template	metabolic cycles	2	hypoxanthine;aspartate;fumarate;glutamate	0.028571429	Metabolic Pathway	9.73246E-06
UMP pyrimidine precursor biosynthesis template	anabolism	7	fumarate;glutamate;aspartate	0.0625	Metabolic Pathway	1.25286E-05

IMP purine precursor biosynthesis template	anabolism	5	glutamate;aspartate;fumarate	0.049180328	Metabolic Pathway	2.84363E-05
Purine Metabolism	Nucleotides Metabolism; Nucleotides Metabolism (Metabolic Pathway); generic; generic	2	hypoxanthine;aspartate;fumarate;glutamate	0.021857923	Metabolic Pathway	2.91961E-05
Glutamine in Cancer Metabolism	Hallmarks of Cancer (7): Deregulated Metabolism; Hallmarks of Cancer (7): Deregulated Metabolism (Hallmarks of Cancer); oncogenic; oncogenic	5	glutamate;aspartate;fumarate	0.045454545	Pathological Process	2.96876E-05
Asp/Asn metabolism template	Aminoacid metabolism and interconvertion	5	glutamate;aspartate;fumarate	0.045454545	Metabolic Pathway	3.69938E-05
Tyrosine degradation template	Aminoacid degradation	4	glutamate;aspartate;fumarate	0.043478261	Metabolic Pathway	4.28621E-05
Aspartate Metabolism	Amino Acids Metabolism; Amino Acids Metabolism (Metabolic Pathway); generic; generic	4	glutamate;aspartate;fumarate	0.041666667	Metabolic Pathway	4.93131E-05
Nicotinate and NAD metabolism template	metabolic cycles	3	glutamate;aspartate;fumarate	0.034482759	Metabolic Pathway	9.12203E-05
Urea cycle and Arginine metabolism template	Aminoacid metabolism and interconvertion	3	glutamate;aspartate;fumarate	0.031914894	Metabolic Pathway	0.000116892
Urea Cycle and Arginine Metabolism	Amino Acids Metabolism; Amino Acids Metabolism (Metabolic Pathway); generic; generic	3	glutamate;aspartate;fumarate	0.03125	Metabolic Pathway	0.000125009
Malonate, Propanoate and beta-Alanine Metabolism	Carboxylic Acids Metabolism; Carboxylic Acids Metabolism (Metabolic Pathway); generic; generic	3	aspartate;glutamate;uracil	0.028571429	Metabolic Pathway	0.000166109
IAA storage via aminoacid conjugation template	Plant hormone storage and degradation	10	glutamate;aspartate	0.074074074	Metabolic Pathway	0.000240139

Pyrimidine Metabolism	Nucleotides Metabolism; Nucleotides Metabolism (Metabolic Pathway); generic; generic	2	aspartate;glutamate;uracil	0.024793388	Metabolic Pathway	0.000259277
Disorders of Phenylalanine and Tyrosine Metabolism	Disorders of Phenylalanine and Tyrosine Metabolism; Disorders of Phenylalanine and Tyrosine Metabolism (Endocrine and Metabolic Diseases); Disorders of Phenylalanine and Tyrosine Metabolism (Endocrine and Metabolic Diseases); nervous system	4	glutamate;fumarate	0.036363636	Disease	0.000592268
Tyrosine byosynthesis template	Aminoacid biosynthesis	5	glutamate;aspartate	0.043478261	Metabolic Pathway	0.000926235
Alanine metabolism template	Aminoacid metabolism and interconvertion	5	glutamate;aspartate	0.043478261	Metabolic Pathway	0.000926235

Supplementary table 6b: Pathway studio pathway analysis of downregulated metabolites in epithelial regions of post-chemo versus pre-chemo ER tissues

PR= poor response; ER= excellent response

Pathway identifier	Pathway name	Entities ratio	Entities pValue	Entities FDR	Reactions ratio	Submitted entities found	Mapped entities
R-HSA-174403	Glutathione synthesis and recycling	0.00744285	0.005431738	0.123030157	0.001206758	C00051	57925;16856
R-HSA-5628897	TP53 Regulates Metabolic Genes	0.013822435	0.011019795	0.123030157	0.001877179	C00064;C00051	58359;57925
R-HSA-8963693	Aspartate and asparagine metabolism	0.013290803	0.011019795	0.123030157	0.001474926	C00064;C00245	58359;15891
R-HSA-4085023	Defective GFPT1 causes CMSTA1	0.001063264	0.012369014	0.123030157	1.34E-04	C00064	58359
R-HSA-15869	Metabolism of nucleotides	0.082402977	0.013116498	0.123030157	0.018771789	C00385;C00064;C00294;C00051	17596;58359;17712;57925
R-HSA-1222538	nitric oxide produced by macrophages	0.013290803	0.01356634	0.123030157	0.001206758	C00051	57925;16856
R-HSA-70268	Pyruvate metabolism	0.013822435	0.016345867	0.123030157	0.001877179	C00051	57925;16856
R-HSA-442660	neurotransmitter transporters	0.017012228	0.016345867	0.123030157	0.002011263	C00064;C00245	58359;15891
R-HSA-156590	Glutathione conjugation	0.016480595	0.019350262	0.123030157	0.001877179	C00051	57925;16856
R-HSA-352230	across the plasma membrane	0.018607124	0.019350262	0.123030157	0.004827031	C00064;C00245	58359;15891
R-HSA-3299685	Reactive Oxygen Species	0.015948963	0.021474194	0.123030157	0.004558863	C00051	57925;16856
R-HSA-1222499	responses of Mtb to phagocytosis	0.02020202	0.027190776	0.123030157	0.003754358	C00051	57925;16856
R-HSA-1614558	cysteine and homocysteine	0.02020202	0.028401571	0.123030157	0.002949853	C00051;C00245	15891;16856
R-HSA-2162123	Prostaglandins (PG) and Thromboxanes	0.022860181	0.029634214	0.123030157	0.004290695	C00051	57925;16856
R-HSA-5579022	Defective GGT1 causes GLUTH	0.001063264	0.03668445	0.123030157	1.34E-04	C00051	16856
R-HSA-210455	Glutamine Uptake And Metabolism	0.003189793	0.03668445	0.123030157	4.02E-04	C00064	58359
R-HSA-112313	uptake and metabolism In glial cells	0.003189793	0.03668445	0.123030157	4.02E-04	C00064	58359
R-HSA-499943	nucleotide di- and triphosphates	0.026581606	0.037473778	0.123030157	0.004424779	C00064;C00051	58359;57925
R-HSA-9711123	Cellular response to chemical stress	0.023391813	0.040249648	0.123030157	0.007374631	C00051	57925;16856
R-HSA-5619063	causes histiocytosis-lymphadenopathy plus	0.003721425	0.042676346	0.123030157	1.34E-04	C00294	17596
R-HSA-71406	and Citric Acid (TCA) cycle	0.023923445	0.047525668	0.123030157	0.004692947	C00051	57925;16856
R-HSA-9026762	maresin conjugates in tissue regeneration	0.004253057	0.048633822	0.123030157	5.36E-04	C00051	57925
R-HSA-9635486	Infection with Mycobacterium tuberculosis	0.02764487	0.050565363	0.123030157	0.005363368	C00051	57925;16856
R-HSA-177162	Conjugation of phenylacetate with glutamine	0.004253057	0.05455706	0.123030157	2.68E-04	C00064	58359
R-HSA-9694548	Maturation of spike protein	0.003189793	0.05455706	0.123030157	4.02E-04	C00221	15903
R-HSA-74259	Purine catabolism	0.031897927	0.055257323	0.123030157	0.004424779	C00385;C00294	17596;17712
R-HSA-9683686	Maturation of spike protein	0.003721425	0.06044624	0.123030157	5.36E-04	C00221	15903
R-HSA-196791	Vitamin D (calciferol) metabolism	0.005847953	0.066301541	0.123030157	0.002413516	C01164	17759
R-HSA-425393	Transport of inorganic cations/anions and amino acids/oligopeptides	0.030834662	0.071968507	0.123030157	0.010056315	C00064;C00245	58359;15891
R-HSA-2142712	Synthesis of 12-eicosatetraenoic acid derivatives	0.006379585	0.072123144	0.123030157	8.05E-04	C00051	57925
R-HSA-1614635	Sulfur amino acid metabolism	0.034556087	0.073724439	0.123030157	0.004827031	C00051;C00245	15891;16856
R-HSA-1428517	The citric acid (TCA) cycle and respiratory electron transport	0.031897927	0.073724439	0.123030157	0.008447305	C00051	57925;16856
R-HSA-156580	Phase II - Conjugation of compounds	0.080808081	0.075149902	0.123030157	0.009654063	C00064;C00051	58359;57925;16856
R-HSA-5619044	Defective SLC6A19 causes Hartnup disorder (HND)	0.006911217	0.077911224	0.123030157	1.34E-04	C00064	58359

R-HSA-5659735	Defective SLC6A19 causes Hartnup disorder (HND)	0.006911217	0.077911224	0.123030157	1.34E-04	C00064	58359
R-HSA-6807062	Cholesterol biosynthesis via lathosterol	0.005316321	0.077911224	0.123030157	5.36E-04	C01164	17759
R-HSA-9023661	Biosynthesis of E-series 18(R)-resolvins	0.00744285	0.08366596	0.123030157	9.39E-04	C00051	57925
R-HSA-2142688	Synthesis of 5-eicosatetraenoic acids	0.007974482	0.089387528	0.123030157	5.36E-04	C00051	57925
R-HSA-5661270	Formation of xylulose-5-phosphate	0.008506114	0.095076103	0.123030157	8.05E-04	C00379	17151
R-HSA-2408499	Formation of selenosugars for excretion	0.006379585	0.095076103	0.123030157	5.36E-04	C00051	57925
R-HSA-2142770	Synthesis of 15-eicosatetraenoic acid derivatives	0.008506114	0.095076103	0.123030157	5.36E-04	C00051	57925
R-HSA-8963684	Tyrosine catabolism	0.007974482	0.095076103	0.123030157	8.05E-04	C00051	57925
R-HSA-156587	Amino Acid conjugation	0.007974482	0.095076103	0.123030157	8.05E-04	C00064	58359
R-HSA-159424	Conjugation of carboxylic acids	0.007974482	0.095076103	0.123030157	8.05E-04	C00064	58359
R-HSA-425366	Transport of bile salts and organic acids, metal ions and amine compounds	0.040935673	0.097798	0.123030157	0.008983642	C00064;C00245	58359;15891
R-HSA-210500	Glutamate Neurotransmitter Release Cycle	0.004253057	0.100731859	0.123030157	0.001072674	C00064	58359
R-HSA-9026766	Biosynthesis of protectin and resolvin conjugates in tissue regeneration (PCTR and RCTR)	0.009037746	0.100731859	0.123030157	0.001072674	C00051	57925
R-HSA-2161541	Abacavir metabolism	0.009037746	0.100731859	0.123030157	9.39E-04	C00294	17596
R-HSA-9683701	Translation of Structural Proteins	0.00744285	0.100731859	0.123030157	0.001474926	C00221	15903
R-HSA-71291	Metabolism of amino acids and derivatives	0.151515152	0.101018195	0.123030157	0.037543577	C00064;C00051;C00245	58359;15891;57925;16856
R-HSA-2262752	Cellular responses to stress	0.04040404	0.103654444	0.123030157	0.022660231	C00051	57925;16856
R-HSA-425407	SLC-mediated transmembrane transport	0.087719298	0.105231406	0.123030157	0.025610083	C00064;C00294;C00245	17596;58359;15891
R-HSA-2408550	Metabolism of ingested H2SeO4 and H2SeO3 into H2Se	0.009569378	0.10635497	0.123030157	9.39E-04	C00051	57925
R-HSA-196836	Vitamin C (ascorbate) metabolism	0.009569378	0.10635497	0.123030157	0.001206758	C00051	57925
R-HSA-2161522	Abacavir transport and metabolism	0.009569378	0.10635497	0.123030157	0.001206758	C00294	17596
R-HSA-5619102	SLC transporter disorders	0.042530569	0.1116204	0.123030157	0.008313221	C00064;C00294	17596;58359
R-HSA-5683826	Surfactant metabolism	0.008506114	0.111945609	0.123030157	0.001072674	C00294	17596
R-HSA-9018896	Biosynthesis of E-series 18(S)-resolvins	0.01010101	0.111945609	0.123030157	0.001206758	C00051	57925
R-HSA-9020265	Biosynthesis of aspirin-triggered D-series resolvins	0.01010101	0.111945609	0.123030157	0.001340842	C00051	57925
R-HSA-8953897	Cellular responses to stimuli	0.041467305	0.113638745	0.123030157	0.026816841	C00051	57925;16856
R-HSA-204174	Regulation of pyruvate dehydrogenase (PDH) complex	0.00744285	0.117503947	0.123030157	4.02E-04	C00051	16856
R-HSA-1614517	Sulfide oxidation to sulfate	0.008506114	0.117503947	0.123030157	8.05E-04	C00051	16856

R-HSA-83936	Transport of nucleosides and free purine and pyrimidine bases across the plasma membrane	0.011164274	0.123030157	0.123030157	0.002145347	C00294	17596
R-HSA-9026395	Biosynthesis of DHA-derived sulfido conjugates	0.011164274	0.123030157	0.123030157	0.001743095	C00051	57925
R-HSA-500753	Pyrimidine biosynthesis	0.011164274	0.123030157	0.123030157	8.05E-04	C00064	58359
R-HSA-446210	Synthesis of UDP-N-acetyl-glucosamine	0.011164274	0.123030157	0.123030157	9.39E-04	C00064	58359
R-HSA-159418	Recycling of bile acids and salts	0.011164274	0.123030157	0.123030157	0.002279431	C00245	15891
R-HSA-9018676	Biosynthesis of D-series resolvins	0.012227539	0.13398687	0.13398687	0.001474926	C00051	57925
R-HSA-8956319	Nucleotide catabolism	0.056353004	0.142904853	0.142904853	0.008447305	C00385;C00294	17596;17712
R-HSA-9694635	Translation of Structural Proteins	0.009569378	0.1448171	0.1448171	0.001877179	C00221	15903
R-HSA-2142753	Arachidonic acid metabolism	0.0579479	0.147224052	0.147224052	0.010324484	C00051	57925;16856
R-HSA-3700989	Transcriptional Regulation by TP53	0.02020202	0.147224052	0.147224052	0.009519979	C00064;C00051	58359;57925
R-HSA-5609975	Diseases associated with glycosylation precursor biosynthesis	0.013822435	0.150185203	0.150185203	0.002145347	C00064	58359
R-HSA-5619115	Disorders of transmembrane transporters	0.051568315	0.151573903	0.151573903	0.010726736	C00064;C00294	17596;58359
R-HSA-9018679	Biosynthesis of EPA-derived SPMs	0.014354067	0.155522186	0.155522186	0.002279431	C00051	57925
R-HSA-8964539	Glutamate and glutamine metabolism	0.014354067	0.160828216	0.160828216	0.001877179	C00064	58359
R-HSA-379724	tRNA Aminoacylation	0.013822435	0.160828216	0.160828216	0.005631537	C00064	58359
R-HSA-379716	Cytosolic tRNA aminoacylation	0.013822435	0.160828216	0.160828216	0.002815768	C00064	58359
R-HSA-379726	Mitochondrial tRNA aminoacylation	0.013822435	0.160828216	0.160828216	0.002815768	C00064	58359
R-HSA-1643685	Disease	0.18394471	0.16532624	0.16532624	0.116519174	C00221;C00064;C00294;C00051	17596;58359;15903;57925;16856
R-HSA-5423646	Aflatoxin activation and detoxification	0.015417331	0.166103457	0.166103457	0.002413516	C00051	57925
R-HSA-2142691	Synthesis of Leukotrienes (LT) and Eoxins (EX)	0.016480595	0.176562224	0.176562224	0.0025476	C00051	57925
R-HSA-9678108	SARS-CoV-1 Infection	0.012759171	0.176562224	0.176562224	0.005363368	C00221	15903
R-HSA-74217	Purine salvage	0.017012228	0.181746077	0.181746077	0.00160901	C00294	17596
R-HSA-212436	Generic Transcription Pathway	0.029239766	0.185051764	0.185051764	0.017430947	C00064;C00051	58359;57925
R-HSA-8963691	Phenylalanine and tyrosine metabolism	0.017012228	0.186899791	0.186899791	0.00160901	C00051	57925
R-HSA-73857	RNA Polymerase II Transcription	0.030834662	0.191902057	0.191902057	0.021319389	C00064;C00051	58359;57925
R-HSA-9694516	SARS-CoV-2 Infection	0.014354067	0.192023527	0.192023527	0.00670421	C00221	15903
R-HSA-9679506	SARS-CoV Infections	0.015417331	0.202181705	0.202181705	0.012201663	C00221	15903
R-HSA-73817	Purine ribonucleoside monophosphate biosynthesis	0.019670388	0.207216464	0.207216464	0.002145347	C00064	58359
R-HSA-382551	Transport of small molecules	0.116427432	0.212124042	0.212124042	0.054035935	C00064;C00294;C00245	17596;58359;15891
R-HSA-74160	Gene expression (Transcription)	0.035087719	0.212696306	0.212696306	0.025878252	C00064;C00051	58359;57925
R-HSA-5663205	Infectious disease	0.066985646	0.213805001	0.213805001	0.043979619	C00221;C00051	15903;57925;16856
R-HSA-196849	Metabolism of water-soluble vitamins and cofactors	0.069112174	0.217360299	0.217360299	0.019308126	C00064;C00051	58359;57925
R-HSA-72766	Translation	0.018607124	0.227063636	0.227063636	0.011128989	C00064	58359
R-HSA-156581	Methylation	0.02020202	0.231953242	0.231953242	0.001743095	C00051	57925
R-HSA-8956321	Nucleotide salvage	0.024455077	0.251227528	0.251227528	0.002949853	C00294	17596

R-HSA-1222556	ROS and RNS production in phagocytes	0.024455077	0.251227528	0.251227528	0.006033789	C00051	57925
R-HSA-8956320	Nucleotide biosynthesis	0.024986709	0.255975825	0.255975825	0.002949853	C00064	58359
R-HSA-8957322	Metabolism of steroids	0.080808081	0.257457972	0.257457972	0.026682757	C01164;C00245	15891;17759
R-HSA-191273	Cholesterol biosynthesis	0.025518341	0.274692421	0.274692421	0.004558863	C01164	17759
R-HSA-5668914	Diseases of metabolism	0.085061138	0.286057761	0.286057761	0.024671494	C00064;C00051	58359;16856
R-HSA-3781865	Diseases of glycosylation	0.029771398	0.297476895	0.297476895	0.010324484	C00064	58359
R-HSA-193368	Synthesis of bile acids and bile salts via 7alpha-hydroxycholesterol	0.028708134	0.301953743	0.301953743	0.005229284	C00245	15891
R-HSA-112310	Neurotransmitter release cycle	0.026049973	0.310828539	0.310828539	0.004827031	C00064	58359
R-HSA-196807	Nicotinate metabolism	0.028176502	0.310828539	0.310828539	0.003888442	C00064	58359
R-HSA-2408522	Selenoamino acid metabolism	0.030834662	0.323945607	0.323945607	0.004424779	C00051	57925
R-HSA-5579029	Metabolic disorders of biological oxidation enzymes	0.028176502	0.323945607	0.323945607	0.0050952	C00051	16856
R-HSA-425397	Transport of vitamins, nucleosides, and related molecules	0.034556087	0.336831883	0.336831883	0.005229284	C00294	17596
R-HSA-211859	Biological oxidations	0.173312068	0.352898298	0.352898298	0.025073746	C00064;C00051	58359;57925;16856
R-HSA-446219	Synthesis of substrates in N-glycan biosythesis	0.036150984	0.353661009	0.353661009	0.006570126	C00064	58359
R-HSA-196854	Metabolism of vitamins and cofactors	0.099415205	0.355117316	0.355117316	0.027353178	C00064;C00051	58359;57925
R-HSA-9018677	Biosynthesis of DHA-derived SPMs	0.038277512	0.366023171	0.366023171	0.007776884	C00051	57925
R-HSA-446193	Biosynthesis of the N-glycan precursor (dolichol lipid-linked oligosaccharide, LLO) and transfer to a nascent protein	0.043062201	0.40567159	0.40567159	0.008447305	C00064	58359
R-HSA-192105	Synthesis of bile acids and bile salts	0.041998937	0.417111911	0.417111911	0.011128989	C00245	15891
R-HSA-194068	Bile acid and bile salt metabolism	0.042530569	0.420879946	0.420879946	0.013274336	C00245	15891
R-HSA-112315	Transmission across Chemical Synapses	0.037214248	0.464376499	0.464376499	0.017967283	C00064	58359
R-HSA-112316	Neuronal System	0.037214248	0.464376499	0.464376499	0.02105122	C00064	58359
R-HSA-392499	Metabolism of proteins	0.125465178	0.493768599	0.493768599	0.061410566	C00064;C00294	17596;58359
R-HSA-8978868	Fatty acid metabolism	0.133971292	0.50020819	0.50020819	0.028828104	C00051	57925;16856
R-HSA-446203	Asparagine N-linked glycosylation	0.061669325	0.527120607	0.527120607	0.017699115	C00064	58359
R-HSA-9018678	Biosynthesis of specialized proresolving mediators (SPMs)	0.074428495	0.593979251	0.593979251	0.016626441	C00051	57925
R-HSA-71387	Metabolism of carbohydrates	0.081871345	0.685630196	0.685630196	0.030705283	C00379	17151
R-HSA-597592	Post-translational protein modification	0.092503987	0.69198253	0.69198253	0.038079914	C00064	58359
R-HSA-556833	Metabolism of lipids	0.337054758	0.771628246	0.771628246	0.11893269	C00051;C01164;C00245	15891;17759;57925;16856
R-HSA-168249	Innate Immune System	0.06539075	0.781862492	0.781862492	0.056181282	C00051	57925
R-HSA-168256	Immune System	0.078681552	0.832031778	0.832031778	0.093188522	C00051	57925
R-HSA-1430728	Metabolism	0.758639022	0.934545845	0.934545845	0.288549209	C00385;C00064;C00294;C00051;C01164;C00379;C00245	17596;58359;15891;17759;17151;57925;17712;16856

Supplementary table 7a: Reactome pathway analysis of upregulated metabolites in epithelial regions of post-chemo versus pre-chemo ER s tissues

PR= poor response; ER= excellent response

Name	Parent Folder	Percent Overlap	Overlapping Entities	p-value	Jaccard similarity
Unsaturated hydroxy fatty acids	Fatty Acyl & Other Lipids	0	Leucate;2-hydroxyvaleric acid	3.25745E-06	0.002967359
Adenosine Deaminase Polymorphism	Sleep Dysregulation (Neurological	7	inosine;glutamine	0.000103397	0.048780488
Uric Acid Synthesis in Gout	System Diseases); digestive system	6	xanthine;inosine	0.000157594	0.042553191
Purine metabolism template	metabolic cycles	2	inosine;glutamine;xanthine	0.001356127	0.020134228
Pentose-Phosphate Shunt	Metabolism; Carbohydrates Metabolism (Metabolic	4	gluconate;beta-D-glucose	0.002718952	0.032786885
Purine Metabolism	Metabolism; Nucleotides Metabolism (Metabolic	1	inosine;glutamine;xanthine	0.003003321	0.015625
Amino sugars synthesis (bacteria) template	Bacteria	3	glutamine;beta-D-glucose	0.003333873	0.03030303
Cholesteryl ester (CE, Che)	Neutral Lipids	0	7-dehydrocholesterol	0.003699583	0.001256281
Pentose phosphate shunt template	catabolilsm	3	gluconate;beta-D-glucose	0.003868543	0.028571429
Ascorbate Biosyntesis	Metabolism; Cofactors and Vitamins	2	ascorbic acid;xylitol	0.006032341	0.023809524
Amino sugars synthesis (Eukaryots) template	Eukaryots	2	glutamine;beta-D-glucose	0.006032341	0.023809524
Phenylalanine metabolism template	Aminoacid metabolism and interconvertion	2	ascorbic acid;glutamine	0.006203981	0.023529412
Cellulose degradation template	catabolilsm	16	beta-D-glucose	0.010421099	0.045454545
Receptor/SGK1 Signaling	Prostate Cancer (Urology/Nephrology	5	glutamine	0.011056805	0.03030303
Epinephrine/Norepinephrine Release	Release Cycles; Neurotransmitter	5	ascorbic acid	0.01154225	0.027777778
Glutamate Release/Uptake Circle	Hormones; Amino Acid Derived Hormones	4	glutamine	0.01269279	0.026315789
Glutamate Release	Release Cycles; Neurotransmitter	4	glutamine	0.013267808	0.025641026
Sleep Regulation	Sleep Regulation	4	glutamine	0.013267808	0.025641026
Antidepressant Action	Sleep Regulation	3	glutamine	0.01499186	0.023809524
SIRT4 Signaling in Aging	Genomic Instability Associated with Aging; Genomic Instability Associated with Aging (Aging Biology); generic; generic; nucleus	3	glutamine	0.01499186	0.023809524
Vitamin C Related Norepinephrine Synthesis	Vitamins Biology; Vitamins Biology (Biological Process); nerve tissue; nervous system	3	ascorbic acid	0.015566208	0.023255814
MTOR/TP53 Regulates Cell Metabolism	Eating Behavior and Metabolism Regulation; Eating Behavior and Metabolism Regulation (Biological Process); Hallmarks of Cancer (7): Deregulated Metabolism; generic; generic; oncogenic; oncogenic	2	glutamine	0.015609432	0.015873016

GABA Release	Neurotransmitter Release Cycles; Neurotransmitter Release Cycles (Nociception Pathways); nerve tissue; nervous system; secretory vesicle	3	glutamine	0.01614039	0.022727273
Glutamine in Cancer Metabolism	Hallmarks of Cancer (7): Deregulated Metabolism; Hallmarks of Cancer (7): Deregulated Metabolism (Hallmarks of Cancer); oncogenic; oncogenic	1	glutamine	0.019926935	0.013157895
Astrocyte Dysfunction and GABA Signaling Deficiency	Epileptiform Disorders; Epileptiform Disorders (Neurological Diseases); nerve tissue; nervous system; synapse	3	glutamine	0.020093348	0.021276596

Supplementary table 7b:Pathway Studio pathwayanalysis of upregulated metabolites in epithelial regions of post-chemo versus pre-chemo ER tissues

PR= poor response; ER= excellent response

Pathway identifier	Pathway name	Entities ratio	Entities pValue	Entities FDR	Reactions ratio	Submitted entities found	Mapped entities
R-HSA-15869	Metabolism of nucleotides	0.0824	2.39E-06	3.40E-04	0.01877	C00262; C00294; C00106; C00025	17596;17368;29985;17568; 17712
R-HSA-83936	Transport of nucleosides and free purine and pyrimidine bases across the plasma membrane	0.01116	9.99E-06	7.09E-04	0.00215	C00262; C00294; C00106	17596;17368;17568
R-HSA-8956319	Nucleotide catabolism	0.05635	3.42E-05	0.001606359	0.00845	C00262; C00294; C00106	17596;17368;17568;17712
R-HSA-8956321	Nucleotide salvage	0.02446	1.03E-04	0.003609551	0.00295	C00262; C00294; C00106	17596;17368;17568
R-HSA-74259	Purine catabolism	0.0319	2.50E-04	0.006075921	0.00442	C00385; C00262; C00294	17596;17368;17712
R-HSA-425397	Transport of vitamins, nucleosides, and related molecules	0.03456	2.87E-04	0.006075921	0.00523	C00262; C00294; C00106	17596;17368;17568
R-HSA-425407	SLC-mediated transmembrane transport	0.08772	3.04E-04	0.006075921	0.02561	C00262; C00294; C00106; C00025	17596;17368;29985;17568
R-HSA-73929	Base-Excision Repair, AP Site Formation	0.00851	5.77E-04	0.00981147	0.00389	C00262; C00106	17368;17568
R-HSA-382551	Transport of small molecules	0.11643	0.001062287	0.015934303	0.05404	C00294; C00106; C00025	17596;17368;29985;17568
R-HSA-73884	Base Excision Repair	0.01329	0.002001877	0.027279641	0.00536	C00262; C00106	17368;17568
R-HSA-74217	Purine salvage	0.01701	0.002273303	0.027279641	0.00161	C00262; C00294	17596;17368
R-HSA-8849932	Synaptic adhesion-like molecules	0.00106	0.00477554	0.042979864	4.02E-04	C00025	29985
R-HSA-6794362	Protein-protein interactions at synapses	0.00106	0.00477554	0.042979864	4.02E-04	C00025	29985
R-HSA-500657	Presynaptic function of Kainate receptors	0.00106	0.00477554	0.042979864	2.68E-04	C00025	29985

R-HSA-5619076	Defective SLC17A8 causes autosomal dominant deafness 25 (DFNA25)	0.00106	0.00477554	0.042979864	1.34E-04	C00025	29985
R-HSA-451307	Activation of Na-permeable kainate receptors	0.00159	0.007156459	0.057251673	2.68E-04	C00025	29985
R-HSA-5578999	Defective GCLC causes HAGGSD	0.00159	0.007156459	0.057251673	1.34E-04	C00025	29985
R-HSA-73894	DNA Repair	0.02924	0.009084682	0.063592775	0.02078	C00262; C00106	17368;17568
R-HSA-888568	GABA synthesis	0.00213	0.009532819	0.066538439	2.68E-04	C00025	29985
R-HSA-8964540	Alanine metabolism	0.00213	0.011904626	0.066538439	5.36E-04	C00025	29985
R-HSA-442982	Ras activation upon Ca2+ influx through NMDA receptor	0.00319	0.014271888	0.066538439	2.68E-04	C00025	29985
R-HSA-5619062	Defective SLC1A3 causes episodic ataxia 6 (EA6)	0.00319	0.014271888	0.066538439	1.34E-04	C00025	29985
R-HSA-5619067	Defective SLC1A1 is implicated in schizophrenia 18 (SCZD18) and dicarboxylic aminoaciduria (DCBXA)	0.00319	0.014271888	0.066538439	1.34E-04	C00025	29985
R-HSA-210455	Astrocytic Glutamate-Glutamine Uptake And Metabolism	0.00319	0.014271888	0.066538439	4.02E-04	C00025	29985
R-HSA-112313	Neurotransmitter uptake and metabolism In glial cells	0.00319	0.014271888	0.066538439	4.02E-04	C00025	29985
R-HSA-9617324	Negative regulation of NMDA receptor-mediated neuronal transmission	0.00319	0.014271888	0.066538439	5.36E-04	C00025	29985

R-HSA-5619063	Defective SLC29A3 causes histiocytosis-lymphadenopathy plus syndrome (HLAS)	0.00372	0.01663461	0.066538439	1.34E-04	C00294	17596
R-HSA-9664535	LTC4-CYSLTR mediated IL4 production	0.00372	0.01663461	0.066538439	4.02E-04	C00025	29985
R-HSA-110331	Cleavage of the damaged purine	0.00372	0.01663461	0.066538439	0.00121	C00262	17368
R-HSA-73927	Depurination	0.00372	0.01663461	0.066538439	0.00121	C00262	17368
R-HSA-5619102	SLC transporter disorders	0.04253	0.018096343	0.067479198	0.00831	C00294; C00025	17596;29985
R-HSA-9026762	Biosynthesis of maresin conjugates in tissue regeneration (MCTR)	0.00425	0.018992799	0.067479198	5.36E-04	C00025	29985
R-HSA-8955332	Carboxyterminal post-translational modifications of tubulin	0.00478	0.021346462	0.067479198	8.05E-04	C00025	29985
R-HSA-110329	Cleavage of the damaged pyrimidine	0.00478	0.021346462	0.067479198	0.00268	C00106	17568
R-HSA-73928	Depyrimidination	0.00478	0.021346462	0.067479198	0.00268	C00106	17568
R-HSA-442742	CREB1 phosphorylation through NMDA receptor-mediated activation of RAS signaling	0.00532	0.023695605	0.067479198	8.05E-04	C00025	29985
R-HSA-5619115	Disorders of transmembrane transporters	0.05157	0.025761392	0.067479198	0.01073	C00294; C00025	17596;29985
R-HSA-916853	Degradation of GABA	0.00532	0.026040235	0.067479198	4.02E-04	C00025	29985
R-HSA-399710	Activation of AMPA receptors	0.00159	0.028380359	0.067479198	6.70E-04	C00025	29985
R-HSA-451308	Activation of Ca-permeable Kainate Receptor	0.00159	0.028380359	0.067479198	2.68E-04	C00025	29985
R-HSA-451326	Activation of kainate receptors upon glutamate binding	0.00213	0.030715982	0.067479198	8.05E-04	C00025	29985
R-HSA-451306	Ionotropic activity of kainate receptors	0.00213	0.030715982	0.067479198	5.36E-04	C00025	29985

R-HSA-70688	Proline catabolism	0.00585	0.030715982	0.067479198	5.36E-04	C00025	29985
R-HSA-9620244	Long-term potentiation	0.00319	0.035373753	0.067479198	8.05E-04	C00025	29985
R-HSA-9662360	Sensory processing of sound by inner hair cells of the cochlea	0.00319	0.035373753	0.067479198	9.39E-04	C00025	29985
R-HSA-8963684	Tyrosine catabolism	0.00797	0.037695914	0.067479198	8.05E-04	C00025	29985
R-HSA-428643	Organic anion transporters	0.00797	0.037695914	0.067479198	0.00121	C00025	29985
R-HSA-438066	Unblocking of NMDA receptors, glutamate binding and activation	0.00319	0.040013601	0.067479198	6.70E-04	C00025	29985
R-HSA-210500	Glutamate Neurotransmitter Release Cycle	0.00425	0.040013601	0.067479198	0.00107	C00025	29985
R-HSA-399721	Glutamate binding, activation of AMPA receptors and synaptic plasticity	0.00425	0.040013601	0.067479198	0.00121	C00025	29985
R-HSA-9026766	Biosynthesis of protectin and resolvin conjugates in tissue regeneration (PCTR and RCTR)	0.00904	0.040013601	0.067479198	0.00107	C00025	29985
R-HSA-2161541	Abacavir metabolism	0.00904	0.040013601	0.067479198	9.39E-04	C00294	17596
R-HSA-174403	Glutathione synthesis and recycling	0.00744	0.04232682	0.067479198	0.00121	C00025	29985
R-HSA-2161522	Abacavir transport and metabolism	0.00957	0.04232682	0.067479198	0.00121	C00294	17596
R-HSA-9659379	Sensory processing of sound	0.00478	0.04232682	0.067479198	0.00174	C00025	29985
R-HSA-977347	Serine biosynthesis	0.00851	0.044635578	0.067479198	9.39E-04	C00025	29985
R-HSA-5683826	Surfactant metabolism	0.00851	0.044635578	0.067479198	0.00107	C00294	17596
R-HSA-9026395	Biosynthesis of DHA-derived sulfido conjugates	0.01116	0.049239736	0.067479198	0.00174	C00025	29985
R-HSA-500753	Pyrimidine biosynthesis	0.01116	0.049239736	0.067479198	8.05E-04	C00025	29985
R-HSA-446210	Synthesis of UDP-N-acetyl-glucosamine	0.01116	0.049239736	0.067479198	9.39E-04	C00025	29985

R-HSA-70635	Urea cycle	0.0117	0.051535148	0.067479198	0.00107	C00025	29985
R-HSA-73614	Pyrimidine salvage	0.01223	0.053826125	0.067479198	0.00134	C00106	17568
R-HSA-8963693	Aspartate and asparagine metabolism	0.01329	0.060672506	0.067479198	0.00147	C00025	29985
R-HSA-70921	Histidine catabolism	0.0117	0.060672506	0.067479198	0.00107	C00025	29985
R-HSA-5628897	TP53 Regulates Metabolic Genes	0.01382	0.060672506	0.067479198	0.00188	C00025	29985
R-HSA-8964539	Glutamate and glutamine metabolism	0.01435	0.0652147	0.067479198	0.00188	C00025	29985
R-HSA-888590	GABA synthesis, release, reuptake and degradation	0.00957	0.0652147	0.067479198	0.00134	C00025	29985
R-HSA-379724	tRNA Aminoacylation	0.01382	0.0652147	0.067479198	0.00563	C00025	29985
R-HSA-379716	Cytosolic tRNA aminoacylation	0.01382	0.0652147	0.067479198	0.00282	C00025	29985
R-HSA-379726	Mitochondrial tRNA aminoacylation	0.01382	0.0652147	0.067479198	0.00282	C00025	29985
R-HSA-196757	Metabolism of folate and pterines	0.01542	0.067479198	0.067479198	0.00402	C00025	29985
R-HSA-5423646	Aflatoxin activation and detoxification	0.01542	0.067479198	0.067479198	0.00241	C00025	29985
R-HSA-2142691	Synthesis of Leukotrienes (LT) and Eoxins (EX)	0.01648	0.071995029	0.071995029	0.00255	C00025	29985
R-HSA-5673001	RAF/MAP kinase cascade	0.01329	0.074246374	0.074246374	0.00684	C00025	29985
R-HSA-5684996	MAPK1/MAPK3 signaling	0.01329	0.074246374	0.074246374	0.00724	C00025	29985
R-HSA-5683057	MAPK family signaling cascades	0.01329	0.074246374	0.074246374	0.00912	C00025	29985
R-HSA-8963691	Phenylalanine and tyrosine metabolism	0.01701	0.076493348	0.076493348	0.00161	C00025	29985
R-HSA-438064	Post NMDA receptor activation events	0.00638	0.076493348	0.076493348	0.00469	C00025	29985
R-HSA-70263	Gluconeogenesis	0.01701	0.076493348	0.076493348	0.00349	C00025	29985
R-HSA-71064	Lysine catabolism	0.01701	0.080974207	0.080974207	0.00174	C00025	29985
R-HSA-156590	Glutathione conjugation	0.01648	0.080974207	0.080974207	0.00188	C00025	29985

R-HSA-71240	Tryptophan catabolism	0.01808	0.080974207	0.080974207	0.00215	C00025	29985
R-HSA-352230	Amino acid transport across the plasma membrane	0.01861	0.080974207	0.080974207	0.00483	C00025	29985
R-HSA-73817	Purine ribonucleoside monophosphate biosynthesis	0.01967	0.085437655	0.085437655	0.00215	C00025	29985
R-HSA-442755	Activation of NMDA receptors and postsynaptic events	0.00851	0.089883745	0.089883745	0.00617	C00025	29985
R-HSA-73621	Pyrimidine catabolism	0.02073	0.089883745	0.089883745	0.00228	C00106	17568
R-HSA-70895	Branched-chain amino acid catabolism	0.01967	0.092100296	0.092100296	0.00308	C00025	29985
R-HSA-72766	Translation	0.01861	0.094312526	0.094312526	0.01113	C00025	29985
R-HSA-1614558	Degradation of cysteine and homocysteine	0.0202	0.098724049	0.098724049	0.00295	C00025	29985
R-HSA-112314	Neurotransmitter receptors and postsynaptic signal transmission	0.01276	0.107495524	0.107495524	0.01113	C00025	29985
R-HSA-8956320	Nucleotide biosynthesis	0.02499	0.107495524	0.107495524	0.00295	C00025	29985
R-HSA-499943	Interconversion of nucleotide di- and triphosphates	0.02658	0.114029204	0.114029204	0.00442	C00025	29985
R-HSA-392499	Metabolism of proteins	0.12547	0.119727234	0.119727234	0.06141	C00294; C00025	17596;29985
R-HSA-70326	Glucose metabolism	0.02233	0.124833597	0.124833597	0.00644	C00025	29985
R-HSA-112310	Neurotransmitter release cycle	0.02605	0.133400994	0.133400994	0.00483	C00025	29985
R-HSA-196807	Nicotinate metabolism	0.02818	0.133400994	0.133400994	0.00389	C00025	29985
R-HSA-5579029	Metabolic disorders of biological oxidation enzymes	0.02818	0.139782402	0.139782402	0.0051	C00025	29985
R-HSA-420499	Class C/3 (Metabotropic glutamate/pheromone receptors)	0.0303	0.148232411	0.148232411	9.39E-04	C00025	29985

R-HSA-446219	Synthesis of substrates in N-glycan biosythesis	0.03615	0.154526233	0.154526233	0.00657	C00025	29985
R-HSA-425393	Transport of inorganic cations/anions and amino acids/oligopeptides	0.03083	0.160782795	0.160782795	0.01006	C00025	29985
R-HSA-9018677	Biosynthesis of DHA-derived SPMs	0.03828	0.160782795	0.160782795	0.00778	C00025	29985
R-HSA-1614635	Sulfur amino acid metabolism	0.03456	0.162860064	0.162860064	0.00483	C00025	29985
R-HSA-416476	G alpha (q) signalling events	0.03456	0.164933218	0.164933218	0.00416	C00025	29985
R-HSA-446193	Biosynthesis of the N-glycan precursor (dolichol lipid-linked oligosaccharide, LLO) and transfer to a nascent protein	0.04306	0.181371026	0.181371026	0.00845	C00025	29985
R-HSA-3928662	EPHB-mediated forward signaling	0.00532	0.18543975	0.18543975	0.00295	C00025	29985
R-HSA-2682334	EPH-Ephrin signaling	0.00532	0.18543975	0.18543975	0.0051	C00025	29985
R-HSA-422475	Axon guidance	0.00957	0.201553192	0.201553192	0.01783	C00025	29985
R-HSA-9675108	Nervous system development	0.00957	0.201553192	0.201553192	0.01783	C00025	29985
R-HSA-112315	Transmission across Chemical Synapses	0.03721	0.213470095	0.213470095	0.01797	C00025	29985
R-HSA-112316	Neuronal System	0.03721	0.213470095	0.213470095	0.02105	C00025	29985
R-HSA-1266738	Developmental Biology	0.01914	0.236877039	0.236877039	0.02481	C00025	29985
R-HSA-2142753	Arachidonic acid metabolism	0.05795	0.236877039	0.236877039	0.01032	C00025	29985
R-HSA-3700989	Transcriptional Regulation by TP53	0.0202	0.236877039	0.236877039	0.00952	C00025	29985
R-HSA-446203	Asparagine N-linked glycosylation	0.06167	0.250271543	0.250271543	0.0177	C00025	29985
R-HSA-1430728	Metabolism	0.75864	0.256438889	0.256438889	0.28855	C00385; C00262; C00294; C00106; C00025	17596;17368;29985;17568;17712
R-HSA-212436	Generic Transcription Pathway	0.02924	0.269079627	0.269079627	0.01743	C00025	29985

R-HSA-73857	RNA Polymerase II Transcription	0.03083	0.274647811	0.274647811	0.02132	C00025	29985
R-HSA-74160	Gene expression (Transcription)	0.03509	0.291149065	0.291149065	0.02588	C00025	29985
R-HSA-9018678	Biosynthesis of specialized proresolving mediators (SPMs)	0.07443	0.292963839	0.292963839	0.01663	C00025	29985
R-HSA-196849	Metabolism of water-soluble vitamins and cofactors	0.06911	0.294774894	0.294774894	0.01931	C00025	29985
R-HSA-9662851	Anti-inflammatory response favouring Leishmania parasite infection	0.01861	0.30913014	0.30913014	0.00349	C00025	29985
R-HSA-9664433	Leishmania parasite growth and survival	0.01861	0.30913014	0.30913014	0.00349	C00025	29985
R-HSA-500792	GPCR ligand binding	0.07283	0.314452687	0.314452687	0.01167	C00025	29985
R-HSA-1643685	Disease	0.18394	0.331395995	0.331395995	0.11652	C00294; C00025	17596;29985
R-HSA-156580	Phase II - Conjugation of compounds	0.08081	0.333688755	0.333688755	0.00965	C00025	29985
R-HSA-9717207	Sensory perception of sweet, bitter, and umami (glutamate) taste	0.04732	0.338859278	0.338859278	0.00282	C00025	29985
R-HSA-9717189	Sensory perception of taste	0.04944	0.345703326	0.345703326	0.00335	C00025	29985
R-HSA-5668914	Diseases of metabolism	0.08506	0.345703326	0.345703326	0.02467	C00025	29985
R-HSA-9658195	Leishmania infection	0.0303	0.347405449	0.347405449	0.00845	C00025	29985
R-HSA-71387	Metabolism of carbohydrates	0.08187	0.359221385	0.359221385	0.03071	C00025	29985
R-HSA-597592	Post-translational protein modification	0.0925	0.364232664	0.364232664	0.03808	C00025	29985
R-HSA-418594	G alpha (i) signalling events	0.05795	0.392041087	0.392041087	0.00912	C00025	29985
R-HSA-196854	Metabolism of vitamins and cofactors	0.09942	0.393646046	0.393646046	0.02735	C00025	29985
R-HSA-5663205	Infectious disease	0.06699	0.485335731	0.485335731	0.04398	C00025	29985

R-HSA-8978868	Fatty acid metabolism	0.13397	0.489540482	0.489540482	0.02883	C00025	29985
R-HSA-388396	GPCR downstream signalling	0.0824	0.493717706	0.493717706	0.02038	C00025	29985
R-HSA-372790	Signaling by GPCR	0.08453	0.507444715	0.507444715	0.03205	C00025	29985
R-HSA-9709957	Sensory Perception	0.10101	0.523522385	0.523522385	0.01837	C00025	29985
R-HSA-71291	Metabolism of amino acids and derivatives	0.15152	0.535302841	0.535302841	0.03754	C00025	29985
R-HSA-211859	Biological oxidations	0.17331	0.586058396	0.586058396	0.02507	C00025	29985
R-HSA-162582	Signal Transduction	0.12919	0.650187278	0.650187278	0.17471	C00025	29985
R-HSA-556833	Metabolism of lipids	0.33705	0.901395364	0.901395364	0.11893	C00025	29985

Supplementary table 8b: Reactome pathway analysis of downregulated metabolites in stromal regions of post-chemo versus pre-chemo ER tissues

PR= poor response; ER= excellent response

Name	Parent Folder	Percent Overlap	Overlapping Entities	p-value	Jaccard similarity
Uric Acid Synthesis in Gout	Gout; Gout (Immune System Diseases); digestive system	9	xanthine;hypoxanthine;inosine	5.16213E-07	0.088235294
Purine metabolism template	metabolic cycles	2	hypoxanthine;inosine;glutamate;xanthine	1.44258E-06	0.029411765
Purine Metabolism	Nucleotides Metabolism; Nucleotides Metabolism (Metabolic Pathway); generic; generic	2	hypoxanthine;inosine;glutamate;xanthine	4.38083E-06	0.022346369
GABRB3 Mutation and Adenosine Deaminase Polymorphism	Sleep Dysregulation; Sleep Dysregulation (Neurological Diseases); nerve tissue; nervous system	7	inosine;glutamate	0.000103397	0.068965517
pathway template	anabolism	7	hypoxanthine;inosine	0.000227817	0.064516129
HPRT1 Deficiency in Lesch-Nyhan Syndrome	Lesch-Nyhan Syndrome; Lesch-Nyhan Syndrome (Inherited/Rare Diseases); endocrine system; generic	5	xanthine;hypoxanthine	0.000247396	0.046511628
Malonate, Propanoate and beta-Alanine Metabolism	Carboxylic Acids Metabolism; Carboxylic Acids Metabolism (Metabolic Pathway); generic; generic	2	glutamate;uracil	0.002851739	0.019607843
Pyrimidine Metabolism	Nucleotides Metabolism; Nucleotides Metabolism (Metabolic Pathway); generic; generic	1	glutamate;uracil	0.003831814	0.016949153
Pyrimidine metabolism template	metabolic cycles	1	glutamate;uracil	0.004373631	0.015873016

AMPA Receptors -> Calcium Influx	Glutamate Receptors; Glutamate Receptors; Glutamate Receptors (Ligand Gated Ion Channels); nerve tissue; nervous system	12	glutamate	0.004432133	0.083333333
NMDA Receptor -> Synaptic Excitation	Glutamate Receptors; Glutamate Receptors; Glutamate Receptors (Ligand Gated Ion Channels); nerve tissue; nervous system	12	glutamate	0.004432133	0.083333333
AMPA Receptors -> Calcium Influx	Glutamate Receptors; Glutamate Receptors; Glutamate Receptors (Ligand Gated Ion Channels); nerve tissue; nervous system	12	glutamate	0.00462494	0.083333333
NMDA Receptor -> Synaptic Excitation	Glutamate Receptors; Glutamate Receptors; Glutamate Receptors (Ligand Gated Ion Channels); nerve tissue; nervous system	12	glutamate	0.00462494	0.083333333
Immunoglobulin Class-Switch Recombination via Classical non-Homologous End-Joining	Immunoglobulins Production; Immunoglobulins Production (Immunological Pathways); lymphatic system; nucleus	6	uracil	0.008662968	0.052631579
HMGB1 and IL1B in Neuronal Hyperexcitation	Epileptiform Disorders; Epileptiform Disorders (Neurological Diseases); nerve tissue; nervous system	6	glutamate	0.009760786	0.052631579

Immunoglobulin Class-Switch Recombination via Alternative End-Joining	Immunoglobulins Production; Immunoglobulins Production (Immunological Pathways); lymphatic system; nucleus	5	uracil	0.009815183	0.047619048
GRM2-4/6-8 (Presynaptic) -> Glutamate Release Attenuation	Glutamate Receptors; Glutamate Receptors; Glutamate Receptors (Ligand Gated Ion Channels); nerve tissue; nervous system	5	glutamate	0.010526316	0.043478261
Glutamate Mediated Neurotoxicity	Toxicity Induced Pathways; Toxicity Induced Pathways (Toxicity Pathways); generic; generic	3	glutamate	0.010627698	0.027777778
GRM2-4/6-8 (Presynaptic) -> Glutamate Release Attenuation	Glutamate Receptors; Glutamate Receptors; Glutamate Receptors (Ligand Gated Ion Channels); nerve tissue; nervous system	5	glutamate	0.010966728	0.043478261
RHO Signaling (Dark)	Visual Phototransduction; Visual Phototransduction (Biological Process); retina; sensory system	5	glutamate	0.010966728	0.043478261
Cochlear Hair Cell Synapse Proteins Mutations (Age-Related/Congenital)	Hearing Loss; Hearing Loss (Otolaryngological Diseases); sensory system; synapse	5	glutamate	0.011056805	0.047619048

Rod Photoreceptor Signaling Impairment in Retinitis Pigmentosa	Retinitis Pigmentosa; Retinitis Pigmentosa (Ophthalmology /Eye Diseases); Retinitis Pigmentosa (Ophthalmology /Eye Diseases); retina; sensory system	5	glutamate	0.011704337	0.045454545
Histamine in Arousal Regulation	Sleep Regulation; Sleep Regulation (Biological Process); nerve tissue; nervous system	4	glutamate	0.012117603	0.04
P2RXs -> Synaptic Transmission	Purinergic Receptors; Purinergic Receptors; Purinergic Receptors (Ligand Gated Ion Channels); nervous system	4	glutamate	0.012188366	0.038461538
Prostaglandin E2 Synthesis Activation via Glutamate in Familial Hemiplegic Migraine	Migraine, Familial Hemiplegic; Migraine, Familial Hemiplegic (Neurological Diseases); nervous system	5	glutamate	0.012351551	0.043478261

Supplementary table 8b: Pathway studio pathway analysis of downregulated metabolites in stromal regions of post-chemo versus pre-chemo ER tissues

PR= poor response; ER= excellent response

Pathway identifier	Pathway name	Entities ratio	Entities pValue	Entities FDR	Reactions ratio	Submitted entities found	Mapped entities
R-HSA-70635	Urea cycle	0.011695906	0.006817654	0.164164667	0.001072674	C00122;C00049	29991;29806
R-HSA-8963693	Aspartate and asparagine metabolism	0.013290803	0.009401592	0.164164667	0.001474926	C00049;C00245	29991;15891
R-HSA-71403	Citric acid cycle (TCA cycle)	0.014885699	0.011585117	0.164164667	0.002279431	C00122;C00042	30031;18012
R-HSA-8963691	Phenylalanine and tyrosine metabolism	0.017012228	0.014811523	0.164164667	0.00160901	C00122;C00049	29991;29806
R-HSA-73817	Purine ribonucleoside monophosphate biosynthesis	0.019670388	0.018384173	0.164164667	0.002145347	C00122;C00049	29991;29806
R-HSA-433137	Sodium-coupled sulphate, di- and tri-carboxylate transporters	0.002126528	0.02272629	0.164164667	6.70E-04	C00042	30031
R-HSA-8956320	Nucleotide biosynthesis	0.024986709	0.028735242	0.164164667	0.002949853	C00122;C00049	29991;29806
R-HSA-5619067	Defective SLC1A1 is implicated in schizophrenia 18 (SCZD18) and dicarboxylic aminoaciduria (DCBXA)	0.003189793	0.033910989	0.164164667	1.34E-04	C00049	29991
R-HSA-5619062	Defective SLC1A3 causes episodic ataxia 6 (EA6)	0.003189793	0.033910989	0.164164667	1.34E-04	C00049	29991
R-HSA-3214842	HDMs demethylate histones	0.002658161	0.033910989	0.164164667	0.002279431	C00042	30031
R-HSA-1234174	Cellular response to hypoxia	0.003189793	0.039459224	0.164164667	0.001340842	C00042	30031
R-HSA-1234176	Oxygen-dependent proline hydroxylation of Hypoxia-inducible Factor Alpha	0.003189793	0.039459224	0.164164667	0.001206758	C00042	30031

R-HSA-71406	Pyruvate metabolism and Citric Acid (TCA) cycle	0.023923445	0.040940901	0.164164667	0.004692947	C00122;C00042	30031;18012
R-HSA-112122	ALKBH2 mediated reversal of alkylation damage	0.003721425	0.044978236	0.164164667	8.05E-04	C00042	30031
R-HSA-112126	ALKBH3 mediated reversal of alkylation damage	0.003721425	0.044978236	0.164164667	8.05E-04	C00042	30031
R-HSA-9694548	Maturation of spike protein	0.003189793	0.050468167	0.164164667	4.02E-04	C00221	15903
R-HSA-5221030	TET1,2,3 and TDG demethylate DNA	0.004784689	0.055929154	0.164164667	6.70E-04	C00042	30031
R-HSA-73943	Reversal of alkylation damage by DNA dioxygenases	0.004784689	0.055929154	0.164164667	0.001743095	C00042	30031
R-HSA-77108	Utilization of Ketone Bodies	0.005316321	0.055929154	0.164164667	4.02E-04	C00042	30031
R-HSA-9683686	Maturation of spike protein	0.003721425	0.055929154	0.164164667	5.36E-04	C00221	15903
R-HSA-1650814	Collagen biosynthesis and modifying enzymes	0.004784689	0.055929154	0.164164667	0.00160901	C00042	30031
R-HSA-73942	DNA Damage Reversal	0.005316321	0.061361338	0.164164667	0.002011263	C00042	30031
R-HSA-916853	Degradation of GABA	0.005316321	0.061361338	0.164164667	4.02E-04	C00042	30031
R-HSA-425393	Transport of inorganic cations/anions and amino acids/oligopeptides	0.030834662	0.062284005	0.164164667	0.010056315	C00049;C00245	29991;15891
R-HSA-1428517	The citric acid (TCA) cycle and respiratory electron transport	0.031897927	0.063823187	0.164164667	0.008447305	C00122;C00042	30031;18012
R-HSA-5625886	Activated PKN1 stimulates transcription of AR (androgen receptor) regulated genes KLK2 and KLK3	0.005847953	0.066764856	0.164164667	0.001474926	C00042	30031

R-HSA-2559580	Oxidative Stress Induced Senescence	0.004253057	0.072139845	0.164164667	0.002413516	C00042	30031
R-HSA-71291	Metabolism of amino acids and derivatives	0.151515152	0.078435833	0.164164667	0.037543577	C00122;C00042;C00049;C00245	29991;30031;15891;29806
R-HSA-2299718	Condensation of Prophase Chromosomes	0.005316321	0.082804787	0.164164667	6.70E-04	C00042	30031
R-HSA-1474290	Collagen formation	0.00744285	0.082804787	0.164164667	0.002011263	C00042	30031
R-HSA-425366	Transport of bile salts and organic acids, metal ions and amine compounds	0.040935673	0.084999728	0.164164667	0.008983642	C00042;C00245	30031;15891
R-HSA-425407	SLC-mediated transmembrane transport	0.087719298	0.086515067	0.164164667	0.025610083	C00042;C00049;C00245	29991;30031;15891
R-HSA-5625740	RHO GTPases activate PKNs	0.007974482	0.088095012	0.164164667	0.002279431	C00042	30031
R-HSA-8963684	Tyrosine catabolism	0.007974482	0.088095012	0.164164667	8.05E-04	C00122	29806
R-HSA-71262	Carnitine synthesis	0.008506114	0.093357253	0.164164667	8.05E-04	C00042	30031
R-HSA-5676934	Protein repair	0.006911217	0.093357253	0.164164667	9.39E-04	C00049	29991
R-HSA-9683701	Translation of Structural Proteins	0.00744285	0.093357253	0.164164667	0.001474926	C00221	15903
R-HSA-6782861	Synthesis of wybutosine at G37 of tRNA(Phe)	0.00744285	0.103798321	0.164164667	0.001072674	C00042	30031
R-HSA-74182	Ketone body metabolism	0.01010101	0.103798321	0.164164667	0.001474926	C00042	30031
R-HSA-2559583	Cellular Senescence	0.00744285	0.103798321	0.164164667	0.004290695	C00042	30031
R-HSA-4839726	Chromatin organization	0.008506114	0.114129061	0.164164667	0.009654063	C00042	30031
R-HSA-3247509	Chromatin modifying enzymes	0.008506114	0.114129061	0.164164667	0.009654063	C00042	30031
R-HSA-500753	Pyrimidine biosynthesis	0.011164274	0.114129061	0.164164667	8.05E-04	C00049	29991
R-HSA-8964208	Phenylalanine metabolism	0.011164274	0.114129061	0.164164667	8.05E-04	C00049	29991

R-HSA-159418	Recycling of bile acids and salts	0.011164274	0.114129061	0.164164667	0.002279431	C00245	15891
R-HSA-212165	Epigenetic regulation of gene expression	0.009037746	0.119253391	0.164164667	0.002279431	C00042	30031
R-HSA-2024096	HS-GAG degradation	0.011695906	0.119253391	0.164164667	0.001743095	C00049	29991
R-HSA-9694635	Translation of Structural Proteins	0.009569378	0.134463796	0.164164667	0.001877179	C00221	15903
R-HSA-389599	Alpha-oxidation of phytanate	0.013290803	0.139480171	0.164164667	0.001072674	C00042	30031
R-HSA-888590	GABA synthesis, release, reuptake and degradation	0.009569378	0.149433061	0.164164667	0.001340842	C00042	30031
R-HSA-8964539	Glutamate and glutamine metabolism	0.014354067	0.149433061	0.164164667	0.001877179	C00049	29991
R-HSA-379724	tRNA Aminoacylation	0.013822435	0.149433061	0.164164667	0.005631537	C00049	29991
R-HSA-379716	Cytosolic tRNA aminoacylation	0.013822435	0.149433061	0.164164667	0.002815768	C00049	29991
R-HSA-379726	Mitochondrial tRNA aminoacylation	0.013822435	0.149433061	0.164164667	0.002815768	C00049	29991
R-HSA-68875	Mitotic Prophase	0.008506114	0.159280325	0.164164667	0.003620274	C00042	30031
R-HSA-9678108	SARS-CoV-1 Infection	0.012759171	0.164164667	0.164164667	0.005363368	C00221	15903
R-HSA-442660	Na+/Cl- dependent neurotransmitter transporters	0.017012228	0.169022984	0.169022984	0.002011263	C00245	15891
R-HSA-6782315	tRNA modification in the nucleus and cytosol	0.014354067	0.169022984	0.169022984	0.004424779	C00042	30031
R-HSA-70263	Gluconeogenesis	0.017012228	0.173855403	0.173855403	0.003486189	C00049	29991
R-HSA-1638091	Heparan sulfate/heparin (HS-GAG) metabolism	0.01754386	0.173855403	0.173855403	0.003888442	C00049	29991
R-HSA-9694516	SARS-CoV-2 Infection	0.014354067	0.178662049	0.178662049	0.00670421	C00221	15903
R-HSA-382551	Transport of small molecules	0.116427432	0.178671821	0.178671821	0.054035935	C00042;C00049;C00245	29991;30031;15891

R-HSA-352230	Amino acid transport across the plasma membrane	0.018607124	0.183443047	0.183443047	0.004827031	C00245	15891
R-HSA-72306	tRNA processing	0.016480595	0.188198522	0.188198522	0.005765621	C00042	30031
R-HSA-9679506	SARS-CoV Infections	0.015417331	0.188198522	0.188198522	0.012201663	C00221	15903
R-HSA-68886	M Phase	0.012227539	0.192928597	0.192928597	0.007776884	C00042	30031
R-HSA-72766	Translation	0.018607124	0.211597364	0.211597364	0.011128989	C00049	29991
R-HSA-1614558	Degradation of cysteine and homocysteine	0.02020202	0.220782534	0.220782534	0.002949853	C00245	15891
R-HSA-15869	Metabolism of nucleotides	0.082402977	0.226398825	0.226398825	0.018771789	C00122;C00049	29991;29806
R-HSA-389661	Glyoxylate metabolism and glycine degradation	0.021796917	0.229869515	0.229869515	0.003083937	C00049	29991
R-HSA-69278	Cell Cycle, Mitotic	0.018607124	0.247752725	0.247752725	0.017296862	C00042	30031
R-HSA-1640170	Cell Cycle	0.019670388	0.256550838	0.256550838	0.023330652	C00042	30031
R-HSA-70326	Glucose metabolism	0.022328549	0.273864734	0.273864734	0.006436042	C00049	29991
R-HSA-8953854	Metabolism of RNA	0.023391813	0.273864734	0.273864734	0.012469831	C00042	30031
R-HSA-193368	Synthesis of bile acids and bile salts via 7alpha-hydroxycholesterol	0.028708134	0.282382352	0.282382352	0.005229284	C00245	15891
R-HSA-1630316	Glycosaminoglycan metabolism	0.030834662	0.286606727	0.286606727	0.010458568	C00049	29991
R-HSA-112310	Neurotransmitter release cycle	0.026049973	0.290808295	0.290808295	0.004827031	C00042	30031
R-HSA-390918	Peroxisomal lipid metabolism	0.032429559	0.303277277	0.303277277	0.004692947	C00042	30031
R-HSA-73894	DNA Repair	0.029239766	0.315545005	0.315545005	0.020783052	C00042	30031
R-HSA-1614635	Sulfur amino acid metabolism	0.034556087	0.347297396	0.347297396	0.004827031	C00245	15891
R-HSA-192105	Synthesis of bile acids and bile salts	0.041998937	0.392400944	0.392400944	0.011128989	C00245	15891
R-HSA-373076	Class A/1 (Rhodopsin-like receptors)	0.041998937	0.392400944	0.392400944	0.010458568	C00042	30031
R-HSA-194068	Bile acid and bile salt metabolism	0.042530569	0.396027489	0.396027489	0.013274336	C00245	15891

R-HSA-2262752	Cellular responses to stress	0.04040404	0.406788406	0.406788406	0.022660231	C00042	30031
R-HSA-5619102	SLC transporter disorders	0.042530569	0.420862956	0.420862956	0.008313221	C00049	29991
R-HSA-8953897	Cellular responses to stimuli	0.041467305	0.42433343	0.42433343	0.026816841	C00042	30031
R-HSA-112315	Transmission across Chemical Synapses	0.037214248	0.438025544	0.438025544	0.017967283	C00042	30031
R-HSA-112316	Neuronal System	0.037214248	0.438025544	0.438025544	0.02105122	C00042	30031
R-HSA-1474244	Extracellular matrix organization	0.014885699	0.470960172	0.470960172	0.005229284	C00042	30031
R-HSA-5619115	Disorders of transmembrane transporters	0.051568315	0.483631176	0.483631176	0.010726736	C00049	29991
R-HSA-195258	RHO GTPase Effectors	0.014885699	0.534439967	0.534439967	0.013140252	C00042	30031
R-HSA-194315	Signaling by Rho GTPases	0.014885699	0.534439967	0.534439967	0.025207831	C00042	30031
R-HSA-9716542	Signaling by Rho GTPases, Miro GTPases and RHOBTB3	0.014885699	0.534439967	0.534439967	0.026414588	C00042	30031
R-HSA-74160	Gene expression (Transcription)	0.035087719	0.562143502	0.562143502	0.025878252	C00042	30031
R-HSA-500792	GPCR ligand binding	0.072833599	0.59589908	0.59589908	0.011665326	C00042	30031
R-HSA-8957322	Metabolism of steroids	0.080808081	0.610659097	0.610659097	0.026682757	C00245	15891
R-HSA-9717207	Sensory perception of sweet, bitter, and umami (glutamate) taste	0.047315258	0.629570659	0.629570659	0.002815768	C00049	29991
R-HSA-9717189	Sensory perception of taste	0.049441786	0.638707196	0.638707196	0.003352105	C00049	29991
R-HSA-71387	Metabolism of carbohydrates	0.081871345	0.656363551	0.656363551	0.030705283	C00049	29991
R-HSA-418594	G alpha (i) signalling events	0.0579479	0.697100782	0.697100782	0.009117726	C00042	30031
R-HSA-392499	Metabolism of proteins	0.125465178	0.796924448	0.796924448	0.061410566	C00049	29991

R-HSA-5663205	Infectious disease	0.066985646	0.796924448	0.796924448	0.043979619	C00221	15903
R-HSA-8978868	Fatty acid metabolism	0.133971292	0.800883553	0.800883553	0.028828104	C00042	30031
R-HSA-388396	GPCR downstream signalling	0.082402977	0.804771789	0.804771789	0.020380799	C00042	30031
R-HSA-1643685	Disease	0.18394471	0.80747166	0.80747166	0.116519174	C00221;C00049	29991;15903
R-HSA-372790	Signaling by GPCR	0.084529506	0.817235451	0.817235451	0.032046125	C00042	30031
R-HSA-9709957	Sensory Perception	0.101010101	0.831227375	0.831227375	0.018369536	C00049	29991
R-HSA-162582	Signal Transduction	0.129186603	0.919609369	0.919609369	0.174711719	C00042	30031
R-HSA-556833	Metabolism of lipids	0.337054758	0.968929528	0.968929528	0.11893269	C00042;C00245	30031;15891
R-HSA-1430728	Metabolism	0.758639022	0.998003705	0.998003705	0.288549209	C00122;C00042;C00049;C00245	29991;30031;18012;15891;29806

Supplementary table 9a: Reactome pathway analysis of upregulated metabolites in stromal regions of post-chemo versus pre-chemo ER regions

PR= poor response; ER= excellent response

Name	Parent Folder	Percent Overlap	Overlapping Entities	p-value	Jaccard similarity	Hit type
Glutamine in Cancer Metabolism	Hallmarks of Cancer (7): Deregulated Metabolism; Hallmarks of Cancer (7): Deregulated Metabolism (Hallmarks of Cancer); oncogenic; oncogenic	5	aspartate;succinate;fumarate	7.5289E-06	0.04477612	Pathological Process
UMP pyrimidine precursor biosynthesis template	anabolism	7	succinate;fumarate;aspartate	1.2529E-05	0.06122449	Metabolic Pathway
Hyperammonemia Type I	Urea Cycle Disorders; Urea Cycle Disorders (Urology/Nephrology Diseases); mitochondria	4	fumarate;aspartate	4.5893E-05	0.04	Disease
Hyperammonemia Type II	Urea Cycle Disorders; Urea Cycle Disorders (Urology/Nephrology Diseases); generic; mitochondria	4	fumarate;aspartate	5.0424E-05	0.03846154	Disease
Citrullinemia Type I	Urea Cycle Disorders; Urea Cycle Disorders (Urology/Nephrology Diseases); mitochondria	4	fumarate;aspartate	5.2769E-05	0.03773585	Disease
Metabolic Reprogramming in Cancer: Overview	Hallmarks of Cancer (7): Deregulated Metabolism; Hallmarks of Cancer (7): Deregulated Metabolism (Hallmarks of Cancer); oncogenic; oncogenic	2	aspartate;fumarate;succinate	5.4323E-05	0.02459016	Pathological Process

Argininosuccinic Aciduria in Urea Cycle Disorders	Urea Cycle Disorders; Urea Cycle Disorders (Urology/Nephrology Diseases)	4	fumarate;aspartate	5.762E-05	0.03636364	Disease
Citrullinemia Type II	Urea Cycle Disorders; Urea Cycle Disorders (Urology/Nephrology Diseases); mitochondria	3	aspartate;fumarate	6.5295E-05	0.03448276	Disease
Argininemia	Urea Cycle Disorders; Urea Cycle Disorders (Urology/Nephrology Diseases)	3	aspartate;fumarate	7.9154E-05	0.03174603	Disease
Nicotinate and NAD metabolism template	metabolic cycles	3	aspartate;succinate;fumarate	9.122E-05	0.03409091	Metabolic Pathway
Urea cycle and Arginine metabolism template	Aminoacid metabolism and interconversion	3	aspartate;succinate;fumarate	0.00011689	0.03157895	Metabolic Pathway
Urea Cycle and Arginine Metabolism	Amino Acids Metabolism; Amino Acids Metabolism (Metabolic Pathway); generic; generic	3	aspartate;succinate;fumarate	0.00012501	0.03092784	Metabolic Pathway
Respiratory Chain and Oxidative Phosphorylation	Electron Transport Chain; Electron Transport Chain (Metabolic Pathway); generic; generic	10	succinate;fumarate	0.00024014	0.07142857	Metabolic Pathway
Formalin and formate degradation template	catabolilsm	7	succinate;fumarate	0.00037806	0.06060606	Metabolic Pathway
Oxo (keto) fatty acids	Fatty Acyl & Other Lipids	0	5-oxo-pentanoic acid	0.00045748	0.0033557	Lipids Ontology

mTOR Signaling Activation by Amino Acids	Fundamental Signalings Changed in Aging; Signal Transduction Pathways; Signal Transduction Pathways (Signal Processing); generic; generic	2	aspartate;succinate	0.00054269	0.02247191	Biological Process
Respiratory chain and oxidative phosphorylation template	anabolism	6	succinate;fumarate	0.0006623	0.04878049	Metabolic Pathway
Mutations in Krebs Cycle Enzymes in Cancer	Hallmarks of Cancer (7): Deregulated Metabolism; Hallmarks of Cancer (7): Deregulated Metabolism (Hallmarks of Cancer); mitochondria; oncogenic; oncogenic	3	succinate;fumarate	0.00097156	0.03174603	Pathological Process
Pentose-Phosphate Shunt	Carbohydrates Metabolism; Carbohydrates Metabolism (Metabolic Pathway); generic; generic	4	gluconate;beta-D-glucose	0.00128846	0.03703704	Metabolic Pathway
Reductive TCA cycle template	metabolic cycles	4	succinate;fumarate	0.00128846	0.03703704	Metabolic Pathway
Proline metabolism template	Aminoacid metabolism and interconversion	4	aspartate;succinate	0.00134494	0.03636364	Metabolic Pathway
scopolin and esculin biosynthesis template	Defense response	4	succinate;beta-D-glucose	0.0014026	0.03571429	Metabolic Pathway
Ubiquinone-Q10 biosynthesis (eukaryotic) template	Eukaryots	4	succinate;fumarate	0.00146143	0.03508772	Metabolic Pathway
Ubiquinone-8 biosynthesis template	anabolism	3	succinate;fumarate	0.00152144	0.03448276	Metabolic Pathway

Ubiquinone-6 biosynthesis (eukaryotic) template	Eukaryots	3	succinate;fumarate	0.00152144	0.03448276	Metabolic Pathway

Supplementary table 9b: pathway studio pathway analysis of upregulated metabolites in stromal regions of post-chemo versus pre-chemo ER regions

PR= poor response; ER= excellent response

Pathway identifier	Pathway name	Entities ratio	Entities pValue	Entities FDR	Reactions ratio	Submitted entities found	Mapped entities
R-HSA-174403	Glutathione synthesis and recycling	0.00744285	0.002565003	0.080432685	0.001206758	C00051	57925;16856
R-HSA-15869	Metabolism of nucleotides	0.082402977	0.002948164	0.080432685	0.018771789	C00385;C00064;C00294;C00051	17596;58359;17712;57925
R-HSA-5628897	TP53 Regulates Metabolic Genes	0.013822435	0.005256768	0.080432685	0.001877179	C00064;C00051	58359;57925
R-HSA-8963693	Aspartate and asparagine metabolism	0.013290803	0.005256768	0.080432685	0.001474926	C00064;C00245	58359;15891
R-HSA-1222538	Tolerance by Mtb to nitric oxide produced by macrophages	0.013290803	0.006496104	0.080432685	0.001206758	C00051	57925;16856
R-HSA-70268	Pyruvate metabolism	0.013822435	0.007856718	0.080432685	0.001877179	C00051	57925;16856
R-HSA-442660	Na+/Cl- dependent neurotransmitter transporters	0.017012228	0.007856718	0.080432685	0.002011263	C00064;C00245	58359;15891
R-HSA-4085023	Defective GFPT1 causes CMSTA1	0.001063264	0.008579547	0.080432685	1.34E-04	C00064	58359
R-HSA-156590	Glutathione conjugation	0.016480595	0.009336001	0.080432685	0.001877179	C00051	57925;16856
R-HSA-352230	Amino acid transport across the plasma membrane	0.018607124	0.009336001	0.080432685	0.004827031	C00064;C00245	58359;15891
R-HSA-3299685	Detoxification of Reactive Oxygen Species	0.015948963	0.010386846	0.080432685	0.004558863	C00051	57925;16856
R-HSA-1222499	Latent infection - Other responses of Mtb to phagocytosis	0.02020202	0.013234766	0.080432685	0.003754358	C00051	57925;16856
R-HSA-1614558	Degradation of cysteine and homocysteine	0.02020202	0.01384146	0.080432685	0.002949853	C00051;C00245	15891;16856

R-HSA-2162123	Synthesis of Prostaglandins (PG) and Thromboxanes (TX)	0.022860181	0.014460309	0.080432685	0.004290695	C00051	57925;16856
R-HSA-499943	Interconversion of nucleotide di- and triphosphates	0.026581606	0.018423592	0.080432685	0.004424779	C00064;C00051	58359;57925
R-HSA-9711123	Cellular response to chemical stress	0.023391813	0.019837847	0.080432685	0.007374631	C00051	57925;16856
R-HSA-71406	Pyruvate metabolism and Citric Acid (TCA) cycle	0.023923445	0.023570587	0.080432685	0.004692947	C00051	57925;16856
R-HSA-9635486	Infection with Mycobacterium tuberculosis	0.02764487	0.025140703	0.080432685	0.005363368	C00051	57925;16856
R-HSA-5579022	Defective GGT1 causes GLUTH	0.001063264	0.025542603	0.080432685	1.34E-04	C00051	16856
R-HSA-112313	Neurotransmitter uptake and metabolism In glial cells	0.003189793	0.025542603	0.080432685	4.02E-04	C00064	58359
R-HSA-210455	Astrocytic Glutamate-Glutamine Uptake And Metabolism	0.003189793	0.025542603	0.080432685	4.02E-04	C00064	58359
R-HSA-74259	Purine catabolism	0.031897927	0.027576264	0.080432685	0.004424779	C00385;C00294	17596;17712
R-HSA-156580	Phase II - Conjugation of compounds	0.080808081	0.02786443	0.080432685	0.009654063	C00064;C00051	58359;57925;16856
R-HSA-71291	Metabolism of amino acids and derivatives	0.151515152	0.028301252	0.080432685	0.037543577	C00064;C00051;C00245	58359;15891;57925;16856
R-HSA-5619063	Defective SLC29A3 causes histiocytosis-lymphadenopathy plus syndrome (HLAS)	0.003721425	0.029742844	0.080432685	1.34E-04	C00294	17596
R-HSA-9026762	Biosynthesis of maresin conjugates in tissue regeneration (MCTR)	0.004253057	0.033926984	0.080432685	5.36E-04	C00051	57925

R-HSA-425393	Transport of inorganic cations/anions and amino acids/oligopeptides	0.030834662	0.036364335	0.080432685	0.010056315	C00064;C00245	58359;15891
R-HSA-1643685	Disease	0.18394471	0.036870856	0.080432685	0.116519174	C00221;C00064;C00294;C00051	17596;58359;15903;57925;16856
R-HSA-1614635	Sulfur amino acid metabolism	0.034556087	0.037297691	0.080432685	0.004827031	C00051;C00245	15891;16856
R-HSA-1428517	The citric acid (TCA) cycle and respiratory electron transport	0.031897927	0.037297691	0.080432685	0.008447305	C00051	57925;16856
R-HSA-177162	Conjugation of phenylacetate with glutamine	0.004253057	0.038095078	0.080432685	2.68E-04	C00064	58359
R-HSA-9694548	Maturation of spike protein	0.003189793	0.038095078	0.080432685	4.02E-04	C00221	15903
R-HSA-425407	SLC-mediated transmembrane transport	0.087719298	0.040422699	0.080432685	0.025610083	C00064;C00294;C00245	17596;58359;15891
R-HSA-9683686	Maturation of spike protein	0.003721425	0.042247179	0.080432685	5.36E-04	C00221	15903
R-HSA-425366	Transport of bile salts and organic acids, metal ions and amine compounds	0.040935673	0.05027602	0.080432685	0.008983642	C00064;C00245	58359;15891
R-HSA-2142712	Synthesis of 12-eicosatetraenoic acid derivatives	0.006379585	0.050503619	0.080432685	8.05E-04	C00051	57925
R-HSA-2262752	Cellular responses to stress	0.04040404	0.053483335	0.080432685	0.022660231	C00051	57925;16856
R-HSA-5619044	Defective SLC6A19 causes Hartnup disorder (HND)	0.006911217	0.054608063	0.080432685	1.34E-04	C00064	58359
R-HSA-5659735	Defective SLC6A19 causes Hartnup disorder (HND)	0.006911217	0.054608063	0.080432685	1.34E-04	C00064	58359
R-HSA-5619102	SLC transporter disorders	0.042530569	0.057876602	0.080432685	0.008313221	C00064;C00294	17596;58359

R-HSA-9023661	Biosynthesis of E-series 18(R)-resolvins	0.00744285	0.05869673	0.080432685	9.39E-04	C00051	57925
R-HSA-8953897	Cellular responses to stimuli	0.041467305	0.058995296	0.080432685	0.026816841	C00051	57925;16856
R-HSA-2142688	Synthesis of 5-eicosatetraenoic acids	0.007974482	0.06276967	0.080432685	5.36E-04	C00051	57925
R-HSA-2408499	Formation of selenosugars for excretion	0.006379585	0.066826938	0.080432685	5.36E-04	C00051	57925
R-HSA-2142770	Synthesis of 15-eicosatetraenoic acid derivatives	0.008506114	0.066826938	0.080432685	5.36E-04	C00051	57925
R-HSA-8963684	Tyrosine catabolism	0.007974482	0.066826938	0.080432685	8.05E-04	C00051	57925
R-HSA-159424	Conjugation of carboxylic acids	0.007974482	0.066826938	0.080432685	8.05E-04	C00064	58359
R-HSA-156587	Amino Acid conjugation	0.007974482	0.066826938	0.080432685	8.05E-04	C00064	58359
R-HSA-210500	Glutamate Neurotransmitter Release Cycle	0.004253057	0.070868586	0.080432685	0.001072674	C00064	58359
R-HSA-9026766	Biosynthesis of protectin and resolvin conjugates in tissue regeneration (PCTR and RCTR)	0.009037746	0.070868586	0.080432685	0.001072674	C00051	57925
R-HSA-2161541	Abacavir metabolism	0.009037746	0.070868586	0.080432685	9.39E-04	C00294	17596
R-HSA-9683701	Translation of Structural Proteins	0.00744285	0.070868586	0.080432685	0.001474926	C00221	15903
R-HSA-2408550	Metabolism of ingested H2SeO4 and H2SeO3 into H2Se	0.009569378	0.074894667	0.080432685	9.39E-04	C00051	57925
R-HSA-196836	Vitamin C (ascorbate) metabolism	0.009569378	0.074894667	0.080432685	0.001206758	C00051	57925
R-HSA-2161522	Abacavir transport and metabolism	0.009569378	0.074894667	0.080432685	0.001206758	C00294	17596
R-HSA-8956319	Nucleotide catabolism	0.056353004	0.07546551	0.080432685	0.008447305	C00385;C00294	17596;17712

R-HSA-2142753	Arachidonic acid metabolism	0.0579479	0.077935299	0.080432685	0.010324484	C00051	57925;16856
R-HSA-3700989	Transcriptional Regulation by TP53	0.02020202	0.077935299	0.080432685	0.009519979	C00064;C00051	58359;57925
R-HSA-5683826	Surfactant metabolism	0.008506114	0.078905233	0.080432685	0.001072674	C00294	17596
R-HSA-9018896	Biosynthesis of E-series 18(S)-resolvins	0.01010101	0.078905233	0.080432685	0.001206758	C00051	57925
R-HSA-9020265	Biosynthesis of aspirin-triggered D-series resolvins	0.01010101	0.078905233	0.080432685	0.001340842	C00051	57925
R-HSA-5619115	Disorders of transmembrane transporters	0.051568315	0.080432685	0.080432685	0.010726736	C00064;C00294	17596;58359
R-HSA-204174	Regulation of pyruvate dehydrogenase (PDH) complex	0.00744285	0.082900336	0.082900336	4.02E-04	C00051	16856
R-HSA-1614517	Sulfide oxidation to sulfate	0.008506114	0.082900336	0.082900336	8.05E-04	C00051	16856
R-HSA-83936	Transport of nucleosides and free purine and pyrimidine bases across the plasma membrane	0.011164274	0.08688003	0.08688003	0.002145347	C00294	17596
R-HSA-9026395	Biosynthesis of DHA-derived sulfido conjugates	0.011164274	0.08688003	0.08688003	0.001743095	C00051	57925
R-HSA-500753	Pyrimidine biosynthesis	0.011164274	0.08688003	0.08688003	8.05E-04	C00064	58359
R-HSA-446210	Synthesis of UDP-N-acetyl-glucosamine	0.011164274	0.08688003	0.08688003	9.39E-04	C00064	58359
R-HSA-159418	Recycling of bile acids and salts	0.011164274	0.08688003	0.08688003	0.002279431	C00245	15891
R-HSA-382551	Transport of small molecules	0.116427432	0.08999769	0.08999769	0.054035935	C00064;C00294;C00245	17596;58359;15891
R-HSA-5663205	Infectious disease	0.066985646	0.090836719	0.090836719	0.043979619	C00221;C00051	15903;57925;16856

R-HSA-9018676	Biosynthesis of D-series resolvins	0.012227539	0.094793394	0.094793394	0.001474926	C00051	57925
R-HSA-212436	Generic Transcription Pathway	0.029239766	0.099990128	0.099990128	0.017430947	C00064;C00051	58359;57925
R-HSA-9694635	Translation of Structural Proteins	0.009569378	0.10264574	0.10264574	0.001877179	C00221	15903
R-HSA-73857	RNA Polymerase II Transcription	0.030834662	0.104065349	0.104065349	0.021319389	C00064;C00051	58359;57925
R-HSA-5609975	Diseases associated with glycosylation precursor biosynthesis	0.013822435	0.10654916	0.10654916	0.002145347	C00064	58359
R-HSA-9018679	Biosynthesis of EPA-derived SPMs	0.014354067	0.110437479	0.110437479	0.002279431	C00051	57925
R-HSA-8964539	Glutamate and glutamine metabolism	0.014354067	0.11431075	0.11431075	0.001877179	C00064	58359
R-HSA-379724	tRNA Aminoacylation	0.013822435	0.11431075	0.11431075	0.005631537	C00064	58359
R-HSA-379716	Cytosolic tRNA aminoacylation	0.013822435	0.11431075	0.11431075	0.002815768	C00064	58359
R-HSA-379726	Mitochondrial tRNA aminoacylation	0.013822435	0.11431075	0.11431075	0.002815768	C00064	58359
R-HSA-74160	Gene expression (Transcription)	0.035087719	0.116588786	0.116588786	0.025878252	C00064;C00051	58359;57925
R-HSA-5423646	Aflatoxin activation and detoxification	0.015417331	0.118169022	0.118169022	0.002413516	C00051	57925
R-HSA-196849	Metabolism of water-soluble vitamins and cofactors	0.069112174	0.119429407	0.119429407	0.019308126	C00064;C00051	58359;57925
R-HSA-2142691	Synthesis of Leukotrienes (LT) and Eoxins (EX)	0.016480595	0.125840776	0.125840776	0.0025476	C00051	57925
R-HSA-9678108	SARS-CoV-1 Infection	0.012759171	0.125840776	0.125840776	0.005363368	C00221	15903
R-HSA-74217	Purine salvage	0.017012228	0.129654358	0.129654358	0.00160901	C00294	17596

R-HSA-8963691	Phenylalanine and tyrosine metabolism	0.017012228	0.133453145	0.133453145	0.00160901	C00051	57925
R-HSA-9694516	SARS-CoV-2 Infection	0.014354067	0.137237187	0.137237187	0.00670421	C00221	15903
R-HSA-9679506	SARS-CoV Infections	0.015417331	0.144761234	0.144761234	0.012201663	C00221	15903
R-HSA-73817	Purine ribonucleoside monophosphate biosynthesis	0.019670388	0.148501339	0.148501339	0.002145347	C00064	58359
R-HSA-5668914	Diseases of metabolism	0.085061138	0.162639733	0.162639733	0.024671494	C00064;C00051	58359;16856
R-HSA-72766	Translation	0.018607124	0.163316796	0.163316796	0.011128989	C00064	58359
R-HSA-211859	Biological oxidations	0.173312068	0.166809268	0.166809268	0.025073746	C00064;C00051	58359;57925;16856
R-HSA-156581	Methylation	0.02020202	0.166984666	0.166984666	0.001743095	C00051	57925
R-HSA-8956321	Nucleotide salvage	0.024455077	0.181513637	0.181513637	0.002949853	C00294	17596
R-HSA-1222556	ROS and RNS production in phagocytes	0.024455077	0.181513637	0.181513637	0.006033789	C00051	57925
R-HSA-8956320	Nucleotide biosynthesis	0.024986709	0.185110495	0.185110495	0.002949853	C00064	58359
R-HSA-196854	Metabolism of vitamins and cofactors	0.099415205	0.208776022	0.208776022	0.027353178	C00064;C00051	58359;57925
R-HSA-3781865	Diseases of glycosylation	0.029771398	0.216855392	0.216855392	0.010324484	C00064	58359
R-HSA-193368	Synthesis of bile acids and bile salts via 7alpha-hydroxycholesterol	0.028708134	0.220313827	0.220313827	0.005229284	C00245	15891
R-HSA-112310	Neurotransmitter release cycle	0.026049973	0.227189996	0.227189996	0.004827031	C00064	58359
R-HSA-196807	Nicotinate metabolism	0.028176502	0.227189996	0.227189996	0.003888442	C00064	58359
R-HSA-2408522	Selenoamino acid metabolism	0.030834662	0.237403196	0.237403196	0.004424779	C00051	57925
R-HSA-5579029	Metabolic disorders of biological oxidation enzymes	0.028176502	0.237403196	0.237403196	0.0050952	C00051	16856

R-HSA-425397	Transport of vitamins, nucleosides, and related molecules	0.034556087	0.247496242	0.247496242	0.005229284	C00294	17596
R-HSA-446219	Synthesis of substrates in N-glycan biosythesis	0.036150984	0.260768871	0.260768871	0.006570126	C00064	58359
R-HSA-9018677	Biosynthesis of DHA-derived SPMs	0.038277512	0.270586357	0.270586357	0.007776884	C00051	57925
R-HSA-446193	Biosynthesis of the N-glycan precursor (dolichol lipid-linked oligosaccharide, LLO) and transfer to a nascent protein	0.043062201	0.302479781	0.302479781	0.008447305	C00064	58359
R-HSA-392499	Metabolism of proteins	0.125465178	0.310668384	0.310668384	0.061410566	C00064;C00294	17596;58359
R-HSA-192105	Synthesis of bile acids and bile salts	0.041998937	0.311802933	0.311802933	0.011128989	C00245	15891
R-HSA-194068	Bile acid and bile salt metabolism	0.042530569	0.314885941	0.314885941	0.013274336	C00245	15891
R-HSA-8978868	Fatty acid metabolism	0.133971292	0.315741454	0.315741454	0.028828104	C00051	57925;16856
R-HSA-112315	Transmission across Chemical Synapses	0.037214248	0.350936135	0.350936135	0.017967283	C00064	58359
R-HSA-112316	Neuronal System	0.037214248	0.350936135	0.350936135	0.02105122	C00064	58359
R-HSA-446203	Asparagine N-linked glycosylation	0.061669325	0.404574826	0.404574826	0.017699115	C00064	58359
R-HSA-9018678	Biosynthesis of specialized proresolving mediators (SPMs)	0.074428495	0.464209601	0.464209601	0.016626441	C00051	57925
R-HSA-8957322	Metabolism of steroids	0.080808081	0.507112818	0.507112818	0.026682757	C00245	15891
R-HSA-597592	Post-translational protein modification	0.092503987	0.55747605	0.55747605	0.038079914	C00064	58359
R-HSA-168249	Innate Immune System	0.06539075	0.651503891	0.651503891	0.056181282	C00051	57925
R-HSA-168256	Immune System	0.078681552	0.709183677	0.709183677	0.093188522	C00051	57925
R-HSA-556833	Metabolism of lipids	0.337054758	0.709353134	0.709353134	0.11893269	C00051;C00245	15891;57925;16856

R-HSA-1430728	Metabolism	0.758639022	0.855482503	0.855482503	0.288549209	C00385;C00064;C00294;C00051;C00245	17596;58359;15891;17712;57925;16856

Supplementary table 10a: Reactome pathway analysis of upregulated metabolites in epithelial regions of post-chemo versus pre-chemo PR tissues

PR= poor response; ER= excellent response

Name	Parent Folder	Percent Overlap	Overlapping Entities	p-value	Jaccard similarity
GABRB3 Mutation and Adenosine Deaminase Polymorphism	Sleep Dysregulation; Sleep Dysregulation (Neurological Diseases); nerve tissue; nervous system	7	inosine;glutamine	0.000103397	0.060606061
Uric Acid Synthesis in Gout	Gout; Gout (Immune System Diseases); digestive system	6	xanthine;inosine	0.000157594	0.051282051
template	metabolic cycles	2	[illegible] e	0.000416646	0.021276596
Purine Metabolism	Nucleotides Metabolism; Nucleotides Metabolism (Metabolic Pathway); generic; generic	1	inosine;glutamine;xanthin e	0.00093853	0.016304348
Amino sugars synthesis (bacteria) template	Bacteria	3	glutamine;beta-D-glucose	0.001582612	0.034482759
Amino sugars synthesis (Eukaryots) template	Eukaryots	2	glutamine;beta-D-glucose	0.002881581	0.026315789
Phenylalanine metabolism template	Aminoacid metabolism and interconvertion	2	ascorbic acid;glutamine	0.002964602	0.025974026
Cellulose degradation template	catabolilsm	16	beta-D-glucose	0.007304317	0.071428571
Phenylalanine and Tyrosine Metabolism	Amino Acids Metabolism; Amino Acids Metabolism (Metabolic Pathway); generic; generic	1	glutamine;ascorbic acid	0.010689217	0.014084507
Androgen Receptor/SGK1 Signaling	Prostate Cancer; Prostate Cancer (Urology/Nephrology Diseases); Prostate Cancer (Urology/Nephrology Diseases); urinary tract	5	glutamine	0.011056805	0.04
Epinephrine/Norepineph rine Release	Neurotransmitter Release Cycles; Neurotransmitter Release Cycles (Nociception Pathways); nerve tissue; nervous system; secretory vesicle	5	ascorbic acid	0.01154225	0.035714286
Dopamine Mediated Glutamate Release/Uptake Circle in Neuron in Migraine	Amino Acid Derived Hormones; Amino Acid Derived Hormones (Hormones Pathways); nerve tissue; nervous system; secretory vesicle; synapse	4	glutamine	0.01269279	0.033333333
Glutamate Release	Neurotransmitter Release Cycles; Neurotransmitter Release Cycles (Nociception Pathways); nerve tissue; nervous system; secretory vesicle	4	glutamine	0.013267808	0.032258065
Adenosine Role in Sleep Regulation	Sleep Regulation; Sleep Regulation (Biological Process); nerve tissue; nervous system	4	glutamine	0.013267808	0.032258065
Agomelatine Antidepressant Action	Sleep Regulation; Sleep Regulation (Biological Process); nerve tissue; nervous system	3	glutamine	0.01499186	0.029411765
SIRT4 Signaling in Aging	Genomic Instability Associated with Aging; Genomic Instability Associated with Aging (Aging Biology); generic; generic; nucleus	3	glutamine	0.01499186	0.029411765
Vitamin C Related Norepinephrine Synthesis	Vitamins Biology; Vitamins Biology (Biological Process); nerve tissue; nervous system	3	ascorbic acid	0.015566208	0.028571429
MTOR/TP53 Regulates Cell Metabolism	Eating Behavior and Metabolism Regulation; Eating Behavior and Metabolism Regulation (Biological Process); Hallmarks of Cancer (7): Deregulated Metabolism; generic; generic; oncogenic; oncogenic	2	glutamine	0.015609432	0.018181818

GABA Release	Neurotransmitter Release Cycles; Neurotransmitter Release Cycles (Nociception Pathways); nerve tissue; nervous system; secretory vesicle	3	glutamine	0.01614039	0.027777778
Sucrose import (cell wall) template	catabolilsm	6	beta-D-glucose	0.018175031	0.043478261
IAA storage via glucoside conjugation template	Plant hormone storage and degradation	6	beta-D-glucose	0.019376565	0.041666667
Glutamine in Cancer Metabolism	Hallmarks of Cancer (7): Deregulated Metabolism; Hallmarks of Cancer (7): Deregulated Metabolism (Hallmarks of Cancer); oncogenic; oncogenic	1	glutamine	0.019926935	0.014705882
Astrocyte Dysfunction and GABA Signaling Deficiency	Epileptiform Disorders; Epileptiform Disorders (Neurological Diseases); nerve tissue; nervous system; synapse	3	glutamine	0.020093348	0.025641026
Aminobenzoate degradation template	catabolilsm	5	glutamine	0.020576839	0.04
Glutamate Overdose and Aura Effect in Familial Hemiplegic Migraine Type 3	Migraine, Familial Hemiplegic; Migraine, Familial Hemiplegic (Neurological Diseases); nervous system; plasma membrane	3	glutamine	0.02137921	0.024390244

Supplementary table 10b: Pathway studio pathway analysis of upregulated metabolites in epithelial regions of post-chemo versus pre-chemo PR tissues

PR= poor response; ER= excellent response

Pathway identifier	Pathway name	Entities ratio	Entities pValue	Entities FDR	Reactions ratio	Submitted entities found	Mapped entities
R-HSA-70635	Urea cycle	0.011695906	0.003797549	0.141006533	0.001072674	C00122;C00049	29991;29806
R-HSA-8963693	Aspartate and asparagine metabolism	0.013290803	0.005256768	0.141006533	0.001474926	C00049;C00245	29991;15891
R-HSA-8963691	Phenylalanine and tyrosine metabolism	0.017012228	0.008336754	0.141006533	0.00160901	C00122;C00049	29991;29806
R-HSA-73817	Purine ribonucleoside monophosphate biosynthesis	0.019670388	0.010386846	0.141006533	0.002145347	C00122;C00049	29991;29806
R-HSA-8956320	Nucleotide biosynthesis	0.024986709	0.016388879	0.141006533	0.002949853	C00122;C00049	29991;29806
R-HSA-5619062	Defective SLC1A3 causes episodic ataxia 6 (EA6)	0.003189793	0.025542603	0.141006533	1.34E-04	C00049	29991
R-HSA-5619067	Defective SLC1A1 is implicated in schizophrenia 18 (SCZD18) and dicarboxylic aminoaciduria (DCBXA)	0.003189793	0.025542603	0.141006533	1.34E-04	C00049	29991
R-HSA-425393	Transport of inorganic cations/anions and amino acids/oligopeptides	0.030834662	0.036364335	0.141006533	0.010056315	C00049;C00245	29991;15891
R-HSA-9694548	Maturation of spike protein	0.003189793	0.038095078	0.141006533	4.02E-04	C00221	15903
R-HSA-9683686	Maturation of spike protein	0.003721425	0.042247179	0.141006533	5.36E-04	C00221	15903
R-HSA-8963684	Tyrosine catabolism	0.007974482	0.066826938	0.141006533	8.05E-04	C00122	29806
R-HSA-5676934	Protein repair	0.006911217	0.070868586	0.141006533	9.39E-04	C00049	29991
R-HSA-9683701	Translation of Structural Proteins	0.00744285	0.070868586	0.141006533	0.001474926	C00221	15903
R-HSA-500753	Pyrimidine biosynthesis	0.011164274	0.08688003	0.141006533	8.05E-04	C00049	29991
R-HSA-8964208	Phenylalanine metabolism	0.011164274	0.08688003	0.141006533	8.05E-04	C00049	29991
R-HSA-159418	Recycling of bile acids and salts	0.011164274	0.08688003	0.141006533	0.002279431	C00245	15891
R-HSA-2024096	HS-GAG degradation	0.011695906	0.090844365	0.141006533	0.001743095	C00049	29991
R-HSA-9694635	Translation of Structural Proteins	0.009569378	0.10264574	0.141006533	0.001877179	C00221	15903
R-HSA-8964539	Glutamate and glutamine metabolism	0.014354067	0.11431075	0.141006533	0.001877179	C00049	29991
R-HSA-379724	tRNA Aminoacylation	0.013822435	0.11431075	0.141006533	0.005631537	C00049	29991
R-HSA-379716	Cytosolic tRNA aminoacylation	0.013822435	0.11431075	0.141006533	0.002815768	C00049	29991
R-HSA-379726	Mitochondrial tRNA aminoacylation	0.013822435	0.11431075	0.141006533	0.002815768	C00049	29991

R-HSA-71403	Citric acid cycle (TCA cycle)	0.014885699	0.118169022	0.141006533	0.002279431	C00122	18012
R-HSA-71291	Metabolism of amino acids and derivatives	0.151515152	0.12439913	0.141006533	0.037543577	C00122;C00049;C00245	29991;15891;29806
R-HSA-9678108	SARS-CoV-1 Infection	0.012759171	0.125840776	0.141006533	0.005363368	C00221	15903
R-HSA-442660	Na+/Cl- dependent neurotransmitter transporters	0.017012228	0.129654358	0.141006533	0.002011263	C00245	15891
R-HSA-70263	Gluconeogenesis	0.017012228	0.133453145	0.141006533	0.003486189	C00049	29991
R-HSA-1638091	Heparan sulfate/heparin (HS-GAG) metabolism	0.01754386	0.133453145	0.141006533	0.003888442	C00049	29991
R-HSA-9694516	SARS-CoV-2 Infection	0.014354067	0.137237187	0.141006533	0.00670421	C00221	15903
R-HSA-352230	Amino acid transport across the plasma membrane	0.018607124	0.141006533	0.141006533	0.004827031	C00245	15891
R-HSA-15869	Metabolism of nucleotides	0.082402977	0.142835465	0.142835465	0.018771789	C00122;C00049	29991;29806
R-HSA-9679506	SARS-CoV Infections	0.015417331	0.144761234	0.144761234	0.012201663	C00221	15903
R-HSA-72766	Translation	0.018607124	0.163316796	0.163316796	0.011128989	C00049	29991
R-HSA-1614558	Degradation of cysteine and homocysteine	0.02020202	0.170638237	0.170638237	0.002949853	C00245	15891
R-HSA-389661	Glyoxylate metabolism and glycine degradation	0.021796917	0.177902673	0.177902673	0.003083937	C00049	29991
R-HSA-425407	SLC-mediated transmembrane transport	0.087719298	0.191005445	0.191005445	0.025610083	C00049;C00245	29991;15891
R-HSA-70326	Glucose metabolism	0.022328549	0.213383328	0.213383328	0.006436042	C00049	29991
R-HSA-71406	Pyruvate metabolism and Citric Acid (TCA) cycle	0.023923445	0.220313827	0.220313827	0.004692947	C00122	18012
R-HSA-193368	Synthesis of bile acids and bile salts via 7alpha-hydroxycholesterol	0.028708134	0.220313827	0.220313827	0.005229284	C00245	15891
R-HSA-1630316	Glycosaminoglycan metabolism	0.030834662	0.223758679	0.223758679	0.010458568	C00049	29991
R-HSA-1428517	The citric acid (TCA) cycle and respiratory electron transport	0.031897927	0.273833	0.273833	0.008447305	C00122	18012
R-HSA-1614635	Sulfur amino acid metabolism	0.034556087	0.273833	0.273833	0.004827031	C00245	15891
R-HSA-382551	Transport of small molecules	0.116427432	0.308977394	0.308977394	0.054035935	C00049;C00245	29991;15891
R-HSA-192105	Synthesis of bile acids and bile salts	0.041998937	0.311802933	0.311802933	0.011128989	C00245	15891

R-HSA-194068	Bile acid and bile salt metabolism	0.042530569	0.314885941	0.314885941	0.013274336	C00245	15891
R-HSA-425366	Transport of bile salts and organic acids, metal ions and amine compounds	0.040935673	0.314885941	0.314885941	0.008983642	C00245	15891
R-HSA-5619102	SLC transporter disorders	0.042530569	0.336125467	0.336125467	0.008313221	C00049	29991
R-HSA-5619115	Disorders of transmembrane transporters	0.051568315	0.390855945	0.390855945	0.010726736	C00049	29991
R-HSA-8957322	Metabolism of steroids	0.080808081	0.507112818	0.507112818	0.026682757	C00245	15891
R-HSA-9717207	Sensory perception of sweet, bitter, and umami (glutamate) taste	0.047315258	0.525179967	0.525179967	0.002815768	C00049	29991
R-HSA-9717189	Sensory perception of taste	0.049441786	0.533990808	0.533990808	0.003352105	C00049	29991
R-HSA-71387	Metabolism of carbohydrates	0.081871345	0.551177711	0.551177711	0.030705283	C00049	29991
R-HSA-1643685	Disease	0.18394471	0.657004584	0.657004584	0.116519174	C00221;C00049	29991;15903
R-HSA-392499	Metabolism of proteins	0.125465178	0.697487564	0.697487564	0.061410566	C00049	29991
R-HSA-5663205	Infectious disease	0.066985646	0.697487564	0.697487564	0.043979619	C00221	15903
R-HSA-9709957	Sensory Perception	0.101010101	0.736684451	0.736684451	0.018369536	C00049	29991
R-HSA-556833	Metabolism of lipids	0.337054758	0.984546915	0.984546915	0.11893269	C00245	15891
R-HSA-1430728	Metabolism	0.758639022	0.99222071	0.99222071	0.288549209	C00122;C00049;C00245	29991;18012;15891;29806

Supplementary table 11a: Reactome pathway analysis of upregulated metabolites in stromal regions of post-chemo versus pre-chemo PR tissues

PR= poor response; ER= excellent response

Name	Parent Folder	Percent Overlap	Overlapping Entities	p-value	Jaccard similarity
Hyperammonemia Type I	Urea Cycle Disorders; Urea Cycle Disorders (Urology/Nephrology Diseases); mitochondria	4	fumarate;aspartate	4.58934E-05	0.040816327
Hyperammonemia Type II	Urea Cycle Disorders; Urea Cycle Disorders (Urology/Nephrology Diseases); generic; mitochondria	4	fumarate;aspartate	5.04241E-05	0.039215686
Citrullinemia Type I	Urea Cycle Disorders; Urea Cycle Disorders (Urology/Nephrology Diseases); mitochondria	4	fumarate;aspartate	5.27694E-05	0.038461538
Argininosuccinic Aciduria in Urea Cycle Disorders	Urea Cycle Disorders; Urea Cycle Disorders (Urology/Nephrology Diseases)	4	fumarate;aspartate	5.76199E-05	0.037037037
Citrullinemia Type II	Urea Cycle Disorders; Urea Cycle Disorders (Urology/Nephrology Diseases); mitochondria	3	aspartate;fumarate	6.52955E-05	0.035087719
Argininemia	Urea Cycle Disorders; Urea Cycle Disorders (Urology/Nephrology Diseases)	3	aspartate;fumarate	7.91541E-05	0.032258065
Glutamine in Cancer Metabolism	Hallmarks of Cancer (7): Deregulated Metabolism; Hallmarks of Cancer (7): Deregulated Metabolism (Hallmarks of Cancer); oncogenic; oncogenic	3	aspartate;fumarate	0.000390594	0.029850746
precursor biosynthesis template	anabolism	4	fumarate;aspartate	0.001425821	0.040816327
Metabolic Reprogramming in Cancer: Overview	Hallmarks of Cancer (7): Deregulated Metabolism; Hallmarks of Cancer (7): Deregulated Metabolism (Hallmarks of Cancer); oncogenic; oncogenic	1	aspartate;fumarate	0.001446523	0.016393443
Pentose-Phosphate Shunt	Carbohydrates Metabolism; Carbohydrates Metabolism (Metabolic Pathway); generic; generic	4	gluconate;beta-D-glucose	0.001709179	0.037735849
Reductive TCA cycle template	metabolic cycles	4	cis-aconitate;fumarate	0.001709179	0.037735849

Tricarboxylic Acid Cycle	Carboxylic Acids Metabolism; Carboxylic Acids Metabolism (Metabolic Pathway); Electron Transport Chain; generic; generic	3	cis-aconitate;fumarate	0.002436913	0.032258065
IMP purine precursor biosynthesis template	anabolism	3	aspartate;fumarate	0.002436913	0.032258065
Pentose phosphate shunt template	catabolilsm	3	gluconate;beta-D-glucose	0.002436913	0.032258065
TCA cycle template	metabolic cycles	3	cis-aconitate;fumarate	0.002706917	0.030769231
Asp/Asn metabolism template	Aminoacid metabolism and interconvertion	3	aspartate;fumarate	0.002894461	0.029850746
Tyrosine degradation template	Aminoacid degradation	3	aspartate;fumarate	0.003187022	0.028571429
Aspartate Metabolism	Amino Acids Metabolism; Amino Acids Metabolism (Metabolic Pathway); generic; generic	3	aspartate;fumarate	0.003492998	0.02739726
Nicotinate and NAD metabolism template	metabolic cycles	2	aspartate;fumarate	0.005221023	0.022727273
Vitamin B6 (Pyridoxine) Metabolism	Cofactors and Vitamins Metabolism; Cofactors and Vitamins Metabolism (Metabolic Pathway); Vitamins Biology; generic; generic	4	aspartate	0.006076389	0.034482759
Urea cycle and Arginine metabolism template	Aminoacid metabolism and interconvertion	2	aspartate;fumarate	0.006138373	0.021052632
Urea Cycle and Arginine Metabolism	Amino Acids Metabolism; Amino Acids Metabolism (Metabolic Pathway); generic; generic	2	aspartate;fumarate	0.00641321	0.020618557
Cellulose degradation template	catabolilsm	16	beta-D-glucose	0.008344152	0.071428571
Pyrimidine Metabolism	Nucleotides Metabolism; Nucleotides Metabolism (Metabolic Pathway); generic; generic	1	aspartate;uridine	0.010314206	0.016393443

Brain Dysfunction in Phenylketonuria	Phenylketonuria; Phenylketonuria (Endocrine and Metabolic Diseases); Phenylketonuria (Endocrine and Metabolic Diseases); mitochondria; nervous system	3	aspartate	0.010420836	0.025

Supplementary table 11b: pathway studio pathway analysis of upregulated metabolites in stromal regions of post-chemo versus pre-chemo PR tissues

PR= poor response; ER= excellent response

Pathway identifier	Pathway name	Entities ratio	Entities pValue	Entities FDR	Reactions ratio	Submitted entities found	Mapped entities
R-HSA-916853	Degradation of GABA	0.005316321	4.10E-04	0.068422429	4.02E-04	C00042;C00025	30031;29985
R-HSA-888590	GABA synthesis, release, reuptake and degradation	0.009569378	0.002597503	0.088420593	0.001340842	C00042;C00025	30031;29985
R-HSA-8849932	Synaptic adhesion-like molecules	0.001063264	0.005727908	0.088420593	4.02E-04	C00025	29985
R-HSA-6794362	Protein-protein interactions at synapses	0.001063264	0.005727908	0.088420593	4.02E-04	C00025	29985
R-HSA-500657	Presynaptic function of Kainate receptors	0.001063264	0.005727908	0.088420593	2.68E-04	C00025	29985
R-HSA-5619076	Defective SLC17A8 causes autosomal dominant deafness 25 (DFNA25)	0.001063264	0.005727908	0.088420593	1.34E-04	C00025	29985
R-HSA-451307	Activation of Na-permeable kainate receptors	0.001594896	0.008581593	0.088420593	2.68E-04	C00025	29985
R-HSA-5578999	Defective GCLC causes HAGGSD	0.001594896	0.008581593	0.088420593	1.34E-04	C00025	29985
R-HSA-112310	Neurotransmitter release cycle	0.026049973	0.011081996	0.088420593	0.004827031	C00042;C00025	30031;29985
R-HSA-888568	GABA synthesis	0.002126528	0.01142845	0.088420593	2.68E-04	C00025	29985
R-HSA-433137	Sodium-coupled sulphate, di- and tri-carboxylate transporters	0.002126528	0.01142845	0.088420593	6.70E-04	C00042	30031
R-HSA-8964540	Alanine metabolism	0.002126528	0.014268491	0.088420593	5.36E-04	C00025	29985
R-HSA-442982	Ras activation upon Ca2+ influx through NMDA receptor	0.003189793	0.017101729	0.088420593	2.68E-04	C00025	29985

R-HSA-5619062	Defective SLC1A3 causes episodic ataxia 6 (EA6)	0.003189793	0.017101729	0.088420593	1.34E-04	C00025	29985
R-HSA-5619067	Defective SLC1A1 is implicated in schizophrenia 18 (SCZD18) and dicarboxylic aminoaciduria (DCBXA)	0.003189793	0.017101729	0.088420593	1.34E-04	C00025	29985
R-HSA-3214842	HDMs demethylate histones	0.002658161	0.017101729	0.088420593	0.002279431	C00042	30031
R-HSA-112313	Neurotransmitter uptake and metabolism In glial cells	0.003189793	0.017101729	0.088420593	4.02E-04	C00025	29985
R-HSA-210455	Astrocytic Glutamate-Glutamine Uptake And Metabolism	0.003189793	0.017101729	0.088420593	4.02E-04	C00025	29985
R-HSA-9617324	Negative regulation of NMDA receptor-mediated neuronal transmission	0.003189793	0.017101729	0.088420593	5.36E-04	C00025	29985
R-HSA-1234174	Cellular response to hypoxia	0.003189793	0.019928178	0.088420593	0.001340842	C00042	30031
R-HSA-1234176	Oxygen-dependent proline hydroxylation of Hypoxia-inducible Factor Alpha	0.003189793	0.019928178	0.088420593	0.001206758	C00042	30031
R-HSA-9664535	LTC4-CYSLTR mediated IL4 production	0.003721425	0.019928178	0.088420593	4.02E-04	C00025	29985
R-HSA-112122	ALKBH2 mediated reversal of alkylation damage	0.003721425	0.02274785	0.088420593	8.05E-04	C00042	30031
R-HSA-112126	ALKBH3 mediated reversal of alkylation damage	0.003721425	0.02274785	0.088420593	8.05E-04	C00042	30031

R-HSA-9026762	Biosynthesis of maresin conjugates in tissue regeneration (MCTR)	0.004253057	0.02274785	0.088420593	5.36E-04	C00025	29985
R-HSA-8955332	Carboxyterminal post-translational modifications of tubulin	0.004784689	0.02556076	0.088420593	8.05E-04	C00025	29985
R-HSA-5221030	TET1,2,3 and TDG demethylate DNA	0.004784689	0.028366918	0.088420593	6.70E-04	C00042	30031
R-HSA-73943	Reversal of alkylation damage by DNA dioxygenases	0.004784689	0.028366918	0.088420593	0.001743095	C00042	30031
R-HSA-442742	CREB1 phosphorylation through NMDA receptor-mediated activation of RAS signaling	0.005316321	0.028366918	0.088420593	8.05E-04	C00025	29985
R-HSA-77108	Utilization of Ketone Bodies	0.005316321	0.028366918	0.088420593	4.02E-04	C00042	30031
R-HSA-1650814	Collagen biosynthesis and modifying enzymes	0.004784689	0.028366918	0.088420593	0.00160901	C00042	30031
R-HSA-112315	Transmission across Chemical Synapses	0.037214248	0.029068347	0.088420593	0.017967283	C00042;C00025	30031;29985
R-HSA-112316	Neuronal System	0.037214248	0.029068347	0.088420593	0.02105122	C00042;C00025	30031;29985
R-HSA-73942	DNA Damage Reversal	0.005316321	0.031166339	0.088420593	0.002011263	C00042	30031
R-HSA-399710	Activation of AMPA receptors	0.001594896	0.033959036	0.088420593	6.70E-04	C00025	29985
R-HSA-451308	Activation of Ca-permeable Kainate Receptor	0.001594896	0.033959036	0.088420593	2.68E-04	C00025	29985

R-HSA-5625886	Activated PKN1 stimulates transcription of AR (androgen receptor) regulated genes KLK2 and KLK3	0.005847953	0.033959036	0.088420593	0.001474926	C00042	30031
R-HSA-451326	Activation of kainate receptors upon glutamate binding	0.002126528	0.036745021	0.088420593	8.05E-04	C00025	29985
R-HSA-451306	Ionotropic activity of kainate receptors	0.002126528	0.036745021	0.088420593	5.36E-04	C00025	29985
R-HSA-70688	Proline catabolism	0.005847953	0.036745021	0.088420593	5.36E-04	C00025	29985
R-HSA-2559580	Oxidative Stress Induced Senescence	0.004253057	0.036745021	0.088420593	0.002413516	C00042	30031
R-HSA-9620244	Long-term potentiation	0.003189793	0.042296908	0.088420593	8.05E-04	C00025	29985
R-HSA-2299718	Condensation of Prophase Chromosomes	0.005316321	0.042296908	0.088420593	6.70E-04	C00042	30031
R-HSA-1474290	Collagen formation	0.00744285	0.042296908	0.088420593	0.002011263	C00042	30031
R-HSA-9662360	Sensory processing of sound by inner hair cells of the cochlea	0.003189793	0.042296908	0.088420593	9.39E-04	C00025	29985
R-HSA-8963684	Tyrosine catabolism	0.007974482	0.045062836	0.088420593	8.05E-04	C00025	29985
R-HSA-428643	Organic anion transporters	0.007974482	0.045062836	0.088420593	0.001206758	C00025	29985
R-HSA-5625740	RHO GTPases activate PKNs	0.007974482	0.045062836	0.088420593	0.002279431	C00042	30031
R-HSA-438066	Unblocking of NMDA receptors, glutamate binding and activation	0.003189793	0.047822103	0.088420593	6.70E-04	C00025	29985
R-HSA-210500	Glutamate Neurotransmitter Release Cycle	0.004253057	0.047822103	0.088420593	0.001072674	C00025	29985

R-HSA-399721	Glutamate binding, activation of AMPA receptors and synaptic plasticity	0.004253057	0.047822103	0.088420593	0.001206758	C00025	29985
R-HSA-71262	Carnitine synthesis	0.008506114	0.047822103	0.088420593	8.05E-04	C00042	30031
R-HSA-9026766	Biosynthesis of protectin and resolvin conjugates in tissue regeneration (PCTR and RCTR)	0.009037746	0.047822103	0.088420593	0.001072674	C00025	29985
R-HSA-174403	Glutathione synthesis and recycling	0.00744285	0.050574723	0.088420593	0.001206758	C00025	29985
R-HSA-9659379	Sensory processing of sound	0.004784689	0.050574723	0.088420593	0.001743095	C00025	29985
R-HSA-977347	Serine biosynthesis	0.008506114	0.053320709	0.088420593	9.39E-04	C00025	29985
R-HSA-6782861	Synthesis of wybutosine at G37 of tRNA(Phe)	0.00744285	0.053320709	0.088420593	0.001072674	C00042	30031
R-HSA-74182	Ketone body metabolism	0.01010101	0.053320709	0.088420593	0.001474926	C00042	30031
R-HSA-2559583	Cellular Senescence	0.00744285	0.053320709	0.088420593	0.004290695	C00042	30031
R-HSA-74160	Gene expression (Transcription)	0.035087719	0.055430692	0.088420593	0.025878252	C00042;C00025	30031;29985
R-HSA-9026395	Biosynthesis of DHA-derived sulfido conjugates	0.011164274	0.058792829	0.088420593	0.001743095	C00025	29985
R-HSA-4839726	Chromatin organization	0.008506114	0.058792829	0.088420593	0.009654063	C00042	30031
R-HSA-3247509	Chromatin modifying enzymes	0.008506114	0.058792829	0.088420593	0.009654063	C00042	30031
R-HSA-500753	Pyrimidine biosynthesis	0.011164274	0.058792829	0.088420593	8.05E-04	C00025	29985
R-HSA-446210	Synthesis of UDP-N-acetyl-glucosamine	0.011164274	0.058792829	0.088420593	9.39E-04	C00025	29985

R-HSA-212165	Epigenetic regulation of gene expression	0.009037746	0.061518989	0.088420593	0.002279431	C00042	30031
R-HSA-70635	Urea cycle	0.011695906	0.061518989	0.088420593	0.001072674	C00025	29985
R-HSA-500792	GPCR ligand binding	0.072833599	0.065162755	0.088420593	0.011665326	C00042;C00025	30031;29985
R-HSA-8963693	Aspartate and asparagine metabolism	0.013290803	0.07235792	0.088420593	0.001474926	C00025	29985
R-HSA-70921	Histidine catabolism	0.011695906	0.07235792	0.088420593	0.001072674	C00025	29985
R-HSA-389599	Alpha-oxidation of phytanate	0.013290803	0.07235792	0.088420593	0.001072674	C00042	30031
R-HSA-5628897	TP53 Regulates Metabolic Genes	0.013822435	0.07235792	0.088420593	0.001877179	C00025	29985
R-HSA-8964539	Glutamate and glutamine metabolism	0.014354067	0.07773814	0.088420593	0.001877179	C00025	29985
R-HSA-379724	tRNA Aminoacylation	0.013822435	0.07773814	0.088420593	0.005631537	C00025	29985
R-HSA-379716	Cytosolic tRNA aminoacylation	0.013822435	0.07773814	0.088420593	0.002815768	C00025	29985
R-HSA-379726	Mitochondrial tRNA aminoacylation	0.013822435	0.07773814	0.088420593	0.002815768	C00025	29985
R-HSA-71403	Citric acid cycle (TCA cycle)	0.014885699	0.080418482	0.088420593	0.002279431	C00042	30031
R-HSA-196757	Metabolism of folate and pterines	0.015417331	0.080418482	0.088420593	0.004022526	C00025	29985
R-HSA-5423646	Aflatoxin activation and detoxification	0.015417331	0.080418482	0.088420593	0.002413516	C00025	29985
R-HSA-68875	Mitotic Prophase	0.008506114	0.08309233	0.088420593	0.003620274	C00042	30031
R-HSA-2142691	Synthesis of Leukotrienes (LT) and Eoxins (EX)	0.016480595	0.085759696	0.088420593	0.0025476	C00025	29985
R-HSA-6782315	tRNA modification in the nucleus and cytosol	0.014354067	0.088420593	0.088420593	0.004424779	C00042	30031
R-HSA-5673001	RAF/MAP kinase cascade	0.013290803	0.088420593	0.088420593	0.006838294	C00025	29985
R-HSA-5684996	MAPK1/MAPK3 signaling	0.013290803	0.088420593	0.088420593	0.007240547	C00025	29985

R-HSA-5683057	MAPK family signaling cascades	0.013290803	0.088420593	0.088420593	0.009117726	C00025	29985
R-HSA-8963691	Phenylalanine and tyrosine metabolism	0.017012228	0.091075032	0.091075032	0.00160901	C00025	29985
R-HSA-438064	Post NMDA receptor activation events	0.006379585	0.091075032	0.091075032	0.004692947	C00025	29985
R-HSA-70263	Gluconeogenesis	0.017012228	0.091075032	0.091075032	0.003486189	C00025	29985
R-HSA-425407	SLC-mediated transmembrane transport	0.087719298	0.095064814	0.095064814	0.025610083	C00042;C00025	30031;29985
R-HSA-71064	Lysine catabolism	0.017012228	0.096364591	0.096364591	0.001743095	C00025	29985
R-HSA-156590	Glutathione conjugation	0.016480595	0.096364591	0.096364591	0.001877179	C00025	29985
R-HSA-71240	Tryptophan catabolism	0.018075492	0.096364591	0.096364591	0.002145347	C00025	29985
R-HSA-352230	Amino acid transport across the plasma membrane	0.018607124	0.096364591	0.096364591	0.004827031	C00025	29985
R-HSA-72306	tRNA processing	0.016480595	0.098999735	0.098999735	0.005765621	C00042	30031
R-HSA-73817	Purine ribonucleoside monophosphate biosynthesis	0.019670388	0.101628472	0.101628472	0.002145347	C00025	29985
R-HSA-68886	M Phase	0.012227539	0.101628472	0.101628472	0.007776884	C00042	30031
R-HSA-418594	G alpha (i) signalling events	0.0579479	0.104060549	0.104060549	0.009117726	C00042;C00025	30031;29985
R-HSA-442755	Activation of NMDA receptors and postsynaptic events	0.008506114	0.106866775	0.106866775	0.006167873	C00025	29985
R-HSA-70895	Branched-chain amino acid catabolism	0.019670388	0.109476366	0.109476366	0.003083937	C00025	29985
R-HSA-72766	Translation	0.018607124	0.112079601	0.112079601	0.011128989	C00025	29985
R-HSA-1614558	Degradation of cysteine and homocysteine	0.02020202	0.117267047	0.117267047	0.002949853	C00025	29985

R-HSA-112314	Neurotransmitter receptors and postsynaptic signal transmission	0.012759171	0.127566199	0.127566199	0.011128989	C00025	29985
R-HSA-8956320	Nucleotide biosynthesis	0.024986709	0.127566199	0.127566199	0.002949853	C00025	29985
R-HSA-69278	Cell Cycle, Mitotic	0.018607124	0.132678102	0.132678102	0.017296862	C00042	30031
R-HSA-499943	Interconversion of nucleotide di- and triphosphates	0.026581606	0.135224678	0.135224678	0.004424779	C00025	29985
R-HSA-1640170	Cell Cycle	0.019670388	0.13776502	0.13776502	0.023330652	C00042	30031
R-HSA-70326	Glucose metabolism	0.022328549	0.147864291	0.147864291	0.006436042	C00025	29985
R-HSA-8953854	Metabolism of RNA	0.023391813	0.147864291	0.147864291	0.012469831	C00042	30031
R-HSA-71406	Pyruvate metabolism and Citric Acid (TCA) cycle	0.023923445	0.15287684	0.15287684	0.004692947	C00042	30031
R-HSA-196807	Nicotinate metabolism	0.028176502	0.157864794	0.157864794	0.003888442	C00025	29985
R-HSA-382551	Transport of small molecules	0.116427432	0.16416091	0.16416091	0.054035935	C00042;C00025	30031;29985
R-HSA-390918	Peroxisomal lipid metabolism	0.032429559	0.165300819	0.165300819	0.004692947	C00042	30031
R-HSA-5579029	Metabolic disorders of biological oxidation enzymes	0.028176502	0.165300819	0.165300819	0.0050952	C00025	29985
R-HSA-8978868	Fatty acid metabolism	0.133971292	0.168366192	0.168366192	0.028828104	C00042;C00025	30031;29985
R-HSA-388396	GPCR downstream signalling	0.082402977	0.171538064	0.171538064	0.020380799	C00042;C00025	30031;29985
R-HSA-73894	DNA Repair	0.029239766	0.172682047	0.172682047	0.020783052	C00042	30031
R-HSA-420499	Class C/3 (Metabotropic glutamate/pheromone receptors)	0.03030303	0.175130335	0.175130335	9.39E-04	C00025	29985
R-HSA-372790	Signaling by GPCR	0.084529506	0.182216824	0.182216824	0.032046125	C00042;C00025	30031;29985
R-HSA-446219	Synthesis of substrates in N-glycan biosythesis	0.036150984	0.182439003	0.182439003	0.006570126	C00025	29985

R-HSA-425393	Transport of inorganic cations/anions and amino acids/oligopeptides	0.030834662	0.189693627	0.189693627	0.010056315	C00025	29985
R-HSA-9018677	Biosynthesis of DHA-derived SPMs	0.038277512	0.189693627	0.189693627	0.007776884	C00025	29985
R-HSA-1428517	The citric acid (TCA) cycle and respiratory electron transport	0.031897927	0.19209988	0.19209988	0.008447305	C00042	30031
R-HSA-1614635	Sulfur amino acid metabolism	0.034556087	0.19209988	0.19209988	0.004827031	C00025	29985
R-HSA-416476	G alpha (q) signalling events	0.034556087	0.194500176	0.194500176	0.00415661	C00025	29985
R-HSA-71291	Metabolism of amino acids and derivatives	0.151515152	0.205123281	0.205123281	0.037543577	C00042;C00025	30031;29985
R-HSA-446193	Biosynthesis of the N-glycan precursor (dolichol lipid-linked oligosaccharide, LLO) and transfer to a nascent protein	0.043062201	0.213489483	0.213489483	0.008447305	C00025	29985
R-HSA-3928662	EPHB-mediated forward signaling	0.005316321	0.218178057	0.218178057	0.002949853	C00025	29985
R-HSA-2682334	EPH-Ephrin signaling	0.005316321	0.218178057	0.218178057	0.0050952	C00025	29985
R-HSA-373076	Class A/1 (Rhodopsin-like receptors)	0.041998937	0.220513595	0.220513595	0.010458568	C00042	30031
R-HSA-425366	Transport of bile salts and organic acids, metal ions and amine compounds	0.040935673	0.222843316	0.222843316	0.008983642	C00042	30031
R-HSA-2262752	Cellular responses to stress	0.04040404	0.229797693	0.229797693	0.022660231	C00042	30031
R-HSA-422475	Axon guidance	0.009569378	0.236700133	0.236700133	0.017833199	C00025	29985
R-HSA-9675108	Nervous system development	0.009569378	0.236700133	0.236700133	0.017833199	C00025	29985

R-HSA-5619102	SLC transporter disorders	0.042530569	0.238989459	0.238989459	0.008313221	C00025	29985
R-HSA-8953897	Cellular responses to stimuli	0.041467305	0.24127306	0.24127306	0.026816841	C00042	30031
R-HSA-1474244	Extracellular matrix organization	0.014885699	0.272648759	0.272648759	0.005229284	C00042	30031
R-HSA-1266738	Developmental Biology	0.019138756	0.277041406	0.277041406	0.024805578	C00025	29985
R-HSA-2142753	Arachidonic acid metabolism	0.0579479	0.277041406	0.277041406	0.010324484	C00025	29985
R-HSA-3700989	Transcriptional Regulation by TP53	0.02020202	0.277041406	0.277041406	0.009519979	C00025	29985
R-HSA-5619115	Disorders of transmembrane transporters	0.051568315	0.281411923	0.281411923	0.010726736	C00025	29985
R-HSA-446203	Asparagine N-linked glycosylation	0.061669325	0.292241987	0.292241987	0.017699115	C00025	29985
R-HSA-212436	Generic Transcription Pathway	0.029239766	0.313494405	0.313494405	0.017430947	C00025	29985
R-HSA-195258	RHO GTPase Effectors	0.014885699	0.317680402	0.317680402	0.013140252	C00042	30031
R-HSA-194315	Signaling by Rho GTPases	0.014885699	0.317680402	0.317680402	0.025207831	C00042	30031
R-HSA-9716542	Signaling by Rho GTPases, Miro GTPases and RHOBTB3	0.014885699	0.317680402	0.317680402	0.026414588	C00042	30031
R-HSA-162582	Signal Transduction	0.129186603	0.318785753	0.318785753	0.174711719	C00042;C00025	30031;29985
R-HSA-73857	RNA Polymerase II Transcription	0.030834662	0.319765411	0.319765411	0.021319389	C00025	29985
R-HSA-9018678	Biosynthesis of specialized proresolving mediators (SPMs)	0.074428495	0.340325113	0.340325113	0.016626441	C00025	29985
R-HSA-196849	Metabolism of water-soluble vitamins and cofactors	0.069112174	0.342352281	0.342352281	0.019308126	C00025	29985

R-HSA-9662851	Anti-inflammatory response favouring Leishmania parasite infection	0.018607124	0.358383542	0.358383542	0.003486189	C00025	29985
R-HSA-9664433	Leishmania parasite growth and survival	0.018607124	0.358383542	0.358383542	0.003486189	C00025	29985
R-HSA-15869	Metabolism of nucleotides	0.082402977	0.374087813	0.374087813	0.018771789	C00025	29985
R-HSA-156580	Phase II - Conjugation of compounds	0.080808081	0.385654656	0.385654656	0.009654063	C00025	29985
R-HSA-9717207	Sensory perception of sweet, bitter, and umami (glutamate) taste	0.047315258	0.391370933	0.391370933	0.002815768	C00025	29985
R-HSA-9717189	Sensory perception of taste	0.049441786	0.398923629	0.398923629	0.003352105	C00025	29985
R-HSA-5668914	Diseases of metabolism	0.085061138	0.398923629	0.398923629	0.024671494	C00025	29985
R-HSA-9658195	Leishmania infection	0.03030303	0.400799549	0.400799549	0.008447305	C00025	29985
R-HSA-71387	Metabolism of carbohydrates	0.081871345	0.413794875	0.413794875	0.030705283	C00025	29985
R-HSA-597592	Post-translational protein modification	0.092503987	0.41929194	0.41929194	0.038079914	C00025	29985
R-HSA-196854	Metabolism of vitamins and cofactors	0.099415205	0.451380227	0.451380227	0.027353178	C00025	29985
R-HSA-392499	Metabolism of proteins	0.125465178	0.549360951	0.549360951	0.061410566	C00025	29985
R-HSA-5663205	Infectious disease	0.066985646	0.549360951	0.549360951	0.043979619	C00025	29985
R-HSA-9709957	Sensory Perception	0.101010101	0.589180544	0.589180544	0.018369536	C00025	29985
R-HSA-211859	Biological oxidations	0.173312068	0.653002062	0.653002062	0.025073746	C00025	29985
R-HSA-556833	Metabolism of lipids	0.337054758	0.718575764	0.718575764	0.11893269	C00042;C00025	30031;29985
R-HSA-1643685	Disease	0.18394471	0.796425481	0.796425481	0.116519174	C00025	29985
R-HSA-1430728	Metabolism	0.758639022	0.996306526	0.996306526	0.288549209	C00042;C00025	30031;29985

Supplementary table 12a: Reactome pathway analysis of downregulated metabolites in stromal regions of post-chemo versus pre-chemo PR tissues

PR= poor response; ER= excellent response

Name	Parent Folder	Percent Overlap	Overlapping Entities	p-value	Jaccard similarity
biosynthesis template	Plants	11	succinate;glutamate	9.3053E-06	0.095238095
precursor biosynthesis	anabolism	4	succinate;glutamate	5.23651E-05	0.044444444
Proline metabolism template	Aminoacid metabolism and interconvertion	4	succinate;glutamate	6.57453E-05	0.04
Glu/Gln metabolism template	Aminoacid metabolism and interconvertion	2	glutamate;succinate	0.000168773	0.025641026
Lysine biosynthesis template	Aminoacid biosynthesis	2	glutamate;succinate	0.000173334	0.025316456
metabolism template	metabolic cycles	2	glutamate;succinate	0.000197053	0.023809524
Lysine degradation template	Aminoacid degradation	2	glutamate;succinate	0.000212015	0.022988506
Lysine Metabolsm	Amino Acids Metabolism; Amino Acids Metabolism (Metabolic Pathway); generic; generic	2	glutamate;succinate	0.000222293	0.02247191
Urea cycle and Arginine metabolism template	Aminoacid metabolism and interconvertion	2	glutamate;succinate	0.000232815	0.021978022
Urea Cycle and Arginine Metabolism	Amino Acids Metabolism; Amino Acids Metabolism (Metabolic Pathway); generic; generic	2	glutamate;succinate	0.00024358	0.021505376
Glu/Gln/Pro Metabolism	Amino Acids Metabolism; Amino Acids Metabolism (Metabolic Pathway); generic; generic	2	glutamate;succinate	0.000249054	0.021276596
Glutathione Metabolism	Cofactors and Vitamins Metabolism; Cofactors and Vitamins Metabolism (Metabolic Pathway); generic; generic	2	glutamate;succinate	0.00026584	0.020618557

Branched Chain Amino Acids Metabolism	Amino Acids Metabolism; Amino Acids Metabolism (Metabolic Pathway); generic; generic	2	glutamate;succinate	0.000295033	0.019607843
Malonate, Propanoate and beta-Alanine Metabolism	Carboxylic Acids Metabolism; Carboxylic Acids Metabolism (Metabolic Pathway); generic; generic	2	glutamate;succinate	0.000295033	0.019607843
Methionine metabolism template	Aminoacid metabolism and interconvertion	1	glutamate;succinate	0.000332071	0.018518519
Glutamine in Cancer Metabolism	Hallmarks of Cancer (7): Deregulated Metabolism; Hallmarks of Cancer (7): Deregulated Metabolism (Hallmarks of Cancer); oncogenic; oncogenic	3	glutamate;succinate	0.000390594	0.031746032
mTOR Signaling Activation by Amino Acids	Fundamental Signalings Changed in Aging; Signal Transduction Pathways; Signal Transduction Pathways (Signal Processing); generic; generic	2	glutamate;succinate	0.000542692	0.023809524
Oxo (keto) fatty acids	Fatty Acyl & Other Lipids	0	5-oxo-pentanoic acid	0.000914748	0.003412969
Metabolic Reprogramming in Cancer: Overview	Hallmarks of Cancer (7): Deregulated Metabolism; Hallmarks of Cancer (7): Deregulated Metabolism (Hallmarks of Cancer); oncogenic; oncogenic	1	glutamate;succinate	0.001446523	0.016949153
mTOR Signaling Activation by Amino Acids	Fundamental Signalings Changed in Aging; Signal Transduction Pathways; Signal Transduction Pathways (Signal Processing); generic; generic	2	glutamate;succinate	0.001990038	0.023809524
Unsaturated hydroxy fatty acids	Fatty Acyl & Other Lipids	0	2-hydroxyvaleric acid	0.002085268	0.001508296

HMGB1 and IL1B in Neuronal Hyperexcitation	Epileptiform Disorders; Epileptiform Disorders (Neurological Diseases); nerve tissue; nervous system	6	glutamate	0.00244858	0.052631579
Cochlear Hair Cell Synapse Proteins Mutations (Age-Related/Congenital)	Hearing Loss; Hearing Loss (Otolaryngological Diseases); sensory system; synapse	5	glutamate	0.002775057	0.047619048
Rod Photoreceptor Signaling Impairment in Retinitis Pigmentosa	Retinitis Pigmentosa; Retinitis Pigmentosa (Ophthalmology/Eye Diseases); Retinitis Pigmentosa (Ophthalmology/Eye Diseases); retina; sensory system	5	glutamate	0.002938296	0.045454545
Prostaglandin E2 Synthesis Activation via Glutamate in Familial Hemiplegic Migraine	Migraine, Familial Hemiplegic; Migraine, Familial Hemiplegic (Neurological Diseases); nervous system	5	glutamate	0.003101534	0.043478261

Supplementary table 12b: Pathway studio pathway analysis of downregulated metabolites in stromal regions of post-chemo versus pre-chemo PR tissues

PR= poor response; ER= excellent response

Accession	Gene	PR-ER-1-2	PR-ER-2-2	PR-ER-3-3	PR-ER-4-1	PR-ER-6-1	PR-ER-6-3	PR-ER-8-1	PR-ER-8-3	PR-ER-9-3	PR-PR-1-2	PR-PR-2-1	PR-PR-3-1	PR-PR-3-3	PR-PR-5-2	PR-PR-6-3	PR-PR-7-1	PR-PR-8-1	PR-PR-8-3	PR-PR-10-3
A0A075B6	IGLV4-69	-2.16313	-0.66502	-1.24345	-3.78661	-0.24036	-2.52091	0.34597	-0.9148	0.467346	-2.38126	-1.57866	0.82387	-0.34504	1.867953	-0.95906	-0.82996	-0.31371	-0.31304	-0.95463
A0A0B4J1	IGHV6-1	-2.14831	-0.12855	0.320185	-3.05864	-1.54713	-2.39529	1.136239	0.060432	0.430717	-2.46255	-1.41702	0.209572	0.514827	0.473383	-1.52116	-1.60163	-0.98859	-0.79072	-3.00657
A0A0B4J1	IGHV3-15	-0.26051	0.442865	0.336082	-0.66667	-0.03833	-0.42358	0.336643	0.420942	0.127729	-1.38971	-0.87	0.463588	-0.06142	1.600186	-1.51464	-1.26235	0.046617	-0.77032	-0.94815
A0A0B4J1	IGHV3-74	-1.60606	0.141382	-0.32022	-1.95885	-1.07568	-1.40162	1.094698	-0.24482	1.788709	-0.80726	-1.16125	1.311303	0.004089	0.416332	-0.84143	-1.55575	-0.34073	-1.19449	-1.37615
A0A0C4DI	IGHV1-18	-1.85844	-0.09007	-1.21557	-3.03809	0.300065	-1.27535	0.353885	-1.28104	0.558227	-1.39964	-1.64527	-0.33873	-0.18095	0.915059	-0.83056	-1.02012	-0.32648	-0.23476	-1.56956
A0A0C4DI	IGHV1-24	-1.87887	-0.58015	-2.10429	-4.38978	0.040252	-2.28021	0.830178	-0.85604	1.583307	-3.90277	-1.5918	2.146061	2.946059	0.101546	-0.98058	-2.23888	-1.07681	-1.64425	-2.46584
A0A0C4DI	IGHV5-51	-2.18235	-0.2211	-2.56457	-1.92747	0.983557	-0.83738	-0.04138	-0.55954	0.992411	-1.48861	-1.67224	1.364334	0.345098	0.874917	-0.91172	-1.35547	0.217609	-0.03791	-0.72212
A0A0C4DI	IGHV2-70I	-2.51225	0.561627	-0.56596	-1.96642	-1.59874	-2.09057	0.898737	-0.98255	1.037999	-2.26836	0.850231	1.936141	2.228635	0.917361	-1.28963	-1.36158	-0.49681	-0.08874	-1.62524
A0AV96	RBM47	0.231316	-0.11543	0.287119	0.026507	-0.19002	0.187501	-0.66915	-0.19531	-0.11832	-0.15385	0.524042	0.606226	-0.64673	0.753395	-0.13902	0.321309	0.787893	0.268012	-0.95947
A0AVF1	TTC26	0.392523	-0.10597	-0.45268	0.396102	-0.51683	-0.14891	-0.09147	-0.11963	0.040847	-0.54727	1.457172	-0.88136	0.03197	-0.43107	-0.40864	-0.01484	-0.20873	-0.85223	0.022139
A0AVT1	UBA6	0.031746	0.40602	0.725004	-0.53992	0.035401	0.153235	-0.44589	0.132598	0.122386	0.17794	-0.11448	0.572487	0.456573	0.82991	-0.21059	0.373393	0.595061	-0.23404	-0.11819
A0FGR8	ESYT2	-0.80795	-0.02354	0.038002	1.346346	-0.18739	-0.15212	-0.01111	0.009745	0.339858	-0.00968	-0.3651	-0.0058	-0.32136	0.336031	1.066674	1.014854	0.289455	0.097019	0.022426
A0JNW5	UHRF1BP	-0.04598	0.5876	0.36632	0.079841	-0.14392	-0.05171	-0.05745	-0.51358	-0.43944	0.094788	-0.71939	0.245171	0.164416	0.715494	-0.42661	0.013613	0.029663	-0.3131	0.612503
A0M8Q6	IGLC7	-2.30474	-0.91862	-1.0703	-2.81099	0.276114	-2.00539	0.797403	0.148742	0.67769	-2.06501	-1.6942	1.134428	0.406668	0.777504	-1.82773	-1.96533	0.055955	-0.51886	-1.72234
A0MZ66	SHTN1	-0.16685	0.121262	0.699108	-0.00399	0.472999	0.496881	-0.46566	0.774308	0.467652	1.067065	0.369217	0.352665	0.659109	0.174745	0.672008	0.052561	0.397653	-0.00704	-0.48256
A0PJW6	TMEM223	0.074915	-0.16614	-0.01266	0.415894	-0.16362	0.036111	0.297207	-0.46772	-0.48714	-1.15244	0.226351	0.033571	-0.14845	-0.24775	0.489916	0.156232	-0.37948	0.025025	-0.70341
A1A4S6	ARHGAP1	-0.40133	-0.31936	0.353256	-0.11052	0.954508	0.735543	-0.33856	-0.51101	-0.55285	0.407152	-1.14341	-0.05068	-0.44941	0.028814	-0.61898	-0.99687	-0.35551	0.152536	-0.511
A1IGU5	ARHGEF3	-0.15736	0.181642	0.032292	-0.58714	0.470963	0.574943	-0.1285	0.19141	0.13864	0.092473	0.236858	-0.21142	0.249778	0.316812	-0.331	0.109295	-0.02673	-0.73115	-0.35723
A1L020	MEX3A	0.122809	0.094547	0.170754	-0.99018	-0.77504	-1.73884	0.123215	0.701834	1.031197	-0.9583	-1.39922	-0.08357	0.080977	-0.24369	0.04367	-0.07718	-0.31148	-1.01646	1.434293
A1L0T0	ILVBL	-0.6603	0.039695	0.711047	0.628939	-0.56816	-0.55935	0.977955	0.731308	-0.43806	-0.38581	-0.8856	0.298929	-0.89805	-0.01152	2.691823	1.219309	0.535562	-0.60139	-0.20047
A1L170	C1orf226	0.291316	-0.17014	-0.19268	-0.67677	-0.383	-0.13806	0.381505	1.033453	0.159939	-0.15593	-1.1028	-0.40862	-0.04646	-0.13788	0.63521	-0.34154	0.47221	-0.53256	0.917258
A1L390	PLEKHG3	-0.24229	-0.20881	-0.34716	0.498527	-0.2205	-0.886	-0.36435	0.775111	0.360815	0.184777	-0.21761	-0.96614	-0.12374	-0.3026	0.962152	-1.06016	0.429108	-0.26254	0.595399
A1L4H1	SSC5D	-1.22807	0.091424	0.408096	-1.10139	-0.36533	-0.46103	0.405565	-2.1678	0.377175	-1.12423	-1.05649	-0.67417	-0.45562	-0.33154	1.911993	-0.87395	-1.76379	-0.98784	0.10064
A1X283	SH3PXD2I	-0.05324	0.354616	-0.26432	-0.81252	-0.60391	-0.40135	-0.30509	-0.03603	0.088355	0.369331	-0.80095	-0.51665	0.837229	-0.57131	-0.77599	0.213199	-0.56526	0.477157	1.117953
A2RRP1	NBAS	-0.58884	0.327348	0.28488	-0.06414	-0.03387	-0.21957	0.342892	0.035416	0.08119	0.314343	0.040041	0.085647	-0.0164	-0.02789	0.139117	0.367905	-0.34526	-0.14496	0.594925
A2RTX5	TARSL2	-0.49082	-0.09804	0.078172	-0.56191	-0.63895	0.105563	-0.0308	-0.98625	0.300419	-0.02211	0.096128	-0.4423	-0.46925	-0.26062	0.18787	0.359366	-0.29382	0.325244	-0.73731
A2RUS2	DENND3	-0.73363	0.841325	0.818086	-0.86584	0.381441	0.351097	0.154217	-1.31205	-0.02424	1.297426	-0.60979	1.520298	0.036253	1.817823	-0.22427	-0.00449	0.487332	-0.02392	-0.61821
A3KMH1	VWA8	-0.53477	-0.04565	-0.78286	-0.48428	-0.4325	-0.41792	0.335671	0.233356	0.264625	0.575135	0.615726	-0.24446	-0.47986	-0.31456	1.540038	0.658117	0.576276	0.679397	-0.42623
A3KN83	SBNO1	0.257509	0.196065	-0.28007	-0.00038	-0.00831	-0.14024	0.571548	0.275405	0.027256	-0.14311	-0.06474	-0.43165	-0.43099	-0.14562	0.295693	-0.30281	0.410484	0.363248	0.152943
A4D1E9	GTPBP10	0.261925	-0.31899	-0.28758	0.469145	-0.10688	0.467427	-0.14636	-0.10631	-0.26306	-0.12195	0.264941	-0.66626	-0.15136	-0.39496	0.029905	0.066513	-0.68984	-1.16263	-0.4046
A4D1P6	WDR91	0.555514	0.121886	0.152331	-0.31573	0.054805	-0.40382	0.04826	-0.71207	-0.2827	0.183703	-0.21961	-0.10698	0.043466	0.035835	0.248883	0.373966	-0.93862	-0.2974	0.087327
A4D1T9	PRSS37	-0.43841	0.826227	0.737884	-0.27159	0.214373	-0.13867	1.53876	0.180253	0.040847	0.398129	-0.39014	0.300562	0.304511	0.150511	0.752653	0.152507	-0.4052	-0.78156	-0.179
A4D1U4	LCHN	0.025663	0.162635	0.053429	0.325798	0.281629	0.240501	0.226514	0.290707	-0.01616	-0.21692	-0.45367	0.609729	-0.16009	0.240204	0.24372	0.22405	0.396507	-0.46742	-0.08774
A5D8V6	VPS37C	-0.73834	0.740116	0.069873	-0.95152	-0.68359	-0.24771	0.130536	0.687326	-0.12921	0.10053	-0.24403	-0.047	0.079709	0.277564	-0.34386	0.707964	-0.00673	0.209742	-0.33214
A5PLN9	TRAPPC1	-0.81088	0.561407	0.331034	-0.1665	0.589747	0.312431	0.03327	-0.2284	0.216091	0.514949	0.120506	0.626703	-0.02336	0.788111	0.047198	0.011047	-0.07383	-0.29781	-0.36467
A5YKK6	CNOT1	0.181961	0.178771	0.039741	-0.15423	-0.07553	0.111453	0.336928	0.107	-0.14661	-0.09084	0.157462	-0.12174	-0.19171	-0.06546	0.217302	0.212073	0.183958	0.046064	0.32586
A6NC98	CCDC88B	-0.88379	1.258981	0.799192	-0.71446	0.208053	0.152197	0.182339	-1.54711	-0.10328	1.316363	-0.79835	0.746914	0.150406	1.847213	-0.64914	-0.57771	0.627427	0.000039	-1.19169
A6NDB9	PALM3	-1.0075	-0.3782	0.117167	0.393092	-2.4711	-2.18477	1.282466	3.309366	0.019914	1.302931	-1.39609	-0.65596	-0.61703	-0.38219	2.675789	-1.80237	0.261541	-1.10924	-1.01653
A6NDG6	PGP	0.233243	-0.02527	-0.29576	-0.15965	0.739624	-0.57868	0.32465	-0.42976	0.262961	0.045645	-0.85148	0.276539	-0.37407	0.005005	-0.61359	-0.14563	0.259703	0.534198	-0.99699
A6NDU8	C5orf51	-0.01596	0.154442	-0.40124	-0.72719	-0.4675	-0.31606	0.127158	-0.52948	-1.76516	0.032522	-0.01646	-0.34722	0.079039	0.406384	-0.44002	0.415656	0.06694	-0.34452	0.295372
A6NED2	RCCD1	0.139798	0.37281	0.024162	-0.4348	-0.03973	-0.18334	-0.20002	0.320729	-0.04557	0.015769	-0.35552	0.239968	-0.04445	0.13646	0.051471	0.085907	-0.7278	0.2217	0.393772
A6NHL2	TUBAL3	0.456427	0.183483	0.227439	-0.21671	0.292395	0.466338	0.033006	0.52356	-0.15723	-0.10085	-0.03488	-0.23689	0.154315	0.091	-0.23569	-0.20008	0.107607	-0.18541	0.745617
A6NHQ2	FBLL1	0.411277	-0.18376	-0.24122	0.186688	0.04082	-0.2789	-0.52665	0.0944	-0.19762	-0.24968	1.142975	0.085753	-0.07455	-0.27461	0.347435	0.003454	0.140456	-0.3397	-0.26493
A6NHR9	SMCHD1	0.430535	0.522132	0.523216	0.233623	0.495329	0.577557	0.213178	0.564093	-0.5263	-0.00765	-0.12534	-0.00237	-0.32408	0.66725	-0.17185	-0.468	0.311107	0.367257	-0.57357
A6NIH7	UNC119B	0.601633	-0.37396	0.01876	-0.11304	0.492942	-0.28502	-0.17586	0.808887	-0.12725	-0.00066	0.054462	-0.24298	-0.29376	-0.0982	-0.42602	-0.3318	-0.03473	-0.65596	-0.08127
A6NJ78	METTL15	-0.85741	0.072096	-0.3895	-0.6238	0.47627	-0.05618	-0.07833	-0.04494	0.00527	0.064467	0.460555	-0.27069	-0.19978	-0.15881	-0.1135	0.859916	-0.28302	-0.84903	-1.54515
A6NKD9	CCDC85C	0.786753	0.007192	-0.65918	-0.07554	0.244402	0.018669	0.294427	1.061657	-0.20054	-0.03297	-0.34293	-0.30229	-0.23672	-0.1458	0.140591	-0.06166	-0.13116	-0.17867	0.148375
A6NMZ7	COL6A6	-2.09938	0.436997	2.02299	-1.73564	2.758608	-1.14759	1.317269	-2.24443	0.412476	-0.16919	-2.91671	-1.07745	-0.41863	-1.4202	0.340227	1.766826	-0.48847	0.59267	-0.20784
A6ZKI3	RTL8C	0.515333	0.464649	-0.07549	-0.68639	-0.34518	-0.54255	-0.06026	-0.02585	0.603173	0.016676	-0.26845	-0.3368	0.095305	0.230812	-0.4142	0.081533	0.326989	0.33835	0.618732
A7E2V4	ZSWIM8	-0.10144	0.497119	0.086411	-0.59079	-0.10854	-0.13111	0.181252	0.056007	0.166116	0.005284	-0.22303	-0.27979	-0.01827	0.5376	0.457623	0.086273	-0.26949	-0.33633	0.470227
A7KAX9	ARHGAP3	-0.16022	-0.19127	0.119954	0.035638	-1.01509	-1.44285	-0.15954	-0.34628	0.064989	-0.56728	0.200601	-0.13913	0.034668	-0.29107	0.061155	-0.48864	0.009008	0.454835	0.543377
A8K2U0	A2ML1	-1.19396	0.835486	-0.00577	-1.15302	-1.96505	-1.48959	0.797574	-1.33077	0.390808	-1.12837	-0.75984	-1.35132	-0.28449	-0.37705	-1.40289	-0.3182	-0.27369	0.299178	-1.0644
A8MVW0	FAM171A2	-0.68754	-0.05763	0.15321	-0.51529	-1.21753	-1.13281	0.177554	-0.11679	-0.06947	-0.37792	-0.69655	-1.30013	-0.0325	-0.55592	0.403055	-0.22416	-0.48462	-0.45641	0.890325
A8MW92	PHF20L1	1.383739	-0.11538	-0.0938	-0.23414	-0.07785	0.18286	0.087065	-0.10754	0.312198	-0.29028	-0.67357	0.426988	0.062443	-0.39425	1.485032	-0.17354	-0.54194	-0.40268	-0.78336
A8MXV4	NUDT19	0.299392	-0.3075	-0.06653	0.486671	0.768518	0.789964	-0.49718	0.251896	-0.42114	-0.4606	0.576915	-0.10002	-0.46293	0.250697	1.032828	0.752102	0.796674	0.174288	-0.64263
A9UHW6	MIF4GD	-0.20333	0.311264	0.105465	-0.60037	-0.48348	-0.39074	-0.32714	-0.75583	-0.034	0.035368	0.190874	-0.67258	-0.00813	-0.39649	-0.58771	-0.34217	-0.6493	-0.65134	-0.47075
B0I1T2	MYO1G	-1.62879	1.388719	1.775457	-0.77536	0.190007	-0.09788	0.897185	-1.20429	0.071701	1.379913	-1.6722	1.235025	-0.19328	1.599247	-0.97214	-0.81082	0.307293	0.697854	-1.07325
B2RTY4	MYO9A	0.202796	0.027508	0.219862	-0.30565	0.428891	0.523691	-0.23128	-0.07911	0.504449	0.031226	-0.50978	-0.27464	0.104987	-0.0274	0.147429	0.194648	0.592451	0.604915	0.100273
B7ZAP0	RABGAP1	-0.22395	-0.01635	0.398214	-0.26408	-0.51789	-0.77936	0.047783	-0.3865	-1.24567	-0.47782	-0.02207	-0.84295	-0.69284	-0.6138	0.599267	-0.32503	-0.53878	0.352681	0.14709
B7ZAQ6	GPR89A	0.385043	0.357368	0.085786	0.008661	-0.38278	-0.28256	0.5376	0.508473	-0.15783	-0.22359	0.161408	0.016909	-0.12671	-0.54383	0.558447	0.215913	0.140756	-0.09521	0.010059
B9A064	IGLL5	-2.29182	-0.62843	-1.08475	-2.35698	-0.22788	-1.89886	0.521618	-0.27353	0.391876	-1.72123	-1.76574	0.962174	0.060242	1.07888	-1.7146	-1.43029	0.052711	-0.72524	-1.8623
C9JLW8	MCRIP1	-0.1369	0.111674	0.118026	-0.24815	0.256862	0.324504	-0.0305	-0.26226	0.029408	-0.15651	-0.28992	0.031571	0.156983	-0.66008	-0.94327	0.265588	-0.05327	-0.50661	-0.09545
H7BZ55	CROCC2	-1.65998	-0.26624	-0.65828	0.237431	-2.063	-1.55105	-0.88118	-1.54478	-0.40647	-0.59905	3.471403	-1.34339	-0.81243	-0.52087	-0.98453	-0.21605	-0.50357	-1.05774	-0.91041
K7EJ46	SMIM22	0.649003	-0.17837	-0.51294	0.144562	-0.5677	-0.46867	-0.32217	0.116342	-0.0608	-0.36092	0.64615	-0.53039	-0.13187	-0.38782	-0.30863	-0.07242	-0.32713	-0.33422	-0.33631
L0R6Q1	SLC35A4	-0.18225	0.799096	0.460814	0.108684	0.542804	0.350962	-0.21077	0.819868	-0.39019	-0.06563	0.10486	-0.58847	-0.50734	-0.31536	0.257382	1.257643	-0.64143	-0.14677	-0.71037
L0R819	ASDURF	0.274726	-0.31783	0.388145	-0.40003	-0.53041	-0.43872	-0.45458	1.263111	-0.33495	-0.09925	-0.44882	-0.83513	0.064009	-0.42959	0.506056	-0.23573	-0.91643	-0.55162	0.029274
O00116	AGPS	0.000641	0.633712	0.005047	0.627678	-0.01424	0.159022	0.416197	0.078609	0.349112	0.501432	0.079541	0.893036	0.06563	0.608686	-0.30996	-0.19148	0.422959	-0.28552	-0.34298
O00139	KIF2A	0.879446	0.122962	0.131925	-0.18773	0.194252	0.492587	-0.12509	-0.02416	-0.28986	-0.19234	-0.03887	-0.21303	0.198275	-0.25338	0.401928	-0.61614	0.288625	0.517896	0.16024
O00142	TK2	-1.2473	0.501795	0.171374	-0.05758	-0.32888	-0.23674	0.134041	-0.76558	0.876745	-0.39985	0.204303	0.085489	0.123739	0.124863	-0.02138	-0.36884	-0.37873	-0.03517	0.137199
O00148	DDX39A	0.093824	-0.03809	0.865191	-0.21193	-0.17098	-0.31342	0.054323	0.829255	0.16498	0.147081	-0.46375	0.162515	-0.53275	0.207413	0.00813	-0.18836	0.273789	-0.06494	0.029406
O00151	PDLIM1	-0.18234	-0.02388	0.625338	-0.60649	-1.00012	-0.29822	-0.39549	1.661802	0.810248	0.892334	0.157671	-0.97736	0.677958	-1.20816	-0.81588	-0.71266	-0.7655	-0.08456	1.498027
O00154	ACOT7	-0.36524	-0.75341	0.379757	-0.01755	0.379936	0.705596	-0.58993	0.309192	1.18743	0.371307	0.232731	-0.18535	0.412085	0.077961	-0.38724	-0.1417	1.395182	0.21613	-0.11096
O00159	MYO1C	-0.28445	-0.13183	-0.13123	-0.77965	0.226977	-0.27964	0.428374	-0.92266	-0.09656	-0.57724	-0.41834	-0.37297	-0.36087	-0.90768	0.058387	-0.49907	-0.69213	0.17285	-0.48639
O00160	MYO1F	-1.63449	1.481151	1.009583	-0.83986	0.315674	0.134977	0.25026	-1.56316	0.00349	1.197134	-1.67373	1.552344	-0.07572	2.203832	-0.67063	-0.60065	0.74817	-0.41176	-0.9551
O00161	SNAP23	-0.40393	0.457463	0.26876	0.367328	-0.2653	-0.55975	0.337977	-1.10729	0.223597	0.543049	0.051549	0.146968	0.132151	0.441758	0.199327	0.016368	0.107944	0.614057	-0.15187
O00165	HAX1	-0.3702	0.627421	-0.32775	0.025628	-0.05381	0.088607	-0.26157	-0.3461	-0.0567	0.702077	0.807265	-0.5221	-0.35225	-0.59815	0.206781	0.597235	-0.52602	-0.05974	-0.81453
O00170	AIP	0.28785	-0.68301	0.35458	0.481048	0.452094	0.471302	-0.70776	0.467343	-0.08827	1.205296	0.104531	-0.1488	0.101874	-0.00369	0.296602	0.283885	0.241954	0.756653	0.180053
O00178	GTPBP1	-0.31994	0.023887	0.19208	0.061447	0.068017	-0.00094	-0.34594	-0.32133	-0.30447	-0.3423	0.135572	0.083264	-0.32804	0.133812	-0.5459	0.024771	0.564032	0.131229	-0.17638
O00182	LGALS9	-0.25258	1.771569	1.110399	-0.25721	0.075464	-0.17575	0.497807	-0.51432	-0.27364	1.446437	-0.74421	1.212529	0.230208	1.548716	0.437597	-1.34614	0.070107	0.563993	-1.64745
O00186	STXBP3	0.052055	0.177398	-0.30604	0.242376	-0.11623	-0.20248	-0.519	-0.08911	-0.66089	-0.0754	0.466628	0.129755	-0.34772	0.207515	-0.21619	-0.52605	-0.32759	-0.4846	-0.62433
O00189	AP4M1	-0.09535	0.22016	0.159383	-0.26792	-0.05873	0.173051	0.143013	-0.22315	0.038346	0.509201	0.254419	0.332504	0.115261	0.538051	-0.17817	0.195013	0.511031	0.318795	0.011551
O00192	ARVCF	2.100318	-1.27502	0.099427	-0.48332	-1.4905	-1.13389	-0.20616	0.124466	-0.4272	-0.39995	-0.60177	-0.79745	-1.31895	-0.1137	0.657268	-0.26881	-1.08638	-0.28558	-0.77054
O00193	SMAP	-0.29404	-0.56555	0.033392	-0.78521	-1.24927	0.012324	-1.45788	0.089707	-0.26514	-0.49543	0.346177	-1.39325	0.235418	-1.10428	0.931176	0.567637	-0.49838	0.185789	-0.40136
O00203	AP3B1	-0.63555	0.467463	0.037504	-0.39756	-0.05768	-0.07808	0.112515	-0.12473	0.117031	-0.24298	0.196888	0.279874	0.09213	0.264818	0.137984	-0.04665	-0.06395	-0.04682	0.469591
O00213	APBB1	-0.42179	0.297787	-0.33066	-0.75651	0.638484	0.626705	-0.04971	-0.34521	0.024276	0.029891	-0.1307	-1.16357	-0.36795	0.536704	-0.47959	-1.24702	0.359928	0.127273	0.331264
O00214	LGALS8	0.455023	-0.11459	0.50068	-0.29861	-0.49131	-0.31326	0.11557	-0.2209	-0.0535	1.224261	0.684531	0.214119	-0.26455	0.483391	-0.56314	-0.13782	0.034577	-0.05208	-0.45205
O00217	NDUFS8	-0.01527	-0.15181	0.274279	-0.25904	0.200751	0.125438	0.4721	-0.70974	-0.58389	-0.71361	0.517654	-0.01891	-0.15808	-0.07851	1.250475	0.029899	-0.67916	-0.4608	-0.72144
O00221	NFKBIE	0.075893	-0.04611	0.141271	-0.52803	-0.35326	0.420534	-0.01979	-0.49852	0.040847	0.359828	0.512476	0.441088	0.268808	0.972853	0.060188	0.140139	0.71456	0.622953	-0.26589
O00231	PSMD11	-0.16597	0.291961	0.317799	0.047725	-0.24039	0.119424	0.114517	-0.01213	-0.22815	0.113132	0.07667	0.212117	0.003351	0.262595	-0.03609	0.427753	0.256902	-0.15862	0.219813
O00232	PSMD12	-0.02492	0.152212	0.173301	0.157932	-0.15907	0.092824	0.195719	0.081329	0.04426	-0.00426	0.019191	0.245214	-0.11629	0.304207	-0.00514	0.421047	0.300756	-0.29921	0.29241
O00233	PSMD9	-0.31588	0.065194	-0.04043	-0.61401	0.948269	-0.48799	-0.28895	0.172589	-0.09426	-0.41407	-0.01174	0.04057	0.047359	0.088008	-1.1106	-0.05999	0.264647	-0.13154	-0.39054
O00244	ATOX1	-0.79409	0.019836	-0.18679	-0.16558	0.456927	-0.07173	0.367965	0.161661	0.140997	-0.09038	-0.41076	-1.14287	0.049951	-0.34965	-0.61978	0.244725	-0.2242	0.588734	-0.03504
O00255	MEN1	0.924622	0.440748	-0.00087	-0.0738	0.203439	0.317996	-0.04044	0.42787	-0.41604	0.260635	-0.12175	-0.1063	-0.65707	-0.11745	-0.06569	-0.24182	-0.23118	0.276293	0.257248
O00258	WRB	-0.74202	0.029115	-0.01542	0.1241	-0.22032	0.386599	-0.07646	0.063173	-0.24932	0.071249	0.25844	-0.38346	-0.00307	-0.33614	0.133857	0.260289	0.002254	-0.01007	-0.3695
O00264	PGRMC1	0.75471	-0.38686	-0.1425	0.52414	0.500297	0.817596	0.358663	0.214906	-0.27096	-0.63138	0.321081	-0.75719	-0.46468	-0.9409	-0.50719	-0.56055	-0.4332	-0.75175	-0.23845
O00267	SUPT5H	0.275662	-0.17083	0.312471	0.397141	0.59558	0.990406	0.233125	2.62157	-0.19219	-0.27493	0.247456	-0.13769	-0.02775	-0.18057	0.776416	-0.13509	-0.31695	-0.223	0.98913
O00268	TAF4	0.32609	-0.15367	0.111219	-0.04483	0.157856	0.530753	-0.28225	0.939176	-0.09274	-0.11562	0.379264	-0.23404	-0.33669	-0.56326	0.510224	-0.55824	-0.16669	0.335732	-0.15294
O00273	DFFA	0.73399	-0.67573	-0.17923	0.123245	-0.14917	-0.03261	0.000203	0.367006	0.935579	0.374313	0.642863	-0.92337	-0.33284	-0.3847	-0.1661	0.942306	0.297854	0.154457	-0.21525
O00291	HIP1	0.060813	-0.05991	0.101355	-0.53937	0.233791	0.827694	0.765433	1.112432	-0.32124	-0.11751	-0.16355	-0.88535	-0.20959	-0.4975	0.363113	-0.47103	-0.89996	-0.66989	-0.15601
O00299	CLIC1	-0.33109	0.181876	-0.23392	-0.45975	0.787436	0.335568	-0.54523	-0.04538	0.029371	0.774949	-0.52518	0.717841	0.66146	0.755396	-0.42069	0.024034	1.891045	0.694847	-0.32295
O00303	EIF3F	-0.15372	-0.12561	0.423441	0.075826	0.266816	0.618183	0.00771	0.291647	0.441517	0.255415	0.256731	-0.40633	0.023182	-0.21149	0.545326	0.418293	-0.08095	-0.29015	-0.01312
O00308	WWP2	0.098446	0.284246	0.242287	-0.52209	0.330935	0.328672	-0.14404	-0.39298	0.279136	0.105274	-0.06426	-0.44025	0.307146	0.196248	0.735418	-0.05701	0.433145	0.708825	-0.48159
O00329	PIK3CD	-1.16732	0.979857	0.711668	-0.27144	0.85331	0.176251	0.226952	-0.96328	0.069522	1.12148	-1.1044	0.898727	0.404876	1.292081	-0.46162	0.319547	0.066222	0.730817	-0.08436
O00330	PDHX	0.539696	-0.21457	-0.01435	-0.08235	0.026959	-0.25099	-0.00831	-0.04894	-0.37264	0.002784	0.373065	0.119389	-0.21706	-0.17298	0.878741	0.869658	-0.03599	-0.19154	-0.93536
O00339	MATN2	-0.75475	-0.91977	-2.14014	-1.41714	-0.79212	-1.39257	-0.21703	0.364414	-0.3036	-0.99116	-0.18053	-1.25993	-1.44234	-0.77225	0.193314	-0.81736	-0.23414	2.31961	-0.80505
O00391	QSOX1	-0.64388	0.210309	-0.33099	0.852309	-0.62086	-0.88916	-0.42616	0.331465	0.742887	-0.84206	-0.25804	0.515854	-0.38318	0.607869	-0.21868	-1.15021	0.29752	-1.37402	-0.1194
O00400	SLC33A1	-0.20594	0.167377	-0.31011	0.72522	0.804011	0.380368	0.92772	-0.68335	0.077063	-0.47299	0.212679	0.28298	0.159252	-0.12906	1.294321	0.061657	0.507621	0.156052	0.087842
O00401	WASL	-0.25099	-1.34127	-1.19859	-0.22924	-0.69673	-0.14305	-1.59152	-0.21175	-0.27404	0.153676	0.229163	-0.80269	-0.03065	-1.93072	0.304211	1.288007	-0.88915	-0.26645	0.143266
O00410	IPO5	0.126279	0.005507	-0.06255	-0.30169	0.334988	0.885699	0.551646	0.563893	-0.3064	-0.30575	0.10981	-0.47986	0.096992	-0.5586	-0.27247	-0.16353	-0.15408	-0.14483	0.594459
O00411	POLRMT	-0.1893	-0.21785	0.424885	0.013741	0.417801	0.270282	0.078835	-0.7969	1.155668	0.084582	0.074493	0.338906	1.108545	-0.08251	0.623576	0.266275	-0.35102	-0.19466	-1.01181
O00418	EEF2K	0.408197	-0.44336	0.202993	-0.39067	0.096902	0.165933	-1.16126	-0.78763	0.054161	0.204848	1.367549	-0.77623	0.269295	-0.65867	0.854253	-0.12377	-0.53374	-0.02082	-0.68362
O00422	SAP18	0.991646	-0.50564	0.12135	0.411831	0.114406	0.422167	-0.48673	0.740027	-0.49918	0.019003	0.569621	0.359405	-0.20247	0.122837	0.461393	0.113525	0.405939	0.0909	-0.5057
O00423	EML1	-0.17808	-0.45212	-0.59641	-0.0904	-0.21084	-0.20403	0.043367	-0.09073	0.433088	-1.10827	-0.37974	-1.69332	-0.39839	-1.10929	-0.46017	-0.65915	-0.60515	0.128334	-0.05877
O00425	IGF2BP3	3.748622	-0.3654	2.122774	1.961116	-2.1282	-0.9591	-0.40961	0.956203	0.507527	1.144007	-2.93862	-2.70759	-1.76945	-0.69736	-1.81859	-3.00786	2.274945	0.978251	0.234763
O00429	DNM1L	-0.07443	0.169885	-0.03597	0.369913	-0.03393	-0.10221	-0.09185	0.342089	-0.42748	-0.29476	-0.19695	-0.1541	-0.02645	-0.25521	0.173138	0.378086	-0.02557	-0.09269	-0.04302
O00442	RTCA	-0.41072	-0.21767	0.434053	0.259631	0.229289	-0.20635	-0.05947	-0.14163	0.136475	0.479966	-0.02262	0.512787	-0.23644	0.589489	0.714785	-0.17875	0.65976	0.17051	-0.12861
O00443	PIK3C2A	-0.36565	0.180868	0.371296	-0.35027	0.294853	0.299474	0.796621	0.252024	0.20765	0.007702	-0.28362	-0.26701	0.020796	0.097257	0.416042	0.085156	0.315166	0.430297	-0.01589
O00458	IFRD1	0.186475	0.300486	0.199585	-1.83378	-0.75921	-0.67427	0.484055	-0.21423	1.013379	-0.71652	-0.56199	0.880479	0.155181	-0.21306	1.388741	-0.48201	-0.66737	-1.03595	-0.80692
O00459	PIK3R2	-0.22895	0.021264	0.14225	-0.03308	-0.36488	-0.78077	0.168822	-0.63053	0.086974	0.214794	-0.52817	-0.04049	0.08018	-0.05153	0.48159	-0.25801	-0.17259	-0.02233	-0.09514
O00461	GOLIM4	-0.27745	0.449884	0.144999	-0.81352	-0.26961	-0.09981	0.599963	0.254873	0.234085	0.096523	-0.42154	-0.91273	0.282383	-0.31217	0.626168	-0.26408	-0.23577	0.977932	1.102876
O00462	MANBA	0.452979	-0.41	0.282798	-0.75184	1.321434	-0.10527	-0.21432	0.099645	0.323554	1.164703	-0.49586	0.773554	0.446545	0.680277	-0.15348	-0.45355	0.085639	0.838162	0.695751
O00463	TRAF5	-0.67194	0.179317	0.145457	-0.42368	0.200117	-0.19892	0.112039	-0.63197	0.336548	0.293998	-0.07173	-0.12835	-0.22753	0.105327	-0.03636	0.481203	0.246879	-0.10488	-0.17877

O00468	AGRN	0.220532	-0.36123	-0.27855	-0.51832	0.368075	-0.41211	0.157199	0.523411	0.010892	1.384586	-0.38409	-0.92514	-0.45535	-0.48962	1.124179	-0.76152	0.728458	2.171327	0.15309
O00469	PLOD2	0.433195	0.001974	0.00289	0.466787	0.365553	1.041879	-0.63305	1.140159	1.442549	0.326868	-0.80126	0.4714	2.657712	0.189322	-1.81664	0.79202	1.237977	-0.11214	3.230044
O00470	MEIS1	0.781298	-1.10386	-0.001	0.346373	-0.50449	-0.424	-0.02926	0.401685	-0.06961	-0.94754	0.663232	-1.28726	-1.5257	-0.96587	0.139012	-0.33786	-1.33282	-1.10742	-0.31736
O00471	EXOC5	-0.18145	0.289522	0.18049	0.042303	-0.06771	-0.02111	0.365703	-0.08801	0.060175	-0.17403	0.222177	0.167399	-0.00614	0.4034	0.211018	-0.00465	-0.24593	-0.11053	0.317576
O00478	BTN3A3	0.169102	0.938996	0.462658	-0.28183	-0.85913	-0.66837	0.652801	-0.63017	0.517636	-0.04504	-0.33933	-0.1972	-0.31798	-0.23873	0.249909	-0.111	-0.49041	1.042951	-0.36484
O00479	HMGN4	-0.43862	-0.67289	-0.0924	-0.9176	-4.47524	-1.05946	-1.12035	-2.15764	-1.38883	-1.04934	-0.08208	-2.31453	-0.20741	-1.69165	0.292116	-0.04169	-2.31186	-0.66166	-0.17935
O00481	BTN3A1	0.331374	0.051811	0.003829	-0.10452	-0.20084	0.154186	-0.09052	-0.83077	-0.28658	-0.17759	0.479589	-0.59324	-0.00081	0.053179	0.059322	0.19653	-1.0659	0.263646	-0.66609
O00483	NDUFA4	0.285052	-0.7944	0.279898	-0.09605	1.177695	0.894008	-0.19098	-0.34396	-0.02764	-1.10481	0.734676	-0.82568	-0.65535	-0.65459	2.058777	1.276529	-1.50707	-0.16267	-0.57618
O00487	PSMD14	-0.02415	-0.08106	-0.01787	0.234581	-0.13795	0.018766	0.100294	-0.01213	-0.07146	0.226345	0.096803	0.289786	0.446057	0.197802	-0.20357	0.31471	0.370577	-0.12431	0.229809
O00499	BIN1	0.271762	-0.90165	-0.1219	-0.68561	-0.3039	-0.19811	0.626014	-0.05857	-0.01832	-0.07085	0.110536	-0.85857	-0.97356	-0.21637	1.024897	0.062132	-0.06605	0.478711	-0.69751
O00505	KPNA3	0.177718	0.25719	-0.05464	-0.39155	0.108097	0.181861	-0.19685	0.281549	0.49642	0.824226	0.326452	0.004472	-0.18822	-0.30555	0.029579	-0.23076	0.002477	0.026073	0.586204
O00506	STK25	-0.03131	0.361417	-0.1079	-0.9729	-0.30465	0.053515	0.148418	-0.24619	0.307736	-0.03387	-0.47705	-0.56903	0.159841	0.169605	-0.06326	-0.17218	0.287168	0.080527	0.731434
O00512	BCL9	0.181658	-0.32569	-0.23008	-0.81896	-0.91437	-0.99913	-0.2183	0.012764	-0.0783	-0.00509	-0.30898	-0.12882	-0.07112	-0.62824	0.592633	0.124016	-0.26732	-0.74573	-0.10154
O00515	LAD1	-0.61993	-0.65414	-0.6623	-0.55918	-2.96656	-2.5782	-0.66452	0.633364	0.422645	0.817326	0.047244	-1.118	-0.0218	-1.32709	-0.87212	-1.47784	-0.33074	-1.52719	-1.9203
O00519	FAAH	-0.31027	-0.17465	-0.46892	0.724857	-0.46683	0.997514	-0.30402	-0.39519	-0.47071	-0.24579	1.263334	-0.16354	0.777759	0.119337	-0.34389	0.388413	-0.6141	-0.82371	-2.2047
O00522	KRIT1	0.118892	0.075584	-0.35619	-0.34214	0.225691	0.266906	0.187337	0.118187	-0.05478	-0.13205	0.036876	0.110683	-0.15166	0.265442	-0.04328	0.044881	0.221407	0.138383	0.314502
O00533	CHL1	0.162951	-0.34035	-0.3631	-1.32532	-1.36996	-2.22505	0.362755	-2.00964	0.007308	-1.54406	2.470519	-0.58685	-1.01446	-0.86296	-1.37018	-1.39245	-0.76173	-1.49533	-1.23978
O00534	VWA5A	0.045631	-0.56414	-0.38151	-0.06008	-0.81597	-0.96802	0.155124	-1.83777	-0.36222	-0.54878	1.293648	-0.4951	-0.09238	-0.11698	-0.67953	-0.31484	-0.71303	-0.44361	-0.25449
O00541	PES1	0.24416	-0.19336	-0.32446	1.034746	0.002041	0.141548	-0.32064	-0.02067	-1.1196	-0.11106	0.578452	-0.44421	-0.47013	-0.32152	-0.35367	0.168682	0.587827	0.539024	-0.36754
O00559	EBAG9	0.177748	1.166053	0.443459	-0.38458	-0.55748	-0.05885	1.126301	-0.80793	0.445112	-0.03725	0.011832	-0.52683	1.844103	-0.04235	0.77777	0.473148	-0.33239	0.194028	-0.20193
O00560	SDCBP	-0.83605	0.447783	-0.35824	-1.32083	-0.22901	-0.22809	0.074741	-0.68189	-0.98653	0.085038	-0.48801	0.163289	-0.13182	0.835352	-0.19886	-1.34151	0.298287	-0.43777	-0.38503
O00562	PITPNM1	-0.37605	0.065191	0.092736	-0.509	-0.29585	-0.29499	0.295978	-0.23008	0.065782	-0.1853	0.378242	-0.04483	-0.29644	0.346237	0.314531	-0.02723	0.309174	-0.03149	-0.17434
O00566	MPHOSPH	0.50594	-0.32825	-0.31499	0.738375	-0.45011	-0.13295	-0.31168	0.605096	-0.56046	0.317247	0.828664	-0.9443	-0.00065	-0.85456	0.079894	0.08822	-0.48439	-0.16917	-0.05706
O00567	NOP56	0.448824	-0.57236	-0.6076	-0.07253	-0.21642	-0.2821	-0.48027	-0.35642	-0.91128	-0.7447	1.288933	-0.20536	-0.60185	-0.30969	0.489421	-0.86095	-0.26766	-0.26266	-0.51916
O00571	DDX3X	-0.16968	0.372003	0.377478	0.06094	-0.11654	0.098316	-0.10464	0.125386	-0.16978	0.058478	-0.00373	-0.00192	0.074166	0.245706	-0.33021	0.319997	0.69744	0.662278	0.44689
O00584	RNASET2	-0.50376	0.589478	-0.76438	-0.71633	0.008147	-0.73471	-0.21186	-0.88286	1.08024	-0.12287	1.3406	1.639925	-0.2837	0.653321	0.173595	-1.21913	0.501596	0.859889	-0.73643
O00592	PODXL	1.429904	-0.70382	-1.10128	-0.83465	-0.64685	-0.77765	1.520501	3.49834	-2.01159	-0.84412	0.441447	0.418393	0.431386	-1.43207	0.126552	-1.07984	-2.33306	-2.01554	-0.65168
O00602	FCN1	-2.051	0.939165	1.690415	-1.02533	-0.17377	-2.69061	-0.89713	-1.40077	-0.03879	3.021021	-1.9107	2.574293	-0.16079	3.489997	-2.14153	-1.86363	1.931442	-0.14602	-2.08912
O00629	KPNA4	-0.03515	0.012544	0.407952	0.203271	0.916252	0.991547	-0.02667	0.027196	-0.28916	0.217765	0.054223	-0.37502	-0.01896	-0.23808	0.101442	-0.43115	-0.1096	-0.51349	-0.12061
O00635	TRIM38	-0.25332	0.471115	0.36936	-0.12638	-0.00028	0.441304	0.185845	-0.4505	0.297144	0.397899	0.181126	0.361037	-0.25029	0.381667	-0.13498	0.085604	-0.17424	-0.39567	-0.15691
O00712	NFIB	0.649699	0.134985	0.289609	-0.33968	0.139241	0.174286	-0.04311	0.341634	-0.33109	-1.24709	-0.28131	-1.0589	-1.41346	-1.22538	1.331938	-0.2199	-1.58565	-0.62331	0.097567
O00743	PPP6C	0.54456	-0.66154	0.920217	0.235139	0.642861	0.669463	0.057828	-0.02068	-1.47653	0.295875	-0.20163	0.553705	0.687198	1.188009	-0.0628	0.545669	0.822927	-0.31293	0.205575
O00748	CES2	0.111167	-0.25251	-0.6182	-0.55146	-1.31195	-0.39522	-0.4377	-1.30449	-0.21543	-0.20978	-0.18525	-0.33302	-0.18709	0.029604	3.338152	0.163034	0.18151	-1.30929	-1.12043
O00750	PIK3C2B	-0.94298	0.34005	0.247667	-0.24967	-0.2939	-0.54492	0.459908	0.054839	0.305273	0.671052	-1.01962	0.184479	0.138212	0.179346	-0.30182	-0.41372	0.189672	-1.02042	-0.82386
O00754	MAN2B1	0.013532	-0.4885	0.55726	-0.17984	0.956175	0.358217	0.279445	0.438264	0.491015	1.299588	-0.84489	0.527301	0.315914	0.349544	-0.42122	-0.19895	0.109861	0.978481	-0.13736
O00755	WNT7A	-1.52959	1.136769	-0.47214	0.126064	-2.09834	-1.78956	0.636431	0.578381	0.63473	-3.73907	-0.54445	-1.16697	-0.35969	-0.72425	0.202055	-1.73801	0.715946	-0.50464	0.018591
O00762	UBE2C	0.522996	-0.92791	0.422638	-0.0528	0.764048	0.923227	0.846478	0.982903	-0.38749	-0.39899	-1.28121	-1.48363	0.326501	-1.48732	-1.64818	-0.34962	0.747908	0.02665	0.549429
O00763	ACACB	-1.76301	-0.50177	-0.44807	-0.45348	-2.15227	-1.63049	0.434069	-2.10135	0.288798	-0.71027	-2.06273	-0.96203	0.045607	-0.77014	-0.40491	-0.69632	-1.28433	0.292409	-0.46877
O00764	PDXK	0.816061	-1.43829	-0.96628	-0.22053	1.201053	-0.26816	-0.64403	-0.65901	1.193243	0.22916	-1.10908	0.755599	-0.16543	1.125773	-0.16063	-0.05662	1.49351	0.502509	0.028431
O00767	SCD	-1.24176	-0.48452	-1.0139	0.026956	1.792813	2.625068	-1.25006	-0.592	0.231706	0.313893	-1.68506	0.749262	0.563295	0.045209	-0.16936	0.010441	1.24739	-0.76224	-0.5609
O14493	CLDN4	1.400908	0.478443	-0.10398	-0.22586	-0.36148	-0.0181	0.069436	0.459784	-0.24416	-0.54353	0.411783	-0.10241	-0.02183	-0.26366	0.486175	0.00077	-0.76444	-0.0215	-0.2938
O14495	PLPP3	-0.02872	0.059148	0.139116	-0.22195	-0.5327	-0.66196	-0.01084	0.096017	0.510451	-0.28383	-0.8541	-0.41809	0.011729	-0.04153	0.061666	0.212852	-0.09954	-0.35959	-0.68889
O14497	ARID1A	0.734713	-0.04205	-0.01613	-0.59138	-0.00254	-0.13517	0.10227	0.560312	-0.57201	-0.07708	0.009891	-0.27987	-0.60824	-0.25897	0.450032	-0.57424	-0.33752	-0.28947	-0.2446
O14498	ISLR	-1.06222	-0.66582	-1.32562	-1.40989	-0.75446	-1.41719	-1.00411	-0.59298	-0.04078	-0.79725	-0.81754	-0.83164	-0.71136	-0.93782	-0.606	-0.42775	-1.14829	-0.47125	-0.02544
O14521	SDHD	0.149404	-0.43422	-0.17617	0.211155	0.061636	-0.01763	-0.14487	0.350043	-0.33381	-0.15713	-0.01514	0.088848	-0.35475	-0.27769	1.8431	0.709956	-0.56848	-0.9908	-0.55946
O14523	C2CD2L	-0.82533	-0.04962	0.037579	0.231418	-0.90244	-0.34167	0.160021	-0.96029	0.311254	0.04022	0.555124	0.099102	-0.01084	0.174455	0.255547	-0.17392	-0.02502	0.184356	-0.91327
O14524	NEMP1	0.324164	-0.35257	0.187144	0.104738	0.660824	0.76072	1.593473	0.534753	0.571044	-1.02686	0.586857	-1.06876	-0.88569	-0.5631	0.978145	-1.34417	0.615876	0.138354	-0.01286
O14530	TXNDC9	-0.27644	0.219136	0.123442	-0.08413	0.245016	0.843968	-0.59216	0.380874	0.107917	0.539025	-0.30005	-0.41539	0.136724	-0.26126	-0.12991	0.591206	0.359313	0.878197	0.995676
O14531	DPYSL4	-0.70623	0.119099	-0.12178	-0.63272	0.062492	-0.59532	-0.06077	-0.00273	0.664741	-0.52949	-0.29325	0.104178	0.636813	-0.54819	-1.15542	0.002363	-0.44855	-0.34321	0.982664
O14545	TRAFD1	-0.94349	0.368908	0.331584	-0.48143	-0.69557	-0.53055	-0.28877	-0.79251	0.436974	0.0022	-0.69822	-0.41347	0.273723	0.02259	-0.03048	0.36197	0.531207	0.782805	0.829359
O14548	COX7A2L	-0.2501	-0.12209	-0.11303	0.423855	1.076131	0.75318	0.267407	0.02132	-0.31534	-0.27914	0.218857	-0.25864	-0.05095	0.046826	2.174731	-1.63707	-0.86879	-0.65026	-0.90447
O14558	HSPB6	-0.91868	-0.649	-1.27115	-0.86821	-1.34314	-2.00733	-0.90091	-2.22581	0.471632	-0.69243	-1.18378	-0.80215	-1.45474	-0.64643	1.316564	-1.98369	-0.67692	0.020355	-0.15727
O14561	NDUFAB1	0.634408	-0.73871	1.021419	0.838454	0.22204	0.267761	1.39042	-0.02071	0.044587	0.059746	0.613869	0.627859	0.363627	0.400593	1.386039	0.079394	0.233465	0.223511	0.086471
O14562	UBFD1	0.730986	-0.4348	-0.51788	-0.56003	-0.59935	-0.31849	-0.77054	0.218117	-0.01241	-0.14082	0.427363	-0.82061	0.294414	-1.00759	-1.0208	-0.22155	0.122906	0.455458	0.419326
O14578	CIT	0.888743	0.072926	0.375259	0.101131	1.124522	1.26173	0.611864	1.664444	0.231541	-0.61497	-0.7444	-0.79359	0.353252	-0.36828	-0.88111	-0.61032	-0.2999	-0.53981	0.496843
O14579	COPE	-0.27127	0.57323	0.039718	0.116928	0.107207	-0.03905	0.662662	0.036874	0.494364	0.451419	-0.05769	-0.22815	0.495814	0.04319	0.404387	0.547503	0.380974	0.281009	0.656422
O14593	RFXANK	-0.05283	0.349367	0.181286	-0.32002	0.869625	-1.25554	0.059133	-0.49439	0.185279	0.732008	-0.53007	0.678347	0.080664	1.118137	-0.60026	-0.25527	0.816028	-0.11985	-1.40988
O14617	AP3D1	-0.65897	0.60962	0.108375	-0.26276	-0.09739	-0.02472	0.027208	-0.15844	0.542495	0.039975	0.096518	0.121871	0.452939	0.21571	0.106193	0.09136	-0.03973	0.101312	0.304067
O14618	CCS	-0.62831	0.02944	-0.06555	-0.1605	0.643623	0.12706	-0.0829	0.887231	0.049267	0.072979	-0.26653	-0.09696	0.233068	-0.13814	-0.75339	-0.31078	0.478116	0.604446	-0.47236
O14639	ABLIM1	-0.26694	-0.83611	-1.51459	-0.63613	-1.14727	-0.83228	-0.89625	0.506387	-0.44869	-1.12549	0.845031	-1.07397	0.561732	-0.88111	0.595413	-1.35419	-0.98503	-0.67569	-0.80485
O14641	DVL2	0.507928	0.062505	0.220485	-0.32006	-0.38994	-0.13375	-0.143	-0.2547	-0.06455	-0.32216	0.368933	-0.47029	0.261726	-0.24719	-0.6755	-0.19987	-0.31517	-0.03866	0.056031
O14646	CHD1	0.171927	0.130072	-0.34804	0.062333	-0.07207	0.169732	-0.09111	0.251981	-0.2302	-0.12034	0.013459	-0.08205	-0.14465	0.20061	0.137948	0.131551	0.582227	0.838639	-0.09525
O14647	CHD2	0.567072	-0.06161	0.05428	0.043704	0.000605	0.219581	-0.28965	0.448165	0.56117	0.05175	0.364522	-0.44211	-0.35539	-0.1816	0.164907	-0.10697	0.124173	0.606263	-0.01102
O14653	GOSR2	-0.73836	0.386051	0.346129	0.613604	0.013036	0.063253	0.296505	0.051234	0.061899	-0.29472	-0.31748	0.248574	0.316857	-0.29915	0.024205	0.634089	0.006724	0.368961	0.35014
O14656	TOR1A	0.332152	0.440217	0.013695	-0.16358	0.223032	0.049181	-0.32194	0.310248	0.732951	-0.34194	0.318481	-0.46482	-0.69674	0.570174	-0.39748	-0.6209	-0.13372	0.135548	0.182905
O14657	TOR1B	0.018176	0.362659	0.032414	-0.21505	-0.13805	0.124853	0.247588	-0.04453	0.159377	-0.01178	-0.65267	0.203646	-0.2043	0.729993	-0.19364	-0.44063	0.062556	-0.24951	-0.44049
O14662	STX16	-0.11696	0.250114	-1.02896	0.476621	0.083633	0.342032	0.347276	-0.07592	-0.1203	-0.17052	0.187246	0.032575	0.291939	-0.08605	-0.14353	-0.25185	0.215883	0.281569	-0.22633
O14672	ADAM10	-0.01254	0.167815	-0.31701	0.460454	-0.19081	-0.18973	0.210781	-0.72445	-0.50361	-0.2895	0.78391	-0.65488	-0.4146	-0.21666	-0.24944	0.455938	0.033715	0.487445	-1.01034
O14678	ABCD4	-0.38501	0.779776	-0.25809	0.262056	-0.7777	-0.18792	-0.30931	0.127045	0.040847	-0.61374	0.617822	-0.28958	0.835967	0.592515	0.052144	0.775528	-0.09947	0.019388	-0.24552
O14681	EI24	-0.06265	0.148057	0.269759	0.932373	-0.38285	-0.35335	0.638603	0.071319	0.161811	-0.24752	0.092782	-0.0291	0.821549	-0.03824	0.112278	0.56255	0.571969	0.732534	0.222831
O14683	TP53I11	-0.83027	-0.04962	0.343602	0.180883	-0.21178	-0.21355	0.073499	-1.41758	0.040847	-1.69791	0.032108	0.150442	-0.01084	0.065911	0.186521	-1.12103	-0.32531	-1.01151	-0.34983
O14686	KMT2D	0.101773	0.044413	-0.20452	-0.23988	-0.87992	-0.38777	-0.00018	0.508758	-0.13005	-0.26164	0.051319	-0.61629	0.264581	-0.46362	0.502627	-0.63753	-0.26137	0.048749	-0.07868
O14727	APAF1	0.319671	0.630319	0.374714	-0.51556	-0.20085	-0.16747	0.650626	0.320155	-0.07036	0.773945	-0.05577	0.590421	-0.55592	2.036489	-0.22514	-0.66287	0.342011	-0.21088	0.334795
O14730	RIOK3	-0.44437	-0.21562	-0.23959	-0.69834	0.03643	0.118252	0.266889	0.167353	0.505555	-0.63647	0.220759	0.07182	0.227546	-0.00771	-0.37078	0.187643	0.761658	-0.2313	-0.3745
O14732	IMPA2	1.11535	-0.0231	0.15497	-0.33357	1.515174	1.490511	-0.30007	-0.5687	0.147339	0.111746	-0.6795	-0.41708	0.180535	0.720954	-0.75813	-0.02366	2.796309	5.146865	-1.31304
O14733	MAP2K7	-0.43255	-0.02018	0.039295	-0.37827	-0.11263	-0.25173	-0.31535	-0.14018	0.23206	-0.02819	-0.03813	-0.20562	0.011799	-0.34948	-0.54537	0.402594	-0.31584	-0.50878	-0.82645
O14734	ACOT8	-0.01633	0.501691	0.133682	0.706613	-0.15604	0.043633	0.283017	0.516001	0.389139	1.048537	-0.67845	0.683897	-0.0317	0.246684	-0.19155	0.234979	0.336144	0.230446	-0.20154
O14735	CDIPT	-0.03213	0.245885	-0.00775	-0.41738	0.457389	0.498201	0.341381	-0.51578	-0.36374	-0.44101	-0.14694	-0.11957	-0.42642	-0.12964	0.37995	-0.03384	-0.4424	-0.49638	-0.27723
O14737	PDCD5	0.08261	-0.67985	0.473104	-0.50698	1.749598	0.940977	-0.52727	0.302068	0.45165	-0.58325	-0.48561	0.482812	-0.42228	-0.24122	-0.16526	0.647968	0.746511	-0.18102	-0.92122
O14744	PRMT5	0.415622	-0.47821	-0.50477	0.962156	-0.27216	0.37793	-0.72981	0.457441	-0.23796	0.086168	0.610643	-0.20394	-0.12224	-0.40457	0.307882	-0.00212	-0.16241	-0.6805	0.016583
O14745	SLC9A3R1	1.548757	-0.85756	-0.3836	-0.03014	-0.03361	-0.99504	-0.28907	0.962676	0.103891	0.261562	0.539933	0.478193	0.402811	0.395845	-1.41121	-1.12475	-0.08297	-0.39928	-0.99664
O14772	FPGT	0.254351	-0.47647	-0.04287	0.290788	0.825199	0.682821	-0.30677	0.970786	-0.01162	0.008487	-0.10632	0.22644	0.024828	0.759931	0.943719	0.306187	0.657243	-0.4618	0.021174
O14773	TPP1	-0.75641	2.083809	0.620873	-0.74575	0.697287	0.734871	0.514369	-0.48228	0.472486	0.288189	-0.32017	0.694384	0.907499	0.151228	0.016284	0.013691	-0.53833	-0.40514	-0.58624
O14776	TCERG1	0.138207	0.126495	0.135175	0.165052	-0.12219	0.165902	-0.19648	0.251061	-0.16327	0.122087	0.202075	-0.43703	-0.34348	-0.38397	0.707742	-0.04008	0.028353	0.28499	0.199499
O14777	NDC80	1.059769	-0.06796	0.952547	-0.33133	1.403583	2.155624	0.18399	2.00096	0.252933	0.338846	-0.98071	-0.37777	0.553304	-0.49952	-0.64658	-0.48108	0.28571	0.090533	-0.04477
O14786	NRP1	-0.72326	0.067016	0.291672	-0.7985	0.138084	0.200773	-0.15192	-1.23842	0.196231	0.423075	-0.07604	-0.10358	-0.14246	0.020683	-0.04765	-0.56739	-0.78441	0.435304	-1.0707
O14787	TNPO2	-0.5374	0.026909	-0.35232	0.032432	-0.48013	-0.50074	-0.06714	0.451852	-0.0944	-0.76658	-0.10789	-0.26	-0.72974	-0.43025	-0.97382	0.09206	-0.44579	-0.67795	0.482178
O14791	APOL1	-1.15895	1.064407	-0.51129	-0.60349	-0.79244	-0.13603	0.139384	-1.33682	0.304369	-0.93151	-1.11068	-0.02931	-0.60806	0.550689	-0.84466	-0.73173	0.995568	0.711975	-0.49875
O14795	UNC13B	-0.01834	0.10532	0.054516	0.049086	-0.22608	-0.29353	-0.30481	0.43854	-0.20126	0.007685	-0.39676	-0.23262	-0.06699	-0.10639	0.1643	0.367712	-0.29693	-0.09995	0.075894
O14802	POLR3A	0.401588	-0.4093	-0.23741	-0.31056	-0.23591	-0.2207	-0.15645	0.017038	-0.16511	-0.3611	0.198892	0.104185	0.355388	-0.63348	-0.09117	0.280596	0.024434	0.015923	-0.16348
O14807	MRAS	-0.10536	-0.11777	-0.11669	-1.27772	-0.38237	-0.47331	-0.13298	-1.18357	-0.21393	-0.80742	0.340934	0.040583	-0.07682	-0.02768	2.529212	-0.31414	-0.13972	-0.48703	0.400103
O14818	PSMA7	0.161503	-0.65896	0.332548	-0.00501	0.851958	0.727284	-0.68388	0.368924	0.02008	0.28975	0.298303	-0.8396	0.022386	-0.5074	-0.04875	0.31634	-0.5114	-0.0673	-0.10046
O14828	SCAMP3	-0.5222	0.045641	-0.12276	-0.21161	-0.30073	-0.43514	0.655883	-0.33606	0.725076	-0.01127	-0.28333	0.00194	-0.36406	-0.25056	0.647411	0.293937	0.187223	-0.06744	-0.41727
O14832	PHYH	0.453276	-0.06144	-0.2948	-0.29127	-0.22862	-0.08654	0.0563	0.175364	0.231318	1.23025	-0.53784	-0.067	-0.07035	-0.56301	1.128001	0.105716	0.772187	2.144035	-0.44839
O14841	OPLAH	0.200882	-0.82804	-0.57599	-0.69169	-0.38008	-0.63483	-0.64137	0.443344	0.056901	-0.17307	-0.53135	0.465094	0.908438	0.262255	0.847878	1.602528	-0.03091	-0.0617	-0.60752
O14867	BACH1	0.029593	0.711299	0.045599	-0.35475	-0.80403	-0.21712	0.254855	-0.56532	0.173402	0.646451	-0.35653	0.301829	0.187348	0.756674	0.018514	-0.27316	1.476347	0.990909	0.803709
O14874	BCKDK	0.369513	0.423118	0.095685	0.269852	0.069178	0.323765	0.198232	-0.71561	0.381788	0.639015	-0.09659	0.431454	0.039052	0.614456	-0.19932	0.922043	0.411893	0.244591	-0.3778
O14879	IFIT3	0.369019	1.59062	3.490064	-0.99148	-0.41863	-0.0254	-0.75172	0.003745	-0.90408	-0.70719	-1.35781	0.62732	0.339281	1.080344	-0.98496	-1.23749	-0.01386	0.196881	-1.73161
O14880	MGST3	-0.4541	-0.2119	-0.28263	-0.21991	0.55403	-0.28776	-0.27939	0.495497	-0.44925	0.83986	-0.15437	-0.2187	-0.4506	0.020097	1.816423	0.548341	-0.81612	-1.24019	-0.40138
O14896	IRF6	-0.11528	-0.47994	-0.6257	-0.34539	0.419667	0.198276	-0.39279	0.82955	-0.64805	-1.12211	1.597337	-0.5853	-0.60316	-0.47496	0.539995	-1.43175	-0.55682	-1.99605	-0.69972
O14907	TAX1BP3	1.221418	-1.09021	-0.23583	-0.53312	-0.00039	-0.59962	-0.79528	0.041028	0.047773	1.331278	-0.0049	0.406361	0.83694	0.0635	0.470578	-0.85138	0.2373	-0.6325	-0.22189
O14908	GIPC1	-0.33268	-0.5143	2.426137	0.189568	-0.80129	-0.36746	0.168976	2.633728	0.011036	0.445935	0.026533	-0.49257	-0.415	-0.57077	1.333686	0.082185	0.217365	-0.37459	-0.10136
O14920	IKBKB	0.025977	0.312815	0.552811	-0.79691	0.435504	0.284332	-0.02514	-0.65545	-0.59067	0.329755	0.013873	0.409282	0.535588	0.731576	-0.09927	-0.45583	0.207657	-0.37285	-0.37336
O14925	TIMM23	0.066648	-0.64381	-0.29748	0.096218	-0.43722	0.019512	-0.29784	1.084511	-0.39952	-0.59594	-0.17714	-0.19374	-0.40069	0.067879	0.590399	0.194822	-0.25878	-0.45146	-0.05737
O14929	HAT1	1.147095	-0.12108	0.153889	0.92653	0.523961	0.770133	-0.30333	0.399125	0.320315	0.758428	-0.78909	-0.45509	-0.54845	-0.02249	-0.4268	0.644227	0.461988	-0.4177	-0.32614
O14933	UBE2L6	0.162088	0.749561	1.554592	0.021435	0.422084	0.884594	0.523679	0.471735	-0.20833	-0.48251	-0.48965	0.231107	1.355593	0.216122	-0.45912	-0.24393	-0.34028	-0.14071	-0.80193
O14936	CASK	-0.23104	-0.83231	0.023139	-0.49201	-1.10956	-1.18019	-0.22283	0.205903	-0.07435	-0.05204	0.544177	-1.02553	-0.03556	-1.25741	0.003844	0.015853	-0.35385	0.567173	0.619968
O14939	PLD2	0.524869	0.152821	-0.60902	-0.49639	-0.47788	-0.41698	0.046329	-0.25219	-0.38541	-0.1164	0.482445	-0.08704	-1.0163	-0.1196	0.731391	-0.54927	-0.39283	-0.30662	-0.77531
O14949	UQCRQ	0.210435	-0.02925	-0.27787	0.353943	0.674234	0.479736	0.063062	-0.42893	-0.47365	-0.59855	0.424571	-0.28667	-0.7203	0.134312	1.689445	-1.29299	-0.70779	-0.51778	-0.9955
O14950	MYL12B	0.194882	0.242003	-0.11819	-0.11793	-0.02237	0.328264	-0.0858	-0.38582	0.432626	-0.02595	-0.8939	0.230923	0.240611	0.084625	-0.70824	0.28677	-0.45331	-0.85693	0.163735
O14964	HGS	-0.0112	0.169345	-0.11124	-0.56242	-0.38272	-0.48412	-0.85321	-0.11592	-0.49344	0.161843	0.098945	-0.36344	0.275308	-0.16275	0.247445	0.294573	-0.18364	-0.01853	0.290348
O14966	RAB29	-0.42806	0.765365	0.360028	-0.53397	0.094752	-0.70896	0.48464	-0.08204	-0.04717	1.515106	-0.23098	0.626104	-0.49952	0.64792	1.194215	-0.87337	0.043991	0.116342	-0.20321
O14967	CLGN	-0.53193	-1.12221	-2.2973	-0.04356	3.298442	2.485659	-0.23374	0.393637	0.096347	-2.68351	-0.26617	0.087536	0.600536	-0.32325	0.265372	-0.79793	-2.46028	-3.29846	-2.42779
O14972	DSCR3	0.269535	0.581618	0.246854	-0.16527	0.175225	0.070518	0.23349	-0.42112	0.231568	0.607595	0.054719	0.644382	0.120401	0.751356	-0.47114	0.569301	-0.15411	-0.22477	-0.30723
O14974	PPP1R12A	0.254096	-0.26682	-0.0793	-0.33796	-1.20284	-0.46972	-0.98403	-0.29615	0.017507	-0.44302	-0.29578	-1.20578	0.36461	-0.78183	0.076877	-0.13756	-0.66094	0.19996	0.826767
O14975	SLC27A2	0.976128	-0.18907	-0.45852	-0.39774	0.770255	0.802347	-0.35666	-1.7497	-0.18026	-0.24692	2.077253	-0.04893	-0.40248	0.883824	-1.45711	-0.21508	-0.17114	-0.98158	-0.38873
O14976	GAK	0.969057	0.366831	0.531461	-0.33255	-0.40853	-0.33636	0.227884	-0.42287	-0.24728	0.680214	-0.00722	0.232682	-0.0474	0.438315	0.002387	0.372771	-0.19341	-0.36655	-0.42977
O14979	HNRNPDL	0.46823	-0.277	-0.05947	-0.31047	0.174833	0.393972	-0.73	0.640052	-0.65883	-0.82461	0.275684	-0.64729	-0.44371	-0.85128	-0.4973	-0.80253	0.001928	0.554447	0.195807
O14980	XPO1	0.482126	-0.03538	-0.04084	0.176994	0.130569	0.42602	0.133148	0.372678	-0.10736	-0.05523	-0.11863	-0.1637	-0.26126	-0.09917	0.497794	0.109022	-0.15574	-0.86137	0.087257
O14981	BTAF1	0.39797	0.029598	-0.6173	0.067332	-0.04859	0.159803	0.423697	0.103044	0.268295	-0.19557	-0.20297	0.212899	-0.62953	0.264729	0.103515	0.031674	0.296477	0.14668	-0.08683
O15013	ARHGEF1	-0.7731	0.003659	0.251051	-0.05497	0.069394	0.227168	0.461556	-0.30056	0.175945	0.441286	-0.27731	-0.08857	0.288068	-0.43052	-0.11448	0.253051	-0.06849	-0.55937	0.807476
O15014	ZNF609	0.483053	-0.30599	-0.29643	-0.49466	-0.45533	0.119743	-0.53675	-0.15563	-0.11107	-0.91763	0.223787	-1.40428	0.019992	-1.29083	-0.73765	0.023842	0.098301	0.841583	0.348026
O15020	SPTBN2	0.220458	-0.7329	-0.19068	-0.11322	-1.23939	-0.7229	0.170558	1.405899	-0.35921	-0.97679	0.754606	-0.88327	0.009662	-1.01659	-0.48656	-1.02954	-0.94029	-1.47595	-1.11123

O15021	MAST4	-0.13571	1.02946	-0.00828	-0.71142	-0.95919	-0.68243	-0.06964	-1.03027	0.040847	-2.19182	0.212958	-0.36728	0.151377	-0.4792	-0.55448	-0.9785	-0.53082	-0.21292	-0.15053
O15027	SEC16A	-0.21872	0.282411	0.055062	-0.44865	-0.52076	-0.26532	0.858101	0.139618	0.312613	-0.28244	-0.04353	-0.24804	-0.04417	-0.0209	0.049769	0.067104	0.069265	0.3831	0.412318
O15031	PLXNB2	-0.06711	0.437699	-0.22215	-0.12372	0.097406	-0.04611	0.124302	-0.2572	-0.12469	0.067472	0.323717	-0.58715	-0.17393	-0.27624	0.212153	-0.02152	-0.44813	0.153404	-0.05621
O15037	KHNYN	0.864395	0.023905	0.316507	-0.84411	-0.67766	-0.31919	0.132898	-0.03146	0.040055	0.014671	-0.03689	-0.33	0.137834	0.099509	0.095295	0.043056	0.067952	-0.26986	0.042539
O15040	TECPR2	-0.13667	-0.06029	0.050935	-0.11912	-0.41983	-0.52418	0.054735	0.293048	-0.55586	-0.72737	0.228886	0.047864	-0.06207	0.016971	0.570972	-0.51743	-0.4435	-0.08225	0.524532
O15042	U2SURP	0.436796	-0.19116	0.128194	0.415308	-0.16088	0.194448	-0.55769	0.868991	0.314176	-0.01864	0.305028	-0.48018	-0.27831	-0.66074	0.094682	-0.27276	0.127921	0.372953	-0.05551
O15047	SETD1A	0.567258	-0.27558	0.306094	-0.36489	0.036411	0.281052	0.292272	0.077914	-0.30513	-0.38234	0.277596	-0.07705	-0.56306	0.140738	0.240223	-0.09572	0.096277	0.107466	-0.18363
O15056	SYNJ2	-0.3629	0.082789	0.094537	-0.44875	-0.50032	-0.52625	-0.08482	-0.06714	-0.5802	-0.30173	0.677925	-0.51147	-0.11514	-0.56616	-0.67504	-0.30289	-0.13406	0.552727	1.408655
O15061	SYNM	-1.79655	-1.42327	-0.94667	-2.40589	-1.93673	-2.54954	-0.20399	-2.26071	-0.51551	-2.25711	-3.15006	-1.34519	-1.42951	-0.75549	-0.31615	-1.19082	-1.99914	-2.41108	-1.8672
O15066	KIF3B	0.035423	-0.06578	-0.01144	-0.32481	-0.42116	0.29887	-0.10186	0.237045	0.156322	-0.09521	0.272011	-0.39311	0.607176	-0.32967	0.470968	0.288133	0.08577	-0.51258	0.234808
O15067	PFAS	1.16053	-0.95237	-0.3163	-0.21443	0.318932	-0.25061	-0.26467	0.046825	0.252172	0.728422	0.589739	-0.58835	-0.29698	-0.28768	-0.09492	-0.49691	-0.40181	0.176283	0.020129
O15068	MCF2L	-0.03512	-0.11301	-0.08105	-0.62496	-0.26404	-0.1319	0.022302	0.808468	-0.25857	-0.1659	1.275119	-1.39783	-0.08683	0.044823	0.347371	-0.14436	-0.43315	-0.36061	-0.59322
O15075	DCLK1	-1.46814	0.408187	1.232695	-1.73559	0.735467	-0.6487	0.872792	-1.21515	0.136631	-0.72346	-1.26613	-0.16689	-0.56031	-1.04984	-0.20238	-0.28368	-0.92603	-0.27624	2.074934
O15078	CEP290	-0.36303	0.209139	0.549	-0.85532	-0.02202	-0.15146	-0.53997	-0.3107	0.484707	-0.73204	1.199631	-0.44811	0.176242	0.314813	0.00315	0.149755	-0.44357	0.069706	-0.09201
O15083	ERC2	0.680029	-0.02393	-0.18262	-0.06628	-0.10013	-0.96824	-0.12107	0.14802	0.497715	-0.14094	0.238667	-0.20397	0.162558	-0.76209	0.034641	0.135612	-0.57771	-0.00843	0.422506
O15084	ANKRD28	1.073372	-0.27809	-0.4666	-0.24809	-0.10792	0.30803	-0.49661	-0.31872	-0.21168	-0.70322	0.259431	-0.15281	0.372447	-0.55203	-0.4649	0.478194	-0.54525	-1.068	-0.34833
O15085	ARHGEF1	-0.47107	0.436708	0.047616	-0.24976	-0.16393	-0.73515	-0.55492	0.16448	0.357609	0.023528	-0.16212	0.11767	-0.12082	0.128437	0.342361	-0.53207	0.039882	0.081491	-0.39333
O15091	KIAA0391	0.582522	-0.40847	-0.01595	1.309041	0.052043	-0.57359	-0.43774	-0.4865	0.807074	0.818537	0.122389	-0.33894	0.040521	-0.37155	-0.13839	1.230773	0.204421	-0.02112	0.215616
O15111	CHUK	-0.39691	-0.439	0.350024	-0.44054	-0.00965	-0.04683	0.470914	-0.40719	0.184272	0.498588	-1.00305	0.82557	0.551211	1.16777	0.425192	-0.58954	0.385447	-0.30456	-0.03275
O15116	LSM1	0.161969	-0.42938	-0.24621	-0.06858	0.834772	-0.21581	-0.01604	-0.12264	0.360477	0.180894	0.254684	-0.04247	0.774586	0.277408	-0.00135	0.040878	1.113541	0.991942	0.140899
O15117	FYB1	-3.07357	1.377488	1.315512	-0.95189	0.725063	0.564841	0.194083	-2.3152	-0.10326	1.397222	-2.11185	0.920698	-0.12731	1.09198	-0.53547	-0.88233	0.10541	0.971185	-1.32518
O15118	NPC1	-0.86172	-0.03094	0.560315	-0.65447	0.076669	0.315458	0.620055	0.007242	0.137185	-0.1545	-0.74691	0.324705	-0.12888	0.867063	0.063424	-0.12155	0.301553	0.093674	-0.20347
O15121	DEGS1	0.162544	0.408031	-0.17999	0.165872	-0.40057	-0.35547	-0.14214	0.565235	1.135335	1.071372	0.052682	0.75894	0.747451	0.48422	0.350444	-0.04358	0.886678	0.2661	0.196017
O15126	SCAMP1	-0.60605	0.251711	0.620898	-0.32223	-0.35563	-0.49081	0.638959	-0.16395	-0.15215	-0.39018	0.40849	0.087504	0.416013	0.052029	0.734973	-0.0108	-0.34087	0.194103	-0.10106
O15127	SCAMP2	-0.45572	0.748165	0.053833	-0.33523	0.052732	-0.16139	1.231187	0.145809	0.289131	-0.00763	-0.21455	0.675367	0.436239	0.425531	0.323743	-0.04515	0.353972	0.36388	-0.27442
O15143	ARPC1B	-0.3622	0.317425	0.998029	-0.52799	0.481738	0.097524	-0.1628	-0.23589	0.298764	0.486148	-0.51508	-0.1411	0.420246	0.978747	-0.21577	0.09836	-0.40927	0.025668	-0.0972
O15144	ARPC2	-0.39987	0.405304	0.660017	-0.41345	0.211996	0.175485	-0.65962	0.003784	0.046951	0.405185	-0.04321	-0.2308	0.117568	0.705092	-0.34007	0.28037	-0.53122	-0.03556	0.424534
O15145	ARPC3	-0.40871	0.442785	0.419055	-0.46882	0.306479	0.078777	0.211102	0.009417	0.437173	0.274261	-0.4082	-0.10072	0.05977	0.820434	-0.46449	0.152098	-0.42721	-0.21779	0.130431
O15160	POLR1C	0.190273	-0.16283	-0.28524	-0.17259	-0.03258	0.185316	0.060934	0.16521	-0.19731	-0.14309	0.167918	0.02358	0.158758	0.105047	-0.10997	-0.18328	0.693694	-0.19969	0.342709
O15162	PLSCR1	0.827628	1.2101	1.584181	-1.0335	1.174463	0.951062	-0.02976	-0.07052	-0.20133	-0.20847	-1.16168	0.66872	-0.29681	1.001036	-0.92866	-0.64429	-0.44442	-0.93348	-1.71633
O15164	TRIM24	1.079832	-0.26354	-0.11456	0.458786	-0.20156	-0.20326	-0.33912	0.25738	-0.30739	-0.15343	-0.03592	-0.78448	-0.11929	-0.35995	0.530809	0.372343	0.052251	0.041441	-0.15499
O15173	PGRMC2	-0.22519	0.339789	-0.52169	-0.29349	-0.288	-0.72035	0.628422	-0.4055	0.221409	-0.45183	-0.00642	-0.38365	-0.03463	-0.39152	-0.18362	-0.007	0.178125	0.711713	0.190644
O15182	CETN3	0.752832	-0.25435	0.106737	-0.536	1.14414	1.08643	-0.5064	0.223213	-0.65097	-0.5731	0.503879	-0.26001	-0.25447	-0.0325	-0.67491	0.381363	0.082922	-0.32893	-0.28202
O15195	VILL	0.103579	-0.61687	-0.75308	-0.10237	-2.21073	-1.43341	-0.29161	-0.30087	-0.31362	-0.8216	1.621861	-1.30944	-0.31068	-0.88084	0.326556	-0.04749	-1.26865	-0.97193	-1.45282
O15211	RGL2	-0.26081	0.112	0.113738	-0.04143	-0.33013	0.093519	0.236072	-0.46298	-0.12373	-0.16043	-0.21722	0.489836	0.165061	0.22003	-0.1022	-0.16719	0.008297	0.069431	-0.07124
O15212	PFDN6	0.428528	-1.06215	-0.00388	0.164663	0.779624	1.022868	-0.81188	1.179749	-0.08806	0.252819	-0.25869	-0.60353	0.201238	-0.52997	0.510722	0.756105	-0.33397	-0.32407	-0.01127
O15213	WDR46	0.094459	-0.15912	-0.57761	0.598339	-0.09518	-0.02226	-0.78656	0.430946	-1.21598	-0.02877	0.979784	-0.25489	-0.20807	-0.43164	0.292334	0.172406	-0.31346	-0.33886	-0.11182
O15226	NKRF	0.553769	-0.0417	-0.11386	0.835924	0.044175	0.303826	-0.39303	0.484806	-0.04468	0.356664	0.404249	-0.5888	-0.24261	-0.49217	-0.71094	0.289561	0.008295	-0.13489	-0.60618
O15228	GNPAT	-0.90098	0.212615	-0.26729	0.383938	-0.07349	-0.13011	0.44095	0.880058	-0.42366	0.876922	-0.03461	0.414318	-0.24248	0.524791	0.18037	0.37861	-0.12677	-0.25262	0.048376
O15230	LAMA5	-0.26785	-0.67247	-0.29374	-1.0557	-0.43543	-1.08914	0.486126	0.083777	-0.34057	0.112575	-0.23381	-0.91446	-0.85386	-0.57948	1.037814	-0.98105	0.215155	1.094559	-0.738
O15231	ZNF185	-0.04532	-1.14561	0.409951	1.00647	-2.08457	-1.35529	-0.45516	-0.08157	-0.06234	-1.34479	0.408984	-1.33736	-0.61753	-1.61316	0.531003	-0.50875	-0.49812	-0.82373	-0.91494
O15234	CASC3	0.358703	-0.2309	0.063671	-0.17925	-0.26088	0.593078	-0.0559	0.302787	-0.02401	0.024095	0.233868	-0.70599	0.60081	-0.28376	-0.17458	0.081204	-0.3545	0.209437	0.614111
O15247	CLIC2	-1.29155	1.248494	0.430383	-0.93172	0.663039	-0.29619	0.276865	-0.91468	-0.08988	0.694727	-1.11606	0.511666	0.270657	0.860475	-0.13029	-0.41897	-0.03812	0.78343	-1.03865
O15254	ACOX3	0.611734	0.412023	1.048894	-0.602	0.209174	0.338089	-0.56665	-0.03875	-0.27763	0.269529	0.048131	0.784284	-0.09301	0.443738	-0.51989	-0.5582	0.110558	-0.5846	-0.0793
O15258	RER1	-0.41657	0.295769	0.018911	0.271945	0.372261	0.526913	0.496838	-0.05545	0.063584	-0.08261	-0.16201	-0.06653	-0.08612	0.086394	0.520377	0.89259	0.971465	0.320215	0.005707
O15260	SURF4	-0.19454	0.498571	0.506992	-0.06855	0.134834	0.085574	0.264588	0.029661	0.390514	-0.06902	-0.26234	0.259802	0.15005	0.289422	0.01873	0.290225	0.213334	0.300903	0.806553
O15264	MAPK13	1.088658	-0.26703	0.144495	-0.49292	0.123584	0.215575	-0.25469	-1.23062	-0.05094	0.150039	-0.23433	-0.37537	-0.62939	0.002904	-0.13816	0.087166	0.687046	-0.48085	-0.40153
O15269	SPTLC1	-0.38216	0.411556	0.194434	1.054397	0.46964	0.666728	0.13893	0.326481	0.262321	-0.32636	-0.23585	0.187421	-0.21556	0.043725	0.336758	0.176066	0.480583	0.106889	0.585997
O15270	SPTLC2	-0.27023	0.372916	0.211477	0.328294	0.109391	0.034617	-0.38523	0.664167	-0.1166	-0.14093	0.242117	0.79958	0.093029	0.542514	0.13581	0.175635	1.225488	0.742658	0.660006
O15294	OGT	-0.01261	-0.42222	-0.02635	0.046292	0.138164	0.54189	-0.00977	0.123125	0.327318	-0.29124	-0.01127	-0.12215	-0.06648	-0.02198	0.039378	0.191164	0.326351	-0.09785	-0.1566
O15305	PMM2	0.300666	-0.91279	0.540333	-0.23967	0.684136	-0.08857	-0.25125	-0.00409	-0.21698	0.383558	-0.77441	0.789224	0.937785	0.181651	0.830262	0.482133	0.771073	-0.17822	0.941528
O15321	TM9SF1	-0.38889	0.105077	0.514573	0.804749	0.267195	0.257242	1.025713	0.356261	0.385298	-0.12134	0.24544	-0.21632	-0.25897	-0.05329	1.372826	0.273301	0.467938	0.149698	0.18021
O15327	INPP4B	-1.19684	-0.01641	0.070239	0.128661	2.143171	1.391415	0.272805	-0.60324	-0.05889	-0.31999	-0.15199	-0.53106	0.294664	-0.03741	-0.53138	-0.48395	0.054739	0.376841	-0.43268
O15344	MID1	0.723707	0.145965	0.194669	-0.42707	-0.54082	-0.52285	0.23358	0.302991	-0.20948	-1.06856	0.104414	-0.54813	-0.03801	-0.86855	0.077252	-0.88052	0.453544	-0.17632	0.080479
O15347	HMGB3	1.037774	-0.19363	0.16046	0.158499	-0.17516	0.504271	-1.17439	0.59433	-0.974	-1.12164	0.111256	0.024287	-0.29264	-0.33369	-0.79967	-0.08381	0.294771	0.127501	0.716892
O15355	PPM1G	1.076169	-1.40505	0.068843	0.842696	0.033455	0.368635	-1.13007	0.493879	-0.1172	0.216923	0.473422	-0.51967	0.239848	-0.31147	0.096274	0.852032	-0.0742	0.015198	-0.00864
O15357	INPPL1	0.116428	0.320515	-0.43214	-0.21871	0.660749	1.002447	-0.39318	-0.11432	0.020632	-0.3025	0.000891	0.178285	0.21786	0.249463	0.048908	0.351713	0.252225	-0.03906	-0.21993
O15371	EIF3D	-0.10311	-0.16001	0.185552	-0.01727	-0.0852	0.401952	-0.4318	0.180363	-0.05462	0.37918	0.171113	-0.39523	0.03943	-0.09004	0.318132	0.394648	0.025185	-0.14776	-0.30725
O15372	EIF3H	-0.12763	-0.43654	-0.08722	-0.19244	0.110875	0.288713	-0.59178	0.215024	-0.4147	0.289251	0.19933	-0.27869	0.067765	-0.45354	0.518911	0.10869	0.075077	-0.19417	-0.13836
O15379	HDAC3	0.269369	0.230804	0.098152	-0.08319	-0.86018	-0.51371	0.471453	0.609842	-0.75802	-0.32703	0.687588	-0.56412	-0.03157	-0.46051	0.436339	-0.74285	-0.1522	0.10343	0.020166
O15381	NVL	0.162682	-0.12677	0.00054	-0.19613	-0.6961	-0.62879	0.176871	0.649073	-0.17884	0.172624	-0.24113	-0.25927	-0.03056	-0.52327	0.580826	0.26502	0.089105	-0.32652	-0.42112
O15382	BCAT2	1.40271	-0.3417	0.023317	0.924862	0.487653	0.640204	0.287613	0.809197	0.084631	0.186866	0.200753	-0.14628	-0.29113	0.26722	2.099614	0.027502	0.023774	0.238784	-0.05562
O15391	YY2	0.645664	-0.44758	0.187368	0.76345	0.13673	0.098414	-0.27492	0.385833	0.345275	0.277987	0.373071	0.234189	0.256417	0.149439	0.402753	0.252193	0.832737	0.34086	0.522345
O15397	IPO8	-0.09393	-0.14363	0.103091	0.029745	0.321857	0.559813	-0.21357	0.003694	0.591983	0.301065	0.051028	-0.28001	-0.31151	-0.05036	-0.14411	0.14184	0.231386	-0.07471	0.628397
O15400	STX7	-0.42995	0.643949	0.216022	-0.15585	-0.21602	-0.61552	0.311256	-1.15854	-0.1359	0.443117	-0.1269	0.165029	0.096761	0.183879	0.808934	0.149847	-0.16912	0.405154	-0.13702
O15417	TNRC18	-0.29563	0.316084	0.335846	-0.1098	-0.23686	-0.00013	0.195981	0.319787	0.050373	0.113897	-0.59133	-0.44426	0.208038	0.261307	0.10787	0.074591	-0.47611	-0.31002	0.144791
O15427	SLC16A3	0.632481	0.894812	0.917496	0.120163	-0.40263	-0.26671	-0.03192	0.093322	0.196581	1.124299	-0.58763	1.546712	1.085928	1.235505	-1.93766	-0.14023	1.117699	0.471376	0.038052
O15438	ABCC3	-0.99335	0.511194	-0.40978	1.306345	0.127717	-0.55706	0.710089	-0.4662	0.040847	0.339251	-0.08243	0.318529	0.236942	-0.48232	-1.16638	-0.35986	-0.42956	-0.1082	-0.64162
O15439	ABCC4	0.071232	-0.19584	-1.31287	-0.99377	0.538347	-0.07576	0.24992	0.814789	-0.11158	0.403682	0.763969	-0.20459	-0.23732	-0.63188	-1.11723	-1.00854	-0.22522	-0.25043	-0.35827
O15446	CD3EAP	0.653043	-0.27534	-0.38155	-0.18391	-0.69876	-0.10984	0.34066	-0.16585	0.663015	-1.15222	0.327217	-0.36326	-0.11548	-0.31407	0.620542	-0.3339	-0.48425	-0.1045	0.037529
O15455	TLR3	-0.38057	0.226806	-0.00828	-0.40315	-0.22728	-0.19011	-0.06964	-0.10273	0.040847	-1.18687	0.13704	-0.49571	-1.25026	-0.09406	-1.26526	-0.54695	0.199105	0.155868	-0.9615
O15460	P4HA2	-0.16925	0.435671	0.163636	0.117448	0.42616	0.919029	-0.36497	0.971811	0.941437	-0.45451	-0.68502	0.018314	2.263945	-0.49452	-1.13043	0.616726	0.636144	0.574375	2.729094
O15484	CAPN5	-0.70085	-0.11273	-0.83312	-0.57479	-0.53206	-0.37318	-0.19699	-0.58134	-0.52183	-1.21898	0.671386	-0.11779	-0.06829	-0.38935	0.772933	-1.92107	-0.64439	-0.61874	-0.81477
O15498	YKT6	0.07444	0.52813	0.074939	-0.54993	0.212892	-0.0966	-0.05718	0.600995	-0.04947	0.249427	-0.66548	-0.20214	0.319433	-0.08927	-0.03123	0.118601	0.083338	-0.02697	0.341752
O15504	NUPL2	0.552241	-0.18192	-0.12097	-0.44463	-0.29211	-0.40139	-0.1233	0.298564	-0.22974	-0.15322	0.631884	-0.62652	-0.0705	-0.31749	0.491918	-0.19835	-0.1838	0.136891	-0.3468
O15511	ARPC5	-0.58412	0.308336	0.106289	-0.6398	0.821976	-0.40837	-0.30935	-0.32402	-0.00483	0.683008	-0.40405	-0.06765	0.188981	0.720546	-0.65643	-0.21953	-0.53106	-0.21047	0.034337
O15514	POLR2D	0.822242	-0.87861	0.077185	0.112767	0.645948	0.531611	-0.53931	0.483701	0.560619	0.012789	0.350496	-1.23816	-0.13871	-0.88731	0.070163	-0.0756	-0.02128	0.495175	-0.03701
O15530	PDPK1	-0.21602	0.358094	0.297953	-0.41318	-0.25039	-0.82471	-0.05859	-0.35522	0.446978	-0.20179	-0.7224	0.707864	0.058931	0.948033	-0.12379	0.011684	1.025544	0.103886	-0.17118
O15533	TAPBP	-0.61503	1.087256	1.117532	-0.24514	0.417625	0.471886	0.070005	-0.6248	-0.18741	-0.23057	-0.68285	0.709756	0.698631	0.510399	-0.8005	-0.89049	0.802823	0.12156	-1.12474
O15541	RNF113A	0.705863	-0.13364	-0.15132	0.075305	0.04231	-0.00671	-0.17639	-0.10662	-0.28715	-0.39383	0.374471	-0.46869	-0.16825	-0.62597	-0.45695	-0.15441	0.661949	0.515291	-0.7291
O15550	KDM6A	0.251891	-0.31216	0.020608	-0.0759	-0.20676	-0.47749	-0.00835	0.579409	-0.22392	-1.00598	-0.05385	-0.02025	-0.14467	0.410839	0.0345	-0.02966	0.45188	0.01856	-0.59329
O15551	CLDN3	1.942255	-0.99652	-0.08831	1.080695	-1.47152	0.489233	-0.46764	0.966133	-0.72491	-2.10754	0.243691	-2.13448	-1.56123	-1.58067	2.119477	-0.13003	-1.16277	-1.87107	0.132258
O43143	DHX15	0.272139	-0.28836	0.334609	-0.15843	-0.021	0.049697	-0.40826	0.263054	-0.17391	-0.05308	0.262258	-0.0901	-0.50246	0.192852	0.028451	0.551576	0.190364	0.037504	-0.03401
O43148	RNMT	1.059672	-0.34832	-0.27785	1.140174	0.115843	0.07583	-1.03458	0.799905	0.03689	0.526924	0.161476	-0.09612	-0.16125	-0.2963	0.861027	-0.62195	0.571989	0.101377	0.329362
O43149	ZZEF1	-0.10182	0.650181	-0.04617	-0.1859	0.026684	-0.01849	0.89604	-0.17988	-0.14154	0.084838	-0.35697	0.518025	0.350296	0.575365	-0.44189	-0.5898	-0.26383	-0.29379	-0.16768
O43150	ASAP2	-0.11614	0.452233	-0.32292	-0.44445	-0.90558	-0.89367	-0.07788	0.690251	0.100095	-0.11668	0.463169	-0.48065	-1.01707	-0.62518	-0.51595	-0.18124	-0.18766	-1.22001	0.158586
O43156	TTI1	0.627105	-0.11836	-0.11914	0.481358	0.175006	0.449907	-0.57392	-0.0706	-0.01747	0.772088	-0.13783	0.144671	0.078622	-0.08597	0.361237	-0.0423	0.57997	-0.60882	0.502278
O43157	PLXNB1	0.927475	-1.05165	-0.5225	-0.36867	-0.3136	-0.23763	0.099695	0.389978	0.497171	-0.89563	0.271528	-0.17445	0.034406	-0.62664	-0.53098	-0.41411	-0.86577	-0.36916	0.106608
O43159	RRP8	-0.11032	0.412914	0.257843	0.745787	0.133805	0.60977	0.353591	0.629835	-0.0707	0.458608	0.450693	-0.00801	-0.11529	-0.27837	0.676804	-0.14783	0.258354	-0.21115	-0.54363
O43164	PJA2	-0.23108	0.411144	0.512578	-0.84449	-0.45891	-0.60775	0.374876	-0.85587	-0.11026	-0.00167	-0.0902	-0.33237	0.1478	0.110163	0.455982	0.058322	0.415692	0.40868	0.781635
O43166	SIPA1L1	-0.24468	0.307741	0.322659	0.029749	-0.82246	-0.97256	-0.15588	0.428109	-0.04998	0.280099	-0.44401	0.008462	0.278246	0.495923	-0.78149	-0.07262	-0.12809	-1.00026	0.421315
O43169	CYB5B	-0.64902	0.381393	-0.06794	0.434477	0.041281	0.229549	0.054098	-0.69264	-0.39062	-0.20656	0.798355	-0.32885	-0.41001	-0.10544	0.419969	0.407505	1.452326	1.268605	-0.06532
O43172	PRPF4	0.323927	-0.20559	0.198817	0.140363	0.314211	0.21778	-0.04868	0.398971	0.428098	-0.25075	0.052441	-0.07962	-0.00942	-0.09907	0.337324	0.36959	0.14349	0.675244	0.089982
O43175	PHGDH	1.511208	-0.18928	-0.20727	0.170472	-0.01825	0.021412	0.183833	0.923292	-0.63802	-0.86188	-1.17209	-0.59184	-0.35782	-0.63193	-0.67762	0.845638	0.011645	-1.44363	-0.17172
O43181	NDUFS4	-0.09746	0.028326	0.182981	-0.80968	1.137533	0.301486	-0.06217	-0.05204	-0.04594	-0.83179	0.448314	-0.12825	0.567669	-0.1053	0.372563	-1.04233	-0.33453	-0.23938	-0.87614
O43182	ARHGAP6	-0.59124	-0.18269	-0.01788	-1.03278	-0.12894	-1.43665	0.344193	-0.41702	0.163418	-0.28277	-0.44561	-1.21284	-0.61666	-0.3983	0.262308	-0.85544	-0.22834	0.502192	-0.13934
O43189	PHF1	-0.08306	0.281503	0.44432	0.100234	0.389685	0.289484	0.077299	-0.32074	-0.0745	0.172123	-0.22859	-0.00576	0.232277	0.199049	0.242196	0.212305	-0.00795	0.461524	-0.51366
O43236	SEPT4	-0.44446	-0.44344	-0.21362	-1.54277	0.558187	-3.42408	0.158162	-1.63388	0.437772	-0.02468	-1.64124	-2.28562	-0.73613	-0.87138	0.786948	-0.79583	-1.12957	0.737856	-0.06338
O43237	DYNC1LI2	-0.31046	0.056949	-0.52732	0.137988	-0.23778	-0.03645	-0.06658	-0.40259	0.292934	-0.23065	0.281304	-0.29291	0.724849	-0.31706	0.184571	0.651813	-0.04142	0.095743	0.452244
O43242	PSMD3	-0.24553	0.262805	0.458355	0.119071	-0.1298	0.082546	0.467964	-0.03577	-0.35279	0.006952	0.080727	0.268606	-0.10495	0.208748	-0.22664	0.163014	0.192557	-0.17792	0.464918
O43252	PAPSS1	0.117253	0.543516	0.922858	-0.3192	-0.53446	-0.34109	-0.54351	0.866266	0.38146	1.092185	-0.10221	-0.04643	0.04618	-0.01582	-0.01643	-0.50247	-0.14145	-0.38544	0.739543
O43264	ZW10	0.308027	0.489675	0.059628	0.404958	0.349546	0.373518	0.367456	0.129941	0.893145	0.206078	-0.15053	0.163238	0.057003	0.056735	0.005483	0.27881	-0.00671	-0.0597	0.234585
O43278	SPINT1	1.75064	-0.60994	-0.07105	-0.07374	-1.04722	-0.54882	-0.65856	0.064077	0.112792	-0.42073	0.344713	-1.241	-0.86373	-1.49523	-0.51026	1.00231	-0.59774	-0.41705	-1.0207
O43290	SART1	0.438487	-0.11766	-0.00926	0.127776	0.023932	0.274313	-0.37052	0.419327	0.296875	-0.14195	0.088606	-0.30505	0.133656	-0.41915	0.16977	0.285728	-0.13524	0.468295	-0.02738
O43291	SPINT2	0.594299	-0.10814	0.479902	0.847251	1.623339	2.18591	0.315781	1.598565	-0.0147	-1.59299	-0.36032	0.382466	0.145045	0.289794	0.852465	-0.36147	-0.61489	-1.10117	2.836974
O43292	GPAA1	0.622349	-0.07223	-0.59674	0.225254	0.153954	0.320617	0.141131	0.524177	-0.07621	0.017626	-0.00063	-0.12143	-0.38347	0.085885	0.326158	0.424792	0.383021	0.275043	-0.00917
O43293	DAPK3	-0.66338	0.290459	0.382042	-0.62665	-0.57303	-0.56044	0.013661	-0.09975	0.891694	0.045015	-0.49849	-0.74837	0.290391	-0.80451	-0.3032	0.009044	-0.65759	-0.05914	1.112528
O43294	TGFB1I1	-1.13854	-0.72531	-0.91934	-1.25512	-1.20971	-1.03484	-0.28264	-1.23114	0.688274	-0.66875	-1.6177	-1.63242	-0.23302	-1.1616	-0.79362	0.178095	-1.75741	-0.04186	1.140453
O43295	SRGAP3	0.770781	-0.19441	0.235927	-0.59567	0.257389	0.464724	-0.08304	0.674178	-0.09257	-1.20175	0.436075	-0.77824	0.132591	-0.31025	0.519277	-0.09658	-0.6552	-0.86993	-0.18602
O43299	AP5Z1	-0.5546	0.974069	0.283551	-0.1562	0.023006	-0.18958	0.561151	-0.17758	0.131222	0.574023	-0.21904	0.037381	-0.29858	0.324846	-0.41734	-0.42622	-0.13067	0.308802	0.215628
O43301	HSPA12A	-1.85727	-0.82035	-0.53535	-2.07995	-0.95716	-1.0785	-0.44495	-1.08785	0.192242	-0.6143	-0.80909	-0.57966	-0.35817	-1.12731	-0.1996	-0.77614	-0.72177	-0.78155	-0.33751
O43306	ADCY6	0.297269	-0.75134	-0.1681	0.166274	-0.08237	0.002287	0.534567	-0.60444	-0.86985	-1.69004	0.34536	-0.26149	-0.82232	-0.31732	0.643459	-0.57236	-0.07691	0.183271	-0.53686
O43310	CTIF	-0.79434	0.170428	-0.15443	-0.38817	-0.67418	-1.41381	-0.13899	-0.55635	-0.19457	-0.25994	0.644266	0.023514	0.108073	-0.21623	0.403688	0.614951	-0.13499	-0.33946	0.051335
O43312	MTSS1	-1.23452	0.909165	0.845163	-0.48346	0.620029	0.096266	0.19621	-1.04258	-0.05203	1.340603	-0.24814	0.890292	0.137954	1.321494	-0.37102	0.354774	0.052447	0.573101	-0.58479
O43314	PPIP5K2	-0.30665	0.104927	0.253207	-0.16775	-0.16496	-0.34372	0.541172	-0.47499	-0.34119	0.2803	-0.49584	0.608642	-0.08301	0.939775	0.673096	0.40864	0.332585	0.261494	-0.04192
O43318	MAP3K7	0.39621	0.231193	0.158003	-0.18214	-0.22046	-0.24876	-0.24791	-0.59947	-0.04677	0.654665	-0.15082	-0.10869	0.211585	0.361011	0.232399	-0.24297	0.507006	0.074982	0.331314
O43324	EEF1E1	0.17573	-0.30948	-0.07878	0.13511	-0.00252	0.043729	-1.1123	-0.45659	-0.28534	-0.23814	0.362342	-0.52977	-0.51705	-0.02469	0.643862	1.450727	0.28686	-0.16077	0.003507
O43347	MSI1	1.12571	-0.70695	-0.99909	-0.88952	-0.14234	0.248357	-0.30598	0.952047	0.218742	-1.44567	-0.73759	-0.51381	0.094532	-0.48573	-1.06801	-0.89059	-0.66931	-1.22295	1.328475
O43353	RIPK2	-0.3756	-0.24644	0.023498	-0.7788	-0.68573	-0.29908	0.023319	-0.27775	-0.58236	0.010643	-0.92183	0.396433	1.124683	0.32139	-1.27692	0.106427	1.575021	0.068187	0.371089
O43390	HNRNPR	0.722438	-0.19671	-0.08509	-0.23607	0.088191	0.054049	-0.60777	0.820955	-0.42207	-0.24745	0.452103	-0.36175	-0.36437	-0.37327	-0.02818	-0.44937	-0.06025	0.305974	-0.2514
O43395	PRPF3	0.420942	-0.05334	0.089641	0.225294	-0.11586	0.429103	0.043845	0.34139	-0.38929	-0.32679	0.237176	-0.32845	-0.36476	-0.48959	0.269744	0.087327	0.179387	0.529733	-0.18274
O43396	TXNL1	-0.43885	-1.01726	0.147537	0.437583	1.335832	-0.3354	-0.73908	-0.41497	1.438272	1.959434	0.295041	0.145185	-0.18209	0.237665	-0.7799	0.149762	0.375195	0.330896	-0.05095
O43399	TPD52L2	-0.35568	0.480547	-0.65946	-0.94125	0.604266	-0.03362	-0.64124	-0.01232	0.12857	0.111332	-0.35784	0.475358	0.984054	-0.17525	-0.10265	0.192175	0.040376	-0.19337	-0.70983

O43402	EMC8	-0.09587	0.51265	-0.07863	0.18246	0.325221	0.236087	-0.17963	-0.06654	-0.14109	-0.03958	0.341671	0.222699	-0.29971	0.024708	0.163475	0.821126	0.063924	-0.0437	-0.05521
O43414	ERI3	-0.33601	-0.80791	-0.04807	-0.02423	1.535224	0.026395	-0.23035	0.026149	1.098731	1.985731	0.095388	0.342196	0.299588	0.271735	-0.19191	-0.6727	0.364544	-0.10757	-0.31851
O43422	THAP12	0.746271	-0.21639	-0.37357	0.384457	0.829135	0.596149	-0.99377	0.365373	-0.07352	0.006327	-0.25193	-0.19662	0.290658	-0.15071	0.666705	0.298003	-0.07927	-0.2172	-0.49146
O43426	SYNJ1	-1.13121	0.859107	0.923643	-0.43348	0.228625	0.108557	0.224408	-0.88131	-0.21077	-0.07147	-0.67543	0.734357	0.167159	0.992653	0.314857	0.388369	-0.12402	0.428048	-0.20247
O43427	FIBP	0.384206	0.276435	0.491969	-0.29887	-0.17437	-0.25317	-0.4048	0.432249	0.017794	-0.0961	0.076738	-0.18072	-0.67812	0.166703	0.263847	0.124349	-0.32483	-0.40444	-0.0274
O43432	EIF4G3	-0.03219	0.118874	0.637231	-0.45476	-0.27267	-0.41625	0.278584	0.363715	0.292903	0.98446	-0.2222	-0.28687	0.004953	0.002889	0.207642	-0.10515	-0.15672	0.3098	0.802962
O43447	PPIH	0.435351	-1.00268	-0.39598	0.72108	1.545539	0.551427	-0.80497	0.321643	0.055634	0.884254	0.200854	0.468163	0.046967	0.543509	-0.01869	-0.10674	0.770225	0.247377	-0.58419
O43464	HTRA2	0.258312	-0.15558	-0.18008	0.350332	0.445634	0.5447	-0.44412	-0.29493	0.350011	-0.20869	0.29391	-0.30986	0.201077	-0.67361	-0.45677	1.66057	-0.93321	-0.42723	-0.85282
O43488	AKR7A2	0.674238	-0.7118	-0.26183	-0.32947	0.508292	-0.12198	-0.90052	0.546871	0.039547	1.505623	0.426284	-0.45098	0.722121	-0.04418	0.924811	0.240945	-0.4148	0.287355	0.603791
O43491	EPB41L2	0.30863	-0.3232	-0.3367	-0.95194	-0.27484	0.145707	-0.79688	-1.74025	-0.42265	-0.67261	1.847722	-0.61331	-0.84198	-0.83804	0.139841	-0.67782	-1.09887	-0.20022	-0.59044
O43493	TGOLN2	-0.93242	0.223679	1.097386	0.26417	-0.37602	-0.1019	1.002315	0.602723	-0.01265	0.274337	-0.08806	-1.4964	0.840926	-1.39036	1.069247	0.458976	0.217509	1.250729	0.565033
O43505	B4GAT1	0.292711	-0.83739	-0.23456	0.135493	-0.10125	0.559857	-0.43669	-0.42782	-0.15913	0.380198	0.18339	-0.87171	-0.46221	-0.90963	-0.97159	-0.70454	0.540923	0.370351	-0.27755
O43516	WIPF1	-0.73888	-0.09261	0.249572	-0.70292	0.032573	-1.95904	0.330442	-2.01142	0.555507	1.197593	-0.80724	0.124455	-0.14362	-0.03303	-0.40807	-0.42755	-0.20679	0.362564	-0.23176
O43520	ATP8B1	-0.64405	-0.53249	-1.33118	0.158199	-1.32082	-1.20969	-0.87416	-0.84365	0.023092	-0.24378	2.132781	-0.72007	-0.67528	-0.7924	-1.84274	-0.19942	-0.58827	-1.12291	-1.50966
O43521	BCL2L11	-0.09533	0.615234	0.396818	-0.60583	0.149617	0.657474	0.602163	1.05898	-0.00087	-1.04098	-0.61523	-0.39724	-0.02068	-0.11516	0.791999	-0.47898	0.122708	-0.83118	-0.22814
O43524	FOXO3	-0.15896	-0.13208	-0.08181	0.020322	-0.05021	0.239202	0.077817	-0.60033	0.065237	-0.42053	-0.4462	-0.0804	-0.00376	-0.01883	-0.06017	-0.7548	0.29641	-0.14669	0.524297
O43556	SGCE	-1.50747	-0.38953	-0.3775	-1.02615	-0.69502	-1.37214	-0.10887	-1.1015	0.326095	-1.63805	-0.60822	-1.19691	-1.42567	-1.44111	-0.52532	-0.22545	-0.99155	0.154489	0.397461
O43567	RNF13	1.242821	0.273528	-0.09319	-0.22374	0.035492	-0.58006	0.379208	-0.39425	0.237762	0.391053	-0.12806	0.369771	0.099548	0.834292	-0.08975	-0.214	0.071688	-0.18175	-0.36323
O43572	AKAP10	-0.32679	0.512642	0.02599	-0.39168	-0.19581	-1.40943	-0.07509	-0.12623	-0.27348	0.017862	0.333579	0.45039	0.146701	0.576774	0.34305	-0.42423	0.097806	0.115101	-0.18572
O43583	DENR	0.103713	-0.27321	0.074654	0.505601	0.305203	0.183619	-0.75234	0.594241	-0.26112	-0.10204	0.449381	-0.9109	0.440059	-0.65955	0.039002	-0.37938	-0.05395	0.514938	0.337084
O43586	PSTPIP1	-1.75051	1.427843	1.46771	-0.82925	0.513285	0.135075	0.431404	-1.16974	-0.06407	1.293432	-1.51009	1.34662	0.023525	2.366039	-0.99403	-0.31514	0.823089	0.446781	-1.12234
O43592	XPOT	0.221872	-0.08493	0.027146	-0.10011	0.323609	0.605222	0.094255	0.28135	0.41445	0.293934	-0.45191	-0.33268	-0.05646	-0.05875	0.102965	-0.04645	0.079603	-0.53739	0.901706
O43598	DNPH1	-0.45834	0.106724	-0.96691	-0.4577	0.572364	-0.10296	-0.11863	0.528148	1.098934	0.533223	-0.58697	0.363689	0.275068	-0.08819	-0.33434	0.384168	-0.29762	-0.41321	-1.09799
O43615	TIMM44	-0.1602	0.167188	0.005696	-0.12678	-0.16363	-0.24327	-0.07475	-0.47702	-0.02707	0.832879	-0.00135	0.01455	-0.14689	-0.3477	0.238807	2.020434	-0.42909	-0.44049	0.118624
O43617	TRAPPC3	-0.46367	0.259225	-0.33758	0.190599	0.371731	0.437961	0.572979	0.349303	-0.06845	0.066322	-0.05065	0.038375	-0.27744	0.387832	-0.20393	-0.33446	-0.00695	-0.03874	-0.15704
O43633	CHMP2A	0.283488	0.08664	-0.83897	-0.04416	-0.00957	-0.12941	-0.49777	-0.28278	-0.46083	0.009949	0.31039	-0.2405	-0.57548	0.221289	0.945675	0.301502	0.032069	-0.14251	-0.96424
O43639	NCK2	0.333086	-0.50004	-0.135	-0.40354	-0.1353	-0.18396	-0.3689	0.551071	0.564264	1.118235	-0.02745	-0.66383	0.34195	-0.11714	0.220445	-0.08211	-0.06719	0.804981	0.721341
O43657	TSPAN6	0.2944	-0.33372	-0.53751	0.959639	-0.47854	-0.24774	0.135452	0.898753	0.082817	-1.91256	0.340122	-0.5928	-0.05655	-0.66318	-1.56237	-0.62812	-0.06782	-0.08385	0.070239
O43660	PLRG1	0.39612	0.000704	-0.42032	0.285698	0.131587	0.455442	0.23157	0.538083	-0.85521	-0.45019	0.507769	-0.3786	0.042313	-0.77945	-0.00664	0.244563	-0.23537	0.578287	0.027303
O43663	PRC1	0.789262	-0.65792	0.197497	0.370045	2.21518	2.032211	-0.58697	1.346729	0.034307	0.310514	-0.26981	-0.45908	0.072568	-0.42881	-1.45927	-0.6202	0.402813	-0.052	0.031003
O43665	RGS10	-0.07476	-0.1718	0.691943	-0.40587	0.987437	0.340977	-0.07063	0.15539	-0.10569	0.122271	-0.6867	0.331753	0.488919	-0.22409	1.952084	-0.05985	0.130594	0.012498	-1.46982
O43670	ZNF207	-0.1155	-0.51883	0.38548	-0.09364	0.143835	-0.3281	-0.10653	0.437036	-0.31522	-0.23844	0.160394	-0.04898	0.041625	-0.09339	-0.3913	-0.02237	0.272455	0.292832	-0.19192
O43674	NDUFB5	0.058339	0.28481	0.644282	0.085239	0.574898	0.239042	0.450484	-0.2682	-0.80895	-0.14813	0.669209	-0.21469	0.02218	-0.03855	1.197376	-0.14318	-0.72694	-0.05193	-0.25786
O43676	NDUFB3	0.340304	0.069435	-0.00163	-0.28794	0.609486	0.588845	0.194681	-0.5865	-0.77825	-1.18845	0.959548	0.029394	-0.06673	-0.25098	0.735135	-0.59264	-0.73483	0.15202	-0.95117
O43678	NDUFA2	0.103375	-0.075	-0.8443	-0.93456	0.92146	0.023946	0.243423	-0.6039	-0.27916	-0.79361	0.368768	0.046396	0.037849	-0.14743	0.51182	-0.74593	0.094239	-0.34381	-0.66549
O43681	ASNA1	-0.98785	-0.17894	-0.22128	0.463747	-0.21629	-0.06491	-0.33806	0.855649	0.061467	0.010135	-0.21218	0.135132	-0.28241	0.123179	-0.70255	0.621078	0.285449	-0.32464	-0.15987
O43684	BUB3	0.257802	-0.28568	0.203764	0.395131	0.008974	0.137094	-0.11139	0.606837	-0.07562	-0.16827	0.347083	-0.16936	-0.41523	-0.40831	0.019774	0.232707	0.101663	0.359324	-0.12564
O43707	ACTN4	0.024248	-0.76425	0.014363	0.281498	-0.18853	0.369901	-1.18987	0.918384	0.081419	-0.71359	0.280241	-0.80122	0.450113	-0.72696	-0.26586	-0.56632	-1.06813	-0.60704	1.885493
O43708	GSTZ1	0.427851	-0.92571	-0.10644	0.373151	1.22522	-0.61933	-0.39203	0.09286	0.831659	2.143219	-0.13418	1.133727	0.301793	0.664334	-0.21904	1.452539	1.047134	-0.57235	0.100908
O43709	BUD23	0.620215	-0.30934	0.161296	0.629457	0.159954	1.120953	-0.46774	0.961709	-0.19156	0.207995	0.313679	-0.64869	-0.22645	-0.69877	0.515368	-0.21039	-0.13519	-0.10509	-0.16883
O43716	GATC	-0.37727	-0.08369	0.038034	1.086907	-0.12834	0.70799	-0.5126	-0.21487	-0.29179	0.040139	0.325351	-0.65488	-0.03923	0.284889	0.703768	0.229668	0.445273	1.003861	0.017905
O43719	HTATSF1	0.401522	-0.64357	0.096046	1.127142	0.20937	0.447395	-0.71408	0.631066	-0.40644	0.145248	0.541103	-0.6809	-0.60452	-0.18756	0.324964	-0.11866	-0.05201	-0.13898	-0.0395
O43731	KDELR3	-1.62569	0.22799	0.317989	-0.11948	-0.69283	0.664538	0.370174	0.364385	0.446196	0.144091	0.235002	-0.53468	0.800849	-0.54385	-0.51661	0.376595	1.1918	2.203762	2.078833
O43739	CYTH3	-0.18161	0.370464	0.281224	-0.46968	0.523121	-0.35084	0.060396	0.962496	0.209595	0.908004	-0.10928	-0.94992	0.140715	0.222048	0.149173	0.613098	0.340749	0.430328	0.415302
O43741	PRKAB2	-0.12477	0.141236	-0.02352	-0.8545	-0.53214	-0.2634	-0.02989	-0.16933	0.023052	-0.10947	-0.08795	0.207593	0.02302	-0.13248	0.291199	0.080497	0.007121	-1.1276	0.60415
O43747	AP1G1	-0.56386	0.49196	0.60415	-0.21363	-0.05145	-0.00179	0.62331	-0.3067	-0.00823	0.135784	0.311896	-0.00416	0.424932	0.093097	-0.12869	0.418599	-0.32615	-0.46247	0.174811
O43752	STX6	0.123962	0.465984	0.109158	0.535629	0.037057	-0.07994	0.290859	-0.0222	0.679064	0.660727	-0.14917	0.616351	-0.07354	0.385719	0.197995	-0.02317	0.028232	0.06161	-0.50925
O43760	SYNGR2	0.015124	0.419754	-0.20818	0.049337	0.392159	0.079254	0.076296	-1.08574	-0.75909	-0.18417	0.085758	0.47194	-0.41139	0.798291	-0.10765	-0.44234	0.014138	-0.62867	-1.63038
O43765	SGTA	-0.10353	-1.3096	-0.14312	0.090331	0.483518	0.670455	-0.02427	0.593205	0.198373	1.301461	0.45982	0.268814	0.236854	0.139419	-0.95665	-0.20692	0.550038	0.09119	0.055039
O43768	ENSA	0.158786	-0.2014	0.163494	-0.74899	-0.55934	-0.52926	-0.39618	-0.44111	0.180011	-0.50909	-0.92867	-0.05051	-0.0143	0.247541	0.903405	-0.41283	0.398795	0.097852	-0.24421
O43772	SLC25A2(	-0.29566	0.805298	0.388031	-0.28125	0.93764	0.056737	0.46767	-0.62998	-0.03698	0.326085	0.455019	0.853366	-0.81628	0.859263	0.266092	-0.09375	-0.33319	0.136936	-0.36156
O43776	NARS	0.016879	-1.14356	0.080402	0.567324	-0.11574	-0.32204	-1.50882	-0.17333	0.214883	0.148646	0.51779	-0.18885	0.024138	-0.33616	-0.15257	1.111207	0.459819	-0.06184	-0.34684
O43795	MYO1B	-0.01494	0.997265	0.451172	-0.43459	0.333685	-0.49321	0.251761	-0.28493	-0.10298	0.94622	-1.55899	-0.35469	-0.10501	-0.97583	-0.81413	0.078413	-0.60559	0.479964	1.435014
O43805	SSNA1	1.02304	-0.27886	0.000405	-0.81938	0.725971	0.515434	-0.2768	0.773063	-0.02989	0.239494	0.52496	-0.0197	-0.34997	-0.25758	-0.09594	-0.51884	-0.20658	0.311413	-0.30019
O43809	NUDT21	0.26307	-0.39912	-0.77689	0.276389	1.19209	0.054875	-1.31848	0.237856	1.12293	0.787325	0.303813	0.398501	0.062577	-0.20645	-0.62388	-0.66378	0.026566	0.299254	-1.03659
O43813	LANCL1	-0.13568	-0.27286	-0.60534	0.548979	-0.25931	-0.08422	-0.58198	0.079673	-0.8615	-0.75632	0.416212	-1.00039	-0.32627	-0.24592	0.470195	0.427137	-0.15731	-0.19992	-0.07761
O43815	STRN	-0.2944	0.249225	0.456171	-0.07466	-0.04282	0.415378	0.527421	-0.03531	0.101557	0.279427	-0.54827	-0.16669	0.366207	0.23537	-0.11717	0.315119	-0.33374	-0.05234	0.26046
O43818	RRP9	0.36221	-0.43211	-0.50412	0.064281	0.292925	0.557759	-0.90049	0.431088	-0.69145	-0.22425	1.160495	-0.65021	-0.2964	-1.05044	-0.17788	-0.55469	-0.33205	0.117144	-0.08887
O43819	SCO2	0.137303	0.371378	-0.163	0.180987	0.362969	0.338163	0.166711	-0.16168	-0.2688	-1.13554	0.439042	-0.56987	-0.38995	-0.92451	0.864422	-0.25379	-0.08553	1.050843	-0.17562
O43822	C21orf2	-0.63634	0.063612	0.028734	-0.50036	-0.62745	-0.42602	-0.33523	-0.21782	-0.00342	-0.80793	0.424956	-0.5747	-0.23131	-0.24275	-0.43354	-1.22379	-0.54034	0.328135	0.298607
O43823	AKAP8	0.405164	-0.34002	0.550429	0.120857	-0.20871	-0.01115	-0.09322	1.146891	-0.35637	-0.65498	-0.29535	-0.17789	-0.62743	-0.06337	0.901617	-0.05069	0.502745	1.037233	-0.52096
O43824	GTPBP6	-0.57338	-0.07564	0.207259	0.077854	-0.13223	-0.36918	0.1586	-0.3299	0.127597	0.332576	0.1909	-0.12829	-0.49423	-0.22535	0.268038	0.030338	0.000888	0.354202	-0.52644
O43826	SLC37A4	0.666783	-0.38668	-0.00383	-0.43653	0.440365	0.042251	-0.04477	-0.25523	0.305863	-0.83116	-0.64975	0.760647	-0.016	-0.21087	-0.4485	-0.3261	0.209881	-0.30094	-0.44117
O43829	ZBTB14	0.682063	-0.22993	-0.00747	-0.05072	0.428992	0.376808	-0.3931	0.684996	-0.1121	-0.39716	0.139328	-1.06335	-0.13851	-0.42209	0.352545	-0.70898	-0.36762	0.417662	-0.08747
O43837	IDH3B	-0.17617	-0.24414	0.493618	0.9201	0.543405	0.496733	-0.66435	-0.02698	0.740344	-0.05309	0.274528	0.152883	0.103556	0.040797	0.745602	0.401867	-0.0015	0.106958	-0.55212
O43847	NRDC	0.36486	-0.33287	0.207686	-0.00496	0.272282	0.386241	-0.67807	0.246101	0.700076	0.502749	-0.63375	-0.37748	0.11092	-0.22282	0.085727	0.566052	-0.01852	-0.39329	0.266442
O43852	CALU	0.100081	-1.19853	-0.05406	-0.07034	0.636948	0.128356	-0.75374	0.801307	-0.31376	0.475065	-0.34688	0.104436	0.817856	-0.05531	1.089193	0.638896	0.429477	0.360546	-0.08233
O43861	ATP9B	-0.3294	0.210111	-0.04009	-0.33472	0.154284	-0.03289	0.109964	-0.85602	0.034447	0.173766	0.02415	0.764559	-0.23722	0.023708	-0.02151	-0.01122	-0.12196	-0.09864	0.528713
O43865	AHCYL1	0.344568	-0.41969	0.089781	0.373746	0.679258	0.806669	-0.65683	0.245512	-0.1317	0.094242	0.564561	-0.14156	-0.00217	0.00546	-0.42125	0.137489	-0.24054	-0.45164	-0.49002
O43896	KIF1C	0.566969	0.690339	-0.00828	-0.88515	-0.46105	-0.48307	-0.06964	0.311131	0.040847	-0.14728	-0.18301	0.00929	1.457693	-0.03812	0.472494	-0.38621	-0.41003	-0.51501	-0.32187
O43909	EXTL3	-0.48317	0.080611	-0.23492	-0.39251	-0.48398	-0.57453	-0.1578	-0.07808	-0.31249	-1.18893	-0.15872	-0.08198	0.121365	0.108927	-0.56397	-0.80452	1.049378	0.850794	-0.17793
O43913	ORC5	1.07261	-0.05261	-0.32554	-0.61835	-0.3986	-0.10193	-0.06516	0.49144	0.258343	-0.90752	0.183211	-0.67069	-1.06175	-0.36846	0.286824	0.016114	-0.85503	-0.33615	-0.22666
O43914	TYROBP	-1.66718	0.969209	0.675751	-0.0046	0.285314	-0.33543	0.017088	-1.07244	-0.1015	1.121078	-0.86404	0.28269	0.08822	0.901039	-0.64595	-0.4106	0.283151	-0.54833	-0.70179
O43920	NDUFS5	0.27348	0.005098	0.274314	-0.6254	0.964028	0.343629	0.041213	0.095396	-0.09676	-0.73676	1.12422	-0.15995	0.078083	0.072074	1.2297	0.131791	-0.84654	-0.08373	-0.81524
O43924	PDE6D	0.38658	0.192373	0.18265	-0.58114	-0.26473	-0.36198	-0.03935	-0.06039	0.408422	0.294414	0.01988	0.328697	-0.07012	1.095097	0.236623	-0.0876	0.040172	-0.41019	-0.05004
O43929	ORC4	0.76789	-0.05952	-0.3898	-0.67352	-0.26668	0.251699	0.398866	0.409846	-0.37608	-0.38061	0.366209	-0.39194	0.037535	-0.33107	0.249575	0.134188	0.015363	-0.14363	-0.10579
O43933	PEX1	-0.18641	-0.03282	0.049712	0.423812	0.545617	0.32412	-0.13727	-0.06174	0.045219	0.24825	0.669254	0.0185	-0.11691	0.027484	0.820133	0.836398	-0.6206	-0.48261	-1.16482
O60216	RAD21	0.528934	-0.36294	0.090448	0.023873	0.521237	0.4766	-0.07594	-0.28896	0.074728	-0.08517	0.11428	-0.61994	-0.08937	-0.26155	0.522951	-0.35851	-0.32408	0.473781	-0.34486
O60220	TIMM8A	0.386527	-0.34263	0.15863	1.115871	0.062247	-0.06357	-0.23154	-0.19029	-0.27471	0.013559	0.314648	-1.0885	-0.28837	-0.55682	0.551489	1.833273	-0.84135	-0.56854	-1.24061
O60229	KALRN	-0.03626	0.371112	0.286592	-0.53404	0.069734	-0.82195	0.073401	0.130253	-0.38589	0.06623	0.625656	0.307401	-0.10442	-0.06393	-0.25694	-0.05985	-0.22706	-0.19066	0.247712
O60231	DHX16	0.318529	-0.10332	-0.09378	0.278596	0.528498	0.717287	-0.46945	-0.25628	-0.16986	0.034093	0.044961	-0.10064	0.06395	-0.30081	0.28449	0.611383	0.286353	0.202145	-0.14373
O60234	GMFG	-1.15015	0.825208	1.104469	-1.18527	0.662857	-0.39369	-0.12818	-1.60177	0.829677	1.887051	-1.35173	0.81152	0.02413	2.318218	-0.878	-0.6228	0.226946	0.365235	-1.03998
O60237	PPP1R12E	-1.15173	0.021621	0.361973	-1.72908	-0.86632	-1.5286	0.115586	-1.7316	0.841345	-1.14631	-1.25942	-0.92513	-0.68987	-1.41866	-0.01382	-0.88369	-1.9214	0.774137	0.274359
O60238	BNIP3L	0.121646	0.119737	0.018981	-0.79548	-0.31505	-1.4704	-0.26246	-0.09342	-0.30523	0.09723	-0.38322	0.076106	0.079021	0.283958	0.012121	-0.39934	0.36144	-0.12156	-0.23414
O60240	PLIN1	-3.2493	-0.79584	-1.19576	-2.11627	-4.05731	-2.57637	0.534975	-3.64176	-0.66482	-1.59502	-2.68652	-1.93307	-1.08481	-1.77445	-1.92904	-1.31292	-2.09491	0.377194	-4.13094
O60244	MED14	0.230099	-0.04336	0.105685	0.125925	0.140763	0.004835	-0.19576	0.521669	-0.10006	-0.21655	0.176811	0.03147	0.001184	0.129642	0.481847	0.22654	0.062461	0.191652	-0.01512
O60245	PCDH7	0.309929	-0.3756	0.25913	-0.24828	-1.22653	-0.81114	0.231896	-0.62143	0.555451	-0.77962	-0.18993	-0.65377	0.662928	-0.40128	0.966662	0.510034	-0.91098	-0.27084	0.375879
O60256	PRPSAP2	0.254532	-0.75926	0.057809	-0.36164	0.0288	0.008947	-0.88137	-0.12703	-0.00602	-0.04329	0.691652	-0.13093	-0.53902	0.185006	-0.34659	-0.27725	0.013465	0.002883	0.1687
O60264	SMARCA5	0.71274	-0.08653	-0.06688	0.153068	0.518618	1.01531	0.040274	0.459608	-0.5192	-0.01047	-0.13941	-0.3164	-0.34546	-0.19675	0.143761	0.015395	-0.11986	-0.0229	-0.17251
O60268	KIAA0513	-1.28095	0.650695	0.577891	-0.82675	0.354058	-0.58142	0.37928	-0.45366	0.460531	0.480413	-0.86818	0.806786	-0.07672	1.477044	-0.71529	-0.77574	1.091673	2.075887	0.194728
O60271	SPAG9	-1.05659	-0.00073	0.267834	-0.06718	-0.3938	-0.1338	-0.18748	-0.82422	0.437748	0.312713	-0.36174	-0.27678	0.569044	-0.18812	-0.3808	0.448419	-0.30037	0.289442	0.435247
O60281	ZNF292	0.999805	-0.19302	-0.42673	-0.03105	-0.72001	-0.27132	-0.26338	-0.39083	0.087108	-0.22536	0.762439	-0.00602	-0.13471	-0.02232	-0.12049	-0.02067	0.037378	0.286829	-0.26591
O60282	KIF5C	0.920694	-0.11572	-0.36297	-1.08531	-1.80982	-1.43294	-0.02328	0.807966	0.803187	0.849498	-0.15121	-1.22406	-0.18048	-0.74331	-1.64641	0.451123	-1.20388	-2.54577	0.898609
O60287	URB1	0.041817	0.020208	-0.71159	0.687496	-0.01076	0.13329	-0.21198	0.154246	-0.42151	-0.4276	0.811554	-0.2039	-0.27178	-0.17167	0.142247	-0.2524	-0.56648	-0.60459	0.108365
O60291	MGRN1	-0.08882	-0.14013	0.00911	0.262193	0.057641	0.236734	-0.42228	0.73332	-0.17766	0.105805	0.691859	-0.33342	-0.21093	-0.15873	0.800955	0.076189	-0.12815	0.906783	0.03216
O60292	SIPA1L3	0.24804	-0.06299	-0.02009	-0.15056	-0.54	-0.19309	-0.166	0.926723	-0.26285	-0.04362	0.223693	-0.75059	-0.22596	0.095903	0.382002	-0.03827	-0.40482	-0.62172	0.939241
O60293	ZFC3H1	-0.13282	-0.08094	0.230797	0.316872	0.526725	0.855458	-0.1117	0.26857	-0.41112	-0.14552	0.043331	-0.24069	-0.189	-0.57754	0.854244	-0.43574	-0.03681	-0.25231	-0.06031
O60299	LZTS3	-0.42374	-0.07247	-0.04302	-0.32556	-0.31224	-0.61983	-0.06539	0.519409	-0.26298	-0.14213	-0.83382	-0.38934	-0.16599	-0.64967	0.495518	-0.34813	-0.61904	-1.34854	-0.24641
O60306	AQR	0.28249	-0.2561	0.116822	0.148246	0.239562	0.407892	0.223342	0.279631	-0.6393	-0.26065	0.151683	-0.03754	-0.06026	-0.11009	-0.01005	0.28069	0.071024	-0.27831	-0.03033
O60307	MAST3	-0.56762	0.600989	0.36177	-0.58962	-0.29529	-0.50081	0.219403	-0.95407	-0.14255	0.06805	-0.85418	-0.10364	-0.02235	0.183027	-0.16107	0.382654	0.396802	0.730649	0.10081
O60313	OPA1	0.38803	-0.11609	0.307297	0.457332	0.167741	0.26684	0.141571	0.159847	-0.21122	0.383172	0.003633	-0.32344	-0.08245	-0.44244	2.865891	0.537851	-0.31788	-0.04969	-0.1294
O60315	ZEB2	-0.40143	0.114367	-0.0029	-0.89175	0.171399	-0.33808	0.267617	-1.00703	0.364251	0.146897	-0.60555	0.980204	-0.22432	1.057799	-0.02855	-0.64123	0.196596	-0.1314	0.069122
O60318	MCM3AP	0.100072	0.440592	0.26278	0.166701	-0.09619	0.344169	0.666207	0.172538	-0.23967	0.362421	0.378928	-0.09718	0.024523	-0.04618	0.816958	-0.09627	-0.07379	-0.05357	0.0872
O60331	PIP5K1C	-0.97341	0.675948	0.620121	-0.48474	0.069444	-0.34554	0.413082	-0.78218	0.013825	0.470455	-0.81975	0.321639	0.042851	0.324619	0.333926	-0.16711	-0.37113	0.574711	-0.01827
O60333	KIF1B	-0.3805	-0.26676	0.467365	0.100248	0.29774	0.48138	0.952281	-0.19273	0.345739	0.868134	-0.64368	0.095002	0.39543	0.356249	-0.0402	0.381036	0.498531	0.10784	0.434697
O60341	KDM1A	0.066816	-0.1588	-0.06425	0.163075	0.163141	0.132087	-0.49975	0.485253	0.354708	-0.22477	0.174768	-0.40015	0.033077	-0.24467	0.2574	0.133533	-0.10652	0.257412	-0.17977
O60343	TBC1D4	-0.0821	0.0903	-0.2475	-0.88937	-0.26597	0.021967	-0.59682	-1.377	0.58702	-0.02425	-0.07927	-0.78459	0.297631	-0.23723	-0.29318	0.137156	-0.63198	-0.10273	-0.00722
O60344	ECE2	-0.53897	0.036437	-0.07045	-0.33121	0.164484	0.15256	-0.17048	-0.36378	-0.22207	1.307874	-0.3162	0.104854	0.118412	-0.26014	0.660426	0.199989	1.315424	0.079777	-0.26503
O60353	FZD6	1.178367	-0.71217	-0.7782	0.43803	-0.20126	-0.05639	-0.43529	0.029984	-0.62859	-1.18111	1.026864	-0.48366	-0.44193	-1.40513	0.756621	-1.36542	-0.75384	-0.73163	-1.516
O60437	PPL	0.577362	-1.2422	-0.6384	-1.13563	-2.88454	-1.66232	-0.66152	0.371088	-1.01916	-1.91162	0.004375	-1.391	-0.78721	-1.37666	0.280713	0.318689	-0.39941	-2.01463	-1.66171
O60443	GSDME	0.086291	0.652837	0.328435	-0.74864	-0.28856	-0.23927	-0.55979	0.29369	0.039377	0.28068	-0.13981	0.077181	-0.19294	-0.05365	-0.21474	-0.27019	-0.48677	0.089532	0.262383
O60447	EVI5	-0.50238	0.52102	0.375154	-0.19504	-0.05235	0.053565	0.296999	-0.23553	0.021709	0.548819	-0.22799	0.219978	0.255767	0.251949	0.217034	0.250117	-0.10927	0.495943	1.01239
O60449	LY75	-0.91442	0.411752	0.358889	-0.95713	-0.16051	-0.30444	-0.05192	-0.35685	0.071096	0.031108	1.677903	-0.44399	-0.46637	0.125646	-0.5076	-0.81837	0.084914	0.14455	-0.95957
O60462	NRP2	-1.2725	0.189335	-0.36719	-1.80233	-0.79146	-0.6563	-0.13507	-0.47451	0.473552	-0.61589	0.456619	-0.1311	0.2912	-0.51944	-1.42352	1.070024	-0.61373	-0.10743	0.305407
O60476	MAN1A2	-0.18922	0.406913	0.310228	-0.12087	0.448142	0.838667	-0.26904	-0.53617	0.265844	0.458163	0.487484	-0.15989	0.145886	0.136538	0.574584	0.215196	0.911116	0.889068	0.00624
O60486	PLXNC1	-0.44118	1.264443	0.020571	-1.05658	0.12508	-0.25677	0.548408	-1.2888	0.324141	0.617954	-1.40141	0.295101	-0.49049	0.661138	-0.14406	-0.37782	-0.3289	0.478848	-0.14534
O60487	MPZL2	0.450159	-0.58973	-0.52506	-0.4088	-1.5775	-0.73488	-0.4136	-0.00457	-0.2289	-0.86598	0.63849	-0.56561	-0.13905	-0.51693	0.80281	-0.04063	0.446823	-1.0838	-1.24862
O60488	ACSL4	-0.05778	0.459432	0.258737	-0.13371	0.039019	0.051562	-0.07147	0.615011	0.607426	0.213527	-0.01815	0.944266	0.124178	0.961912	-0.35117	-0.25683	0.382739	-0.73728	-0.32086
O60493	SNX3	-1.11682	1.135511	0.509949	-0.09151	0.573304	0.121118	0.277158	-0.31612	0.502763	0.599701	-0.41639	1.182755	0.525169	0.991282	-0.19311	-0.08754	0.053958	0.538717	0.233551
O60496	DOK2	-0.98663	1.351328	-0.07757	-0.811	1.442412	0.34021	0.39203	-1.00492	0.015841	1.139965	-1.98728	2.319342	0.183335	2.051311	-0.98293	-0.68291	1.009995	0.015821	-0.60113
O60499	STX10	-0.26554	0.492869	-0.24784	-0.25531	-0.18328	-0.13323	0.298853	0.281047	0.419417	0.766072	-0.24118	0.031061	0.330262	0.318999	1.223465	0.857269	-0.02581	0.481203	-0.43649
O60502	MGEA5	0.175238	-0.38866	-0.44822	-0.18393	0.152332	0.322284	-0.09983	0.54189	-0.02464	-0.12974	0.041263	-0.37256	0.316616	-0.21998	1.322136	-0.43171	-0.14218	-0.71003	-0.0164
O60504	SORBS3	0.033862	-0.24962	0.083332	-1.1549	-0.01337	-0.1476	0.297594	-0.27657	0.259117	-0.60643	-0.84657	-0.33559	-0.61357	-0.59746	-0.1741	0.123041	0.171923	0.255727	0.170741

O60506	SYNCRIP	0.794633	0.185336	0.221867	0.224837	-0.1147	0.163151	-0.64283	-0.18948	-0.15477	0.084417	0.368993	-0.12787	0.505505	-0.53378	0.151201	0.123557	0.159508	0.149325	0.145526
O60508	CDC40	-0.04663	0.323575	0.28888	0.702243	-0.06563	0.055364	-0.53875	-0.09928	0.185909	0.288932	0.382667	-0.56218	0.205723	-0.08125	0.312686	-0.289	-0.2113	0.460864	-0.34199
O60518	RANBP6	0.087882	0.671637	0.867196	-0.15026	0.388844	0.463673	0.090792	0.120073	0.479457	-0.39524	0.190343	0.270538	-0.04725	0.992831	0.99027	-0.00087	-0.01896	0.138123	1.026572
O60524	NEMF	0.383801	0.037461	0.242843	0.440585	-0.42377	-0.25379	0.160132	-0.22801	-0.52841	-0.21233	0.448088	-0.15534	-0.41142	-0.06082	0.493863	0.496694	0.138421	0.241473	0.008957
O60547	GMDS	0.135648	-1.79713	-0.34935	0.663003	0.975829	0.365696	-0.59172	-0.0078	0.14588	-0.38481	0.770208	0.833619	0.928906	0.769298	0.405371	1.694145	-0.23976	-0.54202	-0.04316
O60551	NMT2	0.128606	-0.28402	-0.43233	-0.72942	-0.15992	0.110694	-0.08014	-0.37418	0.281252	-0.65708	-0.32632	-1.45407	-0.29507	-1.57663	-0.56368	-0.01815	-0.10579	-0.60528	-0.27047
O60563	CCNT1	0.233996	-0.2473	-0.01742	-0.13227	-0.29573	0.316185	-0.30688	0.08166	-0.24284	0.006338	-0.47562	-0.52546	-0.06195	-0.74477	-0.00224	-0.3236	-0.37624	-0.3629	0.104631
O60566	BUB1B	1.33124	-1.00502	-0.01233	0.030966	0.942445	0.761814	0.17292	0.424059	-0.31847	-0.47121	-0.64224	-1.20963	0.161689	-1.98436	-1.63081	0.09658	1.036719	-0.32413	0.354831
O60568	PLOD3	-0.07485	0.460303	0.55514	0.153848	0.384769	0.356041	-0.03656	0.919487	0.220945	1.434106	-0.50206	0.737353	0.384882	0.305792	-0.59162	0.639495	-0.20188	-0.04217	0.714993
O60573	EIF4E2	0.158812	-0.08506	0.204223	-0.68672	0.791882	0.315081	-0.17641	0.845223	0.171932	0.871078	0.184561	-0.04654	0.505116	-0.22742	0.546011	0.53226	-0.18793	-0.56718	-0.34908
O60583	CCNT2	1.370401	0.941535	-0.01974	-2.1201	-1.63163	-1.27879	0.14846	-0.05951	0.039542	0.054608	-0.2231	-1.20866	0.11555	0.086223	0.177766	0.654054	0.100338	0.358196	0.57854
O60603	TLR2	-0.95161	0.942831	0.958341	-0.33079	0.304985	-0.10138	-0.0718	-0.52044	-0.11362	2.527014	-0.95486	1.69499	0.165645	1.996245	-0.27671	-0.53181	0.786977	0.227457	-0.70802
O60610	DIAPH1	-0.2207	0.09937	0.537081	0.096932	0.057761	0.298463	-0.01902	-0.0376	-0.10826	0.328472	-0.11197	-0.30602	0.111509	0.127612	0.57348	0.11181	0.009967	-0.46966	-0.18324
O60613	SELENOF	0.432309	0.948343	0.217629	-0.29116	-0.43818	-0.17188	0.252565	0.102044	0.347354	0.527371	-0.06194	-0.27844	0.384278	0.089771	0.109853	0.009984	-0.127	-0.04112	0.30486
O60637	TSPAN3	1.750879	-1.15075	-0.97731	-0.36593	-2.165	-0.91899	-0.65131	0.494199	-0.81193	-2.03023	0.135211	-1.38422	-0.32472	-0.74545	-1.23842	-1.62209	-0.45138	-1.69366	-1.4129
O60645	EXOC3	-0.1995	0.353964	-0.48406	0.083152	-0.1958	-0.248	0.153489	-0.31813	0.061855	-0.40782	0.170422	0.053889	-0.14707	0.279523	0.426511	-0.08177	-0.53383	-0.39934	0.220663
O60664	PLIN3	-1.466	0.810302	-0.33885	-0.94086	-0.50143	-0.39472	-0.01929	-0.53388	0.006669	0.427564	0.240025	-0.72188	0.354067	-0.46964	-1.18488	-0.02598	-0.63836	-0.17797	0.506387
O60669	SLC16A7	0.068939	-0.55992	-0.53298	-1.13289	-1.7852	-1.35224	0.021695	-3.13152	0.394309	-0.51101	-0.28309	-0.83435	-0.94514	0.636326	-1.01524	-1.26913	-0.81594	-0.57711	-0.84481
O60671	RAD1	0.090147	-0.08955	0.161243	-0.00929	1.065949	0.334304	-0.17925	0.76705	0.158131	-0.02091	-0.17361	-0.07336	-0.05545	0.546222	0.257509	-0.12282	0.797077	-0.38358	-0.56118
O60674	JAK2	-0.93772	0.218061	0.201993	-0.22448	0.263214	0.063683	-0.08403	-1.37644	0.074948	0.032395	-0.11106	0.10855	-0.08773	0.358319	-0.11174	-0.30101	0.055104	0.076196	-0.22248
O60678	PRMT3	0.30993	-0.64211	-0.89044	-1.18914	0.250303	0.454262	0.000487	-0.46127	0.040847	0.615255	0.278397	-0.40887	0.361233	0.122502	0.225316	1.43866	0.33221	-0.14494	-0.40773
O60684	KPNA6	0.293941	0.342511	0.488389	0.690302	0.179019	0.63371	-0.42302	0.060946	0.562614	0.236259	-0.0036	0.088753	0.237044	-0.17741	0.03065	0.494717	0.660285	0.150909	-0.23729
O60687	SRPX2	-1.24161	-0.0265	-0.8019	-1.39637	-1.86052	-1.54725	-0.15996	-1.2124	0.655328	-1.06526	-0.48726	-1.5163	-0.50303	-0.83026	-0.80735	-0.36711	-0.96057	-1.45652	0.183119
O60701	UGDH	-0.36939	-0.92078	0.910967	-0.38939	-0.00427	-0.0343	0.217945	0.316036	0.767948	0.077659	0.41843	0.352145	1.480418	0.068437	0.339931	0.718637	1.302317	1.043579	1.636968
O60704	TPST2	-0.5729	0.359105	0.085695	-0.18366	0.027005	-0.09565	0.450283	-0.19766	0.105259	0.078278	-0.34528	0.357389	0.314957	0.232734	-0.24299	0.120043	-0.1902	0.441361	-0.40721
O60711	LPXN	-2.54708	1.072541	0.497422	-0.65781	0.581368	0.333694	0.000299	-1.02204	-0.18486	1.665346	-1.36174	0.712817	-0.0443	0.866511	0.209321	-0.48138	-0.07088	0.397286	-1.35567
O60716	CTNND1	0.361487	-0.19612	-0.2833	0.301732	-0.46518	-0.2424	0.261983	0.444595	0.043272	-0.18034	0.409422	-0.132	0.558062	-0.54185	0.650543	0.72004	-0.16063	-0.0457	-0.14212
O60725	ICMT	0.781272	-0.02209	-0.59234	0.105862	0.451058	0.045278	-0.31004	0.522722	-0.0578	-0.15184	-0.01235	0.072295	-0.4978	0.658099	0.423978	0.089166	0.171001	-0.14834	0.355042
O60749	SNX2	-0.92232	0.370571	1.037815	0.204565	0.301135	0.096659	0.230309	-0.17775	-0.32648	0.18412	-0.2076	0.168922	0.213383	0.097824	0.664698	0.599673	0.097805	0.353388	-0.22435
O60762	DPM1	0.040727	0.370496	-0.50389	-0.3578	0.309097	0.082696	0.202106	0.312795	0.156022	0.26986	0.330329	0.178599	-0.59327	0.268922	-0.42498	-0.58788	0.174242	0.455454	0.039746
O60763	USO1	-0.37329	0.499038	0.748313	-0.24689	0.005909	-0.02412	0.700074	-0.42442	0.220816	0.460599	-0.05981	-0.64133	0.53695	-0.34059	0.463451	0.209215	-0.28548	0.433191	0.3788
O60784	TOM1	-0.95032	0.377784	0.018534	-0.25517	-0.44863	-0.71383	0.167918	-0.81062	0.487144	0.786452	-0.3382	0.275555	0.036713	0.455009	-0.5391	0.418128	0.042211	0.521246	0.656102
O60825	PFKFB2	-0.51185	-0.15206	0.495579	0.438445	-0.37473	-0.51661	-0.29469	-0.09894	0.234229	0.643493	0.056699	0.53873	-0.47386	1.067022	1.414003	-1.02978	-0.19032	-0.8422	-0.63402
O60826	CCDC22	-0.33683	0.72085	0.153466	-0.17309	-0.14858	-0.37077	0.564262	-0.0788	0.006427	0.368558	-0.13006	0.278904	-0.22485	0.378857	-0.09989	0.064288	-0.06772	-0.07407	-0.38113
O60828	PQBP1	1.067333	-0.08984	-1.05148	-0.15325	-0.81549	-0.05228	-0.70747	0.3683	-0.08687	-0.34164	0.123028	-1.27509	-0.73575	-0.97885	-0.37769	0.051179	-0.72615	0.105457	-0.25739
O60830	TIMM17B	0.102959	-0.17838	-0.04535	-0.10398	-0.05305	-0.01827	-0.52235	0.192182	0.588741	-0.1533	-0.52898	-0.10908	-0.15094	-0.00574	-0.18302	0.219519	-0.38365	-0.5294	-0.27738
O60831	PRAF2	-0.80758	0.793358	-0.25048	-0.87568	-0.30759	-0.9944	0.017341	-0.25918	1.352003	1.321749	-0.98209	-0.26482	-0.2663	-0.79245	-0.65144	-0.73963	-0.60442	-0.24856	0.508425
O60832	DKC1	0.750348	-1.04568	-0.39544	0.09429	0.004075	0.097771	-1.56174	0.222998	-0.87063	-0.32939	1.052047	-0.6861	-0.52275	-0.86422	0.082759	-0.13358	0.212903	0.668703	-0.44747
O60841	EIF5B	0.668599	-0.27624	0.108728	0.188455	-0.44593	0.150995	-0.77252	0.517749	-0.03969	-0.00104	0.087758	-0.57171	0.239452	-0.36494	0.046683	0.384777	-0.17622	-0.321	0.275772
O60858	TRIM13	0.191597	-0.08482	-0.13986	0.064476	-0.69743	0.202995	-0.1134	-0.26684	-0.2867	-0.49762	0.349885	-0.42762	-0.16574	-0.69963	-0.08625	-0.3882	0.128589	-0.06348	0.084332
O60861	GAS7	-0.66435	0.292509	0.372198	-0.44781	-0.0847	-0.70781	0.575637	-0.66679	0.169323	0.248407	-0.78952	0.640569	0.394174	0.867692	0.032228	-0.19518	-0.30191	0.691672	0.366559
O60869	EDF1	1.076976	-0.15805	-0.00574	-1.5395	-0.8253	-0.48524	-0.16746	-0.69368	-0.18583	-0.22592	-0.22897	-0.79046	0.184142	-0.31633	-0.03805	0.031649	0.15645	0.856243	0.651884
O60870	KIN	0.878075	0.61647	-0.07244	0.443089	0.286216	0.424525	-0.16026	0.27424	0.082359	0.605585	0.180221	-0.09221	0.244265	-0.10462	-0.03719	0.574392	0.681826	0.706428	0.364525
O60879	DIAPH2	0.512921	0.29742	0.240848	-0.51532	-0.06675	0.016804	0.152772	-0.42875	0.14029	0.613446	-0.16026	-0.07047	-0.6549	0.405981	-0.45611	0.096399	-0.277	-0.32849	-0.40964
O60884	DNAJA2	-0.15369	-0.25774	-0.14092	0.666266	0.396568	0.538673	-0.87636	-0.36443	-0.40076	-0.10476	0.243021	-0.23632	-0.1441	0.356764	0.219965	0.6171	0.383682	0.255381	-0.25871
O60885	BRD4	-0.00194	-0.52595	0.196578	0.309404	-0.40373	-0.14651	0.010268	1.393968	-0.43328	-0.26575	0.108839	-0.76334	-0.32881	-0.54438	1.304624	-0.10186	-0.31789	0.069084	-0.28791
O60888	CUTA	0.236431	-0.24861	-0.33623	-0.58308	0.200383	0.214624	0.559257	0.486665	1.295665	-0.25854	-0.30725	-0.21573	0.079792	0.300719	0.179211	0.17897	0.141214	-0.24032	-0.09281
O60890	OPHN1	-0.25334	0.166842	0.12596	0.311489	-0.58388	-0.1487	0.153897	-0.13539	0.498793	-0.05805	-1.2294	-0.71433	0.101706	-0.60394	0.265579	-0.33011	-0.46487	-0.23271	0.375552
O60906	SMPD2	-0.92474	0.30673	0.193079	1.298238	-0.33155	-0.54799	0.219228	-0.43778	0.040847	0.67404	0.868664	-0.4057	-0.00253	-0.29293	-0.04119	-1.15417	-0.04411	0.077608	-0.40557
O60925	PFDN1	0.225494	-0.9058	0.152102	0.310528	0.901615	1.32117	-0.40115	1.052934	0.162554	0.318235	-0.38529	-0.87245	0.31803	-0.03822	0.504059	0.869078	-0.37316	-0.10025	-0.01215
O60927	PPP1R11	-0.4214	0.342808	0.224549	-0.40202	-0.77556	-0.47547	-0.22072	-1.13217	-0.03857	0.021182	-0.27569	-0.55807	0.001171	0.10271	-0.09987	-0.00302	-0.41788	0.416734	0.305048
O60934	NBN	0.183255	-0.38046	-0.28588	-0.25844	-0.49915	0.001749	-1.22115	0.544329	-0.36181	-1.12289	0.590113	-0.58542	0.578865	-0.36129	-0.85575	0.716486	0.136762	0.324691	0.377225
O60941	DTNB	0.046506	-0.22818	-0.34504	-0.31449	0.013345	-0.64663	-0.13521	-0.33905	-0.11096	-0.38928	0.155229	0.127301	0.03667	-0.93524	0.343621	-0.64697	-0.15265	-0.61634	-0.13709
O60942	RNGTT	0.487139	0.177145	0.197253	0.583445	0.152492	0.469361	0.487658	0.208911	-0.42543	-0.74498	-0.24528	-0.03992	-0.14294	-0.45375	0.649284	-1.30914	0.208023	-0.63426	0.108787
O75027	ABCB7	0.029818	0.292651	0.314822	0.525951	0.178845	-0.03696	0.438654	0.496707	-0.34835	0.309756	0.341629	0.134391	-0.5108	-0.07932	0.863488	1.516814	-0.18746	-0.55852	-0.28377
O75044	SRGAP2	-0.46825	0.676919	0.699911	-0.82267	-0.20319	-0.47462	0.629759	1.020625	0.014062	0.511778	-0.88138	-0.00123	-0.31968	0.178745	-0.57865	-0.50578	-0.60955	-0.34528	0.542853
O75051	PLXNA2	-0.86907	0.438875	-0.00828	-0.445	-0.35443	-0.59234	-0.06964	-0.38119	0.040847	-0.88606	0.05737	-0.38794	0.059239	0.152254	-0.04894	-1.11622	-0.23179	-0.24599	-0.43249
O75052	NOS1AP	0.176079	-1.17129	-0.57802	-0.64624	0.014313	0.227337	0.67235	1.0517	0.184589	0.029316	-0.80582	-0.20568	-0.23258	-0.47201	1.837379	-1.43856	-0.59537	-1.05742	0.188196
O75054	IGSF3	0.083536	-0.16602	0.275558	-0.37069	0.00966	-0.03057	0.060242	1.209286	0.313198	0.199407	-0.26688	-0.30456	0.280548	0.068253	-0.54928	0.024797	0.646055	0.511457	0.439249
O75063	FAM20B	-0.24968	0.485817	-0.51407	-0.04102	0.214334	0.357899	0.067262	-0.00063	-0.12037	0.359205	-0.11263	-0.58199	0.074876	-0.39122	1.652253	0.255515	-0.47149	0.555541	0.779422
O75064	DENND4B	-0.20047	0.382997	-0.14031	-0.04314	0.06516	-0.59636	0.762289	-0.37799	-0.2792	0.244778	-0.65602	0.667466	-0.09959	0.942735	-0.2325	-0.48622	-0.20316	-0.55345	-0.86568
O75071	EFCAB14	-0.65832	0.246609	-0.06639	0.126986	0.247389	0.443912	0.266666	0.025929	0.21575	0.334653	0.459527	-0.39909	0.05477	0.116523	-0.15117	0.153486	0.084127	0.093332	-0.26464
O75083	WDR1	0.867425	-0.65525	0.167231	-0.40757	0.237609	0.202247	-1.31016	-0.43256	0.097156	0.50617	-0.59197	-0.17332	0.467566	0.475362	-0.5874	0.418231	-0.72428	-0.45578	0.238465
O75094	SLIT3	-0.37482	0.477969	-0.11584	-0.36798	0.500171	-0.10432	0.399351	-0.09481	-0.22386	-0.6307	-0.1477	0.060466	-0.39481	-0.30219	1.458937	-0.62886	-0.09824	-0.12977	0.501733
O75095	MEGF6	-1.40975	0.020393	-0.54381	-2.70627	-3.06625	-2.07654	0.1925	-1.52125	-0.43499	-1.00252	2.811246	-2.13444	-0.99111	-0.37194	0.352873	-3.78273	-0.84992	-2.1008	-1.11547
O75110	ATP9A	-0.30651	-0.13285	0.102716	0.558286	0.177613	0.203385	-0.09622	0.50658	-0.56029	-0.25525	-0.09004	0.117086	0.23748	-0.04195	0.28515	0.040884	0.431221	0.371134	0.339982
O75112	LDB3	-2.94007	-1.23629	-0.1897	-2.75096	-2.86583	-3.26145	-0.13558	-4.46791	-0.53696	-2.64462	-1.20841	-2.26133	-1.82676	-0.79574	-0.99009	-1.27691	-1.67438	-0.89728	-2.49674
O75113	N4BP1	-0.46825	-0.31931	0.793451	-0.44213	0.012804	-0.11633	0.105223	-0.71271	0.303626	0.054202	-0.13814	-0.21617	-0.12749	0.333811	0.204505	-0.08721	-0.11815	0.012933	-0.23344
O75116	ROCK2	-0.04971	0.069688	0.13992	-0.78173	-0.31875	-0.44741	0.073783	0.115642	-0.18963	-0.07723	0.089794	-1.11538	-0.15487	-0.54688	-0.07121	-0.00146	-0.93845	-0.12228	0.347593
O75122	CLASP2	0.567961	0.38314	0.542998	-0.7987	0.142984	0.269561	0.433242	-0.44219	0.74474	-0.08545	0.025964	-0.00678	-0.09104	-0.24554	-0.14935	0.33079	-0.34047	0.011013	0.130454
O75127	PTCD1	-0.61534	-0.47425	-0.81483	0.789228	-0.39081	0.077597	-0.41704	-0.5508	0.179413	0.535197	0.307928	-0.19049	-1.46846	-0.37114	0.380346	-0.00601	-0.93922	-0.73397	-0.67632
O75131	CPNE3	-0.4686	-0.23206	0.014779	-0.58199	0.301281	0.05312	-0.19347	-0.65659	-0.19165	-0.55726	-0.48555	0.589557	1.300254	0.833917	-0.77904	0.048565	0.914348	0.363441	-0.28759
O75140	DEPDC5	-0.24869	0.336195	-0.45055	-0.15166	0.300473	-0.49441	0.08582	-0.77675	0.461537	0.126077	0.051516	0.066246	-0.88629	0.121821	-0.49322	-0.64364	-0.0992	0.565461	-0.31967
O75143	ATG13	0.292569	-0.11533	-0.33744	-0.31223	-0.17664	0.19326	0.738721	-0.08124	0.59963	-0.63621	-0.96002	0.665314	0.011434	0.282738	0.447001	-0.90085	0.602581	0.07668	0.069734
O75146	HIP1R	0.105022	-0.24106	0.525676	-0.45131	-0.45414	-0.46152	0.37832	0.401266	-0.07005	-1.16198	-0.24604	-1.25521	-0.2763	-0.62305	-1.34123	-1.35141	0.280919	1.496492	0.345705
O75147	OBSL1	1.685645	-0.66117	-0.16363	-0.25171	-0.24049	-0.10564	-0.59914	-0.08592	0.482653	-1.18521	-0.13649	-0.67433	0.271037	-0.43285	-0.21361	-0.71975	0.133378	-0.61908	0.097494
O75150	RNF40	0.405734	-0.20193	0.289503	0.239537	0.442939	0.491164	0.155538	0.222213	-0.16317	-0.03707	0.157599	-0.49317	-0.27108	-0.29084	0.255964	-0.02275	-0.28831	-0.12981	-0.0419
O75151	PHF2	-0.02294	0.239056	0.075287	-0.56893	-0.26082	-0.11838	0.062341	-0.09136	0.446217	-0.70487	0.521864	-0.46391	-0.10178	0.357265	-0.20281	-0.3192	-0.76173	-0.35938	-0.12227
O75152	ZC3H11A	0.997966	-0.55502	-0.42276	-0.32714	-0.2107	0.273	-0.59908	0.430984	-1.07675	-0.26517	0.695313	-0.47186	-0.1814	-0.1867	-0.11013	0.118766	-0.14757	0.166847	-0.64335
O75153	CLUH	1.310919	-0.03456	0.116603	-0.00636	0.516979	0.942835	-0.18055	0.268216	-0.27272	0.107066	0.022229	-0.57473	0.317358	-0.58312	0.316877	0.176014	-0.18296	-0.45606	-0.56465
O75155	CAND2	0.598824	-0.15351	0.328318	-0.02718	1.369967	1.621983	0.175349	-0.02079	-0.18006	0.404263	-0.11062	-0.09022	-0.07691	-0.50353	-0.7218	0.718716	-1.19528	-1.09461	-1.53724
O75157	TSC22D2	-0.13657	-0.13565	-0.32165	-0.15919	-0.83666	-0.31159	-0.7871	-0.22637	-0.63456	0.40796	-0.19229	-0.62516	0.315736	-0.15717	-0.0194	-0.5292	0.248514	0.568811	0.307239
O75164	KDM4A	0.782252	-0.0472	0.184024	0.175233	-0.1801	0.028007	-0.19447	0.725437	-0.25115	-0.09309	0.024798	0.244309	-0.11487	0.230878	-0.37518	-0.26622	0.121463	-0.59049	0.228803
O75165	DNAJC13	-0.27744	0.431199	0.431266	0.214687	0.350477	0.337267	0.414793	-0.17619	0.335249	0.620096	-0.4157	0.562195	-0.20408	0.937249	0.103793	-0.05797	-0.00292	-0.05202	0.258922
O75170	PPP6R2	-0.77099	0.589728	-0.86129	-0.43859	0.068915	-0.16333	0.128696	-0.70901	0.002147	0.25922	-0.06407	-0.02157	-0.21353	-0.32908	-0.08392	0.250985	0.400509	0.237656	-0.03251
O75173	ADAMTS4	-1.35772	0.045619	-0.85273	-0.78474	-1.00013	-0.57346	0.568172	-0.32345	0.781865	-0.46049	-0.79008	-0.10715	-0.07261	0.160398	-0.09365	-0.79019	0.460913	-0.55602	-0.74236
O75175	CNOT3	0.24021	-0.07507	-0.17714	-0.35277	-0.14791	-0.09181	-0.04333	0.1112	0.085577	0.101489	0.309037	-0.1253	-0.16431	0.099213	0.366015	0.495059	0.116299	0.316141	0.246227
O75179	ANKRD17	0.323851	-0.06245	0.295407	-0.2826	-0.29796	0.215586	-0.42118	0.035418	0.07222	0.17193	-0.10551	-0.50283	-0.18381	-0.325	-0.35403	-0.07168	0.227071	0.020844	0.200932
O75182	SIN3B	-0.02841	-0.65792	0.10021	-0.13022	-0.36246	0.044057	-0.02496	0.802457	-0.61429	-0.42849	-0.33785	-0.35924	-0.54145	0.089994	0.062161	-0.25856	-0.154	-0.07443	-0.39222
O75190	DNAJB6	-0.42414	0.061592	0.351567	0.498845	-0.05336	0.329523	-0.15925	0.260447	0.24762	0.068443	0.893924	-0.61625	-0.09365	-0.32889	0.808498	0.083949	-0.64274	-0.10209	0.046232
O75191	XYLB	-0.01289	0.03077	0.150048	-0.25699	-0.05046	-0.82935	-0.21119	-0.36837	0.109483	0.87313	-0.2969	0.335588	-0.48261	0.614774	-0.06073	-0.83937	-0.34918	-0.8683	-0.76298
O75208	COQ9	-0.01308	-0.15628	-0.87463	0.253546	0.161431	-0.36541	-0.38605	-0.65045	-0.41027	0.001807	0.021058	-0.25659	-0.50964	-0.38137	1.257382	0.679971	-0.43021	-0.46168	-0.76805
O75223	GGCT	-1.36286	1.044485	-0.57556	-1.63215	-0.63051	-1.63123	1.894179	-0.36989	2.434157	2.440009	-1.12295	-1.14155	0.008814	-0.77505	-0.73485	-0.56121	-0.478	-1.34119	-0.86496
O75251	NDUFS7	-0.11886	-0.10801	0.325867	-0.11948	0.074991	0.235413	0.02256	-0.51312	-0.78538	-0.85534	0.568322	0.014569	0.316565	-0.1237	0.918937	-0.39651	-0.49478	-0.34739	-0.59773
O75306	NDUFS2	-0.09044	0.110664	-0.03581	-0.03534	0.177882	-0.13174	0.232283	-0.27609	-0.08133	-0.37478	0.445681	0.224342	-0.16797	0.29102	1.125086	0.137457	0.189774	-0.28877	-0.25898
O75312	ZPR1	0.833433	-1.4303	-0.21349	0.197337	0.418744	-0.04648	-0.31362	0.858684	0.006003	1.00331	-0.05994	0.575343	1.26682	0.093714	-0.03097	0.494761	0.648607	0.040597	0.314622
O75323	NIPSNAP2	0.211964	-0.17057	0.422061	0.70275	0.029054	0.347543	-0.17418	0.12461	-0.37154	0.015214	0.470507	0.125957	-0.12281	0.300236	1.312584	1.232746	-0.00209	-0.37131	-0.43662
O75330	HMMR	0.384658	-0.28008	0.468258	-0.72616	2.672651	1.92358	0.268521	0.286118	0.447703	-0.06953	-1.06495	-0.26374	0.007514	-0.55191	-1.35279	0.259135	0.474451	-1.0567	-0.19223
O75339	CILP	-1.61943	-1.70801	-0.63506	-0.00497	-2.20017	-2.07932	-0.88869	-1.17403	1.018311	-1.79711	-1.75131	-1.02967	-0.24707	-0.69496	-1.15874	-0.58262	-1.10926	-2.42282	0.811753
O75340	PDCD6	0.568916	-0.378	-0.44985	0.1262	-0.43772	-0.2385	-1.10468	-0.13631	0.082118	0.863637	0.678765	-0.66437	-0.29522	-0.20823	0.706844	0.119942	-0.18284	-0.23297	-0.36425
O75347	TBCA	0.125417	-1.62823	-1.0928	-0.12416	1.967523	-0.09129	-0.66617	0.821959	1.093554	0.09814	0.013217	-0.16177	0.379667	-0.16305	0.737244	1.155825	0.586874	0.780058	-0.49973
O75348	ATP6V1G	-0.30556	0.400819	-0.05883	-0.42634	-0.14557	0.003211	-0.22359	-1.11342	-0.37924	0.076512	-0.17461	0.426798	0.060694	0.459728	0.646853	0.098421	0.304252	-0.83953	-1.22579
O75351	VPS4B	-0.44068	0.416749	-0.14925	0.085464	-0.23156	0.029852	-0.1202	-0.65966	-0.50317	0.497854	0.008707	0.239461	0.584166	0.650528	-0.28878	0.321683	0.229367	-0.41409	-0.27052
O75356	ENTPD5	0.297226	0.141026	-0.02194	-0.15457	-0.02976	-0.01047	-0.03694	0.335453	-0.00166	0.197002	0.200511	-0.12683	0.241306	0.212153	-0.49747	-0.31068	0.485057	1.170343	0.390891
O75362	ZNF217	0.054495	0.198037	0.201541	0.164218	-0.17005	0.44018	-0.40926	-0.67092	0.106303	0.019629	0.142932	-0.3727	-0.37399	0.145282	0.003136	0.002678	0.380816	1.811465	-0.81167
O75367	H2AFY	-0.19765	-0.30514	-0.06938	0.158434	0.003381	-0.01937	-0.42086	0.126427	-0.67333	-0.45843	0.411099	-0.35196	-0.76292	-0.06873	0.972458	-0.23289	-0.07408	-0.18235	-0.74758
O75368	SH3BGRL	-1.49583	-0.24635	-1.27122	-1.02634	1.856272	-1.12459	0.423783	0.002127	1.395693	2.417866	-0.7834	-0.39163	-0.3489	0.025388	-1.14036	-0.90496	-0.38035	0.771913	-1.25118
O75369	FLNB	0.278754	0.087898	-0.07066	-0.40066	-0.85864	-0.57767	-0.5798	0.007069	-0.00552	-0.99784	0.273551	-0.52188	-0.04759	-0.41815	0.020764	-0.63624	0.643471	0.402983	-0.35414
O75376	NCOR1	0.184809	0.201354	0.059182	-0.08441	-0.77629	-1.03339	0.643074	0.295355	-0.50212	0.118788	0.561738	-0.38371	-0.15235	-0.2178	0.497388	-0.69323	-0.344	0.224767	0.161587
O75379	VAMP4	-0.99943	0.404609	0.209989	1.173554	-0.56043	-0.74245	0.203251	-0.29402	-0.03617	-0.06794	-0.55036	0.466866	-0.20198	0.649468	0.289636	0.142464	0.284982	0.277527
O75380	NDUFS6	0.052275	-0.0638	-0.64691	-0.27063	0.665918	-0.33012	-0.27013	-0.65225	-0.86536	-1.50703	0.814231	0.139742	-0.05136	0.546175	-0.23853	-0.89271	0.100682	1.330686	-0.11288
O75381	PEX14	0.236624	0.282713	-0.04167	1.177786	-0.18446	0.34436	0.646537	0.833437	0.121348	0.587034	0.106422	0.154493	-0.2868	-0.04218	0.101148	0.710563	0.301562	0.310921	-0.05833
O75382	TRIM3	-0.66811	-0.01907	-0.31568	-0.75921	0.323214	0.060512	-0.33213	-0.15957	-0.83375	0.225915	-0.18733	-0.06008	-0.52591	-0.28836	0.052595	-0.29913	-0.12348	0.442488	0.307209
O75390	CS	0.562294	-0.00333	0.036732	0.565503	0.488183	0.568005	-0.58344	0.255687	-0.03865	0.006473	-0.43533	0.168845	-0.28456	0.132943	1.088859	0.210358	0.258345	0.248501	0.115828
O75391	SPAG7	1.607692	-0.51397	-0.67559	-0.45025	-1.25093	-1.11592	-0.13763	0.352013	0.034035	-0.52792	0.613137	-1.39727	0.252369	-0.61019	-0.08779	-0.21272	-0.69808	0.068468	-0.30393
O75396	SEC22B	-0.48821	0.679405	0.226499	0.323031	-0.02048	0.223412	0.395594	-0.38475	0.154957	0.382614	-0.35559	-0.4574	0.00759	-0.2406	1.806434	0.162222	-0.04152	0.434505	0.341197
O75400	PRPF40A	0.862831	0.026991	0.206327	-0.04298	-0.13491	-0.08129	-0.47524	0.457524	-0.11787	-0.05564	0.041907	-0.08188	-0.36766	-0.22047	0.089348	0.048923	0.247144	0.504738	0.04778
O75410	TACC1	-0.52614	0.313794	0.69816	-0.10319	0.436867	0.118905	-0.34837	-1.09229	0.156105	0.015994	-0.30577	-0.34164	0.127125	-0.2082	-0.61841	-0.23444	0.03656	0.471844	-0.10327
O75414	NME6	0.233665	0.226006	0.095127	-0.10579	0.171978	-0.00895	-0.01106	-0.54058	-0.06867	0.100873	0.129147	-0.4956	-0.14002	0.435663	0.496696	-0.45808	-0.26028	-0.10896	0.221328
O75420	GIGYF1	-0.18667	-0.55638	0.087811	-0.40363	-0.01402	0.182925	0.596976	0.155768	-0.29976	0.128444	-0.53625	-0.10072	-0.52854	-0.05752	1.301653	0.45525	-0.72191	-0.371	-0.16489
O75427	LRCH4	-0.81794	0.279549	0.092125	-0.60461	-0.26354	-0.36798	0.18831	0.135931	0.530823	1.272453	-0.54612	0.321441	0.57615	0.639722	-0.16476	-0.23842	-0.4236	-0.08226	-0.20008
O75431	MTX2	0.54252	0.10641	-0.23792	0.528503	-0.1554	0.082987	-0.19252	-0.00183	0.091138	-0.01494	0.410471	-0.15553	-0.09466	-0.55006	1.408327	0.980578	-0.92966	-0.69552	-0.55536
O75436	VPS26A	-0.37647	0.433663	0.269773	0.241299	0.536866	0.643018	-0.01857	-0.29168	-0.15767	0.005503	-0.06732	-0.18441	-0.12239	0.161183	0.063917	0.551467	0.16378	0.033065	-0.20815

O75439	PMPCB	0.635231	0.039658	0.167028	0.442488	0.338978	0.019375	-0.21952	0.45819	0.347941	0.982878	0.541121	0.195378	0.511151	0.125227	0.942603	0.463353	0.100307	0.415596	0.383971
O75448	MED24	0.207039	-0.07029	0.814314	0.101061	0.157331	0.175549	-0.29805	0.789115	0.023936	0.260357	0.470265	0.147352	-0.11329	0.031777	0.503746	0.239004	0.230405	0.223409	-0.05191
O75449	KATNA1	0.1148	-0.01787	0.005892	0.036777	-0.4595	-1.21908	0.179453	-0.40223	-0.19966	-0.42694	0.111672	-0.20002	-0.34415	-0.31994	0.117871	-0.05577	-0.20729	-0.32867	0.213011
O75475	PSIP1	1.695799	-1.22236	-0.0592	-0.38676	0.665269	1.049568	-0.30898	0.60471	-1.04825	-1.48485	-0.3184	-1.39402	-1.31954	-0.97311	-0.58371	-0.38214	-1.67231	-0.42818	-0.32799
O75477	ERLIN1	0.375262	0.160192	0.050211	-0.12666	-0.35397	-0.06231	0.134308	-0.30478	-0.95739	0.03364	1.209636	-0.26902	-0.67928	0.132674	0.100328	-0.683	0.340222	0.006691	-0.69788
O75487	GPC4	-0.73446	0.331835	-0.5068	-2.05419	0.65625	0.271309	-0.43304	0.419642	0.276267	0.04611	-0.68582	-0.74476	0.013504	-0.98221	-1.7195	-0.51603	-0.46193	-0.79353	-0.19496
O75489	NDUFS3	0.025754	0.206309	-0.08776	-0.11298	0.092256	0.128809	0.286846	-0.32029	-1.16917	-0.66751	0.833773	-0.11091	-0.17763	-0.07602	1.241868	-0.15057	-0.31104	-0.09534	-0.26695
O75494	SRSF10	0.592896	-0.13757	0.376432	-0.04515	0.140252	0.561956	-0.72171	0.395089	-0.59511	-0.48349	0.712569	-0.63483	-0.10072	-0.57233	0.058342	-0.21094	-0.12368	0.333036	-0.21262
O75503	CLN5	-0.29214	-0.00921	0.169884	-0.78976	0.463418	0.292999	0.057012	1.079754	0.404291	0.19941	0.121742	-0.75241	-0.19565	-0.40251	0.549281	-0.07815	-0.49121	0.384429	-0.2472
O75506	HSBP1	0.594953	-0.37329	-0.43469	0.532867	0.145703	-0.34132	-0.37654	0.234871	-0.41375	-0.51331	1.647661	-0.0866	1.163986	-0.4553	0.650156	1.202412	-0.29975	0.139636	0.158847
O75521	ECI2	0.16498	-0.11636	0.039831	1.014887	0.79212	-0.0442	-0.11313	-0.4193	-0.1778	-0.35146	-0.38522	0.106914	0.029908	0.23383	0.283186	0.713538	-0.17452	0.983117	0.139179
O75525	KHDRBS3	-0.00633	0.275958	-0.80071	-1.12593	0.321704	-0.36626	-0.05213	-1.11767	-0.70409	-0.9142	0.432619	-0.23589	-0.44157	-0.93234	0.154669	0.058358	-0.80293	-1.37338	-0.6649
O75528	TADA3	0.995814	0.328428	-0.39406	-0.12691	-0.09263	0.280397	-0.63056	0.082218	-1.12948	-0.59008	0.129524	-0.17275	0.128288	-0.33523	-0.08817	0.511089	-0.00535	-0.23571	-0.00301
O75530	EED	0.544646	-0.2973	-0.1065	0.156098	0.560227	0.67892	-0.00969	0.526854	-0.52446	-0.03773	-0.03174	-0.08674	-0.383	0.496767	-0.24314	-0.21673	0.274845	0.309584	-0.19569
O75531	BANF1	0.540194	-0.42484	0.047536	-0.03335	1.335078	0.546847	-0.47351	0.708924	0.143681	-0.02988	0.147289	0.263575	0.128242	-0.58815	0.708628	-0.07979	0.043349	-0.41731	-0.46782
O75533	SF3B1	0.332705	-0.04755	-0.05831	0.480274	-0.28523	0.170682	-0.27265	0.602822	0.104826	0.014155	0.270324	-0.39097	-0.37404	-0.31947	0.201788	0.012155	-0.17979	0.073618	-0.11734
O75534	CSDE1	0.112552	-0.30013	0.029531	0.06779	0.191318	0.510964	-0.83288	0.268711	0.017932	0.399105	0.028058	-0.20225	0.309081	0.171443	0.149827	0.2129	0.390736	-0.08349	0.497962
O75554	WBP4	0.058547	-0.60738	0.212946	0.561428	0.276461	0.385041	-0.47855	0.705878	0.090773	0.514896	0.812797	-0.47256	0.311252	-0.10565	0.558691	0.27029	0.273637	1.143442	1.212606
O75556	SCGB2A1	1.248319	-0.90672	0.383363	-0.02036	1.913136	1.90262	-0.08878	0.362973	0.595298	-0.41436	0.439051	-0.13189	0.249498	-0.46383	-0.44152	0.606603	1.305912	-0.16306	0.264773
O75558	STX11	-0.91898	1.057069	1.194933	-1.15279	0.514076	-0.7728	0.340124	-1.36948	-0.28453	0.503674	-1.34605	1.566249	0.336003	1.99201	-1.13543	-1.22411	0.980942	0.241375	-1.60893
O75563	SKAP2	-1.24064	0.62048	1.039025	-0.85436	0.597913	0.584516	0.070106	-1.22259	0.057184	1.017904	-1.28302	1.291574	0.627717	1.725431	0.016789	-0.39	0.892568	0.999643	-1.00707
O75569	PRKRA	0.08378	-0.1688	-0.30579	0.024237	-0.34524	-0.7366	0.158154	0.417866	0.22373	0.007217	0.364455	-0.25319	-0.08362	-0.35008	0.199987	0.116296	0.17801	-0.11891	0.360003
O75581	LRP6	-0.81328	0.393045	0.126608	-0.47439	-0.81141	-1.21993	0.135905	0.926993	0.044832	0.020484	-0.383	-0.19208	-0.02926	-0.21557	0.025465	0.165391	-0.15563	0.141878	0.389196
O75582	RPS6KA5	0.796182	-0.90848	-0.2272	-0.05401	0.090492	0.32438	-0.42226	0.05493	-0.31741	-0.05657	0.235988	-0.57216	0.384349	0.209486	1.130247	-0.39486	0.202189	0.196089	-0.97046
O75586	MED6	0.682825	-0.22754	0.109389	0.015383	-0.06296	-0.09504	-0.50095	0.507653	-0.2412	0.200167	0.477457	-0.51769	0.135813	-0.87285	0.447235	0.11274	0.485632	0.477466	0.080636
O75592	MYCBP2	-0.34076	0.372566	0.151808	-0.45486	0.342285	0.65886	0.238101	0.001606	-0.02207	0.074484	-0.33307	0.051583	-0.21919	0.338305	0.336469	-0.4213	-0.08703	0.001294	0.012848
O75608	LYPLA1	-0.89082	-0.41275	0.546939	-1.40622	1.933375	-0.70568	0.20584	-1.1827	-0.05308	0.845732	-1.25812	2.15318	2.229666	0.938605	0.017022	-0.09603	1.195864	-0.0434	-0.56948
O75616	ERAL1	-0.57542	0.682737	0.398244	0.034543	0.058829	-0.68972	0.444313	-0.0751	0.075937	-0.14112	-0.22864	0.376181	0.435686	-0.17855	0.895267	-0.21434	0.251921	0.419305	0.687241
O75636	FCN3	0.006041	0.048936	0.069926	-0.58099	-0.22452	-0.43421	-0.57894	0.452923	0.971568	0.735288	-0.92876	-0.20131	-0.09995	1.068754	-0.42674	0.113174	0.529203	1.128078	-0.35259
O75643	SNRNP20	0.699373	-0.02231	0.118895	0.290587	0.149294	0.231306	-0.32546	0.526472	-0.14834	-0.00229	0.315884	-0.18017	-0.31041	-0.29165	0.053117	0.103479	0.097337	0.156914	0.075611
O75648	TRMU	0.040603	-0.19066	0.073444	0.597478	0.518095	0.553129	-0.46851	-0.06944	-0.16841	0.027981	0.337206	0.203178	-0.07605	0.432942	0.643442	1.322489	0.933993	1.276409	-0.38498
O75663	TIPRL	-0.37249	-0.05578	-0.91889	0.206842	0.14882	-0.27106	-0.62258	0.483505	0.268295	0.800404	-0.44932	-0.29895	-0.22784	-0.13537	0.135697	0.174666	-0.07919	-0.33946	0.186197
O75665	OFD1	0.210201	0.367256	-0.16968	-0.976	0.212759	-0.33395	-0.05071	-0.1448	0.060473	-0.15892	0.535367	-0.65888	-0.17868	-0.25882	-0.19227	-0.16502	0.394945	-0.42628	-0.40849
O75674	TOM1L1	-0.20158	-0.62122	-0.1727	0.042183	-0.10389	0.097473	-0.23257	-0.0013	0.009859	-0.44456	1.062351	-0.27818	-0.57596	0.182131	-0.62308	0.099886	-0.05321	-0.5324	-0.21463
O75676	RPS6KA4	-0.43401	0.413346	0.830961	-0.53746	0.084612	-0.10432	0.670476	-0.14721	0.465956	0.623861	-0.39621	0.417186	0.074625	0.232091	0.427289	-0.31388	-0.09123	-0.271	-0.35445
O75683	SURF6	0.24873	-0.20633	-0.16834	1.051167	-0.37443	0.083031	-0.199	0.238719	-0.32093	-0.91586	0.910259	-0.41139	0.047214	-0.96927	-0.46722	-0.45135	-0.504	0.02687	-0.20225
O75688	PPM1B	0.173093	-0.99721	-0.03885	0.41195	0.873282	0.499361	-0.59939	0.593686	0.396888	1.065495	-0.32149	0.01421	0.206008	0.361837	-0.42729	-0.29156	0.238368	-0.05717	-0.00539
O75689	ADAP1	-0.23032	0.694384	0.392293	-0.5706	0.327122	1.012738	-0.06905	-0.95873	-0.09329	1.170605	0.029988	0.569998	0.05327	1.192659	0.686967	-0.43684	1.661025	0.728623	-0.87769
O75691	UTP20	0.267681	0.108907	-0.13505	0.597354	-0.0152	0.070398	0.365308	0.279374	-0.23731	-0.2205	-0.08322	-0.20287	-0.69219	-0.394	0.659186	-0.02011	0.206648	-0.32974	0.247316
O75694	NUP155	-0.13889	0.24611	0.00168	0.145763	0.027116	0.091332	0.524407	0.023137	-0.42541	-0.05839	0.097062	0.011162	-0.15257	0.06288	0.888292	-0.07033	0.010912	-0.22605	0.153672
O75695	RP2	-0.3927	0.58262	-0.09312	-0.69796	0.078257	-0.03324	1.293727	0.319672	0.733589	2.114809	-0.31277	-0.14443	-0.49742	0.710776	-0.57629	-0.16877	-0.04406	0.347213	-0.52878
O75717	WDHD1	1.247765	-0.25859	0.46916	0.95401	0.317558	0.474962	0.178574	0.455485	0.171235	-0.07361	-0.06564	-0.12318	-0.54824	-0.15219	-0.46835	0.958297	-0.17251	-0.98526	0.113854
O75718	CRTAP	-0.61332	1.278189	0.582072	-0.8109	0.618582	0.503626	0.445806	-0.08132	0.242736	0.365855	-1.11059	-1.12708	0.815802	-0.80643	-0.66906	0.363013	-0.3217	0.925764	2.284076
O75746	SLC25A1	0.406296	-0.37048	-0.20438	0.959681	-0.6262	-0.51114	0.114974	-0.03025	-0.19553	-0.04262	-0.84581	-0.31974	-0.21657	-0.351	1.748038	0.904201	-0.78858	-0.74117	-0.37188
O75781	PALM	0.796664	-0.5234	-0.30938	-1.64229	-0.83365	-0.75561	0.316182	-0.05334	0.416558	-0.18028	-0.94721	-1.28285	-0.23508	-1.48871	1.2664	-1.10772	-1.08754	0.467891	0.391664
O75787	ATP6AP2	0.831245	-0.10181	0.105806	-0.12685	-0.03588	0.132297	0.02783	-0.15409	-0.21371	-0.09764	-0.76269	0.05564	0.096619	0.38521	-0.13263	0.551378	1.098212	0.302345	-0.43531
O75792	RNASEH2	-0.38715	0.295356	0.651636	-0.36574	0.520412	0.529055	0.192937	0.590669	0.408057	0.734156	0.10292	-0.0162	0.544401	0.252491	0.173419	0.224931	0.59016	0.203326	-0.35098
O75794	CDC123	0.574284	0.189343	0.104921	0.19518	0.464031	0.500047	-0.23964	0.728083	0.062288	0.074117	-0.48289	-0.03211	0.261426	0.547217	-0.21648	0.048832	0.970263	-0.08866	0.431713
O75817	POP7	0.781564	-0.28061	-0.0196	0.169788	0.305347	-0.42376	-0.22253	0.659495	-0.48845	-0.81047	0.324442	-0.01713	-0.44158	-0.03737	0.309962	-0.37983	0.111041	0.2488	-0.55201
O75821	EIF3G	-0.18463	0.016105	0.478761	-0.12579	-0.12593	0.165721	0.07612	0.060913	0.176405	0.376598	0.038858	-0.70918	-0.0933	-0.80359	0.407834	0.104812	-0.21307	0.02643	0.002741
O75822	EIF3J	0.468724	-0.60435	0.176978	0.166332	-0.16644	-0.05882	-0.69798	-0.47675	-0.16366	0.405958	0.575977	-0.92032	0.674152	-1.07754	-0.60224	0.752673	0.175413	0.664479	0.260328
O75828	CBR3	0.049275	-0.37155	-0.11646	-1.77058	0.008686	-0.04497	-1.94878	-0.88427	0.835171	-0.59326	-1.32669	-0.51254	0.108775	-0.49914	-1.16907	-0.41601	-0.5284	-0.25259	-0.63636
O75832	PSMD10	0.108558	-0.33563	-0.42595	0.065773	0.963732	0.578193	-0.68455	-0.255	0.031811	0.707582	-0.1308	0.303407	-0.101	0.425051	-0.29908	-0.07683	0.402061	0.166638	-0.06654
O75843	AP1G2	-0.345	0.665015	1.021572	0.288962	-0.16566	-0.25773	0.637873	-0.45569	0.021582	0.507622	0.15062	0.31943	-0.67602	0.624941	-1.08011	-0.70067	0.004723	-0.28412	-0.448
O75844	ZMPSTE2	-0.1574	0.290629	0.696595	0.777818	0.287938	0.1178	0.468438	0.134327	-0.30431	0.185195	-0.23219	0.479338	-0.16491	0.341087	0.268389	0.737677	0.684226	-0.03874	-0.27688
O75864	PPP1R37	0.590228	-0.38379	-0.21723	-0.09269	0.499889	-0.18148	-0.21566	0.27204	0.279363	-1.77566	-0.12293	0.227875	-0.16351	0.226719	0.326771	-0.62092	1.028977	-0.05494	-0.36256
O75865	TRAPPC6	-1.31445	-0.05333	0.048782	0.653614	0.799461	1.153781	-0.39562	0.199519	-0.10166	0.07742	0.171185	0.139339	-0.21487	0.568733	-0.90369	-0.78524	-0.45439	0.235595	-0.98386
O75874	IDH1	-0.05794	-1.63532	0.047974	-1.33234	0.813177	0.288373	-0.05351	0.747784	-0.12327	-0.59885	-0.19884	0.365052	1.1136	0.314758	0.359889	0.227724	-0.04252	0.101455	0.051155
O75879	GATB	-0.39594	-0.17857	-0.29686	1.021652	0.366978	0.17261	-0.21822	-0.25741	-0.03026	0.401165	0.191046	-0.1955	-0.1918	-0.2016	0.03614	0.498962	0.372412	0.352723	0.014561
O75880	SCO1	1.494743	0.408533	0.001975	-0.66752	-0.26799	-0.5732	0.101932	-0.06393	-0.20043	0.414993	1.29288	-0.17776	-0.18835	-0.55412	0.267355	-0.3531	-0.44733	-0.05088	-0.66543
O75881	CYP7B1	-0.72165	0.261185	0.349611	-0.35697	-0.4466	-0.6084	0.501835	-0.93414	0.37468	1.147741	0.114399	-0.7926	-0.04037	-0.15599	-0.41135	-0.60976	-0.71835	1.384846	1.0015
O75882	ATRN	-0.28478	0.340346	0.125736	-0.29377	0.297555	0.061349	0.03873	0.781565	0.348962	0.567535	0.19301	-0.33174	0.202837	-0.00182	0.185965	-0.4062	0.075252	0.439177	0.265643
O75886	STAM2	-0.20735	0.103836	-0.20996	-0.69033	-0.55723	-0.73353	-0.49628	-0.2564	-0.26024	0.199646	0.54158	-0.57978	0.187975	-0.15375	0.363827	0.229711	-0.26115	0.241947	0.511105
O75888	TNFSF13	0.184218	-0.34744	-0.48393	-0.62238	-0.18257	-0.8058	-0.24574	-0.11662	0.636601	-0.48751	-1.41501	-1.42367	-0.63517	-0.48982	3.021674	-0.28077	-0.40719	-0.59469	-1.45705
O75891	ALDH1L1	-0.98534	-0.25317	-0.30135	-1.10887	-1.00906	-1.15481	0.196862	-1.11303	0.724119	-0.71602	-1.4322	-0.91324	0.589297	-0.53642	-0.42631	-0.61712	-0.97218	0.694055	-0.88142
O75900	MMP23A	-0.62262	-0.06535	0.749588	-0.50065	-0.15539	-0.82214	2.196352	-0.16692	0.791784	-0.44799	-0.80717	-1.21425	-0.13996	0.051083	0.57778	-1.26523	-0.78491	-0.97046	-0.00359
O75907	DGAT1	0.56273	-0.20146	-0.45173	-0.23587	-0.40749	-0.17231	-0.31909	0.092728	-0.37114	0.556455	-0.0034	0.007708	-0.08005	0.023447	0.190586	0.869207	0.155463	-1.24474	-0.49708
O75909	CCNK	0.072919	0.342926	-0.29236	-0.40571	-0.36189	0.082003	0.10922	0.217519	0.362195	-0.19266	0.160675	-0.3454	0.215791	-0.07345	0.069771	-0.15128	0.326944	0.697182	0.598598
O75911	DHRS3	-1.17646	0.50456	0.539976	-0.36057	0.095713	0.010472	0.336869	-1.8633	-0.23295	0.237037	0.318949	1.952656	0.0937	0.709923	0.403576	1.774044	2.190734	1.996407	-1.36923
O75915	ARL6IP5	-0.30226	0.652306	0.388091	0.010025	-0.15644	-0.22774	0.225171	-0.65641	0.00628	-0.20757	-0.11505	0.113722	-0.15802	0.236231	-0.1053	-0.30736	-0.08186	-0.25705	0.012448
O75923	DYSF	-0.93654	1.503587	0.972657	-1.06695	0.070496	-0.68262	0.073985	-1.40505	0.12005	1.134758	-0.87057	1.330589	0.069401	2.207825	-0.48261	-0.19351	-0.23889	-0.92615	-0.76287
O75925	PIAS1	0.232218	-0.08602	0.248994	-0.14359	0.048003	-0.02871	-0.25651	-0.20559	0.150102	-0.60677	0.146445	-0.22621	-0.09006	0.056825	-0.17133	-0.18007	-0.10009	0.383838	-0.50786
O75934	BCAS2	0.314409	-0.18972	-0.28025	-0.06892	0.172625	0.147876	-0.13211	0.685194	-0.00407	-0.10929	0.346408	-0.1306	-0.61412	-0.26318	-0.04809	-0.01751	-0.00127	0.466079	-0.01242
O75935	DCTN3	0.113521	-0.04505	-0.15223	-0.44845	-0.0873	0.257963	-0.44888	0.190701	-0.9771	0.123143	-0.70231	-0.61198	0.086462	-0.09583	-0.28912	-0.4055	-0.77421	-0.56048	0.153261
O75937	DNAJC8	0.976096	-0.48926	0.768958	-0.29175	0.885182	1.047512	-1.05567	0.751472	0.195414	0.39473	-0.02969	-0.81126	-0.25378	-0.54458	0.089961	0.411802	-0.21775	0.535791	-0.20945
O75940	SMNDC1	0.461965	-0.1368	-0.31187	-0.20704	0.301626	-0.38272	-0.38551	0.838438	-0.31647	-0.67581	0.451396	-0.44056	-0.01986	-0.29335	0.06303	0.248611	-0.01913	0.211458	0.061937
O75943	RAD17	-0.16341	0.244893	0.153069	-0.2146	0.019735	0.05345	0.159305	-0.30461	-0.01874	0.608032	0.317768	-0.06727	-0.21832	0.150041	0.014452	0.781836	-0.17782	0.435414	-0.58171
O75947	ATP5H	-0.09105	-0.36657	-0.19929	0.346328	0.069076	0.043589	-0.44216	-0.49277	0.054643	-0.13063	0.437194	0.06009	-0.48199	0.171446	1.026922	1.01489	-0.27494	-0.43533	-0.72957
O75955	FLOT1	-0.6797	0.079981	-0.01546	0.584649	-0.12917	-0.05789	0.186909	-0.99912	0.205427	0.549795	0.313331	-0.04624	-0.52539	0.756772	-0.18321	-0.12534	-0.05498	0.011002	-0.47613
O75962	TRIO	-0.22791	0.148589	0.025869	-0.2585	-0.404	-0.25608	0.097771	0.053166	0.076599	0.0191	-0.57655	-0.27293	0.280696	-0.1341	0.078661	0.185154	-0.25751	-0.09311	0.632206
O75964	ATP5L	-0.04727	0.00427	-0.21416	0.428479	0.337143	-0.05215	0.265216	-0.37354	0.486908	-0.05646	0.328963	0.153665	-0.56666	-0.04941	1.147737	1.079644	-0.64789	-0.88327	-0.14284
O75970	MPDZ	0.156917	0.072985	0.156953	-0.65806	-0.53798	-0.34229	-0.06405	0.208731	-0.38969	-0.76082	0.156974	-0.50606	-0.11671	-0.40482	-0.86721	-0.59723	-0.32728	0.00704	0.727794
O75976	CPD	-1.2995	0.017313	-0.155	1.061981	-1.61214	-1.24439	-0.23885	-0.50316	0.250863	0.396568	0.371374	-0.38397	-0.72384	-0.03357	0.672802	-0.02505	-0.52123	-0.762	-0.54643
O75995	SASH3	-0.87778	0.280086	0.991931	-0.5098	-0.14524	-0.1843	-0.21727	-1.1916	0.63271	1.116026	-0.76219	0.313242	0.436489	0.857725	-0.34212	-0.46421	0.27143	0.302435	-0.55521
O76003	GLRX3	-1.20817	-0.01929	-0.04561	0.066054	0.358666	0.866263	-0.44323	0.095756	-0.19288	0.703241	-0.44964	0.096557	0.611271	-0.3529	0.118332	0.659262	0.805982	0.0902	-0.31352
O76011	KRT34	-1.25614	1.256501	-1.59187	-0.91635	-1.92192	-1.39909	-1.11554	-1.06633	0.313232	-3.01233	-1.4771	-0.09063	0.437652	0.176613	-0.41044	-1.85466	-0.83423	-0.70809	1.272297
O76021	RSL1D1	0.292549	-0.76322	-0.45925	0.991961	-0.16425	0.230611	-0.84494	0.054729	-0.77886	-0.56381	0.603174	-1.21203	0.083222	-1.71207	0.379811	-0.17955	-0.31385	0.240281	-0.51715
O76024	WFS1	0.74699	-0.39642	-0.49166	-0.74304	-0.84882	-0.55103	0.548613	0.116258	0.141965	-0.46889	0.445875	-0.99958	-1.24427	-1.19023	-0.47619	0.156926	-0.38409	-0.61449	-0.57001
O76027	ANXA9	0.278349	-0.96174	-0.86702	-1.34356	-2.09091	-0.82129	-0.54745	0.956395	-0.05523	-1.10716	-0.61664	-0.89407	-0.77204	-0.56745	-1.18965	-1.39101	-0.76837	-2.57883	-1.6589
O76031	CLPX	0.373392	0.357448	0.227943	0.912302	0.021145	0.065846	-0.53002	0.100184	-0.63645	0.061262	0.289329	0.025279	-0.53211	-0.22432	0.503642	0.579287	0.288101	0.81473	-0.11349
O76054	SEC14L2	-1.01831	-0.01702	0.06095	0.731461	0.217857	0.636785	-0.46478	-0.50567	-0.01611	0.033398	0.019744	-0.96667	-0.08756	-0.24902	-0.26391	-0.85748	-0.12899	-0.21029	-0.08257
O76062	TM7SF2	0.554161	-0.49106	-0.75369	-0.07568	-1.28024	-0.75564	-0.18338	-0.00741	-0.16951	-1.46135	-0.41698	-0.05032	-0.29178	-0.21279	0.955538	-0.5905	-0.10034	-0.97545	-0.59579
O76070	SNCG	-0.47523	-0.32009	-0.12081	-1.43583	2.478203	1.894918	-0.99135	-2.049	-0.30519	-1.35115	-1.86368	-0.89654	-0.02101	-2.09682	1.270328	-2.35429	-1.51657	-3.45437	-2.39044
O76071	CIAO1	0.159035	0.376664	0.341837	0.25457	0.193693	-0.42901	0.04764	-0.15526	-0.03629	-0.24861	0.189139	0.453549	0.104916	0.567235	0.144673	-0.21476	0.509871	-0.1743	-0.18776
O76074	PDE5A	-0.01394	0.300361	-1.01705	-0.38365	-0.09648	-0.02939	0.396756	-0.6628	0.539318	-0.90884	-0.5014	-1.01432	-0.01003	-0.02772	-0.03246	-0.36941	-0.11384	0.780303	0.265443
O76075	DFFB	0.05532	0.239922	-0.06771	-0.37577	-0.00875	-0.45938	0.090189	0.146574	0.258383	0.016933	0.044481	-0.2969	-0.33355	0.137913	-0.25356	0.379151	-0.22899	0.008755	-0.29342
O76094	SRP72	-0.00218	0.172297	0.324649	0.131422	0.1753	0.302012	0.04785	-0.33134	-0.6132	-0.00025	0.063067	0.33038	0.026415	0.047844	-0.26635	0.66757	0.640702	0.259912	0.027844
O94760	DDAH1	1.623266	-1.34135	-1.99865	-0.8439	0.687293	-0.49681	-0.11207	-0.268	0.26525	-0.52783	1.743222	-1.04846	-0.38101	-0.7237	-0.8782	-1.28376	0.055766	-0.62551	-1.01759
O94762	RECQL5	0.509513	-0.19118	0.018864	0.703815	-0.3706	-0.06017	-0.29861	0.664186	-0.27077	-0.22599	0.229432	-0.26036	-0.04033	-0.45366	-0.54659	0.031691	-0.23009	0.726062	-0.27161
O94763	URI1	-0.12675	-0.63853	-0.04707	-0.39018	-0.57007	-0.38204	0.071964	1.504203	-0.07147	0.516115	0.004057	-0.81082	0.165672	-0.6528	0.502948	-0.31857	-0.41563	-0.51517	0.039613
O94766	B3GAT3	0.362585	0.569684	0.560618	-0.44291	-0.24601	-0.37088	0.281014	-0.69333	-0.19152	-0.22712	-0.13613	-0.42293	-0.21702	-0.07953	0.615135	0.360232	0.435106	1.043213	-1.06896
O94768	STK17B	-0.89219	0.396189	0.371469	-0.47746	0.222185	0.588974	0.120358	-0.56219	0.189124	-0.08134	-0.66206	-0.66108	-0.06292	-0.012	0.060491	0.019355	0.247909	0.749202	-0.24005
O94769	ECM2	0.011248	0.39327	-0.30705	-0.78524	-1.72831	-0.61677	0.35048	-2.11552	-0.47261	-0.05714	0.02762	-1.91199	-0.36872	-0.02536	-0.47729	-0.09549	-0.61455	-0.84262	-0.62664
O94776	MTA2	0.466306	-0.08422	0.322761	-0.02046	0.054383	0.001393	-0.12898	0.345211	-0.40976	-0.13663	-0.03859	-0.24422	-0.0869	-0.23918	0.635386	-0.07973	-0.35928	-0.16039	-0.32051
O94788	ALDH1A2	-1.03214	-0.09668	0.273815	-0.92426	0.626481	0.110297	0.011645	0.020346	0.664741	-4.46364	-0.50609	0.176793	0.489125	-0.23196	-1.33005	0.263054	-0.41665	0.365269	0.605805
O94804	STK10	-1.60529	1.301824	1.726438	-0.43204	0.171472	-0.01715	0.541849	-1.31673	-0.1386	0.938219	-1.11113	1.032931	0.639198	1.609489	-0.68837	-0.29364	0.355948	0.74104	-0.90095
O94806	PRKD3	0.15707	0.227386	0.243078	-0.0709	-0.25232	-0.48475	0.635488	0.782345	-0.74099	-0.29242	0.559863	-0.42286	-0.17061	-0.28664	0.100773	0.139641	-0.16122	-0.14911	-0.34559
O94808	GFPT2	-1.2361	0.226853	0.410645	-1.30993	-0.27126	0.245081	-0.23889	-0.22791	1.139239	0.413006	-1.03538	0.53793	0.787428	0.023736	-0.61621	0.177074	0.440718	0.416427	2.488726
O94811	TPPP	-1.4228	0.071466	-0.3598	-1.53883	-0.78566	-1.36292	0.370354	-0.34472	0.69532	-0.4409	-0.01735	-0.69652	-0.41418	0.377323	-0.05511	-0.67038	-0.31774	-0.21979	-1.61776
O94813	SLIT2	-0.53119	0.290208	0.034299	-0.27781	-0.13085	-0.983	0.050698	0.172404	0.226108	-0.54217	0.390925	0.067473	0.156591	-0.37809	-0.55783	0.682135	-0.09396	0.06732	0.816281
O94817	ATG12	-0.04716	-0.0601	0.152878	-0.0702	-0.23936	-1.39262	0.148525	-0.82969	-0.29987	-0.2164	0.003854	0.071307	-0.10839	0.35153	0.310743	-0.20819	0.271074	-0.44727	-0.4502
O94819	KBTBD11	-0.75613	-0.19193	-0.03589	-0.73658	-0.53573	-0.82533	-0.22706	-1.02761	-0.39464	0.233536	0.362158	0.346417	-0.41447	0.568859	-0.89585	-0.99676	0.354135	-1.26453	-0.79971
O94822	LTN1	-0.09612	0.423255	0.266635	-0.22321	-0.23682	-0.13439	0.149387	-0.11952	-0.27348	0.090132	-0.27181	0.456702	-0.01686	0.500045	0.335166	0.321116	0.245312	-0.3368	0.054958
O94826	TOMM70	-0.01498	-0.08032	-0.15603	0.303827	0.342385	0.433933	-0.8001	-0.30311	-0.29958	0.87929	0.355451	-0.25852	-0.27537	-0.28732	1.493385	0.645465	-0.18511	-0.39766	-0.45489
O94829	IPO13	0.024087	0.725947	-0.29521	-0.1516	-0.21737	-0.01605	0.163971	-0.29998	-0.21505	-0.45712	0.10812	-0.21388	0.466046	-0.18065	-0.31846	-0.27164	0.027734	-0.70761	0.227342
O94830	DDHD2	0.086404	0.450576	0.476603	-0.85863	-0.72346	-0.3605	0.860173	-0.31515	0.005527	-0.57102	-0.35998	-0.1573	0.223696	-0.49435	0.823905	-0.48788	0.386597	-0.14537	0.768969
O94832	MYO1D	-1.08401	-0.7378	-0.81997	-0.26665	-0.61495	-0.75431	-0.39341	-0.79192	0.113654	-0.97724	-0.4222	-0.98046	-0.64837	-0.84558	-0.626	-0.48284	-0.82336	-0.37292	0.421093
O94842	TOX4	0.60214	-0.21454	-0.10954	-0.17977	-0.46398	-0.11591	-0.46919	-0.33917	-0.07419	-0.69674	0.100491	-0.45949	0.029853	-0.66626	0.434723	-0.61553	0.142933	0.463211	0.157798
O94851	MICAL2	-0.89561	0.122283	0.129159	-0.44232	0.423706	0.563222	0.01496	-0.06058	0.488398	-0.49671	-0.414	-0.11156	1.122528	0.048642	-0.28575	0.284586	-0.51987	0.378494	1.406197
O94854	KIAA0754	0.124377	-0.15967	0.198836	-0.33747	-0.25018	-0.29992	-0.38679	-0.06817	0.548838	-0.64566	-0.46145	-0.20568	-0.17843	-0.33159	-0.46271	0.108093	-0.25458	-0.31469	-0.28107
O94855	SEC24D	-1.05118	1.215511	0.544626	-0.75796	-0.22151	-0.39304	1.089341	-0.13498	0.456641	0.109336	-0.47872	-0.17924	0.404862	-0.18211	-0.37127	0.242463	-0.02849	0.780507	1.666731
O94864	SUPT7L	-0.18396	-0.02678	-0.0261	0.324972	0.097391	0.356262	-0.00126	0.119761	-0.29474	0.225819	0.61281	-0.47977	-0.00068	-0.32983	-0.11799	-0.18806	-0.23858	-0.09241	1.175284
O94868	FCHSD2	-0.68154	0.122619	-0.06453	-0.62697	0.117997	0.250243	0.175122	-0.11853	-0.13547	0.194219	-0.53686	-0.1123	0.119212	-0.26895	-0.51414	-0.0015	-0.30506	0.166846	-0.9567

O94874	UFL1	-0.37088	0.42224	0.597626	0.305845	0.162718	0.143663	0.50734	-0.81117	0.235638	0.319182	0.610183	-0.02136	-0.1906	0.168349	0.607897	-0.14375	-0.3687	0.185801	-0.09901
O94875	SORBS2	-1.38304	-0.7762	-2.02037	-1.11229	-2.2694	-1.62811	-1.23166	-1.43969	-0.00906	-1.50401	0.684418	-1.79726	-1.29389	-1.3942	0.659667	-1.22757	-1.97182	-1.24636	-1.51476
O94876	TMCC1	-0.47866	0.522155	0.772459	-0.24	-0.09989	-0.13035	-0.17504	0.361673	0.316012	0.176484	-0.07046	0.096859	0.280838	-0.00499	1.255463	-0.0848	-0.2659	0.229567	0.430304
O94880	PHF14	0.457875	-0.03645	0.089908	0.15634	0.05111	0.022896	-0.36785	0.068345	0.046659	-0.40688	0.813023	-0.79968	-0.33279	-0.33143	-0.03924	-0.20435	-0.49854	0.496295	-0.24545
O94885	SASH1	-0.18376	0.478801	0.180496	-0.21182	-0.43039	-0.45065	0.113497	-0.38271	0.02031	0.17626	-0.58879	-0.18377	0.021049	0.199205	0.262585	-0.12228	0.400164	0.464617	-0.12726
O94886	TMEM63A	-1.43614	0.636018	-0.19262	-0.17397	-0.82053	-0.79811	0.101857	-0.63951	0.245295	-0.54947	-0.43575	-0.16024	-0.44865	0.711316	0.593982	-0.64209	-0.11203	-0.69842	-1.53264
O94887	FARP2	-0.62931	0.328642	-0.09656	-0.49605	-0.84453	-0.60908	-0.03159	-0.07731	0.514624	-0.79615	0.177468	-0.65626	-0.17967	-0.00994	-0.22763	0.387275	0.165399	0.049433	0.127136
O94888	UBXN7	0.689754	-0.59883	-0.25595	0.109314	0.530944	0.45395	-1.0688	0.769353	-0.11986	0.70376	0.078783	-0.69468	0.320787	-0.48851	1.132492	-0.56246	-0.52221	-0.67623	0.159585
O94901	SUN1	-0.68307	-0.16028	0.070309	-0.23096	-0.60135	-0.24616	0.130451	0.382941	0.014589	-0.31545	-0.24044	-0.3336	-0.81914	-0.46655	0.6344	0.259095	0.222572	0.174734	-0.09887
O94903	PLPBP	-0.24224	-0.08565	-0.06578	-0.81034	0.359635	0.010924	-0.22371	-0.06422	-0.04836	0.513246	0.715811	0.005898	-0.1763	0.615477	0.936009	-0.94338	0.494538	0.38566	-0.42683
O94905	ERLIN2	0.096249	0.106342	-0.55979	-0.48156	-0.5355	-0.49498	-0.07059	-1.0115	-0.20564	-0.01749	0.015007	-0.26885	-1.35163	-0.04408	0.250912	-0.82448	0.910148	0.557615	0.081668
O94906	PRPF6	0.55565	-0.24505	0.116633	0.390182	0.136217	0.345042	-0.32706	0.741689	-0.35477	-0.00597	0.24931	-0.20057	-0.07975	-0.43855	0.332854	-0.103	0.046339	0.114634	-0.14367
O94911	ABCA8	-1.54101	0.63451	0.171803	-1.78784	-0.36938	-1.15804	-0.26085	-1.9645	0.330943	-1.37263	-1.90178	-0.3967	-1.17623	-0.3322	0.643217	-1.00807	-1.05816	0.525407	-0.3902
O94913	PCF11	0.769425	0.573858	0.201986	-0.16884	-0.19758	0.139936	0.392886	0.19641	-0.64058	0.116188	0.24818	-0.57624	0.213882	-0.23488	0.686258	0.14132	-0.21653	0.082303	-0.43218
O94915	FRYL	0.094448	0.530448	0.02515	0.184469	-0.31797	-0.1387	-0.03293	-0.40281	-0.14497	0.295391	0.03444	-0.04112	0.17148	0.578321	-0.71297	0.485079	-0.09885	-0.23002	-0.38968
O94919	ENDOD1	0.736496	0.106336	0.03481	-0.03633	-0.5502	-0.71642	-0.32977	-0.97375	0.342284	-0.15262	-0.04098	0.142372	0.344195	-0.18229	0.13546	0.551563	-0.20123	0.208612	-0.73818
O94921	CDK14	-0.65741	-0.02151	0.111717	-0.70677	-0.58408	-0.82493	0.32485	-0.22401	0.511683	-0.36949	-0.51288	-0.56115	0.217154	0.099049	1.863966	-0.52606	-0.19944	-0.75425	1.019364
O94923	GLCE	-0.28757	0.09242	0.186707	-0.12063	0.016584	-0.37894	0.081632	0.810186	0.060338	0.520704	0.003853	0.494062	0.140904	-0.23789	0.09565	-0.50985	0.134916	-0.09591	0.033657
O94925	GLS	-1.64922	0.357717	0.518879	0.161925	-0.39768	-0.522	0.690517	-0.20875	0.152095	1.135991	-1.34476	-0.84549	-0.15864	-1.03014	1.042889	-0.37803	0.674455	0.63967	0.543159
O94927	HAUS5	0.109461	-0.05972	-0.31158	-0.29054	0.446373	0.447142	0.220541	0.455048	0.040847	-1.2904	-0.03057	0.174079	0.15768	0.011676	-0.87441	-1.36818	0.217446	-0.41515	0.068989
O94952	FBXO21	1.093921	-0.75206	-0.26951	-0.50441	-0.48958	-0.09314	0.197641	0.141805	0.087896	-1.72705	0.47477	-0.46224	0.058891	-0.73158	-0.82715	-1.27492	-0.24723	-1.82058	0.428765
O94953	KDM4B	0.014388	-0.07006	-0.55921	0.020273	-0.39913	-0.52057	0.103238	1.199606	0.487478	0.231216	0.286351	0.185809	-0.1727	-0.23567	-1.77499	-0.21021	-0.0222	-0.537	-0.25272
O94956	SLCO2B1	-1.40649	1.068685	0.357204	-0.86219	0.436902	-0.29782	0.447668	-1.0031	0.523069	0.888664	-1.35908	0.487689	-0.0302	0.599515	-0.21242	-0.09577	-1.34228	1.006088	-1.0756
O94964	SOGA1	0.294967	0.322218	0.221574	-0.21253	0.710454	0.762515	-0.09198	0.694555	0.817614	0.553956	0.006895	-1.06724	0.629085	0.113311	0.42605	-0.50481	-0.36805	0.284581	-0.27013
O94966	USP19	0.280148	0.142515	-0.0284	-0.66518	0.301356	0.374246	0.318504	0.282357	0.701821	0.178394	0.039447	-0.16474	0.035938	-0.03956	0.392813	-0.25527	-0.03719	-0.46031	-0.16866
O94967	WDR47	-0.183	0.010755	-0.19132	-0.77945	-0.36351	0.03737	0.468058	-0.24414	0.376503	-0.20787	0.301179	-0.6316	-0.01482	-0.06512	0.32236	0.042137	0.407403	-0.38812	0.064374
O94973	AP2A2	-0.60868	0.905234	0.8284	-0.43537	0.459138	0.353885	0.541806	-0.34768	0.40341	-0.04399	-0.35551	0.244652	-0.07724	0.374314	0.308116	0.538553	0.31561	0.431181	0.000231
O94979	SEC31A	-0.65434	0.345706	0.250899	-0.3147	0.047232	0.00069	0.267125	-0.06983	0.127808	0.147108	-0.4066	0.088162	0.272265	-0.06616	0.307636	0.261572	-0.02162	0.407297	0.88312
O94985	CLSTN1	-0.08301	0.226226	-0.59629	-0.39884	-0.04125	0.031413	-0.33523	0.460345	0.109834	1.155811	0.305786	-0.10895	0.094196	0.091	-0.37459	0.156676	0.334797	1.560289	0.194318
O94992	HEXIM1	0.556176	-0.57816	0.044865	1.735579	-0.59955	0.332274	-0.28949	0.332206	-0.60977	-0.40349	-0.09303	-1.46099	-0.21818	-0.69038	0.22567	1.109299	0.012697	0.383056	-0.7483
O95049	TJP3	0.297007	-0.55346	-0.03425	-0.51111	-0.4075	-0.605	-0.00316	0.087028	-0.69341	0.470719	0.854494	-0.87624	0.131069	-0.70362	0.624694	0.289596	-0.20609	-0.50017	-1.49892
O95071	UBR5	0.168293	0.101768	-0.21787	0.105945	-0.01754	0.312607	-0.57749	0.501082	-0.64504	0.022159	0.024829	-0.01683	0.411228	-0.1629	0.306434	-0.06818	0.138063	-0.06189	-0.0918
O95081	AGFG2	-0.63433	-0.15523	-0.26174	0.08255	-0.64982	0.860899	-0.16646	-0.35248	-0.10955	0.138879	0.402012	-0.57717	-0.36753	-0.21764	0.599681	0.47478	-0.59973	-0.30043	-1.13117
O95104	SCAF4	0.686557	-0.47888	-0.02898	-0.43653	-0.04238	0.290875	0.074453	0.580573	0.003044	0.216162	-0.02773	-0.06691	0.333599	-0.64338	0.261997	0.12235	-0.20599	0.405059	-0.04593
O95139	NDUFB6	0.072119	0.345228	-0.0506	-0.12811	0.470604	0.190949	0.045184	-0.02897	0.829942	-0.32682	0.485776	-0.10834	0.401425	0.050222	0.551543	-0.52691	-0.18873	-0.16114	-0.61667
O95140	MFN2	0.189268	0.288377	0.104065	-0.24269	0.05885	-0.00909	0.218068	0.019191	0.404118	0.168369	-0.09252	0.124075	-0.18174	-0.04159	0.540759	0.835278	0.02007	-0.19797	-0.06391
O95149	SNUPN	0.516986	-0.13613	-0.18125	-0.39465	1.115955	1.522353	-0.39011	0.290101	-0.31873	-0.03654	0.608869	-0.52828	-0.36436	-0.56211	-0.20885	-0.76262	0.418753	0.486492	-0.14179
O95155	UBE4B	0.357363	0.523222	0.383919	-0.35036	-0.18566	0.088726	-0.05778	0.146152	-0.08382	-0.17209	0.288055	-0.20683	0.180038	-0.18874	-0.44733	-0.01545	0.134318	0.405889	0.12959
O95163	ELP1	0.316016	-0.05761	0.69507	-0.28203	-0.0647	0.343482	-0.35706	-0.08021	0.13694	-0.1534	0.018756	-0.27536	-0.16742	-0.03718	-0.30701	-0.36277	-0.23713	-0.50212	-0.11029
O95167	NDUFA3	0.057032	0.22171	-0.13417	-0.08562	0.108392	0.38499	-0.01247	-0.3521	-0.38647	-0.4961	0.825146	-0.08326	0.235002	-0.21048	1.089052	0.084551	-0.81514	-0.14679	-0.05997
O95168	NDUFB4	0.114942	0.410968	0.376917	-0.64591	0.306179	-0.00783	0.291262	-0.46038	-0.47523	-0.76428	0.551557	0.135714	0.121634	0.088529	1.066572	-0.8332	-0.54029	-0.23809	-0.20365
O95169	NDUFB8	0.020882	0.364851	-0.18789	-0.54399	0.014594	0.237068	-0.65112	-0.16711	-1.16356	-1.08147	0.692791	0.013849	0.426153	0.052554	0.926288	-1.02713	-0.47241	-0.08971	-0.26367
O95171	SCEL	-0.37135	-1.43206	-1.6505	-1.76325	-3.51346	-2.09312	-0.76651	-0.55805	-1.42026	-1.35333	-0.56643	-2.05262	-1.48861	-0.92736	0.058581	-1.23891	-0.51419	-1.95435	-3.49815
O95182	NDUFA7	-0.47225	-0.48933	-0.55034	-1.29402	0.806552	-0.36367	-0.19084	-0.2904	0.467045	0.087825	-0.12269	0.237881	-0.20132	-0.13742	-0.11376	-1.42644	-0.33087	-0.95405	-0.76449
O95183	VAMP5	0.341376	1.417126	0.931268	-0.66658	0.0641	-0.37152	1.156194	-1.03124	-0.11159	-0.34874	-0.53889	1.489585	0.319924	1.387582	-0.30232	0.305155	0.459836	0.782575	-0.64133
O95197	RTN3	0.369817	0.56151	0.177683	-0.30282	-0.61457	-0.41615	0.260971	0.430155	-0.49299	0.055796	-0.52112	0.746822	0.197555	1.046586	0.313976	0.409306	0.058898	-0.52184	-0.21476
O95199	RCBTB2	-0.94472	0.245097	0.158159	-0.73514	-0.52252	-0.3613	0.108479	-0.26113	0.360181	0.156383	-0.1695	-0.41371	-0.02129	-0.34454	-0.14106	-0.42838	0.05111	-0.237	0.880685
O95202	LETM1	1.295395	0.167071	0.685615	-0.16003	-0.67072	-0.79633	-0.07401	-0.45657	-0.44175	0.630919	0.453878	0.033813	-0.5261	-0.13691	0.302709	0.279866	-0.50436	-0.3958	-0.53257
O95208	EPN2	-0.03738	-0.11581	0.021108	-0.1928	-0.00759	-0.74996	-0.11345	0.493199	-0.38404	-0.46579	0.289302	-0.50019	-0.07782	-0.57752	-0.41915	-0.17806	-0.44099	-0.18246	-0.18633
O95210	STBD1	-0.08084	-0.41613	-0.04472	-1.69177	-0.5975	-0.50012	0.624149	-0.05249	0.098955	0.72024	-0.5286	0.019228	0.288574	0.766474	-1.28567	-0.30881	0.915918	0.23866	-0.19632
O95218	ZRANB2	0.861553	-0.67498	0.472861	0.456764	0.2578	0.669731	-0.86052	0.303781	-0.54937	-0.25638	0.978068	-1.16135	0.411734	-1.34094	0.483419	0.086359	-0.7727	-0.1096	-0.04979
O95219	SNX4	0.284561	0.495379	0.019036	-0.07336	0.582244	0.578211	-0.05285	-0.30928	0.310934	0.325094	-0.10261	-0.02974	0.355665	0.059074	-0.80935	0.448631	0.076087	-0.03517	0.683947
O95232	LUC7L3	0.642913	-0.35698	0.164981	0.359754	0.271877	0.413755	-0.06885	0.800497	-0.85214	-0.05863	0.515209	-0.62222	-0.37841	-0.45976	0.454329	-0.15843	-0.16592	0.327466	-0.09579
O95235	KIF20A	0.440971	-0.07217	0.146873	-0.1984	0.03107	0.452497	0.372998	0.507	0.401788	-0.23517	-0.695	-0.57538	-0.07039	-0.03905	-0.5873	-0.19612	-0.24501	-0.51089	0.142484
O95236	APOL3	-1.85574	1.69695	1.689163	-0.92903	0.456547	-0.08296	0.526033	-1.81976	-0.4469	-0.21361	-0.9706	0.123248	0.126814	0.226899	-1.79257	0.152768	0.836293	1.118192	-1.71444
O95239	KIF4A	1.011183	-0.1225	-1.38592	0.153065	1.718679	1.923086	0.428961	1.655639	0.124782	0.445829	-0.71983	-0.45737	-0.5734	0.135752	-0.34702	0.299296	0.147916	0.438761	-0.15358
O95248	SBF1	-0.6511	0.616044	0.732686	-0.8002	-0.03712	-0.24938	0.029201	-0.89965	0.041733	-0.16317	-0.47663	-0.27054	0.101039	0.047574	-0.10508	-0.31136	-0.00855	0.587715	-0.22018
O95249	GOSR1	-0.66723	0.484556	0.653543	0.44212	-0.40604	-0.01305	0.757195	0.310875	0.01118	0.156254	0.188085	-0.39039	0.35709	-0.37707	0.163222	0.341309	0.489329	1.687195	0.448786
O95251	KAT7	0.324537	0.34102	-0.56496	-0.09993	0.116776	0.373489	0.713133	1.122962	-0.64186	-0.04819	0.373427	-0.2508	0.177547	0.204844	-0.08969	0.169253	-0.01659	0.474616	-0.56312
O95260	ATE1	-0.55305	0.387592	0.13514	-0.0285	-0.127	-0.33391	0.482641	0.263216	0.552742	0.305004	-0.14043	-0.03619	0.278803	0.290324	1.222555	-0.59361	1.251146	1.050043	-0.29397
O95273	CCNDBP1	0.261537	0.046605	0.040934	-0.02855	-0.38493	0.311237	0.028046	0.081386	-0.17211	-0.0166	0.566333	0.592031	0.195459	0.380829	-0.21035	0.12813	1.126544	0.27802	0.204531
O95292	VAPB	0.315574	-0.21327	-0.20929	0.103031	0.160746	0.296595	0.148022	0.332887	-0.15612	0.162857	-0.17576	-0.87271	0.091046	-0.95655	1.293865	0.531511	0.463422	0.588328	0.206616
O95294	RASAL1	0.512782	-0.98535	-1.1663	1.684454	-1.99262	-0.83685	-0.94176	0.702666	-0.60896	-1.01611	0.152623	-0.82782	-0.65184	-0.47324	1.561895	-0.24174	-0.20644	-1.69157	-1.43499
O95295	SNAPIN	0.74952	0.268183	0.794206	-0.51549	-0.27111	-0.10207	-0.1725	-0.33251	0.238451	0.276473	0.285455	-0.28017	-0.17754	0.211255	-0.16833	0.369167	0.036993	-0.47784	-0.60111
O95297	MPZL1	0.327379	0.379579	0.178348	-0.60529	-0.32466	-0.16998	-0.3153	-0.29947	-0.45133	-0.17257	-0.66851	-0.15768	-0.24208	-0.41862	-0.05864	-0.02046	-0.68372	0.190186	0.438503
O95298	NDUFC2	0.066333	0.2331	0.193121	-0.44519	0.328733	0.190552	0.469171	-0.35248	-0.75737	-0.85969	0.682441	-0.10754	0.042536	0.158143	0.662674	-0.16619	-0.65837	-0.11424	-0.38797
O95299	NDUFA10	0.350204	0.048185	-0.0743	-0.49688	0.369642	0.224873	-0.38186	-0.53772	-0.67036	-0.70569	0.748606	0.333297	-0.3047	0.251033	1.142008	-0.38113	-0.18742	-0.29474	-0.23875
O95302	FKBP9	0.702856	-0.89194	-0.17917	-0.6847	0.574328	-0.3028	-0.28172	0.737412	0.421396	0.812994	-0.1495	-1.44176	0.84648	-0.68528	-0.44851	0.728924	0.520462	1.402673	1.237137
O95319	CELF2	-0.3254	0.589095	0.097533	-1.62397	0.34213	-0.02563	-0.16103	-0.60413	-0.06069	1.02226	-1.24641	1.332876	-0.14726	1.337497	-0.17406	-0.44752	0.127163	0.117791	-0.61694
O95336	PGLS	-0.81073	-0.24592	-0.02068	-0.09973	-0.96897	-0.44414	-0.09884	0.961654	-0.39352	-0.19451	0.077624	-0.7844	-0.35204	-0.30462	-0.55839	0.263687	-0.34563	-0.17474	-0.13505
O95340	PAPSS2	-1.08031	1.076765	0.568912	2.169751	0.03729	-0.12302	0.210479	-0.80921	0.161046	0.202337	-1.05236	1.202654	0.346568	1.323702	-1.22638	1.302293	0.477622	0.207191	0.958771
O95347	SMC2	1.309906	-0.22674	0.431976	0.363471	1.518751	1.863371	1.151157	1.459095	0.018679	-0.39964	-0.61343	-0.52418	-0.14133	-0.50875	-0.67124	0.232825	0.122023	-0.66471	0.34999
O95352	ATG7	0.18527	0.635355	0.393226	-0.27673	0.645504	0.512374	-0.03742	-0.21666	-0.48761	0.602197	-0.39455	0.508284	0.167515	0.915631	-0.40681	-0.10513	-0.36164	-0.49235	-0.01436
O95359	TACC2	-0.36587	-0.32201	-0.21574	-0.16381	-0.89683	-0.45207	-0.31776	0.541551	-0.37623	0.054717	0.301192	-0.96156	-0.24059	-0.05596	2.164584	-0.54594	0.128722	-0.33563	-0.83971
O95361	TRIM16	-0.86563	0.417435	-0.16803	0.256851	-0.66452	-0.97408	0.314577	0.497472	0.615335	-0.29806	-0.56949	-0.199	-0.21656	0.062504	-0.59525	-0.52606	-0.53839	-1.11639	-0.3168
O95363	FARS2	0.33094	0.543998	-0.36996	0.903089	0.630515	0.250968	-0.44076	0.119522	-0.2722	-0.0343	0.21446	-0.25292	-0.40305	0.106605	0.643187	1.474982	-0.57695	-0.54867	-0.07064
O95365	ZBTB7A	-0.28586	0.142683	-0.1895	-0.13972	0.24094	-0.07026	-0.16793	-0.75968	0.220642	-0.2585	0.309009	0.00333	-0.02703	0.194784	0.007671	-0.45009	0.145728	-0.14523	-0.689
O95372	LYPLA2	0.484226	-1.06102	-0.14854	-0.03963	0.192346	-0.28078	-0.78062	0.629442	0.167527	0.961404	0.357947	-0.21585	0.444752	0.285867	0.493372	-0.63907	0.470083	-0.34306	-0.1669
O95373	IPO7	0.181019	0.104584	0.205631	-0.11195	-0.28669	-0.0021	0.253389	0.433608	-0.17864	0.083627	0.016577	-0.2315	0.206202	-0.40513	0.412851	-0.10957	0.430359	-0.35328	0.202245
O95376	ARIH2	0.409562	-0.36071	0.059996	0.127543	0.373707	0.964737	0.26876	0.299166	-0.4533	-0.39068	-0.72078	0.39141	-0.50365	0.490827	0.0949	-1.18353	-0.04977	-0.53147	-0.20891
O95379	TNFAIP8	-1.08957	1.319389	1.115046	-0.0562	0.876736	0.242693	0.155243	-1.31497	-0.2451	1.005187	-0.92611	0.515522	-0.83147	1.012502	-0.48226	-0.30567	-0.39852	0.269897	-0.24347
O95382	MAP3K6	-0.85112	0.453575	0.198939	-0.23421	-0.23054	-0.08088	0.04562	-0.65688	0.068641	0.532957	-0.0779	-0.22741	0.019483	-0.10369	-0.69251	-0.35665	-0.48971	0.085221	0.144276
O95391	SLU7	-0.06632	0.208381	-0.2524	0.487242	-0.32894	0.259361	-0.32038	0.28795	-0.3166	0.086531	0.589438	-0.04784	-0.17141	-0.36896	0.687747	0.177822	-0.15227	0.093316	-0.18599
O95394	PGM3	0.247541	-0.00666	0.222007	0.114834	0.500515	0.101691	0.206631	-0.26117	0.493671	0.608092	-0.87256	0.087501	1.402781	-0.5787	0.085829	0.583687	0.765935	0.543972	1.413288
O95396	MOCS3	-0.03947	-0.43293	-0.82137	0.169749	-0.13827	0.408088	0.14705	-0.43102	0.264059	-0.5859	-0.0527	-0.46551	-0.18004	-0.10457	0.328039	-0.28238	0.174691	-0.19459	0.354042
O95400	CD2BP2	0.975706	-0.1637	0.19267	0.642459	0.053113	0.351389	-0.04754	0.722147	-0.09795	0.219511	0.258561	-0.68515	0.177095	-0.63607	0.163789	-0.11326	-0.19733	-0.11141	-0.25182
O95425	SVIL	-0.52126	-0.26964	0.020393	-0.91467	-0.94575	-1.04154	-0.20608	-0.70398	-0.15727	-0.21967	-0.44678	-1.72133	-1.00306	-0.53515	0.401733	-0.29687	-0.6663	0.519124	-0.07075
O95428	PAPLN	-1.13163	0.004467	-0.09236	-2.46259	0.211898	-0.71964	0.151274	-0.90878	1.401494	-0.08884	-0.42949	-0.69858	-0.7038	0.231376	-0.51096	-1.21394	-0.08416	1.651667	-0.41789
O95429	BAG4	0.975348	-0.04902	0.021314	-0.35093	-0.26499	0.259936	-0.05467	-0.17593	-0.11671	0.316218	0.185481	-0.07357	-0.13484	-0.04128	1.081874	0.004112	0.863385	-0.34753	-0.10853
O95433	AHSA1	-0.04563	-0.13348	-0.65606	0.369689	-0.17251	0.422193	-0.38174	0.269371	0.02364	0.100067	0.090873	-0.29259	0.566894	-0.06635	0.078422	0.81996	0.071047	-0.37835	0.135509
O95436	SLC34A2	0.195872	-1.0528	1.746875	-0.09459	-2.15637	-1.22395	-0.69744	0.49811	0.226855	-0.75952	-1.02069	-0.72147	1.450998	-0.59634	0.299439	1.535036	-1.23311	-1.26785	-0.22653
O95445	APOM	-0.37314	0.485341	0.204229	-0.90976	-0.09718	-0.30308	0.00989	-0.18044	0.863671	0.051374	-0.30318	1.000752	-0.20911	0.884188	-0.16546	-1.14759	0.124218	0.701928	-0.07592
O95453	PARN	0.666289	0.031616	0.035745	0.136548	0.553852	0.257893	-0.30372	-0.12364	0.178943	-0.29886	0.463034	0.007839	0.177132	-0.20615	0.383814	-0.0932	0.112882	-0.01247	-0.33297
O95456	PSMG1	0.643284	-1.05811	0.342729	-0.42234	2.151872	1.275913	-0.07482	0.612141	-0.21537	-0.40157	-0.12516	-0.48209	0.059051	-0.28999	0.28342	1.199929	0.880629	-0.50204	-0.62932
O95466	FMNL1	-1.96661	1.356161	1.275987	-0.71064	0.534535	0.012337	0.218045	-1.61495	-0.30719	1.749302	-1.50683	1.391873	-0.06599	1.955102	-0.55558	-0.61794	0.512364	0.19145	-1.35399
O95470	SGPL1	-0.53792	0.109727	-0.20271	-0.72414	0.106149	0.240345	-0.30872	0.854632	-0.53611	-0.6341	-0.12974	0.589465	0.11092	-0.10994	0.265018	0.768492	0.205122	-0.71338	0.147435
O95477	ABCA1	-1.13121	1.285753	0.848316	-0.56088	-0.01416	-0.17208	0.432562	-0.2784	0.119732	0.461995	-1.09406	1.633604	0.556923	1.299506	-1.02475	-0.57824	0.497197	-0.11302	-0.56741
O95479	H6PD	-0.40022	0.254716	1.467016	0.394889	0.033076	-0.10804	-0.36502	-0.36187	0.324722	0.617599	0.497407	-0.39252	0.17069	0.217809	-0.17932	-0.33287	-0.51532	-0.23996	-0.18672
O95486	SEC24A	-0.81567	0.606484	0.738085	-0.12165	-0.31453	-0.13646	1.93019	-0.59435	0.011483	-0.13878	-0.44479	0.119818	0.449615	0.114787	0.716189	0.225836	0.747023	0.636143	0.413606
O95487	SEC24B	-0.57203	0.481026	0.458863	-0.63674	-0.07274	-0.12514	0.465286	0.097269	-0.66987	0.174295	0.34761	0.162337	0.062119	0.235375	-0.02988	0.096502	0.1437	0.371588	-0.12978
O95490	ADGRL2	0.250038	0.003767	-0.46622	1.418787	0.21723	-0.19306	-0.75288	-0.38484	0.791906	-0.6987	-0.89749	-0.59859	-0.19952	-0.04432	-0.49484	1.83424	-0.46978	0.149677	-0.75418
O95498	VNN2	-1.2848	1.004226	0.339426	-0.9561	-1.75728	-0.87436	0.172389	-2.18986	-0.09822	-0.87509	-0.81139	0.164747	-0.14242	1.928011	-0.38371	-2.30124	0.912438	-0.4024	-0.06816
O95503	CBX6	-0.16677	0.157785	-0.01759	-0.48534	-0.4499	-0.02413	0.134758	0.250173	-0.23601	-0.93142	0.409022	0.029626	-0.15915	-0.00861	-0.34542	-0.61455	-0.01951	0.430597	-0.29973
O95528	SLC2A10	-0.09277	0.477433	0.480746	-0.33785	0.050855	0.044487	0.463112	0.070301	0.00118	-0.48637	0.144429	0.095775	0.395823	-0.16536	-0.86697	-0.28947	1.201985	1.045322	1.404242
O95544	NADK	-0.6264	0.892665	0.802672	-0.61701	1.154109	1.063665	-0.0379	-0.41139	-0.02627	0.638577	-0.09959	0.553328	0.386778	1.128456	0.017915	-0.51491	0.901923	0.711544	-1.38049
O95551	TDP2	0.702998	-0.14565	-0.59384	0.784452	0.595407	0.549062	-0.15869	0.505431	-0.07315	-0.32605	-0.22623	0.080688	-0.0134	0.494401	0.017599	-0.06219	0.210974	-0.42749	0.816789
O95562	SFT2D2	-0.07522	0.209811	-0.05024	-0.39237	-0.26486	-0.32267	0.606577	0.307774	0.040847	-0.70038	-1.74445	0.807819	-0.02836	0.205941	1.164157	-0.32259	-0.00266	-1.85392	-0.63725
O95571	ETHE1	0.518813	-0.25126	0.042276	0.055038	0.859664	-0.27643	-0.33745	-0.1409	0.332665	0.894949	-0.15081	0.726386	0.559102	1.083994	-0.11719	-0.23525	-0.18911	0.185643	-0.66508
O95573	ACSL3	-0.17299	0.287386	0.348181	-0.14533	0.902501	0.774985	0.77312	0.059951	0.21592	-0.01949	-0.05357	1.013484	0.153763	0.603291	-0.3008	0.269625	0.958471	0.18803	-0.40863
O95602	POLR1A	0.32991	0.212905	-0.31506	0.10718	0.151259	0.552359	-0.30218	0.682691	0.046671	0.276788	0.214701	-0.22026	-0.03706	-0.18556	0.085775	-0.28952	-0.14747	-0.17504	-0.05698
O95613	PCNT	-0.01549	0.423338	0.170035	-0.38436	0.82057	0.77362	0.159472	0.125473	0.453322	0.004496	-0.01968	-0.11161	0.047303	-0.37653	0.875294	0.369415	-0.23515	0.335302	-0.2116
O95619	YEATS4	0.775801	-0.08175	0.085121	0.028021	0.464759	0.256382	-0.16328	0.282635	-0.31979	-0.05739	0.336837	-0.44	-0.19756	0.090217	0.341794	-0.20729	0.049395	-0.01108	0.043871
O95625	ZBTB11	0.487203	-0.17939	0.058135	0.021309	0.091653	0.558774	-0.08416	-0.07829	-0.21117	0.205394	0.042408	-0.21012	-0.13875	-1.16019	0.663273	-0.37812	-0.14296	0.181853	-0.07609
O95628	CNOT4	0.397749	-0.69684	-0.00954	0.282283	-0.13318	0.036751	-0.43516	0.335815	0.467953	0.510416	0.182154	-0.03155	0.653842	-0.14388	0.8603	0.292361	-0.35276	-0.26498	0.756115
O95630	STAMBP	-0.07187	-0.04154	0.303857	0.51736	-0.09711	0.104198	-0.61148	0.411374	0.061716	0.415234	0.348176	-0.32206	-0.12332	-0.21619	0.612803	0.877048	-0.53395	-0.66301	-0.08231
O95639	CPSF4	0.005258	0.023314	-0.19824	-0.42996	0.311566	0.150963	0.090408	-0.56721	-0.01437	-0.52597	-0.11193	-0.1007	0.093451	-0.86674	-0.79374	-0.86215	0.656597	-0.14552	0.209129
O95644	NFATC1	-0.46256	-0.03479	0.273358	-0.80344	0.056845	0.258876	0.012638	-0.23921	0.30133	-0.16925	0.065529	0.047195	-0.08026	-0.07796	-0.29106	0.044215	0.229795	0.251435	-0.39094
O95671	ASMTL	-0.2699	-0.01587	-0.98865	0.056732	0.40048	0.244189	-0.66766	-0.5954	0.408933	0.349117	0.011496	-0.34823	-0.61113	0.089062	-0.40007	0.150172	0.713592	0.76264	0.219662
O95674	CDS2	0.087475	0.425312	-0.31296	-1.19722	0.084031	-0.61416	0.178321	-0.45973	0.777852	-0.7372	0.042403	0.239684	-0.47805	0.076244	0.022765	-1.1535	-0.1558	0.219673	-0.38802
O95678	KRT75	-1.84449	0.362427	-0.10342	0.822315	-1.57703	-1.99034	0.079374	-0.93762	0.523334	-0.0878	-2.24356	-0.49742	-0.14955	-0.66449	-2.52949	-1.23557	-1.1385	-3.70686	-1.00431
O95684	FGFR1OP	-0.66951	-0.07813	-0.02141	0.033262	-0.36175	0.354705	1.256616	-0.48198	0.131527	0.566225	0.062305	-0.13475	0.369783	-0.07139	0.739699	-0.30173	0.205295	0.671541	-0.3901
O95696	BRD1	0.052635	0.363578	0.411297	-0.21939	0.119501	0.274855	0.706156	-0.04846	-0.30801	-0.57322	0.705271	-0.0438	-0.06962	-0.31902	-0.53079	-0.19889	-0.53804	1.309443	-0.71227
O95714	HERC2	0.076781	-0.17707	-0.53449	-0.21921	0.214715	0.459342	-0.24764	-0.01039	1.229322	0.039411	0.195179	-0.0399	0.385527	-0.03274	-0.39764	0.294261	0.059563	-0.47183	-0.13227

O95716	RAB3D	-0.50336	-0.91998	0.473071	-0.5532	-0.38437	-0.72861	0.133438	0.017309	-0.3708	0.841311	0.812455	0.856907	0.048012	1.980957	-0.20035	0.122576	0.888567	-0.47469	-0.84457
O95721	SNAP29	0.274894	0.732781	0.239918	-0.27294	-0.31729	-0.37324	0.176852	-0.98196	0.434818	0.077624	-0.02987	0.467551	0.419634	0.749326	-0.85626	0.351658	0.352671	0.394375	-0.8047
O95747	OXSR1	0.909141	0.293877	0.114477	-0.62992	-0.51516	-0.40688	-0.33014	-0.27393	-0.29568	-0.26397	0.096921	0.184236	-0.17169	0.223156	-0.41579	-0.43489	-0.05868	-0.43577	0.357461
O95749	GGPS1	0.051184	-0.14389	0.415342	0.484926	-0.48918	0.243722	-0.21381	1.124775	0.040349	0.440212	-0.2624	-0.73551	-0.15174	-0.4627	0.766508	-0.02785	-0.42494	-0.72252	0.394464
O95757	HSPA4L	0.444805	-0.25255	-0.99901	-0.59287	0.121592	0.947748	0.306443	-0.12746	-0.60302	-0.94729	0.9046	-0.72989	-0.62715	0.020963	-1.00835	0.779701	-0.70817	-0.90755	-0.11436
O95758	PTBP3	-0.35111	0.769856	0.745758	-1.7753	0.273004	0.601723	0.326386	-0.02691	0.166093	-0.59296	-0.71232	0.164668	0.497831	0.648917	0.582304	-0.34316	0.636006	0.730733	-0.0414
O95759	TBC1D8	-0.43649	0.207749	0.103494	0.356637	-0.05629	-0.27533	-0.04443	-1.23198	0.240272	-0.57898	-0.12192	0.396163	0.096358	0.965529	-0.44196	1.698222	0.673029	0.610316	-0.59037
O95772	STARD3N	0.25254	0.572439	0.773395	0.04887	-0.06077	-0.31706	0.565385	0.338923	0.357533	0.46017	-0.20788	1.067559	0.178648	0.656646	-0.42697	0.258376	0.296798	-0.21403	-0.56822
O95777	LSM8	0.040098	-0.28237	-0.05678	0.017694	0.631503	0.346191	-0.5316	0.163659	0.381293	0.349555	0.295471	-0.54121	0.050276	-0.25822	0.089725	0.553592	0.359106	0.421182	-0.05241
O95782	AP2A1	-0.33448	0.387432	0.561201	0.047162	-0.05796	-0.44076	0.493849	-0.24016	-0.32993	-0.27816	-0.32524	-0.22301	0.067293	0.025514	0.548957	-0.30073	-0.56811	-0.05597	0.42022
O95785	WIZ	-0.03811	-0.09192	0.233851	0.202592	-0.64179	-0.22468	0.884745	0.859193	-0.2386	-0.84046	-0.14633	-0.68954	-0.18114	-1.01542	0.662271	0.32129	-0.04105	0.397575	-0.01025
O95786	DDX58	0.948495	0.684981	2.235338	-0.10769	-0.59837	-0.20235	-0.10707	1.017813	-0.58227	-0.50599	-0.84414	-0.06577	0.228326	0.40589	-0.81857	-0.77666	-0.20375	-0.19222	-0.90815
O95793	STAU1	0.430804	-0.2614	-0.17545	0.133475	-0.35319	0.107711	-0.66232	0.547782	0.070838	0.498827	-0.13206	-0.78353	0.018168	-0.73934	0.023363	-0.19965	0.045807	0.127944	0.593732
O95801	TTC4	0.831161	-0.26189	0.197924	0.44816	0.771486	1.244429	-0.36479	0.228165	-0.33679	0.669725	0.541968	-0.283	-0.42407	-0.59332	0.049698	0.506964	0.082065	0.350567	-0.57444
O95810	CAVIN2	-2.35864	0.020712	0.151406	-1.58972	-0.64179	-1.68724	-0.24757	-2.04011	0.456632	-0.79139	-1.07237	-1.4839	-0.77257	-0.903	-0.00687	-1.00368	-0.83638	2.057386	-0.86351
O95816	BAG2	-0.95709	0.004755	-0.73483	-1.88208	-0.35967	0.34014	0.203289	-1.44999	0.125541	-0.891	-0.75332	-0.13758	0.977947	-0.91337	-1.95343	-0.35871	-1.15323	0.903988	1.569782
O95817	BAG3	-0.98151	0.15497	0.147479	-0.98404	-0.10065	-0.80829	-0.28179	-0.76807	0.497062	0.050089	-0.11346	-0.33682	0.87341	-0.67055	-0.14691	-0.30278	-0.19686	0.236586	1.145047
O95819	MAP4K4	-0.50799	0.288289	0.789028	-0.52944	-0.40262	-0.19445	0.543616	0.260232	0.073276	-0.13813	-0.71602	-0.68369	0.580893	-0.08843	-0.1228	0.011728	-0.1524	0.876998	0.876719
O95822	MLYCD	-0.55153	-0.23862	-0.25289	-0.04484	-0.3642	-0.74384	-0.04331	-1.35075	-0.20286	-0.05731	0.609226	-0.24842	-0.60631	-0.5516	0.438875	0.876796	-0.86148	-0.32302	-0.47875
O95825	CRYZL1	0.031891	-0.5893	0.065675	-0.12064	0.524215	0.230997	0.65348	-0.14315	0.367082	-0.1958	0.098345	-0.21633	0.530647	-0.28835	-0.4256	0.357986	0.352089	0.239426	0.277511
O95831	AIFM1	0.627708	-0.05081	0.713134	0.625061	1.65371	1.394671	-0.13579	0.365833	-0.67834	0.050092	0.867735	-0.54109	-0.54465	-0.65902	0.412018	1.054467	-1.00133	-0.77755	-0.60019
O95833	CLIC3	-0.66101	-0.66313	-0.74605	0.302968	0.217695	-0.08232	-0.44577	0.747883	0.245239	-0.02972	-0.77245	-0.05009	-0.59152	-0.4756	0.609966	-0.39687	2.368095	1.355771	0.024747
O95834	EML2	-0.73231	-0.48304	-0.17498	-0.04177	-0.34991	0.189041	-0.42664	-0.41127	0.00402	-0.29378	0.672055	-0.7682	0.469008	-0.34153	1.774041	0.197481	-0.51812	-0.17411	-1.14204
O95837	GNA14	-0.83012	-0.01843	-0.57326	-0.7536	0.030489	-1.39668	0.425359	-1.57157	0.240633	-1.09681	-0.18528	-0.10549	-0.45174	0.123233	-0.05631	-1.01332	-0.29902	-0.40754	-0.4319
O95848	NUDT14	0.074152	-0.86828	-0.57104	0.070647	0.763493	-0.04238	-0.09572	2.142622	0.43363	1.104192	-0.41317	-0.0297	-0.05025	-0.0805	0.123117	0.147459	-0.13568	0.116122	-0.79604
O95858	TSPAN15	0.059302	-0.11165	-0.17061	1.532738	0.559196	0.32357	-0.35311	-0.70855	0.34651	-0.17921	0.480424	-0.57521	0.019091	-0.67748	0.01922	0.815712	-0.09893	-0.85461	-0.30521
O95861	BPNT1	-0.07404	-1.29346	-1.5542	-0.61441	-0.31528	-1.05591	-0.93618	1.187784	0.80619	0.903731	-0.49728	-0.59502	0.224434	-0.12155	0.634331	0.373427	-0.48797	-1.43211	-1.33051
O95864	FADS2	-0.23536	-0.7984	-0.02039	-1.58302	-0.26378	-0.01903	1.353253	0.724028	0.040847	-0.75903	-0.89676	-0.09627	1.423639	0.070314	-0.00793	-1.48826	-0.4883	-0.84082	2.052505
O95865	DDAH2	-0.68678	-0.67262	-1.46175	-1.18585	-0.49249	-1.07702	-0.40197	1.042075	-0.4511	-1.26205	-0.32047	-0.49866	-0.19067	-0.81217	-0.06051	-1.25165	-0.18555	0.052311	0.214297
O95870	ABHD16A	-0.26884	0.701599	0.194264	0.030462	-0.01745	-0.01453	0.114962	-0.26392	-0.37692	-0.00608	-0.10722	0.364	0.327705	0.298847	-0.12197	-0.32358	0.849324	0.370069	-0.21533
O95881	TXNDC12	-0.16774	0.01192	-0.38565	0.023337	1.151597	0.296152	-0.46533	0.561182	0.255574	1.88197	-0.25935	-0.07089	0.517806	-0.18445	-0.1881	0.583711	-0.09139	0.35276	-0.04724
O95905	ECD	0.187794	-0.42479	0.020547	-0.22523	0.197697	0.191629	-0.47758	-0.45166	-0.16563	-0.13644	-0.38946	-0.40556	0.552764	-0.1064	-0.53423	0.170329	0.981299	0.523563	0.340314
O95926	SYF2	1.115894	-0.47991	-0.29396	-0.10923	0.51434	0.552667	-0.40926	0.554143	-0.36462	-0.51064	0.072412	-0.39555	-0.15524	-0.40142	0.054459	-0.49426	-0.42138	-0.18157	-0.50216
O95967	EFEMP2	-1.88453	-0.88383	-0.41696	-1.33369	-1.44899	-1.64128	0.196325	-0.44438	0.933905	-0.40035	0.189162	-0.89852	-0.27537	-0.82954	-0.2428	-0.48089	-1.06255	-0.9773	0.220403
O95983	MBD3	0.857171	-0.15959	0.075226	-0.20852	0.144309	0.233235	1.500932	1.008453	0.229736	-0.12428	-0.69197	-0.17955	-0.05822	-0.60243	0.331102	-1.62119	-0.70883	-0.60747	0.139038
O95985	TOP3B	0.115599	-0.27175	-0.052	-0.49577	-0.21764	-0.05083	-0.11344	0.046642	-0.0776	-0.90662	0.655761	-0.34176	-0.13576	-0.13768	-0.07589	-0.05434	-0.12917	0.379392	-0.19937
O95989	NUDT3	-1.21643	-0.07284	-0.21415	-1.53762	-0.24782	-0.77694	-0.05137	0.543758	0.735598	0.916408	-0.35287	-0.41957	-0.44144	-0.03431	0.796487	-0.77011	0.498855	0.081511	-0.21995
O95994	AGR2	-0.74877	-0.02987	-0.15327	-0.16671	-4.02328	-1.21871	0.355473	-3.12375	-0.50669	-3.0608	3.046181	-0.83237	-1.03108	-0.47571	-1.87205	-2.0031	1.002793	0.699653	-2.10637
O95999	BCL10	0.561482	0.327022	0.250189	0.086194	-0.02133	-0.16081	-0.1459	0.110003	-0.05263	-0.03684	0.068625	0.31611	0.023833	0.7622	-0.75859	-0.06781	0.501623	-0.21359	-0.1441
O96000	NDUFB10	0.067636	-0.28308	0.151874	-0.26129	0.274838	0.358218	0.571814	-0.46281	-0.12805	-0.67385	0.228558	-0.3511	-0.35026	0.008655	0.858664	-0.51192	-0.35455	-0.02412	-0.50855
O96005	CLPTM1	-0.121	0.078	0.027966	0.061349	0.272757	0.762817	1.028795	0.703267	-0.17673	-0.4512	-0.53476	0.292717	0.698817	0.301114	0.926951	-0.44201	0.235185	-0.20759	-0.25109
O96006	ZBED1	-0.22815	0.197345	-0.06096	-0.27877	0.243817	-0.08169	-0.16815	0.092248	-0.26543	-0.80615	0.293445	-0.029	0.170062	0.444028	-0.722	-0.05259	0.56556	1.927886	-0.38567
O96008	TOMM40	0.269413	-0.13595	-0.28065	0.203448	0.051076	0.440918	0.286935	0.388563	-0.3424	-0.32512	0.058581	0.473794	0.460064	0.244138	0.585752	0.242515	0.402884	-0.12455	-0.56388
O96011	PEX11B	-0.36893	0.253526	0.444069	2.160848	0.157993	0.483223	0.467132	0.38577	-0.12432	0.101592	-0.33599	0.317792	-0.58269	0.420877	0.739235	0.196995	-0.22411	-0.53524	-0.64774
O96013	PAK4	0.482736	-0.12926	0.361818	-0.26931	0.111739	0.515378	1.992896	3.134152	0.139506	-0.84431	0.183043	-0.3566	-0.19695	-0.34179	0.767645	-0.91377	-0.18004	-0.70329	2.190597
O96017	CHEK2	-0.30175	0.097188	-0.34055	1.058591	0.049958	0.010767	0.32384	0.710272	0.218677	0.004895	-0.67778	0.103282	-0.22324	0.421619	-0.5734	0.376201	0.9704	-0.62406	-0.32714
O96018	APBA3	-0.27327	0.267137	0.083385	-0.64351	0.292677	-0.08747	0.066497	0.138584	0.160556	0.261338	-0.25788	-0.00659	0.116313	-0.09706	0.299368	-0.66681	0.521441	-0.13381	0.153515
O96019	ACTL6A	0.838891	-0.47234	-0.42337	-0.01785	0.538082	0.810067	-0.71216	0.492513	0.61896	0.237994	-0.1037	-0.39591	-0.31989	-0.22857	0.491493	-0.18149	-0.57707	-0.1853	0.132059
P00167	CYB5A	-0.37991	-0.9383	1.211171	-0.19437	-0.98471	-0.58637	0.09024	-0.65089	-0.07055	0.37462	0.048293	-1.5223	-0.98375	-0.8537	0.408804	1.213054	0.654517	0.856273	-0.3559
P00325	ADH1B	-3.14913	-2.24706	-0.46678	-3.77035	-2.39727	-5.00808	0.963135	-3.52136	0.331645	-2.28823	-3.41811	-1.8143	-2.45015	-1.05808	0.834647	-2.32109	-1.95424	1.624437	-2.60926
P00338	LDHA	-0.53513	0.429839	0.735365	0.457038	-0.64798	-0.2302	0.051538	0.82247	0.004807	-0.167	0.247227	-0.78861	0.802273	-0.43118	-1.22143	0.524367	0.251047	-0.41593	0.948573
P00352	ALDH1A1	-0.66959	-1.25168	0.122625	-2.20142	0.731153	-1.43132	0.966664	-1.58773	1.401494	0.015514	-0.41528	-0.45164	-1.37245	-0.42135	0.139319	-0.97209	-0.75149	0.674473	0.031098
P00367	GLUD1	0.218411	-0.2732	0.733219	0.106065	0.550814	0.426711	-0.97492	-0.43995	-0.07357	0.30784	0.51164	0.246395	-0.06409	0.127504	1.213954	0.90853	0.174446	1.216	-0.52567
P00374	DHFR	-0.41738	-0.55331	-0.35984	-0.14821	2.267366	2.020376	0.16535	-0.08305	0.287126	0.004941	-0.21802	0.24929	0.082096	-0.46994	-0.91853	0.228599	0.799482	0.391533	-0.69107
P00387	CYB5R3	-1.00827	0.231428	0.035842	-0.80298	-0.3013	-0.47817	0.031255	-0.86729	-0.31984	-0.36024	-0.27552	-0.08055	-0.46449	0.060624	-0.0647	0.391384	0.40692	0.601233	0.251249
P00390	GSR	0.629514	-1.33145	-0.03506	0.051305	0.147007	0.106143	-0.64178	-0.13542	-0.01022	-0.37385	0.444734	-0.2412	-0.01704	0.031826	-0.20629	0.278552	0.938181	0.991437	0.092634
P00403	MT-CO2	-0.40132	0.305947	0.205027	-0.08481	0.794498	0.762893	0.326992	-0.38	-0.58571	-0.5875	0.464905	0.291718	-0.1949	0.177203	1.504063	0.586834	-0.43402	-0.3798	-0.32651
P00441	SOD1	-1.21922	0.282815	-0.413	-0.90702	2.040118	-1.18309	-0.13718	-1.50623	2.539026	-0.69573	-1.7722	-1.01418	-0.47288	-0.70956	0.113403	-0.81288	-0.05978	-0.25009	-1.29636
P00450	CP	-0.86504	-0.91228	-0.57079	-0.87675	0.392277	-0.18444	0.085987	-0.10419	1.110858	0.138302	0.378416	0.558442	0.378587	1.721642	-0.09735	-0.62963	0.167327	0.132421	-0.48527
P00488	F13A1	-2.28343	-0.78695	0.7722	-2.0564	0.51576	-0.41882	1.34036	-1.4593	0.693919	-0.09792	0.088979	-0.25589	-1.08528	-0.38964	0.781682	0.353155	-0.74618	0.721618	-0.76369
P00491	PNP	-0.01305	1.008623	-0.52822	-0.71383	0.373548	0.220557	-0.08347	0.106715	0.40422	0.656874	-0.77942	-0.49847	0.023345	0.217022	-1.32709	-0.45585	0.497041	1.100104	-0.58026
P00492	HPRT1	1.066473	-1.02132	0.663727	1.01868	1.901018	1.874113	-0.43862	0.859898	0.35315	0.559078	-0.27979	-0.18454	0.007106	0.333792	-0.39402	1.275267	-0.02096	-0.29831	-0.36018
P00505	GOT2	0.441134	-0.48311	-0.0231	0.941461	0.308896	0.016316	-1.09389	-0.29488	-0.49212	-0.24922	0.931015	-0.60097	0.291932	-0.68778	2.211222	1.032563	-0.77328	0.260249	0.052585
P00519	ABL1	0.160619	0.408447	0.355579	-0.55063	0.058483	-0.29412	0.000102	-0.27933	-0.06384	0.401726	-0.26185	0.323363	-0.00485	-0.34151	-0.742	-0.47312	-0.38276	0.039441	0.033941
P00533	EGFR	0.165519	0.137461	0.244601	-0.56962	-0.12548	0.079004	0.407053	-0.23602	0.59963	-0.00381	-0.54942	0.209914	0.52291	-0.09655	-0.2742	-0.39346	0.618733	-0.22814	0.193844
P00558	PGK1	0.102027	-0.86278	-0.67201	0.303063	1.169002	-0.11378	-0.70244	0.1831	1.038506	1.452715	-0.13264	-0.00492	1.064526	0.293167	-0.74075	0.343879	0.990751	-0.48111	0.522608
P00568	AK1	-1.13367	-0.24417	-1.50669	-1.02314	-0.69277	-0.45774	-0.14855	-0.5802	-0.1718	-1.60033	0.613297	-0.97437	-0.53375	-0.86822	0.643824	-0.52109	-0.18083	-0.39235	-0.58306
P00734	F2	-2.12175	-1.33192	-2.45426	-2.98937	-1.51408	-3.28933	-0.758	-1.19039	-0.21281	-1.59709	-1.22142	1.223183	-1.31993	1.052115	-0.69331	-2.37287	1.085977	-1.32094	-0.97356
P00736	C1R	-1.18713	0.889425	-0.09253	-1.59563	-0.19875	-0.63221	-0.06742	-0.87693	0.681105	0.795316	-0.81883	0.649845	0.244663	0.417384	-0.84929	-0.56113	0.182376	0.219866	-0.02664
P00738	HP	-0.21336	-0.92719	-0.66952	-2.87517	1.599296	-0.11175	0.977793	0.513948	2.539106	2.469324	0.190177	0.859401	0.781714	1.306509	-1.54686	-0.90855	0.19599	0.968877	-0.91431
P00739	HPR	-1.16357	0.458414	-0.68326	-0.84272	-0.2877	0.027446	-0.32638	-0.4707	0.65284	-0.04837	-0.51229	-0.23606	0.091434	0.575932	-0.60867	-0.85651	0.667527	0.733458	0.392813
P00740	F9	-1.58818	0.000654	-1.08443	-1.80987	-1.45145	-2.63369	-0.28399	-1.6366	-0.00328	0.00257	0.060997	0.558054	-0.75689	1.225791	-0.5369	-1.24287	1.374455	-0.07449	-1.93587
P00742	F10	-1.88496	-0.7148	-1.30508	-1.64342	-1.49868	-2.71595	-1.6246	-1.32936	-0.28829	-0.91045	-1.45241	0.862649	-0.99316	1.370509	-0.9329	-0.9671	2.317634	-0.72746	-1.79983
P00746	CFD	-0.98625	0.520996	0.436365	0.02904	-0.61649	-0.27403	0.087131	-0.12934	0.16682	-0.30123	-0.20603	-0.13084	0.320992	-0.05343	0.631224	-0.10896	-0.10731	0.109717	-0.17003
P00747	PLG	-1.50699	-0.26526	-2.23624	-2.01872	-0.73617	-2.08571	-1.37733	-1.3474	-0.6088	-1.45462	-0.66584	2.417944	0.475058	2.858623	-1.09316	-1.77178	2.570031	-0.21281	-1.33322
P00748	F12	-1.19784	1.891324	-0.19768	-1.47134	-1.02403	-1.75541	-0.20449	-0.51108	-0.28956	-0.90819	0.534662	-0.02427	-0.54076	0.438908	-0.32522	-1.32144	-0.64803	0.315092	-0.70501
P00751	CFB	-1.45552	0.607458	-0.69703	-1.73442	-0.60508	-0.59009	-0.62182	-0.85974	0.486182	0.497332	0.503328	-0.42307	-0.46291	0.332324	-0.50955	-0.32894	-0.17371	0.596707	-0.40549
P00761		-0.74695	1.743592	0.8121	0.082623	-1.38293	0.667025	2.087385	0.614714	1.131164	0.176533	-0.37156	-0.13037	1.016404	0.19528	0.660781	0.785748	-0.55712	0.010823	0.813284
P00813	ADA	-1.81173	0.951763	0.579812	-0.71201	0.876071	-0.27746	0.829228	-0.52537	0.739012	-0.02836	-0.86098	0.180221	0.019197	-0.25306	-1.07476	-0.88375	0.200638	0.536173	0.313826
P00915	CA1	-1.85816	-0.3359	-1.31766	-1.39217	2.268959	-2.27673	0.558107	-1.46251	2.254535	1.529292	-2.14633	-0.64226	-0.98436	-0.86515	-1.93198	-1.87599	-1.43313	0.966783	-2.19922
P00918	CA2	-1.51669	0.449516	-0.69868	-0.60443	0.863273	-0.57363	-0.17602	-1.18406	1.343613	1.213188	-1.33323	-0.95735	-0.7069	-1.12324	-1.71837	-1.76671	0.61247	2.621513	-1.35332
P00966	ASS1	0.922082	-1.76674	-0.59763	0.968171	0.05471	0.696681	-0.68881	1.673004	-0.50995	-1.8015	0.168537	-0.43088	-0.06644	-0.55158	-0.04599	0.933713	-0.88665	-1.82237	-0.00235
P00973	OAS1	-0.30996	1.26869	1.379763	-0.15039	1.634445	1.448413	0.31019	-0.49463	-0.2936	-1.25801	-0.42654	-0.30743	0.304801	1.337614	-0.36591	-0.31506	-0.22654	0.02743	-2.70433
P01008	SERPINC1	-1.72147	-1.17408	-1.99123	-1.74384	-1.08515	-1.65599	-0.60201	-0.78242	0.111175	-0.44291	-0.10042	0.129594	-0.66814	0.393723	-0.78082	-1.03137	0.215489	-0.26616	-0.55045
P01009	SERPINA1	-1.14659	0.937967	-1.8281	-2.2382	-0.2158	-1.2676	0.31592	-1.01865	1.296809	0.141541	1.007869	0.844065	0.064283	0.786699	-0.49564	-0.638	-0.14439	0.618684	-0.23828
P01011	SERPINA3	-1.55036	0.371476	-2.26431	-2.57078	0.335966	-1.14502	-0.08561	-0.30177	1.061799	0.909888	0.785532	2.294841	-0.236	2.060103	-0.97788	-1.09088	1.310106	0.756346	0.164694
P01019	AGT	-1.69214	0.635535	-1.76045	-1.98856	-0.4572	-0.97149	0.38449	-0.45199	0.055058	-0.4558	0.087301	-0.03983	-0.7511	0.28681	-1.03764	-1.29231	-0.33492	0.060373	0.079015
P01023	A2M	-0.89642	0.553133	-0.52825	-2.14893	-1.01898	-1.43721	0.107368	-0.69357	0.274149	-1.10027	-1.63332	-0.00823	-0.40876	0.048604	-1.02709	-0.94209	-0.32631	-0.34226	-0.96274
P01024	C3	-1.22975	1.058498	-0.36154	-1.21166	-0.64241	-0.85864	-0.07236	-0.74697	0.658014	0.225197	-0.09304	0.081199	-0.29505	0.288913	-0.30809	-0.90421	-0.00976	0.270361	-0.07512
P01031	C5	-1.56309	0.14346	-0.72446	-1.99872	-0.5948	-1.42624	-0.51058	-0.4929	0.316142	-0.54019	-0.38382	2.106174	0.135871	1.628968	-0.68637	-0.89956	1.728622	0.208516	-0.48151
P01033	TIMP1	-1.40994	0.862255	-0.88772	-0.50964	0.631379	-0.41377	-1.01436	-0.51534	0.580518	2.202574	0.171774	1.693031	0.536626	1.666498	-1.66221	-0.86246	1.085096	-0.8349	-1.25075
P01034	CST3	-1.01144	-0.36196	0.259	-0.72771	0.099922	-0.41494	1.040472	-0.08942	1.095759	0.83914	-0.56262	0.557794	-0.32351	0.536905	0.670638	0.161757	0.150739	-0.67697	-0.93834
P01040	CSTA	-1.8265	3.684894	-0.10518	-1.76873	-1.21534	-0.88719	2.039965	0.010622	1.785778	-0.98159	-1.18348	-0.23672	0.525229	0.108696	-0.67639	-0.7933	-0.46605	-1.15485	-0.39167
P01042	KNG1	-1.68442	0.104718	-1.09649	-2.24602	-0.88178	-1.74184	-0.39678	-0.71213	0.290743	-1.00977	-0.30006	0.082416	-0.9837	0.7199	-0.33106	-1.42166	0.092642	-0.01476	-0.64533
P01111	NRAS	0.83628	-0.23352	-0.38369	0.349965	0.178823	0.605756	-0.56057	-0.84248	-0.11412	0.436201	0.11008	0.57291	0.791969	0.318513	-0.19212	0.472642	0.249399	-0.06231	-0.31283
P01112	HRAS	0.222305	0.248742	0.105392	-0.07444	0.076561	0.238743	0.278246	-0.00978	0.078055	-0.30088	-0.22364	0.436119	0.000108	-0.10929	-0.06832	0.37162	0.267968	0.003437	0.139998
P01116	KRAS	-0.04023	-0.52228	3.665709	-0.43661	-0.34132	-0.4024	-0.10829	-0.13685	-0.28855	2.692695	-0.57711	0.252821	-0.11624	0.828328	0.091412	-0.54555	-0.53807	-0.88802	-0.61115
P01137	TGFB1	-1.1428	0.487816	0.246602	-0.89548	0.40413	-0.14696	0.236226	-1.23248	0.83076	0.344624	-0.41202	-0.36407	-0.2671	0.547986	-1.17403	-0.40563	0.230618	1.474456	-0.23036
P01591	JCHAIN	-2.09639	-0.1911	-1.39792	-2.03259	-0.10114	-1.18201	0.341901	-1.20414	0.068362	-1.65507	-2.45854	0.529803	0.510069	0.201338	-3.2125	-1.7342	-0.18018	-0.095	-2.22927
P01619	IGKV3-20	-3.03158	-0.58917	-0.75027	-2.05423	0.282757	-1.34227	1.45633	-0.0968	0.519417	-1.8946	-1.4186	0.99083	-0.13202	0.639744	-1.81103	-1.25943	-0.19165	-0.28454	-1.73896
P01730	CD4	-1.82354	1.301164	0.877538	-1.07391	0.594432	0.244836	0.169599	-1.778	-0.20348	1.053029	-1.34755	1.049545	-0.17527	0.954862	-0.5344	-1.06065	-0.47695	0.439032	-1.48911
P01742	IGHV1-69	-2.2484	-0.98843	-1.39676	-3.29519	1.982648	0.378931	1.623358	0.138139	0.557917	-1.85499	-0.0733	0.303856	-0.2071	1.252853	-1.79857	-1.37146	-1.54504	-0.93401	-2.40535
P01743	IGHV1-46	-0.24282	0.990234	-0.1381	-0.49286	0.775828	-1.00055	0.358327	-0.73097	0.040847	-2.71597	0.031165	0.776932	-0.29961	0.715238	-0.51809	-1.6299	0.208844	0.326232	-0.44967
P01780	IGHV3-7	-2.52283	-0.97358	0.546229	-2.08724	-0.13156	-1.28072	1.456571	0.118755	0.313363	-1.21018	-1.79356	1.534977	-0.28339	0.413592	-2.12025	-1.40226	-0.77573	-0.99225	-1.83935
P01833	PIGR	-0.81871	-0.79832	-0.25375	-1.9552	-3.13176	-0.81764	-1.01373	-2.45816	-0.25544	-3.05654	-0.44989	-2.16104	-1.10386	-0.0421	0.068476	-2.32464	-0.65519	-0.51531	-2.09312
P01834	IGKC	-2.00107	-0.32809	-1.63041	-2.19445	0.034825	-1.3907	0.844466	0.123267	0.633127	-1.68734	-1.22824	0.766744	-0.10565	0.976688	-1.21084	-0.88385	-0.08843	0.179886	-1.51908
P01857	IGHG1	-2.39702	-0.02065	-1.50342	-2.47834	0.046778	-1.06074	1.253248	0.500363	0.538493	-2.14375	-1.44274	0.827827	0.007084	0.682951	-1.1863	-0.85441	-0.04588	-0.03568	-1.77915
P01859	IGHG2	-1.50922	-0.76502	-0.81864	-1.512	-0.97739	-1.63703	2.250387	0.563091	0.707897	-1.37608	-0.67813	0.308225	-1.05314	0.952523	-1.12912	-1.33932	-0.05259	-0.20086	-1.28635
P01860	IGHG3	-2.92293	-0.22905	0.838293	-2.71581	0.115976	0.131565	0.637052	0.433286	0.156718	-2.07358	-3.17581	0.671005	0.550382	0.945271	-1.99691	-1.73235	-2.05831	-2.14741	-1.68599
P01861	IGHG4	-1.69221	-1.17858	-1.75985	-1.08341	-1.0931	-2.01551	0.074787	-0.80634	1.689893	-1.96437	-1.19141	0.473028	0.049221	1.035851	-0.63035	-1.55229	0.121653	0.110664	-0.93438
P01871	IGHM	-2.10505	-0.34378	-1.62309	-2.36043	-1.88607	-2.36393	0.106654	-1.55461	0.144211	-1.49343	-2.35807	1.214021	2.086283	-0.02485	-3.11367	-1.99893	-0.53448	-0.86133	-2.39622
P01876	IGHA1	-1.67339	-0.94428	-2.05567	-2.42753	0.269541	-0.77756	1.73995	1.5768	0.284819	-1.91736	-1.59116	0.443288	-0.14952	0.952509	-1.76978	-0.92515	-0.04636	0.535634	-1.47487
P01877	IGHA2	-1.20795	-0.3282	-0.58055	-1.00916	0.091107	-1.04365	1.576491	1.093752	0.523188	-1.61915	-0.98539	0.314105	-0.13878	0.880691	-0.93974	-1.68959	0.232297	0.633402	-1.35972
P01880	IGHD	-1.04296	0.533343	0.172521	-2.57485	0.073117	-1.00311	0.246619	-0.96817	0.047606	0.299012	-1.58865	-3.1079	-0.51149	1.180194	-1.43219	-0.22564	0.559666	1.334215	-1.20127
P01889	HLA-B	-2.24266	0.584756	2.248501	1.517646	-0.85271	-1.57346	0.679439	0.053656	-0.02443	-0.50795	-1.3588	1.432987	1.360491	-2.26197	-3.59347	-0.06483	-0.69646	0.1192	-2.68408
P01903	HLA-DRA	-1.23348	0.780327	-0.20347	-0.90613	0.675395	0.20804	-0.14221	-1.35621	-0.94968	-0.26406	0.615284	-0.29054	-1.48578	0.079189	0.260167	-0.52746	-0.44606	-0.32652	-1.73612
P01911	HLA-DRB1	-2.10346	-0.23923	-0.87829	-0.83551	-1.09126	-2.17799	-0.9523	-3.81098	-0.52235	-6.26063	-2.87014	0.373145	-1.60392	1.222402	-0.93618	-6.22318	-1.68778	-3.06055	0.503728
P01920	HLA-DQB1	-2.28894	1.834604	0.176998	-1.01895	2.811699	0.572809	-0.24397	-2.82593	-0.60203	-0.05215	-0.62561	-0.62823	-0.08969	-0.2196	-1.23699	-1.55727	-0.48473	0.110434	-1.91871
P02042	HBD	-1.05412	-0.19686	0.616235	-0.84119	1.113812	-1.60772	-0.18239	-1.14733	1.541642	-0.77787	-1.0221	-0.6172	-0.62492	-0.9014	-1.03839	-1.64901	-0.67725	2.722652	-0.82379
P02452	COL1A1	-3.37317	-0.88281	-0.28123	-3.45962	-1.55315	-2.63279	0.719414	-3.37917	0.27933	-1.62226	-0.5105	-2.42364	-1.93019	-1.51634	0.188549	-1.82299	-1.85026	-1.02475	-1.63796
P02458	COL2A1	-5.02289	-2.74632	-6.41322	-4.9749	-5.89151	-6.50578	-3.02511	0.814119	-4.12048	-5.58783	-0.50555	-3.37653	-4.4842	-2.59585	-3.55426	-4.68696	-2.23246	-6.38517	-2.77286
P02461	COL3A1	-3.2778	-0.45939	0.278579	-2.82531	-1.03609	-1.82504	0.760334	-2.55987	0.391782	-1.29873	-0.95582	-2.16434	-1.88861	-1.43993	0.233614	-1.99996	-1.41768	-0.97759	-1.32048
P02462	COL4A1	-0.61735	0.878468	-1.13837	-2.52774	-2.61018	-1.3274	-0.58159	-2.42224	0.021156	-0.2757	-0.36081	-2.99891	-1.02753	-1.61319	-0.28062	-0.89831	-2.53713	-0.02862	-1.76819

P02511	CRYAB	-1.10194	-1.66308	-0.24837	0.098794	-1.25624	-0.96744	-0.79574	1.494681	0.304811	-0.02467	-2.89418	-1.02857	-0.05753	-0.59338	1.647667	-1.80381	0.757472	-0.37665	-1.45282
P02533	KRT14	-3.19461	1.979274	-0.39565	-1.90163	-1.76271	-1.16141	1.784241	-0.83625	0.409116	-2.56663	-0.63225	1.177083	2.185315	-0.72276	-1.49938	-0.61216	-1.3366	-1.94049	-1.45065
P02538	KRT6A	-3.06382	0.335846	-0.61384	-2.27382	-1.57641	-0.57756	0.886676	-0.91761	0.679636	-0.73877	-2.58099	1.796402	3.538103	-0.66952	-2.82109	-2.26498	-2.02942	-3.24149	-2.00298
P02545	LMNA	-1.10464	-0.18607	-0.35899	0.130427	-0.23397	-0.32675	-0.04493	-0.65172	-0.259	-1.1973	0.081707	-1.05204	-0.32974	-1.33497	0.315553	-0.07197	-0.59528	-0.14982	-0.21939
P02549	SPTA1	-1.51453	0.943541	0.166169	-0.55194	-0.41528	-1.84138	1.391313	-1.70404	0.849527	-0.85607	-1.51145	-1.3548	-0.89857	-1.3178	-1.83402	-1.85509	-1.171	1.911394	-0.7962
P02647	APOA1	-0.97035	-0.49039	-0.61566	-1.68564	-0.87632	-1.21517	-0.63855	-0.47011	0.132108	-0.48137	-0.54251	0.576734	-0.45618	0.777228	-0.07105	-0.81055	0.211262	0.510552	-0.65733
P02649	APOE	-1.95309	1.260047	-0.54574	-2.25839	-0.82882	-1.07815	0.238198	-0.51101	-0.78526	-0.9407	-1.80611	0.539934	-0.8978	0.88486	-0.8892	-2.01449	1.290902	-0.72113	-1.38541
P02652	APOA2	-1.57326	-0.34811	-0.80443	-1.97811	-2.12368	-2.81745	-1.03115	-1.47564	0.904639	-2.11168	-1.3087	0.910619	-1.54797	0.346053	0.134113	-1.611	0.280682	-0.00596	-1.67226
P02654	APOC1	-1.2146	0.486305	-1.77996	-2.10027	-1.47433	-2.33227	-1.90543	-1.58313	-1.58222	-1.9405	-1.46462	1.300708	-2.36791	1.042934	-0.84491	-2.26641	1.98297	-1.28966	-0.74252
P02655	APOC2	-1.56054	0.521067	-1.44773	-1.95877	-0.83687	-1.43934	-0.24409	-0.4719	-0.23242	-1.19955	-0.7947	0.891518	-1.36134	1.241912	-1.47607	-1.84674	0.661541	-0.08578	-1.51123
P02656	APOC3	-1.65939	-0.30707	-0.91242	-1.26515	-0.33285	-0.91455	-0.73378	-0.92602	-0.01059	-1.46967	-0.82526	0.229628	-1.10277	0.278258	-0.63343	-1.55311	0.627833	-0.27038	-1.26657
P02671	FGA	-1.78644	0.249137	-2.01656	-1.9613	-0.65537	-1.37786	-0.83398	-0.96598	-0.44511	-1.18959	-1.71717	2.177708	2.165988	2.867536	-1.65205	-1.20621	3.515906	-0.02288	-0.98482
P02675	FGB	-1.8313	0.434016	-1.97461	-1.99681	-0.36826	-1.2691	-0.94086	-0.61782	-0.64387	-1.6454	-1.70701	2.475809	2.981624	2.867972	-1.4358	-1.21657	3.16076	-0.78967	-1.20156
P02679	FGG	-1.98349	0.532757	-2.23895	-2.09352	-0.43403	-1.34409	-0.88026	-0.64288	-0.76531	-1.59555	-1.87163	2.743592	3.064977	3.100105	-1.88848	-1.20867	3.178736	-0.95339	-1.3709
P02730	SLC4A1	-1.51819	0.918454	-0.04333	-0.72804	-0.39742	-1.77399	1.519196	-1.4747	0.822921	-0.89883	-1.70907	-1.03196	-0.37854	-1.0819	-1.6433	-1.84117	-0.91554	1.612601	-0.57942
P02741	CRP	-1.69056	-0.41744	-0.74917	-3.12494	0.45757	-0.25349	0.217155	-0.07173	1.416487	1.529381	1.938289	0.195685	-1.52019	1.266166	-1.36141	-1.03734	-0.26679	-0.74111	0.704078
P02743	APCS	-3.64681	-2.45873	-2.60928	-4.29893	-2.07815	-4.21499	-0.83321	-3.93518	-0.75439	-2.07722	-2.3313	-0.0994	-3.44797	0.08605	0.373418	-1.73695	-0.04693	-1.28753	-3.13323
P02746	C1QB	-1.64994	1.602379	0.969427	-1.75256	0.167641	-0.07816	0.363363	-1.0143	0.282742	0.058869	-1.04057	1.190768	-0.33661	1.649564	-1.23173	-0.63285	0.081195	0.172144	-1.69817
P02747	C1QC	-1.25991	1.078747	1.615862	-1.20798	0.001891	0.133431	0.301467	-0.90278	0.815704	-0.37496	-0.82994	1.064689	0.119404	1.50434	-1.05275	-0.33553	0.357485	0.079569	-1.36188
P02748	C9	-1.61501	-0.70084	-1.1869	-2.4611	-0.57082	-1.40418	-0.77561	-0.44866	0.681516	0.383231	0.287864	1.582597	-0.04494	1.578521	-0.73692	-1.12397	1.608859	-0.14027	0.097734
P02750	LRG1	-1.44837	-0.36548	-1.51563	-1.73434	-0.02925	-1.31409	1.167251	-0.48451	1.801954	1.979499	1.030284	1.617998	-0.01545	0.836089	-0.67935	-0.53773	0.267501	-0.13438	-0.98201
P02751	FN1	-1.71459	0.398361	-0.22398	-1.29955	-1.11812	-0.83975	-0.06208	0.138677	1.601234	-0.06872	-1.93288	0.501141	1.192343	0.0768	-1.59313	0.213443	0.027638	-0.49085	1.432245
P02760	AMBP	-1.40588	-0.44835	-0.72706	-1.32061	-0.72382	-2.87025	0.087831	-1.73169	0.190856	-0.68864	-0.40903	0.019374	-1.08132	0.086205	-0.31848	-0.8836	-0.14307	-0.46407	-0.67969
P02763	ORM1	-1.01748	-0.44421	-0.92081	-0.92091	0.213616	-1.5111	0.511784	0.464013	2.118887	0.416391	1.737215	0.948642	0.079553	1.034027	-1.08988	-0.91119	0.058082	-0.35018	-0.71313
P02765	AHSG	-2.57197	-1.28229	-2.59452	-3.11313	-2.21872	-4.03656	-1.44092	-0.71498	0.016051	-3.44972	-1.68529	-0.99273	-2.86912	-0.36464	-0.22497	-3.28777	-1.30955	-1.75149	-1.66799
P02766	TTR	-1.6979	-0.7624	-0.58244	-1.50031	-0.7022	-1.99807	-0.7349	-0.46894	-0.1855	-1.67927	-1.32696	0.988848	-1.11867	0.865804	-0.96591	-0.94507	0.458386	-0.66122	-1.60004
P02768	ALB	-1.60959	-1.42491	-1.92165	-2.24087	-0.05871	-1.65185	1.138801	-0.07801	1.085887	-0.92017	-0.14731	0.593853	-0.43573	0.419009	-0.48363	-0.95291	-0.0977	-0.02569	-0.94077
P02774	GC	-1.00851	-1.78631	-1.36462	-1.92936	0.040141	-1.70846	0.951745	-0.27998	1.804815	0.175197	-0.16678	1.653053	-0.40346	2.30181	-0.54734	-1.26506	0.748395	0.277255	-0.78192
P02786	TFRC	0.528062	0.367999	-0.02567	1.781142	1.025466	1.727859	0.677122	0.950084	0.331818	1.705033	-1.45184	0.142888	-0.33014	0.200131	-0.1657	0.759089	1.332673	-0.31876	0.199197
P02787	TF	-1.25428	-1.10681	-1.02676	-1.34482	-0.0314	-1.25474	0.37406	-0.07003	1.26765	-0.2357	-0.24853	0.435447	-0.40686	0.461771	0.033529	-0.84528	0.248881	0.217376	-0.96869
P02788	LTF	-2.37141	-0.58527	1.891354	-2.05204	-1.52776	-2.96289	-0.93697	-3.66386	0.03304	2.030033	-3.18043	1.177694	-2.18181	3.407261	-1.09948	-2.49586	2.2645	-3.64104	-1.76794
P02790	HPX	-1.52636	-0.09235	-1.27028	-1.54877	0.541837	-1.3678	0.779735	0.936771	1.308165	-0.28373	0.572763	0.804806	0.257232	0.807272	-0.17737	-0.67322	0.631839	0.709445	-0.51798
P02792	FTL	-1.0834	-0.22683	0.92691	-1.55813	0.356107	-1.21168	-1.17552	-0.71909	-0.38469	-1.92827	-2.18498	1.701645	2.68884	0.888577	-1.16894	-0.87578	-1.04563	-0.45912	-1.1709
P02794	FTH1	-1.15253	-0.41121	1.239292	-0.93217	0.306977	-0.87898	-0.93202	-0.26613	-0.23133	-1.39969	-1.45485	1.317159	2.767713	1.296234	-0.28227	-0.24255	-0.05461	0.093503	0.420137
P03372	ESR1	0.770666	-0.59134	0.098484	-0.02339	1.176729	1.041455	-0.06711	0.634272	-0.00575	-0.95084	1.459627	-1.69115	-0.30251	-0.33813	0.230288	1.932412	-0.17668	-2.0715	-1.2044
P03891	MT-ND2	0.573365	-0.00687	-0.09506	0.056987	0.849072	0.602497	-0.26141	-0.19701	0.036165	0.111801	0.960764	0.150504	-0.44586	0.035931	0.713927	0.243997	-0.25249	-0.30846	0.657127
P03951	F11	-1.18954	0.407439	-0.03689	-0.78291	-0.96212	-0.3363	-0.07705	-0.5899	0.207685	-0.53627	0.108773	-0.33108	-0.19775	0.417036	-0.31931	-0.37154	0.015371	0.878912	-0.63823
P03952	KLKB1	-1.42931	0.519729	-0.68583	-1.6086	-1.00677	-1.54742	-0.22544	-1.01692	0.059081	-0.06032	-0.10417	0.308622	-0.73574	0.324763	-0.92723	-0.7792	0.40736	0.348112	-1.103
P04003	C4BPA	-0.62679	1.411558	-1.48132	-1.76742	-0.32823	-1.2654	-0.56938	-1.06951	-0.04942	-0.14247	-0.88887	2.023608	-0.05083	2.818873	-1.34	-1.30314	2.522715	0.37571	-0.85379
P04004	VTN	-1.85071	-0.53164	-1.68016	-2.19397	-1.36806	-3.11121	-0.72628	-1.34003	-0.3474	-1.87326	-1.62699	1.463929	-0.96607	1.782883	-0.66117	-2.5935	2.402208	-1.29609	-1.05484
P04040	CAT	-0.45911	-0.01562	0.657104	0.657103	0.992639	-0.07622	-0.29557	-0.27976	1.63138	0.998266	-0.35223	0.114436	-0.5008	1.219027	-0.34231	0.495953	-0.12175	1.140336	-0.59043
P04049	RAF1	0.46403	0.399321	0.295219	-0.60793	0.266355	0.343417	0.04799	-0.34457	0.192031	-0.15037	-0.37038	0.24911	0.039142	0.723339	0.201327	0.271806	0.390945	-0.17805	-0.35123
P04062	GBA	-0.40125	0.137787	-0.17269	-0.43285	0.439872	-0.22186	-0.2013	0.237291	0.604566	1.463626	-0.42177	0.111258	0.956272	0.049791	-0.26629	-0.36291	0.123483	0.271693	-0.93073
P04066	FUCA1	1.088776	-0.48422	-0.11659	-0.38963	0.790134	-0.35129	-0.56435	0.998946	0.658567	1.253358	-0.16012	0.47894	0.619438	0.389604	-0.14879	-0.44967	-0.53449	-0.14121	-0.08912
P04070	PROC	-1.83371	0.071701	-0.68718	-2.49638	-2.15236	-3.19675	0.249052	-1.27486	0.519174	-1.27505	-0.43838	-0.44343	-0.68573	0.165553	-0.36136	-0.8749	0.455555	0.106659	-1.22421
P04075	ALDOA	-0.07758	-0.54693	-0.21859	1.003515	0.265237	0.361709	-0.91113	-0.09889	-0.1446	-0.24761	0.112362	-0.27312	0.367182	-0.14997	-0.38658	0.271779	0.05294	-0.57397	-0.18664
P04080	CSTB	-0.16934	-1.6032	-1.33355	-1.27818	0.198563	-0.47194	-0.31838	-0.95562	1.483431	1.852717	-1.11636	-0.25405	-0.52427	-0.11042	0.881842	-0.757	0.383596	-0.56942	-0.95968
P04083	ANXA1	0.011863	-0.12498	0.54781	-0.73366	0.067333	0.326517	-0.54707	-0.80609	-0.13494	0.748812	0.340153	0.632234	0.024308	0.55389	-1.51283	-0.36799	-0.58248	-1.13144	-0.60369
P04114	APOB	0.335552	1.1934	-0.0679	-1.74016	-1.06293	-1.43576	0.032807	-1.42604	0.582996	0.659951	-1.15316	-0.4368	-0.47361	0.267209	-0.90217	-0.68273	-0.10953	1.095447	-0.82247
P04150	NR3C1	-0.59804	0.21087	0.196888	-0.29257	-0.14823	0.002193	0.526587	-1.28463	0.331797	0.867016	-0.73436	0.160371	0.161081	0.19036	1.826146	0.065273	0.006133	0.630655	-0.22779
P04156	PRNP	0.490549	-0.43296	0.44448	-1.3165	-1.7711	-0.7204	-0.0469	-0.20582	-0.10591	-0.22004	-0.76393	-1.54996	-0.15092	-1.2237	-0.2489	-1.1965	-1.20435	-0.84173	-0.04771
P04179	SOD2	-1.48347	-0.54987	2.624147	-0.20376	1.181983	0.147632	-0.21128	-1.56136	-0.10953	1.965893	-0.45972	0.329581	0.775455	0.558563	0.605243	0.550395	0.499859	1.22169	0.327564
P04181	OAT	-0.8825	-1.11642	-0.28564	-0.05678	0.262186	-0.45818	-0.33905	0.551929	0.673262	1.729704	-0.19911	1.484574	1.724668	0.861355	1.166025	1.636187	1.821374	1.627163	0.882853
P04196	HRG	-1.33897	1.608572	0.095141	-2.1893	-1.08385	-1.44026	-1.11386	-0.22431	-0.03202	-0.96449	0.339308	1.137315	-0.05225	1.499801	0.105915	-1.56684	1.657132	0.702599	-0.66054
P04217	A1BG	-1.20836	-0.52143	-0.51802	-1.52521	0.258884	-0.94526	0.310658	-0.53254	1.17355	0.311323	0.169249	1.057134	0.810647	0.963151	-0.2327	-0.46712	0.467102	0.883924	-0.36651
P04233	CD74	-1.5707	0.769119	1.003498	-0.15076	1.784074	1.921287	-0.17686	-1.1853	-0.39904	-0.18193	1.367097	-0.73743	-0.03075	-0.41053	1.007532	-0.25494	-0.67967	-0.41557	-1.44673
P04259	KRT6B	-3.19586	1.376296	0.063354	-1.9835	-4.25497	-1.71234	1.696011	-2.27951	-0.03559	-1.09522	-2.04986	-0.13194	1.087828	-0.39121	-0.83555	-1.06187	-2.19974	-2.51477	-2.07052
P04264	KRT1	-2.29848	4.067861	0.503357	-1.36642	-2.28445	-1.05082	2.928018	-0.33861	1.300496	-1.81459	-1.10558	-1.28734	0.111257	-0.36676	-0.93562	-0.49714	-0.6161	-1.41299	-0.20136
P04275	VWF	-0.8902	0.587215	0.257209	-1.59971	0.013503	-0.8186	0.799997	-0.97679	0.141105	0.756777	-0.60936	-1.43786	-0.53313	-0.13877	-0.3084	-0.00096	-0.90482	0.81083	-1.33251
P04278	SHBG	-0.3113	0.001376	-0.44261	-0.51734	-0.38293	-0.45767	0.319498	-0.54714	0.147082	-1.27862	-0.52104	1.479202	-0.46419	0.665209	-0.00779	-0.52618	0.615862	0.316656	-0.49861
P04350	TUBB4A	0.133784	0.142334	-0.1073	0.303476	-0.39553	0.363376	0.036486	2.412311	0.736504	2.850683	0.624973	0.190524	0.257196	0.182755	1.632424	-0.50708	-0.2836	-0.24356	0.071947
P04406	GAPDH	0.072196	-1.31534	-0.30948	1.220042	-0.38752	-0.01894	-1.07851	0.731391	0.372277	0.075387	-0.1786	-0.1577	1.121057	0.290041	0.13377	1.125301	0.000414	-0.51331	0.580953
P04424	ASL	-0.18143	0.137963	0.118585	1.081049	0.665764	1.01724	-0.34337	-0.53775	0.173841	0.145007	0.106612	-0.81331	-0.58656	-0.17116	1.998944	-0.18617	0.138077	0.912742	-0.34856
P04439	HLA-A	-0.68695	0.921569	1.814928	-2.42036	0.848181	0.209115	-0.09149	0.193851	-0.45366	1.008002	-1.43843	1.538621	0.928477	1.410219	-0.14553	-0.31593	0.230736	-2.29973	-0.74981
P04440	HLA-DPB1	-0.84877	1.368821	-0.37191	0.010258	0.102366	-0.12065	0.101833	-2.53756	0.259176	0.261503	-1.02193	-0.21382	-0.82254	-0.292	-0.36481	-1.36963	0.122344	0.207027	-0.98337
P04626	ERBB2	-0.53292	-1.03271	-0.80837	0.303839	-1.00386	-0.84815	-0.36488	-0.83465	-0.09075	-0.60095	-0.43482	-0.86388	-0.62215	0.188256	-0.23558	-0.37849	-0.47504	-0.39501	-0.67245
P04632	CAPNS1	-0.40743	-0.50495	-0.58104	-0.06022	-0.24433	-0.05507	-0.43979	0.038355	-0.22465	-0.53693	0.308096	-0.4886	0.088832	-0.46969	0.402706	-0.24931	-0.58995	-0.68271	-0.01775
P04637	TP53	-0.91023	-0.53749	0.103467	-0.50724	-0.41964	-1.7045	-0.00363	1.132269	0.094175	-0.16297	-1.96412	0.810419	-1.90091	-0.34418	-0.97545	-0.84459	-0.09593	-1.5268	-0.70406
P04792	HSPB1	-0.15685	-1.14049	-0.53761	0.132582	0.161357	0.975148	-1.36904	-0.2207	0.077356	-0.41353	-0.58618	-0.4552	-0.08156	-0.05147	-0.09298	0.530508	0.878995	-0.1772	-0.28127
P04818	TYMS	1.625668	-0.97397	0.498399	0.908084	3.024774	2.972325	-0.09042	0.271765	-0.07373	-0.14261	-1.56239	-1.14388	0.059029	-0.88145	-1.54815	0.371679	0.742585	-0.70378	-1.32105
P04839	CYBB	-2.62457	1.864075	1.089972	-1.58163	1.157321	0.492747	0.499374	-2.16844	-0.06136	1.247659	-1.95076	2.611934	-0.38439	2.458246	-0.79018	-0.62778	1.022928	-0.11508	-1.98233
P04843	RPN1	0.04682	0.450448	0.402961	0.448345	0.087766	0.288907	1.17857	-0.05622	-0.04047	0.180907	0.075763	0.352481	0.340763	-0.12707	0.624886	-0.15451	0.153435	0.150688	0.350946
P04844	RPN2	0.027677	0.549132	0.532804	-0.17807	-0.05658	-0.24475	1.187146	-0.36495	0.185327	0.023159	0.081351	0.258357	0.276682	0.032457	0.512089	-0.03872	0.142579	0.290834	0.363764
P04899	GNAI2	-0.96292	0.454411	0.413686	-0.84278	0.319755	0.029646	0.355862	-0.78965	-0.02791	0.415425	-0.49813	0.761561	-0.15871	1.044792	-0.29004	-0.457	-0.35361	-0.22581	-0.36752
P04920	SLC4A2	0.598009	-0.30586	0.540037	0.659743	0.619367	1.240191	0.178374	0.519126	0.547898	0.763123	0.162747	0.040425	0.282455	-0.48155	0.045284	0.119814	-0.89949	-1.24246	-1.05175
P04921	GYPC	-0.79417	0.879611	0.153083	-1.02072	-0.18022	-0.56737	1.428901	-1.76868	0.973882	-0.08792	-0.54726	-0.59299	0.692236	-0.61531	-0.19453	-0.62863	-0.75377	0.556039	-0.53489
P05023	ATP1A1	0.08259	-0.09125	-0.4352	0.136491	-0.4541	-0.33293	-0.01061	0.187277	-0.30782	-0.05763	-0.2291	-0.41602	-0.45394	-0.07289	0.474912	0.2392	0.366211	0.164936	-0.72504
P05026	ATP1B1	0.536328	-0.87593	-0.99173	-0.50942	-1.16346	-0.80675	-0.47121	0.74045	-0.76757	-0.2504	-0.49937	-0.93392	-2.71396	-0.25133	-1.21513	-1.23505	0.360635	-0.84638	-0.9039
P05067	APP	-1.04142	-0.13678	-1.44117	-1.39154	-2.84718	-1.76115	-0.19725	-0.52533	0.693902	-1.47662	-1.12994	-1.55644	-1.47333	-0.69764	-0.39185	-1.65684	-0.633	-1.1456	-0.11818
P05089	ARG1	-2.83018	2.737343	0.166114	-1.26205	-1.46393	-1.87669	1.617822	-0.63462	1.260966	-0.39694	-1.114	-0.80878	-0.27023	1.027372	-1.39662	-1.34829	0.201272	-0.65876	-0.94935
P05090	APOD	-0.02133	0.10784	-0.16574	-1.14684	-1.1711	-0.88646	0.22943	0.181906	0.614708	-0.86985	-0.70074	1.146339	-0.73597	0.710938	-1.10674	-0.62681	0.308893	-0.18662	-0.7418
P05091	ALDH2	-1.34683	-0.51549	-0.0842	0.140646	0.592245	-0.22434	-0.77296	-0.64559	0.293558	0.64188	-1.06893	0.833324	-0.22274	0.842008	1.478597	0.569974	0.434249	1.861646	0.465869
P05106	ITGB3	-1.53221	0.468533	0.200273	-0.21354	0.088407	-1.33911	0.169743	-1.66148	-0.43797	-0.71133	0.944968	-0.36812	-0.26579	-0.45962	-0.99058	-1.69972	0.57096	2.389728	0.213788
P05107	ITGB2	-2.21819	1.69485	0.941132	-0.76329	0.171905	-0.04083	0.214534	-1.54333	-0.88179	1.510338	-1.22177	1.231271	-0.04084	2.141956	-0.56468	-0.84687	0.598584	-0.13249	-1.38457
P05109	S100A8	-3.50204	-0.28176	2.464368	-2.77917	-1.95428	-1.56108	-0.21328	-3.42153	0.978629	3.622736	-3.70827	0.506466	-1.20119	4.016551	-1.45261	-2.08822	1.673885	-2.91044	-1.78265
P05114	HMGN1	0.528061	-0.0678	0.080672	-0.73566	-2.02761	-0.19258	-0.25654	-1.19689	0.220083	-0.37604	0.148418	-0.64752	0.296431	-0.38897	0.432087	0.34494	-0.19549	1.975945	0.800852
P05120	SERPINB2	-2.23741	1.484601	1.029177	-1.68007	0.92372	-0.89993	0.44682	0.905283	1.152183	-0.05788	-1.02852	2.509771	1.663305	1.453195	-1.2814	-1.47021	2.108049	0.012102	0.197206
P05121	SERPINE1	-0.21351	0.280647	0.897191	-0.67336	-0.26768	0.120001	0.028779	-0.23615	0.38108	-0.7915	-2.59094	2.239745	1.320087	1.54115	-0.56534	-0.72201	2.763763	-2.85557	-0.11319
P05141	SLC25A5	0.175854	0.026746	0.050252	0.545853	0.921733	0.98322	-0.17459	0.027278	-0.28796	0.398377	-0.05675	0.400328	-1.10623	0.028604	0.030423	1.585534	-0.03285	0.028302	-0.71747
P05154	SERPINA5	-1.39021	-0.84569	-1.38015	0.149054	-1.42019	-1.31226	0.390065	0.508849	0.245208	-1.0097	-0.99307	-0.72305	-1.22333	-0.53385	0.191058	-1.11299	-0.48322	-0.54942	1.611877
P05155	SERPING1	-1.30588	0.900284	-0.45715	-1.44003	-0.23278	-0.36697	0.377317	-0.16815	0.646594	-0.17917	-0.0828	0.421635	0.092304	0.768791	-0.72086	-0.6367	-0.00906	0.675402	0.022239
P05156	CFI	-0.85456	0.425294	-0.79588	-1.82094	0.157776	-0.5282	-0.38959	-0.0153	-0.03954	-0.19268	0.016757	0.12779	-0.0382	0.575252	-1.18068	-0.8417	0.224769	0.267113	0.656687
P05161	ISG15	1.023742	1.510831	3.358614	-1.24667	-0.40305	0.059581	0.266166	2.029208	0.463928	-0.26492	-1.48647	0.356804	0.422383	0.39374	-1.12378	-1.53209	0.702498	0.548919	-1.65427
P05164	MPO	-1.71591	0.134879	0.738239	-1.77921	-0.52092	-1.46996	-0.04281	-3.36774	-0.05897	2.189009	-2.13596	0.783791	-1.08718	3.135236	-0.84384	-1.92906	1.98111	-2.26659	-1.60828
P05165	PCCA	0.284546	-0.04075	-0.45731	-0.57439	-0.7028	-0.78662	0.081083	-0.24657	-0.07577	-0.11324	1.100964	-0.54897	-0.63226	-0.4218	0.897377	-0.34292	-0.65644	0.24289	0.139267
P05166	PCCB	0.206912	-0.27515	-0.66539	-0.39388	-0.68514	-0.74697	-0.29698	-0.27588	-0.01151	0.06924	0.768211	-0.33664	-0.63841	-0.23367	1.130353	-0.13461	-0.37931	0.063825	0.110665
P05186	ALPL	-0.09055	0.001427	-0.31852	-2.35156	-2.35249	-2.324	1.257124	0.710378	-0.50029	-0.81564	-0.62094	-1.21435	-0.71875	-0.60777	-1.1545	-1.71648	-1.50742	-0.91528	-0.05795
P05198	EIF2S1	0.408042	0.309	0.340026	0.209944	0.031615	-0.05197	-0.38173	0.21698	-0.24798	0.205495	0.094882	-0.19949	0.43979	-0.25353	0.183182	0.082177	0.200826	0.050967	0.134897
P05204	HMGN2	-0.06958	-0.08542	-0.41818	-0.72712	-2.31232	-0.48503	-0.61289	-0.96255	-0.91533	-0.0918	-0.57321	-1.36673	-0.01996	0.629288	0.501393	-0.31965	-0.07227	0.449655	0.430083
P05362	ICAM1	-1.03219	0.009483	0.394236	-1.50835	-0.35344	-0.92593	0.540779	0.43064	0.320798	1.859856	-1.60627	1.31657	1.039975	0.555262	-0.78182	0.911874	0.952865	-0.14688	-0.39534
P05387	RPLP2	0.28673	-0.12525	-0.70009	-0.26195	0.932668	0.181522	-0.75938	0.542235	0.110267	-0.49244	0.199871	-0.26816	0.06272	-0.56626	-0.52551	0.054029	0.026764	0.188024	-0.51916
P05388	RPLP0	0.21671	-0.15793	0.007262	0.361209	0.049384	0.254391	-0.1071	0.181768	-0.27854	-0.11466	0.495823	0.026803	-0.0666	-0.22864	0.034904	-0.00116	-0.16068	-0.3596	0.276035
P05413	FABP3	-2.20228	-0.07528	-0.61313	-2.08024	-0.3486	-1.38256	0.019521	2.89431	-0.28934	-0.82782	-1.57752	-1.06778	-0.77927	-1.17495	0.988023	-1.46955	-0.23272	-0.6025	-2.2447
P05423	POLR3D	0.598082	-0.24146	-0.0496	-0.5565	-0.60121	-0.29017	0.350288	-0.28303	-0.25338	-0.55702	0.065612	-0.23864	-0.15767	-0.45094	-0.05674	0.282683	0.289887	-0.13584	-0.19589
P05452	CLEC3B	-1.77209	-0.08882	-0.86378	-0.74866	-0.26627	-2.38748	0.30133	-0.2515	0.336038	-0.34787	-0.65744	-0.24099	-0.58774	-0.57175	-1.02715	-0.74106	-0.40488	-0.0226	-1.67962
P05455	SSB	1.250957	-0.79939	-0.23134	0.251164	-0.4119	-0.39229	-0.96344	0.551624	-0.22003	0.453118	0.624554	-0.21939	0.001241	-0.37193	0.325177	-0.26042	0.599805	0.302329	-0.06472
P05534	HLA-A	-0.05077	1.388541	1.325346	-0.69234	0.372942	0.528712	-0.79643	-3.78793	-0.35197	0.358197	-0.94534	-0.35781	-0.20899	1.206815	-1.27528	0.188969	-1.51205	0.432672	-3.41354
P05543	SERPINA7	-1.43678	1.189441	-1.19864	-2.08916	-0.82768	-1.22136	0.001842	0.065826	-0.1263	-0.50778	-0.3718	-0.20386	-0.60715	0.467532	-0.27845	-0.86613	-0.36605	0.159945	-0.5027
P05546	SERPIND1	-1.36614	1.341911	-0.6679	-1.71565	-1.37325	-1.50341	0.156415	-0.59718	-0.01719	-0.43593	-0.26184	-0.05545	-1.00953	0.266593	-0.52103	-1.27465	0.795055	0.553649	-0.63271
P05556	ITGB1	-0.13877	0.23096	-0.12601	-0.27066	-0.67816	-0.54079	0.179579	0.025628	0.425534	0.169358	-0.54682	-0.68019	-0.25574	-0.60982	-0.03514	-0.1806	-0.5059	0.031845	0.205719
P05771	PRKCB	-2.56445	1.054565	1.171852	-0.86767	-0.02119	-0.15707	0.812221	0.591222	-0.51564	1.065046	-2.52065	1.206819	-0.11688	1.611678	-0.54249	-1.51328	0.613179	0.300481	-1.08977
P05783	KRT18	0.10433	-0.53035	-1.30516	0.054105	-1.73875	-1.22364	-0.96508	0.673134	-0.96627	-0.74774	0.806036	-0.64239	-0.69521	-0.92338	-1.57952	-1.50048	-0.14686	-1.17458	0.734325
P05787	KRT8	-0.31326	-0.23075	-0.70565	0.708002	-1.69113	-1.04369	-1.10936	0.408661	-0.91821	-1.39967	1.110226	-0.44553	-0.34978	-0.9049	-2.03292	-0.42412	0.33306	-0.47204	0.811147
P05997	COL5A2	-2.33145	0.341906	-0.43194	-3.04047	-1.68584	-1.12158	0.055262	-2.54221	0.384948	-0.7306	-0.69131	-2.20781	-0.51236	-1.52305	-0.54611	-0.3999	-1.71598	-0.93809	-0.88632
P06132	UROD	0.655062	-0.0939	0.205921	0.310544	1.385154	0.766352	-0.136	1.123826	-0.08065	0.367478	-0.37695	-0.41378	0.212105	-0.04715	0.162795	0.306194	-0.59311	0.142343	-0.69136
P06213	INSR	-0.24025	-0.69693	0.005754	-0.59744	-0.24851	-0.5815	-0.14598	-0.53524	-0.26505	0.268428	1.017354	-0.59563	-1.04633	-0.46644	0.145882	1.051432	-0.18147	0.542347	-0.4689
P06239	LCK	-0.33233	0.767509	0.140231	-0.86306	0.381741	-0.00631	0.456344	-0.9293	-0.15962	0.466838	-0.93305	0.013802	0.109651	-0.70926	-1.49	-0.70723	-0.82986	-0.16804	-0.59407
P06241	FYN	-1.00636	0.247285	0.286264	-0.03835	0.131401	-0.02819	0.375271	0.596554	0.08386	0.404716	-0.05295	-0.56104	0.002855	-0.13239	-0.57708	-1.02974	-0.1209	0.755735	0.29807
P06280	GLA	0.253136	-0.37856	0.064303	0.320048	1.05063	0.533466	0.133832	1.155526	0.039599	0.23512	0.371115	1.200694	-0.06618	0.355001	-0.27978	-0.26204	-0.00827	-0.33016	-0.62393
P06310	IGKV2-30	-2.46958	-0.53279	-1.0725	-2.12849	0.008508	-1.32413	0.888548	0.046882	0.142092	-2.28773	-1.47205	1.082212	-0.58058	1.378182	-2.22248	-2.07104	-0.2175	-0.26217	-1.73567
P06396	GSN	-0.52626	-0.80253	0.06461	-0.17216	0.39992	0.354077	-0.41254	0.015752	0.458263	0.325152	-0.53697	-0.22762	0.43304	0.456141	0.045365	-0.07575	-0.3332	0.189546	0.755225
P06400	RB1	-2.20252	0.667759	0.029407	-0.43879	-1.2481	-1.28766	0.383332	1.602732	0.22556	0.500511	0.277829	-0.27915	-0.17477	0.279765	0.501864	-0.63647	0.146028	-0.07761	0.742301
P06401	PGR	1.437886	0.360115	0.078026	-0.68589	-0.27386	-0.14304	-0.45496	-0.02362	-0.58243	-0.15499	1.790123	0.193523	-1.11714	-0.29507	0.060035	-0.1378	0.580617	-0.67945	-0.92413

P06493	CDK1	0.819654	-0.22374	-0.03189	-0.16713	1.368871	1.876508	0.13715	1.269971	0.124782	-0.00023	-0.67965	-0.82984	-0.13607	-0.57387	-0.50958	-0.15158	1.047463	-0.18472	-0.11216
P06576	ATP5B	-0.16134	0.014443	-0.00823	0.523213	0.144083	0.116741	-0.26984	-0.25189	-0.08831	0.373655	0.334402	0.12084	-0.09809	0.064801	1.143775	0.968758	-0.39873	-0.42097	-0.35706
P06681	C2	-1.63421	0.939279	0.182145	-1.4995	0.103616	-0.03793	-0.20102	-0.61806	0.853445	0.335316	0.208793	-0.67493	-0.4309	0.470213	-0.13599	-0.65966	-0.3039	0.291058	-0.88983
P06702	S100A9	-1.74581	0.066669	2.086572	-1.48162	-0.84091	-1.12906	0.0384	-2.12613	0.978352	2.518245	-2.60608	0.338517	-0.80134	2.684001	-0.95883	-1.43551	0.897824	-2.47122	-1.49805
P06703	S100A6	-0.98316	-1.86327	0.521711	1.964014	0.27086	0.211942	-1.04146	-1.6304	0.066774	0.326493	0.375896	-1.21225	-0.87912	-0.46711	0.53111	1.738262	-0.67892	-1.26027	-0.9976
P06727	APOA4	-1.70394	-0.53671	-0.90652	-1.33789	-1.95258	-2.20956	0.018803	-0.30863	-0.57866	-1.95796	-0.92257	1.213142	-0.20961	0.734911	0.454375	-1.52766	0.644799	-0.30215	-0.72811
P06730	EIF4E	0.510476	-0.52548	0.033609	-0.40653	1.129442	-0.17256	-0.47912	0.501594	0.595117	1.346566	-0.06494	0.409572	0.357756	0.393242	-0.41776	-0.1883	0.880255	0.366119	-0.21118
P06732	CKM	-3.45806	-1.39646	-0.47789	-3.25985	-5.04663	-2.86986	0.303956	-5.27069	-0.88423	-4.94718	-4.44055	-2.69858	-2.67616	-2.78843	-5.18362	-4.90615	-2.1518	-4.41677	-4.69586
P06733	ENO1	0.732906	-1.31362	-0.07419	0.888482	0.631836	0.723414	-0.88079	1.124771	0.939143	1.6446	0.777903	-0.32739	0.472112	0.014781	0.176119	1.397451	0.491722	-0.53985	-0.41762
P06737	PYGL	0.098194	-0.39125	0.850989	-0.73767	-0.00768	-0.18639	-0.25106	0.02486	0.116504	0.402846	-0.86237	0.238104	0.721624	1.026281	-4.2E-05	-0.27067	0.024301	-0.17377	0.404033
P06744	GPI	0.5048	-1.34012	0.138872	1.087705	0.761446	1.21495	-0.70421	2.987012	0.06401	0.389562	0.191904	-0.6957	0.787671	0.249188	1.46701	0.659005	-0.1618	-0.75795	0.444839
P06746	POLB	0.29424	-0.15983	-0.36022	-0.43996	0.063832	0.205615	-0.78047	0.058682	-0.62794	-0.73348	0.382262	-0.0056	-0.17391	-0.62226	1.427908	-0.49519	0.424749	0.109768	-0.64642
P06748	NPM1	0.631193	-1.43952	-0.41923	1.250271	0.30629	0.133532	-1.45852	0.108548	-0.40265	-0.23587	0.868599	0.101712	1.405073	-0.78821	0.141183	0.285897	0.171771	0.547746	-0.1803
P06753	TPM3	-0.06879	-0.96268	-0.06433	-0.09637	0.101712	-0.17671	-0.52261	0.109547	0.055152	0.40754	-0.68264	0.003888	-0.18082	0.515677	-0.21749	-0.13085	-0.29093	0.354097	-0.42431
P06756	ITGAV	-0.12814	0.529119	0.0236	-0.34408	-0.21481	-0.38575	0.247631	-0.11487	0.929202	-0.33569	-0.13724	-0.17085	0.680472	-0.6548	-0.91424	0.096506	-0.40338	0.527325	0.883352
P06858	LPL	0.65736	-0.48228	-0.68075	-1.00562	-1.12064	-1.64849	-0.2623	-1.16392	0.553666	-0.99491	-1.7589	-0.35048	-0.2523	0.430082	-0.6367	-1.34945	-0.1632	-0.91477	-0.84291
P06865	HEXA	0.6938	-0.42707	0.455428	-0.01163	1.781596	-0.13584	0.822026	1.3663	0.87202	0.745342	-0.4091	1.238447	1.100921	0.937446	-0.30037	-0.56097	0.894502	1.705716	-0.15645
P07093	SERPINE2	-1.89638	-0.54314	-0.76843	-1.52055	-1.1806	-1.69694	-0.18864	-1.05886	1.012422	-3.474	-0.06611	-0.26894	-1.71191	-0.67212	1.123733	-1.25342	-1.03796	-3.95129	-2.64584
P07099	EPHX1	-1.11709	-0.07454	0.02169	-0.1832	-0.41835	-0.89903	0.382066	-0.58788	-0.25862	-0.11503	-0.69615	-0.22523	-0.26998	-0.44311	1.02951	-0.75842	-0.43335	-0.63083	-0.95164
P07108	DBI	-0.55227	-0.32229	-0.18378	0.296721	2.226268	-0.44258	0.019336	-0.30241	0.552268	2.313378	-0.25944	-0.42383	0.351852	0.204877	1.858627	1.233635	0.289127	0.854763	0.550429
P07195	LDHB	0.627422	-0.39257	1.521349	-1.1198	-0.7097	-0.25452	-0.5583	0.668925	0.080853	-0.13329	-0.0106	-1.14343	-0.2925	-0.75704	-1.43123	0.542367	0.003341	0.312565	0.174508
P07199	CENPB	1.071697	-0.35587	-0.22283	-0.61146	-0.01379	-0.01071	0.231616	0.539038	-0.06538	-0.01289	-0.0585	-0.11263	-0.24884	-0.12917	0.362206	-0.2768	-0.34927	-0.43276	-0.31295
P07203	GPX1	-0.44412	0.401006	0.258748	-0.12927	0.004758	-0.38249	-0.49231	0.042667	-0.19773	0.280406	-0.46598	0.377423	0.520739	0.105105	0.091748	0.004962	-0.93063	-0.40911	-0.8545
P07205	PGK2	-0.02177	-0.20492	-0.69852	0.109242	0.572837	-0.56812	-0.57229	0.010509	0.040847	-0.8575	-0.21541	-0.04269	0.840885	-0.05664	-0.02618	-0.56368	1.08997	-0.99091	0.605638
P07225	PROS1	-1.11667	0.413147	-1.49581	-2.15611	-0.84983	-1.4841	-0.44411	-0.78492	0.637844	-0.18098	-1.53935	1.804712	-0.69476	2.077371	-0.35668	-1.19666	1.629924	-0.33451	-0.59536
P07237	P4HB	-0.26287	0.998061	0.303879	0.219858	0.313723	0.121158	-0.02022	0.222974	0.288929	0.530243	-0.78376	0.699819	1.406971	0.560501	-0.66473	0.342655	0.7375	0.905613	0.660286
P07305	H1F0	-0.4597	-0.50079	-0.62495	-0.61152	-1.97809	-1.10574	-1.01754	-0.25446	0.385564	-2.83137	0.53213	-0.29837	-0.48673	-0.27355	0.247658	0.175454	-0.04428	-0.98156	-1.38585
P07332	FES	-0.11577	0.69313	0.643784	-0.33641	0.239867	-0.08999	0.14334	-0.54758	0.006992	0.575565	-0.54816	0.733201	0.085147	1.444475	-0.35489	-0.17315	0.497277	-0.25343	-0.61912
P07333	CSF1R	-1.04397	2.389325	0.971575	-0.78238	0.716843	0.116109	0.478334	-0.96972	-0.50029	1.914002	-0.04597	0.010551	0.734445	1.003089	-0.34433	0.184036	-0.08612	0.225518	-0.66271
P07339	CTSD	-0.81698	0.373484	-0.15189	-1.50865	1.529416	0.78018	0.469041	0.408008	0.387775	-0.55227	-0.94536	0.039646	-0.71442	-0.47098	-0.61526	-0.02497	-0.70408	-0.94549	-2.34055
P07355	ANXA2	-0.92384	0.226959	-0.20213	-0.59218	-0.18056	-0.65314	0.164394	-0.51919	-0.18325	-0.57504	-0.32362	0.756142	0.058811	-0.22238	-0.95573	-0.71684	0.230287	-0.25982	0.257933
P07357	C8A	-1.53392	0.51896	-1.76566	-1.60459	-0.66935	-1.74179	0.042304	-0.36729	0.0326	-0.43953	-0.38079	0.844812	-0.16381	1.46024	-1.08245	-0.46512	1.224604	-0.65912	-1.41205
P07358	C8B	-1.44677	0.454459	-0.71482	-1.52528	-0.86071	-1.47075	-0.13977	-0.38155	0.290722	-0.22248	0.134395	0.72942	-0.38017	1.043141	-0.6897	-0.55595	0.806201	-0.07767	-0.35564
P07359	GP1BA	-0.94187	0.603737	0.212753	-0.8764	-0.31109	-0.85741	0.642159	-0.29963	0.369282	0.525171	-0.44248	-0.43153	-0.74324	-0.43056	-0.51637	-0.18043	-0.55449	1.550858	0.863815
P07360	C8G	-1.62627	0.078041	-0.93696	-1.70371	-0.64591	-1.40688	0.277114	-0.28847	0.640688	-0.28981	-0.05155	1.393617	0.398207	1.504324	-0.46932	-0.48944	1.063681	-0.90745	-0.96634
P07384	CAPN1	0.022196	-0.56734	0.088553	0.307991	0.320821	0.433022	-0.64764	0.327919	-0.16521	-0.30695	0.379677	-0.32383	0.056221	-0.19051	0.480457	0.325216	-0.37517	-0.53897	-0.18932
P07437	TUBB	0.362036	0.334803	0.193715	-0.16466	0.190847	0.451231	0.009562	0.207346	-0.15801	-0.23221	-0.38448	0.151836	0.245425	0.085721	-0.007	0.021183	0.162351	-0.15774	0.529158
P07451	CA3	-0.98568	-0.91766	-0.16977	-2.12468	-0.91652	-1.19306	-0.26238	-2.01322	-0.17656	-1.65464	-2.125	-1.34126	-1.4021	-1.94554	-1.86194	-2.03232	-1.31386	-0.51538	-0.96466
P07585	DCN	-2.79752	-0.50719	-0.2441	-2.87655	-0.98291	-1.62295	0.409465	-2.0303	0.857546	-0.68901	-1.09104	-1.72839	-1.59236	-1.41458	-0.02781	-1.12476	-1.20032	-0.58316	-1.20988
P07602	PSAP	0.017595	0.814462	0.326276	-1.21758	1.008134	-0.14569	-1.1642	-0.28874	-1.80831	-0.66775	-0.94007	1.079357	-0.17982	0.715519	-0.07552	-1.14735	0.320513	0.628968	-0.92403
P07686	HEXB	0.174505	-0.19753	0.500071	-0.31211	1.063229	-0.19955	0.181427	1.292084	-0.04292	0.639356	-0.64629	0.912397	0.394554	0.821126	0.501612	-0.01997	0.20791	1.1447	-0.28487
P07737	PFN1	0.801288	-0.48006	0.020259	-0.40217	1.385607	0.025899	-0.80179	0.390438	0.483772	1.688305	-0.02398	0.644428	0.915195	1.450842	-0.47766	-0.1322	0.377853	0.188539	-0.40054
P07738	BPGM	-0.72004	0.002665	0.22263	-0.22993	1.172978	-0.80292	0.151987	-0.06498	1.937506	-0.34823	-0.68517	-0.48646	-1.25371	-0.17004	-0.53967	-0.79851	-0.30304	1.745103	-0.42932
P07741	APRT	-1.18034	-1.74737	-1.78876	-1.15362	1.855188	-0.92106	0.318547	-0.43675	0.969474	0.814941	-0.73457	1.888895	-0.29378	2.010079	-0.61846	0.199445	1.443351	-0.42591	-1.36701
P07814	EPRS	-0.07828	-0.27046	-0.30043	0.245685	-0.40573	-0.29413	-0.08779	0.311488	-0.11589	0.231664	0.099601	-0.27144	-0.30693	-0.20038	0.861129	0.714382	-0.07447	-0.21375	-0.26923
P07858	CTSB	-1.30297	0.386719	-0.56902	-1.74959	0.076273	-1.36481	1.276173	0.160134	0.630233	1.127895	-1.39986	1.840718	0.128513	0.825966	-1.45958	-0.43639	0.345734	-0.63218	-1.24473
P07900	HSP90AA	0.263651	-0.26927	-0.26864	0.540187	0.54921	1.156645	-0.40452	-0.00796	-0.52114	-0.27166	0.501711	-0.45065	0.271676	-0.43894	-0.28793	0.279426	-0.06854	-0.35715	0.137159
P07902	GALT	0.801497	-0.6059	0.150014	-0.63298	1.722667	0.127813	-0.3374	-0.72214	0.49124	0.638851	-0.76742	0.301292	0.214463	0.517436	-0.61312	0.507047	0.469094	0.711754	-0.38896
P07910	HNRNPC	0.497708	-0.35859	-0.15082	0.318198	-0.10163	0.509228	-0.55775	0.511578	-0.11937	0.299086	0.755503	-0.4792	-0.03498	-0.59882	-0.01078	-0.10094	-0.04591	0.482565	-0.22553
P07942	LAMB1	-1.20305	0.803658	0.221089	-1.30282	-0.19974	-0.71308	0.474065	-1.36712	-0.10276	0.133188	-0.56695	-2.00059	-0.07177	-1.35652	-0.79337	-0.20881	0.272312	2.285607	0.35152
P07947	YES1	0.043641	0.179589	-0.26785	0.69495	0.230453	0.180989	0.439094	0.404624	-0.15129	-0.08737	0.09162	0.222413	-0.72743	-0.96454	0.595929	0.177137	0.655519	-0.07032	0.00396
P07948	LYN	-1.11619	1.097746	1.20463	-0.86744	0.249339	-0.1377	-0.0744	-0.91157	-0.2961	0.481876	-0.81797	1.945924	0.348051	1.84048	-0.03074	-0.37342	0.616846	0.34503	-0.67566
P07951	TPM2	-2.07907	-1.78103	-0.76818	-0.54201	-1.75296	-1.28714	-1.56157	-0.82299	0.749313	-2.19231	-1.24228	-1.53109	-1.04919	-1.11417	-0.92884	-0.25376	-2.20154	0.059331	-0.64865
P07954	FH	0.561727	-0.23317	-0.01949	0.867132	0.804208	0.964929	-0.82634	0.225218	-0.07248	0.897389	-0.02112	-0.17	-0.05377	-0.2487	2.331186	-1.3208	-0.44046	-0.01559	-0.40625
P07996	THBS1	-1.48845	-1.9628	-1.19158	-1.84393	-1.6874	-2.23488	-1.39171	-1.29801	0.822936	-1.68006	-1.06579	-1.06579	-1.51398	-0.65463	-1.30335	0.880869	-1.3577	-1.82389	-1.35449
P08047	SP1	0.679186	0.134484	0.260816	-0.06536	-0.19058	0.283928	0.085871	-0.11073	-0.39049	-0.07565	-0.36862	-0.07939	-0.27844	-0.21478	0.148735	0.009561	0.063178	0.189047	-0.43149
P08069	IGF1R	0.173103	-1.23466	-0.60451	-0.88531	-1.42385	-1.17371	-0.03182	0.988599	0.302245	-0.17258	0.929747	-0.75322	-0.11682	-0.48193	-0.10679	-0.82631	0.217892	-0.28158	0.127082
P08123	COL1A2	-2.95781	-0.5336	-0.17372	-2.63184	-1.33281	-1.86877	0.750944	-2.69928	0.465429	-1.06822	-0.5671	-2.31476	-1.65202	-1.53498	0.128965	-1.48206	-1.85961	-0.8592	-1.46839
P08133	ANXA6	-1.25817	0.043122	0.28409	-1.52026	-0.2722	-0.24568	0.484824	-0.49966	0.103752	0.11261	-0.29818	0.137455	-0.09579	0.687408	-0.27657	-0.57318	-0.53657	0.23764	0.345129
P08134	RHOC	-0.35458	-0.84916	0.092108	-0.00528	0.537402	0.538396	-0.90577	0.201793	-0.04868	0.591601	0.006412	0.192964	0.057684	0.779235	0.197511	0.54941	0.239476	0.221127	0.706733
P08174	CD55	-2.00453	-1.61557	-0.90868	-0.60066	-3.39194	-2.44811	-1.20911	-2.22605	0.177508	-2.98392	0.256047	-1.46444	-2.63466	-0.57319	0.256131	-1.20953	-1.48938	-2.72139	-2.28107
P08185	SERPINA6	-1.73817	0.822691	-0.79173	-2.09946	-0.83956	-1.24297	0.387296	-0.10501	0.374491	-0.47554	0.287615	0.05698	-0.57578	0.357872	-0.42502	-0.76559	-0.35963	-0.97912	-0.65176
P08195	SLC3A2	-0.23096	1.363798	0.461987	-0.11886	0.005732	-0.40731	0.63496	-0.05601	0.409294	0.209697	-0.61398	1.746895	0.700025	1.066268	-0.32446	0.290109	2.115074	1.392833	-0.69794
P08236	GUSB	1.595498	-0.12603	0.227698	0.074617	1.039861	0.926084	-0.43235	1.369874	-0.30475	0.435104	0.673411	0.941512	-0.36883	0.609902	-0.42314	-1.05793	-0.2739	-0.00929	-0.55535
P08237	PFKM	0.087375	-0.74289	-0.56642	-0.14082	-0.77396	-0.28448	-0.69402	0.338851	0.088326	-0.48458	-0.40848	-1.11782	-1.66479	-0.33072	0.49776	-0.81338	-0.05698	-1.03297	-0.10327
P08238	HSP90AB	0.200014	-0.09246	-0.19774	-0.1252	0.195635	0.596102	-0.03841	0.067996	-0.1116	-0.12494	0.046309	-0.22096	0.298818	-0.1756	-0.15595	0.161234	0.036819	-0.34815	-0.11919
P08240	SRPRA	0.068789	0.301334	0.512962	0.538192	-0.09995	-0.03903	0.360745	-0.20914	-0.20784	0.130212	0.031273	0.544582	0.305888	0.113778	1.530287	0.10005	-0.00949	-0.33491	0.06354
P08243	ASNS	0.088796	0.24829	-0.89489	0.360888	-0.10258	0.483688	0.304259	1.805988	-0.04434	-0.00077	-0.82097	-0.57557	0.351754	-0.45925	-0.33055	2.25994	0.235843	-0.84199	0.050533
P08246	ELANE	-2.55455	-0.38818	2.007147	-1.77976	-1.00084	-3.39039	-0.79875	-2.33512	0.298337	1.973473	-2.7616	0.162111	-1.40231	3.90147	-1.29276	-2.69671	1.541069	-2.69713	-1.06908
P08253	MMP2	-1.66912	-0.59376	-0.16137	-1.48983	-0.32201	-0.53548	-0.4332	-0.74212	0.788026	-0.20984	-1.28441	-0.52833	0.835522	-0.85281	-1.11133	-0.04972	-0.82639	0.221765	1.201227
P08294	SOD3	-0.4026	0.051662	0.674529	-0.71889	-0.34214	-1.10818	0.946474	-0.5079	1.356585	-1.41868	-0.59598	0.100912	-0.74295	0.29315	2.330731	-0.89005	-0.84673	-0.83407	-0.9425
P08311	CTSG	-2.88224	-0.44323	1.703782	-2.33324	-1.39889	-2.86993	2.194483	-4.38719	0.213019	0.613395	-2.57864	-0.15362	-1.96273	2.863667	-0.25242	-2.61302	1.007097	-0.95446	-1.78835
P08397	HMBS	0.861111	-0.41905	0.281929	0.336482	0.964411	0.013119	0.089444	-0.14082	-0.13295	-0.26235	-0.16939	0.218069	1.658454	-0.7401	0.064472	0.773726	0.30214	0.679549	0.459749
P08473	MME	-1.27777	-0.04186	-0.50741	-1.42597	-1.12321	-1.62526	-1.28937	-0.45473	1.273828	0.80498	-1.1551	-0.60189	-0.15506	1.010829	-1.76033	-0.68303	-0.75234	-1.24847	-1.807
P08514	ITGA2B	-1.07276	0.817277	0.486863	0.858644	1.88267	-2.06052	0.616445	-0.81399	0.521523	-0.05953	-1.30595	-3.00017	-0.504	-1.76539	-1.28245	-1.64287	-1.09356	2.527342	0.7388
P08519	LPA	0.058846	-0.48034	-0.64921	-0.15649	-0.09015	-0.48404	0.274979	0.386717	-0.21065	-0.39316	0.553842	-1.12302	-0.53525	-0.51738	-0.08023	-0.67561	-0.752	-1.04982	-4.19804
P08559	PDHA1	0.61445	-0.12728	-0.19315	0.190098	-0.04966	0.020386	0.08353	-0.41969	-0.76303	-0.30694	0.456431	-0.29667	-0.28724	-0.2947	1.154377	0.329703	0.002981	0.118943	-0.63222
P08567	PLEK	-1.72031	1.153607	0.39065	-0.81044	0.644036	-0.10303	0.22349	-0.93797	0.17947	1.406923	-1.35991	1.48841	0.088225	1.342479	-0.58431	-0.64801	0.039969	1.005369	-0.14433
P08571	CD14	-0.83836	3.100137	2.059674	-0.93316	0.876124	0.136904	1.132115	-1.47933	0.04825	2.398828	-1.14036	0.887621	0.466386	0.819647	-0.46728	-0.45896	-0.26788	-0.10901	-1.13122
P08572	COL4A2	-1.0549	0.129924	-0.53095	-1.86845	-0.23889	-1.35041	0.456619	-1.20347	0.31691	-0.1479	-0.41681	-1.24349	-1.18423	-1.52451	-0.13979	-0.55308	0.042749	1.145464	-1.27493
P08574	CYC1	0.779722	0.515172	-0.75445	-0.15481	0.528213	0.906899	0.130555	-0.26836	-0.39176	-0.3857	0.405667	0.14388	-0.08707	-0.00103	1.08191	-1.02914	-0.39998	-0.75706	-1.31726
P08575	PTPRC	-2.04308	1.58788	1.569833	-1.00593	0.654502	0.078049	0.512379	-1.88436	-0.19309	1.202008	-1.72988	0.955469	-0.31261	1.652567	-0.52611	-0.86624	0.274939	0.589669	-1.34932
P08579	SNRPB2	0.611963	-0.99417	-0.47787	0.574477	-0.10436	-0.07248	-0.70849	0.159128	-0.03606	-0.20324	0.542365	-0.06663	-0.3327	-0.05541	0.013209	-0.087	0.210317	0.574195	-0.56614
P08581	MET	-0.25511	0.437537	0.067899	-0.80905	-1.85921	-0.96688	0.632696	-0.56932	-0.76903	-0.87065	0.249246	0.303893	-0.54952	-0.00869	-0.07624	-0.73757	-0.18551	-0.36813	-1.64045
P08582	MELTF	0.555559	1.280268	1.266594	-0.48525	1.247872	0.6506	0.877737	0.113807	0.594429	1.642193	-0.93405	-0.91544	0.907769	-0.604	-0.71225	-0.28206	-0.65884	0.97959	1.225478
P08590	MYL3	-0.61229	-0.81487	-0.4138	-4.01553	-4.97705	-0.61244	-0.1369	-1.0035	0.255566	-4.42488	-5.42641	-1.09941	-1.00082	-2.18562	-3.67724	-4.20175	-2.72061	-6.17555	-4.51379
P08603	CFH	-1.4688	0.993254	-0.59586	-2.13471	-1.11037	-1.13296	-0.32112	-0.81266	0.536175	-0.18402	-0.45848	-0.03259	-0.16504	0.815168	-0.60461	-0.69642	0.182398	0.316368	-0.47589
P08621	SNRNP70	0.880758	-1.05848	0.066303	0.475435	0.234409	0.638599	-1.41719	0.564787	-0.43442	-0.19297	0.704766	-0.42859	-0.31347	-0.4615	0.315113	-0.22443	0.066719	0.441364	-0.18466
P08631	HCK	-1.32693	1.261428	1.425998	-0.68737	0.406323	0.475911	0.624273	-0.81722	-0.55203	1.224756	-1.06628	1.75252	0.128734	1.980258	-0.45907	-0.97895	0.724371	-0.14301	-1.05228
P08648	ITGA5	-1.82781	1.177868	0.493893	-1.17508	-0.31924	0.017405	0.694349	-0.75808	0.025663	0.270485	-1.69498	0.741919	0.815684	0.150711	-0.66374	-0.34489	0.71835	0.370858	0.994615
P08651	NFIC	0.587883	-0.15419	0.3888	-0.21206	0.87695	0.405793	-1.05111	-0.86756	-0.58445	-0.31817	0.87843	-0.49764	-0.31171	-1.15135	0.094546	-0.53376	-0.58179	-0.13075	-0.42688
P08670	VIM	-1.34942	1.386063	0.742388	-1.37177	-0.38658	-0.80433	-0.19399	-0.67462	0.16225	0.314519	-0.26324	-1.01457	0.021484	-0.64395	-0.61053	0.038514	-0.87741	0.762167	0.624457
P08697	SERPINF2	-1.76527	0.0934	-1.85618	-2.6406	-0.931	-1.6457	-0.84084	-0.96682	0.255089	-0.83732	-0.42873	1.617427	0.229698	2.23732	-1.33131	-1.68616	2.536943	0.068562	-1.28071
P08708	RPS17	0.16791	0.264047	0.072316	0.074927	0.040446	0.192281	-0.28533	0.075974	-0.16727	-0.14487	0.485998	-0.44014	0.150928	-0.62181	-0.16527	0.081673	-0.1831	-0.16244	0.160854
P08727	KRT19	-0.44313	-0.00152	0.358272	1.931764	-1.54212	-1.04278	-0.85959	-0.06537	-0.69704	-0.58109	1.167968	-0.30409	0.314619	-0.80476	0.427814	0.366541	0.347346	-0.39209	-0.30176
P08729	KRT7	0.292715	-0.70362	0.054446	2.149059	-2.75164	-2.2891	-1.58659	-0.03265	-1.03885	0.058757	1.221557	-0.64256	-0.21406	-1.07344	0.346069	-0.54076	-1.82195	-3.06716	-0.72029
P08754	GNAI3	-0.33255	0.480933	-0.42949	0.093385	0.214933	-0.07078	0.003585	-0.3	-0.30082	0.237022	-0.39936	0.412027	0.040913	1.083492	0.485638	-0.23926	-0.06129	-0.21235	0.141439
P08758	ANXA5	-1.21918	-0.60895	-0.22038	-0.9881	0.20116	-0.92104	-0.4726	-0.90528	0.185261	0.631836	0.082522	1.498331	0.037778	0.43908	-0.39671	-0.52761	0.236389	0.335831	0.64831
P08779	KRT16	-3.61941	1.176552	0.091442	-0.16612	-3.15693	-1.69374	0.983194	-1.56496	0.079973	-2.18261	-2.56831	-1.47936	-0.89171	-0.41352	-1.39427	-0.79602	-2.35795	-2.92562	-2.77086
P08842	STS	-0.6004	0.474541	-0.14417	-1.23553	-0.54446	-1.14762	1.01148	-0.22889	-0.13683	-0.35439	0.361805	-0.07283	-0.48102	-0.50801	-0.78563	-0.55452	0.339337	-0.00912	-0.98492
P08865	RPSA	0.05509	-0.09825	-0.05989	0.071033	0.14633	-0.03144	-0.84513	0.188686	-0.00428	0.111391	0.49614	-0.10347	0.311524	-0.10383	-0.17255	0.057208	0.075336	0.015992	0.462329
P08962	CD63	0.193415	0.549564	0.193745	-0.43129	-0.85325	-0.6954	0.457476	-0.3338	-0.84155	-0.62386	-0.05219	-0.45868	-0.44188	0.893358	-0.61254	-1.04852	-0.41682	-0.97395	0.052494
P09001	MRPL3	-0.30179	-0.67365	0.414997	1.269334	0.193272	0.222022	-0.48059	-0.38811	-0.17884	0.663493	0.508025	0.176742	-0.56189	0.212114	1.492848	-0.16098	0.125517	-0.43211	-0.65715
P09012	SNRPA	0.379927	-1.14592	-0.151	-0.01047	0.987883	-0.3862	-0.50213	0.302049	1.009451	1.041858	-0.22061	0.412315	-0.82995	-0.02648	-0.53852	-0.39798	0.041987	0.226249	-1.47543
P09104	ENO2	0.287842	-0.36143	-1.48609	0.009138	0.366429	-0.60472	0.088833	0.798665	1.233547	0.851998	-1.23631	0.077746	-0.05195	-0.38241	-0.6591	0.326937	0.315754	-1.67084	-0.50749
P09110	ACAA1	0.061365	0.438654	-0.01714	0.363464	1.086369	0.156645	-0.06819	-1.1466	-0.94909	-0.37702	0.135818	0.886215	0.233043	0.398044	-0.98373	-0.26718	-0.00769	0.013604	-0.93143
P09132	SRP19	-0.43564	0.24793	0.154883	-1.142	-0.35735	-0.42927	-0.05656	-0.31243	0.23966	-0.14386	-0.12834	0.059579	-0.18458	-0.01084	-0.20881	0.79361	1.073145	0.849348	-0.66093
P09211	GSTP1	-0.14017	-0.59619	-1.14927	-0.02032	1.16416	-0.62602	-0.691	2.306811	0.746574	1.492271	-0.33338	0.351295	0.436964	0.673717	-0.16414	-0.46964	0.986676	0.039142	0.161749
P09234	SNRPC	0.51952	-0.5644	-0.82144	-0.49473	1.532925	-0.5353	-0.75896	-0.58914	0.0806	0.388105	0.123805	0.338137	0.115163	-0.05137	-0.73074	-0.98548	0.529416	0.600969	-0.84928
P09237	MMP7	-1.42238	-0.97677	-0.96119	-1.77926	-1.66141	-2.13863	-0.5472	0.914839	0.435386	-1.46082	-2.1025	-0.89329	0.488847	-0.69763	-2.06678	-0.57292	-0.61644	-2.00837	0.333571
P09238	MMP10	-1.76317	-0.37697	-0.62658	-1.11316	-1.96815	-1.54736	-0.48453	-2.59642	0.471719	-2.12466	-2.03508	-0.54294	-0.24671	-0.36688	-1.68198	-0.72243	-1.57317	-2.9699	-0.0131
P09382	LGALS1	-1.47848	-1.37642	-0.48999	-1.18899	0.618335	-0.4839	-0.03361	-0.92427	0.713595	0.977915	-0.83533	0.456386	0.581055	0.242244	-1.08934	0.143095	-0.43231	0.636754	0.09741
P09417	QDPR	0.313288	0.411055	-0.77891	-0.82574	1.091893	-1.06536	0.417068	0.484379	0.907549	1.177469	-0.31292	0.508593	0.203067	0.702316	-1.12738	-0.3145	-0.04454	-0.43772	-1.57937
P09429	HMGB1	-0.19576	-0.43502	-0.27424	0.204741	-1.14694	0.734335	-1.15725	0.184007	0.02503	-0.62023	-0.11174	-0.95275	-0.06073	-0.30504	0.522813	-0.65446	0.045473	0.212184	0.412801
P09455	RBP1	-0.31317	0.222096	-1.84896	-1.20791	-0.27362	-2.17745	-0.08539	0.637082	1.424397	2.366082	0.300539	-0.00102	-0.1264	0.155455	0.750488	-0.43683	-0.55997	-1.96733	-0.24613
P09467	FBP1	-1.34809	0.010054	0.718049	-0.03833	0.844935	0.33986	0.781742	-0.51284	-0.34464	2.102029	-0.14384	0.242676	0.691336	1.256102	-1.36828	-0.68039	0.53758	0.216079	-2.06987
P09471	GNAO1	0.572167	-0.50217	-0.64962	-1.06652	-1.55694	-1.45323	-0.03358	-1.98593	0.453519	-0.53076	-0.47457	-0.76086	-0.43249	-0.08756	0.273831	-1.04135	-0.76247	-0.93884	0.377888
P09486	SPARC	-1.13056	0.892914	0.71507	-0.78205	0.438221	0.34072	-0.21227	0.504873	1.15304	0.317849	-0.89476	-1.60936	1.275182	-1.04033	-1.25231	0.550691	-0.71286	1.514295	2.890501
P09493	TPM1	-1.04551	-1.97758	-0.72292	-0.97636	-0.78913	-1.15334	-1.63964	-1.69997	0.480472	-1.60846	-1.78136	-1.2461	-0.91053	-0.77681	-0.82066	-0.2011	-1.61004	-0.60279	-0.40986
P09496	CLTA	-0.36121	-0.14917	0.71507	0.172374	-0.10672	-0.26277	-0.72762	0.568528	-0.04248	0.370382	0.176497	0.903959	0.165648	0.866212	-0.13696	0.477762	0.125579	0.419622	0.082273
P09497	CLTB	-0.12092	-0.51177	1.022043	0.833337	0.914816	0.392375	-0.84498	0.392381	-0.22648	-0.03734	-0.05982	0.181764	1.939431	-0.15235	1.434974	0.879496	-0.3565	0.106677	0.273464
P09525	ANXA4	-1.16061	-0.17707	0.623334	-0.03382	-0.49674	-0.57521	0.765585	0.897443	0.175615	1.55876	-0.32863	0.32104	-0.39152	0.127169	0.240211	-0.65634	1.63345	1.502185	-0.18808
P09543	CNP	-0.22588	0.475036	0.697799	0.206197	-0.47434	-0.51519	-0.04167	-0.31891	-0.183	-0.12752	-0.16066	0.16805	0.020509	0.056382	-0.30985	-0.04701	-0.17056	0.328323	-0.16856

P09601	HMOX1	-0.75758	1.252452	0.997498	-0.39284	0.146052	-0.14008	0.732741	-1.10216	1.13047	0.193908	-1.16026	1.238791	0.230716	0.84419	-0.29578	-0.47547	-0.67755	0.069232	-1.22441
P09619	PDGFRB	-1.02024	-0.12246	-0.07434	-0.80047	-0.22346	-0.98568	0.000756	-1.1895	0.056048	0.033551	-1.09121	-0.6766	-0.56271	-0.88712	-0.14458	-0.99475	0.130598	-0.07361	
P09622	DLD	0.220746	-0.96449	0.038943	0.777443	0.817034	0.485223	-0.45081	0.277675	-0.15139	0.041014	0.66525	-0.65491	-0.05636	-0.73908	2.40754	1.877553	-1.16301	-0.31951	-0.69775
P09651	HNRNPA1	0.392514	-0.07542	-0.10444	0.418068	0.471278	0.857012	-0.45977	0.600681	0.043701	0.012528	0.70041	-0.16659	-0.07694	-0.27227	-0.12145	-0.60933	-0.15143	0.397757	0.423214
P09661	SNRPA1	0.590924	-0.56318	-0.49701	0.3245	0.383364	0.168788	-0.4014	0.192191	0.12719	0.009197	0.246363	0.194415	0.027956	-0.00091	-0.0084	-0.20431	0.469902	0.355882	-0.47194
P09669	COX6C	-0.05463	-0.06408	-0.00485	-0.07363	0.931495	0.708265	-0.02869	-0.75398	-0.6369	-0.81292	0.667521	-0.09803	-0.0672	-0.10001	1.807745	0.531872	-0.32185	-0.37883	-0.72084
P09758	TACSTD2	1.475684	-1.9974	-1.39167	0.881539	-1.70337	-0.14194	0.190696	1.122533	-1.59278	-0.37397	0.026141	-0.76973	-1.75119	-0.78795	1.401096	0.205883	-0.70666	-1.67315	-0.5812
P09769	FGR	-1.21869	1.189601	1.073559	-0.52247	0.233153	-0.72824	0.143156	-0.80097	-0.237	1.772592	-1.03435	1.450193	0.031717	2.882199	-0.52801	-1.18348	1.378166	-0.72809	-1.40364
P09871	C1S	-1.45407	0.792253	-0.16084	-1.98923	-0.21447	-0.59804	-0.25879	-0.74698	0.779185	1.005472	-1.04761	0.275196	0.321142	1.024363	-0.9993	-0.45425	0.136871	0.578563	-0.1155
P09874	PARP1	0.68947	-0.93196	-0.44488	0.352085	-0.36806	-0.09905	-0.25267	0.702052	-0.33344	0.431862	-0.32132	-0.64694	-0.34554	-0.88763	0.582542	-0.2598	-0.77982	-0.47752	-0.67026
P09884	POLA1	1.58225	-0.2728	-1.07141	0.152127	1.422539	1.183107	-0.13923	0.056856	1.765026	-1.58622	-0.27739	-0.63585	-0.42999	-0.36523	-1.05709	-3.0755	0.252188	0.537408	-0.18104
P09913	IFIT2	0.169151	2.021309	2.934867	-0.83132	-0.31051	0.181221	-0.97889	-1.16785	-0.61749	0.081254	-0.97007	0.258223	0.097451	1.168525	-0.63606	-1.40716	0.020664	0.494051	-1.52001
P09914	IFIT1	0.566107	1.748946	2.828349	-1.1783	-0.41385	-0.02736	-0.44611	0.255675	-0.45404	-0.56197	-1.81425	0.644333	-0.02138	1.105551	-0.86797	-1.61661	-0.2568	-0.15082	-1.8735
P09917	ALOX5	-0.92691	1.127933	1.08321	-1.08121	0.855633	0.42419	0.447249	-1.13668	-0.21698	0.962018	-1.0452	1.082605	0.097342	2.289061	-0.85641	-0.91042	0.565896	-0.21476	-1.34215
P09936	UCHL1	0.263132	-1.3824	1.568371	-3.45481	-1.84681	-1.62947	0.012106	2.561453	-0.29281	-0.1647	-2.72941	-1.27161	0.029149	-1.37828	-1.53063	-1.45423	-1.90834	-1.7402	1.22285
P09960	LTA4H	-0.53495	0.680974	-0.33481	-0.10763	-0.32151	-0.19035	0.075179	0.609186	-0.49659	1.172241	0.123822	-0.29833	-0.07686	0.632692	0.177432	-0.68157	-0.33457	-0.25776	-0.11326
P09972	ALDOC	-0.38414	-0.5166	-0.3027	0.815064	0.076782	-0.30728	-0.62111	-0.37223	0.086066	-0.16952	-0.22222	-0.14726	0.373934	0.069974	-0.40324	0.34605	0.343216	-0.27076	-0.15254
P0C0L4	C4A	-1.06066	0.198069	-0.67926	-2.724	-0.59603	-1.38819	-0.30083	-1.49607	0.928684	-1.67632	-0.64816	1.152144	-0.50425	1.281598	-0.40503	-1.69552	1.220507	-0.33455	-0.76783
P0C0L5	C4B	-0.42516	1.016244	-0.29047	-1.67757	-0.44335	-0.63809	-0.07811	-0.74204	0.219095	-0.1341	-0.17455	0.167767	-0.39328	0.902768	-0.91059	-1.11587	0.218405	0.03944	-0.1239
P0C2W1	FBXO45	0.055333	0.033983	0.053405	-0.00349	0.27135	0.723144	0.572369	0.373186	-0.08474	-0.41738	-0.08462	0.152717	0.018963	0.328857	0.729271	-0.1635	-0.04274	-0.03664	0.116006
P0C7P0	CISD3	0.006092	0.431789	0.567759	0.01915	1.167767	0.257518	0.774163	-1.31769	-0.20363	-0.68732	0.184839	-0.22316	0.551434	0.383505	0.365079	0.269978	0.110983	-0.08372	-0.36061
P0C7T5	ATXN1L	0.121546	0.038826	0.096775	-0.84708	-0.43853	-0.71148	0.156812	0.134825	-0.52218	-0.11332	0.209584	-0.17528	-0.07109	-0.0562	-0.07651	0.056315	-0.51544	-0.05131	-0.04325
P0C7X5	ZNF806	-0.55709	-0.03394	-0.24383	0.221299	0.60966	-0.10478	-0.2373	0.220501	-0.32168	-0.23788	0.575468	0.380344	-0.32986	-0.79215	-0.39141	-0.09199	-0.98965	-0.66095	-0.50873
P0C870	JMJD7	-0.88362	0.046893	-0.13346	-0.63362	-0.55675	-0.35311	0.201698	-0.52724	-0.00589	-0.056	0.300679	0.111681	-0.10044	-0.71179	-0.79057	-0.05747	-0.8697	-0.82254	0.14682
P0CG38	POTEI	-1.25381	1.391871	0.627477	-1.04315	1.260479	0.077334	0.446145	-1.27293	0.194654	0.994381	-0.95027	0.935617	-0.01399	0.237655	-0.25342	-0.5078	-0.7215	0.282056	-0.95746
P0CG39	POTEJ	-0.39783	-0.59599	0.156289	-0.41231	0.385457	-0.46447	-0.61222	-0.33267	0.279874	0.286396	-0.51822	-0.22521	0.195931	1.572601	-0.22636	-0.15035	-0.05673	0.232207	0.222661
P0DJI8	SAA1	-1.55113	-0.09395	-4.07407	-4.97704	2.869774	1.42989	-1.02909	-1.37842	2.445323	2.464433	3.259294	-0.67561	0.150524	0.698363	-1.89913	-0.97934	-0.38133	-0.39697	-1.41892
P0DMN0	SULT1A4	-1.6487	0.20857	1.023078	-0.68757	1.680668	0.654154	0.035024	-0.49671	-0.64121	-0.24583	-0.21452	2.152879	1.325727	1.364033	-0.26292	0.18095	1.052866	-0.07322	-0.97668
P0DMV9	HSPA1B	0.045545	-0.63363	-0.38775	0.337376	0.693093	0.760119	-0.77583	-0.2633	-0.43745	-0.54414	-0.09657	0.013836	-0.43	0.333716	0.825328	0.072575	0.720141	-0.00587	-1.01619
P0DP23	CALM1	0.436157	-2.12615	0.58277	0.664315	1.29056	0.703108	-1.35024	1.495493	0.325965	1.394564	0.884988	-0.35112	0.988545	0.052324	0.543528	1.119526	0.041567	0.626758	0.360951
P0DPB6	POLR1D	-0.02806	0.27499	0.2036	-0.35342	-0.53254	0.766206	0.182186	0.197944	0.185254	0.406313	-0.62683	0.013527	-0.10836	-0.04349	0.307692	0.179094	0.496105	-0.37729	-0.72536
P10109	FDX1	-0.44307	0.577872	-0.02863	-0.62879	0.024508	-0.70646	-0.21658	0.413408	0.760098	0.094013	-0.16075	0.456269	0.374029	0.23543	0.165332	-0.14372	0.350746	0.787996	-1.13069
P10114	RAP2A	-1.00768	1.04303	0.287884	-1.45609	0.430239	-0.29142	0.599276	-0.37949	-0.2014	0.611946	-0.6153	0.641979	-0.16539	0.715815	-0.06049	-0.50312	-0.33096	0.049418	-0.29748
P10155	TROVE2	0.372776	-0.39565	-0.15372	-1.29408	-0.3225	-0.47111	-0.40883	-0.11423	-0.17736	-0.25693	0.628627	-0.60153	-0.40061	-0.4893	1.024594	-0.7224	-0.96507	-0.74552	-0.14369
P10253	GAA	-0.0174	-0.31012	0.158774	0.321006	0.884756	0.072999	0.534389	0.588107	1.176607	0.23443	0.012522	0.676261	0.764137	0.526288	0.216478	-0.5245	0.186237	0.91382	0.227266
P10301	RRAS	-0.27239	0.076196	-0.63675	-0.38228	-0.42909	-0.90416	0.30496	-1.31776	0.485959	-0.49389	-0.14109	0.093877	-0.33144	-0.13452	0.084688	-0.48625	-0.25105	0.009671	0.084899
P10398	ARAF	-0.32578	0.163692	-0.66172	0.060011	-0.67312	-0.22386	0.494459	0.535163	-0.33095	0.923454	-0.13658	0.042533	-0.135	0.644035	-0.10787	-0.07194	0.163866	-0.34028	-0.54593
P10412	HIST1H1E	0.133767	-0.63914	-0.14549	-0.38323	0.304081	-0.71449	-0.73254	-0.91442	-0.26056	-2.31315	-0.2365	-0.51618	-1.06309	0.172692	0.315438	-0.21452	0.31513	0.627046	-0.33856
P10451	SPP1	-2.88721	-3.2132	-2.76787	-4.56843	-1.77775	-3.20225	-0.93447	-1.32099	-0.17589	-3.48517	-1.27223	-1.53335	-4.31907	-0.19451	-0.08848	-2.6867	-1.42446	-1.1348	-1.58214
P10515	DLAT	0.351689	-0.11745	-0.448	0.144289	-0.09781	-0.11002	-0.10639	-0.4718	-0.12627	-0.0849	0.244414	0.483557	0.805304	0.024101	0.692508	1.150018	0.02695	-0.26322	-0.75052
P10586	PTPRF	0.388228	-0.21759	0.387044	-0.4203	0.808702	0.530751	0.215085	0.032065	0.489953	0.730722	-0.48892	-0.69406	-0.38601	-0.35666	-0.5179	0.062884	-0.29017	-0.6752	-0.65164
P10588	NR2F6	-0.12301	-0.23724	-0.19273	0.004786	-1.19596	-0.79522	0.276897	1.090348	0.071582	-0.85692	0.051238	-1.4549	-0.12645	-0.82605	-0.51028	0.542935	0.287472	0.280781	0.031248
P10599	TXN	-0.11928	1.619345	0.34756	-0.50372	1.319817	-0.45784	0.876021	-0.24731	1.85988	0.025167	0.418513	-0.33691	0.011202	0.856302	-0.21152	-0.23395	0.283141	-0.57645	-0.75717
P10606	COX5B	0.087353	-0.65419	0.031694	0.070621	1.516638	0.616563	-0.20187	-0.51347	-0.01083	-0.37354	0.421096	0.319413	0.143418	0.317	1.554767	0.524549	-0.19408	0.12224	-0.73062
P10619	CTSA	-0.23439	0.195062	0.260216	-2.15277	1.125155	-0.00126	-0.05999	0.616648	0.98887	-0.2106	-1.0638	-0.33763	0.082595	-0.14972	0.117621	-0.29192	-0.54599	0.770207	0.068018
P10643	C7	-2.19466	0.376224	-0.43624	-1.96222	-0.49943	-0.96504	0.290356	-0.62989	-0.34283	-0.48009	0.148101	1.163519	-0.39853	0.770294	-0.7203	-1.07784	1.516193	-0.64622	-0.99451
P10644	PRKAR1A	-0.68965	0.36293	0.331502	0.343229	-0.13854	-0.28694	0.202007	0.195123	-0.32537	-0.17438	0.601043	0.463139	-0.2509	0.535795	-0.04034	0.194477	0.262759	-0.06111	-0.59683
P10768	ESD	-0.57913	-1.0188	-0.76176	-0.59275	1.189307	-0.76122	-0.77264	0.098886	1.156399	1.222749	-0.34712	0.149601	0.59227	0.418696	0.360021	-0.19949	0.71592	0.693869	0.472161
P10809	HSPD1	0.777175	-0.37512	-0.32117	0.685752	-0.02411	-0.20791	-0.79453	0.029856	-0.35194	0.212028	0.473329	0.212619	0.249343	-0.04324	0.448399	0.97907	0.454706	-0.02223	-0.29491
P10909	CLU	-1.26019	-0.65429	-1.71547	-2.03372	-1.05171	-1.33093	-1.45167	-0.95554	-0.35488	-1.93025	-0.79364	0.545685	-0.65115	1.370357	-0.88856	1.814522	-1.75965	-1.56326	
P10915	HAPLN1	-1.06671	1.206833	-0.64985	-2.42403	-5.15301	-2.1164	-0.20666	1.161519	0.142406	-1.28769	-1.45346	-2.73857	-0.57566	-0.74873	-0.81109	-1.17459	-1.00308	-2.77236	-3.64602
P10916	MYL2	-1.28103	-3.95908	-0.56505	-3.91623	-6.1129	-4.2149	-0.0596	-5.35306	-1.07972	-3.82987	-5.26678	-2.55495	-6.01478	-3.74063	-5.27922	-4.35502	-2.69705	-6.91638	-3.69229
P11021	HSPA5	-0.06957	0.339399	0.575161	-0.35994	0.421604	0.135593	0.194517	0.376616	0.323852	0.231407	-0.08375	1.228088	0.739907	0.676514	0.028011	-0.01765	0.759449	0.776479	0.262911
P11047	LAMC1	-0.75963	-0.04097	-0.66452	-1.48893	-0.53141	-1.35441	0.177543	-0.93268	-0.38842	-0.08274	-0.43063	-1.45519	-0.77093	-1.15865	0.521324	-0.84905	0.057105	1.223884	-0.72832
P11117	ACP2	0.356951	1.055521	0.894709	-0.29756	0.390398	-0.3844	-0.03758	-0.53075	-0.43751	0.460333	-0.64929	1.375513	0.719372	1.586483	-0.72947	-0.01808	0.869213	0.642711	0.058744
P11137	MAP2	0.289334	-0.59719	-0.26689	-0.94242	-1.6983	-0.78421	-0.45007	0.761045	0.164439	-1.31681	-1.42313	-0.19458	-0.20058	-0.08287	-1.26985	-0.82385	-0.77424	0.734086	
P11142	HSPA8	0.18298	-0.11425	0.259229	-0.01865	0.221046	0.297601	-0.32449	0.167993	0.1872	0.412495	0.145957	-0.09885	0.490585	-0.09642	-0.01062	0.279074	0.204784	0.126026	0.130554
P11166	SLC2A1	0.575563	-0.24325	-0.41552	0.978214	-1.10399	-1.09192	0.303369	1.316449	0.150756	-1.35195	-0.86664	0.910089	0.086442	0.320926	-1.21191	-1.03607	1.47403	-0.32309	0.167563
P11169	SLC2A3	-2.3404	0.70699	0.505435	-1.10911	-0.33934	-0.89988	-0.1158	-0.96969	-0.01865	0.40462	-2.73728	2.550462	-0.10051	2.151421	-1.05115	-1.3295	2.511092	0.151606	-0.4788
P11171	EPB41	-0.50493	0.550826	0.085767	-0.33641	-0.46553	-0.96641	0.592675	-0.4879	0.784118	-0.3982	-0.92892	-1.14877	-0.33434	-0.81435	-0.40724	-1.04741	-1.01919	1.023474	-0.57825
P11172	UMPS	0.151414	-0.60263	-0.15976	1.007055	0.514352	0.791263	-0.30909	0.216221	0.256587	0.341896	-0.30067	0.057307	-0.12932	-0.09684	1.078678	0.449814	0.44276	-0.76326	-0.22935
P11177	PDHB	0.61567	-0.30494	-0.23858	0.342382	0.038812	0.113321	-0.21069	-0.26669	-0.52988	-0.40836	0.352079	-0.01952	-0.45877	-0.21196	1.099043	0.413885	0.125169	0.322693	-0.44101
P11182	DBT	0.668582	-0.28229	-0.828	-0.01807	-0.28301	-0.12235	-0.22422	-0.06704	-0.53483	-0.02255	0.039527	-0.2329	-0.23535	-0.24052	1.32825	1.019012	0.192319	-0.18941	-0.78356
P11215	ITGAM	-2.09285	0.724561	1.391779	-0.77935	0.174272	-0.72164	0.001497	-1.84198	0.089404	1.626843	-1.15944	1.605538	-0.44561	2.365391	-0.84031	-0.9676	0.842494	-0.87917	-1.44679
P11216	PYGB	0.274431	-1.20004	-0.30412	0.163118	-0.53084	-0.92578	-0.94568	0.552325	0.262624	0.512773	-1.21291	0.06553	0.135448	0.091787	1.258056	-0.54334	-0.1093	-1.02428	0.04259
P11217	PYGM	-0.94717	-1.45756	-0.27697	-1.86027	-0.88057	-1.78308	0.021442	-0.76571	0.688577	-0.48846	-2.16818	-0.35428	-0.84983	-0.42852	-0.50686	-1.1169	-0.3669	-0.92228	-0.58147
P11233	RALA	0.311321	-0.24375	-0.17809	-0.51409	0.156702	-0.09366	-0.1749	0.076279	0.046872	0.046492	0.115182	-0.01497	-0.14306	-0.2773	0.195589	0.312265	0.096339	-0.27348	-0.20212
P11234	RALB	-0.06137	-0.58328	0.242274	-0.35362	0.080172	-0.3147	0.083391	-0.11438	0.271179	0.301218	-0.37349	0.276488	-0.04007	0.655302	0.282936	-0.05255	0.174443	0.360088	0.608412
P11274	BCR	0.266323	0.510214	-0.44596	0.16616	-0.32791	-0.31948	-0.21516	-0.26505	-0.12469	0.107122	-0.05519	-0.3627	-0.35633	-0.08576	0.367865	-0.09206	-0.15325	0.039122	-0.21892
P11277	SPTB	-1.61328	0.958703	0.225313	-0.70634	-0.38385	-1.65077	1.358558	-1.16817	0.806816	-0.92864	-1.61018	-1.43735	-0.7031	-1.03739	-1.75697	-1.81136	-0.95459	1.768384	-0.72011
P11279	LAMP1	-0.70188	0.015795	-0.13808	-0.9117	-0.2675	-0.19458	0.553504	0.213721	-0.7991	0.179837	-0.47733	0.99702	0.164726	0.624395	-0.19096	-0.5938	0.338695	-0.40598	-0.39047
P11310	ACADM	0.65374	-0.55996	0.671367	-0.2337	0.574986	0.427209	-0.06075	0.02374	0.219194	0.101049	0.878928	0.061328	0.336773	-0.12847	2.21535	-0.1486	-0.52273	0.097442	-0.79224
P11387	TOP1	0.566713	-0.37094	0.083851	0.051323	-0.2695	0.265842	-0.01042	0.652225	-0.53397	0.072069	0.27979	-0.32814	-0.2902	-0.51851	-0.09282	-1.13087	0.377477	0.796628	0.177384
P11388	TOP2A	0.96425	-0.69353	0.659849	0.330492	1.873461	2.242936	0.869404	0.880411	0.097743	0.185063	-1.22385	-0.48606	-0.23135	-0.51828	-1.03018	-0.07586	0.305587	-0.44158	0.083482
P11413	G6PD	-0.76896	0.111062	0.398116	0.231237	-0.24631	0.038283	-0.07407	0.217548	-0.4227	0.489562	-1.1225	0.341097	-0.48423	1.923956	-1.19246	-0.32419	0.954581	0.281134	-0.15915
P11441	UBL4A	-0.07039	-0.03213	-0.4771	-0.18545	0.720801	0.320213	0.822636	0.83851	-0.24572	-0.15551	0.065593	-0.05395	-0.53741	-0.08676	0.43913	-0.04908	1.369876	0.344589	-0.73774
P11498	PC	-0.984	-0.42666	0.020825	-0.32914	-0.5813	-0.3766	0.137318	-0.62453	0.14874	-0.84339	-0.57876	-0.19146	-0.96901	-0.32443	0.088875	2.497204	-0.14764	0.648488	-0.92792
P11532	DMD	-1.18389	-1.15308	-0.52323	-1.17732	-1.41569	-1.78373	0.173145	-1.3782	0.145743	-1.55023	-0.11051	-0.92894	-1.09426	-0.52796	-0.36819	-1.33371	-1.49851	-0.76708	-0.14196
P11586	MTHFD1	1.276663	-0.276	-0.24005	0.271454	-0.18378	0.260154	-0.28355	0.043702	-0.53207	0.020628	0.10182	-0.01635	-0.40798	0.230488	-0.59514	-0.41321	-0.00254	-0.60273	-0.58312
P11678	EPX	-3.19374	1.005836	1.095588	-1.92388	0.771688	-1.4398	1.865118	-2.01151	0.351109	3.157332	-1.75134	-1.83399	-1.8026	-0.36064	-2.14296	-1.0775	-1.14353	-0.9945	-1.68347
P11717	IGF2R	-1.01053	0.685171	0.651348	0.084374	-0.22899	-0.05734	0.433724	-0.50824	-0.20362	0.47058	-0.78547	-0.23485	-0.10226	0.310556	0.266746	-0.21028	-0.28165	0.255664	0.304558
P11766	ADH5	-0.30247	-0.95544	-0.44677	-1.22171	-0.24868	-0.17543	-0.77626	-0.27669	-0.28706	-0.85325	-0.00843	-0.44747	-0.42422	-0.27431	-0.16416	-0.61896	-0.2097	-0.0325	0.169717
P11802	CDK4	0.921256	0.263277	-0.33819	0.181447	0.770212	0.947379	-0.39006	0.022123	-0.41501	-0.79183	-0.3633	-0.63315	0.471735	-0.73676	-0.3561	-0.67569	-0.22973	-0.57436	0.205336
P11908	PRPS2	0.899303	-1.09187	0.129292	0.123549	0.362134	-0.13427	-1.22354	-0.3006	0.071459	-0.11327	0.168165	-0.17914	0.108402	0.36672	-0.81456	0.509544	0.173632	0.121682	0.277699
P11940	PABPC1	0.134392	-0.22573	0.510145	-0.08361	0.112909	0.472351	0.05078	-0.01263	-0.1012	0.069917	-0.11307	0.148529	-0.04953	-0.01974	-0.21185	-0.07059	0.130652	-0.02981	0.013216
P12004	PCNA	1.497044	-1.22224	0.166101	0.899769	1.835239	1.923436	-0.33072	1.785123	0.164161	0.673749	-0.0263	-0.64725	0.180075	-0.16547	-0.58292	-0.33408	0.739461	-0.08438	-0.37542
P12035	KRT3	-1.29398	-0.90969	-3.38698	-0.67852	-0.86543	-1.09726	1.00983	1.919637	0.017813	1.296629	-1.60499	-0.787	-0.61904	0.013575	3.57897	-0.85917	-0.6878	-2.24208	-1.15482
P12081	HARS	-0.27875	0.05558	0.503467	0.719269	-0.04212	0.233289	-0.58554	0.473297	-0.05392	-0.08932	-0.02276	-0.8505	0.374339	-0.53444	0.247603	0.498156	-0.35821	0.354298	0.331434
P12107	COL11A1	-1.51423	-1.21142	-1.39227	-0.98723	-1.87603	-1.60027	-1.3856	-0.54534	-0.23939	-2.97824	-2.5983	-1.75051	-0.55477	-1.22261	-1.81886	0.627574	-2.12595	-2.62524	-0.34272
P12109	COL6A1	-1.58009	-0.18717	-0.21984	-2.06272	-0.77986	-0.84668	0.332184	-1.56026	0.414556	0.132843	0.691918	-1.37333	-0.5025	-1.09691	0.041912	-0.08324	-1.07087	-0.33853	-0.46209
P12110	COL6A2	-1.36386	-0.05727	-0.14739	-1.55974	-0.69393	-0.74855	0.393811	-1.30565	0.425507	0.230173	0.71017	-0.6811	-0.23246	-0.50035	0.206293	-0.00079	-0.50707	-0.21739	-0.25315
P12111	COL6A3	-1.5357	-0.19057	-0.41526	-1.74718	-0.89793	-0.95846	0.143515	-1.556	0.377041	0.238635	0.845139	-1.40068	-0.36759	-1.02964	-0.10209	-0.06303	-0.92445	-0.21535	-0.44455
P12235	SLC25A4	0.112848	-0.92287	-0.51624	0.928115	-0.08364	-0.03326	-0.40564	-0.06898	-0.48106	-0.28079	0.31776	-0.52917	-1.11957	-0.00999	0.168309	1.558361	-0.29007	-0.32794	-0.48394
P12236	SLC25A6	0.075138	0.146816	-0.24481	0.442912	0.528818	0.884375	0.133703	-0.03629	0.140687	0.228219	-0.76934	0.446164	0.104581	0.058686	-0.00137	1.516151	0.196793	0.210758	-0.57728
P12259	F5	-0.30317	0.86816	-0.43992	-1.21854	-0.253	-1.7061	-0.86844	-1.4309	-0.11152	-0.21192	-1.30513	1.184125	-0.43815	1.745848	-1.13009	-0.39086	2.615688	1.324551	-0.88562
P12268	IMPDH2	0.308833	-0.81766	-0.29839	0.121614	-0.0222	0.097586	-0.65778	0.148135	-0.28623	0.080267	0.278408	0.32223	0.806417	0.434177	-0.05753	0.060499	0.265963	-0.59422	0.118833
P12270	TPR	0.280719	0.127442	-0.05975	0.395325	0.08204	0.30296	-0.2102	0.285691	-0.47441	-0.36269	0.219958	-0.42016	-0.29911	-0.50438	0.860945	-0.35973	-0.49726	-0.02444	0.141789
P12277	CKB	0.594633	-0.63189	-0.53928	-0.90574	-1.07168	-1.80873	-0.36264	1.905479	0.415181	-0.37321	0.144261	-0.07151	-0.04685	-0.20456	1.995754	-1.85911	-0.73424	-1.21129	-0.13719
P12314	FCGR1A	-1.15838	1.715532	1.043184	-1.1657	0.5057	-0.234	0.236449	-1.30051	-0.17338	0.498649	-1.41456	1.060136	0.624225	1.131445	0.093904	-0.05239	0.05757	0.621254	-0.80418
P12318	FCGR2A	-2.12959	0.942433	0.546057	-0.68064	0.858011	0.488908	-0.16201	-1.63343	-0.03621	1.200464	-1.54953	1.084033	-0.0402	0.491022	0.009088	0.234803	-0.31056	0.171024	-1.49261
P12429	ANXA3	1.144001	-1.9944	0.524462	-0.48873	-1.12236	-1.35443	-1.06307	1.412473	-0.65498	0.584261	-0.35173	0.762025	-1.03936	2.361112	-0.87662	-2.41542	-1.11139	-2.82474	0.24525
P12532	CKMT1A	0.817223	-0.37153	-0.49188	1.063699	-1.14255	-0.57292	0.358226	-0.69732	-0.23886	0.015165	0.825465	-0.04643	0.108095	-0.41028	1.613921	-2.34365	-1.37317	-1.72953	-0.8743
P12694	BCKDHA	1.138747	-0.3497	-0.23314	0.456891	-0.63569	-0.77192	-0.26015	-0.275	0.286499	-0.40558	0.745625	-0.12795	-0.36083	-0.07318	1.766543	0.236955	-0.95428	-0.73591	-1.08241
P12814	ACTN1	-0.16887	-0.63952	-0.31376	-0.40996	-0.96016	-0.63029	-1.03199	0.287745	0.583122	-0.45382	-0.18218	-0.91798	0.772531	-0.40563	-0.40958	0.062307	-1.06853	-0.27974	1.051099
P12821	ACE	-1.25922	0.340409	-0.23382	-0.99789	-0.43651	-1.10467	0.486274	-0.48783	0.00659	0.079779	-0.74804	-0.49824	-0.26316	-0.53	-0.84028	-0.8448	-1.266	-0.18214	-0.76925
P12830	CDH1	1.393422	-0.90604	-0.86471	0.947969	-1.27687	-0.74509	-0.47173	0.496232	0.248599	-0.36259	0.31545	-1.30471	-0.66026	-0.53859	1.762292	1.175861	-0.48877	0.545793	-1.25649
P12883	MYH7	-1.25741	-3.22906	-0.43385	-3.82476	-6.41177	-5.60764	-0.09898	-6.3713	-3.10239	-5.3492	-6.29002	-3.44612	-4.18537	-3.46698	-5.70414	-4.90618	-3.2356	-6.87695	-4.78536
P12931	SRC	-0.10011	-0.1544	-0.34808	-0.11084	-0.21586	-0.60319	0.019321	0.337467	0.01719	0.171966	-0.30939	0.051769	-0.14532	-0.03447	-0.79411	-0.83908	0.840522	0.766102	0.000844
P12955	PEPD	-0.306	-0.77134	-0.04816	0.06886	2.356665	1.213409	0.196591	1.377925	0.99435	1.167314	-0.95832	-0.05667	1.077975	-0.45226	0.23613	0.02738	-0.42681	0.289415	1.008732
P12956	XRCC6	0.161711	0.004864	-0.21307	-0.00099	0.238479	0.386257	-0.43111	-0.14712	-0.46214	-0.38688	0.806864	-0.37949	-0.39657	-0.3571	0.052439	0.149404	0.007474	-0.00164	-0.43591
P13010	XRCC5	0.15039	-0.15082	-0.25862	0.025797	0.240768	0.528233	-0.37285	-0.13325	-0.51736	-0.29254	0.772992	-0.14312	-0.39008	-0.14151	0.251797	0.337881	0.102389	-0.11172	-0.35418
P13051	UNG	0.914526	0.20735	-0.13026	1.162606	0.402556	0.515802	-0.57776	0.022833	-0.34387	-1.26292	0.052589	-0.19875	-0.14191	-0.10065	0.324646	1.953215	-0.29969	-0.71217	-0.29125
P13073	COX4I1	-0.23238	0.103083	0.388757	0.01846	0.54364	0.688192	-0.01321	-0.14139	-0.47425	-0.63846	0.515556	-0.12802	-0.06314	0.044132	1.788118	0.823768	-0.29182	-0.14543	-0.51595
P13284	IFI30	-0.75874	0.214196	2.056557	-0.55964	0.526379	-0.01433	1.138862	0.242964	-0.05884	0.898212	-1.08294	1.794039	1.403621	1.428259	-0.36525	-0.61893	-0.10608	0.802368	-1.94803
P13473	LAMP2	1.810438	-0.48518	0.035431	-0.52561	0.357173	0.384909	-0.04973	0.164424	-0.44004	1.135789	-0.13305	0.480265	0.015392	0.403823	-0.68505	-0.14694	0.346304	0.762899	-0.43619
P13489	RNH1	-1.49965	0.533258	0.030817	-0.99007	-0.02067	0.397088	0.511128	0.666208	-0.01702	0.433506	-0.22481	-0.04706	0.046424	0.113906	-0.42223	-0.22936	0.044941	0.377087	0.211089
P13497	BMP1	-0.93673	-0.23485	-0.12111	-0.25439	-0.32638	-0.50073	0.408705	-0.32054	0.300759	-0.84535	-0.72211	0.276867	0.196531	-0.16088	0.116116	-0.58793	1.888697	-0.15731	0.767056
P13498	CYBA	-2.77025	1.523732	1.133463	-1.12792	-0.35895	0.024578	0.090355	-2.37904	-0.77598	1.767627	-1.39622	2.524944	-0.36156	2.100937	-0.50866	-0.94401	0.672015	-0.46579	-1.95738
P13591	NCAM1	-1.16606	-1.7123	-1.61514	-2.32209	-1.0396	-1.06885	1.002277	1.935922	-0.25678	-0.38728	-0.91337	-1.05606	-0.03792	-0.99019	0.944494	-0.66949	-1.97446	-3.23734	-1.295
P13611	VCAN	-1.57473	-0.78264	-0.42608	-1.73588	-1.64156	-1.19983	-0.48157	-0.61037	0.831931	0.366077	-1.30673	-1.4421	0.737198	-0.74942	-0.45576	0.01936	-0.75874	1.668739	2.12423
P13639	EEF2	0.185214	-0.34677	0.174622	0.178164	-0.11109	0.148967	-0.459	0.065786	0.06679	0.553869	0.449616	-0.03972	0.233204	-0.25122	-0.02684	0.052956	-0.08914	-0.19556	0.450085
P13645	KRT10	-2.76691	3.465165	0.742188	-1.59143	-1.79155	-1.04676	2.882448	-0.53613	1.560555	-1.96951	0.251675	-1.18714	0.041552	-0.17656	-0.66203	-1.06353	-0.75507	-1.07571	-0.21266
P13646	KRT13	-2.69313	-0.4615	1.289781	0.993752	-1.85694	-1.07894	-0.28388	-0.28102	0.04153	-0.7902	0.961742	-0.45583	-0.11111	-0.98061	-0.17373	0.736453	0.316303	-0.33287	-0.19622

P13647	KRT5	-2.9884	2.949989	0.475666	3.195809	-2.65532	-1.13474	2.500485	1.466838	1.086744	1.108853	-1.52789	-0.24815	0.907634	-0.38554	3.480607	-1.31645	-1.38366	-2.30796	-0.08928
P13667	PDIA4	0.005012	0.062631	-0.04481	1.090766	0.251223	0.09133	-0.54877	0.703102	0.024059	0.314804	-0.37751	-0.03207	0.281258	-0.2703	-0.23344	0.400464	-0.25598	-0.06423	0.119366
P13671	C6	-1.91408	0.828285	-1.31589	-1.98319	-0.71031	-1.40632	-0.80306	-0.55601	-0.12853	-0.81328	0.091573	2.069884	-0.26638	1.799666	-0.74459	-0.70142	2.00528	0.536677	-0.85029
P13674	P4HA1	0.578133	0.086363	-0.43186	0.089305	-0.23299	0.037117	-0.85707	1.131087	0.115829	-0.04856	-0.30974	0.09667	2.138088	0.096771	-1.78724	0.330859	1.760403	0.806847	1.396391
P13686	ACP5	-0.99363	-0.1867	-1.19879	-1.50112	-1.16408	-1.47847	-0.08915	-0.77819	0.558713	-0.90643	-1.55241	-0.13555	-0.50232	-0.09641	-0.77544	-1.80524	-0.18141	-1.15702	-0.77895
P13693	TPT1	-0.66624	0.183893	-0.21694	-0.4997	0.481519	0.160125	-0.32794	0.350469	0.651901	1.442829	-0.39853	0.268553	1.071299	0.399353	-0.52867	-0.06323	0.992682	0.406965	0.591705
P13716	ALAD	-0.30301	-0.79972	0.319975	0.035275	0.626761	-0.44864	0.004653	-0.17383	0.768858	-0.48763	0.473095	-0.65038	-0.36147	-0.21065	0.660937	-0.30773	-0.75049	0.127826	-0.00969
P13747	HLA-E	-1.3958	0.905851	1.247866	-0.20655	0.578319	0.116689	0.377637	-1.28699	-0.17998	-0.94974	-0.45011	-0.37314	0.72798	0.259379	-0.30933	0.107572	0.33143	0.133578	-0.76544
P13760	HLA-DRB1	-2.43122	2.213717	1.492308	-1.53268	-4.61059	-0.71749	-0.00304	-2.22259	-0.83516	1.080212	-3.63319	-2.32647	-1.93616	-1.66131	-3.93968	1.733112	-1.12411	-5.21434	-1.78761
P13762	HLA-DRB4	-1.26977	1.986585	-0.00966	-3.76159	-2.71149	-0.41196	0.478485	-2.64325	-0.0974	1.010749	-4.7879	-1.60509	-1.1688	-2.13129	-2.77268	0.497464	-1.62048	-3.04844	-2.09692
P13796	LCP1	-2.83503	1.700116	0.700697	-1.22265	0.989786	0.373712	0.318823	-1.48782	-0.18048	1.736794	-1.6954	-0.06321	-0.20204	1.132255	-0.67718	-0.94109	-0.0342	0.452156	-1.05974
P13797	PLS3	-1.90781	1.607751	1.432036	0.362081	1.136561	0.73492	0.84443	-0.22253	0.6166	-0.48402	-1.14583	-1.09418	0.367633	-1.51122	-0.83297	-0.039	-0.86812	0.646787	2.412234
P13798	APEH	0.480083	-0.95365	0.284048	-0.27476	0.689509	0.458018	-0.06945	0.553145	0.486418	0.359203	0.075671	-0.04569	0.936467	-0.15376	-0.2296	-0.18303	-0.59405	-0.50795	-0.19035
P13804	ETFA	0.288468	0.246947	-0.52944	0.966038	1.22943	0.715676	-0.28148	0.038674	-0.01695	0.330924	0.780457	-0.09062	0.022021	-0.09655	0.456755	0.146906	-0.35728	0.359994	-0.46015
P13807	GYS1	-0.178	-0.42462	-0.28154	0.219708	-0.32238	-0.74421	-0.61882	-0.99162	0.007266	-0.15859	-0.80381	0.711171	-0.08286	1.610027	-0.66966	-0.48468	2.218633	-0.06823	-0.3953
P13861	PRKAR2A	0.200157	-0.50767	0.037464	-0.71125	-0.16686	-0.29469	-0.39338	0.041468	0.033884	-0.12536	0.192497	-0.38282	0.144631	-0.19495	0.591597	-0.16803	-0.35964	0.135621	0.266953
P13928	ANXA8	-2.16997	0.532284	2.490177	0.449181	-0.79463	-0.54242	1.763208	1.803008	0.225997	0.347367	-0.30902	1.52647	0.554566	1.013887	-1.24746	-0.16607	0.91731	0.265826	1.016583
P13929	ENO3	0.671742	-0.07857	-1.16771	-1.68334	0.155131	0.342994	-0.50872	-0.3432	0.040847	1.377018	-0.20773	-0.35802	0.052825	0.105808	-0.34986	1.288007	0.048669	-0.50783	-0.18026
P13984	GTF2F2	0.080121	-0.06947	-0.23399	-0.3751	-0.01181	0.490561	-0.75691	0.457839	-0.07902	0.48907	0.427685	-0.32419	0.536584	-0.59452	0.717871	-0.12176	-0.16723	0.523854	0.117733
P13995	MTHFD2	-0.20261	0.01964	-0.55752	0.056985	0.44519	-0.04105	-0.78895	0.701232	0.11776	-0.64832	-1.31977	1.011374	0.535383	0.550176	-0.95319	1.362046	0.450383	-0.68115	-0.59265
P14136	GFAP	3.01913	-1.31302	-1.20408	-1.68324	-1.84629	-1.54146	-0.70573	-0.75867	-0.95271	-1.62725	-1.82029	-1.3376	-0.4143	-1.31193	-1.41476	-1.98773	-1.82291	-3.18621	-2.05987
P14174	MIF	0.958619	-1.88692	-0.16733	0.857856	0.219886	-0.30565	-0.54383	0.358018	1.169859	0.885171	0.677616	0.033724	0.547943	0.145711	-0.01398	0.490661	0.425316	0.123668	-0.01501
P14314	PRKCSH	0.161314	-1.13949	0.683074	-0.24751	0.229553	-0.13439	-0.89612	1.572732	-0.06662	0.909636	-0.07502	-0.24569	-0.09908	0.001504	-0.06435	0.34097	-0.05892	0.414232	-0.24406
P14317	HCLS1	-2.15001	1.275191	1.297003	-0.8464	0.042416	0.453216	-0.5086	-1.84189	-0.2718	1.603269	-1.28634	0.245636	0.626625	0.91219	-0.06861	-0.19369	0.055669	1.156862	-0.85671
P14324	FDPS	0.060683	-0.69039	-1.15812	-0.16604	-0.78582	-0.61186	-0.3107	0.192257	-0.71316	-0.17136	0.240203	-0.95633	-0.21469	-0.68755	0.425257	0.411655	-0.61133	-1.02301	-0.51684
P14373	TRIM27	0.639203	-0.18809	0.544108	-0.04372	-0.09379	0.09225	0.382693	-0.52359	0.022513	-0.57558	0.384377	0.468152	1.260586	0.370581	-0.38776	0.161718	0.804779	0.124417	-0.3419
P14384	CPM	-1.08326	1.105903	-0.44811	1.299952	0.086667	-0.15783	0.040914	-0.95282	-1.18346	0.519795	-0.86251	-0.11473	-0.06442	0.015626	-0.22198	-0.84041	0.233903	1.3749	-0.77446
P14406	COX7A2	-0.02214	-0.2191	0.525384	-0.06171	1.000054	1.116435	-0.43021	-0.61064	0.034203	-0.63487	0.790797	0.170699	-0.02771	0.249827	1.778482	1.177636	-0.35429	-0.13559	-0.57656
P14543	NID1	-1.05362	-0.59356	-0.57192	-1.89199	-0.63366	-1.22675	0.078175	-1.77923	-0.36044	0.216513	-0.30867	-1.29688	-0.89971	-0.66917	-0.1583	-0.50511	-1.21963	-0.48864	-1.77101
P14550	AKR1A1	-1.13187	-0.69067	-1.36781	-1.04458	1.807371	-0.43747	0.366717	0.528915	0.856175	1.678979	-0.49796	0.294107	0.430468	0.30042	-0.89516	-0.32628	0.404893	0.164201	-0.3438
P14618	PKM	0.249367	-0.58238	0.264539	0.720035	0.261785	0.644039	-1.28931	0.747707	-0.06327	0.027086	0.18096	0.154824	0.824089	0.468068	-0.45677	0.27006	0.888	0.360233	0.656142
P14625	HSP90B1	0.006052	0.213789	0.047742	0.155785	0.115931	-0.0012	0.156171	0.12176	-0.18584	-0.5528	-0.25916	0.851334	0.585139	0.39906	-0.0921	-0.42334	0.354926	0.231633	0.260812
P14649	MYL6B	0.142202	-0.03	-0.83095	-1.35022	0.008735	-0.37582	0.013976	-0.38161	-0.28706	-1.17335	0.651057	-1.19349	-0.28336	-0.72678	-1.70618	-1.50418	-1.06628	-0.61131	0.047877
P14735	IDE	-0.05119	-0.09101	-0.10863	-0.12827	0.568144	0.269541	0.096869	0.51664	0.678308	0.533894	0.000452	0.059296	0.839324	-0.06165	-0.001	-0.10263	0.401455	-0.0215	-0.10273
P14780	MMP9	-2.55688	-0.81014	2.00137	-1.17955	-0.6134	-1.40494	-0.47841	-2.29532	0.369475	2.769858	-2.21743	-0.33166	-1.35363	3.01562	-1.085	-1.86886	1.108398	-2.38069	-1.21125
P14854	COX6B1	0.028147	-0.78995	-0.06132	-0.13373	1.37605	0.622506	-0.25749	-0.12784	-0.1222	0.555841	0.850438	0.38169	-0.12211	0.916933	2.051287	1.601011	0.086216	-0.67867	-0.76856
P14859	POU2F1	0.024525	0.030645	-0.06419	-0.51226	0.018133	0.211106	0.188721	0.68188	0.307826	-0.12202	0.068611	-0.08974	-0.03954	-0.03185	0.104814	-0.56728	0.090026	-0.34302	-0.02754
P14866	HNRNPL	0.545459	-0.36271	0.372049	0.298385	0.266083	0.732595	-0.38633	0.907106	-0.13257	-0.09268	0.398219	-0.21645	-0.07016	-0.25739	0.138965	0.02408	0.110694	0.330171	-0.13787
P14868	DARS	0.632474	-0.35635	-0.16491	0.026645	-0.10091	-0.12442	-0.2648	0.48133	0.201274	0.223872	0.201282	0.227314	0.355973	0.118431	0.352891	1.036918	0.080304	-0.55111	-0.01153
P14902	IDO1	-1.57469	0.254048	-0.57837	-1.75735	-1.42362	-0.75023	-0.25307	-1.74417	0.294501	-0.35307	-0.48806	-0.54025	-0.56596	0.793996	-0.61171	-1.13627	0.670369	0.087576	-2.15074
P14923	JUP	0.10135	0.656148	-1.05663	0.841294	0.254578	-0.01671	-0.0552	0.108313	-0.29903	-0.56018	0.387317	-0.40636	0.013741	-0.99009	0.569139	0.482091	-0.37165	-0.78982	-0.79363
P14927	UQCRB	0.170004	-0.57364	0.022893	0.806628	0.86822	0.899195	-0.31178	0.134695	-0.35728	-0.94327	0.481888	-0.01316	-0.45536	-0.0042	1.67845	-1.0778	-0.65788	-0.3803	-1.49881
P15056	BRAF	0.06024	-0.12504	0.44992	0.761588	-0.30231	-0.2588	0.229312	0.316784	-0.24219	-0.02515	0.071242	0.309511	0.09466	0.615083	1.536747	0.069879	0.717667	-0.1105	-0.2206
P15088	CPA3	-2.90863	-1.52289	0.156237	-3.62513	-1.72903	-2.26591	3.577165	-3.6822	-0.52543	-2.3583	-2.74095	-1.58544	-1.89049	-0.96539	0.338909	-2.32587	-0.96945	1.090053	-2.00803
P15090	FABP4	-1.22162	-0.85827	-1.24528	-2.07256	-3.32944	-4.40384	1.200526	-3.55452	-0.43281	-1.68423	-4.53019	-2.51406	-1.48182	-1.97429	-3.03562	-1.50549	-1.00436	1.00125	-2.56785
P15104	GLUL	-0.12066	0.294447	0.680566	-0.71687	0.534338	0.151776	-0.25643	-0.60602	0.605523	0.369109	0.770176	0.860932	-0.15101	1.452197	0.512674	0.354264	-0.6799	-0.42361	-1.09955
P15121	AKR1B1	-0.42497	-0.60833	-0.76891	-1.45705	2.381796	-0.77003	1.225115	0.919182	1.296421	1.801359	-1.22273	-0.15158	-0.56524	-0.05333	-0.90899	-1.0096	2.256144	0.35686	-0.55785
P15144	ANPEP	-1.1619	0.733932	0.25361	-0.80834	-0.57096	-1.18211	0.136105	-0.65856	0.214516	2.273058	-1.49236	0.307254	0.08528	1.228026	-0.90952	0.171822	-0.10548	0.258553	-1.3037
P15169	CPN1	-1.00333	0.519536	0.472839	-0.7638	0.004307	-0.98682	0.038388	-0.58437	0.116843	0.609059	-0.27487	0.323243	-0.20019	0.982526	-0.3165	-0.55901	0.630734	-0.01015	-1.76508
P15170	GSPT1	0.137762	-1.08846	0.182357	-0.24717	0.520629	0.77927	-1.11147	0.030697	0.240777	0.283885	0.07251	0.325813	0.730669	0.276545	-0.02163	0.43917	0.868161	-0.03942	0.191038
P15259	PGAM2	-2.2584	-1.04235	-0.37367	-0.49733	-0.37809	-3.3763	1.227556	0.593566	0.358002	0.365749	-0.68881	-0.56482	-0.32826	-0.08972	3.206874	-0.23423	-0.15003	0.183686	0.769205
P15260	IFNGR1	-0.12843	0.670878	0.332698	-0.15796	-0.14379	-0.34109	0.55794	-0.78053	0.179406	1.423295	-0.54886	0.298981	-0.12893	0.433105	0.126722	1.929623	-0.18079	-0.53401	-0.51644
P15289	ARSA	0.313932	-0.29294	0.245344	-0.52578	2.388039	-0.21497	-0.23179	-0.57737	0.324279	0.939712	0.143001	0.825805	0.362217	0.34812	-0.48849	0.058525	0.225126	0.502589	-0.21291
P15291	B4GALT1	-0.12906	0.041147	0.348514	-0.22856	-0.06219	-0.28011	-0.34802	-0.67713	-0.06276	0.130803	-0.04141	-0.20884	-0.01349	-0.06546	-0.84815	0.126516	0.979061	1.268592	0.195595
P15309	ACPP	1.026665	-0.3823	-0.22726	-0.26468	3.364991	1.593754	-0.2494	0.099674	0.418367	0.395491	-1.57024	-0.00842	0.285405	0.472049	-1.99408	3.482636	0.010404	-1.3772	0.071486
P15311	EZR	-1.60685	-0.3012	0.362908	1.20565	-0.01269	0.432933	-0.42821	-0.44496	-0.34252	-0.39288	0.693005	-0.34	-0.0709	-0.37348	1.516879	0.287214	0.633979	0.887059	-0.13918
P15313	ATP6V1B1	-0.44871	-0.57619	-1.3682	-0.1342	-0.69311	-0.13011	-0.38557	-1.44282	1.094897	-0.10274	0.487344	0.067702	0.578843	-0.33595	2.181496	0.098588	-0.57316	-0.33105	-1.19433
P15328	FOLR1	1.683423	-0.91229	-0.96765	2.250754	2.745052	2.230331	-0.63848	-1.17235	-0.76902	-5.13409	0.803782	-2.43413	-0.15467	-0.57028	4.24609	0.588553	-1.24871	-3.30773	-2.97462
P15336	ATF2	0.536355	-0.2192	-0.55105	-0.38256	-1.50097	-0.58401	-0.4336	0.961052	0.07278	-0.31431	1.288236	-1.66007	-0.16022	-0.54551	0.023025	0.016202	-0.43948	0.623357	-0.72424
P15374	UCHL3	-0.89354	0.112416	-0.18862	-0.42231	0.072927	0.785199	0.368442	0.687782	0.488318	0.219427	0.139612	-0.40833	-0.10885	0.00245	0.575057	-0.55824	-0.12851	0.0365	0.094649
P15408	FOSL2	-0.74793	0.214064	0.547634	0.738817	0.499666	0.66009	0.646447	0.272695	0.340262	-1.10984	0.148763	0.699668	1.289446	0.208156	-1.1828	0.64078	0.113614	0.128758	1.167318
P15498	VAV1	-2.0674	1.692886	1.467887	-1.05802	0.401615	0.139944	0.441516	-1.56714	-0.1371	1.244171	-0.76023	1.433691	0.004229	1.920104	-0.43333	-0.70231	0.308965	-0.03522	-1.2619
P15502	ELN	-1.83093	-1.68352	-0.11439	-2.35581	-2.40331	-3.39919	0.66665	-4.41526	0.086129	-2.98305	-2.26226	-2.1087	-2.0103	-1.17384	5.405258	-2.37142	-2.17916	-0.53008	-3.34737
P15529	CD46	1.106348	-0.25115	-0.57514	-0.86144	-0.81392	-0.57381	-0.16692	-0.29784	-0.12846	-0.63859	0.582368	-0.63508	-0.15254	-0.37344	1.29364	0.051169	-0.36897	-0.68181	-1.33174
P15531	NME1	-0.08389	-1.85145	-0.1926	1.072902	0.740457	0.509473	-0.61674	1.090022	0.598435	0.796272	0.750207	-0.48293	1.220996	-0.53066	0.142984	0.727377	-0.82133	-0.30405	0.923245
P15559	NQO1	-2.31425	-0.65832	-0.33665	3.439292	-3.11544	-1.44816	-0.48251	0.819265	-0.4887	-0.07782	-0.65236	-0.78415	-0.14	0.329596	-0.2392	-1.28358	-1.18365	-0.2922	-1.50299
P15586	GNS	0.282475	0.501629	-0.13378	-0.77592	1.810625	0.001419	1.826404	-0.04294	0.735449	0.789304	-0.854	1.551494	0.194068	0.910077	-1.10016	-1.34112	0.716567	0.703851	-0.87702
P15735	PHKG2	0.068491	0.233666	-0.11547	-0.06829	-0.21663	0.161243	-0.09784	-0.63237	-0.03014	-0.53812	-0.05926	-0.15465	-0.95215	0.666158	-0.6675	-0.24198	0.053996	-0.60671	-0.07045
P15848	ARSB	-0.49906	0.155147	0.18237	-0.2865	0.699156	-0.14886	-0.217	-0.17117	0.172921	0.318375	-0.12203	0.891139	0.103606	0.625789	-0.40229	-0.18374	0.219271	0.918154	-0.37409
P15880	RPS2	0.386019	-0.39489	-0.13147	0.296026	0.081823	0.113492	-0.43108	0.212703	-0.04184	-0.45511	0.53315	0.498853	-0.23201	0.085978	-0.17037	0.150873	0.251337	-0.13756	0.115037
P15882	CHN1	-0.85886	0.345937	-0.13391	-0.74226	-0.47821	-1.18619	0.509059	-0.44695	0.326956	-0.48897	-0.29712	-0.37832	-0.34837	-0.62037	-0.68043	-0.94259	-1.01833	-0.06973	-0.12266
P15907	ST6GAL1	-0.40253	-0.24534	-0.18326	-0.95484	-0.02863	-0.57548	-0.02302	-0.09189	-0.1385	-0.21824	-0.02006	-1.06538	-0.39202	-0.62393	2.020153	0.097261	-0.57343	-0.12278	-1.02508
P15924	DSP	0.438318	0.475911	-0.98663	1.051293	0.49673	0.296873	0.254112	0.447668	-0.45078	-1.09206	0.56366	-0.30109	0.464838	-1.09918	-0.00755	0.139073	-0.3877	-1.27329	-0.93252
P15927	RPA2	0.984609	-0.71851	0.123711	-0.79349	0.675036	0.676017	0.079928	0.79166	0.499094	0.239347	0.781099	-0.67619	-0.29555	-0.34522	0.222661	-0.151	-0.09164	0.046023	0.134362
P15941	MUC1	-0.02157	-1.47189	0.60289	-0.18179	-1.6457	-1.01023	-0.66722	-0.34459	0.070053	-1.38067	0.038674	-0.47212	0.624796	-0.48731	1.752646	-0.08627	-0.2328	-2.62282	-1.27309
P15954	COX7C	-0.03393	-0.76375	-0.34854	-0.21433	0.614824	0.825418	0.181273	-0.63812	-0.83097	-1.04818	0.283841	-0.09117	0.098084	0.305841	1.93663	0.819392	-0.29922	-0.58325	-0.65412
P16035	TIMP2	-1.37122	0.518738	0.317076	-1.3318	-0.31579	-0.93238	0.255225	-0.66804	0.043677	-0.18925	-1.21403	0.290482	0.053911	0.504048	-1.06786	-0.34222	0.111031	-1.12113	-0.15088
P16050	ALOX15	-0.88497	1.587795	2.397173	-0.8232	-0.17343	-0.20843	1.462212	-1.40669	0.04825	0.782155	-1.63636	1.512853	0.645486	0.567883	-1.02921	-0.45972	0.559573	-1.16515	-0.84287
P16066	NPR1	0.077071	-1.13802	-0.6496	0.7418	-0.87829	-0.9205	-0.38219	-0.7488	-0.448	-1.67762	-0.32315	-0.79167	-0.10067	-0.52559	1.891077	-0.62039	-1.43386	-2.32667	-1.49489
P16070	CD44	-1.57288	1.245925	1.368431	0.463513	-0.70989	-0.59328	0.718659	-1.0126	-0.25671	0.895219	-1.49739	0.851857	0.824349	0.916992	-0.4897	-0.27999	0.235865	0.281857	-0.54921
P16083	NQO2	-1.26454	-0.54628	-1.12653	-0.39443	2.268225	0.239132	0.146787	-0.23301	0.877798	0.620917	-0.77553	-0.30855	0.077369	0.224669	-0.26204	0.136304	0.059864	0.229385	-0.67077
P16104	H2AFX	0.832826	-0.05202	0.360868	0.175712	0.696529	0.486315	0.221024	0.171887	-0.12022	-0.37061	0.01045	-0.34516	-0.63125	0.226726	0.199999	-0.58971	-0.23659	0.627129	-0.56049
P16112	ACAN	-0.67013	-2.77448	-1.7051	-0.8814	-4.35039	-0.91842	-0.85543	0.808187	-0.84849	-3.98354	0.184981	-1.36329	-3.15286	-0.92435	0.801733	-2.86106	-0.79443	-2.38424	-2.53497
P16144	ITGB4	0.433645	-0.83839	-0.0276	-0.22357	-1.49871	-1.58389	0.558667	0.635696	-0.41035	0.677591	1.525297	-1.01386	-0.80939	-0.96853	-0.0494	-0.76043	-1.13194	-1.42	-1.90465
P16150	SPN	-1.92543	1.285973	0.711244	-1.41877	0.172637	0.067604	0.207275	-1.43961	-0.31626	0.911807	-2.05616	-1.03101	-0.11998	1.445411	-1.16246	-0.86479	0.410366	0.918557	-2.11148
P16152	CBR1	-0.12533	-0.42648	0.556279	-1.00834	2.004207	-0.85116	0.250313	-0.65678	0.978234	1.156823	-0.57483	1.085554	0.38252	1.094996	-0.45528	0.186337	1.494926	-0.17645	-0.5297
P16157	ANK1	-1.62302	0.882859	0.11169	-0.7813	-0.35062	-1.60226	1.156159	-1.63015	0.961591	-0.67382	-1.55505	-1.5198	-0.84959	-0.9266	-1.53505	-1.45821	-0.75351	1.630511	-0.96014
P16219	ACADS	-0.58114	-0.6274	0.483112	0.325479	1.450417	0.211776	-0.46653	0.160623	0.339234	0.233741	1.078997	0.857697	0.142865	0.788606	-0.82394	-0.44528	-0.01914	0.425232	-0.25686
P16220	CREB1	0.591867	-0.26634	0.014244	-0.5786	0.424015	0.008474	-0.38627	0.121837	0.122186	-0.39664	-0.17719	-0.42074	-0.57248	-0.3679	-0.38304	-0.33964	-0.46015	0.438587	-0.18731
P16234	PDGFRA	-1.78608	0.525073	1.547465	-0.75675	-0.65897	-1.04804	1.300808	-0.88715	0.360181	0.742493	-0.78256	-0.64353	-0.60581	-0.66229	-0.95732	-0.48829	-0.18549	0.04939	0.570258
P16278	GLB1	0.749105	-0.58917	0.044144	0.046977	0.535792	0.317436	-0.02785	1.600806	0.041848	1.207149	0.048525	0.431799	0.973775	0.027077	0.148129	0.026595	-0.05259	1.279229	1.095481
P16284	PECAM1	-1.18787	1.54469	1.078256	-1.25689	0.359373	-0.66966	1.257903	-1.45005	0.281495	0.512933	-1.47755	0.336528	-0.22434	0.575241	-0.27861	-0.08943	-0.37479	0.853743	-0.88723
P16298	PPP3CB	-0.0822	0.240093	0.9265	-0.72081	0.405649	-0.32779	0.870767	-0.34955	0.257758	0.092512	0.019482	-0.00354	0.208456	0.081835	-0.20816	-0.77152	-0.41381	0.362604	0.090672
P16333	NCK1	0.454266	-0.0552	0.705455	-0.44616	0.594996	0.392304	-0.14796	-0.24876	0.387319	0.962199	0.007024	0.50608	0.314979	0.486559	0.368073	0.02526	0.435612	0.3282	-0.12944
P16383	GCFC2	0.262745	0.573391	-0.62947	-0.19223	-0.08557	0.092613	0.260867	0.260433	0.612465	-0.22323	0.418885	-0.30176	1.06364	0.223486	0.542977	-0.17355	-0.37576	-0.70459	-0.56999
P16401	HIST1H1B	0.783979	0.411895	0.710863	0.186105	0.050999	-1.35655	-0.69532	-0.34053	-1.0735	-1.87242	-0.72189	-0.17424	-0.00823	0.347487	-0.00217	-0.20925	1.396237	1.308193	0.834571
P16402	HIST1H1D	0.860002	0.388646	0.189979	0.129819	0.62577	0.361945	0.421063	0.073115	-0.23053	-2.07655	-1.3205	-0.85631	-0.4136	0.688075	0.430237	0.600854	0.538156	0.522363	-0.35849
P16403	HIST1H1C	-0.17385	0.57271	-0.98292	-0.21764	0.715918	0.035111	-1.4837	-0.6547	0.378987	-2.00862	0.068361	-0.7635	-0.37683	0.118663	0.140965	0.445862	0.140158	0.688548	-0.51869
P16422	EPCAM	1.477137	-1.1436	-1.30524	-0.29869	-0.71266	0.510757	-0.32741	0.728289	-1.06464	-0.82686	1.373381	-1.68327	-0.72608	-1.1104	0.800845	-0.40673	-0.82644	-1.4634	-0.9927
P16435	POR	-0.31531	0.558169	0.16346	-0.32948	-0.09743	0.112029	0.096521	0.346722	-0.04717	0.613473	-0.03625	0.42766	-0.21188	0.306005	0.358466	-0.53331	0.613743	0.075849	-0.41024
P16452	EPB42	-1.58811	0.694995	0.261663	-0.90608	-0.26076	-1.66757	1.327857	-1.90894	0.895691	-0.86639	-1.37259	-1.26664	-1.16849	-1.2431	-1.70952	-1.2721	-1.69697	1.918855	-0.85996
P16455	MGMT	0.163106	-0.34263	-0.51696	0.395838	-0.16111	0.700755	-0.68228	0.04049	0.234207	0.057912	0.210268	-0.75076	-0.23122	-1.18742	0.319778	0.522232	-1.01833	-0.49905	-1.084
P16520	GNB3	-0.48774	0.392312	-0.03604	-0.1109	-0.03949	-0.25271	0.266694	-0.0125	0.313602	-0.02695	-0.17751	-0.08233	-0.5859	0.364898	0.61211	0.124853	-0.06579	-0.03696	0.542483
P16591	FER	-1.72945	-0.26244	0.010003	-0.2627	-0.27856	-0.66639	0.113986	0.134991	0.421136	0.449359	-0.48579	-0.51425	0.019616	-0.63223	0.39857	0.610648	0.089826	-1.26432	0.670037
P16615	ATP2A2	-0.36919	0.422638	0.144896	0.465641	0.0529	0.112949	0.333012	-0.28942	-0.07865	-0.14017	0.078289	0.560781	-0.1409	0.390021	-0.22863	0.424378	0.343613	-0.01823	0.298015
P16671	CD36	-2.92023	0.764908	-1.13885	-2.9016	0.039127	-1.45348	0.979856	-2.693	-1.64027	0.36943	-2.19485	-1.11172	-2.39177	-0.9869	-1.79848	-1.39047	-1.90339	0.088634	-2.26296
P16885	PLCG2	-2.01362	0.552596	0.379746	-0.74122	0.190405	-0.23295	0.630672	-1.02475	-0.30891	0.927259	-1.04114	0.161942	-0.33827	0.990202	0.476109	-0.40954	-0.31124	0.206829	-1.20682
P16930	FAH	0.570642	-1.05539	-0.76327	-0.08764	0.981059	-0.74278	0.125236	-0.04809	0.369452	0.815553	0.3839	-0.35617	0.608353	-0.52187	-0.02686	-1.21528	-0.48363	0.548656	1.235515
P16949	STMN1	1.210203	-2.13584	-0.05721	0.146419	1.272212	0.963704	-0.50307	0.522979	-0.94835	-0.7739	-1.5041	-1.16521	-0.78931	-0.358	-0.86833	-0.82933	-1.21248	-1.10955	0.068517
P16989	YBX3	0.26812	-0.01012	-0.06209	0.066945	-0.63667	-0.32515	-0.30006	-0.0503	-0.2736	-0.74086	0.261528	-1.5133	0.300388	-0.77056	-0.23174	0.67837	-0.86833	-0.37223	-0.02133
P17028	ZNF24	-0.17582	0.107163	0.05762	0.248897	0.234765	0.282762	-0.33981	-0.38714	-0.08027	-0.40251	0.740396	-0.17277	-0.12584	-0.14806	-0.86002	-0.16829	-0.63073	-0.26752	-0.21122
P17029	ZKSCAN1	0.13278	-0.11608	-0.1379	-0.15842	-0.8053	-0.01352	-0.03548	-0.46337	-0.18759	-0.15986	0.525883	-0.12322	-0.07868	-0.74118	0.101548	-0.18409	-0.85934	-0.46213	0.135503
P17066	HSPA6	0.010392	0.293978	0.091332	-0.08301	0.329406	0.145533	0.408037	0.040073	0.042314	0.128111	-0.1703	0.52072	0.002305	0.214009	0.18724	0.039441	0.52981	0.263256	-0.04211
P17096	HMGA1	0.118786	-0.50587	0.125994	0.391601	-3.02122	-0.47629	-0.55586	-0.11026	-0.26919	-1.40767	0.231898	-0.83582	0.191626	-0.91269	1.291746	0.326953	0.282437	0.644361	-0.56882
P17098	ZNF8	0.220141	0.094579	-0.29755	0.204686	-0.339	-0.05848	-0.23341	-0.45605	0.026876	0.533262	0.473899	-0.11876	-0.11113	-0.56632	-0.12108	0.204124	-0.65725	-0.30999	-0.64453
P17152	TMEM11	-0.145	0.466279	0.158377	-1.15091	-0.14357	-0.28138	0.564301	-0.11076	-0.43551	0.081068	0.31946	-0.10053	-1.06976	-0.05028	0.416293	0.41586	-0.44264	-0.55945	0.056521
P17174	GOT1	0.27321	-0.82604	-0.18893	-0.37697	1.543851	0.481755	0.62237	0.697826	0.53669	0.208945	-0.14922	-0.2949	0.102482	-0.49813	0.829755	0.292461	-0.45152	-0.14437	-0.53097
P17213	BPI	-2.0146	-0.17944	0.620556	-1.47216	-0.42768	-2.5034	-1.12819	-1.50203	0.118801	0.999506	-1.42237	1.82332	-0.92713	3.546991	-0.49378	-2.18123	1.289851	-0.53916	-1.28904
P17252	PRKCA	-1.4964	-0.67935	-1.17345	0.819978	-0.39199	-0.68041	-0.53801	-1.33941	-0.033	-0.49566	-0.57147	-0.30805	-1.43769	0.019658	-0.47342	0.625851	-0.64191	-0.49756	-0.39328
P17275	JUNB	-1.08548	0.538216	0.189901	-0.70326	-1.09451	-0.64121	0.521509	0.314154	0.464903	-0.16562	-0.69468	-0.75553	0.496108	-0.50373	-0.27741	-0.14868	-0.29463	-0.44371	1.184097
P17301	ITGA2	0.362889	-0.33406	-0.28448	-0.93429	-1.33243	-1.31328	-0.43744	0.944847	0.144302	0.199264	0.077197	-0.53206	0.065082	-1.07123	-0.15146	-0.31576	0.851044	0.26442	0.02956
P17302	GJA1	0.171439	0.259465	-0.00501	-0.82409	-0.43715	-0.23901	-0.32722	-0.75997	0.499126	-0.96238	-0.47696	-1.34768	0.46147	-0.49172	-1.14416	0.043432	-1.54437	0.40208	1.683748
P17480	UBTF	0.396731	-0.37766	-0.28018	0.864435	-0.27152	0.308969	-0.83468	0.054546	-0.36299	-0.24441	0.403062	-0.82805	-0.38221	-0.71561	0.036885	-0.12177	-0.25391	0.47832	0.025254

P17535	JUND	0.244847	-0.08998	0.210595	0.426172	-0.72834	0.158464	-0.07364	-0.09049	-0.01905	-0.71198	-0.05849	-0.13725	-0.12233	0.143258	-0.30048	-0.46537	0.284651	-0.21686	-0.01705
P17540	CKMT2	-0.17221	0.439849	-0.86445	-0.43103	-1.54584	-0.81912	-0.74933	-1.36706	0.194524	-0.662	-0.29223	-1.12179	-0.69104	-0.90565	2.066457	-0.14085	-0.15666	-0.40904	-0.45676
P17568	NDUFB7	0.02324	-0.04708	-0.48807	-0.46131	0.301611	-0.08082	0.49732	-0.45056	-1.61165	-1.18561	0.271987	0.366943	-0.20897	-0.07066	0.751925	0.030622	-0.31266	-0.01812	-0.69123
P17612	PRKACA	-0.60746	-0.15198	0.112995	-0.31094	-0.20723	-0.44349	-0.12179	0.67174	-0.32352	-0.1234	-0.03215	-0.13605	-0.73495	0.029055	0.822088	-0.05036	-0.45739	-0.46826	-0.52324
P17655	CAPN2	-1.43629	-0.6978	-0.16119	-0.58082	-0.96284	-0.73592	-0.52363	-0.02939	-0.48157	-0.52185	0.497937	-0.44852	0.159548	-0.67244	-0.21805	-0.08293	-0.52924	-0.54714	0.422148
P17661	DES	-3.94691	0.202363	-0.06643	-4.04143	-0.94288	-1.8104	-0.59658	-2.4922	0.105482	-3.35503	-4.16942	-1.00512	-1.05289	-0.87046	-0.75955	-3.34503	-1.60705	-1.76872	-0.2918
P17676	CEBPB	0.784906	0.40129	0.47748	-0.11224	0.073011	0.818473	0.360976	-0.40922	-0.09224	-0.32376	-0.18282	1.089541	0.189094	0.56574	-0.13854	-0.16338	1.324748	1.469821	-0.5105
P17706	PTPN2	0.411078	-0.39907	0.626895	0.916891	0.939594	0.921518	-0.42403	0.703685	0.592446	0.998175	-0.11824	0.139895	0.172511	0.393308	0.963468	0.037764	1.049093	0.395501	0.111566
P17812	CTPS1	0.839289	-0.98554	0.442859	1.084404	0.511363	1.095782	-1.25283	0.410693	0.09547	0.227887	-0.73453	0.216655	0.354236	0.092834	-0.05876	-0.59263	0.570046	-0.69589	-0.63785
P17813	ENG	-1.1111	0.686527	0.324326	-1.14403	-0.45865	-0.7476	0.150659	-1.02926	-0.18245	0.818371	0.398054	-0.86372	-0.66675	-0.24282	-0.51402	-0.58471	-0.62179	-0.04421	-0.36097
P17844	DDX5	0.608839	0.174477	0.382103	0.627827	-0.12092	0.134843	-0.4022	0.681228	-0.18441	-0.05621	-0.01362	-0.67566	-0.11782	-0.39884	-0.30233	-0.15209	0.134559	0.479886	0.208354
P17858	PFKL	0.659958	-0.37434	-0.22045	-0.11573	0.146365	0.545646	-0.89322	0.419579	0.160528	-0.07988	-0.03023	0.742293	0.166562	0.659934	0.035436	0.320406	0.962538	-0.58921	-0.23153
P17900	GM2A	-0.78913	1.374591	1.033843	-0.55873	-0.0434	-0.67281	0.298411	-0.64431	-0.24411	0.220925	-1.94147	-0.18143	-0.02352	0.190911	-1.11942	-0.3716	0.08662	-0.10091	-1.56964
P17931	LGALS3	-0.32911	0.25047	-0.63588	0.657668	-0.21295	-0.66566	0.645387	-0.42639	-0.37825	-0.21118	-0.14779	-0.53013	-0.61611	-0.73103	0.972906	0.064192	0.514931	1.227464	-0.62909
P17936	IGFBP3	-2.05901	-0.26753	-1.2628	-1.85717	-1.32714	-1.89893	0.485835	-1.27402	0.645743	-2.6577	-1.32751	2.178231	0.203792	-0.14568	-1.21653	-1.32759	-0.15382	-1.40005	-1.74244
P17948	FLT1	-0.22337	0.155046	0.185549	-0.20232	-0.34679	-0.07682	-0.27209	0.170575	0.040847	0.256011	-0.3821	-0.30564	-0.20497	0.515747	-0.62067	-0.09498	1.070813	0.244013	0.192213
P17980	PSMC3	-0.02917	0.208778	0.261226	0.258152	-0.07568	0.144565	0.073426	0.224727	0.123834	0.084306	-0.01974	0.275728	-0.01974	0.194409	-0.05388	0.275642	0.386792	-0.07921	0.200005
P17987	TCP1	-0.31398	0.064632	0.13495	0.010861	-0.12531	0.25617	-0.47237	-0.73454	-0.16756	-0.23835	0.337796	-0.05446	0.183257	-0.03692	-0.05696	-0.12894	-0.12801	-0.35558	-0.14299
P18031	PTPN1	-1.08019	0.017488	0.947776	0.316454	0.824113	0.487841	0.538967	-0.20528	-0.09721	0.74697	-0.80373	0.509556	0.090142	0.705051	0.153988	0.311888	1.010065	1.001409	0.100474
P18074	ERCC2	0.131985	0.270211	0.428795	0.131332	0.219943	0.608322	0.116209	0.296142	-0.3037	0.171393	0.181787	0.081465	-0.16809	-0.13641	0.207765	0.276172	0.340639	0.066812	0.254413
P18077	RPL35A	0.254316	-0.46869	-0.54866	0.54876	-0.12503	0.083702	-0.21627	0.104163	-0.45691	-0.44559	0.330338	0.477103	-0.37689	0.243372	0.431471	-0.22303	0.357672	-0.27163	0.082867
P18084	ITGB5	-0.81146	-0.04122	0.013057	-0.44767	-0.0044	-0.16431	0.407712	0.411634	0.517981	-0.44639	-0.92537	-0.24088	0.17928	-0.06365	-0.57413	0.085705	-0.46575	0.398918	0.760123
P18085	ARF4	0.05506	-0.59894	0.018337	-0.99488	1.483579	-0.07383	0.104945	-0.0631	0.446805	0.677413	-0.24038	1.306932	0.653672	0.606808	-0.05673	0.314834	0.998982	1.063012	0.741712
P18124	RPL7	0.388565	-0.27472	-0.02108	0.505285	0.039784	0.120783	0.014337	-0.0147	-0.20745	-0.80329	0.378345	-0.03269	-0.39237	-0.07833	-0.04448	-0.21685	0.176677	-0.16931	-0.20706
P18206	VCL	-0.30425	-0.41568	-0.7832	-0.42823	-0.46835	-0.51362	-0.78085	-0.47498	0.096349	-0.55495	-0.30421	-0.95452	0.200159	-0.694	-0.49307	-0.31353	-1.09893	-0.37445	0.171401
P18428	LBP	-1.31559	0.097909	-0.97138	-1.97392	-0.11471	-0.34073	0.163471	-0.58572	0.8133	0.099086	1.31578	0.592289	-0.01741	1.05531	-0.21623	0.065225	0.167031	0.756423	0.514494
P18433	PTPRA	-0.34923	-0.53747	0.208325	-0.10456	-0.07672	-0.29555	-0.20828	0.35507	0.32768	0.631395	-0.36663	-0.50428	-0.49457	-0.5429	-0.10886	-0.61387	-0.04882	-0.07018	-0.06171
P18440	NAT1	-0.51366	0.276347	-0.0812	-0.35035	0.118889	-0.36251	0.197069	-0.01071	-0.13017	-0.7883	0.718433	0.52613	-0.01964	0.899267	0.047941	-0.63046	0.448218	-0.3086	0.025915
P18583	SON	0.51183	-0.14261	-0.43333	-0.33876	-0.30248	-0.01114	0.076966	0.388048	-0.43191	0.138851	0.133723	-0.65148	-0.10939	-0.45056	0.441545	0.05877	0.038784	0.469698	-0.12396
P18615	NELFE	0.898378	-0.1327	0.066546	0.235643	-0.29033	0.280099	0.082869	0.309754	-0.84538	-0.36137	-0.06396	-0.94382	-0.29167	-0.40315	0.389218	0.102079	-0.10751	0.080689	0.043847
P18621	RPL17	0.342782	0.01491	0.282617	0.322596	-0.04595	-0.00771	-0.00047	-0.14571	-0.40265	-0.6592	0.393963	-0.31208	-0.23071	-0.42292	-0.1175	-0.06335	0.129669	0.052215	-0.15434
P18669	PGAM1	0.639934	-1.06199	-0.06235	0.007993	1.29751	-0.45345	-0.45839	0.703606	0.953017	1.16578	-0.79342	0.531484	1.811725	0.769567	-0.18934	-0.22277	1.255197	0.16664	1.297755
P18754	RCC1	0.355931	-0.60057	0.56805	-0.27926	0.418629	0.447804	0.102536	0.760136	-0.70854	-0.05354	0.22155	-0.56019	-0.32652	-0.56981	0.054305	0.12804	0.666078	1.023416	-0.50832
P18827	SDC1	-0.62292	-0.07773	0.020375	-0.90675	-0.74155	-1.19635	0.041871	-0.12955	1.105286	-1.42048	-1.33957	-1.96608	-0.44877	-0.76762	-1.33663	0.365844	-1.1169	0.106893	-0.58132
P18846	ATF1	0.94573	-0.35808	0.01046	0.190361	0.289611	0.551352	-0.07203	0.581544	-0.50166	-0.07591	-0.32432	-0.36114	-0.30983	-0.42163	-0.31734	-0.74577	0.002314	0.811005	-0.0811
P18858	LIG1	1.444443	-0.35841	-0.44852	0.44732	0.641808	1.515735	0.626016	0.873759	-0.38926	-0.41007	-0.33872	-0.50143	-0.5351	-0.76516	-0.14053	-0.96032	0.046017	-0.54937	-0.49671
P18859	ATP5J	0.285758	-1.34683	-0.02081	0.766219	1.298482	0.376879	-1.03307	-0.05434	-0.98758	-0.31485	0.691653	0.179375	0.297264	0.046683	2.139822	1.625786	-0.72654	0.070284	-1.09599
P18887	XRCC1	0.219852	-0.60033	-0.42056	0.524617	-0.25903	0.386459	0.234962	0.449803	-0.09905	-0.72161	0.170241	0.015238	0.07731	-0.35665	0.702809	-0.31787	-0.20319	0.115806	-0.49827
P19012	KRT15	-1.05907	-0.51017	-0.5791	3.580001	-2.03539	-3.83948	-0.384	-3.39879	0.394094	-2.85013	-0.02001	-0.6518	-0.77605	-1.9423	-3.92042	2.08462	-2.93048	-1.04268	-3.93212
P19013	KRT4	-2.76313	0.258535	2.71989	-1.66779	-2.28955	-1.01262	-0.75656	-0.46932	0.471719	-2.55943	-1.0827	-1.00876	-1.8113	-0.83599	-1.0827	-0.65453	-1.82707	-4.39659	-0.53623
P19021	PAM	-1.53157	0.814493	-0.58726	-0.87789	-1.11729	-1.42115	0.525068	0.066196	0.61351	-0.32127	-0.55625	0.019436	-0.31665	-0.35142	-0.02896	-1.23946	-0.73993	-1.61755	0.457538
P19022	CDH2	-0.83207	-0.5995	-2.15008	-2.15421	-2.16791	-1.39975	0.534471	0.934253	-0.81616	-1.20994	0.662559	-1.03012	0.212677	-0.84812	-2.19799	-1.58549	-0.71678	-2.41102	0.395307
P19075	TSPAN8	-3.21502	-1.49164	-1.64196	-4.93133	-3.78211	-1.90933	-0.79604	-0.61368	-0.36761	-3.17975	2.824773	-3.34491	-1.70797	-2.06049	1.606244	-2.71725	-1.59502	-2.82143	-2.72144
P19086	GNAZ	-0.15489	0.274026	-0.07282	-0.38443	-0.74952	-0.98603	-0.07163	0.286751	0.233506	-0.09456	0.109789	-0.31267	0.001556	0.440047	-0.21268	-0.33451	-1.08507	-0.25977	-0.38571
P19105	MYL12A	-0.484	-0.5589	-0.08082	-0.10724	0.072027	0.498854	0.01444	-0.36072	0.196475	0.127899	-0.47814	0.430121	0.073157	0.537276	-0.52579	0.594742	-0.30981	-0.19382	-0.25327
P19174	PLCG1	-0.11782	-0.01383	-0.03656	-0.84646	-0.31551	-0.16353	-0.20246	0.23548	-0.51829	0.15334	-0.77043	-0.67151	0.226559	-0.50436	-0.4394	-0.70018	-0.53847	0.434433	1.025205
P19320	VCAM1	-1.46085	0.075061	0.784757	-1.09195	-0.30817	-0.7554	0.215662	-1.1193	-0.4277	-0.23999	-1.39487	-1.25507	0.269668	-0.28975	-1.16159	0.211646	-1.06939	0.082271	1.463117
P19338	NCL	1.18894	-0.87189	-0.48547	-0.21146	0.304732	0.547574	-1.24279	0.546958	-0.80841	-0.04788	0.537851	-0.62183	-0.03917	-0.88708	0.072025	-0.16533	-0.18917	-0.09106	-0.2873
P19367	HK1	-0.26818	0.73162	0.145788	-0.02573	-0.18551	-0.39212	0.004336	0.103442	-0.32896	0.332712	0.157242	0.57969	-0.18995	0.408899	0.120045	0.86576	0.642794	0.439025	-0.01763
P19387	POLR2C	0.438986	0.078174	-0.50505	0.100859	0.103004	0.418945	0.485998	0.24942	-0.81045	0.103327	0.252443	-0.36743	-0.48678	-0.50185	0.189835	-0.42643	-0.18923	-0.02767	0.091015
P19388	POLR2E	0.316756	-0.14685	-0.3227	0.037711	0.312777	0.272377	-0.83259	0.568943	0.013047	0.397079	0.296963	-0.08131	-0.24605	-0.02018	0.150837	-0.18549	-0.17555	0.115231	0.179776
P19397	CD53	-1.95841	1.101702	0.851443	-1.01736	0.734865	-0.58986	0.206377	-1.13197	-0.02895	1.26918	-1.65923	1.04303	-0.23954	1.353296	-0.39769	-0.44648	-0.45235	0.01282	-1.94248
P19404	NDUFV2	0.3111	-0.16627	-0.11008	-0.16467	0.812142	0.184237	-0.09403	-0.31649	0.176426	-0.44554	0.374776	0.358856	0.493007	0.002417	0.512325	-0.31529	0.003285	-0.05007	-0.56916
P19440	GGT1	1.620754	-0.03093	0.293007	-0.43025	-0.42801	-0.423	0.693302	-1.72324	-0.35913	-0.46416	-0.53989	-0.35274	-0.05981	-0.33575	-0.27867	-0.7194	0.440984	2.778302	-1.44553
P19447	ERCC3	0.194945	0.499113	0.119981	-0.18017	0.257117	0.320348	-0.17621	0.458592	-0.25243	0.30514	0.289252	-0.03821	1.084433	0.120758	-0.06801	0.111439	0.395366	0.451646	-0.20797
P19474	TRIM21	-0.85742	0.609317	1.739481	-0.22892	0.978282	1.308106	0.373655	-0.56446	-0.73321	0.095502	-0.60064	0.422731	0.046815	0.754129	-0.6433	-0.27135	-0.12191	-0.34402	-0.61718
P19484	TFEB	-0.14885	0.324111	0.316689	-0.34816	-0.17692	-0.18642	0.08263	-0.33749	0.18039	0.608739	-0.06945	0.3278	-0.13699	0.351381	-0.32789	-0.47849	0.271213	-0.14276	-0.40624
P19525	EIF2AK2	1.249535	0.022477	0.700382	-0.16076	-0.11916	0.600762	-0.81963	1.204992	-1.41668	0.103255	-0.4652	0.537734	0.016095	0.515706	-0.6295	0.070653	-0.1819	-0.34956	-0.72389
P19544	WT1	1.380204	-0.77605	-0.28342	-0.29339	1.384421	1.52292	-0.14448	0.863022	-0.18972	-1.59876	0.871755	-0.58471	-0.25726	-1.58299	-1.11383	0.346021	-0.0146	-0.75753	-0.34886
P19623	SRM	-0.30369	-0.59773	-0.58899	-1.37426	-0.2749	-0.3376	-0.35644	0.230607	0.355749	0.905519	-0.08212	-0.21538	-0.05546	-0.30527	-0.88504	0.147623	0.384521	-0.06189	0.569969
P19634	SLC9A1	-0.02749	0.118667	0.207843	0.063382	-0.29092	-0.2632	0.092413	0.723855	0.270186	0.33172	-0.37752	-0.08336	-0.09304	-0.50147	-0.77379	-0.28799	-0.42187	-0.51353	0.066333
P19652	ORM2	-0.54515	-0.27239	-1.32638	-1.86165	0.345825	-1.16019	0.623871	-0.62045	1.129671	-0.44682	1.773137	0.989108	0.315241	0.353969	-0.84691	-1.36914	-0.04872	-0.87	-1.27934
P19784	CSNK2A2	-0.19598	0.267737	-0.25933	0.47474	0.268926	0.34401	-0.29212	-0.04154	0.115535	0.037921	-0.01174	-0.05225	0.077369	0.315362	0.225012	0.348398	0.565321	0.127866	0.092262
P19793	RXRA	0.127475	0.358844	0.154329	-0.76738	0.318729	0.07297	0.387623	-0.18472	0.125488	-0.13221	0.014288	0.145219	0.028216	0.380134	0.171804	-0.13227	0.059787	-0.00432	-0.86933
P19801	AOC1	-1.72079	-0.16373	-0.42489	-1.76823	-1.9476	-1.11974	0.13355	-1.52909	-0.14674	-1.25598	-0.54012	-1.60609	-0.41127	-0.56903	-0.22773	-0.38267	3.168803	4.965597	-0.99768
P19823	ITIH2	-1.08988	0.389075	0.085765	-1.89911	-1.09372	-1.67673	-0.26791	-1.10739	0.397508	0.531098	-1.20303	-0.07642	-0.0773	0.491003	-0.6128	-0.92483	-0.16711	1.261798	-0.28769
P19827	ITIH1	-0.99349	0.263866	-0.63447	-2.01471	-0.6448	-1.3095	-0.69432	-1.20709	0.165453	0.864977	-1.26643	-0.50584	0.318293	0.288013	-0.6368	-1.04817	-0.35158	1.888194	-0.1864
P19838	NFKB1	-0.90404	0.588466	0.187734	-0.72568	-0.07955	-0.18714	0.378796	-0.39721	0.015823	0.758583	0.275661	0.39656	-0.15312	0.529962	-0.81268	-0.63592	0.04811	-0.14912	0.015652
P19878	NCF2	-2.37293	1.090472	1.031937	-1.10003	-0.33649	-0.84983	0.212236	-1.54229	0.050499	1.673478	-1.61201	2.11221	-0.08599	2.667954	-0.96006	-0.87659	1.540642	-1.0891	-1.53995
P19971	TYMP	-1.50192	1.519704	1.477022	-0.48825	1.263834	0.361875	-0.10205	-0.64122	-0.02515	0.999201	-1.45829	0.630426	1.281641	0.262771	-0.73397	-0.30256	-0.18005	0.096401	-0.93682
P20020	ATP2B1	-0.31949	0.444722	-0.34941	0.800935	-0.47723	-0.58836	0.355392	0.051029	-0.16712	-0.70829	0.745817	0.174531	0.321596	-0.19133	0.062005	-0.69527	-0.85299	-0.3286	-0.20404
P20036	HLA-DPA1	-2.28627	-0.93107	0.942983	-1.36576	1.407603	1.073177	-1.1261	-2.95822	-0.56915	-2.17625	0.793685	-0.54364	-1.09426	0.467076	0.900917	-0.15822	-0.19413	0.308108	-1.69571
P20039	HLA-DRB1	-3.40823	-3.37727	-0.4618	-1.37355	3.620497	2.490015	-0.16401	-2.23678	0.523069	0.535924	-2.48453	-2.49215	-3.58762	0.139995	-0.7548	-0.81549	0.553977	2.181835	-2.51586
P20042	EIF2S2	0.567994	-0.28587	-0.14109	0.012574	-0.00087	-0.08376	-0.38135	0.174836	-0.2059	0.138729	0.067317	-0.56915	0.576103	-0.5432	0.067762	0.219534	0.018743	-0.0978	-0.11406
P20073	ANXA7	-0.5437	-0.09291	0.061276	-0.24973	0.11243	0.425459	0.48532	-0.0333	0.059698	0.044248	-0.10947	0.345598	-0.37397	0.209662	0.642545	-0.04417	-0.21581	-0.4651	-0.39039
P20132	SDS	-2.23879	1.82246	1.21147	-1.12534	0.661059	-0.12936	0.741169	-1.26956	0.129918	1.066207	-2.21749	3.03179	0.391793	2.054336	-0.83633	-0.69026	0.959583	0.51092	-0.25092
P20290	BTF3	-0.56505	0.149605	-1.0113	-0.06093	0.247091	-0.34677	-0.95665	-0.1013	-0.5599	-0.61807	0.559822	-0.46376	0.292276	-1.07415	-0.6748	-0.13219	-0.17887	0.128932	-0.00486
P20292	ALOX5AP	-1.63924	1.303455	1.14545	-0.25118	1.764662	1.603382	0.162203	-2.36004	0.064642	1.299867	-0.74088	0.967172	0.010838	2.507619	-0.19364	0.242175	-0.09198	-1.5639	-1.33131
P20336	RAB3A	0.115088	0.179521	-0.00979	-0.25405	-0.53199	-0.97388	0.196248	-0.00094	-0.34378	0.268536	0.576378	0.333334	-0.06083	0.628099	0.476325	0.328647	-0.07383	-0.2298	-0.04385
P20338	RAB4A	0.084695	-0.18695	-0.38053	0.476251	-0.41176	-1.10948	-0.14155	-0.18821	-0.76259	-0.1886	0.234984	0.067335	-0.48844	0.104567	0.741255	-0.19501	-0.2152	-0.40708	-0.63283
P20339	RAB5A	0.813977	-0.23608	0.010849	-0.27915	0.080591	0.017569	-0.70988	-0.60411	0.148092	0.126736	-0.26374	0.348085	-0.38637	0.118485	0.116983	0.358171	-0.22257	-0.27302	-0.17386
P20340	RAB6A	-0.00523	0.133661	-0.13097	0.014824	0.664271	0.645514	0.305832	0.185049	0.140687	0.012801	-0.23798	0.362325	0.134805	0.308754	0.599666	0.616684	0.407158	-0.1143	-0.02345
P20585	MSH3	-0.00542	-0.54866	-0.38492	0.406433	-0.53277	-0.07265	0.221303	0.619205	0.024214	-0.27588	0.723982	-0.30435	-0.59576	-0.18851	0.802558	0.872159	0.068796	-0.00229	-0.15042
P20591	MX1	1.426883	1.646267	3.443593	-0.9996	-0.02011	1.327152	0.38893	2.373811	-0.20384	-0.61545	-1.85639	1.038704	1.054405	0.682745	-1.47577	-1.98816	-0.03098	-0.12671	-1.96061
P20592	MX2	-1.20391	2.025687	3.192219	-1.34775	0.292551	0.406685	0.070425	-0.20758	0.04729	0.281819	-1.96266	1.072375	0.062667	1.38719	-0.80965	-1.1161	-0.13569	1.069746	-1.5443
P20594	NPR2	0.729087	-0.09364	-0.19339	-0.10859	0.315598	-0.79687	-0.27208	0.231902	0.114273	-0.09741	0.188883	-0.5318	0.101747	-0.55604	-0.10427	0.333415	-0.52181	0.060409	0.417912
P20618	PSMB1	-0.00811	-0.14829	0.21812	-0.36748	0.326866	0.395965	-0.25495	-0.05762	-0.11278	0.095758	0.153179	-0.40944	0.032449	-0.47069	-0.20054	0.45162	-0.53894	0.022148	-0.23918
P20645	M6PR	0.283859	0.611992	-0.15383	0.058394	-0.8093	-0.3758	-0.06509	0.097613	0.193465	-0.52032	-0.40727	-0.3737	0.250526	0.25142	0.35126	-0.22967	-0.04122	-0.71052	-0.98567
P20674	COX5A	-0.03993	-0.2581	-0.30823	-0.10067	1.315434	0.738273	0.015475	-0.2807	0.076506	-0.35122	0.366071	0.641241	0.183416	0.252483	0.975122	0.961901	-0.13971	-0.26739	-1.09918
P20700	LMNB1	0.514068	0.54051	0.001287	-0.96169	0.122075	0.214965	0.382847	0.490292	-0.67724	0.028585	0.400898	-0.62299	-0.4494	-0.26884	0.514242	0.129022	-0.3754	0.300784	-0.0733
P20701	ITGAL	-1.70028	1.248779	1.516228	-0.91261	0.483605	0.212158	0.158539	-1.56955	0.030703	1.064807	-1.61966	0.836714	-0.16136	1.164083	-0.37225	-0.56323	0.692974	0.609026	-0.62995
P20702	ITGAX	-1.78304	1.35103	0.097521	-0.95169	0.429286	0.2845	-0.59571	-1.79122	-0.82987	0.986059	-1.76228	0.490823	-0.25008	1.008658	-0.70416	-0.86537	0.207018	-0.39034	-1.6888
P20742	PZP	-1.00089	-0.04389	-0.17608	-1.26883	-0.49449	-1.09311	0.358744	-1.56899	0.515859	-0.0268	-1.49037	0.405326	-0.31442	0.983832	0.04797	-1.16026	0.476087	0.535813	0.243956
P20774	OGN	-3.74778	-2.1123	0.281064	-2.62664	-1.10489	-3.36332	0.821091	-3.24809	1.019388	-3.1552	-1.88546	-1.88198	-3.48758	-0.74116	0.429667	-2.6848	-1.33133	-1.72933	-2.7277
P20810	CAST	-1.16326	-0.41206	-0.79336	-0.85084	-0.84936	-1.69714	-1.0726	-0.7352	-0.5645	-0.56831	-0.212	-0.45218	0.485691	-0.40162	0.494931	0.139967	-0.13638	0.278287	0.242022
P20839	IMPDH1	-1.12057	0.030746	1.466018	-0.22771	0.636627	0.034827	-1.02022	0.305123	0.34791	1.765161	-0.61894	1.04819	0.478015	1.543199	-0.00421	-0.59505	0.819851	-0.03331	-0.23913
P20851	C4BPB	-1.44948	0.555381	-0.52804	-1.38283	-0.77709	-1.28234	-0.58539	-1.82601	-0.02442	-0.22911	-1.19457	2.434024	-1.20898	3.081933	-1.21876	-1.37001	2.027766	0.441222	-0.78443
P20908	COL5A1	-1.96488	0.373973	0.259034	-1.35517	-0.03858	-0.19689	0.490255	-0.262	0.905105	-0.6071	-0.3852	-0.40595	0.327704	-0.54078	-0.61307	-0.3615	-0.97897	-0.33779	0.798037
P20930	FLG	-2.77357	3.627235	-1.01562	-2.78476	-3.63064	-2.21318	1.673614	-1.14045	0.967794	-2.58789	-1.29081	-2.27448	-0.53006	-0.87091	-1.02585	-1.45617	-1.48008	-1.55664	-1.25368
P20936	RASA1	-0.46162	0.239389	0.299864	0.021561	-0.4701	-0.53628	-0.27933	-0.05838	0.012375	0.014685	0.053887	0.00929	0.319351	0.03182	0.480413	0.208146	0.296874	-0.37992	-0.15118
P21127	CDK11B	0.509823	0.140905	0.830668	0.53268	0.579117	1.160371	-0.04359	1.041616	-0.06611	0.554432	0.191273	-0.31491	-0.53516	-0.23456	0.342261	0.301969	0.338541	0.275833	0.068861
P21266	GSTM3	-0.9658	-0.43358	-1.59583	-0.37481	-0.50094	-1.10504	-0.54463	1.122541	-0.53146	-0.21492	-1.9141	-1.09397	-1.34111	-0.39396	-0.08905	-0.69097	2.547195	3.319587	0.131302
P21281	ATP6V1B2	-0.49097	0.503705	0.31965	-0.34197	0.250624	0.046594	-0.19342	-0.5451	0.110779	0.229724	-0.54166	1.001356	0.306567	0.716892	-0.44646	-0.06566	0.34857	0.027564	-0.29794
P21283	ATP6V1C1	-0.64121	0.560778	0.275677	-0.12376	-0.18269	-0.08212	-0.39427	-1.08521	0.252663	0.608962	-0.33927	0.983587	0.570792	0.757827	0.648287	-0.41227	0.229638	-0.57459	-0.97293
P21291	CSRP1	0.165309	-1.44944	-1.85596	-1.05056	-1.11804	-1.55681	-1.42636	-1.99711	-0.94974	-1.13107	-0.42197	-1.10433	-0.97756	-0.73723	-0.04203	-1.10853	-1.65681	-1.64418	-0.09041
P21333	FLNA	-0.71741	-0.06928	-0.57137	0.262502	-0.66748	-0.63626	-0.7134	-0.85725	0.550915	-0.78284	-1.28495	-0.38951	-0.02564	-0.29982	-0.74208	-0.39106	-0.38943	-0.09545	0.589753
P21359	NF1	-1.66861	0.223036	-0.09818	0.376694	-0.21497	-0.24098	0.174244	-0.00237	0.980357	-0.02311	-0.48711	-0.0559	0.054991	-0.11899	-0.34999	0.710196	0.653336	0.536482	0.425076
P21397	MAOA	-1.17213	-0.08922	-0.05387	-1.85332	-1.42004	-2.01543	0.9012	-1.42988	0.274631	-0.59668	-0.30408	-1.10696	-1.10819	-0.87328	-0.50967	-1.10916	-0.92836	0.471066	-0.24911
P21399	ACO1	-0.9503	-0.6749	-0.5182	-0.49507	-0.24238	-0.27827	0.325608	-0.28441	0.442727	-0.10625	-0.52696	-1.0381	-0.03546	-0.69553	-0.51186	0.529865	-0.52235	0.165073	1.117482
P21589	NT5E	-0.03941	-0.6396	-0.03686	-1.47419	-1.36603	-1.14116	-0.16218	-1.32587	-0.01385	-0.70023	-0.17187	-1.39389	-0.27286	-1.18863	0.61176	0.1653	-1.09043	-0.66343	-0.56791
P21675	TAF1	0.063244	-0.34392	-0.08418	-0.20706	0.186046	-0.01445	-0.56692	0.529667	0.100545	0.365946	-0.04551	-0.11647	0.134855	-0.16976	0.416287	-0.36387	-0.33906	0.195525	-0.27533
P21695	GPD1	-3.27352	-1.15205	-1.50853	-1.96603	-2.08337	-3.14398	0.73985	-3.07782	-1.76854	-1.64195	-2.1354	-1.52437	-1.21472	-1.77216	-1.23415	-1.62768	-1.96002	0.031171	-3.01167
P21709	EPHA1	0.128336	-0.18126	-0.23956	0.760699	-0.33184	-0.21409	-0.29492	1.06446	-0.07732	0.227729	-0.20917	-0.4931	0.120529	-0.03681	0.787171	0.358303	1.922726	-1.56499	0.564152
P21730	C5AR1	-2.21274	2.95423	0.864951	-1.14549	0.271939	-1.11788	0.749436	-2.6608	-0.24561	1.271231	-2.13619	1.735289	0.159208	2.197098	-1.32024	-1.26204	0.163822	-0.05358	-2.44583
P21757	MSR1	-1.37539	2.607549	0.979056	-1.00727	0.54819	-0.33994	0.96451	-1.49097	-0.03319	-0.12429	-1.30989	1.620046	0.265268	1.810288	-0.33165	-0.0708	0.369403	0.250553	-1.19688
P21796	VDAC1	-0.49565	0.395828	0.294258	0.556382	0.224782	0.329012	0.336928	-0.19235	-0.25859	-0.17865	0.21983	0.118542	0.124849	0.148281	0.925862	1.642099	0.417175	0.018958	-0.24307
P21810	BGN	-1.06547	-0.2661	0.209031	-1.77081	-0.6371	-0.60816	-0.25222	-1.13119	1.084847	-0.84402	0.141013	-0.77985	-0.31645	-0.32344	0.091221	-0.89944	-0.83364	-0.63125	-0.39643
P21817	RYR1	-0.15873	1.454527	1.079333	0.099513	-0.11074	-0.04438	0.4633	0.205204	2.18125	-0.17348	0.245988	-0.62307	-0.27728	0.775602	0.851593	-0.00651	0.472677	-0.52911	1.39907
P21860	ERBB3	0.670904	-0.24832	0.018042	-0.18662	1.777634	1.525009	0.171877	-0.00054	-0.39897	-0.40364	-0.18313	-0.25066	-0.63283	-0.21525	-0.55618	0.046016	0.152175	-0.86938	-0.59137
P21912	SDHB	0.640889	-0.61973	-0.35025	0.060965	0.089296	-0.34524	-0.37734	-0.74135	-0.91179	-0.43775	-0.19626	0.374933	-0.27132	-0.22121	2.051232	1.396568	-0.28769	-0.35777	-0.72038
P21926	CD9	0.677854	-1.13356	-2.37698	0.087178	-1.08107	-0.81652	-1.46896	0.261288	-1.41589	-0.38143	-0.25417	-0.60011	0.116198	-1.07197	1.044612	1.779367	-1.50047	-1.909	-0.53616
P21941	MATN1	-4.53448	-2.61846	-1.17491	-3.57373	-5.43613	-3.87231	-0.57525	1.144841	-0.58643	-2.80564	-6.19196	-3.29972	-3.49657	-1.27721	-2.6135	-2.43487	-2.8915	-7.23616	-4.65188
P21953	BCKDHB	0.977936	-0.0322	-0.30569	-0.03874	-0.0831	-0.25667	-0.34497	-0.78281	-0.10361	-0.30585	0.383928	-0.13312	-0.15037	-0.11457	0.795705	-0.43523	-0.20965	-0.28032	-0.12882

P21964	COMT	-0.14211	0.774978	0.989448	0.240868	-0.35237	-0.70811	-0.13429	-1.12885	-0.76391	-0.18045	0.255787	0.908391	-0.53522	1.004985	-0.67937	-0.39485	1.497782	0.713502	-0.41858
P21980	TGM2	-1.75526	0.92214	2.169261	-2.34427	-1.15916	-0.98039	0.477309	-0.88354	1.100339	-0.45864	-1.42521	0.551914	0.742796	0.45971	-1.06249	-1.80199	0.576246	-0.73711	-0.91816
P22033	MUT	0.23361	-0.24764	0.296626	0.064608	-0.3865	-0.27995	-0.61692	0.256289	-0.29274	-0.39138	-0.03362	-0.58433	-0.00811	-0.35221	1.22884	0.537181	-0.36208	0.384202	-0.4986
P22059	OSBP	0.112698	-0.34109	0.171383	-0.09735	-0.45623	-0.34061	-0.0953	-0.1056	-0.07972	-0.23595	0.312875	-0.05119	0.658704	-0.43825	0.414025	0.007434	0.347801	0.20479	0.211188
P22061	PCMT1	0.119504	-0.82368	-0.05274	0.156491	1.065213	-0.01627	-0.41314	0.075397	0.723073	0.91869	0.024181	-0.19424	-0.08111	0.175035	1.018878	-0.12925	0.389975	0.079302	-0.16608
P22087	FBL	0.288473	-0.71329	-0.60676	0.045798	-0.15238	-0.24876	-0.8479	-0.15659	-0.97101	-0.74566	0.876111	-0.13443	-0.41611	-0.5776	0.316842	-0.91918	-0.22546	-0.48653	-0.56569
P22102	GART	0.769281	-0.94148	0.300123	-0.33157	0.530453	0.925682	-0.96271	0.269013	-0.15168	0.546358	0.007707	0.755758	0.904552	0.599573	0.082873	0.384066	0.799237	-0.64294	-0.08377
P22105	TNXB	-2.49836	-0.88047	-0.83644	-2.56639	-2.02232	-2.40265	-0.44046	-2.40683	-0.16944	-1.67188	1.07228	-1.5029	-1.01722	-0.96404	-0.25408	-1.29377	-0.56304	-1.31383	-1.37883
P22223	CDH3	1.062804	-0.25418	-0.15246	0.103684	-1.16703	-0.75609	0.363727	0.887958	-0.40485	-1.36628	-0.18512	0.286329	0.112332	-0.52832	0.612946	-0.28252	-0.40185	-0.64173	0.187297
P22234	PAICS	1.365699	-1.4481	0.865611	0.606521	0.362291	0.559509	-0.95354	0.083103	0.058021	-0.03994	0.507466	-0.29335	0.481479	-0.36573	-0.92638	-0.19764	0.794609	0.051294	0.087022
P22307	SCP2	-0.12408	0.753631	0.3152	0.161484	0.870767	0.529061	-0.61345	0.192946	-0.23817	0.581875	-0.35499	0.936604	0.166025	0.36915	-0.7409	0.184065	-0.34408	-0.64301	-1.21043
P22314	UBA1	-0.02374	-0.44054	-0.81347	-0.2585	-0.51812	-0.66968	-0.64728	0.840178	1.270571	1.973876	-0.15344	-0.25948	-0.47879	-0.06356	0.116138	0.133849	0.016104	-0.03287	-0.50939
P22352	GPX3	-2.24604	-1.98986	-1.21991	-2.07241	-1.67716	-1.31343	-1.28774	-2.20326	-0.76159	-1.30625	-0.23605	-0.09048	-1.57275	-0.4115	0.842408	-0.48447	-0.10328	-0.62842	-2.04721
P22392	NME2	-0.07353	-0.91165	-0.02252	1.113798	0.565585	0.413894	-0.66509	1.16596	0.136182	0.305214	0.271365	-0.60654	0.979724	-0.46518	0.781228	1.09382	-1.23959	-0.61766	0.847899
P22413	ENPP1	-0.8399	-0.31054	0.099006	-0.24018	-1.84802	-1.42313	-0.38937	-1.44752	-0.04381	-2.11952	-1.43713	-0.12096	0.142033	-0.33524	3.103281	-0.39049	-0.02453	-0.20439	0.437906
P22415	USF1	0.125897	0.222619	0.809782	-0.50384	0.561664	0.486897	0.053774	-0.31672	-0.03304	0.310454	0.265332	0.377811	-0.22985	0.08487	0.385705	-0.15716	-0.05816	1.383757	-1.10145
P22570	FDXR	0.231556	-0.11438	0.075812	-0.35648	-0.31793	-0.58909	0.294359	0.445493	-0.11565	0.060844	0.933733	-0.77249	-0.53336	-0.74249	-0.32476	-0.21296	-0.8149	-0.42912	-0.67174
P22626	HNRNPA2	1.169961	-0.35905	0.359229	-0.19387	0.43097	0.77977	-0.46113	0.779091	-0.42013	0.141672	0.570699	-0.54353	-0.25044	-0.34463	0.028126	-0.12183	0.114585	0.512051	-0.38581
P22670	RFX1	-0.1021	0.261668	-0.08918	-0.14326	-0.11678	-0.11056	0.678294	1.04841	-0.21652	-0.04875	0.121922	-0.27971	-0.37047	-0.14559	0.863002	-0.04906	-0.01256	0.142198	-0.12497
P22676	CALB2	-1.41835	-0.88175	2.685967	-0.87298	-1.47207	-1.32467	0.070337	-0.04126	1.256494	1.053825	-1.55662	-0.34549	0.646605	-0.73255	-2.2451	-1.97828	-0.01597	0.17615	3.53137
P22681	CBL	0.018826	0.480584	0.620883	-0.53254	0.306971	0.073883	0.056783	0.090326	-0.52723	0.766758	-0.59655	0.083324	0.036132	0.823247	0.173712	0.122161	-0.0576	-0.29624	-0.26175
P22694	PRKACB	0.359283	-0.81853	-0.23016	-0.90413	0.142362	0.404418	0.688083	-0.83581	-0.6191	-0.62489	-0.44975	-0.14135	-1.33224	-0.16379	-0.23514	-0.96018	-0.79747	-0.26095	0.35164
P22695	UQCRC2	0.181241	-0.10531	-0.1856	0.543991	0.496463	0.694094	0.150951	-0.19059	-0.34766	-0.5549	0.511551	0.230503	-0.10825	0.267351	1.756856	-0.83643	-0.38999	-0.3622	-0.96028
P22732	SLC2A5	-2.88586	2.081775	0.884695	-0.99	0.521141	-0.51285	0.519232	-1.41566	0.474225	1.247269	-1.00863	1.989174	2.041948	1.296139	-1.65847	-0.72501	0.621337	-0.14486	-0.52756
P22735	TGM1	0.095599	0.440545	-1.42185	2.089052	-1.38275	-1.30312	0.489018	-0.57405	-0.238	-1.29433	0.572053	1.532919	1.388792	0.320879	-1.41978	-2.18284	-1.76191	-2.81413	-2.5155
P22792	CPN2	-1.00333	0.770943	-0.67385	-2.08823	-0.42789	-0.90785	-0.21955	-0.67798	-0.32516	-0.24322	-0.21657	0.264506	-0.16849	1.262599	-0.62951	-0.91648	0.871876	0.240623	-1.35507
P22830	FECH	-0.48679	0.565735	0.67194	0.518974	0.083317	-0.50969	0.376612	-0.25041	-0.53402	-0.49986	0.211136	-0.12859	-0.58816	-0.056	0.303007	-0.41558	0.035559	0.599108	0.183849
P22891	PROZ	-2.05883	-0.61146	-0.02268	-1.0287	-1.62795	-2.23899	-0.12114	-0.83959	0.661479	-2.16706	-1.28156	1.416741	-0.50945	0.792805	-0.25382	-1.62462	0.001318	-1.43622	-1.84559
P22897	MRC1	-1.11419	0.126059	0.436958	-1.55696	0.493603	-1.27846	1.426178	-1.26401	0.199698	-0.62022	0.349255	0.713655	-0.77745	0.576894	-0.34871	0.40378	-0.973	-0.48403	-1.46226
P23025	XPA	0.066931	-0.11705	0.289926	-0.14078	0.862362	0.649227	-1.11283	0.600559	-0.00548	-0.4649	0.505813	0.141773	-0.12943	0.166069	0.365866	-0.63697	0.595795	0.732337	0.606729
P23141	CES1	-1.27844	-0.26396	-1.01203	-3.17512	-1.24707	-1.89274	0.439764	-2.11713	1.244132	-0.67819	-1.40316	-0.13219	-0.08428	-0.94746	-2.28558	-1.04772	1.402542	1.516666	0.605673
P23142	FBLN1	-2.15962	0.235567	-0.45606	-1.64109	-1.79677	-0.56523	-0.75894	-0.05267	1.245212	-0.76459	-2.31679	-1.44802	0.565436	-1.27155	-1.18667	0.69278	-0.51822	0.07522	1.742276
P23193	TCEA1	0.840202	-1.27983	-0.15184	-0.54665	-0.10435	0.287671	-0.89339	0.230316	-0.73773	-0.27268	0.384186	0.392184	1.534635	-0.7777	0.245717	0.302097	0.263595	0.558795	-0.13558
P23219	PTGS1	0.496098	-0.5873	0.277481	0.900547	-0.07866	-0.07733	0.723068	2.001723	-0.0386	-1.00942	0.166242	-0.03462	-0.45733	-0.36198	1.911112	-0.50618	0.182343	-0.72432	-0.40189
P23229	ITGA6	0.303921	-0.1008	-0.35275	-0.24637	-0.81541	-1.2378	0.461428	-0.44258	-0.01874	1.354977	1.285971	-0.95851	-1.09713	-1.05254	-0.26783	-0.47651	-1.33551	-0.12378	-1.35574
P23246	SFPQ	0.242734	-0.43116	0.196017	0.370637	0.134344	0.313995	-0.38642	0.328935	-0.38986	0.123364	0.292074	-0.45991	-0.32463	-0.26438	0.252084	-0.22322	-0.06539	0.645292	-0.10231
P23258	TUBG1	-0.35639	0.412128	-0.35673	-0.15269	-0.06628	0.955766	0.098473	-0.26118	0.100981	-0.0955	0.650434	0.126294	0.022097	-0.23206	-0.47751	0.477649	0.198881	0.231783	0.041972
P23284	PPIB	0.16281	-0.49883	-0.00183	0.088276	0.970758	0.122647	-0.02744	-1.16195	0.511906	1.058132	-0.09337	-0.17406	0.465561	-0.27158	-1.29835	0.009583	0.779315	1.096073	-0.07879
P23297	S100A1	0.302707	-2.26689	-0.41025	0.873511	2.544293	1.772827	-0.87678	1.441177	0.605026	0.696118	-1.79176	-0.55748	-0.10217	-0.5188	4.449863	-1.58319	-1.4794	-2.88603	-3.00539
P23368	ME2	-1.39343	1.254214	0.874869	0.712579	0.245516	-0.07994	0.126662	-0.1948	0.282352	1.384709	-0.18873	0.68058	-0.18868	0.666636	-0.11953	0.298993	-0.794	-0.42046	-0.88531
P23378	GLDC	-1.42753	-1.31884	-0.70255	-0.45512	-1.84061	-2.20765	-0.63619	0.454191	-0.24497	-0.17982	-1.9354	1.350466	1.960086	0.985379	3.418988	-0.01886	0.222157	-0.42753	-1.74476
P23381	WARS	-0.47045	0.234416	1.16132	-0.69272	0.671179	0.430244	-0.44734	-0.62788	-0.30379	-0.38393	-0.70779	0.132765	1.483451	0.002014	-0.89316	-0.79375	1.412765	1.270936	-0.72593
P23396	RPS3	0.198155	-0.30096	-0.08639	0.011779	0.265204	0.179604	-0.25037	0.183637	0.072842	0.060459	0.447481	0.389502	0.044234	0.216094	-0.04796	0.212261	0.20711	-0.30497	0.259525
P23443	RPS6KB1	-0.26505	0.430789	0.059486	0.570054	-0.3245	0.019486	0.024431	-0.34602	0.238662	-0.16843	-0.58847	-0.06028	0.259684	0.100376	-0.44502	0.839223	0.005434	0.064112	0.583673
P23458	JAK1	0.112749	0.11547	0.680745	-0.42877	0.394074	-0.21303	0.821357	-0.3898	-0.38303	0.826317	-0.36531	0.253713	-0.65073	0.358521	-0.17426	-0.51145	-0.26673	0.015016	-0.36854
P23469	PTPRE	-0.92914	0.509372	0.164637	-0.52543	0.123703	-0.291	0.419471	-1.09449	-0.27438	1.248201	-0.81312	0.656274	-0.7251	1.155529	-0.73162	-0.14576	0.437491	-0.02975	-0.69923
P23470	PTPRG	-0.50874	-0.1583	-0.14519	-0.11185	-0.43423	-0.31072	-0.03643	-0.04035	-0.03207	-0.46973	-0.07249	-0.70564	-0.07238	-0.67611	0.25345	-0.4798	-0.44208	-0.14209	-0.17869
P23497	SP100	1.085652	0.540995	1.021527	-0.99517	0.203768	0.054649	-0.14559	0.821282	-0.27924	-0.22892	-0.68904	-0.19601	0.156213	-0.00499	0.088101	0.454544	-0.40621	0.150072	-0.36182
P23508	MCC	-1.08472	0.511362	0.070085	-0.76138	-0.6199	-0.76636	0.200437	-0.57943	0.121867	-0.10006	0.173601	-0.64179	0.162855	-0.81661	-1.01593	0.012145	-0.38053	1.080033	0.497049
P23511	NFYA	0.380795	-0.21431	-0.2882	-0.1764	-0.48982	0.348823	0.142838	0.115278	-0.17871	-0.31973	-1.02503	-0.80628	0.16199	-0.50596	0.01421	-0.32573	-0.30371	-0.18146	0.046002
P23526	AHCY	0.886592	-1.43647	-0.73735	0.371001	0.807341	-0.08172	-0.65861	1.405784	0.155665	0.503211	0.080212	0.119962	0.498689	-0.40149	0.118522	1.90524	0.008114	-0.45299	-0.05241
P23528	CFL1	0.389007	-0.75217	-0.09015	0.072044	1.241866	0.639186	-0.79466	-0.10012	0.016629	0.726719	-0.68739	0.435015	0.932319	0.760675	-0.57281	0.097509	0.453531	0.063226	0.288895
P23588	EIF4B	0.749155	-0.64945	-0.45221	-0.11782	-0.19814	0.48237	-0.7469	0.55904	0.033123	0.162713	0.310008	-1.13179	0.821105	-1.03854	0.091008	-0.25338	-0.55515	0.058522	0.555828
P23610	F8A1	1.107431	0.185349	-0.01494	-0.5059	-0.18991	-0.39322	0.101136	-0.34301	0.12331	0.444273	-0.18775	-0.06897	-0.07798	0.366147	-0.09075	0.265894	-0.4447	-0.19434	-0.22715
P23634	ATP2B4	-0.10919	-0.1304	-0.59541	-0.01427	-0.34368	-0.39534	0.15329	1.08277	-0.38604	-0.50105	-0.00194	-0.33862	-0.08388	-0.44278	0.78485	-0.62481	-0.84053	-0.75629	0.26093
P23743	DGKA	-0.38925	0.340753	-0.17183	-0.44449	-0.11948	-0.08625	-0.32313	-0.83796	-0.25807	0.23573	-1.22027	0.11514	-0.11263	0.903183	-0.88138	-0.79981	-0.10022	0.376123	-0.30735
P23786	CPT2	0.427873	-0.03257	0.234732	0.050769	0.461092	0.436052	-0.15982	-0.28993	-0.33832	0.104884	0.671629	0.238538	-0.32622	0.434501	0.745167	1.770847	-0.57187	-0.72866	-1.03668
P23919	DTYMK	1.224369	0.421133	-0.62603	-0.46625	0.049686	0.262733	-0.052	0.885847	0.114258	-0.03275	-0.28071	0.182626	-0.65294	0.370705	0.176347	0.401229	0.342597	-0.33436	0.109415
P23921	RRM1	0.888861	-0.41403	0.139247	0.129367	2.220352	3.363794	-0.47241	0.789794	0.245599	-0.28882	0.040466	-0.45046	0.118395	-0.29803	-0.21392	-0.10107	1.040042	-0.30859	0.641589
P23946	CMA1	-2.22897	-1.20636	-0.74018	-2.20159	-2.12179	-3.70081	0.786885	-2.8186	-0.44308	-2.04338	-3.84464	-1.40323	-1.34928	-1.50569	0.517609	-1.84547	-3.33983	1.133379	-3.65497
P24043	LAMA2	-1.48748	-0.99718	-1.14403	-0.55671	-1.17968	-1.92349	0.498459	-2.15959	-0.10375	-0.91315	0.599577	-1.41221	-1.44178	-0.44039	-0.0588	-0.79636	-0.92409	-0.12687	-1.75848
P24158	PRTN3	-1.94979	0.656469	1.098691	-1.64962	-1.07516	-2.12297	-0.60732	-1.20486	0.058556	4.182673	-1.46026	0.066694	-0.25991	4.05294	-2.04238	-1.9408	2.119811	-0.56607	-1.44795
P24310	COX7A1	-1.60281	-0.43129	-0.99885	-0.64502	-0.486	-0.91518	-0.6115	-0.43219	-0.00247	-0.86228	1.823239	-1.27032	-0.52459	-0.76352	0.189	-0.52224	-0.9464	-0.35985	-0.15156
P24347	MMP11	-1.22686	-1.10033	0.650231	-0.39623	-0.96105	-1.98483	-0.54144	-0.26451	0.349055	-1.99462	-1.39177	-1.10892	-0.69902	-0.95382	-0.70873	-0.12939	-1.02071	0.028009	-0.64249
P24386	CHM	0.41258	0.04287	0.120215	-0.10126	0.047362	0.121832	-0.30697	-0.19944	0.063021	0.460028	0.288873	-0.24299	-0.66734	0.344404	-0.43879	0.192426	0.080608	0.096838	-0.55965
P24468	NR2F2	0.90041	-0.46275	-0.41833	-0.30089	-0.2373	-0.13906	0.30695	-0.25214	0.269107	-0.42084	0.189962	-2.31766	-2.24762	-0.99819	-0.92297	-0.71608	0.73455	1.343082	-0.72658
P24534	EEF1B2	0.309835	0.309186	-0.24395	-0.00987	-0.07149	0.019921	-0.52873	0.15081	-0.32849	-0.12355	0.233853	-0.54574	0.769163	-0.65243	0.236617	0.58513	-0.38553	-0.16039	0.120096
P24539	ATP5F1	-0.19798	0.150672	0.033354	0.472424	-0.01573	0.128743	0.300388	-0.39829	0.004877	-0.18253	0.382876	-0.01256	-0.56249	-0.01801	1.394427	1.073799	-0.41841	-0.58692	-0.30462
P24557	TBXAS1	-1.99687	1.717438	1.198473	-0.5577	0.937535	0.229467	0.883992	-1.56325	-0.09846	0.709664	-0.84881	1.791833	-0.06315	1.466802	-0.18359	-0.67402	0.310707	-0.00799	-1.69166
P24666	ACP1	0.92303	-1.1922	-0.63517	-0.29679	0.505898	-0.31328	-1.01217	0.427259	0.137265	1.048054	0.043774	0.212747	-0.01941	0.218976	0.374494	0.389103	0.531121	-0.63019	0.152394
P24752	ACAT1	0.098425	-0.32431	0.13998	0.167996	0.638957	-0.09191	-0.53221	-0.20002	-0.18043	0.548582	-0.01875	0.044461	0.345598	-0.43839	1.578942	0.672241	-0.91602	0.278444	-1.00346
P24821	TNC	-1.7409	-0.2394	-0.34721	-0.7819	-1.14652	0.219515	-0.77762	-0.57345	1.162809	-0.9075	-0.852	-0.72402	1.517343	-0.7548	0.687761	-0.37668	-1.68811	-2.12256	1.757341
P24844	MYL9	-1.25538	-0.69225	-0.64924	-1.78622	-0.57242	-0.49892	-0.32549	-1.24805	0.332579	-1.18702	-1.09614	-0.39128	-0.23148	-0.27373	-0.69013	-0.253	-1.02693	-0.64872	0.374069
P24928	POLR2A	0.426346	0.249393	0.368765	-0.03509	0.153078	0.350601	0.241759	0.403043	-0.02518	-0.11983	0.352525	-0.44364	-0.08353	-0.32505	0.143279	-0.29441	-0.2374	0.147743	-0.20613
P24941	CDK2	0.551157	-0.31833	0.291485	0.330559	0.937947	1.372432	0.16997	0.50181	-0.1409	-0.32096	-1.16835	-0.10178	0.331449	-0.26766	-0.24041	-0.45603	0.6811	-0.30346	0.558161
P25054	APC	-0.74228	-0.16661	-0.04234	-0.60122	-0.665	-0.50361	-0.20656	-0.28279	0.065372	-0.05014	0.561984	-0.90432	-0.09627	-1.1975	0.785335	-0.66963	-0.30914	0.55649	0.727584
P25098	GRK2	-0.51281	0.361243	0.608565	-0.3953	0.431811	0.465757	-0.31766	-0.32308	-0.58377	0.853191	-0.60347	0.53268	0.089745	0.837881	-0.27947	0.144427	0.234251	0.217178	-0.76503
P25205	MCM3	2.186999	-0.71964	-0.31199	-0.17196	1.533124	1.907373	-0.18009	1.032437	-0.45531	-0.63811	0.065796	-0.54641	-0.48649	-0.71785	-0.25173	0.558954	-0.30872	-0.53127	-0.63378
P25208	NFYB	0.697949	-0.41864	-0.01672	-0.74291	0.40562	0.72794	-0.36446	0.685713	-0.54868	-0.44792	0.127219	-0.36225	-0.27085	-0.84117	-0.21144	-0.40811	-0.46326	0.162334	-0.40112
P25311	AZGP1	-1.51419	1.056806	-0.16525	-1.5888	-0.54464	-1.64241	2.192292	-0.66304	1.54407	-0.82964	-0.65134	0.124871	0.541788	0.30556	-0.67728	-0.92553	0.18659	0.080193	-0.6114
P25325	MPST	-0.06966	-1.5246	0.059192	0.17891	0.326508	-0.82648	0.068523	-0.71249	1.080839	1.13087	-0.4182	-0.13264	0.29984	-0.23314	-1.25081	0.789517	0.101744	1.054438	0.258921
P25391	LAMA1	-0.09376	-0.07468	0.348578	-0.39574	-0.95693	-1.68457	0.177193	0.400971	0.241206	-1.08319	-0.0108	-0.65881	-0.3099	-0.05168	-1.26113	-0.91846	1.094557	2.858085	-0.7143
P25398	RPS12	0.062623	0.243042	-0.10758	-0.03449	0.220856	0.186733	-0.03683	-0.0582	-0.00378	0.26619	0.695327	-0.07203	0.15801	0.035911	-0.1163	-0.21228	0.241904	0.016115	0.007955
P25440	BRD2	1.018249	-0.12914	0.108801	0.305131	0.65279	0.744399	-0.05397	-0.1062	-0.08815	0.089141	0.341025	-0.25108	-0.29292	-0.37385	0.133637	0.395418	-0.14128	0.33921	-0.46914
P25445	FAS	-0.66863	0.185479	0.083689	-0.63923	-0.70348	-0.69565	0.166234	-1.01678	-1.53538	-0.15296	-0.2098	-1.02231	-0.21221	-0.42404	0.305528	-0.51544	-0.84217	0.223868	0.631294
P25490	YY1	1.030302	-0.35878	-0.12922	0.39951	-0.48491	0.166466	0.392614	0.420112	-0.32746	-0.03019	0.281303	-0.24382	0.099592	0.145716	0.155225	0.006916	0.458794	0.350573	-0.10886
P25685	DNAJB1	-0.43109	-0.74103	1.156	0.2598	-0.1717	0.122965	0.338447	2.192128	-0.57443	-0.15431	-0.46404	-0.20183	-0.42442	-0.0969	1.492482	0.822151	0.252208	-0.39913	-0.59476
P25686	DNAJB2	-0.32858	0.021416	-0.26227	-0.44656	0.349605	0.209808	-0.29402	0.435773	-0.6782	0.09126	-0.1267	-0.0741	-0.53905	-0.17332	-0.39207	-0.55619	0.594976	1.029213	-0.20543
P25705	ATP5A1	-0.15678	0.292369	-0.0304	0.400768	0.030538	-0.05759	0.285672	-0.54342	-0.34474	0.314288	0.393429	-0.0025	-0.1779	-0.05661	1.049716	0.887049	-0.5271	-0.36969	-0.50159
P25774	CTSS	-1.31022	0.949938	0.074909	-1.68269	0.36079	-0.14839	0.290826	-0.22617	-0.04116	0.617565	-1.24945	0.182267	-0.198	0.258864	-0.58313	-1.38065	-0.66455	-0.39656	-1.88531
P25786	PSMA1	0.017477	-0.49623	0.444898	0.076503	0.430877	0.742912	-0.13529	0.366952	0.329489	0.184171	0.233906	-0.83651	0.206459	-0.47613	-0.04185	0.34709	-0.78677	0.023937	-0.27728
P25787	PSMA2	0.1716	0.062241	0.674893	0.204095	0.211649	0.600085	-1.13313	0.416763	-0.10837	0.284617	0.135146	-0.69825	0.206705	-0.34662	-0.04722	0.373011	-0.45196	0.108922	0.482031
P25788	PSMA3	0.203267	-0.73382	0.472547	0.101996	0.554134	0.636999	-0.54773	0.444723	-0.34187	0.251177	0.25132	-0.69073	0.041147	-0.39135	-0.00047	0.466707	-0.49115	-0.18027	0.053996
P25789	PSMA4	0.164208	-0.70411	0.158488	-0.43399	0.740289	0.579316	-0.7581	0.322544	-0.03115	-0.08023	0.351129	-0.73227	0.217013	-0.65948	-0.20182	0.651135	-0.30031	-0.18345	0.006437
P25815	S100P	-2.60915	-0.38818	1.896003	-2.67294	-2.52285	-3.90811	-0.14891	-3.30781	0.298337	2.017232	-3.38591	0.876408	-1.40231	4.125987	-1.46472	-3.05281	2.283909	-3.38808	-2.05802
P25940	COL5A3	-1.28714	1.164738	0.899783	-2.15714	-1.31429	-1.66404	1.221311	-1.34312	0.089577	-0.51836	-1.36698	-2.45554	-0.62114	-1.71668	-0.08199	-0.32695	-2.42593	-0.55705	-2.08622
P26006	ITGA3	0.352046	-0.65235	-0.63429	0.847775	-0.87259	-0.49994	0.011933	0.922332	-0.11984	0.448397	0.528093	-1.06854	-0.35946	-0.268	-0.0557	-1.27756	-0.69127	-1.57808	-0.56486
P26012	ITGB8	1.808323	-0.25386	-0.44324	-0.05115	-0.22276	-0.29777	-0.13106	0.822098	-0.44883	0.449283	0.049442	0.332958	1.011491	-0.60749	1.246158	0.844424	-0.12751	-0.51126	-0.41797
P26022	PTX3	-1.22441	-0.20921	-1.00132	-1.12243	-1.27548	-1.85953	-0.59935	-1.53794	-0.06095	1.500093	-0.65306	-0.15027	-0.30711	0.549531	-0.49604	-0.46276	0.0389	1.085575	-1.23775
P26038	MSN	-0.52614	0.181117	0.589614	0.323285	0.657952	0.733068	-0.3069	-0.03112	0.33949	1.107881	-0.31863	-0.20459	0.937481	0.115409	-0.71438	-0.02268	-0.56906	0.351999	0.647845
P26045	PTPN3	0.172859	0.016846	-0.19068	-0.2285	0.048088	-0.33486	0.099054	0.665044	-0.33109	-0.63985	0.105142	-0.21707	0.10339	-0.48654	0.161799	-0.42487	-0.57982	-0.36657	-0.66032
P26196	DDX6	0.072373	-0.07324	-0.10863	0.235833	0.066963	0.220611	-0.04674	0.358607	-0.25302	-0.42536	0.012829	0.041678	0.194084	-0.04074	0.293029	-0.04407	0.046776	0.214644	-0.11115
P26232	CTNNA2	0.383709	0.209275	-1.51395	0.276701	-0.43016	-0.0616	0.293739	-1.00557	0.236483	-1.09466	-0.46769	-0.90022	0.08742	-0.37107	-1.17522	-1.09238	-0.15983	-0.28309	0.793259
P26358	DNMT1	0.379946	-0.02218	1.222727	-0.34103	0.338929	0.841148	-0.43771	0.998207	-0.68677	-0.26617	-0.17008	-0.69429	-0.17904	-0.58204	-0.78397	0.48922	-0.45942	-0.14651	-0.55053
P26368	U2AF2	0.86051	-0.57543	-0.39397	0.235599	0.252878	0.275803	-1.17518	0.737719	-0.00597	0.307423	0.469862	0.001485	-0.32597	-0.16293	0.270354	-0.22869	0.21175	0.494493	-0.07425
P26373	RPL13	0.348213	-0.05049	0.064267	0.53192	-0.23581	0.08265	-0.03829	-0.01391	-0.38725	-0.53578	0.326977	-0.71892	-0.10755	-0.83088	0.091611	-0.02089	-0.16916	-0.12407	0.003815
P26374	CHML	0.365226	-0.00601	-0.1412	-0.21726	0.482142	0.491933	-0.3857	-0.00404	0.133939	-0.29287	0.191346	-0.71718	0.051469	-0.31306	-0.50802	-0.73579	-0.03018	-0.82479	0.162847
P26440	IVD	1.3129	-0.69631	-0.01245	1.322947	-0.07709	0.018315	-1.11579	-0.4868	-0.3319	-0.62911	1.477527	0.164495	0.369217	-0.01046	0.365407	0.764355	0.488587	0.560322	-0.76646
P26447	S100A4	-3.17532	-0.42932	0.038447	-1.73024	-1.49359	-1.79131	-0.48753	-2.74569	-0.56841	-0.50949	-3.12897	0.286	-1.29476	0.107131	4.275187	-1.45749	0.398107	-2.03496	-3.23137
P26572	MGAT1	-0.97701	0.73912	0.816852	0.542284	0.587247	0.966368	1.730259	-0.67446	0.428147	0.081255	-0.66132	0.500633	0.079952	0.557907	0.361838	0.040944	0.599808	0.543349	0.263613
P26583	HMGB2	-0.17116	0.373068	0.389669	0.627959	-0.25604	1.397269	0.294883	0.535693	-0.39933	-0.30211	-0.20934	-0.30409	0.042971	1.292058	-0.8559	0.795361	-0.2852	0.161728	-0.54191
P26599	PTBP1	0.371198	0.091548	0.448794	-0.06535	0.419457	0.441406	-0.32403	0.124845	-0.65309	0.163691	0.75382	-0.36228	0.154715	-0.30851	-0.04675	-0.0392	-0.05975	0.178145	-0.55158
P26639	TARS	0.418982	-0.96947	0.359528	0.764185	0.800844	1.197101	-0.46138	0.618111	0.674575	0.483113	-0.16669	-0.47748	0.505731	-0.58056	-0.08089	0.926776	-0.01143	0.073537	0.680351
P26640	VARS	-0.05124	0.192153	-0.08465	-0.26283	-0.31077	-0.03766	-0.15115	-0.5561	-0.20861	-0.08633	0.319915	-0.09944	-0.02295	-0.17602	0.823876	-0.15436	0.217026	0.175983	-0.07917
P26641	EEF1G	0.407082	0.139645	0.233601	0.428131	-0.22941	-0.08788	-0.1056	0.16167	-0.28688	-0.03733	0.221754	-0.09751	0.805445	-0.44899	0.360151	0.374306	0.177235	0.171208	0.179627
P26885	FKBP2	-0.07724	-1.14646	-0.19971	0.256443	0.63135	-0.16694	0.153846	-0.30123	0.734167	0.47201	0.032673	0.856722	-0.39539	0.387193	0.169929	0.49947	0.462365	0.593518	-0.90672
P26927	MST1	-0.94414	-0.43891	-0.20413	0.015162	-0.70889	-0.92043	0.099396	-0.84432	0.048257	-0.8347	0.495784	0.528048	-0.18315	1.05996	-0.09335	-0.22534	0.120911	-0.25227	0.081873
P27105	STOM	-1.75233	0.653647	1.129662	-0.98669	-0.13585	-1.19415	1.112947	-1.20968	0.135385	0.264355	-0.65319	0.646054	-0.47547	1.721363	-0.55721	-1.17888	0.001694	0.673843	-0.7015
P27144	AK4	0.699301	0.37429	-0.90668	1.029358	-0.64794	-0.97355	-0.21216	0.11336	0.437451	0.75868	0.178537	0.495378	1.015867	0.044915	-1.78808	2.070859	0.670205	0.270336	-0.14475
P27169	PON1	-1.39761	0.321126	-1.40311	-1.25072	-0.88013	-1.44722	0.422066	-0.30359	-0.11826	-0.90936	-0.60605	0.46368	-1.04866	-0.04691	-0.809	-1.3084	0.141276	0.132691	-1.22515
P27338	MAOB	-2.27932	0.2297	0.477033	-1.63654	-0.84904	-1.62053	0.601507	-1.54295	0.274631	-0.22608	1.644049	-0.77754	-0.90728	-0.59803	-1.00972	-1.30987	-0.63733	-0.46954	-0.26384
P27348	YWHAQ	0.555338	-1.04512	-0.175	-0.4311	-0.42679	-0.08134	-1.1368	0.810762	-0.41897	-0.13075	-0.38667	-0.68719	0.344024	-0.72922	-0.38804	0.988103	-0.27359	-0.28164	0.617463
P27361	MAPK3	-0.60566	0.263345	0.20468	-0.33289	0.260753	0.437343	-0.19934	-0.48574	-0.38093	-0.03949	-0.30325	0.110304	-0.05414	0.156518	-0.10147	-0.32084	0.006909	-0.42457	-0.42736

P27448	MARK3	0.55173	0.052188	0.33036	-0.17625	0.066156	-0.20659	-0.05231	0.462596	-0.14069	0.084642	-0.40987	0.435978	0.034662	0.115218	-0.12473	-0.03879	0.105176	0.132167	0.217777
P27449	ATP6V0C	0.290566	0.460518	0.680712	-0.37476	0.313166	-1.12414	0.283924	-1.13053	-0.24629	0.419365	-1.70638	1.798196	-0.11934	1.171567	-1.00007	0.195311	0.740491	-0.53766	-0.58681
P27487	DPP4	-2.27696	0.016344	0.070501	-2.74654	-0.95145	-1.35419	0.160069	-2.62247	-0.75794	-1.9518	1.561747	-0.68272	-0.88706	-1.11953	-2.53686	-1.91001	0.720246	-0.64966	-0.26822
P27540	ARNT	0.051164	-0.28095	-0.10542	-0.34169	-0.50337	-0.32018	-0.54204	0.301143	-0.1107	0.362832	0.297594	-1.28863	-0.02261	0.192027	0.910295	-0.10678	-0.28389	-0.18809	0.249847
P27635	RPL10	0.249056	-0.26136	0.001671	0.304182	0.055654	0.162642	-0.21738	-0.10813	0.347991	-0.80714	0.270364	0.448042	-0.29696	-0.01758	-0.17719	-0.21467	0.424493	0.019264	-0.01924
P27658	COL8A1	-3.81826	-3.34127	-1.36415	-3.15135	-4.23821	-3.22202	-1.05205	-2.12765	0.558727	-2.20929	-2.8487	-3.44176	-1.58496	-0.79738	1.232692	-1.10353	-2.03376	-1.67553	0.095158
P27694	RPA1	1.575237	-0.58776	-0.08631	-0.34066	0.674171	0.736873	-0.79822	1.054529	0.020055	0.281328	0.341232	-0.22878	-0.45028	-0.05624	0.335779	-0.22103	-0.11846	-0.44824	-0.02744
P27695	APEX1	0.651919	-0.75171	-0.50764	0.226217	0.700393	-0.07694	-0.30558	-0.01404	0.612583	1.00302	0.555457	-0.19896	0.599318	-0.32416	-0.17254	0.064559	-0.21707	-0.24274	-0.45878
P27701	CD82	0.337609	0.449874	0.229447	0.574896	-0.70321	-0.77891	-0.79491	-0.66469	-0.44853	0.063041	0.019644	-0.36354	0.221425	0.221217	1.038735	0.655215	-0.5146	-0.99673	-1.34915
P27707	DCK	0.491242	0.521989	1.647362	-0.33854	0.822752	0.439924	-0.47034	-0.0156	-0.01117	0.305372	-0.82347	0.113356	0.214808	0.81985	-0.13161	-0.22306	0.728768	0.471177	-0.25271
P27708	CAD	0.807174	-0.25866	-0.74501	0.223893	-0.34328	0.184527	0.357538	0.573002	-0.39684	-0.04179	-0.36768	-0.09796	-0.15964	-0.16342	-0.39262	0.851319	-0.59633	-1.2002	0.190362
P27797	CALR	-0.58129	-1.19886	0.391822	-0.05532	0.604328	0.265405	-0.77335	1.670508	0.296991	1.497207	-0.08984	0.54528	0.492838	0.750077	1.079172	0.525355	0.953387	0.95984	0.302138
P27816	MAP4	0.577989	-0.3705	-0.17983	-0.93161	-0.83577	-0.58017	-0.52467	0.087789	-0.11736	-0.26078	-0.4155	-1.25093	1.014708	-0.89212	-0.16429	0.322467	-0.99183	0.599487	1.26648
P27824	CANX	0.093493	0.27807	0.155773	0.687175	1.213423	1.190936	-0.11509	-0.87738	-0.28292	-0.22024	0.022015	1.166581	2.161224	0.356441	1.068762	0.387584	0.50988	-0.15119	-0.23835
P27986	PIK3R1	-1.20179	0.760036	0.604965	-0.31871	0.212856	0.014295	0.46416	-0.58337	0.840099	0.952828	-0.4845	0.201217	0.259012	0.691093	0.50751	-0.69229	0.080629	0.47261	0.118786
P27987	ITPKB	-0.29083	0.146331	1.069869	-0.88039	-0.6814	-1.65415	0.540004	-0.49192	0.360181	0.02862	-0.36363	-0.29841	0.829252	-0.57176	0.173081	-1.08242	0.115364	-0.03603	0.338979
P28062	PSMB8	-0.39935	0.469037	0.94375	-0.05114	0.551653	0.860318	-0.40375	-0.43067	0.371132	-0.06518	0.066498	-0.40196	0.321712	-0.09036	-0.39258	0.77726	-0.58485	0.075811	-0.3997
P28065	PSMB9	-1.36752	-0.31281	0.249057	-0.56411	0.459923	0.24085	-0.14738	-1.15473	0.125998	0.092349	0.143601	-0.22705	0.041947	-0.13611	-0.86321	0.000899	-0.49663	0.297825	-0.93358
P28066	PSMA5	0.064951	-0.26397	-0.21232	-0.18607	0.50332	0.337318	-0.2131	0.207088	-0.2465	0.191218	0.18035	-0.4413	0.076178	-0.38493	-0.37413	0.273307	-0.35962	-0.34402	-0.35882
P28068	HLA-DMB	-1.83179	1.079759	0.493244	-1.0309	1.480152	0.993235	0.316198	-1.54415	-0.32421	1.103735	-0.16686	-0.16674	-0.13716	0.53671	0.263609	-0.18096	-0.07877	-0.01674	-0.8216
P28070	PSMB4	0.179265	0.096489	0.265823	-0.14277	0.579966	0.461126	-0.40264	0.046191	0.442966	0.216076	0.034141	-0.59061	0.129869	-0.19386	-0.10428	0.109462	-0.19462	0.258967	0.203163
P28072	PSMB6	0.637394	-0.38347	-0.41397	-0.2504	0.038336	0.064271	-0.29608	0.806187	1.053369	0.280104	0.553797	-0.63698	0.216594	-0.56453	-0.05932	0.431815	-0.68337	-0.20813	0.420009
P28074	PSMB5	0.514686	-0.42373	-0.41779	0.194846	-0.90713	-0.52206	0.10228	0.892337	0.749238	-0.25297	0.375346	-1.06415	-0.49392	-0.95943	0.530376	-0.31141	-0.45766	-0.20855	0.766085
P28161	GSTM2	-2.32398	-0.42911	-1.75779	-1.54109	-0.08826	-1.58734	-0.76999	-1.17399	0.176557	-0.01702	-0.94419	-0.50351	-1.15606	-0.95622	0.047931	-1.03792	0.468338	-0.37061	-0.52223
P28288	ABCD3	0.323721	0.321594	0.092208	0.942592	-0.03542	0.092187	0.391155	0.597033	0.160726	0.621179	-0.30481	0.444236	-0.44358	0.435344	-0.09512	-0.30814	0.747759	0.388775	-0.33738
P28289	TMOD1	-1.01693	-0.73599	-0.28523	-1.48557	-0.4852	-0.52809	0.261549	1.937635	0.143267	-0.16632	0.365773	-0.64232	-0.22001	-0.9114	-0.32262	-1.21978	-1.15502	-1.33606	-1.57343
P28290	SSFA2	-0.04841	0.392557	0.01914	-0.01573	-0.34179	-0.45465	-0.37615	-0.80737	0.190153	0.46253	-0.11116	-0.30121	-0.06521	0.125028	1.01661	0.85417	0.609998	0.148018	-0.13483
P28300	LOX	-0.90971	0.93518	0.093759	-0.51996	0.070269	-0.14156	0.495863	-0.02386	1.589636	-0.01636	-1.48576	2.143603	0.019415	0.804021	-1.23189	-0.5149	0.126732	-0.64933	0.01031
P28331	NDUFS1	0.11906	0.123592	-0.04769	-0.50294	0.245428	0.154479	0.249513	-0.23491	-0.59618	-0.93452	0.576721	0.267943	0.121535	-0.06125	0.985009	-0.3372	-0.40241	-0.14743	-0.56521
P28340	POLD1	1.299958	-0.04748	-0.21304	0.037272	0.795293	1.404774	0.019682	0.925714	-0.53921	-0.42059	0.024549	-0.04246	0.219283	-0.40177	-0.39116	0.43843	-0.12174	-0.77325	-0.98842
P28347	TEAD1	0.644819	-0.44798	-0.90596	0.633909	0.426787	0.512488	-0.48051	1.246478	-0.09373	0.315973	0.266126	-0.41689	0.171064	-1.05269	-0.12786	0.124807	0.120551	0.080579	0.744845
P28370	SMARCA1	1.416179	0.056909	-0.17518	-0.03847	0.132633	0.493774	0.224059	1.059271	0.924417	-0.14763	0.007756	-1.24734	0.158144	-1.02392	0.901595	-0.1325	-0.52849	-0.55417	0.588016
P28482	MAPK1	0.36388	0.28144	0.379727	-0.27018	0.228361	0.031971	-0.59831	-0.29413	-0.1312	0.473644	0.162242	-0.10008	0.203431	0.105669	0.061264	-0.02202	0.03321	0.29178	-0.09173
P28676	GCA	0.316386	-0.16222	0.127552	-0.01343	-0.32912	-0.98076	0.303534	-0.62385	0.736648	1.475897	-1.21058	0.947431	-0.62939	2.45669	-0.31396	-1.29392	0.997458	-1.13048	-0.57328
P28702	RXRB	0.126991	-0.15868	-0.16432	-0.49974	0.133728	0.625558	-0.00826	-0.23145	-0.09171	0.046414	-0.04591	-0.38637	-0.40437	-0.78793	0.657395	-0.1808	-0.35318	-0.15097	0.077999
P28715	ERCC5	0.99907	-0.12467	-0.03935	-0.31033	0.446607	1.26062	-0.22554	0.409547	-0.26963	-0.18354	0.45208	-0.35791	-0.38689	-0.02968	0.600442	-0.40834	-0.59372	-0.02975	-0.25978
P28799	GRN	-0.85216	-0.09845	0.14779	0.02106	0.495063	0.995212	0.437591	0.63097	0.197562	0.100276	-0.87561	0.702816	0.096343	0.103105	-0.69728	0.151236	0.06753	0.528519	-0.37718
P28827	PTPRM	-0.39764	0.059289	0.191771	-0.21494	0.466312	-0.84117	0.138641	-1.12981	0.283716	-0.03609	-0.04248	-1.07768	-0.1807	0.104073	0.839283	-0.0202	0.178923	0.68092	-0.02175
P28838	LAP3	1.424423	-0.8437	1.821943	-0.52511	1.038607	-0.18598	-0.27893	-0.35358	0.517733	0.708849	-0.63212	0.311172	0.56043	0.83559	-0.55766	0.71312	-0.1578	0.172378	-0.9943
P28906	CD34	-1.66008	-0.03619	0.64525	-0.8365	-0.28113	-1.15564	0.387483	-1.7391	0.569634	-0.85231	-1.14003	-0.8765	-0.64686	-0.4104	1.306519	-0.30787	-1.28144	-0.02447	-1.44923
P28907	CD38	-1.93957	1.694621	1.37892	-1.12164	0.613611	-0.41509	2.621634	-1.74187	-0.05828	-0.66113	-0.79374	0.118041	0.264427	0.373311	-1.5918	-1.05568	-0.49529	1.016405	-1.48135
P29034	S100A2	-0.8168	-2.87539	-0.49777	3.920909	-2.40453	-0.93783	-1.35219	0.808957	-1.0776	-2.86283	-1.23595	-1.98996	-2.01059	-0.92383	3.622239	-1.35144	-2.60327	-3.62347	-0.05494
P29083	GTF2E1	0.689546	-0.4039	-1.4248	-0.38592	0.143996	0.14314	-0.09209	0.367451	0.016981	0.356284	0.13593	-0.1909	-0.18634	-0.10212	-0.38466	0.242948	0.055522	0.370341	0.706864
P29084	GTF2E2	1.047268	0.566897	-0.21046	-0.20447	0.149284	0.28499	-0.04518	0.236611	-0.06994	-0.59663	0.018036	-0.68434	0.318578	-0.51026	-0.54421	-0.63921	0.540589	0.5821	0.076517
P29144	TPP2	0.426046	0.151613	0.16213	-0.30993	0.353822	0.713439	-0.08461	0.030338	0.144671	0.188458	-0.1247	-0.33729	-0.21191	-0.06886	0.295378	-0.18392	-0.20035	0.100618	0.101726
P29218	IMPA1	-0.86539	0.171088	-0.31713	-0.2251	0.82979	-0.17089	-0.31154	-0.05184	0.505885	0.820168	0.004227	0.575137	0.175257	-0.15849	-1.36087	0.003751	0.700817	0.145737	-1.01242
P29317	EPHA2	-0.35902	0.377209	0.7748	-0.79317	-0.59024	-0.56622	0.09665	0.113389	0.338103	-0.1229	0.076052	-1.03935	0.383087	-0.39449	-1.19182	0.091034	-0.22671	0.504614	1.200535
P29323	EPHB2	0.608151	0.523368	0.235394	-0.83289	-0.34951	-0.32561	0.157124	0.674121	-0.19779	-0.0414	-0.88747	-0.31056	-0.54443	-0.36024	-0.96757	-1.0804	-0.12278	0.070633	0.477835
P29350	PTPN6	-0.38219	0.247438	1.007875	-0.47143	0.224214	0.15662	-0.64153	-0.7546	-0.34345	1.103794	-1.01784	0.837848	-0.45217	1.824904	-0.15782	0.118346	0.36544	-0.12605	-1.1343
P29353	SHC1	-0.63787	-0.40149	0.089836	-0.11885	-0.12292	0.145999	0.568802	-0.07602	0.270298	0.443004	-0.33846	-0.69353	-0.02878	-0.52884	0.811448	-0.1139	-0.16976	0.14311	0.441604
P29372	MPG	-0.5873	-0.16373	-0.52277	-0.52009	0.397819	0.019568	-0.29518	0.068437	-0.20205	-0.0023	0.478316	-0.73207	-0.00093	-0.16395	-0.15334	0.150089	-0.25843	0.560499	-0.2302
P29373	CRABP2	-1.64447	-1.48874	-2.63149	-0.28234	2.546279	-0.31489	-0.73244	-0.40022	0.756377	0.723184	-1.89748	-0.22008	-0.51223	0.151416	-3.25913	-0.67522	-0.8708	-1.62337	-1.29076
P29374	ARID4A	0.963168	-0.14847	-0.07077	-0.12392	-0.03908	-0.04082	-0.15921	-0.15887	-0.13247	-0.67341	0.14555	0.27191	-0.31955	0.060479	0.000836	-0.79477	-0.11428	0.083403	-0.16604
P29375	KDM5A	0.666802	0.233666	-0.02098	-0.26301	-0.19691	-0.29916	-0.06792	0.202903	0.131273	0.114707	-0.11978	-0.20988	0.871842	-0.25139	0.306218	0.441866	-0.19735	0.063797	-0.44033
P29401	TKT	-0.35598	-0.55964	-0.28831	0.309271	-0.57253	-0.1347	-1.30671	-0.39546	-0.6333	-0.36186	0.349793	-0.49291	0.075491	0.120106	0.349482	-0.75031	-0.36476	-0.48865	-0.42779
P29466	CASP1	-0.99125	0.96037	0.808989	-0.24802	0.35528	-0.07609	-0.12175	-1.47765	-0.29688	1.642166	-0.60961	1.279962	0.047906	1.583408	0.062981	-0.36281	0.468945	-0.03113	-0.73503
P29474	NOS3	-0.65773	0.261092	0.006854	-0.63003	0.177997	0.348201	-0.08906	-0.45273	0.099926	-0.12133	-0.63464	-0.67618	-0.17522	1.176496	0.038015	-0.15701	-0.2967	0.254553	-0.58217
P29508	SERPINB3	-1.15568	0.335451	0.186927	-1.48626	-0.6707	0.049495	-0.25063	-0.96543	0.704025	-0.18832	-2.12423	-0.0425	-0.41451	-0.9077	-0.32293	-0.37139	-0.17838	-0.2054	-0.50889
P29536	LMOD1	-1.73791	-1.64353	-1.54784	-2.53725	-2.31423	-2.8559	-0.81639	-3.04851	0.099659	-0.73295	-1.92654	-1.60548	-1.74317	-0.49131	-0.49704	-1.00522	-1.75805	-0.58023	-0.6894
P29558	RBMS1	0.732561	-0.08295	-0.15801	0.602122	-0.06052	-0.58181	-0.23124	-0.45609	-0.13693	-0.02049	-0.16391	-0.06451	0.16678	-0.12139	-0.36626	-0.08725	0.397237	0.027925	-0.14882
P29590	PML	-0.20776	0.466	0.664323	-0.24486	-0.27369	-0.09122	-0.01644	-0.0278	-0.50338	-1.08909	0.690432	-0.36028	-0.07835	-0.39147	-0.35944	-0.62051	-0.84565	-0.35303	0.439661
P29597	TYK2	-0.48166	0.301712	-0.02146	0.037069	0.314617	0.146928	0.224874	0.77417	-0.31062	-0.12507	0.012072	0.014761	0.078698	0.326566	-0.56546	0.411306	-0.06675	0.62151	-1.01068
P29622	SERPINA4	-1.72471	0.118919	-0.79707	-1.24584	-0.98523	-1.36034	-0.20783	-1.04318	0.609919	-1.43185	-0.87952	0.019959	-0.48799	0.000118	-0.42013	-0.86726	-0.2963	-0.11117	-0.75995
P29692	EEF1D	0.498903	0.812403	0.114295	0.275682	-0.12024	0.105176	0.039538	0.045411	0.144625	0.584873	0.062593	-0.55783	1.110155	-0.90779	0.111481	0.198823	-0.24233	0.543401	0.071298
P29728	OAS2	0.235738	1.107338	2.615643	-0.81591	0.407994	0.441542	0.143339	0.370431	-0.18938	-0.40483	-1.85976	1.28216	0.273575	0.993851	-1.06484	-0.90521	-0.16366	-0.10639	-1.62974
P29762	CRABP1	-0.88912	-0.05356	-0.26019	-1.34269	-2.49369	-2.30222	-0.26861	2.254187	0.142299	-3.84131	-1.89598	0.620804	-0.58743	-0.14818	-1.79436	1.304519	-0.89117	-2.33368	-2.01416
P29966	MARCKS	-0.93215	0.846656	-0.79162	-1.91308	-0.17	-0.72748	-0.37971	-1.0419	0.010249	-0.0367	-0.88673	0.151459	0.238134	-0.21473	-0.73473	-0.67266	-0.67222	0.38721	1.089595
P29972	AQP1	-1.92633	0.474622	1.867284	-1.41426	-0.38524	-0.62523	1.062964	-0.86994	1.193891	-0.26511	-1.71749	-0.47857	0.416527	-0.28892	-0.09307	-0.79528	-1.02615	0.345991	-0.48966
P29992	GNA11	-0.76766	-0.30875	-0.62313	-0.73419	-0.50342	-0.78715	0.159249	-0.97117	-0.2256	-0.48846	0.41752	-0.35993	-0.59548	-0.32397	-0.03665	-0.71609	-0.77168	-0.47581	-0.30573
P30038	ALDH4A1	0.917754	-1.0972	-0.30159	0.091556	-0.06267	-0.30378	-0.8412	0.476455	-0.38626	0.343473	0.459492	0.521879	0.234287	0.70653	1.789537	0.76143	-0.78835	-0.74366	0.169433
P30040	ERP29	0.514377	0.177659	0.114573	0.241975	0.643484	0.074985	-0.40674	0.677868	0.314406	0.040035	-0.40793	-0.30163	-0.0988	0.074005	-0.74014	-0.10627	0.051923	0.174466	-0.00229
P30041	PRDX6	-0.16891	-1.40554	-1.75568	-0.45946	1.132623	-0.58576	0.021395	1.131805	0.901302	0.95443	-0.71825	-0.49829	-0.09764	-0.42357	0.551716	-0.30315	-0.56057	-0.74373	-0.48136
P30042	C21orf33	1.023877	-1.15707	-0.23361	0.822957	2.982265	0.094534	-1.03237	-0.84015	0.402348	0.833001	-0.3264	0.419731	0.829737	-0.25605	0.822684	0.907033	-0.41241	-0.38623	-0.88429
P30043	BLVRB	-0.85395	0.343339	0.054065	-1.08552	1.392888	-0.59802	0.803962	-0.97664	1.088928	0.939352	-1.27197	0.969593	-0.6578	0.986011	-0.4517	-0.86473	-0.28614	0.143658	-1.53781
P30044	PRDX5	-0.53417	-1.09538	-1.43686	-0.81228	-0.49579	-0.98771	-1.26401	0.777809	0.102223	-0.98829	0.875265	-0.42839	-0.27157	0.210373	1.363416	0.552509	-0.51623	-0.91055	-1.25585
P30046	DDT	1.024709	-0.29103	-0.24953	0.003853	1.15309	-0.67234	-0.05755	-0.57284	0.320597	0.191601	0.347764	-0.10699	0.327461	0.229732	0.43555	0.528024	0.635374	0.926985	-1.26475
P30047	GCHFR	1.326768	0.096673	0.075736	-0.44204	-0.4889	-0.04546	0.051357	-0.50101	-0.78447	-0.51938	0.69613	-1.24907	0.081113	-0.43613	-0.8357	-0.099	-0.56113	-0.689	-0.11377
P30048	PRDX3	0.398164	-0.39606	-0.37061	0.125532	0.688955	0.175453	-0.71895	0.334147	-0.15376	1.196781	0.860752	-0.27788	-0.11887	-0.4154	0.680848	1.582168	-0.32697	0.513085	-0.80951
P30049	ATP5D	-0.53563	0.250381	-1.42362	-0.37057	0.509582	-0.39578	0.252877	-0.49083	-0.1822	0.666871	-0.1043	-0.00569	-0.61349	-0.17066	0.234743	0.701674	-0.46858	-0.31495	-1.1841
P30050	RPL12	0.162646	-0.35755	-0.09272	0.044564	0.366306	0.120681	0.022384	-0.06029	0.12299	-0.17219	0.183614	0.18884	-0.01324	-0.55146	-0.01302	-0.12015	0.036386	-0.03806	-0.09208
P30084	ECHS1	-0.56123	-1.01506	-0.00298	0.927312	1.201091	0.907475	-1.00527	1.263544	0.185778	1.317083	0.480354	0.283566	0.347034	-0.13686	1.537347	2.559325	0.13322	0.284636	-0.41258
P30085	CMPK1	-0.30482	0.309899	-0.6724	-0.61664	0.204921	-0.04088	0.307371	0.313265	1.262704	0.526131	-1.0992	0.235999	0.209681	0.27907	-0.38909	-0.70429	0.636007	-0.05814	-0.35778
P30086	PEBP1	0.260556	-1.0676	-0.70067	-0.2331	1.196861	-0.52637	0.157827	-0.05414	1.514437	0.22897	-0.00727	-0.85619	-0.56653	-0.49533	0.238542	-0.57595	0.175341	-0.39657	-0.40093
P30101	PDIA3	0.542837	0.497471	0.261728	0.245557	-0.0088	0.017599	-0.66792	0.038141	-0.02108	0.090246	0.064967	0.177129	0.737876	0.037642	-0.4613	0.473632	0.730103	0.530461	0.460403
P30153	PPP2R1A	0.052618	-0.11495	0.271167	-0.17736	-0.19682	0.142	-0.07293	-0.21305	-0.56979	-0.2108	0.120905	-0.50205	-0.0644	-0.07685	0.226183	0.149016	0.020409	-0.15952	0.279422
P30154	PPP2R1B	-0.22428	0.299744	0.449039	-0.18196	-0.3166	-0.0213	0.151263	0.027829	-0.58236	-0.19796	-0.5736	-0.49965	0.3701	0.124867	-0.86824	0.356664	0.823073	0.214156	0.297541
P30260	CDC27	-0.24581	0.064453	0.999214	0.335741	-0.02844	0.158906	0.372015	-0.07647	0.562855	-0.21345	0.460079	-0.08525	-0.07081	0.046778	0.025916	0.744039	0.376217	0.488322	0.185159
P30273	FCER1G	-2.59147	2.31312	1.94429	-0.72948	0.42678	-0.26622	1.404365	-1.6654	-0.28088	0.387555	-1.65256	1.735995	0.449575	1.493512	0.00625	0.024624	0.786545	0.47939	-1.52922
P30405	PPIF	-0.00382	0.176163	-0.74404	-0.13356	2.192939	-0.10319	-0.76255	-0.60732	-0.15068	2.525016	0.00298	0.88478	0.069129	0.295596	-0.75416	-1.04716	0.678903	0.071433	-1.31132
P30414	NKTR	0.81931	-1.05669	0.016799	-0.25502	-0.40715	-0.05989	-0.27873	0.44376	-0.35623	-0.19862	0.293148	-1.03825	-0.29634	-0.10155	-0.22371	-0.38247	0.100444	1.030968	0.424641
P30419	NMT1	-0.01563	-0.08745	-0.11587	0.071057	-0.094	0.325227	-0.45617	-0.12558	0.154586	-0.12424	0.306431	0.031479	-0.0045	-0.11307	-0.24449	0.605073	0.0735	-0.06233	-0.24806
P30466	HLA-B	-0.01618	1.436731	1.263952	-0.72152	0.556135	0.957915	0.594059	-3.28314	-0.96995	-2.62778	-3.06798	0.395039	0.277344	0.719215	-0.26179	0.920584	0.699859	1.3844	-0.44529
P30481	HLA-B	-1.2218	2.182906	-0.05168	-0.63896	-2.59614	-1.74594	0.561899	-1.30184	0.273422	0.305244	-0.61098	-1.39404	0.115908	-1.90628	-3.50842	-0.2922	-2.5063	-1.09294	-3.59335
P30499	HLA-C	-0.83721	0.337197	1.606492	0.027467	-0.3939	-0.39944	1.150931	0.56549	0.425933	-0.02023	-0.46396	-0.42218	0.25946	-0.49004	-0.42748	0.54285	-0.17105	-0.42103	1.672136
P30504	HLA-C	-1.91774	1.076469	0.654349	-0.69305	0.501785	1.185495	-0.51246	-0.34023	-0.2209	-1.12972	-0.90601	0.319829	-0.17276	0.894115	0.078628	-2.67868	-1.01509	0.096528	-3.62219
P30511	HLA-F	-0.98226	1.726199	0.999886	-0.42769	0.628923	0.545076	0.608106	-0.64994	-0.1271	-0.94849	-1.27688	-0.08977	0.585139	0.053206	-0.90013	-0.96191	0.455261	0.940398	-0.88001
P30519	HMOX2	-0.25905	0.853508	0.83429	-0.24366	-0.18351	-0.27547	0.730933	0.172101	-0.64609	0.214165	-0.02315	-0.18671	0.272299	-0.06257	0.008855	0.462296	0.686532	0.611476	0.012516
P30520	ADSS	-0.15074	-0.66692	-0.01811	0.009454	0.614594	-0.00718	-0.5261	0.653006	0.856537	0.97536	-0.46146	-0.07743	0.045175	0.847216	0.250299	-0.52288	0.624143	-0.81416	0.219218
P30533	LRPAP1	0.491023	-0.00115	1.025575	-0.58716	0.495844	1.104984	-0.52601	0.695929	0.471124	-0.10263	-0.46601	-0.21467	0.165184	-0.00872	-0.4843	-0.14282	-0.34839	-0.024	0.0352
P30536	TSPO	-0.71999	-0.053	0.094225	-1.00363	0.756815	0.082132	-0.17832	-0.17326	0.145726	0.407474	0.694047	1.200817	-0.18171	1.17146	0.160917	0.649935	0.290396	-0.76692	-0.1718
P30566	ADSL	-0.35606	0.291048	0.086838	-0.26053	0.179711	0.149321	-0.18606	-0.49725	0.025822	0.1838	-0.28729	-0.25676	-0.12234	-0.33693	-0.09438	0.588489	0.353503	0.952431	-0.10611
P30613	PKLR	0.138883	-0.12148	-0.30912	0.631905	0.573387	-0.66114	-0.37008	-0.8283	0.151554	-0.11821	-0.27603	-1.14641	0.171747	0.556508	-0.87806	-1.07118	0.454692	0.994233	-0.12282
P30622	CLIP1	-0.47887	-0.28734	0.523134	0.512086	-0.10593	0.058552	-0.50059	-0.0111	-0.18088	-0.43319	0.155324	-0.40935	0.440073	-0.20897	0.469984	-0.3489	-0.19811	0.22769	0.159228
P30626	SRI	0.268553	-0.8685	-0.39284	-0.48279	0.706249	-0.77354	-0.49378	0.356941	0.711625	1.511026	0.420302	1.026562	0.778607	0.232165	-0.18812	0.159408	0.235741	0.20034	0.40386
P30679	GNA15	-0.42536	0.438697	-0.42737	-0.26261	0.492218	-0.16344	-0.11267	-1.53349	-0.07576	-0.21582	-0.58498	1.393253	-0.89651	1.134637	-1.05323	-0.373	0.288764	-0.01273	-0.2148
P30711	GSTT1	-2.35731	-0.84569	-0.73496	1.210404	-1.55767	-1.65522	-0.97419	-2.81025	0.684883	-1.07124	0.063004	-0.89467	0.099859	-0.45076	0.536428	0.245048	-0.44225	-3.50666	-0.23441
P30740	SERPINB1	-1.10835	0.407181	0.215153	-0.1438	-0.92892	-0.35107	-0.41788	-0.71461	-0.15255	1.432906	0.264029	-0.0196	-0.30436	2.287157	-0.46536	0.350752	-0.33843	-0.70618	-0.7805
P30837	ALDH1B1	-0.26213	-0.35224	0.328329	0.565743	-1.50342	-1.19337	-1.50553	-0.09271	1.513427	0.359515	0.017915	-0.17359	0.804681	-0.61034	-0.80672	0.323034	-1.62081	-1.82451	1.123751
P30876	POLR2B	0.472775	-0.00509	0.639566	-0.02137	0.154199	0.29557	-0.03453	0.369015	-0.09384	-0.16259	0.297928	-0.50086	-0.11233	-0.35359	-0.0603	-0.11501	-0.12305	0.017989	0.079488
P31040	SDHA	0.384286	-0.22734	0.111797	-0.0515	-0.31256	-0.29571	0.111335	-0.21342	0.151374	0.647936	0.010559	0.143401	-0.09712	-0.23927	1.817948	1.378717	-0.48739	-0.27792	-0.65604
P31146	CORO1A	-2.03375	1.298776	1.726803	-1.09358	0.704301	0.154839	-0.15271	-2.19965	-0.40275	1.707797	-1.81953	1.018698	0.054735	2.149695	-0.62172	-0.54115	-0.06721	0.751034	-1.41067
P31150	GDI1	-0.89361	0.269261	-0.39033	-1.14659	0.219774	0.064602	-0.34552	0.02714	0.410054	0.140823	-0.10371	-0.8555	-0.16219	-0.55913	-0.45887	0.173763	0.334927	0.716005	-0.1603
P31151	S100A7	-1.57563	0.221659	-0.1715	-1.44416	-0.38174	0.961292	0.133699	-0.99609	0.018033	-0.72533	-2.05274	-0.03332	-0.26915	-0.41836	-0.78741	-1.83397	-0.70626	-0.28145	-1.70675
P31153	MAT2A	0.495341	-1.63799	0.723596	1.508427	1.349793	1.102336	-0.97829	0.804477	0.210885	0.493178	0.137103	-0.35186	0.579291	-0.36855	0.540531	0.384866	0.124236	-0.09269	0.475975
P31321	PRKAR1B	-0.11579	0.759242	0.139443	0.137386	0.981208	0.520785	-0.49019	0.744174	-0.25584	0.088417	0.180721	-0.13329	0.513398	-0.72155	0.252445	0.175134	0.21559	0.121227	-0.78168
P31323	PRKAR2B	-0.87414	-0.75445	-0.74561	-1.65261	-1.03881	-0.98379	-0.41323	-1.72064	0.07622	-0.88036	0.560751	-0.92461	-1.51151	0.011148	-0.47934	-1.14578	-0.68704	-0.717	-1.11426
P31327	CPS1	-0.01919	0.431039	0.076117	-0.5453	-1.65619	-1.28664	-0.20814	-0.46046	-0.15349	-0.15722	-0.95301	-0.97565	0.152814	0.757518	-0.42751	0.109327	1.80266	1.44963	0.205767
P31350	RRM2	0.847311	-0.69989	-0.77083	0.156058	0.504903	0.47334	-0.52138	0.532837	0.393494	1.034635	-1.1705	-0.87421	-0.16803	0.006441	-0.63822	1.109395	0.536442	-0.24321	-0.09718
P31431	SDC4	0.330682	-0.87093	-0.19422	-0.2225	-0.83479	-0.26918	0.257341	1.822132	0.370913	0.698755	0.335292	-1.88485	-0.22015	-0.03977	1.130446	-0.5688	0.475893	0.128586	0.745664
P31483	TIA1	0.852488	0.11451	-0.27371	-0.0576	-0.33851	-0.05603	-0.01084	0.641958	-0.39777	-0.37138	-0.41403	-0.77058	0.06791	0.088029	-0.04053	-0.95886	-0.02437	-0.25342	-0.15642
P31689	DNAJA1	0.56932	-0.3508	-0.17819	0.359638	0.582509	1.132881	-0.6656	0.007498	-0.45951	-0.41286	0.302597	-0.22476	-0.20868	0.144066	-0.39069	0.220254	0.277196	0.119989	-0.16612
P31749	AKT1	0.237801	0.153539	0.320104	0.524965	0.003588	-0.05081	-0.2983	0.43106	-0.65686	0.07308	0.133761	0.125213	0.055304	0.430027	0.007039	-0.02269	0.520482	0.101288	0.839366
P31751	AKT2	-0.72774	0.229716	0.898586	-0.56829	0.281773	0.420173	0.592223	2.201331	-0.23634	-0.14706	-0.51174	0.039776	0.430776	0.038761	1.029094	-0.68587	0.00506	-0.30071	1.616396

P31930	UQCRC1	0.141163	-0.01374	-0.2771	0.681159	0.239661	0.677618	-0.03405	-0.13339	-0.29079	-0.53565	0.486139	0.171651	-0.12811	0.22292	1.659242	-0.54541	-0.31102	-0.2157	-0.8274
P31937	HIBADH	0.844144	-0.82842	-0.30714	-0.37446	1.047085	-0.38457	-0.2131	-0.29958	1.469499	-0.20361	0.07636	-0.19071	-0.26568	-0.35018	0.836291	1.45422	-0.7943	-0.37799	-0.91457
P31939	ATIC	0.022435	-1.2473	-0.29308	-0.08876	1.196721	0.557923	-0.7089	0.920241	0.365522	0.383188	-0.21322	0.524794	0.583399	0.225728	-0.07135	0.553217	1.030395	-0.12607	0.173498
P31942	HNRNPH3	0.671802	-0.19641	-0.18407	-0.7451	0.156088	0.413923	-0.49999	0.608105	-0.17813	-0.04835	0.474211	-0.28016	-0.49397	-0.37945	-0.38251	-0.13291	-0.13237	0.345873	-0.01138
P31943	HNRNPH1	0.261088	0.136661	0.289499	0.192323	0.213732	0.40771	-0.25215	0.564527	0.141759	0.189771	0.21425	-0.00131	-0.03252	-0.06169	0.269632	-0.51051	0.08545	0.407116	-0.06131
P31944	CASP14	-1.58658	2.968773	0.044289	-0.41851	-0.72079	-1.26974	2.509259	-0.53738	1.527144	-0.92169	-0.02656	-0.35783	0.38524	0.055743	-0.41614	-0.72486	-0.06471	-0.17148	-0.63285
P31946	YWHAB	0.334361	-0.78285	0.296777	0.136155	0.034641	0.189668	-0.88768	0.612706	0.218947	0.851308	0.212409	-0.71442	0.654782	-0.27274	0.430353	-0.31399	-0.19781	0.188977	0.292977
P31947	SFN	1.144415	-0.97765	-0.11413	0.109553	0.415924	0.759832	-1.04906	0.230352	0.120638	0.162753	-0.35397	-0.88565	0.071631	-0.69329	-0.3782	0.035615	-0.24327	-0.90354	-0.11283
P31948	STIP1	0.687768	-1.42391	-0.11129	0.019951	-0.0848	0.082582	-1.26724	-0.30448	-0.47819	-0.03762	0.281086	-0.17175	0.993384	-0.46429	-0.2583	0.66951	0.382652	-0.01477	-0.57982
P31949	S100A11	-1.71707	0.507466	-1.15795	-1.02942	-0.30466	-2.17508	0.014023	-1.09501	0.089547	0.316968	-2.09182	2.03025	-1.4089	2.189272	-0.88169	-1.51985	2.217073	-1.69607	-1.07387
P31994	FCGR2B	-2.69277	1.024957	0.803524	-0.48198	0.144201	-0.05405	-0.04392	-0.67632	0.076047	1.921477	-1.12003	-0.15236	0.491824	0.864785	-0.2459	0.070661	0.124221	0.542076	-0.01614
P32004	L1CAM	0.954187	-0.92496	0.212625	0.007513	0.530382	0.126749	1.239746	1.833873	-0.67772	-2.15207	-1.24177	-0.72899	1.023846	-0.23108	1.601561	-1.81893	-0.02505	0.657351	0.848117
P32019	INPP5B	-0.02338	-0.14554	0.226652	0.178494	0.633492	1.145132	-0.18674	0.056258	-0.22804	0.293472	-0.47343	-0.13469	-0.01308	0.5031	-0.17218	0.196475	-0.27589	-0.82235	-0.63935
P32119	PRDX2	-0.84905	-0.30479	-0.63388	-0.99044	0.789807	-0.73276	-0.31147	0.855093	1.765721	-0.23425	-0.24343	-1.10203	-0.9444	-1.38768	-0.1038	-1.10583	-0.38623	0.638103	-0.53735
P32121	ARRB2	0.39064	1.086156	0.32469	-0.7104	0.372514	-0.386	-0.86141	-0.42371	-0.90378	1.589636	-0.7356	1.044326	-0.28781	1.955808	-0.74438	-0.29504	0.573006	-0.3025	-1.4128
P32321	DCTD	0.223372	-0.81849	-0.14783	-0.64739	1.190665	0.257194	-0.45889	0.968412	0.244589	-0.10639	0.795101	-0.23414	0.204106	-0.4103	-1.1312	0.096569	-0.32625	-0.6941	0.680314
P32322	PYCR1	-0.92762	0.70493	-0.61296	-0.18015	-0.68967	-0.45278	1.11717	-0.45606	0.219911	-0.12548	-0.47137	-0.76805	0.52625	-0.90672	-0.49243	1.211543	0.525695	0.435342	1.680891
P32418	SLC8A1	-1.8114	1.414647	0.118665	-0.02574	0.322011	-0.13844	0.503273	-1.29343	0.19531	-0.01659	-1.52304	1.161451	0.176994	0.767404	2.370222	-1.01635	0.300189	0.587786	-1.25686
P32455	GBP1	1.300975	1.544374	0.821946	-0.5089	0.07334	0.413104	-0.61924	-0.49268	-0.10329	-0.33595	-1.14853	-0.35811	1.227394	-0.61343	-0.51479	-0.12299	-0.3292	0.237298	0.003834
P32456	GBP2	-0.89633	1.421407	1.373037	0.281852	0.531741	-0.02322	-0.59583	-1.51602	0.117207	0.669612	-1.16941	0.137354	0.714482	0.029348	-0.57076	-0.11061	0.247251	0.59648	0.640399
P32519	ELF1	-0.77709	0.30364	0.186203	-0.87817	-0.54477	-0.10255	0.240793	-0.45229	0.157103	0.302998	0.511329	-0.30247	0.004441	0.115797	-0.0257	-0.56509	0.32002	0.292731	0.219464
P32780	GTF2H1	-0.10886	0.149475	0.047655	-0.20302	0.378155	0.306823	0.351487	0.27343	0.711651	-0.37241	0.291031	0.285074	0.287465	0.106465	-0.12129	0.30501	-0.06391	0.421089	0.325888
P32856	STX2	-0.91503	0.174104	0.026994	-0.5105	-0.40775	-0.44997	0.261158	-1.32172	0.063799	-0.12329	0.503902	-0.67481	-0.01321	-0.30646	-0.80541	-0.09674	-0.18243	-0.13541	0.107065
P32929	CTH	1.567587	-1.06227	0.08285	-0.18036	0.027356	0.23666	-0.6259	0.000916	-0.16545	-0.59575	1.566377	-0.69338	0.644357	-0.07285	-0.59598	2.129628	2.598132	2.99055	0.363741
P32942	ICAM3	-1.90831	0.643695	1.120826	-1.59793	0.010631	-0.06652	0.165007	-1.47598	0.075206	1.503602	-1.04866	0.132326	-0.39217	2.24199	-0.51584	-1.26962	0.173307	0.676899	-0.99046
P32969	RPL9	0.330296	-0.10632	-0.29197	0.246278	0.120195	0.115709	-0.40624	0.142241	-0.4579	-0.10552	0.545983	0.201581	-0.15182	-0.15797	0.174864	0.133232	0.142156	-0.23672	0.148742
P33121	ACSL1	-1.42798	0.701428	0.17281	-0.51261	-0.4838	-0.99115	0.367307	-1.18926	0.024245	0.299251	-1.53819	0.922622	-0.37053	1.675595	-0.65709	-0.46712	0.734979	0.087233	-0.79643
P33151	CDH5	-1.26151	0.625444	0.037874	-1.34661	-0.00292	-0.70779	0.648031	-0.85705	0.151176	0.650173	-0.97938	-1.31003	-1.12066	-1.02237	0.655559	-0.03936	-1.10997	0.660539	-0.59291
P33176	KIF5B	-0.02411	0.075849	-0.02971	-0.07203	-0.22824	0.246063	-0.72318	-0.18297	-0.15064	-0.07525	-0.04497	-0.12164	0.223487	0.006431	-0.06046	0.108815	0.087326	-0.20836	0.395046
P33240	CSTF2	0.567684	-0.3504	-0.08126	0.104674	0.146273	0.034284	-0.42104	0.527796	-0.31658	0.280339	0.642051	-0.27985	0.030985	-0.28796	-0.24087	0.222611	-0.06366	0.556918	-0.04136
P33241	LSP1	-2.15616	1.529664	0.653775	-0.64139	0.586897	-0.30604	-0.70361	-1.64714	-0.53071	0.729282	-1.47472	0.518333	0.905983	1.311959	-0.59021	-0.76233	0.495561	-0.03034	-1.59062
P33316	DUT	0.241738	-0.63844	-0.36853	-0.08465	0.979463	0.226599	0.227894	-0.61049	0.377772	0.674353	0.108919	-0.39578	0.355088	-0.30636	-0.45479	-0.01665	0.295306	0.091424	-0.74878
P33402	GUCY1A2	-1.09493	-0.52895	-0.66324	-1.13531	-0.26566	-2.64435	-0.07585	0.037602	-0.01135	-0.48647	-0.84426	-0.88223	-0.71353	-0.797	-0.74372	-0.67756	-1.02116	-0.30042	-0.83718
P33527	ABCC1	0.216018	0.163561	0.490022	-0.28273	-1.06333	-1.19334	0.229342	0.237792	-0.31632	-0.35529	0.324052	0.132617	0.299602	-0.0462	-0.4883	0.195029	0.200181	0.144754	0.146097
P33897	ABCD1	-0.13607	0.990494	1.330817	0.815278	0.582781	0.402789	0.540373	0.060634	-0.02343	0.218475	-0.99508	1.030036	-0.24513	0.715881	-0.70422	-0.44814	0.096889	-0.39939	-0.55755
P33908	MAN1A1	-1.21564	0.84904	1.126258	-0.96038	1.031575	0.354788	1.444711	-0.92509	0.448985	0.702845	-0.87431	0.026807	0.239534	0.487468	-0.89745	-0.22012	0.100894	0.548475	0.378804
P33981	TTK	0.563133	-0.00019	0.06628	0.120888	-0.00318	0.828455	0.357536	0.334918	-0.58712	-0.08709	-0.73282	-0.23299	-0.26382	-0.17917	0.192027	-0.19158	0.442915	0.391283	-0.40177
P33991	MCM4	2.035104	-0.80463	-0.3658	-0.17813	1.497304	2.010583	-0.106	1.085113	-0.39687	-0.71963	-0.16985	-0.49044	-0.1372	-0.60193	-0.20179	0.428752	-0.29743	-0.63635	-0.55135
P33992	MCM5	2.175093	-0.49112	-0.15968	-0.09304	1.609286	1.952107	-0.02778	0.795301	-0.57354	-0.74028	-0.06656	-0.56557	-0.18214	-0.5759	-0.17847	0.653829	-0.15187	-0.51374	-0.64057
P33993	MCM7	2.224527	-0.37609	-0.05991	-0.14698	1.479592	1.918867	0.50176	1.193739	-0.49547	-0.86491	-0.43104	-0.64833	-0.17384	-0.73598	-0.17188	0.457763	-0.0205	-0.78162	-0.44327
P34059	GALNS	0.368543	-0.37008	0.241078	0.13493	1.502837	0.080478	-0.3303	0.510397	0.561527	1.199799	-0.13518	0.411455	0.449409	0.631995	-0.25998	-0.11155	0.111192	0.739676	0.674722
P34741	SDC2	-1.09194	-0.34835	-0.3225	-1.95404	-2.36388	-2.60142	0.367194	-2.77482	0.064817	-1.08386	0.47668	-1.95253	-0.80905	-1.49752	-1.28935	0.30996	-0.94002	0.471703	-0.51885
P34896	SHMT1	0.620224	-0.32926	-0.84315	0.698398	0.340879	0.75754	-0.35633	0.598078	-0.20876	-0.1859	0.579116	-0.58415	0.149253	-0.08341	1.130267	-0.87436	-0.38359	-1.32807	-1.04139
P34897	SHMT2	1.197505	-0.24652	-0.27032	-0.11998	0.087351	0.336425	-0.3015	0.962789	-0.72692	-0.42531	0.678992	0.257748	0.468263	-0.32778	-0.75675	0.028127	0.499099	-0.35984	1.107733
P34910	EVI2B	-1.94731	1.529615	0.671737	-1.37067	0.445644	-0.13396	0.92727	-1.96547	0.220051	1.697427	-2.27452	1.523053	0.4777	1.738096	-1.13947	-1.05649	0.56868	0.172343	-1.37342
P34913	EPHX2	-0.19485	0.431841	-0.8059	-0.4961	-0.18367	0.062317	-0.0858	-0.16827	-0.56411	-0.65709	0.797929	-0.53885	-0.65586	-0.74923	-1.04445	-0.08512	-0.53324	-0.55203	-0.03985
P34932	HSPA4	-0.45856	-0.82862	0.062838	0.468616	0.91793	0.617923	-0.87728	0.417837	0.161967	0.791993	0.097673	-0.40666	0.414031	-0.43147	0.606474	0.477522	0.062668	-0.0491	-0.17843
P34949	MPI	-0.57669	-0.09251	0.152916	-0.44056	0.629598	0.440129	-0.01055	0.260212	0.209267	0.090705	0.378368	0.013995	-0.59261	0.095299	-0.85606	-0.13302	0.42903	-0.58183	-0.13145
P35052	GPC1	-0.29413	-0.65839	-0.15536	-1.11954	-0.37452	-0.45274	-0.59885	-0.28792	0.702184	-0.63251	-0.25995	-0.79046	-0.44526	-1.07507	0.251721	0.5111	-0.38595	0.062922	0.357837
P35080	PFN2	0.363255	-1.01582	-0.40585	-0.75741	1.214081	0.633967	0.059367	1.477119	1.190597	0.719829	0.665227	-0.44982	-0.12329	-0.36015	-0.47006	-1.4677	1.135548	0.001763	-0.03043
P35221	CTNNA1	0.659802	-0.60222	-0.87397	0.456812	-0.13356	-0.1864	-0.4929	0.515825	-0.46135	-0.66424	0.500963	-0.36772	0.388681	-1.03682	0.721027	0.475354	-0.41268	-0.11808	-0.16531
P35222	CTNNB1	0.83655	-0.28921	-1.01949	-0.4702	-0.27795	-0.29845	0.209175	0.685467	0.071533	-0.40052	0.352127	-0.6945	0.366218	-0.88164	0.444002	0.634862	-0.74	-0.39278	0.187037
P35232	PHB	0.146143	0.079552	-0.50854	0.599344	-0.52276	-0.4698	0.245389	0.169192	-0.79271	0.133752	-0.19128	0.315626	0.350329	-0.00584	0.448056	1.475908	-0.55104	-0.54077	-0.06788
P35236	PTPN7	-1.15776	0.725974	0.971855	-0.66567	0.301503	0.387417	0.214567	-0.60005	-0.3211	0.715307	-0.93904	0.033281	0.213393	1.461858	-0.70502	-0.6647	0.356047	0.167237	-1.12016
P35237	SERPINB6	-0.75995	0.415598	-0.09546	-0.07129	-1.16795	-0.12078	-0.01266	0.988846	0.456132	0.353557	0.296336	-0.4159	-0.0196	0.051019	0.03425	0.439653	-0.73889	-0.47323	0.55324
P35240	NF2	-0.21648	0.135264	0.411212	-0.03483	-0.07851	0.042222	1.452716	-0.13354	0.163198	0.113602	-0.03047	-0.83372	-0.18933	-0.52069	-0.16033	-0.41601	0.44819	0.897884	0.246663
P35241	RDX	0.354073	-0.28134	-0.08337	0.573124	0.409531	0.295374	0.048504	0.917973	0.023663	0.09531	-0.21522	-0.86379	0.323871	-0.40148	-0.25642	-0.5051	-0.70518	0.537866	0.497742
P35244	RPA3	1.023374	-0.64656	0.212091	-0.23727	0.862968	0.762498	-0.93969	1.130979	-0.12322	0.378318	0.36993	-0.37926	-0.35758	0.379166	-0.01995	-0.16931	0.255618	-0.57642	-0.04611
P35249	RFC4	1.230191	-0.11627	-0.20922	0.280207	0.812942	0.984864	0.246554	0.666774	-0.64266	-0.3395	-0.202	-0.33801	-0.29831	-0.18252	-0.07958	-0.0377	0.094995	-0.50143	-0.44599
P35250	RFC2	1.188451	0.023201	0.017882	0.008074	0.45527	1.019338	-0.05015	0.218387	-0.26591	0.088421	0.007984	-0.36082	-0.31534	-0.0443	0.063046	0.317388	-0.02694	-0.51732	-0.31366
P35251	RFC1	1.431142	-0.65004	1.053614	-0.03979	-0.45404	0.46132	0.10048	0.172747	-0.30518	-0.0924	0.166246	-0.88411	-0.19902	-0.74836	-0.36116	0.186817	-0.78268	-0.28248	-0.44458
P35268	RPL22	0.267116	-0.25028	-0.20754	0.174469	-0.11735	0.006052	0.121698	0.016259	-0.03495	-0.24498	0.338996	-0.18648	-0.81001	-0.33644	-0.05607	0.107949	0.244061	-0.13145	-0.10454
P35269	GTF2F1	-0.14347	-0.98277	-0.13969	-0.61807	-0.30393	0.61144	-0.72212	0.556356	0.213739	-0.02258	0.53448	-1.23104	-0.28915	-1.21432	0.661579	0.02398	-0.7948	0.361152	0.254863
P35270	SPR	0.527748	0.153635	-0.17124	-0.1666	1.380207	0.323717	0.603233	-0.28545	-0.49498	0.26649	-0.15793	0.505463	0.369399	0.367973	-0.61008	0.148915	-0.46947	-0.40904	-0.74858
P35442	THBS2	-1.37169	-0.92923	-0.67176	-2.04013	-1.26677	-1.26496	-1.00317	-1.36046	1.299884	-1.68108	-2.54261	-0.21569	-0.15049	-0.58476	-1.28917	0.553558	-0.67332	-1.90802	1.087838
P35475	IDUA	0.597979	-0.16156	0.202432	-0.60216	-0.44825	-0.88002	-0.03696	0.513663	-0.12212	0.571868	-0.16363	-0.55738	-0.55737	-0.42551	-0.63741	0.263814	-1.11482	-0.77436	-0.35018
P35520	CBS	2.288771	-1.65993	1.074983	-0.06282	1.914392	1.900213	-0.47087	0.563696	-0.90996	-1.94668	-1.87178	0.354085	0.769304	-0.89721	-1.97656	0.937162	0.638869	-1.77811	-1.3912
P35527	KRT9	-2.06045	4.362235	0.79209	-1.08611	-2.42958	-0.99388	3.022758	-0.14598	1.537533	-1.97544	-1.76403	-1.43721	0.223903	-0.34228	-1.18567	-0.07741	-0.62811	-1.54303	-0.25916
P35542	SAA4	-0.86815	0.804355	-0.79186	-2.66151	-0.30317	-0.99143	1.600891	0.329983	0.957804	0.36221	0.195839	1.382159	-0.25629	0.844959	-0.79168	-1.3487	0.413123	0.503413	-0.73959
P35555	FBN1	-3.0372	-1.67064	-0.49271	-2.47031	-3.40015	-2.85096	-0.86848	-2.73924	-0.88889	-1.39683	-0.87347	-2.84546	-1.87545	-1.44978	0.399901	-0.99561	-1.69851	-1.4824	-2.22142
P35556	FBN2	-1.65136	-1.1533	-2.07267	-2.49851	-2.24505	-3.31206	-1.81566	-0.47357	0.607798	-2.70447	-0.46298	-2.42831	-1.13411	-0.83638	-1.08758	-1.76293	-2.35637	-3.23482	0.098502
P35558	PCK1	-2.4068	-0.48527	-0.90367	-1.11462	2.803356	3.737429	-0.40027	-0.99429	0.320181	1.046298	-1.98702	-1.38083	-0.13477	-0.58312	-0.67934	-3.29104	-0.91631	-2.51555	-1.40086
P35573	AGL	0.697929	-1.22988	-0.59926	0.368264	0.069231	0.091998	-0.86299	-0.19906	0.080677	-0.22622	-0.05212	-0.1956	0.257026	0.112898	0.055584	0.15548	-0.34861	-0.95593	-0.40658
P35579	MYH9	-0.78235	0.480358	-0.00058	-0.11391	-0.14912	0.160279	-0.08287	-0.74321	0.481132	-0.23218	-0.62613	-0.22558	0.450424	0.229318	-0.8754	0.541119	-0.56219	-0.12976	0.72823
P35580	MYH10	0.319262	-0.29399	-0.5997	-0.9126	-1.23417	-0.78793	-0.35816	-0.83707	0.630659	-0.95588	0.045603	-0.9899	-0.02171	-1.17624	-1.0957	0.066112	-1.122	-0.54743	1.272458
P35606	COPB2	-0.56099	0.306781	0.206446	0.345682	-0.08774	-0.10303	0.229862	0.008013	0.169337	0.07021	-0.27371	0.027596	0.430126	0.084551	0.763033	0.436627	0.457051	0.416082	0.733623
P35609	ACTN2	0.114207	-1.21871	-0.44694	-0.21007	-0.4109	0.813752	0.043396	1.154245	-0.18245	-0.98712	-0.06227	-0.79738	0.231385	-1.16624	0.038483	-0.70221	-1.55461	-1.31966	1.762342
P35610	SOAT1	-0.67122	0.634686	0.263465	0.202033	0.575454	0.509705	0.325882	-0.85533	0.223641	0.274597	-0.35457	1.555131	-0.52466	0.864731	-0.07415	1.061217	0.342497	0.223453	-0.09204
P35611	ADD1	0.12738	0.041748	0.347651	-1.00848	-0.55199	-0.38598	0.015714	0.150068	0.102743	-0.26455	-0.39997	-1.2022	-0.1666	-0.65401	-0.36273	-0.92318	-0.79741	0.44209	0.338257
P35612	ADD2	-0.77611	0.871464	0.536723	-1.42204	-0.49183	-1.23597	0.335892	0.768541	0.577108	-1.11442	-1.01422	-0.55944	1.089393	-0.73799	-1.4181	-1.2608	-0.93531	1.206595	1.818503
P35613	BSG	0.073489	-0.29436	0.202734	-0.44991	-0.07651	0.259895	-0.05839	0.624689	0.254288	0.955808	-0.6273	0.176455	0.605348	0.213409	-0.42085	-0.54291	0.457605	0.477506	0.81963
P35625	TIMP3	-2.82374	-1.59385	-0.759	-3.19515	-1.71136	-3.00122	-0.11501	-2.97934	-0.06674	-2.70289	-2.51288	-0.89964	-2.35419	-0.83176	3.460929	-2.02607	-1.08301	-0.86673	-3.15659
P35637	FUS	1.227406	-0.46679	0.314142	0.212464	0.19337	0.667673	-1.40128	0.959037	-1.01584	-0.74246	0.151894	-0.93415	-0.3776	-0.569	0.504696	-0.1846	0.005509	0.021064	-0.21315
P35658	NUP214	0.142806	0.190878	-0.14172	0.00126	-0.20257	0.005645	0.212318	0.216518	-0.22401	-0.10729	0.218414	-0.2354	0.113125	-0.28475	1.126604	-0.19276	-0.24148	-0.00439	0.20888
P35659	DEK	1.49097	-0.99425	-0.07395	0.959941	0.240735	0.869759	-0.69558	-0.06841	-0.82677	-0.53456	-0.10994	-0.56629	-0.09374	-0.62033	0.796509	0.134057	-0.81523	-0.39233	-0.46494
P35749	MYH11	-3.39021	-1.92367	-1.19744	-3.28624	-1.91536	-3.26133	-0.4068	-4.2251	-0.61318	-3.15989	-3.30115	-1.16704	-2.29361	-0.73753	-0.54681	-2.70688	-2.42749	-3.48715	-3.9794
P35813	PPM1A	0.660795	-0.88985	-0.20176	0.364612	0.513126	-0.24238	-0.4182	0.060902	0.206124	1.192264	0.033407	-0.58346	-0.1146	0.33859	0.530632	0.018179	-0.02318	0.215258	0.328475
P35858	IGFALS	-1.64715	-0.08516	-0.50795	-1.03606	-1.10484	-1.72886	-0.20061	-0.54581	-0.19169	-1.41954	-0.94633	0.506541	-0.73425	0.518244	-1.04503	-1.16443	0.108822	-0.20555	-1.02375
P35869	AHR	0.010656	0.600262	0.175172	-0.33161	-0.18199	-0.4816	0.1735	-1.52055	0.35755	1.867	0.196906	-1.58646	-0.12611	-1.25346	-0.64871	1.316292	-0.44833	1.268219	0.734155
P35908	KRT2	-2.71276	3.590312	0.972271	-1.70315	-2.32927	-1.10299	2.812857	-0.56997	1.203982	-2.16245	0.635146	-1.75932	0.139273	-0.28741	-0.56719	-1.21321	-0.86143	-0.9136	-0.1557
P35914	HMGCL	1.103063	-0.85419	0.680626	0.214967	2.500435	1.068183	-0.59623	0.701081	0.565081	1.585999	0.918841	0.968205	0.363093	0.780706	0.775971	0.067166	-0.17825	0.427918	-0.04877
P35998	PSMC2	-0.07073	0.191497	0.69571	-0.02434	-0.01735	0.418524	0.211002	0.143303	0.149649	0.239808	0.14348	0.279387	0.076677	0.232605	0.064137	0.286608	0.267838	-0.07084	0.216252
P36222	CHI3L1	-1.08928	0.208592	0.669838	-1.33441	-0.32081	-0.80649	0.155197	-1.56154	0.547706	2.071151	-1.24766	0.203695	-0.76246	1.256962	-0.18478	-1.65732	-0.29115	-2.56486	-1.43083
P36269	GGT5	-1.32903	-0.73822	0.364793	-2.061	-1.13023	-1.95557	0.793179	-1.34211	-0.0884	0.200275	-1.41227	-1.39468	-1.05729	-1.23931	0.434464	-0.82371	-1.36907	0.863153	-0.99465
P36404	ARL2	0.918455	-0.38205	0.023603	0.323573	0.670261	-0.15742	-1.43558	-0.49178	-0.19182	0.086041	-0.77274	0.523773	-0.32139	0.276217	-0.12353	0.86657	0.465676	-0.51567	-0.04503
P36405	ARL3	0.804545	-1.318	-0.95068	-0.2865	0.996922	-0.21695	-0.95893	-0.40833	1.019807	0.31426	-0.29198	0.323882	-0.45271	0.317878	-0.08687	0.066366	1.014357	0.632306	0.436042
P36507	MAP2K2	-0.48796	0.561697	-0.16578	-0.40083	-0.06567	0.072058	-0.31926	-0.15486	0.047173	0.487499	0.305087	0.26872	-0.05324	0.778059	-0.18056	-0.36348	0.256806	-0.42321	0.125449
P36542	ATP5C1	-0.13761	0.028452	0.114447	0.581734	0.133546	0.037825	0.585249	-0.44866	-0.23156	0.346952	0.406899	0.116125	-0.21218	0.189213	1.347793	1.170634	-0.50072	-0.41297	-0.40069
P36543	ATP6V1E1	-0.69121	0.484704	0.096869	-0.41141	-0.10886	-0.20726	-0.34801	-1.14348	-0.16504	0.39444	-0.20436	0.529535	0.113783	0.463621	0.49178	-0.0706	0.038151	-0.37107	-0.55193
P36551	CPOX	0.142046	-0.38018	0.291148	0.710021	0.754573	0.342124	-0.54423	0.490017	1.39392	2.193693	-0.19336	-0.21126	0.434742	-0.29838	1.547913	0.292107	0.519421	0.331061	-0.27341
P36578	RPL4	0.372838	-0.10494	0.129207	0.59291	-0.16076	0.071435	-0.09707	0.056628	-0.22989	-0.6242	0.450456	-0.80172	-0.2013	-0.45266	0.016551	-0.21414	-0.23413	0.058654	-0.03012
P36776	LONP1	-0.01498	0.400232	-0.25948	-0.00189	-0.4022	-0.27574	-0.23592	-0.16088	-0.22193	0.320406	0.690782	0.30292	0.078523	-0.01611	0.077009	0.517681	-0.18498	-0.47052	-0.68184
P36871	PGM1	0.298454	-0.52453	-0.58391	-0.40165	1.025578	-0.12537	0.336137	-0.17239	0.696947	-0.10175	-0.56329	-0.23908	1.081177	0.391817	-1.45557	0.311036	0.713703	0.375269	2.098262
P36873	PPP1CC	0.759368	-1.59277	0.287733	-0.24608	0.346542	-0.04127	-0.34394	0.583145	-1.2568	-0.12099	-0.66984	-0.0572	-0.41762	-0.34917	0.378127	-0.2568	0.462679	0.562341	0.134953
P36915	GNL1	0.532381	-0.00759	-0.69389	0.006748	0.020627	0.647139	-0.36178	-0.52075	-0.58742	-0.30592	0.002741	-0.2228	0.116046	-0.21833	0.089776	0.138855	0.51383	-0.0577	-0.015
P36952	SERPINB5	-1.34513	1.207799	-0.13455	-0.58788	-2.23884	-1.1955	1.347215	1.347163	0.712325	-0.90845	-1.88231	0.240328	-0.04545	-0.77481	-2.41215	-1.34492	-0.63521	-2.3752	0.240707
P36955	SERPINF1	-1.9231	-0.31282	-0.6328	-1.88101	-1.15494	-1.14753	-0.43016	-1.01603	1.038479	-0.89537	-0.76706	-0.83329	-0.23077	-0.30178	-0.53844	-0.20672	-0.85211	-0.00502	0.437312
P36957	DLST	-0.25596	-0.10754	0.170165	0.257576	0.295041	0.150938	-0.04023	0.306218	-0.27381	-0.0915	0.366906	0.390038	0.082222	-0.08349	1.078542	1.08563	-0.1663	-0.05578	-0.14801
P36959	GMPR	1.159801	-0.39254	-0.6679	-0.56349	0.261923	-0.24795	-0.58283	0.007488	0.527026	-1.02423	0.733512	-0.34085	0.493368	-0.7645	-1.19568	1.38525	-1.09178	-0.49708	-0.97807
P36969	GPX4	-0.8361	-0.44843	0.25851	-0.8455	0.221046	-0.43963	-0.82412	-0.28012	-0.05905	0.940277	-0.52023	-0.32483	-0.47714	-0.19045	-0.51745	-0.44429	-1.02088	-0.22733	-0.15736
P37108	SRP14	0.090058	-0.04413	-0.11848	-0.3942	-1.04615	-0.58729	-0.57569	0.058279	0.093701	-0.38063	-0.1818	0.292071	-0.04723	0.230228	-0.03615	-0.22387	0.843418	0.579423	-0.07543
P37198	NUP62	0.149311	0.326821	-0.02154	0.054276	0.041326	0.12347	0.004689	0.298323	-0.52487	-0.271	0.303538	0.117025	-0.17048	0.021691	0.910418	-0.12406	-0.00894	-0.17581	0.256991
P37235	HPCAL1	-0.48215	-0.72199	-0.13437	0.138227	0.275036	-0.18467	-0.59881	0.16612	0.784948	1.062445	0.198312	0.766207	0.299262	0.708095	0.341656	0.283194	-0.20017	-0.10295	-0.66544
P37268	FDFT1	0.367674	-0.28023	-0.60959	-0.40036	1.110569	1.507528	0.104264	-0.40973	-0.41956	-1.4813	-0.16253	-0.57386	-1.43699	-0.23108	-1.05878	-0.95764	-0.6074	-0.72885	-0.02611
P37275	ZEB1	-0.26816	0.287309	0.01345	-0.15632	-0.47909	-0.76328	-0.23675	-0.50537	0.235129	0.399641	-0.15866	-0.83461	0.168409	-0.05397	0.132313	0.348079	0.131868	0.253952	0.238389
P37802	TAGLN2	-0.31916	0.288	0.280521	-0.37504	0.050659	0.208098	-0.38918	0.601741	0.355005	0.645361	-0.78004	-0.65566	0.573329	-0.61301	0.205755	-0.00188	-0.30232	-0.49298	0.59913
P37837	TALDO1	-0.90245	-0.03922	-0.30924	-1.01698	1.850533	1.392349	-0.97314	0.991363	-0.00361	0.326309	0.172488	-0.80232	-0.20154	0.291814	0.209461	0.424393	-0.04238	0.130629	-0.85118
P37840	SNCA	-0.85785	0.720172	-1.20634	-2.1449	-0.01429	-1.75055	-0.55127	-1.08819	0.025278	-1.29947	-0.67127	-0.81031	-0.96511	-0.97527	-1.70345	-2.20067	-0.32905	-0.49117	-1.80309
P38117	ETFB	0.223723	-0.22656	-0.42316	1.116202	1.794115	0.34568	-0.23153	-0.07733	-0.07488	0.695713	0.309809	0.24592	0.24939	0.166405	0.008736	0.072124	-0.05291	0.278421	-0.6993
P38159	RBMX	0.630377	-0.05521	-0.02939	0.111842	-0.14862	0.512407	-0.04023	0.770544	-0.28043	-0.45351	0.554885	-1.53287	-0.42992	-1.19409	-0.03988	-0.39155	-0.97487	0.215548	0.016774
P38432	COIL	0.11302	-0.11116	-0.23752	0.747643	-0.72045	0.29405	-0.23387	0.203977	-0.39592	-0.84639	0.333768	-0.40374	-0.19074	-0.93067	-0.39821	0.471047	-0.0335	-0.41224	0.133274
P38435	GGCX	-0.29792	0.290802	0.25554	-0.2848	-0.27859	0.228411	-0.267	0.371304	-0.14912	0.301896	-0.59278	-0.12155	-0.37472	-0.43698	1.212947	0.137639	0.65879	0.252269	-0.18326
P38571	LIPA	-0.22307	0.437081	0.033192	0.329729	0.289034	-0.65823	0.116748	-0.78992	0.007266	-0.52347	0.010872	-1.04946	-0.14804	0.205365	0.183722	0.260347	-0.04258	0.019963	-1.56593

P38606	ATP6V1A	-0.65514	0.586877	0.011899	-0.3758	-0.21123	-0.066	-0.28409	-0.84874	0.098308	0.345187	-0.36587	0.829203	0.103118	0.68685	0.600372	-0.07596	0.118586	-0.47618	-0.64932
P38646	HSPA9	-0.50067	-0.40356	0.318073	0.861251	0.35364	0.211999	-0.63495	-0.15213	-0.59428	0.44091	0.160416	0.270965	7.98E-05	0.149796	1.614292	1.75394	1.151522	1.053768	-0.80984
P38919	EIF4A3	0.504828	-0.224	-0.17309	-0.05914	0.183299	0.192344	-0.56317	0.186414	-0.59803	-0.04004	0.21576	0.038177	-0.33078	0.123536	-0.09849	0.067988	0.460678	0.115422	-0.08829
P38935	IGHMBP2	1.07319	-0.12959	-0.14995	0.518891	-0.01376	-0.09147	-0.67892	-0.04229	-0.11649	0.033406	-0.34648	-0.59529	0.363953	-0.27338	0.181907	0.539707	0.043941	-0.51901	-0.66381
P39019	RPS19	0.49131	-0.69041	-0.4652	0.046336	0.154012	0.332117	-0.34012	0.12433	0.508069	0.305153	0.806968	-0.44205	-0.03508	-0.33711	0.040303	0.167139	-0.48128	-0.55754	-0.70486
P39023	RPL3	0.337099	-0.19603	0.20029	0.511405	0.015184	0.286579	-0.04826	0.143537	-0.45739	-0.73509	0.532897	-0.12405	0.025234	-0.33257	0.1713	0.053845	-0.17495	0.019977	-0.19782
P39059	COL15A1	-1.56111	0.861598	0.740156	-2.27007	0.170912	-0.50358	0.712891	-1.60795	1.079464	1.008211	-0.25525	-1.4747	-0.46902	-0.56218	-0.5126	-0.37784	-0.90956	-0.76763	-1.57231
P39060	COL18A1	-0.15224	0.20447	-0.51797	-0.8411	-0.31399	-0.83121	0.641632	0.142103	0.206587	-0.88897	0.197792	-0.92719	-0.60751	-0.83644	0.392935	-0.42226	-0.14851	1.283024	-0.2385
P39656	DDOST	0.054475	0.396612	0.109698	-0.17228	-0.13099	-0.08247	0.726281	-0.18719	0.395766	0.024986	0.170152	0.272789	0.363905	0.06318	0.475247	-0.13845	0.126336	0.377441	0.073183
P39687	ANP32A	0.820425	-1.02907	-0.71554	0.600072	0.524278	0.36941	-1.34822	0.864269	-0.76456	0.04974	0.264282	-0.69732	-0.25528	-0.61676	0.135075	0.447893	0.092177	-0.04742	-0.23356
P39748	FEN1	1.322268	-0.36756	0.503562	0.614144	0.62377	1.038434	0.002165	0.836683	-0.42769	-0.50349	-0.07857	-0.06575	0.12315	0.066386	-0.29706	0.152676	0.530133	0.453947	-0.73848
P39880	CUX1	0.072275	0.454985	-0.07047	0.150888	0.355758	0.651112	0.262324	0.415609	-0.05055	-0.20258	-0.1603	-0.40454	0.188802	0.029586	0.428357	0.349377	0.034658	0.125917	0.933523
P40121	CAPG	-0.53421	-1.44588	0.092804	0.507319	1.0467	0.49516	-1.28904	0.150584	0.161849	1.423821	-2.24185	-0.05093	0.598364	0.187829	1.036365	0.459545	-0.1313	-0.52848	-0.49819
P40123	CAP2	0.650741	-0.65341	-0.51841	0.67472	-1.1125	-0.57751	-0.40403	-0.59935	0.224872	-1.34279	-0.60175	-1.36117	-0.24822	-0.63864	-0.15801	-1.28556	-1.05989	-0.7711	0.868165
P40189	IL6ST	-0.28548	0.184853	0.340106	-0.69685	0.048495	-0.23186	0.167878	-0.81244	0.120623	0.679556	0.311869	-0.21878	-0.12454	0.147512	0.095608	-0.00671	-0.20015	0.017098	-0.71073
P40199	CEACAM6	-0.26143	1.089837	2.150264	-0.67715	-0.60819	0.016638	0.383919	-0.7539	-0.0082	-0.4615	-0.54672	0.154167	0.2817	1.206763	-0.78166	-0.43569	1.002473	-0.02049	-1.52047
P40222	TXLNA	0.218255	0.090724	0.886124	0.741663	1.084268	1.265772	-0.08687	0.514305	0.126886	0.236828	-0.09313	-0.78423	0.396088	-0.76382	-0.34519	0.349637	-0.19467	0.199419	0.425641
P40227	CCT6A	-0.31922	-0.11764	0.092641	0.087073	-0.25856	0.358864	-0.63647	-0.62636	-0.0292	-0.10069	0.06924	-0.10973	-0.20023	-0.15967	0.070221	-0.04931	-0.14248	-0.47484	0.031756
P40261	NNMT	-1.66625	0.533156	0.038374	-1.89159	0.069236	0.248281	0.300295	-0.42664	0.939505	1.037985	-1.91283	0.12479	1.451073	-0.11852	-0.95032	1.735771	0.914458	0.965128	2.126667
P40306	PSMB10	-0.70055	0.312736	-0.17151	-0.5311	0.73009	0.429353	0.285101	-0.79636	1.146459	0.528196	0.043683	-0.0127	0.117446	0.051704	-0.85118	0.547291	-0.12999	0.073313	-0.67932
P40424	PBX1	0.484171	-0.46589	-0.40012	0.087084	-0.43183	-0.05118	0.13492	0.359804	-0.27663	-0.91417	0.62619	-1.00537	-0.7186	-0.74828	0.483042	-0.14806	-1.07272	-0.88714	-0.10573
P40425	PBX2	0.512625	0.038796	-0.25477	-0.04688	-0.17416	-0.22595	-0.45188	-0.68294	-0.6152	-1.02245	0.757168	-0.6868	-0.2802	-0.53595	-0.00531	-0.53685	-0.19229	0.671763	0.841519
P40426	PBX3	0.917744	-0.71929	-1.11862	0.345971	-0.43906	0.208452	-0.13457	0.912609	-0.62353	-0.69589	0.981936	-1.55977	-0.50669	-0.94355	0.496989	-0.21612	-1.67103	-1.07192	-1.18123
P40429	RPL13A	0.420322	-0.22147	-0.20655	0.399835	-0.0416	-0.18836	-0.01118	-0.06002	-0.13394	-1.01829	0.55474	0.197166	-0.06936	-0.08866	0.150731	-0.3038	0.272951	0.05237	-0.24609
P40616	ARL1	-0.25062	-0.38283	0.428073	0.235918	1.055465	0.196866	-0.59937	0.196049	1.394236	0.333804	0.074705	0.896102	0.5544	0.592207	1.184018	0.604746	1.071124	0.783842	0.646069
P40692	MLH1	1.03509	-0.13245	0.702017	0.211595	0.141924	0.487131	0.037695	0.22347	-0.3903	-0.02057	-0.02453	-0.27149	-0.18029	-0.08086	0.09211	0.825631	-0.22859	0.002296	0.038355
P40763	STAT3	-0.46763	0.164714	0.783415	0.635982	0.070831	0.427668	0.143561	-0.66875	0.392364	0.056959	0.073728	0.470257	0.535325	0.800708	-0.07243	1.356747	0.721757	0.105037	0.137247
P40818	USP8	0.277096	0.27183	0.632824	0.405225	-0.38694	0.353612	-0.3364	-0.57662	-0.26744	-0.10518	0.263022	-0.17686	-0.26956	-0.151	-0.05813	0.276273	0.267826	0.087892	0.064812
P40855	PEX19	0.225269	-0.09071	-0.22546	0.099033	0.049971	-0.06593	-0.56034	0.161037	0.146367	0.036317	-0.07248	-0.43797	0.052055	-0.00957	0.503384	-0.09022	0.172615	-0.20114	-0.2716
P40925	MDH1	-0.62389	-0.28602	-0.88045	-0.78002	0.632711	0.069112	-0.7807	0.690739	0.168952	0.023088	-0.01581	-0.87227	-0.10862	-0.77693	-0.28425	-0.03496	-0.91108	-0.42581	-0.06419
P40926	MDH2	0.247399	-1.24139	-0.72676	-0.0493	1.977297	-0.33351	-0.71011	0.058205	0.900852	1.700417	0.365166	0.255021	0.84699	-0.38228	1.254036	-0.39122	-0.68838	-0.21504	-1.27392
P40937	RFC5	1.292255	-0.01308	0.041007	0.489698	0.752338	0.86379	-0.08073	0.430449	0.370918	-0.22906	-0.00619	-0.45139	-0.20049	-0.08229	-0.06503	0.067769	-0.06692	-0.41032	-0.40696
P40938	RFC3	0.755716	-0.47318	0.352951	0.017311	0.35245	0.789547	0.478817	0.406674	-0.83464	-0.05108	-0.11226	-0.19299	-0.97212	-0.11172	0.127553	-0.45438	0.235044	0.17058	-0.32632
P40939	HADHA	-0.34968	0.150338	0.233468	1.034382	0.274626	0.031654	0.015368	-0.08424	-0.10253	0.002379	-0.20742	0.418936	-0.41275	0.288361	0.952041	0.938494	-0.46032	-0.40063	-0.10972
P41091	EIF2S3	0.401331	-0.11044	0.087346	0.267156	0.018859	0.426588	-0.10768	0.338931	0.07138	0.153246	0.123128	-0.36098	-0.20521	-0.35117	0.456065	0.367632	-0.13215	-0.02925	0.077977
P41208	CETN2	0.607689	0.44127	-0.42679	-0.22851	1.334743	0.875159	-0.84278	0.289559	-0.14485	-1.32808	1.821792	-0.85738	0.862871	-0.68487	-0.38934	0.143236	-0.15757	0.457267	-0.95246
P41212	ETV6	-0.11396	-0.04662	0.227374	0.101642	-0.30202	-0.49687	-0.03169	0.538111	-0.91338	-0.20801	0.151291	0.020495	-0.24308	0.178478	0.207281	0.428919	-0.21145	-0.95629	-0.07338
P41214	EIF2D	-0.34572	-0.0075	-0.76118	-0.43645	-0.20395	-0.35461	-0.02017	-0.37424	0.962471	0.293525	0.575997	-0.18944	-0.20235	-0.20894	0.770633	-0.46173	-0.40969	-0.44533	-0.45806
P41218	MNDA	-2.36979	0.927797	1.129404	-1.68699	-0.02013	-0.58347	-0.6832	-2.01487	0.044791	2.05627	-1.77096	2.305609	-0.64321	3.257769	-0.54606	-1.26042	1.572109	-0.72271	-1.54291
P41219	PRPH	-2.18555	-1.88896	-2.4344	-1.38246	-0.30406	-1.80227	-1.93322	-1.27597	0.683138	-2.26724	-2.52155	-0.5096	-2.07464	-0.89756	-0.67619	-2.36067	-0.98059	-2.33351	3.084092
P41223	BUD31	0.912858	-0.44282	-0.24105	-0.03622	0.603491	-1.12987	-0.56976	0.558334	-0.04222	0.195208	0.474172	0.002136	0.093315	0.336256	0.708121	0.76238	0.302517	-1.16764	-0.12318
P41226	UBA7	0.334194	0.33245	0.944106	-0.39371	-0.79978	-0.26569	0.190832	-0.22434	-0.07622	0.328713	-1.03305	0.355585	0.045877	0.519385	-0.55743	-0.0662	-0.13306	-0.10809	-0.36374
P41227	NAA10	-0.03271	0.279516	0.079533	0.514134	0.184571	0.860407	-0.18479	0.23486	-0.31304	0.41274	0.228053	-0.53013	0.255671	-0.35675	-0.63194	0.352351	0.196624	-0.16908	0.235783
P41229	KDM5C	0.95737	-0.16274	0.326946	-0.10162	-0.47522	-0.50622	0.417187	0.626253	-0.1456	-0.43028	0.491979	-0.06755	-0.36874	-0.05229	-0.82022	0.394245	0.186245	-0.15372	-0.07566
P41236	PPP1R2	-0.23248	-0.06277	-0.13098	-0.9179	0.130016	-0.55423	0.175089	0.269123	-0.33674	-0.24805	-0.22214	-0.21184	-0.11755	-0.56577	1.315428	-0.44065	-0.17237	-0.40965	-0.48951
P41240	CSK	-0.56049	0.804328	0.348303	-0.68943	0.208869	0.103437	-0.46737	-0.66252	-0.18817	0.694644	-0.55689	0.94118	-0.15411	1.243384	-0.3896	-0.66966	0.557398	0.056732	-0.15014
P41250	GARS	0.655987	-0.54897	0.531001	-0.49663	0.136003	0.219258	-0.79052	0.995196	0.081754	0.050349	-0.54451	0.079663	0.996724	-0.42066	-0.04304	0.997617	0.864644	0.281239	1.027142
P41252	IARS	-0.16018	0.13472	-0.1044	0.434109	-0.01282	0.088057	0.084864	0.255299	-0.59742	-0.45467	0.314491	-0.35385	-0.14904	-0.07903	0.124401	1.042064	-0.28235	-0.589	0.173301
P41743	PRKCI	0.764884	-0.90262	0.168532	0.916238	0.187352	0.346039	-1.01539	-0.13055	-0.56395	-1.06308	-0.06677	-0.30488	0.029171	0.361427	1.160466	0.280227	0.281726	-0.2906	-0.86563
P42025	ACTR1B	0.517589	-0.1807	0.116833	-0.21274	-0.126	0.234526	-0.50176	0.131375	-0.22165	-0.34552	0.258735	-0.21537	-0.11654	0.155138	-0.16704	-0.16955	-0.59393	-0.6488	-0.0359
P42126	ECI1	1.424948	-1.71134	-0.46258	0.021796	1.327203	1.175534	-0.73005	0.120309	-0.57855	-0.31384	0.50677	-0.76337	-0.49159	-0.44966	0.784516	1.979576	-0.45268	0.24946	-1.1398
P42166	TMPO	0.802449	-0.3956	-0.39632	0.030092	0.3556	0.791664	0.46463	1.325098	0.049343	-0.87334	0.27419	-0.11597	0.635343	-0.32654	-0.89211	-0.71731	0.217388	0.420195	-0.4237
P42167	TMPO	0.631334	-0.54236	-0.43607	0.497913	-0.61335	0.008424	-0.10423	0.00398	-0.56856	-0.84591	-0.11852	-0.85284	0.029603	-0.46696	0.08982	-0.71242	-0.72601	-0.11063	0.00099
P42224	STAT1	0.285644	0.8991	1.509793	-0.65885	0.047707	0.34417	-0.00754	0.692691	-0.41551	-0.69739	-1.19863	0.58245	0.5056	0.652394	-0.69693	-0.76545	0.649271	0.184315	-1.00708
P42226	STAT6	-0.01851	0.381476	0.757466	-0.5166	-0.25335	-0.18206	0.086172	-0.19754	1.160625	0.138622	-0.19585	0.468776	-0.41971	0.46753	-0.30628	-0.55659	0.3364	-0.333	-0.01757
P42229	STAT5A	-0.74959	0.390881	0.891664	0.177934	-0.20068	-0.251	0.337088	-1.23046	-0.27367	0.39801	-0.50941	0.480039	-0.60502	0.980112	-0.17433	0.130842	0.110242	0.168631	-0.34353
P42285	MTREX	0.490469	0.147528	-0.09599	0.297009	0.214239	0.263781	-0.31685	0.378901	-0.45091	-0.38603	0.398663	-0.08921	-0.05178	-0.3221	0.397118	-0.1782	-0.09402	0.065895	0.083039
P42330	AKR1C3	-1.73452	-0.58281	-0.45115	-0.07779	-0.84259	-1.41446	0.296196	-1.58813	0.350892	-1.69059	-0.62745	-0.58996	-0.96776	-0.82572	0.178641	-1.43101	0.99431	-0.28437	0.016991
P42331	ARHGAP2	-2.09704	0.683384	0.956854	-1.26444	0.12333	-0.34761	-0.26428	-1.97319	-0.08703	1.038048	-0.9681	0.841637	-0.48064	1.764319	-0.60733	-1.13029	0.024225	0.429131	-1.57495
P42336	PIK3CA	-0.3184	0.181226	0.325685	-0.19445	0.008548	-0.47125	-0.35653	-0.37873	0.409458	-0.04383	-0.50174	-0.49748	0.049194	0.079339	0.271602	0.119404	0.23795	-0.52662	0.110352
P42338	PIK3CB	-0.40261	0.199732	0.252452	0.262647	-0.40651	-0.33973	0.387137	-0.07524	0.212146	0.386091	-0.26839	0.229828	0.123637	0.811741	1.034373	-0.17894	0.539713	-0.24518	-0.47314
P42345	MTOR	0.324323	0.287262	0.119027	-0.17852	-0.22842	-0.12159	0.152125	0.078648	0.518248	0.218086	0.17209	-0.02314	-0.3125	0.022152	0.103899	0.303796	-0.1654	-0.38576	0.063541
P42356	PI4KA	-0.21044	0.618598	0.374712	-0.40272	0.099554	-0.12034	0.655782	-0.63031	-0.43602	0.011087	-0.31313	0.073011	-0.10591	0.104691	-0.06642	0.117423	0.008552	0.690979	-0.00116
P42566	EPS15	0.046145	0.631635	1.011099	-0.21786	0.376017	0.135292	0.148179	-0.3656	0.012938	0.32616	-0.39394	0.243546	0.011838	0.141647	0.409508	0.370165	-0.32945	0.099746	-0.1669
P42574	CASP3	-0.40516	-0.40254	0.956856	0.008711	1.42534	0.906649	0.119271	0.230994	0.067332	0.318677	-0.19736	-0.00384	0.398882	0.450746	-0.97557	0.257495	0.430951	0.270274	1.026025
P42575	CASP2	0.710237	-0.23115	0.059572	0.300691	-0.02285	0.474446	-0.02485	0.420512	-0.22165	-0.27046	-0.29175	-0.03975	0.050693	-0.23636	0.404548	0.2741	-0.06998	-1.01135	0.024533
P42677	RPS27	0.266345	0.159146	0.42672	0.079292	0.248938	-0.18432	-0.07784	0.230364	0.724021	0.202049	0.186307	0.270379	-0.15509	0.343532	-0.63716	-0.03303	0.869775	-0.24124	-0.32021
P42684	ABL2	-0.21748	0.589322	-0.01319	-0.36401	-0.24277	-0.38656	0.388215	0.275864	0.230385	-0.06456	-0.64085	0.190902	-0.09312	0.164415	0.10151	-0.34759	0.029423	-0.39652	-0.26041
P42685	FRK	0.028218	0.696826	-0.09402	0.789057	1.389987	1.927063	-0.24462	-0.61684	0.064045	0.147622	0.137781	-0.02106	0.519679	-0.34729	1.78928	0.512188	0.00039	-0.46984	-0.61539
P42694	HELZ	0.065771	0.524559	-0.61317	-0.25304	-0.13272	0.09579	0.509695	-0.34999	0.414227	0.330352	-0.1468	0.200025	-0.13783	0.417928	-0.14022	0.739446	0.564268	0.120787	-0.37944
P42695	NCAPD3	0.320846	-0.26082	0.014659	0.508403	1.106624	0.783343	-0.13063	0.552338	-0.23731	0.280907	-0.36644	-0.11848	-0.00562	-0.09475	0.043187	0.40061	-0.06784	0.147892	-0.31778
P42696	RBM34	-0.01512	0.123353	-0.7843	1.171792	0.001062	0.186516	-0.96165	0.393636	0.029484	-0.49068	0.35574	-0.41767	-0.32834	-0.53346	-0.26107	-0.57612	0.243566	-0.084	-0.45507
P42704	LRPPRC	-0.10749	-0.17694	-0.34442	1.159261	-0.00343	0.265725	-0.13977	0.28904	-0.34119	0.720535	0.470524	0.188193	-0.09751	-0.29407	0.821593	-0.01638	0.077601	-0.43305	-0.10601
P42765	ACAA2	-0.85037	1.25403	0.578159	0.094738	0.658891	0.177189	0.365082	-0.3698	0.473844	0.719585	-0.50081	0.000479	0.391192	-0.10695	0.076181	-0.66186	-0.4851	0.389364	0.029783
P42766	RPL35	0.272251	-0.64557	0.359195	0.466614	-0.62864	0.697325	-0.49314	0.289868	0.189199	-0.4119	0.233015	-0.55893	0.204163	-0.12298	0.811334	0.909538	0.157914	-0.84521	0.065945
P42768	WAS	-1.65052	0.309259	0.343122	-0.71361	0.339653	0.266043	0.019756	-1.43626	0.270056	1.404572	-1.04648	0.494358	0.295204	0.986673	-0.02806	-0.02549	-0.08168	0.415119	-0.87948
P42771	CDKN2A	1.207742	-0.31637	0.738561	0.754933	0.54627	0.843886	0.197523	0.815724	0.990387	-0.18874	-0.62422	-0.07923	-0.64228	0.006433	-0.24815	0.856526	-0.48392	-0.65791	0.93929
P42773	CDKN2C	-0.86218	0.283601	-0.07353	-1.93674	0.739367	0.98606	0.337809	-1.51963	0.205688	-0.58494	-1.42217	-0.77039	-0.19939	-0.6916	0.001342	-0.56942	-1.19541	0.082887	-0.88038
P42785	PRCP	0.357093	0.092021	0.064496	-0.29088	0.112567	0.031555	-0.9089	0.201682	-0.19181	0.151309	-0.35683	-0.40664	0.267707	0.327694	0.044185	0.146378	0.196861	0.441961	-0.12826
P42858	HTT	1.038028	0.120908	0.094279	-0.35432	-0.36492	-0.4596	0.350665	-0.55829	0.394646	0.158564	-0.59895	0.17438	-0.33358	0.246467	-0.45664	0.277094	-0.33863	-0.55996	-0.34811
P42892	ECE1	0.441566	0.050788	-0.0317	-0.43244	-0.01561	-0.31631	0.104294	1.836032	-0.27083	0.142377	-0.4883	-1.13306	-0.8378	-0.31788	-0.6531	0.424415	1.019439	0.680995	-0.02265
P42898	MTHFR	0.0284	0.034074	0.389352	-0.76543	0.101072	-0.29904	-0.02326	-0.45716	-0.02621	1.25428	0.036537	0.727448	-0.11561	1.228729	0.248779	-0.8634	0.790845	0.27444	-0.33896
P43003	SLC1A3	-0.45209	-0.31824	-0.46352	0.002432	-0.65568	-0.18065	-0.47266	-2.70089	0.016692	-0.37893	-0.20222	-2.04443	-0.18124	-0.22403	3.060181	0.249294	-1.85317	-1.60976	-1.6262
P43007	SLC1A4	0.260414	-0.79906	-0.35082	-1.45234	0.166019	-0.01739	1.997531	0.064993	0.040847	-0.0648	-0.69904	-0.76166	-0.91615	-0.89131	-0.53412	0.125186	-1.39148	-0.68022	-0.73155
P43034	PAFAH1B	1.356161	-1.17574	0.529918	-0.04715	0.631335	0.615013	-0.99052	0.496105	0.398038	0.202056	0.292207	-0.23879	1.196211	-0.02252	0.056342	0.222861	-0.09198	0.041129	0.640028
P43121	MCAM	0.404048	-0.13509	-0.86428	-1.16057	-0.94542	-1.30638	0.822585	0.634561	-0.53312	-0.28608	-1.45981	-0.88995	0.057557	-1.51523	-0.54245	-0.72998	-2.77175	-0.49948	-0.57408
P43155	CRAT	0.63367	-0.37694	0.121355	-0.96287	-0.00426	-0.20665	-0.20374	-1.58606	0.206828	0.50147	0.224383	-0.10585	0.263516	-0.41507	-0.80237	-0.08773	-0.88176	-0.02677	0.347136
P43243	MATR3	0.350471	0.019096	0.183025	0.104417	0.007675	0.250318	0.035862	0.519258	-0.49985	-0.37096	0.56542	-0.53687	-0.2672	-0.42409	0.168951	-0.19947	0.079051	0.715784	0.063357
P43246	MSH2	1.528687	-0.44631	0.395056	0.261043	0.416481	0.742023	0.559811	0.906656	-0.8476	-0.32319	-0.16641	-0.55078	-0.80194	-0.52037	-0.04774	0.581462	-0.67802	-1.02124	-0.46157
P43250	GRK6	-0.41148	0.562344	0.630847	-0.50779	0.754574	0.001936	0.173841	-0.64752	-0.10295	0.938976	-0.65133	0.77851	0.170254	1.438829	-0.17331	0.276767	0.572463	0.211067	-0.19754
P43251	BTD	-0.35462	0.107283	-0.8769	-1.36586	-0.07287	-0.87942	0.16503	-0.31446	0.322998	0.57446	-0.38122	0.053551	-0.28998	-0.25636	-0.66189	0.250317	-0.13136	-0.10521	-0.48591
P43304	GPD2	1.849844	0.000554	-0.63753	0.902765	-0.71591	-0.45762	-0.32415	0.169018	-0.83217	-0.55113	0.936457	0.040608	-0.60268	0.315116	0.349834	0.970014	-0.58125	-1.23742	-0.62847
P43307	SSR1	0.209123	0.519864	-0.16541	0.312474	0.221204	0.189838	0.809186	-0.8152	-0.22729	-0.22525	0.099086	0.168221	0.209245	-0.11826	0.833595	0.1536	-0.19236	0.277673	-0.02939
P43353	ALDH3B1	-1.14445	0.393426	0.16911	-2.77158	-0.06795	-0.86197	-0.03789	-1.25409	-0.68203	0.226153	0.776043	1.188925	-0.04557	1.505471	0.416179	1.377752	0.510584	-0.93256	-1.56395
P43358	MAGEA4	-2.75723	-0.70063	1.158409	-1.75481	-2.27219	-1.74095	-0.76639	-2.05101	0.753744	2.485667	-2.2845	-0.93278	-0.62255	-0.64612	-2.25637	-1.66978	0.417108	0.601821	1.91059
P43378	PTPN9	-0.02439	-0.1794	0.18201	-0.05426	0.658086	0.364642	0.140156	0.08629	-0.0768	0.006793	0.008004	0.166215	0.033798	0.441739	-0.24558	-0.0045	0.324925	0.057541	0.544626
P43403	ZAP70	-1.92364	1.004152	0.62887	-0.24047	1.613193	1.113942	0.111535	-2.1237	-0.05973	0.93126	-1.95083	-0.79643	-0.05549	-0.43695	-0.6886	-1.15682	-0.20397	1.392924	-0.83011
P43405	SYK	-0.18199	0.595805	0.004919	-0.71031	0.651656	0.521332	-0.13392	0.025161	-0.16472	0.946334	-0.79103	1.111907	-0.64356	1.541755	-0.10961	-1.2584	0.130717	-0.8898	-1.38027
P43487	RANBP1	1.510284	-1.82887	-0.30177	0.559896	1.070111	-0.27726	-1.43908	-0.42644	0.089791	1.12356	-0.3688	0.014305	0.098654	0.276204	-0.56157	0.940068	1.623749	0.766946	-0.17996
P43490	NAMPT	-0.00615	0.743241	2.027481	0.149682	0.216986	0.089811	0.538454	0.584624	0.348792	1.506366	-0.75998	0.952711	0.986356	1.483389	0.132715	1.117024	0.770394	-0.52357	0.900036
P43652	AFM	-0.73139	-0.52291	-1.28252	-1.53247	-0.61114	-1.516	0.75497	0.2583	1.039115	-0.81845	0.007834	0.204578	-0.31811	0.522257	-0.35298	-0.88228	-0.05231	0.412743	-0.87863
P43686	PSMC4	-0.08265	0.20221	0.559596	0.138925	-0.09783	0.349119	0.048932	0.274243	0.018299	0.163574	0.043221	0.163118	0.059413	0.132295	-0.13889	0.43224	0.17288	-0.09237	0.660094
P43897	TSFM	0.658994	-0.16291	0.001319	0.706087	1.334237	1.126943	-0.66875	0.27882	-0.44496	-0.05802	-0.11401	0.372369	0.418069	-0.24112	0.469012	0.915992	0.143616	-0.45554	-0.02308
P45877	PPIC	-0.88936	0.311609	0.3246	0.142646	1.158987	0.868406	0.197095	0.158185	0.226526	0.482719	-0.1143	-0.01399	0.584665	0.120473	-0.77275	0.479984	0.303625	0.613323	1.322321
P45880	VDAC2	-0.1329	-0.11748	-0.41318	0.153863	0.487947	0.661101	-0.29815	-0.29792	-0.60713	-0.33095	-0.06889	0.064118	-0.47805	0.178444	0.973564	1.475689	0.467068	0.023481	-0.6311
P45954	ACADSB	-0.9756	-0.24461	-0.06226	1.479399	-0.7024	-0.5965	-1.78177	-1.13766	-0.10138	-0.35205	0.384629	0.084002	-0.10669	0.20021	1.31493	1.84884	0.3464	0.322467	-0.99535
P45973	CBX5	1.327013	-1.42348	-0.33521	0.308818	-0.10336	0.952345	-0.48555	0.574781	-0.41273	0.116407	0.697911	-1.00276	-0.53842	-0.16102	0.74851	-0.62938	0.237671	0.1769	0.602882
P45974	USP5	0.163791	-0.48465	-0.66265	-0.26927	-0.78854	-0.50913	-0.68763	0.500748	-0.05499	0.207615	-0.36415	-0.12473	0.365952	-0.03281	-0.03767	0.841525	-0.16055	-0.66983	0.222406
P45984	MAPK9	-0.33337	0.027503	-0.728	0.276643	0.517144	0.914449	-0.44841	-0.53945	0.336495	-0.32251	0.213891	-0.15438	-0.14999	0.054622	-0.16944	0.231829	-0.00928	-0.39688	-0.286
P45985	MAP2K4	0.008549	0.463247	-0.06062	-1.06353	-0.31956	-0.08165	0.042679	-0.45228	-0.45221	0.111312	-0.0513	0.040274	0.056435	0.347513	-0.41963	-0.53318	-0.033	0.088139	0.057866
P46013	MKI67	1.004965	0.279349	-0.72712	0.243431	0.549774	1.496299	0.67269	1.769523	0.437816	-0.48556	-0.70957	-0.24195	1.071261	-0.68453	-0.66519	0.245077	-0.27587	-0.04329	-0.31181
P46019	PHKA2	-0.01079	0.49692	-0.37745	0.054936	-0.1634	0.055002	0.572011	-0.2932	-0.03014	-0.18343	-0.36543	0.033175	-0.58104	0.77567	-0.56563	0.089153	0.383094	-0.95883	-0.39372
P46020	PHKA1	-0.60088	-0.81837	-0.85786	-0.1929	-1.1619	-0.32311	-0.48101	-0.08327	-0.73432	-1.49194	0.889599	-0.74092	-0.49445	-0.56314	0.422276	-0.81103	-1.24353	-1.9939	-1.40372
P46060	RANGAP1	0.001358	0.158505	0.076791	0.087909	-0.06875	0.041386	0.126962	0.080834	-0.66661	-0.1304	0.160706	-0.23844	0.143229	-0.13049	0.559577	-0.07335	0.059123	0.141359	-0.1116
P46063	RECQL	0.436773	0.224153	2.348933	-0.80667	-0.01715	-0.06424	-0.32363	0.135306	-0.07046	0.248663	-0.29197	-0.08595	-0.11517	-0.13415	0.072147	0.758533	-0.16366	-0.05753	0.658302
P46087	NOP2	0.724405	-0.16146	-0.82773	0.295693	-0.49161	-0.23887	-0.45702	0.272817	-0.37677	-0.44145	0.151256	-0.14737	0.571713	-0.50864	-0.24579	0.300297	-0.20877	-0.06756	-0.38587
P46100	ATRX	0.529043	0.076264	0.299638	0.200411	-0.19935	0.223242	0.157717	0.233581	0.151138	-0.18798	0.428092	-0.85386	-0.38383	-0.35529	-0.18046	-0.16708	-0.33052	0.312786	0.134358
P46108	CRK	0.227223	0.071796	-1.49151	-0.79863	1.056923	-0.37185	-0.17214	0.478529	-0.04196	1.14208	-0.26996	-0.19431	0.258863	-0.30701	-0.48602	-0.23208	-0.12554	-0.08771	-0.19263
P46109	CRKL	0.602168	-0.60677	0.150088	-0.00562	0.399969	-0.34475	-0.78592	-0.1974	0.779892	0.644386	-0.00732	0.228949	0.120335	0.51345	-0.32911	-0.13241	0.731309	0.701199	-0.30713
P46199	MTIF2	-0.2116	-0.06085	0.058471	1.024942	-0.07841	-0.21267	1.002377	-0.00992	-0.37787	0.468013	0.516763	-0.0243	0.027109	-0.22765	1.03109	0.579201	-0.67402	-0.41563	-0.58948
P46379	BAG6	-0.40792	0.374185	0.089041	0.228671	-0.13008	0.419197	0.441837	0.285946	0.144416	0.00722	-0.19888	0.019633	-0.0905	0.025186	0.12456	0.440298	0.780172	-0.16202	0.076762
P46459	NSF	-1.01019	0.734862	0.356394	0.73052	-0.01167	0.148163	0.682428	-0.28976	-0.12556	0.177304	-0.29414	0.870693	0.229775	0.83444	0.231207	0.317681	0.357631	-0.10239	-0.0808
P46531	NOTCH1	-0.18944	0.402061	0.129026	-0.30176	-0.01181	0.431701	0.16521	0.105149	0.080621	0.218631	-0.50153	-0.36772	0.12991	0.522678	-0.2169	0.030761	0.445871	1.04215	0.065125

P46734	MAP2K3	-0.4405	-0.02977	0.144755	-0.32054	-0.37136	-0.41542	0.412794	0.004218	-0.33628	0.349164	0.282638	0.094089	-0.5312	0.817501	-0.18038	-0.70884	0.20428	-0.60646	-0.377
P46736	BRCC3	-0.57804	-0.05719	-0.0096	-0.21645	0.155272	-0.09464	-0.25832	0.029854	0.041328	-0.04346	0.419066	0.019588	-0.00318	-0.08461	-0.09319	0.748066	-0.23505	-0.28569	-0.28078
P46776	RPL27A	0.230931	-0.12657	-0.20184	0.173961	-0.07534	-0.11491	-0.52168	-0.11138	-0.16287	-0.28397	0.565499	-0.05178	0.061266	-0.17381	0.139694	0.070085	0.186579	-0.14214	-0.17244
P46777	RPL5	0.189561	-0.03028	0.108304	0.433808	-0.18311	0.23398	-0.18799	0.140859	-0.20173	-0.4024	0.268319	-0.12645	-0.0776	-0.45271	0.155415	-0.05178	0.042054	-0.15044	-0.08199
P46778	RPL21	0.622397	-0.19552	-0.07284	0.249437	-0.08716	-0.30339	0.317551	-0.0017	-0.23341	-0.5735	0.241044	0.174825	0.112646	0.051811	0.293205	0.139302	0.209109	0.01363	-0.04002
P46779	RPL28	0.084715	-0.16209	-0.35068	0.306933	-0.58753	-0.24817	0.5119	-0.21502	-0.19787	-0.41433	0.175143	0.600927	-0.33993	0.185435	-0.03609	0.021216	0.410334	-0.09907	-0.50729
P46781	RPS9	0.27268	-0.36377	-0.17055	0.420717	-0.03288	0.530926	-0.80073	0.059555	-0.31595	-0.37491	0.556839	0.14872	-0.1102	-0.04555	-0.09981	0.138916	-0.00546	-0.19675	0.102819
P46782	RPS5	0.300069	-0.0053	0.155318	0.373093	-0.07354	0.334877	-0.52123	0.132952	0.223435	-0.21931	0.168712	0.141706	-0.518	-0.08397	0.030646	0.183745	0.150003	-0.01303	-0.11163
P46783	RPS10	0.337876	-0.33181	0.018396	0.095018	-0.01859	0.258684	0.161137	0.267592	0.377917	0.087602	0.412121	-0.15506	0.016163	-0.17348	-0.09298	0.05752	0.045099	-0.1988	-0.41561
P46821	MAP1B	-1.46776	-0.0915	-0.74888	-1.9465	-1.29658	-1.58002	0.081463	0.365058	-0.10902	-0.16955	-0.59347	-1.40065	0.088015	-1.35139	-0.14558	-0.93786	-1.69899	-0.30546	1.11052
P46926	GNPDA1	-0.09317	-0.72732	0.140477	-0.06181	0.848226	0.416954	-0.37151	0.55448	0.343704	0.728713	0.397934	-0.14808	0.122912	-0.15924	0.452925	0.197354	-0.49624	0.007589	-0.21288
P46934	NEDD4	-0.41088	0.699362	-0.04389	-0.28905	-0.1794	-0.42486	0.250817	-0.69337	0.173094	0.812625	-0.75436	-0.22681	-0.07412	0.603707	-0.79354	0.096861	0.388158	0.94059	1.052194
P46937	YAP1	1.055444	-0.3596	-0.95868	-0.39734	-1.25055	-0.72963	-0.97622	0.103166	0.724395	-0.44305	0.096756	-1.01087	0.41313	-0.77781	-0.38119	0.418958	-0.12564	-0.1162	-0.1368
P46939	UTRN	-0.0311	0.104759	-0.05389	-0.43261	-0.26486	-0.54843	-0.15974	-0.89174	0.042813	-0.56657	-0.40072	-0.53986	-0.40095	-0.4918	0.515704	-0.15294	-0.19955	0.200629	-0.32623
P46940	IQGAP1	-0.40208	0.639935	0.128946	0.39644	0.19366	0.206686	0.059472	-0.20885	0.061351	0.305661	-0.03985	0.370522	0.673565	0.226487	-0.5073	0.070269	-0.36295	-0.44996	-0.32361
P46952	HAAO	-0.53166	0.269111	0.004815	-0.19309	-0.00507	-0.63749	-0.21791	-0.77332	0.312563	0.311673	-1.61336	-0.34455	0.244498	0.240308	0.230962	-0.4383	-0.41997	-0.05121	-0.66625
P46976	GYG1	-0.85782	-0.59946	-0.42339	-0.43841	1.46451	0.181504	-0.99557	-0.90694	0.364628	0.012408	-0.77289	0.035045	-0.05272	0.792875	-0.15382	-0.45409	-0.28477	-0.84066	-0.85798
P46977	STT3A	-0.44496	0.610586	0.284108	0.160144	-0.17094	-0.15696	1.199587	-0.06872	-0.296	0.008701	0.161064	0.304598	0.718806	-0.24915	0.767809	-0.18565	0.145397	0.26309	0.537006
P47712	PLA2G4A	-1.15195	0.27329	-0.20514	-1.32797	-0.10316	-0.61324	0.700657	-0.241	-0.84993	-0.39212	-0.97635	0.269233	-1.00259	0.016149	-0.82214	-0.67264	-0.86854	-1.32056	-1.14178
P47736	RAP1GAP	0.342494	-0.47665	-0.44829	-0.68823	-1.12789	-0.46924	-0.1521	0.701611	-0.12481	0.100889	-0.06866	-0.81331	-0.33929	-0.08701	1.638364	-0.10053	-0.46111	-0.6254	0.170396
P47755	CAPZA2	-0.42629	0.228783	0.452255	-0.39288	0.421794	0.169149	0.086385	0.482327	0.142525	0.568279	-0.15329	-0.02925	0.161467	-0.00198	0.274888	0.732339	-0.18989	-0.05907	0.125766
P47756	CAPZB	-0.23216	0.210177	0.455485	-0.65233	0.521936	0.023904	-0.22592	0.053195	0.388477	0.75427	-0.31308	0.013404	0.198311	0.232212	-0.12728	0.213122	-0.12172	-0.00096	0.065272
P47895	ALDH1A3	-1.53324	0.302395	1.390221	-0.82447	1.478141	-0.25716	0.527249	-0.34172	1.241933	0.797419	-1.9127	1.593729	1.601145	0.549195	-1.43565	0.027468	0.697457	1.16372	3.106901
P47897	QARS	0.181585	0.252087	0.30595	-0.13197	-0.03104	0.342673	-0.04136	0.20453	-0.146	-0.16197	-0.36975	-0.13992	0.281649	0.053317	0.390318	0.248672	-0.16061	-0.30045	0.1201
P47914	RPL29	0.073969	-0.11886	0.248227	0.054119	-2.20747	0.252759	-0.00985	0.038236	0.217169	-1.25842	-0.36212	-0.47395	0.042996	-0.92776	0.54172	0.424005	-0.21069	-0.45413	-0.28784
P47929	LGALS7	-1.83843	0.626289	-0.402	1.564696	-1.18287	-1.25316	0.882569	2.196532	0.25999	-1.9203	-1.63307	-0.79674	-1.05692	-0.67354	-2.50811	-1.52922	-0.49952	-0.89344	4.043535
P47974	ZFP36L2	0.776431	-0.35001	-0.53479	-0.27513	-0.57649	-0.68946	-0.34887	-0.23948	-0.49834	0.654994	0.117787	-0.6782	-0.28582	-0.28379	0.582456	0.65643	-0.86253	-0.84643	-1.14101
P47985	UQCRFS1	0.049588	0.265651	-0.29342	0.250001	0.669911	0.16124	0.130775	-0.27096	-0.38711	-0.46681	0.456352	0.382766	-0.3007	0.256638	1.516268	-1.14302	-0.12728	-0.05142	-0.68951
P48047	ATP5O	0.021895	-0.01853	-0.07586	0.330199	0.303234	-0.14976	0.14506	-0.50953	-0.35992	0.178356	0.27197	0.191053	-0.3776	-0.01009	1.27057	0.923909	-0.46527	-0.69126	-0.66011
P48059	LIMS1	-0.49832	-0.49501	0.468631	-0.45304	0.451806	0.029191	-0.81618	-0.10444	-0.67581	-0.02109	-0.79877	0.110589	-0.12273	0.178656	0.203786	-0.10745	-0.27804	0.524068	0.388654
P48061	CXCL12	-2.98305	-1.28322	-0.37764	-1.37676	-1.65952	-2.09458	-0.01286	-3.24283	0.004397	-2.78648	-3.45388	-0.11002	-1.91561	-0.16009	3.158531	-1.7378	-0.52916	-2.42741	-2.79277
P48147	PREP	-0.6413	-0.48589	-0.1613	0.405474	0.38569	-0.94606	0.06435	-0.3369	1.110679	2.277683	-0.90992	0.852959	0.434011	0.589754	0.321148	-0.69726	0.882496	-0.63885	-0.12906
P48163	ME1	-1.76054	0.379096	0.979349	-1.48443	0.899456	-0.70364	1.42265	-1.44014	0.729886	-0.25613	-0.09836	-0.5288	-0.54409	-0.4678	-0.40325	-0.83841	-1.25074	-0.05611	-1.1555
P48200	IREB2	0.536337	0.140923	-0.1204	0.941252	-0.21982	1.061154	0.027783	0.670476	0.004173	0.791946	0.033238	-0.00779	0.312409	0.39545	-0.80779	-0.50832	0.876908	0.056962	-0.06222
P48380	RFX3	0.35234	-0.77984	-0.954	-0.19315	0.081098	0.13036	0.31044	0.23545	-0.3278	0.59245	0.893579	-0.1359	-0.63778	-0.22205	-0.23447	-0.46633	-0.59486	-0.32136	0.017557
P48382	RFX5	-0.36749	-0.14169	-0.07091	-0.35202	0.274558	-0.41898	-0.41689	0.929321	0.289154	0.403714	-0.32022	-0.30069	0.065034	-0.42552	0.159419	0.350949	-0.09617	-0.22894	-1.25299
P48426	PIP4K2A	-0.86221	0.951905	1.09017	-1.06304	0.993122	0.593203	0.375072	-0.70832	0.414498	0.417746	-0.4604	0.060198	-0.17695	0.356568	-0.3362	-0.4308	-0.1279	0.840564	0.210872
P48444	ARCN1	-0.53926	0.205794	0.043454	0.218991	-0.25667	-0.18696	0.435119	-0.05575	0.20265	-0.05016	-0.21783	-0.21845	0.495647	-0.20715	0.711687	0.571104	0.333467	0.376692	0.509231
P48449	LSS	-0.31349	0.260506	0.122179	-0.21769	0.591533	0.862121	0.686451	0.20467	0.364319	-0.41647	-0.68931	0.613074	0.208313	-0.19525	-0.57116	0.308044	0.117453	0.011163	0.167219
P48454	PPP3CC	-0.23073	0.45611	0.169504	-0.29386	0.435191	-0.53034	0.016407	-0.62753	0.268186	-0.40523	-0.55414	-0.08843	0.138856	0.127883	-0.41557	-0.40747	0.354143	0.18468	0.785241
P48506	GCLC	-0.01684	0.189761	-0.09285	0.431779	0.226935	-0.03769	-1.20156	-0.36909	-0.08003	0.160567	-0.49916	-0.83605	-0.64156	-0.25891	0.021709	-0.03904	-0.58881	0.090693	-0.81502
P48507	GCLM	0.891855	-0.07091	-0.48344	0.477153	0.982733	1.206671	-0.14029	-0.43269	-0.12051	-0.29415	-0.56307	0.231682	-0.09476	1.183029	1.085256	-0.25628	0.117356	-0.32549	-0.72115
P48509	CD151	0.433711	-1.16491	-0.61348	0.36059	0.895142	1.192832	0.283565	0.402041	-0.46318	-0.29613	0.226481	-0.81934	-0.8702	-0.58702	0.757676	-0.13324	-0.68638	-0.21109	-0.57095
P48553	TRAPPC1(	-0.08187	0.61762	1.055121	-0.23693	-0.58423	-0.36889	0.163056	-0.01141	0.044424	0.16998	-0.39381	0.480173	0.543857	0.689509	0.223631	0.100597	0.097542	-0.2554	-0.64107
P48556	PSMD8	-0.1971	0.086717	0.239746	-0.07437	-0.27893	0.255086	0.324502	0.180491	-0.24221	0.074336	-0.07466	0.311857	-0.12168	0.296156	-0.12007	0.376087	0.372773	-0.36143	0.285396
P48594	SERPINB4	-0.49775	1.635128	0.343602	-0.23908	-0.25755	-0.18361	0.073499	-0.47819	0.615335	-1.83814	0.017432	-0.07938	0.199122	-0.26289	-0.62312	-0.09488	-0.70134	-0.64634	-0.69496
P48595	SERPINB1	-2.40687	0.837268	1.252936	-1.10475	-0.24752	-2.13026	-0.38139	-1.27565	0.066268	3.369217	-1.02228	0.857876	-0.16361	3.249431	-0.62203	-1.33342	1.3434	-0.53437	-1.62793
P48634	PRRC2A	0.80334	-0.44507	-0.59214	-0.14686	-0.44873	0.128366	-0.59753	-0.34795	-0.22714	-0.1808	0.087998	-1.05842	0.977207	-0.94472	0.736267	0.769564	-0.03613	0.438353	0.035672
P48637	GSS	0.737767	-0.58703	0.492953	0.630073	1.154418	-0.06131	0.01195	1.172897	0.831484	1.227791	0.006809	-0.38156	0.456697	-0.25446	-0.28205	-0.51993	-0.37046	0.30108	0.378586
P48643	CCT5	-0.281	-0.0909	-0.10096	-0.03252	-0.09456	0.313051	-0.79193	-0.71175	-0.33924	0.112347	0.368266	-0.13656	0.048277	-0.18764	-0.14945	-0.14125	-0.15894	-0.2499	-0.08369
P48651	PTDSS1	0.237971	-0.13131	-0.41372	-0.31205	-0.23605	-0.25817	0.250873	0.963527	-0.31508	-0.45955	-0.17754	1.414144	-0.02605	0.046019	0.237931	-0.52916	0.259247	-0.29319	-0.63848
P48681	NES	0.669256	0.943695	-0.16306	-1.31653	-0.22061	-0.65597	-0.01507	0.60293	0.122425	0.988015	-0.66833	-1.63004	0.283643	-1.19398	-0.24583	-0.00647	-1.16917	1.700936	0.88425
P48723	HSPA13	-0.56223	0.189599	0.387002	-0.70556	-0.12887	-0.49314	1.339028	-0.03553	1.077192	-0.24606	-0.87633	0.260254	0.612333	0.200028	-0.21667	-0.15193	0.583517	1.418053	0.274441
P48729	CSNK1A1	0.43688	-0.67368	0.579705	-0.03001	0.068076	0.078579	0.373853	0.310609	-0.55557	-0.36636	0.167073	0.127315	-0.1154	0.137191	0.7304	0.104568	0.60027	-0.29864	-0.27282
P48730	CSNK1D	0.51326	-0.76792	0.085872	-0.30087	0.173388	0.186751	-0.11844	0.045347	0.040847	-0.29514	-0.36856	0.049554	0.200381	-0.06249	-0.01052	0.020298	0.358048	0.687	0.223446
P48735	IDH2	1.11468	-2.03918	0.183329	1.459872	-0.07954	0.076121	-0.87699	0.686707	-0.30767	0.388782	-0.43619	0.528377	0.7716	0.459955	1.448848	0.264378	-0.11235	0.064772	0.573822
P48736	PIK3CG	-0.90249	1.372992	0.844036	-0.21039	0.692701	0.41487	0.347669	-1.08849	-0.0388	1.042851	-1.43087	0.805086	0.056052	1.107768	-0.50469	-0.5179	0.36492	-0.09008	-0.93184
P48739	PITPNB	1.020519	-0.44562	0.774186	0.048435	1.283522	0.406666	-0.34589	0.143224	0.487045	1.127099	0.014197	-0.70083	0.723496	-0.2987	-0.08209	0.905985	0.144041	0.763156	0.429978
P48960	CD97	-1.09986	-0.03074	-0.00336	-0.95711	1.053701	-0.03136	0.458327	1.067453	0.390187	0.358951	0.200383	0.842807	-0.14593	0.839994	-0.09877	0.221736	0.545972	0.318489	-0.28818
P49005	POLD2	0.484633	-0.5085	0.933322	0.054114	0.944089	0.99734	0.247126	0.508819	-0.27849	-0.17758	0.284783	0.182347	-0.20509	0.341027	-0.7308	0.226944	0.110627	-0.27033	-0.91309
P49006	MARCKSL	1.360229	-0.50805	-0.35835	-0.46588	-0.89632	-1.59208	-0.07229	0.19626	0.203938	0.367315	0.269142	-0.96485	0.139453	-1.23388	-1.04468	-1.68097	-1.29717	-0.12395	0.782629
P49023	PXN	0.075768	0.004215	0.036007	0.087451	-0.32338	-0.41128	-0.85935	0.053092	0.368887	0.279128	0.468883	-0.08637	0.646807	0.610877	0.00358	-0.62021	0.241304	0.478628	0.220218
P49069	CAMLG	-0.44643	0.41153	-0.52352	0.313551	-0.07413	-0.21406	-0.07922	-0.30344	-0.91202	-0.0212	0.748676	-0.95361	-0.01799	-0.41959	0.247355	0.030677	-0.10546	0.39472	-0.43041
P49116	NR2C2	1.178903	-0.92683	-0.3108	-0.60885	0.182782	0.503977	0.769341	0.619583	0.509994	-0.21941	0.291457	-0.50585	-1.05939	0.110844	-0.20435	0.021784	-0.53018	-0.43552	-0.4017
P49137	MAPKAPK	0.242499	-0.10579	0.481863	0.159318	-0.06367	-0.73654	-1.27212	-0.16859	-0.55822	0.427567	0.16664	0.103776	-0.34097	0.95664	-0.12445	-0.42561	0.113018	-0.1726	0.538615
P49184	DNASE1L	-0.63463	-0.22604	0.40804	-0.51915	0.775649	0.031586	-0.00313	-0.50965	-0.30783	0.012695	-0.52359	-0.1575	-0.73053	0.390097	-0.71323	-0.12671	0.150638	0.295152	-0.43538
P49189	ALDH9A1	-0.10314	-0.75741	0.493399	-0.48751	0.877674	-0.04792	-0.06809	-0.29158	0.675181	0.580126	0.133065	0.104617	0.071814	0.037638	0.925984	-0.0306	-0.55217	-0.13022	-0.19069
P49207	RPL34	0.521868	-0.32516	0.389518	0.38347	-0.2906	0.049024	0.032116	0.181389	-0.70418	-0.66577	0.256302	-0.93492	-0.45876	-0.86407	0.021711	-0.08499	-0.16094	-0.39018	0.229665
P49247	RPIA	0.285644	-0.11473	-0.63716	0.195097	-0.15073	0.043605	-1.04325	0.133956	-0.31762	0.271252	0.17628	-0.42784	-0.18535	0.217854	0.025736	0.060214	0.010583	-0.02666	-0.24028
P49257	LMAN1	-1.19302	0.63801	0.132156	0.171543	-0.68415	-0.5212	1.432156	-0.57524	0.076585	0.112135	0.365676	-0.31434	0.377959	-0.34573	-0.37099	0.54863	-0.05344	0.336106	0.350587
P49321	NASP	1.457831	-1.08213	0.199551	0.289439	1.025581	1.408753	-0.62215	0.980945	-0.24946	-0.11586	-0.54235	-0.97081	-0.00312	-0.82014	-1.06513	0.407969	0.055817	0.195072	0.263975
P49327	FASN	-0.36459	-0.78935	-0.66883	-0.35028	-1.10717	-0.43042	-0.00258	-0.77146	-0.59316	-0.90599	-0.85476	-0.41457	0.259004	-0.52892	-0.99025	-0.78679	0.053848	-0.98601	-0.83042
P49354	FNTA	0.187781	-0.03939	-0.067	-0.39851	0.333703	0.397629	-0.94992	0.148875	-0.29252	0.19604	0.286062	0.18069	0.340658	0.40615	0.727851	0.139454	0.055591	0.225654	0.205799
P49356	FNTB	0.293359	-0.48218	-0.28447	-0.30346	0.204224	0.405853	-0.64082	0.286319	0.065757	0.502697	0.190415	-0.28632	0.394214	-0.41512	0.298592	0.303308	-0.29765	0.204845	0.66775
P49368	CCT3	-0.29513	0.028625	0.020609	0.142536	-0.16272	0.33858	-0.59287	-0.66202	-0.26305	-0.07339	0.313696	-0.11425	0.029914	-0.16257	0.033594	0.037219	-0.08399	-0.31588	-0.15344
P49406	MRPL19	-0.38881	0.136988	0.1137	1.297361	0.330695	0.428155	-0.25169	-0.3316	-0.43041	0.596457	0.661615	0.040914	0.398347	-0.12075	0.935161	-0.74215	-0.39676	-0.2312	-0.65989
P49407	ARRB1	-1.17092	0.698157	0.691764	-0.44286	0.686396	0.254625	-0.13162	-1.08631	-0.67193	2.083194	-0.53472	0.240652	-0.48145	0.792404	-0.35772	-0.62512	0.105607	1.12591	-0.75427
P49411	TUFM	0.444589	-0.45977	0.145276	0.873363	0.543232	0.453789	-0.62562	-0.30606	-0.4102	0.189029	0.411647	0.137807	-0.00923	0.170871	0.925311	0.593799	-0.09254	-0.20408	-0.56641
P49418	AMPH	-0.09692	-0.32528	-0.31272	-0.17642	-0.93177	-0.89871	0.038279	-0.17554	-0.04364	-0.34826	1.805655	-0.36624	-0.65347	-0.30403	0.321176	-0.3638	-0.86525	0.176254	0.434474
P49419	ALDH7A1	-0.46517	-1.63843	-0.52862	1.405743	0.683637	0.638381	-0.90773	1.579307	-0.03132	-0.10138	0.727885	-0.11903	0.704081	-0.08876	2.845715	1.846695	-0.37391	-0.10112	0.659973
P49441	INPP1	-0.75634	-0.0033	0.505576	-0.69521	0.000398	-0.61095	-0.27991	-0.30911	0.294712	0.116698	-0.25631	-0.60293	0.370375	0.236122	-0.03099	-0.15389	-0.27929	0.562085	0.251479
P49448	GLUD2	0.162035	0.199144	0.212067	-0.24267	0.322508	-0.07518	-0.11565	-0.90414	-0.18024	0.70564	0.460447	0.1275	0.05075	0.657108	0.164284	0.356034	0.746581	0.360483	-0.33716
P49454	CENPF	0.7459	-0.03677	0.861139	-0.29527	0.29572	0.320032	1.293407	1.032583	0.088425	-0.50091	-1.01454	-0.73698	0.006756	-0.63875	-0.81431	-0.17482	-0.94816	-0.86968	0.425813
P49458	SRP9	0.628965	-0.57362	0.112647	0.089579	-0.7017	-0.53957	-0.03232	0.219391	0.34463	-0.48164	0.406272	-0.28025	0.08813	-0.01032	-0.1586	0.211447	0.777905	0.238364	-0.23788
P49459	UBE2A	0.263643	-0.06017	0.102733	0.099422	0.939854	1.178202	-0.00552	0.9628	-0.28694	0.019482	-0.23834	-0.08928	-0.5484	-0.03644	0.05239	0.316334	-0.03133	-0.16796	-0.32018
P49585	PCYT1A	0.153715	-0.00596	0.401645	0.58329	0.444132	0.718434	-0.01641	-0.16789	-0.21921	0.210653	0.122435	-0.05622	-0.04634	-0.04137	2.084013	-0.14131	-0.07613	-0.37875	0.177704
P49588	AARS	-0.18304	-0.62375	-0.3753	0.654954	0.911319	0.983318	-0.23907	0.507581	-0.01933	-0.15628	-0.40012	0.160378	0.60609	-0.17121	-0.03954	1.073767	-0.23908	-0.24559	0.397095
P49589	CARS	-0.63779	-0.57436	0.31539	-0.52426	0.748225	1.170947	-0.0897	1.04792	0.145382	0.66992	-0.23955	-0.22344	0.585741	-0.25502	0.683927	0.200335	0.143435	0.44176	0.112185
P49590	HARS2	-0.77933	-0.09645	0.720144	0.801471	-0.12573	-0.13253	-0.29764	0.229982	-0.49256	-0.05805	0.144454	-0.04575	-0.03414	-0.02472	1.672603	0.871426	0.05039	0.294753	0.108368
P49591	SARS	0.687783	-1.04605	0.071982	0.256048	0.834052	0.856512	-0.90087	0.267391	0.130047	0.495396	0.259427	0.156284	0.391831	0.113086	-0.08092	0.900495	0.154531	0.132393	0.195516
P49593	PPM1F	-0.51109	-1.0356	0.861055	-0.57741	0.448535	-0.19224	0.044794	-0.65386	0.518984	0.708807	-0.35509	-0.45982	-0.07487	0.408434	0.024956	0.345156	-0.22571	0.730128	0.323295
P49641	MAN2A2	0.76391	-0.05251	-0.40959	-0.22739	0.133106	0.321534	-0.08279	0.044814	0.268295	0.199473	-0.4808	-0.19131	-0.18311	0.011592	0.367853	-0.05499	0.328251	0.835946	-0.04218
P49642	PRIM1	1.499288	-0.39387	-0.24288	0.0407	1.145835	1.659199	0.040667	0.28413	-0.45619	-0.55354	-0.0464	-0.3426	-0.28261	-0.32887	-0.44867	0.174167	-0.14781	-0.81284	-0.82692
P49643	PRIM2	1.324932	0.670061	0.622191	0.247749	1.419665	1.387085	-0.09944	-0.28569	-0.41706	2.195126	-0.00949	0.010637	0.13771	3.322399	-1.55526	-1.73279	1.431862	-0.66356	-2.15456
P49662	CASP4	0.037428	-0.05659	0.636633	-0.7409	0.364167	-0.44957	0.078744	-0.16694	0.096322	1.628066	-0.67787	1.012675	0.562063	1.617035	0.148974	0.367332	0.687129	0.330152	0.423807
P49674	CSNK1E	0.089226	0.282574	0.113555	-0.28961	-0.08141	-0.06361	0.222562	-0.16171	0.087096	-0.74429	0.485411	-0.15133	0.07899	-0.46867	-0.41945	-0.40412	0.340244	1.532001	0.325842
P49711	CTCF	0.565339	0.340253	-0.31295	0.234102	0.315508	0.495911	-1.43566	0.039921	-0.5352	-0.91715	0.463907	-0.29517	-0.03122	0.090664	-0.13475	0.053822	-0.03425	0.867928	-0.03889
P49720	PSMB3	0.285511	-0.54307	0.519639	0.218915	0.688769	0.614146	-0.59223	0.286023	0.18184	0.353939	0.297173	-0.48667	0.16756	-0.32031	-0.09371	0.572987	-0.42021	-0.38846	0.257404
P49721	PSMB2	0.098467	-0.43905	0.360763	-0.30635	0.358149	0.323067	-0.47965	0.046102	0.245115	0.356495	0.708542	-0.86184	0.055349	-0.13186	-0.33556	0.590014	-0.42452	-0.03085	-0.21762
P49736	MCM2	2.171936	-0.72138	-0.27584	-0.23813	1.546391	1.538862	-0.17232	0.912763	-0.9567	-0.93107	-0.35546	-0.55406	-0.49308	-0.36159	-0.16506	0.243632	-0.1423	-0.72049	-0.40353
P49746	THBS3	-0.17981	-0.34017	-0.82928	-0.75755	-1.076	-0.72443	-0.13236	0.694216	0.641211	0.144961	-1.08003	-0.90158	-0.15354	-0.49464	1.010277	-1.32407	-0.69178	-1.46343	0.605031
P49747	COMP	-1.30611	-2.28949	-1.47465	-2.17246	-1.76612	-2.5194	-0.86056	-0.207	2.070988	-3.27372	-3.54627	-0.43625	-0.56378	-1.14686	-1.49487	-2.39186	-1.18982	-3.5803	-0.71323
P49748	ACADVL	0.250111	0.476203	0.526446	0.05777	1.295227	0.700147	0.04183	-0.01224	0.050008	1.029973	0.537362	0.830039	0.559722	0.431329	1.197146	-0.21153	-0.07038	0.257807	-0.82136
P49750	YLPM1	0.119973	-0.02724	-0.18298	0.02053	-0.20398	-0.20391	-0.4687	0.765328	-0.0976	0.003815	0.649218	-0.49364	0.157333	-0.43573	0.098197	0.081883	-0.52953	0.391691	0.522749
P49754	VPS41	-0.20764	0.632625	0.28662	0.330653	0.186121	0.144023	0.159935	-0.32408	-0.14786	-0.10421	-0.32719	0.778552	-0.07907	0.612657	-0.28223	-0.28444	-0.05681	-0.34185	0.220593
P49755	TMED10	-0.47774	0.585773	0.18483	-0.0368	-0.01081	-0.00923	0.434451	-0.17763	-0.13352	-0.42248	-0.0185	0.395849	0.101119	0.159033	0.327143	0.228361	0.52327	0.425866	0.215565
P49756	RBM25	0.857634	-0.46016	0.118043	0.265754	0.017107	0.148649	-0.09026	0.490227	-0.36963	0.237444	0.316075	-0.19053	-0.3959	-0.4131	0.332087	-0.11195	0.267486	0.744037	0.114062
P49757	NUMB	0.161588	0.372964	-0.03703	-0.12635	-0.76035	-0.78906	-0.51175	-0.01474	0.558969	0.486789	0.208137	-0.42873	0.606584	0.108155	0.686003	0.390115	0.017323	0.64838	0.056703
P49760	CLK2	0.421027	-0.05821	-0.13461	0.06904	0.386138	0.134988	-0.17981	0.808407	-0.15263	-0.08594	-0.00886	0.102142	-0.28525	-0.04861	0.338118	-0.28027	0.163589	0.488882	-0.17088
P49761	CLK3	0.610874	-0.19062	0.22724	0.060554	0.611071	0.339456	-0.34428	0.355186	0.300585	0.287364	0.356088	0.457286	0.460416	0.397468	0.153346	0.000603	0.783953	0.407285	-0.14353
P49768	PSEN1	0.104921	0.130224	0.115208	-0.27614	0.048625	-0.0213	-0.30442	0.146045	-0.13932	-0.06901	-0.09853	0.358511	0.061421	0.077298	-0.42845	-0.02424	0.38584	-0.07577	0.288833
P49770	EIF2B2	-0.23655	0.098776	0.040212	0.442926	0.222468	0.666341	0.088984	0.193887	-0.12884	-0.14765	0.183693	-0.04772	0.002553	-0.10639	0.318207	1.102388	-0.24908	-0.33724	-0.1178
P49773	HINT1	0.226194	-0.14793	0.130733	-0.27646	0.725791	-0.14956	-1.16081	-0.0981	0.751957	0.371305	0.364995	0.069555	-0.24997	0.274961	-0.22304	0.41043	1.836729	0.393992	-0.56322
P49788	RARRES1	-1.97109	0.85703	1.35848	-0.71398	3.088651	2.445432	0.27352	-1.41677	-0.12691	-0.1946	-0.79104	0.775687	0.286984	0.442726	0.615218	-0.10036	1.875105	0.048987	-1.25208
P49789	FHIT	-0.05776	-0.1926	-0.03757	0.133593	0.922766	0.9639	-0.08147	0.017744	-0.04292	0.144302	1.790998	-0.36201	-0.16697	-0.32872	1.537388	-0.06112	-1.07795	-0.21413	0.674289
P49790	NUP153	0.996473	-0.39007	-0.6387	0.605729	-0.03518	0.292181	-0.89383	-0.36548	-0.84073	-0.67821	0.119868	-0.64025	-0.13992	-0.73416	0.572197	-0.25098	-0.38225	-0.0125	-0.11909
P49792	RANBP2	0.211688	-0.02917	0.010267	0.130401	-0.03747	-0.01565	-0.1191	0.284916	-0.3874	-0.18934	0.351673	-0.20778	-0.09362	-0.19893	0.952042	-0.0407	-0.10162	-0.11007	0.241459
P49795	RGS19	-0.95375	0.931953	0.805954	-0.79813	0.227815	0.043481	0.395416	-0.67588	-0.63983	0.810783	-0.92702	0.574353	0.56813	1.420686	-0.24748	-0.19303	0.12635	0.475829	-0.70758
P49815	TSC2	-0.079	0.443266	0.064251	-0.48832	-0.17394	-0.05427	0.445745	-0.14165	0.009276	0.104731	0.154546	-0.14493	-0.28521	-0.24137	-0.1563	-0.19535	-0.39725	0.108226	0.174838
P49821	NDUFV1	0.438831	0.013608	0.314791	-0.19852	0.389708	0.398315	0.518874	-0.21615	0.433362	-1.02992	0.716606	0.347624	0.094785	0.032072	0.981061	-0.1637	-0.37548	0.051688	-0.4994
P49840	GSK3A	-0.1032	0.17596	-0.09707	-0.4273	0.028694	-0.00442	0.288494	0.88023	0.656745	-0.33369	-0.32128	-0.14601	-0.16089	0.167292	-0.0697	-0.9544	-0.01892	0.130329	-0.3014
P49841	GSK3B	-0.02232	0.34503	0.028336	-0.9581	0.058488	0.077263	0.303798	-0.06819	-0.06063	0.137877	-0.38304	0.396175	0.090802	0.096042	0.359279	-0.24788	-0.14354	-1.00245	0.27302
P49848	TAF6	-0.11155	0.330202	-0.35312	0.056611	0.046292	0.032845	0.168549	0.782658	0.379628	-0.03456	0.050183	0.022862	-0.28247	-0.16308	0.601294	-0.23531	-0.26717	0.167594	-0.23854

P49902	NT5C2	-0.05325	-0.55189	0.731771	0.067688	-0.6253	-0.595	-0.61985	0.395369	0.298526	0.435009	0.341661	0.189794	-0.10509	0.20895	-0.11528	0.308604	0.657784	0.319952	0.390064
P49903	SEPHS1	0.311776	-0.53706	-0.28006	-0.01901	-0.13002	-0.16382	-0.93752	0.327437	-0.26591	-0.36445	0.105317	-0.17779	0.245843	-0.20525	0.362135	-0.02207	0.547277	-0.15695	-0.11347
P49908	SELENOP	-1.07807	0.307444	-0.85233	-1.06743	-1.02893	-1.66926	-0.18132	-1.18028	0.346876	-0.83005	-0.27462	1.538968	-0.01843	0.897839	-0.04643	-1.02245	1.409345	-0.01022	-0.63271
P49913	CAMP	-3.15629	0.033642	2.884495	-1.49674	-1.78578	-3.09916	-0.59383	-2.31536	0.460475	2.432224	-2.2775	-0.21875	-1.50472	0.515452	-1.84596	-2.41935	1.52047	-2.55151	-2.04528
P49914	MTHFS	0.557787	-0.48498	-0.11271	0.541129	1.720785	0.05272	-0.4237	1.469671	0.023312	0.454453	-0.02975	0.064741	-0.37146	1.132249	0.028963	-0.93078	1.744302	0.196816	-0.44376
P49915	GMPS	0.818425	-0.92378	0.495896	1.106071	2.178392	2.185273	-1.52035	0.797435	-0.01159	1.004453	-0.18655	0.040699	0.187596	-0.18037	0.664524	0.811512	0.185687	-0.27341	0.255682
P49916	LIG3	-0.12686	-0.84976	-0.07555	0.568867	-0.44	-0.20144	0.575706	-0.32534	0.031653	-0.64039	0.479474	-0.00239	-0.08532	-0.4639	0.652083	0.026261	-0.14508	0.103886	-0.2593
P49959	MRE11	0.162793	-0.44111	-0.27925	0.221738	0.347517	0.558785	-0.04669	0.636775	-0.6538	-1.79015	0.806496	-0.04584	0.21422	-0.3418	0.344784	0.89197	-0.20049	0.15591	-0.02512
P49961	ENTPD1	-1.72118	0.432778	-0.03477	-1.71098	0.566771	-0.37698	1.009305	-2.0472	0.257729	0.056446	-1.3089	-0.47936	-0.16806	-0.34331	-0.01328	-0.60061	-0.98474	0.107361	-1.05559
P50135	HNMT	-2.01354	0.783866	-0.27344	-1.00766	-0.21639	-0.58592	0.497345	-1.56881	-0.51273	0.924763	-1.18916	-0.21082	-0.16465	-0.36339	0.125252	0.314774	-0.09087	1.419436	0.049988
P50148	GNAQ	-0.37001	0.177275	-0.45055	-0.40158	-0.07691	-0.44689	0.08582	-0.99982	0.461537	-0.51002	-0.01869	0.134285	-0.4283	0.122321	-0.24311	-0.59861	-0.29047	-0.04701	-0.38317
P50151	GNG10	-0.93647	0.957772	0.168454	-1.17396	0.638384	-0.174	1.02254	-0.46679	-0.0667	0.811133	-0.21278	0.296637	0.086249	0.379589	-0.15933	-0.50971	-0.00024	-0.0932	-0.42302
P50213	IDH3A	-0.06803	-0.90985	0.540701	0.976996	1.3995	0.430964	-0.44504	-0.15477	0.032917	0.445496	0.454317	0.675583	0.120547	0.633729	0.678985	0.440312	0.175577	0.271421	-0.8319
P50225	SULT1A1	0.156223	-0.20744	0.950009	-1.1867	3.75112	2.425254	0.600496	-1.60258	-0.28021	-0.16619	-1.37347	1.249637	0.484876	0.764298	0.971388	-0.0579	0.323793	-0.08337	0.163803
P50238	CRIP1	1.046262	-1.04853	0.259477	2.152571	-3.39028	0.760468	-1.58405	-0.71875	-1.2095	-0.92837	0.422975	-1.73255	0.544776	-1.21872	1.126619	1.30501	-2.65182	-0.67359	-2.34815
P50281	MMP14	-0.42805	0.724355	0.034102	-0.83105	-0.27235	-0.44573	0.743653	0.482296	0.034817	-0.7226	-1.0248	-0.29827	0.624001	-0.88045	-0.66412	0.08139	-0.19494	0.88261	1.335203
P50336	PPOX	-0.22206	0.109202	-0.55634	-0.3878	-0.25464	0.044716	0.147666	-0.32446	-0.39258	-0.23133	0.350187	-0.19143	-0.08174	0.027918	0.832909	-0.3733	-0.46132	-0.64718	-0.66635
P50395	GDI2	-0.14064	0.251887	0.243569	-0.10694	0.712779	0.80343	-0.20656	0.003947	0.031698	0.518469	-0.27201	-0.83395	0.002819	-0.47482	0.024569	0.29639	-0.24588	0.196991	0.437051
P50402	EMD	-0.26674	-0.00095	-0.08971	-0.09695	0.509039	0.572569	0.18022	-0.63136	-0.03247	-0.41761	0.050149	-0.49695	-0.69294	-0.39791	0.05996	0.166113	0.536606	0.979157	-0.03664
P50416	CPT1A	-0.22314	0.48721	-0.01543	-0.28486	0.52538	0.343394	0.134567	-0.93852	0.019121	0.085513	0.516217	0.473682	-0.21519	0.793446	0.844654	1.557109	0.745769	-0.07668	-1.36148
P50440	GATM	-1.22274	0.148532	-0.57696	-0.31308	0.129195	0.265216	-0.52202	-0.20445	-0.22924	-0.18157	1.111107	-0.2829	-0.00165	-0.40271	-0.1595	-0.4074	-0.91065	-0.64739	-0.44144
P50452	SERPINB8	-1.13954	0.795336	0.344081	-0.25238	-0.41501	-0.59889	-0.45618	-0.53162	1.254936	1.697395	-0.30222	0.23515	0.711918	1.172057	-0.79073	0.802787	-0.01201	-0.82327	-0.00597
P50453	SERPINB9	-1.80069	0.765679	1.17833	-0.32394	0.044643	-0.07483	-0.72972	-1.35687	0.250353	1.369095	-1.80269	-0.35765	0.25307	-0.19717	0.321452	-0.32549	-0.57063	0.048998	-0.39642
P50454	SERPINH1	-0.6305	1.298079	0.204468	-0.79723	0.53239	0.380479	0.365134	0.36983	0.754756	0.567839	-0.48982	-0.3563	1.04768	-0.81844	-1.36937	0.365275	0.030184	0.930168	1.751359
P50479	PDLIM4	-1.41778	-1.41328	-0.54254	-1.50435	-1.30403	-1.16243	-0.82176	-0.00432	0.396346	-0.12932	0.199533	-0.86504	0.292929	-0.97146	-0.87308	-0.06934	-1.29345	-0.51516	1.22948
P50542	PEX5	0.02786	0.132024	-0.52076	-0.41416	-0.64708	-0.41447	-0.77938	0.007761	1.759604	0.181397	-0.08512	-0.25503	-0.30084	0.186263	-0.18721	0.381967	-0.02736	0.124919	0.372321
P50548	ERF	0.363694	0.25151	0.069282	-0.43645	-0.4416	0.093285	-0.04031	2.611202	-0.38604	-0.69512	0.018948	-0.33438	0.460618	-0.81013	1.491105	-0.93188	-0.97455	-0.85128	0.263467
P50552	VASP	-0.82156	-0.04761	-0.0538	-0.40679	-0.11687	-0.06424	-0.28657	-0.66953	-0.07881	0.73504	-0.59154	0.411024	0.445056	1.48146	-0.00313	-0.6075	0.665991	-0.38575	-0.10878
P50570	DNM2	-0.91732	0.519955	0.483802	-0.86744	-0.20679	-0.37987	0.385829	0.087458	-0.33559	0.364644	-0.42238	0.240663	-0.34199	0.433934	-0.57566	0.298072	0.407142	0.215068	-0.22645
P50579	METAP2	0.153423	-0.23154	0.60938	0.301924	-0.05005	-0.01675	-0.39058	0.27316	0.493913	0.395016	0.714915	-0.66173	0.609161	-0.52815	0.599642	0.08664	-0.44792	0.446792	0.457219
P50583	NUDT2	0.377471	0.443341	-0.78495	-0.63809	-0.81587	-1.04775	-0.36206	-1.16141	0.102234	0.115895	-0.19413	-0.5376	-0.56911	0.444385	-1.61335	0.694837	0.314317	0.052769	-0.30613
P50613	CDK7	-0.04091	0.119243	0.244897	0.070668	0.27392	0.298652	0.039189	0.368601	0.116238	0.122584	0.347053	-0.0253	0.274894	0.852989	0.195271	0.18239	0.633587	0.202522	0.046628
P50747	HLCS	-0.3779	0.207796	0.258385	-0.61815	0.033628	0.85131	0.08146	0.589726	0.014153	-0.08388	-0.04254	-0.15363	0.228554	-0.06314	-0.22197	-0.00376	0.582942	0.190513	0.059303
P50748	KNTC1	1.388053	0.094487	0.057057	0.695585	0.954691	1.484239	0.330927	0.580285	0.093338	-0.09139	-0.58544	-0.35736	-0.41307	0.087709	-0.55002	0.045017	0.201855	-0.78321	-0.05401
P50749	RASSF2	-0.73601	0.383379	-0.25823	-1.33055	-0.02216	-0.50745	0.060476	-0.833	0.390328	1.2415	-1.02032	0.854614	0.271674	1.544421	-0.19281	-0.18442	-0.23044	-0.41846	0.04901
P50750	CDK9	0.4157	-0.1115	-0.4937	-0.03685	0.089327	0.23237	-0.70888	0.36489	0.413595	0.20477	0.060736	-0.35998	-0.16494	-0.01231	0.041301	0.115926	0.409641	0.531344	0.367763
P50851	LRBA	0.090526	-0.52639	-0.98754	-0.1366	0.220213	0.104716	-0.35094	-0.13199	-0.44051	-0.40446	0.713882	-0.57237	-0.01339	-0.35491	-0.19926	0.641941	-0.34278	-0.8127	-0.59806
P50895	BCAM	0.88884	-1.55673	-0.99454	-1.18288	-1.05262	-0.51425	0.591014	0.935136	-1.3218	-2.16892	-0.31822	-1.82704	-0.29605	-0.8383	0.812804	-1.8351	-1.80739	-2.12934	-1.27459
P50897	PPT1	0.388105	0.030731	0.295439	-0.76045	-0.12677	0.236413	0.455998	1.423154	1.558317	0.96694	-0.41813	-0.1952	-0.23498	0.198977	-0.018	0.125835	-0.52105	-0.45796	-1.0086
P50914	RPL14	0.317034	-0.0691	-0.14681	0.158394	-0.08322	0.02549	-0.20744	-0.09908	-0.21122	-0.14276	0.50592	0.066028	-0.23908	0.184099	0.111644	-0.2546	0.076316	-0.29167	-0.26548
P50990	CCT8	-0.3492	-0.05736	-0.03506	-0.28927	-0.18235	0.184059	-0.71998	-0.58372	-0.12463	0.117564	0.262352	-0.13158	0.007436	-0.13435	-0.22251	-0.03532	0.269097	-0.30473	-0.11569
P50991	CCT4	-0.34781	0.097139	-0.00262	-0.10348	0.151074	0.407323	-0.61231	-0.76821	0.017287	-0.1272	0.242836	-0.17066	0.114462	-0.203	-0.14449	-0.14828	-0.13731	-0.4928	-0.25041
P50995	ANXA11	-0.01789	0.048942	-0.2241	0.294317	-0.45975	-0.24238	0.139697	0.411161	-0.13002	-0.13403	-0.22777	-0.16793	-0.47534	0.577787	0.752083	0.155863	0.268912	-0.21484	-0.11263
P51003	PAPOLA	-0.51348	0.084943	-0.73843	0.115197	0.23367	0.312275	-0.25313	0.231088	0.036117	0.294874	0.147793	-0.14868	0.461799	0.029829	0.806912	0.340853	0.630941	1.467722	0.199119
P51114	FXR1	0.660903	-0.19823	0.204488	0.002636	0.615788	0.889411	-0.86823	0.28036	-0.01064	0.147401	-0.09161	0.078007	0.278129	-0.32014	1.179967	-0.87531	-0.07344	-0.24285	0.234685
P51116	FXR2	0.430214	-0.44187	0.240508	-0.57193	-0.38188	-0.05063	-0.1269	-0.1049	0.340445	-0.1097	-0.47367	-0.09164	0.317419	-0.20077	-0.16975	-0.10606	-0.27482	-0.45713	0.132738
P51148	RAB5C	-0.4644	0.566469	0.501265	-0.05857	0.141424	-0.09916	0.435944	-0.5818	-0.19603	0.280551	-0.3047	0.872676	0.192145	0.603293	-0.22271	0.326436	-0.01961	0.283801	-0.17602
P51149	RAB7A	-0.28262	0.417048	0.30468	0.425616	0.395253	0.150638	0.472989	-0.0834	0.160253	0.808973	-0.48351	0.65568	-0.08556	0.722404	0.637864	-0.07532	0.283452	-0.309	-0.18907
P51151	RAB9A	-0.47424	0.411176	0.030777	-0.00328	0.535322	0.025272	0.317138	-0.79203	-0.35151	-0.08727	-0.73951	0.898203	0.112366	0.89701	-0.77928	0.575991	1.637207	0.842902	-0.16298
P51153	RAB13	-0.40912	-0.15243	-0.04953	0.696809	0.542428	0.15664	-0.02136	0.283388	-0.0453	0.49947	-0.75046	0.40049	0.243927	0.608369	1.081465	-0.21666	0.132175	-0.32519	0.286747
P51159	RAB27A	-0.40394	0.201968	1.437517	-1.47478	0.002705	-0.25956	0.35238	-2.22814	0.133405	0.779892	-0.37392	0.844999	0.57049	2.58526	-0.51579	-0.24014	1.390438	1.030463	-0.33026
P51178	PLCD1	0.172221	-0.11829	-0.24706	-0.2879	-0.24692	-0.29884	-0.59538	-0.5313	-0.53527	-0.41115	0.52182	0.088999	0.16084	0.009839	0.182224	-0.59493	-0.68728	-0.24619	0.20888
P51397	DAP	0.085834	-0.17566	-0.14989	1.196752	-0.47339	-0.29884	0.427539	-0.95327	-0.01034	-0.78958	-0.25978	-0.35511	0.145268	0.240253	0.775533	-0.14268	-0.70769	0.546048	0.025635
P51398	DAP3	-0.33263	0.011095	-0.15833	0.737233	0.1609	0.022663	0.338024	0.115811	0.489847	0.294302	0.357331	0.020079	-0.49739	-0.25551	1.078556	-0.21923	-0.33592	0.091462	-0.45302
P51452	DUSP3	-0.79815	0.387366	0.191453	-0.08117	0.186884	0.173078	-0.34347	-0.29535	0.702945	0.580523	-0.35911	0.456662	0.312974	0.396813	-0.61566	0.043253	0.530161	-0.10722	0.191649
P51511	MMP15	0.305335	0.124732	-0.18925	-0.03626	0.191981	1.259593	-0.19727	0.213911	-0.04813	-0.08039	-0.26606	-0.33754	0.194703	0.428799	-0.23032	-0.13353	-0.25968	-0.03273	0.487591
P51531	SMARCA2	0.820208	0.37041	0.115643	-0.11947	0.87094	0.56489	-0.52817	-0.57115	-0.05767	0.080137	0.272242	-0.20147	-0.37945	-0.20359	1.261063	0.191016	-0.32049	0.516142	-0.3857
P51532	SMARCA4	0.513238	-0.23107	-0.0162	-0.07823	-0.31665	-0.08507	-0.09722	0.865822	-1.08327	-0.06778	-0.09687	-0.46371	-0.50414	-0.2238	0.186969	-0.55717	-0.25299	-0.37618	-0.04414
P51553	IDH3G	-0.1874	-0.51836	0.337318	1.021514	0.802385	0.350501	0.244733	-0.43509	0.272958	0.205143	0.345683	0.438002	-0.42819	0.175554	0.560522	0.208642	0.0593	-0.1991	-1.15701
P51570	GALK1	-0.11594	0.549737	-0.25127	-0.1665	0.055379	0.024135	0.173209	-0.57029	-0.07549	-0.02255	-0.67361	0.384718	0.115799	0.572966	-0.08773	0.067964	0.032271	-0.0901	-0.07313
P51571	SSR4	0.026036	0.263337	0.094881	0.296681	0.287659	0.139489	1.101186	-0.47917	-0.05228	-0.20922	-0.12329	0.338492	0.176441	0.194557	0.88671	0.175644	0.092033	0.200634	0.01898
P51572	BCAP31	-0.53605	0.840455	0.500321	1.630583	0.665088	1.004975	0.38026	-0.2482	-0.78044	-0.27485	0.079393	-0.06611	-0.44573	0.291267	-0.30044	0.039761	0.020947	-0.13902	-0.66951
P51580	TPMT	0.662062	-0.25738	-0.01933	1.421523	0.612466	0.130318	-0.12494	-0.22767	0.551222	-0.07979	-0.7554	0.820095	0.44995	0.954981	-0.07599	1.055995	-0.25699	-1.20678	-0.45881
P51606	RENBP	-1.05716	1.344216	0.918418	-0.41296	1.593168	0.458047	0.462956	-0.63878	-0.22294	0.785855	-1.19247	1.825066	0.317578	1.811212	-0.48393	-0.60728	0.392855	0.131627	-0.68259
P51608	MECP2	-1.01395	-0.24427	-0.26083	-0.89373	-0.52418	-1.1695	-0.33979	-1.04845	-0.22079	-1.12947	-0.31118	-1.30977	-1.11734	-1.1971	-0.27483	-0.88934	-0.29734	1.000954	-1.25286
P51610	HCFC1	0.525932	0.252541	-0.04656	0.268738	0.22954	0.359236	0.194301	0.463976	-0.36169	-0.18772	0.09471	-0.22522	-0.38077	-0.16899	0.036868	-0.4789	0.155398	0.333426	-0.2713
P51617	IRAK1	0.539865	-0.48065	0.148878	-0.29687	0.347578	0.515524	0.312078	-0.37979	-0.01932	0.687734	0.25986	0.066851	-0.61035	0.196418	-0.22581	-0.23452	0.988712	-0.14106	-0.96665
P51636	CAV2	-1.90313	0.092833	0.122624	-1.91073	-1.26718	-1.65416	0.540302	-2.21598	-0.46887	-1.53005	-0.48954	-1.51818	-0.84543	-0.70402	-0.24984	-0.97397	-1.01046	0.337056	-0.50639
P51648	ALDH3A2	-0.55263	-0.07191	-0.44404	0.397113	-0.17705	-0.4128	0.176748	0.623609	-0.4182	-0.7729	0.31089	-0.31108	-1.26605	0.0701	0.654201	-1.30923	-1.03382	-1.38219	-1.6456
P51649	ALDH5A1	1.041767	-2.08922	-0.87741	1.187307	-0.10158	-0.18503	-1.54622	0.526964	-1.31742	-1.00093	1.479422	0.079283	0.283963	-0.47706	1.335037	1.32922	-1.09903	-0.63458	-1.68981
P51659	HSD17B4	-1.22736	1.072925	0.748191	0.127447	0.056942	-0.0806	0.395545	0.076162	-0.08173	0.722782	-0.6351	1.140292	-0.24443	0.579357	0.627248	0.404479	0.482805	0.669863	-0.15537
P51665	PSMD7	-0.29316	0.284163	0.398405	-0.04437	-0.15392	-0.02145	0.076295	0.101937	0.374133	0.034483	-0.01415	0.13661	0.146669	0.285861	-0.41874	0.332603	0.038019	0.081649	0.006624
P51668	UBE2D1	-0.21262	0.078135	0.335808	-0.37309	0.293622	0.065473	-0.15745	0.460279	0.123879	0.492272	-0.82162	0.651587	0.073878	1.186062	-0.57622	-0.09457	0.362992	0.019215	0.38001
P51687	SUOX	0.333993	-0.48743	0.479219	-0.0571	1.433153	0.274613	0.105879	-0.06668	0.010476	-0.20537	-0.01724	-0.70895	0.331862	-0.41079	0.429634	-0.23617	-0.939	0.372363	0.414031
P51688	SGSH	1.146229	0.032004	-0.08756	0.205041	0.318446	0.24376	0.364759	0.293537	-0.29689	0.290561	0.473673	-0.00189	-0.35667	-0.30966	0.312783	-0.24113	-0.96707	0.199824	0.452897
P51689	ARSD	-0.34978	0.537686	-0.33467	-0.46703	-0.93129	-1.07863	0.561042	-0.39994	0.005468	-1.08457	0.760788	0.029119	0.116161	-0.28227	-1.46099	-0.28418	-0.14931	-0.71178	-0.23168
P51692	STAT5B	-1.44375	0.483434	0.528647	-0.16814	-0.49469	-0.23198	-0.51889	-0.22762	-0.2587	0.637906	-0.92042	0.162753	-0.79038	1.281646	-0.22934	-0.35902	-0.09348	0.167904	0.013678
P51784	USP11	-0.30673	-0.35578	-1.43789	-0.62026	-0.6634	-0.3267	-0.47317	0.974674	0.862783	1.276463	-0.34417	-0.18833	-0.20123	-0.16699	-0.44762	-0.61291	0.228582	-0.14304	-0.00259
P51790	CLCN3	-0.10859	0.31358	0.087985	0.079453	-0.21516	-0.61736	0.203998	-0.36108	-0.19261	0.363491	0.466786	0.196916	-0.14204	0.071873	-0.96266	0.219942	0.222154	0.272539	-0.12396
P51798	CLCN7	-0.24419	0.902237	0.925917	-0.26229	0.022966	-0.59563	0.779469	-0.42868	0.167016	0.285632	-0.72208	1.102562	0.606932	0.983681	0.095197	-0.32793	0.290756	0.239613	-0.44882
P51809	VAMP7	-0.43737	0.98017	0.854809	-0.33067	0.767986	0.713819	0.209174	-0.25023	-0.50833	0.710957	-0.82708	0.404842	-0.14162	1.040683	0.226654	0.142111	0.311592	-0.05643
P51812	RPS6KA3	-0.60131	0.342761	0.558261	0.213669	0.485495	0.598537	0.010553	-0.70588	-0.45443	0.512678	0.09581	0.587859	-0.18946	0.839318	-0.60804	-0.53427	0.101991	-0.01729	0.028256
P51825	AFF1	1.195216	-0.50873	0.150158	0.163874	0.888582	0.238934	-0.38285	-0.02192	-0.12618	-0.04767	0.780695	-0.53378	-0.0577	-0.19226	2.254293	0.462533	-1.01713	0.280789	-0.72202
P51828	ADCY7	-0.03362	0.446232	0.540154	-0.32949	0.428915	0.406785	0.317095	-0.21362	0.547848	0.048266	-0.16985	-0.10487	0.081475	0.292631	-0.99066	0.420561	-0.64111	0.137619	-0.53027
P51858	HDGF	-0.17243	-1.14196	-1.24509	-0.21683	1.176296	-0.96532	-1.1734	0.590235	0.312902	0.814245	-0.21979	-0.6287	-0.35455	-0.57965	0.643584	-0.78234	-0.19388	-0.37524	-0.96915
P51884	LUM	-2.2469	-0.96367	-0.33198	-2.70904	-0.44547	-0.74212	0.039374	-1.91795	1.157678	-0.14887	-0.29788	-1.35353	-1.00861	-1.0869	-0.1935	-0.8704	-1.19177	-0.67927	-1.19682
P51888	PRELP	-2.97196	-1.22275	-0.73837	-2.45846	-1.53161	-2.71321	0.369576	-1.89045	-0.5136	-1.7747	-1.11023	-1.4335	-1.93503	-0.72761	0.417135	-1.53824	-1.52055	-1.39072	-2.78542
P51911	CNN1	-4.3813	-1.89658	-1.96117	-3.21408	-1.88652	-2.80857	-1.97129	-3.3479	0.360384	-2.98929	-3.41711	-1.63842	-1.62039	-1.26019	-0.29051	-2.37174	-3.10251	-3.08452	0.186437
P51946	CCNH	0.029989	-0.12358	0.074238	-0.15643	0.409466	0.357553	-0.22404	0.626362	-0.24746	-0.04139	0.179197	0.012043	-0.2274	0.087883	0.089705	-0.19143	0.365423	0.253176	0.132413
P51948	MNAT1	0.121992	-0.05973	0.039132	0.096712	0.824536	-0.05511	-0.22244	0.102181	-0.07998	0.038051	0.134211	-0.16629	-0.04332	0.173706	0.682952	0.284991	0.512379	0.41124	-0.09258
P51965	UBE2E1	0.282962	-0.61555	0.083964	0.055148	0.438509	1.118923	-0.23333	1.266988	-0.24808	-0.03577	0.2699	-0.63944	0.087314	-0.38475	0.140536	0.710663	-0.32796	-1.0334	-0.10856
P51970	NDUFA8	0.37037	-0.06529	0.257627	-0.06866	0.452112	0.630475	0.228933	0.472778	-0.22905	-0.93181	0.37743	0.081006	-0.3036	0.109507	0.097552	-0.06593	-0.02934	0.314936	-0.19016
P51991	HNRNPA3	0.586781	-0.47343	-0.26851	0.488173	-0.01607	0.174734	-1.08669	0.309941	-0.61105	-0.17593	0.444036	-0.29079	-0.37202	-0.03781	0.230239	0.186176	0.15022	0.35094	-0.07351
P52209	PGD	-0.21071	-0.31629	0.351448	-0.51938	0.391232	-0.07497	-0.42306	0.835082	-0.06049	1.256699	-0.62407	0.135452	-0.03071	1.228275	-0.94813	-0.48573	0.517752	-0.44348	-0.05845
P52272	HNRNPM	0.206086	0.1248	0.086778	0.080915	-0.02615	0.30986	0.002657	0.074662	-0.05426	-0.09382	0.478747	-0.10926	-0.19939	-0.27147	0.154686	0.000369	0.021116	0.561284	-0.01591
P52292	KPNA2	0.302587	0.611053	0.08303	0.634278	0.557681	1.07646	0.103705	0.521203	0.522547	-0.37597	-0.92578	-0.23634	0.156018	-0.33812	-0.71922	0.689618	0.022089	-0.64877	-0.0485
P52294	KPNA1	0.205759	0.136564	0.85472	-0.27749	-0.22942	0.412688	-0.5462	0.110619	0.261565	0.46361	-0.15876	0.226096	0.131998	-0.01028	0.460859	-0.19294	0.43955	0.052404	0.080929
P52298	NCBP2	0.404504	-0.08734	0.260889	-0.08959	0.626624	0.669862	-0.84996	1.073062	0.154172	-0.84607	0.386402	-0.01436	0.58264	-0.18594	0.433269	0.421965	0.139978	0.125605	-0.02818
P52306	RAP1GDS	-0.08774	0.428684	0.165818	-0.55436	0.222559	0.335085	-0.33173	-0.24175	0.922113	0.71047	-0.16278	-0.11058	-0.00967	0.057236	-0.79844	-0.16404	-0.29148	0.045825	0.5644
P52333	JAK3	-0.23153	-0.40459	0.967826	0.341743	-1.08383	-0.93641	-0.16772	-0.20104	-0.28688	0.171518	-0.25902	-0.90585	-0.2855	-0.30213	-0.32189	-0.08205	0.04685	0.133482	0.475369
P52434	POLR2H	-0.13864	-0.56456	-0.45643	-0.21702	1.19713	-0.0913	-0.6223	0.529178	0.083184	0.186211	-0.21608	0.167705	-0.39612	0.304887	-0.16806	-0.23245	0.407819	-0.17408	-0.34838
P52564	MAP2K6	0.609166	-0.54679	-0.15587	-0.40248	-0.59986	-0.24629	-0.13478	0.192744	-0.41657	0.019502	-0.39286	-0.39645	-1.52814	-0.4086	-0.35515	0.053513	-0.05083	-0.62946	-0.25214
P52565	ARHGDIA	-1.28722	-0.48526	-0.86824	-1.27863	1.319681	-1.01345	1.602771	-0.92291	1.262383	3.091778	-0.93071	1.242845	-0.9084	1.266562	-0.93047	-0.45944	0.67752	-0.58812	-1.45707
P52566	ARHGDIB	-3.52337	-0.44329	-0.85393	-2.36253	1.596186	-1.67842	0.919236	-2.44997	0.621107	4.106733	-2.47562	1.800687	-1.16654	3.151527	-0.89937	-1.35837	1.097629	-1.08653	-2.69895
P52594	AGFG1	-0.32135	0.547306	0.580789	-0.91746	-0.41964	-0.51091	0.20014	-0.28539	-0.78697	0.428478	0.035673	0.115872	0.992663	0.781988	0.429687	0.351765	-0.15755	0.447507	0.265941
P52597	HNRNPF	0.842821	0.194527	-0.14398	0.206932	0.689529	0.869941	-0.72822	0.616413	-0.63627	0.240365	0.675522	-0.06444	-0.25638	0.052189	-0.68882	0.070092	0.20315	0.345672	-0.50046
P52630	STAT2	-0.06595	1.12563	1.197759	-0.72479	-0.03379	0.30246	-0.29325	-0.20532	0.158164	-0.14151	-0.52814	0.145615	0.515712	0.409849	-0.50183	-0.03676	0.652892	0.920686	0.347385
P52655	GTF2A1	1.001906	-0.29998	-0.74723	-0.06424	0.281007	0.515437	-0.73676	0.413181	-0.55158	-0.78332	-0.1867	0.127226	0.323723	-0.42141	-0.38149	0.041592	-0.01208	-0.30147	-0.6583
P52657	GTF2A2	0.563969	-0.35897	-0.08205	-0.0044	0.47027	0.460211	-0.20441	0.454654	-0.27254	-0.3298	-0.05064	-0.19351	0.085016	-0.33358	-0.31026	0.482864	-0.03723	-0.50568	-0.40964
P52701	MSH6	1.946164	-0.45328	0.327078	0.232672	0.660723	1.038662	-0.19251	1.102973	-0.29862	-0.4057	-0.09364	-0.71502	-0.70345	-0.32176	-0.30131	0.248226	-0.92034	-1.30233	-0.54636
P52732	KIF11	1.283301	-0.49419	0.503404	0.043969	1.134839	1.560164	0.542039	1.249073	0.626412	0.328609	-0.91868	-0.60218	0.018989	-0.58794	-0.65332	0.387301	0.527533	-0.8168	-0.12507
P52735	VAV2	-0.27062	-0.04926	0.094048	-0.98427	-0.36165	-0.43234	0.565502	1.392347	0.202806	-0.11696	-0.16982	0.069321	0.198799	-0.14922	-0.7025	-0.6809	0.35662	-0.52317	0.597006
P52747	ZNF143	0.362086	-0.44752	-0.06943	0.034502	-0.89884	0.388085	-0.11189	0.485917	-1.37173	-0.23621	0.323041	-0.80975	-0.57696	0.576828	0.324273	-0.0425	0.061052	0.493414	-0.85237
P52756	RBM5	0.617611	0.12749	-0.00083	-0.21166	0.20391	0.545561	-0.76777	0.491267	-0.3966	-0.22282	0.644189	-0.56315	-0.07531	-0.14946	-0.0973	-0.27119	-0.75213	0.088172	-0.10548
P52758	RIDA	-0.48999	-0.1185	0.234687	-0.64956	0.598193	-0.18989	-0.05198	0.198515	-0.24243	-0.95139	0.396071	0.343188	0.076216	-0.47947	-1.16417	-0.27247	-0.10289	-0.2498	-1.33952
P52788	SMS	-0.154	0.272955	-1.10747	-0.46461	0.201938	-0.15735	-0.98622	0.267774	-0.51751	-0.3549	0.510962	0.029755	0.547618	0.184777	-1.35065	-0.82236	0.497279	0.447989	0.384362
P52789	HK2	0.672855	-0.48873	-0.53974	0.658943	-0.75731	-1.69294	-0.38591	0.713766	-0.41145	-0.07678	-0.46865	2.240354	0.105684	1.651554	-0.75722	-0.49838	1.12678	-1.73581	-0.62842
P52790	HK3	-1.91581	2.085017	1.533197	-1.07379	-0.4473	-1.46146	0.233064	-1.45425	-0.45901	1.157638	-1.61558	1.988506	0.986011	2.482928	-1.00521	-1.3907	0.853823	-0.38803	-2.22473
P52815	MRPL12	-0.05272	-0.52856	-0.2107	0.879323	0.811891	0.637229	-1.27483	-0.73224	-0.21906	0.202614	0.335623	-0.21204	0.161644	-0.61725	-0.1174	0.271179	-0.11523	0.060626	-0.94827
P52888	THOP1	-0.25579	0.06083	-0.19791	-0.24431	0.45458	0.827835	-0.1599	-0.1243	0.025091	0.246226	0.325556	-0.87457	0.01318	-0.28545	-0.77197	0.218995	-0.57623	-0.36493	0.017355
P52907	CAPZA1	-0.16449	0.391341	0.36632	-0.62253	0.503934	0.228507	0.013292	0.063182	0.548143	0.759212	-0.38602	-0.00932	0.266072	0.202452	-0.0783	0.198716	-0.18941	0.163589	0.107055
P52926	HMGA2	-1.70251	-1.02149	-1.2343	0.046601	-2.87225	-3.62242	-0.98839	-0.47845	0.725807	-2.13718	-1.44331	-3.04424	-1.40747	-0.8578	0.573428	-2.99751	-2.07167	-3.25525	1.641709
P52943	CRIP2	0.625849	-1.8384	-0.88081	-0.30617	-0.86206	-0.20454	-1.17564	-0.09349	-1.22247	-1.82735	0.011521	-1.13396	-0.80082	-1.77011	0.200936	-1.15128	-1.32147	-2.22201	-0.45796

P52948	NUP98	-0.04674	0.261026	0.180544	0.038416	0.105069	0.194931	0.349546	0.154425	-0.09008	-0.18534	0.183208	0.051249	-0.14515	-0.06483	0.676956	-0.27174	0.139674	-0.0481	0.064684
P53004	BLVRA	-1.30448	0.683908	0.171547	-0.95665	1.198404	0.067777	0.311698	0.207802	0.171462	0.347275	-0.81365	0.955825	0.259895	1.178269	-0.28559	-0.2255	0.563423	0.726114	-0.0339
P53007	SLC25A1	0.881021	0.174288	0.003663	0.696319	0.045947	-0.19271	-0.2826	-1.22209	-0.02671	-0.44916	0.636338	0.15631	-0.11292	0.156015	0.547984	0.986231	-0.15677	0.258241	-0.77605
P53041	PPP5C	0.754375	-0.52891	-0.37734	0.338042	0.60075	0.406974	-1.03301	0.549565	-0.31042	-0.03228	0.081436	-0.29704	0.871309	-0.31173	0.421747	0.035336	0.043624	0.38356	-0.14838
P53350	PLK1	0.770436	-0.53933	0.0778	-0.24589	0.600956	0.535034	0.193653	0.606117	-0.04463	0.109136	-1.06405	-0.58837	-0.12356	0.048653	0.050451	0.550919	0.349819	-0.6461	-0.30404
P53355	DAPK1	-0.42811	1.21272	0.201603	-0.7008	0.205701	-0.13446	-1.06538	-0.37828	0.676973	0.478838	-0.25333	0.411093	-0.29737	0.590918	-0.41433	-0.0387	0.57915	1.58327	-0.29666
P53365	ARFIP2	-0.49389	0.149573	0.772977	-0.60764	1.197263	1.096604	0.875947	0.566627	-0.16499	0.054228	0.530186	-0.46445	-0.22087	-0.74872	-0.76583	-0.10618	-0.07101	0.102097	0.320548
P53367	ARFIP1	-0.58248	-0.02543	0.33724	-0.43937	0.199406	0.233192	-0.57859	0.022369	0.017622	0.067936	0.321407	-0.18009	0.317077	-0.09382	-0.55964	0.099306	-0.06408	-0.15896	-0.18898
P53370	NUDT6	0.788418	0.028079	-0.68252	-0.61194	-0.49653	-0.27385	-0.42637	-1.25572	0.504431	-0.36556	1.366927	0.08304	0.394251	-0.32658	0.108851	-0.04021	-0.4717	-0.54039	-0.37874
P53384	NUBP1	-0.4627	0.302882	0.239298	-0.23908	0.654367	-0.18379	0.141961	0.068236	-0.00253	0.133748	-0.06928	1.007585	0.286695	0.62742	-0.03712	-0.20095	0.818371	0.253363	-0.38744
P53396	ACLY	-0.54112	0.484752	0.661574	0.682454	-0.31037	0.084987	-0.0865	-0.42496	0.065123	0.420297	-0.02589	0.334085	0.65291	0.519939	0.240553	0.690989	0.281353	0.097912	0.297763
P53582	METAP1	0.860321	-0.53109	-0.55565	-0.26927	-0.16343	-0.19413	-0.56833	-0.25861	0.101342	-0.42424	0.251908	-0.05669	0.565859	0.167355	0.187833	-0.12135	1.233415	0.876075	0.306073
P53597	SUCLG1	0.142053	-0.76192	-0.1772	1.020715	0.571401	-0.1025	-0.46828	0.111409	-0.17835	0.188222	0.372433	-0.03427	0.02703	0.288694	1.289596	0.236869	-0.15597	0.251829	0.01431
P53602	MVD	-0.8395	-0.52862	-0.84277	-0.25297	0.943718	0.291229	-0.10624	-0.41651	0.588254	0.932077	-0.48482	-0.03649	0.128115	0.16546	-0.27455	1.08796	-0.02868	-0.2167	0.229045
P53609	PGGT1B	-1.08172	-0.14242	0.348464	-0.78305	0.141125	0.156255	-0.32252	-0.01655	0.263933	0.54881	0.072037	0.327597	0.765714	0.605746	1.040142	0.478524	0.376657	-0.14317	-0.87544
P53611	RABGGTE	0.21232	-0.05036	0.617818	-0.05989	0.106892	0.53276	-0.05415	0.30907	0.125221	0.586524	-0.07111	-0.47022	-0.10245	-0.09921	0.118846	0.191083	0.364296	0.105419	-0.2164
P53618	COPB1	-0.51408	0.513482	0.351054	0.105973	-0.01783	0.163212	0.527171	0.064541	-0.01481	-0.00094	-0.15613	0.040272	0.240628	0.106429	0.356302	0.624525	0.49976	0.242354	0.873368
P53621	COPA	-0.4701	0.482283	0.237385	0.190005	0.048391	-0.00594	0.464224	-0.05406	0.082965	0.04139	-0.06482	0.027157	0.350067	0.144142	0.478801	0.395006	0.448182	0.273403	0.712199
P53667	LIMK1	-0.33288	0.393639	-0.15246	-0.44554	-0.22125	0.339007	0.212895	-0.13416	-0.28813	0.393579	-0.61368	-0.01528	0.323604	0.134245	-0.55428	-0.34238	-0.11919	-0.28942	-0.01843
P53671	LIMK2	-0.00903	0.127946	-0.07468	0.435267	-0.11308	0.00317	0.41684	-0.31891	-0.141	-0.50738	0.178453	0.141625	-0.13922	0.576571	-0.34088	-0.04008	-0.12623	0.012306	-0.35901
P53675	CLTCL1	-1.19693	-0.39239	-0.68622	-0.15501	0.092014	0.184097	-0.65536	-0.01926	-0.18142	0.268877	0.075924	0.293099	-0.18786	0.547188	-0.09028	0.533676	1.769686	-0.07129	-0.14454
P53677	AP3M2	-0.53041	0.224509	0.203302	-0.71363	-0.2481	-0.74997	-0.21802	-0.57316	-0.08916	0.268878	0.446921	0.102816	0.407552	0.066529	-0.00688	-0.13514	0.219519	0.28443	0.747171
P53680	AP2S1	-0.50938	0.796293	0.521847	-0.06898	0.348753	0.257922	0.478991	-0.10414	-0.22427	-0.08985	-0.19562	0.291368	0.152578	0.396945	0.53083	0.33398	0.072564	0.309455	0.320908
P53701	HCCS	-0.11976	-0.10403	-0.14397	0.390792	0.446643	-0.28882	-0.5157	-0.78928	-0.3632	-0.49495	0.893302	0.136699	-0.14086	-0.29612	-0.4148	0.973698	-0.14126	-0.28262	-0.30966
P53804	TTC3	0.484241	-0.13329	0.057462	0.280747	0.123185	-0.25471	-0.37537	0.260696	0.015358	0.050879	0.164252	-0.03694	-0.08939	-0.26952	0.129988	0.104373	0.112201	0.980943	0.24964
P53814	SMTN	-1.76535	-0.43312	-1.54247	-1.46299	-1.29766	-1.29295	-0.13726	-1.81759	0.350359	-1.60813	-2.29974	-0.9668	-0.29007	-0.7869	-0.48734	-0.86439	-1.17034	-1.15146	-0.2303
P53985	SLC16A1	-0.13475	0.926456	0.509343	-1.50725	-0.23726	-0.97145	0.127489	0.476812	-0.26681	0.155958	-0.68521	-0.22079	1.29399	-0.94223	-1.1907	-1.12768	-0.61116	1.132274	1.731174
P53990	IST1	-0.57026	0.36141	-0.30009	-0.83898	0.122127	0.220518	0.00538	-0.83098	0.250357	0.013829	0.329285	-0.2158	-0.04103	0.204364	0.023661	0.069696	-0.60953	-0.30909	0.08499
P53992	SEC24C	-0.07743	0.461599	0.046285	-0.03161	0.208554	0.180809	0.412249	0.098892	0.249676	0.309803	-0.00066	-0.02633	0.043636	-0.0878	0.62988	0.293417	0.061424	0.129561	-0.15188
P53999	SUB1	-0.26828	-0.61499	-0.01508	0.223145	0.924961	0.399515	0.026939	-0.78366	0.011631	0.844213	-0.51464	-1.13674	-0.03546	-0.64385	1.738102	-0.57288	-0.04516	0.703588	-0.64694
P54098	POLG	-0.09468	0.033848	0.164135	0.1365	-0.34367	-0.24627	-0.18282	-0.18927	-0.23014	-0.00302	0.754467	-0.22082	-0.36739	0.032482	-0.39103	-0.52695	-0.26858	-0.21236	-0.35169
P54105	CLNS1A	0.429509	-0.00884	0.985531	-0.39133	0.037525	0.312685	-0.99368	0.118215	-0.57331	-0.91028	-0.16457	-0.04111	0.680629	0.563194	-0.42728	0.709504	0.793267	-0.20755	-0.67442
P54136	RARS	-0.16804	0.071581	0.08699	0.600348	-0.23992	0.071258	-0.10891	-0.0771	-0.42251	0.088529	0.251529	-0.3245	0.12017	-0.21602	0.604247	0.68121	0.250703	-0.13385	0.121813
P54198	HIRA	0.673578	-0.14384	-0.0244	0.249094	-0.36923	-0.27819	0.00217	0.016632	-0.21545	-0.20939	0.229475	-0.22015	-0.18511	0.040364	0.409215	-0.14516	0.224204	0.351621	-0.34636
P54252	ATXN3	-0.21281	-0.75624	-0.0054	0.02772	0.542819	-0.07081	-0.42447	0.757637	0.014971	0.546579	-0.33417	-0.18248	0.250731	0.333133	0.821645	0.82331	-0.0113	-0.03459	0.130154
P54253	ATXN1	-0.44833	-0.02041	-0.17913	-0.29432	0.592577	-0.24817	-0.2064	-0.22249	-0.30447	-0.39611	-0.11168	0.293792	-0.35936	-0.05828	-0.88235	-0.09726	-0.01931	0.029515	0.143033
P54259	ATN1	0.401613	0.23863	0.030066	-0.40679	-0.76956	-0.4531	0.170046	-0.06933	0.2514	-0.95585	-0.72213	-0.38808	-0.19802	0.108779	-0.3322	0.65659	-0.43321	-0.58907	-0.33792
P54277	PMS1	0.964338	-0.31581	-0.22198	0.463109	-0.19657	-0.18265	-0.13738	0.242477	-0.21405	-0.21705	0.112998	-0.81675	-0.07473	-0.08137	-0.1722	0.539258	-0.60629	-1.19032	-0.23479
P54278	PMS2	-0.15149	-0.24493	-0.06019	-0.53977	0.162957	0.388186	-0.47059	0.37889	-0.16603	-0.15458	0.135095	-0.30793	-0.05226	-0.47835	0.461611	1.057499	-0.03627	-0.18429	-0.34258
P54284	CACNB3	0.142192	-0.15353	-0.29758	0.324811	0.505383	0.466224	-0.21656	0.218189	0.112335	0.166422	1.049525	-0.12664	-0.14337	-0.33501	0.520819	-0.4736	-1.47702	-0.34982	-0.78269
P54289	CACNA2D	0.434408	0.25939	0.081624	-1.33981	-0.72039	-1.37045	0.145758	-1.62679	0.425206	-0.23504	-0.78868	-1.25794	-0.51535	-1.05503	-0.80378	-0.48413	-0.86837	-0.11881	-0.06063
P54577	YARS	0.444103	-0.03917	0.611558	0.833412	0.771045	1.040085	-0.4582	0.633483	-0.34498	-0.2606	-0.09156	0.017617	0.196206	0.002829	-0.12694	1.05999	0.255049	0.357496	0.019919
P54578	USP14	0.19567	-0.05439	-0.12377	0.020114	-0.3638	-0.31408	-0.32676	0.653269	0.041286	0.586922	0.011215	-0.48907	-0.05996	-0.61521	-0.31664	-0.24725	0.449479	-0.02435	-0.01535
P54619	PRKAG1	0.229829	-0.26676	0.153268	0.223739	-0.13369	0.216689	-0.64698	-0.19556	-0.53015	-0.50648	0.018986	0.002438	-0.22058	0.319619	-0.0279	-0.74031	0.174864	-0.63976	0.363235
P54652	HSPA2	-1.02503	0.342003	-0.14079	0.181541	0.692412	0.822555	0.694666	2.13401	0.209713	0.02509	-0.51039	-0.31958	0.38859	-0.53806	0.078463	-0.15796	2.6701	2.500538	0.566387
P54687	BCAT1	-1.44755	-0.96227	-0.96525	-1.21691	-1.01185	-1.16144	-1.16522	0.544385	-0.03244	-1.02588	-0.88723	1.369701	3.162088	-0.36184	-2.34636	3.051575	2.240122	1.520346	-0.40251
P54709	ATP1B3	-0.13504	0.434004	0.14078	0.54472	0.14	-0.03734	0.468098	-0.51857	-0.21441	-0.14	-0.45599	0.261305	0.340749	-0.05004	1.311245	1.285322	0.690334	0.558839	-0.51929
P54725	RAD23A	-0.05084	-0.84676	0.027767	-0.27513	0.474462	-0.28823	-0.24389	0.979389	0.194137	1.180027	0.009836	-0.84573	-0.48564	-0.50057	1.146188	0.766925	-0.18055	0.005063	-0.28963
P54727	RAD23B	0.433425	0.200331	-0.47764	-1.08761	1.044386	-0.04376	-0.47541	0.375333	-0.17752	0.131157	0.234548	0.041761	-0.05899	-0.08867	-0.77936	0.144418	-0.14489	-0.32462	-0.40346
P54753	EPHB3	-0.12645	-0.27305	-0.09052	-0.38203	-0.68231	-0.96801	-0.59449	-0.43792	0.133558	-0.15873	0.096953	-0.44178	-0.31661	-0.23463	-0.39979	-0.59012	-0.7789	-0.23341	0.724458
P54760	EPHB4	0.315581	-0.23628	-0.46196	-0.68886	-0.323	-0.01643	0.874515	0.751157	0.112416	-0.30429	-0.21768	-0.84504	-0.27693	-0.41009	0.346315	0.267478	-0.55603	-0.85306	0.059683
P54802	NAGLU	-0.22563	0.030746	0.061608	0.042297	0.822655	0.993824	0.461527	0.297031	0.212497	0.359166	0.00715	0.551551	0.139266	0.251587	-0.15484	0.112276	0.21192	1.044601	0.666027
P54803	GALC	0.029558	1.061749	-0.03348	-0.08749	-1.35995	-1.24397	0.5405	0.656025	-0.39447	0.187098	0.923275	-0.64406	-0.9188	-0.13684	-0.75799	0.013275	-0.03395	0.535575	0.421971
P54819	AK2	0.314936	-0.83908	-0.16976	0.633232	2.70902	1.311966	-0.91717	0.461806	0.240953	0.921524	0.643843	-0.54278	0.173605	-0.55619	0.520526	1.239142	-0.84097	-0.22355	-0.97256
P54886	ALDH18A1	-0.6374	0.066444	0.1568	0.777141	0.17914	0.115588	0.047729	0.229244	0.105977	0.52751	-0.41401	-0.39898	0.018542	-0.53166	1.528562	1.404541	0.464149	0.57956	0.653083
P54920	NAPA	0.143258	0.66327	0.596453	-0.35322	-0.05899	0.429402	0.397988	-0.30556	-0.19552	0.008831	-0.46644	0.687814	0.119563	0.749216	0.611443	-0.73534	0.290795	-0.12507	-0.55826
P54922	ADPRH	-0.82166	1.038912	0.646893	-0.03816	0.699784	-0.04342	-0.35291	0.062259	0.189198	0.730651	-1.06569	-0.46199	0.965551	-0.14517	0.526801	-0.03973	-0.60167	-0.65902	-0.14379
P55008	AIF1	-1.83388	-0.3492	0.171051	-0.62794	2.773282	-0.34917	0.320638	-1.98418	1.005249	3.331765	-0.57681	0.913099	-0.51614	1.409097	-0.72438	-0.82644	0.286095	0.754603	-2.3322
P55010	EIF5	0.110507	-0.00562	-0.01199	-0.25926	-0.24262	-0.09496	-0.1548	0.185969	-0.16238	0.188861	0.298682	-0.16855	0.339941	-0.15148	0.151626	0.65754	0.545737	0.2571	0.479103
P55011	SLC12A2	-0.78282	-0.24929	-0.20681	-0.59175	-0.65855	-0.71563	0.250103	-0.67911	0.507159	0.008982	1.619342	-0.25446	-0.16468	-0.73163	-0.53408	-0.34462	-0.43829	-0.38636	-0.81998
P55036	PSMD4	-0.08113	0.165382	0.318869	-0.18905	-0.0736	0.075829	-0.49294	0.305576	0.250956	0.243213	0.106985	-0.41322	0.331787	-0.1847	0.002221	0.304378	-0.05329	0.201203	0.184047
P55039	DRG2	-0.54213	-0.02908	0.421966	-0.11234	0.000353	0.459028	-0.27317	-0.20649	0.109515	-0.02387	0.717364	0.263984	0.082682	0.340512	-0.28028	-0.31542	0.284721	-0.15599	0.21743
P55058	PLTP	-1.67449	-0.11305	-0.59749	-0.53507	-0.7669	-0.85454	0.270456	0.687436	0.832648	0.50279	-0.5787	0.426616	0.035789	0.328507	-0.61425	-1.10208	-0.2047	-0.11166	-0.24445
P55060	CSE1L	0.341012	-0.28126	0.004739	0.331819	0.465422	0.973675	0.333679	1.000229	-0.57345	-0.1647	-0.40751	-0.44972	-0.19047	-0.48535	0.397925	0.02638	0.778645	0.255214	0.769645
P55072	VCP	0.192822	-0.89152	0.487617	0.18638	0.390574	0.478725	-0.50699	0.047601	0.243435	0.220403	0.25577	-0.52426	0.096642	-0.33941	-0.63009	0.203358	-0.33764	0.506443	0.130694
P55081	MFAP1	0.595062	-0.00849	-0.55497	0.148549	-0.0668	0.189167	-2.09907	0.333102	-0.04713	-0.50473	0.590288	-0.91555	-1.10761	-0.68363	0.108308	0.027173	-0.18676	0.648836	-0.02769
P55083	MFAP4	-2.22942	-1.89219	-1.17183	-2.70147	-2.35877	-3.73608	-0.31909	-2.16098	-0.05177	-3.55605	-3.10943	-0.65458	-2.80119	-0.89062	0.240713	-3.18918	-1.08998	-2.02675	-2.42629
P55084	HADHB	-0.45788	0.226966	0.203445	1.085641	0.187922	-0.0079	0.125158	-0.06975	-0.3387	0.028973	-0.1352	0.302116	-0.32021	0.260319	0.826896	0.880892	-0.52791	-0.41278	-0.0832
P55145	MANF	-0.38667	-0.15142	0.365617	-0.88237	0.08822	0.250504	0.432558	-0.45489	0.329485	-0.31598	0.297175	1.204514	0.560071	0.571971	-1.42885	-0.25005	-0.30644	0.42183	-0.19382
P55160	NCKAP1L	-1.50973	1.499239	1.242707	-0.67224	1.200064	0.671273	0.143486	-1.60031	-0.25845	1.264818	-1.48523	1.2336	-0.06644	1.438748	-0.525	-0.38376	-0.48721	0.012273	-1.30737
P55196	AFDN	0.107247	-0.77593	0.479005	-0.33247	0.061715	0.194656	-0.3688	0.319775	0.479003	0.764129	0.991788	-0.69862	0.310907	-1.08067	1.3309	-0.24031	-0.58374	-0.62062	-0.45878
P55198	MLLT6	-0.159	0.053717	-0.15812	0.086061	-0.43711	-0.18358	-0.16147	-0.30641	-0.32393	-0.64818	1.196103	-0.46791	-0.08336	-1.15538	-0.556	-0.15025	-1.18242	-0.67072	-0.12142
P55199	ELL	-0.20511	0.151281	0.325916	-0.0385	-0.03347	0.33707	0.079714	0.061639	-0.01562	0.570171	-0.05649	0.366897	0.066662	0.531951	-0.33742	0.474963	0.625635	0.146311	0.230015
P55201	BRPF1	0.666714	-0.06568	-0.00962	-0.77967	0.12745	0.702835	-0.1253	0.007984	-0.09865	-0.4408	-0.22174	-0.47467	-0.11613	-0.10682	-0.00475	-0.21989	-0.01768	-0.08733	-0.16898
P55209	NAP1L1	0.655876	-0.29647	0.337017	-0.01562	0.460807	1.148415	-0.37285	0.317044	0.439021	0.39167	0.05754	-0.14965	0.613628	-0.17208	-0.38094	-0.00532	0.631359	0.746506	0.587345
P55212	CASP6	-0.00286	0.081638	0.387655	0.029647	0.62092	0.517883	0.176068	1.024843	0.21698	-0.06302	0.322084	-0.26594	0.282533	0.354769	-1.15757	0.587339	0.087523	0.459504	0.791633
P55263	ADK	-0.21739	-0.16745	-0.13111	0.473849	1.437334	0.581545	-0.5963	-0.34944	-0.18511	0.000299	-0.09603	0.294833	-0.06269	0.751773	0.55055	0.063169	0.762038	-0.36234	-0.62155
P55265	ADAR	0.46217	-0.00854	0.60099	0.11724	-0.20187	0.015915	-0.25911	0.979526	-0.16291	-0.1475	-0.1489	-0.18152	-0.10946	-0.10901	0.533248	-0.37047	0.020094	0.102261	-0.28721
P55268	LAMB2	-0.47017	-0.9496	-1.04809	-1.51294	-0.67478	-1.52826	0.207485	-0.54637	-0.3346	-0.31417	-0.16615	-1.02817	-1.13689	-0.71148	0.904479	-1.34824	-0.64693	-0.31019	-1.82485
P55285	CDH6	-0.67914	-0.55833	-1.92982	0.297473	1.153025	1.15495	1.073039	1.41731	-0.40868	-1.95391	1.053066	-0.50454	2.642528	-1.15154	-1.45542	0.467699	-2.11805	-2.9992	-0.34372
P55287	CDH11	-1.55135	0.117898	0.026067	-1.24706	-0.84406	-1.23793	0.404132	-0.88305	0.385818	0.269923	-0.66959	-1.03896	0.048525	-1.12224	-1.04101	0.224455	-0.63775	0.888033	1.258044
P55290	CDH13	-0.40554	-0.68272	-0.64601	-1.08566	-0.70395	-1.72251	-0.62744	-1.42805	0.618454	-0.50123	-0.80252	-1.42806	-0.51238	-0.61546	-0.69394	0.008891	-0.81715	-1.08051	-0.78644
P55327	TPD52	0.221782	-0.57812	-1.43328	-0.68647	0.286423	-0.25372	-1.38895	-0.66101	-0.45299	-1.65307	-0.38545	0.895967	-0.87211	-0.29795	-1.41759	0.939972	0.435574	-0.60753	-1.26692
P55345	PRMT2	-0.10241	-0.28822	-0.72332	-1.49806	-0.43712	-0.52527	-0.45985	-0.88267	-0.20082	-0.76918	-0.16978	-0.83953	-0.41567	0.06071	-0.53891	-1.01147	-0.67732	-1.52808	0.571053
P55735	SEC13	-0.41448	0.618488	-0.09076	-0.49788	0.133296	-0.17681	0.713303	-0.07678	-0.06569	0.012836	-0.1932	-0.50526	0.686518	-0.03916	0.286824	0.264832	0.119073	0.359372	1.026345
P55769	SNU13	0.170358	-0.77282	-0.71309	-0.25544	0.145641	-0.41657	-0.74447	-0.69534	-0.10861	-0.17933	0.820957	0.19532	-0.12179	0.027281	0.237091	-0.73225	0.270256	-0.26719	-0.65615
P55786	NPEPPS	-0.38297	0.115127	0.19593	0.809446	-0.30388	-0.01661	0.073967	0.320268	0.006416	-0.00628	-0.26854	-0.66737	0.373395	-0.71154	-0.48668	0.47666	0.113804	0.118159	0.644309
P55795	HNRNPH2	0.29657	0.153747	0.247748	0.41559	0.014501	0.257341	-0.22616	0.274195	5.08E-05	0.119137	0.287046	-0.08995	-0.3557	0.248836	-0.54291	0.010705	0.135737	0.283495	0.007461
P55809	OXCT1	1.385258	-0.82452	-0.44901	-1.07244	0.560055	-0.33398	-0.61759	-0.87453	0.137006	0.082203	0.313453	-0.28684	-0.14492	-0.23628	1.101696	0.056256	-0.69252	-0.34504	-0.80086
P55884	EIF3B	-0.32035	0.331145	0.310731	-0.02855	-0.15548	0.342772	0.093739	0.260714	0.051894	0.310372	0.226166	-0.4648	-0.37785	-0.41662	0.565327	0.177615	0.182157	-0.32183	-0.12741
P55899	FCGRT	-0.71045	2.131844	0.074025	-0.52201	-0.09091	-0.18632	1.733602	-0.75753	0.475112	0.1664	-0.69301	2.198622	0.244899	1.853721	-0.55684	-0.30375	0.010382	0.308911	-0.5697
P55957	BID	0.083349	0.188244	0.464846	0.002545	-0.5425	-0.42145	0.200991	-0.21135	0.759859	0.74044	0.224024	0.079081	0.091557	0.328626	0.380858	-0.32254	-0.22958	-0.3804	-0.9635
P56182	RRP1	0.332022	0.110055	-0.99044	0.401878	0.176744	0.497134	0.004514	0.118752	0.348079	0.093227	0.526492	-0.21206	0.124955	-0.56677	-0.23235	-0.07258	0.112643	-0.27257	-0.58424
P56192	MARS	0.248367	0.051136	-0.24526	0.332471	-0.02506	0.204955	-0.15852	-0.33387	-0.11456	-0.14597	-0.02931	-0.21434	-0.14239	0.099322	0.653121	0.587433	0.221137	-0.2839	-0.05072
P56199	ITGA1	-1.04567	0.131967	0.065247	-1.38204	-0.29414	-0.95333	-0.20866	-1.53314	-0.05472	-0.14078	-1.02307	-1.61815	-0.17048	-1.27112	-0.37965	0.428258	-1.74143	1.28976	0.191476
P56377	AP1S2	-0.418	0.607034	0.151311	-1.37107	0.289911	-0.29029	-0.38609	-0.56316	-0.20141	0.450959	-1.11351	0.84744	-0.34411	1.022523	-0.46851	-0.65902	0.612461	0.47418	0.018821
P56378	MP68	-0.02481	-0.25141	-0.17136	0.32744	0.038369	0.48884	-0.10858	-0.34518	-0.08713	-0.79411	0.839284	-0.09849	-0.32876	-0.01482	1.465682	0.830651	-0.27492	-0.32619	-0.431
P56385	ATP5I	-0.07522	0.25391	0.130643	0.438805	-0.04821	-0.27189	0.34849	-0.78131	-0.2512	-0.00884	0.318187	-0.1522	-0.6356	0.06917	1.167759	0.380265	-0.69316	-0.49741	-0.70058
P56524	HDAC4	0.182198	-0.01976	-0.04096	-0.35476	-0.54367	-0.21462	0.138452	-0.15242	-0.47357	0.135892	0.414437	-0.03299	0.067248	0.063727	-0.21042	0.1329	-0.19112	0.141404	-0.07269
P56537	EIF6	-0.31453	0.226814	-0.33526	0.088423	0.19413	-0.17758	0.513164	0.141313	0.602627	-0.08	0.128998	-0.16553	0.305473	0.190805	0.233775	-0.33192	0.440898	0.170036	-0.36696
P56545	CTBP2	0.221606	-0.17864	-0.14307	0.409893	0.496152	0.584801	-1.04049	1.085999	-0.8738	-0.20088	0.658771	0.173095	0.714443	-0.32206	0.921165	0.627383	0.376007	0.239724	-0.02644
P56556	NDUFA6	0.139238	-0.38412	0.122578	-0.29583	0.825726	0.156017	-0.11096	-0.38844	0.037722	-0.43819	0.787062	-0.24682	-0.06287	-0.0642	1.112148	0.175377	-0.75674	0.385612	-0.64077
P56559	ARL4C	0.457674	-0.15668	0.098007	-0.14604	0.334414	-0.13392	-0.33913	-0.12528	0.079793	-1.46455	-0.74468	-0.52412	0.085953	-0.40605	0.160933	-1.11399	-0.35736	-1.68445	-0.16439
P56589	PEX3	-0.01808	0.530445	0.613729	1.15189	0.278377	0.151767	0.513953	0.049814	0.679064	0.417364	-0.08308	-0.03664	-0.40032	-0.06387	-0.03092	0.012687	-0.34186	0.149675	-0.59782
P56705	WNT4	-1.12346	-0.43838	-0.30479	-1.55886	-2.1377	-1.26393	-0.05062	-2.05528	0.517416	-0.45995	-1.62654	-2.11616	-0.17311	-0.33026	-0.38819	-1.24359	-0.98959	1.391251	1.137284
P56747	CLDN6	2.33451	-0.32936	-1.24639	-2.02983	-4.1037	-2.79476	-0.04852	1.888923	0.210851	-3.95252	-2.35703	-0.91828	0.101233	-0.55126	-1.90333	-2.14635	1.833419	2.15356	2.77318
P56937	HSD17B7	0.448314	-0.24039	0.038413	0.044495	0.149322	-0.28131	-0.1708	-0.37704	-0.04699	-0.16648	0.099977	-0.0215	-0.0646	-0.25093	0.117202	0.609665	-0.64642	-0.79565	0.38846
P56945	BCAR1	-0.18576	0.222437	0.530907	-0.7651	-0.45612	-0.17282	0.766416	0.093756	0.771749	-0.01574	-0.01234	-0.95607	0.712122	-0.69206	-0.06132	0.157621	-0.84426	0.189754	1.108342
P56962	STX17	0.284867	0.239635	-0.38009	-0.35205	-0.30279	-0.11756	0.319399	-0.37218	-0.41141	-0.74841	0.001296	-0.01696	-0.00377	0.207532	0.057573	-0.04629	-0.16248	-0.36723	0.020775
P57060	RWDD2B	0.259157	0.304246	0.038564	-0.24091	0.628987	0.468604	0.32486	0.186343	-0.41518	0.118834	0.465179	0.507741	-0.29661	0.312601	-0.31383	-0.31451	-0.08829	0.560452	-0.93309
P57076	C21orf59	0.817389	-1.14328	-0.48524	-0.5081	0.075829	0.396811	-0.16403	-0.4534	-0.88656	0.069912	1.145099	-0.38677	0.96463	-0.29594	0.23069	-0.08829	0.109246	-0.25339	-0.55014
P57081	WDR4	0.236879	0.174269	-0.18171	-0.49269	0.58741	0.410352	0.246243	0.129303	0.659989	-0.18873	-0.3797	0.3216	0.2923	-0.12924	-0.01821	0.146158	0.543467	-0.78501	-0.25302
P57088	TMEM33	0.378997	0.572071	0.834109	-0.37424	-0.25389	-0.40885	1.064875	-0.26037	-0.01716	0.183621	0.441112	0.301086	-0.54965	0.362223	-0.09196	0.057396	-0.36968	-0.73936	-0.37684
P57105	SYNJ2BP	-0.09631	0.289842	-0.88691	0.513629	-0.45705	-0.43871	0.608229	0.056132	0.228926	-0.00982	0.664714	-0.28352	0.058787	0.178827	0.977388	0.930265	0.129062	-0.01534	0.124008
P57678	GEMIN4	0.072003	0.283158	-0.18095	0.319908	0.328086	0.45891	0.158068	0.263202	-0.31153	-0.44251	0.692846	-0.30864	-0.25003	-0.18628	0.018307	-0.3321	0.066667	-0.47377	-0.13198
P57727	TMPRSS3	1.272314	-0.44281	-0.30199	-0.09628	-0.21273	0.27543	-0.01668	1.370373	-0.38147	-0.90141	0.066387	-0.44566	-0.3005	-0.33822	1.080319	-0.53296	-0.65122	-1.00819	-0.97173
P57735	RAB25	1.020338	-0.56226	-0.77986	1.166784	-0.44924	-0.31852	-0.84127	-0.81616	-0.3416	0.178939	1.79423	-1.52834	-1.22995	-0.42932	1.911853	-1.96952	-0.36867	-2.47386	-2.06877
P57737	CORO7	-1.33838	1.244788	0.951875	-0.76482	0.267519	-0.22163	0.176736	-0.91116	0.198575	1.15494	-0.6449	1.2876	0.083892	1.806585	-0.54502	-0.31862	0.411103	0.085135	-0.62565
P57740	NUP107	0.067631	0.031396	-0.29886	0.235738	0.144076	0.231373	0.163048	0.255367	-0.13447	-0.11049	0.325668	0.168893	-0.07334	-0.06271	0.688561	-0.19371	0.052153	-0.15777	0.394181
P57764	GSDMD	0.802709	-0.12263	-0.00703	-0.60708	0.547235	0.52119	-0.09499	-0.44543	0.122997	0.247486	-0.51848	0.511751	0.425542	0.694356	-1.06081	0.47117	0.026265	-0.45568	-0.57854
P57772	EEFSEC	0.297937	-0.09108	0.163069	0.437351	1.054423	0.794953	0.075373	0.17046	0.001832	0.338754	-0.12059	0.594511	-0.33072	0.242766	0.27613	-0.40875	0.255062	0.022423	0.127028
P58005	SESN3	0.160959	-0.0468	-0.49455	-0.22092	-0.78057	-0.90541	0.303924	-0.96959	-0.00991	0.111952	0.897925	-1.17299	0.015712	-1.50367	1.190543	-0.66073	-0.49767	0.093647	-0.12107
P58107	EPPK1	1.969911	-0.82957	-2.3505	0.480352	-1.36585	-0.90463	-0.94067	0.251338	-0.68031	-1.17001	0.290023	0.398035	1.147882	-0.67007	-1.02389	-0.77616	-0.69576	-2.24019	-1.55268

P58335	ANTXR2	-2.03146	0.169805	-0.39349	-0.56788	0.207891	-0.63448	0.149241	-0.88416	0.292515	0.060557	-0.34769	0.269525	0.319301	0.302339	-0.67426	-0.03954	-0.42811	0.357639	1.230088
P58340	MLF1	-0.23334	-0.36986	-1.40695	-1.18992	0.891655	0.290003	-1.24668	-0.41233	-0.39474	-1.60564	2.897631	-1.53888	-0.56165	-0.19939	-0.48384	-0.87816	-1.42295	-0.64353	-0.67214
P58546	MTPN	-0.27845	-0.5855	-1.5479	-0.95857	0.767439	-0.68599	-1.65262	0.253126	0.040847	2.217883	-0.16669	-0.03145	-0.16001	0.503653	0.104359	-0.23671	-0.19306	-0.33273	-0.62277
P59998	ARPC4	-0.25107	0.377893	0.108136	-0.37368	0.420705	0.305991	-0.65553	-0.00645	-0.03021	0.560393	-0.21286	-0.26555	0.147919	0.714446	-0.43386	0.239009	-0.50887	-0.18411	0.449296
P60033	CD81	-1.43776	0.215166	-0.68175	-1.49828	-0.2795	0.386918	0.014444	-1.14245	-0.47261	-0.5765	0.058379	-0.17184	-0.61397	-0.41515	-0.12288	-0.50829	-0.55385	-0.22283	-0.80574
P60174	TPI1	0.494579	-1.51716	-1.11956	0.047342	0.73984	-1.2311	-0.03841	-0.59965	1.585327	1.28975	-0.88078	0.03914	1.006387	-0.09283	-0.25207	0.524437	0.643817	-0.62094	0.262982
P60228	EIF3E	-0.33843	0.258512	-0.01695	-0.16311	-0.06438	0.385934	-0.04941	0.115711	-0.0618	0.044137	0.316004	-0.43951	-0.098	-0.24782	0.508554	0.181729	-0.04521	-0.41476	-0.11685
P60468	SEC61B	-0.19764	1.049404	-0.05156	0.400746	-0.17042	0.154968	1.019501	-0.00252	0.422622	0.114687	0.098237	-0.18412	0.51411	-0.36269	0.961158	0.303177	0.405118	0.602604	0.559272
P60484	PTEN	0.164649	0.455292	0.542397	-0.9455	0.125474	0.476654	0.015535	-0.29941	0.033815	0.310983	-0.50354	-0.11332	-0.0341	0.695865	-0.10307	0.341262	-0.08099	0.134229	-0.0828
P60510	PPP4C	0.302662	0.41606	-0.14882	0.076307	0.751543	-0.01808	-1.16789	0.516539	0.802162	0.372147	0.160393	-0.26194	0.04832	0.285816	-0.3755	0.271639	0.299655	0.19541	-0.04746
P60520	GABARAF	0.124565	0.364384	0.252832	-0.21017	0.32693	-0.57919	-0.17344	-0.4975	-0.09099	0.76384	0.150238	-0.09043	-0.31051	0.328579	-0.60248	-0.28809	-0.24618	0.017129	-0.32397
P60660	MYL6	-0.5757	0.269606	-0.64527	-0.31672	-0.23689	-0.13841	-0.23318	-0.79266	0.399839	-0.09737	-0.68884	-0.27639	0.277644	0.371151	-0.81873	0.100618	-0.47573	-0.54041	0.180719
P60842	EIF4A1	0.281476	0.193968	0.203503	-0.00585	0.265156	0.621167	-0.21567	0.061021	-0.14817	0.275191	-0.24489	0.324752	0.424054	0.422021	-0.28887	0.009308	0.308839	-0.18907	0.401182
P60866	RPS20	0.015081	-0.22594	-0.11541	-0.08915	-0.30974	0.264214	-0.121	0.204735	0.930681	0.238277	0.154673	0.643705	0.218864	0.493346	0.463341	0.703162	0.429671	-0.4768	0.254951
P60880	SNAP25	1.573884	0.496438	0.180527	-1.09054	-0.61627	-0.3887	0.237608	-0.48436	0.080252	0.837422	-0.93821	-0.37271	0.385915	-0.8318	-1.06037	0.838705	-0.09285	1.639685	0.986213
P60891	PRPS1	0.004149	0.143202	0.518518	-0.18175	0.499026	1.03932	-0.07878	-0.20636	-0.10166	0.085518	0.188128	0.288932	0.093715	0.493776	-0.17772	-0.05114	0.350458	0.347423	-0.1237
P60900	PSMA6	0.090943	-0.57564	0.427997	0.020381	0.509088	0.602336	-0.33389	0.231871	0.435233	0.08626	0.199721	-0.68918	-0.14348	-0.2775	-0.01003	0.548186	-0.48903	-0.01249	-0.06676
P60903	S100A10	-0.91463	-0.11748	-0.10726	-1.09494	-0.51188	-0.84683	0.344275	-0.60879	-0.3687	-0.72503	-0.38999	0.594193	-1.07679	0.19911	-1.44519	-1.26781	0.481035	-1.27281	-0.40377
P60953	CDC42	0.142974	-0.67993	-0.01783	-0.03068	0.689438	-0.11634	-0.20818	-0.15951	0.334523	0.378137	-0.00689	1.477048	0.226391	1.134847	-0.0887	-0.25283	0.54412	0.129816	-0.36706
P60981	DSTN	-0.22698	-1.49926	-0.50435	0.206816	-0.1119	-0.61284	-0.72581	-0.16875	0.249184	-0.34421	-0.16746	-0.0836	-0.0947	0.003427	-0.05904	0.274098	0.874564	0.607929	0.724298
P60983	GMFB	0.275876	0.133134	-0.5846	-0.46358	0.336651	-0.23152	-0.69436	0.036904	0.239146	0.203691	-0.09714	-0.43586	0.251433	-0.48852	-0.54638	0.445928	0.04335	0.406773	0.614837
P61006	RAB8A	-0.7644	-0.17053	1.180131	-0.02942	-0.05592	-0.17749	0.802339	0.740346	-0.63607	0.18371	-0.85694	0.982491	-0.47289	0.779775	-0.62885	0.263179	0.466867	-0.09639	-0.37982
P61009	SPCS3	-0.63525	0.795312	0.298889	0.031452	0.334115	-0.54387	0.407714	-0.35849	-0.03284	-0.369	-0.09225	0.28104	0.299807	0.19769	-0.82861	-0.2376	-0.2958	0.715707	-0.03831
P61011	SRP54	-0.29099	0.189837	0.097586	-0.37063	-0.00073	0.120787	-0.09682	-0.42106	-0.32611	0.106777	0.323183	0.242288	0.298613	0.023954	-0.58079	0.447219	0.671686	0.672663	0.129899
P61018	RAB4B	-0.77677	0.228429	0.793633	-0.52823	-0.02355	-0.36568	0.456392	0.035293	-0.36105	0.305753	-0.12276	0.853877	0.203952	0.86304	0.658557	-0.33663	-0.39244	0.115689	0.180097
P61019	RAB2A	-0.09806	-0.48233	0.006984	-0.56668	0.047583	0.265545	0.267261	-0.28002	-0.02439	-0.25024	-0.14443	0.618926	0.913917	0.173187	0.102977	0.490361	0.489425	0.244495	0.287831
P61020	RAB5B	-0.02877	-0.17272	0.276008	-0.00166	0.227812	0.066467	0.033031	-0.19888	0.100612	-0.07783	-0.13712	0.231319	0.250859	0.276966	0.367904	-0.36929	-0.26733	-0.36256	0.07835
P61024	CKS1B	0.903884	-0.16707	0.265341	0.077893	0.263047	2.421784	0.709773	2.443474	0.272796	-0.25241	-1.09231	-0.21379	0.137198	-1.12744	-0.62931	-0.02855	0.12929	-1.22954	-0.28324
P61026	RAB10	-0.12003	-0.07579	0.019797	0.608493	0.502708	-0.01566	0.230569	-0.11357	-0.71792	-0.07175	-0.53215	1.153857	-0.39591	0.77296	0.349068	0.469268	-0.11189	-0.75326	-0.21988
P61077	UBE2D3	-0.31244	0.255996	0.21989	0.064021	0.258915	0.045433	0.155971	0.150425	-0.09576	0.154284	-0.06019	0.020709	0.223135	0.450179	-0.20813	0.263167	0.46758	-0.58515	-0.00391
P61081	UBE2M	0.471465	-0.10265	-0.04513	-0.19034	-0.19638	0.107257	-0.29795	0.483296	-0.68301	-0.18559	0.340394	-0.41244	0.007423	-0.39212	1.073335	-0.00712	0.026356	-0.73889	-0.20773
P61086	UBE2K	-0.22074	0.079021	-0.67829	-0.75482	0.405062	0.118417	-0.71335	0.622779	0.457848	0.500431	-0.205	-0.66243	-0.06212	-0.71893	-0.62733	-0.02826	-0.3484	-0.67639	-0.36871
P61088	UBE2N	0.070319	0.029271	-0.58158	-0.06707	0.166303	0.016147	0.002489	0.265665	-0.18224	-0.35561	0.213462	-0.78488	0.439105	-0.36366	-0.03424	0.047977	-0.3475	0.074249	0.131352
P61106	RAB14	-0.19102	0.427173	-0.11923	-0.43654	0.036489	-0.38844	0.14143	-0.38887	-0.31438	-0.04046	0.34329	0.575708	0.007562	0.438462	-0.14797	-0.16844	0.095097	0.091139	-0.13376
P61158	ACTR3	-0.35502	0.418243	0.653105	-0.39012	0.201774	0.080122	-0.61155	-0.09088	0.079746	0.317708	-0.14919	-0.34352	0.509103	0.696116	-0.29168	-0.01642	-0.32666	0.114764	0.197203
P61160	ACTR2	-0.37492	0.20642	0.762445	-0.2554	0.167292	0.024028	-0.17635	0.014603	-0.09314	0.427638	-0.30719	-0.22413	0.277672	0.685382	-0.33319	0.157938	-0.38779	0.056018	0.310001
P61163	ACTR1A	-0.12618	0.022596	0.410757	-0.2765	-0.09699	0.048486	0.074238	0.047811	0.181264	0.130858	-0.80181	-0.10907	0.085882	0.465214	-0.03671	-0.14989	-0.33209	-0.30143	0.522518
P61201	COPS2	-0.0497	-0.20482	0.111581	0.053778	-0.42486	0.014239	-0.48159	-0.14771	-0.17618	-0.15487	0.382532	-0.90436	-0.41499	-0.50574	0.312222	0.349976	-0.47148	-0.40601	0.307236
P61221	ABCE1	-0.11486	-0.15218	0.242788	0.419072	0.464679	0.420996	-0.471	0.002912	0.387766	0.182801	0.607205	0.169422	-0.11625	-0.07411	-0.66589	0.389668	0.103324	-0.08808	0.255447
P61225	RAP2B	-1.20576	1.613281	1.096966	-0.49118	1.109674	0.592537	0.783685	-1.14405	-0.28094	0.348855	-0.53509	1.441953	-0.46011	0.868631	-0.61725	-0.83972	-0.38599	0.178331	-0.54924
P61244	MAX	0.264441	-0.12969	-0.32148	-0.55334	-0.24556	0.194397	-0.13999	0.179843	0.115835	-0.8599	-0.10196	-2.15826	-0.21076	-0.93149	-0.04495	0.008981	-0.88652	0.272836	-0.4477
P61247	RPS3A	0.223091	-0.28752	-0.09874	0.057269	0.126262	0.187456	-0.65556	0.112628	-0.18278	-0.1191	0.427993	0.09131	0.196665	-0.03345	-0.09773	0.195906	0.124951	-0.17668	0.025047
P61254	RPL26	0.156048	-0.22009	-0.27334	-0.12994	-0.38161	-0.07498	0.000191	-0.03491	-0.16841	-0.06673	0.494655	-0.02104	-0.29514	-0.19867	0.444287	0.416769	0.13301	0.190257	-0.44828
P61289	PSME3	-0.12309	-0.19608	0.610014	0.853074	0.809226	0.721976	-0.32886	0.385466	-0.55945	0.244811	0.090097	-0.3046	0.1276	-0.48985	0.291478	0.470972	0.10606	0.315637	0.416732
P61313	RPL15	0.346713	0.0486	0.30424	0.493364	-0.04253	0.168568	0.049758	0.235969	-0.67998	-0.56324	0.570461	0.209641	-0.35614	-0.03654	-0.01245	0.065153	0.193396	-0.20011	0.118286
P61353	RPL27	0.322919	0.024071	-0.17656	0.460402	-0.78774	0.405867	-0.01404	0.084867	-0.06744	-0.94206	0.391973	-0.64785	-0.02943	-0.82332	0.610343	0.557508	-0.81525	-0.28613	-0.09638
P61421	ATP6V0D	-0.20509	0.925521	0.918766	-0.29726	-0.17126	-0.53434	0.455136	-0.75218	-0.72798	0.358196	-0.65469	1.416675	0.109102	1.387609	-0.25681	0.668814	0.932615	0.349249	-0.65411
P61457	PCBD1	0.859974	-2.4024	-0.97235	0.854528	1.69526	1.444346	-1.05043	0.536671	-0.8894	0.428123	1.093002	-0.50775	-0.04795	-0.61143	1.369481	1.564307	-0.1387	0.026029	-1.01973
P61513	RPL37A	-0.14698	0.300942	0.130264	-0.06974	0.29175	0.877963	0.247016	-0.13296	0.070472	0.17882	0.295174	0.494777	0.18451	0.733342	0.585606	0.053804	0.290596	-0.37245	-0.18522
P61586	RHOA	0.075519	-1.82556	-0.50312	-0.22694	0.851433	-0.023	-1.22489	-0.03341	-0.16069	0.503904	-0.06447	1.177384	0.112009	1.461531	-0.11157	0.297646	0.169345	0.079976	0.401988
P61599	NAA20	0.268346	0.894028	-0.13161	-0.31257	-0.1424	0.054038	0.092878	-0.04402	0.131921	0.247406	-0.0259	-0.10366	-0.28853	0.536545	0.604237	-0.02308	-0.35109	-0.27179	-0.08365
P61601	NCALD	-0.49959	-1.31092	-0.00259	-0.50342	0.187447	-0.11824	0.080862	-0.73836	-0.02668	-1.01037	0.921479	0.67253	0.801886	0.063473	-0.60767	-0.03162	-0.04254	-1.00897	-1.50072
P61604	HSPE1	0.962849	-2.90961	0.079385	0.688779	1.173751	1.048665	-2.1989	0.606256	-1.17142	-0.95959	0.420708	-0.69386	-0.5046	-0.74876	1.027152	2.325882	-0.53821	-0.61788	-1.76233
P61619	SEC61A1	-0.12228	0.451019	0.129327	0.249181	0.126422	0.105129	0.855387	-0.05575	0.323616	0.211721	0.166668	0.378379	0.059456	-0.29278	0.92328	0.12324	0.262489	0.162569	0.743824
P61626	LYZ	-2.44185	0.854449	1.867832	-3.09301	-1.96759	-1.79729	0.5069	-1.5675	0.462323	2.866244	-1.93609	0.008331	-1.09009	1.099334	0.300505	-2.25172	0.074112	-1.35177	-1.20623
P61758	VBP1	0.404208	-0.70804	0.206524	-0.06102	0.921751	0.704576	-0.85735	0.551976	0.846974	0.311277	-0.32958	-0.21909	0.187907	-0.43627	-0.01619	0.617316	-0.25431	-0.09966	-0.10592
P61764	STXBP1	0.313454	-0.33794	0.074714	-1.00927	-0.37972	-0.19716	0.040535	-0.63403	0.267414	-0.1457	-0.29469	-0.75929	-0.56979	-0.67662	0.201883	-0.27091	0.596559	1.136206	-0.6164
P61769	B2M	-0.31097	0.896913	1.428962	-1.39547	1.715853	-0.56312	0.602007	-1.44893	0.180362	-1.02882	-0.7578	1.832071	0.203563	1.514195	-0.43659	-1.03103	0.798215	0.927732	-2.8428
P61803	DAD1	0.29353	0.544855	-0.11091	-0.46513	0.051493	-0.48507	0.834355	-0.58396	0.255766	-0.06111	0.326477	0.378166	0.040046	0.035804	0.267201	-0.11062	0.022021	0.022897	0.364379
P61812	TGFB2	-0.23389	-1.32363	-1.41416	-2.07708	-1.91458	-2.65974	-0.41697	-0.43877	-0.40434	-1.34372	-0.65995	-2.12942	-1.27413	-0.58968	1.098001	-2.4999	-1.55715	-2.27685	-1.32094
P61923	COPZ1	-0.23756	0.356973	-0.13584	-0.21171	0.024409	0.215739	0.043597	0.003428	0.194491	0.439129	0.338536	-0.11273	0.526019	0.169179	0.349988	0.834689	0.3604	0.348511	0.335419
P61927	RPL37	0.419497	-0.13257	-0.3398	-0.32746	-1.26118	-0.08421	-0.23116	-0.50577	0.05211	-0.72807	0.2827	-0.61124	-0.02151	-0.65414	0.273671	0.26732	-0.08813	-0.5212	-0.79199
P61956	SUMO2	1.719434	-0.90825	0.745904	-0.31378	-0.51358	-0.57379	-0.32562	-0.58727	-0.01918	0.830955	0.457909	-0.55416	0.659202	-0.58648	0.796238	0.050671	-0.5337	-0.06789	0.501767
P61960	UFM1	0.026552	-0.55411	0.411791	0.255663	0.762921	1.018901	-0.40998	-0.12435	0.183086	0.903417	0.278037	-1.05449	0.474544	-0.64236	0.064629	0.774595	-0.77559	-0.34273	-0.25897
P61962	DCAF7	0.50977	-0.05745	0.151144	0.723961	0.283502	0.582731	0.347748	0.982724	-0.24654	-0.43466	-0.08719	-0.13525	-0.12524	0.203051	-0.20054	0.103126	0.213907	-0.03067	0.429939
P61964	WDR5	0.828466	-0.12969	0.146096	0.248738	0.519145	0.343571	-0.26187	0.808152	-0.1828	-0.27636	0.545277	-0.04907	-0.18602	-0.15748	0.229777	0.214165	0.18649	0.182639	0.098909
P61966	AP1S1	-0.71868	0.3504	-0.37553	0.271777	-0.18224	-0.07502	0.258143	0.025697	1.257238	0.320205	0.311928	-0.57864	-0.12647	-0.06864	0.073734	0.390979	-0.53699	-0.739	0.3234
P61970	NUTF2	-0.94912	0.140516	-0.20952	-0.84395	0.321218	0.053268	-0.21087	0.691431	-0.61131	-1.05955	-0.36109	-0.37309	-0.1016	-0.24099	-0.54017	-0.05693	0.239135	-0.41857	-1.03996
P61978	HNRNPK	0.305106	-0.11925	0.130073	0.014897	0.374958	0.78257	-0.32574	0.598566	-0.10424	0.075568	0.608285	-0.56117	-0.13856	-0.4751	-0.07255	-0.03867	-0.24735	0.327374	0.131335
P61981	YWHAG	0.186085	-0.29649	0.395466	-0.43045	0.09339	0.284538	-0.28617	0.246191	0.176502	0.503083	0.057729	-0.81653	0.352606	-0.2626	0.364847	-0.19938	-0.18703	0.099671	0.313126
P62068	USP46	0.279305	0.158345	0.4185	-0.15241	-0.24218	-0.07185	0.042895	-0.1312	-0.00488	-0.27981	0.092826	0.016531	0.117492	0.023006	-0.12356	0.474689	0.50367	-0.45328	-0.36515
P62070	RRAS2	-0.17649	-1.18238	-0.70769	-0.68326	0.271085	-0.31797	0.125734	0.183087	0.22373	-0.47555	0.039074	-0.2906	-1.15758	-0.29213	-0.12004	0.28007	0.596076	0.389885	-0.01677
P62072	TIMM10	0.512659	0.207122	-0.69708	-0.10513	0.124944	0.059432	0.181073	0.16129	0.612465	0.04104	0.402231	-0.30755	0.083143	-0.1014	0.148069	-0.15851	-0.42152	-0.31802	-1.19039
P62081	RPS7	0.408762	-0.23231	-0.02412	0.276647	0.043548	0.357068	-0.41845	0.176049	-0.11852	-0.1827	0.546243	-0.58506	0.065367	-0.60283	0.074496	0.272716	-0.267	-0.12681	0.1829
P62136	PPP1CA	0.223773	-0.15573	-0.05333	-0.03753	0.619345	0.368072	-0.28673	0.465558	0.165285	-0.04581	-0.06784	0.202817	0.05825	0.340241	-0.05839	-0.03581	0.542454	0.321628	-0.28182
P62140	PPP1CB	0.660512	-1.29612	-0.27853	0.061938	-0.06514	-0.21949	-1.19414	0.255687	0.503487	-0.17267	0.490619	-0.01174	0.052521	0.534945	0.196648	0.232526	0.363086	-0.07931	0.258411
P62166	NCS1	0.226931	-0.14723	0.064526	0.067187	0.625069	0.308963	0.173543	0.334551	0.0734	0.150991	-0.35567	0.01822	-0.14854	-0.36585	-0.11892	0.396977	-0.25501	-0.7577	0.71241
P62191	PSMC1	-0.08883	0.372486	0.428641	0.156265	-0.01782	0.455076	0.031002	0.131588	-0.02953	0.172093	0.044709	0.257683	0.316337	0.136216	-0.02469	0.326059	0.276942	-0.14088	0.354705
P62195	PSMC5	-0.0255	0.120056	0.379222	0.050944	-0.1347	0.078883	-0.0535	0.021603	0.162432	0.097564	0.022904	0.308924	-0.02848	0.340424	-0.08033	0.277286	0.288489	-0.05769	0.296324
P62241	RPS8	0.349328	-0.2527	-0.11587	0.410596	0.08124	0.113922	-0.94979	0.086471	0.035684	-0.34443	0.435114	0.010348	-0.17235	-0.13393	-0.11482	-0.02519	0.162417	-0.11211	0.044125
P62244	RPS15A	0.308181	-0.22181	0.19994	0.249619	-0.05942	0.162645	-0.58196	-0.0492	0.535624	-0.19753	0.396674	0.177333	0.524666	0.121218	0.018781	0.121751	0.317017	-0.02811	-0.03025
P62249	RPS16	0.209335	-0.6973	-0.4485	0.305136	0.141145	0.165752	-1.45724	0.175522	-0.52113	-0.13414	0.287663	0.593588	-0.18164	0.323331	-0.05954	0.329808	0.283175	-0.4691	0.301324
P62253	UBE2G1	0.459726	0.251985	0.24237	-0.58038	0.162173	-0.57581	-0.14025	0.673269	0.270916	0.966545	-0.19021	-0.27723	-0.09356	-0.3815	-0.45206	-0.6583	-0.04522	0.225983	0.296793
P62256	UBE2H	-0.33297	-0.37486	0.069132	0.83835	1.179317	1.383273	-0.21217	1.330453	0.005878	-0.74292	-0.33969	0.007698	0.127425	-0.08591	0.993152	0.384877	-0.15804	-0.67751	0.776856
P62258	YWHAE	0.752117	-0.86499	-0.12422	-0.10755	0.454269	0.542303	-0.44706	0.782708	0.324074	0.458913	0.596733	-0.63444	0.518682	-0.72831	-0.08337	0.053963	-0.75321	-0.21635	0.601066
P62263	RPS14	0.176025	-0.35151	-0.30911	-0.04043	0.221918	-0.27441	-0.47519	-0.446	-0.03206	0.027645	0.316036	-0.0664	-0.12174	-0.26085	-0.22851	0.046387	0.197698	-0.17016	-0.26874
P62266	RPS23	0.38391	-0.36921	0.523008	0.378537	-0.62032	0.049238	-0.11474	0.055564	-0.15085	-0.05427	0.438083	0.442246	-0.4432	0.261611	0.314951	0.080127	0.747606	-0.26306	-0.57388
P62269	RPS18	0.014235	-0.16493	0.086506	-0.06806	0.017192	0.262326	-0.21456	0.179385	0.037331	-0.25569	0.633401	-0.23948	-0.11928	-0.47256	-0.13786	0.116759	-0.27652	-0.13121	0.294807
P62277	RPS13	0.256541	-0.30019	-0.29129	0.364873	0.091149	0.198355	-0.35364	0.211132	0.223758	-0.26991	0.696339	-0.14979	0.072639	-0.42213	0.079029	-0.00735	-0.07684	-0.04417	-3.5E-05
P62280	RPS11	0.387073	-0.63893	0.00852	0.517149	-0.1672	0.159714	-0.63106	0.07477	-0.30274	-0.21264	0.422817	0.551826	-0.33437	0.331001	-0.24968	0.071614	0.512322	-0.15034	-0.31195
P62310	LSM3	0.416383	-0.65025	-0.28844	0.278002	1.065279	0.184852	-0.58654	0.79586	-0.25734	-0.4199	0.323312	-0.46428	0.05916	-0.48291	-0.00257	0.448136	0.187719	0.243198	0.005419
P62312	LSM6	0.724628	-0.56316	0.179127	0.538257	0.986157	0.766402	-0.08924	1.063911	-0.13662	-1.05155	0.614361	-0.03017	0.015342	0.363051	-0.45244	0.476353	0.065414	-0.22743	-0.52715
P62314	SNRPD1	0.730085	-0.67596	-0.04282	0.146294	0.444969	-0.10362	-1.13637	0.120284	0.154092	0.331013	0.518961	0.773625	0.111411	0.585712	0.000194	0.12428	-0.01466	0.32562	0.137249
P62316	SNRPD2	0.441068	-0.65474	-0.33857	0.266883	0.624281	0.130837	-0.55183	0.410045	0.401327	-0.01086	0.463618	0.206256	-0.37969	-0.33314	0.167179	-0.08861	-0.02275	0.246911	-0.32112
P62318	SNRPD3	-0.1751	0.707052	0.557664	-0.0992	0.231405	-0.47325	0.279736	0.103124	0.06655	0.102067	-0.54539	1.306897	0.099061	0.936052	-0.52165	-0.71778	0.867787	-0.03799	-0.85775
P62328	TMSB4X	-1.81773	0.159842	0.177447	-0.77586	-0.35584	-1.50989	-0.23806	-0.22773	0.378988	-0.23603	-1.44892	-0.15695	0.363149	0.568531	-0.34578	0.676968	-0.55033	0.321966	0.35304
P62330	ARF6	0.342775	-0.32165	-0.33468	0.320682	0.629019	-0.17068	-0.51494	-0.52837	-0.08627	-0.25112	0.426425	1.085637	-0.06322	1.091061	-0.5497	0.586459	0.579441	-0.38922	-0.42072
P62333	PSMC6	-0.10282	0.349678	0.215606	0.003619	-0.16368	0.01298	-0.03461	0.015039	-0.04489	-0.11425	0.085161	0.332117	0.17819	0.354676	0.126946	0.278493	0.392489	-0.06041	0.2104
P62341	SELENOT	-0.59865	0.438798	0.372673	0.258412	0.999604	0.968602	0.082178	-0.24502	0.054149	0.170439	-0.22686	0.502853	0.203342	0.387554	0.423601	0.240309	0.105127	-0.55687	0.055613
P62424	RPL7A	0.359531	0.094335	0.1201	0.558169	0.008583	0.16	0.081733	0.057523	-0.80171	-0.90211	0.441511	-0.36952	-0.17915	-0.43681	0.028966	0.025398	0.017562	0.266571	-0.22899
P62495	ETF1	-0.08961	0.072559	0.008894	0.168358	-0.18916	0.192868	0.21705	-0.33964	-0.03827	0.047641	0.224809	-0.31397	0.603725	-0.46389	0.161133	0.59673	0.604775	0.438547	0.161301
P62699	YPEL5	-0.14933	0.214922	0.371205	0.398815	0.224705	0.253802	0.057852	0.173671	-0.10141	0.288141	0.411373	0.266086	0.114837	0.256627	-0.11902	-0.06036	0.22572	-0.03289	0.60679
P62701	RPS4X	0.273067	-0.30897	0.151794	0.333853	-0.08134	0.143526	-0.60817	0.176993	-0.19418	-0.15245	0.602805	0.238012	-0.02998	0.12434	-0.06921	0.179707	0.267683	0.007821	0.12142
P62714	PPP2CB	1.573516	-2.41889	-0.23209	-0.06731	0.227494	0.452104	-0.42392	0.408855	-0.31979	-0.19902	0.375209	-0.16026	0.216503	0.81	0.205834	-0.11863	1.018789	0.222679	1.14556
P62745	RHOB	-0.20392	-0.2907	-0.06096	-0.06913	0.300646	0.208299	-0.16815	0.113616	0.147339	0.163898	-0.26283	0.686333	-0.18845	0.798567	-0.33968	0.22814	0.345918	-0.02926	0.267202
P62750	RPL23A	0.385024	-0.1204	-0.12384	0.176366	0.04714	-0.04765	-0.63997	-0.26217	-0.91556	-0.65096	0.394219	-0.67436	-0.28206	-0.96892	0.172993	-0.20913	-0.64372	0.425254	0.052004
P62753	RPS6	0.37032	-0.30012	0.142369	0.240189	-0.01138	0.161422	-0.54999	0.084532	-0.14796	-0.45695	0.616081	-0.15815	0.218787	-0.29349	-0.19208	0.008881	0.125053	-0.06638	-0.11943
P62760	VSNL1	0.153522	-0.12912	0.159039	-0.29631	-0.05431	-1.56786	0.096817	-1.19159	0.89962	2.294074	0.32095	-0.51693	0.172976	-0.61419	-0.62034	0.301446	-0.73189	0.548134	1.293322
P62805	HIST1H4A	0.653488	-0.23577	0.148055	0.262612	0.451129	0.784446	0.511794	0.700232	-0.67454	-0.59129	0.05296	-0.41607	-0.87795	0.242922	0.553692	-0.04421	-0.43581	0.103111	-0.2689
P62820	RAB1A	-0.1499	0.107175	0.150956	-0.00657	0.448398	0.287891	0.187785	-0.06432	-0.05653	-0.10301	-0.18645	0.776776	0.270596	0.565183	0.328779	0.401182	-0.0691	-0.13593	0.088489
P62826	RAN	0.961444	-1.32494	-0.05227	0.735808	1.018933	0.143735	-0.51881	0.573408	0.767907	0.408853	-0.12907	1.576445	0.102264	1.207245	-0.01716	-0.04986	2.128886	0.085382	-0.12943
P62829	RPL23	0.202887	-0.59631	-0.95406	-0.13753	0.276361	-0.49073	-0.16128	-0.2647	0.277041	0.159082	0.138831	0.66281	0.108527	0.172894	-0.365	-0.60442	0.702105	-0.09073	-0.2393
P62834	RAP1A	-0.44477	-0.66259	0.020119	-0.09444	0.75811	-0.13182	0.008687	-0.73456	0.193151	0.347555	0.005838	1.074943	-0.19189	0.962457	-0.16368	-0.21132	-0.12125	0.27891	0.102964
P62841	RPS15	0.5214	-0.43838	-0.44697	0.065962	0.10381	-0.0859	-0.51765	-0.11258	0.952996	-0.60928	0.516066	0.246471	-0.5701	-0.49492	-0.61652	0.01517	-0.42021	0.657715	-0.59768
P62847	RPS24	0.428367	-0.07701	0.102206	0.3303	0.239804	0.265557	-0.43908	0.363375	-0.04753	-0.67732	0.63859	0.591791	-0.16861	0.468613	0.012047	0.532041	0.468272	0.212358	0.018922
P62851	RPS25	0.181049	0.13426	-0.62221	-0.04902	-0.298	0.445917	-0.13787	0.303207	2.467346	-0.20975	0.134229	0.890767	0.03754	-0.06186	-0.43368	0.517113	0.600555	-1.27713	-0.14516
P62854	RPS26	0.180512	-0.07541	0.112885	0.097897	0.342874	0.224106	-0.1591	0.127194	-0.2155	-0.0999	0.479618	0.458049	-0.12666	0.191707	-0.11952	0.438236	0.263632	-0.38052	0.277685
P62857	RPS28	-0.31383	0.1556	-1.14482	-0.35473	0.578422	-0.48668	0.08092	-0.7856	0.574707	0.940773	0.516717	-0.5938	0.213493	-0.73988	-1.02618	-0.37139	-0.22732	-0.371	-0.41046
P62873	GNB1	-0.37113	0.14435	0.40916	-0.22277	-0.03969	0.110646	0.251118	0.268171	0.050081	0.494015	0.064111	0.217238	0.196334	-0.36093	-0.60406	-0.19563	0.334295	0.880729	0.617388
P62879	GNB2	-0.46439	0.211163	0.260538	-0.29607	0.021458	-0.17449	0.054568	-0.34289	0.057266	0.141795	-0.02028	-0.20866	-0.03503	0.254832	0.041306	-0.26886	-0.47019	0.027008	-0.13203
P62888	RPL30	0.239194	0.423664	-0.15243	0.23763	-0.30285	0.248986	0.093008	0.218568	-0.72085	-0.2427	0.284446	0.463814	-0.16415	-0.10422	0.36211	0.295669	-0.1333	-0.15613	0.280537
P62899	RPL31	0.182998	0.238457	0.065632	-0.02014	-0.11731	-0.11117	0.13184	-0.23709	-0.14814	-0.46986	0.042979	-0.25118	-0.05175	-0.41424	0.130564	0.032585	-0.39216	-0.04218	-0.25177
P62906	RPL10A	0.291419	-0.06524	0.028696	0.470809	-0.03983	0.074406	-0.04881	0.110283	-0.25	-0.27492	0.495808	-0.5732	0.042952	-0.53059	0.34879	0.036168	-0.46153	-0.14269	0.080856

P62910	RPL32	0.158999	0.325681	-0.25582	0.301426	-0.34909	0.217494	0.801686	0.210885	-0.31642	-0.43207	0.683946	0.142807	-0.03522	-0.84112	-0.04902	-0.02937	-0.67892	-0.42327	0.189487
P62913	RPL11	0.459516	-0.88755	0.196465	0.327533	-0.00177	0.269127	-0.24359	0.239932	0.0979	-0.02778	0.453112	0.27026	-0.14417	0.134932	0.02071	0.245527	0.653639	-0.39239	-0.35372
P62917	RPL8	0.28376	-0.03976	-0.4983	0.413345	-0.10696	0.119382	0.220075	0.009543	-0.12619	-0.71987	0.535501	-0.63828	-0.08718	-1.29787	-0.01773	-0.25218	-0.44974	0.039396	-0.07022
P62937	PPIA	0.252175	-1.60951	0.245344	-0.2111	1.705233	0.065253	-0.23179	0.197077	0.766391	1.771173	0.112711	0.037815	-0.14928	0.055717	-0.47637	0.015435	1.21773	0.382692	-0.47863
P62942	FKBP1A	0.669449	-1.05349	0.524181	0.491474	0.287806	0.013341	-0.3309	0.302991	0.634363	1.762954	0.499136	-0.72529	0.241436	-0.29384	0.243609	0.017685	0.286826	0.536408	0.737944
P62993	GRB2	0.501057	-1.04286	0.782371	0.068075	0.668195	0.300105	-0.08626	0.40854	0.583143	1.122436	-0.32073	1.336218	0.801107	1.118802	-0.54924	0.313335	0.349044	0.402451	-0.48835
P62995	TRA2B	0.530128	-0.70688	0.07448	0.450021	0.197948	0.62321	-0.91668	0.638848	-0.05663	-0.15983	0.203155	-0.25646	-0.61373	-0.41519	0.290458	-0.36616	-0.06725	0.144983	0.032305
P63000	RAC1	0.262804	-0.11649	0.370863	-0.74001	0.521162	-0.38865	-0.10733	-0.93337	-0.11632	0.60724	-1.30867	0.986007	-0.48331	1.4703	-0.44463	-0.13421	0.235888	-0.35612	-0.30579
P63010	AP2B1	-0.47115	0.424321	0.515845	0.080211	0.076113	-0.14946	0.493626	-0.11236	-0.38449	-0.21993	-0.20352	-0.18845	0.084446	-0.01964	0.317253	0.382253	-0.24097	-0.10547	0.566222
P63027	VAMP2	-0.00243	0.170747	-0.08506	-0.28982	-0.18058	0.017694	0.227458	-0.39115	-0.08594	0.089808	-0.05254	-0.02981	-0.80427	0.546397	-0.35154	0.221277	-0.57507	-0.44566	0.022793
P63096	GNAI1	-0.27639	0.217134	-0.17117	-1.09111	-0.4454	-0.39808	1.06072	0.493391	-0.58304	-0.94967	-0.35434	-1.14178	-0.57701	-0.59314	0.891238	-0.41443	-0.7691	0.632164	-0.26583
P63098	PPP3R1	0.020908	-0.84421	0.426298	-0.50861	1.136642	-0.06231	-0.54888	-0.04915	0.486753	1.063219	0.380246	-0.11779	0.42658	0.522307	0.068527	0.438621	-0.51031	1.00318	0.68063
P63104	YWHAZ	-0.02195	-1.07504	-0.21282	0.17663	0.093043	0.418406	-1.41205	-0.02336	0.277896	0.212084	-0.10265	-0.40254	1.385094	-0.62144	0.019196	-0.18186	0.003458	-0.07572	-0.23165
P63151	PPP2R2A	0.469819	-0.50036	0.363545	-0.1378	-0.18084	0.074495	-0.67265	-0.53101	0.648649	-0.17542	0.666495	-0.21434	0.244939	0.104299	-0.56663	-0.0792	0.924643	0.393242	0.761908
P63165	SUMO1	0.483138	-0.6492	0.504266	-0.32305	-0.7146	-0.84997	-0.74896	-0.25596	-0.49662	-0.09606	0.33309	-1.0555	0.737049	-1.01522	0.92828	0.540402	-0.74595	-0.17828	0.54255
P63167	DYNLL1	-2.4295	-0.68187	0.110599	-0.00868	0.947811	0.183909	0.076095	0.619203	0.683075	0.241117	0.505749	0.673016	0.132877	0.500669	0.012576	0.058669	0.993746	-0.21316	0.308335
P63173	RPL38	-0.0139	0.073855	-0.10879	-0.20686	-0.12995	-0.50402	-1.31267	-0.34282	0.327313	0.616962	0.185663	0.89152	0.323127	0.521134	-0.4407	-0.12337	1.007624	-0.16492	0.183706
P63208	SKP1	0.147724	-0.73709	0.089209	0.250193	0.46781	0.208721	-0.63108	1.099129	0.362658	0.053806	0.402909	-0.51563	0.299088	-0.49428	0.598304	0.23106	-0.09831	0.340396	0.088392
P63218	GNG5	-0.11617	0.80507	-0.00992	-0.5811	0.63106	-0.06013	0.425775	0.110918	0.258383	0.267188	-0.67742	-0.31094	-0.39552	0.001853	-0.40787	-0.00019	-0.70894	-0.49092	0.023155
P63220	RPS21	0.336959	-0.58707	-0.91981	-0.38266	0.211285	-0.34146	-1.14203	-0.01029	0.340043	-0.0344	0.526365	-1.07816	0.569595	-1.02761	-0.15812	-0.27677	-0.29992	-0.08579	-0.33969
P63241	EIF5A	0.576918	-0.61684	-0.07457	-0.37803	0.649135	-0.05737	-0.88265	-0.04978	0.355067	1.362525	-0.07706	0.324073	0.466518	0.14473	-0.62799	-0.39335	0.619631	-0.03523	-0.2486
P63244	RACK1	0.191707	-0.16582	0.062549	0.232231	0.043037	0.268951	-0.61296	0.167216	-0.38772	-0.03252	0.440599	-0.09588	0.041721	-0.05245	-0.08381	0.215383	0.019416	-0.18484	0.110379
P63279	UBE2I	0.511854	-0.89198	-0.33526	-0.00888	0.101158	0.042281	-1.98612	0.164722	-0.84538	-0.58439	0.058197	-0.41758	-0.33814	-0.39773	0.019631	0.031286	0.085189	-0.0547	-0.33315
P63316	TNNC1	-0.68583	-3.96878	-0.55027	-3.98507	-4.29032	-5.32987	0.091893	-4.12827	-0.58285	-2.88351	-3.34682	-2.44344	-4.08607	-1.68885	-1.21334	-2.85686	-3.78919	-2.48714	-4.62154
P67775	PPP2CA	0.387476	-1.20058	0.594266	0.675576	0.881605	0.310018	-0.47515	0.80943	0.31985	0.545287	0.557127	-0.09608	0.914364	0.709248	0.701277	0.347456	0.687882	0.522227	0.838599
P67809	YBX1	0.945114	-0.47525	0.848686	0.712517	0.126345	0.558385	-0.518	0.521318	0.109907	-0.45719	0.174624	-0.78401	0.406292	-1.5741	0.112236	0.293934	-0.79995	-0.29036	0.115632
P67812	SEC11A	-0.16595	0.613524	0.864796	0.443917	0.280913	0.452646	0.48511	0.172109	0.103789	-0.1624	-0.0185	0.631296	0.06844	0.586325	-0.36226	0.090565	0.440046	0.082357	0.534978
P67870	CSNK2B	0.15358	0.533495	-0.22447	0.157505	0.003651	0.4791	-0.16265	0.384659	0.270663	0.037925	0.12774	-0.37767	0.499244	-0.17125	-0.13215	0.035398	0.644379	0.03376	-0.17397
P67936	TPM4	-0.62309	-1.54297	0.131164	-0.08284	-0.31889	-0.68912	-1.38655	1.179082	0.773221	0.073449	-0.81257	-1.04632	0.272443	-1.01398	-1.07692	0.228591	-1.10296	0.640001	0.358578
P68036	UBE2L3	0.369613	-0.14249	0.216784	0.360353	-0.06626	-0.58364	-0.93795	-0.66665	-0.52999	-0.05815	0.105799	-0.29423	0.177507	-0.37147	-0.3917	0.125059	0.30224	0.612729	-0.28274
P68133	ACTA1	-0.8684	-0.64336	0.122067	-0.47361	0.095395	-0.45585	-0.64031	-0.11898	0.753883	0.156278	-0.85692	-0.09831	0.076289	0.725019	-0.31114	-0.08858	-0.1182	0.025498	0.352492
P68366	TUBA4A	0.281084	0.087602	0.348563	-0.05448	0.148062	-0.22527	0.301217	0.525967	0.125928	-0.24478	-0.2236	-0.39246	-0.17122	0.101367	0.192548	0.161751	0.273152	-0.29655	0.186847
P68371	TUBB4B	0.275253	-0.25871	-0.21237	0.037792	-0.18437	-0.04252	0.273064	0.384057	0.137389	-0.39139	0.307703	-0.10002	-0.36181	-0.00465	-0.41903	-0.53387	0.060547	-0.62293	-0.24382
P68400	CSNK2A1	0.21566	-0.42655	-0.10349	0.606635	-0.23024	0.051073	-0.81444	0.532803	-0.21816	-0.1381	0.317005	-0.2952	0.364149	0.107973	0.225487	0.135644	0.370682	0.02282	0.036748
P68402	PAFAH1B:	0.547749	-0.43941	-0.62398	-0.51117	0.211583	-0.33846	-0.56979	0.136466	0.381605	1.519302	-0.32003	0.989866	-0.29423	0.436909	0.002845	0.57887	0.641505	-0.49751	-0.04862
P68871	HBB	-0.8772	-0.14406	0.66884	-0.17872	1.227223	-1.4845	-0.29999	-0.88748	2.006805	-0.52908	-0.89615	-0.87014	-1.03223	-0.87542	-1.09925	-1.63148	-1.25644	2.698044	-0.71742
P69849	NOMO3	-0.48672	0.463111	1.74E-05	0.220378	0.120695	0.311255	0.329805	-0.18981	0.142173	0.007551	0.433013	0.057723	0.302138	0.102914	0.145627	-0.30311	0.140334	0.641157	-0.07874
P69891	HBG1	-0.00557	-0.19134	-0.67554	-1.02735	-1.74458	-2.54285	0.237218	-2.85896	-0.28732	-0.88957	-2.05876	-1.4686	-0.93936	-1.15663	-1.11052	-1.36085	-2.76047	-0.43306	-3.85805
P69892	HBG2	-0.65147	-0.50891	-0.31399	-2.78235	0.019132	-1.56764	0.288133	-1.86661	0.352039	-1.14397	-1.37995	-2.33058	-1.05475	-1.98867	-0.86192	-2.54526	-0.93742	-1.84798	-1.93261
P69905	HBA1	-0.99749	0.022325	0.391415	-0.45018	1.179286	-1.305	-0.29441	-0.74834	2.039993	-0.67816	-0.98914	-0.63256	-1.06385	-0.7913	-1.05709	-1.42257	-0.82736	2.010629	-0.80791
P78310	CXADR	1.080428	-0.69017	-0.24226	-0.43704	-1.42549	-0.72769	-0.10987	0.279299	-0.91989	-0.18356	0.216669	-0.51744	0.227813	-0.61825	-0.04557	0.186308	-0.57291	-1.1401	-0.8026
P78314	SH3BP2	0.336936	0.398386	0.425495	-0.11911	-0.28463	-0.33996	-0.00964	0.119374	-0.0205	0.91698	-0.3692	0.001584	0.25647	0.46183	-0.46298	0.924523	-0.08663	-0.69157	-0.68486
P78316	NOP14	0.666941	-0.12752	-0.67879	0.336489	-0.34697	-0.0314	-0.22331	0.153698	-0.70237	0.231868	0.52728	-0.31416	0.123227	-0.98123	0.15998	-0.149	-0.58053	-0.23417	-0.06729
P78318	IGBP1	-0.34754	-0.56402	-0.50186	-0.04488	0.23383	0.146923	0.003987	0.204587	1.138904	0.827713	0.011204	-0.15242	-0.03477	0.353994	-0.51457	0.170408	0.225591	0.073345	0.064614
P78324	SIRPA	-1.04611	1.178819	1.161861	-1.09724	-0.07184	-0.37077	1.019864	-1.44249	-0.21465	1.225426	-1.18497	-0.0724	0.014423	0.978819	0.328413	-0.33364	-0.23332	0.101184	-0.38096
P78330	PSPH	0.465445	-0.37136	-0.14551	0.187445	-0.00037	-0.43556	-0.45831	1.350102	0.046484	0.811257	0.918723	-0.86761	-0.34297	-0.49712	-0.58694	0.745535	-0.12629	-0.00169	-0.15816
P78332	RBM6	0.243369	0.061402	0.544811	-0.04474	-0.03552	0.055995	-0.29065	0.245248	0.001736	-0.11909	0.415348	-0.6267	-0.5262	-0.57462	-0.14036	-0.18631	-0.19848	0.170352	0.127868
P78344	EIF4G2	-0.17777	0.247401	0.344977	-0.21228	0.137177	0.350703	0.165744	0.457791	-0.14121	0.449965	-0.64438	-0.07173	0.425797	0.074712	-0.07225	0.168286	0.057428	0.013537	0.639451
P78345	RPP38	-0.17806	0.181932	0.067617	-0.16545	0.079517	0.093285	0.184932	0.150667	0.194889	0.315116	-0.04937	0.161903	-0.02142	0.202399	-0.39722	0.130698	-0.02547	-0.05301	-0.26859
P78346	RPP30	0.553197	-0.29963	-0.02341	0.286676	0.06293	-0.12161	-0.5423	0.260453	-0.19344	-0.72809	0.343476	0.050437	-0.01817	0.223288	0.326082	0.145945	-0.06871	0.255999	-0.43544
P78347	GTF2I	0.644211	-0.74047	-0.60862	0.473052	0.172158	0.751086	-0.1027	1.667708	-0.55149	-0.35624	0.09608	-0.4024	0.056243	-0.54332	0.966474	-0.95019	-0.62401	-0.54424	0.599882
P78356	PIP4K2B	-0.37298	0.271478	0.150598	-0.30686	-0.2294	-0.26829	0.000622	-0.34107	0.355174	0.071009	0.308413	-0.27049	-0.26125	-0.19264	-0.46156	-0.45382	-0.51157	0.202384	0.258817
P78357	CNTNAP1	-1.01317	0.408915	0.300759	-1.11302	-0.34514	-0.5322	-0.11351	-0.04518	0.482522	-0.4095	-0.1784	-0.3608	0.264679	-0.39758	-0.3175	-0.65547	-0.23156	0.024006	0.657561
P78362	SRPK2	0.030508	-0.10351	-0.22336	-0.37712	0.632056	0.498644	-0.48305	1.100204	0.283456	0.155127	-0.65362	-0.50324	0.015779	-0.43159	0.15978	-0.03375	-0.67276	-0.73133	0.123561
P78371	CCT2	-0.31558	0.011211	-0.15847	0.055801	0.144141	0.552078	-0.93861	-0.56282	0.169437	-0.223	0.297585	-0.12342	0.091655	0.015175	-0.01482	-0.0748	-0.09498	-0.30948	-0.03952
P78382	SLC35A1	1.090997	-0.10235	-0.07366	0.59278	0.301361	-0.04922	-0.73276	-1.55122	-0.22561	-0.02473	-0.44587	0.301985	0.248227	0.174758	0.743401	0.741397	0.326814	-0.04524	-0.42396
P78386	KRT85	-0.31236	0.428852	0.074575	1.326097	-2.06497	-1.29639	-0.90727	0.05896	-1.13407	-2.77961	-1.93074	-0.26694	-0.67098	-0.50589	-0.87208	-1.22432	-1.24432	-2.24916	-0.96534
P78406	RAE1	-0.09576	-0.04013	-0.27471	0.345978	0.253495	0.376547	-0.09524	0.309155	0.353488	0.189163	0.060635	-0.09598	-0.11045	0.014632	0.670278	-0.03338	0.132474	-0.00691	-0.33006
P78410	BTN3A2	0.089592	-0.32424	0.194008	-0.83684	0.161606	0.325958	-0.04598	0.027202	-0.06019	-0.07817	-2.05286	-0.50726	-0.03128	-0.32807	-0.2935	-0.74913	-0.41273	0.77899	0.756878
P78417	GSTO1	-1.8704	0.224477	-1.09616	-1.71311	1.779228	-0.63712	-0.47106	0.574046	1.002706	1.589831	-0.62137	0.058903	0.38721	0.704158	-0.95825	-0.66465	-0.02834	-0.46129	-1.02953
P78524	ST5	-0.32674	0.096608	-0.03838	-0.67262	-0.14993	-0.44781	-0.4409	-0.265	0.52931	-0.26441	-0.00096	-0.46355	0.139346	-0.00292	-0.12155	-0.08262	-0.53259	0.149434	0.109111
P78527	PRKDC	0.831549	-0.06999	-0.00762	-0.03496	0.001059	0.132626	-0.07674	0.347014	-0.35723	-0.17337	0.268333	0.16778	0.107663	-0.07887	0.449191	-0.05238	0.18701	0.011139	0.19644
P78536	ADAM17	-0.31469	0.676719	-0.22213	-0.29526	-0.34734	-0.52232	0.51425	-0.26933	-0.02232	-0.06474	0.179743	0.329133	0.646992	-0.07679	-0.21192	0.98031	0.129964	0.144182	0.128625
P78537	BLOC1S1	0.189044	0.043563	0.017362	-0.34435	-0.08099	-0.10787	0.005864	-0.45549	-0.02186	-0.18319	0.275775	0.201683	0.02546	0.46211	0.147984	-0.69344	0.317026	0.28991	0.227462
P78539	SRPX	-2.38367	-0.69478	-0.28859	-0.98405	-1.37074	-3.03383	0.01099	-1.30859	0.273472	-0.63044	-1.29326	-1.08164	-0.69624	-0.19283	1.788174	-1.46848	-0.68463	-0.59113	-0.87506
P78549	NTHL1	0.890733	-0.50458	-1.67704	-1.12402	-0.10727	-0.28277	-0.40153	0.347592	0.040847	-1.3964	0.470684	-0.89089	-0.64247	-0.21736	0.113834	-0.22381	-0.05268	0.734506	-0.70442
P78559	MAP1A	-0.81385	0.330027	-0.46324	-0.80938	-1.15905	-0.79333	-0.5922	-0.69423	-0.04561	-0.27939	0.811908	-1.10044	0.257968	-0.92123	-0.3261	0.023702	-0.80605	0.238223	0.730925
P78563	ADARB1	-0.27151	0.480316	0.197757	-0.03385	0.254262	-0.08218	0.324829	-0.19012	-0.22375	0.039052	-0.47156	0.452617	-0.02952	-0.11268	0.002529	-0.60455	0.058566	0.316109	0.254006
P79483	HLA-DRB3	-1.1002	0.027605	-0.23481	-0.08277	-0.04869	0.466468	-0.35211	-0.2837	-0.55492	-0.41049	0.824903	-0.82472	-0.44136	-0.02323	0.20746	-1.10201	-0.22908	0.357328	-1.91111
P80188	LCN2	-1.57046	0.874562	0.979367	-0.9696	-0.10277	-0.92608	-0.22877	-2.6759	-0.03333	1.073512	-1.36015	1.739158	-0.24446	2.239085	-0.92103	-1.94728	1.220657	-2.34227	-1.50452
P80217	IFI35	0.184634	0.374932	0.544417	0.027474	0.772393	-0.08411	0.009366	0.304495	0.257002	0.51605	-0.9229	0.527061	1.246974	0.8763	-0.98336	0.088854	0.486692	0.266978	-0.86951
P80303	NUCB2	-0.54596	-1.00657	-0.07263	-0.3632	1.499125	0.949942	-0.1701	0.037169	0.201363	0.250103	0.128004	0.323402	0.796981	0.213237	0.701575	0.642743	0.43906	1.358156	0.092004
P80404	ABAT	0.669893	-0.05348	0.341553	-0.04726	-0.38953	-0.87094	-0.15262	-0.26782	-0.44622	-0.12617	0.880056	0.390062	-0.16909	-0.00869	-0.84337	0.541963	-0.52111	-0.97661	-0.42923
P80511	S100A12	-4.71355	-1.35952	1.558178	-2.07425	-2.32091	-3.36103	-0.3208	-5.07109	-0.42519	1.563652	-3.93379	-0.47933	-1.65989	3.743421	-0.67357	-2.19459	1.186307	-4.90638	-2.69601
P80723	BASP1	-1.66895	-0.51037	0.929856	-2.09241	-1.29495	-1.74837	0.831903	-2.52818	1.172504	0.532254	-1.03541	0.059881	-0.66883	0.754833	-1.50821	0.621268	-0.23175	-0.87865	-0.09736
P81605	DCD	-1.41773	1.636613	0.447719	-1.27273	-2.26411	-0.9909	1.697202	-0.33159	0.619085	-1.35484	-1.17517	-0.66509	0.266941	-0.40627	-0.65167	-0.90839	-0.96995	-0.84214	-0.37429
P82094	TMF1	-0.3452	0.558517	0.372624	0.635735	-0.02633	-0.09667	0.276047	-0.22639	-0.34344	-0.73141	0.328877	-0.23151	0.298561	-0.25124	-1.06346	-0.40351	0.343642	0.904514	0.088139
P82650	MRPS22	-0.21031	-0.09992	-0.33002	0.852576	0.235137	0.449539	0.08188	0.063192	-0.64627	-0.04022	0.474785	-0.21636	-0.0021	-0.37814	1.237966	-0.12812	-0.43488	-0.17908	-0.22878
P82663	MRPS25	-0.02468	-0.0119	-0.16859	0.30802	0.334409	0.450338	-0.10418	0.327099	0.342184	-0.41018	0.467361	0.280499	-0.11068	0.081232	1.178088	-0.60483	-0.33295	0.308097	0.034396
P82664	MRPS10	-0.39782	-0.18511	-0.20921	0.997457	0.034681	0.278091	-0.11875	0.375393	-0.75247	0.418003	0.602782	-0.00807	-0.18405	-0.06116	1.396914	0.300341	-0.4479	-0.0737	-0.36175
P82673	MRPS35	-0.15729	-0.54881	-0.05255	0.921101	0.258904	0.433307	-0.1472	0.219764	-0.63018	0.529528	0.711914	-0.38994	-0.01124	-0.27307	1.179726	0.127952	-0.07598	0.225519	-0.13886
P82675	MRPS5	-0.52073	-0.17949	-0.38327	1.042147	-0.05149	0.438005	0.009348	0.054507	-0.44261	0.154298	0.606149	-0.27987	-0.28864	-0.24284	1.158662	-0.02199	-0.57612	-0.02172	-0.4306
P82912	MRPS11	-0.84006	0.231877	0.150798	-0.54214	0.098331	-0.13794	0.145073	-0.46038	-0.0038	0.586667	0.612329	0.337873	-0.16697	0.42729	0.475071	-0.15494	-0.00287	0.027318	-0.62629
P82914	MRPS15	-0.47102	-0.62065	-0.54057	1.211552	0.035434	0.336641	-0.73342	-0.02754	-0.31907	0.281187	0.690045	-0.19604	-0.23489	-0.15508	1.146663	-0.37795	-0.2417	-0.27743	-0.93467
P82921	MRPS21	-0.23758	-0.00754	-0.22419	0.713079	0.124411	-0.04215	0.215673	-0.31899	-0.10536	-0.08523	0.706908	0.018704	0.003491	-0.43181	0.562392	0.121169	-0.48242	-0.30504	-0.86166
P82930	MRPS34	-0.44528	-0.34137	-0.4053	0.904866	-0.17602	0.383339	0.223214	0.095986	-0.10656	-0.15372	1.057371	-0.31597	-0.19649	0.255262	1.364801	0.000512	-0.35069	-0.25447	-0.14772
P82932	MRPS6	0.024784	-0.43265	-0.18782	-0.00742	0.70634	0.581225	-0.13358	0.401298	-1.24656	0.490714	0.53197	-0.11933	-0.01445	-0.20586	1.185926	-0.42166	0.124715	-0.12414	-0.67403
P82933	MRPS9	-0.08458	0.006031	-0.02016	0.994591	0.054948	0.250051	0.223109	0.019663	-0.13952	0.018482	0.292929	-0.21388	-0.21578	-0.25283	0.999505	-0.11396	-0.18772	-0.1311	-0.48448
P82979	SARNP	0.297053	-0.14132	-1.29552	-0.19534	0.53517	0.392399	-0.77123	-0.13069	0.176521	-0.40985	-0.06418	-0.29269	-0.58792	-0.24141	-0.22424	-0.31662	-0.24818	0.758421	-0.14551
P83110	HTRA3	-0.9792	0.446616	-0.3551	-0.56613	-1.23122	-0.94668	-0.16911	-0.59277	0.808648	-1.85234	-2.10382	-0.70699	0.827157	-0.8111	-0.55688	0.311934	-0.78268	-0.27794	1.991701
P83111	LACTB	-1.52318	1.777732	0.819934	0.239069	0.330626	-0.28293	0.032482	-1.27418	-0.49301	0.98883	-0.75078	1.092545	-0.00588	1.129815	-0.67019	0.242007	0.551815	0.11741	-0.81506
P83436	COG7	-0.52529	0.383654	0.02891	0.38927	0.103334	0.071218	0.18198	-0.40343	0.274417	0.018823	-0.06576	0.023378	0.167009	-0.12141	0.670989	0.435168	-0.0598	0.126237	0.579616
P83731	RPL24	0.371095	-0.55328	-0.82846	0.242219	-0.09555	-0.15765	-0.11046	0.060297	0.272109	-0.48692	0.3402	0.045567	0.043861	-0.95385	0.393236	0.023239	-0.05483	-0.46305	-0.53291
P83916	CBX1	0.44414	-1.33846	-0.02737	1.030818	1.116175	-0.39786	-0.77934	0.888717	0.326353	-0.11259	0.075158	-0.08278	-0.33936	0.02776	0.257408	-0.27427	0.271652	1.31239	0.130418
P84022	SMAD3	-0.13189	-0.07463	0.177795	-0.10755	-0.29906	-0.73605	-0.47986	-0.27899	0.503888	0.141494	0.465914	-0.00065	-0.10321	-0.00479	-0.78036	0.664876	0.620423	-0.04894	-0.71459
P84085	ARF5	0.194686	-1.08612	0.356055	0.132491	1.051812	0.455365	-0.74274	0.085705	0.741819	1.012452	-0.1255	0.695321	0.343952	0.895971	0.434304	0.750213	0.461382	0.040404	0.469631
P84090	ERH	1.183588	-1.27153	0.529	0.924543	0.197007	0.582504	-0.69687	1.282769	-0.49997	-0.02822	1.149745	0.360916	-0.3882	0.273969	0.858008	0.671566	0.542326	0.972949	-0.18527
P84095	RHOG	-1.42454	0.95558	0.489942	-0.7083	0.698969	0.28013	0.365904	-0.48521	-0.53756	1.01902	-0.67217	1.138568	0.019793	1.242943	0.003805	-0.4928	0.408757	-0.06352	-0.41318
P84098	RPL19	0.085609	-0.1101	-0.54505	0.315558	-0.16987	-0.11136	-0.32443	-0.16547	0.433984	-0.49541	0.415976	-0.91454	-0.19369	-0.99751	0.132402	0.102156	-0.47264	-0.07538	-0.20909
P84101	SERF2	0.663529	0.219913	-0.26849	-0.1697	-2.36805	0.45107	-0.55561	-0.46735	-0.07817	-0.45381	-0.04913	-1.66099	0.449745	-1.10055	-0.16522	0.866916	-0.69193	1.149545	0.667368
P84103	SRSF3	0.4607	-0.22015	-0.07236	0.287109	0.183466	0.613827	-0.40772	0.704065	-0.5126	-0.26799	0.433385	-0.64373	-0.46521	-0.66519	0.136528	0.036264	-0.43903	0.279705	-0.03726
P84157	MXRA7	-1.26673	-0.96683	-1.12479	0.591797	-0.65983	0.018233	-0.04294	0.37097	-0.15202	-0.81561	0.476082	-1.10803	0.162743	-1.27192	-0.57213	-0.13329	-0.50504	0.45233	0.408574
P84243	H3F3A	0.626574	-0.18102	0.694861	0.237968	0.481029	0.666025	0.348063	0.387532	-0.44814	-0.6196	0.198069	-0.55147	-0.70459	0.100642	0.374796	-0.18802	-0.46218	0.424666	-0.64171
P85037	FOXK1	-0.04002	0.001228	-0.18538	-0.53429	-0.09487	0.243161	0.080445	0.53026	0.245187	0.206905	0.451993	-0.4984	-0.30675	-0.40476	0.169262	-0.15205	0.112029	0.133513	-0.13307
P85298	ARHGAP8	0.544039	-0.63453	0.033602	0.281156	0.099471	-0.25015	-0.36049	0.106921	-0.34978	-1.10169	-0.32158	-0.97179	0.480419	-0.10465	0.481769	1.52146	0.302484	0.922577	-1.64523
P86790	CCZ1B	-0.60244	0.48516	0.276128	-0.21877	0.245545	0.507107	0.069565	0.282044	0.047588	0.179189	-0.2883	-0.02074	0.085513	0.175436	0.115195	0.633493	0.012201	0.086262	0.715764
P98082	DAB2	-1.34082	0.715092	0.921811	-0.86669	0.242169	-0.23728	1.055506	-1.24817	0.416567	0.586547	-0.60523	0.894922	-0.00102	0.900583	-0.26066	0.337492	-0.76383	0.268389	0.247028
P98095	FBLN2	-1.88916	-0.31571	-1.1089	-2.1575	-1.97537	-1.17383	-0.16879	-0.18186	-0.0604	0.495743	-2.77845	-1.69399	-1.81244	-1.3257	-0.72016	0.457189	-1.14546	0.768681	-1.1917
P98155	VLDLR	-0.66112	0.294393	-0.01281	-0.18457	0.462183	0.054991	-0.28774	0.45057	0.049329	0.305581	1.144849	0.469928	0.045777	0.645052	0.177233	0.060822	0.405294	1.117895	0.050674
P98160	HSPG2	-1.23419	0.020437	-0.26273	-1.29466	-0.68481	-1.08177	0.495032	-0.24994	0.840382	0.673757	-0.86641	-1.11135	-0.36968	-0.89414	0.185249	-0.6164	-0.32939	0.606527	0.453075
P98170	XIAP	0.266184	-0.39717	0.481342	-0.39802	0.252855	0.282864	-0.39509	-0.3071	0.219782	0.392191	0.623708	-0.09026	-0.15242	0.362196	-0.12324	0.551197	0.568866	0.534238	-0.50338
P98171	ARHGAP4	-1.64022	0.946383	0.903952	-0.99057	0.973179	0.855662	0.43216	-0.80897	-0.16091	0.828898	-1.3545	0.989604	-0.20325	1.147348	0.555775	-0.74594	-0.2694	0.048366	-1.34556
P98172	EFNB1	-0.27219	0.014619	-0.10546	-2.66227	-0.69886	-0.03234	-0.22594	0.249996	-0.01793	0.445858	0.429934	-0.7782	-0.16587	-1.93142	-0.55912	0.10526	-1.00993	-0.6647	0.044668
P98175	RBM10	0.489476	-0.26174	-0.51728	0.316881	-0.29654	-0.13338	-0.81761	0.833674	0.122627	0.237155	0.545375	-0.76352	-0.0421	-0.42884	-0.00728	0.202468	-0.21542	0.720344	-0.10757
P98179	RBM3	-0.11435	-0.06717	-0.24498	0.625721	-0.27308	-0.1877	-1.45216	-0.86703	-0.2854	-0.43341	-0.05045	-1.30816	0.020033	-0.83354	-0.74049	0.060726	-0.91857	0.154138	0.207302
P98194	ATP2C1	-0.02647	0.301062	0.114407	0.496748	0.756137	0.549111	-0.26126	-0.18943	0.325936	0.641288	-0.20366	0.291935	0.357616	0.233379	1.483947	0.198929	0.409204	0.111505	-0.13225
P98196	ATP11A	-0.27917	0.759168	0.345214	-0.28574	0.160786	0.088674	-0.05555	-0.32705	0.196316	1.111281	-0.41743	0.511605	0.258327	1.942556	-0.21467	-0.3214	1.784527	2.0606	-0.58804
P99999	CYCS	0.363034	-0.14357	-0.39444	-0.76719	0.77587	-0.84856	-0.20211	-1.26595	-0.1978	0.517357	-0.81267	-0.77697	-0.67277	-1.1922	0.740637	0.68885	-1.34517	-0.56728	-1.56565
Q00013	MPP1	-1.5853	1.249803	-0.18292	-0.21128	0.604246	-0.27169	-0.99061	-1.32835	0.575116	0.825307	-1.26467	1.496253	-0.12837	0.858564	-0.38461	-0.54329	0.154422	0.710699	-1.08342
Q00059	TFAM	1.057	-0.54943	-0.02555	0.487454	0.4846	0.678394	-0.69475	0.001696	-0.85552	-0.31126	0.875408	-0.38765	-0.16236	-0.60315	0.687731	-0.87568	-0.11663	-0.18626	-1.12357
Q00169	PITPNA	1.479727	-0.47675	0.57151	-0.11824	1.505242	0.194683	-0.32278	0.223428	0.392025	0.764812	0.577946	-0.59894	0.902314	-0.01533	0.044123	-0.37548	-0.60037	0.186484	0.97588
Q00325	SLC25A3	0.355181	-0.19911	-0.04628	1.009339	0.234489	-0.2911	-0.35175	0.183745	-0.26016	-0.29587	0.0995	0.270123	-0.39522	0.109583	1.331985	0.498168	0.053583	-0.38458	0.284454
Q00341	HDLBP	-0.41052	0.612859	0.054738	-0.52076	-0.15639	-0.19536	0.174226	-0.05826	0.494578	-0.11086	-0.44222	-0.50045	0.425105	-0.41863	-0.19432	0.488315	0.035001	0.650952	1.143025

Q00403	GTF2B	0.595411	0.385581	0.102488	-0.35337	-0.03839	-0.03601	0.380354	0.263022	-0.21101	0.090135	0.525624	-0.25422	-0.16841	-0.23152	-0.10145	0.191842	0.122301	0.167906	-0.33748
Q00534	CDK6	-0.64657	0.128613	-0.02243	-1.44089	-0.25786	-0.4502	-0.10761	-0.45914	0.055558	0.134293	-0.4917	-0.71185	0.141327	-0.71462	-0.76414	-0.82998	-0.57133	0.132121	0.31349
Q00535	CDK5	0.231835	-0.02939	0.098128	1.242578	0.167847	0.003808	-0.17029	0.239893	0.126788	0.83234	-0.35686	0.292283	-0.73016	0.232028	-0.04008	0.710231	0.075131	-0.23795	-0.65313
Q00536	CDK16	0.500218	-0.2015	-0.19156	-0.18261	-0.22394	-0.27966	-0.03614	0.764906	0.040847	1.990367	0.366421	-0.2858	-0.17624	0.126189	0.130798	0.329806	0.503446	-0.54327	-0.40641
Q00537	CDK17	0.418965	0.384112	0.498852	-0.5302	-0.4647	-0.00444	0.24129	0.008782	-0.07227	-0.01939	-0.90243	-0.28833	0.488172	0.353879	-0.91702	0.092036	0.438462	0.837959	0.615109
Q00577	PURA	-0.61787	-0.62663	-0.276	-0.66853	-0.51011	-0.68997	-0.70326	-0.50772	0.219572	-1.00324	0.562946	-0.79663	-0.92763	-0.78572	0.376122	-0.11052	-0.37848	-0.27802	-0.64244
Q00587	CDC42EP	-0.25348	0.195084	-0.47987	-0.43839	-0.60702	-1.0022	-0.62882	0.198571	0.401309	0.148192	0.277745	-0.88343	0.24722	-0.85422	-0.58981	-0.16659	-0.88819	0.883179	1.196679
Q00610	CLTC	-0.56238	0.621286	0.443954	0.052802	-0.13804	-0.13911	0.42678	-0.12509	-0.12275	0.039511	-0.07422	0.625234	0.134049	0.497868	0.092841	0.311019	-0.08099	-0.28893	0.048542
Q00613	HSF1	0.883069	-0.56995	-0.39083	-0.42367	-0.54738	0.151763	-0.23397	-0.00363	-0.44001	-0.38092	0.265529	0.098524	0.414078	-0.01225	-0.59163	0.189588	0.411692	-0.01288	-0.52252
Q00653	NFKB2	-0.28707	-0.09974	-0.02688	-0.70373	-0.26941	-0.28533	0.038837	0.137857	0.328834	0.780405	-0.22912	0.445406	-0.02036	0.094282	-0.83985	-0.4835	0.45506	-0.44122	-0.44654
Q00688	FKBP3	0.396215	-0.56569	-0.46362	0.684255	-0.28391	-0.17872	-0.02949	0.200061	-0.14966	-0.33648	0.334897	-0.83772	-0.24424	-0.82558	0.404103	0.892387	-0.52396	-0.13181	0.352886
Q00722	PLCB2	-1.53201	0.659445	1.326406	-0.50162	0.157342	0.269446	-0.02413	-1.10916	-0.12106	1.48017	-1.18349	0.590939	0.937865	1.497733	-0.32543	-0.51428	0.472588	0.47365	-1.10542
Q00765	REEP5	-1.16182	0.559727	0.531528	-0.48492	-0.14488	-0.21463	0.429666	-0.71167	-0.77784	-0.4274	0.229025	-0.05155	-0.19791	-0.15681	1.012715	0.339765	0.433271	0.611256	-0.59997
Q00796	SORD	0.322266	-0.80521	-0.60304	0.019291	-1.02448	-0.85137	-1.28253	0.447649	-0.36106	-1.09277	0.220568	-0.80031	-0.2767	-0.763	0.681546	0.771753	-0.47461	-0.9185	-0.73407
Q00839	HNRNPU	0.983047	-0.89261	-0.0419	0.473295	0.075363	0.558722	-1.00766	0.750414	-0.18093	-0.02749	0.324449	0.161525	-0.37659	0.144873	0.3752	-0.43033	0.34821	0.026959	-0.47564
Q00978	IRF9	0.832596	-0.09673	-0.16093	-0.11291	-0.14083	0.243978	0.067798	1.2501	-0.61369	-0.43171	-0.1166	-0.05546	-0.30225	-0.06728	-0.73536	-0.27552	0.015258	0.501603	-0.54971
Q01081	U2AF1	0.272492	-0.08448	0.331935	0.424495	0.31387	0.29977	-0.86005	0.397464	-0.63565	0.39001	0.059994	-0.35067	-0.43284	-0.35061	0.197603	-0.31109	0.408465	0.457942	-0.17021
Q01082	SPTBN1	-0.32153	-0.12372	0.162035	-0.56082	-0.55105	-0.69463	0.490488	0.560722	-0.17603	-0.20217	-0.0375	-0.96606	-0.60348	-0.7501	-0.03261	-1.0044	-0.91635	-0.1184	-0.04012
Q01085	TIAL1	-0.00197	-0.44715	-0.14589	-0.04345	0.135927	0.298194	-0.35393	0.49368	0.635931	0.139038	-0.00195	-0.03448	-0.08982	-0.19197	-0.11414	0.090108	0.143869	0.199649	-0.65652
Q01105	SET	1.341169	-1.80946	0.268406	0.18923	0.645013	0.759479	-1.5421	1.417048	-0.45963	0.00842	1.02064	-1.03759	0.050475	-0.69443	0.066616	-0.24886	-0.38499	0.244278	-0.08411
Q01130	SRSF2	0.691808	-0.40158	0.636489	0.340619	0.326427	0.739591	-1.7403	0.837231	-0.03396	0.03174	0.785491	-1.29922	-0.15226	-0.84481	0.596497	0.482378	-0.30102	0.721602	0.266958
Q01167	FOXK2	0.471044	0.305642	0.287456	0.180748	-0.54841	-0.5579	0.140922	-0.49665	-0.10152	-0.21117	0.15268	0.18725	0.123528	0.008229	-0.39094	0.158156	-0.04208	0.075969	0.033399
Q01196	RUNX1	-1.17941	0.463287	0.290102	-1.21241	-0.06687	-0.58949	0.268516	-0.41335	0.809754	-0.2267	-0.23851	0.123281	0.247663	-0.19464	-1.66013	0.518227	0.259275	0.766182	1.253205
Q01201	RELB	-0.49357	0.265977	0.363476	-0.71147	-0.41607	-0.0735	-0.02719	0.393459	0.118939	0.77198	-0.05705	0.182406	-0.24884	0.277149	-0.34166	-0.50902	0.333154	-0.44702	-0.50489
Q01415	GALK2	0.108134	0.190932	-0.031	-0.91201	0.140058	-0.38834	-0.04341	-0.92244	-0.2608	-0.44302	-0.21302	0.166389	-0.03489	0.022653	-0.46258	-0.21261	-0.13075	-1.23251	0.05996
Q01432	AMPD3	-1.53724	0.433653	1.037354	-0.9806	0.001163	0.024477	-0.11578	-0.80162	0.174071	1.060215	-0.27661	0.894992	-0.04209	1.607939	-0.5799	-0.77876	0.18857	-0.13662	-0.97679
Q01433	AMPD2	0.085563	0.174516	0.083038	-0.19699	-0.71771	-0.49707	-0.91777	-0.10487	0.289881	0.249061	-0.00703	-0.38116	0.436131	-0.0762	-0.06165	0.009207	-0.12376	0.335262	0.547343
Q01469	FABP5	-1.02815	1.262436	-1.01514	-1.1778	0.849917	-0.92493	0.517511	0.434584	1.052972	1.411809	-1.2587	-0.36524	0.76061	0.070387	-1.79912	-1.6281	-0.0614	-0.05381	-2.15954
Q01484	ANK2	-1.35703	-0.18373	0.325501	-1.33335	-0.62937	-1.63123	0.144061	-1.88249	0.151477	-0.97521	0.274237	-0.58342	-0.68168	-0.3924	0.899737	-1.50458	-0.67928	-1.43291	-1.15933
Q01518	CAP1	-0.71558	0.394464	0.413721	-0.04462	0.508477	0.655	-0.26704	-0.44034	0.074324	0.651406	-0.75033	-0.38817	0.514553	0.159885	-0.05905	0.393915	-0.53491	-0.05124	0.058775
Q01581	HMGCS1	-0.60656	-0.01533	0.391477	-0.05305	0.229097	-0.32402	-1.17417	-1.01831	0.040847	-1.77595	-0.7966	-0.08081	-0.03841	-0.07076	-0.72303	-0.90688	0.75448	-0.00177	-0.2681
Q01650	SLC7A5	-0.42662	-0.14031	-0.10167	-1.12224	-0.30956	-0.51309	-0.69395	0.376662	0.785867	0.25186	0.368012	0.539507	0.529529	0.190981	-0.9515	0.578327	0.716863	0.621081	-0.29649
Q01658	DR1	1.132071	-0.58196	0.322267	0.012754	0.515523	0.532641	-0.62208	0.135538	-0.13825	0.295353	0.00351	-1.00715	0.414603	-0.44054	-0.19658	-0.34326	-0.10916	0.381386	-0.63171
Q01664	TFAP4	1.961944	-0.16545	-0.09906	0.024518	-0.35002	-0.28948	0.030525	0.586441	-0.60914	-0.27161	0.095194	-0.77123	-0.00141	-0.41302	0.332741	-0.33539	-0.15449	0.511303	-1.34776
Q01740	FMO1	-1.57394	-0.37946	1.238848	-0.85357	-1.75224	-0.84149	-0.21944	-1.35706	0.515094	-1.18089	-0.79821	-1.66261	-1.77988	-1.63109	-1.38324	-0.29076	-1.95047	-0.44789	-0.62898
Q01780	EXOSC10	0.624707	0.128625	0.088618	-0.14249	0.204503	0.403723	-0.02166	0.422421	0.902744	-0.14858	0.465126	-0.54336	-0.90028	-0.28221	-0.12622	0.240573	-0.08827	0.176963	0.019639
Q01804	OTUD4	-0.08377	-0.23503	-0.21125	-0.31949	0.030169	0.502173	-0.36574	-0.4029	-0.04197	-0.05461	0.868457	-0.56462	-0.2086	-0.52963	-0.44083	0.863453	-0.48669	-0.57452	-0.33576
Q01813	PFKP	0.421445	-0.73275	-0.37761	1.159015	-0.29546	0.326717	-1.20875	0.808237	0.118392	-0.48743	0.405693	0.313354	0.533405	0.141385	-1.00588	0.660671	1.94625	-1.14684	-0.32192
Q01814	ATP2B2	-0.35413	0.755416	-0.42494	-0.48146	-0.87589	-0.73436	-0.45071	-0.22791	0.001453	-0.31233	1.780452	0.107425	1.25881	-0.93211	2.461614	0.391417	1.271664	-0.06436	-0.86193
Q01831	XPC	0.89127	-0.12638	0.022056	-0.87964	0.46464	0.471469	-0.55894	0.069048	-0.25053	-0.22608	0.677839	-0.51387	0.201451	-0.82325	-0.89668	0.00261	-1.16353	-0.29118	-0.55304
Q01844	EWSR1	0.102718	0.09523	-0.14301	0.51561	0.516067	0.231648	-0.52869	0.142282	-0.55276	-0.50305	0.481269	-0.46495	-0.45833	-0.00131	-0.00182	-0.70844	0.202599	0.491949	-0.68618
Q01968	OCRL	-0.13981	0.564972	0.056583	-0.16787	0.061097	0.312878	0.247387	0.170255	0.836053	0.194876	-0.12876	-0.16281	0.258908	0.049239	-0.58896	0.143707	-0.15531	-0.1863	0.117652
Q01970	PLCB3	-0.14401	0.079532	0.495506	-0.01348	0.042236	-0.39916	-0.41588	-0.04092	0.434488	0.126476	-0.26321	-0.30467	0.538489	-0.29785	0.376755	0.932523	-0.54263	0.170646	-0.05552
Q01973	ROR1	0.185981	0.318635	1.450148	-0.92979	0.167685	-0.43723	0.046563	1.276225	0.193707	0.529372	-2.3802	-0.0526	0.421807	-1.35611	-0.06351	-0.53858	-1.91795	0.600274	2.404133
Q01974	ROR2	-1.05623	1.254116	0.84409	-1.43712	-0.25502	-0.78361	0.948984	-0.85521	1.242294	0.180755	-1.85333	-1.15675	0.148367	-0.73798	0.696657	0.313852	-1.44273	0.665257	1.611932
Q01995	TAGLN	-2.119	-0.97935	-1.296	-2.31894	-2.09542	-1.51568	-1.82299	-1.37331	1.2603	-1.57886	-2.44903	-0.68054	1.491902	-1.21101	-0.79597	-0.70499	-1.91549	-1.20959	0.945184
Q02040	AKAP17A	0.196317	0.520441	0.160101	-0.15341	-0.28684	-0.55143	0.098884	0.458741	0.096361	-0.06409	0.589077	-0.1438	-0.26069	0.050423	-0.48705	0.141662	0.233011	0.83896	-0.35534
Q02083	NAAA	0.641004	1.068836	0.842443	-1.57743	-0.19788	-0.73178	0.237489	-1.64008	0.073263	0.459445	0.050039	-0.18666	-0.07771	0.196611	-0.78515	-0.72917	-0.59742	0.630666	-1.77832
Q02108	GUCY1A3	-0.55643	0.392959	-0.04972	-0.90497	-0.08003	-0.97919	0.450493	-0.42656	0.522905	-0.2251	-1.51804	-0.57783	-0.20511	-0.59538	-0.40514	-0.71122	-0.30615	0.155289	-0.89692
Q02127	DHODH	-0.49526	0.170565	-0.35556	0.219129	0.276683	0.191207	-0.20095	-1.5196	-0.19113	-0.0251	0.559425	0.152356	-0.2885	-0.17755	0.279739	1.988829	-0.50364	-0.58196	-0.9752
Q02153	GUCY1B3	-0.92813	0.299315	0.145814	-1.42481	0.044304	-0.43798	-0.0009	0.143906	0.639366	-0.11713	-1.64628	-1.77093	-0.51032	-1.09206	-0.43873	0.210668	-0.67184	0.51823	-0.44485
Q02218	OGDH	-0.46476	-0.20495	0.505874	-0.04596	-0.15853	-0.42015	-0.52609	-0.09277	-0.13259	-0.07358	0.20539	0.183769	-0.03345	0.114527	1.985189	0.519985	0.078737	0.11944	-0.30371
Q02224	CENPE	0.333263	0.21272	-0.12815	0.30769	-0.36428	-0.0918	-0.25518	0.343107	0.048266	0.065624	0.33057	-0.70187	0.141426	-0.50984	-0.13968	0.153325	-0.05523	-0.41765	-0.00469
Q02241	KIF23	0.847247	-0.23619	-0.06119	0.177364	1.030437	1.458967	-0.11855	1.384892	0.090621	-0.26463	-0.67069	-0.34895	0.412328	-0.68801	-1.30296	-0.27924	0.081403	0.566875	0.16199
Q02252	ALDH6A1	0.365368	-1.35603	-1.1863	0.911315	-0.12992	-0.35586	-0.99324	-0.51179	-0.23398	-0.10946	1.243083	-0.55084	-0.69217	0.145821	0.876424	2.016286	-0.39086	-0.06621	-0.55795
Q02318	CYP27A1	-0.71415	1.589605	0.804898	-1.53489	-0.6459	-1.28826	-0.1737	-1.48668	-0.54997	0.306543	-0.68065	1.04131	-0.45482	1.0858	-1.56978	-1.57857	0.713877	-0.43688	-1.03292
Q02338	BDH1	0.812915	-1.34806	-0.33735	-0.53909	1.591823	1.291862	-1.28726	-1.00646	-0.43338	0.065958	1.198101	-0.6447	0.043036	-0.03129	4.507426	1.476752	-0.88401	-1.22974	-1.79205
Q02388	COL7A1	-1.80821	-0.57211	-1.06139	-1.3053	-1.49806	-2.51	0.650502	0.751079	-2.18773	-0.64046	2.069467	-1.02427	-0.59293	-0.34961	-0.92929	-1.39799	-0.51429	-1.39119	-1.0595
Q02413	DSG1	-1.50637	2.9823	0.604199	-0.84063	-1.99495	-1.22674	2.028498	-0.85908	0.950655	-1.39469	-0.78195	-0.74996	0.079359	-0.35362	-0.82917	-0.55086	-0.68405	-0.86905	-0.78184
Q02447	SP3	0.615971	0.234071	0.013305	0.372249	-0.19386	-0.48102	-0.01386	0.477598	-0.23598	-0.61854	0.594728	-0.32479	-0.07692	-0.5203	0.060688	0.226144	-0.07428	0.347058	0.100384
Q02487	DSC2	0.625516	-0.49251	-1.14337	-0.14214	0.208748	-0.3252	-0.82491	-0.0505	-0.04986	-0.15651	0.710032	-0.54338	0.273997	-0.47338	-0.65634	0.552209	-0.33257	-0.97356	-0.85369
Q02543	RPL18A	0.236718	0.031129	-0.0035	0.60399	-0.38735	0.201893	0.016145	0.288086	-0.22435	-0.61431	0.625501	-0.2462	-0.18259	-0.00536	0.387587	0.031049	0.272376	-0.04125	-0.03349
Q02750	MAP2K1	-0.02686	0.394037	0.44776	-0.16335	0.149198	0.335352	-0.09335	-0.49965	-0.82014	0.047113	-0.51144	0.75963	0.566534	0.921844	-0.578	0.137978	0.181549	-0.48205	-0.36927
Q02790	FKBP4	1.059912	-1.20667	-1.59327	0.046364	-0.22031	0.106306	-1.36462	-0.37018	0.175144	0.141021	-0.23855	0.028685	1.064336	-0.40951	-0.59824	1.204867	-0.2703	-0.9831	-0.55854
Q02809	PLOD1	0.20173	0.702899	0.386564	-0.60185	0.22827	0.033517	-0.37616	0.375313	0.549524	0.110502	-0.74068	0.733638	0.874789	0.24787	-1.17496	0.612004	1.332909	0.884707	1.452708
Q02818	NUCB1	-0.09877	-0.72506	0.447324	0.152662	0.480926	0.190625	-0.37934	0.153378	-0.18139	0.024276	0.252849	-0.37412	1.097475	-0.02976	1.287431	0.912677	0.110172	1.270228	0.525936
Q02833	RASSF7	0.038291	-0.19841	0.044079	-0.25657	0.14095	0.508888	0.137036	1.030932	0.037694	0.063845	-0.0541	-0.2366	0.132513	-0.03831	0.387644	-0.1602	0.273286	-0.35877	-0.14721
Q02878	RPL6	0.407142	-0.00085	0.15278	0.510733	-0.0535	0.173489	0.07745	0.035339	0.039654	-0.67695	0.416977	-0.19414	-0.09459	-0.24605	0.126168	-0.29706	-0.24789	-0.04959	-0.1664
Q02880	TOP2B	1.040788	-0.43634	-0.26318	0.174665	0.349829	0.581328	-0.29722	0.337776	-0.58872	-0.5463	0.093581	-0.51219	-0.56095	-0.26901	-0.29008	-0.57077	-0.66906	-0.28837	0.114317
Q02952	AKAP12	-1.14038	-1.30734	-0.3056	-2.23521	-1.95655	-2.45107	-0.54426	-1.55019	-0.24034	-0.7806	-0.8218	-1.7027	-0.75665	-1.50031	0.541079	-0.49732	-0.78877	-0.05377	-0.41262
Q02978	SLC25A11	1.12815	0.264293	-0.30925	-0.19735	-0.09416	-0.33573	0.676676	-0.41386	-0.27793	0.204158	0.142344	0.446787	0.485846	-0.00575	1.175174	0.379324	-1.05537	-1.00351	-0.57588
Q03001	DST	-0.89926	0.745797	0.361391	-0.47148	0.117841	-0.0968	0.085193	0.167189	-0.32666	-0.15985	-0.45792	-0.37241	0.084688	-0.07469	-0.7734	-0.61891	-0.65467	-0.02404	0.942482
Q03111	MLLT1	0.739907	-0.3585	-0.18777	-0.30455	-0.6477	-0.04836	0.097379	0.419759	-0.0976	-0.39984	0.195335	-0.49899	-0.34881	-0.22446	0.462625	-0.24792	-0.33453	0.019083	0.207111
Q03112	MECOM	0.677461	-0.45843	-1.08568	-0.03909	-0.58454	0.658071	-0.53604	0.355147	-0.49744	-2.14705	0.33339	-1.0276	-0.57331	-0.81388	1.09301	-0.59313	-0.44648	0.120366	0.089127
Q03113	GNA12	-0.61406	0.483638	0.279997	-0.85057	-0.09583	-0.59947	-0.28756	-0.30061	0.057267	0.385619	0.71937	0.172414	-0.19021	-0.21703	-0.42298	-0.24603	0.070178	0.438558	-0.05067
Q03135	CAV1	-2.09805	-0.17387	-0.10878	-2.59691	-1.27114	-2.12043	0.660595	-2.23409	-0.11272	-1.27288	-1.35156	-1.26537	-0.95345	-1.48042	-0.13108	-1.0082	-1.2059	0.143912	-0.8438
Q03154	ACY1	0.55164	-0.75375	-0.38082	-0.74311	0.685653	-0.48841	-0.04492	-1.12083	-0.02145	0.168277	0.147293	-0.4978	-0.44808	-0.1062	0.065607	-0.22151	-0.03461	-0.64895	-0.32937
Q03164	KMT2A	0.265932	-0.2715	-0.31344	-0.38193	-0.21842	-0.08527	-0.17671	0.491008	-0.3285	-0.02348	0.006371	-0.13844	-0.17429	-0.24491	-0.02908	-0.33362	-0.11256	-0.05188	-0.10008
Q03169	TNFAIP2	-0.78396	-0.50475	0.08306	-0.87599	0.298703	0.057945	-0.12218	-0.3702	-1.14583	0.424627	-0.19331	-0.23139	-1.4025	0.466097	1.468529	1.275456	-0.06388	-1.05855	-0.73679
Q03188	CENPC	1.067932	0.054645	0.723181	-0.69255	0.162332	0.380703	0.522098	0.368484	-0.33477	-0.01975	-0.07755	-0.2422	-0.00362	-0.11679	0.037262	0.536482	-0.58798	0.657301	-0.07346
Q03252	LMNB2	-0.80747	-0.22538	-0.5397	-1.01173	-1.05987	-0.95836	0.098663	-0.78967	-0.46736	-0.50445	0.759083	-1.36841	-1.0194	-1.41626	-0.57812	-0.83079	-1.13109	-0.05542	-0.55884
Q03426	MVK	-0.0723	0.169689	-0.55744	0.089229	0.633253	0.458023	-1.13344	-0.0221	-0.07747	-1.03658	-0.32368	0.362086	-0.01392	0.068493	0.02351	-0.06525	0.273771	-0.34662	0.513306
Q03468	ERCC6	0.168221	0.422265	0.079663	-0.24744	0.173547	0.279242	0.310491	-0.13312	0.022359	-0.16411	0.244115	0.176803	0.075082	0.314925	0.036321	0.269723	-0.11638	0.038977	0.006022
Q03518	TAP1	-0.73582	1.368267	1.137797	-0.74166	0.37028	0.342351	0.37958	-1.2931	-0.13762	-0.53687	-0.90998	0.587667	0.424672	0.453185	-0.88423	-0.69634	0.549279	-0.04144	-1.27717
Q03519	TAP2	-0.70884	1.74776	1.341289	-0.44967	0.632793	0.64308	0.738303	-1.33516	-0.50708	-0.28355	-1.15579	0.857248	0.720232	0.535397	-0.97588	-0.54342	0.903189	0.255078	-1.32672
Q03692	COL10A1	-3.71279	-2.07866	-1.71348	-4.28492	-3.54743	-4.017	-0.27537	-3.23105	0.902508	-1.78639	-2.92576	-2.529	-4.79588	-0.96424	0.282329	-1.44815	-1.39572	-4.30234	-1.97237
Q03701	CEBPZ	0.948333	-0.16094	-0.36611	0.744463	-0.28733	-0.11438	-0.34402	0.30573	0.129633	0.194274	0.459695	-0.19987	-0.01712	-0.44657	0.178449	0.098702	-0.25352	-0.39555	-0.18084
Q04206	RELA	0.062417	-0.03678	0.370606	-0.04114	0.432545	0.623174	-0.38433	0.064044	0.097027	0.214929	0.211256	0.409479	0.335885	0.124542	-0.27065	0.065863	0.294479	-0.04835	-0.09992
Q04323	UBXN1	0.339934	-0.20027	0.166379	-0.11942	-0.23	-0.25664	-0.49685	0.23204	0.406952	0.905664	0.065549	0.51182	0.673526	0.04174	-0.39457	0.059497	-0.03948	-0.24507	-0.21199
Q04446	GBE1	-0.33493	-0.65374	-0.00069	1.102438	-0.2184	-0.34807	-0.60291	-0.01868	0.394466	-0.07408	-0.24413	-0.29436	0.234992	0.277945	0.441413	-0.20948	0.332823	-0.46074	0.092574
Q04609	FOLH1	1.113361	-0.21633	0.092359	-1.0928	-0.21383	-0.06462	0.229584	1.640694	-0.01994	0.118626	-0.2301	-0.63468	-0.34305	-0.60423	0.877814	-0.76686	-0.50256	-0.47589	-0.11058
Q04637	EIF4G1	0.528797	0.100536	0.379009	0.394522	0.395855	1.008763	0.06987	0.411239	-0.07913	0.351945	-0.31349	-0.34297	0.172518	-0.42523	0.680968	0.117297	-0.03059	-0.18411	0.346201
Q04656	ATP7A	0.42757	0.093921	0.404542	-0.00867	-0.56511	-0.26616	0.068717	-0.26492	-0.07164	-0.39641	0.035464	0.151398	0.069245	0.033904	0.13841	-0.1656	0.244864	-0.09886	-0.52408
Q04695	KRT17	-1.22492	-1.3101	-2.34809	-0.12321	-2.53281	-1.72818	-0.93749	0.688788	-1.46209	-2.96474	2.02098	0.193234	1.283976	-1.7092	-4.07138	-2.27312	-1.7899	-2.16991	2.007071
Q04721	NOTCH2	0.096184	0.157907	0.477938	-0.92057	-0.1188	0.12178	0.823939	-0.12779	0.312613	0.123826	-0.05389	-0.44776	-0.58454	0.24865	0.162934	-0.37017	-0.30739	0.288807	0.641936
Q04724	TLE1	-0.24966	-0.26334	0.235414	0.382719	0.654645	0.426775	-0.10336	0.794121	-0.30399	0.01637	-0.16428	-0.40431	-0.10252	-0.22205	0.043039	0.475966	0.287655	-0.3575	-0.30191
Q04726	TLE3	-0.40458	0.038071	0.074128	-0.37914	-0.55491	0.419355	-0.10004	-0.30216	-0.26559	0.885149	0.025233	-0.93314	-0.03553	0.159977	0.176907	-0.64931	0.033752	0.618597	-0.11923
Q04759	PRKCQ	0.485861	-0.2709	-0.1	0.299371	-0.39935	-0.03491	-0.05621	0.767283	0.216974	0.295051	-0.07606	-1.35961	-0.07079	-0.28661	0.853103	0.143593	-0.74818	-0.51928	-0.01352
Q04760	GLO1	0.595814	-0.9556	-1.67115	-0.38642	1.724288	-0.24906	0.512983	-0.83265	1.62766	1.083042	-0.8818	0.272336	0.326188	0.37487	0.651333	-0.29089	2.037107	0.359868	-0.88284
Q04837	SSBP1	-0.10567	-1.06624	-0.09783	2.351567	0.430864	0.227958	-0.99337	1.058336	-0.50904	0.177594	-0.15775	-0.38798	-0.6567	-0.32771	1.692364	1.213983	-0.35192	-0.6402	-0.47725
Q04864	REL	-0.07559	0.474345	0.289491	-0.13153	-0.02557	0.112184	0.094323	-0.36302	0.104631	0.63292	-0.08042	1.020342	0.112524	1.383707	-0.04802	-0.46398	0.205291	-0.14465	-0.20428
Q04912	MST1R	-0.4845	-0.79893	-1.02682	-0.11979	0.190219	0.154236	-1.40598	0.033425	0.635931	-0.10364	0.212682	0.148581	0.034433	-0.04132	0.344371	0.438987	-0.28022	-0.04373	-0.81366
Q04917	YWHAH	-0.32656	0.188941	0.265683	0.369492	0.688573	0.704509	-0.61083	-0.55065	0.093656	0.790102	0.144651	-0.33519	0.263007	0.201364	-0.33128	0.122487	0.260795	0.391839	-0.11813
Q05048	CSTF1	0.109459	-0.12108	0.60454	0.336273	0.205164	-0.13004	0.008659	0.526996	-0.56056	-0.29589	0.654117	-0.38372	0.198061	-0.39674	-0.01793	-0.27759	0.077255	0.331347	0.045391
Q05084	ICA1	0.334682	-0.13215	-0.05327	-0.29783	-0.03805	-0.09625	-0.02437	-0.06112	-0.2558	0.274605	0.683813	-0.0808	-0.13603	-0.33357	1.029274	0.653817	-0.06727	-0.27824	-0.23552
Q05086	UBE3A	0.512493	0.088813	-0.38991	-0.28754	-0.36148	-0.14335	-0.18903	-0.59183	-0.59709	-0.22771	0.458866	-0.05208	-0.06272	-0.23871	-0.35939	0.497559	0.615273	0.056853	0.042112
Q05193	DNM1	-0.98623	-0.12395	0.964759	-1.35436	-0.16821	-0.91049	0.34685	-0.18488	0.098157	0.536661	-0.21882	-0.49352	-0.2933	-0.61229	-0.57127	-0.17027	-0.94406	0.849307	0.981579
Q05209	PTPN12	-0.06574	0.469687	0.506205	-0.84556	-0.06981	-0.45121	0.264031	-0.22781	1.905077	0.70899	-0.18693	0.355553	0.675996	0.665091	-0.09547	-0.28027	0.072097	-0.17763	-0.0182
Q05397	PTK2	0.64165	-0.16806	0.17422	-0.88146	-0.57597	-0.42161	-0.42838	-0.18912	0.836208	-0.06258	-0.11458	-0.06305	0.657196	-0.32202	0.178344	0.471749	-0.052	-0.10041	-0.15739
Q05469	LIPE	-1.91258	-0.83323	-1.04586	-1.99579	-2.30844	-2.63599	0.893246	-1.25988	-0.61239	-0.74625	-2.52158	-1.226	-1.34023	-0.54238	-1.6355	-0.79479	-1.58212	0.218064	-3.39103
Q05513	PRKCZ	0.045047	-0.5339	0.551203	0.160397	-0.29173	0.385526	-1.16287	0.459242	0.08841	0.328405	0.114114	-0.37162	0.253336	-0.56193	-0.59913	0.622765	1.009477	0.201121	-0.59369
Q05519	SRSF11	0.333415	-0.52131	-0.02292	0.143142	-0.18002	0.214046	-1.26666	0.392732	-0.51091	0.467581	0.418565	-0.18189	-0.35714	-0.14709	-0.07849	0.142653	-0.08432	0.392429	-0.08144
Q05639	EEF1A2	0.217397	-0.11222	-0.04044	0.57788	-0.21999	-0.46933	-0.11557	-0.06034	-0.02853	0.241109	-0.02968	0.714001	0.324875	0.46079	0.049207	0.224167	1.127833	0.205023	0.080641
Q05655	PRKCD	0.542692	-0.03274	0.54729	-0.26854	-0.26302	-0.1171	0.27316	0.595308	-0.0857	0.192083	-0.34609	0.849186	-0.63308	1.879222	-0.01544	-1.08769	0.544737	-0.89319	-1.02476
Q05682	CALD1	-0.87464	-0.47878	-0.84565	-1.17274	-1.84195	-1.18566	-1.09049	-0.84808	0.805094	-0.75895	-1.60303	-2.0967	0.379287	-1.50845	-0.61771	0.30577	-2.01522	-0.01418	1.318551
Q05707	COL14A1	-2.63513	-1.36668	-1.2923	-2.36853	-1.34584	-2.67873	0.127846	-3.06205	-1.02363	-2.02746	1.047396	-1.4366	-2.53552	-0.7082	0.150342	-1.85646	-0.60265	-1.59426	-1.49155
Q05823	RNASEL	-0.89737	1.401576	1.097679	-0.47851	-0.42844	-0.97425	1.159286	-0.32507	1.319181	-0.08786	-0.20298	0.335865	0.239003	0.72834	-0.8733	-0.53772	-0.10864	-0.08425	0.156222
Q05932	FPGS	0.135603	0.208059	0.986608	-0.28444	-0.02354	1.058279	-0.16299	-0.05807	-0.10143	0.084353	0.218932	-0.10392	-0.37331	0.222206	-0.34418	0.049167	0.184201	0.14686	0.036973
Q05D32	CTDSPL2	1.620401	0.652414	-0.55875	-0.05233	-0.30061	-0.00477	-0.38588	-0.01741	-0.23451	-0.76479	0.009926	0.001008	-0.88512	-0.31974	-0.49348	-0.00413	-0.14936	0.383378	-0.25055
Q06033	ITIH3	-0.1518	0.59647	-0.32036	-2.17406	-1.06585	-1.5336	-0.68262	-0.85757	0.586311	1.990539	-0.84444	0.401973	1.00471	1.160322	-0.34184	-1.17511	0.352058	2.305135	-0.07543
Q06124	PTPN11	0.477999	-0.48791	-0.13729	0.32202	-0.52286	-0.07696	-0.45037	0.393493	-0.13033	-0.02287	0.177599	-0.62505	0.233947	-0.61792	0.178661	0.148005	0.182497	0.165424	0.57693
Q06136	KDSR	-1.07291	-0.20085	0.51055	0.267518	-0.44381	-0.32857	0.30904	-1.0163	0.355174	-0.15734	0.272899	-0.0672	-0.40292	-0.37029	-0.43615	-0.15851	-1.07911	-0.6588	0.138982
Q06187	BTK	-1.27733	1.024298	1.210162	-0.90328	0.326298	-0.08888	0.083792	-1.3049	0.230339	1.247	-1.35667	1.39581	-0.16781	1.671637	-0.91141	-0.89254	0.7295	-0.3177	-1.00303

Q06190	PPP2R3A	-0.04995	0.156153	0.168873	0.277304	0.534723	0.409633	-0.03748	0.162896	0.100257	0.194377	-0.86684	-0.40758	0.05549	0.29718	0.797472	-0.12705	-0.24046	-0.03129	0.126708
Q06203	PPAT	1.271338	-0.73487	0.521523	-0.30508	0.493132	0.032065	-0.40472	0.046567	0.584052	-0.15553	-0.16237	-0.2294	0.07909	-0.51781	-0.60934	0.26152	0.383592	0.0694	0.166416
Q06210	GFPT1	0.489861	-0.78505	0.029709	-0.04614	-0.17058	0.25991	-0.84416	0.503274	0.149115	0.50607	-0.57875	0.54507	0.309312	0.050711	0.680296	-0.41748	0.814713	-0.55468	0.189519
Q06265	EXOSC9	0.984282	-0.35926	0.226876	-0.06777	0.82349	0.958354	-0.42097	0.400982	-0.4727	-0.34951	0.415133	-0.36196	-0.28439	-0.58108	-0.23639	0.285711	-0.30081	0.364523	-0.36505
Q06278	AOX1	-0.73606	0.165728	1.473816	-1.74239	-0.75311	-1.07887	0.48072	-1.13082	-0.01867	-1.00298	-0.68604	-0.82458	0.130237	-0.55852	1.132815	-1.15124	-0.80959	1.01782	0.015744
Q06323	PSME1	0.208225	-0.84374	-0.51208	-0.19482	1.330885	0.393455	-0.63265	0.561349	0.346937	0.804423	0.042155	-0.44733	-0.14257	-0.02238	-0.50371	1.341584	-0.13079	-0.25593	-1.30143
Q06330	RBPJ	0.486591	0.357605	0.047206	-0.34304	0.057256	0.06748	-0.07724	0.119022	0.172266	0.32637	0.190486	-0.13864	-0.51482	0.455399	-0.5135	-0.14507	0.087883	-0.02235	-0.11802
Q06481	APLP2	-0.48924	0.073754	-0.92468	-0.5722	-1.14691	-0.86071	-0.76319	-0.60574	0.162756	-1.04869	-0.55178	0.059777	0.633361	-0.34404	-0.77949	-1.55078	-0.16557	-0.34799	-1.01153
Q06546	GABPA	0.362859	-0.24851	0.195192	-0.09201	-0.24652	-0.05232	-0.03833	0.356339	-0.14531	0.001423	-0.00107	-0.22562	-0.39788	0.076467	0.024529	-0.27271	0.019729	0.121338	-0.15176
Q06547	GABPB1	0.321628	-0.01868	-0.0018	0.200559	0.002512	0.06617	-0.17236	0.647255	-0.06711	0.290834	0.345042	-0.24726	-0.25671	-0.14642	0.090092	-0.41508	-0.19792	0.232964	0.200333
Q06587	RING1	-0.17828	0.413619	0.126108	-0.07544	0.327184	-0.01775	0.26603	-0.26924	0.07864	-0.35783	-0.48169	0.202856	0.78674	0.419528	-0.13825	-0.16005	-0.03821	-0.30943	-0.36046
Q06710	PAX8	0.21501	-1.64726	-0.01117	-0.22679	-0.91542	-0.33652	0.728296	2.019525	-0.59338	-1.20453	0.010946	-0.92403	0.285121	-0.61657	1.665439	0.231012	-0.36425	0.247434	-0.1182
Q06787	FMR1	0.333582	-0.38436	0.253611	0.074801	0.624677	1.301984	-0.36482	-0.08722	-0.56645	-0.23115	0.150405	-0.5538	-0.09949	-0.72303	0.157651	1.146586	0.234324	0.521618	-0.73065
Q06828	FMOD	-0.89586	-1.93104	-2.38947	-2.4353	-1.01697	-1.07525	-1.46305	0.036847	-0.29176	-1.22846	0.451254	-0.98562	-0.3755	-0.26651	0.520115	-0.57986	-0.38509	-0.82032	-0.57654
Q06830	PRDX1	-0.255	-0.13505	-0.30677	-0.68396	0.973735	0.050555	-0.28298	-0.13347	0.359372	0.248261	-0.49328	0.451273	0.166262	-0.26269	-0.18287	-0.50692	0.539331	-0.47313	-1.06869
Q07002	CDK18	0.362942	-0.44825	0.005522	0.670028	-0.05517	-0.15897	-0.4775	0.460549	-0.097	0.436863	-0.28089	-0.0636	0.323299	0.607916	0.575525	-0.48479	0.659073	-0.46201	-0.49457
Q07020	RPL18	0.458849	-0.1427	-0.19379	0.316867	-0.05763	-0.12698	-0.3165	-0.08814	-0.08716	-0.77081	0.379719	0.422043	-0.53157	0.194042	0.242598	-0.2478	0.083476	0.080575	-0.13597
Q07021	C1QBP	1.356141	-0.22687	-0.55461	-0.33904	0.193701	-0.08894	-0.4462	0.138328	-0.05421	1.575183	0.61676	0.515964	0.77344	0.135634	-0.12124	0.347134	-0.52047	-0.96449	-1.03319
Q07065	CKAP4	-1.15982	1.289855	0.427678	0.391941	-0.2733	-0.24633	1.157167	0.180033	0.607785	-0.09044	-0.4419	-0.4721	0.799806	-0.6284	-0.88176	-0.1784	-0.38015	0.866143	2.043458
Q07075	ENPEP	0.135109	1.22541	0.659571	-0.3513	0.069809	-0.80482	0.497107	-0.34796	0.518388	-0.03792	-1.06873	-1.20087	0.377597	-0.85274	-0.22358	0.492023	-1.09758	0.167337	-0.14275
Q07092	COL16A1	-3.78335	-1.90248	-3.15531	-2.99295	-4.95474	-4.57803	-1.87493	-2.00785	0.308422	-2.60871	-1.72111	-3.72455	-3.00071	-1.04745	-0.38547	-1.96176	-1.12615	-1.00935	-0.60071
Q07157	TJP1	0.477006	-0.67721	1.603912	0.147132	-0.57789	-0.44086	-0.4712	0.513433	-0.17174	-0.31409	-0.29184	-1.12277	-0.07751	-0.94024	0.22649	0.471433	-0.5705	0.653715	0.504632
Q07352	ZFP36L1	-0.1411	0.429692	-0.23609	-0.12361	0.055475	0.385418	0.246496	0.44901	0.343031	-0.76479	-0.01773	-0.12546	0.567147	0.232699	-0.18635	-0.23719	0.321753	0.345923	0.226488
Q07507	DPT	-3.19663	-0.01077	0.667438	-2.48009	-0.72072	-0.26209	0.491234	-2.01539	-0.34113	-0.65616	-0.45428	-2.53717	-1.31394	-1.75618	0.192154	-1.80539	-1.70526	-0.09191	-1.74476
Q07617	SPAG1	-1.39284	-0.05323	-0.53171	0.047073	-0.03469	1.323412	-0.01969	0.416493	-0.16333	0.121906	1.990774	0.172713	0.132512	-0.4059	-1.13105	0.252615	-0.7008	-0.94242	-1.54669
Q07666	KHDRBS1	0.486359	-0.03362	0.358951	0.368329	0.121695	0.531916	-0.62866	1.045615	-0.30594	-0.2027	0.400758	-0.2901	-0.13261	-0.55265	-0.08573	-0.0353	-0.19658	0.413593	0.109073
Q07812	BAX	-0.34598	0.582787	-0.22091	-0.78284	-0.10644	0.27536	0.098885	-0.1666	-0.30719	-0.11283	-0.13293	0.115884	-0.01854	0.462408	0.300844	-0.26571	-0.06214	-0.1035	-0.3584
Q07817	BCL2L1	0.132428	0.077465	0.531456	-0.04529	0.011917	0.022867	0.06811	1.166234	-0.31043	-0.5621	0.340501	-0.07958	0.656991	-0.47018	0.790204	0.302793	-0.41466	-0.92255	0.273926
Q07820	MCL1	-0.26659	0.394593	0.321314	-0.51482	0.144725	-0.06296	0.435435	0.417027	0.405682	0.190901	-0.32936	-0.41088	-0.53742	-0.0394	0.722493	-0.1755	-0.10733	-0.22869	-0.31976
Q07864	POLE	0.615647	-0.0011	-0.10357	-0.69266	0.98932	0.627951	-0.32599	0.265164	-0.40852	-1.26608	0.235503	-0.47202	-0.17514	-0.27178	-0.94389	0.493837	0.028192	0.157745	0.047171
Q07866	KLC1	0.16194	0.131227	-0.10214	-0.06815	-0.1654	0.125756	-0.62499	0.007353	-0.6442	0.28938	-0.33089	0.012961	0.074132	0.110146	-0.34413	0.065418	0.091426	-0.45323	0.628122
Q07889	SOS1	-0.20138	-0.06915	-1.19979	-0.68724	-0.5546	-0.7045	1.155707	0.300031	0.335604	-0.13714	-0.38419	-0.24224	-0.00164	-0.14812	-0.36416	0.38811	-0.67583	-0.43127	-0.20528
Q07954	LRP1	-1.18002	0.934553	0.563233	-0.56872	-0.43913	-0.62794	0.829638	-0.31652	0.477423	0.130609	-0.7534	0.12242	0.439305	0.351638	0.267611	-0.18112	-0.31796	0.475166	0.965247
Q07955	SRSF1	0.648311	-1.38387	-0.01735	0.111603	0.221168	0.321201	-1.4079	0.452209	-0.38084	-0.14091	0.543577	-0.47287	-0.26543	-0.38294	0.083376	0.21842	-0.04044	0.393247	-0.23738
Q07960	ARHGAP1	-0.77368	0.434931	0.165318	0.278882	0.012703	0.150941	-0.11937	-0.57968	-0.00725	0.014228	-0.38125	0.076899	-0.15108	0.33139	0.060924	0.289155	-0.1307	-0.10174	0.686437
Q08170	SRSF4	0.816729	-1.15821	-0.09381	-0.08148	0.644278	0.569619	-0.78715	0.730296	-0.6491	0.33404	0.56467	-0.91472	-0.0852	-0.65001	0.091088	0.45804	-0.14298	0.811807	0.031835
Q08174	PCDH1	-0.20513	0.14726	0.016784	-0.61915	-0.66416	-0.41905	-0.0429	-1.20838	-0.51602	0.233982	-0.17327	-1.1529	-0.25996	-1.09257	1.509131	-0.91557	-0.80287	-0.92978	-1.49572
Q08188	TGM3	-1.58574	2.449589	0.456146	-1.4515	-1.33991	-1.14398	1.443816	-0.8409	1.617098	-0.33357	-0.50298	-0.42227	-0.05731	-0.16276	0.212348	-0.19691	-0.29065	-0.6314	-0.25434
Q08209	PPP3CA	-0.56458	-0.48365	0.019706	-0.83468	0.286002	0.093916	-0.68728	-0.08169	-0.28934	0.363544	0.369666	-0.6556	0.097401	-0.07857	0.179218	-0.09937	-0.83186	-0.14716	0.984966
Q08211	DHX9	0.286974	-0.22664	0.037868	0.357521	-0.06269	0.12192	-0.3918	0.617531	0.051343	-0.13845	0.183588	-0.23822	-0.33344	-0.13544	0.248428	0.047829	-0.04179	-0.02894	0.140982
Q08257	CRYZ	0.805696	-0.94322	0.375141	0.394025	0.431011	0.416254	-1.20163	-0.77432	-0.23195	-0.8886	0.570139	-0.8658	-0.60413	-0.55956	1.099837	2.239047	0.249032	0.873762	0.251456
Q08345	DDR1	0.780014	-0.57934	0.127838	0.107951	0.064424	-0.0415	0.815229	-0.34854	0.497715	-0.20288	0.034558	-0.48055	-0.08105	-0.82009	0.45079	1.168351	-0.60153	-1.4106	0.111977
Q08357	SLC20A2	-0.25247	0.089075	0.083968	-0.13902	-0.61336	-0.66103	0.008935	-0.92166	0.233783	-0.35624	-0.20401	0.046551	-0.25938	-0.95241	1.120815	-0.66719	0.458349	1.301918	-0.36868
Q08378	GOLGA3	-0.15417	0.29015	0.144781	0.035187	-0.21428	-0.39179	0.560508	-0.2328	0.352548	-0.48931	-0.22038	-0.51824	0.262853	-0.37584	0.445846	-0.3448	0.057047	0.577548	0.424973
Q08379	GOLGA2	-0.18504	0.504976	0.506955	-0.50766	-0.06107	-0.03741	0.542006	-0.44433	0.232214	-0.28075	-0.12344	0.011735	0.182061	-0.15201	0.149281	-0.04602	0.327334	1.052957	0.335544
Q08380	LGALS3BI	1.534071	-0.51351	0.254699	0.198125	-0.33042	-0.06186	-0.18725	2.134078	0.398685	-0.32889	0.305906	-0.40924	0.506106	-0.15762	-0.72928	-0.20013	-0.3428	-0.44683	-0.67017
Q08397	LOXL1	-0.66564	-0.56052	-0.58314	-1.52081	-0.91925	-1.28172	-0.47075	-1.11627	0.825601	-0.64563	-1.74574	-0.41765	-0.48567	-0.10967	-0.45497	-0.51824	-0.23651	-0.95929	-0.42483
Q08426	EHHADH	-0.33973	0.246505	0.0107	0.987956	1.023696	0.897757	-0.34093	-0.12756	-0.00855	0.998326	-0.34813	0.460331	-0.29228	0.25632	1.086969	1.112951	-0.04753	-0.40857	-0.37669
Q08431	MFGE8	-0.83363	-1.11239	-0.93712	-1.1247	-1.66227	-1.46742	-0.86319	0.013121	-0.65676	-1.34965	-1.23817	-0.82634	0.117086	-0.88694	-0.069	-0.95683	0.737697	-0.57501	-0.7581
Q08495	DMTN	0.127349	0.429492	0.20197	-0.93662	-0.2467	-1.72492	0.32098	-0.45501	0.260314	-0.80515	-0.69457	-0.28613	-0.45246	-0.40246	-0.75477	-1.60861	0.362757	2.011605	-0.38884
Q08499	PDE4D	-1.38442	0.194506	0.251344	-0.42357	-0.69961	-0.45512	0.05956	-0.48199	0.040847	-1.172	-0.4622	-0.32829	-0.10776	0.061514	0.654367	-0.43049	-0.06029	-0.10801	0.376862
Q08554	DSC1	-2.07281	3.553782	-0.01653	-1.82905	-1.26821	-1.03721	2.008466	-0.78925	0.092337	-1.45778	-0.75228	-0.92125	0.325672	-0.73354	-1.14237	-0.54453	-0.96393	-0.66444	-0.76806
Q08623	PUDP	-0.72014	-0.15092	-0.56266	-0.90856	-1.22433	-0.57214	-0.10456	0.26931	0.316271	-0.06567	0.149612	-1.10407	-0.41455	-0.8859	-0.8668	-0.31399	-0.57072	-0.64008	-1.11411
Q08722	CD47	0.342362	0.147362	1.120557	0.771512	0.081692	-0.09365	0.349071	0.731916	-0.834	-0.14818	-0.4917	-0.08279	0.222349	0.056359	0.309467	-0.52138	-0.48724	-1.12443	-0.1281
Q08752	PPID	-0.26753	-0.45391	-0.21237	-1.82308	0.876754	1.372397	-0.89822	0.284279	-0.69949	0.408274	0.956102	-0.93337	-0.73814	0.081964	-0.81171	0.029561	-0.06599	-0.52114	-1.27671
Q08945	SSRP1	1.598433	-0.59467	-0.40335	0.500686	-0.2602	0.081489	-0.53271	0.562371	-0.39754	-0.54743	0.21114	-0.15995	0.384856	-0.53072	0.436549	0.268124	-0.09184	-0.23541	-0.0995
Q08999	RBL2	-0.19259	0.049775	-0.14337	-0.046	-0.23078	-0.35105	-0.05088	-0.42181	0.026573	0.141276	0.443559	-0.34287	-0.13716	-0.40594	0.184781	0.033575	-0.38195	0.168051	-0.02403
Q08AD1	CAMSAP2	-0.17907	0.293809	0.061709	-0.90511	-0.783	-0.56823	0.009972	0.227266	0.263594	0.606578	-0.39876	-0.71128	0.095966	-1.26085	0.584846	-0.54975	-0.63858	-0.00802	0.43874
Q08AE8	SPIRE1	-0.21217	0.296539	-0.46074	-0.46877	0.268367	-0.26022	-0.90054	0.311323	0.193809	0.161069	-0.81298	-0.40958	0.301237	0.038104	-0.04295	-0.42915	0.15299	0.115715	-0.19387
Q08AF3	SLFN5	-0.71173	0.650784	0.856258	1.272477	-0.17253	-0.17922	-0.4499	-0.00829	-0.48637	-0.24125	0.983858	-0.02576	-0.03864	-0.69116	-0.07625	0.260999	-1.14854	-0.50179	-0.19562
Q08AM6	VAC14	-0.27239	0.34187	0.213029	0.384375	-0.39704	-0.18892	0.206817	-0.24358	-0.07179	-0.16634	-0.35866	-0.06323	-0.50723	-0.00266	0.017979	0.010065	-0.8695	-0.66324	0.091169
Q08J23	NSUN2	0.541439	-0.27283	-0.32089	0.131943	-0.5289	0.041329	-0.84596	-0.24135	-0.40446	0.010021	-0.16083	-0.04232	-0.35848	-0.16103	0.311531	0.773219	0.648273	-0.50986	-0.19687
Q09028	RBBP4	0.960954	-0.73971	0.345316	0.528005	0.51017	0.591312	-0.27259	0.757914	-0.49743	-0.02755	0.107421	-0.94836	0.047967	-0.55632	0.527013	0.298652	-0.19287	0.382717	0.225446
Q09161	NCBP1	0.139176	0.127961	0.046834	-0.37026	0.744555	0.92599	-0.13035	0.508708	0.020009	-0.21896	0.183912	-0.05206	-0.39139	-0.22484	0.044397	0.113535	0.063197	0.149663	0.092342
Q09328	MGAT5	0.24669	-0.02194	-0.00251	-0.15495	-0.51498	-0.59691	0.280722	0.790293	-0.40147	-0.6853	-0.20453	-0.24361	-0.22543	-0.56981	0.9966	-0.1518	0.122747	-0.27449	1.023854
Q09472	EP300	-0.11952	0.212022	-0.0107	-0.69227	0.019454	-0.38696	-0.12804	-0.76391	0.252754	0.092351	-0.07278	-0.33186	-0.38539	-0.11726	-0.01425	0.389031	0.297141	0.179475	-1.26639
Q09666	AHNAK	0.753708	-1.04134	-0.94069	-0.04266	-0.69952	-0.56491	-0.67314	-0.57718	-0.47383	-1.16826	-0.22984	-0.69919	0.387509	-0.80843	0.924652	0.328113	-0.33426	-0.1771	-0.14977
Q0IIM8	TBC1D8B	-0.52565	0.29399	0.229127	0.467898	-0.23448	-0.0616	0.152165	-0.29026	0.177558	0.078269	-0.75894	-0.01166	0.35381	-0.44028	-0.51713	0.358464	-0.2882	0.088399	1.449258
Q0JRZ9	FCHO2	-0.58118	0.572325	0.617956	-0.00664	-0.07976	-0.49163	0.31717	-0.44715	0.322916	0.123963	-0.44914	-0.35282	0.086951	0.147168	0.079747	0.713777	-0.24432	0.439785	-0.68817
Q0VD83	APOBR	-1.58452	1.119115	0.796038	-0.21096	0.139905	0.515446	0.089485	-1.22551	-0.94595	2.159163	-0.63456	0.342502	0.632434	1.188898	-0.66159	-0.03916	0.539545	-0.26284	-1.02542
Q0VDF9	HSPA14	0.35593	-0.14602	0.191968	0.341356	0.587891	1.131373	-0.73675	0.352269	-0.36618	-0.31665	-0.20226	-0.11613	-0.09547	-0.03638	0.406589	0.475427	-0.26194	-0.66061	-0.19821
Q0VF96	CGNL1	-0.78421	0.063648	-0.48533	-0.56715	-1.25033	-1.24441	0.067893	-1.15952	0.15366	-0.52994	-0.56274	-1.37351	-1.07778	-0.49593	0.224941	-0.28403	-0.30292	0.487889	-0.61871
Q0ZGT2	NEXN	-1.94041	-0.97988	-0.82549	-0.72259	-1.02514	-1.05345	-1.40338	-1.5625	1.171445	-0.51109	-1.0877	-2.0724	-0.16644	-1.40852	-0.77285	0.062852	-1.98761	0.408676	1.254103
Q10469	MGAT2	-0.48393	1.239043	0.737417	-0.34419	-0.01057	-0.17554	1.666016	-0.28466	0.312636	0.337561	0.007948	0.047295	0.577175	0.003974	0.693657	0.365651	0.547018	0.45426	0.381897
Q10471	GALNT2	-0.34166	0.999009	0.234193	0.728944	0.067224	-0.07984	0.700237	-0.0534	0.392302	0.645303	-0.24154	-0.23573	0.511763	-0.12583	1.134878	-0.48734	0.197453	0.243846	0.564444
Q10472	GALNT1	-1.30391	0.732797	0.158417	0.469861	0.098984	0.063118	0.581802	-0.54906	-0.19222	0.297252	-0.43506	-0.36187	0.520337	0.381963	-0.67746	0.282104	-0.25433	-0.00999	0.866761
Q10567	AP1B1	-0.55789	0.760829	0.679294	0.025483	0.467099	0.13601	0.073673	-0.51261	-0.1564	0.016976	-0.01653	0.511343	0.131265	0.399857	0.091607	0.043518	-0.1269	-0.08428	-0.31387
Q10570	CPSF1	0.556946	0.304805	0.116622	0.021461	0.363652	0.412503	0.205204	0.038044	-0.10245	-0.10167	0.485844	-0.15333	-0.23357	0.015753	0.057385	0.073318	-0.12474	0.216016	-0.18529
Q10588	BST1	-2.94382	1.167269	1.212142	-0.57338	-0.65973	-1.06808	0.33456	-1.49626	-0.0884	-0.10176	-2.58407	0.510038	0.47971	2.325224	-0.35928	0.472384	-0.20098	-0.71285	0.357678
Q10589	BST2	0.104981	0.609463	0.879811	-1.53527	-0.75283	0.300556	0.612637	1.935919	-0.54495	-0.46889	-1.40868	-1.79977	-0.24106	-0.30607	-1.20488	-0.29753	-0.55868	-0.63186	-2.16912
Q10713	PMPCA	0.70688	-0.6034	0.46137	0.558461	0.584967	0.40368	0.096781	0.279718	0.234959	1.236791	0.624584	0.06925	-0.10989	0.124214	0.490468	0.555337	0.251869	0.380777	0.269896
Q11201	ST3GAL1	-0.34781	0.423792	-0.16478	-0.1301	-0.34502	-1.07826	-0.45478	-0.50571	0.040847	1.798662	0.060918	0.192319	0.103631	0.407423	-0.33666	-0.45203	0.012419	-0.14907	0.046945
Q12756	KIF1A	1.565022	-0.79974	0.462247	-0.43302	-1.1883	-1.27126	0.178815	1.329595	-0.40485	0.016995	-1.02175	0.0556	0.575795	-0.18463	0.395886	-0.06239	-0.12494	-1.65429	0.915297
Q12765	SCRN1	1.054897	-0.52722	0.035919	-1.15419	0.615444	-0.18419	-0.38141	1.125372	0.607975	0.261052	0.09897	-0.4921	0.169625	-0.52962	0.175394	-0.41119	0.508949	0.083821	0.027771
Q12767	TMEM94	-0.32863	0.423792	0.224626	-0.47883	-0.20486	-0.8228	-0.1859	-0.03285	-0.18585	0.453554	0.79733	0.241976	0.103631	0.120181	-0.33356	-0.08613	-0.18943	-0.20619	-0.06004
Q12768	WASHC5	-0.43781	0.627159	0.553004	0.003786	-0.00336	-0.08546	0.336765	-0.40933	-0.03182	0.013124	-0.52911	0.377992	0.216979	0.655496	-0.08101	-0.11852	0.09652	-0.26982	-0.01616
Q12769	NUP160	0.161749	0.28317	0.055563	0.075015	0.077238	0.142038	0.070848	0.185163	-0.66642	-0.1977	0.173596	0.120671	-0.28092	0.287728	0.779167	-0.05715	0.186123	-0.01902	0.117868
Q12774	ARHGEF5	0.049637	-0.33481	-0.11196	0.516163	-0.73204	-0.23904	-0.26424	0.600461	-0.47956	-0.11677	-0.35807	-0.66148	-0.28399	-0.27032	1.055993	0.350273	-0.3462	-0.74606	0.076339
Q12778	FOXO1	0.190208	-0.0678	0.289472	0.239366	-0.04576	0.090875	-0.46169	0.165686	-0.29916	0.331813	0.347951	-1.05978	-0.21689	-0.14809	0.493673	-0.68659	0.117665	1.197092	-0.35815
Q12788	TBL3	0.528994	-0.08924	0.042219	0.086709	-0.25117	-0.04142	0.092815	0.291146	0.61746	-0.2216	0.810846	0.023642	-0.33781	-0.32613	0.095013	0.392121	0.083579	0.08751	-0.16957
Q12789	GTF3C1	0.562882	-0.07369	-0.06661	0.274096	-0.05668	-0.08146	0.086677	0.432705	0.139638	-0.18165	0.073815	-0.19649	-0.04334	-0.22728	0.303635	-0.13405	-0.39962	0.183622	0.092183
Q12791	KCNMA1	0.376967	0.785958	0.067653	-0.42226	-0.39792	-1.72801	0.686517	-0.5717	0.040847	-0.18883	0.154459	-0.37312	-1.26382	-0.10186	-0.10778	-0.11969	-0.60913	-0.34404	-0.65561
Q12792	TWF1	0.089457	-0.19723	0.133935	-0.09494	0.114357	-0.05999	-0.73113	0.883252	0.047041	0.174772	0.264718	-0.68453	0.263558	-0.55923	0.014013	-0.28821	-0.17702	-0.36091	0.471497
Q12797	ASPH	0.100881	0.355573	0.206609	-0.92443	-0.28836	-0.21807	0.038478	-0.3845	0.071506	-0.47692	-0.09398	-0.03917	0.067689	-0.46553	-0.10917	-0.32598	1.292307	1.132814	0.435786
Q12800	TFCP2	0.536559	-0.35055	-0.20794	-0.11901	0.002933	0.167707	-0.8955	0.386079	0.708531	0.294304	0.16263	0.254455	0.052326	0.045469	0.087072	-0.43382	0.275088	0.426715	0.116078
Q12802	AKAP13	-0.5444	0.259383	0.38539	-0.54199	-0.11087	-0.33312	0.695769	-0.13055	0.160569	0.512899	-0.14739	-0.07282	-0.00934	0.393564	-0.6484	-0.31172	0.175352	0.548861	0.600789
Q12805	EFEMP1	-1.29972	-0.70628	-1.15166	-0.95029	-1.14138	-2.16614	-0.01757	-0.81808	0.153329	-1.00597	-2.26341	-0.61274	-0.61762	-0.19834	-0.09127	-0.44487	-0.48269	-1.53202	-0.80858
Q12816	TRO	0.295261	0.069086	-0.24987	0.193387	-0.46411	-0.23447	0.023965	-0.20545	0.364039	-0.08137	-0.25219	-0.67253	-0.12512	-1.06839	-0.76675	-0.34298	-0.4751	0.526194	1.430753
Q12824	SMARCB1	0.569019	0.035901	-0.43319	-0.04614	0.072415	0.297562	0.056238	0.545762	-0.54076	-0.34636	0.247665	-0.44027	-0.62035	-0.22089	0.422104	-0.43427	-0.44263	-0.03081	-0.02846
Q12830	BPTF	0.725799	0.049161	-0.01674	-0.05895	-0.19244	-0.03299	-0.00295	0.21814	-0.18529	-0.4369	0.132218	-0.4496	0.03187	-0.69218	-0.06266	-0.02721	-0.12888	0.083654	-0.24592
Q12840	KIF5A	-0.32793	-0.06004	0.093377	-0.01137	0.048099	0.222949	-0.04572	1.280497	0.150854	-0.01333	-0.469	-1.26325	0.125864	0.519217	0.69324	-0.18358	-0.50901	-0.01702	0.478786
Q12841	FSTL1	0.675554	0.772038	0.762222	-0.11219	0.620807	0.71184	0.086995	1.49996	-0.44117	-0.35024	-0.15117	0.220679	0.303461	0.101888	-1.39517	-0.2423	0.375653	-0.03326	1.199445
Q12846	STX4	-0.10528	0.144453	-0.02322	-0.1165	-0.21263	-0.23622	0.004592	-0.2311	-0.06347	-0.09534	0.643268	0.04907	-0.57552	-0.13298	-0.29815	-0.25504	-0.6728	-0.3134	-0.88628
Q12849	GRSF1	-0.09947	0.13894	0.796917	-0.25563	0.14481	0.237947	-0.51837	-0.29889	0.057951	0.029427	0.324568	-0.54791	-0.36372	-0.37638	-0.6368	0.089661	0.973961	0.709678	-0.20684
Q12851	MAP4K2	0.088247	0.943777	-0.71197	-0.47649	0.117832	0.120081	0.586721	-0.00552	0.040847	-0.36656	-0.52642	0.207562	0.267681	0.572302	0.437766	0.128258	0.186986	0.262655	-0.25397
Q12857	NFIA	1.773048	-0.59487	-0.20483	-0.30662	-0.29316	0.369138	0.218535	0.051695	-0.08663	-1.16933	0.26817	-0.84007	-0.02056	-1.08355	0.487773	-1.27968	-0.7683	-0.64328	0.457588
Q12860	CNTN1	-1.19862	-0.33334	-0.74515	-0.92735	-1.22664	-1.14972	-0.53554	-1.18528	0.375276	-0.86089	-0.4252	-0.95792	-0.37518	-0.77334	-1.17722	-0.64332	-0.66197	0.590648	0.41101
Q12872	SFSWAP	0.288787	-0.22252	-0.29677	0.111484	-0.32449	-0.08428	-0.02471	0.675346	-0.3477	-0.02421	-0.09465	-0.36234	0.10211	-0.26306	0.278543	-0.15119	-0.1191	0.402646	0.086229
Q12873	CHD3	0.86815	-0.30643	-0.43095	0.341602	-0.17607	-0.08705	0.179455	0.580782	-0.37741	-0.16455	0.23709	-0.56165	-0.26828	-0.59529	-0.00382	0.058123	-1.10233	-0.59144	0.376082
Q12874	SF3A3	0.316091	-0.17603	-0.02322	0.527876	0.101689	0.417231	-0.57436	0.457837	-0.25075	0.18628	0.482772	-0.25159	-0.30337	-0.44146	0.34819	0.072343	-0.23151	0.034647	-0.05719
Q12882	DPYD	-1.32585	0.136723	0.292984	-0.37239	1.269223	0.107788	-0.15942	-0.70398	0.242715	1.169656	-0.49922	0.390046	1.36093	0.532767	-0.67039	0.344151	-0.10412	0.624425	1.246583
Q12884	FAP	-1.84696	0.829802	0.292823	-1.04879	0.10075	-0.46736	-0.16915	-0.48292	0.600251	-0.06438	-2.02032	-1.41725	1.249723	-1.65052	-1.30191	0.664299	-1.00714	1.161265	2.757817
Q12888	TP53BP1	0.719259	-0.19763	-0.28673	0.725878	-0.98232	-0.63187	-0.19716	0.034566	-0.05608	-0.24522	0.478514	-0.74194	0.326143	-0.60752	0.445696	0.149476	0.06431	0.513985	0.774222
Q12893	TMEM115	-0.27133	1.354073	0.653517	-0.45666	0.147454	0.273889	1.300125	-0.28044	0.004771	-0.07741	-0.06033	-0.43773	0.557428	-0.56291	0.302478	-0.25286	-0.65199	0.643598	0.160773
Q12899	TRIM26	0.700094	0.097543	-0.0122	-0.13852	0.30258	0.309852	-0.47839	-0.99358	-0.20248	-0.16293	0.220122	-0.5095	0.606784	-0.07353	0.271262	0.48011	0.163865	0.49666	-0.31892
Q12904	AIMP1	-0.06207	-0.15973	-0.1114	0.102268	-0.28941	-0.25504	-0.38925	-0.07854	-0.52738	0.021737	-0.021	-0.40407	-0.12489	-0.29762	0.687725	0.470645	0.051848	-0.24501	0.00845
Q12905	ILF2	0.355342	-0.16997	-0.37612	-0.04245	-0.0581	-0.08122	-0.54193	1.078963	-0.5371	-0.1219	0.586613	-0.41565	-0.46728	-0.89054	-0.10054	0.029853	-0.34169	0.031263	0.116881
Q12906	ILF3	0.226406	-0.17432	-0.15257	-0.05046	-0.30517	-0.18521	-0.1811	1.007839	-0.29617	-0.41578	0.473896	-0.88147	-0.25308	-0.9843	-0.18046	-0.20109	-0.65414	0.231931	-0.09092
Q12907	LMAN2	-0.51084	0.532732	0.233635	0.673314	0.007206	-0.02161	0.815582	-0.01063	-0.03491	-0.23184	-0.03457	0.552835	0.731759	0.363125	0.611287	0.643382	1.204146	1.162884	-0.02563
Q12912	LRMP	-1.35727	1.146962	0.79024	-1.26467	0.579865	-0.15115	0.432048	-1.83786	-0.04483	0.70022	-1.08398	0.322029	-0.09587	0.859874	-0.41267	-0.4524	-0.385	0.971321	-0.83497
Q12913	PTPRJ	-0.84666	1.27505	0.981018	-0.36872	0.472885	0.172408	0.325101	-0.22053	-0.20477	1.050749	-1.06469	1.238898	0.346432	1.879892	-0.30944	-0.7282	0.726956	0.401301	-0.87536
Q12923	PTPN13	-0.19503	0.322676	0.020344	-0.03884	-0.41283	-0.45456	0.12215	-1.4388	0.185785	-0.61832	-0.6778	-1.17409	-0.21978	-0.38862	-0.26181	1.103454	-0.15334	0.402724	-0.00864
Q12929	EPS8	-0.04473	0.375436	0.273267	-1.19314	0.037058	-0.29531	0.686293	-0.54349	0.134722	0.207161	-0.20609	-0.26449	-0.20333	0.086034	-0.39656	0.981583	-0.18994	0.329894	0.220428

Q12931	TRAP1	0.327569	-0.30102	-0.18358	0.147374	-0.19184	0.039448	-0.2627	-0.17825	-0.4577	-0.00536	0.127889	-0.04032	0.158947	-0.40425	0.921008	1.47982	-0.01318	-0.3325	-0.40749
Q12933	TRAF2	0.856392	-0.0856	0.590447	-0.72584	0.126699	0.46844	0.655541	0.717696	-0.17052	-0.58865	-0.57695	-0.59704	0.028875	-0.05155	-0.72016	-0.37973	0.313756	-0.37974	0.149771
Q12955	ANK3	0.980044	-0.36813	-1.10207	0.295344	-0.11511	0.053395	-0.03152	0.957586	-0.48843	-0.7841	1.518368	-1.0634	-0.33271	-0.82502	-0.13099	-0.65392	-0.50114	-0.63245	-1.1624
Q12959	DLG1	0.449991	-0.65981	-0.14368	0.329822	-0.06832	-0.19742	-0.54776	0.445655	0.327749	0.259534	0.560496	-0.75946	0.000741	-0.73206	1.622805	0.339775	-0.38782	0.135065	0.475652
Q12962	TAF10	-0.10398	-0.01712	-0.63383	-0.29996	0.050871	0.276823	1.017202	-0.26347	0.348084	0.77416	0.112582	-0.76552	-0.02217	0.233613	-0.15138	-0.06954	-0.20628	0.363344	-0.4277
Q12965	MYO1E	-0.12406	0.23157	-0.01969	-0.01836	0.003809	0.11165	-0.17398	-0.65562	0.137813	0.199911	-0.45379	0.50497	-0.13753	0.29854	-0.64044	-0.13647	0.645099	0.253239	0.195651
Q12972	PPP1R8	0.965406	-0.35713	-0.44864	-0.06081	0.245679	0.303096	-0.65714	0.656287	0.69707	0.17228	0.508316	-0.11526	0.233514	-0.52139	-0.18357	-0.35009	0.12149	0.16426	0.210482
Q12979	ABR	-0.20987	1.08025	0.707506	-0.41536	0.218219	0.097789	0.086334	-0.33355	-0.15686	0.719988	-0.52564	0.686504	0.414845	1.08518	-0.86434	-0.02576	0.162745	-0.01453	-0.02401
Q12980	NPRL3	-0.28832	0.339195	0.37632	-0.24902	0.557511	-0.24722	-0.17442	-0.16262	0.00716	0.512895	-0.27724	0.001272	0.208464	0.561846	-0.20981	0.005639	-0.26245	-0.40297	0.272841
Q12981	BNIP1	-0.35678	0.854458	0.318345	0.170985	0.3103	-0.24939	0.720819	-0.02784	-0.69573	0.4409	0.020175	0.265591	0.207462	-0.11006	0.612416	0.125675	0.281043	0.256666	0.416334
Q12983	BNIP3	0.467149	-0.4577	0.041296	0.269493	-0.30865	-0.06113	-0.50178	0.693883	0.082127	0.250971	0.914341	0.162637	0.131418	0.25043	-0.05497	0.026801	0.792842	0.37931	-0.0856
Q12986	NFX1	0.119258	0.337945	0.453872	-0.83873	-0.09741	0.612562	0.213766	-0.43274	0.074118	-0.31535	0.48443	-0.21866	0.207059	-0.37698	-1.10483	0.273747	-0.3167	0.762221	0.046963
Q12996	CSTF3	0.182242	0.146743	0.309782	0.294074	0.211008	0.053403	0.051896	0.657035	-0.19618	-0.11887	0.714879	-0.33825	-0.04707	-0.59061	-0.40372	-0.18717	-0.1246	0.097301	0.182809
Q12999	TSPAN31	-0.03024	-0.23363	0.285776	0.368632	-0.836	0.746747	0.619593	-0.15353	-0.03262	-1.1069	0.170559	-0.23158	-0.15748	-0.23176	0.747213	-0.63125	0.34078	0.540672	0.202639
Q13011	ECH1	0.285721	-0.71866	0.063077	0.541941	2.404971	1.964815	-0.33929	0.967461	0.365377	0.356534	-0.04333	0.709762	0.457094	0.333038	1.04814	0.711293	-0.02111	0.419188	1.34113
Q13017	ARHGAP5	0.447249	0.269685	-0.36361	-0.4851	-0.13179	-0.08813	-0.18562	0.154856	-0.25798	-0.47864	0.455741	-0.52253	-0.34605	-0.28756	0.144441	-0.04874	0.155498	0.069336	0.821319
Q13029	PRDM2	0.887927	-0.12993	-0.16349	0.039829	-0.32837	-0.80636	-0.01234	0.301315	0.04114	-0.04102	-0.2392	-0.19623	-0.00278	-0.09044	1.469753	0.187542	0.131189	0.040603	-0.32495
Q13033	STRN3	-0.08576	-0.65209	-0.2348	-0.29796	-0.63127	-0.49824	0.024467	0.351848	0.387743	0.015375	0.252648	-0.32669	0.086815	-0.11683	0.49438	0.159788	0.249482	0.149652	0.346666
Q13042	CDC16	-0.33525	-0.0878	0.185117	0.173271	0.252604	0.871632	-0.32983	0.493898	0.238184	-0.26615	0.443923	-0.20728	-0.11384	-0.05271	-0.03016	0.143337	0.217364	-0.22682	0.357555
Q13043	STK4	-0.65742	0.06238	0.152219	-0.88401	-0.16614	0.010761	-0.18595	-0.59858	-0.20923	0.737782	-1.08157	0.4966	-0.36219	1.137195	-0.36058	-0.75202	0.250821	-0.07295	-0.01598
Q13045	FLII	-0.08611	0.189143	0.678446	-0.14951	-0.12661	0.069866	-0.45509	-0.18305	-0.3747	-0.08985	0.242461	0.569051	-0.12659	0.957902	-0.25928	-0.03109	0.181832	-0.70135	-0.3993
Q13049	TRIM32	0.500635	0.313322	-0.31436	-0.3906	0.333817	-0.01674	-0.57253	0.366813	-0.24791	-0.3572	0.123394	-0.4367	0.31799	-0.54193	-0.07619	0.295698	-0.1509	-0.39874	-0.36482
Q13057	COASY	-0.20056	0.317877	-0.29314	0.215854	0.852187	1.243151	-0.39988	-0.29337	-0.36044	0.839821	0.724977	0.318109	-0.69675	0.467243	0.050891	0.990125	0.2929	-0.25582	-0.22278
Q13075	NAIP	-1.88571	1.721178	1.235949	-0.96693	0.842732	0.324601	0.304974	-1.42442	-0.22808	1.667215	-0.78142	1.792424	-0.14357	1.695703	-0.4049	-0.23697	0.122533	0.099945	-1.00101
Q13084	MRPL28	-0.32775	0.182258	0.518992	1.103139	0.457275	0.094787	-0.75839	-0.40307	0.518655	0.198433	0.080978	0.238138	0.117538	0.130771	0.80479	0.056877	-0.17103	-0.09633	-0.59477
Q13085	ACACA	0.046579	-0.11201	0.050187	0.294476	0.026856	0.480027	0.10268	-0.50028	0.061165	-0.01037	-0.47843	-0.15666	0.194319	-0.33528	-0.48835	-0.09096	0.166363	-0.66858	-0.28018
Q13094	LCP2	-1.10199	0.944174	0.981275	-1.0116	1.236158	0.514746	0.11125	-1.09301	0.339886	1.425844	-1.21853	1.011673	0.032913	1.059673	-0.59316	-0.04944	0.764668	0.63819	-0.47395
Q13098	GPS1	-0.08835	0.071304	-0.11176	-0.14454	-0.34658	-0.21414	0.143774	-0.25118	0.850682	-0.11979	0.330021	-0.62509	-0.25661	-0.73168	0.103173	0.152908	-0.4349	-0.14113	0.356588
Q13099	IFT88	-0.60855	0.486939	-0.15702	-0.78834	-0.56998	-0.35903	0.119144	-0.63119	0.117553	-0.84398	1.501878	-0.58098	-0.24439	-0.68897	-0.50456	-0.76505	-0.73273	-0.26032	-0.58142
Q13103	SPP2	-2.60508	-1.97778	-0.87099	-1.05757	-3.82319	-3.07059	-1.30499	-1.24123	-0.92326	-2.42254	-3.86368	-1.02818	-4.01134	0.274358	0.009894	-3.10231	-0.43853	-2.20003	-2.04645
Q13107	USP4	0.229467	0.385293	-0.06344	-0.29298	0.213918	0.674056	-0.3708	0.517409	0.141963	0.343338	-0.20002	0.417673	-0.03939	0.81091	0.022485	-0.10834	0.067225	-0.63525	-0.08905
Q13114	TRAF3	0.256822	0.034563	0.300175	-0.39445	-0.35986	-0.69475	0.231566	0.155047	0.018893	0.183815	0.454281	-0.15431	0.14989	-0.11209	-0.52009	-0.02698	-0.20828	0.138125	0.281187
Q13123	IK	0.705547	0.255999	0.226364	0.014705	0.017307	0.42936	-0.28552	0.284809	-0.18938	-0.4313	0.532654	-0.58961	-0.19461	-0.33248	-0.00313	0.017112	-0.18608	0.584419	-0.29975
Q13126	MTAP	1.064467	-0.07056	-0.42536	0.162321	1.470826	0.04145	-0.50363	0.15784	-0.22399	-0.63284	-0.28553	-0.32547	0.297872	-0.25167	-0.17767	0.912021	-0.37606	-0.39325	-0.09854
Q13127	REST	0.267816	-0.4364	0.447182	0.038108	-0.06751	0.294222	-0.27563	0.005754	-0.61969	-0.32463	-0.01063	-1.03626	0.097768	-0.24806	0.698376	1.537789	-0.70985	1.239607	0.01024
Q13129	RLF	0.634539	-0.06643	-0.02271	1.181525	-0.17141	0.793038	0.163946	0.841434	-0.38518	-1.17749	-0.26639	-0.53496	-0.29464	-0.21582	0.052182	-0.10609	-0.10815	-0.03014	0.216817
Q13131	PRKAA1	0.088878	0.102411	0.064904	-0.16287	-0.16537	-0.03954	-0.45277	-0.5262	0.033032	-0.47012	-0.07895	-0.0916	-0.46591	0.362092	0.104389	-0.18878	0.22035	-0.24223	0.138715
Q13136	PPFIA1	-0.01203	0.124017	0.059805	0.064566	0.251851	0.493071	-0.06851	-0.01117	0.350936	0.229961	-0.36513	-0.30883	0.364194	-0.18026	0.169878	0.676627	0.049727	0.763577	0.228862
Q13137	CALCOCO	-0.95296	0.2698	0.550722	0.383805	0.426951	0.68982	-0.34387	0.027116	0.061017	0.598053	-0.48983	-0.3576	0.295481	0.110951	-0.45822	0.644537	-0.30446	0.13621	0.256953
Q13144	EIF2B5	-0.18069	0.129395	0.227612	0.367234	0.341284	0.157392	0.154096	0.070768	0.560393	-0.14106	-0.18272	-0.01827	0.028197	-0.1096	0.44228	0.703222	-0.39793	-0.30729	-0.22344
Q13148	TARDBP	0.65083	-0.38781	0.056373	-0.07801	0.199927	0.154691	-0.32183	0.590891	-0.36701	0.318139	0.500689	0.007934	-0.73669	0.044012	-0.1947	0.012251	0.306078	0.273204	-0.07304
Q13151	HNRNPA0	0.579544	-0.39187	0.07634	0.187816	-0.92634	0.072092	-0.51453	0.635059	-1.37173	-0.47236	0.4917	-0.44715	-0.49054	0.093063	0.404812	-0.27997	0.432286	0.466884	0.176856
Q13153	PAK1	-0.6431	0.320703	-0.53944	-0.68999	0.335987	0.610217	-0.11939	-0.16637	-0.57418	-0.27513	-0.49649	-0.79029	-0.10928	0.02637	-0.58631	-0.05447	-0.27125	-0.32935	-0.55142
Q13155	AIMP2	-0.24867	0.245522	-0.06782	-0.13363	-0.1151	0.099692	-0.62189	0.190091	-0.3677	0.196389	0.306872	-0.2864	-0.39137	0.075941	0.804372	0.751819	0.015408	-0.21071	-0.12983
Q13158	FADD	0.520299	-0.54751	-0.00137	-1.05167	0.535903	0.6394	-0.05777	-0.15254	0.060532	0.591111	0.025842	0.150673	0.136891	0.470987	-0.7374	0.448367	0.600301	0.040136	0.468133
Q13162	PRDX4	-0.11632	0.053979	0.196629	0.710591	0.536937	0.606177	0.116953	0.158407	0.377821	-0.52317	-0.9572	-0.74381	0.896446	-0.56296	-0.26223	0.37626	0.148777	0.98512	0.125991
Q13164	MAPK7	-0.1959	0.107701	-0.55034	-0.43407	-0.04304	-0.38985	-0.06686	-0.24119	-0.48768	-0.23825	-0.022	0.282333	-0.26976	0.581326	-0.53743	0.090219	-0.13228	0.046278	0.34149
Q13177	PAK2	0.166627	0.63351	0.362266	-0.06232	0.226963	0.567632	-0.15287	0.159512	0.148162	0.540336	-0.41264	-0.03416	0.631627	-0.04175	1.143986	0.056808	-0.11041	0.008567	-0.00077
Q13185	CBX3	1.767115	-1.28378	0.13881	0.284252	1.197108	0.702105	-1.09368	1.720642	-0.02924	0.636635	0.063062	-0.31918	-0.58956	0.230039	0.705698	0.076781	0.731276	0.472296	-0.30632
Q13188	STK3	-0.13817	-1.70869	0.010955	-0.73848	-0.65959	-0.72448	-0.62829	-0.60316	0.040847	-0.24455	-0.27367	-0.17811	0.623614	-0.31157	-0.25505	-0.15851	0.417747	0.063178	0.296518
Q13190	STX5	-0.22668	0.286852	0.25866	0.242313	0.198263	0.073319	0.521931	-0.02324	0.288895	-0.07308	0.036995	0.513312	0.407108	0.207348	0.584033	0.027477	0.113551	0.59609	0.145377
Q13191	CBLB	-0.21076	0.517296	0.304699	-0.60875	0.47299	0.171319	0.160823	-0.36768	0.039687	-0.20578	-0.40533	0.217534	0.229403	-0.08968	0.45347	0.506685	-0.00309	-0.09565	-0.38645
Q13200	PSMD2	-0.16183	0.351021	0.303426	-0.06363	0.012288	0.582299	-0.20088	0.152169	0.18269	0.412984	-0.00395	0.151223	0.269291	0.14238	0.095465	0.132352	0.330635	-0.15404	0.445832
Q13201	MMRN1	-0.97459	0.078526	1.269733	-0.47896	0.65415	-1.41499	1.059006	-0.83577	0.040847	0.53775	-1.18988	-0.6453	-1.59705	-0.38549	-1.00498	-0.77557	-0.02568	0.997609	0.238715
Q13206	DDX10	0.577934	-0.11805	-0.43453	0.353504	-0.46816	-0.85574	-0.56618	-0.10248	-0.24921	-0.21673	1.109051	0.163545	0.289746	-0.23701	-0.19881	-0.00831	-0.01448	-0.23513	-0.29217
Q13217	DNAJC3	-0.35751	0.269592	0.087423	-0.76449	0.576445	0.349684	-0.46574	-0.1112	-0.60901	0.379965	-0.11712	0.993235	0.161249	0.710188	-0.26572	-0.22156	0.713947	0.467232	0.014705
Q13228	SELENBP	-0.5241	-1.11405	-0.80387	-0.80204	1.123792	-1.22571	0.524277	-0.34664	1.832289	-0.45894	-1.03712	-0.72001	-1.46645	-0.07976	2.036273	-1.29627	-0.94635	-0.7699	-1.27054
Q13232	NME3	0.83761	-0.64755	-0.02323	-0.09143	-0.47556	-0.59355	-0.1531	-0.73325	-0.31651	-0.23871	1.151108	-0.45671	0.328277	-0.40091	1.25927	-1.45628	-1.06018	-0.47498	-0.37054
Q13242	SRSF9	0.420744	-0.64715	-0.29803	0.60536	-0.28992	0.106387	-1.37126	0.796123	-0.77541	-0.69107	0.655997	-0.59062	-0.28761	-0.60549	-0.54627	-0.13058	0.352702	0.624782	0.047041
Q13243	SRSF5	1.005199	-0.83678	-0.28494	0.145569	0.229277	0.049216	-1.24854	0.555286	-0.39535	0.141007	0.842218	-1.23957	-0.44617	-0.74176	0.513462	-0.3363	0.224133	0.736908	-0.07903
Q13247	SRSF6	0.897105	-0.91179	0.100579	0.623996	0.14203	0.30959	-1.05403	0.879659	-0.40819	-0.05823	0.618216	-0.64236	-0.04608	-0.73505	0.65137	-0.56182	0.256753	0.686028	-0.0053
Q13257	MAD2L1	0.33973	-0.32922	-0.29211	-0.06471	0.353419	-0.01659	0.301157	0.721317	-0.40671	-0.28288	0.588418	-0.95299	-0.71899	-0.52632	-0.31429	-0.88967	0.291727	-0.13388	0.456683
Q13263	TRIM28	0.773612	-0.43635	-0.75771	0.204989	-0.30383	0.127708	0.085027	1.055913	-0.20131	-0.42729	0.317389	-0.58578	-0.65211	-0.52213	0.812553	-0.61915	-0.03248	-0.11063	-0.12325
Q13277	STX3	-0.10774	0.163428	0.242869	0.373273	-0.37715	-0.47251	0.118026	-0.01472	-0.3511	0.257721	-0.61581	0.370969	0.68889	0.932346	0.200507	-0.68677	0.704923	0.823475	-0.59275
Q13283	G3BP1	-0.20405	0.131058	-0.11966	0.487588	0.430668	0.445389	-0.14276	0.347529	-0.68617	-0.21259	-0.09816	-0.40227	0.207315	-0.14079	0.399091	0.902262	0.250975	0.007863	0.085151
Q13287	NMI	0.361744	0.323278	0.70613	0.221878	0.897856	0.076559	-0.15375	0.184707	-0.20743	0.842753	-0.86724	0.608413	0.599091	0.907212	-0.91035	0.446041	0.365107	0.160534	-0.9172
Q13303	KCNAB2	-0.54284	0.270196	0.750748	-0.91316	0.110673	0.03266	0.024969	-0.44545	-0.08112	0.926253	-0.83851	1.586777	-0.10572	2.31802	-0.49034	-0.24245	0.245298	0.19306	-1.02447
Q13308	PTK7	0.27536	-0.08042	-0.32622	-0.3654	-0.88788	-0.63522	-0.07673	0.25314	0.323279	-0.39296	-0.41906	-1.23792	0.240258	-0.80415	-0.77058	0.615825	-0.76723	0.245299	1.180045
Q13310	PABPC4	0.293147	-0.47934	-0.01787	0.358488	-0.24967	0.07035	-0.82024	0.638676	-0.27941	0.112649	-0.07818	-0.08099	-0.25302	-0.30508	0.366862	0.209806	0.425213	-0.50579	-0.04982
Q13315	ATM	0.437581	0.734099	-0.04179	-0.2852	-0.2795	-0.65544	0.678884	-0.06733	-0.95261	-0.4623	0.140082	0.397458	0.159451	0.307829	-0.14566	0.149178	-0.24364	-0.11151	0.145926
Q13322	GRB10	0.313043	0.527837	0.0087	-1.47155	-0.53707	-0.93488	0.819416	0.45904	0.27507	0.279758	-0.2586	-0.45869	-0.26516	-0.29327	-0.01554	-0.09438	-0.29998	-0.19164	0.095305
Q13325	IFIT5	0.93348	0.459112	1.159858	-0.80103	-0.60129	-0.09123	0.146512	0.674229	-0.08713	-0.24665	-0.6813	0.278799	0.111448	0.61837	-0.30577	-0.87075	0.187634	-0.35693	-0.75165
Q13330	MTA1	1.049175	-0.30764	-0.63882	0.45569	0.156463	0.57083	-0.39508	1.096997	0.201624	-0.32099	0.620045	-0.52855	-0.10069	-0.3135	0.36244	0.146792	-0.06079	-0.02837	0.371205
Q13332	PTPRS	-0.39904	-0.82598	-0.81287	-0.51036	0.582408	0.378308	0.115014	-0.05804	0.592854	0.10747	-0.52865	-0.35598	-0.04155	-0.60426	-0.12233	-0.88903	-0.43965	-0.52983	-0.64345
Q13347	EIF3I	-0.31582	0.030435	0.396764	0.043597	-0.02751	0.330505	0.374171	0.141495	-0.13058	0.209852	0.214405	-0.38952	-0.30555	-0.32417	0.293698	0.468719	-0.16364	-0.27894	-0.06521
Q13356	PPIL2	0.279109	0.37547	-0.20958	-0.14025	-0.21788	-0.12071	-0.00677	-0.26101	-0.14025	0.435992	0.395548	-0.45246	-0.19395	-0.0768	-0.0127	0.002514	-0.02289	0.406722	0.051532
Q13362	PPP2R5C	-0.03857	-0.09432	0.168229	0.104532	0.426378	0.668051	0.037431	0.162667	-0.12215	-0.2441	-0.30874	-0.82168	-0.11403	0.283741	0.418998	-0.00705	-0.1063	0.148566	-0.12426
Q13363	CTBP1	1.739925	-0.62912	-0.00223	-0.46232	-0.04372	-0.25863	-0.82979	0.35096	-0.19807	-0.18529	0.458042	-0.32652	-0.23943	-0.09468	0.292767	0.12041	-0.54643	-0.0871	0.175778
Q13367	AP3B2	-0.25444	0.319058	-0.12411	-0.49634	0.037155	-0.12487	0.233208	-0.32493	0.379761	-0.31937	0.320179	0.382273	-0.14775	0.256358	-0.29069	-0.11494	0.417999	-0.48983	0.526818
Q13371	PDCL	0.359589	0.166613	0.299889	0.039464	0.136344	-0.01739	0.205608	-0.27041	0.013113	0.706823	-0.08795	-0.11406	0.164582	-0.09705	-0.27925	0.380641	0.862325	0.255938	0.868415
Q13393	PLD1	-0.23095	0.510255	0.100199	-0.25442	0.366586	-0.32434	0.305673	-0.62664	-0.12784	-0.25876	-0.16703	0.263111	-0.22787	1.016077	-0.38191	-0.77646	-0.04185	0.059546	-0.05878
Q13395	TARBP1	-0.39781	0.222432	0.262339	0.058795	0.327686	0.572723	0.203132	-0.35615	0.280566	0.201087	-0.41965	0.117446	0.117711	0.242593	0.662702	0.1382	0.090184	-0.46866	-0.70672
Q13404	UBE2V1	0.12467	-1.0307	-0.1252	-0.15968	1.671304	-0.15547	-0.37348	-0.70648	1.098016	1.833064	-0.6412	-0.23281	0.60252	-0.15494	-0.13785	-0.40085	0.462951	0.070519	-0.36157
Q13405	MRPL49	-0.09881	-0.47103	0.3092	0.647363	0.715428	0.653557	-0.32715	-0.4173	0.452874	0.547573	0.366903	-0.14712	0.273985	-0.27606	0.585124	0.048647	-0.16783	0.043719	-1.06619
Q13409	DYNC1I2	0.185707	0.333772	-0.13407	-0.05138	-0.21798	0.171818	0.040592	-0.22398	0.15305	-0.05404	0.155979	-0.17791	0.215915	-0.21904	0.142532	0.736793	-0.27568	-0.06046	0.617087
Q13416	ORC2	0.780551	-0.54851	-0.48743	-0.45087	-1.13654	-0.27453	-0.38616	0.338247	0.037827	-0.42469	0.260816	-1.23762	-0.22145	-0.54924	0.307858	-0.30293	-0.24342	-0.43733	-0.10502
Q13418	ILK	-0.78102	0.126387	-0.26257	-0.82449	0.067445	-0.07884	-0.22716	-0.40797	0.519734	-0.20235	-0.60886	0.093478	-0.14067	0.168577	-0.15197	-0.31616	-0.22012	0.305025	0.183075
Q13421	MSLN	1.561444	-1.46458	-0.90831	-0.10908	-0.99236	-0.52763	-0.66976	1.118799	-0.58044	-1.43522	0.467782	-0.19256	0.799065	-0.71867	0.694076	0.681308	-1.24348	-2.88664	-1.98777
Q13422	IKZF1	-0.51133	0.453514	0.330093	-0.77193	0.236944	0.056278	0.577753	-2.05807	0.040847	0.313561	-0.00854	-0.46437	-0.16697	0.866154	-0.42198	-0.8308	-0.01524	0.068188	-0.5233
Q13423	NNT	0.307081	0.115171	0.275563	0.367906	-0.14437	-0.09401	0.026974	-0.7148	-0.40706	0.164758	0.017705	-0.01984	-0.69642	0.10198	2.077068	0.564277	-0.99044	-0.62516	-0.16086
Q13424	SNTA1	0.514838	0.002702	-0.69957	-0.67317	-0.72123	-0.18917	1.211749	-0.15586	0.07968	0.83612	0.468641	-1.73196	0.144395	-0.88167	0.089129	-0.01154	-0.49146	-0.38588	-0.02329
Q13425	SNTB2	-0.2604	-0.14915	0.201257	-0.46173	-0.08419	-0.45644	0.378436	-1.17887	0.321906	-0.8804	-0.42354	-0.68395	-0.08675	-0.49322	0.913245	-0.12569	-0.34419	0.255231	0.073075
Q13426	XRCC4	-0.42607	0.264223	-0.23086	0.164632	0.176084	0.851648	-0.27245	1.03975	-0.3387	0.109055	0.202112	-0.50092	0.108969	-0.11805	0.606233	0.086483	-0.26632	-0.01506	0.165947
Q13427	PPIG	1.173961	-1.05518	0.119852	-0.0416	-0.32567	-0.22068	0.327087	0.373677	0.018734	0.338421	-0.01098	-0.07983	-0.7579	-0.84779	0.377953	0.100093	-0.01122	0.325733	-0.38959
Q13428	TCOF1	-0.30046	-0.9556	-0.28529	0.554957	-0.80541	-0.06969	-0.48712	-0.41056	-0.33658	-0.21492	0.441451	-1.32762	0.141155	-1.22294	1.040724	0.130581	-0.8978	0.470131	0.110191
Q13435	SF3B2	0.372797	-0.18914	-0.14376	0.58374	-0.2617	0.099137	-0.40513	0.595427	-0.53986	-0.21447	0.291002	-0.80027	-0.02926	-0.79348	0.587827	0.047205	-0.43252	0.317239	-0.24045
Q13438	OS9	-0.32329	0.316925	-0.24154	0.420877	0.306508	0.418634	0.567259	0.111989	0.059796	0.215221	-0.25434	0.283913	0.355854	-0.18671	0.960693	-0.49211	-0.09982	-0.00768	0.493492
Q13439	GOLGA4	0.324231	0.15106	0.29895	-0.13649	0.11667	0.085218	0.051456	-0.52138	-0.12175	-0.18854	0.354438	-0.3889	0.450887	-0.40977	0.209073	0.620067	-0.37847	0.194796	0.129311
Q13442	PDAP1	0.160623	-0.11146	-0.40286	-0.58558	-1.77935	0.112611	-1.12279	-0.51256	-0.25139	-0.14854	0.044175	-1.12414	0.343811	-0.73857	0.025972	1.244589	-1.24464	-0.85549	0.124271
Q13443	ADAM9	0.718542	0.283654	0.580358	-0.10726	0.140258	0.278055	0.049568	-0.32027	0.351912	0.033728	-0.98415	0.129572	0.248707	0.210939	0.619417	-0.62336	0.9859	0.512436	-0.17469
Q13444	ADAM15	0.246598	0.398321	0.546095	-0.43162	-0.40592	0.227447	0.495642	-0.00727	-0.10163	-0.02353	-1.17157	0.509895	0.281611	0.21511	-0.44049	-0.92232	0.175994	-0.55189	-0.62745
Q13445	TMED1	-0.91887	0.934979	0.191256	-0.57581	-0.28181	-0.55801	0.586566	0.841906	-0.25972	-0.05717	-0.00968	-0.19002	-0.06854	-0.60109	-0.51677	0.30719	0.208883	0.443798	0.530251
Q13449	LSAMP	-1.76963	1.338315	-0.25091	-1.85187	-0.70081	-1.39473	0.122877	0.720977	0.106127	0.378695	-1.42361	-1.60398	-1.63329	-1.59481	-0.02376	-1.11025	-1.39329	1.114971	1.071122
Q13451	FKBP5	-0.86216	-0.6509	0.922465	-0.72721	2.070003	1.317529	-0.32639	-0.77869	0.375592	2.503552	0.114108	0.81875	0.280418	1.257089	-1.26037	0.618538	0.234266	0.855917	0.282701
Q13454	TUSC3	1.444473	-0.18198	0.246629	-1.0119	-1.00444	-1.18446	-0.26878	-0.79647	0.109875	-0.94078	0.981477	-0.80502	0.080455	-0.49314	-1.85802	-0.64607	-0.19214	0.302352	0.639649
Q13459	MYO9B	-1.03438	1.194325	0.597362	-0.2785	-0.25215	-0.38456	0.529728	0.872933	0.182366	0.520218	-0.74089	0.710211	0.120887	0.982934	-0.78445	-0.05867	0.121954	0.003748	0.576606
Q13464	ROCK1	-0.89648	0.41068	0.299206	-0.71638	0.113378	0.108792	-0.13127	-0.14181	0.090878	0.476257	-0.30937	-0.63247	-0.10364	0.362525	0.168909	0.121755	-0.50167	-0.37399	-0.13072
Q13469	NFATC2	0.045939	0.183205	0.355253	-0.57187	0.141892	-0.17683	0.482264	-0.41721	0.286529	-0.04584	0.136116	0.014388	-0.0239	0.00312	0.006594	0.133903	-0.23037	0.874167	-0.02781
Q13480	GAB1	-0.02142	0.495901	0.101613	-0.47791	-0.80743	-0.30857	0.413487	0.021519	0.258689	-0.83879	-0.93687	-0.04344	0.259589	-0.97269	-0.54934	-0.35557	-0.0027	0.258474	0.806457
Q13485	SMAD4	0.103142	-0.86187	0.653414	0.007257	-0.46829	-0.12476	-0.20311	-0.28361	0.28445	0.020999	0.291554	-0.44038	0.056325	-0.08154	0.382569	0.485497	-0.48235	-0.37849	0.672671
Q13488	TCIRG1	-0.35729	1.447432	0.908937	-0.69871	-0.2671	-0.51088	0.507669	-0.64906	-1.02061	-0.1254	-1.01626	1.353106	-0.13021	1.364839	-1.18507	1.006777	0.34064	-0.38898	-0.93832
Q13490	BIRC2	0.23833	-0.17372	-0.14979	-0.62417	0.531146	0.020665	-0.28036	0.511782	0.063358	-0.60827	0.007463	-0.64654	-0.21739	-1.01366	-0.34285	0.011919	0.283888	-0.01957	0.138209
Q13492	PICALM	-0.60543	0.850906	0.861129	-0.04482	0.010954	-0.24801	-0.01405	-0.57162	-0.14777	0.239559	-0.21927	0.04889	0.26247	0.175416	0.348667	0.961738	-0.31577	0.446116	0.162397
Q13496	MTM1	0.017714	0.055171	-0.31191	-0.41168	0.939572	0.611821	-0.25893	-0.34029	-0.23502	0.105375	0.974329	-0.29186	-0.65854	-0.38639	-0.11523	-0.36842	0.007321	0.17042	-0.50255
Q13501	SQSTM1	-0.09477	-0.21207	0.43814	1.005365	0.981637	0.89925	-0.50096	-0.5584	-0.49394	-0.21735	-0.80282	-0.33971	-1.03712	-0.16308	0.647862	0.12686	-0.26493	-0.22074	-1.0627
Q13503	MED21	0.003611	0.041067	-0.45786	-0.21914	-0.36481	0.187268	-0.18675	0.352838	0.015656	-0.00831	0.831117	0.102698	0.068282	-0.24094	0.529606	0.333797	-0.02876	1.220518	-0.0095
Q13505	MTX1	0.324036	0.19556	0.255656	0.694276	0.210735	0.219728	0.004803	0.414918	0.2112	-0.59737	0.210137	-0.18091	-0.06242	-0.05411	1.110998	1.432659	-0.19489	-0.31199	-0.14363
Q13506	NAB1	0.403701	-0.29188	0.066558	-0.34995	-0.32029	-0.40725	0.030675	-0.1435	-0.23368	-0.09635	0.397615	-0.27151	-0.7504	-0.36973	0.055022	0.612885	-0.23686	-0.57253	-1.06951
Q13509	TUBB3	-0.86223	0.212746	0.093865	-1.28907	-0.59185	-0.96824	0.01078	0.676068	0.465449	-0.21352	-1.73427	0.005752	0.210888	-0.25288	-0.82391	-0.15871	-0.30952	-0.47954	1.940949
Q13510	ASAH1	-0.97908	1.680544	0.269886	-0.67268	0.232043	-0.69873	0.301824	-0.99947	-0.62939	-0.92924	-0.57497	0.639475	-0.07553	0.604991	-0.0578	-1.26048	-0.10514	-0.16696	-1.55022
Q13523	PRPF4B	0.825962	-0.03728	-0.49	0.918844	0.024393	0.238803	-0.74791	-0.09328	-0.52279	-0.02187	0.667012	-0.42061	-0.05628	-0.6963	0.090155	-0.15471	-0.4356	-0.00912	0.134962
Q13526	PIN1	-0.06656	-1.3844	-0.52137	-0.67255	0.020121	-0.6563	-0.78203	0.07932	0.402522	0.606807	-0.72878	0.120713	-0.85664	-0.39483	-0.43918	0.527855	0.229488	-0.94624	-0.38258
Q13530	SERINC3	-0.25902	0.348728	0.214166	0.038391	0.286991	0.265303	0.321655	-0.10492	0.566734	-0.03101	-0.0311	0.35356	0.682889	0.115277	0.514022	-0.59827	0.502688	0.266229	-0.12737

Q13535	ATR	-0.25596	-0.03384	0.127858	0.407829	0.286048	0.629487	-0.07162	0.55157	0.019655	0.467051	-0.08012	-0.274	0.170839	0.18916	-0.04752	0.301116	-0.25978	-0.9145	0.085734
Q13546	RIPK1	0.126242	0.179879	0.572891	0.181016	0.302179	0.364086	-0.23226	0.24696	0.23337	0.297444	-0.15983	0.501951	0.716246	0.623706	0.032978	0.30372	-0.07986	-0.2281	0.379287
Q13547	HDAC1	0.793891	-0.51737	-0.00411	0.307653	0.047433	1.166952	-0.69481	0.749552	-0.44827	-0.26502	0.314572	0.092024	-0.52283	-0.33674	0.455276	0.185085	-0.03705	0.053244	-0.48782
Q13555	CAMK2G	0.044309	0.16894	0.28939	-0.41369	-0.19389	0.323878	0.358415	-0.2777	0.448071	0.330855	-0.4894	0.407481	0.089961	0.976816	-0.4276	-0.65745	-0.0452	0.320269	-0.81657
Q13557	CAMK2D	-0.8514	0.199833	0.097515	-1.38851	-0.05002	-0.5535	0.190722	-0.14862	0.066876	-0.53403	-0.8495	0.049438	-0.69348	-0.00459	-0.70173	-0.3732	0.988627	1.113412	0.198591
Q13561	DCTN2	0.180021	0.070711	0.209223	-0.39234	-0.0314	0.223702	-0.32269	0.049903	0.023324	-0.10247	-0.5844	-0.66987	0.062589	-0.20151	0.024034	-0.32497	-0.57612	-0.30815	0.078286
Q13563	PKD2	-0.33477	1.044482	0.200638	-0.44346	0.431692	-0.12513	0.483209	-0.32248	0.288097	0.045311	-0.46147	-0.12061	0.448875	-0.5896	-0.94616	-0.05313	0.032832	1.142296	1.250662
Q13564	NAE1	-0.10213	-0.45573	-0.37044	0.406092	-0.41404	-0.01482	0.148271	-0.00547	-0.50681	-0.478	-0.21063	-0.84091	-0.3151	-0.39862	0.337772	0.249847	-0.11324	-0.42945	0.076362
Q13568	IRF5	0.019037	-0.28894	-0.15562	-0.27723	0.513587	0.638012	-0.52347	-0.17723	0.0862	1.013898	-0.3989	0.686185	-0.24075	0.446954	-0.36676	-0.30216	0.607562	0.086794	-1.34903
Q13572	ITPK1	-0.62774	-0.44289	0.221372	-0.34074	-0.1635	0.017728	-0.45322	-0.0304	0.295895	0.106701	0.810272	0.416921	0.12455	0.754985	-0.55247	-0.03967	1.945732	0.98498	-0.75721
Q13573	SNW1	0.456974	0.418415	0.062086	0.139779	-0.24852	0.22893	0.109257	0.314183	-0.30378	-0.3792	0.459087	-0.51876	0.242528	-0.56974	0.327226	-0.06299	-0.50064	0.459853	0.122101
Q13574	DGKZ	-0.64464	0.557461	0.349374	-0.16378	0.197266	-0.20548	0.018946	0.146054	-0.11005	0.119484	-0.02393	-0.09169	0.032331	0.446816	-0.20915	-0.15089	0.160232	-0.06055	-0.24387
Q13576	IQGAP2	-1.88615	1.154204	0.351432	-1.29303	0.348365	-0.73184	0.643231	1.162303	-0.57051	0.459973	-0.67786	0.7355	-0.53178	1.376282	-0.26748	-1.01572	-0.01699	-0.3234	-0.2645
Q13586	STIM1	-1.06076	0.777768	0.401605	-0.53959	0.20889	0.334398	0.168223	-0.81264	0.066478	-0.08383	-0.62368	-0.07956	-0.06122	0.219663	0.521253	0.420352	-0.02052	0.316092	-0.33774
Q13595	TRA2A	0.614869	-0.67941	-0.00478	0.075746	-0.2544	0.117684	-0.25935	0.659437	-0.26048	-0.36327	0.538829	-0.18681	0.806792	-0.49304	-0.50329	0.327679	-0.03968	0.511885	-0.59546
Q13596	SNX1	-0.27966	0.673621	0.277868	-0.2948	-0.27549	-0.09123	-0.09992	-0.4831	-0.05231	0.252902	-0.35413	-0.11463	0.288542	0.061614	-0.25335	-0.17158	-0.00908	0.29194	0.29577
Q13601	KRR1	0.313521	-1.00148	-0.80461	0.529637	-0.40636	0.245718	-0.663	0.615443	-0.17968	0.279836	0.511668	-0.28684	-0.33644	-0.40403	0.276478	-0.22198	0.205579	0.240253	-0.47908
Q13608	PEX6	-0.00336	-0.12811	0.083227	0.596371	0.06162	0.261451	-0.46932	-0.78109	0.019477	0.444781	0.342878	-0.01636	-0.1223	0.115755	0.276415	0.452624	-0.29836	-0.72257	-1.61146
Q13610	PWP1	0.46201	0.333904	0.464195	0.873042	0.553417	0.820235	0.28548	0.462433	-0.21322	0.000617	0.283314	-0.77393	0.285213	-0.73915	-0.27788	-0.05971	0.072677	0.449115	0.734837
Q13613	MTMR1	0.119967	0.078395	0.847215	-0.03554	0.076714	0.347497	-0.24879	-0.07128	-0.52189	0.099908	0.257963	-0.47885	-0.63197	-0.16678	-0.00434	-0.23568	0.084188	0.3818	-0.21873
Q13614	MTMR2	-0.15908	0.364897	0.556224	-0.2666	-0.30348	0.063303	0.574038	0.440648	0.188112	-0.59618	-0.46611	-0.50364	-0.00803	-0.14336	0.111579	-0.16896	0.453615	0.37949	0.450072
Q13615	MTMR3	-0.55264	0.206899	0.059773	-0.30756	-0.08139	-0.27471	-0.44143	-0.33008	0.076905	0.277241	-0.12003	-0.18276	-0.03219	0.681539	-0.11092	-0.71052	0.200086	0.585043	0.047288
Q13616	CUL1	0.041152	-0.07865	-0.00761	0.599092	0.159227	0.406131	-0.08011	0.46051	0.427389	-0.02948	0.178375	-0.74105	-0.16021	-0.48517	0.874132	0.387121	-0.75827	-0.75503	0.112723
Q13617	CUL2	-0.22397	0.384907	0.610367	0.275775	0.118782	0.472477	0.168934	-0.14413	-0.0066	0.183099	-0.3087	-0.38574	-0.06707	-0.31289	0.727778	0.808909	-0.13812	-0.43908	0.012237
Q13618	CUL3	-0.02744	-0.02671	-0.13761	-0.2639	0.142385	0.305466	0.053873	-0.05033	-0.1126	-0.10546	-0.00061	-0.76028	-0.24376	-0.72202	0.302668	-0.0985	-0.95096	-0.66842	0.396254
Q13619	CUL4A	-0.25442	-0.04388	0.03689	-0.41055	0.219294	0.786467	0.219849	0.432695	-0.61319	0.023495	0.193985	-0.72208	-0.14096	-0.56161	0.56288	0.000384	0.030513	-0.19531	0.404814
Q13620	CUL4B	0.752443	-0.0238	0.530462	0.089679	0.363563	0.282686	-0.15124	0.160373	-0.02204	0.111518	-0.02751	-0.77573	-0.48341	-0.76346	0.258091	-0.14466	-0.62488	-0.07488	0.077744
Q13625	TP53BP2	0.764221	-0.41813	-0.23712	-0.85278	-1.13272	-0.95254	0.043462	0.431751	0.464545	-0.02602	-0.6826	-0.82075	-0.16646	-0.22749	1.396045	-0.4075	-0.02071	-0.29056	0.210336
Q13627	DYRK1A	0.895085	0.280401	-0.11765	-0.59296	0.207972	0.592521	0.53165	0.304638	0.11358	-0.21862	0.230559	0.208553	0.509909	0.551179	0.087164	-0.52085	0.715875	-0.03922	0.184761
Q13630	TSTA3	1.308926	-0.01533	-0.71852	-0.35898	0.829887	-0.00019	-1.64356	0.176223	0.936921	1.232904	-0.40393	0.859295	-0.03841	0.722802	0.216549	0.189803	0.390257	-0.50093	-0.18417
Q13636	RAB31	-1.01025	0.265863	0.617417	-0.62574	0.056305	0.127906	0.108125	-0.35108	-0.28323	0.195238	-1.03454	0.896032	0.426747	1.169143	-0.74708	0.201372	0.396996	0.143712	0.577611
Q13637	RAB32	-0.10657	0.740909	0.455781	0.78816	0.362907	0.171242	0.010422	-0.63744	-0.46222	0.945192	-0.95969	0.833638	-0.39687	1.417573	0.407004	-0.18115	0.052573	-0.50141	0.114929
Q13641	TPBG	0.170885	-0.39972	-0.40886	-0.42606	-1.09997	-0.72872	-0.2083	-0.90045	-0.26327	-0.39139	-0.13123	-0.23357	-0.09996	-0.42678	-0.10001	-0.72815	0.065675	0.301238	1.662923
Q13642	FHL1	-1.23496	-1.21105	-0.94086	-2.41347	-1.69045	-2.72822	-0.65336	-1.14278	-0.21939	-1.91485	-2.62056	-1.07424	-1.47323	-1.04481	-0.57766	-1.86614	-2.77357	-1.18595	-2.39557
Q13643	FHL3	-1.02191	0.641083	0.344821	-0.13677	-0.24855	0.276643	-0.48589	-0.37099	0.681558	-0.02812	-1.25568	-1.68396	0.734314	-0.81101	-0.7702	0.695113	-0.65605	0.608828	1.930591
Q13671	RIN1	-0.1885	0.099622	0.015007	-0.56929	-1.05061	-0.38653	0.092724	-1.88841	-0.02552	-0.39294	0.27191	-0.02532	-0.17867	-0.13233	-0.76159	0.040017	-0.6611	-0.61379	0.481328
Q13683	ITGA7	-0.64687	-1.0679	-1.04629	-1.17976	-1.18703	-1.94648	0.539185	-1.2369	-0.10884	-0.69071	-1.05231	-1.00994	-2.2248	-1.00434	-0.36259	-1.15193	-1.92496	-0.08185	-1.89379
Q13685	AAMP	-0.1044	0.164792	0.243596	-0.3708	0.035672	0.531494	0.153366	0.44362	0.441625	-0.03346	-0.32754	0.205746	0.111315	0.014141	0.253279	0.130638	0.994247	0.54682	0.159546
Q13724	MOGS	-0.27009	0.27764	-0.38546	-0.08805	-0.31301	-0.32234	0.508984	0.240681	-0.03684	0.165981	0.014637	-0.07818	-0.27262	0.100872	0.409398	0.075691	-0.51442	-0.20437	0.40608
Q13740	ALCAM	1.576174	-0.38212	-0.21587	-0.95708	0.519252	0.822849	-0.47406	-2.17728	0.384724	1.331053	1.889799	-1.56898	-1.04672	-1.16271	0.337667	-0.66424	-1.8777	-0.72005	-2.29082
Q13751	LAMB3	-1.4541	-1.74821	-2.25189	-1.11108	-1.86055	-1.40172	-0.34074	0.033132	-0.64004	-0.7966	0.961841	-1.03589	-1.15372	-0.44567	-0.09926	-1.97137	-0.69908	-1.91423	-1.04643
Q13753	LAMC2	-1.75938	-1.41601	-2.98478	-1.31648	-2.41439	-1.46349	-0.58339	0.045144	-0.88641	-0.8826	1.403107	-1.38835	-1.30626	-0.55602	-0.33417	-2.29857	-0.90972	-2.88106	-1.68664
Q13769	THOC5	0.283065	0.078498	-0.36892	0.366787	0.758888	1.053483	-0.52483	0.431899	-0.20652	0.399855	0.74297	-0.17464	-0.19716	-0.12491	0.338542	-0.28401	0.379309	0.42834	-0.13429
Q13795	ARFRP1	-0.69061	0.479578	0.190961	-0.48312	0.838694	1.022897	0.618235	0.045972	0.264236	0.021408	-0.50469	0.037611	0.297443	-0.06064	0.177754	0.408441	0.379314	1.030403	0.062113
Q13796	SHROOM:	0.112516	0.249198	0.291931	-0.30119	-0.2257	-0.64219	0.318988	-0.31822	0.074322	0.029385	0.461791	-0.43372	-0.12278	-0.42764	-0.39116	0.104926	0.576465	1.083079	0.272102
Q13797	ITGA9	-0.09657	-0.3898	-0.71767	-1.16811	-0.3328	-0.726	-0.15786	-1.84257	-0.57848	-0.46058	0.884465	-0.03323	-0.31116	0.085115	-0.32529	-0.20465	0.12937	-0.84117	-1.96099
Q13813	SPTAN1	-0.15741	-0.30213	0.156562	-0.41437	-0.67281	-0.73959	0.338906	0.870688	-0.26221	-0.2917	0.096875	-1.0474	-0.4182	-0.92612	-0.18031	-1.02274	-0.98989	-0.31333	-0.10984
Q13823	GNL2	0.107117	0.135715	0.039324	0.964094	0.144314	0.31627	-0.13958	-0.38155	2.826289	-0.13487	0.198322	-0.24976	0.042136	-0.63402	-0.22608	0.774153	0.019879	-0.09319	-0.48102
Q13825	AUH	-0.21613	-0.22943	0.186212	-0.44646	0.147833	0.206632	-0.60116	-0.69686	-0.37169	-0.34049	-0.29585	-0.30207	-0.87708	-0.12506	1.420562	1.765971	-0.92575	-0.26127	-0.40732
Q13835	PKP1	-0.48203	2.510439	-0.9456	-0.51354	-1.49185	-1.73007	1.075376	0.129518	0.823778	-0.41292	-0.75074	-0.49287	0.341066	-0.41074	0.176045	-0.52228	-1.60576	-0.88467	-0.56799
Q13838	DDX39B	0.708443	-0.17873	-0.44916	0.007484	0.52733	0.456637	-0.89299	-0.59907	-0.63149	-0.03334	0.735668	-0.13268	-0.06011	-0.17651	-0.22636	-0.25948	0.244713	0.301411	-0.03818
Q13867	BLMH	-0.70988	0.477996	-0.29928	-0.58142	0.344997	-0.463	0.823883	0.008668	0.144421	0.518235	-0.56044	-0.87058	0.691095	-0.09405	-0.46936	0.236166	-0.42817	0.268922	0.6927
Q13868	EXOSC2	0.814013	-0.42212	0.124135	-0.04859	0.789687	0.318192	-0.68129	0.285129	-0.64422	-0.11289	0.24226	-0.29317	-0.30641	-0.28121	-0.30958	0.290862	0.074138	0.174258	-0.05955
Q13884	SNTB1	-0.20804	-0.39665	-0.71413	-0.68228	-0.61708	-0.96949	-0.36704	0.493284	0.014802	-0.49147	0.017625	0.649375	-0.0255	0.335431	-0.24884	0.097885	0.556525	-0.1532	-0.84075
Q13885	TUBB2A	-0.78994	0.211242	0.215226	-1.07219	-0.4712	-0.24804	0.581285	1.282848	0.210309	-0.35922	-1.41329	0.148542	-0.05675	-0.13935	-0.67547	-0.29601	-0.23852	-1.01237	1.484613
Q13889	GTF2H3	0.374763	-0.47453	0.000636	0.194129	0.669511	0.169708	-0.33489	0.421634	0.023513	0.130565	0.1798	0.538209	-0.08982	0.33045	0.265896	0.146455	0.422953	-0.12158	-0.4631
Q13895	BYSL	0.096441	0.059664	-0.49054	0.106983	-0.34412	-0.23193	0.206424	0.252639	0.490263	0.520621	0.404783	-0.55581	-0.24435	-0.5265	-0.16524	-0.12092	-0.06489	-0.15907	-0.05868
Q13905	RAPGEF1	-0.05313	0.472991	0.135472	-0.51265	0.207948	-0.30105	0.336968	-0.16108	-0.12141	0.219327	-0.02011	0.456953	0.079213	-0.14228	-0.56014	-0.67347	-0.46437	-0.03352	0.015581
Q13938	CAPS	1.809519	-0.82394	-2.40329	-1.12773	-1.88954	-1.77053	-2.51149	-1.61524	-0.74189	-2.5631	3.421644	-1.05256	-1.47345	-1.49499	1.210865	-2.72974	-0.06773	-0.32351	-3.39732
Q13948	CUX1	-0.33773	0.512372	0.112058	0.34138	0.890151	1.064954	1.016558	-0.07601	0.187756	-0.02084	0.133279	-0.21315	0.329255	-0.42949	0.4112	0.293505	0.07997	0.643404	0.258938
Q13951	CBFB	-2.04136	0.676459	0.213221	-0.74631	0.686762	-0.30182	-0.20732	-0.64191	-0.06283	0.77227	0.060608	-0.04367	0.104672	0.507514	-0.42557	0.153061	0.310708	0.58562	-0.25066
Q13952	NFYC	0.822612	-0.117	0.24645	-0.21749	0.636774	0.43938	0.260435	0.807392	-0.14482	-0.12737	0.181987	-0.30707	-0.34981	-0.26488	-0.18582	-0.60497	-0.36516	0.069465	-0.1749
Q13976	PRKG1	-1.42848	-0.62808	-0.24297	-1.46359	-0.61884	-1.27716	-0.10047	-1.54242	0.004643	0.039332	-1.18296	-1.86864	-0.95743	-0.97244	-0.32106	-0.01559	-1.54615	0.766277	1.593201
Q14004	CDK13	0.597338	-0.18903	-0.15903	-0.59035	-0.57096	-0.31468	-0.26186	-0.88236	-0.39864	0.155514	0.452131	-0.36971	0.205113	-0.44525	0.572997	-0.28344	-0.07574	0.640149	-0.18339
Q14005	IL16	-2.35443	1.236074	0.794091	-1.25093	0.544045	-0.29991	-0.01103	-1.72882	-0.12008	1.37691	-1.66558	-0.29477	-0.21744	0.927498	-0.18716	-0.65691	-0.20615	1.048456	-0.94555
Q14008	CKAP5	0.14478	-0.01854	0.424725	-0.16708	0.127223	0.351117	0.135592	0.58274	-0.18966	0.079902	0.205969	-0.55569	-0.14266	-0.51958	-0.0895	0.367437	-0.17316	0.160012	0.157185
Q14011	CIRBP	-0.01811	-0.00338	-0.55836	0.278694	-0.44236	-0.2475	-0.75521	0.43634	-0.55851	-0.90854	1.16451	-1.69677	-0.21671	-1.78148	0.196274	-0.27875	-1.24051	0.562508	-0.00798
Q14012	CAMK1	0.377463	-0.47036	-1.37251	-0.47304	0.441933	0.338991	-0.3633	-0.34709	0.040847	-0.60879	-0.39672	0.855336	-0.67663	0.942199	-0.35807	-0.17307	0.078985	0.090913	-0.51593
Q14019	COTL1	-2.44999	1.80712	0.816958	-0.5437	0.826119	0.459461	0.346339	-0.0179	0.290249	1.94472	-0.7895	0.093128	0.206791	0.513415	-0.52766	0.001201	-0.73622	0.511138	-0.10211
Q14103	HNRNPD	0.602657	-0.12943	-0.3022	-0.09162	0.007101	0.24725	-0.80022	0.478115	-0.04176	-0.0989	0.574095	-0.41751	-0.39149	-0.46532	-0.25282	-0.8452	-0.2402	0.452563	-0.01899
Q14108	SCARB2	0.537932	1.12845	1.276448	-0.96761	-0.29028	-0.74535	0.560428	-0.27235	-0.05123	0.643434	-0.36306	0.73765	0.152672	0.209828	-0.94423	-0.64038	-0.09825	0.237067	0.174343
Q14112	NID2	-1.26343	0.482805	0.100064	-1.83758	-0.01253	-0.87679	0.472895	-1.09122	-0.22711	0.268259	-0.44337	-1.6694	-0.14038	-1.05631	0.587993	-0.1894	0.62279	2.195392	-0.10407
Q14116	IL18	-1.39875	0.81632	-0.84904	-0.17167	0.963178	-0.09371	-0.58653	-0.43969	0.22106	0.404485	-0.60155	1.095529	0.481874	0.667847	-0.75948	-0.78654	1.205302	-0.3256	-1.14456
Q14118	DAG1	0.052247	-0.41554	-0.52175	-0.89666	-0.58131	-0.93858	0.176674	-0.46741	0.209911	-1.02869	-0.26739	-0.85765	-0.30333	-1.43162	0.152266	-0.16835	-0.73755	-0.43875	-0.11236
Q14126	DSG2	0.415714	-0.98406	-1.40297	0.484449	0.133494	-0.15366	-0.04182	0.536276	-0.22589	-0.13856	0.57946	-0.57578	-0.02279	-1.02955	0.625418	0.681535	-0.60547	-1.07146	-0.88494
Q14134	TRIM29	-1.76517	-1.0549	0.505768	-0.96995	-3.40441	-3.01052	-1.43097	0.059191	-0.32338	0.147989	-1.3836	1.065962	3.249654	-1.60921	1.742098	-0.50414	-0.51532	-1.0916	-3.12989
Q14137	BOP1	0.405672	0.063859	-1.09473	0.421904	-0.19027	-0.02594	-0.51407	0.168881	1.443835	-0.22172	0.42055	-0.38073	-0.40229	-0.47371	-0.24896	0.215507	0.324974	0.482331	-0.54715
Q14139	UBE4A	0.104519	0.37124	0.181011	0.046292	-0.27014	-0.12626	0.210852	0.001258	0.029978	-0.22262	0.29501	0.184204	0.657222	-0.1656	0.087764	-0.05141	0.046641	-0.29226	0.127623
Q14141	SEPT6	-0.54598	-0.48816	1.126949	-0.29817	0.705056	0.114284	-0.59339	-0.59398	-0.90427	0.147246	-0.98529	0.021079	-0.32236	0.754815	-0.50218	0.153857	-0.39661	0.090278	-0.51473
Q14142	TRIM14	-0.39686	0.784329	1.372904	-0.70736	0.412924	0.672702	0.495957	-0.27463	-0.1205	0.138677	-0.99438	0.516093	0.681257	0.627116	-0.73496	0.614661	-0.07109	0.57197	-0.46041
Q14145	KEAP1	0.033078	0.199598	-0.03686	-1.10211	-0.32127	-0.67965	0.046794	0.8894	0.293913	0.210489	0.060714	-0.32356	0.239672	-0.58408	-0.84578	0.051947	-0.45362	-0.37854	-0.58927
Q14146	URB2	0.012115	-0.00336	-0.23417	0.61094	-0.0253	-0.00598	-0.04251	0.040461	-0.32889	-0.49649	1.01384	-0.36696	-0.30171	-0.16198	-0.03856	-0.76628	-0.50547	-0.35215	0.165203
Q14147	DHX34	0.830129	-0.18551	-0.10824	0.077623	0.198545	0.143111	-0.14815	-0.33931	-0.32715	-0.34902	0.360704	-0.37213	-0.38078	-0.26206	-0.17272	-0.02598	0.610699	0.195077	-0.63957
Q14149	MORC3	-0.50829	0.174015	0.992519	-0.48471	0.421318	0.856525	-0.17639	-0.07257	0.808443	0.175848	0.223425	0.404763	0.612211	0.300974	0.409456	0.040836	0.361286	0.408979	-0.42891
Q14151	SAFB2	0.220578	-0.02306	-0.38794	-0.24959	-0.50102	0.066097	-0.32231	0.540674	0.080921	-0.30592	0.844415	-1.10492	0.129343	-0.9369	-0.29532	-0.39713	-0.6025	0.124048	-0.78755
Q14152	EIF3A	-0.27856	0.275285	0.183492	-0.05764	-0.18383	0.363966	0.32736	0.173081	-0.14577	0.051517	0.250583	-0.82354	-0.06066	-0.73209	0.714487	0.225197	-0.48851	-0.27341	-0.03296
Q14155	ARHGEF7	-0.26551	0.366979	0.096494	-0.75222	-0.02131	-0.08262	-0.06569	0.028476	-0.00166	-0.03476	-0.31689	0.050446	0.156375	0.015523	-0.11576	-0.43709	0.18257	-0.03011	0.188669
Q14156	EFR3A	0.161204	0.46919	0.118636	-0.23843	0.29609	-0.12801	0.433848	-1.0601	-0.46595	0.12385	-0.33177	0.097651	0.229087	-0.06984	0.380456	0.064675	0.171213	0.458134	-0.39097
Q14157	UBAP2L	0.292976	-0.12919	-0.18391	-0.13622	-1.20008	-0.12976	-0.54295	0.242178	0.121514	0.00747	-0.13026	-1.13003	0.458595	-0.57272	0.850133	0.412845	-0.26679	0.040933	0.458943
Q14160	SCRIB	1.305009	-0.37242	-0.76951	-0.29095	-0.18328	-0.42574	-0.4215	0.166113	-0.30779	0.015173	0.007717	-0.05633	0.46256	-0.37076	-0.14449	0.201253	-0.51104	-0.53827	-0.9216
Q14161	GIT2	-0.81377	0.802007	0.694684	-0.72007	-0.00625	0.019944	0.518073	-0.55244	-1.77792	0.421294	-0.47207	0.433491	-0.12971	0.785157	-0.09527	-0.32778	-0.05874	0.295349	0.152686
Q14164	IKBKE	-0.19499	0.214911	-0.0145	-0.92197	0.028993	-0.73447	-0.04448	-1.04978	0.428858	0.678201	-0.46997	0.329575	0.042317	0.542839	-0.30045	-0.72776	0.035004	-0.68592	-0.09882
Q14165	MLEC	-0.26943	0.39463	0.140483	0.635453	-0.09451	-0.36959	0.849471	0.081835	-0.52078	-0.27952	0.457347	0.122288	-0.13163	0.088506	0.448575	-0.28192	0.380003	0.226819	0.497482
Q14166	TTLL12	-0.1898	-0.21126	-0.0992	-0.22177	0.519596	0.111706	-0.44939	-0.35243	0.088181	0.254128	0.005854	0.365099	0.403374	0.59852	-0.41123	1.442013	0.603572	-0.04987	-0.49586
Q14181	POLA2	1.164649	-0.08549	-0.15663	0.120562	1.233019	1.353979	-0.23202	0.601438	-0.40294	-0.29323	0.04839	-0.12844	-0.20624	0.0328	-1.33289	-0.26595	0.388158	-0.13995	-0.10996
Q14185	DOCK1	-0.1431	0.023119	0.043905	-0.33986	-0.58188	-0.35689	0.526504	-0.33006	0.134588	0.042164	0.678514	-0.62224	-0.15412	-0.8737	-0.15978	-0.29007	-0.56658	0.104456	0.477064
Q14191	WRN	0.978025	-0.15457	-0.0493	0.165323	-0.12769	0.4586	-0.20747	-0.29928	-0.05297	-0.59395	0.368284	-0.65369	-0.21178	-0.41961	0.175704	-0.19478	-0.13553	0.122877	-0.2345
Q14192	FHL2	0.873728	-0.56092	-0.33726	-0.42757	-0.7776	-0.16358	0.126397	0.103185	0.155252	-0.96595	-0.4294	-1.65895	0.071939	-1.30227	-0.26476	0.588063	-1.99472	-1.56105	0.471216
Q14194	CRMP1	2.419229	-0.62791	-0.162	-0.94352	0.297892	-0.65383	0.028753	0.716009	0.254408	-1.27612	0.823118	-0.00117	0.659823	-0.55612	-0.74882	-1.00959	-0.64338	-0.89865	2.057492
Q14195	DPYSL3	-0.90286	-0.76729	0.173752	-1.37714	-0.18301	-0.49106	-0.79985	-0.22547	0.902179	-0.83443	-0.24786	-0.04155	1.743776	-0.8191	-0.91052	0.00909	-0.94571	-0.41785	1.957466
Q14197	MRPL58	-0.3189	0.018543	0.104306	0.67808	0.476313	0.2886	-0.18074	-0.24476	0.214796	0.392981	0.583189	0.119928	0.103687	-0.5618	0.600755	-0.36476	0.018207	-0.18609	-0.68596
Q14202	ZMYM3	0.506465	-0.19011	-1.85121	0.557499	0.211615	0.739008	0.461793	1.361687	0.073587	-0.61358	0.357379	-0.79204	-0.89078	-0.52303	-0.3399	-0.46882	-0.53213	-0.08815	0.005382
Q14203	DCTN1	-0.08459	0.47791	0.105629	-0.32699	-0.25017	0.003074	0.303325	-0.07308	-0.08499	-0.06073	-0.62709	-0.50862	0.02759	-0.1277	-0.16004	-0.2737	-0.64021	-0.44369	0.316459
Q14204	DYNC1H1	0.201131	0.365647	-0.12002	-0.02601	-0.13563	0.18543	0.307278	-0.31551	-0.07839	-0.22784	-0.07524	0.125618	-0.02042	0.166329	0.081769	0.554065	-0.03082	-0.41654	0.50653
Q14232	EIF2B1	0.45697	-0.0131	0.05867	-0.0583	0.658853	0.328879	-0.32739	-0.10515	0.255319	-0.09428	0.068136	-0.21016	-0.00043	-0.18344	0.033645	-0.33493	-0.07037	-0.08745	0.197468
Q14240	EIF4A2	0.298358	-0.44909	-0.72276	0.096061	-1.20694	-1.58867	-0.58272	0.449988	0.062046	0.30061	0.582231	-1.00305	-0.0945	-0.27505	0.853152	-0.06717	-0.00155	-0.15545	-0.19021
Q14241	ELOA	0.739872	-0.05383	-0.58548	-0.35023	-1.04421	-0.13416	-0.24105	0.574971	-0.02445	0.481965	0.113107	-0.41433	0.157095	-0.5282	-0.08537	-0.38435	0.328151	0.868367	-0.28783
Q14244	MAP7	0.818095	-0.67222	-0.23138	0.922051	-1.04007	-0.4363	-1.02024	-0.92397	0.248302	-0.24292	0.669191	-0.97008	0.535949	-1.14556	1.106826	0.546439	-0.24239	0.135196	-0.66501
Q14247	CTTN	-0.10657	-0.4555	0.063758	-0.06591	-0.85917	-0.0049	-1.12695	0.074835	-0.54674	-0.614	0.29885	-1.20865	1.125328	-1.4378	-0.05717	0.788584	-0.27001	0.205409	0.29727
Q14249	ENDOG	0.609919	-0.09635	1.613742	-0.7621	-0.31358	-0.51628	-0.02892	-0.15208	0.040847	0.495179	-0.28634	0.041588	1.71762	-0.40023	-0.96166	-0.35194	0.254994	-0.63731	-0.08341
Q14254	FLOT2	-0.81215	0.176142	-0.3024	0.549406	0.048092	-0.058	-0.28236	-1.00751	-0.52583	0.759596	0.454974	-0.03218	-0.58944	0.774963	-0.35031	-0.08143	0.116314	0.099468	-0.37273
Q14257	RCN2	0.686225	-1.46882	-1.00804	0.501185	-0.01645	0.058069	-1.00517	1.835622	0.116548	-0.18977	0.126354	0.529545	1.79611	0.063402	-1.13325	-0.73199	0.04951	-0.11226	-0.21053
Q14258	TRIM25	0.060696	0.419124	0.739698	-0.65554	0.389381	0.653536	-0.30015	0.120266	-0.23639	0.097617	0.330446	0.345816	0.378065	0.250116	-0.15771	0.345628	-0.15415	-0.16414	-0.56617
Q14289	PTK2B	-0.81438	0.351932	0.797107	-0.76715	0.293267	0.086984	-0.015	-0.83602	-0.96668	1.068078	-0.78058	0.914851	0.412957	1.743412	-0.89569	-1.01228	0.594928	-0.14447	-0.7114
Q14314	FGL2	-1.54063	0.271471	0.571544	-1.51478	0.283459	0.066787	-1.09566	-1.75879	0.127609	0.692409	-0.78358	-0.84623	-0.88959	-0.19581	0.023373	-0.84723	-0.44742	0.004445	-2.12278
Q14315	FLNC	-1.7039	0.958159	0.384412	-0.74899	-0.56088	-0.4562	0.122043	0.358025	1.027938	-0.83675	-1.5467	0.082491	1.512908	-0.76215	-1.27294	-0.89616	-0.54604	0.615834	2.497972
Q14318	FKBP8	0.218832	0.033644	-0.21478	0.661384	0.099296	0.222068	0.023332	-0.07986	-0.0612	-0.14517	0.179404	-0.0655	-0.29132	0.354355	-0.21864	0.302687	0.109525	-0.33388	0.091267
Q14320	FAM50A	0.939418	-0.0371	0.586223	0.512454	1.141505	1.202478	-0.34103	0.539471	-0.45798	0.218311	0.041605	-0.55386	0.353451	-0.03355	0.056654	0.569286	1.231377	1.731968	-0.08675
Q14331	FRG1	0.203588	0.253428	0.336185	0.285681	-0.16891	0.402738	0.129435	-0.00603	-0.02888	0.133916	0.125807	-0.09471	0.051986	0.07798	-0.62712	0.294298	0.141358	0.353798	0.03936
Q14344	GNA13	-0.7123	0.542319	-0.15087	-0.27631	0.212303	-0.18468	0.23608	-0.279	-0.33896	-0.04023	-0.40198	0.284056	-0.47941	0.825146	-0.31226	-0.16828	-0.34842	0.208131	-0.47725
Q14376	GALE	0.048566	-0.82396	0.298615	0.038299	0.113926	-0.13401	-0.37006	0.642455	0.972673	1.210376	0.06562	0.886314	0.474606	0.215205	1.037798	-0.69924	0.988426	-0.13197	-0.48129
Q14393	GAS6	-1.65926	-0.81011	-1.98186	-1.67138	-1.65304	-2.10493	-1.81748	-0.50095	-0.23412	-1.83677	-1.66732	-0.90529	-2.28943	-0.48159	0.761416	-2.02306	-0.92908	-2.76833	-1.66242
Q14432	PDE3A	0.692654	-0.62129	-0.21991	-1.12129	-0.66831	-1.77108	-0.37302	-1.1911	0.043986	0.238883	-0.24746	-0.69426	-0.37119	-0.70565	1.98076	-0.25228	-0.64368	-0.0585	-0.23167

Q14435	GALNT3	1.309063	-0.5431	-1.6736	0.786422	-1.14115	-0.70952	0.046536	0.06472	0.334147	0.622047	1.067566	-1.39498	-0.70297	-0.10436	2.510492	0.179664	0.088005	-1.38402	-1.80597
Q14444	CAPRIN1	0.567381	-0.24454	0.711323	0.555986	0.194113	0.633202	-0.8065	0.556878	-0.47979	-0.46322	0.020124	-1.00793	-0.01237	-0.94699	0.82501	0.393501	-0.22018	-0.0921	0.276846
Q14451	GRB7	-0.48108	-0.94796	-0.53451	-0.69306	-0.18804	0.077801	-0.21957	-0.36918	-0.2057	-1.04688	0.019322	-0.88069	-0.75932	0.35564	-0.35367	0.327302	-0.62364	-0.94311	-0.71515
Q14457	BECN1	-0.41909	0.694443	0.541626	-0.28631	0.156557	0.395394	0.102691	-0.73936	0.258617	0.215075	-0.47314	0.339558	0.452757	0.412023	-0.09784	0.480924	0.01998	-0.15553	-0.3333
Q14493	SLBP	2.177556	-0.25509	-0.58078	-0.60752	-0.68351	0.125989	-0.13488	0.325852	0.082864	-0.32508	-0.05881	-1.14915	-0.02801	-0.43601	-0.18298	-0.26142	-0.41938	0.38159	-0.29421
Q14498	RBM39	0.572079	-0.10372	0.05451	0.137878	0.138744	0.471062	-0.4985	0.57561	0.149202	0.099159	0.539927	-0.21509	-0.6217	-0.35622	0.083793	-0.5105	0.201893	0.375484	-0.27568
Q14511	NEDD9	-0.22874	0.221533	0.354649	0.092722	-0.0205	-0.09767	-0.46397	-0.60485	-0.08957	-0.1565	-0.54177	-0.23812	-0.22791	0.880582	0.086226	-0.62513	0.141182	-0.36845	0.418178
Q14515	SPARCL1	-0.61689	0.692103	0.399051	-0.48364	-0.30773	-0.28271	0.347844	-0.71847	0.020102	0.355882	-0.99573	-0.58969	-0.19721	0.155498	-0.18368	-0.21733	-0.77906	1.390341	-0.73857
Q14517	FAT1	-0.00342	0.241594	-0.06763	-0.38676	-0.35598	-0.06789	0.237321	-0.8714	0.118566	-1.08245	0.040624	-1.06463	0.011626	-0.29152	-0.70643	0.107931	-0.17197	1.478634	0.620244
Q14520	HABP2	-0.22111	-0.26556	-0.07167	-0.55932	-0.91491	-1.40629	-0.17405	-0.34982	0.178981	-0.31289	0.851732	1.505864	-0.75392	2.242934	-0.85544	-0.29647	1.642469	0.234593	-0.16005
Q14527	HLTF	0.487428	-0.24191	0.429668	0.257983	0.502236	0.694949	0.40384	1.18692	0.730079	0.852464	-0.27711	-0.74372	0.210193	-0.29879	1.645879	0.853614	-0.82711	-0.5333	-0.22819
Q14534	SQLE	-0.41603	-0.50076	0.407068	-0.52404	1.106406	0.692822	-0.00558	0.094674	-0.08292	0.028712	-0.08949	1.740551	0.601237	-0.15968	0.017084	-0.03249	0.802699	-0.1472	0.047538
Q14554	PDIA5	-0.41784	0.256223	-0.25714	-0.24084	-0.51063	-0.42003	-0.01691	0.318418	0.949509	0.860192	-0.3569	-0.16764	0.582733	-0.26965	0.743051	-0.0284	-0.04585	0.200149	0.305187
Q14558	PRPSAP1	0.62132	-0.24356	-0.04774	0.044463	0.478681	0.376986	-0.70819	-0.0424	-0.53993	0.070755	-0.11447	-0.05646	-0.16923	0.571841	-1.04836	0.316982	0.292704	0.259337	-0.00436
Q14562	DHX8	0.001741	-0.4026	0.589154	0.15171	0.282334	0.411632	-0.09616	0.181781	-0.33152	0.131329	0.411797	-0.28536	-0.05568	-0.25624	-0.08263	-0.14154	-0.01749	0.116722	0.122586
Q14566	MCM6	2.61768	-0.78727	-0.41724	-0.15974	1.609984	1.90661	-0.23029	1.242794	-1.20107	-0.98048	-0.14915	-0.97683	-0.57121	-0.82333	-0.16768	0.639461	-0.10734	-0.61871	-0.55748
Q14571	ITPR2	-0.58489	0.812688	0.416245	-0.84987	0.332972	-0.03927	0.319031	-0.47549	1.236819	0.627447	-0.03863	0.378806	-0.36511	0.47382	-0.05932	-0.54712	-0.47454	-0.10803	0.350101
Q14573	ITPR3	0.089775	-0.34683	0.062446	-0.08547	-0.1036	0.279074	0.547126	0.955817	-0.13046	0.251174	0.233195	-0.37647	-0.37044	-0.38672	0.842503	0.24318	0.613441	-0.79536	-0.39763
Q14574	DSC3	-3.03833	1.454303	0.190514	-0.65465	-1.14792	-0.73903	0.978046	-0.63307	0.836336	-1.58552	-0.68766	-0.29664	-0.47045	-0.89718	-1.65554	-0.77796	-1.18575	-0.94922	0.328851
Q14624	ITIH4	-1.09682	0.671252	-0.80589	-2.28338	-1.36883	-1.96551	-0.48411	-0.7258	0.682716	0.213653	0.605041	0.430396	0.000775	0.733066	-0.37006	-0.73734	0.573195	0.976057	-0.13318
Q14642	INPP5A	-0.66205	0.036368	-0.01065	-0.3061	-0.36876	-0.41138	-0.00903	0.224357	0.413534	-0.07441	-0.1413	-0.84368	-0.18474	-1.10895	-0.12073	-0.47215	0.023178	-0.05599	0.109859
Q14643	ITPR1	-1.42839	0.495847	-0.20481	-1.2614	-0.28013	-0.77261	-0.07807	-1.37174	-0.21421	-0.08058	-0.46593	-0.04119	-1.11218	0.192293	-0.31631	-1.0087	-0.8952	-0.21574	-1.18075
Q14644	RASA3	-0.87248	0.659383	0.198071	-0.69216	0.109222	-0.34242	0.377624	-0.21758	-0.32311	1.001318	-0.67183	0.127081	-0.04727	0.241487	-0.77911	-0.10742	-0.47904	0.817334	0.756382
Q14651	PLS1	0.667945	-1.32502	-0.78566	0.678543	-0.53365	0.317644	-1.69622	-0.56038	-1.38545	-1.16233	1.090032	-1.24835	-0.81087	-0.80696	-1.22541	-0.79912	-0.05874	-1.4051	-0.84085
Q14653	IRF3	0.586005	0.221945	-0.01574	-0.67715	-0.03395	-0.20826	1.139655	0.14939	0.576116	0.277031	-0.08826	0.441073	0.423789	-0.38827	-0.1483	-0.14046	-0.10998	0.194006	-0.83022
Q14667	KIAA0100	-0.44851	0.154877	0.105994	0.297654	0.036747	-0.37197	-0.41288	-0.07644	1.16213	-0.20183	0.7306	0.440178	0.103552	0.216392	-0.34491	0.290236	0.322213	-0.20855	-0.40039
Q14669	TRIP12	-0.25528	0.577148	0.391559	-0.60247	-0.00786	0.626294	0.553573	0.156312	0.011538	0.119027	0.12516	0.006744	0.281681	0.035032	0.238368	0.038828	-0.01576	0.083215	-0.03277
Q14671	PUM1	0.702896	-0.27403	0.190091	-0.31135	-0.0856	0.211718	-0.36146	0.233182	0.015358	0.293704	0.06275	-0.4619	0.056143	-0.10274	0.168458	0.431215	0.147078	-0.02114	0.389959
Q14676	MDC1	0.542959	-0.15917	-0.39865	-0.12363	-0.09148	0.654152	0.323493	-0.62578	-0.91798	-0.5477	-0.20319	-0.58372	-0.09673	-0.4614	-0.59898	0.677058	-0.30623	-0.22906	-0.13418
Q14677	CLINT1	-0.42363	0.407056	0.304057	0.12648	-0.25338	-0.19555	-0.03346	-0.04244	-0.21959	0.267792	0.218592	-0.31478	0.280414	-0.04165	0.651909	0.846383	-0.16247	-0.01547	-0.20577
Q14678	KANK1	-0.61825	-0.33842	-0.16428	-0.80412	0.738639	1.171678	-0.18092	-1.21503	-0.08522	-0.3035	-0.19347	-0.26764	-0.5847	-0.95061	0.506619	0.108083	0.256221	0.544036	-0.22012
Q14683	SMC1A	0.689042	-0.09276	0.0408	0.129662	0.72479	1.02039	-0.17486	-0.03539	-0.38427	-0.06125	0.030801	-0.64071	-0.5574	-0.339	0.441725	-0.17476	-0.29527	0.126491	-0.34773
Q14684	RRP1B	1.037315	-0.10763	-0.58498	-0.3544	-0.18815	0.141535	0.152763	-0.14954	0.5722	0.049196	0.283774	-0.31068	0.140344	-0.32626	0.106885	-0.32448	0.16798	0.388642	-0.40243
Q14687	GSE1	-0.29899	0.303664	0.087053	-0.08164	0.150525	0.138448	0.307845	0.291903	-0.22086	0.192985	0.536226	0.278603	0.041627	-0.1383	-0.29972	0.052232	-0.10124	0.031556	-0.51561
Q14689	DIP2A	-0.05603	-0.45814	0.549883	-0.52567	-0.01227	-0.05439	-0.9192	-0.46482	1.015038	-0.37303	-0.44257	-0.01956	-0.07672	0.014159	0.214008	0.043071	0.064309	-0.06577	0.076979
Q14690	PDCD11	0.103917	0.220821	-0.35296	0.737634	-0.51351	-0.34011	-0.3002	0.226839	0.112173	-0.06801	0.622731	-0.06836	-0.23035	-0.45642	0.218544	-0.31745	-0.11487	-0.3698	-0.21379
Q14692	BMS1	-0.01029	0.172347	-0.43983	0.325781	-0.10953	-0.00089	-0.06955	0.335535	-0.77519	-0.33252	0.503538	-0.10378	0.148088	-0.22992	0.042467	0.16179	-0.13895	-0.03675	0.144638
Q14693	LPIN1	-0.35474	0.22394	0.21801	-0.57943	0.02964	-0.82392	0.37018	-0.27274	0.221066	-0.26778	-0.9149	0.112391	0.087137	-0.15792	0.917316	-0.22704	-0.03825	-0.39264	0.531114
Q14694	USP10	0.027297	0.007213	-0.1827	-0.18076	0.706291	0.771547	-0.73994	-0.02818	-0.05231	0.091119	0.279608	-0.29841	0.187831	-0.0883	0.43428	0.366414	-0.18273	-0.47568	0.286178
Q14696	MESD	0.177518	-0.93534	-0.31242	0.737403	0.772751	-0.39882	-0.2248	-0.37014	0.492128	2.54801	-0.10118	1.174531	0.482968	1.126758	-0.96525	-0.24327	0.935131	0.33482	-0.37441
Q14697	GANAB	0.264817	-1.0175	0.434723	0.148185	0.198953	0.08764	-0.3231	0.882495	0.339474	0.326539	0.340446	0.233874	0.641727	0.075807	0.299163	0.67176	0.296423	0.514211	0.103484
Q14699	RFTN1	-0.63087	0.320145	0.281536	-1.72269	0.306841	-0.07983	0.268652	-1.86627	-0.01986	0.513844	0.118672	-0.47975	-0.28289	-1.10013	0.111275	-0.65774	-1.03062	0.608643	-0.42996
Q14728	MFSD10	0.610456	0.172915	-0.14803	-0.63904	-0.7571	-0.77608	0.319787	-0.12678	0.058159	0.322041	0.201963	0.522415	-0.47551	0.530908	-0.49781	0.314891	-0.44607	-0.91235	-0.18563
Q14738	PPP2R5D	0.172903	-0.1722	-0.21068	0.142046	-0.14609	0.389787	-0.20023	-0.13967	-0.11899	-0.31633	-0.08754	-0.36982	0.434923	0.123429	0.115656	0.053663	0.125429	-0.33943	0.109032
Q14739	LBR	0.33934	0.289186	0.807571	-0.48312	-0.25428	-0.483	-0.25637	0.08067	0.150283	0.850332	-1.35855	0.804818	-0.4227	1.341813	-0.18505	-1.09088	0.576808	1.105069	-0.21815
Q14746	COG2	-0.67824	0.300699	0.170517	-0.10077	0.304611	0.150355	0.394596	-0.04659	0.601344	0.474741	-0.24036	-0.03509	-0.30855	-0.24026	0.845013	-0.12001	0.005699	0.093865	0.439148
Q14761	PTPRCAP	-0.6844	0.413045	-0.05009	-0.58511	0.119528	0.046567	0.179234	-1.28978	-0.54881	0.228464	-2.00696	-1.49458	-0.29313	-1.6331	-1.11051	-0.3698	-0.8996	1.376096	-1.34433
Q14764	MVP	-0.22356	-0.37803	0.169988	0.603695	0.042689	0.281964	-0.96548	-0.76529	-0.0551	-0.11335	-0.37134	0.585364	0.129906	0.658307	-1.18228	1.474776	0.012612	-0.40493	-0.04525
Q14766	LTBP1	-0.55042	-1.32745	-1.40762	-1.38566	-2.12833	-1.69763	-0.45304	-0.55277	-0.50619	-0.79534	-0.68661	-1.97717	-1.66295	-0.69726	0.351294	0.016323	-1.92809	-0.88221	-0.91704
Q14767	LTBP2	-1.18304	-1.72254	-1.08913	-2.09015	-2.64091	-2.16707	-1.0079	-1.31507	0.680953	-1.40004	0.081782	-2.01578	-1.31673	-1.30728	0.662578	-0.04901	-2.29377	-1.12579	-0.8798
Q14781	CBX2	1.045299	-1.04732	-0.49832	-0.31442	-1.505	0.341615	-0.19517	1.188568	-0.34238	-0.60371	-1.44656	-1.25852	-1.24129	-0.94758	-1.33789	-1.38495	-0.76455	-1.99002	0.389706
Q14789	GOLGB1	-0.3907	0.584221	0.537765	0.461085	0.337603	0.377933	0.392276	-0.17404	0.151979	0.484018	-0.16273	-0.59541	0.586753	-0.56035	0.986999	0.474051	0.114084	0.60458	0.565397
Q14790	CASP8	-0.60712	-0.18374	0.489884	-0.35355	-0.19857	-0.40248	0.103013	0.501762	-0.53623	0.269837	0.127548	-0.11236	-0.57158	0.756747	-0.52623	0.087437	0.196943	-0.73752	0.196626
Q147X3	NAA30	-0.2576	0.134627	0.050999	-0.28102	-0.22651	-0.12513	0.252554	-0.0166	0.504367	0.112294	-0.29889	0.111985	0.240421	0.108006	0.1032	-0.15879	0.704364	-0.82785	-0.15307
Q14814	MEF2D	-0.4755	0.234412	0.197927	-0.30655	-0.22269	-0.36833	0.553311	-0.31623	0.301886	0.627891	-0.25211	-0.43731	0.097762	-0.28249	0.841896	-0.0766	0.031903	0.27234	-0.34418
Q14839	CHD4	0.567416	-0.10063	0.06101	0.048008	-0.0003	0.503893	-0.01344	0.900929	-0.22723	-0.13647	-0.01258	-0.48215	-0.28093	-0.42387	0.615036	-0.17848	-0.12352	0.07451	0.219974
Q14847	LASP1	-0.43795	-0.16575	-0.00893	-0.0771	0.018476	-0.46851	-0.62131	-0.9976	0.312563	0.044838	-0.50584	-0.22767	0.321721	0.159082	-0.52609	0.042734	-0.30832	0.053813	-0.17904
Q14849	STARD3	-0.94724	0.950615	0.723892	-0.74131	-0.19023	-0.45336	0.411162	-1.30665	-0.20085	0.73551	0.346166	0.650473	-0.1424	1.036351	-1.06631	-0.43474	-0.23125	-0.23807	-0.2059
Q14865	ARID5B	0.404226	-0.16826	0.192178	0.365487	-0.08327	0.364378	-0.56111	0.270842	-0.00629	-0.15352	0.178892	-0.71946	-0.07955	-0.15106	0.483188	1.124715	-0.85562	-0.07342	0.291581
Q14914	PTGR1	0.236038	-0.33972	-1.37441	-1.16544	1.932642	0.96064	0.087478	1.235837	0.031244	-0.14425	0.17658	-0.38585	-0.20925	-0.28641	-0.86929	0.005195	-0.83766	-1.64732	0.082963
Q14919	DRAP1	0.829234	-0.28428	-0.23909	-0.61447	-0.05312	0.451522	-0.50471	-0.18832	0.026845	0.035777	-0.09644	-0.53156	0.152791	-0.3804	-0.13463	0.334113	-0.24553	-0.12042	0.218925
Q14934	NFATC4	0.274387	0.276117	0.115682	-0.45786	-0.15879	-0.64686	0.075293	-0.01949	0.117557	-0.31353	-0.5185	-0.69105	0.107394	-0.3037	-0.24913	0.365442	-0.17868	-0.38555	0.743518
Q14938	NFIX	-0.1274	-0.63682	-1.11771	-0.53495	-0.18736	-0.88337	-0.26362	0.865173	-0.64396	-0.67987	0.341358	-1.73126	-0.47338	-0.64159	1.826436	-0.58681	-0.87641	-0.25574	-0.04015
Q14956	GPNMB	-3.11212	0.20571	-0.4236	-1.0464	-1.64661	-1.58798	0.088007	-2.06696	0.244181	-0.3838	-1.34315	0.225352	-0.02491	-0.01511	-1.87329	-1.27269	0.690325	-0.31057	-2.48558
Q14966	ZNF638	0.514184	-0.34241	-0.21609	0.14191	-0.05714	0.235588	-0.16631	0.762615	-0.50444	-0.28089	0.486346	-0.54186	-0.00958	-0.4144	0.272574	-0.12615	-0.38379	-0.00615	-0.02981
Q14974	KPNB1	-0.17263	0.544449	0.203768	-0.02015	-0.26877	0.105972	0.051112	0.036653	-0.02751	0.136247	0.243702	-0.23256	0.077766	-0.05624	-0.22667	0.438559	0.046095	-0.0834	0.231546
Q14978	NOLC1	1.785957	-1.46287	-1.31213	0.072682	-1.48705	-0.0849	-0.8979	0.022875	-1.2022	-1.02594	1.157528	-1.62493	-0.09412	-1.71678	0.123393	-0.77129	-0.55955	0.460024	-0.75569
Q14980	NUMA1	0.563407	-0.16255	-0.19553	0.417853	0.779	1.039795	0.352786	0.663905	-0.5421	-0.7223	-0.00647	-0.63847	-0.00865	-0.77141	0.486574	0.744625	-0.96015	-0.43367	-0.31345
Q14997	PSME4	0.250221	0.112598	0.056987	0.071363	0.19969	0.270795	0.243223	0.3121	0.071276	-0.06239	-0.59903	0.201486	0.095504	0.149989	-0.04147	-0.35223	-0.11268	-0.56487	0.19473
Q14999	CUL7	0.783749	0.027387	-0.69243	0.005618	-0.85536	-0.70281	0.262694	0.078283	0.205876	-0.52791	0.024641	-0.79978	0.21119	-0.411	-0.15997	-0.30987	-0.23966	-0.36944	0.694554
Q14BN4	SLMAP	-0.49523	-0.60619	-0.53218	-1.22393	-0.79771	-0.84646	-0.49368	-1.0341	-0.14123	-1.17016	-0.32809	-0.57005	-0.87039	-0.59732	-0.5791	-0.78504	-0.45057	-0.37666	-0.23697
Q14C86	GAPVD1	0.188834	0.582639	0.150076	-0.7585	-0.60698	-0.90855	0.2269	-0.09796	-0.26667	0.066026	-0.75753	0.207854	0.054852	0.419918	-0.62176	-0.00084	0.627553	-0.08455	-0.17444
Q14CX7	NAA25	0.459322	0.187731	0.054496	-0.15393	-0.2411	-0.01438	-0.2504	0.192898	-0.15011	-0.20734	-0.00909	-0.42925	0.30122	-0.41358	0.116591	0.410801	-0.15565	-0.498	0.165655
Q15003	NCAPH	1.226456	-0.27888	0.263104	-0.19123	0.667645	1.29971	0.588548	1.79457	0.215251	-0.52865	-0.88628	-0.50028	0.180526	-0.24685	-0.78933	-0.41148	0.286713	-0.7393	0.535675
Q15005	SPCS2	-0.55983	0.023286	0.106276	-0.02238	0.327684	0.171529	1.636291	-0.19844	0.147688	-0.84749	-0.28177	0.336936	0.277644	0.37342	-0.54367	-0.34466	0.035209	0.360115	-0.15776
Q15006	EMC2	0.195631	0.596538	0.146628	-0.14758	0.138129	0.278487	0.604866	0.015554	-0.06444	-0.34116	0.197639	0.140225	-0.15519	-0.038	0.137743	0.244671	0.027469	-0.1798	-0.38501
Q15007	WTAP	0.075359	-0.41131	0.098339	0.381607	0.345448	0.75648	-0.69688	0.109066	-0.14858	0.253809	0.212726	-0.29003	0.284428	-0.16515	-0.05211	-0.32833	0.08371	0.326843	-0.14529
Q15008	PSMD6	-0.1846	0.502466	0.463669	0.234164	-0.16845	0.042771	0.01166	0.094464	-0.62597	-0.05305	0.046613	0.105128	0.050611	0.272453	-0.07072	0.153454	0.277472	-0.0176	0.325141
Q15014	MORF4L2	1.038401	-0.25871	-0.0209	-0.38145	0.501771	0.447534	-0.60306	0.201874	-0.04277	-0.18557	0.337847	-0.98061	-0.13335	-0.47127	-0.26928	-0.00847	-0.0448	0.476747	0.317051
Q15018	ABRAXAS	-0.70162	0.33417	0.229555	-0.12355	0.02578	0.483816	0.434332	-0.05164	0.275645	-0.30679	0.258026	-0.15287	0.253847	-0.31259	0.153922	0.634599	-0.25052	0.138059	-0.16865
Q15019	SEPT2	0.044012	-0.50426	0.131815	-0.68885	-0.24771	-0.29594	-0.65746	0.030584	0.057178	-0.26058	0.097129	-0.6243	0.103859	-0.40828	-0.03179	0.33779	-0.5395	0.106302	0.705312
Q15020	SART3	0.283987	-0.22956	-0.23514	0.404413	-0.51872	-0.30284	-0.69527	0.493534	-0.53061	-0.50209	0.230307	-0.51434	-0.44961	-0.35211	0.116646	0.009605	-0.35804	-0.04492	0.462604
Q15021	NCAPD2	0.752645	-0.29576	0.351848	0.061097	0.780934	0.916328	1.20221	1.333858	0.118277	-0.25496	-1.10393	-0.40329	-0.28979	-0.09114	-0.81591	0.218504	-0.0601	-0.62433	0.283699
Q15022	SUZ12	0.333905	0.032258	-0.15752	0.233764	0.37806	0.434387	-0.08335	0.610279	0.030473	-0.05705	-0.30908	-0.12199	-0.27324	-0.09779	0.392671	-0.16798	0.226055	1.004324	-0.10572
Q15024	EXOSC7	0.667354	-0.20901	-0.08175	-0.23401	0.825351	0.849457	-0.51548	0.342942	-0.50118	-0.1074	0.497782	-0.1577	-0.2576	-0.22555	-0.54772	-0.14975	-0.00696	0.175122	-0.13032
Q15025	TNIP1	-0.81725	0.424605	0.243632	-0.36143	0.07946	0.152552	0.233688	-0.34209	0.702945	0.663689	0.231189	0.357048	0.977234	0.300908	-0.28172	-0.38625	0.373849	-0.0997	0.126516
Q15027	ACAP1	-1.49695	1.026501	0.014928	-0.85847	0.588539	0.306721	0.764996	-1.41679	0.308934	0.848845	-0.97446	-0.08676	-0.2785	1.085095	-0.8265	-1.66618	0.058633	0.839502	-0.82472
Q15029	EFTUD2	0.54935	-0.45303	0.047563	0.391861	0.228374	0.285207	-0.603	0.428927	-0.03658	-0.09667	0.372597	-0.01616	-0.22644	-0.15939	0.179636	0.005432	0.140942	0.301377	0.019149
Q15031	LARS2	0.483851	-0.26548	0.559387	0.47029	-0.21521	-0.26209	0.107064	-0.71025	-0.6705	-0.2486	0.626595	-0.0584	-0.32502	-0.08954	0.235923	0.148603	-0.58115	-0.37619	-0.60364
Q15035	TRAM2	-0.61393	0.033466	-0.18433	-0.42191	-0.21273	-0.54259	0.192337	0.018191	0.504336	-0.09646	-0.79191	-0.51808	0.372028	-0.31224	-0.69504	0.175232	-0.50058	-0.46871	0.80233
Q15036	SNX17	-0.59285	0.784125	-0.04186	0.112874	0.398744	-0.05773	-0.09978	-0.44725	0.270535	0.119717	-0.52559	0.544315	0.81529	0.583764	-0.2594	0.145809	-0.38378	0.109686	0.248777
Q15041	ARL6IP1	0.228731	1.165642	1.084175	0.024008	0.363617	0.397591	0.184569	0.385751	-0.81167	-0.31772	-0.44338	0.954754	0.535397	0.26694	-0.59412	-0.36348	0.439211	0.280876	0.796846
Q15042	RAB3GAP	0.007124	0.488814	0.359614	-0.17266	-0.19696	-0.18341	-0.03834	0.152026	0.045321	0.387051	-0.32417	0.252893	0.557822	0.138646	0.815888	0.300144	0.01253	-0.48644	0.367184
Q15043	SLC39A14	0.111581	0.174033	0.75626	-0.52826	0.272663	0.245127	0.739999	0.226444	0.174968	0.00892	0.129702	-0.53016	0.086608	-0.2696	0.57044	0.122355	0.525918	0.960556	1.436904
Q15046	KARS	-0.29477	-0.49165	0.108495	0.033494	0.513598	0.624335	-1.12948	-0.6303	-0.2522	-0.03682	0.228981	-0.55122	-0.06447	-0.12137	0.464039	1.007907	-0.44064	-0.18934	0.106513
Q15047	SETDB1	0.232849	-0.11412	-0.09709	-0.46994	-0.19943	-0.21335	-0.131	0.231403	0.029894	0.128892	0.151044	-0.8072	-0.12066	-0.226	0.298834	-0.54365	-0.03294	0.076156	0.154282
Q15048	LRRC14	0.866797	0.714451	0.115267	-0.41487	0.026882	0.262985	0.323638	-0.52571	0.285833	-0.33652	-0.17759	0.100495	0.116886	0.058379	-0.12263	0.219064	-0.6175	-0.05667	-0.30146
Q15050	RRS1	0.120729	-0.17149	-0.37189	0.829507	-0.14981	-0.05805	-0.19451	-0.4809	-0.88221	-0.68992	1.12911	-0.03486	0.834969	-0.98974	0.087891	0.238682	-0.21431	0.721917	-0.26414
Q15051	IQCB1	0.583898	-0.43158	-0.38511	-0.92021	-0.48679	-0.17353	-0.28277	-0.17122	-0.17209	-0.37825	0.266249	-0.63479	0.204381	-0.43418	0.28175	-0.16993	0.647761	-0.10743	-0.4917
Q15052	ARHGEF6	-1.34364	1.099441	0.706491	-0.64819	0.399686	0.292862	0.088162	-1.55192	-0.01056	1.182887	-1.01261	1.20963	0.040262	1.399287	-0.2002	-0.39627	0.404774	0.154825	-0.89144
Q15054	POLD3	1.666864	-0.52745	0.186451	0.347008	1.128518	1.530494	-0.2702	1.164791	-0.59361	-0.62654	0.324767	-0.69855	-0.27408	-1.08608	-1.0176	0.513213	-0.25775	-0.71985	-0.80399
Q15056	EIF4H	0.432043	0.197322	-0.24182	-0.36232	0.514231	1.060088	-0.83293	0.331178	-0.3765	0.406246	-0.14099	-0.39654	0.922787	-0.35322	-0.1106	0.131235	-0.01569	-0.15366	0.595459
Q15057	ACAP2	0.131253	0.426502	0.353471	0.014353	0.08816	0.37173	-0.05661	-0.33329	-0.08389	0.637431	-0.52711	-0.02025	-0.00975	0.825301	0.926129	-0.58667	-0.16001	-0.25662	-0.17894
Q15059	BRD3	1.348619	-0.17581	-0.19941	-0.41779	-0.10379	-0.05091	-0.17747	0.891946	-0.46468	-0.34293	0.329144	-0.63167	-0.49048	-0.46594	-0.36402	-1.15934	-0.68905	-0.21522	-0.1219
Q15061	WDR43	0.459282	0.06304	-0.13935	0.917564	-0.442	-0.30349	-0.06526	0.411772	-0.88776	-0.05398	0.885842	-0.52014	-0.15646	-0.78301	0.063008	0.31699	-0.53404	-0.43971	-0.02849
Q15063	POSTN	-1.63742	-1.03091	-0.52928	-1.35916	-2.16585	-2.5972	-0.99801	-0.77545	1.425139	-0.78796	-2.704	-1.53323	-1.06001	-1.03575	0.184051	0.248195	-1.33829	-1.93832	-0.0209
Q15067	ACOX1	0.456625	-0.59588	0.212557	1.118144	-0.17296	0.073142	-0.76884	0.926704	1.135682	0.354164	-0.24102	0.937494	0.287077	0.9146	-0.51134	0.423521	0.567527	-0.23688	0.186358
Q15070	OXA1L	-0.21658	0.524308	0.487922	0.75722	-0.48519	-0.42124	0.300405	-0.4276	0.124877	0.19437	0.428748	-0.10016	0.604545	-0.51755	1.25795	0.766745	-0.39702	-0.33482	-0.8455
Q15075	EEA1	-0.43137	0.394914	0.863441	-0.353	0.256166	-0.15786	-0.66017	-0.62554	-0.19054	-0.11038	-0.37976	-0.79847	0.459831	-0.33089	0.258921	0.346839	-0.12208	1.093096	0.862102
Q15080	NCF4	-1.94106	0.986576	1.021143	-1.00755	-0.16194	-0.63046	-0.54033	-1.4272	0.080378	1.540104	-1.87752	1.910834	0.151211	2.80847	-0.90421	-1.18168	1.190474	-0.85442	-1.9686
Q15084	PDIA6	0.416256	0.137064	0.206604	-0.22106	0.019122	-0.26182	-0.39113	0.465021	0.01872	0.517331	-0.21221	0.124689	0.602669	-0.24938	-0.17384	0.737501	-0.0798	0.15094	-0.13139
Q15102	PAFAH1B:	1.149025	-1.07573	-0.23213	-0.51025	-0.21659	0.205491	-0.84354	2.417499	0.306119	-0.23677	-0.37338	-0.15538	0.176159	-0.12714	0.085567	-1.55736	-0.37214	-0.68077	-0.03734
Q15113	PCOLCE	-0.80061	-0.05934	-0.71958	-1.01268	0.244117	-0.52111	0.199917	-0.54131	0.885066	0.279598	-0.04465	0.352079	-0.47432	0.30502	-0.04344	-0.21356	0.417517	-0.26141	0.076471
Q15118	PDK1	0.939389	-0.26634	-0.48346	0.5041	-0.64625	-1.17679	0.950013	0.028775	-0.40791	0.618953	-0.43351	0.147803	0.24066	0.134882	-0.34341	0.097278	0.226374	-0.34342	-0.01516
Q15119	PDK2	-0.36363	0.037129	-0.18908	0.209485	0.378545	-0.08067	-0.32807	0.021053	1.076327	0.400932	-0.07542	0.037039	0.159266	-0.33465	0.956033	-0.55388	-0.3581	-0.65435	-0.31639
Q15120	PDK3	0.966799	-0.16583	-0.7181	0.696856	-0.30989	-0.57762	-0.46197	-2.14995	0.036827	-0.26525	1.05946	-0.11639	-1.09152	0.671223	-0.72338	-0.48294	-0.20054	-0.08468	-0.24402
Q15121	PEA15	-0.00523	-0.0936	-1.13581	-1.07498	0.110844	0.048305	-0.70952	0.709096	0.138107	0.90243	-0.57881	0.166447	-0.29632	0.297752	0.191249	-0.82546	0.128089	-0.81098	-0.62009
Q15124	PGM5	-1.32994	-1.08394	-0.67057	-0.24682	-1.27713	-1.39437	-0.72613	-0.20299	-0.58646	-1.35753	0.297502	-1.28831	-0.52032	-0.78774	0.573784	-1.43036	-1.15331	-0.79207	-1.34417
Q15126	PMVK	0.022969	-0.70552	-1.71373	-0.41033	0.133158	-0.47713	-0.53593	0.482354	0.262755	1.17995	-0.55108	0.743283	-0.07958	0.727677	0.017018	-0.17446	0.663121	-0.64339	-0.60195
Q15139	PRKD1	-0.69896	0.358153	0.116509	0.007976	-0.31961	-1.07516	0.242937	-0.42994	0.322744	-0.45375	-0.21905	-0.39116	0.003563	-0.41701	-0.71391	0.10146	-0.44651	0.375869	1.37488
Q15147	PLCB4	0.337566	0.039875	-0.01203	-0.27851	-0.45475	-0.554	-0.15601	1.216176	0.1026	1.114567	0.12397	-0.40986	-0.01687	-0.12146	-0.11244	-0.7423	-0.60922	0.191137	1.099853
Q15149	PLEC	0.462302	0.115883	0.069734	-0.45734	-0.7655	-0.68911	-0.03137	-0.16349	-0.04864	-0.3252	-0.39888	-0.14185	0.495212	-0.53799	-0.65032	0.655091	0.308497	0.141758	-0.05123
Q15154	PCM1	0.333959	-0.43118	-0.62186	-1.42894	-0.26147	0.010752	-0.26344	-0.45977	-0.20675	-1.0283	0.729434	-0.35195	-0.40411	-0.20731	-0.39896	-0.35299	0.255555	0.22538	-0.32857

Q15155	NOMO1	-0.39417	0.307809	0.289777	-0.10031	0.158909	-0.09674	0.987981	-0.19564	-0.23332	-0.00911	0.168995	0.272514	0.061413	0.140202	0.327053	-0.04275	0.229987	0.1911	0.029713
Q15165	PON2	-0.14115	0.382341	-0.2766	-0.42353	0.113666	0.062155	-0.27506	0.147267	1.259614	0.317722	-0.33647	0.014524	-0.0958	-0.37035	0.753108	0.590009	0.175856	0.62059	-0.32318
Q15172	PPP2R5A	0.080081	-0.25884	-0.37248	-0.45525	-0.56117	-0.75575	-0.51348	-0.19364	-0.59767	0.441379	-0.20231	-0.27487	-1.02134	0.727762	1.166563	-0.01849	-0.39722	-0.57408	-0.24191
Q15173	PPP2R5B	-0.15376	0.259789	0.203964	-0.28318	-0.3335	-0.17477	0.255533	-0.05974	0.120459	0.417166	-1.09925	0.269418	0.818744	0.498181	0.540336	0.263411	-0.15203	-0.71958	0.900206
Q15181	PPA1	0.106352	-1.3981	-0.70455	0.005129	2.122217	0.782718	-0.89433	1.108141	0.476725	0.778567	0.045552	0.610919	1.100884	0.024535	-0.87045	1.53163	0.559967	-0.86066	-1.45637
Q15185	PTGES3	0.39613	-0.63685	-0.75815	-0.11711	1.38741	1.164735	-2.33509	0.298833	-0.77007	0.50813	0.66458	0.414653	0.163182	0.469215	-0.53543	-0.61866	1.003114	-0.53592	-0.58412
Q15198	PDGFRL	-0.23881	-0.90808	-1.00183	-1.22825	-1.56727	-1.85981	-0.29092	-0.125	0.343405	-0.691	-1.29361	-1.20061	-0.87284	-0.45913	0.590839	-0.94421	0.515742	-1.47149	0.31987
Q15208	STK38	-0.15948	0.099468	0.449271	-0.55845	0.496078	0.589721	0.161165	-0.22838	0.589841	0.828606	-0.4018	0.148058	-0.0543	0.781078	-0.17591	0.288358	0.763784	0.605541	0.315462
Q15233	NONO	0.527237	-0.29866	0.07618	0.358353	0.210616	0.341896	-0.29523	0.425064	-0.14023	-0.08927	0.3789	-0.05506	-0.01378	-0.19614	-0.08884	-0.23535	0.184611	0.511254	-0.14368
Q15257	PTPA	-0.04214	0.08081	-0.11473	-0.74987	0.263684	0.140482	0.196282	0.846211	-0.11324	-0.08517	0.400569	-0.85209	-0.31701	-0.52294	-0.43585	-0.281	-0.63229	-0.11483	0.404215
Q15262	PTPRK	0.569274	-0.36328	-0.24248	1.104247	0.034141	-0.1589	-0.33206	-0.63757	-0.51695	-0.80572	1.098227	-1.02804	-0.4996	-0.03295	1.015599	1.258226	-0.08941	0.31393	-0.66615
Q15269	PWP2	0.527647	0.110078	-0.56482	0.356447	-0.23127	-0.26121	-0.45188	0.098801	0.06515	-0.0783	0.708819	0.103828	-0.09649	-0.20315	0.159228	0.107876	-0.00206	-0.25766	-0.47466
Q15274	QPRT	0.153072	-1.18063	0.107625	0.877676	1.255822	0.365575	0.178594	0.458442	0.235227	-0.81306	-0.98758	-0.83955	0.387776	-1.35325	-1.93252	-0.55641	0.456068	1.572331	-0.18899
Q15276	RABEP1	0.359863	0.032742	0.655417	-0.71147	0.14912	0.350952	-0.20851	-0.34925	0.94423	0.384317	-0.50145	-0.21752	0.35873	-0.07754	-0.14173	-0.22959	-0.73826	0.059896	0.490797
Q15283	RASA2	-0.06962	0.24964	0.372776	-0.45834	0.367681	0.400148	0.066701	-0.41946	0.221522	0.456006	-0.40702	0.252491	0.048058	1.064988	0.349039	0.300406	0.036913	0.33664	-0.13794
Q15286	RAB35	-0.12982	0.166474	0.135003	0.031674	0.476611	0.08282	0.189603	-0.0149	-0.16264	-0.07051	-0.50957	0.870176	-0.36058	0.661235	0.109098	-0.20043	0.109939	-0.36011	0.069944
Q15287	RNPS1	0.357301	-0.25903	0.197659	0.376833	-0.17289	0.209449	-0.69194	0.338696	0.37573	-0.41346	0.424906	-0.39554	-0.25031	-0.11352	0.161594	0.323411	0.014611	0.079867	-0.42953
Q15291	RBBP5	0.603969	-0.13691	0.021991	-0.05993	0.141979	0.514414	-0.46004	0.895854	0.609202	-0.46722	0.098825	-0.35833	0.18294	-0.22258	0.686793	-0.50129	-0.30142	-0.20351	-0.18662
Q15293	RCN1	0.191358	-1.08073	0.547619	0.51418	0.612036	0.309964	-0.82506	1.023077	0.031462	0.375841	0.306859	-0.33271	0.45481	0.438065	-0.96756	1.234343	0.743933	0.507456	0.098209
Q15311	RALBP1	0.08423	0.166737	-0.09736	0.23334	5.73E-05	0.183826	-0.0258	-0.37078	-0.46071	0.049191	-0.03853	0.227421	-0.40208	0.173172	0.94395	-0.04735	0.046773	-0.51937	-0.57633
Q15323	KRT31	-0.63463	4.495679	-1.01548	-1.5076	-4.18409	-0.99933	-0.18059	-0.93826	0.636389	-1.21771	-3.2597	-0.48541	-0.55859	-0.87271	-1.73886	-1.13544	-0.90958	-3.3416	-1.23736
Q15326	ZMYND11	0.450252	-0.222	-0.32502	0.174782	0.13649	0.239046	-0.09448	0.027576	-0.58856	-0.98388	-0.58054	-1.18005	-0.05685	-0.44397	0.428184	-1.19066	-0.1886	0.048624	-0.65381
Q15334	LLGL1	-0.16029	0.662667	0.1869	-0.48102	-0.25707	-0.12317	0.514426	-0.04974	0.094754	0.261597	-0.162	-0.45115	0.296898	-0.17984	-0.08033	-0.26439	-0.3894	-0.01584	0.767913
Q15345	LRRC41	-0.41515	0.388942	-0.1235	1.237809	0.696145	0.40768	-0.017	-0.50234	-0.04475	0.06965	0.563147	-0.72795	-0.55622	-0.01247	-0.34939	-1.28939	-1.10745	-0.23536	-0.43132
Q15363	TMED2	-0.68133	0.633721	0.229346	0.223377	-0.02813	-0.01097	0.252201	-0.07206	-0.04131	-0.35649	0.323045	0.345038	0.147707	0.258527	0.341525	0.213797	0.552476	0.738755	0.725092
Q15365	PCBP1	0.245068	0.028879	-0.45198	-0.66051	-0.04704	-0.22407	0.30513	0.63839	-0.27814	0.409297	-0.07613	0.27147	-0.21553	0.419564	-0.2499	-0.24688	0.023497	-0.19149	-0.16307
Q15366	PCBP2	-0.0035	-0.19663	-0.92488	-0.4897	-0.28419	-0.46607	-0.39872	-0.19609	0.124359	0.619646	-0.29854	-0.13859	-0.0261	0.037097	-0.36599	-0.58683	0.883427	0.137445	-0.08964
Q15369	ELOC	0.246907	-0.40099	-0.00601	0.026955	-0.0655	0.252656	-0.25762	0.238689	0.027868	0.084754	-0.11778	0.063233	0.675381	0.421879	0.217501	0.338108	0.725959	0.130086	-0.0114
Q15370	ELOB	0.730147	-1.62364	0.413635	0.310522	0.80274	-0.21743	-0.30552	-0.57191	0.984438	1.369051	-0.46211	0.947467	0.029302	0.874197	-0.23863	0.264201	1.531534	-0.17241	0.028241
Q15382	RHEB	0.421303	-1.58217	-0.55775	0.894678	1.631891	0.217831	-1.60944	0.287987	0.189232	1.175794	-0.82066	1.483528	0.499975	1.092461	0.816456	0.714227	0.625076	-0.39001	-0.0034
Q15386	UBE3C	-0.92816	0.326632	0.17194	0.901595	-0.2088	-0.19483	-0.23981	-0.04144	-0.47599	0.185179	-0.24469	0.114024	0.234001	0.226659	0.425461	0.972301	-0.29487	-0.82302	-0.05811
Q15388	TOMM20	-0.23198	-0.42165	0.184655	0.692809	0.018088	0.251114	-0.30901	-0.08784	0.129855	-0.22871	0.276561	0.177704	0.620958	0.388323	0.349092	0.538698	0.374868	0.482264	-0.37435
Q15392	DHCR24	0.755487	-0.53443	1.369028	1.365038	2.241108	2.370855	-0.34924	-0.56471	0.004734	0.166109	-0.2386	0.824783	0.992999	0.343249	1.061294	0.271231	-1.13136	-1.42535	-2.25499
Q15393	SF3B3	0.343183	0.026963	-0.0465	0.42943	0.047224	0.089717	-0.18807	0.379127	-0.22091	-0.013	0.260319	-0.354	-0.43265	-0.20692	0.178167	0.172493	-0.16098	-0.06556	0.029723
Q15397	PUM3	0.347488	-0.08449	-0.28709	0.695322	0.005532	0.279979	-1.20266	0.136506	-0.81609	-0.65896	0.298738	-0.11566	-0.73956	-0.47248	-0.47404	-0.19073	-0.00774	-0.33144	-0.16262
Q15404	RSU1	-0.95031	0.19518	-0.84756	-1.25392	0.424223	-0.22774	0.138045	-0.46239	0.133427	-0.48424	-0.48525	-0.73609	0.02815	-0.38572	-0.28445	0.098683	-0.74442	0.341335	-0.40319
Q15417	CNN3	-0.05809	-1.31892	-0.74506	-1.02324	-0.90068	-0.87302	-1.23994	-0.88409	1.010158	-0.05181	-1.22841	-1.19905	0.368016	-1.08735	-0.43189	0.86564	-0.63189	-0.06599	1.095798
Q15418	RPS6KA1	0.33273	0.282505	0.086492	-0.16423	0.058931	0.222085	0.481972	0.414529	0.349737	0.926832	0.039397	0.826592	-0.28986	1.036048	0.162468	-0.15122	0.754178	-0.12235	-0.38332
Q15424	SAFB	0.479223	-0.32273	0.023694	-0.08984	-0.07904	0.455123	-0.67176	0.40175	-0.63989	-0.02023	0.584086	-1.17501	0.055881	-1.06438	0.494391	-0.44287	-0.78757	0.373746	-0.1206
Q15427	SF3B4	0.212485	-0.33238	-0.3889	0.4789	-0.02384	0.256964	0.027149	0.594275	-0.62938	-0.09951	0.186439	-0.34236	-0.44938	-0.18599	0.441137	0.091089	-0.09866	0.33126	-0.22449
Q15428	SF3A2	0.722889	0.052922	0.365506	0.613864	0.003592	0.115553	-0.02436	0.356026	-0.21969	0.014931	0.560452	0.144106	-0.39319	-0.40442	0.212908	-0.27672	-0.076	0.527539	-0.29288
Q15435	PPP1R7	-0.614	0.292846	-0.36907	-0.88769	-0.08459	-0.37051	-0.18026	0.038833	-0.8321	0.113261	0.276796	-0.42158	-0.1063	-0.14288	-0.37328	0.429311	-0.56015	0.020077	0.042244
Q15436	SEC23A	-0.93177	0.944028	0.409181	-0.26783	-0.0795	-0.08171	0.515781	-0.19973	0.206751	0.182388	-0.30215	-0.34198	0.391918	0.019126	-0.29091	0.312759	0.104805	0.524268	1.020198
Q15437	SEC23B	-0.24529	-0.23695	-0.12206	0.072051	-0.099	-0.1112	0.679009	0.180577	0.566052	0.148108	-0.11325	0.418278	0.287864	0.146429	1.042007	0.334963	0.314945	0.196436	-0.88793
Q15438	CYTH1	-0.88499	1.298443	0.776665	-0.54058	0.182523	-0.07939	0.447652	-0.64809	-0.09988	0.519439	-1.24379	0.451006	0.094845	0.722491	-0.70009	-0.40814	-0.39814	0.328116	-1.46808
Q15459	SF3A1	0.301251	-0.06658	-0.05326	0.49361	-0.01626	0.195301	-0.45424	0.448259	0.138095	0.034849	0.362944	-0.22144	-0.27609	-0.53134	0.259447	-0.11627	-0.39877	0.255284	-0.1949
Q15477	SKIV2L	-0.36358	0.216826	-0.23356	0.248857	-0.44865	-0.20345	0.24369	-0.24853	0.392538	-0.29684	0.370834	-0.13484	-0.21607	-0.02637	0.585237	0.030316	-0.129	-0.12168	0.362361
Q15485	FCN2	-1.62018	0.371915	-0.27542	-1.7798	-0.80016	-1.78242	-0.12508	-0.93159	0.328934	1.641392	-0.13399	1.348367	0.070409	2.296293	-0.40646	-0.25832	2.085839	1.314367	-0.59207
Q15526	SURF1	-0.10581	0.05553	-0.0104	0.153069	0.07836	-0.39941	0.325339	-0.52072	-0.4296	-0.16891	-0.31198	0.548907	-0.03407	0.499457	0.235111	0.234825	-0.24967	-0.36338	-0.53792
Q15528	MED22	0.729992	0.361788	0.277708	-0.48892	0.325438	0.3668	0.468183	0.459554	-0.0947	0.479549	0.176428	0.437539	0.587273	0.353004	-0.50884	-0.17574	0.197416	0.09914	-0.4712
Q15542	TAF5	-0.01501	0.069966	-0.11233	0.115765	-0.00457	-0.20525	-0.04059	0.661215	0.102893	-0.05505	-0.1191	0.090598	0.029477	0.105656	0.370846	-0.40705	-0.05637	-0.06337	-0.60012
Q15545	TAF7	0.130637	0.018731	0.078222	-0.39203	-0.09707	0.353804	-0.15487	0.356324	0.157802	0.25124	0.255955	-0.93048	0.204989	-0.35806	0.231667	0.464753	-0.42549	0.08792	0.295701
Q15554	TERF2	-0.27766	0.230495	0.028612	0.589037	0.459781	0.536472	-0.20935	-0.15875	-0.39894	-0.41826	0.432824	-0.39758	-0.21574	-0.48598	0.215934	0.096055	0.018606	0.669344	-0.26285
Q15555	MAPRE2	-0.62043	0.184	0.629245	-0.92107	-0.52537	0.019785	-0.21414	-0.20642	-0.47755	1.156341	-0.76898	-0.1485	0.232645	0.064269	-1.06119	-0.25622	-0.61666	0.811591	0.95721
Q15582	TGFBI	-1.20894	0.292179	-0.40633	-1.38022	-0.42904	0.53655	-0.74142	-0.69755	1.246351	0.790156	0.438957	-0.62142	0.884376	-0.66817	-0.63175	-0.35199	-0.74345	-0.34338	1.105036
Q15596	NCOA2	0.602269	-0.42795	-0.24253	-0.8592	-0.19348	0.288239	0.297331	-0.31428	1.354665	-0.45572	-0.28664	-0.41465	1.040475	-0.57083	0.191202	0.138644	-0.25281	0.070536	-0.09607
Q15599	SLC9A3R:	-0.04963	-0.99177	-0.61457	-1.0431	-0.66494	-1.05096	0.122917	0.013083	4.788404	-0.9027	0.533753	-0.80628	0.081672	-1.14019	0.784072	0.30632	-0.30164	0.710697	-1.0909
Q15628	TRADD	-0.88078	0.053686	0.430285	-0.12584	0.088835	0.127616	0.218367	-0.93513	-0.0774	0.40962	-0.38214	0.611889	0.165531	0.811713	0.070417	-0.15234	0.196982	-0.56926	-0.04444
Q15629	TRAM1	-0.37734	0.126784	0.226477	-0.11895	0.322417	0.441143	0.506401	-0.453	-0.11164	-1.17506	-0.57749	1.86799	0.250005	0.160022	0.444044	1.029709	-0.06525	-0.11384	-0.45183
Q15631	TSN	0.272597	-0.51572	-0.30228	-0.34	-0.91289	-1.20586	-0.60723	0.282708	0.472378	-0.25243	0.663677	-0.65647	-0.25069	-0.42821	0.281085	-0.47955	-0.30306	-0.399	0.230621
Q15633	TARBP2	0.087375	0.107918	0.141049	-0.73752	-0.48267	0.41622	0.35558	-0.06417	0.15403	0.214689	-0.11679	-0.11783	0.01588	0.135923	0.213359	0.025837	0.249818	0.422309	0.35242
Q15637	SF1	0.449951	-1.1702	0.072682	0.068785	0.267243	0.19853	-0.97458	0.405964	-0.007	-0.06066	0.092716	-0.11252	0.37862	-0.39123	0.169046	0.155582	-0.2963	0.289203	-0.03927
Q15642	TRIP10	-0.14488	-0.03835	0.05648	-0.84655	-0.14514	0.16346	-0.13026	-0.59278	0.457459	0.806231	0.115003	-0.95519	0.034825	-1.11715	-0.60061	-0.03654	-0.6835	-0.19766	-0.00042
Q15643	TRIP11	-0.44894	0.10138	0.298908	0.30333	0.894098	1.100958	0.49069	-0.39143	-0.14166	-0.1639	-0.26666	-0.1228	0.626459	-0.19149	1.535134	0.694243	0.113552	0.567814	0.046245
Q15645	TRIP13	0.456393	-0.08003	-0.45428	-0.7972	0.124433	0.763956	0.240954	-0.12421	0.20939	-0.30573	-0.7771	0.036089	0.100005	0.009025	-0.81575	-0.2322	0.722135	-1.2329	-0.17447
Q15646	OASL	0.069414	1.41492	1.446431	-0.51087	0.147443	0.735503	0.099166	-0.23454	-0.16212	0.044534	-1.10032	0.599545	0.558626	0.781238	-1.24798	-0.31653	0.051257	0.35847	-0.88818
Q15648	MED1	-0.13058	0.374783	0.453972	0.032829	-0.06825	0.075314	0.453053	0.287114	0.227995	-0.4265	-0.28278	0.019602	0.059801	0.079313	0.47309	-0.11547	-0.17085	0.341728	-0.03105
Q15650	TRIP4	-0.02645	0.029263	-0.04254	0.328882	-0.33235	0.183329	-0.42516	-0.82986	-0.06279	-0.33914	-0.13248	-0.09267	0.229948	0.042992	0.245466	-0.00693	0.34413	-0.03014	0.133547
Q15652	JMJD1C	0.426325	0.024123	-0.06697	-0.04038	-0.07326	0.118921	-0.18315	0.323155	0.056102	0.305018	-0.11288	-0.53572	-0.26676	0.107785	0.121777	-0.05489	0.085834	0.550861	-0.21171
Q15653	NFKBIB	-0.24878	-0.61669	-0.02649	-0.10557	0.392275	1.556416	-0.36669	1.498932	-0.19766	-0.07088	0.232733	0.030891	0.62321	-0.63632	0.420335	0.022582	-0.66904	0.212154	0.837008
Q15654	TRIP6	-0.06544	-0.0858	-0.2837	-0.18988	-0.30951	-0.02116	0.018244	0.29497	-0.02994	-0.65883	0.342005	-0.52053	0.858335	-0.244	0.922383	0.098586	-0.49874	-0.55079	0.057458
Q15661	TPSAB1	-0.54231	-0.10355	-0.85125	-0.517	-0.60039	-1.53671	0.523343	-0.56349	0.87549	-1.03836	-0.57323	-0.366	-0.27706	-0.63261	0.388823	-1.43352	-0.47285	-0.23917	0.279348
Q15678	PTPN14	0.504343	-0.19867	-0.26688	-0.5111	-0.29311	-0.53948	0.008144	0.43511	-0.20759	-0.83537	0.404921	-0.97138	0.053648	-0.77176	0.230867	-0.05682	0.198451	-0.07789	0.846132
Q15691	MAPRE1	1.053229	-0.05563	-0.26379	-1.15385	0.236485	0.254588	-0.56368	0.212621	-0.1959	0.51792	-0.13927	0.049826	0.884056	-0.11384	-0.31877	-0.07159	0.139371	0.078875	0.510829
Q15714	TSC22D1	-0.45053	-0.42492	-0.45111	-1.65219	-1.24117	-1.06135	0.566207	0.402918	0.632952	-0.72583	-0.70839	-0.33592	-0.08865	-0.63008	0.704089	-0.76719	-0.12292	-0.08619	0.811185
Q15717	ELAVL1	0.333536	-0.15805	-0.55872	-0.45007	-0.04169	-0.03785	-0.11741	0.244933	-0.39239	0.120179	0.380781	-0.19978	-0.47288	-0.3716	-0.38175	-0.03022	0.09717	0.139242	-0.06579
Q15723	ELF2	-0.29802	0.053386	-0.15227	-0.28225	-0.29824	-0.72834	-0.29313	0.330641	0.523747	-0.59612	-0.5397	-0.08841	-0.06314	0.07599	-0.2405	-0.89396	-0.10546	-0.65187	-0.30228
Q15738	NSDHL	-0.41143	0.348498	0.473934	0.813421	0.669501	0.32991	0.201748	-0.34058	-0.03586	-0.09049	0.119124	-0.00196	-0.37209	-0.23613	-0.71096	0.049694	1.075613	0.317977	-0.40698
Q15742	NAB2	0.570191	-0.56076	-0.03131	-0.78469	-0.07549	-0.04913	-0.50081	0.356765	-0.69059	-0.23621	-0.2552	-0.72433	-0.59227	-0.5166	-0.33761	-0.75974	-0.32821	-0.19491	0.116044
Q15746	MYLK	-1.19123	-0.69444	-0.75213	-1.67978	-1.23942	-1.05376	-0.5419	-1.71698	0.286624	-0.62949	-1.29395	-1.01904	-0.69759	-1.21158	-0.2283	-0.36012	-1.37963	-0.9544	0.198423
Q15750	TAB1	0.22716	0.008719	-0.06846	-0.28988	-0.06645	-0.15893	-0.78009	-0.21771	-0.3877	0.646257	-0.46121	-0.04473	0.423061	0.254374	0.161162	-0.13302	0.166227	0.035619	0.088409
Q15751	HERC1	-0.08434	0.733632	0.69482	-0.20394	-0.2252	0.308938	1.112448	-0.23583	-0.03626	0.311425	-0.29318	-0.35944	0.40304	0.014906	0.057713	0.185629	0.265461	-0.31287	-0.21765
Q15758	SLC1A5	1.692453	0.078359	-0.61718	0.362366	-0.10406	0.259633	0.814093	0.237333	-1.47992	-0.5191	1.1356	-0.29396	0.244792	-1.23719	-1.36567	0.305392	0.065028	-0.07691	-0.75916
Q15771	RAB30	-0.48115	-0.02219	0.073684	-0.27583	0.179499	-0.57028	0.10935	-0.43734	0.378928	-1.47434	-0.91429	-0.6817	-0.24385	-0.52702	0.287106	-0.62949	-0.23834	-0.41496	0.222291
Q15773	MLF2	0.18823	0.116679	-0.8567	0.255824	-0.72852	-0.05104	-0.05316	0.410267	0.417975	-0.77113	0.210922	-0.60034	-0.38482	-0.21722	-0.12773	0.901179	0.161419	0.740313	-0.43359
Q15776	ZKSCAN8	0.552966	0.370011	0.140793	0.485642	-0.36449	-0.24094	0.130505	-0.13631	0.096195	-0.05635	-1.11505	-0.14551	0.31941	-0.11548	0.03583	0.312301	-0.23025	-0.1396	0.552036
Q15785	TOMM34	0.341029	-0.72237	-1.24415	-0.4676	-0.27529	-0.13232	-0.46147	0.346797	0.26525	0.146597	0.388516	-0.55522	-0.07052	-0.58105	-0.39968	-0.93782	-0.13335	-0.93894	-0.10594
Q15788	NCOA1	-0.16469	0.508826	-0.03415	-0.28789	-0.57618	-0.59473	-0.24255	-0.14539	0.040847	-0.04073	0.153039	-0.59346	-0.06428	0.620728	0.669617	-0.19497	-0.49042	-0.07776	-0.28034
Q15796	SMAD2	-0.09876	0.000332	-0.02255	-0.22919	-0.44456	-0.65265	-0.8534	-0.12798	-0.46892	0.509643	0.388908	-0.14323	-0.13531	-0.19141	-0.61302	0.269735	-0.01136	-0.74735	0.296411
Q15797	SMAD1	-0.20294	-0.05547	-0.53526	-0.44	0.449944	-0.02532	0.025313	-0.18759	0.198987	-0.02667	0.02781	-0.64461	-0.15482	0.016785	-0.03364	0.548519	-0.0943	-0.65421	-0.23982
Q15811	ITSN1	-0.59972	0.739276	0.43306	-0.94518	-0.19355	-0.42569	-0.00328	0.062449	0.186937	-0.24421	-0.24553	-0.01253	0.148042	-0.26709	-0.04231	0.161307	-0.43421	0.665361	0.272899
Q15813	TBCE	-0.49244	-0.51366	-0.46122	0.482068	-0.60985	-0.38089	-0.14815	-0.05689	-0.0744	0.478938	-0.51225	0.268471	0.234045	0.101357	0.108978	0.721373	0.700332	-0.12669	0.062555
Q15814	TBCC	0.306628	-0.75731	-0.35303	-0.21007	-0.02805	0.26362	-0.46789	-0.00194	-0.33489	-0.12704	-0.00904	-0.51652	0.431168	-0.09944	0.403717	0.151607	-0.32075	-0.32804	-0.04793
Q15819	UBE2V2	-0.00013	-0.0848	-0.18083	-0.5986	1.089186	-0.53658	0.410351	-1.51341	0.107712	1.349037	0.06922	-0.02509	-0.10501	-0.48596	-0.40451	-0.66364	-0.33741	0.597815	-0.49984
Q15833	STXBP2	-0.50578	1.173355	0.299972	-0.09588	0.137789	0.290085	0.037175	-0.5989	0.465088	0.988052	-0.53357	0.754063	0.539133	0.930637	-0.6336	0.780243	0.209571	-0.65696	-0.60666
Q15836	VAMP3	-0.00947	0.379428	0.14826	-0.4495	-0.01046	-0.27811	0.210756	-0.28505	1.037484	0.050937	0.066033	-0.07274	0.028679	0.168834	0.273853	0.138051	0.108183	-0.06056	-0.24665
Q15843	NEDD8	0.464467	-0.2002	0.224032	0.425943	0.070451	0.003317	-0.42462	-0.17856	0.037921	0.460095	0.134725	-0.87786	0.260166	-0.38785	0.231286	0.1819	-0.02694	0.222627	-0.00648
Q15847	ADIRF	-1.94813	-0.45641	-0.07538	-0.90298	1.448632	0.317853	0.35198	-3.91994	0.608208	-2.86241	-3.29904	-0.19858	-0.84464	-0.73235	1.856042	-2.70611	-0.58608	-1.69464	-1.985
Q15853	USF2	0.013241	0.259025	-0.19581	0.055045	-0.86248	-0.10478	-0.05389	0.240414	0.061028	0.6041	-0.50775	-0.22255	-0.25589	-0.24278	0.280344	0.084683	0.026939	-0.04843	-0.28949
Q15904	ATP6AP1	0.466334	1.013622	0.1756	-0.32299	-0.09298	0.087562	0.526968	-0.60168	-0.31729	0.001005	-0.27069	1.21623	0.271937	1.232107	-0.48956	0.511388	1.057734	0.55879	-0.43229
Q15906	VPS72	0.850955	-0.15486	0.083274	0.230461	0.251197	0.481539	-0.31806	0.364747	0.653318	0.393717	0.191759	-0.688	-0.28366	-0.39656	0.27061	0.284871	-0.56353	0.183508	-0.02179
Q15907	RAB11B	-0.08794	-0.35511	0.182408	-0.08254	0.451501	0.311556	0.174404	-0.11243	-0.15484	0.073338	-0.02051	0.383207	0.350416	0.526463	-0.13811	0.405856	-0.11519	0.280731	0.073965
Q15910	EZH2	0.002422	0.246488	-0.21583	0.142594	0.357481	0.498862	-0.57346	0.439592	-0.29299	0.10418	-0.12218	0.330384	0.603767	0.18101	0.380303	-0.02094	0.156551	0.62185	-0.26217
Q15911	ZFHX3	0.224982	-0.0086	-0.31038	0.071757	-0.47877	-0.33067	-0.29055	0.236971	-0.27122	-0.6767	0.352819	-0.54649	-0.11348	-0.85073	-0.33324	-0.20351	-0.99468	-0.19642	-0.73293
Q15942	ZYX	-0.78606	-0.34345	0.040792	0.159366	-0.63362	-0.35123	-0.723	-0.04969	0.826732	0.377951	-0.79719	-0.29972	0.736852	0.141398	-0.43721	0.080818	-0.44738	0.119707	1.028824
Q16134	ETFDH	-0.7794	0.398361	0.090144	-0.16601	0.414874	-0.08762	0.501568	-0.80314	-0.33248	-0.12626	0.785861	0.116995	-0.51487	0.32743	-0.17103	0.655219	0.200489	1.010636	-0.51383
Q16181	SEPT7	0.168048	-0.70391	0.53932	-0.92659	-0.40698	-0.27706	-0.75068	-0.05006	0.093556	-0.17366	0.019224	-0.93315	-0.22684	-0.57435	-0.06194	0.29359	-0.58285	0.365996	0.737976
Q16186	ADRM1	-0.19728	-0.13421	-0.19836	-0.31633	-1.20414	-0.03918	0.68174	0.445458	-0.19669	0.676019	0.232979	-0.27298	-0.41232	-0.55266	-0.00821	0.117206	-0.15579	-0.24728	0.258
Q16204	CCDC6	1.083819	-0.22181	0.208679	1.162513	0.066929	0.765574	-1.26901	1.066507	0.04951	-0.68739	-0.23739	-0.87088	-0.12181	-0.85238	-0.16168	1.485341	-0.12381	-0.7703	-0.37598
Q16222	UAP1	-0.46127	-0.74722	0.476964	-1.03773	0.055233	0.022203	0.175941	0.564377	1.097051	0.770351	-0.50808	0.358506	0.888023	0.072217	0.013353	0.1218	-0.08981	0.02253	0.898554
Q16254	E2F4	-0.05782	0.120643	0.124827	0.29936	0.273478	0.365392	-0.28096	-0.31564	-0.24805	-0.15462	0.594328	-0.79498	-0.10741	-0.34178	0.302734	0.521641	0.270696	0.641566	0.335018
Q16270	IGFBP7	-0.61404	-0.1023	0.122494	0.04228	-0.39639	0.317739	0.206702	-0.65064	0.007017	-0.07788	0.32721	-1.6051	0.023911	-0.66184	-0.33548	-0.18917	1.361324	2.578905	0.162654
Q16352	INA	-0.69012	0.641926	1.342269	-1.15433	-0.57156	-2.06805	0.296029	2.191803	0.320268	0.036432	-0.82936	-1.22237	0.756044	-0.71983	-1.11269	-0.22097	-1.00241	0.821467	2.846246
Q16363	LAMA4	-1.29943	0.641376	-0.46724	-1.82634	-0.36081	-0.73068	0.338571	-1.41035	0.5099	-0.27752	-0.96969	-1.49292	-0.17869	-1.11801	-0.94687	-0.25222	-0.84887	0.850001	0.01719
Q16394	EXT1	-0.24413	0.189536	-0.12942	-0.17718	0.074839	0.15203	0.23276	-0.02467	0.204011	0.871023	-0.63235	0.230621	0.558555	-0.09697	0.254215	0.720678	0.750404	1.479071	-0.07382
Q16401	PSMD5	0.664837	0.253433	-0.37402	-0.32301	-0.20544	0.045054	0.051671	0.285593	-0.82262	-0.65722	0.791015	-0.63125	-0.25794	-0.30727	-0.44156	-0.66016	-0.49734	-0.56608	0.37447
Q16512	PKN1	-0.70714	0.474683	0.672209	-0.08809	-0.03213	-0.23024	0.299603	1.812355	-0.17088	0.332967	-0.29758	0.36269	-0.30388	0.754213	1.086151	0.107746	0.128782	-0.08936	-0.29269
Q16513	PKN2	1.039773	0.131305	0.219374	0.040404	-0.08482	0.223625	-0.3646	-0.04122	0.140171	-0.11386	-0.03371	-0.51075	0.342321	-0.24186	-0.00433	0.484284	0.296302	0.333057	-0.29306
Q16527	CSRP2	-0.89467	-0.8296	-0.70607	-0.79294	-1.53144	-1.86928	-0.66647	-0.60266	0.638548	-1.2546	-1.40056	-2.03138	-0.22674	-0.71249	-0.44202	0.319352	-1.73439	-1.29001	0.223015
Q16531	DDB1	0.124969	-0.19832	-0.22124	-0.16184	-0.01856	-0.19028	-0.23353	-0.08	-0.42407	-0.40002	0.124344	-0.82971	0.031039	-0.83709	0.507805	0.162051	-0.97952	-0.53141	-0.18066
Q16537	PPP2R5E	0.27245	-0.32347	0.58038	-0.05246	-0.41042	-0.48214	-0.39126	0.100126	-1.03902	-0.15396	0.270767	-0.2508	0.480804	0.021557	0.750292	-0.37784	-0.14665	-0.10441	0.3278
Q16539	MAPK14	0.135874	0.597212	0.312246	-0.13948	0.000875	-0.15156	-0.00821	-0.49074	-0.79143	0.478421	-0.77112	0.419989	-0.03473	1.400485	-0.25204	-0.68019	0.498247	-0.16481	0.296715
Q16540	MRPL23	-0.56589	-0.00511	-0.10409	0.869991	0.31364	0.287223	-0.15071	-0.92514	-0.32558	-1.44109	0.790817	0.091714	0.122752	0.008497	0.96221	0.079666	0.158015	0.592606	-0.62669

Q16543	CDC37	-0.52036	-0.1875	-0.22152	-0.48388	-0.10486	0.069063	-0.8961	0.760421	-0.30502	0.389422	0.237564	-0.82534	0.119825	-0.55568	-0.8459	0.672452	0.282548	0.054568	0.040039
Q16555	DPYSL2	-0.21756	-0.4622	0.397109	-1.08109	0.400123	-0.52538	0.173416	0.266777	0.508139	0.490149	-0.55621	0.113769	0.424417	0.18539	0.025688	-0.29639	-0.47985	0.17121	0.980222
Q16576	RBBP7	0.945326	-0.69365	0.346561	0.643362	0.592959	0.60315	-1.10852	0.732629	0.244576	0.010493	0.157381	-0.405	0.219106	0.240754	0.210704	0.134501	0.326781	0.013805	-0.3403
Q16584	MAP3K11	-0.00989	0.334993	0.481568	-0.46008	0.918839	0.409478	0.062378	-0.68544	0.006687	0.409292	-0.34916	0.860344	0.125971	0.876571	0.281704	0.003447	0.467544	-0.05913	-0.58603
Q16585	SGCB	-1.43263	0.462306	0.003583	-0.2586	-0.34761	-1.42446	0.128269	-0.90631	0.143523	-0.79809	-0.53401	0.03564	-0.27288	-0.15987	-0.20376	-0.56187	-0.22366	0.489421	-0.1124
Q16595	FXN	0.741506	-0.38575	0.129043	0.016636	0.29429	1.04063	-0.47328	0.476836	-0.15411	-0.36894	1.101657	-0.96797	0.053125	-0.6299	0.512036	0.383822	-0.82666	0.241443	-0.3768
Q16610	ECM1	-2.11396	0.350285	-0.89241	-1.43248	-1.11741	-0.77149	-0.18646	-0.70371	0.334287	-1.62179	-1.32717	1.514264	1.056873	1.30065	-1.24287	-0.39876	1.732425	-0.97471	-0.46444
Q16611	BAK1	-0.73453	0.91698	1.035911	-0.01262	1.236003	1.777305	1.635928	0.47819	-0.11996	-0.31604	-0.06511	0.075868	0.104469	0.496873	0.881314	0.584432	1.055173	0.723018	0.19117
Q16625	OCLN	0.579598	-0.61118	-1.09449	0.178573	-1.25237	-0.43448	-0.25376	0.091571	-0.29697	-1.11381	1.117919	-0.3001	0.272631	-0.56868	1.792346	0.148804	-0.22251	-0.97581	-0.65585
Q16629	SRSF7	0.501171	-0.35162	-0.06926	0.313752	-0.04778	0.274112	-0.33342	0.603236	0.132649	-0.17735	0.678173	-0.39557	-0.12361	-0.43904	0.302405	0.086222	-0.15969	0.303196	-0.02245
Q16630	CPSF6	0.692788	-0.16637	-0.17046	0.065032	0.177968	0.420888	-0.7762	0.49962	-0.03524	0.037164	0.732427	-0.56152	-0.15673	-0.68675	0.084635	-0.23869	-0.14327	0.410999	-0.11211
Q16643	DBN1	-0.05153	-0.13789	-0.48425	0.359312	-0.38053	-0.20861	-0.70213	0.745416	0.550992	-0.14284	-0.71968	-0.6797	1.21295	-0.73223	-0.07711	-0.01317	-0.16353	0.481365	1.248049
Q16644	MAPKAPK	0.564333	0.268062	0.303046	-0.5089	0.190431	-0.24739	0.008481	-0.52785	-0.01196	0.878668	-0.19642	0.714125	-0.08068	1.467993	-0.08307	-0.95391	0.648062	-0.41264	-0.48793
Q16647	PTGIS	-1.85459	1.185631	1.506162	-1.22674	0.649711	0.363875	1.251859	-0.24496	1.034962	0.347668	-1.29763	0.247108	0.988629	-0.0524	-1.30266	-0.59087	-0.22766	1.105558	2.334419
Q16656	NRF1	0.376301	0.41063	-0.4034	-0.05982	-0.05474	0.349496	0.257907	0.565697	0.282929	-0.06782	-0.19603	-0.00983	0.225794	0.266843	-0.01794	-0.98114	0.202939	0.601767	0.067779
Q16658	FSCN1	-1.19611	-0.3829	0.644305	-0.90949	-0.47784	-0.42429	-0.46014	1.276696	0.774345	0.845664	-0.36705	-0.35265	0.558027	-0.50865	-0.70095	0.364717	0.425	0.13947	1.385359
Q16666	IFI16	0.510137	0.278053	0.847601	-0.28544	0.587285	0.689441	-0.49967	-0.39291	-0.14151	0.358685	0.505904	0.224459	0.157949	0.10532	0.193658	-0.38545	-0.80927	-0.259	-0.41504
Q16678	CYP1B1	-1.75251	1.395347	0.924671	-1.45674	-0.48877	0.357795	1.167381	-1.62908	0.34699	1.687088	-2.0265	-0.88	-0.92209	-0.62293	-1.5642	-0.9681	-1.49026	0.49677	1.998044
Q16698	DECR1	-0.90947	-0.53732	0.127846	-0.35435	1.621099	0.45251	0.125107	0.135263	0.232885	0.096409	0.035494	0.144177	-1.03956	1.260107	-0.61113	1.023187	0.726569	0.145384	-0.31636
Q16706	MAN2A1	-0.92053	0.744483	0.383075	-0.35515	0.437987	0.335946	0.909872	-0.44613	0.042067	-0.59519	-0.30465	0.130177	0.193413	0.046301	0.416993	1.009534	-0.01568	0.303086	0.247764
Q16718	NDUFA5	0.026401	0.009928	0.021414	-0.14843	0.333671	-0.24276	0.238564	-0.31665	0.483806	-0.46697	0.551307	0.318528	0.099006	0.102259	1.292093	-0.47378	-0.40229	-0.40079	-0.82801
Q16719	KYNU	-2.0729	1.644678	0.85083	-0.85647	0.843846	0.045223	-0.03118	-1.26302	-0.61702	1.630907	-2.86085	0.192394	0.005611	0.769787	-0.93316	-0.68878	0.178279	-0.51161	-1.48766
Q16740	CLPP	-0.11014	-0.26174	-0.00732	-0.42395	0.337526	0.437708	-0.38777	-0.43394	-0.00742	0.730476	0.466678	0.053757	-0.36334	0.144024	-0.09829	0.154	-0.19989	-0.18664	-0.58883
Q16762	TST	-0.09416	-1.34249	0.425572	0.781969	1.637168	0.875212	-0.95649	0.32934	0.160657	0.821257	1.053704	-0.33986	0.441459	-0.25168	-1.53898	1.537098	-0.44478	0.503816	0.02963
Q16763	UBE2S	0.44517	-0.10161	-0.27573	-0.30638	0.307256	1.366443	0.223502	0.716921	-0.18773	-0.24097	-0.7959	-0.70499	-0.07971	-0.15173	-0.26582	0.731142	0.047514	-0.75984	-0.12569
Q16774	GUK1	-0.26144	-0.42583	-1.03178	-0.72536	0.625522	-1.87853	-0.91093	-0.2407	2.16956	3.050849	-0.72436	0.324165	-0.31799	0.556603	-0.08769	-0.70266	0.912787	-0.1833	-0.02093
Q16775	HAGH	-0.06277	-1.20248	-0.26486	-0.07952	0.01461	-0.96782	-0.50793	-0.82485	0.991637	0.133026	0.343939	-0.51801	0.36178	0.298463	0.430484	0.428984	-0.52618	-0.06095	-0.65394
Q16787	LAMA3	-3.08675	-1.90655	-2.08036	-0.77963	-2.77295	-1.74317	-0.55556	-0.74038	-1.72988	-1.47697	1.410495	-1.37233	-1.18585	-0.68603	2.410854	-2.69563	-1.47517	-2.38366	-2.05565
Q16790	CA9	1.659802	0.108297	-0.54549	0.920952	-1.72817	-1.84987	0.72619	1.412698	-0.25787	-1.13117	0.552631	-0.49907	0.668311	-0.0789	-1.09258	-1.83292	-0.779	-1.27116	0.286516
Q16795	NDUFA9	0.174986	-0.43083	-0.18072	-0.44616	0.207786	0.161894	-0.17073	-0.32436	-0.42608	-0.75435	0.472107	0.260371	0.183486	0.194232	0.794516	0.144332	-0.58038	-0.31522	-0.37609
Q16798	ME3	-0.11879	-0.09709	-0.26206	-0.5295	-1.04774	-0.8944	-0.72694	-1.34674	-0.12999	-0.77835	0.512532	-0.81324	-0.04973	-0.06262	-1.23809	1.643731	-0.67714	-0.76451	-0.67549
Q16799	RTN1	-1.60786	0.56151	0.255667	-0.71045	0.697751	0.818363	0.064303	-1.42112	-0.43031	2.45448	-0.58411	0.500998	-0.71453	0.310297	0.177244	-0.21437	-0.03641	0.58603	-0.3523
Q16822	PCK2	0.396672	0.782364	0.204047	-0.15701	0.501233	0.413301	-0.29632	-0.39404	-0.24135	0.151788	-0.30758	0.453931	-0.05222	0.478086	0.024308	2.506532	-0.30118	-0.35442	-1.20864
Q16831	UPP1	-2.12645	1.387982	1.309886	-1.06661	0.372349	-0.17483	0.272255	-1.01215	-0.16312	1.514606	-1.57605	1.641935	0.024258	2.494525	-0.93353	-0.77336	1.068021	-0.39373	-1.5203
Q16832	DDR2	-1.19811	0.155468	-0.17712	-0.78044	-0.57924	-0.66085	-0.06346	-0.71284	0.542651	-0.24121	-1.7754	-0.32231	0.386962	-0.44131	-0.77827	-0.21344	-0.92218	-0.54466	1.006669
Q16836	HADH	1.430066	-1.04347	-0.19147	0.14546	0.59049	0.686187	-1.05363	-0.28885	-0.89979	-0.2293	1.293597	-0.1759	-0.0178	-0.67968	0.523502	-0.11194	-1.59803	-0.57406	-1.19861
Q16850	CYP51A1	-0.02108	-0.17736	-0.4488	-0.3211	0.437867	0.112091	-0.34343	-0.19536	-0.87791	-1.26984	0.474139	-0.17027	-0.25548	-1.04243	0.626204	-0.00043	-0.42445	-0.72841	-0.46712
Q16851	UGP2	-0.41465	0.17582	0.564289	-0.00037	0.019459	0.375563	-0.43149	0.262019	0.347636	0.139382	-0.29158	-0.40121	0.411832	-0.12502	-0.20181	-0.07925	-0.57363	-0.2504	0.935456
Q16853	AOC3	-1.93657	-0.61579	-0.99161	-1.71526	-1.40023	-3.01694	0.633323	-3.25716	-0.4499	-1.5879	-2.89583	-0.74624	-0.79512	-1.51807	-0.60178	-1.47084	-1.14425	0.825281	-2.07047
Q16854	DGUOK	0.433808	-0.27075	-0.06511	0.080185	0.436872	0.046478	0.060217	0.578706	0.147105	0.281268	-0.40166	-0.18801	-0.14241	-0.19814	0.182213	0.558014	-0.7139	-0.36104	0.543136
Q16864	ATP6V1F	-0.49226	0.370233	-0.18989	-0.52698	0.006375	0.345204	-0.07308	-0.07304	0.090036	0.125594	-0.15217	0.597349	0.235443	0.680756	0.079332	0.187528	0.263218	-0.18676	-0.61869
Q16881	TXNRD1	0.339919	-1.23186	0.336873	0.40778	0.173669	-0.13048	-0.87261	-0.14344	-0.60589	0.105394	-0.37201	0.246373	0.663436	0.515448	0.196684	-0.7117	0.007834	-0.48974	-0.17075
Q16890	TPD52L1	0.307024	-0.12048	0.0919	-0.07362	0.379394	-0.10778	-0.04869	-1.01459	-0.16425	0.077145	0.315575	-0.89178	-0.48795	-0.97583	1.304417	0.447579	1.741925	1.055706	-0.627
Q16891	IMMT	0.307474	0.207547	0.255944	0.421837	-0.21809	0.016979	0.238011	0.011666	-0.47676	-0.08753	0.574359	-0.29369	-0.17085	-0.38725	1.284022	0.938644	-0.99016	-0.6408	-0.24082
Q17RN3	FAM98C	-0.44436	0.171004	0.847572	-0.84498	-0.9375	-0.58314	0.44706	-0.0284	-1.01132	-0.81743	0.210255	-0.24469	-0.11859	0.266669	0.18209	-0.50308	-0.34239	-1.73894	-0.71783
Q1ED39	KNOP1	0.871535	-0.11829	-0.34593	0.250725	-0.93866	-0.86328	-0.67762	-0.58389	-0.32144	-0.07105	0.726701	-0.49681	-0.0147	-0.43066	-0.56261	0.316154	-0.1991	-0.57362	-0.71302
Q1KMD3	HNRNPUL	0.956612	-0.6569	0.350514	0.450609	-0.03363	-0.18464	-0.76753	0.422122	-0.14577	-0.14967	0.209894	0.204031	-0.08527	0.199686	0.531761	0.408113	0.176767	0.441929	-0.05882
Q24JP5	TMEM132	0.123387	0.204534	-0.28775	-0.56486	-1.04247	-0.67239	0.252747	0.650033	0.31833	0.285431	-0.16012	-0.62013	-0.15244	-0.38007	-1.04405	-0.13693	-0.24364	0.133473	-0.28079
Q27J81	INF2	-0.29831	0.218243	-0.33861	0.175799	0.372329	0.744122	0.007813	-0.09411	0.312002	-0.15785	0.306383	-0.2226	-0.22517	-0.38364	-1.14393	0.286432	0.077542	-0.22011	-0.82646
Q29RF7	PDS5A	0.962494	-0.14802	0.13858	-0.0408	-0.0154	0.151777	-0.01539	0.011787	-0.25533	-0.18524	-0.02835	-0.01728	-0.39682	0.036623	-0.0188	0.078542	0.128416	0.1637	-0.61806
Q2KHR3	QSER1	1.360412	-0.48615	-0.22364	-0.2355	0.55187	0.233922	0.368009	0.887809	0.093181	-0.83275	-0.08916	-0.68897	-0.29887	0.136553	-0.08982	0.295415	-0.10446	-0.28557	0.164558
Q2KHT3	CLEC16A	-0.21724	-0.28206	0.15133	-0.14846	-0.00582	-0.01338	0.27201	-0.3706	0.278212	-0.14798	-0.35904	0.31907	-0.47431	0.166224	-0.26291	-0.29361	0.183353	0.32811	-0.03881
Q2M1P5	KIF7	0.1943	0.049152	-0.16671	-0.40788	-0.8735	-0.37493	0.778859	-0.20494	0.158182	0.34473	-0.63687	-1.33085	-0.13168	-1.82591	-0.06421	-0.25473	-0.54706	-0.03775	0.558451
Q2M2I8	AAK1	-0.76949	0.596706	0.324438	-0.44773	-0.08777	-0.11982	-0.08924	0.195137	0.821334	0.261666	-0.74395	-0.06423	-0.0156	-0.30079	2.627105	0.216188	-0.25518	0.003462	0.096946
Q2M385	MPEG1	-1.45095	2.360125	0.500895	-0.88334	0.189714	-0.38081	0.849549	-1.16805	0.095893	0.344583	-0.74861	0.119481	-0.26513	-0.43921	-0.98973	-0.95051	-0.9156	0.04854	-1.46785
Q2M389	WASHC4	-0.39873	0.979518	0.695545	-0.11228	0.010629	-0.23173	0.128732	-0.30118	-1.08522	0.165754	-0.33093	0.422659	-0.00485	0.617071	-0.05118	-0.10828	0.162716	-0.06863	0.061871
Q2NL82	TSR1	0.84505	0.265049	0.108893	0.249591	-0.09507	-0.00766	-0.01246	0.085736	-1.02127	0.097375	-0.42044	-0.02695	-0.05453	-0.08317	-0.65626	-0.364	0.122667	-0.44128	-0.2147
Q2PPJ7	RALGAPA	-0.23504	0.409258	0.028549	0.764347	-0.83768	-0.66543	-0.31733	-0.1344	0.270871	0.416027	0.200498	-0.19152	0.057396	0.344747	0.197316	-1.0595	-0.41891	-0.58961	-0.33887
Q2PZI1	DPY19L1	0.970189	0.067385	0.173314	-0.42403	0.494094	0.005024	0.009751	-0.26752	0.064635	0.034246	-0.04102	-0.06413	0.412021	-0.11401	-0.81419	0.25322	0.270214	0.079907	0.819609
Q2TAA2	IAH1	-0.82095	0.44812	0.123758	-1.73649	0.312465	-0.66992	0.156342	0.515957	0.709731	-0.24233	-0.79973	-0.68659	-0.18074	-0.28412	0.513759	0.607496	-1.28237	0.262908	-0.57167
Q2TAA5	ALG11	-0.87271	0.613392	-0.39414	0.507484	0.115833	-0.42192	0.210641	0.155355	-0.00264	0.688168	0.061542	0.108051	0.027253	0.009226	0.518355	0.103283	-0.01317	0.217275	0.67087
Q2TAK8	MUM1	-0.53207	0.3352	0.017992	-0.67467	0.109654	0.531412	-0.33471	-1.61652	-0.03138	-0.61407	1.175566	-0.60232	-0.147	0.152877	-0.17349	-0.0714	-0.36346	0.027643	-0.24104
Q2TAL8	QRICH1	0.712234	-0.78062	0.253578	-0.20755	-0.38256	-0.2299	0.489621	0.447765	0.953356	-0.08592	0.400564	-0.49752	0.613118	0.0773	-0.25016	-0.32308	-0.14552	-0.16537	0.013123
Q2TAY7	SMU1	0.317021	-0.00913	0.431029	0.09813	0.120905	0.330821	-0.24694	0.221948	-0.22297	-0.14143	0.364405	-0.45622	-0.64634	-0.37524	-0.14028	0.012678	0.028547	0.312296	-0.19869
Q2TAZ0	ATG2A	-0.20163	0.454388	0.375361	-0.39583	0.718599	0.946028	0.34956	0.123441	0.002996	-0.48323	0.016128	0.467964	0.557708	0.338004	-0.43622	-0.06419	-0.15722	-0.04396	-0.62889
Q2TB10	ZNF800	-0.2818	-0.22264	-0.34963	0.467587	-0.42915	-0.0617	-0.54593	-0.15703	-0.47508	-0.4627	0.407695	-0.92542	-0.41983	-0.84009	0.864079	-0.98694	-0.78638	-0.25486	-0.8224
Q2TB90	HKDC1	-1.96858	-0.89846	-0.34755	-0.30881	0.801418	1.18661	-0.41092	1.581509	-0.30838	-0.70835	1.828187	-1.44409	-1.01652	-1.11996	3.222924	1.287696	-0.49064	-1.12116	-0.09976
Q2TBE0	CWF19L2	0.470019	-0.02216	0.077502	0.134789	-0.25346	-0.29373	-0.38498	0.205737	-0.16943	0.125845	-0.37633	-0.1777	0.618864	-0.24846	0.442801	0.417611	0.267669	0.301717	-0.02346
Q2VPB7	AP5B1	-0.53561	0.722659	-0.08756	-0.12457	-0.02769	-0.00531	0.810859	-0.19697	0.127456	0.173861	-0.42234	0.367094	0.274636	0.639886	-0.10883	-0.38715	0.059067	0.095405	-0.2085
Q2VPK5	CTU2	-0.12852	0.186053	-0.04096	-0.13875	-0.05819	0.439703	-0.44486	-0.59992	-0.10197	0.043784	0.555967	0.319994	0.019789	0.012291	-0.02322	-0.915	-0.36277	-0.63529	-0.07315
Q32MZ4	LRRFIP1	-0.25459	-0.15585	0.403689	-0.18484	-0.58584	-0.27506	-0.67548	0.046223	-0.50325	0.237811	0.658059	-0.318	0.330778	0.340225	-0.05874	0.090371	-0.01809	-0.23339	-0.37345
Q32NB8	PGS1	0.60259	0.317499	0.125977	0.277093	0.225822	-0.49119	0.108692	-0.85317	-0.12433	0.254197	-0.02643	0.445883	-0.11109	0.107538	-0.12083	0.131998	0.22837	0.764923	-0.43298
Q32P28	P3H1	-0.98049	1.345877	0.536544	-0.77164	0.632875	0.590879	0.214256	-0.13904	0.659247	0.448602	-1.13087	-0.8078	0.841229	-1.05493	-1.41718	0.325667	-0.57212	0.967314	2.156765
Q32P41	TRMT5	0.364333	0.139561	-0.37494	0.296672	-0.76392	-0.56954	-0.22022	0.150693	0.107484	-0.1735	1.003585	-0.34148	-0.16292	-0.13186	0.254954	-0.19962	-0.12146	-0.48675	-0.11684
Q32P44	EML3	0.437591	0.374629	0.479363	-0.77612	-0.19401	-0.48069	-0.05237	-0.84141	0.167755	0.403836	0.228366	-0.10524	0.442507	-0.12612	0.058478	0.300895	-0.5773	0.249694	-0.2897
Q3B726	TWISTNB	0.526394	0.136442	0.17465	-0.2649	0.654863	0.448229	-0.30319	0.057162	0.12142	0.393506	0.797142	0.087698	0.210792	0.397374	-0.48616	0.124941	0.424739	-0.11593	-0.29862
Q3B7J2	GFOD2	0.252861	-0.31623	-0.31963	-0.23793	-0.37091	-0.6835	-0.13592	-0.54237	-0.04364	-0.68769	1.198064	-0.47987	-0.78508	0.250838	0.7087	-0.50516	-0.18956	0.530571	0.706008
Q3B7T1	EDRF1	0.203875	0.114207	-0.06615	-0.00886	0.396798	0.99305	-0.03921	0.211676	-0.55337	-0.28604	0.177581	0.203984	-0.14919	-0.25347	-0.19009	0.047193	0.10975	0.146519	0.001721
Q3KQU3	MAP7D1	-0.34966	-0.19009	0.498346	0.494583	-1.09704	-0.05837	0.034914	0.386621	0.88256	0.050368	-0.6891	-0.99838	0.354274	-0.44056	-0.38754	-0.17382	-0.81025	0.148171	0.471929
Q3KQV9	UAP1L1	-0.56188	-0.04178	-0.07519	-1.1663	1.085286	-0.22417	0.343987	1.263113	0.97227	0.47684	-0.82482	1.280943	0.344684	0.433816	-0.60263	-0.28417	0.397826	0.707665	0.342269
Q3KQZ1	SLC25A35	0.548152	-0.3294	-0.25932	0.330825	1.064889	0.730613	-0.64946	0.079939	-0.32614	0.094867	-0.05496	0.713147	-0.15462	0.384656	0.340217	0.688238	-0.38202	-0.39778	-0.77845
Q3LXA3	TKFC	0.968792	-0.34606	-0.65603	0.512726	-0.37071	-0.37107	-0.33676	0.305919	0.189926	-0.28581	0.493001	-0.75697	-0.69005	-0.27959	-0.25819	0.664779	-0.94364	-1.14253	-0.57547
Q3MHD2	LSM12	-0.13323	0.139635	-0.37833	-0.05954	0.306662	0.141869	-0.86019	0.481144	-0.79181	0.241793	-0.04916	0.460907	1.353676	0.395171	0.320958	0.870787	0.579662	0.429645	0.182148
Q3MIP1	ITPRIPL2	0.643553	-0.4249	-0.30066	0.121475	-0.55712	-0.54497	-0.51476	-0.71734	-0.45374	-0.44991	1.010348	-0.42179	-0.38248	-0.19959	0.78211	-0.13018	-1.10633	-0.32185	-0.79985
Q3MIX3	ADCK5	0.13328	0.13177	-0.17179	0.22974	-0.50165	-0.51927	0.600757	0.123977	-0.12973	-0.11463	-0.33403	1.052045	-0.05646	0.370981	-0.15045	-0.07046	-0.17313	-0.4275	-1.09455
Q3SXM5	HSDL1	-0.9138	0.680257	-0.45925	-0.34518	-0.47902	-0.69513	0.821529	0.328016	0.078849	0.366264	0.77073	-0.14071	-0.8309	-0.21001	-0.24168	0.090106	-0.24783	-0.7885	0.528679
Q3SY69	ALDH1L2	-1.84888	-0.4815	-0.50917	-0.81643	-0.69534	-0.76248	0.701953	-0.15968	0.450007	0.265905	-1.80586	-0.7755	0.864608	-1.05705	-0.86022	-0.26186	-0.86675	0.320143	0.943216
Q3SY84	KRT71	-0.29317	3.281079	0.492842	-1.23405	-1.16215	-0.45189	1.90581	0.710181	0.040847	-0.04712	-0.07916	-0.71914	0.538299	0.158326	-0.41094	-0.01099	-0.2842	-0.52154	0.427999
Q3SYG4	BBS9	-0.25832	0.164215	0.087966	-0.23096	-0.11089	-0.14807	0.31568	-0.32685	0.435539	0.133714	-0.06055	-0.52182	0.229497	-0.35856	-0.12741	0.484083	-1.05247	0.062166	0.935999
Q3T906	GNPTAB	-0.62971	0.484495	0.08471	-0.03384	0.161416	0.085904	-0.23944	-0.23492	-0.01415	0.627145	-0.6542	0.278528	0.40731	0.046769	-0.2479	-0.22651	0.402748	0.236075	-0.36723
Q3V6T2	CCDC88A	-1.33119	0.659784	0.78468	-0.88294	0.004366	-0.25338	0.228153	-0.70504	0.601713	0.926252	-0.97219	0.517696	0.214683	0.871936	-0.56362	-0.3686	-0.08083	-0.03647	-0.04091
Q3YEC7	RABL6	0.211911	0.056747	-0.35148	-0.50721	-0.11648	-0.06911	-0.23185	0.106647	-0.53573	-0.29204	0.095408	-0.14191	0.215573	0.23481	0.066208	-0.05788	0.218426	0.141613	0.370366
Q3ZAQ7	VMA21	0.640778	0.52971	0.353008	0.179493	0.712091	0.089511	0.300816	-1.04579	-0.15807	-0.11995	0.020264	0.242267	0.154392	0.152515	0.304301	-0.07464	0.470182	-0.11189	-0.40146
Q3ZCQ8	TIMM50	-0.16682	-0.28403	0.890492	0.558943	-0.27747	0.218797	1.831065	3.506418	0.298047	-0.47607	-0.58226	-0.51344	-0.41881	-0.16355	0.738929	-0.02718	-0.59144	-0.18437	1.287192
Q460N5	PARP14	0.406493	0.870461	1.396088	-0.58481	0.800819	1.187396	0.39019	1.21687	-0.05251	0.731443	-0.50058	0.186402	0.353744	0.394505	-1.09515	-0.21774	0.026243	0.107195	-0.64424
Q495W5	FUT11	0.093813	0.237468	0.22339	0.13352	0.38653	-0.13661	0.001118	0.535208	0.006431	0.092545	-1.42134	-0.14383	0.149712	0.187855	-0.04974	0.408508	-0.02302	0.417193	0.873664
Q49A26	GLYR1	0.882517	-0.30123	-0.2152	-0.25974	-0.48661	-0.11589	0.290748	0.1353	0.292247	-0.58736	0.596916	-0.59733	-0.79541	0.015785	0.197771	0.055812	-0.05047	0.522058	-0.56675
Q49MG5	MAP9	0.491773	-0.32807	-0.50801	-1.49844	-0.57724	-0.51095	-0.85351	-0.6929	-0.07222	-0.14925	1.550686	-0.96185	-0.17584	-0.59905	-0.0227	0.22738	-0.11452	0.108278	-0.55931
Q4ADV7	RIC1	-0.63987	0.615338	0.149067	0.058266	0.224847	-0.06837	0.194789	-0.29696	0.285312	0.251685	-1.12485	0.889997	0.176876	0.108547	-0.87122	-0.01087	-0.23118	-0.76624	0.650893
Q4G0F5	VPS26B	-0.11581	0.53696	0.409236	0.120683	0.137167	-0.45116	0.497212	-0.17498	0.576112	-0.33665	0.242373	-0.14991	0.351303	0.220929	0.089658	-0.06849	-0.66761	-0.3476	0.133947
Q4G0I0	CCSMST1	0.177608	0.350666	-0.18165	0.311594	-0.73495	-0.27317	-0.4002	0.17461	-0.29301	-0.1895	0.719875	-0.82647	0.19104	-0.48372	0.13833	0.254526	-0.33128	-0.40356	-0.62991
Q4G0J3	LARP7	0.112616	-0.44136	-0.51573	0.155178	-0.297	0.111674	-0.40496	1.255393	1.251346	-0.02198	0.279302	-0.91019	-0.29577	-0.36047	1.146268	0.05085	-0.39642	-0.36717	-0.14846
Q4G0N4	NADK2	0.227724	-0.46787	-0.41414	0.101047	1.034651	0.463555	-0.59786	-0.11024	0.411598	1.135117	-0.16992	0.048127	0.054621	0.184126	0.599673	0.370611	-0.14215	0.43783	0.668669
Q4G0X4	KCTD21	-0.15375	0.421293	-0.1344	-1.13111	0.215049	-0.79657	0.365222	-0.47133	0.162518	0.202789	-0.1382	0.71847	-0.03625	0.132015	-0.40891	-0.32936	-0.34134	0.073522	-0.02865
Q4G176	ACSF3	-0.61338	0.357833	-0.16701	0.316395	0.4081	0.240199	-0.08003	-0.29009	0.783724	0.390472	0.792316	-0.40673	-0.53085	-0.04267	0.75362	0.514151	-0.59975	-0.13558	-0.13981
Q4J6C6	PREPL	-0.0023	-0.70776	0.127097	1.038052	0.525271	0.793205	-1.30989	0.21956	-0.47038	0.441526	0.42729	-1.11125	-0.06626	-0.47488	0.863276	0.341138	-0.1937	-0.03394	0.415339
Q4KMP7	TBC1D10B	0.232633	0.288884	0.09007	-0.12337	0.018216	0.225091	-0.41469	-0.49437	-0.15248	-0.26476	-0.10068	-0.20928	-0.32818	0.057841	-0.24118	0.064981	-0.21134	0.063384	0.168559
Q4KMQ1	TPRN	0.259693	0.001593	0.296387	-0.56074	-0.44562	-0.37909	-0.25546	0.292039	0.453502	-0.59633	-0.36262	-0.35539	0.09327	-0.40318	-0.01034	-0.50932	0.020473	-0.15984	-0.33737
Q4KMQ2	ANO6	-0.64426	0.825766	-0.22486	-0.02689	-0.539	-0.96841	0.252323	-0.6093	0.944158	-0.17072	-0.40338	0.269533	-0.31717	-0.05072	-0.62793	-0.54849	-0.41839	0.084201	0.366465
Q4L180	FILIP1L	-1.41212	0.516174	0.109854	-1.08412	-1.01359	-0.08315	-0.67573	-0.69249	1.071576	-0.59036	-0.75991	-1.66687	0.986034	-1.55478	-1.12987	0.360725	-1.49908	-0.37805	1.955408
Q4LDE5	SVEP1	-1.7007	0.467613	0.193911	-1.43719	-0.41141	-0.65798	0.367955	-1.20064	0.546048	0.174676	-2.49254	-0.42803	0.643317	-0.28822	-0.7434	-0.05006	-0.48768	0.608928	1.823433
Q4LE39	ARID4B	0.424639	-1.08503	-0.29009	-0.37456	-0.27051	0.2188	-0.42757	0.903613	-0.05596	-0.18743	0.189501	-0.23732	-0.38328	0.177899	0.459117	0.192312	-0.04744	0.124337	-0.07005
Q4V328	GRIPAP1	-0.20464	0.032626	-0.00334	0.124637	-0.28202	-0.33641	-0.55938	-0.41725	-0.04378	0.676735	-0.14185	-0.1899	-0.10467	0.243855	-0.62401	0.107119	-0.28807	-0.52138	-0.81586
Q4V9L6	TMEM119	-2.14787	-0.42408	-0.59704	-2.29373	-0.8352	-1.27622	0.534489	-1.5563	0.632508	0.46005	-2.38904	-3.02451	-2.65933	-1.49096	0.495768	-1.03807	-2.09822	-0.12186	-0.25223
Q4VCS5	AMOT	-0.10071	-0.04542	0.206577	-0.62761	-1.30212	-0.50053	-0.09339	0.581385	0.432974	0.317113	-0.64928	-1.17113	-0.02627	-0.67148	-0.70774	0.385333	-0.26959	0.075619	0.174335
Q4ZHG4	FNDC1	-1.11532	0.861904	0.343602	-0.30601	-0.76724	0.173298	0.073499	-0.88472	-0.08594	-1.54273	-0.07371	-0.02597	-0.10279	-0.34404	-0.28245	-0.09394	-0.4985	-1.04347	-0.63442
Q4ZIN3	TMEM259	0.198154	-0.02565	0.208549	-0.28825	0.238448	0.491939	-0.33468	-0.54874	-0.02948	0.777487	-0.06768	-0.35051	0.27045	-0.35173	0.283134	-0.31259	-0.497	-0.27103	-0.57755
Q504Q3	PAN2	-0.14078	0.27564	-0.21747	0.31075	0.315427	0.214151	-0.35265	0.395096	-0.10943	-0.06931	0.155215	0.184684	0.387571	0.091071	0.273873	0.283753	0.239239	0.841377	0.135774
Q52LJ0	FAM98B	-0.06792	-0.04972	-0.77259	0.292007	-0.60017	-0.28206	-0.9402	-0.17763	-0.22702	-0.34127	0.300016	-0.40963	-0.22034	-0.50133	0.735856	0.197466	0.103055	0.145843	0.222266
Q52LW3	ARHGAP2	0.547284	0.197791	0.207394	0.014951	0.108451	0.894486	-0.4893	0.307786	-0.38324	-0.67165	0.218701	-0.28259	1.620298	0.00313	0.420908	0.402691	-0.13893	-0.71794	-0.30406
Q53EL6	PDCD4	0.166125	0.162984	0.044924	-0.01468	-0.51527	-0.66478	-0.6034	-0.32592	-0.80166	0.037095	0.821031	-0.65575	-0.32305	-0.66283	0.542656	-0.52224	-0.73591	-0.42826	-0.72366
Q53EP0	FNDC3B	-0.2428	0.366473	-0.07161	-0.63899	0.030968	0.502384	1.161952	0.008104	0.207369	-0.04801	-0.50326	0.198367	0.619362	0.409438	-0.5248	0.020351	0.446654	0.829307	1.117414
Q53ET0	CRTC2	-0.24333	0.110488	-0.04522	-0.62631	-0.58888	-0.76479	0.250777	-0.19117	-0.19304	0.209093	0.078211	-0.29744	-0.05537	-0.08899	0.661763	0.345704	-0.62777	-0.08364	-0.46408

Q53EU6	GPAT3	-0.07869	0.274166	0.403541	-0.22358	0.112337	0.414599	0.323338	-0.40025	0.208102	0.434439	-0.0704	0.378193	0.121829	1.724985	-0.19065	0.072117	1.648131	0.85881	0.482747
Q53F19	NCBP3	1.432946	0.420886	0.358102	-0.19144	0.120321	0.43604	-0.74776	0.669026	-0.17832	-0.25161	0.508736	-0.48509	0.232595	-0.23776	-0.48113	-0.14948	-0.05805	0.509333	0.251148
Q53FA7	TP53I3	0.085119	-0.4866	-0.36417	-0.30757	1.047753	-0.06477	-0.82201	1.186361	0.297775	-0.03433	-0.30176	-0.9912	0.171442	-0.40131	-0.38755	1.361946	-1.23982	-0.61971	1.330734
Q53FT3	HIKESHI	-0.88933	-0.30629	0.155292	-1.25681	1.60803	0.329417	-0.03328	-0.29479	0.521215	-0.09584	-0.07565	-0.19594	0.674065	0.127578	-0.379	0.526053	-0.06014	0.293035	-0.58087
Q53G44	IFI44L	2.775135	2.112765	0.506654	-0.4592	-0.66601	-0.9032	1.057091	-2.09952	-0.52673	-0.05344	-0.28436	2.045249	0.15305	-0.54094	-0.39176	-0.42015	-0.75488	-1.08372	-2.36537
Q53GG5	PDLIM3	-1.66532	-1.06753	0.21622	-1.19381	-1.76267	-0.53621	0.458104	-0.94756	1.508905	-1.13985	-1.63095	-1.84791	2.154425	-1.03771	-0.75169	0.149343	-1.66311	-0.05614	2.446478
Q53GL0	PLEKHO1	-0.40081	0.125878	-0.07514	0.02665	-0.20541	-0.03348	0.186965	-0.47001	-0.79683	0.225109	-0.37586	0.246424	0.453728	0.463836	0.120287	-0.08088	0.471848	0.644482	-1.3069
Q53GL7	PARP10	0.39969	0.50258	-0.24255	-0.28106	-0.24477	0.206597	0.081633	-0.10534	0.183448	0.064118	-0.59317	0.266477	0.400228	0.36362	-0.53938	0.342277	0.222115	-0.19678	-1.25887
Q53GQ0	HSD17B1:	0.328329	0.120325	0.338259	0.716527	0.046147	-0.23369	0.548673	-0.18631	-0.42079	-0.12828	0.513798	0.406676	-0.57594	0.220061	0.121407	0.380189	0.136175	-0.40953	0.011811
Q53GS7	GLE1	0.029059	0.024528	-0.00129	-0.17611	0.040447	-0.13621	-0.05553	0.081731	-0.33386	-0.0204	0.06887	-0.12381	0.04911	-0.13753	0.654211	-0.11615	-0.41061	-0.46911	-0.21652
Q53GS9	USP39	0.540534	0.120623	0.437632	0.335049	0.167948	0.123963	-0.8317	0.654305	0.089223	-0.08534	0.170305	-0.09753	0.021001	-0.19762	0.122654	-0.12143	-0.28942	-0.1918	0.01774
Q53GT1	KLHL22	-0.14293	-0.06709	-0.15318	-0.05971	-0.31821	-0.51995	-0.32778	-0.58245	-0.22559	-0.35411	-1.3578	-0.13106	-0.12959	0.216201	0.013956	-0.42928	-0.22104	-0.58451	0.110394
Q53H12	AGK	0.289629	0.083497	0.009845	1.398283	-0.47284	-0.47431	0.206788	0.329181	-0.1484	0.238114	0.546402	-0.38894	-0.26162	-0.45476	1.022011	0.390156	-0.5184	-0.82714	-0.38997
Q53H47	SETMAR	0.494365	-0.37409	0.039208	-0.34511	-0.60584	0.292046	-0.22325	-0.10091	-0.23059	-0.49938	0.10631	-0.90998	-0.5029	-0.23336	-0.00048	-0.30598	-0.57191	-0.2703	0.041197
Q53H82	LACTB2	0.101959	0.224144	0.119038	-0.4175	1.320378	0.117926	0.19905	0.066017	0.464636	0.867369	-0.46067	1.407792	0.314226	-0.0429	-0.17906	2.098386	-0.29647	-0.63245	-0.86911
Q53H96	PYCR3	0.866599	-0.38324	-2.09292	-0.38913	-0.08282	0.043274	-1.04801	-0.97343	1.106252	-0.37509	0.252188	0.080192	0.528173	-0.56416	-0.65243	0.783644	-0.31025	-0.61633	-0.61804
Q53HC5	KLHL26	0.261967	0.086014	0.057167	-0.07983	-0.23234	-0.31149	0.082784	-0.45754	-0.53445	0.062016	0.598069	0.158846	-0.10639	0.365076	-0.20254	-0.17025	-0.27941	0.313914	-0.48671
Q53HC9	EIPR1	0.002088	-0.09624	-0.12668	-0.1158	-0.59548	-0.59276	-0.8428	-0.02505	-0.20952	0.002328	-0.24709	-0.18305	0.065866	-0.34151	0.346255	0.05985	-0.61218	-0.49075	0.053164
Q53HL2	CDCA8	0.870919	-0.30163	-0.17695	-0.12246	1.116572	1.481328	-0.33021	0.731154	-0.18965	-0.12728	-0.39259	-0.17442	0.234192	-0.02484	-1.08815	-0.1116	0.096146	0.251271	0.51222
Q53HV7	SMUG1	0.71677	0.175545	-0.38294	-0.46007	0.238331	-0.38718	0.016697	-0.61658	0.040847	-1.10317	0.612411	0.470514	0.876653	-0.29151	-0.63477	-1.14506	0.376483	-1.22264	0.512461
Q53LP3	SOWAHC	0.249385	0.115675	-0.18192	-1.14015	0.197066	-0.44287	0.062084	-0.31816	-0.06519	0.015394	-0.18087	-0.91134	-0.25799	-0.24733	0.136828	0.463098	0.337326	-0.58482	-0.27911
Q53QZ3	ARHGAP1	-1.66696	1.250238	0.721993	-0.71125	0.455755	0.209069	0.321133	-1.15785	-0.03637	1.256829	-1.16564	0.87277	-0.18229	1.652142	-0.48718	-0.59755	0.071041	0.812804	-1.30177
Q53R41	FASTKD1	0.760093	-0.21072	-0.02005	-0.5953	0.101625	-0.34787	-0.16345	0.051965	-0.19302	0.173631	0.238316	-0.10387	-0.11944	0.193235	1.481224	0.17707	0.510036	0.01417	0.364748
Q53SF7	COBLL1	0.105036	0.163952	-0.1313	-1.27691	-0.92774	-0.9789	-0.15119	-0.98032	0.297646	-0.25157	-0.57183	-0.53836	0.020559	-0.74665	-0.28377	-0.37706	0.893228	2.028234	0.625504
Q53T59	HS1BP3	-0.39416	0.273906	-0.6242	-0.7356	-0.10587	-0.35025	0.557109	-0.24664	-0.23907	0.894964	-0.21118	0.203183	-0.10564	0.622214	-0.26382	0.033965	-0.16599	-0.22135	0.05124
Q53TN4	CYBRD1	0.48459	-0.2763	-0.00589	-1.47279	-0.41471	-1.58734	0.485381	-0.70707	-0.14336	-0.51539	-0.0278	-0.82821	-1.18907	-1.61811	1.054133	-0.18183	-2.02374	-0.58772	-0.18475
Q562E7	WDR81	0.308267	0.564645	0.344988	-0.59583	0.08101	-0.07518	0.737567	-0.60409	-0.18378	0.579952	0.120919	0.117683	-0.18715	-0.17646	-0.16666	-0.35489	-0.78244	-0.01552	-0.20168
Q562R1	ACTBL2	-0.72734	-0.28587	-0.05412	-0.28203	0.124319	-0.25884	-0.54282	-1.96666	0.574753	0.442346	-0.65228	-0.15552	0.37291	0.720742	-0.15327	0.225611	-0.12625	0.240477	0.435031
Q567U6	CCDC93	-0.09457	0.763699	0.518379	-0.38015	-0.20857	-0.39878	-0.04812	0.024551	-0.32321	0.591862	-0.0441	0.19134	-0.11196	0.298202	-0.31956	0.113204	-0.21457	-0.21469	-0.04013
Q56VL3	OCIAD2	0.159201	0.172557	1.254864	1.40361	-0.86224	-0.83643	0.343421	0.563678	0.536704	-0.3573	0.277433	-1.13731	0.335308	-0.58103	-0.42695	0.722355	-0.70154	-0.77953	-0.3083
Q587I9	SFT2D3	1.424148	0.293629	-1.31333	-1.20375	-0.81738	-0.1754	0.942614	-0.52467	0.040847	0.276097	0.029991	-0.56116	-0.00582	-0.18563	-0.54867	-0.86421	-0.09478	0.143518	-0.44509
Q58A45	PAN3	-0.11837	0.255033	0.038024	-0.22901	0.284661	0.428346	0.096791	0.519831	0.242132	0.739544	-0.35841	-0.32079	0.129667	-0.00526	0.209466	0.233649	0.527184	-0.16617	0.084173
Q58DX5	NAALADL:	1.294529	-0.20145	-0.31191	0.183248	-0.35999	-0.74919	-0.39547	-0.47778	0.286752	-1.12676	0.818434	-0.68597	-0.66943	-0.72031	-0.00778	-0.81435	-0.18848	-0.30758	0.019341
Q5BJD5	TMEM41B	0.078623	-0.00153	0.581036	-0.82834	1.140144	0.859037	0.396544	0.325308	0.068658	-0.29036	-0.39079	0.145801	-0.15121	-0.3276	0.040474	-0.80001	1.214694	0.711723	-0.11409
Q5BJF2	TMEM97	0.576468	-0.86012	-0.38767	0.260356	0.378142	-0.08057	-0.17074	0.396049	-0.98969	-0.01169	0.144984	-0.06885	-1.76809	-0.17259	-0.48506	-0.02894	-0.21269	-0.08977	-0.35316
Q5BJF6	ODF2	0.085805	-0.03667	-0.24951	-0.24409	-0.72601	-0.92277	0.71092	-0.06415	0.497715	-1.2383	1.998901	-0.58139	-1.81575	-0.25285	-0.71832	-1.15416	-0.60749	0.125214	-0.03922
Q5BJH2	TMEM128	0.812174	0.260163	0.06116	-0.59025	0.288174	-0.03281	0.221468	-0.48038	0.334253	-0.06577	0.132848	-0.64263	-0.00444	0.063304	0.065195	0.080276	-0.17124	0.740951	0.563471
Q5BJH7	YIF1B	-0.73943	-0.04052	-0.14458	0.445082	-0.44724	0.509331	-0.38774	-0.61945	0.06961	0.190804	-0.56558	-0.74802	0.150544	-0.21926	0.587115	-0.55607	-0.91763	-0.7117	-0.05769
Q5BKY9	FAM133B	0.772499	-0.48855	-0.2701	0.28426	-0.79419	0.334722	-0.61144	0.580082	-0.21958	-0.26879	-0.00303	-0.9345	-0.15648	-0.42485	0.679202	-0.08787	-0.55992	-0.12343	-0.02897
Q5BKZ1	ZNF326	0.789996	0.376476	-0.03355	0.143569	-0.16086	0.352896	0.391037	1.071359	0.467617	-0.23233	1.255413	-1.58561	-0.53158	-1.04043	-0.24527	-1.12294	-0.37144	0.47417	-0.09328
Q5C9Z4	NOM1	-0.06324	0.013814	-0.60829	1.884452	-0.23469	0.538552	-0.33012	0.283619	-0.45075	0.061558	0.197958	-0.70335	-0.2151	-0.65624	-0.29035	-0.5746	-0.77402	-0.84217	-0.23105
Q5D862	FLG2	-1.6028	1.857733	-0.40841	-2.09763	-2.23991	-0.97812	1.553865	-1.01768	1.022613	-1.67406	-0.35857	-1.66512	-0.13259	-0.27026	-0.88078	-1.28573	-1.40402	-0.82322	-0.64709
Q5EB52	MEST	-0.10947	-0.27305	-0.00792	-0.32792	-0.20176	-1.07246	0.943148	0.252292	0.823778	-1.10526	-0.73244	-0.03454	-0.31661	-0.06776	-0.00221	-1.38674	-1.27999	-0.39504	0.750272
Q5EBL4	RILPL1	-0.89561	0.119767	0.291714	-0.39066	-0.22921	-0.03757	0.137696	0.079104	0.105818	0.243618	-0.08253	-0.36061	0.323458	-0.14115	-0.4546	-0.78849	-0.41875	0.31205	0.736023
Q5EBL8	PDZD11	0.284412	-0.67613	-0.16754	-0.26796	0.194813	0.013246	-0.49723	0.170348	0.263414	0.284572	0.238716	-0.14835	-0.28467	0.017424	-0.16257	0.825612	0.146267	0.075274	-0.45149
Q5EBM0	CMPK2	0.390749	1.289154	2.537029	-0.92421	0.015996	-0.20706	0.428774	0.219347	0.084856	-0.49396	-1.35452	0.510834	0.44128	0.630667	-0.31118	-1.3193	-0.44533	-0.7243	-1.31085
Q5F1R6	DNAJC21	0.1647	-0.49215	-0.13773	-0.86408	0.023758	0.111152	0.142802	-0.49109	-0.27753	0.210811	0.392673	-0.71965	-0.90884	-0.57808	-0.22782	0.56477	0.052256	-0.01657	-0.10371
Q5GFL6	VWA2	-0.42318	0.43383	-0.04567	-0.46215	-0.64552	-0.72825	0.398841	0.566638	0.408553	-0.13968	-0.05475	-0.31607	-0.06876	0.158747	-0.42311	-0.05679	-0.49608	-0.41019	0.251563
Q5GLZ8	HERC4	-0.45445	0.360561	-0.29739	-0.35488	0.182839	0.344428	-0.3423	-0.36567	0.454246	0.383488	0.194872	-0.16675	0.090046	0.266614	0.352795	-0.60317	-0.07221	-0.48852	-0.23885
Q5H8A4	PIGG	0.575414	0.059231	0.077254	-0.39466	-0.1214	-0.43798	0.187265	0.051979	0.314788	-0.58389	0.146412	0.148208	-0.04655	0.171912	-0.47285	0.219597	0.086739	-0.15706	-0.10091
Q5H9R7	PPP6R3	0.478865	-0.06864	0.494874	-0.11111	-0.16077	0.491315	0.047791	-0.05014	-0.57331	-0.3661	-0.06909	-0.01277	0.731875	0.27779	-0.3173	0.789089	0.313838	-0.27653	-0.16829
Q5H9U9	DDX60L	0.304829	0.444464	0.553178	0.541844	0.015648	-0.18509	0.258619	0.41088	-0.14474	-0.49727	0.009888	0.589965	-0.12013	0.711142	-0.58008	-0.02561	0.220653	0.086266	-1.07797
Q5HYA8	TMEM67	-0.68092	0.529906	0.175388	-0.27866	-0.21474	0.206552	0.18371	0.017875	0.269688	0.23151	-0.11836	0.228809	0.300715	-0.39093	0.327265	-0.34938	0.266054	0.095023	0.470732
Q5HYI7	MTX3	-0.57296	-0.0908	-0.92315	0.393762	-1.18858	-0.92821	-0.15397	-1.24003	-0.04365	-0.35722	1.064591	-0.28728	-0.88117	-0.40184	1.232512	0.970313	-0.80921	-0.47445	-0.28181
Q5HYI8	RABL3	0.089026	-0.29066	0.007068	-0.08639	0.371003	-0.06653	-0.75745	0.763781	-0.1865	0.365808	0.016603	0.299351	0.21669	0.034777	1.28654	0.025067	0.255172	-0.0197	-0.04858
Q5HYJ3	FAM76B	0.569017	-0.13511	0.438852	-0.22376	-0.27832	-0.59132	-0.29047	-0.01711	0.439692	-0.80795	-0.3862	0.057925	-0.05489	0.379863	0.072903	0.027061	-0.29792	0.414618	-1.22501
Q5HYK3	COQ5	0.177264	0.001947	-0.06139	0.060689	0.016209	-0.29102	0.333091	-0.08966	0.199391	0.274895	0.662346	-0.41001	-0.08073	-0.14318	1.092147	0.457788	-0.06906	-0.42982	-0.30377
Q5HYK7	SH3D19	-1.18773	0.620164	-0.37929	-1.37985	-0.14886	-0.68865	0.195664	-0.70254	-0.30692	0.321156	-0.48846	-1.00936	0.376907	-0.81764	-0.44385	-0.29079	-0.84131	1.127936	1.460875
Q5IJ48	CRB2	1.372334	-0.21515	-0.23078	-0.12924	1.05709	0.746695	0.237111	0.791472	-0.14596	-0.47852	0.299324	-0.45734	0.11215	-0.50641	-1.93634	-1.05386	-0.84995	-1.38904	1.039775
Q5J8M3	EMC4	-0.09659	0.866957	-0.13254	0.073644	0.359184	0.391118	0.334555	-0.14812	0.153864	-0.21905	0.159141	0.16977	0.420777	-0.0103	0.337724	0.336037	0.040318	-0.61538	0.36262
Q5JPH6	EARS2	0.123876	-0.14165	-0.08611	1.08931	-0.63757	-0.63969	0.048709	-0.50287	-0.13871	-0.85372	-0.32661	-0.58807	-0.01774	-0.72622	1.146862	0.032007	-0.13175	-0.09088	0.139743
Q5JPI3	C3orf38	0.301588	-0.17175	0.16127	0.150074	-0.01101	0.48469	-0.31875	0.277791	0.034529	0.23301	0.865161	0.116238	0.144107	0.280209	0.434079	0.577814	0.184225	0.763483	0.130204
Q5JRA6	MIA3	-0.62669	0.471354	-0.14365	0.175012	-0.40209	-0.42446	0.702003	0.209606	0.194402	0.269205	0.210772	-0.5461	0.130594	-0.28345	0.523598	0.237526	-0.329	0.450634	0.490042
Q5JRX3	PITRM1	0.638447	-0.81225	0.136017	0.75549	1.351952	0.692312	-1.07063	0.182784	0.349633	1.562262	0.066683	0.614838	1.175904	-0.29736	1.262799	1.497439	0.294701	1.077909	0.601962
Q5JSH3	WDR44	-0.17189	-0.16765	0.097445	-0.26318	0.530584	0.451799	-0.48957	-0.22039	0.068966	0.871626	-0.21383	-0.12583	0.507446	-0.13473	0.17764	-0.42858	-0.26791	0.30872	0.454082
Q5JSJ4	INTS6L	0.316372	-0.07689	-0.09385	-0.24556	0.017082	-0.75846	0.046007	1.041586	0.324653	-0.33447	0.604968	0.177347	0.145302	0.002466	-1.09869	-0.23089	0.227825	-0.09364	0.317184
Q5JSL3	DOCK11	-1.69353	1.10306	0.715083	-1.19488	0.485468	-0.01526	0.553311	-0.71263	0.657614	0.548308	-1.47255	0.539111	0.166496	0.824061	-0.05284	-0.37525	-0.0357	0.871888	1.50165
Q5JSP0	FGD3	0.363745	0.690687	0.663792	-0.91227	0.434554	0.462038	-0.11586	-1.2716	-0.40734	0.901005	-0.78283	0.821627	0.060975	1.878521	-0.89972	-0.26253	0.469198	-0.1042	-0.82846
Q5JSZ5	PRRC2B	0.745852	-0.23035	-0.18463	-0.52742	-0.60724	-0.04644	-0.17078	-0.53325	-0.1674	-1.02492	0.455873	-1.18716	-0.2162	-1.03966	-0.0828	-0.09405	-0.96133	-0.41962	-0.17942
Q5JTD0	TJAP1	-0.06096	0.154171	0.297551	-0.36132	-0.29447	0.187388	0.101666	0.005038	-0.10672	0.149584	-0.10193	-0.57245	-0.2049	-0.29766	1.028364	0.339772	0.421424	0.327269	0.085637
Q5JTH9	RRP12	-0.24426	-0.33133	-0.65152	0.915259	-0.56496	-0.21068	-0.32429	0.148726	-0.52803	0.105596	1.181055	0.056698	0.079469	-0.61839	0.010509	-0.72789	-0.07376	-0.46445	-0.30781
Q5JTV8	TOR1AIP1	-0.49521	0.486242	0.311571	-0.00766	-0.31855	-0.24398	0.318503	-0.03207	-0.59813	-0.0946	0.365035	-0.64059	-0.58374	-0.18869	1.11313	-0.09622	-0.55193	-0.04779	-0.80427
Q5JTZ9	AARS2	0.381496	0.294778	-0.03239	-0.0065	0.043395	0.321044	-0.41334	-0.04822	0.247499	-0.30165	0.533876	-0.13482	0.212173	-0.32207	0.479335	0.325117	-0.14361	0.162115	0.342278
Q5JVF3	PCID2	0.059105	0.028087	-0.53115	-0.1812	0.291814	0.540925	-0.4066	-0.07997	0.050227	0.715179	-0.00312	-0.379	-0.21743	-0.30284	0.131953	0.410837	0.175249	-0.39194	0.153804
Q5JVS0	HABP4	-0.54335	0.458987	0.11883	-0.32171	-0.55261	-0.69061	-0.33807	-0.15849	0.201459	0.337276	-0.7217	-0.12556	0.034445	-0.23943	-0.6964	0.343533	0.000582	0.300254	0.351521
Q5K4L6	SLC27A3	-0.21609	0.712251	-0.01125	0.97248	0.026303	0.454808	0.399254	-0.41296	-0.69453	-0.40789	-0.86102	0.118797	-0.18942	0.30744	1.738383	-0.37057	0.233405	-0.88592	-0.75529
Q5K651	SAMD9	1.252569	0.739321	1.119774	-0.8816	-0.02328	0.390669	0.264331	1.659519	-0.04174	-0.10382	-0.89508	0.22635	0.082958	0.70027	-0.45796	-0.1173	-0.2085	-0.42612	-0.5583
Q5KU26	COLEC12	-1.98057	0.759286	0.856051	-0.82264	-1.19853	-2.02783	1.868236	-0.98304	0.47326	0.122229	-1.42857	-0.27554	0.400234	0.046904	-0.56286	0.409598	-1.2871	1.598967	1.220402
Q5M775	SPECC1	-0.359	0.14735	0.411473	0.00939	-0.2071	0.488163	-1.04671	-0.64346	0.307646	0.177095	-0.34958	-0.59464	0.89701	-0.5958	0.693057	-0.35768	-0.60157	-0.42892	-0.04688
Q5MIZ7	PPP4R3B	0.398696	0.658555	0.443281	-0.04068	-0.20439	0.037017	-0.22071	0.73394	-0.61289	-0.45299	0.356095	-0.38113	0.030632	-0.04073	-0.52679	-0.09753	-0.40919	-0.36114	-0.03068
Q5MNZ6	WDR45B	0.515042	-0.12542	-0.93956	0.215028	0.440987	-0.64904	-0.35347	-0.6615	-0.1141	-0.17919	0.54431	0.327942	-0.17144	0.305897	-0.65044	-0.19767	0.661431	0.090538	0.041992
Q5MNZ9	WIPI1	-0.6196	0.579807	0.383304	-0.14715	-0.39506	-0.60498	0.286732	-0.88225	0.06817	0.821117	-1.04181	-0.29041	-0.11476	0.683402	0.081782	0.073858	-0.68632	0.300093	0.098244
Q5NDL2	EOGT	-0.17224	0.281222	0.059303	-0.13519	0.592475	0.226913	0.243262	-0.28993	0.271458	0.074999	0.325574	-0.05018	0.354991	0.014121	-0.08345	0.136466	-0.69699	0.379887	1.379721
Q5PRF9	SAMD4B	-0.4073	-0.29465	0.077749	-0.95376	0.04065	0.279874	0.398276	2.745998	0.361242	-0.06527	0.10873	-0.57073	-0.14376	0.279522	0.045449	0.094302	-0.42697	-0.25514	0.65895
Q5PSV4	BRMS1L	0.493517	-0.25542	-0.12794	-0.14468	-0.38041	-0.51764	-0.15683	0.236741	0.002369	-0.47231	0.258515	-0.23248	-0.22765	-0.23195	-0.09935	-0.2213	-0.16828	0.056887	-0.34423
Q5QJ74	TBCEL	-0.25756	0.044904	0.100891	-0.13622	-0.17079	-0.56162	-0.35995	0.180585	0.339276	-0.0402	0.227085	0.168919	0.972987	0.079689	-0.18904	-0.04294	0.171017	0.53392	0.596148
Q5QJE6	DNTTIP2	0.764986	-0.13379	-0.50039	0.516789	-0.44352	0.124336	-0.38683	-0.11463	-0.32108	-0.35125	0.653867	-0.42892	-0.60149	-0.82726	0.516488	-0.1239	-0.10408	0.180255	-0.13218
Q5R372	RABGAP1	-0.46356	0.57015	0.545741	-0.18853	-0.07632	-0.2903	-0.0565	-0.36232	0.088865	-0.37013	-0.40127	0.059012	0.044731	0.305425	0.770285	0.005428	-0.19951	0.470128	0.201416
Q5R3I4	TTC38	-0.89061	0.205334	-1.39747	-0.49352	-0.33204	-0.84388	-0.02309	-0.87676	0.040847	0.184631	0.634636	0.103589	1.185181	-0.36447	-0.84977	-0.00982	-0.02738	0.971713	-0.22305
Q5RKV6	EXOSC6	0.473021	-0.105	-0.09615	-0.33011	0.545646	0.698185	-0.43373	-0.12828	0.265167	-0.39626	-0.00271	0.084408	0.017335	-0.53077	-0.58737	-0.12615	0.013782	-1.05001	0.208382
Q5SGD2	PPM1L	-0.40845	0.132228	0.116907	-0.21468	-0.71641	-1.15253	0.181851	-0.63705	-0.05258	-0.0229	-0.21104	0.075897	-0.12575	0.159397	0.995947	-0.32233	-0.25964	-0.21625	-0.54322
Q5SNT2	TMEM201	0.093306	-0.3273	-0.23032	-0.58624	-0.07818	-0.19391	-0.2024	-0.2278	-0.41851	-0.43623	1.036711	-0.65202	-0.12518	-0.38985	1.261976	0.488245	0.377129	-0.31965	0.048731
Q5SQI0	ATAT1	0.443723	-0.06931	-0.01634	-0.17006	-0.38124	0.092612	-0.03291	-0.53841	-0.1864	-0.29968	0.756377	-0.37887	-0.08489	-0.3611	-0.07591	0.016698	0.007696	-0.09041	0.857503
Q5SQN1	SNAP47	-0.23096	-0.1187	-0.09151	-0.04759	-0.12669	-0.02407	-0.13191	0.046078	-0.31105	-0.28751	0.325211	-0.00031	-0.03823	-0.09752	0.170256	0.253773	0.186992	-0.06286	-0.14673
Q5SRE5	NUP188	-0.03869	0.211018	-0.03918	0.082496	0.101374	-0.02209	0.105527	0.195164	0.587674	-0.30493	0.189279	-0.15372	-0.31391	0.155008	0.894122	-0.16338	-0.14997	-0.43878	0.272551
Q5SRE7	PHYHD1	-1.77142	0.339098	0.034478	-0.69645	0.820637	0.282146	0.115521	0.865051	0.094158	0.817433	0.242131	-0.21871	0.121664	0.19082	-0.04799	-0.85443	-0.07171	0.596123	0.112458
Q5SRH9	TTC39A	1.976791	-0.39484	-0.69647	-0.03343	-0.46432	-0.34593	-0.15706	-0.83454	0.252082	0.278243	0.102399	-0.39184	0.068096	-0.31092	-0.05995	0.688575	-0.21712	-0.27778	-0.47331
Q5SRI9	MANEA	0.012234	-0.18883	0.898801	-0.31275	1.142264	-0.14878	0.921848	-0.89455	0.357494	0.369718	-0.54911	-0.30667	0.211559	0.141768	0.310375	-0.15941	-0.08663	0.191359	-0.27788
Q5SSJ5	HP1BP3	-0.56139	-0.22403	-0.9988	-0.63221	-1.07879	-1.46372	-0.23426	0.513406	0.135422	0.919172	-0.10928	-0.46623	-1.23087	-0.27098	0.233677	-0.46507	-0.46688	-0.11015	-0.7673
Q5ST30	VARS2	-0.18365	-0.15363	0.084951	0.728963	1.045604	0.996131	-0.10748	-0.92686	-0.09094	-0.36092	0.492026	-0.26454	-0.08626	0.175401	1.029172	0.483085	-0.07825	0.067705	-0.50277
Q5SW79	CEP170	-0.57625	0.331512	-0.51947	-0.69005	-0.72355	-0.7291	-0.16406	-0.43834	0.152012	0.761019	-0.56805	-0.16677	0.306691	0.100749	0.370532	-0.25747	-0.16833	0.397226	0.998946
Q5SW96	LDLRAP1	-0.4353	0.534889	-0.58296	-0.64468	0.523858	0.008428	0.606806	-0.09481	0.169089	0.027581	-0.21564	0.061516	0.187344	-0.14029	-0.21598	-0.29277	-0.47816	0.26104	0.950905
Q5SWX8	ODR4	-0.85208	0.185476	0.303394	0.8501	-0.31337	-0.03055	0.272166	-0.51392	-0.18702	-0.24103	0.363756	-0.1713	-0.44355	-0.03393	0.06858	-0.67682	0.166519	-0.3096	0.105916
Q5SXM2	SNAPC4	1.148023	-0.7293	-0.37308	-0.32648	-0.47631	-0.09073	0.017352	0.302369	-0.04329	-0.98947	-0.16089	-0.96041	0.057992	-1.4467	-0.4138	-0.66934	-1.35283	-1.40261	0.007216
Q5SXM8	DNLZ	0.450028	-0.08626	0.589209	0.100187	1.068954	-1.07153	-0.74697	0.430531	0.283918	0.807714	-0.00679	0.10238	1.020476	-0.32138	1.678037	0.363621	0.053981	-0.00126	-0.15706
Q5SY16	NOL9	0.549482	-0.08521	-0.49737	0.173751	0.346711	-0.20138	-0.19988	0.913682	-0.27945	-0.19633	0.777403	-0.2192	-0.42632	-0.73252	0.138367	-0.13715	-0.10723	0.429434	0.34726
Q5T011	SZT2	-0.13133	-0.65488	-1.20356	0.009292	0.350969	0.363115	-0.56678	-0.79304	0.080677	-0.39611	-0.18922	-0.19308	0.264694	-0.04494	0.013073	-0.48744	-0.47716	-0.47996	-0.67167
Q5T0F9	CC2D1B	0.437417	0.55874	0.029247	-0.17377	0.048221	0.342652	0.941263	-0.09343	-0.96053	0.351329	-0.09395	0.122298	0.38002	0.105922	-0.14732	0.486342	-0.03311	-0.01687	-0.19166
Q5T0N5	FNBP1L	0.978328	-0.80354	-0.34241	-0.50602	-0.4646	0.099011	-0.22657	1.049956	0.204113	-0.30826	-0.12002	-1.41572	0.032929	-0.56991	-0.4541	0.303428	-0.44477	-0.19204	0.550814
Q5T0Z8	C6orf132	-0.07152	-0.56117	-0.38077	-0.17902	-1.25462	-1.08043	-0.03047	0.371556	0.457513	-1.76369	-0.0694	-0.39536	0.677731	-0.56973	0.816627	0.753139	-0.04116	-0.82246	0.414184
Q5T160	RARS2	0.44811	0.944202	0.376914	1.326063	0.067569	0.297968	-1.07153	-0.81999	-0.26185	0.250979	0.005361	-0.22026	-0.0018	-0.00757	0.723507	0.674364	-0.2658	0.043765	-0.38136
Q5T1M5	FKBP15	-0.60602	1.022784	0.673461	-0.42307	0.102506	0.042111	0.255811	-0.39303	-0.14917	0.493342	-0.4538	1.044061	0.233474	1.161484	-0.43981	-0.19045	0.375278	0.053774	-0.08104
Q5T1V6	DDX59	0.078616	0.257154	0.123176	0.268191	0.185497	-0.00394	-0.30164	0.034093	-0.18372	0.59541	-0.04028	0.035651	0.156823	0.19583	0.162028	-0.05702	-0.15596	-0.11961	0.12384
Q5T200	ZC3H13	0.235628	-0.54734	0.127161	0.231622	0.242899	0.307884	0.00216	-0.06094	-0.01	0.396942	0.512546	-0.91703	0.219098	-0.66684	0.164879	-0.51137	-0.50138	0.3697	0.126854
Q5T280	SPOUT1	0.742328	-0.34184	0.259745	-0.58056	-0.56796	-0.04444	-0.34684	0.040049	-0.27954	-0.09985	0.267556	-0.35922	-0.05097	0.036906	-0.24553	-0.50663	-0.78894	-0.38996	-0.00525
Q5T2E6	C10orf76	-0.53643	0.647414	0.01947	-0.13457	0.075246	0.1124	-0.4594	-0.05571	0.358985	0.142112	-0.14327	-0.11185	0.606352	-0.12123	0.375038	-0.09076	0.171134	0.251437	0.045247
Q5T2T1	MPP7	0.818845	-0.09487	-0.13342	0.648991	0.456579	0.304715	-0.50031	0.190161	-0.16264	-0.56584	0.137735	-0.17455	0.1324	-0.15655	1.812135	0.558086	-0.15528	-1.00535	-1.20759
Q5T3F8	TMEM63B	-0.35963	0.007703	0.116586	-0.17518	0.488839	0.0047	0.571436	-0.1865	0.303252	0.024036	0.42417	0.480431	-0.08839	0.269094	0.418085	-0.0316	0.2683	0.777015	-0.0845
Q5T3I0	GPATCH4	0.983028	-0.45554	-0.41009	0.108492	-1.24451	-0.32768	-0.42371	-0.01325	-0.28889	-0.59346	0.713158	-1.65178	-0.0424	-0.68356	1.277764	-0.36336	-0.26005	-0.3416	-1.21618
Q5T3U5	ABCC10	-0.49969	0.187587	-0.05947	-0.65532	-0.3291	0.031266	0.224149	-0.37463	0.079256	0.037269	0.006013	0.328448	-0.19955	0.221145	-1.09325	-0.30303	0.19763	-0.11698	0.471976
Q5T440	IBA57	-0.36114	-0.24115	-1.22647	0.114499	-0.6271	-0.62269	-0.36961	0.862983	0.060052	0.766724	0.542343	0.497106	-0.25461	0.132053	0.645812	0.483219	-0.02001	0.150753	-0.37245
Q5T447	HECTD3	-0.02915	0.517183	0.52532	-0.31514	0.146468	0.084357	0.302736	-0.35099	-0.05346	0.434051	-0.23476	0.486364	0.056539	0.624235	-0.27798	-0.17923	-0.10351	-0.32565	-0.16483
Q5T4B2	CERCAM	-0.71031	0.384878	-0.00273	-0.92823	0.417854	-0.09589	0.263724	0.049318	0.214656	-0.04207	-1.0797	-0.54294	0.434408	-0.51289	-0.83353	0.078998	-0.65548	0.366975	0.996665

Q5T4S7	UBR4	-0.2491	0.320371	0.381832	-0.41426	-0.10253	-0.03395	0.389859	0.287043	-0.05032	0.225688	0.070246	0.025705	0.014535	0.11424	0.221976	0.257758	-0.12458	-0.665	0.14643
Q5T5C0	STXBP5	-0.63415	0.131708	0.287772	-0.22939	-0.15121	0.199801	0.059971	-0.65066	0.098462	0.18626	-0.23684	-0.56318	-0.22132	0.920747	0.042363	-0.49094	-0.07592	0.092565	-0.05086
Q5T5P2	KIAA1217	0.884306	-1.0827	-1.24899	-0.0629	-0.79077	-0.47258	-0.68704	0.188928	-0.05143	-0.57097	-0.04023	-0.28153	1.263981	-0.90472	0.577213	1.131882	-0.54249	-0.46866	0.040231
Q5T5U3	ARHGAP2	-0.60659	0.227517	0.208302	-0.22008	-0.29916	-0.14659	0.46213	0.114249	0.13647	0.131998	-0.51216	-0.70717	0.098969	-0.44704	0.111348	0.366779	-0.12289	-0.05417	-0.12826
Q5T5X7	BEND3	0.377076	-0.10284	-0.02169	0.268292	0.281095	1.14201	-0.06764	0.100769	-0.47762	-0.57189	0.149087	0.244872	-0.17364	-0.0796	-0.32841	0.240382	-0.24154	0.013456	-0.04491
Q5T5Y3	CAMSAP1	0.414566	-0.22542	0.413295	-0.6757	-0.65139	-0.74072	0.171389	-0.10177	0.06213	0.069699	0.128369	-0.44045	-0.07586	-0.1739	-0.20171	-0.17561	-0.07049	-0.32864	0.076666
Q5T653	MRPL2	-0.58096	-0.11485	0.190237	1.326901	0.161698	0.342694	-0.37179	-0.37488	0.232461	-0.21929	0.641301	0.305709	0.137129	0.353815	0.731704	-0.28799	0.058541	-0.07563	-0.84947
Q5T6F2	UBAP2	0.579838	-0.30103	-0.19039	-0.41429	-0.64081	0.001103	-0.42279	-0.14151	-0.01182	-0.01349	0.094509	-1.26797	-0.01951	0.150974	0.229496	0.773962	-0.00167	0.670615	-0.04265
Q5T6V5	C9orf64	-0.21701	0.0626	-0.43893	-0.74919	0.575754	1.159647	-0.10569	0.132078	-0.48947	-0.3625	0.744485	-0.85676	-0.27266	-0.73824	-0.83634	-0.07146	-1.1619	-0.99886	0.533859
Q5T749	KPRP	-0.60287	0.283501	1.265996	-0.67965	-0.48479	-0.28286	2.944874	-0.05461	0.772065	-0.15546	-0.62844	-0.33484	0.91014	-0.36566	-0.43975	-0.178	-1.05987	-1.21448	0.132873
Q5T7V8	GORAB	-0.67536	0.106641	0.304309	-0.07154	-0.14147	-0.41368	0.481586	-0.31695	0.172287	-0.37696	-0.60757	-0.37204	-0.11134	-0.41113	1.068988	-0.10678	-0.53264	0.053478	0.156753
Q5T7W0	ZNF618	0.433446	-0.21365	-0.61342	0.031645	-0.18007	0.326053	-0.81887	0.427449	-0.58024	-1.67845	0.20296	-1.08165	0.193917	-0.94031	-0.4522	0.146225	-0.44269	0.690858	0.839685
Q5T8D3	ACBD5	-0.18623	0.838752	0.312179	-1.24105	0.468454	0.602452	-0.05461	-0.07694	-0.1708	0.458156	-0.63785	1.309969	-0.24387	0.716341	-0.18866	0.251083	1.257875	0.193856	-0.43589
Q5T8P6	RBM26	1.036084	-0.39672	-0.17782	-0.45193	-0.24678	0.1106	-0.13848	0.258145	-0.12337	-0.0858	0.846869	-0.53398	-0.46744	-0.43737	0.102737	-0.49409	0.021393	0.428688	-0.09304
Q5T9A4	ATAD3B	0.515345	0.479082	0.17146	0.704089	1.729916	1.372337	0.388822	0.11412	-0.52498	1.188712	0.248555	-0.45698	-0.16172	-0.65577	0.454232	1.461049	0.697556	0.723051	-0.38774
Q5T9L3	WLS	0.570322	-0.0533	-0.33333	-0.16304	-0.79953	-0.61352	0.822845	1.529013	0.337892	-0.45485	0.131571	-1.30976	0.27276	-0.62502	-0.27651	-1.44243	-0.28671	0.056732	1.944535
Q5TA45	INTS11	-0.02134	0.513183	-0.1519	0.298421	-0.14925	-0.21019	0.243395	0.760639	-0.28183	-0.18043	0.216807	-0.07136	-0.48292	0.045985	-0.29441	0.271965	0.20552	-0.1432	0.278273
Q5TA50	CPTP	-0.07478	0.422716	0.190897	-0.69166	0.107277	0.661233	0.090209	0.392246	0.101012	0.440075	-0.33038	-0.24817	0.051769	0.104901	-0.18618	-0.0448	0.951199	0.942974	-0.29234
Q5TAQ9	DCAF8	-0.53617	-0.15976	-0.14599	0.182773	-0.29912	-0.16548	-0.23623	-0.02785	-0.11985	0.140143	0.334926	-0.56742	-0.11684	-0.55304	0.955682	0.175504	-0.17823	-0.4432	-0.0087
Q5TAX3	ZCCHC11	1.211338	-0.36915	0.08473	-0.46811	-0.4919	0.095553	-0.37113	0.325111	-0.43309	0.004159	0.205624	-0.89647	0.011254	0.105683	-0.30647	0.577774	-0.65132	-0.38727	-0.61299
Q5TBA9	FRY	-1.08028	0.501374	0.432328	-0.21042	-0.05742	-0.50285	0.228991	-0.37684	0.135559	-0.0362	-0.22553	-0.14348	0.185341	0.166856	-0.11643	-0.13011	-0.31867	0.051789	0.213031
Q5TBB1	RNASEH2	-0.41215	0.316145	0.066751	-0.48594	0.824504	0.749588	0.01969	0.969828	-0.26629	0.988128	-0.14242	-0.50765	-0.65969	0.27327	-0.29163	-0.17234	0.089502	0.463309	-0.52733
Q5TC12	ATPAF1	-0.08603	0.071282	0.094117	1.013166	1.589319	1.944565	-0.0519	0.000181	-0.33252	0.0439	0.208011	0.264407	-0.04645	0.006415	1.30808	1.927398	0.978229	0.635061	-0.29038
Q5TCQ9	MAGI3	-0.13312	0.332524	-0.40746	-0.36076	0.422693	0.188213	0.074343	-0.11657	-0.57418	-0.9611	-0.86034	-0.87842	0.211267	0.007157	-1.06832	1.029508	-0.16053	-1.06937	-0.47854
Q5TCZ1	SH3PXD2/	-1.27836	0.611603	0.213595	-1.62711	-0.45119	-0.22048	0.122917	-0.64363	0.869233	-0.2992	-0.58644	-0.4121	0.624346	-0.4696	-1.01597	0.175307	-0.1027	0.704938	1.752572
Q5TDH0	DDI2	0.311123	0.474086	0.053987	-0.41772	-0.36775	-0.79904	-0.208	-0.15274	0.772232	0.743982	-0.20781	-0.59873	-0.22482	-0.36845	-0.20557	-0.41905	0.176753	0.92207	-0.63535
Q5TEJ8	THEMIS2	-0.95143	1.317478	0.821896	-0.33528	0.041501	-0.24804	-0.17739	-0.76868	-0.85834	1.749743	-1.4757	1.478383	0.82768	1.166662	0.823461	-0.35816	1.05139	0.430626	-0.75436
Q5TEU4	NDUFAF5	-0.29054	0.01151	0.033532	0.141152	0.429692	0.15161	-0.16294	0.810203	0.21243	1.020851	-0.25214	0.147118	0.1546	0.420254	0.378242	0.842411	-0.38848	-0.10706	0.306533
Q5TFE4	NT5DC1	-0.11709	-0.04552	0.369467	1.251411	1.974107	0.714577	-0.2058	-0.42302	0.44978	0.910045	-0.44278	-0.11058	0.440437	-0.01541	0.788174	-0.08572	0.625777	0.951488	0.107289
Q5TGL8	PXDC1	-0.32889	0.436052	-0.17094	-0.5574	-0.36144	-0.36817	0.31153	-0.78334	-0.15111	-0.51259	-0.77838	-0.67287	-0.08609	-0.39819	0.176453	-0.37497	-0.74127	0.619159	1.106481
Q5TGY1	TMCO4	0.246293	0.154186	0.030453	-0.44772	0.463067	0.153501	-0.0795	0.615621	-0.09589	0.179994	0.279912	0.159815	-0.52963	0.17238	0.32786	0.907113	-0.37467	-0.27768	0.222207
Q5TGY3	AHDC1	1.098404	-0.18228	-0.42019	-0.59292	0.12474	0.012552	-0.2373	0.737469	0.013233	-0.44203	0.041634	0.317312	-0.01429	0.289437	0.870166	-0.95238	-0.34488	0.011652	-0.11614
Q5THJ4	VPS13D	-0.30179	-0.2129	0.853766	-0.42713	-0.09566	-0.09114	0.130467	0.082225	-0.07854	0.146334	0.227974	-0.01164	-0.1407	-0.2661	-0.14834	0.513476	0.545599	0.408223	0.242224
Q5TZA2	CROCC	-0.4941	-0.04568	-0.5972	-1.07716	-1.14324	-1.40226	-0.0102	-0.36139	-0.19493	-0.50495	2.499568	-1.11734	-0.67442	-0.8474	-0.6963	-0.61047	-1.39041	-0.33657	-0.91102
Q5U5X0	LYRM7	-0.30222	-0.74102	0.153298	-0.25188	0.913726	0.430446	-0.27007	0.113108	-0.70054	-0.30297	0.114754	-0.09776	-0.00615	0.04474	2.443758	0.470532	1.145192	0.299217	-1.83358
Q5U651	RASIP1	-0.664	0.194934	0.223794	-0.06358	0.095284	-0.39093	0.648736	-0.90205	-0.31556	0.383107	-0.34069	-0.27679	-0.27284	-0.53139	0.415988	-0.25247	-0.58632	0.785534	-0.48562
Q5UCC4	EMC10	0.046873	0.539529	1.080584	0.121262	0.311778	0.035615	1.411147	0.358781	0.201943	-0.09249	0.188488	-0.22426	0.546751	0.258403	0.013921	0.460558	0.125785	-0.18166	-0.22776
Q5UIP0	RIF1	1.8321	-0.34624	-0.14912	-0.50098	0.091808	0.628963	0.25157	0.548088	-0.15821	-0.26594	-0.10038	-0.47789	-0.13747	-0.33311	-0.16235	0.180914	-0.15807	-0.18475	-0.02346
Q5VIR6	VPS53	0.398431	0.158334	0.060996	-0.17648	-0.05262	-0.26717	0.25187	-0.03813	-0.20268	0.633737	-0.16259	0.130839	0.079069	0.063963	0.736332	0.33829	-0.49534	-0.28782	-0.325
Q5VSL9	STRIP1	-0.04243	0.274079	-0.11151	-0.1622	0.033484	0.045529	0.02611	0.326818	-0.88948	0.618146	-0.07367	0.018598	-0.20594	-0.06473	-0.20762	0.164344	0.040906	-0.00928	0.05937
Q5VT06	CEP350	-0.08855	-0.03993	0.319416	-0.19494	0.285239	-0.04505	-0.18958	-0.18654	-0.05286	0.418316	0.04211	-0.37958	-0.04467	-0.1444	0.416576	0.091628	-0.22556	-0.45878	-0.31566
Q5VT25	CDC42BP/	-0.36933	-0.17625	-0.77817	-0.18155	-0.20803	-0.43636	-0.69293	-0.56117	0.186336	-0.36421	-0.7309	-0.90305	0.37863	-0.88683	1.31544	0.269531	-0.6129	-0.60228	0.35961
Q5VT52	RPRD2	0.41051	-0.42322	-0.27811	0.045721	0.044352	-0.02783	0.020353	0.821305	-0.44566	-0.50293	0.181119	-0.63145	-0.51082	-0.51357	0.805671	-0.65863	-0.59637	-0.10715	-0.33907
Q5VT66	MARC1	1.16761	-0.48037	-0.84134	0.359216	-1.346	-0.85947	-0.77945	0.014335	-0.40223	-0.13245	0.28022	-0.60854	-0.22355	-0.29998	0.076845	-1.33508	-1.0727	-1.44705	-1.13899
Q5VT79	ANXA8L1	-1.17126	0.897814	1.004448	-0.22054	-0.7695	-0.32511	0.839118	3.067521	1.914023	2.147205	-0.95066	0.738975	1.199446	0.071688	-0.28731	-1.60647	0.327765	-0.84009	0.467424
Q5VTL8	PRPF38B	0.645044	-0.10801	-0.51006	-0.76318	0.321389	0.251365	-0.69181	0.030434	-0.13806	-0.3439	0.280312	0.043314	-0.51743	0.153997	-0.03868	-0.23409	0.059725	0.01147	-0.7615
Q5VTQ0	TTC39B	-0.31491	0.332413	0.171561	0.16917	0.262796	0.830184	0.268054	-0.6088	-0.05144	-0.43768	0.238092	0.163852	0.262994	0.134465	0.026986	-0.23764	-0.37998	0.490243	0.469859
Q5VTR2	RNF20	0.435634	-0.05801	-0.26529	0.148593	0.479106	0.743252	-0.04288	0.540142	-0.14753	0.109489	0.154166	-0.32572	-0.0471	-0.41659	0.241024	0.245701	-0.42498	-0.10766	0.034073
Q5VU43	PDE4DIP	0.003653	0.569838	0.354539	-0.5811	0.918579	0.791091	0.082959	-0.1059	0.229861	0.541348	-0.08667	-0.31282	0.366219	-0.25811	0.665423	0.53328	-0.24438	0.030702	0.383367
Q5VU97	CACHD1	0.40377	-0.06005	0.100757	-0.00883	0.174171	0.099638	-0.37329	-0.2909	-0.06752	-0.03154	0.492536	-1.01239	-0.05774	-0.60476	-0.51894	-0.23638	-0.26848	-0.06393	-0.43775
Q5VUA4	ZNF318	0.448695	-0.14961	-0.0083	-0.12972	-0.20606	0.184312	-0.09116	0.121432	-0.01457	-0.32696	0.243038	-0.56243	-0.33674	-0.20875	0.038951	-0.66926	-0.10298	0.603338	-0.07685
Q5VUB5	FAM171A1	0.235962	-0.15607	-0.19091	-0.24263	-1.27706	-0.45873	-0.06713	0.400547	0.126616	-0.17174	-0.17765	-1.5851	-0.00497	-0.4742	1.159758	-0.51837	-0.32518	-0.34711	0.350848
Q5VV41	ARHGEF1	0.618363	-0.82194	-0.25828	0.049969	0.043975	0.69039	0.003646	0.525735	-0.18267	0.79487	0.709577	-0.41526	-1.64241	-0.48283	0.665688	1.017383	1.268865	0.022191	-0.62429
Q5VV42	CDKAL1	0.432109	-0.07989	-0.23763	0.535996	0.08104	-0.1047	0.119072	-0.35993	-0.12619	-0.56749	-0.46825	-0.0505	0.272361	-0.01975	0.359119	0.025006	-0.26607	-0.33666	-0.2714
Q5VVJ2	MYSM1	0.07372	-0.08933	0.190757	-0.33059	0.627321	0.66896	-0.31265	-0.11443	0.064292	0.215231	0.326068	-0.53118	-0.17541	-0.19477	0.421478	0.579475	-0.22248	0.445361	-0.85097
Q5VVQ6	YOD1	-0.43277	0.763337	-0.26642	-0.91255	-0.43083	-1.17613	0.535497	0.099379	1.767688	0.057313	-0.68079	-0.91292	-0.99925	-0.09284	-1.11576	-0.39508	-0.85942	0.011446	-0.63369
Q5VW32	BROX	-0.62626	-0.54104	-0.15522	0.455554	-0.76343	-0.46349	-0.57012	-0.00899	-0.55469	0.507462	0.056637	-0.80323	-0.01723	-0.75295	1.878483	-0.09214	-0.85482	-1.51864	-0.80387
Q5VW36	FOCAD	-0.01303	-0.01237	0.044695	0.491326	0.04896	0.009262	-0.12794	-0.58042	-0.26256	-0.48605	0.071235	-0.49939	-0.11619	-0.18039	-0.19124	0.63379	-0.7284	-0.80457	0.922364
Q5VW38	GPR107	-0.14885	0.33272	0.492024	-0.38871	0.275532	-0.27699	0.792196	0.024834	0.682657	0.001956	-0.57346	-0.09282	-0.02106	-0.06514	0.890364	0.096459	-0.07483	0.62469	0.436567
Q5VWG9	TAF3	-0.1961	-0.25899	-0.1752	-0.10751	-0.38407	-0.28413	-0.50882	0.622857	-0.00127	-0.17547	-0.06568	-0.24745	0.083355	-0.31734	0.038249	0.119943	-0.38189	0.283076	0.470014
Q5VWJ9	SNX30	-0.1968	0.712621	0.756245	-0.10569	0.569287	0.288238	0.221751	-0.03643	0.225852	0.41148	-0.67776	0.507084	0.053698	0.627347	-0.24414	-0.19788	-0.05751	-0.05913	0.706876
Q5VWN6	FAM208B	0.417115	0.033674	-0.03256	-0.18176	0.238358	0.075174	-0.11193	0.530881	-0.03132	0.127383	0.113697	-0.294	-0.32246	-0.09111	0.450538	0.080736	-0.05457	0.464156	0.040041
Q5VWQ0	RSBN1	0.397863	0.048152	-1.39196	0.142036	-0.06065	0.452605	0.134719	0.777165	-0.40198	-0.41939	0.157753	-0.12933	-0.15135	-0.24907	-0.0445	-0.5879	-0.10773	-0.35717	-0.31477
Q5VWQ8	DAB2IP	-0.05384	0.125298	0.151978	-0.86644	-0.52053	-0.35243	0.537431	0.477031	0.029898	-0.48999	0.296822	-0.32154	0.067097	-0.5336	0.360526	-0.18578	0.864299	0.659204	0.273487
Q5VWZ2	LYPLAL1	-0.932	-0.13852	-0.41006	-1.08429	-0.80952	-0.524	-0.44196	0.483492	0.040847	2.756661	1.317294	0.121322	0.308334	0.62854	-0.84628	-0.63647	0.177623	0.149701	-0.004
Q5VYK3	ECM29	0.564778	0.129136	0.494852	-0.5237	0.943345	1.06143	0.029167	-0.07385	0.401506	0.129148	0.134271	0.100684	-0.15668	0.037222	-0.72024	-0.24142	-0.14873	-0.43731	0.085859
Q5VYS8	ZCCHC6	-0.28161	-0.0243	0.412187	-0.32225	0.436376	0.584389	-0.20752	-0.30341	0.119049	0.828069	-0.18255	0.114018	0.223696	0.519237	0.034373	0.488873	0.243245	0.37982	0.177587
Q5VZ89	DENND4C	0.215639	0.740625	0.487692	0.197588	0.206438	0.034145	-0.06084	-0.3425	0.021463	-0.56819	-0.07835	0.311915	-0.07841	0.542096	-0.11587	-0.09353	0.160062	-0.30137	-0.38655
Q5VZE5	NAA35	-0.27401	0.464654	0.121451	-0.28974	0.076271	0.236116	0.066437	0.155708	0.211298	-0.19563	-0.05138	-0.10951	0.216527	-0.29087	0.309227	-0.32239	-0.06822	-0.28715	0.528593
Q5VZK9	CARMIL1	0.75305	-0.24739	-0.36023	0.088622	-0.55903	-0.14288	-0.77257	-0.91105	-0.02385	-0.97886	-0.16379	-0.3697	0.505769	-0.26209	0.956492	0.343112	-0.06186	-0.69282	-0.46058
Q5VZL5	ZMYM4	0.189846	-0.23633	-0.35165	0.614215	-0.07541	0.546382	0.142058	0.648993	-0.39006	-0.24803	0.135223	-0.72615	-0.07614	-0.33138	0.323935	-0.43257	-0.43412	0.152631	-0.1302
Q5W0B1	RNF219	0.546712	-0.17714	0.021748	-0.02457	-0.01876	0.327884	-0.28667	0.419296	-0.06608	0.100325	-0.10462	-1.10546	0.00497	-0.39608	0.25567	-0.36133	0.110466	-0.33114	0.150936
Q5W0U4	BSPRY	0.255002	-0.27295	-0.05081	-0.13452	0.143516	0.626466	-0.26547	-0.30001	-0.18443	-0.42206	1.042994	0.018198	-0.07786	-0.08587	-1.11411	0.024255	0.120249	-0.91055	-1.49757
Q5W0V3	FAM160B1	-0.3836	0.345525	-0.03272	-0.44373	-0.05994	0.274506	0.697152	-0.39746	0.290454	0.092915	-0.40917	0.03774	0.000428	0.10147	0.317417	0.080291	-0.10008	-0.34672	0.299916
Q5W111	SPRYD7	-0.58995	0.199498	0.515939	0.018604	-0.74202	0.311398	0.5373	0.025663	-0.14016	0.463536	0.158164	-0.05484	-0.09418	0.054637	1.247787	0.833037	-0.26147	0.452808	0.426461
Q5XKP0	MIC13	0.112355	0.369734	-0.40964	-0.16458	-0.14456	-0.15238	-0.21204	-0.01434	-0.55301	-0.14881	0.704742	-0.37182	-0.09637	-0.69347	1.006156	0.989036	-0.92332	-0.37012	-0.52148
Q5XPI4	RNF123	-0.12428	0.474091	0.230036	-0.69627	0.085197	-0.41301	-0.07384	-0.3775	-0.05229	-0.43204	-0.1652	-0.21171	-0.71321	-0.19881	-0.25029	-0.2872	-0.75656	0.046007	-0.27324
Q5XUX1	FBXW9	-0.95978	0.02386	-0.37259	-0.12059	-1.0022	-0.48684	-0.20362	0.178589	-0.55056	-0.16687	0.799653	-0.93983	-0.39783	-0.33317	-1.21564	-0.64068	-0.64534	-1.25373	-0.17874
Q5XXA6	ANO1	1.179727	-0.17469	-0.35099	-0.98651	-0.3716	-1.34094	-0.26681	0.158257	0.143395	-0.93844	0.276676	-0.86414	-0.1026	-0.25778	0.272108	-0.36253	-0.28239	-0.18143	0.574553
Q5Y7A7	HLA-DRB1	-0.10508	1.872097	0.338834	-0.2607	-0.23237	-0.73815	0.76257	-0.31661	-0.27348	-2.43372	3.117879	0.141286	-0.11126	0.458296	-0.14809	-2.68255	0.449327	1.854424	-0.20494
Q5ZPR3	CD276	-0.3473	1.18263	-0.16906	-0.80078	-0.42812	0.019427	0.480914	0.35116	-0.16697	-0.42387	-0.7016	-0.40984	0.52761	-0.45384	-0.35841	-0.09536	-0.59197	0.463994	0.844926
Q63HN8	RNF213	0.41865	0.911088	1.326173	0.407078	-0.10956	0.020059	0.168864	-0.1382	-0.1612	0.101455	-0.85969	0.597281	0.003639	0.664197	-0.70875	0.21096	0.483615	-0.1504	-0.86322
Q63HR2	TNS2	-1.13471	0.331993	-0.24695	-1.433	0.437164	-0.40617	0.222879	-1.15909	0.037552	-0.00834	0.035485	-0.42091	-0.82448	-0.23889	0.36898	-0.68684	-1.09593	0.24319	-0.03369
Q63ZY3	KANK2	-1.45985	0.213293	-0.34114	-1.36354	-0.18385	-0.62204	-0.12716	-0.97588	0.368769	-0.41815	-0.79454	-1.56348	-0.4355	-0.93496	-0.07959	-0.1319	-1.44089	0.293788	0.183702
Q641Q2	WASHC2/	-0.00503	0.241518	0.239045	-0.07435	-0.25244	0.068276	-0.03687	-0.27025	-0.00613	0.23011	-0.38688	0.023478	0.60155	0.02553	-0.16339	0.230019	-0.01981	0.467874	0.530698
Q641Q3	METRNL	-1.02153	0.694852	1.058234	-0.76298	-0.34471	-0.29535	0.343081	-0.73597	-0.38361	0.604605	-1.67081	0.315184	0.053885	0.32677	-0.23823	-0.63762	-0.07823	-1.29687	-1.09248
Q643R3	LPCAT4	0.169689	0.194301	1.168584	0.157447	-0.31351	-0.23896	0.453714	-0.07663	0.530398	0.085585	0.451861	-0.0273	-0.00343	0.097905	-0.16632	-0.0577	0.237205	-0.49704	-0.69863
Q658P3	STEAP3	-0.66321	0.609027	0.577812	-0.60695	-0.44124	-1.59001	0.171087	-0.83445	0.063751	0.471295	0.711431	1.204404	-0.00509	1.064242	1.199337	-0.94367	-0.05538	-1.14802	-1.8473
Q658Y4	FAM91A1	-0.28497	0.35346	-0.17181	0.049405	-0.06475	0.263796	-0.7801	-0.31573	-0.37249	-0.47965	-0.05281	0.379953	0.691127	0.388019	0.115562	0.270647	0.421337	-0.71702	-0.53677
Q66GS9	CEP135	-0.60296	0.522414	0.387108	-0.2216	-0.31921	-0.17583	0.301846	-0.40183	0.071096	-0.71599	1.783377	-0.45576	-0.67787	0.08331	-0.73347	-0.17197	-0.2176	0.214786	-0.28845
Q66K14	TBC1D9B	0.304317	0.333355	-0.14336	0.314441	0.559283	0.54826	-0.1719	-0.23433	-0.58647	0.047512	-0.03123	0.079741	-0.80728	0.319433	0.342428	0.294234	-0.1739	-0.64923	-0.27869
Q66K74	MAP1S	-0.08185	0.407876	0.449855	0.002699	0.040507	-0.23182	-0.3459	0.190355	-0.33913	0.079382	-0.09081	0.228222	-0.45495	0.307754	-0.20572	0.430867	-0.08599	0.05163	0.003241
Q66K79	CPZ	-0.75259	0.611849	0.204259	-0.96294	-1.24159	-0.46732	0.540223	-0.70589	0.534351	-1.16749	-0.38464	-0.60008	-0.10559	-0.50623	0.605615	0.151822	-0.38652	-0.90499	-0.13658
Q66LE6	PPP2R2D	-0.13081	-0.33941	0.144788	-0.68278	-0.21023	0.221566	-0.33092	-1.03515	-0.22559	-0.14685	-0.05252	-0.23117	-0.497	0.137399	0.179116	-0.11483	0.238235	-0.15498	0.283623
Q66PJ3	ARL6IP4	0.616631	-0.41166	-0.55511	0.036497	-0.12914	0.28883	-0.1702	0.343432	-0.18224	-0.11005	0.612859	-0.63919	-1.34759	-0.64033	0.340671	-0.00963	-0.25671	0.214808	-0.6644
Q674X7	KAZN	0.804536	-0.25949	-0.23987	-0.71535	-0.41868	0.221132	-0.47686	0.930484	-0.15579	-0.52573	1.70201	-0.97269	-0.15084	-0.5798	1.205851	0.243434	-0.24369	0.474007	-0.09785
Q676U5	ATG16L1	0.063943	-0.10181	-0.05433	-0.49023	-0.22092	0.080463	-0.16337	-0.15926	-0.10454	0.018237	0.59432	-0.35968	0.673268	-0.11268	0.551523	0.322142	0.080047	-0.52347	-0.08825
Q684P5	RAP1GAP	-0.46452	0.319261	0.416744	-0.32478	-0.07033	-0.13237	0.228423	0.112449	-0.16882	-0.2678	-0.7489	0.371675	0.109639	1.001495	-0.59183	-0.41342	0.50561	0.099742	0.229335
Q687X5	STEAP4	-0.13883	0.070535	1.614616	0.008366	1.116018	0.403183	0.329431	-1.76513	0.076012	1.391856	0.070981	0.267624	-0.41374	0.479396	0.83291	-0.69007	-0.61501	0.325003	-0.08464
Q68CP4	HGSNAT	-0.29589	0.470247	0.574581	-1.33593	-0.15151	-0.41932	0.406566	-0.80765	0.375545	-0.27576	-0.17386	0.381439	0.149216	0.580441	0.096777	-0.41894	0.467258	1.917837	0.545071
Q68CP9	ARID2	0.667838	-0.74309	-0.03642	-0.11698	-0.07737	0.033622	0.141381	0.452936	-0.51214	-0.30742	-0.15374	-0.71607	-0.91522	-0.16256	0.232097	-0.52092	-0.21836	0.205622	-0.1834
Q68CQ4	DIEXF	0.323738	-0.07312	-0.74749	0.164358	-0.49812	-0.51319	-0.67307	0.577274	-0.21403	0.142018	0.614289	-0.25206	-0.25741	-0.26672	0.273413	-0.62322	-0.49725	-0.82613	-0.11533
Q68CQ7	GLT8D1	-0.71881	0.288943	0.539415	-1.02666	0.422575	0.050912	0.141728	-0.24196	-0.59804	-1.3299	0.213112	-0.49486	0.473879	-0.76557	-0.08238	-0.37491	-0.33426	0.530946	0.974892
Q68CR1	SEL1L3	-0.28749	0.283501	0.29536	-0.67991	-0.37322	-0.10698	0.731895	0.022632	0.489278	1.468758	-0.20439	-0.61264	0.91014	-0.6369	-1.11119	-0.45651	-0.78211	-0.05695	-0.05319
Q68CZ2	TNS3	0.128276	0.223806	0.154784	-0.49538	0.146597	0.326826	-0.5931	-0.54474	-0.54188	0.480509	-0.94866	0.246535	0.038686	0.384438	-0.3288	0.417316	0.166964	-0.61361	-0.94802
Q68CZ6	HAUS3	0.348953	0.178916	0.01496	-0.24649	0.325101	0.760597	0.13493	0.321927	0.079527	-0.02575	-0.12873	0.124249	0.34932	0.155024	-0.49722	0.423209	-0.01889	-0.0523	-0.16525
Q68D10	SPTY2D1	-0.32917	0.278836	0.298013	-0.58948	0.037505	0.363662	0.179539	-0.08303	0.039604	-0.36461	0.319268	-0.38008	-0.01939	-0.24018	0.19461	0.117797	0.860015	1.071493	0.301706
Q68D91	MBLAC2	-0.10028	-0.37175	-0.58111	-0.70056	-0.58934	-0.77028	0.485667	-1.27001	0.136303	-0.04598	0.85337	-0.42605	-0.67737	-0.31207	0.446681	0.264978	-0.30445	-0.09086	-0.64002
Q68DH5	LMBRD2	0.456458	0.015912	-0.17681	-0.3297	-0.27411	0.03576	-0.02371	0.066549	0.18865	-0.15522	-0.15927	-0.12319	-0.16966	0.244564	0.668616	0.235803	-0.23226	0.245834	-1.39275
Q68DK2	ZFYVE26	-0.28839	1.341226	0.199326	-0.3938	0.186517	-0.29199	1.031214	-0.27926	0.784948	0.132809	-0.33191	0.421341	0.997085	0.539472	-0.13067	0.220479	0.095143	0.059206	-0.04019
Q68DK7	MSL1	0.417374	-0.0311	-0.37655	0.459439	-0.29587	0.000154	-0.46404	-0.03501	0.137071	-0.44066	0.107074	-0.20282	0.460888	0.205175	0.314011	-0.02962	-0.14968	-0.63905	-0.82005
Q68DQ2	CRYBG3	-0.50009	0.408143	0.192523	0.066539	0.025249	-0.08375	0.22228	-0.79766	0.138483	1.02082	0.398583	0.106733	-0.16537	0.204826	0.050507	0.019438	-0.17646	0.466271	-0.08607
Q68E01	INTS3	0.158063	0.004756	-0.05184	-0.18039	-0.35442	-0.36126	0.240069	1.067832	-0.18115	0.23846	0.458906	-0.39726	-0.5943	-0.31293	0.15312	-0.46091	-0.23106	-0.3016	0.147105
Q68EM7	ARHGAP1	0.05506	0.104234	0.423599	-0.62478	-0.27684	-0.32149	-0.03341	-0.82984	0.176364	0.144267	-0.4723	0.191048	-0.1917	0.050539	-0.15743	-0.47543	-0.04851	0.312401	0.062188
Q69YN2	CWF19L1	0.142039	0.453084	0.339443	0.83768	-0.33565	0.325873	-0.07867	0.460707	-0.28198	-0.36125	-0.32017	-0.00042	-0.07409	0.197016	0.67054	-0.44641	0.48248	-0.18578	-0.33368
Q69YN4	VIRMA	0.071029	0.30546	-0.21786	0.262246	0.27975	0.64955	-0.03611	-0.00576	-0.64209	0.333979	0.253073	-0.19446	0.230358	-0.23641	-0.11655	-0.10416	-0.18097	-0.15685	0.062361
Q69YQ0	SPECC1L	0.148839	-0.06659	-0.15234	-0.41721	-0.4192	-0.0227	-0.00435	-0.20921	1.551581	-0.0994	-0.20621	-0.99392	0.322673	-0.89114	-0.46516	0.363919	-0.65579	0.112178	0.373031
Q6AI08	HEATR6	-0.0331	0.084785	-0.00146	-0.00658	-0.38353	-0.22759	0.023939	-0.61635	-0.06272	-0.54923	-0.23901	0.045549	-0.43614	0.09769	0.104725	-0.06632	-0.31082	-0.53016	0.51334
Q6AZY7	SCARA3	0.819176	-0.39412	-0.36656	-0.99959	-0.25041	0.065298	-0.39908	-0.51368	-0.20989	-0.35606	0.469668	-1.39543	0.099154	-1.23844	0.267988	-0.09526	-0.90067	-0.23217	-0.28233
Q6BCY4	CYB5R2	0.093561	-0.28074	0.033117	-1.33198	0.054981	-0.10607	-0.53085	0.021116	0.584716	-0.7082	0.723026	-0.51799	-0.06986	-0.79627	-1.0814	-0.878	-0.44091	-0.01108	0.480012
Q6BDS2	UHRF1BP	0.010163	0.875752	-0.00746	-0.28311	0.440294	0.872794	0.034302	-1.1312	0.160405	0.061782	-0.32838	0.582528	0.219772	0.504097	-0.43501	0.314515	0.63224	0.550493	-0.73364
Q6DD88	ATL3	-0.55916	0.520955	0.384881	-0.19217	0.127222	-0.23664	0.318089	-0.16002	-0.7574	-0.05771	-0.3058	0.736815	0.265586	0.591052	0.476539	0.663107	0.143382	-0.33841	0.09885
Q6DKI1	RPL7L1	0.476024	-0.19802	-0.26127	0.626046	0.593862	0.229543	-0.4722	0.221535	-0.15951	0.091103	0.589434	0.096722	-0.20386	-0.32954	-0.35951	-0.03076	0.05759	0.037921	-0.82055

Q6DKJ4	NXN	0.831239	-0.25847	-0.52625	-0.33671	-0.45948	-0.13594	0.340428	0.361963	0.058562	-0.34585	-0.21367	-0.38849	0.168002	-0.47298	-0.50334	-0.11585	-0.14757	-0.35824	0.651224
Q6DKK2	TTC19	0.449848	-0.26298	0.447861	-0.30482	0.207484	0.026641	-0.25372	0.021297	-0.24597	-0.08214	0.678813	-0.51787	0.214393	-0.1578	-0.01463	-0.31873	-0.85706	-0.56603	-0.76305
Q6DN90	IQSEC1	0.007946	0.210532	-0.25524	-0.81626	-0.20873	-0.5185	0.011222	-0.71677	-0.24494	-0.80554	-0.04768	-0.05793	0.149986	0.353631	-0.35839	-0.02007	-0.49555	-0.23765	-0.28096
Q6DT37	CDC42BP	0.761996	-0.98584	-0.00624	0.460959	-1.04526	-0.17487	-0.49813	-0.27586	-0.68796	-0.36597	0.197363	-1.03785	0.183865	-0.38138	-0.03086	0.244432	-0.18419	-0.46225	-1.59184
Q6EMK4	VASN	0.227779	-0.01516	-0.15563	-1.74298	-0.26695	-0.84275	0.912256	0.276168	0.224688	-0.40204	-0.45291	-0.48504	-1.25617	-0.80272	-0.10887	-0.58669	-0.56123	0.337164	-0.03757
Q6FI81	CIAPIN1	-0.42605	-0.05131	-0.83521	0.067713	-0.18847	0.078566	-0.30525	0.078353	0.249661	-0.0443	0.132727	-0.32097	0.410806	-0.19425	-0.02324	0.416945	0.447329	0.206856	0.60902
Q6GMV2	SMYD5	0.070883	0.244564	-0.06494	-0.69973	0.639207	0.06765	-0.04722	1.007795	0.26165	0.255321	0.534071	-0.13652	0.097015	-0.21415	-0.65109	-0.21252	-0.23359	-0.04801	0.9291
Q6GMV3	PTRHD1	-0.26538	-0.83976	0.051194	0.038306	-0.75542	-0.49332	-0.9542	1.08153	-0.25197	0.189205	0.246518	-0.27361	0.43039	-0.23045	0.29556	0.829425	-0.71172	-0.78087	0.227399
Q6GQQ9	OTUD7B	0.709417	-0.44824	-1.54359	-0.0296	-0.00122	-0.23705	-1.36511	-0.0086	-0.33724	-0.29816	0.274541	-0.69973	-0.07828	-0.33232	0.695924	0.168752	-0.61796	-0.80533	-0.10708
Q6GTX8	LAIR1	-2.0045	1.964249	1.196817	-0.80709	-0.19423	0.532086	0.950684	-1.25803	-0.14345	1.183073	-0.93668	0.835933	0.048487	1.889286	-0.71595	-0.64567	-0.01788	0.84486	-0.91792
Q6GYQ0	RALGAPA	0.283044	-0.73091	-0.26771	-0.55697	-0.17477	-0.42435	-0.15365	-0.13449	-0.05091	-0.51958	-0.01378	-0.27551	-0.41775	-0.02194	0.610237	-0.01613	-0.08468	-0.30476	0.131636
Q6I9Y2	THOC7	0.062639	-0.66569	-0.41411	0.304446	0.900335	0.937822	-1.09381	0.211998	-0.1679	0.418907	0.363536	-0.0714	-0.24945	-0.15345	0.176167	-0.35602	0.229581	0.346598	-0.27977
Q6IA17	SIGIRR	-0.4242	-0.10335	0.189253	-0.34865	1.683608	2.023625	-0.37358	0.307727	-0.37828	0.226373	1.015632	-0.28348	-0.79587	-0.12713	-1.01035	0.958255	-0.45773	-0.3323	-1.52669
Q6IA69	NADSYN1	-0.19285	-0.01419	0.627875	0.382909	0.545519	0.491009	-0.36698	0.768069	-0.08428	0.756436	0.089509	0.281448	-0.44588	0.695098	-0.10875	1.170668	-0.50543	-0.41667	-0.44238
Q6IA86	ELP2	0.085168	0.571866	-0.918	-0.10552	-0.0012	0.408164	0.998997	0.047973	-0.74582	-0.03671	0.3145	-0.35393	0.278943	0.034704	-0.42794	-0.33712	-0.27729	-0.34254	0.583014
Q6IAA8	LAMTOR1	0.208664	0.682987	-0.04162	-0.24971	0.00185	-0.25561	0.116068	-0.29844	1.077997	0.240274	-0.15476	0.288644	-0.08514	0.444287	-0.18313	0.277362	-0.11021	0.134721	-0.13343
Q6IAN0	DHRS7B	-1.25472	0.431482	-0.01233	0.089137	-0.09476	-0.14375	0.359971	-0.10067	-0.3009	-0.50234	0.898376	-0.06342	-0.6895	-0.37916	0.4883	-0.24501	-0.5003	-0.54317	-0.37458
Q6IBS0	TWF2	0.130747	0.489382	0.742891	-0.58138	0.595185	0.0843	-0.61732	-0.09651	-0.04933	0.855828	-0.53836	0.050324	0.595773	0.553925	-0.18393	0.174559	-0.10286	0.013882	0.273289
Q6IBW4	NCAPH2	0.668967	-0.07433	-0.0907	-0.2367	0.334845	1.516263	-0.20462	0.172907	-0.32947	0.083012	-0.40795	-0.23449	-0.21196	-0.06342	0.078076	-0.14631	-0.16429	-0.18693	-0.02633
Q6IC98	GRAMD4	-1.10756	1.349006	1.020846	-0.29718	0.614633	0.322872	0.656684	0.15133	1.364686	-0.27486	-0.58385	1.040406	0.578782	1.374097	0.261454	-0.78112	0.474238	0.890225	-0.51559
Q6ICG6	KIAA0930	-0.77271	0.362302	-0.1559	-0.38036	0.07646	-0.39477	0.296044	-1.06342	0.321061	-0.01155	-0.41606	0.4931	-0.24071	0.242996	-0.41177	-0.20386	0.4477	0.035702	-0.67157
Q6ICL3	TANGO2	-0.51974	0.122819	0.342867	-0.32347	0.730111	-0.924	-0.12691	-0.56294	0.524049	1.009819	-0.19584	0.086577	-0.13215	0.834833	-0.32821	0.022834	0.037299	0.521261	-0.30654
Q6IN84	MRM1	-0.87292	0.381021	-0.00897	0.262937	0.044752	-0.33175	0.254443	-1.54431	-0.21762	-0.36709	1.418385	0.318796	0.021664	-0.0558	0.020826	-0.28382	-0.47394	-0.49779	-0.45072
Q6IN85	PPP4R3A	0.613909	-0.51779	-0.10155	0.015573	0.481592	0.78768	0.080876	0.932338	-0.14292	-0.48764	0.134902	-0.40826	0.041071	-0.23718	-0.07212	0.010217	0.294436	-0.08269	0.130325
Q6IPR1	ETFRF1	0.128036	0.169483	-0.14158	-0.35564	-0.23588	-1.0248	1.342024	-0.49322	0.615335	3.484509	-0.13019	-0.12855	-0.64466	-0.44556	-0.0405	0.53396	-1.40609	-1.17784	-1.14392
Q6IQ22	RAB12	0.404988	0.140521	0.279977	-0.81047	0.697498	0.695674	-0.04506	-0.9948	0.125284	0.402033	-0.56956	0.808845	0.166504	0.475017	0.058089	-0.2162	0.776119	0.078565	-0.09271
Q6IQ23	PLEKHA7	0.013601	-0.25782	-1.12109	-0.67504	0.447967	0.39371	-0.36776	0.453294	0.040847	-1.02337	0.308624	-0.89757	-0.46262	-0.68231	0.494915	-0.18863	0.122716	0.413177	-0.76926
Q6IQ49	SDE2	0.344728	-0.46243	-0.38507	-0.31262	-0.83314	-0.31438	-0.94508	0.046828	-0.20393	0.213937	-0.16796	-1.14321	-0.4471	-1.08608	0.929619	-0.16186	-0.69828	0.054363	-0.13524
Q6ISB3	GRHL2	0.782506	0.085565	-0.02767	0.494594	-0.63456	-0.03529	0.230951	0.380783	-0.52664	-1.63605	0.43155	-0.36324	-0.13903	-0.63929	0.053402	0.019584	0.248113	-0.24455	-0.62044
Q6JBY9	RCSD1	-2.60178	1.241422	1.074218	-1.24113	0.803224	-0.98381	0.027652	-2.38449	-0.23498	1.487678	-1.37298	0.294031	-0.18728	1.649549	-0.08326	-0.76549	0.165679	0.512653	-1.1153
Q6JQN1	ACAD10	0.61271	-0.11709	-0.3667	0.068836	-0.79562	-0.78735	0.459028	-0.75757	-0.01464	-0.16859	-0.24936	-0.29032	0.032148	-0.11177	0.316603	1.631451	-0.36622	-0.34211	0.050592
Q6KB66	KRT80	-1.1419	1.266762	0.990307	0.006834	-2.48653	-1.88644	1.220553	1.016451	-0.54312	-1.08604	0.842499	-0.81709	-0.31303	-1.37153	-0.94154	-1.18737	-1.36757	-2.81006	-0.98357
Q6KC79	NIPBL	0.580524	-0.0679	0.019204	0.029962	-0.10636	0.164977	0.508618	-0.17292	-0.0296	0.116693	0.077581	-0.39028	-0.75534	-0.22364	0.180348	-0.07974	-0.05735	0.209523	-0.15024
Q6L8Q7	PDE12	0.05168	-0.23031	0.229082	-0.21544	-0.00975	0.028948	-0.2675	0.14086	-0.17487	-0.04233	0.696893	-0.24648	-0.26428	-0.07497	0.383025	-0.11788	0.294294	0.110173	-0.35898
Q6N022	TENM4	-1.48466	0.316686	-0.00364	-0.71609	-1.17402	-1.94787	-0.25286	0.946891	0.116037	-0.44736	0.493493	-1.13511	0.605995	-1.00859	-0.93293	0.401905	-1.0437	0.332855	0.996487
Q6N063	OGFOD2	-0.34038	0.015183	-0.45034	0.433559	-0.10772	-0.51715	0.197271	-0.77671	0.205427	-0.20798	0.446731	-0.06392	-0.26811	0.344047	-0.00642	-0.3221	-0.07828	0.191861	-0.56873
Q6N069	NAA16	-0.79693	0.549454	0.089537	-0.21104	-0.04828	-0.31236	0.274061	0.125974	5.098457	-0.36705	0.319758	-0.03953	0.138311	0.551752	-0.14977	-0.57464	0.014751	0.097968	0.123045
Q6N075	MFSD5	0.181815	0.660778	0.252648	0.010853	0.413803	-0.04809	0.645186	-0.20443	0.261191	-0.43616	0.158488	0.483052	0.175621	0.416518	0.342627	0.130979	0.683098	0.516032	0.735527
Q6NSJ0	MYORG	2.001805	-0.30449	-0.06104	-0.47953	-0.11902	-0.00515	-0.28463	-0.21869	-0.20629	-0.26585	0.590427	-0.35125	0.077651	-0.55796	-0.52943	0.010702	-0.19996	-1.04836	-0.04266
Q6NTF9	RHBDD2	1.021771	-0.37221	-0.0777	-1.08542	0.312209	0.503825	0.260833	1.14141	-0.24393	0.459969	0.516463	-0.93606	-1.36674	-0.44515	-0.3331	-0.85622	-0.91791	-1.6263	0.90177
Q6NUK1	SLC25A24	0.816356	0.711602	0.007999	0.386871	0.050478	-0.07253	-0.1534	-0.10636	-0.89895	-0.229	0.607533	0.028533	-0.33087	0.197073	0.42596	1.610712	-0.30709	-0.69647	-0.26202
Q6NUK4	REEP3	-0.18797	0.396806	-0.08078	0.124943	-0.12763	0.002473	-0.14983	-0.4159	-0.18651	-0.07588	-0.29195	-1.3171	-0.15274	-0.87839	-0.12031	-0.21939	-0.58327	-0.0072	0.599328
Q6NUM9	RETSAT	-0.8684	0.656547	0.0809	-0.41335	-0.20566	-0.39234	-0.46628	-0.46118	-0.01511	-0.46525	-0.45233	0.028702	-0.58566	0.267419	-0.6065	-0.38247	-0.09398	-0.20395	-0.83727
Q6NUQ1	RINT1	-0.47473	0.42856	0.376964	-0.03441	0.223495	-0.14147	-0.09381	-0.05671	-0.64706	0.406406	0.276076	0.289812	-0.21085	0.079373	0.309522	0.19983	0.394039	0.302175	0.570671
Q6NUQ4	TMEM214	-0.69169	0.548954	-0.03394	0.426695	0.39716	0.367308	1.518902	-0.25324	0.49043	0.035816	0.006405	-0.28876	0.445274	-0.28827	-0.2362	0.535504	-0.40257	0.145752	0.834358
Q6NV74	KIAA1211L	0.773652	-0.58291	-0.99357	-0.1902	-1.10335	-0.99315	-0.6053	0.660149	-0.49938	-0.62261	1.11359	-1.17755	-0.23421	-0.26624	1.661677	0.329778	-1.34378	-1.57797	-0.44367
Q6NVY1	HIBCH	0.428772	-0.56515	-0.50679	0.629527	-0.72847	-0.6626	-0.49164	0.58398	-0.474	-0.18964	0.526569	-0.57921	-0.16511	-0.51691	2.455165	1.252137	-1.2177	-0.57903	-0.35112
Q6NW29	RWDD4	-0.84356	0.267123	-0.14493	-0.40022	-0.17873	0.024966	0.229217	-0.14861	0.066962	0.211718	-0.22402	-0.20215	-0.46244	0.098592	-0.02565	0.615648	-0.41455	-0.51263	-0.07909
Q6NW34	NEPRO	-0.44571	0.726933	-0.01628	0.916823	-0.97027	-0.50661	0.433329	0.596023	-0.27476	-0.21635	-0.41968	-0.53857	0.446987	-0.30832	1.070768	-0.2372	-0.00615	0.796462	0.086109
Q6NWY9	PRPF40B	0.405263	-0.64067	-0.17145	0.321293	0.432236	0.343591	-1.30565	0.184594	0.056962	-0.0648	-0.0423	-0.11636	-0.0051	-0.32403	0.582629	0.423466	-0.11959	-0.60447	-0.25708
Q6NXE6	ARMC6	0.044317	0.482841	-0.02887	0.629195	-0.92566	-0.53531	-0.00369	-0.30231	0.009812	0.05918	-0.14655	-0.67754	-0.42963	-0.08457	-0.0863	0.122849	0.86004	0.078116	0.185419
Q6NXG1	ESRP1	0.413383	-0.25733	-0.62461	-0.62132	0.102197	0.398477	-1.01744	0.306699	-0.47008	-0.1888	0.166269	0.068243	0.420227	-0.43999	0.224567	0.847556	0.572778	-0.5168	-0.61187
Q6NXR4	TTI2	0.580557	-0.09008	-0.34851	-0.95273	-0.36031	0.663864	0.207517	-0.11112	-0.33509	-0.25271	-0.07648	-0.14737	0.429289	-0.0549	0.740576	0.025612	0.382326	-1.58076	0.353734
Q6NXT6	TAPT1	0.812014	-0.16036	-0.14909	-0.52662	-0.19608	-0.2535	0.036677	-0.59852	0.011045	-0.32177	0.113993	-0.05442	0.021036	-0.21393	0.315974	0.527922	-0.63692	-0.15092	-0.96319
Q6NY19	KANK3	-0.97299	0.31903	-0.011	-0.86261	-0.38408	-0.26037	0.524209	-1.19471	0.14195	0.141089	-1.04921	-0.61852	-0.36992	-1.92854	0.552235	-0.71846	-0.64632	0.655859	-0.72584
Q6NYC1	JMJD6	-0.08897	0.147434	-0.1058	-0.13224	0.347532	0.324352	0.092269	-0.23556	0.632607	0.092862	-0.37095	0.674521	-0.03217	0.372887	-0.31391	0.817328	0.673519	0.731036	-0.26859
Q6NYC8	PPP1R18	-0.4671	0.543297	0.673935	-0.37935	-0.3598	-0.37046	-0.68935	-0.92359	0.895642	1.249842	-1.34963	0.14043	0.563895	0.031666	-0.53648	-0.04235	0.154449	1.126993	0.185104
Q6NZI2	CAVIN1	-1.99671	-0.23796	0.073187	-2.449	-0.81556	-1.53176	-0.19239	-2.0279	-0.19594	-1.29697	-1.07924	-1.5259	-0.49522	-1.47573	-0.13109	-0.88608	-1.13769	0.909559	-0.47687
Q6NZY4	ZCCHC8	0.563992	-0.46912	-0.03537	0.089231	0.106083	-0.06673	0.029255	0.562207	0.203423	-0.44013	0.420639	-0.06411	0.063785	-0.22691	0.250497	-0.36179	-0.14472	0.300007	-0.12378
Q6P087	RPUSD3	0.773501	-0.28071	0.114781	0.330749	-0.18903	0.105134	-0.3227	-0.78169	-0.38794	0.041204	0.259795	-0.65915	-0.10437	-0.31812	-0.20559	-0.22258	-1.04523	-0.99597	-0.64984
Q6P158	DHX57	-0.04128	-0.11744	0.289919	-0.09323	-0.19958	-0.27933	-0.34802	0.586871	-0.08215	-0.18547	0.337572	-0.12503	0.144196	-0.48936	0.358662	0.110993	-0.22087	-0.28206	0.380545
Q6P161	MRPL54	-0.20992	-0.23976	-0.40955	0.63793	-0.29414	-0.25038	0.026953	-0.29987	-0.28989	0.688918	0.853729	-0.21103	0.302938	-0.3837	0.46176	-0.36655	-0.1231	0.079777	-0.70197
Q6P179	ERAP2	-1.70347	-1.31884	2.302449	-1.67574	2.399465	1.571621	0.153643	0.040071	-0.45561	-2.68655	0.49671	-0.63267	0.156871	-0.94374	-0.57016	1.906851	-1.47702	-3.30153	0.455993
Q6P1A2	LPCAT3	-0.18516	0.4517	0.114141	-0.21075	-0.19424	-0.73836	0.130537	0.183382	-0.34999	-0.16661	-0.82385	0.478307	0.013492	0.711391	0.960774	0.447878	0.091428	-0.0828	-0.30164
Q6P1J9	CDC73	0.775796	-0.04718	-0.20758	-0.45638	0.005231	0.132803	-0.05835	0.406072	-0.94929	-0.19767	0.058316	-0.32319	-0.21877	-0.17068	0.37393	-0.20972	-0.15455	0.133108	-0.20931
Q6P1K2	PMF1	1.117742	0.036887	-0.03467	-0.29504	0.312543	-0.12212	0.208114	0.925996	0.374577	0.018681	-0.39569	-0.24599	-0.28791	0.257837	0.223026	0.170771	-0.07862	-0.76547	-0.2364
Q6P1M0	SLC27A4	-0.45298	0.672674	-0.04188	-0.55307	0.179913	0.133319	0.870115	0.392096	-0.10579	-0.05513	-0.43981	0.470342	-0.16808	0.34668	-0.1056	-0.62104	-0.04428	-0.31301	0.097951
Q6P1M3	LLGL2	0.459178	-1.09071	-0.64808	-0.60179	-1.0194	-0.72078	-0.05455	1.109941	-0.18286	0.060884	0.19427	-0.33685	-0.21022	-0.20684	1.194155	0.109645	-0.00942	-0.33232	-0.37774
Q6P1N0	CC2D1A	-0.57753	0.126186	0.972929	-0.63916	-0.08561	0.165933	0.994145	2.081766	0.022657	0.269355	0.294661	-0.1684	-0.58626	-0.20462	1.950474	0.647765	-0.14124	-0.23175	-0.56833
Q6P1N9	TATDN1	-0.41945	-0.20792	-0.19168	0.298664	0.696755	0.01901	0.215706	0.13645	0.466179	0.832229	-0.1102	-0.14228	-0.09559	-0.19409	1.184838	0.906305	-0.01285	-0.54887	-0.84738
Q6P1X5	TAF2	0.125212	0.152481	-0.03556	-0.24256	0.316605	0.875036	0.199465	0.513551	0.40912	0.379717	0.062206	0.176804	0.279541	0.121281	0.44172	-0.19131	-0.07856	-0.16234	-0.11727
Q6P1X6	C8orf82	1.071325	-0.90902	0.051029	0.175543	-0.41575	0.074153	-1.09329	-0.58735	-0.17309	-0.40506	0.015563	0.215377	0.754539	-0.25108	1.255045	0.700855	0.297717	0.154738	-0.03801
Q6P2C8	MED27	0.371985	-0.01969	0.174021	-0.4225	-0.04144	0.423385	0.149518	0.434466	-0.13491	-0.55307	-0.08887	-0.1417	-0.01579	-0.0508	-0.10561	0.136064	-0.42622	-0.34458	0.345016
Q6P2E9	EDC4	-0.24947	0.208683	-0.0566	0.339988	0.291332	0.198	0.154891	-0.27294	-0.10515	-0.14691	0.12107	0.166137	-0.19519	0.189888	0.118875	0.662359	0.463696	0.382306	0.1118
Q6P2P2	PRMT9	-0.04915	0.049512	0.309899	-0.22401	0.622777	1.174583	-0.03661	0.074085	0.162081	-0.25389	0.04279	-0.33634	0.145447	0.39244	-0.62583	0.31946	0.509622	0.508362	0.165393
Q6P2Q9	PRPF8	0.702753	0.025454	0.10414	0.340554	0.125075	0.253513	-0.40928	0.455573	0.154704	-0.06992	0.350333	-0.10236	-0.29055	-0.19523	0.149472	-0.12993	0.039469	0.156266	-0.0708
Q6P3W7	SCYL2	-0.07997	0.234628	0.332585	0.198099	-0.12944	-0.289	-0.76849	0.121081	-0.43907	-0.07698	-0.10088	0.015571	0.04752	-0.13153	0.001429	-0.15498	0.018457	-0.14553	0.409078
Q6P3X3	TTC27	-0.62463	0.384824	-0.40696	0.648918	-0.08212	0.339636	-0.15084	-0.25068	-0.2426	-0.02324	-0.065	0.035238	-0.45025	0.095052	0.17078	0.571348	-0.41232	-0.88561	0.252485
Q6P4A7	SFXN4	-0.5546	0.056893	0.091077	0.481989	0.195782	0.237543	-0.05942	-0.24361	-0.26428	-0.39611	0.216058	0.314261	-0.03896	-0.2066	0.562999	1.678512	-0.037	-0.41018	-0.09965
Q6P4E1	CASC4	-0.37323	0.609983	-0.03256	0.230458	-0.30508	-0.35469	0.580773	-0.75506	-0.10446	-0.3571	0.128092	-0.79331	0.325458	-0.67354	-0.14354	0.63645	0.83833	1.499027	0.761369
Q6P4Q7	CNNM4	-0.16068	-0.34844	-0.15116	1.194853	-0.57704	-0.3987	-0.16358	0.734359	-0.35642	-0.59875	0.170177	-0.0434	-0.47027	0.23078	-0.34489	0.826529	0.669588	-0.5798	-1.09176
Q6P4R8	NFRKB	0.486052	-0.03034	0.196332	-0.16005	-0.02994	-0.04663	0.315492	0.275918	0.097913	-0.38726	-0.2276	0.113014	-0.09115	0.123068	0.266648	-0.37562	0.193673	-0.54166	-0.1219
Q6P587	FAHD1	1.067699	-0.24563	0.146294	0.206574	0.959918	0.481496	-0.35043	0.160814	0.063735	0.190002	0.568354	0.481073	0.021315	0.280509	-0.06502	0.694893	0.001459	0.309609	0.295832
Q6P5R6	RPL22L1	-1.19695	-0.07956	-0.18466	-1.31369	0.154046	-0.23775	0.034699	-0.94461	-0.03528	-0.31022	0.017335	1.890994	0.010673	0.290516	-0.31588	-0.4278	-0.12242	-0.06759	-0.12274
Q6P6C2	ALKBH5	0.495882	-0.02112	-0.02145	-0.02547	-0.31354	-0.63589	-0.01067	0.592701	-0.56332	-0.06675	0.369789	-0.15156	-0.0843	0.453552	-0.50703	-0.28527	-0.03938	-0.475	0.254662
Q6P996	PDXDC1	-0.25682	0.42474	0.034253	-0.26269	0.708136	0.799241	0.101083	-0.2878	0.247959	0.05049	-0.05987	-0.10485	0.790122	-0.32861	0.199544	1.055694	0.824982	1.316392	0.278532
Q6P9B6	TLDC1	-0.87615	0.530209	0.024336	-0.21356	-0.62185	-1.30115	-0.18162	-0.2773	-0.0581	-0.01549	-0.16877	-0.17922	0.092415	-0.33336	-0.00425	0.611237	-0.11408	0.08621	0.768215
Q6P9B9	INTS5	-0.1358	-0.02561	0.525528	-0.00196	-0.35467	-0.13145	-0.39962	0.308127	-0.23662	0.291985	0.629	-0.44434	0.050493	0.187939	0.325285	0.126786	0.128151	0.155077	0.066287
Q6PCB0	VWA1	-0.21084	0.232175	-0.05194	-0.44382	0.241783	-0.44514	-0.07282	-0.30174	-0.01897	1.060303	0.011173	-0.37025	-0.84971	-0.47924	-0.29714	-0.45751	0.905817	1.638831	0.345515
Q6PCB5	RSBN1L	0.655369	-0.15555	0.040665	-0.72003	0.573699	1.229644	0.137986	0.462413	0.115312	0.279428	0.249272	-0.47783	-0.26357	0.180005	0.772407	-1.5437	-0.60552	0.330343	-0.44212
Q6PCB7	SLC27A1	-0.93743	0.672674	-0.08362	-0.49461	-0.61873	-0.93645	0.532076	0.435035	-0.13683	-0.10697	0.956974	0.105304	-1.2287	-0.09737	-0.08624	-0.44225	-0.31278	-0.49114	-0.98631
Q6PCB8	EMB	-0.68447	0.365956	-0.02362	-0.34737	0.462908	-0.38808	0.138598	-0.21885	0.215399	0.03393	-1.17929	0.389566	-0.41434	0.913571	-0.67506	0.093272	-1.0507	-1.11333	0.102736
Q6PCE3	PGM2L1	-1.06747	-0.07554	-0.25884	-0.79417	-0.5229	-0.01276	-0.4844	0.158295	0.135291	-1.69452	0.501361	-0.81413	0.423	-0.10243	-1.1238	1.308777	-1.66316	-1.27955	0.604012
Q6PD62	CTR9	0.643005	0.013269	0.367928	-0.17844	0.141773	0.523632	0.052722	0.655444	0.048844	-0.18748	0.30097	-0.04287	-0.19708	0.153301	0.19835	-0.28514	-0.06302	0.115499	0.010981
Q6PD74	AAGAB	0.297051	0.303896	0.244326	-0.22342	0.60218	0.979292	-0.98671	-0.01915	-0.06157	-0.32178	0.009005	0.097539	0.280108	0.610323	-0.09497	0.541831	0.715978	-0.31141	0.688476
Q6PFW1	PPIP5K1	-0.50105	0.313166	0.009241	-0.06173	-0.38343	-0.16546	0.077495	-0.65975	-0.15855	-0.57523	0.579221	0.157334	-0.13733	0.478977	-0.0715	-0.21993	0.171266	-0.18173	-0.23418
Q6PGP7	TTC37	-0.48398	0.268054	-0.34422	0.48965	-0.33394	-0.27383	-0.44944	-0.33377	-0.31869	-0.42115	0.393838	-0.15541	-0.35037	0.00537	0.503387	-0.09646	-0.02557	-0.44115	0.318342
Q6PI48	DARS2	0.334055	-0.60093	-0.44559	1.130934	0.14694	0.135679	-0.61468	0.570718	0.051689	0.695054	-0.10733	0.037437	-0.05849	0.267107	1.862213	1.113046	-0.72257	-0.92884	-0.57474
Q6PI78	TMEM65	-0.37776	0.800581	0.367155	-0.12231	-0.76102	-0.61145	-0.80125	-0.76855	-0.2106	-0.60865	-0.96195	0.198482	0.572687	-0.32791	0.862564	0.799113	-0.11051	-0.11744	-0.15462
Q6PII3	CCDC174	1.030629	-0.12251	-0.34861	-0.0556	-0.68664	-0.3516	-0.40703	0.452833	0.087898	-0.69167	0.30167	-0.54947	-0.13731	-0.35028	0.011912	-0.18438	-0.58384	-0.05828	-0.17663
Q6PIJ6	FBXO38	-1.11302	0.475994	-0.04511	-0.42831	-0.8437	0.560441	0.336507	-0.78075	0.124117	-0.32085	0.618004	-1.04064	0.573363	0.117545	0.526622	0.721932	-0.77684	0.123437	0.356425
Q6PIU2	NCEH1	-1.21668	1.851295	1.880767	-0.49311	0.554372	0.288524	0.733174	-1.0443	0.73213	-0.43439	-0.47089	1.400735	-0.46521	0.746738	0.231337	-0.38922	0.480789	0.455784	-1.03093
Q6PIY7	PAPD4	0.3713	-0.29116	-0.24722	0.361156	0.131122	-0.01709	-0.4892	-0.30886	-0.17341	-0.49422	0.889939	-0.04493	-0.08563	0.173617	0.491167	0.167904	0.130313	0.502808	-0.31754
Q6PJ69	TRIM65	0.761837	0.225028	-0.08468	-0.58066	-0.09386	-0.01792	0.267264	-0.01227	0.299066	0.380874	-0.69198	0.029701	0.09662	0.290896	-0.20258	0.105769	-0.06243	-0.36957	-0.17682
Q6PJF5	RHBDF2	-0.09501	0.843483	0.323034	-0.37287	-0.07891	0.089031	0.073006	-0.38814	-0.33203	0.531005	-0.04652	0.895958	0.16854	0.574917	-0.28527	-0.37051	0.043432	-0.5649	-0.01735
Q6PJG2	ELMSAN1	0.559915	-0.13407	0.416733	0.556672	-0.14372	-0.24241	-0.04074	0.621839	0.012146	0.165068	-0.1327	-0.10433	0.269382	-0.1771	0.015451	0.145981	0.23951	0.403005	0.15931
Q6PJG6	BRAT1	0.099349	0.537218	0.092919	-0.55296	0.013045	0.354715	-0.27189	0.192653	-0.0102	-0.10988	0.25171	-0.44091	-0.37684	-0.23375	-0.42564	-0.13749	0.191392	-0.00885	0.122004
Q6PJI9	WDR59	-0.62932	0.123966	-0.22609	-0.07976	0.107714	0.319726	-0.00092	-0.03601	-0.17367	0.230454	0.605052	-0.46233	-0.26919	-0.10576	0.082257	-0.46328	-0.26634	-0.59488	-0.19845
Q6PJT7	ZC3H14	0.58626	-0.07689	-0.29841	0.03932	-0.06727	0.560575	0.074151	0.611875	0.550387	-0.0887	0.636022	-0.7772	-0.17218	-0.83193	0.45815	0.191704	-0.56671	0.323184	0.094188
Q6PJW8	CNST	0.457628	0.196699	-0.34797	-0.02023	-0.08236	0.193834	-0.21636	0.130376	0.268295	0.054472	-0.85873	-0.56307	0.114413	0.351249	0.522445	-0.09054	0.330064	0.685076	-0.21294
Q6PK18	OGFOD3	1.006581	0.156508	0.000456	0.961358	0.184847	-0.04217	-0.05488	-0.02675	-0.61787	-0.34237	-0.53934	-0.20845	-0.1385	-0.05904	-0.395	0.045717	-0.30467	0.548805	0.268598
Q6PKC3	TXNDC11	-0.13076	0.460857	0.2356	-0.38323	0.784204	0.504011	0.696475	-0.14445	0.017595	-0.08439	-0.42307	0.034165	0.197812	0.078476	0.157627	-0.34858	0.601392	0.777936	0.328192
Q6PKG0	LARP1	-0.3557	-0.27558	-0.35948	0.401831	-0.40324	0.133496	-0.46271	-0.14222	-0.30351	-0.37799	0.314796	-0.54427	-0.0975	-0.5691	0.408274	0.176979	-0.07631	-0.14163	-0.45738
Q6PL18	ATAD2	1.058768	-0.64706	0.228235	-0.33067	2.320881	2.379118	0.415218	0.308951	1.059088	0.353576	-1.13609	-0.17684	-0.21144	-0.45531	-0.11992	2.241556	0.06755	-0.08907	-0.86631
Q6PL24	TMED8	-0.55015	0.026046	0.085081	0.049516	-0.26761	-0.56459	0.176694	-0.36543	-0.21474	0.298007	0.068613	0.440736	0.073891	0.897143	0.036831	-0.07605	0.393971	-0.23061	-0.18531
Q6PML9	SLC30A9	0.08674	-0.21317	0.032708	-0.06424	-0.84738	-0.90859	-0.21564	-0.76562	0.088205	0.028897	1.06065	-0.42901	-0.47653	-0.08573	0.311348	1.082605	-1.0246	-0.77216	-0.82333
Q6Q0C0	TRAF7	-0.13989	0.654685	0.099044	0.054545	0.178879	-0.09973	0.259452	-0.5078	-0.18986	-0.40288	-0.16125	0.384297	0.447085	0.336495	-0.02122	-0.13603	0.473412	0.064406	-0.04869
Q6QNY0	BLOC1S3	0.535347	-0.09305	-0.0157	-0.58533	0.39587	0.503389	-0.23156	-0.45126	0.069593	-0.01999	0.333086	-0.04103	0.063352	-0.41943	0.030062	0.013313	-0.166	0.061532	-0.02979
Q6R327	RICTOR	0.102662	0.491995	-0.1145	-0.27741	-0.34732	-0.19945	0.551632	-0.36254	0.308157	-0.13607	-0.28097	-0.13281	0.421837	-0.06214	0.643651	-0.05067	-0.01217	-0.38535	0.345113
Q6RFH5	WDR74	0.263001	-0.1498	-0.03247	0.164728	-0.12135	-0.00619	-0.53619	0.223473	-0.36361	-0.44112	0.011782	-0.22603	-0.01784	-0.21873	0.469066	0.060792	-0.0468	-0.07641	-0.5726
Q6RW13	AGTRAP	-0.25134	0.39926	-0.55009	-0.86606	0.326064	0.171141	-0.11117	-0.03438	0.06106	0.60572	-0.22752	0.647188	-0.24131	0.825325	-0.89431	0.376627	0.452247	-0.21975	-0.2947
Q6SPF0	SAMD1	-0.06964	-0.3683	-0.43	-0.31934	-0.70267	-0.05616	-0.56761	0.889152	0.422308	-0.01231	-0.21014	-1.06189	-0.02711	-0.23068	0.640048	0.130196	-0.21689	-0.06469	-0.40239
Q6STE5	SMARCD3	0.452201	-0.06615	-0.12771	-0.24737	-0.90636	-1.0026	0.491918	0.698164	-0.24448	-0.6153	-0.19584	-1.11117	-0.39743	-0.54496	0.126942	-1.74309	-0.19725	-0.49544	-0.26213

Q6SZW1	SARM1	-0.2486	0.313769	0.112595	-0.64521	-0.48785	-0.75988	0.234364	-0.58717	0.08051	-0.16116	-0.13043	-0.42535	-0.58172	-0.1901	-0.70385	-0.39793	-0.37872	0.020159	1.033932
Q6UB35	MTHFD1L	0.571463	-0.30009	0.370867	0.777662	0.128408	0.131999	-0.06621	-0.51145	0.245364	-0.42393	0.587628	0.317318	0.286699	-0.53041	-0.46252	1.598834	0.53452	0.361282	0.0665
Q6ULP2	AFTPH	-0.85612	0.074523	0.046679	-0.15727	-0.49417	-0.23047	-0.02948	0.357059	0.122693	-0.24567	-0.12144	-0.07303	0.054797	0.135471	0.457286	-0.11133	0.270068	-0.56848	-0.28464
Q6UN15	FIP1L1	0.718461	-0.40757	0.058678	0.183689	-0.30343	0.363156	0.012703	0.182366	0.707867	-0.61461	0.630972	-0.41397	-0.20291	-0.2542	-0.06561	-0.08494	-0.1742	0.4675	-0.23112
Q6UUV7	CRTC3	-0.61199	0.030334	0.04654	0.052087	-0.75273	-0.76727	-0.17188	-0.16491	0.153603	-0.17588	-0.82368	-0.35627	0.062269	-0.20888	0.356175	0.057748	0.146869	-0.50537	0.051216
Q6UUV9	CRTC1	0.080446	0.403661	-0.15633	-0.03928	-0.41045	-0.64889	-0.2633	-0.16736	-0.21812	0.111579	0.317455	-0.0286	-0.10782	1.021003	-0.2399	0.229739	0.112213	-0.04526	0.181831
Q6UVK1	CSPG4	-0.53915	0.325402	-0.48579	-0.99685	-0.402	-0.14828	0.321296	-0.36421	-0.35873	0.176815	-0.19947	-0.70386	0.196739	-0.1766	0.448645	0.041975	-0.66392	-0.3491	0.165117
Q6UVY6	MOXD1	-0.40057	-0.39038	-0.60376	-1.54593	-1.23043	-0.76992	-0.20305	-2.18734	0.476288	-0.3602	-2.03567	-2.58955	-0.68061	-1.42436	0.00055	-1.05771	-1.16478	-0.65266	0.822018
Q6UW02	CYP20A1	-0.47199	0.387745	0.142715	-0.40278	0.448536	0.205875	0.565734	-0.03446	0.229887	-0.5391	0.085937	-0.29586	0.048271	-0.04583	0.643173	0.198125	-0.27698	-0.01433	-0.24855
Q6UW63	KDELC1	-0.37078	0.46065	0.535924	-0.97415	1.157685	1.159393	-0.46194	-0.05893	1.429206	0.218762	-0.45354	-0.50511	0.423242	0.35645	-0.1226	-0.31521	0.268928	1.178897	1.884215
Q6UW68	TMEM205	-1.14026	-0.01978	0.061284	-0.31556	-1.42922	-0.7655	0.348256	0.215051	-1.01132	-0.72759	0.933584	-0.12627	-0.48424	0.019491	-0.00813	-0.65749	-0.33183	-0.95158	-1.20422
Q6UWE0	LRSAM1	0.375473	0.062291	0.214131	-0.57232	0.144395	-0.07551	-0.94631	0.342226	-0.87007	0.030897	0.406554	-0.18574	-0.20901	0.128749	-0.12128	-0.6201	-0.42276	-0.26016	-0.09817
Q6UWH4	FAM198B	-1.81875	0.917781	-0.11684	-0.80927	-0.00534	-0.04864	0.145964	-0.87882	0.287505	0.505883	-1.06867	-0.10307	0.203715	-0.03024	-0.82266	0.239203	-0.9091	-0.52312	0.983605
Q6UWJ1	TMCO3	-0.47841	0.089952	-0.09096	-0.56913	0.202167	0.050132	-0.33952	-0.16612	0.043209	1.010447	0.545674	-0.29128	-0.10272	-0.19622	0.192259	-0.588	0.411456	0.75057	0.326381
Q6UWP7	LCLAT1	0.397448	0.950029	0.084399	0.498966	-0.18296	0.053854	-0.09059	0.713989	0.040847	-0.23288	-0.08164	0.01929	0.921877	0.080402	0.476292	0.53187	-0.14346	-0.55336	0.065587
Q6UWP8	SBSN	-0.63822	2.522128	-0.19585	-0.98224	-1.28063	-0.95895	1.877638	-0.9274	0.171341	0.177512	0.413365	-1.01498	-0.23022	-0.45349	-0.28316	-0.60885	-0.69993	-0.59574	0.503443
Q6UWR7	ENPP6	-1.02725	1.4598	0.738305	-0.82267	0.175186	-0.15128	-0.02578	-3.39567	-0.34594	-0.78417	-0.14137	0.355526	-0.28185	0.329933	-1.75976	-0.5127	0.13031	-1.08467	-1.27644
Q6UWY5	OLFML1	-3.94668	-1.29583	-1.33298	-2.78408	-1.84935	-2.70761	-0.4771	-3.14754	-0.18752	-1.56814	-0.66112	-2.01423	-3.24508	-1.31187	0.337769	-1.65766	-1.1747	-0.35548	-1.39423
Q6UWZ7	ABRAXAS	-0.0232	-0.21171	-0.4526	0.394207	-0.25843	0.19754	-0.34268	0.467635	-0.12175	-0.83018	0.46815	-0.84687	0.028186	-0.67076	0.432323	0.557071	-0.92665	0.004183	-0.46622
Q6UX04	CWC27	1.236509	-1.19359	-0.1459	0.238015	-0.37011	-0.16036	-0.54191	0.634629	-0.22475	-0.03999	0.333013	-0.63598	-0.28323	-0.54063	0.744767	-0.07271	-0.11787	0.326483	-0.28569
Q6UX06	OLFM4	-1.60928	-0.92579	0.445026	-2.06051	-0.61549	-1.99598	0.04947	-3.7496	0.346231	0.174638	-1.49863	0.004621	-2.28644	0.497321	-0.98983	-2.02183	1.660517	-1.98466	-1.03565
Q6UX07	DHRS13	0.19282	-0.18195	-0.0581	-0.0382	0.058745	0.833648	-0.2848	-0.22233	-0.16578	-0.16601	0.616481	-0.11256	-0.06346	0.299132	-0.17516	0.339029	0.244868	0.122052	-0.94127
Q6UX53	METTL7B	-0.21546	0.08721	-1.02912	1.0117	-0.47063	-0.24465	0.236358	-0.42102	-0.56043	-0.72612	1.38571	0.048135	-0.96526	1.114166	-0.76294	-1.13205	-0.14365	-0.94874	-1.47614
Q6UX71	PLXDC2	-0.92408	1.205213	-0.05319	-1.96359	0.116051	0.045437	0.584294	-0.44353	0.606853	-0.02071	-0.84844	-1.01324	-0.05564	-0.48441	-0.36782	-0.08523	-1.03336	0.086508	1.610037
Q6UXD5	SEZ6L2	0.230977	0.275612	-0.01469	0.389105	-2.0218	-0.56967	2.008758	0.499596	-0.28688	0.465861	0.249924	-0.79409	0.04226	-0.06548	-1.42391	0.374725	0.609175	1.185282	-0.05732
Q6UXI9	NPNT	-0.70459	-0.55419	-0.76821	-1.5326	-2.32214	-1.53501	-0.39446	-1.62244	0.171695	-2.49268	-0.8373	-1.51798	-1.30034	-0.73068	-0.77648	-1.23379	-1.5299	-0.72159	-0.10085
Q6UXN9	WDR82	0.780139	-0.5427	0.015697	0.116552	0.178242	0.220689	-0.64651	0.211317	-0.80996	-0.33621	0.284911	-0.36524	0.028464	-0.03276	0.103044	-0.456	-0.03704	-0.02383	0.01819
Q6UXV4	APOOL	-0.02929	0.097827	0.442637	-0.51285	-1.08655	-0.89301	-0.03333	0.009892	0.202713	0.16617	0.164643	-0.74033	-0.33775	-0.43682	1.541934	0.778488	-0.4529	-0.65073	-0.29726
Q6V0I7	FAT4	-0.38206	0.08813	-0.17927	-0.30452	-0.1264	-1.21193	0.12309	-0.81677	0.200031	-0.01218	0.039644	-0.13329	0.286916	-0.53429	-0.56484	0.321886	-0.00142	0.060977	0.044574
Q6V1X1	DPP8	-0.21568	0.186214	0.119675	0.029441	0.055559	0.30627	-0.29006	-0.49191	-0.10585	0.454089	-0.31455	-0.08763	0.009174	0.369596	-0.07064	-0.1783	0.295059	-0.1158	0.179102
Q6VMQ6	ATF7IP	0.677354	-0.17855	0.172233	-0.43458	-0.038	-0.36543	-0.44295	0.590861	-0.68883	-0.06724	0.002939	-0.79113	0.067408	-0.60463	0.250996	-0.56868	-0.46721	-0.06698	-0.03112
Q6VN20	RANBP10	0.210921	-0.2664	0.048191	0.187716	0.155206	0.394577	-0.58031	-0.28293	0.005973	-0.02075	0.687047	0.04961	0.005168	0.329018	0.114046	0.14976	0.405283	-0.04975	-0.05804
Q6VY07	PACS1	-0.38245	0.10729	-0.08429	-0.63958	-0.01076	0.057667	-0.23569	-0.22165	0.543919	-0.21883	0.205138	-0.13056	0.284952	-0.02184	0.012421	0.061593	-0.39281	-0.01931	0.380212
Q6WCQ1	MPRIP	-0.81966	-0.19173	-0.38876	-0.73351	-0.99983	-0.80977	-0.36573	-0.49876	0.00359	-0.18195	0.474383	-1.12458	-0.77435	-0.86549	-0.39531	-0.11775	-0.50138	-0.17616	-0.08595
Q6WKZ4	RAB11FIP	-0.57429	0.62989	-0.10862	-1.26899	-0.76384	-0.02144	0.098985	-0.60114	0.046052	0.502612	-0.1886	0.346098	0.056198	0.805094	0.539578	-0.77139	0.634889	0.388648	0.452375
Q6XQN6	NAPRT	1.621397	-0.49894	-0.64372	-0.19228	-0.85803	-0.94362	-0.5056	-0.49475	0.008177	0.155164	-0.96514	0.814919	0.874461	0.676399	-0.47011	-0.16154	0.595969	-0.3073	-0.94719
Q6XUX3	DSTYK	-0.08524	0.181049	0.494174	-0.30493	0.922063	0.476842	0.146331	0.000504	0.154932	-0.10375	-0.03551	0.219806	0.148488	0.881747	-0.37357	-0.19787	0.580597	0.425466	-0.1681
Q6XZF7	DNMBP	-0.68983	0.379711	0.158575	-0.47141	-0.32703	-0.78074	0.146825	-0.3035	0.186854	0.446689	-0.19558	0.021824	0.238198	0.308543	-0.44129	-0.37584	-0.06458	0.596698	0.73859
Q6Y1H2	HACD2	0.045521	-0.10772	-0.3182	0.815025	0.567601	0.878047	0.558557	0.649255	-0.25418	-0.3839	0.016961	-0.27762	-0.03855	-0.86008	2.863822	0.645142	0.262861	-0.6459	-0.82777
Q6Y288	B3GLCT	0.34979	0.103862	0.450056	-0.60474	0.747802	0.671022	0.005556	-0.55172	0.48291	0.680787	-0.1366	-0.08988	0.167335	-0.08381	1.243013	0.127912	0.979639	0.373788	0.48699
Q6Y2X3	DNAJC14	0.236003	0.305956	0.295224	-0.19191	0.269756	0.077524	0.194455	-0.32954	0.195012	0.106372	-0.29586	0.321186	0.094969	0.561806	0.599842	0.16991	0.178559	-0.1654	0.255238
Q6Y7W6	GIGYF2	0.139789	0.130939	0.196407	-0.77181	0.002033	0.173414	0.488735	-0.04915	0.078909	0.311486	-0.12173	-0.23255	0.149413	-0.23148	0.282689	0.352067	-0.15858	0.088703	0.016024
Q6YHK3	CD109	-0.61189	0.11355	-0.39552	-0.86383	-0.39422	-0.71983	-0.25084	-0.21158	0.70324	-0.34172	-0.67489	-0.25072	1.456141	-0.79973	-0.69419	0.736153	-0.76233	-0.13818	0.61828
Q6YHU6	THADA	-0.21831	-0.02972	-1.85375	0.205927	-0.09406	-0.34097	-0.53508	0.315294	-0.14903	0.054597	0.095715	0.086995	-0.69595	-0.17439	-0.41962	-0.84212	-0.01132	-0.93429	-0.22
Q6YN16	HSDL2	1.000996	0.179629	-0.2745	-0.02107	1.523628	1.390975	-0.0244	-0.85069	-0.37971	-0.68771	0.842319	-0.28019	-0.69442	0.341816	0.377239	-0.19442	-0.66834	-0.51235	-1.02
Q6YP21	KYAT3	-0.33186	-0.55029	-0.03393	0.603852	-1.02865	-1.20226	-0.24953	0.003327	-0.3891	-0.16633	0.193876	-0.13758	-0.41759	-0.46729	0.768832	-0.17861	-0.88198	-0.65193	0.269561
Q6ZMG9	CERS6	1.070444	0.400533	0.148626	-0.00024	0.029425	0.335767	-0.00665	0.697181	-0.09371	-0.48481	-0.27636	0.698809	0.156079	0.166442	-0.8016	-0.08177	0.117605	0.223828	0.518777
Q6ZMI0	PPP1R21	0.07282	0.451962	0.34822	-0.73925	-0.24822	-0.48679	-0.23363	0.091352	-0.34698	0.195939	0.008001	-0.06438	0.363789	-0.05415	-0.13321	-0.29481	-0.38208	-0.0882	-0.11184
Q6ZMP0	THSD4	-1.04629	-1.64289	-0.98912	-0.69013	0.088923	0.035062	-0.63863	-1.48661	-1.02804	-2.28221	1.559548	-0.89777	-2.00704	-1.1118	0.489451	-1.29705	-1.28184	-1.22386	-0.82882
Q6ZMZ3	SYNE3	-1.0975	0.256856	0.193276	-1.09799	-0.06632	-0.78648	0.586589	-0.57459	-0.17714	0.343383	-0.8847	0.121728	-0.23852	0.412235	0.261654	-0.74086	-0.32691	0.6	-0.61573
Q6ZN28	MACC1	1.325844	-0.39953	-0.00437	0.627559	-0.36926	-0.5356	0.145336	0.476745	0.334014	-0.27292	0.092416	0.157724	-0.01619	0.108795	1.013451	-0.20159	-0.57683	0.004056	-1.36002
Q6ZNB6	NFXL1	0.262408	-0.17709	-0.56624	-0.07994	-1.03097	-0.1477	-0.03326	-0.84509	-0.26513	0.11059	1.19087	-1.95451	-0.0591	-0.59453	-0.34281	0.310053	-1.02599	0.146037	-0.44288
Q6ZNC8	MBOAT1	1.673008	-0.43177	-0.26653	0.051657	1.123229	-0.07593	-0.25119	0.525445	-0.04104	-0.87093	0.667056	0.465769	0.062588	-0.20882	-0.24184	0.49277	-0.21631	-1.3567	-1.90756
Q6ZNJ1	NBEAL2	0.073757	0.268912	0.361778	-0.12059	0.156554	-0.1388	0.040498	-0.61142	0.288807	0.648432	-0.4744	1.093722	0.128136	1.529932	-0.3266	-0.33172	0.403254	-0.488	-1.07239
Q6ZNL6	FGD5	-1.15152	0.214551	-0.03783	-0.82088	-0.25609	-0.30825	-0.29735	-0.16159	0.073136	0.330369	-0.24963	-0.28983	-0.13177	-0.7132	-0.53446	-0.39867	-0.32269	-0.49016	-0.94204
Q6ZNW5	GDPGP1	-0.35876	0.155236	0.104105	-0.40115	-0.21941	-0.5796	-0.32846	-0.04852	-0.02968	-0.13977	0.329719	-0.42595	-0.05228	0.139506	0.02731	-0.60735	-0.33952	0.039882	-0.47962
Q6ZPD9	DPY19L3	-0.55754	0.261626	0.120377	-0.36559	0.165297	-0.09605	0.019635	-0.2731	-0.14681	0.578009	0.431327	-0.35006	0.039534	0.306319	0.183613	-0.11491	-0.07998	0.631758	0.518017
Q6ZRP7	QSOX2	0.129797	0.359232	0.050747	-0.62851	-0.16056	-0.53417	-2.48225	-0.18561	0.084573	0.185562	-0.13605	0.049091	0.377392	-0.2556	0.290961	-0.02408	-0.07592	0.101818	0.593232
Q6ZRS2	SRCAP	0.637793	0.206563	-0.3867	0.042447	0.394246	0.627182	-0.26504	0.21504	-1.12948	-0.0879	-0.34811	-0.31099	0.195279	-0.33816	-0.00529	0.034055	-0.29468	-0.00671	-0.1729
Q6ZRV2	FAM83H	0.736159	-0.55114	0.272785	-0.46774	-0.77254	-0.33296	0.010806	-0.08474	0.326128	-0.73041	-0.03326	-0.17066	0.396617	0.006633	0.724991	-0.02825	-0.28625	-0.50956	-0.98922
Q6ZS17	RIPOR1	-0.93151	0.513862	0.377289	-0.46338	0.24982	-0.18175	0.068509	-0.59478	0.199326	0.6002	-0.87044	0.314646	0.262722	0.221067	-0.36142	0.040631	0.248399	0.433504	0.396502
Q6ZS30	NBEAL1	-0.21552	-0.38855	-0.17852	-0.25204	-0.18238	-0.34394	-0.25638	-0.5272	0.717329	-1.4764	-0.51807	0.127448	0.397669	0.632005	-0.06852	0.160619	0.576774	0.140616	0.086793
Q6ZS81	WDFY4	-1.27405	2.631776	0.822139	-0.48786	0.618351	0.100557	0.368413	-1.17108	0.136902	1.471369	-1.4445	0.890971	-0.08058	1.322708	-0.18914	-1.01886	0.428644	0.100251	-1.08506
Q6ZSR9		-0.68606	0.089059	0.177034	-0.49455	-0.41839	-0.70167	0.162053	0.228493	0.240709	0.686992	-0.60102	-0.08249	0.650611	0.248135	2.369047	0.414452	-0.33936	0.279425	0.115489
Q6ZSS7	MFSD6	0.057383	-0.1946	0.06913	-0.78473	0.005069	-0.63121	0.444644	0.102473	0.089567	0.590918	-0.25139	-0.47952	-0.57038	-0.84338	0.136968	0.643252	-0.07175	-0.22942	-0.43696
Q6ZSZ5	ARHGEF1	-0.69884	0.795863	0.225937	-0.75809	-0.27739	-0.21024	-0.10565	-0.37594	0.142707	0.53476	-0.7142	0.282595	0.237312	0.689755	-0.68423	0.31599	0.091026	0.494097	0.012813
Q6ZT07	TBC1D9	-0.28829	-0.10164	-0.19774	-0.4305	0.140906	-0.08962	-0.16208	-0.20731	0.178227	-0.29768	-0.75427	0.172637	0.1574	0.259563	-0.30782	-0.21442	0.606546	-0.85204	-0.09188
Q6ZT12	UBR3	0.620369	0.306874	-0.95349	-0.3372	-0.49861	-0.55687	0.046011	-0.10525	0.398487	0.1867	-0.09074	0.010012	-0.17064	-0.22971	-0.02409	0.875663	-0.07753	-0.3267	-0.1923
Q6ZT21	TMPPE	0.053431	-0.28148	-0.74868	0.320148	0.13754	-0.31217	1.819172	-0.63048	-0.02225	-0.14025	-0.34044	0.630148	-0.32764	0.378913	-0.05711	-0.11789	-0.01681	-0.18786	-0.21995
Q6ZU35	KIAA1211	-0.38727	-0.61788	-0.42575	-1.15171	-0.81811	-2.37418	-0.29445	-0.06993	0.547363	-0.90408	-1.41268	-1.24596	-0.46888	-0.36197	-0.08616	-0.40304	-1.01348	-0.3228	0.258228
Q6ZU65	UBN2	0.286906	-0.26535	-0.18992	0.514642	-0.64554	0.220802	-0.17544	0.810214	-0.01236	-0.22301	-0.15426	-0.27836	-0.11402	-0.81289	0.632003	0.137506	-0.27132	-0.40527	-0.00423
Q6ZU80	CEP128	-0.38841	0.534813	-0.20545	-0.57058	-0.66901	-1.05205	-1.14276	0.277367	0.040847	-0.64964	0.920832	-0.4035	-0.50254	0.121937	-0.19299	-0.73511	-0.31871	0.283513	-0.19135
Q6ZUJ8	PIK3AP1	-1.32844	1.334944	0.654489	-0.85342	-0.40824	-0.81746	0.152518	-0.98249	-0.16175	0.848819	-1.76821	2.199566	0.696576	1.899456	-0.39099	-0.69945	1.194312	-0.49512	-1.13731
Q6ZUM4	ARHGAP2	-0.70625	1.196316	1.073741	-0.16669	-0.0976	-0.34151	-0.21059	-1.02547	-0.65382	0.819235	0.254258	0.757778	0.027113	0.686466	-0.11763	0.487849	0.574329	0.138404	-0.68023
Q6ZUT6	CCDC9B	0.016835	0.378748	0.071983	-0.96915	-0.22888	-0.50945	0.093588	-0.11957	-0.55052	-0.32116	0.305365	-0.1259	0.298653	-0.40374	-0.60523	-0.66555	0.136327	-0.50836	-0.9767
Q6ZUX7	LHFPL2	-1.94651	0.510057	-0.00358	-0.43136	-1.09878	-0.63306	0.31541	-1.14023	0.350144	0.11585	-1.09101	-0.67364	0.322064	0.106948	0.64826	-0.40823	0.034165	0.187478	0.539334
Q6ZVK8	NUDT18	-0.50366	-0.23201	0.395881	-0.09322	0.701071	0.387259	-0.20827	-0.42985	-0.90427	0.164332	-1.35894	0.379278	-0.19832	0.357944	0.081072	-0.1167	0.163944	-0.68053	-0.34325
Q6ZVM7	TOM1L2	-0.8471	0.098671	-0.62616	-0.6899	-0.58812	-0.84376	-0.49991	0.74444	0.289888	0.306918	-0.04796	-0.48613	-0.31587	-0.38065	0.26733	-0.28261	-0.32358	0.067705	0.233215
Q6ZW49	PAXIP1	0.186701	-0.14862	-0.06287	0.03193	0.422662	0.46315	0.222311	0.729374	0.274593	0.152498	-0.0976	-0.15939	-0.12414	0.05472	0.516149	0.051013	0.070108	-0.88422	0.241002
Q6ZXV5	TMTC3	-0.41462	0.50859	0.371018	-0.35154	-0.21241	-0.37307	0.526612	-0.22213	0.237279	-0.38894	-0.29834	-0.52446	-0.22641	-0.57266	-0.2989	-0.48317	0.252637	0.393742	0.889323
Q709C8	VPS13C	-0.56411	0.316113	0.077466	-0.34472	0.308754	-0.0587	0.19036	-1.03168	-0.29174	-0.32489	-0.1017	-0.11838	-0.27761	-0.05766	-0.31507	0.113753	0.203587	0.243328	0.034235
Q709F0	ACAD11	-1.06489	0.458974	0.128818	0.223829	0.079279	0.360738	0.408487	0.275199	0.728651	-0.4877	0.501483	0.060191	0.257546	0.130511	0.131238	-0.00956	0.379327	0.479734	0.246723
Q70CQ2	USP34	0.168317	-0.06266	-0.7968	-0.26329	-0.20856	-0.10413	-0.17677	0.32999	-0.07813	-0.2061	-0.00819	0.052675	0.116221	-0.12295	0.174775	0.621806	0.166227	0.106087	0.430524
Q70CQ3	USP30	-0.08506	0.281301	0.241393	-0.26209	-1.13557	-0.71947	0.413567	-0.31336	0.322054	-0.4631	0.133615	-0.30722	-0.00907	-0.22649	0.254025	0.360325	-0.18878	0.952138	0.112765
Q70E73	RAPH1	-0.10296	-0.1094	-0.00291	-1.09183	0.067757	0.115716	0.466355	-1.09601	-0.01568	-0.19703	-0.95796	0.575852	-0.37524	0.412734	-0.03123	-0.09017	0.392753	0.845908	0.575836
Q70EL1	USP54	0.367863	0.032092	0.288303	-0.91437	-0.15409	-0.50945	-0.11681	0.750889	-0.36023	-0.08756	0.517503	0.090452	-0.29669	-0.27414	0.174618	-0.07004	-0.46349	-0.39714	0.08272
Q70EL4	USP43	0.739999	-0.31816	0.042873	0.058565	0.5106	-0.30741	-0.14908	0.256516	-0.3316	-0.25172	0.867841	0.588286	-0.5047	-0.04385	-0.40763	0.006663	0.217286	0.131587	-0.19723
Q70IA6	MOB2	-0.62625	0.050393	-0.32614	-0.63308	0.985072	1.103779	-0.06395	0.259305	0.221145	0.149931	-0.14877	0.247415	-0.5497	0.239063	0.044866	0.554028	-0.13695	-0.58005	0.0183
Q70J99	UNC13D	-1.59754	0.821354	1.705723	-0.63174	0.043192	-0.31038	0.754542	0.773648	-0.18297	0.545006	-1.44557	0.768837	-0.55511	2.336336	-0.94845	-0.73409	1.413928	0.870584	-0.28536
Q70UQ0	IKBIP	-0.86477	1.534525	0.232639	-0.06106	0.604732	0.53377	0.868109	-0.0288	0.033072	-0.05429	-0.84239	-0.14266	0.962702	-0.45353	-1.57465	0.295753	0.084721	0.685681	1.519929
Q70Z53	FRA10AC1	0.636547	-0.11191	0.035712	0.391788	0.155226	0.117079	-0.2322	0.35379	-0.15636	0.115228	-0.01884	0.056858	-0.47757	-0.43038	-0.14334	-0.66582	-0.26938	0.015099	-0.1377
Q712K3	UBE2R2	0.048462	-0.14241	0.10007	-0.05903	0.246529	0.328346	-0.29962	0.298567	-0.30136	-0.25964	0.008863	-0.67548	-0.22035	-0.29424	-0.65973	-0.06431	-0.19325	-0.23036	-0.00349
Q719H9	KCTD1	-0.3312	-0.71271	-0.01387	1.790971	1.250398	0.918094	-0.76516	0.32309	-0.11165	-0.44241	-0.09322	-0.55756	0.20155	-0.87716	0.447883	0.36189	-0.11426	-0.5293	0.702142
Q71RC2	LARP4	0.260892	0.284613	1.127511	-0.13531	-0.16745	0.117938	0.134085	-0.61743	0.147581	0.800116	-0.1275	-0.32405	-0.35361	-0.0622	-0.42702	-0.17162	0.373877	0.98918	0.001574
Q71SY5	MED25	0.117474	-0.07903	0.414868	0.00881	0.005506	0.090303	-0.17087	0.092257	0.083426	-0.10759	0.416455	0.01126	-0.16181	-0.12484	0.812123	0.38267	0.352213	1.151139	-0.46228
Q75N03	CBLL1	-0.27482	0.074396	-0.23234	-0.02481	0.011161	0.324291	-0.1444	-0.3897	-0.07494	-0.10314	0.140715	-0.79038	0.065702	-0.23775	0.389242	-0.55624	-0.28892	-0.72899	-0.20452
Q75N90	FBN3	-0.03988	-0.18775	-0.90274	-2.69157	-2.12043	-2.01056	-0.00551	0.286565	0.266795	-1.05678	0.55975	-2.11215	-0.64261	-0.82803	-1.46971	-1.34667	-1.09772	-2.75583	0.862743
Q75QN2	INTS8	0.122864	-0.41723	0.003332	-0.55445	-0.13566	-0.62213	-0.25336	0.735745	0.110728	-0.04163	0.557841	-0.20586	-0.29668	-0.27462	0.365094	0.049914	-0.10992	0.048128	0.518817
Q75T13	PGAP1	0.220836	-0.21429	0.017602	-1.01761	-0.63265	-1.67261	0.098628	-0.17893	-0.06407	-0.85289	-0.22902	-0.80599	0.015318	-1.11367	1.737715	-0.18427	-0.79588	0.408598	0.57652
Q76FK4	NOL8	0.153071	-0.0201	-0.33735	0.722108	-0.21072	0.198459	-0.25893	0.181901	-0.46628	-0.24281	0.954683	-0.64852	-0.2068	-0.83305	0.240391	-0.27611	-0.59591	-0.44952	0.15771
Q76M96	CCDC80	-1.10385	0.478598	0.166598	-1.40173	-0.58806	-0.79034	-0.16426	-0.48273	1.596946	-1.09025	-2.09764	0.302977	0.560155	-0.57198	0.153888	-0.56007	-0.50339	-1.25746	1.519344
Q76N32	CEP68	0.135433	-0.30836	-0.0839	-0.3347	-0.27487	-0.70148	-0.2064	0.296417	-0.0856	0.113437	0.855759	-0.3788	-0.03696	-1.23959	1.061962	-0.10919	-0.51473	-0.49187	-0.35976
Q7KYR7	BTN2A1	0.419905	0.19033	0.2016	-0.36791	-0.1509	0.016335	0.259954	-0.33939	-0.22559	-0.82577	-2.22246	0.186994	-0.03726	0.458452	0.526332	0.127747	-0.04682	-0.47856	-0.14628
Q7KZ85	SUPT6H	0.178738	-0.07828	-0.10266	0.076755	0.27474	0.283399	-0.13768	0.576269	-0.19819	0.277396	0.285104	-0.33035	-0.04617	-0.38998	-0.00524	0.031311	-0.02055	0.448462	-0.0414
Q7KZF4	SND1	-0.25534	0.331053	0.2716	0.129753	0.460907	0.536527	0.168227	-0.05429	0.039111	0.288638	-0.1822	0.041003	0.297965	-0.29139	0.558857	0.323915	-0.26395	0.178108	0.309824
Q7KZI7	MARK2	0.163096	-0.03279	-0.12869	-0.6317	-0.34627	-0.62268	0.041935	0.109987	-0.34378	-0.0053	0.078813	0.405291	0.067167	0.462884	0.643486	0.042983	0.195592	-0.46295	-0.3278
Q7KZN9	COX15	0.327456	0.011346	-0.3612	-0.01759	-0.48421	-0.43615	-0.20162	0.011716	-0.48618	0.826562	0.633987	0.529132	-0.3443	-0.10292	0.429086	0.43328	0.269986	-0.19496	-1.26896
Q7L014	DDX46	0.136597	-0.22721	-0.01252	0.624094	-0.16817	0.192816	-0.72296	0.275863	-0.52535	0.282208	0.246449	-0.35047	-0.12724	-0.26508	0.701211	0.024167	0.103065	0.248358	0.01138
Q7L099	RUFY3	-0.50112	0.122548	0.733579	-0.63533	-0.18524	0.05527	-0.18131	-0.43095	-0.25137	-0.58834	-0.48399	-0.11216	-0.47141	0.156972	-0.54123	0.049543	-0.10745	0.090504	-0.03411
Q7L0Y3	TRMT10C	0.231634	-0.18107	-0.1672	0.625586	0.219886	0.174114	-0.18447	0.006484	-0.12253	1.240729	0.096823	-0.03769	-0.36818	-0.10705	1.45078	0.198855	0.127344	-0.11406	-0.01623
Q7L1Q6	BZW1	-0.06115	0.313579	0.334724	-0.74079	-0.24854	-0.23147	0.137354	0.141939	-0.29744	0.016795	-0.59792	-0.01214	0.202477	0.304828	-0.34152	0.454592	0.44786	-0.17619	1.204092
Q7L1V2	MON1B	-0.22042	0.399399	0.011465	-0.11015	0.45292	0.225305	0.300114	-0.11388	0.20828	0.518863	-0.5257	0.188515	-0.29219	0.924842	-0.16591	0.483418	0.01162	-0.32285	0.222289
Q7L1W4	LRRC8D	0.348577	-0.26355	0.276962	2.343618	0.833337	0.818177	-0.18998	-0.08061	0.125646	0.250729	0.051161	0.139851	0.33732	0.218756	1.1009	0.210334	1.336171	-0.11597	-0.30152
Q7L266	ASRGL1	0.947391	-0.63994	-0.15822	0.296355	2.787341	1.889031	-0.79585	0.455327	0.140174	-0.08969	2.241145	0.393044	-0.12118	-0.26439	-0.26071	0.066242	1.256763	-0.03223	0.283121
Q7L2E3	DHX30	0.069082	-0.26954	-0.16535	-0.38954	-0.2298	-0.13717	-0.26393	0.282597	-0.31366	-0.18583	0.000849	-0.2782	-0.10681	-0.30422	0.048505	-0.11863	-0.40108	-0.59476	-0.1691
Q7L2H7	EIF3M	0.05132	0.185491	-0.04045	-0.0581	0.135676	0.551082	0.137914	0.283993	-0.25502	0.184939	0.322172	-0.2203	0.227176	-0.03543	0.483721	0.180328	0.028161	-0.44687	-0.01439
Q7L2J0	MEPCE	0.254195	-0.06875	-0.07336	-0.36305	-0.46813	0.53903	0.132048	1.4023	0.198749	0.023434	0.259506	-0.64506	-0.38847	-0.45323	1.162265	0.579196	-0.58239	-0.41488	-0.12502
Q7L311	ARMCX2	0.052635	0.070544	-0.23548	-0.45681	0.134803	0.42422	0.246894	0.517359	1.302061	-0.41044	0.306061	-0.51336	-1.20853	-0.5964	0.539187	0.083563	0.267611	0.316719	0.272334
Q7L3B6	CDC37L1	-0.01764	0.145868	0.021073	-0.37082	0.174006	0.547769	0.14811	-0.16913	0.152608	-0.3384	0.062169	-0.2289	-0.09987	0.372527	0.019818	-0.83848	-0.0182	-0.27675	0.565215
Q7L3T8	PARS2	0.85566	-0.08559	-0.24838	0.652516	0.879374	-0.06019	-0.74581	-0.05611	-0.1433	-0.01646	0.395118	-0.11262	-0.00251	-0.12781	0.499893	1.063658	-0.35428	0.189858	-0.63063
Q7L4I2	RSRC2	0.543016	-1.02714	-0.37541	0.163975	-0.83385	-0.0622	-0.75069	0.481834	0.676145	-0.35074	0.293681	-1.13798	-0.06487	-0.60145	-0.00522	-0.16134	-0.29485	-0.11494	0.076264
Q7L523	RRAGA	-0.01039	0.851125	0.970687	0.004339	0.833621	0.504115	0.185497	0.274284	0.202539	0.03178	-0.52054	0.910723	0.219744	1.287943	-0.00396	0.327826	0.465713	-0.43232	0.017042
Q7L576	CYFIP1	-0.03989	0.714094	0.182046	-0.45919	0.174886	-0.00611	0.193484	-0.2411	-0.21373	0.265135	-0.05279	0.349303	-0.02005	0.220455	0.09186	0.259415	-0.10534	-0.17248	-0.10026

Q7L591	DOK3	-1.54292	1.098912	0.979653	-1.18843	-0.13046	-0.06916	-0.40394	-1.3009	0.066883	1.636383	-1.16135	1.779499	0.03068	1.479641	-0.42731	-0.08057	0.88815	0.029436	-1.21813
Q7L592	NDUFAF7	0.238242	-0.26791	-0.22257	0.235467	-0.13989	-0.17949	-0.16816	0.262609	-0.32179	0.552752	0.093459	-0.42251	-0.23728	-0.13961	-0.54343	0.421195	-1.48599	-1.07375	-0.15209
Q7L5D6	GET4	-0.67221	0.134333	0.595817	0.371379	-0.33996	0.400869	0.261275	0.624269	0.359124	0.382025	-0.07566	0.10618	-0.45997	0.18932	0.013966	0.701555	0.991965	-0.25477	-0.06914
Q7L5N1	COPS6	-0.39592	-0.12118	-0.10026	0.386547	-0.2907	0.198763	-0.01558	-0.06071	-0.15498	0.333779	0.287479	-0.80802	0.413641	-0.4564	0.240817	0.257969	-0.48744	-0.29196	0.111364
Q7L5N7	LPCAT2	-1.4412	0.589958	0.578848	-0.23838	-0.32577	-0.81814	-0.41069	-0.92362	0.013172	0.10535	0.280864	1.172243	-0.39792	1.515944	-0.48035	0.588287	0.298669	-0.75877	-1.06961
Q7L5Y1	ENOSF1	0.627871	-0.59411	0.292567	0.404519	2.017549	1.171592	0.263317	-0.22076	-0.20667	-0.06657	-0.394	0.022997	-0.4064	0.014258	0.126637	-0.29921	-0.27166	-1.00924	-1.77545
Q7L5Y9	MAEA	0.081253	0.093929	-0.00319	0.425682	0.094806	0.360502	-0.01648	-0.15535	-0.03372	-0.01195	0.122156	0.082965	-0.13878	0.292258	0.403882	0.347487	-0.31502	-0.33124	0.123981
Q7L775	EPM2AIP1	0.927658	-0.44715	-0.59448	-0.55971	-0.56341	-0.65171	0.489489	0.304837	0.557034	-0.60857	0.30873	-0.40956	-0.32904	-0.47701	-0.54483	0.128065	-0.96315	-0.9211	0.626088
Q7L7V1	DHX32	-0.87839	-0.02549	0.0665	-0.49972	0.100098	0.441713	-0.01254	-0.06697	-0.08237	-1.14154	-0.01796	-0.01784	0.237364	0.128577	-0.37533	-0.02283	0.472077	-0.59944	-0.415
Q7L7X3	TAOK1	-0.56121	0.820282	0.024589	-0.27999	-0.17619	-0.04692	0.140423	0.139467	0.01195	-0.04253	0.068533	-0.23513	0.005095	0.349572	-0.05538	0.17008	0.207785	0.349046	0.225847
Q7L804	RAB11FIP	0.4555	0.374218	0.320586	-1.35276	-0.39818	-0.89241	0.195515	-0.57973	-0.44328	-0.05381	0.335114	0.222112	0.074827	-0.1705	-0.70326	-0.17194	0.28408	-0.3269	0.213467
Q7L8L6	FASTKD5	0.116529	-0.06656	0.175265	0.505897	0.478765	0.425587	-0.12108	0.554652	0.16027	0.754723	-0.04204	-0.01894	-0.00212	0.16644	0.386396	0.064688	0.106479	-0.16155	0.572169
Q7L9B9	EEPD1	-0.23749	0.569886	0.513807	-0.06734	0.569886	-0.26612	0.89384	0.801238	-0.16665	0.207757	-0.40373	0.446167	0.159853	0.593623	0.185782	-0.10096	0.326279	0.665356	-0.42101
Q7LBC6	KDM3B	0.427618	-0.59998	-0.02659	0.197618	-0.12371	0.526578	-0.29287	0.401394	-0.72924	-0.20697	0.197393	-0.40839	-0.14602	-0.29245	0.318204	0.249417	0.039599	-0.23446	-0.05113
Q7LBR1	CHMP1B	0.578508	0.039408	-0.69768	-0.86514	0.924159	0.563094	-0.65876	-0.01308	0.031475	0.321941	0.291552	0.177032	-0.3507	0.633373	-0.36224	-0.77178	-0.06348	-0.57172	-0.97182
Q7LDG7	RASGRP2	-1.35624	0.456934	0.940966	-1.04389	-0.08226	-0.8328	0.201907	-1.74985	0.033855	0.982639	-0.94378	0.533804	0.205567	2.274752	-0.71465	-0.79977	1.132641	0.305936	-0.94915
Q7LG56	RRM2B	-1.03541	-0.09342	-0.42051	-0.32594	-0.23071	-0.15462	-0.49389	-0.45374	0.505555	-0.96961	0.101833	0.179127	0.847915	0.137224	-0.83827	-0.24792	0.301894	-0.14361	-0.9427
Q7LGA3	HS2ST1	-0.24124	1.030485	2.049853	0.061146	0.759969	0.148467	1.822596	0.198751	0.169089	0.215134	-0.17951	0.039078	-0.10333	-0.08872	-0.06053	-0.31183	0.471762	1.427391	1.556972
Q7RTP6	MICAL3	1.212792	0.25346	-0.60565	0.003042	-0.52312	-0.18962	0.007976	0.015282	0.016139	-0.74838	0.9533	-0.75749	0.55018	-0.37587	0.316011	-0.31788	-0.43327	-0.07337	-0.59511
Q7RTS9	DYM	-0.37138	0.454548	0.084722	0.085454	0.27495	0.257858	0.617253	-0.36142	-0.08367	0.314213	0.583125	-0.29608	-0.17775	-0.38231	-0.09428	0.572194	-0.5619	0.615927	0.036098
Q7RTV0	PHF5A	0.886867	-0.33678	-0.1792	0.703894	0.387566	0.190542	-0.5303	-0.12365	-0.14509	0.03151	0.460763	0.343674	-0.10893	-0.26166	0.248398	-0.21639	0.37566	0.384275	-0.16213
Q7Z2K6	ERMP1	0.086574	-0.26966	-0.51768	0.99634	-0.01254	0.619725	0.053255	0.440548	-0.63554	-1.20602	0.237811	-0.64846	-1.27404	0.129496	1.053152	-0.70811	0.338943	-0.96093	-0.64637
Q7Z2T5	TRMT1L	0.28666	0.202504	-0.3165	0.594835	-0.45669	-0.41791	-0.41508	0.050979	-0.79658	0.158257	0.721826	-0.25348	-0.78717	0.081855	-0.27332	-0.52777	-0.43897	-0.32027	-0.98139
Q7Z2W4	ZC3HAV1	0.584444	0.16225	0.353005	0.139885	-0.22753	-0.23324	0.034549	0.244676	0.268587	0.457536	-0.18693	-0.06784	-0.00264	0.068399	-0.07485	0.209682	-0.22772	-0.04965	-0.1306
Q7Z2W9	MRPL21	-0.29535	-0.45613	0.040079	0.368002	0.416751	0.125848	-0.1472	-0.21382	0.070546	0.571936	0.092276	0.012553	-0.63142	-0.11419	0.654773	-0.08272	0.350886	-0.17051	-0.46249
Q7Z2Z2	EFL1	0.074764	-0.12828	-0.42381	0.540349	0.340786	0.556553	-0.02393	0.209777	0.181982	0.000587	0.12864	0.506372	-0.3563	0.572893	-0.31621	-0.25958	-0.15697	-0.38024	-0.11163
Q7Z304	MAMDC2	-0.84513	-0.65821	-0.89149	-1.09532	-1.15348	-2.32634	-0.32212	-1.29375	0.235628	-1.32621	-0.61607	-0.94782	-0.5928	-0.73452	-0.4574	0.189665	-0.65861	-1.38083	-0.41418
Q7Z333	SETX	0.516627	0.001998	0.026178	0.086237	0.63636	0.397242	-0.15158	0.007498	-0.19716	-0.19792	0.231724	-0.3081	-0.04345	0.008255	-0.1172	-0.58055	-0.0122	-0.1236	0.190712
Q7Z388	DPY19L4	0.381779	0.186769	-0.0006	-0.6419	-0.59735	-0.4604	-0.17723	-0.03945	0.519003	-0.26258	0.069984	-0.82772	0.055047	-0.12511	0.033241	0.097654	0.532436	-0.04173	0.67496
Q7Z392	TRAPPC1	-0.65914	0.284946	0.420388	-0.02095	0.378017	0.185218	-0.10785	-0.36382	0.277148	0.188557	0.046897	0.723299	-0.1962	0.784895	-0.16847	0.205665	0.177964	0.406411	-0.25804
Q7Z3B4	NUP54	-0.05117	0.345469	-0.22661	-0.08328	0.124413	0.340848	-0.09458	0.135637	0.289298	0.124848	0.471869	0.063919	-0.18849	0.071762	0.559864	-0.06452	0.011731	0.017046	0.244508
Q7Z3C6	ATG9A	-0.04664	0.445001	-0.70784	-0.46074	-0.27337	-0.05701	-0.87737	-0.01782	-0.15064	-0.40262	-0.36324	0.474479	0.110569	-0.38599	-0.33847	0.214878	0.335077	-0.28877	0.712575
Q7Z3D4	LYSMD3	-0.36531	0.242315	-0.18815	-0.66429	0.126564	-0.58078	0.101474	0.475365	0.089912	0.197354	0.213668	-0.23457	-0.00557	-0.19794	-0.03185	0.130496	-0.21971	1.071332	-0.15631
Q7Z3D6	DGLUCY	1.043265	-0.44486	-1.76683	-0.71038	-0.60402	-0.63072	0.131006	0.687468	0.994626	-0.47515	0.23994	-0.10966	-0.25704	-0.87457	0.71969	0.290755	-0.90728	0.032124	0.525826
Q7Z3E2	CCDC186	-0.45967	0.398282	1.386206	0.161837	-0.29302	0.154296	0.044358	-0.05701	0.620477	-0.1212	-0.26269	-0.33145	0.637542	-0.05344	0.263875	0.098037	-0.15227	0.062896	0.097418
Q7Z3E5	ARMC9	-0.65952	0.448225	-0.25671	-0.75601	0.149688	0.474679	0.119519	0.019699	0.502172	0.137322	-0.31959	-0.57591	0.209107	-0.33366	-1.35635	-0.06822	-0.38621	0.127369	2.670062
Q7Z3J2	C16orf62	-0.0112	0.775869	0.245113	-0.11421	0.020369	-0.1358	0.264497	-0.20513	-0.4101	0.318056	0.02174	0.332755	0.20212	0.53804	-0.44154	0.373116	-0.03293	-0.38242	0.049027
Q7Z3K3	POGZ	0.234024	-0.06778	-0.17399	-0.19288	-0.02765	-0.13568	-0.04386	0.567586	0.571505	-0.25892	-0.10231	-0.27287	-0.36791	-0.37999	0.33476	-0.42827	0.10355	0.767979	0.125007
Q7Z3T8	ZFYVE16	-0.35697	0.473074	-0.08037	-0.22223	-0.08687	-0.23806	0.058448	-0.2044	0.101456	-0.16402	-0.10503	0.196366	0.254469	0.100725	0.288018	0.185551	0.371195	0.407618	0.520928
Q7Z3U7	MON2	0.07287	-0.25016	-0.06471	0.308168	0.548881	0.666923	0.341977	-0.31707	2.384661	-0.2821	0.072439	-0.23451	-0.03477	0.126083	0.387062	-0.4562	0.007475	0.211505	0.457348
Q7Z3V4	UBE3B	0.182939	-0.1303	-0.00836	0.734082	0.115633	-0.19395	-0.4544	-0.15564	-0.0064	0.241574	0.407841	0.004842	-0.10876	0.222754	0.272268	-0.86095	0.07857	0.332636	0.056341
Q7Z3Y8	KRT27	0.668274	-0.29617	-0.4371	-0.2569	-1.76468	-1.10126	-0.04957	0.77024	-0.04652	-0.33907	0.662566	-1.11146	-0.82839	-0.19437	-0.69271	0.02865	-0.08776	0.533754	-2.37378
Q7Z401	DENND4A	-0.3616	0.381969	0.268932	0.21332	0.271271	0.622561	0.068554	-0.41845	-0.03666	0.277393	-0.12808	0.403397	-0.05596	0.201836	-0.5874	-0.84504	-0.18306	0.032053	0.22991
Q7Z403	TMC6	0.071764	0.433282	0.890715	-0.55046	-0.05422	-0.40553	0.724801	-0.52649	0.040847	0.24813	0.643758	0.125845	-0.4432	0.224854	-0.55451	-0.86947	0.285429	0.49814	-1.31085
Q7Z404	TMC4	0.025707	-0.36166	-0.71877	0.804124	-0.82662	-0.15451	-0.31479	0.341803	-0.44436	-0.43983	1.335579	-1.43655	-0.66988	-0.38856	-0.39722	-0.10248	-0.4368	-1.20222	-1.37582
Q7Z406	MYH14	0.212552	-0.55827	-1.17666	0.353675	-0.97323	-0.05278	0.03807	0.76035	-0.22917	-1.8671	0.007869	-0.41117	0.075689	-0.68133	1.689879	-1.01126	0.609492	-0.60744	-0.54145
Q7Z417	NUFIP2	-0.14245	-0.36216	-0.11436	0.202482	-0.93956	0.014412	-0.50346	0.067329	-0.88691	0.263131	0.130303	-0.41252	0.032076	0.02686	-0.05326	0.71084	0.578813	0.665971	0.59602
Q7Z434	MAVS	-0.4549	-0.08788	-0.60244	0.235885	-0.64869	0.265582	-0.1615	0.160819	0.913352	1.283477	-0.08241	-0.8032	0.535705	-0.45644	0.865011	-0.11376	-0.35073	0.228415	0.213314
Q7Z449	CYP2U1	-0.37802	0.194261	0.180547	-0.48449	-0.03751	-0.54155	0.025011	-0.95931	0.11006	-0.25223	0.553587	-0.06975	-0.12341	-0.25428	-0.98571	-0.83551	-0.96001	0.010919	0.434913
Q7Z460	CLASP1	0.503425	-0.14283	0.175349	-0.62759	-0.87753	-0.83592	0.142523	-0.01349	-0.18945	0.018358	-0.27794	-0.50961	0.040482	-0.24651	0.520271	0.539979	-0.37273	0.160943	0.445188
Q7Z478	DHX29	-0.18469	0.327992	-0.00344	0.025064	-0.29321	-0.00739	0.942025	0.095326	-0.22788	-0.35407	0.280325	-0.20716	0.240824	-0.25543	1.427213	0.063625	-0.02672	0.376768	0.226276
Q7Z4G1	COMMD6	0.103275	0.39218	0.473693	0.050545	0.241407	-0.34825	0.020918	0.45678	0.084195	0.714668	-0.08598	0.580479	0.010001	0.501977	-0.39325	0.492809	0.012201	-0.19858	-0.57478
Q7Z4G4	TRMT11	1.032064	-0.08364	-0.14092	0.613019	-0.01573	0.18195	-0.49425	0.088931	-0.11458	0.62669	-0.20248	0.043899	0.2271	-0.2482	0.865359	0.46289	0.232362	-0.09799	0.381476
Q7Z4H7	HAUS6	0.002012	-0.16387	0.206015	-0.1382	-0.12232	-0.01546	-0.03685	0.573616	0.1513	-0.54468	-0.40362	-0.03286	0.221817	0.151139	-0.62856	-0.09818	0.183342	-0.10093	0.090309
Q7Z4H8	KDELC2	0.722356	0.270082	0.260888	-0.61266	0.133108	-0.33318	0.281816	-0.275	0.03576	-0.78049	-0.49485	-0.21652	0.157057	-0.21371	-0.67763	0.092922	-0.41877	-0.21613	0.535208
Q7Z4L5	TTC21B	-0.16905	-0.09501	-0.43522	0.006763	-0.37674	-0.90816	-0.54088	-0.16322	-0.46937	-1.32668	0.628442	-0.27628	-0.03634	0.10021	-0.10652	-0.08583	-0.75235	-0.91068	-0.17796
Q7Z4Q2	HEATR3	-0.43906	0.197612	0.31403	1.165416	0.926084	0.765247	-0.82205	0.14161	0.038606	-0.42155	-0.11149	0.511214	0.134882	0.75368	0.078741	-0.2298	0.755175	0.263644	-0.16618
Q7Z4S6	KIF21A	0.023085	0.009392	-0.35564	-0.29328	-0.07671	-0.26512	-0.08251	-0.28223	0.040847	-0.81877	0.848016	-0.31434	-0.51833	0.046061	-0.60198	-0.61092	0.356809	0.148898	-0.33014
Q7Z4V5	HDGFL2	0.247969	-0.2343	-0.31624	0.13882	0.162954	0.801518	-0.35478	0.325937	-0.00846	-0.32915	0.54799	-1.3092	-0.327	-0.68614	0.323753	-0.2276	-0.70145	-0.08024	-0.39726
Q7Z4W1	DCXR	0.230848	-1.22646	0.030582	0.620184	0.808759	0.340715	-0.3292	0.030941	-0.15206	-0.14446	-0.64721	0.662448	0.55358	0.692224	0.623561	1.129615	0.011164	0.352022	0.099366
Q7Z569	BRAP	0.043696	0.195096	-0.01432	-0.40721	0.215812	0.445142	-0.06088	0.267995	0.451541	-0.18733	0.030369	0.09707	0.374413	0.103893	-0.3052	0.129481	0.174517	-0.15215	0.193464
Q7Z589	EMSY	0.293582	-0.10696	0.332218	-0.02233	-0.0131	-0.55427	-0.07076	0.383065	-0.3472	-0.43719	-0.00687	-0.05608	-0.21873	0.117131	-0.06879	0.0815	-0.3125	0.149967	-0.36839
Q7Z5G4	GOLGA7	0.051937	0.687402	0.106058	-0.47157	-0.00876	-0.20312	0.169323	-0.36191	-0.38927	-0.15735	0.001759	0.028623	-0.13547	0.216156	0.325808	0.627937	-0.06245	0.341297	-0.2742
Q7Z5J4	RAI1	0.528227	-0.05638	0.564045	-0.50136	-0.47711	-0.12837	-0.28115	-0.0425	-0.45701	-0.30145	0.976319	-0.62955	-0.01883	0.245937	-0.1227	0.142992	-0.85318	-0.79085	-0.11359
Q7Z5K2	WAPL	0.559883	0.018066	-0.01034	-0.09309	0.181631	0.408717	-0.05584	0.249373	-0.31859	0.209478	-0.01155	-0.12302	-0.12031	0.027334	0.709583	0.37	0.44464	0.177494	-0.31618
Q7Z5L7	PODN	-1.58681	-0.10337	0.695519	-2.47866	-0.48854	-1.14537	1.022557	-1.4848	0.396568	-1.14015	-1.91609	-1.19962	-1.24006	-0.4939	0.822367	-1.1286	-0.81572	-1.60921	-0.9228
Q7Z5L9	IRF2BP2	0.544314	-0.52799	-0.84189	-0.03419	-0.87162	-0.14244	-0.13103	0.083183	-0.02437	0.078829	-0.1389	-1.28165	-0.14463	-0.49151	0.769518	-0.11545	-0.8089	-0.27375	-0.12773
Q7Z5N4	SDK1	-0.22199	0.686124	0.156898	-0.56012	-1.37665	-0.90267	0.564598	0.103966	0.329399	0.011008	-0.10665	-1.0663	0.017412	-0.10493	-0.55457	-0.0249	-0.52984	-0.31869	0.405066
Q7Z5R6	APBB1IP	-1.92952	1.108128	0.975805	-0.67622	0.165356	0.190765	0.289618	-1.37125	-0.91632	1.705576	-1.76053	1.206882	0.124898	1.977335	-0.21598	-1.20807	0.622858	-0.42836	-1.0158
Q7Z6B0	CCDC91	-0.41244	0.263844	-0.24489	-0.53761	-0.49287	-0.42463	-0.49731	0.052573	-0.73033	-0.2517	0.227549	-0.09853	-0.02972	-0.29048	0.563757	0.469594	-0.99327	-1.07673	0.089422
Q7Z6B7	SRGAP1	-0.29392	0.169211	0.224564	-0.30404	0.603167	0.480474	0.156502	-0.09064	0.250436	-0.31892	-0.91141	-0.85317	0.128412	0.147883	-0.21629	0.089574	-0.32901	0.142349	0.576942
Q7Z6E9	RBBP6	0.920922	-1.06548	-0.26132	0.182759	-0.99762	-0.52345	-0.69203	0.450432	-0.02329	-0.46913	0.271338	-0.92985	0.329996	-0.84885	0.715539	-0.25691	-0.14115	0.415827	0.091016
Q7Z6I6	ARHGAP3	-2.35125	1.976869	1.09438	-1.02924	0.583921	0.125918	0.854746	-1.86601	0.176147	1.922992	-2.12342	1.232889	0.146815	1.542357	-0.83602	-0.5795	0.492258	0.50534	-1.53966
Q7Z6J0	SH3RF1	-0.71454	0.031886	0.239175	-0.7367	-0.55813	-0.30504	0.127767	0.886817	0.481546	-0.12995	-0.47331	0.079947	0.482272	-1.04514	-1.13029	0.056307	-0.51418	-0.56085	1.367067
Q7Z6J4	FGD2	-1.20121	1.144767	1.467734	-0.72499	0.231219	0.160616	1.632417	-0.7588	-0.55203	0.698983	-0.7267	1.216405	1.095886	1.617798	-0.27687	-0.63683	0.680791	-0.17272	-0.69879
Q7Z6J9	TSEN54	1.221777	-0.10457	-0.17983	-0.2162	0.119116	0.036308	-0.26576	0.386479	-0.17229	-0.67021	0.273756	-0.18908	-0.04394	0.058892	-0.04774	-0.02486	-0.11735	-0.07774	0.060834
Q7Z6K3	PTAR1	-0.03234	0.554172	0.128186	-0.80201	0.195566	0.381948	0.25251	-0.17965	-0.1047	0.045597	0.295251	0.110623	0.260468	0.214099	0.028291	0.563879	-0.06267	0.005797	-0.10849
Q7Z6K5	ARPIN	0.731739	0.091523	-0.46382	-0.55612	0.383073	-0.23471	-1.06666	-0.36346	-0.39714	0.186176	-0.23599	-0.79603	0.1269	-0.70167	-0.927	-0.95133	-0.75333	-0.11114	-0.40751
Q7Z6L1	TECPR1	-0.16573	0.609475	0.573098	-0.1597	0.202264	0.13048	1.241973	-0.71379	0.414227	0.163518	-0.66419	0.86858	0.357056	0.539444	0.17679	0.328372	0.135708	0.002943	0.193799
Q7Z6Z7	HUWE1	0.1927	-0.09462	-0.44579	-0.20857	-0.46035	-0.31516	0.174349	0.057431	-0.16957	0.065871	0.196832	-0.31697	-0.23915	-0.34246	-0.64416	0.106697	-0.21751	-0.63848	0.1679
Q7Z739	YTHDF3	-0.0633	-0.312	0.255333	-0.27099	-0.21661	0.523382	0.092274	-0.31326	-0.75891	0.110825	0.074153	0.017912	0.527539	-0.21009	0.29143	0.40611	0.136486	0.341985	0.378256
Q7Z794	KRT77	-2.48256	0.557528	-0.41228	-0.99434	-2.14974	-1.42737	1.554709	-0.72735	0.832116	-1.33178	-0.60546	-1.45933	-0.36977	-0.39805	-0.60286	-0.72418	-1.10635	-1.42462	-0.60402
Q7Z7A1	CNTRL	0.150433	1.71923	3.801026	-1.05868	-0.06275	0.614838	0.750612	0.278144	-0.22413	-0.2733	-0.64238	0.56877	0.838326	0.309454	-0.62814	-1.97048	-0.20583	0.120751	-2.2881
Q7Z7A3	CTU1	-0.02125	0.001132	-0.0583	-0.00568	-0.01994	0.452656	0.059649	-0.08364	-0.4535	-1.08341	0.680693	-0.4796	-0.06896	0.104991	0.234384	-0.76908	-0.31856	-0.40583	-0.19079
Q7Z7A4	PXK	0.018689	0.266398	-0.27592	-0.77479	-0.06748	0.005485	0.055647	-0.1116	0.214625	0.042811	-0.04347	-0.33246	0.24376	0.728652	0.636096	0.047568	0.079795	2.18134	0.294276
Q7Z7B0	FILIP1	0.578795	0.533693	-0.12889	-0.63907	-0.27739	-0.83429	0.068749	-1.05501	0.28265	-0.21013	-0.2231	-0.59895	0.032673	-0.94058	1.228112	-0.78144	-0.95698	-0.31492	0.171425
Q7Z7D3	VTCN1	1.149116	-0.99146	0.271635	3.58379	3.528613	3.502639	-0.90377	-3.64226	-0.36737	-1.73574	-2.39094	-1.17079	0.254063	-1.76707	0.368178	4.203154	0.093569	-0.57158	-3.95066
Q7Z7E8	UBE2Q1	0.130585	0.003764	0.195601	-0.10545	-0.21669	0.133383	-0.5413	0.187099	-0.00764	0.060785	-0.22149	-0.04712	0.270326	0.175402	0.812736	0.047736	0.160633	-0.05669	0.129969
Q7Z7F0	KHDC4	0.306117	-0.14157	-0.29756	-0.59571	-0.13437	-0.45714	0.070143	0.399903	-0.14158	-0.27193	0.298173	-0.4398	-0.39899	-0.95954	-0.54299	-0.40673	-0.28138	-0.42051	-0.37392
Q7Z7F7	MRPL55	-0.24125	-0.19016	0.395305	0.750298	0.102135	0.472202	-0.19227	-0.49751	-0.0516	0.342991	0.584118	-0.22769	0.824343	-0.51626	0.885678	-0.13102	-0.43711	0.155926	-0.75596
Q7Z7G0	ABI3BP	-2.27055	-1.00254	0.833263	-1.88544	-0.90004	-1.49525	0.048394	-1.71923	0.326313	-0.57126	-2.04896	-1.27421	-0.90866	-0.71965	2.434441	-1.46848	-0.77932	-0.71382	-1.27649
Q7Z7G8	VPS13B	-0.26034	-0.0497	0.526286	0.355545	0.182762	0.313256	-0.65147	-0.20027	0.124254	0.416586	-0.44036	-0.33386	0.009451	-0.65975	1.008529	0.171263	0.223878	0.374306	-0.12971
Q7Z7H5	TMED4	-0.31447	0.122643	0.185817	-1.03223	-0.48528	-0.05504	0.564063	0.46616	-0.38273	-0.85911	0.100167	-0.11087	-0.2105	0.143811	0.760667	-0.22087	0.580164	0.128904	-0.45821
Q7Z7K6	CENPV	-1.03397	-1.07924	-0.30054	-2.10458	-1.01988	-0.54571	0.335034	-0.69213	-0.47878	-1.08888	-0.79268	-1.00594	-0.08996	-1.86613	-2.97392	-1.45347	-1.31141	1.166428	0.613269
Q7Z7L1	SLFN11	0.480248	0.767714	0.143133	-0.42291	0.734086	0.350351	-0.35607	-0.46437	-0.29963	0.37145	-0.15175	0.420274	1.024865	0.06751	-0.84412	-0.26495	-0.12458	-0.81674	-0.03738
Q7Z7M9	GALNT5	-0.77742	0.629115	0.275904	-1.28747	0.140333	0.310814	0.157297	-0.44456	0.727281	-0.33605	-1.36256	-0.39061	0.921742	-1.58384	-2.76555	0.16736	-0.91876	0.424045	1.759409
Q7Z7N9	TMEM179	0.111516	0.169159	-0.06681	-0.19604	-0.57549	-0.52606	0.037767	-0.22254	-0.13864	0.056698	-0.56356	0.389705	-0.05982	0.079592	0.475543	-0.06013	0.168485	-0.19168	-0.71163
Q86SF2	GALNT7	-0.12265	0.572067	0.819015	0.670148	0.268936	0.394244	0.362803	0.216088	-0.12535	0.552484	0.964421	-0.40986	-0.2127	0.133025	-0.9432	-0.8464	-0.14674	-0.04428	0.571917
Q86SK9	SCD5	1.607579	-0.40459	-0.86282	-1.26938	-1.27618	-0.66954	1.000808	1.674653	0.339101	1.158993	0.157371	-0.88303	-0.6659	-0.69856	-0.38086	-1.43408	-0.98958	-2.29819	-0.7222
Q86SQ0	PHLDB2	-0.61034	0.636617	0.313866	-1.21693	-0.06759	-0.73523	0.15176	1.419182	0.255208	2.146678	-1.551	-0.59844	0.582018	-0.60779	-0.61772	-0.63814	-0.8502	0.788432	1.302473
Q86SQ7	SDCCAG8	-0.52668	0.14446	-0.10796	-0.54846	-0.2979	-0.27335	-0.21574	-0.83708	-0.27546	-0.05086	0.118926	-0.15355	-0.12757	0.084725	-0.49422	-0.10876	0.299305	0.091049	0.088588
Q86SR1	GALNT10	-0.73455	0.301368	0.415756	-0.12006	0.126307	0.126191	0.243334	-0.44954	0.179127	0.072701	-0.321	0.716745	0.044288	-0.0036	0.739736	0.646085	-0.02743	-0.29006	-0.25408
Q86SX6	GLRX5	-0.63577	-0.4562	-0.71555	0.617435	0.634959	0.476141	0.158606	0.453361	0.2564	-0.01112	0.269821	-0.0723	0.289885	0.223235	0.049127	1.195684	0.638344	0.435814	-0.11323
Q86SZ2	TRAPPC6	-0.37208	-0.14543	0.3601	-0.15474	0.448147	-0.10477	0.189137	-0.7132	0.306077	0.127173	-0.36311	0.38319	0.153156	0.582442	0.287476	0.456881	0.159364	-0.54982	0.157614
Q86T24	ZBTB33	0.894979	-0.54165	-0.05468	-0.06274	-0.52347	0.430576	-0.65497	0.856769	-0.19825	0.01334	-0.11688	-0.70667	-0.0099	-0.58081	-0.45078	-0.06278	-0.68697	-0.06415	0.197978
Q86T65	DAAM2	-0.36543	0.024853	0.049029	0.427706	-0.72693	-0.98812	-0.1752	-0.71509	0.451141	-0.37425	-1.29946	-0.899	-0.15665	-0.79373	-0.77522	0.360022	-0.87992	-0.21327	0.071497
Q86TB9	PATL1	0.432473	-0.06414	0.13728	-0.33706	-0.17631	-0.49957	-0.40152	-0.00107	-0.44081	-0.01696	-0.61478	0.254154	0.675371	0.238531	-0.53104	0.527414	0.601851	0.274323	-0.37831
Q86TG7	PEG10	2.104291	-0.69531	-0.73793	-0.79668	-0.71651	0.259084	-0.44422	-0.17356	-0.23118	-1.48614	-0.91815	-1.81483	0.016758	-0.93154	2.107871	0.850007	0.1654	-0.66683	-1.20767
Q86TI0	TBC1D1	-0.3412	0.85655	0.550183	-0.49346	-0.10551	-0.07706	0.347512	-0.49556	-0.16246	0.461817	-0.19894	0.158229	0.386918	0.690141	-0.96406	-0.3591	-0.25174	-0.3927	-0.10663
Q86TI2	DPP9	-0.40736	-0.02473	0.198332	0.00774	0.114824	-0.03731	-0.48627	-0.53706	0.421542	0.439159	0.174717	-0.24414	0.18751	-0.23892	0.081367	-0.03596	-0.41727	-0.27214	-0.01806
Q86TM6	SYVN1	-0.49757	0.170992	-0.05693	0.287461	-0.2236	0.493639	0.816339	0.464139	0.857745	-0.0387	-0.00272	-0.14635	-0.01633	-0.15787	0.327644	0.107898	0.006269	-0.05781	0.099475
Q86TP1	PRUNE1	-0.39539	-0.69737	-1.31042	-0.30791	-0.61251	-0.30674	-0.54557	1.0982	0.809444	0.046881	-0.72064	-0.33109	-0.40786	-0.22757	1.25165	-0.60587	0.020597	-0.60543	-0.54847
Q86TU7	SETD3	-0.29775	0.088278	-0.07577	0.131345	0.233075	0.263769	-0.95909	0.121005	-0.95124	0.178587	0.57226	-0.1417	0.065326	0.167628	0.353751	0.030339	0.506295	0.106002	0.010814
Q86TV6	TTC7B	-0.52444	0.021504	0.172767	-0.30585	0.215763	-0.23854	0.17814	-0.36513	-0.1056	-0.41725	-0.39213	0.396294	-0.11753	-0.05028	-0.36353	-0.69094	0.172156	0.443364	0.055924
Q86TW2	ADCK1	-0.54848	0.143594	-0.15431	-0.51965	-0.11806	-0.39248	0.044329	-0.08263	0.309231	0.510954	-0.14701	0.261788	-0.45129	0.132065	-0.22011	0.674979	-0.2564	-0.36355	-0.72174
Q86TX2	ACOT1	0.54995	0.132133	-0.71086	0.770697	-1.14314	-1.60035	-0.14886	-0.57752	-0.24197	-0.46229	0.232362	-0.76844	-0.36564	-0.32176	-0.98813	-0.05154	-0.67278	0.303258	-0.09271
Q86U06	RBM23	1.145528	0.012302	0.101323	-0.76619	0.129056	-0.63261	-0.15171	0.97354	-0.18377	0.080449	-0.05821	-0.04709	-0.29324	0.008316	0.066045	-0.10247	-0.0104	-0.19875	0.358878
Q86U28	ISCA2	-0.25732	-0.3684	-0.53514	-0.19811	0.091396	-0.96772	-0.84461	-0.11057	0.040847	0.20601	0.595312	0.54666	0.277227	0.339196	0.707311	-0.11739	0.313996	0.602222	-0.35965
Q86U38	NOP9	0.314452	-0.09898	-0.88483	0.518475	-0.04415	-0.01948	0.551676	0.034102	-0.16286	-0.05878	0.12595	-0.17078	-0.08839	0.045434	-0.04929	0.508464	-0.14508	-0.25585	-0.03476
Q86U42	PABPN1	0.964346	-0.06369	-0.52641	0.252115	0.020689	0.34593	-0.28583	0.592801	0.038625	-0.26046	0.439134	-0.79981	-0.16298	-0.50337	0.164587	-0.31252	-0.25884	0.016619	-0.05893
Q86U44	METTL3	0.167651	-0.87781	-0.11922	-0.10565	-0.25424	-0.20611	-0.48743	0.260103	0.723643	-0.19295	0.368621	-0.51945	-0.26458	-0.33196	0.251712	0.13406	-0.48951	-0.0731	0.260519
Q86U70	LDB1	0.076737	-0.04619	-0.39783	0.729925	-0.23002	0.188631	-0.17903	0.976714	-0.56991	-0.56034	0.184759	-0.88988	-0.22588	-0.46243	0.059447	-0.9919	-0.25892	-0.26669	0.087417
Q86U86	PBRM1	0.886191	-0.38937	-0.19269	-0.17662	-0.14383	0.043353	0.205087	0.70447	0.054021	-0.27127	0.144229	-0.35123	-0.47488	-0.23964	0.211134	-0.88103	-0.13133	0.041559	0.04932

Q86UA1	PRPF39	0.28352	-0.29279	0.235159	0.052849	0.237822	0.277846	0.025814	0.417605	-0.97298	-0.23325	0.514176	-0.14495	-0.13121	-0.00626	0.140819	0.117935	0.327626	0.058834	-0.30757
Q86UD1	OAF	-1.83852	0.570494	-0.12328	-1.31691	-0.95672	-1.54321	0.159993	-0.10172	0.209024	0.104259	-1.11146	0.78985	0.137275	1.312945	-0.68364	-0.49531	1.27465	-0.81041	-1.15664
Q86UE4	MTDH	-0.10366	-0.03509	-0.18074	-0.2553	-0.77482	-0.21305	0.325377	-0.34741	-0.00042	0.015864	0.086121	-0.69063	0.844965	-0.48409	0.71318	-0.08424	-0.52035	0.496509	-0.37265
Q86UE8	TLK2	0.563658	0.13747	-0.01227	0.168973	0.002864	-0.65089	0.058187	0.280854	-0.15335	-0.33782	-0.12362	-0.14701	-0.11568	-0.07011	-0.46261	0.493512	-0.3243	-0.3529	-0.06554
Q86UK7	ZNF598	0.802651	-0.21595	0.329367	-1.23062	-0.80207	-0.22007	-0.36547	-0.24575	-0.09973	0.224051	0.339525	-0.50992	-0.12457	-0.34276	0.506594	0.431908	0.265387	1.192709	-0.00829
Q86UL3	GPAT4	-0.09796	0.598505	0.966591	-0.39498	0.09635	0.068406	0.772611	-0.20051	0.040847	0.248305	-0.22944	0.666079	0.97559	0.451346	0.754385	-0.55939	1.053074	0.153074	0.389765
Q86UP2	KTN1	0.061578	0.165272	-0.18673	0.642344	-0.16794	-0.16183	-0.09013	-0.03729	-0.29404	-0.15123	0.171472	-1.15914	-0.1588	-0.6809	0.48445	0.259675	-0.66072	0.224743	0.245508
Q86UQ4	ABCA13	-0.13465	0.734197	0.148878	-0.52451	0.423238	-0.04187	0.312078	-0.10653	-0.09683	0.352049	-0.04727	0.452561	0.213559	0.335924	0.041157	0.172465	0.049094	-0.09057	-0.0116
Q86US8	SMG6	0.434887	0.506541	-0.05121	-0.54777	0.315682	-0.01587	0.244325	0.189457	0.244443	-0.57699	-0.02863	-0.15119	0.247979	-0.07761	-0.76859	-0.45523	-0.1806	-0.08734	0.552362
Q86UT6	NLRX1	-0.27298	0.227694	-0.39134	0.021695	-0.72978	-0.60366	0.66923	-0.76649	-0.28309	-0.69964	1.306109	0.196752	-0.03098	0.333095	-0.70268	0.175053	-0.78783	-0.6399	-0.59157
Q86UU0	BCL9L	0.417226	-0.03024	0.105448	0.228535	-0.58106	-0.25458	-0.28044	0.135374	-0.22842	-0.3478	0.067991	-1.39036	-0.12417	-0.28248	0.536956	0.524751	-0.27502	0.491024	0.19203
Q86UU1	PHLDB1	-0.08155	-0.09259	-0.11956	-0.66431	-0.32214	-0.90172	-0.27799	-0.20968	0.687566	-0.03842	-0.63702	-0.67693	0.570712	-0.64303	-0.16204	-0.34178	-0.69609	1.558946	1.465576
Q86UV5	USP48	0.731422	-0.28818	0.368542	-0.17988	0.234481	0.760596	-0.44565	1.242409	-0.18713	0.674855	0.527446	-0.5373	-0.3103	-0.14034	0.085781	-0.1608	-0.05949	-0.08439	0.121194
Q86UW6	N4BP2	-0.06183	0.515924	0.455821	-0.05163	-0.06088	0.034499	0.568732	-0.36613	-0.34458	0.045377	-0.04195	0.415269	0.08572	0.490709	-0.13961	-0.08025	0.128846	0.19588	0.476675
Q86UX2	ITIH5	-2.02328	-0.10132	-0.46731	-1.88947	-0.70237	-1.34686	0.370165	0.379998	0.372441	-0.18156	-0.46431	-1.09644	-0.3663	-0.74896	-0.87449	-0.93964	-0.10872	0.855216	-0.04195
Q86UX7	FERMT3	-1.82777	1.16861	1.299893	-0.58495	0.238675	-0.21896	0.295993	-1.59141	0.292786	1.195486	-1.46316	1.285473	-0.27907	1.899324	-0.69054	-0.62366	0.026763	0.571838	-1.17847
Q86UY6	NAA40	0.270743	-0.05358	-0.15944	0.628741	-0.43792	0.130037	-0.05149	-0.02222	-0.46756	-0.36938	0.646593	-0.48512	-0.63059	0.233065	0.223286	-0.13252	0.474511	-0.32636	-0.82275
Q86UY8	NT5DC3	0.307366	0.338334	0.046032	0.33471	-0.68491	-0.24636	0.459031	-0.59929	0.186545	-0.30059	0.125278	0.026585	0.322691	0.05266	0.018617	0.893429	0.410279	0.227017	0.418624
Q86V21	AACS	0.348816	-0.11449	-0.45631	0.389327	0.761559	1.03033	0.476704	1.336973	-0.02709	0.31771	-0.11941	0.183805	0.162454	0.496191	-0.67437	-1.28573	0.574107	-0.3988	-0.03772
Q86V48	LUZP1	-0.46438	-0.05299	0.164541	-0.78582	-0.74997	-0.52415	-0.18449	0.145854	0.710415	0.314974	-0.684	-1.05753	0.25457	-0.80261	-0.27357	-0.03629	-0.30522	0.5928	1.045808
Q86V59	PNMA8A	0.439047	-0.14251	-0.15137	-0.62139	-0.14876	0.183949	0.067085	0.682609	-0.40417	-1.37553	0.194188	-0.43467	0.164	-0.01339	1.060419	-0.47227	0.233124	-0.53733	-0.02046
Q86V81	ALYREF	0.805579	-0.38809	-0.5883	0.274772	0.052705	0.347962	-1.03768	0.255988	-0.09899	-0.20113	0.150834	-0.38491	-0.40638	-0.38269	-0.50028	-0.1566	0.050518	0.132909	-0.8722
Q86V88	MDP1	0.297105	-0.00762	-0.08161	-0.0639	0.293711	0.131943	-0.23777	0.086849	0.224549	0.173015	-0.24412	0.263553	0.048957	0.467369	-0.24662	-0.05361	0.621171	-0.15733	-0.2294
Q86VB7	CD163	-1.76933	3.02564	2.931441	-1.49726	0.941475	-0.01303	1.720637	-1.41582	0.183136	1.602853	-1.47347	2.191843	0.824164	2.410785	-0.78009	-0.22656	0.462484	0.048588	-1.32803
Q86VI3	IQGAP3	0.582316	-0.03608	0.234401	0.348678	1.063882	0.828901	-0.11836	0.906275	0.322873	0.185018	-0.63745	-0.26303	0.343692	0.266458	-0.88115	-0.19696	0.498894	-0.13858	0.859023
Q86VM9	ZC3H18	0.224827	-0.2	0.076364	0.086202	0.207163	0.486633	-0.94547	0.308872	-1.555	-0.15535	0.286774	-0.93798	-0.20225	-0.63513	0.354567	0.522648	-0.45143	0.219046	0.271801
Q86VN1	VPS36	-0.48466	0.050019	-0.43636	-0.39936	-0.32788	-0.27117	0.047528	-0.28051	-0.05623	0.277331	0.543298	-0.16837	-0.31263	0.188088	-0.31367	0.380936	0.20798	0.258381	0.470871
Q86VP1	TAX1BP1	0.360571	-0.43913	0.964925	-0.6869	-0.40222	-0.26069	0.530694	1.034773	0.776712	0.575049	-0.29175	-0.46258	0.002393	-0.36008	0.063592	0.071881	0.061511	-0.50169	-0.64306
Q86VP3	PACS2	-0.10235	0.329702	0.143054	-0.45701	-0.36394	-0.51436	-0.38208	-0.29996	0.153429	0.375815	-0.29287	-0.07744	0.11099	-0.34348	0.483927	-0.71749	-0.31554	0.083549	0.070905
Q86VP6	CAND1	0.634241	-0.00779	0.005552	-0.02478	0.018167	0.334166	0.044555	0.107279	-0.04776	-0.40003	-0.13062	-0.79249	-0.13456	-0.76728	0.028267	-0.27796	-0.80322	-0.33918	0.683069
Q86VR2	RETREG3	-0.69429	0.943074	0.318506	-0.04344	-0.24129	-0.32706	1.22505	-0.20804	0.462899	-0.34284	-0.24842	0.218241	0.148924	0.11426	-0.36669	-0.22882	-0.03608	0.075232	0.361835
Q86VS8	HOOK3	-1.22099	0.39526	0.06217	-0.94901	-0.1849	-0.17836	-0.13502	-0.86583	0.787018	-0.34507	-0.4908	-0.00853	0.852945	0.061674	-0.08744	-0.02833	0.142707	0.469014	0.782976
Q86VW0	SESTD1	0.371865	-0.02063	0.215596	-1.0637	-0.29971	-0.60208	0.094606	-1.2343	0.140002	-0.42009	-0.26868	-0.56393	-0.06715	-0.48777	0.203981	-0.48596	-0.09985	-0.32981	0.406808
Q86VX2	COMMD7	0.203634	0.511441	1.098255	-0.09477	0.463647	-0.68131	-0.15249	0.188379	0.192445	0.393302	0.067185	0.389222	-0.29715	0.49509	-0.50309	0.070347	-0.03125	-0.117	-0.12616
Q86VY4	TSPYL5	-0.08342	-1.09499	0.053126	-1.23712	-0.70288	0.257319	-0.68323	0.670142	0.703269	1.375195	-1.84967	-0.40445	1.115385	-0.61881	0.121192	0.307884	-0.53171	-0.41368	1.67683
Q86W34	AMZ2	0.374335	-0.05337	0.234395	0.569692	0.011025	-0.54087	-0.39048	0.173022	-0.14754	-0.22867	-0.09986	-0.58195	-0.11236	-0.10574	-0.8324	-0.05006	0.40468	-0.29113	-0.16769
Q86W42	THOC6	0.301673	0.111684	0.376788	0.544098	0.607696	0.545539	0.102095	0.488063	-0.23217	0.232173	0.692783	-0.16038	-0.3089	-0.09101	-0.29328	-0.30922	0.481856	0.283695	-0.19326
Q86W50	METTL16	1.454061	0.077955	-0.38595	-0.34969	-0.28312	-0.25172	-0.09695	-0.10289	0.182131	-0.00144	0.653817	-0.13249	-0.20246	-0.38477	-0.42624	-0.60919	-0.14584	0.091901	-0.2733
Q86W56	PARG	0.357786	0.424736	-0.10391	-0.5789	-0.07808	0.220865	0.28424	-0.0943	-0.88948	0.709468	-0.35234	-0.18177	-0.5898	0.154876	-0.27715	0.557973	0.317497	-0.26134	-0.05241
Q86W92	PPFIBP1	-0.60941	-0.17936	-0.26675	0.156563	-0.19035	-0.31277	0.035921	-0.28751	0.610247	-0.28818	-0.41537	-0.94974	0.786202	-0.96264	0.079975	0.658082	0.44654	1.311742	1.06443
Q86WA6	BPHL	0.873485	-1.39392	-1.38662	0.645345	-0.02663	-0.13826	-0.21293	0.532655	-1.04772	-0.7293	-0.01585	-0.38764	-0.31146	-0.27696	1.254761	2.564455	-1.07026	-0.37654	0.053599
Q86WA8	LONP2	-0.36645	0.047788	0.114624	-0.58284	0.123177	0.013418	-0.25617	-0.06878	-0.10598	-0.04247	-0.40021	-0.45276	-0.16843	0.164585	-0.06152	-0.16638	0.156825	0.037979	-0.59197
Q86WB0	ZC3HC1	0.130862	0.024389	0.124282	0.418796	0.340367	0.42462	0.244899	0.038835	0.32064	0.226074	0.186309	-0.39219	0.276755	-0.12063	0.503144	-0.05172	-0.17215	-0.00463	0.341687
Q86WG5	SBF2	-0.39497	0.162255	0.139634	-0.66722	0.132508	-0.31135	0.084489	-0.11135	-0.06324	0.054213	0.183919	0.178294	-0.03246	0.10159	0.4311	-0.41166	-0.09139	0.03976	0.016391
Q86WJ1	CHD1L	0.592913	0.144095	-0.27551	0.372031	0.016916	0.042889	-0.25276	0.277956	-0.00769	-0.74199	0.715405	-0.21158	0.178088	-0.14831	0.21357	0.262159	-0.02062	0.131716	-0.24752
Q86WN1	FCHSD1	-1.07016	0.405061	0.675157	0.201909	-0.46883	-0.03251	-0.11423	-0.09666	0.019645	0.109063	-0.20708	-0.32386	-0.15698	-0.12063	-0.14928	0.006911	0.109423	0.251036	0.496691
Q86WQ0	NR2C2AP	1.26223	-0.98628	-0.0416	-0.01269	1.17615	0.155019	-0.15896	-0.34701	1.156354	0.787028	0.119094	0.602579	0.259333	0.221447	-0.86735	0.216577	0.900762	-0.20511	-0.07392
Q86WR0	CCDC25	1.377563	-0.78171	-0.23279	0.083569	0.311593	0.450987	-0.64487	0.431752	-0.23499	0.050842	0.614795	-0.8798	-0.25302	-0.92448	-0.53785	0.792928	-0.26771	-0.10196	-0.01154
Q86WR7	PROSER2	0.427814	-0.11205	-0.02956	-0.23078	-1.10149	-0.24676	0.127868	0.722078	0.006883	0.420459	-0.3031	-0.44158	-0.05703	-0.32966	0.019051	0.206901	2.4479	2.306505	1.019028
Q86WT1	TTC30A	0.175418	-0.07616	-0.45268	0.303883	-0.40269	-0.17731	-0.09147	-0.17309	0.040847	-0.62524	1.829152	-0.32967	-0.31303	-0.64913	-0.32524	-0.42362	-0.33107	-0.64884	-0.3338
Q86WU2	LDHD	-1.40799	0.293337	0.282007	-0.36655	0.220044	0.120631	0.338043	-0.1532	0.545718	0.493695	-0.29395	-0.07147	0.390202	0.30057	1.235012	0.178353	-0.29949	-0.27361	-1.50265
Q86WV6	TMEM173	-0.94952	0.412262	1.141228	-0.35715	-1.14415	-0.6721	0.220136	-1.01776	-1.54619	-0.39873	1.460997	-0.80878	-1.82148	0.102525	0.633163	-0.62119	-0.43142	-1.51537	-1.31519
Q86WX3	RPS19BP	-0.10864	0.114566	0.204464	-0.27299	-0.3845	-0.01561	0.321616	0.168443	0.538589	-0.18955	0.908333	0.061262	0.457426	0.295851	0.134755	0.477781	0.414888	1.744032	0.517008
Q86X02	CDR2L	0.132822	-0.07442	0.22737	0.725705	-1.2498	-0.65419	0.118088	0.251547	0.199392	0.355973	-0.55479	-1.02352	0.133579	-0.91706	-1.05733	0.214693	0.503724	0.136985	-0.6326
Q86X10	RALGAPB	0.214181	0.099143	0.120046	0.239334	-0.26451	-0.65673	0.748715	-0.14473	-0.47866	0.148208	-0.01956	-0.07686	-0.22561	0.306373	0.533003	-0.32444	-0.06113	-0.36372	-0.17805
Q86X27	RALGPS2	-0.20115	0.357469	0.233588	0.036199	0.552427	0.284505	0.376712	-0.11181	0.315362	0.00974	-1.02309	-0.52717	0.481053	-0.72489	0.548636	0.306141	0.125496	0.601642	0.925304
Q86X29	LSR	0.367427	-0.71936	-0.36392	0.209777	-1.14052	-0.20796	0.456271	1.309763	-0.75347	-0.26621	0.714943	-0.493	-0.58323	-0.76024	1.167154	0.301818	-0.14651	-0.24347	-0.40539
Q86X52	CHSY1	-0.53811	0.852649	0.252127	-0.5894	0.187132	-0.09338	0.567518	-0.33507	0.310806	0.112033	-0.84505	-0.14665	0.530916	-0.30208	-0.76784	0.197236	0.302675	0.418243	1.760034
Q86X55	CARM1	-0.73945	0.176783	-0.0996	-0.48194	-0.02302	-0.16706	-0.48591	0.517295	-0.19437	-0.06554	-0.10903	-0.00152	-0.33958	-0.08662	-0.67435	0.485371	-0.03372	-0.18133	0.506319
Q86X76	NIT1	-0.02909	-1.51312	-0.41441	0.443677	0.911791	-0.22825	-1.08319	-0.47246	-0.22103	0.610352	-0.08774	0.141132	0.052847	0.393909	1.050091	1.077905	-0.09523	-0.38407	0.109139
Q86X83	COMMD2	0.154744	0.643051	0.484423	0.138437	0.362265	0.594398	0.506102	0.192843	-0.58936	0.615142	-0.0856	0.285435	-0.48626	0.394907	-0.60235	0.011545	-0.0093	-0.3431	0.345834
Q86XA9	HEATR5A	-0.15473	0.265997	0.362679	-0.09348	0.013125	0.159487	0.204633	0.017879	0.023298	0.122409	-0.70075	-0.08965	0.252146	0.451082	0.13584	-0.14588	-0.47739	-0.16472	1.000582
Q86XI2	NCAPG2	1.077587	-0.72288	0.188216	0.612249	1.350242	1.142614	0.43533	0.63142	-0.13455	0.019374	-0.074	0.026804	0.687201	0.764848	-0.02574	-0.14528	0.452175	-0.28327	0.666808
Q86XL3	ANKLE2	0.428121	0.221847	-0.01974	0.009014	-0.43633	-0.3574	-0.2427	0.3475	-0.02695	-0.0284	0.084331	-0.41559	0.509371	-0.16611	-0.46705	-0.1063	-0.00105	-0.38627	1.025839
Q86XN8	MEX3D	-0.36707	-0.00822	0.102148	0.085408	-0.46829	-0.9536	-0.5302	0.066359	0.368887	-0.04333	0.335684	-0.14799	-0.00092	0.337184	0.285193	-0.84826	-0.09924	0.458244	-0.51962
Q86XP3	DDX42	0.583366	0.062376	0.167316	0.893722	-0.27772	-0.03442	-0.55893	0.449084	-0.1254	-0.18376	0.605013	-0.46835	0.00712	-0.51605	0.019068	0.061516	-0.35171	0.085775	-0.13102
Q86XR8	CEP57	0.864346	-0.45199	-0.08453	-1.06105	0.948641	0.523294	-0.67526	-0.47178	-0.16185	-0.20164	0.671346	-1.29783	-0.23951	-0.82136	-0.59222	0.913627	-0.37294	-0.08215	0.631452
Q86XX4	FRAS1	0.081715	1.522595	-0.07464	-1.13108	-0.62296	-1.04498	1.094575	0.761887	1.767688	-0.94682	-1.31536	-1.00526	-0.33513	-0.05513	-0.80375	-1.6168	-0.75611	-0.79301	0.371362
Q86XZ4	SPATS2	0.467493	-0.12102	-0.27292	-0.3162	-0.2726	-0.04795	-0.20469	0.770391	0.081649	0.513543	-0.46893	-1.13454	0.074336	-1.62081	-1.19098	-0.70305	0.047703	0.209246	1.0306
Q86Y07	VRK2	0.008317	0.703166	0.225106	-0.20473	0.79661	0.83027	0.057654	0.33896	0.349422	0.088855	0.583978	-0.14978	0.109361	0.21112	-0.27567	0.225364	0.358126	0.513176	0.072547
Q86Y37	CACUL1	-0.38222	0.024128	-0.21484	0.06712	0.044172	0.422395	-0.07576	0.069806	0.071639	-0.08003	-0.69176	0.329028	-0.07428	0.174012	0.185841	0.75849	0.181507	-0.38349	-0.34502
Q86Y56	DNAAF5	-0.36139	0.029274	-0.50441	-0.2171	-0.74946	-0.46847	-0.06536	-0.04675	-0.0137	-0.06359	1.051279	-0.53133	-0.24838	-0.86298	-0.6654	0.149901	0.145195	-0.88533	-0.2497
Q86Y79	PTRH1	0.057249	-0.04025	-0.09085	0.960234	-0.11866	0.227006	-0.54225	0.46312	-0.31416	-0.29639	1.255958	-0.917	-0.19217	-0.3146	0.458943	-0.19784	-0.21711	0.514504	-0.10124
Q86Y82	STX12	-0.45136	1.58244	0.458821	-0.36934	0.144539	-0.10094	0.570277	-0.37949	0.328698	0.545938	-0.39266	0.623982	0.588379	0.626205	0.16095	0.02731	0.317684	0.822282	0.423035
Q86YJ6	THNSL2	-0.34767	0.770666	-0.33449	-0.45176	-0.39503	-0.36746	0.013044	-0.88459	0.040847	-1.71601	-0.51802	-0.59095	0.314067	-0.26594	-0.67001	0.10348	-0.36806	-1.00906	0.117687
Q86YM7	HOMER1	0.068097	-0.03064	-0.09399	-0.25345	-1.54313	-0.79614	0.059102	-0.17819	0.401529	0.002976	0.52737	-0.07224	0.003279	-0.17923	-1.6374	-0.04856	1.024397	1.50527	-0.539
Q86YP4	GATAD2A	0.501947	-0.6133	-0.64161	-0.00238	-0.88394	-0.10773	-0.10293	0.111832	-0.31599	-0.24138	0.002712	-1.04045	-0.56119	-0.54136	-0.1069	-0.19647	-0.4045	0.199415	-0.33992
Q86YR5	GPSM1	0.603397	0.418921	0.374753	-0.87502	-0.4187	-0.4419	0.307722	0.160056	0.120217	0.200417	-0.41636	-0.22772	0.243317	-0.46334	-0.74341	-0.01477	-0.34696	0.1583	0.995173
Q86YS6	RAB43	-0.80864	0.156095	0.56454	0.026279	0.177425	0.280336	0.458841	-0.15689	0.154386	0.757561	-0.13422	0.672386	0.449938	1.113986	-0.43851	0.136862	0.193946	-0.19336	-0.42942
Q86YS7	C2CD5	0.144523	0.134347	1.38155	-0.48086	-0.40548	-0.52799	0.067872	0.358226	-0.49987	-0.07898	0.338278	-0.19086	-0.42501	-0.07949	0.057067	0.282916	-0.54658	-0.2365	-0.31405
Q86YT6	MIB1	-0.35768	0.314306	0.081315	-0.06187	0.090593	-0.14333	0.057301	-0.38484	0.26359	-0.54604	-0.02321	-0.30659	0.097351	-0.17997	-0.30054	-0.09654	-0.16099	0.083867	0.203237
Q86YV0	RASAL3	-1.41778	1.375235	1.270051	-1.06539	0.380877	0.324813	0.615251	-1.13884	-0.55005	0.710014	-1.16928	0.064379	-0.09476	0.927747	-0.35639	-0.31844	-0.13307	0.530522	-1.05628
Q86YV9	HPS6	-0.0198	0.522235	0.270564	-0.17941	0.358974	0.58038	1.512073	-0.37308	0.040847	-0.31984	-0.07163	0.271385	0.628707	0.210411	-0.02371	-0.4529	-0.0162	-0.35114	-0.23735
Q86YZ3	HRNR	-2.0676	3.845768	0.048245	-1.9133	-3.14455	-1.79586	2.777642	-1.08571	0.944762	-1.88226	-2.27918	-2.09552	-0.55963	-0.73175	-1.63186	-0.6567	-1.21878	-2.10259	-0.95089
Q8IU81	IRF2BP1	0.450406	-0.68303	-0.34811	-0.38344	-0.52156	0.017924	-0.12502	0.686502	-0.49744	-0.92015	0.245905	-0.63644	-0.15457	-0.24931	0.87026	-0.80818	-0.73135	-0.14069	-0.345
Q8IU85	CAMK1D	0.072129	-0.30116	-0.2	-1.09674	0.010331	-0.23129	-0.461	0.561524	-0.46577	0.350722	-0.7076	0.698815	-0.11663	0.609763	-0.64391	0.094077	0.478654	-0.38003	0.321142
Q8IUC4	RHPN2	0.446814	-0.43568	0.031191	1.009458	-0.21182	0.59623	-0.00231	1.679105	0.688542	-0.24084	-0.85835	0.159753	0.135176	-0.23009	0.163326	0.986642	1.404781	-0.37906	0.886559
Q8IUD2	ERC1	0.402028	0.165928	-0.31397	-0.41256	-0.27777	-0.49524	-0.72808	0.392373	0.268377	0.207506	-0.40205	-0.28887	0.790174	-0.39389	0.072518	0.276706	-0.28495	0.325133	0.615193
Q8IUD6	RNF135	-0.16787	0.034708	0.235281	-0.23007	-0.04429	-0.52524	0.165202	-0.82149	-0.1355	0.090217	0.247297	0.834366	-0.29218	0.394937	-0.59923	-0.31135	-0.14641	-0.88589	-0.85694
Q8IUF8	RIOX2	0.031689	0.020277	0.413187	-1.00717	0.747913	1.133747	-0.17057	-0.05444	-0.35484	1.154293	0.910926	-0.28045	0.122939	-0.20482	0.728681	-0.60058	-0.29381	-0.24375	-0.96263
Q8IUH3	RBM45	0.858087	-0.11613	-0.09398	0.361675	0.19024	-0.30091	-0.05897	0.060472	-0.09722	-0.82991	0.937583	0.234192	-0.36619	0.170224	0.342923	-0.95161	-0.28349	0.461951	-0.08273
Q8IUH4	ZDHHC13	-0.05473	0.079229	0.138899	0.082032	0.523468	0.529439	0.126452	0.663003	0.22908	0.099431	0.028437	-0.5726	0.010107	-0.03602	2.577896	-0.12035	0.151901	-0.11806	0.314898
Q8IUH5	ZDHHC17	-0.2903	0.532169	0.299175	-0.50679	0.370684	0.244024	0.754218	-0.07807	0.239988	0.032948	-0.04591	-0.61323	0.348751	0.308305	0.707241	0.288092	0.616663	0.849503	0.937358
Q8IUI8	CRLF3	-1.26069	0.300634	0.909462	-0.24264	0.571113	0.014413	0.077327	-0.99747	-0.17617	1.473925	-1.0851	0.946636	-0.34002	1.161118	-0.42146	-0.32351	0.43487	0.730057	-0.48432
Q8IUL8	CILP2	-1.03758	0.033058	-0.50293	-0.55298	-0.64156	-0.72357	-0.27633	-0.5108	0.624796	-0.89709	-1.20751	-0.04072	0.116241	-0.69905	-0.85844	-0.55432	-0.8975	-0.51751	0.660055
Q8IUR0	TRAPPC5	-0.3589	0.713775	0.289301	0.163337	0.190288	-0.01321	0.267066	-0.34939	0.414227	0.301171	-0.35097	0.388453	0.238527	0.589228	-0.19522	0.226583	-0.04441	0.031297	-0.30229
Q8IUR7	ARMC8	-0.19129	0.631055	0.293012	0.553096	0.15865	0.252999	0.215809	-0.09596	0.938434	0.354189	-0.20451	0.317312	0.235167	0.233534	0.678999	0.099113	0.070908	-0.46231	-0.201
Q8IUW5	RELL1	-0.10914	0.595562	0.355434	-0.57591	-0.41889	-0.74259	-0.01344	-0.2761	-0.2077	0.815064	-0.49761	-0.50002	-0.31627	-0.00798	0.066507	0.152739	-0.21724	0.294619	0.369267
Q8IUX4	APOBEC3	-0.17425	-0.40178	-0.01129	0.236123	0.340498	-0.43792	0.080535	-0.25775	-0.30447	-0.53563	1.242885	-0.54019	-0.71188	-0.25625	-1.31819	1.06146	0.710871	0.535322	-1.24744
Q8IUX7	AEBP1	-1.74929	-0.52345	-0.35629	-1.54024	-1.8985	-1.12516	-0.56266	-0.93588	1.359746	-1.08428	-0.5107	-1.50261	0.105417	-1.13242	-1.03336	0.288813	-1.33373	-0.93748	0.650365
Q8IUZ5	PHYKPL	-0.19272	0.374937	-0.39562	0.291026	0.331713	-0.26612	-0.11072	-0.28454	-0.04558	-0.29301	0.174067	0.198265	0.227207	0.35643	-0.19703	-0.43223	0.555442	0.860666	-0.05507
Q8IV04	TBC1D10(	-0.33414	0.032736	-0.04726	-0.7305	0.467283	0.629871	0.057264	-1.31541	-0.16071	0.113318	-0.69819	-0.4961	-0.18186	0.506162	-0.42737	-0.21578	-0.07741	0.07335	-0.89945
Q8IV08	PLD3	-0.2934	1.213362	0.453827	-0.95333	0.880948	-0.0625	0.785404	3.085638	-0.51124	-0.79595	-0.92579	-0.16691	0.722292	-0.77285	-0.37466	-1.4045	-0.704	-0.09433	2.819467
Q8IV36	HID1	0.925918	-0.42654	0.285342	0.781363	-0.65058	-0.51967	1.324968	0.473262	-0.44878	-1.49052	-0.03194	-0.48012	-0.09912	-0.97592	-0.92495	0.918999	-0.86748	-0.86106	-1.56542
Q8IV38	ANKMY2	0.556986	0.206539	-0.02399	-0.6739	0.225752	-0.21108	0.2874	0.820744	0.269776	-0.98651	-0.44651	-0.44014	0.060169	-0.18144	-0.13117	-0.21985	0.206613	0.804553	0.228785
Q8IV50	LYSMD2	-0.57466	0.751015	0.558685	-0.54384	-0.49164	-0.7054	0.205854	-0.93126	-0.02412	1.72124	-0.22851	0.381572	0.130214	0.925495	-0.59177	-0.303	0.177476	0.339023	-1.41905
Q8IVB4	SLC9A9	-0.86062	0.863624	0.975818	-1.18645	0.410876	-1.18298	0.630543	-1.61228	0.224595	0.919828	-0.06746	1.599453	0.113495	0.935044	0.844099	-0.00461	-0.16094	0.289978	-0.43622
Q8IVB5	LIX1L	-1.0459	0.119201	-0.22852	-1.05811	-0.13929	-0.71307	-0.37494	-0.66773	0.466763	0.502518	-0.47006	-1.34397	0.178878	-0.82506	-0.21222	-0.19091	-1.10114	1.168427	1.749292
Q8IVD9	NUDCD3	0.964138	-0.22609	-0.58547	-0.6996	-0.32163	-0.00422	-0.93806	0.747636	-0.27799	0.356531	0.114288	-0.44725	-0.04408	-0.43941	-0.14784	0.072852	-0.4001	-0.10711	0.30933
Q8IVF2	AHNAK2	0.242337	-0.78422	-1.49982	0.962604	-0.03419	-0.00371	-0.44135	0.058552	-0.42906	-1.75655	0.32445	-0.84541	0.272291	-0.94502	1.137234	-0.1289	-0.15043	-1.917	-1.19584
Q8IVF7	FMNL3	0.061706	0.477206	-0.5052	-0.96459	0.53834	0.724774	-0.04912	-0.46804	0.139595	0.547145	-0.49158	-1.11015	-0.08935	-0.63508	0.433794	-0.57436	-0.88078	-0.1133	0.295841
Q8IVG5	SAMD9L	-0.69748	1.450656	1.358294	-0.88345	0.209169	0.203862	0.329359	0.047592	0.203494	-0.20626	-0.71819	0.61761	0.062722	0.879713	-0.62432	0.082051	-0.07321	0.047672	-1.00354
Q8IVH4	MMAA	0.153338	-0.07509	-0.19087	0.743544	-0.12308	-0.48634	0.144188	-0.56561	-0.30609	-0.1599	0.020349	-0.38171	-0.02674	0.018378	0.226319	0.55572	-0.193	0.446054	-0.47675
Q8IVH8	MAP4K3	0.081269	-0.57918	-0.1759	-0.54721	-0.86544	-0.16004	-0.8164	0.673838	-0.32222	-0.75507	0.194093	-0.78864	0.134033	-0.56305	-0.15151	0.705884	-0.00963	0.087987	0.488071
Q8IVI9	NOSTRIN	0.559581	0.067371	0.029777	-0.14448	-0.3282	-0.10307	-0.17923	-0.02646	-0.31242	0.023162	1.355317	-0.60061	-0.03479	-0.57296	-0.23537	-0.41365	-0.87033	-0.06925	-1.19301
Q8IVL5	P3H2	-0.48912	-0.51806	0.141102	-0.16363	-0.6654	-0.78247	0.19991	1.133956	0.166685	0.243612	-0.31663	-0.22091	-0.28622	-0.12137	-0.81443	-1.00789	2.273622	1.608064	0.166701
Q8IVL6	P3H3	-0.12838	1.097662	0.034151	-0.81556	-0.01848	0.10646	-0.14349	0.04214	1.101736	-0.40908	-0.66383	-0.89732	1.326352	-0.60702	-1.55274	0.327262	-1.03807	0.760341	2.374782
Q8IVM0	CCDC50	-0.37969	0.048281	-0.05558	-0.42622	-0.33562	0.379432	-1.1563	-0.25938	0.153012	0.187281	-0.23066	-1.1184	0.15837	-0.90327	0.918747	0.060281	-1.00854	0.493365	0.854104
Q8IVP5	FUNDC1	0.148264	0.028137	0.120046	0.426779	0.380338	-0.89147	0.748715	-0.31556	-0.2616	-0.09749	-0.35009	-0.623	0.05994	0.120584	-0.04007	-0.14935	0.701368	-0.33695	-0.0974
Q8IVS2	MCAT	0.105943	-0.48136	0.405256	0.47265	-0.20285	-0.3585	0.049009	-0.71016	0.157688	-0.06528	1.167919	0.166456	0.222934	-0.03538	0.082945	0.848475	0.508559	0.180282	-0.37851
Q8IVT2	MISP	-0.59525	-0.73743	-0.7472	-0.33215	-0.24381	-0.0536	-0.56559	0.224936	-0.41117	-0.84307	1.117469	-0.8988	-0.41667	-0.50438	-0.75756	-1.68533	-0.58401	-1.56349	-1.03903
Q8IVT5	KSR1	-0.34591	0.177235	0.327571	-0.40071	-0.95351	-0.32645	0.555903	-0.56559	0.437199	-0.61476	-0.06207	-0.00899	0.166048	-0.12953	0.198479	-0.559	0.321553	0.398764	0.424131
Q8IVU3	HERC6	0.488856	-0.0818	-0.69487	-0.77861	0.099852	-0.26388	0.539589	1.037802	0.523828	0.231135	0.093252	-0.21894	0.207863	-0.93314	-1.31843	-0.17089	-0.46732	0.529247	-1.43042

Q8IW35	CEP97	0.09716	-0.21573	-0.56997	-0.89582	-0.02548	0.08865	-0.10301	0.002332	0.095257	-0.30756	0.621652	-0.75448	-0.07406	0.270066	0.469399	-0.5402	-0.19211	-0.0245	0.077827
Q8IW45	NAXD	0.081668	-1.12071	-0.30501	-0.32014	-0.28574	-0.34222	-0.19411	0.242949	-0.1156	-0.08858	1.229789	-0.38673	-0.54966	-0.39954	0.959653	-0.32372	-0.28267	-0.04103	-0.09511
Q8IW92	GLB1L2	1.250885	-0.20983	-0.4717	0.229078	-0.91363	-0.55862	-0.33649	-0.68428	-0.38759	-0.50132	0.531799	-0.06609	-0.03124	-0.2793	1.128023	0.395445	-0.5052	-2.07122	-0.95371
Q8IWA0	WDR75	0.124306	0.018892	0.390128	0.710488	-0.15166	0.012771	-0.01644	0.349586	-0.23416	0.177534	0.371688	-0.09967	-0.03126	-0.27595	0.415409	0.625088	0.019956	-0.55161	0.085145
Q8IWA4	MFN1	0.108713	0.160593	-0.08443	0.115726	1.005474	0.731248	-0.00769	-0.03366	0.415342	-0.61163	-0.39536	-0.53236	0.412176	-0.21218	0.859885	-0.17876	-0.47445	-0.50442	0.276923
Q8IWA5	SLC44A2	-0.11037	-1.15374	-0.37508	-1.05997	-0.70569	-0.32375	0.508677	0.53436	-1.13123	-0.61215	-0.3073	-0.61908	-0.95835	-0.66761	-0.76577	0.78413	-0.33201	-0.2862	-0.24507
Q8IWB1	ITPRIP	0.052445	0.990234	0.060772	0.404499	-0.28223	-0.80091	-0.09018	-0.11647	0.040847	0.200018	-0.02153	0.344681	-0.29961	1.1872	-0.76844	0.023245	0.452183	-0.25196	-0.10972
Q8IWB7	WDFY1	0.038229	0.911357	0.022531	-0.50671	-0.13133	0.072222	-0.07247	-0.23503	0.3403	-0.12572	-0.44006	0.121213	0.218119	0.540557	-0.22038	0.294341	-0.58449	-0.2096	0.486309
Q8IWB9	TEX2	0.356298	0.184095	-0.2056	-0.01095	-0.21047	-0.08855	-0.10649	0.203458	-0.28931	-0.00399	0.09129	-0.23614	-0.08917	-0.06663	-0.04065	-0.14849	-0.29462	-0.50401	-0.92097
Q8IWC1	MAP7D3	-0.08916	-0.16232	0.208977	0.28633	-0.20653	0.53691	-0.11402	-0.28407	0.362904	0.443394	-0.09921	-0.74631	0.406576	-0.22788	0.634443	0.494731	-0.02017	0.112207	-1.02973
Q8IWE2	FAM114A1	-0.28884	0.513201	-0.37565	-0.92895	-0.26195	-0.25264	-0.11815	-0.22021	0.32258	0.230175	-0.38173	-0.80285	0.846221	-0.76341	-0.47228	1.183642	-0.33145	0.266118	1.477042
Q8IWE5	PLEKHM2	0.029141	0.201182	-0.03393	-0.38298	-0.29387	-0.59888	-0.02995	0.024585	-0.3333	0.034427	-0.13989	0.230489	0.427835	0.506527	-0.17358	0.160638	0.018146	0.002839	0.448667
Q8IWF6	DENND6A	0.456997	0.285876	0.167023	-0.36482	0.069523	0.445154	0.096376	0.242687	-0.3602	0.097659	-0.88828	0.056128	0.340025	-0.75375	-0.42242	0.248582	-0.11254	0.116032	0.038138
Q8IWI9	MGA	0.567953	0.43097	-0.02648	-0.00225	0.058463	0.12383	0.072156	0.064752	-0.34628	-0.41666	-0.18531	-0.24411	0.220089	-0.34648	0.444675	-0.18986	0.011549	0.026952	0.076191
Q8IWJ2	GCC2	0.085269	0.205567	0.250398	0.439626	0.289543	0.345534	-0.12698	0.248411	-0.25978	0.013744	0.051874	-0.45538	-0.01275	-0.22803	0.257705	0.080213	0.082979	0.893926	0.292622
Q8IWL3	HSCB	-0.14543	-0.2218	0.477429	0.658804	0.285364	-0.24568	-0.05844	-0.41498	0.191953	-0.12273	0.862279	-0.50529	0.792071	-0.08987	0.34316	1.059692	-0.07608	0.973916	-0.9131
Q8IWR0	ZC3H7A	0.64279	-0.3833	-0.38476	-0.75404	-0.24475	0.140204	-0.02612	-0.45068	0.252252	-0.34664	0.566243	-0.70353	-0.0259	-0.41802	0.006892	0.001388	0.231596	0.163232	0.017304
Q8IWS0	PHF6	1.191257	0.46054	0.570824	0.73089	-0.08496	0.724854	-0.62371	0.945289	-0.15833	0.278908	0.992814	-0.84755	0.205338	-0.33207	-0.89294	0.009721	0.04673	-0.1669	-0.41776
Q8IWT0	ZBTB8OS	0.375354	-0.24013	0.657618	-0.1838	0.961956	0.236186	-0.24936	-0.22542	0.420857	1.64425	-0.78286	0.21936	0.468116	0.818458	0.473182	0.064384	0.264594	0.043849	0.119412
Q8IWT3	CUL9	-0.23143	0.322835	0.069767	-0.3714	-0.49025	-0.9462	-0.71469	-0.28774	-0.05232	0.884866	0.00256	-0.04237	0.910854	0.168225	-0.11603	0.190541	-0.31436	-0.09994	0.290218
Q8IWT6	LRRC8A	-0.19435	0.814115	0.146441	-0.42169	0.623024	0.192023	0.880992	-0.21762	0.040847	-0.3137	0.137598	0.075063	0.181599	0.271558	-0.0681	-0.5923	0.319752	-0.15412	0.087233
Q8IWU2	LMTK2	0.557388	0.349119	-0.01218	-0.66795	-0.32173	0.176729	-0.19271	0.432192	0.559125	0.120666	-0.20624	-0.0782	0.130229	0.325134	-0.44134	0.477094	0.341913	-0.94176	-0.70412
Q8IWU6	SULF1	0.940833	-0.7221	0.287522	-1.69261	2.182647	1.438084	-0.23248	-0.86558	0.444393	-1.08968	-2.1059	-0.57748	0.930659	-1.07332	-1.50503	1.092193	-0.80168	0.237869	1.124768
Q8IWV7	UBR1	0.201508	-1.34603	-0.24785	0.159802	-0.13215	-0.11663	0.338204	-0.48204	-0.02225	-0.2195	-0.18238	-0.01112	-0.38646	0.138092	-0.05579	-0.0559	0.668177	0.081716	0.170205
Q8IWV8	UBR2	0.164957	0.393653	0.237739	-0.15946	0.192741	0.242236	0.416021	0.163481	0.044834	0.187238	0.244951	0.230078	0.418548	0.043515	-0.10076	0.648594	-0.06002	-0.64514	0.017718
Q8IWW6	ARHGAP1	0.143569	0.248793	0.684965	-0.97887	-0.26677	-0.13383	0.237527	-0.07571	-0.13225	-0.11852	-0.23862	0.531559	0.018849	0.424255	0.35384	0.20025	0.530796	0.403612	0.010331
Q8IWX8	CHERP	0.358185	-0.48065	0.06042	0.164308	-0.48429	0.298751	-0.46815	1.128566	0.000186	-0.11931	0.355113	-0.59969	-0.26154	-0.20136	0.121804	-0.12146	0.278105	0.377236	-0.06494
Q8IWY9	CDAN1	0.61497	-0.24969	0.01555	0.290865	-0.50408	-0.19778	-0.01138	-0.52403	0.005392	-0.98117	-0.58572	-0.45112	-0.04322	-0.16245	-0.12456	-0.57213	1.259627	0.095347	0.267914
Q8IWZ3	ANKHD1	-0.48578	0.360735	0.145571	-0.01964	-0.23332	0.101138	-0.28134	0.352033	0.097507	-0.00892	0.084049	-0.10059	0.408454	-0.02801	0.627068	-0.17036	0.031259	0.05693	-0.16969
Q8IWZ8	SUGP1	0.451466	-0.16196	-0.05191	-0.03714	-0.20835	0.293394	0.046225	0.30026	-0.05554	0.281412	0.33677	-0.86565	-0.60147	-0.5099	-0.18366	0.39231	-0.08416	0.497701	-0.11865
Q8IX01	SUGP2	0.365253	-0.23121	-0.21466	0.067103	-0.52831	-0.38502	0.507008	-0.41475	0.040847	-0.40188	0.539169	-0.48571	-0.66363	-0.33899	-0.84769	0.070346	-0.21793	0.469409	-0.20703
Q8IX03	WWC1	0.136821	0.214822	0.057363	-0.01412	-1.69767	-0.45263	-0.02405	-0.29642	-0.30885	-0.16241	-0.06882	-0.59632	-0.4615	0.088804	-0.1651	-0.05714	0.234984	-0.91298	0.049695
Q8IX04	UEVLD	-0.65762	0.508516	1.152744	-0.49962	0.414491	0.821826	0.37735	-0.18766	0.054824	-0.08411	-0.23642	0.086058	0.050793	0.021225	0.409456	0.485004	-0.10391	-0.36568	-0.24343
Q8IX05	CD302	-1.01598	0.901266	1.363999	-2.11997	0.036265	-1.25293	0.7864	-0.91719	0.205781	0.580459	-0.56227	1.038727	-0.67949	0.759781	-0.27751	-0.43564	0.453435	1.57463	-0.54255
Q8IX12	CCAR1	0.371392	-0.33326	0.224098	0.21383	0.194603	0.345944	-0.87718	0.632632	-0.09523	0.180711	0.587241	-0.21567	-0.14689	-0.41837	0.253641	-0.14848	0.004262	0.204155	-0.27967
Q8IX18	DHX40	0.301013	-0.07889	-0.26664	-0.45174	0.060379	-0.04784	0.106182	0.166879	-0.38713	-1.34664	0.594067	0.232835	-0.1948	0.159116	-0.72296	0.036454	0.218693	0.30432	-0.17864
Q8IXB1	DNAJC10	0.247575	0.505743	-0.32957	-0.17501	-0.46761	-0.27876	0.185748	0.882527	0.245208	0.534043	0.153519	-0.5562	-0.03138	-0.42635	0.244069	-0.37541	-0.5862	-0.61729	0.386064
Q8IXH7	NELFCD	0.734794	0.112528	0.38423	0.174573	-0.05122	0.388323	0.515775	0.584195	0.058574	-0.11675	-0.30986	-0.07802	-0.0432	-0.20691	0.18436	-0.04159	-0.25883	-0.64696	0.323512
Q8IXI1	RHOT2	-0.12426	0.173878	0.12213	-0.14699	0.088385	-0.26436	-0.66764	-0.12022	-0.06888	-0.1671	0.859858	0.249913	0.149619	-0.22008	0.28205	0.931593	-0.43662	-0.20604	-0.30148
Q8IXI2	RHOT1	-0.3499	0.545115	-0.18767	0.926993	-0.02478	-0.44921	0.310212	0.174672	0.176284	0.322423	0.466755	0.043605	-0.52566	0.184425	0.214948	0.679175	0.060906	-0.08148	-0.24645
Q8IXJ6	SIRT2	-0.40959	-0.27037	-0.09785	-0.28778	0.758009	0.807147	0.2332	1.077355	0.480101	0.331558	-0.2858	0.183118	0.362999	0.489668	0.372881	-0.86015	-0.38598	-0.34152	0.882239
Q8IXK0	PHC2	0.54298	-0.20363	0.171065	0.351618	0.370127	0.464402	0.485903	0.581408	-0.44204	0.26327	0.172026	0.150374	-0.48278	0.192308	-0.33895	-1.20327	0.077647	-0.13134	0.364758
Q8IXK2	GALNT12	0.26023	0.145852	-0.26826	0.400896	-0.0815	0.095252	-0.2013	-0.85208	-0.14535	-0.17022	1.96748	-0.62731	-0.15065	-0.98142	-0.19254	0.323148	-0.47992	-0.0905	-0.15408
Q8IXL6	FAM20C	-0.78858	0.779837	0.406408	-0.19773	-0.57827	-0.34626	1.368079	0.027606	0.281712	0.311881	-0.5263	-0.16017	0.296785	0.654037	1.358303	-0.16227	0.266348	-0.67028	-0.2718
Q8IXM2	BAP18	0.131366	-0.13722	0.035211	-0.34629	0.631944	0.076962	0.493215	0.698633	-0.0858	-0.28063	0.06071	0.304566	0.222125	0.147964	-0.07897	-0.52888	0.051023	-0.00559	-0.06809
Q8IXM3	MRPL41	-0.54372	-0.06171	0.587921	0.90826	0.457855	0.650133	-0.33033	-0.46742	0.004752	0.348279	0.854358	-0.63208	0.112858	-0.47136	0.917571	0.126676	-0.63727	0.031702	-0.23352
Q8IXM6	NRM	0.456511	0.113759	-0.49225	-0.72826	-0.25783	-0.20475	0.129797	-0.79125	-0.97477	-0.75446	0.181268	-0.00187	0.151835	0.265813	-0.06278	0.295871	0.075742	0.039488	0.274477
Q8IXQ4	GPALPP1	0.041555	-0.05284	0.11225	-0.56016	0.31058	0.923476	-0.06639	1.048821	-0.26365	0.422239	0.57064	-0.50345	-0.02329	-0.13598	0.64209	0.539887	-0.10172	0.075572	-0.00767
Q8IXQ5	KLHL7	0.437805	-0.25908	-0.0886	0.043279	0.065133	0.144633	-0.10485	0.779558	-0.03491	-0.07012	0.279928	-0.27536	-0.01202	-0.61408	-0.20508	-0.19401	0.114753	-1.04001	-0.55149
Q8IXQ6	PARP9	0.612667	0.58712	1.241013	-0.79617	0.490314	0.69184	0.036314	1.071647	-0.02806	0.313899	-0.25993	0.747532	0.17711	0.682776	0.223128	-0.40853	-0.01169	-0.34202	-0.71254
Q8IXS6	PALM2	-0.25648	-0.70858	-0.76351	-1.30307	-1.48802	-1.26003	-0.16979	0.857912	0.284826	-0.07237	0.651374	-1.0447	0.518681	-1.12362	-0.87104	-0.16811	-1.246	0.253916	0.492789
Q8IXS8	FAM126B	-0.11927	0.242257	-0.19031	-0.15333	-0.6381	-0.57585	0.014428	-0.40913	-0.09573	-0.05319	-0.4995	0.669028	-0.21219	-0.41923	0.341891	-0.18699	0.369623	0.260558	-0.61556
Q8IXT5	RBM12B	0.510547	-0.14021	-0.18831	-0.14877	0.004374	0.131802	-1.10423	0.377118	0.06751	-0.09957	0.498562	-0.19214	-0.17732	-0.37192	0.061786	-0.50507	0.264692	0.373384	-0.00145
Q8IXW5	RPAP2	0.108085	0.303955	0.384128	-0.42459	-0.06863	-0.01377	0.544555	0.082917	0.003392	-0.07294	-0.08626	-0.51513	0.081705	-0.097	0.000421	-0.29512	0.503324	0.594381	0.601721
Q8IY17	PNPLA6	-0.90646	0.75682	-0.09502	-0.21071	-0.26029	-0.52209	0.350753	-0.27412	0.518744	0.227874	-0.36258	0.376049	-0.34043	0.435074	-0.41527	0.86597	-0.05458	-0.33692	-0.38983
Q8IY18	SMC5	0.336808	0.5216	0.033911	0.014705	-0.08031	-0.10626	0.16667	0.201319	-0.3242	-0.42024	-0.06185	-0.10018	-0.01423	-0.21142	0.401205	0.413199	-0.11766	-0.24145	-0.06996
Q8IY21	DDX60	0.416144	0.426487	1.274719	-0.95596	-0.11554	0.439904	-0.14299	0.266383	-0.04471	-0.68241	-0.0475	0.478726	0.160056	0.501308	-0.84019	-0.34748	0.017139	-0.50523	-1.37721
Q8IY22	CMIP	-0.04466	0.285599	0.0842	-0.28265	-0.19508	-0.1432	0.490958	-0.67523	0.518628	-0.12917	0.679963	-0.07586	0.133972	0.61728	0.028779	0.180843	0.096274	0.514916	-0.47026
Q8IY31	IFT20	-0.72368	0.12982	0.013462	-0.14576	0.024028	-0.08892	-0.09731	-0.27569	-0.16633	0.128538	0.698264	-0.26594	0.111786	-0.20918	0.755551	0.637556	0.008922	-0.34882	-0.04225
Q8IY33	MICALL2	-0.72361	-0.19809	0.310274	-1.25002	-1.10085	-0.43869	-0.48189	-0.57198	-0.04186	0.062414	0.120742	-1.73962	-0.69101	-0.62392	-0.30135	-0.15946	-0.69742	-0.11681	-0.30059
Q8IY34	SLC15A3	0.05221	0.007811	0.043452	-0.31213	-0.22666	-0.40567	-0.41965	-0.19046	0.003878	1.003217	-0.35659	0.965075	-0.12032	0.278745	-1.21475	0.24698	0.243133	-0.56851	-1.61622
Q8IY37	DHX37	0.116172	0.69264	-0.56987	0.189793	0.190259	0.140463	-0.06944	0.27799	0.773852	-0.2324	0.352167	-0.02243	0.522697	-0.04678	-0.76345	-0.18747	-0.0134	0.255607	0.642593
Q8IY47	KBTBD2	1.135416	-0.12738	-0.18655	-0.03442	0.065143	0.171146	-0.11799	0.524203	-0.25115	0.689309	-0.29993	-0.01011	-0.74137	0.199825	-0.245	0.313569	-0.00678	-0.3627	-0.22602
Q8IY67	RAVER1	-0.05972	-0.14198	-0.15151	-0.19562	-0.00718	-0.09075	0.336942	0.659561	0.218591	0.381044	0.74489	-0.04872	-0.02842	0.145769	-0.52615	0.622109	0.272371	0.405124	-0.11596
Q8IY81	FTSJ3	0.115217	0.099083	-0.56727	1.730479	-0.53726	-0.34501	-0.29916	-0.01645	-0.53682	-0.10049	0.313144	-0.49769	-0.13427	-0.89782	-0.47825	0.337643	-0.36973	-0.1029	-0.73201
Q8IY95	TMEM192	-0.17319	0.6918	-0.63088	-0.89411	0.092978	-0.35229	0.162599	-0.68013	-0.10142	0.142601	0.462092	0.575321	-0.42757	0.450723	-0.76059	-0.28098	0.094677	0.371991	-0.63135
Q8IYB1	MB21D2	0.002109	-0.00715	0.330428	-0.43586	0.518996	0.684373	-0.07398	-0.09606	-0.05057	-0.19784	0.078806	-0.75526	0.30896	-0.29176	2.101138	0.253996	0.260068	0.564288	0.690519
Q8IYB3	SRRM1	0.705258	-0.71022	0.53313	-0.21843	0.072048	0.142012	-1.09582	0.443052	-0.55419	0.333588	0.324063	-1.01968	-1.01536	-0.74454	-0.03622	-0.33465	-0.6521	0.457702	-0.27445
Q8IYB5	SMAP1	0.243597	-0.27425	0.171636	-0.28221	-0.28765	-0.24223	-0.00499	0.087667	0.86089	0.221942	-0.05011	0.16317	-0.36098	0.129088	-0.08446	-0.36176	0.121492	0.463274	-0.19017
Q8IYB7	DIS3L2	-0.31505	-0.061	0.022017	-0.29485	0.165217	0.426525	0.024919	0.110746	0.04188	0.076457	0.59347	-0.10482	0.529669	-0.16493	-0.27975	0.577183	-0.2497	0.052061	0.304141
Q8IYB8	SUPV3L1	-0.02961	-0.37689	0.66814	0.350704	0.397451	0.626004	-0.21403	0.193127	0.242329	0.240635	0.27817	-0.39967	-0.19384	-0.2691	1.239124	0.475915	-0.24597	-0.21319	-0.67846
Q8IYD1	GSPT2	0.153953	-0.06508	-0.23332	0.133834	-0.5103	-0.75825	-0.03084	-0.78444	0.1463	0.425962	0.749587	-0.30015	-0.04812	-0.08662	-0.68123	0.368662	0.942325	0.542808	0.757011
Q8IYI6	EXOC8	-0.34884	0.456081	-0.0822	-0.03868	-0.22842	-0.22637	0.273014	-0.1195	0.111167	-0.06552	0.188468	-0.09707	0.205288	0.351722	0.330169	-0.00882	-0.29309	-0.30174	0.246523
Q8IYJ3	SYTL1	0.818375	0.054867	-0.18476	0.623434	-0.65028	-0.49712	-0.41155	-0.85357	-0.75253	0.532654	1.860719	-0.36828	-0.20357	0.7231	-0.79552	-0.29818	-0.31292	0.039726	-1.7778
Q8IYM9	TRIM22	-0.10863	0.823536	1.123725	-0.95282	0.822402	0.976323	0.17211	-0.92071	1.101784	-0.52794	0.293063	-0.00537	-0.13419	0.368296	-1.21777	0.019716	-0.43475	0.684784	-0.47798
Q8IYQ7	THNSL1	-0.43266	-0.13972	-0.49431	0.341429	0.621978	0.36057	-0.24207	0.702463	-0.29154	-0.11909	0.507021	-0.98413	-0.41171	-0.63261	1.862965	0.432265	-1.11989	-0.67523	-0.21793
Q8IYS1	PM20D2	2.79267	-1.2973	-1.05387	0.198567	-0.56974	-0.08387	-1.58662	-1.48616	0.37122	0.171892	1.258913	-0.26723	-0.9826	0.337106	0.032448	0.315253	-0.19466	-0.82795	-1.69809
Q8IYS2	KIAA2013	0.293725	0.370166	-0.11504	-0.06228	0.236579	-0.02845	0.786795	0.084481	-0.69543	0.278522	0.141407	-0.03774	0.315422	-0.07869	0.609831	0.569095	0.885425	0.882717	0.068266
Q8IYU2	HACE1	-0.63934	0.349224	0.195337	-0.28336	-0.32785	-0.29648	0.303811	-0.58329	-0.08989	-0.14307	-0.02542	-0.20553	0.022094	0.209003	0.524135	-0.09327	-0.76189	-0.13666	-0.27013
Q8IYU8	MICU2	0.165795	0.028139	0.127758	-0.25919	0.58889	0.147482	0.231822	0.019266	0.039788	-0.1286	-0.15559	0.197123	0.102889	0.114738	0.084822	0.360196	0.696083	0.418514	0.097316
Q8IZ07	ANKRD13	-0.30664	-0.21146	0.361062	0.186605	-0.23851	-0.40462	0.008687	-0.20739	0.302294	0.909971	-0.78815	-0.35741	-0.26129	0.876902	0.51648	0.253123	0.028897	0.035094	0.097894
Q8IZ21	PHACTR4	1.066017	-0.28469	-0.44934	-0.47163	-0.31775	-0.00164	-0.63797	-0.02071	-0.02312	0.230804	-0.19046	-0.38985	0.27752	-0.17301	0.104093	0.619742	0.259586	0.775486	-0.20241
Q8IZ40	RCOR2	0.399645	-0.01156	-0.02052	-0.45963	0.002573	-0.75938	-0.13491	0.511622	0.298282	0.382127	-0.19483	0.521868	0.124333	0.231506	0.429907	0.028704	-0.14902	0.060431	0.858704
Q8IZ52	CHPF	-0.29839	0.707149	0.094514	-0.95084	-0.37554	-0.20415	0.28597	-0.18145	0.319015	-1.57439	-0.96553	-0.30303	0.950145	-0.29327	-0.9883	0.023177	0.025881	-0.15926	1.151999
Q8IZ69	TRMT2A	1.055835	-0.13633	-0.04301	0.342012	-0.40362	-0.43178	-0.49309	-0.43581	-0.19323	0.243781	0.1195	0.02955	-0.77193	-0.12735	-0.161	0.947833	0.817627	0.803126	-0.85655
Q8IZ73	RPUSD2	0.777006	-0.43333	0.247186	0.321759	0.540098	0.499189	-0.45093	0.354837	0.073343	0.413384	0.390601	-0.18613	0.339919	0.370011	-0.22951	1.198219	0.449187	0.133639	0.664591
Q8IZ81	ELMOD2	-0.45866	0.204042	-0.07558	0.580694	0.683847	0.712363	-0.54477	-0.05846	-0.04343	0.010522	0.47274	0.177346	-0.55087	0.337808	-0.78564	0.347168	0.076586	-0.03964	0.641992
Q8IZ83	ALDH16A1	0.035377	0.689339	0.085698	-0.2252	-0.44804	0.061706	1.07049	-0.14484	0.616381	0.148396	0.023668	-0.45424	-0.13714	0.025083	-0.23552	-0.49505	-0.20756	0.1613	-0.28388
Q8IZA0	KIAA0319L	0.287317	0.680997	0.030728	1.445091	-0.20774	-0.06168	-0.32685	-0.13944	-0.04301	0.600937	0.34864	-0.3762	-0.26002	0.087957	0.23312	-0.31067	0.113254	0.123119	-0.38814
Q8IZD4	DCP1B	0.139493	0.162406	-0.08071	-0.45273	0.050445	-0.74539	0.082416	-0.45355	0.105856	-0.69754	0.008224	-0.01051	0.382818	-0.54198	0.136258	0.140206	0.04979	-0.09254	0.048182
Q8IZE3	SCYL3	0.334192	-0.03888	-0.16935	0.730958	0.385567	-0.43054	-0.73584	-0.2694	-0.00177	0.084969	-0.15334	-0.19702	-0.13866	-0.04451	0.514401	0.151852	-0.1527	0.436611	-0.48915
Q8IZH2	XRN1	-0.6239	-0.04992	0.747582	-0.11372	0.22946	0.315711	-0.12283	-0.15972	0.496624	0.161595	-0.58839	0.151392	0.268698	0.118166	0.678217	0.21375	-0.26005	-0.24011	-0.03748
Q8IZL8	PELP1	0.262634	-0.32961	-0.29061	-0.19015	-0.21714	-0.1401	-0.17311	0.428546	-0.19239	-0.42416	0.512845	-0.42312	0.720303	-0.18783	-0.07503	-0.19971	0.176598	-0.15186	0.10187
Q8IZP0	ABI1	0.158412	-0.52765	0.122935	-0.55017	-0.17152	-0.34912	0.996317	-0.00904	-0.82274	0.375948	0.197244	-0.14943	-0.13335	-0.03905	0.211381	0.000312	-0.17202	-0.08793	-0.17585
Q8IZQ1	WDFY3	0.146608	1.341226	0.522228	-0.54467	-0.01448	-0.41423	3.203924	-0.47036	0.040847	0.192944	-0.22887	0.525489	0.997085	0.966398	-0.08173	-0.2206	0.075944	0.326675	-0.02119
Q8IZQ5	SELENOH	1.344186	-0.31728	-0.13167	0.05315	0.219394	-0.13231	-0.3197	0.224474	-0.43817	0.157025	-0.10869	-0.81764	0.07793	-0.21363	-0.17889	-0.62553	-0.9966	0.355993	-0.81118
Q8IZR5	CMTM4	-0.64324	-0.36376	-0.34173	0.327718	-1.48401	-0.7204	-0.55649	-0.42645	0.036686	-1.09182	0.95827	-1.03969	-0.28491	-1.30816	1.159276	0.76728	0.032398	0.327212	-0.69875
Q8IZV5	RDH10	-0.73944	-0.58488	0.379972	-0.45224	-0.86964	-0.78019	0.339877	0.441222	0.114327	-0.37116	-0.62172	1.007414	-0.58964	0.071771	0.038714	-0.57893	3.907332	5.476071	-0.88421
Q8N0U8	VKORC1L	0.279944	0.34625	0.066089	-0.55263	0.256205	0.725134	0.072065	0.000888	0.14641	0.068675	-0.00437	-0.00556	0.347752	0.222693	-0.58996	0.054386	0.206276	-0.69011	0.417359
Q8N0V3	RBFA	-0.67372	-0.42107	-0.13481	0.186072	0.19589	-0.51966	-0.20977	0.020997	-0.1165	0.44134	0.167137	-0.40131	0.067732	0.154001	1.092711	1.30736	-0.06731	0.054061	-0.09549
Q8N0W3	FUK	-0.85347	-0.22163	-0.46834	0.23222	-0.66424	-0.66678	-0.23184	-0.83074	0.70611	-0.38453	0.807619	-0.77012	-0.32216	-0.21804	0.829792	0.21688	-0.69745	-0.9017	-0.57386
Q8N0X4	CLYBL	-0.104	-0.68639	0.057453	0.126799	0.353863	-0.03089	-0.64045	0.150903	-0.50546	0.326001	1.11144	-0.98741	-1.37405	-0.71926	1.78857	-0.4671	-1.28533	-0.13405	-0.89711
Q8N0X7	SPART	-1.5363	0.492063	-0.02803	-1.92412	-0.80348	-1.10909	-0.2676	0.057976	0.627782	1.721416	0.285158	0.115901	0.292172	-0.02853	-0.20969	-0.6508	-0.53886	-0.02883	0.309763
Q8N0Z3	SPICE1	0.453412	-0.13131	-0.21025	0.085481	-0.23873	-0.59054	-0.29385	0.413518	0.591031	0.251304	0.254754	-0.66318	-0.2733	-0.00395	0.22684	0.211647	-0.37921	-0.35007	-0.14574
Q8N0Z6	TTC5	0.955229	-0.15176	-0.0272	0.137134	0.080076	0.253849	-0.21303	0.269519	-0.08323	0.287855	0.068299	-0.26273	-0.16754	0.065952	-0.11041	-0.12381	0.239669	-0.20475	0.339246
Q8N108	MIER1	0.518004	0.005508	0.000104	-0.2867	0.399053	0.41718	-0.07242	0.467958	-0.22941	0.097776	-0.06944	0.062801	-0.30681	0.163684	0.166484	0.211021	-0.04469	0.435131	-0.06709
Q8N118	CYP4X1	0.563904	-0.35097	-0.36998	-1.04552	-0.36258	-1.09639	-0.01873	-1.46343	-0.36222	-1.30013	0.228854	-0.3237	-0.38292	-0.75746	-0.30513	0.42737	-1.10647	-0.39286	-1.04041
Q8N122	RPTOR	0.223442	0.085097	-0.33121	-0.30019	-0.24722	-0.04357	-0.9178	-0.02918	0.588741	-0.14883	0.027672	-0.06619	-0.6193	0.145718	-0.34133	-0.06491	-0.05116	-0.31983	-0.36791
Q8N128	FAM177A1	0.344231	-0.1782	0.174278	-0.26474	-0.85746	0.057523	-0.0179	0.096217	0.417975	-0.82754	0.078562	-1.40428	-0.53445	-0.09363	0.060538	0.835152	0.775538	2.449014	0.164132
Q8N129	CNPY4	-0.15628	0.786091	0.652321	-1.12352	1.376016	-0.31089	1.065749	0.423464	0.065113	-1.13476	-0.19161	0.852468	0.266645	0.79984	-0.25447	0.577434	0.275758	-0.44418	-0.10862
Q8N137	CNTROB	0.270945	0.240338	-0.45659	-0.96889	-0.21984	-0.65769	-0.00048	-0.95005	0.557377	-0.72718	0.362578	-0.12249	-0.0774	0.224082	-0.51427	-0.57287	-0.35467	0.350595	-0.75958
Q8N142	ADSSL1	0.405084	-0.2676	0.001397	-0.05515	0.097823	0.549685	0.62249	0.425538	0.208482	0.25229	0.990463	0.36148	-0.07422	0.755877	-1.39964	-0.01259	0.454746	0.20804	-0.19331
Q8N163	CCAR2	0.855332	0.031324	-0.40176	-0.30655	-0.40584	-0.09964	0.084014	0.160796	-0.52888	-0.75452	0.999051	-0.20862	-0.329	-0.4277	-0.95087	-0.53119	0.19994	0.35238	0.779144
Q8N183	NDUFAF2	-0.02575	-0.08383	-0.21127	0.703878	-0.13457	-0.27018	-0.02067	-0.81499	-0.30361	-0.05676	0.438957	-1.00636	-0.1121	-0.52749	0.89222	0.332407	-0.53	0.326297	-0.70579
Q8N196	SIX5	-0.64974	-0.24888	-1.12309	-1.90999	-1.71739	-1.0408	-1.25993	-0.07115	0.735598	-0.23184	-0.55687	-1.00354	-0.26504	0.655008	0.783592	-0.1057	-0.46262	0.05389	0.418507
Q8N1B4	VPS52	0.121439	0.54053	-0.05563	-0.27227	-0.05911	-0.08319	0.528603	-0.03008	-0.39575	0.33885	0.078937	0.188169	0.470881	0.019396	0.774136	0.105755	-0.27489	-0.46358	-0.14864
Q8N1F7	NUP93	-0.17033	0.200999	-0.1809	0.190392	-0.07712	0.072774	0.120926	0.174583	-0.14336	-0.05967	0.299577	-0.04297	-0.10449	-0.02147	0.785419	-0.05244	0.071794	-0.12498	0.287999
Q8N1F8	STK11IP	-0.32824	0.331276	0.089958	-1.01637	-0.05471	-0.07941	0.162707	-0.31807	0.082157	0.552859	-0.309	0.36008	-0.50931	0.594372	-0.73415	-0.1345	0.223461	0.361388	0.12714
Q8N1G0	ZNF687	0.937858	-0.44728	-0.63459	0.131913	0.122207	0.07598	0.093113	1.625711	-0.04891	-0.68028	-0.16273	0.087911	0.122451	-0.56878	0.595179	-0.01951	-0.01867	-0.25593	-0.03085
Q8N1G2	CMTR1	0.668655	0.021408	0.361553	0.09086	0.331576	0.521655	-0.5688	0.318431	-0.1419	-0.654	0.053622	-0.02032	-0.07833	-0.01261	-0.08039	-0.28143	1.089007	1.684888	0.368955
Q8N1G4	LRRC47	0.61568	-0.26585	-0.11274	0.011118	-0.24897	0.003474	-0.49053	-0.08158	-0.40525	0.073112	0.383844	-0.71401	-0.879	-0.37702	0.301942	0.729046	0.329244	0.296602	-0.28645
Q8N1I0	DOCK4	-0.77174	1.057641	0.098445	-0.50901	0.43313	0.068562	-0.36574	-0.4526	-0.17007	0.478157	-0.66546	0.747442	0.206562	0.773677	0.01437	-0.18193	0.051792	-0.43115	-0.34045
Q8N1N4	KRT78	-2.07777	3.450842	0.600018	-1.69724	-1.69746	-0.67777	2.878355	-0.35672	0.953114	-0.53548	-0.26174	-0.9375	-0.07459	-0.08974	-0.47879	-0.25054	-0.82819	-1.03883	0.031175

Q8N1S5	SLC39A11	-0.13768	0.181701	0.117583	0.211486	-0.01643	-0.65017	-0.27041	-0.32196	-0.18349	0.459064	0.285718	0.436014	-0.0047	0.381753	-0.38055	0.491841	-0.00892	-0.36877	-0.58192
Q8N1W1	ARHGEF2	0.198238	0.049693	0.318684	-0.73855	0.094559	-0.5352	0.194183	-0.30354	-0.06519	-0.15656	-0.39322	0.007874	-0.15322	-0.15537	0.858566	-0.12589	0.841694	-0.44177	-0.08209
Q8N201	INTS1	0.014992	0.221554	0.027929	-0.0907	-0.16329	-0.14939	0.86412	0.799631	0.751457	0.189711	0.583977	-0.25142	-0.19271	-0.23995	0.466015	0.026615	-0.14911	-0.01649	0.274786
Q8N257	HIST3H2B	1.266003	-0.5703	0.248717	0.107151	0.763461	0.444734	-0.26948	0.780943	-0.24764	-0.38313	-0.0167	-0.08028	-0.67579	0.606117	0.32397	-0.3395	0.149357	0.443933	-0.52529
Q8N271	PROM2	-0.53488	-0.55493	-0.46409	-0.2432	-1.22029	-0.90947	-0.19597	-1.5628	0.337117	-2.53774	0.6987	-0.80488	-0.6986	-0.43528	0.597645	-1.69147	0.130537	-0.67582	-1.20481
Q8N2F6	ARMC10	0.117958	-0.09091	-0.89939	0.184245	0.033684	-0.00091	-0.39744	0.019787	-0.13264	0.029797	1.055797	-0.49988	-0.50108	-0.21741	0.105892	0.865521	-0.97835	-0.43578	-0.77069
Q8N2G8	GHDC	-1.18827	0.94231	1.217037	-0.17414	-0.16318	-0.46804	0.770495	-1.15935	-0.97337	-0.01731	0.324064	0.520234	-0.09505	0.296111	-0.97547	0.10078	0.08383	0.136218	-0.54285
Q8N2H3	PYROXD2	0.901314	-0.08919	-0.30864	0.442905	0.189521	-0.01907	-0.2656	-0.78771	-0.57619	-0.34671	1.435046	-0.27246	-0.07222	-0.13708	-0.39552	-0.34484	-1.25354	-0.26628	0.24662
Q8N2H4	SYS1	-0.03458	0.08106	0.067993	0.005826	0.006216	0.287025	-0.11778	0.341416	0.238208	0.168749	-0.53231	-0.16398	0.040167	-0.52853	0.783405	-1.17367	0.540499	0.462176	0.251338
Q8N2K0	ABHD12	-0.09918	0.309597	0.995596	0.212689	-0.27554	-0.21917	0.669202	0.095458	-0.74939	0.310029	-0.23966	0.665242	0.083501	0.458101	0.345759	-0.22763	0.54993	0.649416	-0.2696
Q8N2M8	CLASRP	0.074536	-0.00693	0.138398	0.109807	-0.21661	0.610406	-0.09787	0.544193	-0.11285	-0.61526	0.397597	-1.07151	-0.41301	-0.32221	-0.04895	0.371539	0.316162	0.295779	-0.11942
Q8N2S1	LTBP4	-0.23579	-0.28848	-1.34704	-2.07874	-1.69875	-1.67349	-1.54023	-1.09823	-1.65826	-1.19894	1.02628	-0.68502	-0.6361	-0.41222	1.074015	-0.99864	-0.48968	-1.54195	0.117259
Q8N2U9	PQLC1	-0.29134	0.909113	0.450045	-0.00761	0.786437	-0.25081	0.103583	-0.15831	0.089739	0.515408	-0.79078	0.21445	-0.04644	0.563466	-0.45596	-0.02372	-0.46119	-0.33686	-0.33674
Q8N335	GPD1L	1.09946	-0.95939	-0.83941	-0.80652	0.112833	0.006809	-0.7907	-1.16314	-0.38726	0.288333	-0.40629	-0.21194	-0.635	-0.36554	0.560398	-0.14527	-1.52466	-1.68986	-1.66696
Q8N357	SLC35F6	-1.24756	0.403347	0.076406	-0.04128	-0.21245	-0.24758	0.313243	-0.47352	-0.10305	0.01779	-0.0107	0.715542	-0.25206	0.509368	-0.23892	-0.30863	-0.39717	0.110144	-0.25643
Q8N392	ARHGAP1	-0.73631	0.755612	0.073932	-0.21449	0.176793	-0.16436	0.211866	-1.07938	-0.20478	0.596436	0.835833	0.333414	0.138795	0.178625	-0.46706	0.118264	-0.19836	0.602406	0.488148
Q8N3C0	ASCC3	-0.41348	0.494811	0.514069	0.461344	0.020581	0.059882	0.160493	-0.7229	0.511674	0.220175	-0.02905	0.189827	0.166428	0.027016	0.316071	-0.02243	0.214453	0.117352	0.019188
Q8N3D4	EHBP1L1	-0.33471	0.316469	0.608209	-1.00149	-0.11586	0.010958	0.15684	-1.43443	0.541554	0.671261	-0.28177	0.866168	0.108439	0.713725	-0.36323	0.100451	0.187494	-0.12152	-0.47019
Q8N3E9	PLCD3	-0.38824	-0.71709	-1.65091	1.02459	-0.92053	-0.99246	0.194269	-0.67177	-0.71735	-1.31707	0.450563	-0.04888	0.20813	-0.24258	1.015011	0.430895	-0.19174	-1.10159	-0.89135
Q8N3F8	MICALL1	-0.11505	0.259481	-0.34338	-0.41661	0.104928	0.275392	0.452734	-0.07295	1.643862	-0.76017	0.547676	-0.14085	0.13847	0.303727	-0.76222	-0.49706	0.352763	-0.00604	0.088861
Q8N3P4	VPS8	0.031398	0.401388	0.091271	0.171905	-0.14634	-0.20929	0.785359	-0.13031	-0.20764	0.389656	0.048218	0.302714	0.077895	0.375907	0.10336	-0.18946	-0.4305	-0.45847	0.293612
Q8N3R9	MPP5	0.665575	-0.15398	-1.07253	-0.13666	-0.78237	-0.50144	0.50279	0.570815	-0.13956	-0.68015	-0.04388	-0.66613	-0.00066	-0.24259	0.138854	-0.888	0.188128	0.065473	0.403392
Q8N3U4	STAG2	0.678486	0.276156	-0.21738	-0.23687	0.964497	1.265131	-0.39021	-0.06968	-0.17018	-0.03046	0.30559	-0.5937	-0.71592	-0.10825	-0.07137	-0.02702	0.181751	0.250462	-0.24287
Q8N3V7	SYNPO	-0.80835	-0.52469	-0.99187	-0.38908	-0.41727	-0.09081	-0.77279	-0.85033	0.97296	0.057652	0.548388	-1.28435	0.854328	-0.78886	-0.38091	-0.32114	-0.65228	-0.22662	1.052563
Q8N3X1	FNBP4	0.645692	-0.22214	-0.03867	-0.33413	0.093994	0.833404	0.029339	0.821629	-0.22752	-0.22951	0.063943	-0.33847	-0.21567	0.127992	0.228588	0.200485	0.012638	0.180798	-0.48184
Q8N3Y1	FBXW8	1.129705	0.37233	-0.24925	0.222209	-0.34852	-0.82129	-0.06082	0.043253	0.351796	-0.15786	-0.23701	-0.1589	0.446655	0.013352	-0.00252	0.371015	-0.36321	-0.35089	0.585808
Q8N436	CPXM2	-1.3018	1.81585	-0.05369	-1.28005	-1.968	-1.31631	1.295255	-0.54816	0.898072	-1.08313	-0.8368	-1.074	0.073802	0.247007	0.196379	-0.37174	0.24944	-1.14877	-0.55428
Q8N442	GUF1	-0.50059	0.046217	0.142938	-0.12693	-1.15588	-1.43418	-0.55962	-0.24875	-0.39676	0.391092	0.9025	-0.19892	-0.25983	0.25085	-0.0071	0.074067	-0.1938	-0.71845	-0.09352
Q8N465	D2HGDH	0.179348	-0.47316	-0.84079	-0.41258	-0.90585	-0.48331	-0.17283	0.497185	-0.18224	-0.24911	1.339213	-0.27977	-1.32063	-0.20303	-0.32598	0.358442	-0.16857	0.105599	-0.92748
Q8N490	PNKD	0.472	0.643276	0.287807	0.034696	0.437168	-0.26352	-0.11479	-0.11576	0.301788	-0.42716	0.128971	-0.09115	0.649986	0.889785	-0.04768	-0.2171	0.062824	0.14745	-1.1493
Q8N4A0	GALNT4	-0.24678	-0.0849	-0.28453	1.069293	2.583921	1.983419	-0.30093	-1.17663	-0.54328	-1.1525	1.019814	-0.34409	-1.01879	0.411811	-0.98156	-0.97226	0.099016	0.296523	-0.4182
Q8N4C6	NIN	-1.09216	0.567835	-0.01616	-0.17736	0.133974	-0.32892	0.375111	-0.30368	0.142404	0.129828	-0.2374	0.079138	-0.18273	0.685833	0.266373	-0.01965	0.601443	0.890141	-0.67864
Q8N4C8	MINK1	-0.06096	0.45619	-0.11281	-0.83616	-0.71642	-0.41915	-0.30385	-0.40927	0.640436	0.160991	0.454623	-0.21027	0.605167	-0.50283	0.333429	0.23974	0.099201	-0.38273	-0.46761
Q8N4H5	TOMM5	0.612697	-0.18463	-0.58414	0.407617	0.080609	0.208965	0.190797	-0.04884	-1.18696	-0.2769	0.355613	0.306123	0.420134	0.005991	0.932201	0.331726	0.375331	-0.09114	-0.77176
Q8N4N3	KLHL36	0.125977	-0.08389	-0.13597	0.089612	0.077513	-0.05053	0.405335	-0.22106	-0.9884	-1.53808	0.379894	-0.08838	-0.35032	-0.18508	0.570337	-0.19008	-0.40762	0.032171	-0.42792
Q8N4P3	HDDC3	-0.03827	-0.38447	-0.14768	1.519191	0.813538	0.199844	-0.29635	0.229932	0.242698	0.110857	1.437978	-0.50569	-0.11745	0.094932	0.986102	-0.20453	-0.76988	0.015387	-0.14976
Q8N4Q0	ZADH2	-0.03527	-0.19665	-0.03504	-0.00041	0.666286	0.379375	-0.33414	-0.4007	-0.05978	-0.16633	1.407356	-0.37595	-0.00258	0.296532	-0.40294	0.938142	-0.1248	-0.48094	0.086527
Q8N4Q1	CHCHD4	1.349837	-0.34077	-0.69629	-0.55903	0.477731	0.363916	-0.19899	-0.26439	-0.40965	-0.48907	0.555554	-1.36033	-0.40404	-1.1551	-0.06651	1.303047	-1.88945	-0.94961	-0.88699
Q8N4T8	CBR4	0.589173	-0.51112	-0.74243	0.144406	0.886395	0.205819	-0.47044	-1.04234	-0.64504	-1.51683	1.440711	0.025768	-0.36182	-0.14364	-1.73135	1.100314	-0.99151	-1.18503	-1.27948
Q8N4X5	AFAP1L2	-0.94655	-0.77868	0.471553	-0.24739	-0.41312	-0.80678	-0.73058	2.532461	0.476334	-0.1373	-0.46297	-0.6056	1.120027	-0.23025	-0.50491	-0.03208	-1.0614	-0.22758	0.220401
Q8N511	TMEM199	0.113098	0.04959	0.024311	0.23848	-0.83708	-0.76019	-0.0719	-0.1062	-0.23363	0.292715	-0.3353	0.218488	0.108702	0.068361	-0.19518	0.875268	0.892631	0.43135	-0.44903
Q8N531	FBXL6	0.039644	0.357489	0.024939	-0.18819	-0.04959	0.30039	0.15016	-0.11106	-0.22338	0.037066	-0.46988	0.27065	0.313199	-0.0424	0.100195	-0.0268	0.50565	-1.14671	0.448614
Q8N543	OGFOD1	0.827015	-0.3134	-0.07474	0.359587	1.011884	0.516216	-0.34577	0.430097	-0.18566	-0.05563	0.2853	-0.22758	-0.02185	-0.0804	-0.05672	0.008127	0.301566	0.258427	-0.07044
Q8N556	AFAP1	0.350186	0.099551	0.255305	-0.53282	-0.8621	-1.02444	-0.21221	-0.34352	0.19552	-0.32976	-0.73452	-0.86424	0.026958	-1.04355	-0.68028	-0.04417	-0.02667	0.42734	0.466853
Q8N568	DCLK2	0.110499	0.300608	0.346744	-0.75064	-0.1531	-0.42786	0.306338	-0.36976	0.040847	-0.43233	-0.70106	-0.66906	-0.51695	0.058581	0.25022	0.536788	-1.36729	0.178377	-0.92327
Q8N573	OXR1	0.279821	-0.1081	-0.80762	-0.33366	-0.44545	-0.67085	-0.27729	-0.89925	0.432742	-0.40416	0.193486	-0.20516	0.951009	-0.2533	0.151261	-0.59725	0.272644	0.502754	-0.29504
Q8N584	TTC39C	-1.06112	0.270503	0.182951	-0.9233	0.016239	-0.44655	0.255578	0.477002	0.781142	1.090312	-0.7303	-0.51773	0.175644	-0.45378	1.775147	-1.19544	-0.4696	0.522179	0.683639
Q8N5A5	ZGPAT	0.590541	-0.0666	0.470385	-0.44223	0.096754	0.748652	0.848114	1.069933	0.163864	-0.27046	-0.35452	-0.46376	1.179181	-0.07668	0.453877	0.448232	0.243557	1.162733	-0.04122
Q8N5C6	SRBD1	-0.42697	0.232585	-0.04085	-0.01709	0.171007	0.119897	0.586586	0.24756	0.017695	-0.16807	0.485334	-0.29065	-0.18819	-0.05379	-0.09241	-0.06076	-0.46641	0.253317	0.326005
Q8N5D0	WDTC1	-0.58628	0.22631	-0.08665	-0.29459	-0.31413	-0.20384	-0.22802	-0.21252	-0.30719	-0.22941	0.064849	-0.13058	-0.05989	0.445853	0.936763	-0.48166	-0.41204	0.333932	-0.73406
Q8N5G0	SMIM20	0.627717	-0.14236	0.248419	0.396637	-0.1917	-0.11653	0.154117	-0.24137	0.390711	0.268418	0.667355	-0.90251	-0.15593	-0.92267	0.31271	1.229983	-0.94246	-0.1499	-0.23629
Q8N5G2	MACO1	0.086816	-0.22369	-0.17729	-0.35766	0.012898	0.053484	-0.03578	-0.0545	0.076564	-0.65053	-0.01609	-0.41824	-0.19385	-0.63657	0.710275	-0.19556	-0.12986	0.536637	0.314484
Q8N5H3	FAM89B	0.226611	-0.04925	-0.16191	-0.84397	-1.38256	0.320999	-0.00117	-0.06252	-0.35813	-0.35712	0.025157	-0.40319	-0.82249	-0.85746	0.010385	0.133787	-0.14201	-0.33813	-0.4944
Q8N5I2	ARRDC1	0.039327	0.220363	0.451772	-0.25773	-0.02222	-0.38948	0.337301	-0.44205	-0.04032	-0.06116	0.405699	0.131992	-0.36179	0.369163	-0.09335	-0.57663	-0.12273	-0.40808	-0.97067
Q8N5J2	MINDY1	-0.48019	0.118736	-0.00828	-0.36508	-0.75144	-0.62873	-0.06964	-0.16731	0.040847	-0.41299	-0.00122	-0.44902	0.022469	0.7726	1.453524	-0.72505	-0.0943	0.544272	-0.67635
Q8N5K1	CISD2	-0.41267	0.724039	0.250529	-0.45778	0.255107	-0.07644	0.366666	-0.12029	-0.70462	-0.66177	0.294837	1.01568	0.302821	0.492062	-0.33488	-0.57454	0.802087	0.606294	-0.34559
Q8N5M1	ATPAF2	0.037773	-0.06624	0.004233	0.419003	-0.19661	-0.3819	-0.30929	0.971586	-0.13555	0.148998	0.442413	0.006477	-0.3231	0.195447	-0.04981	1.399708	-0.99327	0.085346	-0.17831
Q8N5M4	TTC9C	-0.0782	-0.26928	-0.15711	0.020466	0.311032	0.168208	-0.45702	-0.09592	-0.29881	0.172419	0.989127	-0.24084	-0.00064	0.098967	-0.35405	-0.38264	0.455955	-0.71174	-0.58477
Q8N5M9	JAGN1	1.017672	-0.29064	-0.21169	-0.47132	0.396465	0.394999	-0.27502	-0.03007	0.573285	-0.69774	0.57371	0.301775	-0.20517	-0.03753	-0.17559	-0.1009	-0.04369	-0.61364	-0.12181
Q8N5N7	MRPL50	-0.28674	0.080578	-0.35369	0.579717	0.04891	0.154934	0.797906	-0.42177	-0.93116	0.280275	0.630743	-0.15488	0.086138	0.064745	0.565395	-0.26926	-0.16799	-0.38904	-0.58574
Q8N5V2	NGEF	0.41498	0.313634	-0.43074	-0.45435	0.390311	0.543162	0.447613	0.055696	0.040847	-1.48779	0.016674	-0.45978	-0.1054	-0.5006	-1.63817	-1.29566	0.891833	-0.07439	-0.35839
Q8N612	FAM160A2	-0.13429	0.492848	0.128442	-0.26803	-0.07089	-0.10625	0.183877	-0.12102	-0.25119	-0.18173	-0.13965	0.004748	0.076099	0.856098	0.359603	-0.0046	0.47145	0.293906	0.233805
Q8N653	LZTR1	-0.19545	0.243338	0.111169	-0.19636	0.482047	-0.13367	0.542411	-0.14487	-0.02679	-0.58088	-0.58845	0.357556	0.204753	0.683069	-0.17295	-0.35433	0.320087	0.075297	0.07318
Q8N668	COMMD1	-0.4786	-0.15435	0.091535	-0.23425	0.2601	0.58493	0.01006	0.035076	0.157618	1.439678	-0.3689	0.308972	-0.13195	0.488037	-0.04604	0.047574	0.542249	-0.65079	-0.2586
Q8N684	CPSF7	0.877123	-0.03436	0.119515	0.418529	-0.28167	-0.43753	-0.2955	0.395276	0.242845	-0.13147	0.43709	-0.62566	0.224924	-0.49867	0.266875	-0.00683	-0.34052	0.24611	0.03946
Q8N6G5	CSGALNA	-0.67153	0.338086	0.361336	-0.7295	0.106181	0.033863	-0.02305	-0.28909	-0.05688	0.211889	-0.29899	0.461459	0.162588	0.573621	-0.54911	0.035238	0.844215	0.243233	0.181679
Q8N6G6	ADAMTSL	-1.69075	0.382859	0.25621	-0.81709	-0.46994	-0.142	0.546958	-1.22416	0.421484	-0.74407	-0.19464	-0.4251	-0.48847	-0.43053	0.636263	1.210097	-0.87832	0.199425	-0.80252
Q8N6H7	ARFGAP2	0.204796	0.055545	-0.25769	-0.03063	-1.12285	-0.34622	-0.60303	-0.43448	-0.03278	0.415676	-0.31339	-0.11362	0.358438	0.092093	0.627535	0.556472	0.240158	0.506272	0.089471
Q8N6M0	OTUD6B	0.398023	-0.17861	0.627311	-0.32673	-0.27085	0.175976	0.288598	-0.32573	0.322247	0.282815	0.131006	0.227787	0.489591	-0.31603	0.022163	1.311272	-0.14679	-0.36937	-0.74373
Q8N6R0	METTL13	0.365123	-0.05585	-0.69777	0.814721	-0.24615	-0.19794	-0.31532	0.287877	-0.61575	-0.2932	0.488426	-0.28061	-0.30553	-0.22442	-0.24008	-0.61329	-0.25411	-0.37871	-0.20876
Q8N6T3	ARFGAP1	-0.25931	0.629112	0.39515	-0.00335	-0.11174	0.283231	1.061427	0.43346	-0.09024	0.330037	-0.36618	-0.07146	0.416814	-0.07516	0.391652	0.040929	0.320433	0.748834	0.943218
Q8N6T7	SIRT6	-0.56738	0.102777	-0.05707	-0.34567	0.351021	0.170167	-0.35862	-0.45076	0.002097	-0.0741	1.080294	-0.29095	-0.06945	0.334417	-0.04518	0.027472	-0.39781	-0.04216	-0.24586
Q8N6Y2	LRRC17	-0.56781	-1.1782	-1.08927	-0.54238	-1.85941	-1.12009	-0.74637	-1.08527	-0.49877	-1.30057	-0.43416	-1.60641	-0.83367	-1.91135	-0.73366	-1.64988	-0.92188	-1.27398	-1.19123
Q8N766	EMC1	0.02611	0.658102	0.20422	-0.06491	0.370898	0.167388	0.567258	-0.13844	0.022199	-0.03425	0.151247	0.210546	-0.09237	0.195855	0.331228	0.26759	0.118344	-0.1976	-0.09199
Q8N7H5	PAF1	0.564576	-0.23847	0.085135	-0.17932	0.094535	0.276713	0.102264	0.914369	-0.0514	0.059951	0.247698	-0.13934	-0.9143	-0.19713	0.316411	-0.18593	-0.26443	0.305105	0.173952
Q8N7R7	CCNYL1	-0.50314	1.016081	0.359565	-0.05113	-0.05613	-0.44052	0.572024	0.127108	-0.0605	-0.15303	-0.50762	0.543559	-0.04546	0.520198	0.562678	0.075362	0.6874	1.17211	0.266891
Q8N806	UBR7	0.297905	-0.23621	-0.21817	0.140668	1.663032	1.180167	-0.53816	0.624722	-0.19907	0.357275	0.048123	-0.17175	-0.26769	-0.23982	-0.48347	0.35871	0.454447	0.114246	-0.24856
Q8N884	CGAS	0.949351	0.220096	0.144894	-0.37249	0.435238	0.731661	-0.23202	-0.65763	0.323255	0.380486	-0.50975	-0.29542	0.501785	-0.12233	-0.96164	-0.38129	0.275534	-0.47966	0.174087
Q8N8A2	ANKRD44	-1.10366	1.045982	0.729137	-0.59101	0.368142	-0.04831	0.446281	-1.20856	-0.11358	0.841122	-1.31527	0.798468	-0.48592	1.557249	-0.33772	-0.62117	0.141184	0.19377	-0.60092
Q8N8A6	DDX51	-0.10137	0.465493	-0.5573	0.586073	0.17644	0.143109	-0.52212	0.358597	-0.23578	-0.64818	0.448199	-0.00227	-0.25855	-0.00253	0.225906	-0.56552	-0.5688	-0.55689	0.380564
Q8N8L6	ARL10	0.160927	0.368277	-0.30116	-0.6654	-0.66107	-0.77768	0.065267	0.033036	0.671735	-0.10255	-0.14529	0.267946	0.240066	0.022537	-0.70196	0.027602	0.129779	0.979096	0.490679
Q8N8N7	PTGR2	-0.37606	-1.27548	-0.10088	-0.02872	0.266026	-0.58325	-0.29517	-0.07813	0.602078	0.582985	-0.22393	0.668618	-1.17457	1.059278	-0.53552	-0.03298	0.808613	0.016341	-0.99198
Q8N8R3	SLC25A29	-0.16605	-0.09689	0.128569	-0.6037	0.067208	0.702108	-0.39855	0.802751	0.499733	-0.43026	-0.26594	0.282993	0.190937	-0.0131	1.956623	-0.21769	0.078887	0.507049	-0.57712
Q8N8R5	C2orf69	-0.30304	0.309065	-0.38985	-0.53526	0.084536	0.130519	0.23039	0.087328	0.2387	0.168669	-0.12475	-0.41958	-0.08506	0.23723	-0.04882	-0.27942	-0.14366	0.186642	-0.78033
Q8N8S7	ENAH	-0.89503	-0.60396	-0.68226	-0.6443	-1.80246	-1.18957	-0.41923	0.783143	0.655815	0.319532	-0.60644	-1.4093	1.062865	-1.45659	-0.17446	-0.28659	-1.49507	-0.82809	1.528697
Q8N960	CEP120	-0.3555	0.283497	0.123826	-2.38879	-0.10551	-0.2628	0.032861	-0.84595	0.853445	0.068799	0.246797	-0.69069	-0.09708	0.205754	-0.43798	-0.50808	-0.52845	0.374682	-0.20907
Q8N983	MRPL43	-0.52623	-0.10294	0.379787	0.734236	0.17164	0.612052	-0.21489	-0.40213	-0.3876	0.514199	0.48142	-0.38964	0.075984	-0.32335	0.46769	-0.10389	-0.25229	-0.0368	-0.2958
Q8N9B5	JMY	-0.50038	0.467201	-0.81123	-0.18335	-0.52722	-0.47157	0.906852	0.243673	-0.1136	-0.15398	-0.18825	-0.12483	0.24396	-0.33334	0.242511	1.24401	1.07569	0.478986	0.029873
Q8N9F7	GDPD1	-0.89865	0.328429	-0.29606	-0.49899	-0.04224	-0.82834	0.310338	-0.00747	-0.48415	-0.20844	-0.29495	0.816949	-0.10237	0.839535	-0.14483	-0.01393	0.16762	0.019415	-0.30664
Q8N9N2	ASCC1	-0.17965	0.201322	0.857391	0.283322	0.249671	0.026678	-0.47836	-0.38069	0.148789	0.535958	0.280665	-0.23587	0.18635	-0.6166	0.100057	0.094877	0.215333	0.191024	0.195903
Q8N9N5	BANP	-0.04578	0.316338	0.059444	-0.24836	0.518174	-0.00082	0.098365	0.010078	0.258567	0.155819	0.318484	0.094698	0.205618	-0.06686	-0.20168	0.260287	-0.22608	-0.2914	0.090465
Q8N9N7	LRRC57	0.874087	-0.18314	-0.34395	-0.10064	-0.26946	-0.50802	-0.34051	-0.73302	-0.03546	0.179092	0.525587	-0.32665	-0.19171	-0.27243	-0.30746	0.81603	0.016033	-0.11184	-0.58279
Q8N9N8	EIF1AD	0.332632	-0.17968	0.087368	-0.88812	-0.56441	0.378358	0.153951	-0.09807	0.343862	-0.23105	-0.91086	-0.55479	-0.04471	0.027951	-0.80699	-0.00469	-0.34194	-0.08708	-0.08218
Q8N9R8	SCAI	0.924719	-0.22453	-0.55146	-0.41403	-0.27744	-0.40592	-0.8266	0.526958	0.258343	-0.72194	0.550925	-0.40147	-0.34882	-0.43627	0.266281	-0.09528	-0.16811	-0.29124	-0.25488
Q8N9T8	KRI1	-0.47194	-0.11917	0.11029	-0.74339	-0.7452	-0.48106	0.158991	0.812374	-0.04652	0.190955	0.346031	-0.81484	-0.33615	-0.76751	-0.22629	1.088044	-0.3132	0.273341	-0.54234
Q8N9V3	WDSUB1	0.158197	-0.3598	0.238741	0.161038	0.329642	-0.31584	-0.24315	0.529691	-0.26943	0.215289	0.136715	0.092981	0.112627	0.014092	0.974562	0.374935	0.37589	0.150432	-0.38023
Q8NAF0	ZNF579	0.509886	-0.3658	-0.44654	-0.05017	-1.41428	-0.51235	0.016585	0.782589	-0.08654	-0.27548	1.344725	-1.05881	-0.14706	-1.25998	1.141798	-0.05513	-0.22367	0.016409	-0.13738
Q8NAN2	MIGA1	0.340926	0.549339	0.109374	0.353684	0.002971	0.03859	0.091729	-1.17923	0.013941	0.375598	0.36254	-0.199	-0.49785	0.172525	1.444028	0.253131	1.244002	1.507261	-0.47647
Q8NAP3	ZBTB38	0.160036	-0.10229	-0.12789	0.02718	0.363507	-0.49641	0.10346	0.231884	-0.08715	-0.38183	0.245718	-0.71161	-0.29143	-0.06581	0.890745	-0.40857	0.147935	0.159429	-0.01823
Q8NAT1	POMGNT2	0.484891	0.30263	-0.13204	-0.40506	-0.04727	-0.33246	0.03191	0.07877	-0.55061	-0.80944	1.137284	-0.2763	0.183822	-0.31488	0.256865	-0.08138	-0.12258	-0.74351	-0.28731
Q8NAV1	PRPF38A	0.946401	-0.17836	0.303708	0.06249	0.397314	0.197612	0.260367	0.392	-0.25958	0.286337	0.672459	-0.24019	-0.2366	-0.00053	0.158234	-0.04809	0.330572	0.177156	0.070257
Q8NB16	MLKL	-1.07706	1.034395	0.343417	-1.42146	0.690548	0.484651	0.27898	-1.19796	-0.08866	1.151252	-1.16378	0.868202	-0.10745	1.406096	-1.24084	-0.35705	0.129459	0.513896	-1.09044
Q8NB37	GATD1	0.144721	0.025158	-0.00873	-0.93	1.806275	-0.50854	0.090359	0.754905	0.309107	0.504821	-0.26794	0.850378	0.139051	0.866003	-0.00374	-0.03527	0.054015	0.525267	-0.26139
Q8NB49	ATP11C	0.235909	0.12695	0.9052	-0.88938	-0.12104	-0.22501	0.868548	-0.29531	1.037484	0.015548	-0.26445	0.262654	-0.10325	0.141939	-0.24063	-0.37089	0.209815	0.014223	-0.61828
Q8NB78	KDM1B	0.032293	-0.02938	0.030753	0.424743	0.483891	0.331793	-0.36603	0.253147	0.244549	-0.17059	0.257654	-0.16692	-0.08413	-0.14309	0.263426	0.763222	-0.35625	-0.55615	-0.30826
Q8NB90	SPATA5	0.088375	-0.24613	-0.25604	-0.25568	-0.21352	-0.07936	-0.19576	-0.41951	-0.14519	-0.32755	0.037299	0.031045	-0.30868	-0.17934	-0.1342	0.366865	-0.26742	-0.42412	-0.37579
Q8NBF2	NHLRC2	-0.54445	-0.23419	-0.50812	-0.37446	-0.27562	0.030226	-0.10411	-0.00602	0.722336	-0.02464	-0.10363	-0.23341	-0.10648	-0.28386	0.015346	0.225757	-0.21373	-0.5205	-0.43662
Q8NBF6	AVL9	1.238151	-0.40033	-0.32225	-0.71418	-1.10198	-0.08653	-0.18782	0.145886	-0.14121	0.005838	-0.42107	-0.46455	-0.02749	0.089488	-0.27416	0.078653	0.425397	-0.65252	-0.12582
Q8NBI6	XXYLT1	0.87991	0.018906	0.463456	0.022563	-0.35402	-0.51504	0.171229	0.184565	-0.01487	0.003501	-0.61188	-0.32859	0.101995	-0.12727	-0.01509	-0.61577	0.049658	-0.48013	0.526579
Q8NBJ4	GOLM1	-0.39217	0.542194	0.803444	-0.64043	0.900688	1.211796	0.595534	0.220501	0.145536	0.559393	-0.27635	-1.6428	0.135543	-1.54422	-0.72934	-0.2557	0.566215	1.462892	0.337762
Q8NBJ5	COLGALT	-0.87808	0.694239	1.326683	-0.17751	0.351495	-0.04912	-0.0891	0.510446	0.206146	0.879249	-0.69906	1.332464	0.56681	1.193647	-1.14067	0.854071	0.614429	0.327266	0.293198
Q8NBJ7	SUMF2	0.169684	-0.41065	0.944971	0.777719	0.98085	0.729902	-0.4012	0.593243	0.645793	0.804934	0.341206	0.223802	0.546896	0.403352	0.651724	0.244281	0.426937	0.591743	0.082745
Q8NBJ9	SIDT2	-1.09768	0.233709	0.270299	-0.1986	-0.13532	-0.01798	0.306583	-0.03422	0.040011	0.352748	-0.86814	1.140109	0.12282	1.216447	0.092144	-0.08121	-0.22306	-0.39376	0.391451
Q8NBK3	SUMF1	0.950177	-0.11382	0.076711	-0.67166	0.564744	-0.10171	-0.32691	-0.00683	0.420432	0.646495	-0.20972	0.941988	0.039908	0.606525	-0.40627	0.003547	0.287761	-0.12822	-0.24125
Q8NBL1	POGLUT1	-0.12443	0.321195	-0.09094	-0.41906	-0.05436	0.053223	0.347471	0.580065	-0.27057	0.938046	-0.12486	-0.38406	0.010531	-0.20458	0.02793	-0.61076	-0.11856	-0.39772	0.085367
Q8NBM4	UBAC2	-0.28307	-0.04123	-0.20942	-0.74468	-0.17388	0.407877	0.853988	0.202353	-0.00984	0.037862	0.029649	-0.55423	-0.37544	-0.88646	0.326668	-0.2921	0.895398	0.40759	0.848505
Q8NBM8	PCYOX1L	-0.08434	-0.66958	-0.02456	-0.032	0.652906	1.123269	-0.15768	-0.17122	0.550144	0.139155	0.712455	0.123814	-0.21511	0.617139	-0.90191	-0.06226	-0.45184	-0.57695	0.076364
Q8NBN3	TMEM87A	-0.34169	0.129747	-0.17701	-0.07809	-0.78026	-0.44643	0.331659	-0.20356	-0.23873	-0.6662	-0.18429	-0.3611	0.253859	-0.96303	1.646142	1.22217	1.125206	0.725776	0.561241
Q8NBN7	RDH13	0.039852	-0.10317	-0.84983	0.766062	-1.12859	-0.80249	-0.46396	0.26163	-0.46007	-0.63749	1.377738	-0.67964	0.054838	-0.60704	2.408595	0.953465	-0.46261	-1.0498	-1.10331
Q8NBP0	TTC13	-0.41022	0.429833	0.262886	-0.03908	-0.01974	-0.10838	0.221439	0.314078	0.620477	0.010736	-0.32606	-0.10885	0.241757	0.015839	0.520042	0.00175	0.103238	0.117381	0.431243
Q8NBQ5	HSD17B11	-0.90297	0.578398	1.198769	-1.07052	0.639297	0.085534	0.217085	-0.12928	0.774938	1.755435	-0.35011	0.552333	0.091926	1.342433	-0.59744	0.086226	-0.12039	-0.47311	-0.40785
Q8NBS9	TXNDC5	-0.13628	-0.34087	0.181148	0.200209	0.381852	-0.39804	1.059498	-0.54324	0.416464	-0.01043	-0.564	-1.00647	0.822788	-1.33759	-0.67489	-4.6E-06	-1.08849	1.103271	0.664066
Q8NBU5	ATAD1	0.492148	0.103842	0.177108	0.315998	-0.40262	-0.14235	-0.32268	-0.19835	-1.23351	0.066525	0.26175	-0.25582	-0.07102	0.26242	1.092579	0.543091	0.045966	-0.35492	-0.07319

Q8NBX0	SCCPDH	0.130588	-0.07832	-0.56666	0.121739	-0.1036	-0.06336	-0.22131	0.051694	0.029894	0.213717	0.025794	0.748572	0.161169	0.42092	0.846148	0.076284	0.160236	-0.54819	-0.36431
Q8NBZ7	UXS1	-0.3048	0.297527	0.100281	0.244582	-0.15922	-0.58736	-0.08073	0.066521	-0.35174	0.464134	0.192588	-0.15056	0.074747	-0.01054	-0.19038	-0.41441	1.143002	1.007979	-0.33093
Q8NC42	RNF149	0.07787	-0.11818	0.205254	-0.60595	-0.03021	-0.47876	0.296629	0.050785	0.031082	0.065124	-0.52894	0.360548	0.087072	0.328294	-1.17867	-0.31217	1.039906	-0.22	-0.42536
Q8NC44	RETREG2	-0.67445	0.477256	-0.08506	-0.31667	-0.30607	-0.11341	0.227458	-0.24109	-0.08594	0.146873	-0.26942	0.124307	-1.08775	0.556106	-0.22488	0.507832	-0.18273	-1.04966	0.013877
Q8NC51	SERBP1	1.302523	-0.55156	-0.44316	0.04314	-1.12461	-0.00107	-1.21496	0.194601	0.061538	-0.57828	0.487181	-1.05871	0.036024	-0.91325	0.006439	0.049736	-0.23729	-0.44883	-0.46652
Q8NC56	LEMD2	0.122427	0.062029	-0.26731	-0.10399	-0.54627	0.042934	0.217765	0.074534	-0.15722	-1.08548	0.017108	-0.54292	-0.32789	-0.75305	0.039727	-0.21861	0.18784	-0.06982	-0.05081
Q8NC60	NOA1	-0.40066	0.042657	0.092873	0.392307	-0.07362	0.440768	-0.45241	-0.25809	-0.09363	-0.42554	0.390043	-0.10798	-0.16184	0.206084	0.025285	-0.44984	0.388032	-0.27595	-0.14774
Q8NC96	NECAP1	0.254733	0.004522	0.423375	-0.28821	-0.16791	-0.62953	-0.1459	-0.40986	0.669489	-0.09329	-0.55939	0.028969	-0.03542	-0.02417	0.078947	0.560829	0.260795	-0.27617	-0.30885
Q8NCA5	FAM98A	-0.33403	-0.04203	-0.20566	-0.11935	-0.16244	-0.33983	0.977981	-0.13431	0.803497	-0.1908	-0.15577	-0.42257	0.186774	-0.49784	-0.03112	0.337364	-0.49094	-0.2473	0.418437
Q8NCC3	PLA2G15	0.009345	-0.16666	0.407212	-0.05842	0.954773	-0.47401	0.066796	-0.20011	0.176003	0.298368	-0.72684	-0.36324	-0.24055	0.331871	-0.3122	-0.09422	-0.36861	0.927225	-0.94686
Q8NCE2	MTMR14	-0.3264	0.465767	0.024955	-0.62088	0.428411	0.037439	-0.77182	-0.23402	-0.00639	0.742478	-0.46153	0.501461	0.193774	1.118116	-0.65019	0.160325	0.344873	-0.01967	-0.32005
Q8NCF5	NFATC2IF	1.237973	-0.27654	-0.16184	-0.01564	-0.04963	1.263679	-0.10246	0.321517	-0.32954	-0.10201	-0.38708	-0.39815	-0.20411	0.031066	0.095063	-0.05284	-0.34483	0.224116	-0.19896
Q8NCG7	DAGLB	-0.30183	0.68593	0.593301	-0.3272	0.247786	0.150164	-0.08053	-0.21573	-0.1903	1.041655	-0.30732	1.041559	0.006424	1.049588	-0.26591	-0.10649	1.214351	0.176228	-0.23155
Q8NCH0	CHST14	-0.30929	0.743231	-0.51456	-0.53313	-0.82966	-0.4785	-0.71759	-0.44343	0.581309	-0.30362	-0.52253	-0.90119	0.11318	-0.04835	-0.42499	-0.0145	0.219377	0.778996	0.694869
Q8NCL4	GALNT6	0.860871	-0.04716	0.102045	-0.88187	0.241824	0.352266	-0.22757	0.833769	0.010352	0.638564	1.04435	-0.06155	-0.85587	-0.07628	0.230698	-0.80341	0.183313	-0.09214	-0.06344
Q8NCM8	DYNC2H1	-1.25553	-0.14827	0.273971	0.339017	-1.20663	-1.33981	0.029451	-0.29258	-0.18038	-0.81928	1.432299	-0.3026	-0.16467	-0.00586	-0.31742	-0.85519	-0.51048	-0.52777	0.305804
Q8NCN4	RNF169	1.057889	-0.49115	0.002907	-0.31512	0.94861	1.008104	-0.60486	0.227414	-0.40994	-0.54649	0.451764	-1.17054	-0.08252	-0.8306	-0.25262	-0.10508	-0.43739	0.00135	-0.55974
Q8NCN5	PDPR	-0.24671	-0.72014	0.163975	0.543372	0.106017	-0.14522	-0.5816	-0.07345	-0.26875	0.417459	0.574364	0.045486	0.216104	-0.19414	1.607209	1.680222	-0.61944	-0.16299	0.009379
Q8NCW5	NAXE	0.097142	-1.0365	-1.03004	-0.16809	-0.50898	-0.23141	-0.51317	1.05141	-0.16569	-0.26539	-0.37224	-1.32877	-0.29239	-0.57507	1.61282	0.452399	-0.71012	-0.71303	-0.66899
Q8ND04	SMG8	-0.16637	0.176278	0.282001	-0.03856	-0.10686	-0.11978	0.684955	0.077627	0.695599	-0.02021	0.159563	0.036253	0.089822	0.052129	-0.46654	-0.85974	0.16127	-0.25947	0.381066
Q8ND24	RNF214	-0.16431	-0.29038	0.021491	-0.08212	-0.40492	-0.33537	-0.83992	0.159388	0.124333	0.051835	0.220632	-0.58151	0.744565	-0.01204	-0.13654	0.199896	-0.3016	-0.04517	0.10478
Q8ND30	PPFIBP2	-0.31299	-0.18896	-0.15686	-0.64227	0.060249	0.412934	-0.13524	0.167283	-0.08104	0.228935	0.439653	-0.08452	-0.96944	0.183601	0.4838	-0.55303	-0.23099	-0.77134	-0.56
Q8ND56	LSM14A	0.281434	-0.33826	-0.10733	-0.03216	-0.21821	-0.28519	0.078588	1.181999	0.255751	-0.07632	0.796931	-0.04303	0.401384	-0.19806	0.485464	0.107573	0.303182	0.278253	0.052024
Q8ND71	GIMAP8	-1.08021	1.498558	2.193198	-0.84824	0.802771	0.610001	0.042326	-1.52141	0.022233	0.878556	-0.96745	1.434493	-0.42455	1.13404	-0.17961	-0.21139	0.155577	0.726027	-1.26155
Q8ND76	CCNY	-0.12456	-0.34057	0.1366	-0.01621	0.093603	-0.21643	-0.29607	-0.1788	-0.07704	-0.11241	0.406987	-0.38222	-0.04031	0.225895	0.439535	-0.43393	0.187966	0.326287	-0.10459
Q8ND82	ZNF280C	1.040769	-0.27339	-0.35164	-0.41173	-0.62777	0.141336	-0.2491	-1.32756	-0.32978	0.444043	0.706473	-0.12602	0.04503	-0.81448	1.085562	0.352066	0.701445	0.471215	-0.55062
Q8ND83	SLAIN1	0.260556	0.010099	-0.48877	-0.66928	-0.29175	0.212677	0.305138	-0.24748	0.040847	-0.37702	1.703429	-1.26328	-0.12549	-0.57842	-0.00667	-0.09434	-0.52965	-1.00588	-0.08139
Q8ND90	PNMA1	-0.34511	-0.02185	-0.90871	-0.27634	-0.32105	-0.38682	-0.30289	1.099694	0.236713	0.259584	-0.57643	-0.01504	-0.03728	-0.27617	0.518412	-0.60161	-0.50594	-1.45213	1.821011
Q8NDA8	MROH1	0.950075	0.02896	-0.26917	-0.78114	-0.42844	-0.34995	0.237826	0.619648	0.934802	0.309352	-0.16246	0.064279	0.443201	0.176358	-0.86338	0.510031	-0.57145	-0.518	-0.76729
Q8NDF8	PAPD5	-0.12186	-0.16171	-0.32387	0.07384	-0.41703	-0.50828	-0.17044	0.184092	0.499226	-0.0016	0.602393	0.128407	-0.24833	-0.26461	-0.0897	0.556959	0.085679	0.71034	-0.61288
Q8NDH3	NPEPL1	-0.49926	-1.01539	-0.24287	1.039294	1.009252	0.676406	-0.67724	0.554149	0.060017	-0.76759	0.641328	-0.33703	-0.48634	-0.18805	0.313003	0.61778	-0.45873	0.126645	-0.10433
Q8NDI1	EHBP1	0.230629	-0.33614	-0.38733	-0.76113	-0.68136	-0.72371	-0.92049	-0.07113	0.300322	0.501184	-0.25566	-0.84399	0.08186	-0.27273	0.860818	0.625932	-0.50021	-0.17744	0.207582
Q8NDT2	RBM15B	0.889817	0.369051	-0.30029	-0.21981	-0.51316	-0.14072	-0.18545	0.229679	-0.48499	-0.55412	0.252223	-0.43811	0.045186	-0.49124	-0.19394	-0.80714	-0.01386	0.369867	0.153702
Q8NDX5	PHC3	0.144039	-0.10358	-0.19285	0.261246	0.109392	-0.21853	-0.49131	0.668861	-0.17222	-0.36335	0.480697	-0.78618	-0.10845	0.361422	0.659815	0.138826	0.204006	0.440131	-0.27452
Q8NDZ4	C3orf58	0.584628	0.001399	0.195021	-0.39399	0.797308	0.770435	-0.20724	-0.36268	0.764895	0.627416	0.137187	-0.12267	0.016989	-0.02786	1.869473	-0.80502	0.246351	-0.10279	0.144348
Q8NE01	CNNM3	1.167752	-0.47705	-0.36196	0.013984	-0.02533	0.108747	0.019288	0.643025	-0.40824	-0.20945	-0.38494	-0.02842	-0.31757	-0.13349	0.04084	-0.30081	-0.35123	-0.56772	-1.02835
Q8NE62	CHDH	0.608885	-0.94995	-0.75314	-1.38485	-1.08404	-1.28094	0.274484	-0.07724	-0.21621	-1.39439	1.863166	-0.20977	0.331741	-0.91445	-0.85869	-1.70655	0.006051	0.419407	-0.98407
Q8NE71	ABCF1	0.618083	-0.13592	0.025232	0.043263	-0.12483	0.150866	-0.72636	-0.6251	-0.19291	-0.14018	-0.06915	-0.09398	0.127396	-0.15488	0.269471	0.182542	0.429274	0.616311	0.153493
Q8NE86	MCU	0.455016	0.207507	-0.2515	0.082768	-0.06812	0.173268	0.239479	-0.03085	-0.13218	-0.04742	-0.21666	-0.27192	-0.6212	0.410894	-0.56377	0.939701	1.759313	0.651705	0.041078
Q8NEB9	PIK3C3	-0.46519	0.2454	0.799378	-0.11322	0.104315	0.177053	-0.25225	-0.51674	0.886411	0.564043	-0.39455	0.37347	-0.19455	0.239712	0.008293	0.42996	-0.08884	-0.33943	-0.14033
Q8NEC7	GSTCD	-0.20462	-0.12292	0.272056	-0.59187	0.151143	-0.04576	0.046569	0.187052	0.040847	-0.59853	0.725878	-0.05824	0.292312	-0.30481	-0.08595	-1.48702	-0.39305	0.670461	-0.2903
Q8NEF9	SRFBP1	-0.1708	-0.37464	-0.68062	0.453909	-1.1851	-0.12729	-0.46288	-0.67192	-0.06819	-0.0906	1.373527	-0.7966	-0.4029	-0.68352	0.242769	0.035365	-0.29482	-0.49254	-0.68653
Q8NEJ9	NGDN	0.504439	-0.21457	-0.06859	0.459482	-0.44295	-0.15592	-0.61198	0.192537	-0.25893	-0.21992	-0.1338	-0.45666	-0.06879	-0.41127	0.23184	-0.30621	-0.01464	-0.6824	-0.26636
Q8NEN9	PDZD8	-0.33639	-0.06372	-0.06207	-0.14021	-0.19771	-0.71023	-0.3418	-0.48699	0.361682	0.188908	0.267101	0.086716	0.047588	0.609247	0.544529	0.111716	0.595469	0.523099	-0.25069
Q8NEU8	APPL2	-0.81582	0.138627	0.361591	-0.23422	0.280227	-0.12318	-0.22622	-0.308	-0.06977	0.145148	-0.01063	0.161505	0.151867	0.227809	0.043259	-0.0162	0.207564	0.641295	0.527787
Q8NEW0	SLC30A7	-0.05921	0.897125	0.446625	-0.18472	0.462646	0.096171	0.106722	0.01135	0.065113	-0.0501	-0.44846	0.605619	0.382474	0.521235	-0.14876	0.3667	0.379509	-0.42497	0.500837
Q8NEY1	NAV1	-0.4433	0.269025	-0.31954	-0.78758	-0.26168	-0.46464	0.157314	0.222484	0.171707	0.539258	-0.67624	-0.4643	0.546467	0.390673	-0.20991	-0.66334	-0.72705	0.745131	2.213059
Q8NEY8	PPHLN1	0.646689	-0.77752	0.084092	0.140205	0.454502	0.863394	-0.56254	0.670833	-0.1909	0.045066	0.713622	-0.64615	-1.06649	-0.67235	0.081364	-0.51167	-0.06998	0.520667	-0.09356
Q8NEZ2	VPS37A	-0.20932	0.824353	0.894197	-0.62518	-0.025	-0.68431	0.51937	-0.21233	-0.52658	0.195929	-0.12223	0.408047	0.2191	0.446062	-0.41864	-0.02184	0.857884	0.287333	0.31061
Q8NEZ3	WDR19	-0.09061	0.033196	-0.44265	-0.46955	-0.65237	-0.59691	-0.02075	-0.34362	0.040847	-0.99293	1.337012	-0.63195	-0.2877	-0.30779	-0.44144	-0.10147	-0.70302	-1.08756	0.066857
Q8NEZ4	KMT2C	-0.1028	-0.20501	-0.10134	0.295239	0.145773	0.108336	-0.12346	0.213467	-0.08158	0.060898	0.149865	-0.10994	0.010562	-0.20265	0.383325	-0.02356	-0.03086	0.623531	-0.718
Q8NEZ5	FBXO22	-0.81081	0.077543	-0.79602	0.593472	0.574988	0.540293	-0.31376	0.070953	0.079476	-0.09802	0.615135	-0.46808	0.320259	-0.42458	-0.04623	0.041139	-0.46288	-0.10716	-0.45505
Q8NF37	LPCAT1	0.448514	0.536948	0.22535	-0.21664	-0.25677	-0.10967	0.514429	-0.20283	0.335427	1.598411	-0.6307	1.015436	0.240299	0.597549	-0.36816	-1.19125	0.826241	0.163683	-0.0135
Q8NF50	DOCK8	-1.91125	1.459273	1.159508	-0.8598	0.445658	0.076696	0.466053	-1.3529	-0.32726	1.091728	-1.12443	0.937241	-0.06012	1.498274	-0.30672	-0.80837	0.466856	0.444063	-1.19556
Q8NF91	SYNE1	-0.73598	0.195266	-0.09551	-1.02723	-0.31241	-0.52776	0.048344	-1.07031	-0.02552	-0.33105	0.151941	-0.68287	-0.02766	0.344744	-0.892	-0.54653	-0.53578	-0.00156	0.466429
Q8NFA0	USP32	-0.42487	0.318598	0.029586	-0.01642	-0.09081	-0.12916	0.017697	-0.04131	-0.29606	0.1401	0.210379	0.111791	0.217093	0.662963	-0.10386	1.166569	0.162099	-0.51797	-0.30422
Q8NFC6	BOD1L1	1.659783	-0.90713	0.578885	-0.23773	-0.71217	0.160059	1.127064	0.023227	-0.13044	-0.26151	0.174912	-1.48416	-0.73948	-0.89409	0.095491	-0.27696	-1.15782	-0.26593	0.201683
Q8NFD5	ARID1B	-0.25983	-0.59795	-0.35358	-0.0105	-0.32151	0.069121	-0.22698	-0.22774	0.754868	-0.60683	0.364327	-0.56665	-0.05747	-0.47839	0.884732	-0.59852	-0.53224	0.136108	0.065171
Q8NFF5	FLAD1	0.091999	-0.10767	-0.41246	0.188511	-0.74726	-0.77729	0.019885	0.520425	-0.35631	-0.0864	0.108454	0.131592	0.370404	-0.22356	1.407399	-0.4343	-0.18432	-0.94644	-0.93022
Q8NFG4	FLCN	-0.3754	0.263549	-0.00031	-0.99142	-0.06434	-0.92189	0.166315	0.016346	0.282677	0.273214	0.016557	0.420312	-0.00531	0.282209	-0.99569	-0.10988	0.283535	0.068051	0.048467
Q8NFH3	NUP43	0.172443	0.240901	0.658405	0.135452	0.318857	-0.13527	-0.3926	0.302961	0.270875	-0.07218	-0.00676	0.177553	-0.20853	-0.43971	0.485474	0.003755	0.297813	0.013636	0.223832
Q8NFH4	NUP37	0.658697	-0.48697	0.242751	0.344891	0.936998	0.221118	-0.48629	0.556408	0.109806	0.146634	0.229232	0.209357	0.330181	0.561131	0.97096	0.295589	0.489515	0.423076	0.425159
Q8NFH5	NUP35	0.041304	0.143315	-0.26296	-0.2815	-0.26297	-0.21031	0.480377	0.135212	-0.5251	-0.16125	0.198301	-0.17603	0.267799	-0.09813	0.952596	-0.26142	-0.04618	0.023744	0.156982
Q8NFH8	REPS2	-0.30871	0.139052	0.263517	-0.55734	0.09661	0.378695	0.191279	-0.08738	0.173281	0.562048	0.219578	-0.27598	-0.11688	0.407427	1.04327	-0.10772	-0.14084	-0.02408	0.136816
Q8NFI3	ENGASE	-0.31947	0.201181	0.111046	-0.21313	0.054154	0.086376	-0.58606	-0.74361	-0.05642	-0.18381	0.255439	-0.19875	-0.59913	-0.04398	-0.08549	0.244871	-0.20511	0.647167	0.036162
Q8NFJ5	GPRC5A	-1.73492	-0.05935	-0.01729	1.358815	-1.41854	-0.78329	0.057262	0.96551	-0.19165	0.00497	-1.49754	1.359128	0.129409	0.421837	-0.3568	1.265512	1.318537	-2.05879	-1.67429
Q8NFJ9	BBS1	-0.65895	-0.06614	-0.08266	-0.72317	-0.23321	-0.06635	-0.27349	-0.25326	0.400775	-0.44881	0.620046	-0.90262	0.146516	-1.08766	-1.1413	-0.86482	-0.31674	-0.52823	0.367292
Q8NFP9	NBEA	-0.42177	0.315026	-0.04609	-0.42034	-0.30656	-0.2721	-0.20762	-1.93866	0.077053	-0.05333	1.179218	-0.24384	0.291722	-0.46874	0.03021	-0.73204	0.401498	1.703002	0.497053
Q8NFQ8	TOR1AIP2	0.021302	0.697218	1.090235	0.365401	-0.57831	0.217385	0.92603	0.408091	-0.48152	0.257536	0.074675	-1.50376	0.165792	-0.77931	0.641866	-0.01005	-0.49328	-0.09269	0.484903
Q8NFT2	STEAP2	4.71E-05	0.135557	0.130089	-0.54947	-0.14871	-0.41203	0.840114	-1.14382	0.233147	0.152354	-0.38237	0.556183	0.362362	-0.17892	-0.35668	2.408759	-1.16122	-0.58466	0.263893
Q8NFU3	TSTD1	0.291646	-1.33348	-2.30401	-0.90245	-1.05725	-0.47025	-0.78646	0.186979	-0.67307	-0.18915	-0.02721	-1.88558	-0.83949	-0.67453	0.626823	0.401841	-1.3466	-1.77819	-1.44057
Q8NFV4	ABHD11	0.240169	-0.15422	-1.06587	0.990906	-0.73336	-0.9267	-1.30092	-0.54654	-0.49013	-0.22119	0.34869	-0.36241	-0.63429	-0.51485	2.362423	0.811054	-0.65976	-0.9349	-1.59967
Q8NFW8	CMAS	0.864475	-0.45674	2.37543	-0.8516	-1.70982	-1.50285	-1.11867	-0.05409	-0.55576	0.636951	0.338594	-0.65124	-1.06098	-0.20162	0.397663	0.332089	-0.56251	-0.56808	-0.21393
Q8NFZ5	TNIP2	-0.63561	0.523374	1.129382	-0.22323	-0.20439	-0.18238	0.294988	-1.0612	-0.01917	0.036759	-0.26403	-0.26943	-0.34348	0.994727	-0.66985	-0.81867	0.012018	-0.41105	9.62E-05
Q8NFZ8	CADM4	0.499008	-0.19724	-0.1839	-0.251	-0.93959	-1.21966	0.433503	1.122041	0.195403	0.006815	0.44793	-0.76516	-0.11723	-0.22101	0.81993	-0.62033	-0.62916	-0.52049	-0.06606
Q8NG11	TSPAN14	-0.27329	1.096545	-0.37745	-0.515	-0.69848	-0.74278	-0.89048	-0.29155	0.040847	-0.27201	1.20162	0.728507	-1.04873	0.697803	0.363885	0.059988	0.333856	0.409533	-0.70043
Q8NHG7	SVIP	1.212902	-0.32128	-0.39365	-0.01846	-0.49154	-0.02008	-0.06948	-0.36639	-0.64237	-0.02971	-1.02908	-1.13891	-0.4504	-0.1229	-0.23075	0.585612	-0.02041	0.208618	-1.05998
Q8NHH9	ATL2	0.02911	0.004393	0.430498	0.051058	-0.68323	-0.57237	0.271457	0.85121	0.168685	-0.2621	-0.49446	-0.04574	-0.27019	-0.19758	-0.20983	0.006929	-0.25335	-0.33182	-0.06631
Q8NHJ6	LILRB4	-1.61617	1.166789	0.80136	-0.87581	-0.1613	-0.2128	0.057766	-1.2511	-0.23061	0.723879	-1.45742	0.54822	-0.14113	1.458931	-0.59585	-0.83909	0.392449	-0.2589	-1.52468
Q8NHL6	LILRB1	-2.16547	2.164294	1.144601	-1.06937	0.418058	0.049505	0.54293	-1.13852	1.379802	2.287928	-0.96362	1.879128	0.843381	1.849157	-0.16629	-0.62465	-0.0653	0.781296	-1.31675
Q8NHM5	KDM2B	0.083251	-0.08236	0.060456	0.188631	0.132494	-0.12696	-0.11256	0.727144	-0.15071	-0.01925	-0.55947	0.107605	0.141542	0.157166	0.553617	0.118153	0.158325	0.143644	-0.21191
Q8NHP6	MOSPD2	-0.41233	0.982769	0.430519	0.019158	0.140563	0.166678	0.174505	-1.10728	-0.16727	-0.28146	-0.22846	0.749662	0.078292	0.942204	-0.28148	-0.03504	0.337024	-0.06137	-0.36622
Q8NHP8	PLBD2	0.165504	-0.13473	-0.22058	-0.11991	0.821999	-0.02974	-0.10079	0.601391	0.19407	0.249895	-0.75922	0.076269	0.07771	0.186277	0.258715	-0.55178	-0.17557	0.313662	-0.17458
Q8NHQ8	RASSF8	0.206583	0.099497	-0.28675	-0.42001	-0.33895	-0.43941	0.06897	-1.08648	0.060039	-0.80013	-0.1736	-0.04767	-0.00721	0.034438	-0.24984	-0.26727	-0.20114	0.318618	0.112994
Q8NHQ9	DDX55	1.068704	-0.03891	-0.03665	-0.10411	0.153761	-0.1198	-0.21312	0.000852	0.442077	-0.20359	-0.23563	0.054464	-0.30923	0.441238	-0.64558	-0.80959	0.647983	0.320866	-0.13749
Q8NHU6	TDRD7	0.13601	0.261684	0.358061	-0.44983	0.998087	1.600255	-1.23247	1.049482	-0.24089	0.04046	-0.09193	0.305263	0.055022	0.3017	-0.12237	0.029061	-0.07357	-0.36847	0.293214
Q8NHV1	GIMAP7	-1.90809	1.210734	1.597905	-0.71052	1.190302	0.271422	0.170841	-2.36716	0.140476	1.268868	-1.29527	1.320181	-0.46686	1.496703	-0.47335	-0.60514	0.248831	1.108921	-1.48642
Q8NHV4	NEDD1	0.074011	-0.03888	-0.04994	-0.22877	-0.45796	0.272824	-0.47035	-0.65937	-0.13004	-0.62747	0.33985	-0.29016	0.226229	-0.27918	-0.11682	0.08995	-0.27951	-0.11234	0.276642
Q8NHY2	RFWD2	0.062521	-0.2459	-0.1428	-0.25354	0.029926	-0.64653	-0.25722	0.029494	0.056304	-0.08164	0.6356	-0.73637	-0.11103	-0.43588	0.444655	0.108169	-0.60217	-0.06165	0.45332
Q8NI08	NCOA7	0.41913	0.362084	0.895165	-0.37873	-0.1342	-0.58244	-0.33356	-0.59741	0.180024	0.532402	-1.31083	0.076246	0.280262	-0.16508	-0.11246	0.661336	0.079342	0.017818	0.286671
Q8NI22	MCFD2	-0.33656	0.242708	0.046602	0.637345	0.160828	-0.10072	-0.07419	0.433975	-0.18126	0.236252	0.169919	-0.52684	-0.26164	-1.28843	-0.17787	0.557034	-0.36542	-0.1372	0.32236
Q8NI27	THOC2	0.435936	-0.37051	-0.09049	0.298604	0.686428	0.830689	-0.47084	0.333456	-0.32508	0.50028	0.666661	-0.24368	-0.30125	-0.34113	0.054668	-0.10937	0.471895	0.519166	-0.31082
Q8NI35	PATJ	1.088769	-0.06699	0.397147	0.16884	-0.3517	-0.40838	1.229807	0.591441	-0.14727	-0.13989	0.323794	-0.12635	-0.20553	-0.31675	0.574467	0.321205	0.36548	0.423735	-0.7581
Q8NI36	WDR36	0.561548	0.449074	0.263272	0.324041	-0.26553	-0.09428	0.897933	0.076034	0.35362	-0.13664	0.779649	-0.26926	-0.23	-0.71137	0.101712	0.08122	-0.05614	-0.1309	-0.2439
Q8NI60	COQ8A	-0.1293	-0.46348	-0.05837	-0.02597	-0.9629	-1.18297	-0.49761	-1.43253	0.216857	-0.34926	0.042399	-0.53716	-0.01462	-0.33593	1.542774	-0.07553	-0.6385	-0.98567	-1.43401
Q8TAA9	VANGL1	0.767689	0.265544	-0.04783	0.614795	-0.29493	-0.08766	0.039348	0.518742	-0.79935	-1.06094	-0.06201	-0.77331	0.051834	-0.65707	0.353723	-0.51872	-0.03761	0.266953	-1.21417
Q8TAB3	PCDH19	1.44674	-0.26065	0.164197	-0.36825	0.719904	1.709084	-0.89723	0.170204	0.003632	-0.29867	0.195815	0.245442	0.471841	-0.39844	-0.40764	-0.05265	-0.22546	-0.99886	0.926936
Q8TAD4	SLC30A5	-0.11815	0.140937	0.144292	-0.10229	0.280519	-0.22481	0.535255	-0.55278	0.055229	0.407518	-0.24653	0.394958	0.013187	-0.05099	1.506155	-0.06428	0.514164	-0.10743	0.091299
Q8TAD8	SNIP1	0.367832	-0.3672	-0.05776	0.278465	0.109096	0.344653	-0.41103	0.289404	-0.2185	-0.57277	0.112503	0.106681	0.093232	-0.27691	0.284228	-0.10601	0.672295	0.687849	-0.12809
Q8TAE8	GADD45G	-0.40449	-0.00027	0.256494	0.695537	0.198953	0.192524	0.156497	-0.02899	-0.54596	-0.06392	0.597002	-0.15379	0.251094	-0.29451	0.672492	-0.15821	-0.25642	0.156565	-0.67446
Q8TAF3	WDR48	0.667746	-0.379	0.482388	-0.21604	-0.32646	-0.47837	-0.53273	-0.0238	0.140026	-0.1977	0.149048	-0.50118	-0.08517	0.326002	0.1225	0.187356	-0.49569	0.04607	0.412861
Q8TAG9	EXOC6	-0.24137	0.261217	-0.25152	0.211571	0.06948	0.084406	0.58883	0.024114	0.171946	0.161279	0.476497	0.112971	-0.81731	0.70408	0.365628	0.063765	-0.14118	-0.47284	-0.75151
Q8TAQ2	SMARCC2	0.132478	0.06833	0.094826	0.186506	0.468634	0.786668	0.131729	0.793783	-0.08506	0.037621	0.157772	-0.63819	-0.41586	-0.46078	0.58245	-0.59167	-0.44501	0.013566	-0.19186
Q8TAT6	NPLOC4	0.390688	-0.14811	0.373577	0.021715	0.007314	-0.00937	-0.45293	0.191317	-0.00778	0.068179	-0.11447	-0.19577	-0.47628	-0.08044	-0.73805	-0.26541	0.15494	0.204383	0.005849
Q8TB03	CXorf38	0.220399	0.48299	0.321393	0.108453	-0.22496	0.062412	0.165984	-0.20185	-0.38563	0.095679	0.189841	0.546047	0.175644	1.007945	-0.40485	-0.13288	0.529227	0.694562	-0.51537
Q8TB22	SPATA20	-0.40375	-0.50979	-0.1857	1.105592	-1.50969	-1.10746	-1.24077	-1.7487	-0.03459	0.416821	0.834588	-0.52858	-0.54984	-0.31367	0.212754	1.316949	-1.31765	-0.73835	0.098352
Q8TB24	RIN3	-1.42634	1.069778	0.918713	0.038081	0.510287	-0.06123	0.427582	-1.16772	-0.00566	0.053854	-0.53557	1.323729	0.101048	1.615242	0.076598	0.105031	0.016897	-0.34467	-0.80354
Q8TB36	GDAP1	0.8173	0.166637	0.189587	-0.10939	-0.39483	-1.33526	-0.40943	-0.90876	0.078735	-0.03394	0.599423	0.936002	1.670842	-0.11099	0.32404	-0.10503	-0.14548	-0.3942	1.155358
Q8TB37	NUBPL	0.318717	-0.50094	-1.2779	0.933019	-0.5284	-0.77804	-0.29865	-1.17214	-0.19066	-0.55389	1.083877	-0.5118	-0.50511	-1.39447	0.395103	0.94907	-1.15154	-0.37012	-1.42446
Q8TB45	DEPTOR	0.827983	0.062682	-0.22076	0.536739	0.673006	0.699732	-0.30138	-1.01621	-0.37185	0.164382	0.622201	-0.74577	-0.78569	0.215957	0.154723	-0.02867	0.031996	-0.41629	-0.57751
Q8TB61	SLC35B2	-0.29367	0.627619	0.378697	-0.19037	0.061632	0.346329	0.359303	-0.1817	0.334304	-0.30409	-0.59134	-0.17705	-0.43137	-0.31904	0.370601	0.281916	0.330982	0.464484	0.202572
Q8TB72	PUM2	0.02173	0.518835	0.025717	-0.29494	-0.49139	-0.19698	0.120655	0.415835	0.202331	0.061576	-0.38065	-0.19843	-0.12458	0.336037	0.369809	0.293163	-0.03298	-0.81219	0.101821
Q8TB96	ITFG1	-0.34809	-0.02199	-0.03344	0.698234	-0.03289	0.068752	0.053364	0.510165	-0.21289	-0.27228	0.092557	0.1804	-0.079	-0.0585	0.77569	-0.47096	0.479282	0.601323	0.41674
Q8TBA6	GOLGA5	-0.32841	0.852307	0.217173	0.074869	0.482836	0.742472	0.929571	-0.166	0.290721	0.1096	-0.32289	-0.39061	0.714695	-0.43362	0.258663	0.121597	0.240195	0.720124	0.605725
Q8TBC4	UBA3	-0.03843	-0.35729	-0.0032	0.614704	-0.29758	-0.26273	-0.89094	0.276623	-0.63944	-0.4223	-0.20746	-0.59179	-0.07836	-0.2036	0.254219	-0.11817	-0.08847	-0.26668	0.136761
Q8TBC5	ZSCAN18	-0.03807	-0.32681	-0.35254	-0.32423	-0.40881	-0.42112	-0.47528	0.470979	-0.06175	-0.49437	0.372099	-1.56957	0.11683	-0.65639	-0.88548	0.278801	-0.86279	-0.71531	0.378529
Q8TBM8	DNAJB14	-1.12158	0.302736	0.046342	-0.43311	0.064498	-0.05817	0.311319	-0.48545	0.177974	0.236104	0.030604	-0.79898	-0.14456	-0.16686	0.046377	-0.06645	0.099648	-0.01427	-0.06343
Q8TBN0	RAB3IL1	-1.01132	0.004648	1.60737	-0.67688	0.042139	-0.46163	-0.43432	-0.39873	-0.11526	0.324712	-0.87651	0.466037	0.30002	0.637365	-0.30881	0.098675	0.020926	0.507015	0.326795
Q8TBX8	PIP4K2C	-0.26001	-0.0706	-0.51036	0.039817	-0.60842	-0.37462	-0.97124	0.946103	0.730506	0.578361	-0.31054	-0.00788	-0.18465	0.379306	0.840287	-0.75283	0.436192	-0.59146	0.186116
Q8TC05	MDM1	-0.58644	-0.34029	-0.91956	-0.06801	-0.52893	0.019792	-0.7285	-0.94108	-0.15569	-0.45311	2.136961	-0.90175	-0.55227	-0.15225	-0.40804	-0.1752	-1.40567	-0.40035	-0.63495
Q8TC07	TBC1D15	0.309118	0.45008	0.382707	0.177992	0.386455	0.403563	0.478692	-0.07013	-0.02972	-0.09705	-0.15916	0.250384	-0.19079	0.31728	0.320166	0.299939	0.278621	-0.12188	-0.08064
Q8TC12	RDH11	0.543178	0.315879	0.190058	0.124159	-0.18556	-0.40403	0.211581	0.690596	0.236317	-0.1285	0.235461	0.708123	0.248489	0.700305	-0.1586	-0.59783	0.665289	-0.43128	-0.25324
Q8TC71	SPATA18	-1.78011	-0.46441	-0.28035	-2.95727	-2.30806	-1.77705	0.01103	-2.12947	-0.16271	-2.14518	3.652145	-2.17024	-0.92737	-0.74251	-1.18769	-0.6601	-0.88694	-1.85168	-1.14346

Q8TCB0	IFI44	3.042815	0.767509	1.797708	-0.51236	-0.59751	-0.52014	-0.06964	-0.93592	0.040847	0.061507	-1.06372	1.264654	0.109651	-0.044	-1.39776	-0.27686	-0.01033	-0.23055	-0.84396
Q8TCD5	NT5C	0.453896	0.057386	-0.43841	0.015624	0.343088	0.403205	-1.03484	-0.14053	0.130817	-0.29986	0.839183	-0.33168	0.078105	0.009799	0.002613	0.173078	-0.45816	-0.16463	0.363238
Q8TCE6	FAM45A	-0.41058	0.57055	0.126941	-0.12183	0.047155	-0.17125	0.268056	0.143972	0.098768	0.424664	0.545533	-0.02651	0.167795	-0.00917	0.163827	0.440403	0.108021	1.034356	0.0306
Q8TCF1	ZFAND1	-0.5876	0.043631	0.415326	-0.26677	-0.23519	-0.03037	0.655611	-0.17984	0.208132	-1.01291	0.049083	0.188594	0.010936	-0.18577	-0.1724	0.125403	0.208613	-0.05567	-0.0966
Q8TCG1	KIAA1524	0.223251	0.070354	1.122434	-0.37809	0.485231	0.881357	1.392285	1.176844	-0.36589	0.50647	-0.76231	-0.2436	-0.21088	-0.00683	-0.76457	-1.09787	0.532438	-0.62174	0.588566
Q8TCG2	PI4K2B	0.320513	0.147786	0.015113	-0.11785	0.147242	0.106699	-0.26441	0.246485	-0.07019	0.210328	-0.04615	0.120065	0.346496	-0.44476	-0.52956	0.144052	0.085865	0.405375	0.097557
Q8TCJ2	STT3B	0.64569	0.501986	0.355905	-0.61892	-0.15926	-0.31255	0.820495	-0.52976	0.142045	-0.25126	0.172965	0.237024	-0.19891	0.173369	0.034542	0.213278	0.076441	0.104248	0.194294
Q8TCS8	PNPT1	0.492764	0.104835	0.811292	0.436361	-0.00755	0.030359	-0.51246	0.496981	0.169358	0.605286	0.099692	-0.08801	-0.23225	-0.13577	0.836894	0.748977	-0.83348	-0.8809	-0.51793
Q8TCT9	HM13	-0.0501	0.570631	0.463494	-0.30942	0.259262	0.02554	0.555779	-0.01895	-0.15701	0.654722	-0.48185	0.840063	0.422944	0.255439	-0.37585	0.143563	1.050483	1.193415	-0.03229
Q8TCU6	PREX1	-1.35187	1.049284	0.31922	-0.85682	0.558957	0.13177	0.150051	-0.67727	0.046515	1.348313	-1.31811	1.0429	-0.37929	1.746327	-0.35613	-0.59923	0.444473	-0.10072	-0.80371
Q8TCY9	URGCP	-0.06956	0.280612	0.365343	-0.54713	0.187853	0.446195	0.31927	0.676375	0.200395	0.173498	-0.14859	-0.20552	0.214701	0.077943	0.098153	0.446022	0.584542	0.362098	0.544479
Q8TD06	AGR3	2.589151	0.100891	-1.06852	-1.15607	-0.93692	-0.66678	-1.00955	-0.03209	-0.68145	-2.26241	3.711852	-0.29238	-0.21036	-0.20163	-1.37504	-1.43587	0.380292	-0.95293	-2.4096
Q8TD16	BICD2	-0.56954	0.176939	-0.00056	0.002288	0.279144	0.520113	0.014789	-0.0895	-0.18419	0.312128	0.239033	-0.45762	0.136492	0.040736	-0.22905	0.091053	-0.12981	0.351601	0.366278
Q8TD19	NEK9	-0.95881	0.485034	0.328195	-0.30412	-0.25344	-0.01859	0.336497	-0.25589	-0.04381	0.047379	0.061507	0.010603	0.124436	0.212701	-0.42853	-0.21248	-0.08411	-0.19596	0.317618
Q8TD22	SFXN5	-0.32943	0.093964	0.47971	-0.82045	0.506369	1.014316	-0.2967	1.059386	0.291882	0.005105	0.585757	0.51041	-0.14977	0.066163	-0.73572	0.099327	-0.38318	-0.02181	0.51856
Q8TD26	CHD6	0.094528	-0.06026	0.097732	0.031975	-0.39032	-0.05123	0.13252	0.664779	-0.06859	0.440269	-0.13985	-0.71704	-0.22278	0.402166	0.500423	0.018635	-0.31766	0.062962	0.297885
Q8TD30	GPT2	-0.44936	0.08686	0.019478	1.010111	-1.10478	-1.10708	-0.2568	0.446395	0.090439	0.781133	-0.45196	-0.09885	-0.23888	0.103917	-0.24707	0.237497	0.492052	-0.31855	0.160453
Q8TD43	TRPM4	0.35107	-0.04583	-0.21774	0.522677	-0.6075	-0.58244	-0.28519	-0.80818	-0.3302	0.002884	0.186808	-0.10344	-0.19557	-0.52341	0.881208	0.356582	-0.1253	-0.61938	-0.80074
Q8TD55	PLEKHO2	-1.4269	0.591834	0.988343	-0.75862	0.533939	-0.19859	-0.01183	-1.53207	0.486368	1.156662	-0.87958	1.406519	0.112946	1.452203	-0.28678	-0.44294	0.417956	0.430758	-0.77791
Q8TDB4	MGARP	-2.29932	0.272776	-0.12563	-1.29412	-1.09251	-0.43425	-0.18477	-1.0612	0.449801	-1.52032	1.077564	-1.65636	0.396952	-2.04948	-0.39856	-0.58933	-0.81528	2.019489	2.701474
Q8TDB6	DTX3L	0.618175	0.752485	1.228277	-0.77562	0.630004	1.060546	0.089416	1.120185	0.434997	0.348675	-0.4683	0.4526	0.465584	0.425483	0.180477	-0.33727	-0.20384	-0.19058	-0.61159
Q8TDD1	DDX54	0.190993	0.220169	-0.47605	1.212858	0.062263	-0.03153	-0.03068	-0.21352	-0.72026	-0.52611	0.47854	0.046562	0.316929	-0.38696	-0.43305	0.52844	-0.04419	0.081434	-0.49311
Q8TDH9	BLOC1S5	0.521306	-0.29339	-0.47037	-0.19804	0.370917	0.245444	-0.65893	-0.82116	-0.20539	-0.16328	-0.19552	-0.10226	0.019241	-0.19371	0.24117	-0.23285	-0.56968	-0.45617	-0.28167
Q8TDJ6	DMXL2	-0.549	0.512204	0.454462	-0.46351	0.292585	-0.00296	0.690145	-0.70391	-0.0065	0.814198	-0.54774	1.089851	-0.07837	1.160079	-0.57637	0.247434	0.655543	0.153274	-0.67789
Q8TDM6	DLG5	0.135873	0.420332	-0.15288	-0.78648	-0.13766	0.060917	-0.11495	-0.02365	-0.17451	-1.12973	0.136195	-1.04122	-0.27609	-0.7013	-0.33011	-0.20245	-0.58955	-0.34393	-0.34229
Q8TDN6	BRIX1	0.069578	-0.05059	-0.11961	0.122762	-0.04168	-0.09595	-0.28292	0.296026	-0.11144	-0.71775	0.680081	0.012363	-0.03322	-0.75697	0.150945	0.179164	-0.2425	0.059494	0.432899
Q8TDP1	RNASEH2	-0.54887	-0.18577	0.295161	-0.20423	1.146155	1.202392	0.019773	0.141424	0.194014	0.321192	-0.01523	-0.2002	-0.1053	0.493433	-0.04415	0.192117	0.44956	1.367455	-0.84658
Q8TDQ7	GNPDA2	0.799507	-0.39921	-0.00975	-0.49104	-0.61162	-0.82483	-0.43121	0.23572	-0.01855	0.548097	0.310572	-1.20887	0.243621	-0.57594	-0.40658	1.110808	-0.9994	-0.08819	1.201641
Q8TDR0	TRAF3IP1	-0.20109	-0.32951	-0.5566	-0.36298	-1.22227	0.019788	-0.5217	-0.1354	-0.17603	-0.78299	2.245404	-1.52295	-0.70925	-1.10315	0.092516	-0.28614	-0.93658	-0.52343	-0.09603
Q8TDW0	LRRC8C	-0.0756	0.719389	1.196556	0.179684	0.938055	0.303743	1.4314	-1.01276	0.292563	0.444121	-0.9065	0.554509	1.406702	0.641347	1.196957	-0.22329	-0.24151	-0.07914	-1.17598
Q8TDX7	NEK7	-0.05311	0.894028	0.168887	-0.4672	0.125147	-0.00021	-1.60634	-0.3235	0.160642	-0.22485	-0.38361	0.146052	-0.28853	0.903577	0.667576	-0.38849	0.120904	-0.27808	-0.06349
Q8TDY2	RB1CC1	-0.09938	0.520436	0.953015	-0.37804	0.479385	0.617293	0.942352	-0.17179	0.025067	-0.28338	-0.20315	0.334593	2.045893	-0.25125	0.486872	0.447376	-0.35165	-0.15973	0.23138
Q8TDY4	ASAP3	-0.25216	-0.00493	0.207329	-0.78464	-0.46917	-0.81462	0.065059	0.257369	0.303382	0.425712	-0.97116	-0.20105	-0.22076	-0.23681	0.677861	0.046291	-0.66808	-0.23654	1.121447
Q8TDZ2	MICAL1	-1.24784	0.731082	0.367701	-0.9278	0.325056	0.209352	-0.23493	-1.16422	0.174262	1.310892	-1.16592	0.168996	-0.0457	0.621104	-0.50869	-0.41696	0.258065	0.04186	0.282964
Q8TE02	ELP5	0.301031	0.321823	0.221445	-0.53385	0.338296	0.916458	0.265312	-0.39783	-0.01881	-0.13497	0.086988	-0.13642	0.121462	-0.01127	0.099144	-0.24641	-0.04492	-0.30052	0.291454
Q8TE57	ADAMTS1	-0.76519	-0.40522	0.039165	-0.47193	-0.90358	-1.10627	-0.16606	-1.60812	0.311757	-0.35793	-1.37896	-1.01989	-0.03859	-0.18061	-0.97215	0.028432	-1.19435	-1.09244	0.821833
Q8TE68	EPS8L1	0.299626	-0.95616	-0.62498	0.828903	-0.51604	0.208104	-1.46056	0.104974	0.223927	0.584705	0.481393	-0.7745	0.125218	-0.28417	1.128921	0.782752	0.601589	-0.05694	-0.31516
Q8TE77	SSH3	0.101745	-0.47772	0.339651	-0.66595	0.097019	0.380952	-0.22999	0.259046	-0.54003	-0.5509	0.454365	-0.06875	0.016392	-0.15006	-0.27705	0.43133	0.121799	-0.70655	-0.5972
Q8TE82	SH3TC1	-0.64013	1.032455	0.274063	-0.67196	-0.05946	-1.1206	1.194379	-0.89665	0.09106	1.949027	-0.45863	1.14874	0.661884	0.723011	-0.23186	-0.40306	-0.3913	-0.31665	-1.44989
Q8TEA1	NSUN6	-0.01139	0.408329	0.179774	-0.29622	0.355048	0.667696	0.430038	-0.12471	-0.00852	-0.34009	-0.31288	-0.15668	0.518021	0.097358	0.034181	-0.13039	0.376784	-0.60677	0.172734
Q8TEA7	TBCK	-0.0534	0.422508	0.289285	-0.67669	-0.06405	-0.37443	-0.37457	0.300702	-0.35138	0.504807	-0.06453	0.148367	0.298694	0.031482	-0.37287	0.008793	-0.73743	-0.29045	0.034306
Q8TEB1	DCAF11	0.545938	-0.02905	-0.04135	0.160304	0.04996	0.299648	-0.49209	-0.16302	-0.16067	0.547715	0.28426	-0.20691	0.228197	-0.18072	0.141031	0.027898	0.187681	-0.45358	-0.34236
Q8TEB9	RHBDD1	-0.3527	-0.17629	-0.20396	0.234193	-0.38077	-0.29692	0.034772	-0.47004	0.038397	-0.1564	0.379852	-0.31352	-0.10591	-0.38435	0.704431	-0.20803	-0.50315	-0.16939	-0.03105
Q8TED0	UTP15	0.260464	-0.17137	-0.11087	0.477977	-0.02015	0.009027	-0.77512	0.275512	-0.39535	-0.19783	0.574014	-0.13648	-0.15766	-0.65943	0.620148	0.394128	-0.23584	-0.28747	0.004341
Q8TED1	GPX8	0.834293	0.980898	0.288428	-0.14786	0.056289	-0.05996	0.466002	0.078542	0.805057	0.020663	-0.65917	-0.55273	0.537101	-0.86227	-1.0246	-0.09985	0.266987	0.813384	2.13759
Q8TEH3	DENND1A	0.290745	-0.12977	0.065507	-0.3049	-0.6031	-0.15784	0.266745	-0.49346	0.077687	-0.11979	-0.13747	0.158762	-0.38176	0.342588	-0.47286	-0.48141	-0.07593	0.169622	0.447116
Q8TEM1	NUP210	0.873575	0.240553	-0.2669	0.205943	1.101031	1.10126	0.59261	0.645874	-0.35221	0.402972	0.155986	0.435637	0.01134	0.278895	-0.32448	0.331319	-0.63942	-0.97692	-0.81459
Q8TEP8	CEP192	0.659406	0.097524	0.026195	-0.10271	-0.25165	-0.38932	-0.10903	0.164862	-0.10208	-0.15969	-0.2148	-0.18038	-0.09233	-0.06358	0.31063	0.172302	0.021071	-0.25541	-0.01411
Q8TEQ0	SNX29	-0.67784	0.582851	0.247747	-0.93162	0.138461	0.031881	0.401836	-0.6658	-0.32328	0.434262	-0.28963	0.225658	-0.38065	0.315476	-0.14878	0.141372	-0.23215	0.255206	0.240188
Q8TEQ6	GEMIN5	-0.41554	0.026943	-0.00858	0.471527	0.25447	0.532048	0.232223	-0.06447	0.053866	-0.24089	0.571336	-0.38964	0.070984	-0.34526	0.279468	0.082483	0.027818	-0.17341	-0.13534
Q8TEQ8	PIGO	0.42479	0.002378	-0.16118	0.324581	0.140729	0.097266	-0.60623	0.116069	0.084992	-0.50692	0.036552	-0.65398	0.048901	-0.42122	0.316133	1.244242	0.145188	-0.01526	-0.4925
Q8TER0	SNED1	-1.61411	0.154629	-0.35924	-1.50146	-1.3749	-1.39725	0.260637	-1.69156	0.520758	-0.79992	0.03648	-1.16947	-0.42283	-0.47882	-0.77458	-0.52928	-0.73529	-1.21642	-0.25351
Q8TER5	ARHGEF4	0.30164	0.438736	0.288611	-0.78067	-0.45633	-0.36742	0.382654	0.807022	0.150044	0.338325	-0.03665	-0.38371	-0.24722	0.031204	-0.47706	-1.08626	-0.14453	-0.03718	0.363896
Q8TEU7	RAPGEF6	0.043434	0.328872	0.375519	-0.69025	0.12098	0.12047	-0.35976	-0.25786	0.11442	0.239279	0.031359	0.299157	0.337155	0.906237	0.347079	-0.0585	0.246996	0.07389	-0.39737
Q8TEV9	SMCR8	-0.40743	0.736509	0.555807	-0.36126	0.115345	-0.51466	0.303077	-0.67336	-0.07149	0.501618	-0.22416	1.372374	-0.06173	1.017397	-0.90175	-0.05043	0.285694	-0.11343	-0.86929
Q8TEW0	PARD3	-0.04663	-0.59118	0.077599	-0.3472	0.020394	0.214875	-0.27379	0.081105	0.584278	-0.37942	-0.07347	-1.19527	0.275174	-0.44089	0.775008	0.103235	-0.70756	-0.1377	-0.08563
Q8TEW8	PARD3B	0.392391	-0.18276	-0.14936	-0.8887	-0.19491	-0.80412	0.040088	0.427245	-0.05217	-1.49046	0.302316	0.257958	-0.04094	0.10098	0.484908	-0.29714	-0.27888	-0.86774	-0.6755
Q8TEX9	IPO4	0.437906	0.014582	-0.39529	0.376898	-0.46491	-0.37337	0.044345	0.20377	1.216056	-0.17123	0.290672	-0.04803	-0.24916	-0.19609	0.175416	0.266166	0.633577	-0.78184	0.291461
Q8TF01	PNISR	0.328709	-1.56164	0.018943	0.266738	0.252349	0.560521	-0.85449	-0.18192	-0.21838	0.152669	0.823814	-1.22453	-0.35551	-1.55899	0.366901	-0.17789	0.054675	0.665899	-0.32709
Q8TF05	PPP4R1	0.073512	0.771678	0.229798	0.35215	-0.04083	0.384537	-0.34564	0.353006	0.084391	0.086789	-0.17269	-0.1155	0.312185	0.262293	-0.92219	-0.05898	0.696409	0.133084	0.46957
Q8TF42	UBASH3B	-0.02033	0.398113	0.362163	-0.76469	0.662302	-0.29285	-0.06059	-0.1255	-0.06672	0.155108	-0.41343	1.298242	-0.45789	1.288142	-0.71611	-0.76324	0.974983	-0.04502	-0.5722
Q8TF46	DIS3L	0.403072	-0.16155	-0.30073	-0.45295	0.344536	0.474877	-0.13295	-0.59144	-0.15865	-1.01639	0.047813	-0.35736	0.026602	0.136062	-0.2535	-0.05803	0.65501	0.086334	-0.41491
Q8TF66	LRRC15	-0.74283	0.516294	-0.64347	-1.53304	-0.75008	-0.84112	0.301027	-0.09066	0.187808	-0.09831	-0.12989	-0.14092	0.128324	-0.00101	-0.35824	-0.28209	-0.48855	0.032373	-0.48164
Q8TF68	ZNF384	0.608594	-0.29669	-0.48663	-0.15909	-1.19626	-1.08156	0.040198	1.030865	-0.44705	-0.07243	0.280094	-0.50674	-0.42983	0.090226	0.11902	-0.18574	0.055658	-0.24388	-0.68004
Q8TF72	SHROOM:	-0.04765	-0.26695	-0.43348	-0.53675	-0.44205	-1.02298	-0.78798	-0.08083	-0.46095	0.470345	0.668411	-0.11333	-0.84035	0.197735	-0.25121	0.852474	0.083359	-0.44288	-0.2843
Q8TF74	WIPF2	-0.00959	0.161984	0.181179	-0.17784	0.185635	-0.29769	0.093273	-0.14608	0.046741	-0.11588	-0.35703	-0.15652	-0.11462	0.09396	0.006074	-0.00864	-0.21621	-0.80171	0.218791
Q8WTS6	SETD7	-0.63429	0.175232	-0.06031	-0.76071	-0.12024	-0.71678	0.140639	-0.35808	0.444695	0.568687	-0.96661	-0.90317	0.146508	-0.74211	-0.16626	-0.08228	-0.34608	0.357956	0.70949
Q8WTT2	NOC3L	0.37644	0.097213	-0.61626	0.893453	-0.13605	-0.22548	-1.10605	-0.20959	0.041509	0.117207	0.571361	-0.20181	0.223725	-0.6486	-0.13329	0.225599	-0.28351	-0.26491	-0.79088
Q8WTW3	COG1	-0.52358	0.391876	0.590144	-0.08756	0.184441	0.297648	0.277724	-0.36374	0.02513	0.186069	-0.10219	-0.02607	-0.0172	-0.0742	0.79392	0.138756	-0.19266	0.294083	0.185706
Q8WU39	MZB1	-2.66578	0.008301	-0.36931	-2.96055	0.782877	-1.61753	0.564159	-1.31571	0.915138	-2.35105	-2.56854	-0.82311	-0.58067	-0.19567	-1.0545	-1.34154	-0.77963	1.499553	-0.81136
Q8WU76	SCFD2	-0.47608	0.329324	0.257874	0.336064	-0.19775	-0.2387	0.120749	0.069386	0.618627	0.424168	0.425211	-0.12168	0.048504	-0.00201	0.828451	0.664611	-0.29417	0.347127	0.681217
Q8WU79	SMAP2	-0.72165	0.222757	0.488858	-0.3901	0.257066	-0.09853	-0.331	-0.75052	-0.05072	1.24345	-0.36774	0.65308	0.208482	0.944636	-0.34873	-0.14563	0.209081	0.662796	-0.28866
Q8WU90	ZC3H15	0.292656	-0.20085	0.005154	-0.35766	-0.63437	-0.41239	-0.62264	0.15681	-0.1948	0.018326	0.61938	-0.79723	-0.10107	-0.57869	0.539184	0.305419	-0.30882	-0.15284	0.091354
Q8WUA2	PPIL4	0.837538	-0.73991	0.120624	0.304041	-0.16275	0.023742	-1.0294	0.081775	-0.86192	0.238752	0.992185	-0.23235	0.27774	-0.38812	0.041652	0.393884	0.351159	0.572072	0.194901
Q8WUA4	GTF3C2	0.887081	-0.75484	0.251288	-0.06478	-0.31305	-0.02225	0.525244	0.611261	-0.2303	-0.45665	0.041871	-0.18511	-0.41429	-0.12156	0.38196	-0.08131	0.009942	-0.18589	0.037649
Q8WUA7	TBC1D22/	-0.31202	0.284621	0.666733	0.120491	0.353315	0.361277	0.071469	-0.48641	0.170136	0.524357	-0.09137	0.035888	0.629793	-0.13661	0.03932	-0.3779	-0.08689	-0.12304	-0.23448
Q8WUB8	PHF10	0.577547	-0.42289	0.028762	0.232881	-0.03071	0.09916	-0.49583	0.350602	-0.74088	-0.2887	0.133101	-0.87446	-0.35371	-0.49934	-0.28855	-0.97662	-0.42628	-0.40494	-0.38643
Q8WUD1	RAB2B	-0.18258	-0.03259	0.201039	-0.42704	-0.47326	-0.52328	-0.22632	-0.12118	0.154347	0.000681	-0.14297	-0.17008	0.034218	0.340951	-0.36705	0.041895	-0.00897	0.089997	-0.74331
Q8WUD4	CCDC12	1.18699	0.151605	-0.48293	-0.26863	-0.3587	0.329362	-0.91179	0.866571	-0.12687	-0.30312	0.486451	-1.44268	0.43196	-0.99219	0.512726	0.10039	-0.75712	0.146806	0.08561
Q8WUF5	PPP1R13L	0.175588	-0.66917	-0.49575	-0.71091	-1.13304	-0.61825	-0.33566	-0.16096	0.112206	-0.90536	0.09006	-0.82658	0.495663	-0.66075	-0.46163	-0.83447	-0.08125	-0.68536	0.270741
Q8WUF8	FAM172A	-0.74121	-0.81446	-0.51954	0.496989	-0.24591	-0.86451	-0.32381	-0.30207	-0.37308	-0.26101	0.629446	-0.36352	-0.62456	0.165079	1.069294	-0.03305	-0.13555	0.077328	0.073875
Q8WUH2	TGFBRAP	0.093772	0.289398	0.179876	-0.1023	-0.26405	-0.32999	0.189425	-0.0082	-0.194	0.37629	0.040078	0.124879	-0.09531	0.45067	0.080716	-0.1404	-0.24595	-0.29079	-0.01987
Q8WUH6	TMEM263	-1.11312	0.040658	0.056799	-0.20491	-1.40143	-0.25497	0.266155	-0.72344	0.339764	0.239155	-0.49519	0.10637	0.576904	-0.01621	0.276545	-0.29113	0.707902	0.575084	1.497055
Q8WUI4	HDAC7	0.221079	-0.37949	0.044309	0.180837	-0.17428	0.074465	-0.25581	0.35859	-0.15779	-0.10129	-0.02367	-0.36413	-0.20324	-0.32076	-0.30355	-0.24654	-0.27532	-0.26137	0.180446
Q8WUK0	PTPMT1	0.171729	0.467877	-0.08676	1.189436	0.278619	0.154432	-0.08928	-0.54829	-0.14889	0.489947	0.071416	-0.26918	-1.55554	0.077131	-0.77238	1.53365	-0.42615	-0.9944	-0.92386
Q8WUM0	NUP133	0.028189	0.307373	0.100012	0.231191	-0.07585	0.076098	0.378884	0.260259	-0.13803	-0.16397	0.053845	0.090561	-0.33702	-0.06542	0.675527	-0.14564	0.202622	-0.16294	0.296446
Q8WUM4	PDCD6IP	0.753589	0.235378	0.174373	-0.61479	0.037022	0.187718	-0.00574	-0.51867	-0.296	-0.26129	-0.084	0.060891	0.120251	0.044392	-0.35579	0.193089	-0.56282	-0.8336	-0.14334
Q8WUN7	UBTD2	0.309604	0.018461	-0.0264	-0.28239	-0.58925	-0.82363	0.075556	0.271705	0.33835	-0.4154	-0.31581	0.061169	0.185404	0.025426	0.208161	-0.04226	0.71447	0.365901	0.405551
Q8WUP2	FBLIM1	0.4071	-0.3684	-0.04337	-0.24338	-0.67201	-0.34161	-0.25449	0.155955	-0.13319	0.056152	-0.04954	-1.05032	0.261133	-1.76723	-0.31209	0.288101	-0.34265	0.180511	1.119006
Q8WUQ7	CACTIN	0.329471	-0.32709	-0.47352	-0.00206	0.179379	0.170571	-0.49796	0.339006	0.215114	-0.49812	0.16605	-0.87723	-0.84502	0.119534	-0.07788	-0.32578	-0.4609	0.272871	-1.17842
Q8WUW1	BRK1	0.135183	0.576214	-0.12724	-0.86473	0.087544	0.031286	0.097482	-0.07284	-0.07198	0.323888	0.268003	-0.19078	-0.63337	0.003208	-0.3395	0.102492	-0.2188	-0.06784	-0.08942
Q8WUX9	CHMP7	0.092183	0.553502	0.791343	-0.20134	-0.08089	0.03743	0.203471	-0.14043	0.025813	0.345856	-0.40156	-0.07694	0.382923	0.205816	-0.82481	-0.2845	0.561347	0.631905	0.786548
Q8WUY1	THEM6	1.517599	0.170706	-1.03866	-0.61944	-0.06103	0.3217	-0.81817	-0.54975	-0.99333	-0.54315	-0.04945	0.447342	-0.1898	-0.07511	0.548909	-1.09491	-0.39012	-1.33865	-1.50863
Q8WUY3	PRUNE2	0.799119	0.333596	-1.32335	1.123657	1.475965	1.650454	-1.2052	-0.13163	-0.0043	0.101832	0.423453	-0.58067	-0.04445	-0.46671	0.529964	-0.2232	-0.04114	-0.24674	-0.33184
Q8WUY8	NAT14	0.5501	-0.02503	-0.26083	-0.04633	-0.16824	-0.22614	0.646022	0.465037	0.080741	-0.94158	0.752129	-0.79115	-0.33212	-0.63374	0.819626	-0.2133	-0.20485	-0.35475	0.95878
Q8WUZ0	BCL7C	0.250333	0.043438	-0.50769	0.25305	-0.29264	-0.04917	-0.12085	0.277081	0.102603	0.011124	0.260342	-0.29897	-0.24603	-0.42662	0.069075	0.033669	-0.28426	-0.00673	-0.81349
Q8WV22	NSMCE1	0.535589	-0.02066	-0.09059	0.247534	-0.41265	-0.03339	-0.12328	0.27855	0.044657	-0.22649	0.674646	-0.18933	-0.14038	-0.06844	-0.01575	0.045531	0.032153	0.007113	0.075075
Q8WV41	SNX33	-0.71459	0.728814	0.216436	-0.56705	0.038971	-0.40517	-0.18455	-0.18132	0.946289	-0.33482	-0.04342	0.01129	0.661486	-0.05168	-0.22809	-0.61194	0.004304	-0.09861	0.065229
Q8WV92	MITD1	-0.5073	-0.1977	-0.2226	-0.22682	0.309001	0.440481	-0.0531	0.983068	0.29105	-0.00575	0.416504	-0.34182	-0.12065	0.218707	-0.39023	-0.05656	-0.34564	-0.71483	0.053212
Q8WVB3	HEXDC	-0.37091	0.028449	-0.02944	0.247937	0.020721	-0.62145	0.194939	-1.60371	-0.29191	-1.46822	0.426621	-0.543	-0.21665	0.025042	-0.61495	-0.76085	-0.9118	-0.90891	-1.32847
Q8WVB6	CHTF18	0.085662	-0.30397	-0.17967	-0.20473	0.044955	0.784385	-0.316	0.54394	-0.32347	-0.29146	-0.11337	0.226325	0.125943	0.215934	0.487786	0.367178	0.467586	-0.45425	-0.60345
Q8WVC0	LEO1	0.919122	0.057352	-0.8785	-0.14253	-0.07864	0.30633	-0.53936	0.434402	0.376928	-0.02473	0.223909	-0.3645	0.499166	-0.12909	0.05002	-0.03337	-0.22039	0.313251	0.11214
Q8WVC6	DCAKD	-0.24428	1.108826	0.190997	-0.25078	-0.50441	-0.42371	0.261624	-0.86174	0.182834	-0.65589	1.708782	-0.66328	-0.26881	-0.41478	-0.79281	0.438686	-0.51236	-0.14945	-0.23863
Q8WVF1	OSCP1	-0.80975	-0.3398	-0.59769	0.264925	-0.17414	0.374304	-0.15161	-0.37545	-0.2889	-0.35032	1.589649	-0.2336	-0.20121	-0.67887	-1.14354	-0.27566	-0.39551	-1.09523	-0.27971
Q8WVJ2	NUDCD2	-0.50514	-0.54045	-1.69618	-0.562	-0.06657	-0.08642	-0.41403	0.289743	0.387716	0.965749	1.015254	0.289167	-0.50449	0.07415	-1.00998	0.40767	0.283598	-0.01742	-1.11242
Q8WVK2	SNRNP27	0.736473	-0.15063	-0.08553	-0.05229	0.030462	-0.01244	-0.25143	0.518403	0.157077	0.278889	0.122298	-0.38199	-0.33541	-0.4969	0.191585	-0.21712	0.155414	0.161226	0.673583
Q8WVM0	TFB1M	-0.66574	0.18229	-0.31298	0.768958	0.214448	-0.19913	-0.23306	-0.29723	-0.39624	-0.15838	0.729761	0.05122	-0.08939	0.064386	-0.11486	-0.14132	-0.48442	-0.51785	-0.60036
Q8WVM7	STAG1	0.671696	-0.01119	0.179732	-0.0168	0.437098	0.709237	0.155024	-0.01792	-1.19418	0.058179	-0.0877	-0.12867	0.038626	-0.42049	0.892335	-0.41205	-0.6896	-0.24167	-0.03893
Q8WVM8	SCFD1	-0.4152	0.492623	0.09994	0.091087	0.018362	-0.1155	0.696459	-0.11392	0.10943	0.185478	0.034544	-0.44791	0.121284	-0.466	0.56557	0.558499	0.044046	0.566426	0.474744
Q8WVQ1	CANT1	-0.28345	0.354709	0.468519	0.479848	0.088404	0.084076	0.023601	-0.02114	0.498886	0.37068	0.098764	0.181775	0.446958	0.007379	0.221272	0.148859	0.309433	0.170155	-0.29028
Q8WVR3	C7orf43	-0.52743	-1.10681	-0.93617	-0.77925	-0.05461	-2.29715	0.215436	-0.26334	0.635662	-0.13114	-0.6369	0.832306	-0.40686	0.705383	-0.28455	-0.62248	0.241312	-0.50213	-0.18554
Q8WVT3	TRAPPC1:	-0.41668	0.598663	0.419761	-0.07353	0.397757	-0.05517	0.21763	-0.17747	0.165029	0.271199	-0.04906	0.367841	-0.28199	0.479961	0.033744	0.096597	-0.32165	-0.45704	-0.00576
Q8WVV4	POF1B	1.23484	1.695493	-1.85734	1.505143	-1.58652	-0.70327	-0.06002	-0.56543	-0.85765	-1.00211	1.306027	-1.05354	-1.13894	-1.07599	0.810008	-2.12041	-0.70151	-1.82875	-1.40315
Q8WVV9	HNRNPLL	0.580118	0.053954	-0.00217	-0.11297	-0.56454	-0.35869	-0.16238	0.439252	-0.0596	-0.07665	0.231763	-0.03734	-0.26271	-0.089	-0.05003	0.025623	-0.04252	-0.32333	0.145752
Q8WVX9	FAR1	-0.8778	0.193324	0.148129	-0.23195	0.915683	0.412752	0.190951	-0.21336	0.017652	0.220396	-0.29808	0.901578	0.177073	0.55101	-0.02917	0.02497	0.346095	0.567366	-0.20525
Q8WVY7	UBLCP1	-0.79561	0.144963	0.292703	0.472678	0.356945	0.507156	-0.74279	-0.12225	0.174619	-0.14094	0.134066	0.059625	0.316743	-0.14883	0.271996	-0.23085	-0.45865	-0.22789	0.312501
Q8WW12	PCNP	0.023536	0.205453	0.218436	-0.59647	0.097466	-0.03578	-0.24647	-0.75985	-0.11308	-0.09924	-0.30799	-0.14172	0.368444	-0.08699	1.318205	-0.52328	-0.00267	0.166593	-0.22148
Q8WW22	DNAJA4	-0.12239	0.329599	-0.16254	-0.96477	0.108953	-0.41176	0.021309	-0.62504	0.631279	-0.78526	1.078304	0.460862	-0.18139	0.246632	-0.29507	-0.76037	0.036383	0.399874	-0.2547
Q8WW59	SPRYD4	-0.02247	0.668142	0.305992	0.341985	2.132574	-0.2743	-0.33574	-0.16913	0.076514	0.956599	0.037109	0.430043	0.518338	0.306245	0.361332	0.494205	0.526824	0.030126	0.15635
Q8WWC4	MAIP1	0.362301	-0.20476	0.40525	0.022917	-0.05988	-0.08036	0.086796	-0.02814	-0.40146	-0.32776	0.590759	-0.07989	-0.0477	-0.14286	0.674604	0.788071	0.150812	-0.31355	-0.52765
Q8WWH5	TRUB1	-0.25123	-0.17412	0.244845	-0.55203	-0.41295	-0.05088	-0.01156	0.214591	-0.03304	-0.083	0.482031	-0.51447	0.367252	-0.33659	0.18922	0.2829	0.392048	0.064529	-0.08458
Q8WWI1	LMO7	0.798575	-0.70807	-0.99011	-0.72644	-0.81299	-0.433	-0.54178	0.764781	0.378922	-0.54987	0.335496	-0.8859	0.584188	-1.23921	-0.6398	-0.45887	-0.86039	-0.69966	0.234322
Q8WWI5	SLC44A1	-0.20646	-0.44011	-0.68705	-0.59257	-0.4567	-0.02289	0.473851	-0.82257	0.114612	-0.48038	0.586846	-1.03019	-1.74449	-1.02903	-0.32654	0.518005	-0.78664	-0.6133	-0.62315

Q8WWM7	ATXN2L	-0.033	-0.15516	0.126406	-0.07554	-0.52748	0.024701	-0.63096	-0.21719	-0.14557	0.200603	-0.0216	-0.42166	0.69612	-0.62419	-0.20079	0.54947	0.15024	0.145008	0.163629
Q8WWM9	CYGB	-1.18491	0.128808	-0.03341	-0.48103	-0.15567	-1.16764	-0.11613	-0.77778	0.309594	0.200837	-0.30047	-0.73217	0.076839	0.271727	-0.05225	0.20621	-0.48544	0.338066	0.197673
Q8WWN8	ARAP3	-0.07351	0.181915	-0.24807	-0.41352	-0.27458	-0.91065	0.134011	-0.13711	0.050746	0.6743	1.200761	-0.06371	0.172217	0.273626	0.760593	-0.1738	-0.42228	-0.45274	-0.06362
Q8WWP7	GIMAP1	-1.81472	1.549773	2.165075	-0.83976	0.95407	0.14335	0.653306	-2.03889	-0.07892	1.17219	-1.52485	1.454501	-0.37027	1.580249	-0.34052	-0.74301	0.434986	1.014267	-1.19488
Q8WWQ0	PHIP	0.44706	-0.32263	-0.39466	-0.08942	-0.82806	-0.59197	0.24563	-0.19784	1.987938	-0.54052	-0.28723	-0.32214	-0.0815	-0.31116	1.687152	-0.46816	-0.17394	-0.03112	-0.35631
Q8WWV3	RTN4IP1	-1.00149	0.055984	0.75742	1.250628	0.06128	-0.05609	0.621077	-0.70103	-0.11347	0.38338	0.418195	-0.05287	-0.29184	-0.02356	1.789669	-0.22984	-0.22239	-0.7921	-1.21114
Q8WWX9	SELENOM	-0.69345	0.41939	0.605874	-0.71001	0.93994	0.013789	-0.41634	-0.58775	-0.24221	0.402475	-0.30417	-0.50411	0.479251	-0.42754	-0.58544	0.327553	0.140129	1.151198	1.21276
Q8WWY3	PRPF31	0.375178	-0.0954	-0.15927	0.364084	-0.24819	-0.01899	0.016913	0.342032	-0.06884	-0.27191	0.362401	-0.45822	-0.43619	-0.27981	0.009888	-0.10686	-0.14335	0.063295	0.107089
Q8WX92	NELFB	0.515884	-0.26698	-0.12947	0.272099	0.05418	0.250383	0.184436	0.623335	-0.23069	-0.04307	-0.60341	-0.25316	-0.46825	-0.31795	0.349692	0.176275	-0.22142	-0.44626	0.464953
Q8WX93	PALLD	-1.24557	-1.246	-1.0268	-0.98396	-1.6017	-1.15912	-1.54017	-0.45777	0.684335	-0.77816	-1.17987	-1.23769	-0.19858	-0.96409	-0.85155	0.39915	-1.30866	-0.49928	1.189592
Q8WXA3	RUFY2	-0.44971	0.861172	-0.46845	-0.67498	0.211825	0.276242	0.17524	-0.23244	0.133011	-0.26324	0.005991	0.189192	0.79872	0.097199	-0.07672	0.730701	-0.21801	0.077566	-0.04525
Q8WXA9	SREK1	0.5347	-0.53071	-0.9237	0.023045	-0.34062	0.00564	-1.39923	0.651787	0.429419	-0.09176	0.457533	-0.8913	-0.13379	-0.79142	0.4988	-0.01046	-0.26166	0.215873	0.006112
Q8WXD5	GEMIN6	-0.07211	0.031714	0.237682	-0.46029	-0.21715	-0.05117	-0.0303	0.403031	0.469614	-0.27326	0.09771	-0.20164	-0.0393	0.14701	-0.10633	-0.06918	0.492533	-0.31187	-0.14361
Q8WXE0	CASKIN2	0.115406	-0.6227	0.392108	-0.38373	-0.69033	-0.91521	-0.39295	0.681243	0.1289	-0.25129	-0.37211	-0.83029	-0.04131	-0.69746	0.270956	0.200022	-0.47	0.370157	0.353276
Q8WXE9	STON2	0.985834	-0.50636	-0.61371	0.201323	0.025197	0.55704	0.202993	0.193108	-0.74112	-1.87048	0.139241	-0.41703	0.514671	-0.30386	2.0217	0.583509	-0.37497	-0.41236	-0.43744
Q8WXF1	PSPC1	0.577857	-0.34783	-0.71089	-0.54844	-0.32931	-0.39706	-0.62761	0.667071	0.077352	-0.14918	0.710714	-0.1103	-0.07394	-0.5203	0.403061	-1.28394	-0.08409	0.603325	-0.47671
Q8WXF7	ATL1	-0.47601	0.105159	-0.05842	-0.38362	-0.12821	-0.22215	0.566333	0.239193	0.286127	0.433583	-0.35976	-0.11306	-0.27992	-0.16383	-0.5375	-0.36806	-0.83931	-0.50267	1.628679
Q8WXG6	MADD	-0.4349	0.568433	0.065862	-0.12577	-0.18066	-0.15028	0.910266	0.013933	-0.22859	0.529288	0.159808	0.050193	-0.00925	0.326776	-0.1655	0.105556	-0.06994	-0.05638	-0.18615
Q8WXH0	SYNE2	0.610222	-0.47743	-0.36416	0.176382	-0.5072	-0.24284	-0.14324	-0.23667	-0.46195	-1.02446	1.292406	-1.09462	0.309343	-0.66107	-1.01515	0.440882	0.078448	1.010338	-0.53593
Q8WXI4	ACOT11	1.246185	-0.85851	-0.08546	-0.24908	-0.36073	0.198443	-0.29313	-0.05061	-0.55976	0.035807	0.035427	-0.06214	0.164106	0.949781	1.650704	-0.27459	1.143676	-1.26094	-0.89542
Q8WXI7	MUC16	-1.29712	0.489576	-0.2033	0.280742	-1.28154	-1.84556	0.08643	0.967766	-0.15347	-1.7227	-0.32413	-0.83977	-0.49327	-1.29666	-2.20118	1.328136	-1.15419	-1.15835	-1.65808
Q8WXI9	GATAD2B	0.390129	-0.71893	-0.05617	0.046972	-0.22054	0.415332	-0.08133	0.748668	-0.07106	-0.21498	-0.06083	-0.67067	-0.01617	-0.46325	1.04717	-0.12629	-0.81864	0.045151	-0.1876
Q8WXW3	PIBF1	0.021927	0.098614	-0.29128	-1.20411	0.3618	0.230427	-0.05429	-0.41404	-0.00453	-0.69804	0.890145	-0.52857	-0.02373	-0.14065	-0.52368	-0.2176	0.169437	0.082542	-0.30429
Q8WXX5	DNAJC9	1.260041	-0.34619	-0.02481	-0.34997	1.073298	1.514816	-0.38459	1.100639	-0.42567	-0.20784	-0.23574	-0.88412	0.06137	-0.70372	-0.18284	-0.01381	-0.21393	-0.22354	-0.48288
Q8WY22	BRI3BP	1.096899	0.455772	-0.00592	0.412624	-0.06913	-0.43058	-0.72048	-0.14737	-0.20987	-0.08807	0.501563	0.333767	-0.59792	0.979561	-0.51723	-0.81551	0.672559	1.089934	-0.90121
Q8WY36	BBX	0.31669	-0.904	-0.30178	-0.33488	-0.67495	0.568241	-0.23277	0.611435	-0.51486	0.039033	-0.30507	-0.56867	-0.50227	-0.15583	0.297304	-0.20469	-0.23911	0.15815	-0.35377
Q8WYA0	IFT81	0.368559	-0.56622	-0.55522	0.022748	-0.63415	-0.51014	-0.3747	0.166876	0.040847	-0.99247	1.420192	-0.83859	-0.23046	-0.61863	-0.34213	-0.52727	-0.38174	-0.65576	-0.02572
Q8WYA6	CTNNBL1	0.429281	0.071946	0.194419	-0.044	0.113762	0.355573	-0.51065	0.695981	0.091449	-0.21616	0.501419	-0.14755	-0.11419	-0.22421	0.103146	-0.14393	0.199153	0.212689	0.251694
Q8WYJ6	SEPT1	-1.73916	0.201929	0.287709	-1.22739	1.302071	0.786742	-0.06717	-2.46144	0.453739	0.679488	-1.55945	-0.83552	-0.5512	-0.2443	-0.69677	-1.55476	-0.5467	1.711615	-0.71662
Q8WYL5	SSH1	-0.36537	0.237044	0.141805	-0.08226	-0.29413	0.598425	0.621704	-0.91187	0.010822	0.065666	0.170825	0.488364	0.280651	0.050315	0.187894	-0.09087	0.089972	1.158289	0.399141
Q8WYP3	RIN2	-0.84836	0.284062	0.055733	0.18509	-0.37662	-0.62589	0.003122	-0.44122	0.184967	-0.9081	-0.64725	-0.30255	-0.11948	0.131072	-0.09931	-0.53432	-0.28221	-0.31151	0.108295
Q8WYP5	AHCTF1	0.350345	-0.42803	-0.29187	0.027684	-0.04428	0.164698	0.001073	0.25695	-0.44108	-0.0182	0.136302	-0.3233	-0.32753	-0.2769	0.878738	-0.4111	-0.09765	0.156277	0.055325
Q8WZ42	TTN	-2.6142	0.746732	-0.44102	-0.65048	-0.50058	-0.64429	0.042376	0.250075	0.533271	0.197397	-1.12419	-0.52579	0.952735	0.046243	-0.34953	-0.48317	0.313726	0.500602	0.014264
Q8WZ74	CTTNBP2	0.6471	-0.54118	0.204694	0.081647	0.388218	0.644524	-0.58938	1.477889	-0.48928	-0.40043	-0.08131	-0.90574	-0.09421	-0.31859	0.136436	0.357813	-0.07213	0.160686	-0.11989
Q8WZ82	OVCA2	1.354452	-0.38075	-0.02376	0.155592	0.269039	0.478278	0.025055	-0.13604	0.326618	0.471992	-0.14766	0.303289	0.058793	0.103526	-0.56598	-0.956	0.252763	0.896529	0.332585
Q8WZA0	LZIC	0.276282	-0.62688	-0.28013	-0.75592	0.675593	-0.16875	-0.45867	0.082183	-0.36144	0.246276	-0.06791	-0.38584	-0.52647	-0.57393	-0.43293	0.125155	0.255099	0.192091	-0.36853
Q8WZA1	POMGNT1	-0.23623	0.006131	-0.06113	0.242577	-0.10166	0.170533	0.10389	-0.38963	-0.19003	-0.22407	0.026038	-0.8297	-0.1829	-0.24561	1.128395	0.189562	0.441402	0.366943	-0.31379
Q8WZA9	IRGQ	-0.36364	-0.36627	-0.74232	-0.68623	-0.79272	-0.35511	-0.10388	0.873219	0.446478	-0.11877	-0.11344	-0.64262	-0.42696	-0.29817	0.04108	-0.69935	-0.13649	-0.51794	0.331904
Q92466	DDB2	-0.49562	0.052727	-0.6544	-0.39298	0.907214	0.641309	-0.46774	-0.19196	-0.13049	-1.06019	0.35702	-1.20725	-0.22934	-1.33643	-1.13128	-0.21859	-1.01819	0.202802	-0.87771
Q92485	SMPDL3B	1.485902	-1.06896	-1.76987	-0.21407	-1.30817	-0.43236	1.418772	2.099446	0.624858	-0.17869	-0.20336	-1.02538	-0.46036	-0.33985	0.548088	-0.62498	-1.11673	-1.81941	-0.18432
Q92499	DDX1	-0.02308	-0.44751	-0.18545	0.202673	-0.4397	-0.16462	-0.73261	0.022724	-0.29373	-0.25782	0.419226	-0.44598	-0.08494	-0.31454	0.611634	0.2826	0.037559	-0.03743	0.190774
Q92503	SEC14L1	-0.40463	0.57007	-0.11759	-0.1819	-0.27314	-0.6105	0.409593	-0.65929	-0.29478	0.229129	-0.30079	0.47191	-0.1195	0.777114	0.144604	-0.12578	-0.08507	-0.09351	0.161474
Q92504	SLC39A7	0.053593	0.227859	-0.13082	0.060773	0.113989	0.221138	1.651898	-0.21941	0.240247	-0.5044	-0.86161	-0.28902	0.406078	-0.8208	1.001176	0.843125	0.552047	0.736838	0.389725
Q92506	HSD17B8	0.369179	-0.22148	-0.78055	0.020351	0.592154	-0.10575	-0.2274	-1.81629	-0.29864	-1.19789	1.294039	-0.33385	-0.65781	-0.51426	-0.86192	1.333052	-1.02466	-0.96041	-1.35258
Q92508	PIEZO1	-0.60568	0.413453	0.014187	-0.25898	0.130765	-0.44327	0.328707	-0.7312	0.055391	0.154641	-0.68752	0.011487	0.228083	-0.16096	-0.27838	0.233632	0.17971	-0.2583	-0.32765
Q92520	FAM3C	-0.73943	0.644283	0.127642	0.230258	-0.24901	0.267285	0.480253	-0.18391	-0.05249	1.146179	-0.18441	-0.88753	-0.5154	-0.55035	0.830631	1.105075	0.018232	1.236291	0.666595
Q92522	H1FX	0.737998	0.566317	0.153478	-0.5824	-0.92107	0.891796	0.044349	0.990016	0.219561	-0.65846	-0.55066	-0.55838	-0.51155	-0.1599	-0.15699	-0.02636	-0.18906	-0.54826	-0.70298
Q92530	PSMF1	-0.02948	0.014069	-0.46679	-0.29652	-0.58546	-0.3924	-0.21758	0.37271	0.233263	0.38265	-0.07024	-0.87862	0.003935	-0.05922	-0.12275	-0.4171	-0.22161	0.031137	0.084755
Q92538	GBF1	-0.12652	0.657679	0.405461	-0.05926	-0.03969	0.156903	0.718639	-0.20187	-0.23114	0.170675	-0.25579	0.021524	0.156385	0.057404	-0.04063	-0.1138	0.292466	0.357632	0.601252
Q92539	LPIN2	-0.53237	0.015453	0.11977	0.137794	-0.18934	0.354198	-0.37301	0.162051	-0.30577	-0.15129	-0.17236	0.113088	-0.31242	0.358799	-0.61126	-0.40973	1.069137	0.619219	0.123089
Q92540	SMG7	-0.03828	0.02764	0.050555	-0.14313	-0.54015	-0.31837	0.030629	0.470596	-0.28877	0.041948	-0.25833	-0.26806	-0.36241	-0.16237	-0.14597	-0.02429	-0.29422	-0.10155	-0.01784
Q92541	RTF1	1.179182	-0.41815	0.402621	0.585535	-0.33851	-0.16068	-0.80422	0.215866	-0.35938	-0.03	0.310792	-0.61697	0.402812	-0.37782	-0.01714	0.662139	0.025222	0.594294	0.025013
Q92542	NCSTN	0.401653	0.469599	0.418384	0.362437	0.13693	0.092714	0.540482	0.623729	-0.20307	0.091788	-0.10277	0.271416	0.301518	0.405807	0.281781	0.02707	0.170271	0.254964	0.405897
Q92544	TM9SF4	0.342872	0.334624	0.535723	0.214663	0.098232	0.459348	1.142025	0.477613	0.08847	0.102742	-0.40804	0.132236	-0.04776	-0.13873	0.887892	0.685874	0.044175	0.236815	0.600522
Q92545	TMEM131	-0.08727	0.136875	0.062729	-0.47655	-0.40299	-0.30544	0.058634	0.375907	0.162578	-0.05767	0.293676	0.227153	-0.1531	0.402541	0.591033	0.142761	-0.0952	0.544731	0.075231
Q92547	TOPBP1	0.554083	-0.31938	0.237275	0.511266	1.158015	1.916171	0.24488	0.956828	-0.04817	-0.20731	0.089726	-0.41529	-0.14951	-0.58617	0.165453	0.217356	0.375437	-1.43982	0.207205
Q92551	IP6K1	0.083451	0.289002	0.262291	0.018166	0.503315	0.454961	0.030153	0.073963	0.037103	0.203341	-0.21128	0.637732	0.192742	0.369153	0.146932	-0.31666	0.138284	-0.02188	0.533241
Q92552	MRPS27	-0.33739	-0.07196	-0.4366	0.857325	-0.05076	0.125056	-0.1076	-0.05772	-0.1377	0.197111	0.458495	-0.09586	0.040859	-0.3228	1.208835	-0.08437	-0.2482	-0.16309	-0.07904
Q92556	ELMO1	-1.40515	1.066286	1.064915	-0.94374	0.283012	-0.10267	0.544424	-1.44356	-0.34079	0.768101	-0.39079	0.467029	-0.64037	1.149397	-0.16669	-0.07656	-0.4259	0.085418	-0.67332
Q92558	WASF1	-0.16462	0.116917	-0.02709	0.248349	-0.92634	-1.31801	-0.26929	-0.58067	0.498633	-0.10772	-0.31588	-0.37481	-0.25954	-0.3324	-1.32926	-0.68825	-0.07213	-0.69585	0.410003
Q92560	BAP1	0.331486	-0.05952	0.07784	-0.28454	0.005662	0.562024	-0.11829	-0.00224	-0.08754	0.800119	0.225721	-0.19154	0.006537	-0.16062	0.51755	0.354994	0.391071	-0.1405	-0.08302
Q92562	FIG4	-0.37675	0.1378	0.237369	0.220522	-0.64114	-0.11151	-0.12852	-0.18512	0.184918	0.045501	0.060794	-0.80434	0.01918	-0.22329	-0.35487	-0.30463	-0.52393	0.227398	-0.07902
Q92563	SPOCK2	-1.7584	-0.76588	-1.83807	-3.25346	-1.04538	-1.72344	-1.2572	0.050678	-0.14758	-1.76523	-2.29732	0.373242	-0.75461	-0.45614	-1.00394	-1.18368	-1.24523	-3.66716	-0.92697
Q92567	FAM168A	-0.47028	0.17678	-0.10743	-0.80318	-0.50061	0.341269	0.257995	-0.0948	0.094666	-0.25936	-0.33039	-1.16613	-0.08851	-0.43597	-0.19616	-0.41463	-0.07557	0.353683	0.06154
Q92572	AP3S1	-0.42263	0.898153	0.426385	-0.60675	-0.39788	-0.33401	0.65	-0.20864	0.077561	0.128774	0.104029	-0.52197	0.821862	-0.2351	0.306374	-0.16235	-0.41262	0.389345	0.414577
Q92574	TSC1	-0.14467	0.461191	-0.2766	-0.52898	0.079829	-0.49326	0.747442	0.347602	-0.03684	-0.18701	-0.45487	0.213482	-0.08698	0.125358	-0.21031	-0.55327	-0.59853	0.103523	0.604364
Q92575	UBXN4	0.80588	0.319581	-0.0425	-0.23274	-0.34778	-0.64991	0.363999	0.197246	-0.22701	-0.35889	0.012321	-1.07494	0.238473	-0.67448	0.249432	0.253048	-0.28812	0.477087	0.128587
Q92576	PHF3	1.340619	-0.02927	-0.18393	0.064207	-0.62414	-0.7579	0.008687	0.624667	-0.22819	-0.2367	0.200204	-0.64901	0.551706	-0.59273	1.010517	-0.18232	-0.15794	0.546976	-0.51991
Q92581	SLC9A6	-0.15266	0.554762	0.120504	0.121915	0.280034	0.018484	0.565841	-0.10554	0.250408	-0.00909	-0.48737	0.326843	-0.66507	0.217252	-0.30744	0.466064	-0.31548	0.326136	-0.40254
Q92597	NDRG1	-0.07004	-0.05934	-1.48594	0.358107	-1.05398	-0.69761	-0.71228	-1.3407	-1.67893	-1.23612	-1.2472	0.900605	0.734127	0.194951	1.14833	0.779885	1.234395	0.06617	-1.09897
Q92598	HSPH1	-0.46064	-0.42147	-0.50208	-0.3267	1.368803	1.475644	-0.83479	0.048875	0.300391	1.150735	0.808463	-0.20141	0.803862	-0.39003	-0.62944	-0.37149	0.055953	-0.34673	0.316318
Q92599	SEPT8	0.294715	-0.97752	-0.18021	-0.33349	-0.19406	-0.16621	-0.49947	0.869996	-0.35648	-0.35559	0.323727	-0.80979	0.266681	-0.87059	0.395301	-0.01524	-0.34558	-0.19772	0.131421
Q92600	CNOT9	0.334481	0.087531	-0.5232	-0.27922	-0.07793	0.148064	-0.14883	-0.01964	0.053226	-0.06164	0.651898	-0.05057	0.341123	0.183664	0.370214	0.366391	0.257195	0.350282	0.442978
Q92604	LPGAT1	-0.48386	0.515529	-0.06958	-0.13132	-0.1552	-0.37781	0.21069	-0.19924	-0.07185	0.341802	-0.14222	0.591033	-0.56236	0.829869	0.109572	0.142468	0.224314	-0.14186	-0.04231
Q92608	DOCK2	-1.87457	1.245154	1.032185	-0.79826	0.521331	0.109918	0.055587	-1.89045	0.310978	1.209044	-1.68241	1.12756	-0.27437	1.79771	-0.48575	-0.72886	0.146642	0.441114	-1.36695
Q92609	TBC1D5	0.876225	-0.0761	-0.2443	-0.36335	-0.37759	-0.34985	0.268786	-0.44137	0.398487	0.03976	0.229916	-0.21047	-0.0342	0.080311	0.242669	0.292072	-0.50592	-0.30243	-0.21584
Q92610	ZNF592	0.362536	0.19473	-0.10117	-0.32848	0.056391	0.035747	-0.45763	-0.53029	0.263895	-0.14482	-0.08703	-0.03854	-0.03056	0.030794	0.168268	-0.06244	0.085481	0.72457	-0.66084
Q92614	MYO18A	-1.05912	0.706205	0.705978	-0.59778	0.486894	0.351841	0.482845	-1.05331	-0.39822	0.170642	-0.24093	0.279367	0.198289	0.819713	-0.46761	0.185667	0.237465	0.26047	-0.29658
Q92615	LARP4B	0.48782	0.610255	-0.47202	0.147933	-0.07471	1.080998	-1.19822	0.168898	0.014569	0.183952	0.203589	0.013393	0.944077	-0.06863	0.199625	0.729877	0.784388	0.033574	-0.3647
Q92616	GCN1	0.385216	0.089262	0.005968	0.894575	-0.0096	0.34906	0.502602	0.143933	-0.08388	-0.32401	0.000236	-0.05539	0.07604	-0.28419	0.046849	-0.15928	0.094016	-0.27076	0.394628
Q92619	ARHGAP4	-1.91207	0.552901	1.473822	-0.75016	0.391058	0.227633	0.036158	-1.69477	-0.40554	1.39841	-1.2505	1.050061	-0.34783	1.574945	-0.45504	-0.29311	0.32417	0.749537	-0.91228
Q92620	DHX38	0.098207	0.114147	-0.30128	-0.1667	-0.08987	-0.00767	-0.55596	-0.13565	0.041509	0.071527	0.160759	-0.17705	-0.17269	0.20875	0.029452	0.522164	-0.05136	-0.0376	0.188349
Q92621	NUP205	0.052121	0.171077	-0.16054	0.229888	0.026615	0.129628	0.161145	0.35442	-0.66127	-0.16121	0.243	0.067431	-0.17817	0.071602	0.878627	-0.0397	0.016394	-0.35698	0.233956
Q92625	ANKS1A	0.050773	0.203739	0.467465	0.158238	-0.31652	-0.21327	-0.17258	-0.47101	0.083708	0.233235	0.143233	0.205298	0.075208	0.200983	0.127378	-0.16245	0.4088	-0.17046	0.157681
Q92626	PXDN	-1.20536	0.670734	0.570771	0.53917	-0.63721	-0.56732	0.886642	1.179793	0.435501	-0.13743	-0.67999	-1.0209	0.230852	-0.24672	-0.56276	0.231334	-0.6882	-0.29818	1.064857
Q92629	SGCD	-1.75096	0.158221	-1.32037	-0.79697	-1.16968	-1.46633	-0.40366	-1.27767	0.194031	-1.50352	-0.54685	-0.85182	-0.84432	-0.67271	-0.47672	-0.52453	-1.00188	0.363497	0.495929
Q92636	NSMAF	-0.08657	0.542805	0.335249	-0.54927	-0.05391	0.209777	0.368759	-0.3862	0.0898	0.296546	-0.52841	0.212419	0.257596	0.266739	0.012701	0.125304	-0.10331	0.275801	0.456784
Q92643	PIGK	0.10966	0.355587	0.104918	0.271892	-0.38642	-0.0216	0.564864	0.643883	0.189042	-0.06358	0.381723	-0.32079	-0.21713	-0.1428	0.238887	0.217015	0.290649	0.081517	0.326034
Q92665	MRPS31	-0.16692	0.209531	-0.18228	0.580364	0.201723	0.485884	-0.04358	0.001924	-0.04656	0.263421	0.097769	-0.21177	-0.12927	-0.49333	1.022247	-0.1252	-0.30104	-0.32003	-0.41648
Q92667	AKAP1	0.036268	-0.23815	-0.53093	0.37448	-0.79842	-0.50117	-0.28683	-0.65971	-0.12552	-0.37979	0.684069	-0.72795	-0.25772	-0.65469	0.66112	0.29436	-0.75647	-1.05056	-0.89874
Q92673	SORL1	-0.51835	0.048489	-0.65484	-0.04465	-0.59138	-0.58479	0.179219	0.190903	-1.03667	-0.2169	1.127519	-0.78883	-0.18508	-0.00612	0.421474	1.304646	-0.46063	-1.02355	-1.52144
Q92688	ANP32B	0.665312	-1.09055	-0.03504	0.326439	1.414764	1.480041	-1.12927	0.870478	-0.57443	0.535491	0.586657	-0.42301	-0.18821	-0.4882	-0.1245	0.61625	0.225053	0.179751	-0.24256
Q92692	NECTIN2	0.590397	-0.36849	0.406509	-0.41527	-0.15354	-0.43181	1.083324	0.772036	0.502623	0.232006	-0.45596	-0.2083	0.312878	-0.46909	0.536299	-0.74167	-0.10174	-0.03115	-0.10019
Q92696	RABGGTA	-0.03127	0.24796	0.047677	0.019299	-0.61353	-0.39521	-0.14998	0.048253	-0.21812	0.446063	0.01775	-0.23492	-0.54906	0.17832	0.062791	0.057065	-0.0554	-0.33473	-0.26299
Q92729	PTPRU	0.862872	-0.3115	-0.39441	-1.145	-1.63346	-1.19753	-0.04219	-0.4803	-0.36249	-1.13182	0.827868	-0.32778	-0.4141	-0.13038	0.779027	0.03587	-0.48249	-0.39647	-0.78465
Q92733	PRCC	0.690956	-0.50526	0.076079	0.069669	-0.5736	-0.24047	-1.13546	1.090441	-0.44983	-0.18176	0.782173	-0.67519	-0.19382	-0.14138	0.710693	0.06487	-0.02537	0.751266	-0.16495
Q92734	TFG	-0.10819	0.336239	0.157671	-0.22462	0.308523	0.240386	-0.36899	0.144512	0.515502	0.428554	-0.34708	-0.00963	0.615056	0.015932	0.910304	0.194997	-0.00164	0.304519	0.496766
Q92736	RYR2	-0.84683	-0.51295	-0.07706	-0.90985	-0.4128	-3.97314	0.014739	-2.68054	0.407504	0.577179	-1.59197	0.186408	-0.97006	0.668691	-0.38822	-2.52306	-0.66317	-1.5009	-2.14108
Q92738	USP6NL	0.195772	-0.12714	-0.34725	0.016264	-0.45486	-0.82584	-0.08745	0.245837	0.340758	0.77865	0.68993	-0.46967	-1.10888	-0.61491	0.130934	-0.40455	-0.23112	-0.65253	-0.27068
Q92743	HTRA1	-1.62969	-0.74085	-0.60077	-1.44999	-1.42593	-1.26712	-0.13551	-1.27756	1.472567	-1.33666	-1.08075	-0.4836	-0.0651	-0.2848	0.274023	-1.18784	-0.4892	-1.26954	-1.02367
Q92747	ARPC1A	0.114501	-0.44672	-0.67241	0.075914	-0.54805	-0.25151	-0.7762	0.626886	-0.46333	-1.07105	0.57674	-1.06791	0.090395	-0.69492	-0.40593	0.024737	-0.0991	-0.16806	0.650126
Q92759	GTF2H4	0.250079	0.059053	0.118111	-0.04403	0.693486	0.449281	-0.02715	0.087985	-0.15083	0.143572	0.347893	0.07922	0.311255	-0.08054	-0.09148	0.3473	0.535099	0.345825	0.208166
Q92766	RREB1	0.364521	-0.23962	-0.07824	0.015841	-0.32156	0.045007	-0.19526	0.115075	0.005446	-0.18221	0.221611	-0.69713	-0.15274	-0.52033	1.078703	-0.19226	-0.56869	-0.00996	-0.43743
Q92769	HDAC2	0.197119	0.226099	-0.17744	0.446679	-0.13073	0.405404	-0.17012	0.617003	-1.05163	-0.39646	-0.5083	-0.39139	-0.56536	-0.27289	0.825696	-0.11251	-0.23511	-0.10318	-0.02199
Q92783	STAM	0.152198	0.231955	0.065293	-0.47646	-0.32341	-0.43117	-0.21503	0.000338	-0.3227	0.277645	-0.39288	-0.25291	-0.02431	-0.20644	0.509651	0.109866	-0.43894	0.076405	0.606541
Q92785	DPF2	0.828031	-0.3462	0.095819	0.128002	0.243328	0.762375	-0.50894	0.606894	-0.03792	0.109019	-0.02715	-0.64037	0.042297	-0.07449	0.678413	-0.51697	-0.17518	0.408523	0.035232
Q92791	P3H4	-0.18506	0.789733	0.260598	-0.9651	-0.08535	0.019213	0.025861	-0.04203	0.672202	-0.40806	-0.52193	-0.50346	0.792028	-0.63373	-1.22922	0.371714	-0.48476	0.345793	1.744397
Q92793	CREBBP	0.379971	0.261623	0.218666	-0.20274	-0.416	-0.28344	-0.00233	-0.07113	0.146533	-0.21751	0.041556	-0.2766	-0.08123	-0.30208	-0.04284	-0.14901	-0.1234	0.557984	-0.14577
Q92794	KAT6A	0.513739	0.196131	-0.0798	-0.72253	0.34035	0.210405	0.204719	0.025448	-0.14981	0.278056	0.035167	0.138396	0.281313	0.201159	-0.53755	0.084013	0.082618	0.115325	-0.03019
Q92796	DLG3	0.13761	-0.24619	0.094805	0.202755	0.638531	0.6589	-0.42264	0.321182	-0.30687	0.216456	0.613167	-0.93591	-0.0975	-0.48811	0.618473	-0.00359	-0.28598	-0.07834	0.032192
Q92797	SYMPK	0.413725	0.316484	-0.11343	0.029547	0.162889	0.245143	-0.06069	0.291552	-0.60051	-0.08929	0.409285	-0.1324	-0.08154	0.108201	0.152934	-0.01649	-0.20587	0.209281	0.105409
Q92804	TAF15	0.648962	0.00273	0.595336	1.011471	0.396194	0.566629	-1.29796	0.116069	-0.4195	0.069036	1.346293	-1.06473	0.734487	-0.99736	0.041955	0.239288	-0.30078	1.449776	-0.09976
Q92805	GOLGA1	0.138053	0.526682	0.204949	-0.48815	0.229331	0.278272	0.239856	-0.06848	0.188017	-0.31724	0.04039	-0.21204	0.531603	-0.29156	0.08679	0.441666	-0.25866	0.646988	0.258554
Q92817	EVPL	0.580237	-0.71945	-1.24684	-0.19922	-1.84564	-0.97254	-0.82242	-0.08419	-0.45181	-1.92397	0.444898	-1.06264	-0.28832	-1.20709	-0.3615	0.334405	-0.07864	-1.44752	-1.78016
Q92820	GGH	0.362174	1.07987	-0.08612	-0.76356	1.964467	0.874323	0.777915	0.01175	0.048801	0.160819	-1.55674	1.463345	0.48616	0.897775	-0.62722	-0.78848	0.666639	1.203813	0.09633
Q92823	NRCAM	0.582327	-0.18289	-0.47631	-0.34971	-0.85258	-0.37466	-0.22564	-0.26187	0.206376	-0.4398	0.553571	-1.09552	-0.01466	-0.35727	-1.66743	0.255421	-0.83999	-1.03958	-1.27929
Q92828	CORO2A	-0.46119	-0.36329	-1.02916	0.373225	-0.27548	-0.25358	-0.33784	0.611735	-0.44932	-0.85833	-0.23232	-0.71035	-0.19776	-0.43883	0.284344	0.584484	-0.50348	-1.58604	-1.58649
Q92830	KAT2A	0.200007	0.07977	-0.1919	-0.12932	0.045252	0.528997	0.126834	0.494299	-0.11094	-0.78788	0.172516	-0.53094	-0.23312	-0.26447	-0.16135	-0.04658	-1.46195	0.098222	-0.42259
Q92835	INPP5D	-1.66241	0.843321	1.262314	-0.80959	1.072916	0.935631	-0.6712	-2.34419	0.357274	0.982726	-1.3058	1.153977	0.099706	1.697184	-0.23901	-0.58563	0.299861	0.511148	-1.40448
Q92841	DDX17	0.420697	-0.0879	-0.25981	0.385848	0.385793	0.438091	-0.45616	0.42875	-0.22968	-0.227	0.334421	-0.06862	-0.20154	-0.12536	0.154434	-0.1322	0.32271	0.65965	-0.26331
Q92844	TANK	1.534475	0.14836	0.174216	-0.65559	-1.08673	-1.02293	0.204744	-0.3212	-0.10202	0.368017	-0.24559	-0.24758	0.158313	0.237319	-0.44425	-0.55226	-0.32013	-0.69362	0.039165
Q92845	KIFAP3	-0.22658	0.098242	-0.06752	0.026838	-0.23551	0.14215	0.278263	-0.248	-0.12909	-0.21852	0.324666	-0.04659	0.047022	0.216632	1.200002	0.100905	-0.01254	-0.06441	0.060937
Q92851	CASP10	-0.57576	0.549724	1.174568	-0.33514	0.08824	-0.06256	-0.04793	0.558268	0.182829	1.007817	-0.32903	0.639842	0.222501	1.165554	-0.43111	-0.1199	0.491283	0.05197	-0.72079

Q92854	SEMA4D	0.338529	0.003736	0.262968	-0.79833	0.911609	0.731314	-0.36281	-0.0384	-0.10581	0.216628	0.263977	-0.08097	-0.17948	0.400672	-0.22325	0.158817	0.341828	0.1802	-0.86044
Q92859	NEO1	0.004269	-0.05056	-0.019	-0.62114	-0.63112	-0.41694	0.136936	0.892448	0.040847	-0.65737	-0.63324	-0.7522	-0.32939	-0.4546	-0.84656	-0.58965	-0.35801	-0.44654	-0.31124
Q92870	APBB2	0.108654	-0.06032	-0.06012	-0.26157	-0.08356	-0.21392	-0.09728	-0.25258	-0.01057	-0.27059	0.460505	-0.62821	-0.1194	-0.65294	0.309598	0.210632	-0.73707	-0.32189	0.349146
Q92878	RAD50	-0.08607	0.006024	-0.29268	0.228478	0.409942	0.758277	-0.18071	0.521459	-1.26802	-1.87679	0.687984	-0.05194	0.182166	-0.12048	0.366995	0.875289	-0.03985	0.09983	0.04286
Q92879	CELF1	0.683965	-0.16353	-0.04214	-0.12016	0.321022	0.323578	-0.10916	0.941421	-0.27608	0.046026	0.264796	-0.09677	-0.41253	0.191465	-0.42093	-0.40925	0.19988	0.024676	-0.23855
Q92882	OSTF1	-0.65156	1.147207	0.963269	-0.57421	0.716103	0.62542	-0.2834	-0.58683	0.318907	1.531818	-0.77375	1.112618	0.426844	2.155408	-0.49173	-0.18035	0.384378	0.284481	-0.38318
Q92888	ARHGEF1	-0.33539	0.568641	0.716024	-0.62453	0.104417	-0.01986	0.686989	0.663735	-0.03968	0.488467	-0.40694	0.40771	0.259551	0.926428	0.192142	-0.49514	0.00933	0.21928	-0.23126
Q92889	ERCC4	0.280508	0.4685	-0.4546	-0.36458	0.600544	0.485389	-0.22773	0.034562	-0.1293	-0.13624	0.302083	-0.00236	0.741344	-0.05174	-0.06954	0.076473	-0.28764	-0.17336	-0.55586
Q92890	UFD1	0.314494	-0.34645	-0.23421	-0.23187	0.106197	-0.20577	-0.31122	-0.20695	0.236107	0.170954	-0.24859	-0.32281	0.073994	-0.50948	-0.35545	0.147464	-0.12421	0.149143	0.012301
Q92896	GLG1	-0.85152	0.737327	0.378589	-0.35736	0.494086	0.415908	0.54589	-0.22408	-0.08253	0.232576	0.082731	-0.89261	0.205286	-0.46246	0.131326	-0.03935	-0.6401	0.601889	1.05368
Q92900	UPF1	0.248562	-0.18319	0.379951	0.148414	-0.30735	0.124623	-0.14727	0.050277	0.35004	-0.23262	0.028056	-0.27324	-0.3708	-0.05695	-0.39386	0.344314	0.116359	-0.34187	-0.26315
Q92905	COPS5	0.089182	-0.07433	-0.16827	-0.22821	-0.07586	-0.11644	-0.18068	-0.19455	-0.15289	0.079583	-0.05794	-0.27195	-0.18072	-0.24668	-0.03278	0.183766	-0.26498	-0.29342	0.061119
Q92917	GPKOW	0.149724	0.080187	0.416829	-0.67044	-0.24231	-0.3331	0.217413	0.071898	-0.35316	0.921301	-0.02363	-0.11442	-0.12981	-0.2509	-0.53243	0.089521	-0.08643	-0.32024	-0.29263
Q92922	SMARCC1	1.259068	-0.63682	-0.06783	-0.6122	-0.69557	-0.39131	-0.14067	0.503691	-0.40485	-0.48036	-0.27961	-0.80117	-0.07765	-0.52567	0.428867	-0.7104	-0.82256	-0.51156	-0.00933
Q92925	SMARCD2	0.628965	0.072353	0.000776	0.482527	0.006915	0.252154	-0.3941	0.027756	-0.18684	0.024796	0.11994	-0.22203	-0.10449	-0.3176	0.711472	-0.23171	-0.135	0.094778	-0.65051
Q92930	RAB8B	-0.69144	0.98582	0.794771	-0.27532	0.255198	0.032452	0.558389	-1.08071	-0.04299	0.59691	-0.83132	1.264925	0.347901	1.343655	-0.66769	-0.23625	0.538059	0.177585	-0.30616
Q92934	BAD	0.48647	-0.24849	-0.31211	0.473716	-0.7148	0.028665	-0.08871	1.351404	-0.10766	-0.25828	0.550509	-0.77249	-0.03315	-0.37455	0.37659	1.053627	-1.38322	-0.83107	-0.06738
Q92945	KHSRP	0.161482	-0.10868	-0.36212	-0.11999	-0.09072	0.03631	-0.8351	0.155834	-0.51334	-0.01663	0.602528	-0.66783	-0.21665	-0.84033	-0.22957	-0.7658	-0.68065	0.008784	-0.40641
Q92947	GCDH	-0.51223	-0.56584	-0.28283	0.400277	-0.0452	-0.25144	-0.22283	1.540181	0.243204	0.121982	-0.49302	-0.42848	-1.35227	-0.24336	2.633578	1.112692	-0.54469	-0.23663	-0.82962
Q92954	PRG4	-0.94767	0.456833	0.326499	-1.10325	-1.01542	-1.15106	-0.04672	-1.46025	0.335995	-0.34759	-0.90086	0.641008	-0.30242	0.978024	-0.66406	-0.75812	1.102429	0.18484	-1.51252
Q92963	RIT1	-0.21154	0.183818	0.521621	-0.23659	-0.12982	-0.035	0.02026	1.020617	0.096901	0.382879	-1.27739	1.040732	0.121854	1.317088	-0.17987	0.041731	0.355173	-0.21144	-0.15025
Q92968	PEX13	-0.10512	0.779837	-0.0314	-0.32805	-0.23871	0.124963	0.510532	-0.27381	0.588741	-0.23263	-0.58997	-0.16968	0.296785	0.081403	-0.0691	0.068042	-0.15549	-0.99676	-0.47888
Q92973	TNPO1	-0.12081	0.147486	-0.14869	0.125938	-0.03404	0.394322	0.974674	0.31581	0.008182	-0.34826	0.190947	-0.05042	-0.00512	0.113238	0.418913	0.109602	0.114492	0.057357	0.342837
Q92974	ARHGEF2	-0.05261	0.444884	0.130789	-0.57393	-0.06394	0.111311	0.411233	0.19782	-0.12969	0.484251	-0.15236	0.348954	0.131215	0.52044	1.19193	-0.01817	-0.14639	-0.36058	-0.49496
Q92979	EMG1	0.214342	0.250422	0.070185	0.798106	-0.08321	-0.28442	0.053381	-0.02256	0.158863	-0.00507	-0.08493	0.034659	0.103555	-0.04503	-0.18332	-0.20513	0.215513	0.112185	-0.35811
Q92989	CLP1	1.04992	-0.17611	0.026009	0.187723	0.173955	0.61462	-0.36796	0.383568	-0.27968	-0.35234	0.568681	-0.27343	-0.27106	-0.12765	0.35651	0.157127	-0.24656	-0.08948	-0.31759
Q92990	GLMN	0.661673	-0.17342	-0.05986	0.331622	0.004449	-0.00219	-0.16588	0.527385	-0.60231	-0.44798	-0.09092	-0.27786	-0.32249	-0.16004	-0.6714	0.395901	0.523966	-0.11314	0.398338
Q92993	KAT5	0.581062	-0.10285	-0.30225	-0.06993	-0.25142	0.371256	0.122222	0.572631	-0.22434	-0.11903	0.332718	0.272116	-0.16617	0.41697	-0.01013	0.000956	1.167371	-0.07863	0.203949
Q92994	BRF1	0.277718	0.437012	-1.32129	-2.04746	-0.38229	-1.00694	-0.45185	0.22	-0.21741	-1.24094	-0.66414	2.08223	3.265755	2.514567	-1.39228	-1.28217	2.652994	-0.19366	-0.96835
Q92995	USP13	0.402399	0.014379	-0.08553	-0.12972	0.835049	0.625069	-0.26023	-0.12741	0.384369	0.902613	0.284236	0.029629	0.213846	-0.35677	-0.6031	0.061332	0.285872	0.032404	-0.2849
Q92997	DVL3	0.197347	0.175546	-0.04316	-0.49673	-0.48947	-0.37168	-0.55692	-0.10726	0.726362	0.313282	0.009464	-0.18463	-0.09119	-0.14616	0.437355	0.110371	0.066859	0.040323	0.434253
Q93008	USP9X	-0.12872	0.303528	0.435588	-1.13077	-0.11289	0.02003	0.054979	-0.08105	-0.01959	0.004505	0.071093	0.09645	0.128421	0.17146	-0.24657	0.386968	0.138294	-0.28543	0.155829
Q93009	USP7	0.478906	-0.24688	0.194732	-0.13758	-0.19976	-0.28164	-0.73598	0.218012	-0.41229	-0.29501	0.399434	0.115347	0.453228	0.02052	0.259095	0.237621	0.26753	0.174304	-0.19046
Q93034	CUL5	0.157174	-0.30617	-0.26122	-0.00249	-0.52823	-0.50587	-0.11016	-0.3866	-0.18192	-0.37486	0.447565	-0.45786	0.198214	-0.59219	0.17216	0.01757	-0.43886	-0.26337	-0.08987
Q93050	ATP6V0A1	-0.54911	0.57814	0.57279	-0.2252	-0.14327	-0.30911	0.511038	-0.75457	0.243582	0.260163	-0.25941	0.827276	0.314792	0.809472	0.149029	-0.21022	0.87202	0.651843	-0.49518
Q93052	LPP	0.037405	-0.90933	-0.46139	-0.21632	-1.00304	-0.90854	-0.70781	-1.01122	0.247852	-0.46068	-0.59418	-0.87216	0.497831	-0.86275	0.831508	0.34432	-0.81475	-0.17139	0.261871
Q93062	RBPMS	0.714733	-0.24486	0.196142	-1.57178	0.079041	0.923406	0.110269	-1.08816	0.279176	-1.22948	-0.70433	-1.76195	-0.28345	-1.35767	-0.9815	0.329172	0.531322	0.381397	-0.11946
Q93063	EXT2	-0.0222	0.068286	-0.68581	-0.18047	0.046603	-0.16722	0.458475	0.494563	0.25641	-0.18497	0.048775	-0.87941	0.512608	-0.72394	-0.51211	-0.30818	0.3198	1.325133	0.487403
Q93074	MED12	0.324976	-0.04355	-0.02291	-0.08028	0.134561	0.139384	0.197273	0.278716	-0.20393	-0.34761	0.118347	-0.45603	-0.01738	-0.49123	0.245677	-0.17654	-0.62426	-0.1861	-0.06709
Q93084	ATP2A3	-1.12205	0.755021	0.469724	-1.5485	0.215588	-0.54413	0.743446	-1.60587	0.444301	0.283151	-1.86935	-0.03507	-0.83612	1.681337	-1.13502	-1.46827	0.116944	1.1233	-1.02843
Q93100	PHKB	-0.57459	-0.55561	0.042092	0.221472	-0.5718	-0.37313	0.001521	-0.73933	0.128037	-0.45604	0.182667	0.173954	-0.08101	0.992016	-0.5542	-0.01083	0.164915	-0.99555	-0.32628
Q969E2	SCAMP4	-0.67897	0.356528	0.132291	-0.18828	-0.21659	-0.20225	0.211254	-0.72146	0.007071	-0.35374	0.857664	0.400664	-0.423	-0.19812	-0.31192	-0.3915	-0.06959	-0.45271	-0.34932
Q969E8	TSR2	0.579962	0.12985	0.440432	-0.83475	0.373712	-0.17906	-0.07986	0.347974	-0.1788	-0.20606	-0.03413	-0.19437	-0.18131	0.035266	0.019694	-0.61875	0.609132	-0.23318	-0.69749
Q969F1	GTF3C6	0.376409	0.367895	-0.10941	0.283264	0.128281	0.21537	0.188772	0.495797	-0.07289	0.188538	0.135604	0.295476	0.139856	0.270566	0.181197	-0.97289	0.723945	0.05375	1.151813
Q969F9	HPS3	-0.0692	0.660474	0.29784	-0.25106	0.524557	0.824297	0.400184	-0.4622	0.027473	0.311051	-0.52551	0.548779	0.289241	0.278887	0.158994	-0.1119	0.184597	-0.90591	0.191516
Q969G3	SMARCE1	0.690244	-0.30416	0.067713	-0.13413	0.273528	0.191319	-0.07506	0.756777	-0.01089	0.27352	0.060501	-0.37194	-0.46771	-0.34443	0.639103	-0.42904	-0.28156	-0.05668	-0.25573
Q969G5	CAVIN3	-1.82321	-0.36251	-0.91298	-1.49102	-1.11921	-1.45169	-0.53399	-1.5644	0.491665	-0.9493	-1.4274	-1.66774	-0.64894	-0.9377	-0.09762	-0.54259	-1.65984	-0.08947	-0.28185
Q969H4	CNKSR1	0.132397	-1.45861	0.030849	0.517911	0.225901	0.629435	-0.36864	0.70683	-0.09313	0.343509	-0.07253	-0.20625	0.556784	-0.1202	1.39287	0.280305	-0.12488	-0.00466	-1.33321
Q969H8	MYDGF	-0.04131	-1.30385	0.605673	-0.19809	1.594819	-0.05995	-0.13596	-1.81711	1.612726	2.243144	-0.76397	1.43248	0.44649	0.732927	-0.64467	-0.04951	-0.09205	0.383991	0.090386
Q969J3	BORCS5	0.723353	0.034304	-1.83262	-0.54071	0.021043	-0.21966	-0.12659	-0.2279	0.820846	0.696323	-0.25857	-0.3782	1.32882	0.35823	-0.17235	0.057111	0.072993	-0.0542	-0.44019
Q969M1	TOMM40L	0.076389	-0.0289	0.092325	0.015833	0.552821	0.727455	-0.11022	-0.78191	-0.25532	-0.2026	-0.14809	-0.00862	0.044819	0.15256	2.203471	0.276895	-0.4917	-0.66002	0.089207
Q969M7	UBE2F	0.232732	-0.46411	-0.39038	-0.47682	-0.59945	-0.44863	-0.42051	0.502068	0.29635	-0.72386	0.248698	-0.40628	-0.08582	-0.33431	0.692262	-0.01777	-0.93867	0.284775	0.584532
Q969N2	PIGT	0.040707	0.373177	0.505777	0.320243	0.097481	0.046549	0.867722	0.751351	-0.2213	0.112609	0.060412	-0.2469	0.122712	0.172824	0.338045	0.199801	0.201823	0.1726	-0.0564
Q969P0	IGSF8	-0.30732	-0.99647	-0.64269	-0.79034	-0.84479	-1.1726	0.390777	0.390556	0.149038	-0.93538	-0.50117	-0.63339	-0.71788	-0.22455	1.021447	-0.41675	-1.0282	-0.46734	-1.20565
Q969Q5	RAB24	-0.30206	0.731007	0.450087	-0.35449	0.553364	0.531146	0.217994	-1.19123	0.036141	0.267828	-0.29413	0.620935	0.262333	1.258814	0.170275	1.406431	0.576627	0.35854	-0.62595
Q969R5	L3MBTL2	-0.05256	-0.19831	0.006665	0.327845	0.196521	0.171726	-0.34841	-0.01523	-0.00989	0.064456	0.057161	-0.11031	-0.03782	0.280421	0.380719	0.200536	0.421554	-0.36668	-0.77993
Q969R8	ITFG2	-0.11184	0.454263	0.01977	-0.41138	-0.10938	-0.20169	0.389489	0.084503	0.285797	0.156038	-0.78078	-0.0579	-0.14857	-0.07479	0.329479	0.083077	-0.51291	-0.67983	0.032695
Q969S2	NEIL2	0.336365	-0.07945	-0.41338	-0.67337	-0.68151	-0.63128	-0.04838	-0.30224	-0.39402	-0.36383	2.355774	-0.91769	-0.29867	-1.9729	-1.69491	-0.67933	-1.25913	0.092466	-0.64073
Q969S3	ZNF622	-0.29373	-0.6901	-0.00306	0.235721	-0.31235	-0.2029	-0.36687	0.042663	-0.26319	0.073654	-0.03659	-0.46619	-0.4383	-0.44863	0.788294	0.087855	0.242788	-0.16302	-0.11005
Q969S8	HDAC10	0.158314	0.436497	-0.12721	-0.17197	-0.34306	0.096471	-0.01804	-0.65984	0.050829	0.271914	-0.23049	0.548982	-0.20313	0.342991	0.109789	0.39789	-0.43063	-0.17799	-0.11534
Q969S9	GFM2	-0.65827	0.204519	0.021332	0.839902	-0.66946	-0.88605	-1.12513	-0.35244	-0.24378	0.017152	1.488767	-0.15818	-0.15506	0.27261	1.302418	0.610663	-0.08962	-0.05728	-0.68228
Q969V3	NCLN	-0.38381	0.449219	0.543098	0.155793	-0.02728	-0.07877	1.101214	-0.4276	0.275892	-0.00797	0.133405	0.097424	-0.0325	0.172588	0.195365	0.067013	0.131764	0.237426	0.03287
Q969V5	MUL1	-0.05148	-0.18162	0.122734	-0.39371	-0.04295	0.358033	-0.35451	0.662641	0.027495	0.20178	0.15482	0.042826	-0.19901	-0.00761	-0.15592	0.849289	-0.26978	0.300625	-0.12824
Q969X1	TMBIM1	-0.01521	0.493138	0.38441	-0.46272	-0.49875	-0.29656	0.348352	-0.31139	0.162055	-0.97122	-0.4797	0.378017	-0.47219	0.772451	0.509372	-0.36455	0.016524	0.505094	-0.45818
Q969X5	ERGIC1	-0.68659	-0.66407	-0.24328	1.478686	-0.28422	0.07805	0.20778	-0.26636	-0.51203	-1.04206	0.61441	-0.3909	-0.41141	-0.16329	1.1907	0.000613	-0.13826	-0.27481	0.027201
Q969X6	UTP4	0.385843	0.012872	-0.19478	0.886009	0.145081	0.036441	0.184402	0.398825	-0.19996	-0.2728	0.732994	0.118331	-0.12808	-0.15376	0.335577	0.238781	0.038609	-0.50774	-0.2507
Q969Y2	GTPBP3	-0.49173	0.223615	-0.44992	-0.72359	-0.46931	-0.66077	-0.3517	-1.2415	0.041309	-0.02825	0.848493	-0.1787	0.187048	-0.10428	0.429401	1.535851	-0.12542	-0.22286	1.077081
Q969Z0	TBRG4	0.120112	-0.13236	-0.07563	0.013519	0.540003	0.974836	-0.10528	0.362182	-1.31119	0.315814	0.641423	-1E-04	-0.12645	-0.48215	0.230498	0.708156	0.853321	0.193221	-0.55495
Q969Z3	MARC2	-0.21978	-0.09028	-0.39319	0.95056	0.19266	-0.25106	-0.25492	-1.12964	-0.21377	-0.79484	1.058112	-0.92448	-0.40183	-0.24197	0.117253	0.692833	-0.41911	-0.64233	-1.19549
Q96A00	PPP1R14A	0.141657	-0.17789	-0.11638	-0.70231	-0.47995	0.493037	-0.03791	-0.94274	0.289633	-0.17307	0.529718	-1.83387	-0.32379	-0.71218	0.002096	0.075607	0.368849	0.051456	0.584468
Q96A19	CCDC102A	-0.30552	-0.2232	-0.15591	-0.7036	-0.54432	0.054867	-0.73417	-0.49448	0.239506	-1.59689	-0.10749	-1.38236	-0.12384	-0.61872	-0.26782	-0.00125	-0.45498	-0.25316	0.335833
Q96A26	FAM162A	0.025428	-0.11787	-0.17237	0.499023	-0.336	-0.38662	-0.76764	0.050165	-0.75389	0.278244	0.233511	-0.05012	0.096553	0.099409	1.784971	0.609479	0.145666	-0.51979	-0.2149
Q96A33	CCDC47	-0.35697	0.424559	-0.02078	0.825061	-0.01968	-0.119	0.053998	-0.34395	-0.44881	-0.157	0.325002	0.382079	0.325621	0.012072	0.743945	0.634199	0.729176	0.435373	-0.20949
Q96A35	MRPL24	-0.21009	-0.01751	0.015621	0.804328	0.295768	0.263499	0.468929	-0.28267	-0.3457	0.263979	0.363683	-0.04844	0.001933	-0.31039	1.351243	0.11179	-0.36402	0.148931	-0.65392
Q96A49	SYAP1	0.033272	-0.59551	-0.06576	-0.16749	0.403918	0.223575	-0.14625	-0.13735	-0.37271	0.369462	0.211226	-0.54699	0.009907	-0.55208	-0.55211	0.233915	1.272668	2.319963	-0.08252
Q96A57	TMEM230	0.580588	-0.35329	0.086552	-0.10635	-0.00523	-0.10006	-0.0279	0.590783	0.2137	0.482396	-0.31379	0.189392	-0.28488	-0.48809	0.257651	-0.43248	-0.11037	-0.26074	0.328756
Q96A58	RERG	0.717061	0.10134	0.071812	-0.40531	-0.32297	-1.47082	0.334069	-0.91452	0.128478	-0.05224	-0.34089	-0.07489	-0.06156	0.01986	-0.28843	-0.42827	-0.93454	-0.4465	0.170434
Q96A65	EXOC4	-0.18362	0.308759	0.243117	0.071987	-0.09929	-0.15056	0.302765	-0.04419	-0.24486	-0.07128	0.226312	0.005919	0.188824	0.256629	0.409347	-0.05294	-0.37907	-0.52204	0.246321
Q96A73	KIAA1191	-0.16167	0.26799	-0.12166	-0.23785	0.462705	-0.83278	0.07498	-0.66302	-0.03132	-0.53725	0.685404	-0.04389	-0.06772	-0.075	-0.3955	0.200946	-0.16396	-0.18275	-0.60375
Q96A83	COL26A1	-1.25797	-0.73301	-2.30897	-4.19545	-1.65735	-3.53915	1.100343	1.757528	0.102851	-2.83549	-3.05139	-1.53749	-1.02783	0.401341	-0.82521	-2.26885	-0.44928	0.738083	0.419792
Q96AA3	RFT1	0.102973	-0.14837	-0.33208	-0.49886	-0.04059	-0.31021	0.00548	0.504719	1.643862	-0.39827	0.227328	0.043691	-0.08105	0.306341	-0.48948	-0.33813	0.312642	0.022486	0.108865
Q96AB3	ISOC2	-0.21476	-0.34231	-0.15594	0.060245	0.112944	0.106261	-0.08251	0.332819	-0.08936	0.19671	-0.25645	-0.01155	-0.04399	0.011587	1.074581	0.510067	0.489483	0.625267	-1.08472
Q96AB6	NTAN1	-0.17582	0.240287	-0.14016	-0.15422	0.946402	-0.15714	-0.24777	0.047719	0.160019	0.206807	-0.58142	0.22146	0.116948	0.567686	-0.20149	-0.21385	0.085435	0.014581	-0.14693
Q96AC1	FERMT2	-0.63168	0.029644	-0.24498	-1.11442	-0.54763	-0.56332	-0.08302	-0.62277	0.252379	-0.55116	-0.80725	-1.34963	-0.01076	-0.9933	0.007139	0.013165	-0.65197	0.756328	1.166483
Q96AD5	PNPLA2	-0.48058	0.205077	0.278438	-0.4236	-0.01871	0.162616	0.291377	-0.05844	0.074867	0.031879	-0.82625	0.22496	0.066768	0.205924	0.222516	-0.11359	0.008061	-0.03363	-0.99824
Q96AE4	FUBP1	0.983741	-0.69062	-0.12352	0.041292	0.531546	0.735477	-0.60033	0.621918	-0.7397	-0.33687	-0.00617	-0.31414	-0.35604	-0.57016	-0.19081	-0.08737	-0.00553	0.329531	-0.34005
Q96AE7	TTC17	-0.05008	-0.19882	-0.30171	-0.17471	-0.24191	-0.34422	0.151804	0.963703	0.180735	0.165719	-0.23656	-0.72954	-0.19317	-0.31655	0.620656	-0.36831	0.146243	0.258841	0.523526
Q96AG3	SLC25A46	-0.48803	0.062932	-0.16249	-0.01943	-0.71143	-0.42594	0.649299	-0.87823	0.096225	-0.6326	0.917443	-0.45814	-0.91024	-0.19099	0.492331	0.88958	-0.35061	-0.0397	0.296561
Q96AG4	LRRC59	-0.84354	0.602909	0.386669	0.263994	-0.13212	0.238187	0.676877	0.027236	-0.06283	0.109645	-0.22589	-0.75374	0.232464	-1.26836	-0.67659	0.628923	0.565463	0.815375	1.075427
Q96AJ1	CLUAP1	-0.12012	0.184832	0.0812	-0.55328	-0.52453	-0.13152	-0.14053	-0.16264	0.040847	-0.71296	-0.08753	-0.12201	0.217545	0.235099	0.253636	0.236681	-0.50271	-0.18743	-0.39802
Q96AJ9	VTI1A	-0.66592	0.15395	0.751629	-0.31347	-0.00314	0.081661	0.466957	-0.67689	-0.07491	-0.03445	-0.42703	0.003689	0.581557	0.004053	0.630009	0.339724	0.03449	0.109324	-0.40163
Q96AN5	TMEM143	-0.0263	-0.02893	-0.11221	0.442004	0.032843	-0.08569	0.011285	-0.42705	-0.42426	-0.28535	0.547082	-0.23448	-0.28379	0.135094	1.326731	-0.42666	-0.13261	-0.25267	-1.48945
Q96AQ6	PBXIP1	-0.69535	0.09885	-0.70054	-0.01379	-0.74989	-0.2579	-0.00503	0.402866	0.10804	0.884458	-0.72627	-0.90192	-0.22681	-0.65829	0.555264	-1.03549	-0.09647	-0.93057	0.162092
Q96AQ8	MCUR1	0.29061	-0.2601	0.405367	0.857821	-0.03096	-0.1712	0.202834	-1.40813	-0.23822	-0.21283	0.056627	-0.54078	-0.311	-0.3268	1.70844	2.580869	-0.71579	-0.31889	-0.70621
Q96AX1	VPS33A	-0.14787	0.655292	0.380525	0.164431	0.057347	-0.04353	0.159261	-0.10122	-0.44053	0.286993	-0.1667	0.163028	0.128896	0.286116	-0.17682	-0.08998	-0.14107	-0.10665	0.290076
Q96AX9	MIB2	0.105244	-0.35341	0.019901	-0.05802	0.56801	0.154057	-0.39239	0.506656	0.136163	0.553336	0.358208	-0.27742	-0.03082	0.346058	0.341857	0.887461	-0.07844	0.036168	-0.42804
Q96AY3	FKBP10	-0.36991	-0.75496	-0.37233	0.049276	0.347033	-0.16267	-0.99392	0.531383	0.781134	1.33874	-0.22352	-0.91492	1.410016	-0.58705	-1.02104	0.679285	0.482082	1.230704	1.896473
Q96AY4	TTC28	-0.33695	0.111455	-0.04315	-0.385	-0.67122	-0.47002	-0.09856	-0.39817	0.294429	-0.34337	0.436413	-0.85089	0.023019	-0.57234	-0.05228	-0.21516	-0.51511	0.107753	0.285357
Q96AZ6	ISG20	-1.40433	0.550015	0.024995	-0.68392	0.100885	-0.07783	0.286905	0.567269	0.504115	-0.27168	-0.87915	-0.84789	-0.18345	0.041618	-1.07914	-2.02371	-0.24462	0.438148	-1.28518
Q96B23	C18orf25	-0.38238	-0.33822	0.760944	0.209303	-0.74911	0.212652	-0.36574	0.050743	-0.27457	-0.4428	0.058786	-0.7731	1.809538	0.061734	-0.13106	1.47458	-0.94501	-0.19438	0.281981
Q96B26	EXOSC8	1.166342	-0.52875	0.113001	-0.18592	0.793315	0.610001	-0.51909	0.450247	-0.28176	0.479085	0.521857	-0.47887	-0.22426	-0.07572	-0.23178	0.334218	0.283219	0.643477	-0.48701
Q96B36	AKT1S1	0.574086	-0.02696	0.0874	-0.11593	0.06629	0.245666	-0.06727	-0.10703	0.005036	-0.33911	-0.24953	-0.61437	-0.25085	-0.83831	0.420871	0.208206	-0.00182	0.317054	-0.03881
Q96B49	TOMM6	0.239527	-0.56587	-0.47592	0.026857	-0.61817	-0.20058	-1.18862	-0.25518	0.826653	-0.41265	0.024465	0.117311	0.576189	-0.0807	0.78508	-0.00343	0.199761	-0.03153	-0.07213
Q96B70	LENG9	-0.39781	0.268641	-1.01259	-0.16995	-0.30753	-0.39371	-0.04741	-0.58097	0.040847	-0.23592	1.183385	-0.06072	0.113373	-0.37653	-0.93035	-1.15169	-0.65589	0.012929	0.484111
Q96B97	SH3KBP1	-0.86826	0.139286	-0.02981	-1.10401	-0.50229	-0.12811	-1.03689	-1.53193	-0.28973	0.855373	-0.68604	-0.3513	-0.50405	0.545393	-0.45332	-0.7428	-0.02389	0.750612	-0.21756
Q96BD5	PHF21A	0.477998	-0.33055	0.047949	-0.00088	-0.78931	-0.29062	-0.15396	0.299083	0.197382	-0.07245	0.009469	-0.26308	0.022435	0.519033	-0.28844	0.047135	-0.00615	-0.13256	0.119603
Q96BH1	RNF25	-0.00057	0.162746	0.148494	-1.24678	0.024428	0.228037	0.19686	0.574227	0.064127	0.020151	-0.11455	-0.11494	-0.22635	-0.31241	0.360257	0.312406	0.107109	0.099641	-0.10629
Q96BJ3	AIDA	0.176478	-0.48507	0.218621	-0.71228	-0.1913	-0.48626	-0.33873	0.135558	0.694947	0.01558	-0.4314	-0.53805	0.498157	-0.59398	0.632027	0.371293	-0.55981	0.447139	0.461452
Q96BJ8	ELMO3	0.531726	-0.22914	-1.01029	0.198543	-0.26268	0.088979	0.072928	-0.49759	-0.51797	0.223051	1.257854	-0.51824	-0.56143	-0.2049	0.661709	-0.02651	0.504652	-0.88787	-0.52437
Q96BM9	ARL8A	-0.20049	-0.18011	0.584319	-0.72574	-0.04319	-0.29086	0.223204	0.487683	-0.18627	0.057402	-0.35726	0.855036	-0.45176	1.067283	-0.06043	-0.51985	-0.2054	-0.63217	0.045969
Q96BN8	OTULIN	-0.23304	0.517974	1.149684	-0.50196	0.110481	0.459736	0.921673	-0.61876	-0.01053	0.617922	-0.47571	-0.18078	0.7059	0.370206	-0.0138	0.289962	-0.06182	-0.11198	-0.00537
Q96BP3	PPWD1	0.102268	-0.72004	0.110778	0.775058	0.338593	0.461653	-0.5457	0.666946	-0.11102	0.069646	0.328117	-0.2059	-0.03252	0.189357	0.539574	-0.09989	0.397791	0.610872	0.144412
Q96BQ5	CCDC127	0.550233	-0.33656	1.09519	0.467284	-0.58544	-0.45762	-0.08857	-0.70202	0.091226	0.004474	-0.32037	-0.93616	-0.4942	-0.36253	0.727214	0.938333	-0.65534	-0.4963	0.074578
Q96BR5	COA7	2.11552	-0.44101	-0.09968	-0.07175	0.963288	0.947131	-0.22949	0.497437	0.311924	0.52797	0.720652	-0.74708	0.030867	-0.5459	-0.12578	0.811847	-1.0278	-0.14731	-1.76567
Q96BW5	PTER	0.055329	0.198181	-0.16271	0.209322	0.363151	-0.44512	-0.12838	0.113511	-0.09439	-0.0517	0.835709	-0.46009	-0.12803	0.326802	0.18722	0.300321	-0.26419	0.145011	0.043078
Q96BW9	TAMM41	0.993952	0.137983	-0.08742	-0.0584	0.699866	0.352091	-0.40314	-0.49808	-0.2925	-0.33553	0.18851	-0.66354	-1.32331	0.05948	-0.60339	1.117872	-0.96016	-0.55221	-0.09025
Q96BX8	MOB3A	-1.38941	0.880556	0.895796	-0.62777	0.467921	0.524459	0.235177	-0.91945	-0.16373	0.97006	-0.365	0.472071	0.342426	2.169149	-0.88012	-0.2962	0.628573	0.213732	-0.11742
Q96BY6	DOCK10	-1.46953	0.878719	0.32988	-0.55321	0.638029	0.523665	-0.00128	-1.33398	-0.37613	1.242232	-0.56266	0.147898	-0.31619	0.41333	-0.11981	-0.13652	-0.47802	0.220321	-0.93078
Q96BY7	ATG2B	-0.62307	0.653213	0.261083	-0.3303	-0.02921	-0.09206	0.311026	-0.1592	0.225272	0.060833	-0.0751	0.271268	0.401271	-0.10665	0.078262	-0.13204	-0.00929	-0.26594	0.202854
Q96BZ8	LENG1	0.487289	-0.2373	0.289138	0.093483	-0.83679	0.273537	-0.40886	0.330166	-0.10079	0.111414	0.2821	-1.04441	-0.21437	-0.20666	1.032506	0.708473	-0.98827	-0.42276	-0.32552
Q96BZ9	TBC1D20	-0.6015	0.860568	0.220044	-0.28482	-0.62066	-0.43848	0.102405	0.162193	0.58085	-0.27198	0.073717	-1.95369	0.167871	-0.41769	-0.06137	-0.36674	-0.309	0.158843	1.153134
Q96C01	FAM136A	1.391051	-0.41247	0.028655	0.816476	0.289959	0.273494	-0.24521	1.207606	-0.35453	-0.19251	0.546346	-0.00418	0.073214	-0.59947	-0.41148	0.26406	-0.88843	-1.50852	-0.42897
Q96C10	DHX58	0.026703	0.319372	0.622594	0.061092	-0.46069	0.037302	0.187451	-0.58835	0.095747	-0.40664	0.101124	0.214977	-0.21038	0.397515	-1.58736	-0.06129	0.597537	0.922453	-0.69025

Q96C11	FGGY	-0.37995	-0.15554	-0.06102	0.439194	1.00339	0.601678	-0.23703	0.114117	0.098625	-0.19376	-0.09081	-0.24953	-0.08668	-0.19797	0.043942	0.444212	-0.09766	-0.2064	-0.17868
Q96C19	EFHD2	-1.04347	0.230615	0.56566	-1.07547	1.158965	-0.17742	-0.52339	-0.2901	1.028366	3.389191	-0.85417	0.905405	0.206126	1.594909	-0.82139	-0.60213	0.50427	-0.04707	-1.16826
Q96C23	GALM	-0.56098	0.004327	-0.30374	-1.01927	1.922351	-1.17033	0.238457	0.073508	0.986943	1.900832	-0.8985	1.134631	-0.04304	0.09226	-1.10725	-0.54169	0.461221	-0.09652	-0.39305
Q96C24	SYTL4	0.269253	0.146558	-0.05578	-0.78928	-0.94968	-0.39842	-0.22963	-0.68379	0.00622	0.070327	0.080325	-1.29038	-0.31008	0.034659	-1.02148	0.351275	-0.18667	-0.28563	-0.48329
Q96C36	PYCR2	-0.57511	-0.41418	-0.83007	0.246295	-0.88621	-0.92866	-0.02685	-0.67017	-0.13955	1.489924	-0.21904	-0.31861	-0.33672	-0.03404	0.146975	0.563998	-0.23624	-0.22666	-0.22549
Q96C86	DCPS	0.021269	-0.52467	0.018231	0.338555	0.409488	-0.11087	-0.01489	0.81093	0.337907	0.183696	0.422108	0.047147	0.649809	-0.07673	0.241075	0.4611	0.278057	0.731725	-0.29634
Q96CB8	INTS12	0.325724	-0.41703	-0.43907	-0.14688	-0.8685	-0.63704	-0.40613	0.707186	-0.04626	-0.04846	0.054083	-0.74117	-0.09132	-0.37824	0.317643	-0.12912	-0.19759	0.399706	0.200059
Q96CB9	NSUN4	0.9546	-0.28369	0.058373	0.040484	-0.13484	0.278287	-0.16303	0.604159	-0.27996	0.294255	0.145348	-0.08323	-0.13599	-0.73232	0.767739	0.108869	0.027758	-0.48177	-0.69415
Q96CC6	RHBDF1	-1.16584	-0.33867	-0.47071	-1.03145	-1.17769	-1.15573	-0.09962	-1.04787	0.190577	-1.16387	0.447995	-0.47974	-0.67048	-1.07116	-0.34813	-0.48185	-0.1611	0.351809	-0.13127
Q96CD0	FBXL8	-0.26684	0.122207	0.062729	-0.04591	0.143004	-0.03495	0.104506	-0.55476	-0.09553	-0.54193	-0.02793	-0.13362	-0.41371	0.020085	-1.06065	-0.71294	-0.472	-0.94912	0.188769
Q96CF2	CHMP4C	1.316073	-0.46745	-1.00699	-1.38458	-0.22606	0.648599	1.23616	0.493069	0.040847	-0.41466	0.534108	-0.26489	-0.3118	-0.51176	0.001488	-0.06518	-0.04552	-1.47846	0.045941
Q96CG8	CTHRC1	-1.73899	-0.73578	-0.42004	-2.14105	-1.48214	-0.658	-0.87754	0.14933	1.205695	-0.02133	-2.07088	-1.01697	0.397688	-0.94674	-1.37994	0.032864	-2.07306	-0.33602	1.731196
Q96CM8	ACSF2	-0.99108	-0.11788	0.365787	3.12974	-0.37385	-0.34077	-0.28158	0.887416	0.310438	1.137164	-0.0727	-1.21849	-1.21041	-0.68794	0.838247	1.17471	-0.52072	0.639277	-0.04588
Q96CN4	EVI5L	-0.86272	0.488643	-0.2051	-0.49909	-0.27835	-0.36192	0.46869	-0.80956	0.434609	-0.17448	-0.1001	-0.17073	0.204155	-0.47224	-0.15331	0.178626	-0.23689	-0.3115	-0.04844
Q96CN7	ISOC1	0.088404	-1.3895	-0.98937	-1.07989	0.588795	0.561607	-0.94984	-0.13573	-0.80377	-0.68527	0.709909	-0.62305	-0.86732	-0.22561	0.185794	-0.33341	0.247464	0.006565	-0.26305
Q96CN9	GCC1	-0.49933	0.377544	0.240213	0.260426	0.728783	0.611845	0.279457	0.217277	0.159234	0.215189	-0.21424	0.125473	0.232751	-0.05938	1.098528	0.350816	-0.21278	0.232211	0.256209
Q96CP6	GRAMD1/	-1.00501	0.119631	-0.28588	-0.25575	-0.81437	0.256419	0.074481	-0.22063	0.236483	0.185343	-0.22204	-1.37148	0.117542	0.029807	-1.20219	-0.06713	-0.37621	0.092484	0.295278
Q96CS2	HAUS1	0.26271	0.484962	0.36438	0.012697	0.596449	0.575712	0.158057	0.103184	0.161256	0.018175	0.161151	-0.27183	-0.25558	-0.21185	-1.08841	0.194503	0.303249	0.479522	-0.09629
Q96CS3	FAF2	-0.00884	0.162625	0.331562	1.128616	0.455402	0.617834	0.588686	-0.31634	0.033126	-0.23188	-0.13963	0.312782	1.219953	-0.41179	0.543073	0.402838	0.353355	0.136123	0.081693
Q96CT7	CCDC124	0.471607	-0.18321	-0.13409	-0.51273	-1.07773	-0.15924	0.07649	-0.12903	-0.04899	-0.25503	0.752036	-0.90754	-0.15913	-1.12969	-0.33883	0.833716	-1.105	-0.26757	0.273082
Q96CU9	FOXRED1	0.645328	0.07361	0.767825	-0.12994	0.154442	0.064197	-0.37045	-0.66673	-0.41154	-0.63064	0.360763	-0.17865	-0.04559	-0.11662	0.183237	0.211942	-0.32265	-0.41813	0.131903
Q96CV9	OPTN	-0.57358	-0.07748	0.809919	-0.29632	0.406766	0.651379	-0.25771	0.135353	0.704561	0.455001	-0.10828	-1.03648	0.137223	-1.12733	-0.38918	0.247331	-0.10973	0.432904	0.859091
Q96CW1	AP2M1	-0.43532	0.490394	0.584629	0.041484	0.363001	0.220918	0.58527	-0.29822	-0.19207	-0.1798	-0.21046	0.053984	-0.01158	0.110566	0.593309	0.190789	-0.10659	0.227651	0.227619
Q96CW5	TUBGCP3	-0.4215	0.316608	-0.15622	-0.52475	0.305748	0.40913	0.114255	-0.3867	0.789115	-0.0783	0.35289	0.037072	-0.22992	-0.15929	0.097994	-0.14149	0.249614	-0.11918	-0.02485
Q96CX2	KCTD12	-1.19555	0.172937	0.479248	-0.15868	-0.05891	-0.54108	0.029609	-0.13344	-0.23997	-0.00723	1.035772	-0.54483	-0.05953	-0.46269	0.392994	-0.56387	-1.08072	-0.52287	-0.85692
Q96D15	RCN3	-1.24718	-0.12578	-0.21603	-0.71627	1.101234	0.802929	-0.27032	0.262588	0.997808	1.245418	-1.07585	-0.8411	1.685005	-0.68577	-0.98612	1.393508	-0.35005	1.500369	1.764265
Q96D31	ORAI1	-0.19622	0.174864	0.260716	0.174814	0.294767	0.48334	0.938678	-0.5277	-0.09225	-0.22746	0.572676	0.017222	-0.21164	0.053955	-0.44958	-0.92535	-0.43394	1.156007	-0.75125
Q96D46	NMD3	-0.03129	0.243783	-0.30857	0.263679	0.875861	0.715702	0.117532	-0.22505	0.660865	-0.47659	0.336111	-0.0795	-0.15255	0.126278	1.145713	-0.04182	0.598625	0.208578	0.016415
Q96D53	COQ8B	0.194328	-0.12927	0.186491	-1.02596	-0.46637	-0.58356	-0.09061	0.158552	-0.66796	-0.77999	0.652462	-0.29343	0.307109	-0.60642	0.86143	-0.68772	-0.72598	-0.65928	2.072078
Q96D71	REPS1	0.136269	-0.15229	0.173881	0.276235	-0.51978	-0.36485	-0.08301	-0.36783	0.005708	-0.05609	0.260643	-0.49715	-0.02747	-0.53544	1.039526	-0.09664	-0.2578	-0.23489	-0.40019
Q96D96	HVCN1	-1.8555	0.766022	0.037053	-0.96862	0.295862	-0.63174	0.537564	-2.10789	0.267328	0.409243	-1.23303	1.383292	-0.21439	0.97244	-0.42872	-1.2797	0.349576	0.858965	-0.90383
Q96DA6	DNAJC19	0.824667	-0.30787	-0.12004	-0.27432	-0.25455	-0.34746	-0.36283	-0.15469	-0.56528	-0.34046	0.286401	0.176911	-0.39579	-0.39564	0.625375	1.656435	-0.32344	-0.49597	-1.26549
Q96DB5	RMDN1	-0.2337	0.383569	0.790576	-0.42107	0.73772	0.421384	-0.46771	-0.28942	-0.06267	-0.13225	0.268545	-0.35606	0.623777	-0.64691	-0.73304	-0.17143	-0.65559	0.305947	0.05898
Q96DC8	ECHDC3	1.00742	-0.11794	-0.17459	0.079948	-0.78141	-0.70065	-0.51081	-0.22284	0.514043	0.057173	-1.31004	-0.29599	-0.41093	-0.01649	0.824546	2.23705	0.498701	1.152315	-1.87473
Q96DE0	NUDT16	0.106065	0.20264	0.384589	0.222253	1.006866	0.577929	-1.33656	-0.06737	1.019552	1.041245	-0.1611	0.212097	0.156011	0.566678	0.519202	0.031989	0.004082	0.333119	-0.07435
Q96DF8	ESS2	0.659804	-0.04392	0.065025	0.26798	-0.78395	-0.10504	-0.2302	0.012012	0.501879	0.063315	-0.05296	-0.53786	-0.23695	-0.00416	-0.33924	0.154848	-0.19771	0.361233	-0.0816
Q96DG6	CMBL	-1.34596	-1.19513	-0.44045	-2.60769	1.007682	0.22298	0.056618	0.330519	0.879801	0.555675	-0.37398	-1.78945	-1.33442	-0.7913	0.805712	0.645864	-0.50335	0.301874	1.038307
Q96DH6	MSI2	-0.78545	-0.75247	-1.41535	0.122338	-0.55103	-0.02253	-1.01033	0.022032	-0.25616	-0.33331	1.156746	-0.87916	-1.0343	-0.79126	-0.04173	-0.24429	-0.68512	-0.4263	-0.37446
Q96DI7	SNRNP40	0.644233	-0.24844	0.230735	-0.01229	-0.09807	0.305185	-0.21102	0.15493	0.003453	-0.182	0.436916	-0.23489	-0.3478	-0.19655	0.540495	-0.17128	0.108392	0.287506	-0.30028
Q96DM3	WDR98	-0.48857	-0.21151	0.333333	-0.60043	0.199478	0.135847	0.054078	-0.15972	0.274498	-0.77165	-0.63262	0.443795	-0.16356	0.462271	-0.03845	0.276542	-0.35436	-0.07017	0.048445
Q96DT6	ATG4C	0.402992	0.156863	0.237012	-0.32834	0.345397	0.530641	-0.39143	-0.08119	0.027799	1.020063	0.624334	-0.31157	-0.73057	0.50914	-0.28872	0.442699	0.092161	0.116788	-0.80052
Q96DT7	ZBTB10	-0.03938	-0.08813	-1.06089	-0.54559	0.45531	0.506339	1.261227	-0.39298	0.128725	0.097559	-0.46693	0.374955	0.324443	-0.08177	-1.3198	0.024082	0.509386	0.81525	0.54185
Q96DV4	MRPL38	-0.42282	0.134016	0.549603	0.675672	0.166898	0.127209	-0.1659	-0.39466	0.254051	0.348167	0.656491	-0.04526	0.40095	-0.15198	0.482693	-0.02366	-0.30529	0.167705	-0.42252
Q96DX4	RSPRY1	-0.3601	0.236417	0.369223	-0.94181	0.111538	-0.30251	0.431701	-0.53879	0.190733	0.020438	0.034665	-0.33036	0.058334	0.17667	0.214872	0.392759	0.094121	1.060932	0.395125
Q96DZ1	ERLEC1	-0.16037	0.055903	0.15515	0.602246	-0.1281	-0.32807	0.81129	-0.20976	-0.29238	-0.3923	0.266123	-0.81218	0.448704	-0.23798	0.564564	0.090043	-0.64988	0.497564	-0.22462
Q96E11	MRRF	1.120845	-0.38357	-0.23244	-0.06296	0.830289	0.23256	-0.62711	-1.07351	-0.83996	-0.21712	0.187952	-0.07302	0.188172	-0.2356	-0.36374	0.697013	0.073292	-0.61052	-0.65031
Q96E29	MTERF3	0.321056	-0.24872	-0.26897	0.8611	-0.47457	-0.04833	-0.25127	-0.60509	-0.38577	-0.34408	0.308301	-0.34538	-0.24019	-0.24924	0.537498	0.269408	0.048678	-0.02729	-0.384
Q96E39	RBMXL1	1.861811	-0.34997	-0.80643	0.415561	-0.81454	-0.48097	-0.65866	1.027432	-0.70232	-1.60799	0.153401	-1.65115	-1.27588	-1.06549	-0.14335	-0.13947	-1.25499	-0.23628	-0.63947
Q96E52	OMA1	1.003645	-0.22851	-0.394	0.064639	-0.8464	-0.71599	-0.37705	0.17141	-0.20463	-0.31095	1.037624	-0.69302	-1.42995	-0.42833	0.969363	1.185656	-0.97314	-0.91886	-1.31322
Q96EB1	ELP4	0.293338	0.076574	-0.22469	-0.63838	0.71371	0.496514	0.091179	-0.29949	-0.28933	-0.31292	-0.54786	-0.43821	-0.22393	-0.01259	-0.42407	-0.13177	-0.38677	-0.23771	0.09423
Q96EB6	SIRT1	0.19898	-0.21361	-0.0138	-0.2953	0.474093	0.879514	-0.17554	0.543766	-0.44056	-0.22592	-0.06473	-0.45456	0.029654	-0.24751	-0.14286	0.652294	0.635583	0.857978	0.090285
Q96ED9	HOOK2	-0.35657	-0.59562	-0.4909	0.340016	-0.33763	0.096802	-0.45686	0.387646	-0.41139	-0.27402	0.345853	-0.45206	-0.41461	-0.65387	0.023021	0.363979	0.098017	0.201133	-0.39413
Q96EE3	SEH1L	0.137792	-0.19595	-0.19011	0.116021	0.401143	0.391258	-0.44929	0.216795	0.12336	0.13577	0.321668	0.167588	-0.36521	0.196968	0.653732	-0.19195	0.2659	-0.26894	-0.02229
Q96EH3	MALSU1	-0.05584	0.58495	-0.05589	0.892881	0.574246	0.634052	-0.298	-0.69312	0.216373	0.644819	0.588885	0.199436	-0.5858	0.201281	0.184797	0.766326	-0.06634	-0.78938	-0.50929
Q96EI5	TCEAL4	1.170623	-0.00469	-0.33732	-0.6779	-0.61359	0.128319	-0.30266	0.853912	0.07968	0.296275	0.959199	-1.4265	0.414283	-1.50711	-0.27222	-0.06182	-0.86529	0.186878	-0.47737
Q96EK5	KIF1BP	0.290157	-0.42056	-0.09698	0.299731	1.332285	1.411527	-0.01623	0.114814	-0.37839	0.200576	-0.32736	-0.03233	0.028514	-0.07654	-0.17677	0.38654	0.350837	-0.15765	0.362392
Q96EK7	FAM120B	-0.55586	-0.27107	0.151276	0.099474	-0.33766	0.037702	0.054911	-0.76425	-0.10153	0.519637	0.733611	-0.16132	0.112103	-0.11053	0.491501	-0.20173	-0.04521	0.060668	-0.07307
Q96EK9	KTI12	-0.11875	-0.96507	0.112661	-0.61306	-0.20663	0.509822	0.347471	0.269438	0.040847	0.771401	0.013197	-0.24771	-0.59611	-0.33242	-0.55456	0.207476	0.164602	-0.13116	-0.16541
Q96EL3	MRPL53	-0.18471	-0.09943	0.198896	0.998609	0.964288	-0.02327	-0.29275	-0.60698	-0.0087	0.726217	0.761602	-0.29077	0.248436	-0.68147	0.222398	-0.40802	-0.64452	-0.09425	-0.9931
Q96EM0	L3HYPDH	-0.36289	0.28836	-0.17156	-0.67235	0.143076	-0.35226	0.061373	-0.5946	1.332798	0.943183	-0.4483	-0.89763	0.028148	-0.4982	-0.3889	0.049318	-0.27186	1.038799	1.378234
Q96EP0	RNF31	0.026745	0.424902	0.027504	-0.80946	-0.23055	-0.33156	1.082197	0.02793	1.037484	0.2047	0.22388	-0.1613	-0.22493	0.383034	-0.04654	0.281214	-0.4411	-0.0853	-0.2292
Q96EP5	DAZAP1	0.448993	-0.44849	0.119984	0.459622	0.542432	0.658596	-0.63257	0.17265	0.173929	0.200542	0.309617	0.147692	-0.03678	0.184211	0.221179	-0.29711	0.151339	-0.23219	-0.71759
Q96EQ0	SGTB	-0.47634	0.339179	0.068575	-0.49788	0.055657	0.008302	-0.11625	-0.34409	-0.32805	0.14735	-0.18028	1.010055	0.218294	0.989241	-0.8193	0.184568	0.483728	0.617552	-0.55811
Q96ER3	SAAL1	-0.87347	-0.0167	0.236866	0.072532	0.214086	0.358013	-0.30562	0.764055	-0.13981	0.065778	0.264412	-0.5729	0.160681	-0.38324	0.135924	0.463675	-0.35193	-0.5151	0.576962
Q96ER9	CCDC51	0.701168	0.034843	0.116846	-0.35313	-0.25244	-0.12733	0.566035	-0.48574	-0.01664	-0.21103	0.762091	-0.02036	0.048195	-0.47345	-0.08782	-0.0694	-0.66608	-0.17931	-0.6268
Q96ES7	SGF29	1.379747	-0.38932	0.043306	-0.1689	0.290645	0.705408	-0.52478	0.144422	-0.33969	-0.24099	0.279993	-0.3024	0.100021	-0.37093	-0.12586	0.110136	-0.13836	-0.00995	-0.15638
Q96EU6	RRP36	0.969352	-0.00766	-0.05457	0.251381	0.271281	0.354566	-0.50518	0.342648	0.541665	0.049069	1.282018	-0.77068	0.618101	-1.02186	0.475869	-0.04294	-0.24951	0.209276	-0.00248
Q96EV2	RBM33	0.176904	-1.2011	-0.25187	0.708301	-0.23465	-0.18823	-0.77149	0.15941	-0.48947	-0.26716	-0.05484	-0.34481	0.369289	-0.16228	0.38788	0.055232	0.109644	0.144606	-0.0752
Q96EV8	DTNBP1	0.172703	-0.04662	0.260473	0.281848	0.224544	0.09597	-0.17102	-0.46411	0.042881	0.166539	0.336126	0.072987	-0.01587	-0.32728	0.095461	-0.2425	0.193719	-0.09483	-0.21056
Q96EW2	HSPBAP1	-0.22274	0.249747	0.615375	0.059784	0.57761	0.692198	-0.48076	-0.07384	0.404403	1.033076	-0.21225	0.151436	0.0456	0.462782	1.032634	-0.98598	-0.18811	0.324875	-0.31123
Q96EX1	SMIM12	0.533138	-0.54142	-0.07442	0.847615	1.301999	1.175233	-0.28205	1.495078	0.154932	0.864933	-0.06104	-0.41299	-0.18582	-0.20992	0.95146	0.14075	-0.79081	0.230934	-0.19962
Q96EY1	DNAJA3	0.337385	-0.03368	-0.24315	0.079373	-0.27113	-0.27617	-0.16855	0.096728	-0.41662	-0.25495	0.313472	0.15187	-0.04707	-0.06944	0.898165	0.320059	0.799487	0.358288	-0.86986
Q96EY4	TMA16	0.173888	-0.1393	0.005427	0.494778	0.04432	0.27738	-1.31014	-0.56613	-0.12253	-0.00323	0.660397	-0.66695	-0.32769	-0.01129	-0.38714	0.322059	-0.35302	-0.17529	-0.6318
Q96EY5	MVB12A	-0.4415	-0.0043	-0.07442	-0.83242	-0.6876	-0.21758	0.148563	0.8562	0.014199	0.260256	-0.48178	0.204521	-0.27142	0.659222	-0.50119	-0.3583	0.314652	-0.71486	0.181443
Q96EY7	PTCD3	-0.14933	-0.35579	-0.22741	0.555263	0.20945	0.364651	-0.13276	-0.0695	0.029673	0.29595	0.667837	0.128989	-0.34463	-0.18639	0.920004	-0.28977	-0.25834	-0.28373	-0.27464
Q96EY8	MMAB	1.038404	0.00667	-0.88617	1.701514	1.306147	0.647985	-0.32238	-1.24445	-0.35164	0.237868	-0.15277	0.446605	0.011106	-0.39794	1.232545	-0.6759	-0.38534	-0.02626	0.17036
Q96EZ8	MCRS1	0.196482	0.017608	0.52984	-0.26408	0.269751	0.701469	-0.1586	-0.03426	0.146617	0.240162	0.269445	0.32432	0.101788	0.593141	0.018321	-0.03602	0.347893	0.698669	-0.68036
Q96F07	CYFIP2	-0.4371	-0.32321	0.078996	-0.18541	0.131705	0.019904	0.012662	0.034593	0.347555	0.471531	0.025298	-0.30172	-1.02227	0.817594	0.418612	0.134298	0.032376	0.118647	-0.51369
Q96F15	GIMAP5	-2.28308	1.54382	0.940236	-1.29522	0.860992	0.23896	0.480737	-1.67365	0.934667	0.242824	-0.34632	1.493579	0.034295	1.392818	-0.62329	-1.05046	0.159489	0.476714	-0.71507
Q96F24	NRBF2	0.588314	0.101418	0.291013	-0.37576	-0.43885	0.044877	0.135147	-0.39173	-0.05281	1.518616	-0.66719	0.485779	-0.0334	1.271372	-0.54541	-0.21824	1.130074	0.919911	0.476381
Q96F44	TRIM11	-0.11674	-0.06487	-0.04757	0.477692	0.016745	-0.08594	0.241667	0.331039	0.132883	0.594988	0.388479	0.010711	0.086855	0.321299	-0.06897	0.057204	0.536364	-0.25072	0.204231
Q96F45	ZNF503	-0.31408	-0.08543	-0.36589	-0.4563	-0.75096	-0.47069	-0.20119	-0.15874	-0.11168	-0.86426	0.354714	-1.29615	-0.01094	-0.75074	-0.10018	0.214667	-0.77692	-0.63745	-0.0055
Q96F63	CCDC97	-0.08797	0.113944	-0.02112	-0.13069	-0.10049	-0.3637	0.482234	1.159282	-0.19196	-0.26781	-0.36015	0.116406	-0.15888	0.409876	0.146641	-0.8714	-0.31697	-0.16267	0.189767
Q96F86	EDC3	0.293745	0.888366	-0.22052	0.095251	0.651392	0.637867	-0.16303	0.241273	0.036752	-0.88733	0.352915	-0.06555	0.323798	-0.15361	-0.40436	-0.21687	0.670816	-0.09998	0.114988
Q96FH0	BORCS8	0.437234	0.40453	0.279123	-0.17613	0.131	-0.4517	0.588597	-0.28007	0.464147	0.141233	0.05828	0.439762	0.170766	0.553933	0.117756	-0.00201	0.255118	0.072081	0.019921
Q96FK6	WDR89	0.576878	0.059089	-0.20124	0.34748	-0.31895	-0.11256	-0.15714	-0.13665	-0.18519	-0.24346	0.470565	-0.45296	-0.74304	-0.09781	0.360284	-0.05857	-0.4203	-0.07911	0.11343
Q96FN4	CPNE2	-0.27272	-0.55668	-0.23697	-1.04786	0.503778	-0.10221	-0.3887	-1.14778	0.386246	0.112609	-0.49545	0.714086	-0.81146	1.658505	-0.08253	0.419837	0.546323	0.186496	0.311986
Q96FQ6	S100A16	-0.19016	0.261118	-0.06384	0.358901	-0.03087	0.53297	-0.30405	-0.65355	1.034833	-0.0985	-0.1312	-0.35056	0.270965	-1.12683	0.204516	0.126412	-0.39392	-0.63942	-0.85214
Q96FS4	SIPA1	-0.49924	-0.05705	0.677125	-0.4218	0.405125	0.068003	0.437849	-0.56469	1.01489	0.818419	-0.47065	0.890016	0.338247	1.108363	-0.13574	0.134607	0.016193	0.475935	-0.32185
Q96FV2	SCRN2	-0.39555	-0.51807	0.804119	-0.59095	1.339921	-0.7511	1.913161	-0.5618	-0.30524	0.206484	0.26813	-0.67638	-1.10216	-0.13897	-0.50295	-0.36107	-0.3591	0.263637	-0.30514
Q96FV9	THOC1	0.253264	0.087647	-0.21731	0.296395	0.445851	0.606263	0.046418	0.57943	-0.51869	0.137937	0.674056	-0.15919	-0.70733	-0.0437	-0.07437	-0.47465	0.442642	0.606483	-0.29023
Q96FW1	OTUB1	-0.13666	-0.35327	0.177679	0.105318	-0.50736	-0.19851	-0.07975	0.746746	0.091166	0.173185	-0.00854	0.118347	0.236712	0.220965	-0.03283	0.205622	0.277764	-0.22406	-0.0367
Q96FX7	TRMT61A	0.040458	0.289974	0.214112	0.083232	-0.14641	-0.13419	0.444152	0.019644	0.413657	0.581522	0.141961	0.125254	-0.02035	0.128156	-0.04866	-0.0389	0.320318	0.018284	-0.91234
Q96FZ2	HMCES	0.319729	-0.44497	0.121492	0.59847	1.079876	1.463712	-0.47032	0.318868	0.065437	0.439043	0.179092	-0.47716	-0.08228	0.190287	0.890917	0.567109	-0.1388	-0.57215	-0.64984
Q96FZ7	CHMP6	-0.5209	0.379161	0.241747	0.374079	-0.28118	-0.16097	-0.22712	-0.90606	-0.22137	0.13003	-0.02576	-0.49663	-0.09179	-0.00747	-0.51003	-0.36599	-0.16993	0.435815	-0.00089
Q96G01	BICD1	0.802524	0.077661	-0.55626	0.020622	-0.20168	-0.08461	-0.18758	0.376954	-0.38517	-0.41863	0.882796	-0.84557	0.03194	-0.83632	-1.11145	0.059102	-0.08062	-0.51903	0.579182
Q96G03	PGM2	-0.35871	-0.48612	0.276745	-1.15966	1.515604	-0.62871	0.191389	-0.06757	0.481663	0.641224	-0.42178	-0.73401	0.230981	0.129984	-0.03841	0.109912	-0.54161	0.608031	1.114687
Q96G21	IMP4	0.086814	0.371771	-0.31278	0.349739	-0.2864	0.14118	-0.16188	0.274988	0.181664	0.072925	0.899182	-0.10778	-0.25335	-0.15349	0.114046	0.061638	-0.19718	-0.29829	0.087659
Q96G23	CERS2	-0.1051	-0.05476	0.127553	0.100839	-0.04022	0.339023	-0.43819	0.418915	-0.08944	0.085101	-0.3994	0.301671	0.212107	0.175582	1.455947	-0.06765	0.349174	0.565798	-0.2857
Q96G25	MED8	0.355986	-0.16451	0.667176	-0.0069	0.098739	-0.14333	1.355291	0.436003	0.040847	-0.36041	0.44813	-0.19896	-0.4195	-0.32287	0.11699	0.353672	0.067655	0.290867	-0.01666
Q96G28	CFAP36	0.464638	-0.07678	-0.28401	-0.0902	-0.31029	-0.22914	0.081103	0.636499	-0.10453	0.417758	1.039374	0.025444	-0.27625	-0.11701	-0.72645	0.273091	-0.1302	-0.51475	-0.38384
Q96G46	DUS3L	-0.26156	0.216107	0.130194	0.025867	0.02904	0.35102	-0.06806	-0.31443	-0.04833	0.114036	-0.4824	0.42455	-0.5948	-0.36797	0.273321	0.017166	-0.7207	-0.14699	-0.42797
Q96GA3	LTV1	-0.35537	-0.01969	-0.06306	0.519254	-0.42638	-0.04373	0.084628	-0.20858	-0.05662	0.753329	0.055015	-0.62311	-0.0732	-0.42176	-0.57829	-0.18352	-0.12383	0.222004	-0.07596
Q96GA7	SDSL	-0.81449	0.410253	-0.34798	-0.0733	0.169334	-0.56991	0.149738	0.288621	0.295529	-0.6706	-0.50317	1.243851	0.092379	-1.1492	0.891654	-0.78615	-0.30017	-0.49176	0.358491
Q96GC5	MRPL48	-0.6369	0.079586	-0.4416	1.25051	0.453489	0.463544	-0.76877	-0.38045	-1.02423	-0.16389	0.353966	0.148991	0.41018	-0.06807	0.607116	-0.1212	-0.17018	0.294252	-0.34009
Q96GC9	VMP1	-1.10872	0.546634	0.799524	0.470002	-0.14494	0.051094	1.46967	0.131852	0.15649	-0.2126	-0.67958	0.916594	0.650133	0.427011	0.119533	1.315959	0.436045	-1.59731	0.209654
Q96GD0	PDXP	0.073183	0.466888	0.043233	-0.09467	0.665734	-0.32081	-0.03883	-0.36288	-0.14409	0.472832	0.066668	0.19726	0.187525	-0.11832	-0.26129	-0.03295	-0.08499	0.297565	0.247011
Q96GG9	DCUN1D1	0.753481	-0.76423	-0.09338	0.110346	1.276674	1.06677	-0.54253	0.074083	0.149156	0.57833	-0.11186	-0.34458	-0.58223	-0.32997	1.13569	0.159055	-0.03344	-0.55094	-0.42015
Q96GK7	FAHD2A	-0.10045	0.022162	-0.0078	0.058503	0.671452	0.128338	-0.16653	0.469722	-0.04256	0.128588	0.332669	-0.14904	0.009969	0.104764	0.413009	0.663054	-0.18645	-0.19716	0.166244
Q96GM5	SMARCD1	0.403143	-0.12272	-0.26797	-0.33641	0.218354	0.112191	0.031273	1.058194	-0.51023	-0.1816	0.225082	-0.68123	-0.16991	0.037477	0.007994	-0.99352	-0.13794	0.287337	0.483977
Q96GM8	TOE1	0.783927	0.00998	-0.24055	-0.76711	0.696202	0.763318	-0.21206	-0.42466	-0.0833	-0.58117	-0.13665	0.14788	-0.20662	-0.52207	0.193339	-0.3182	0.028477	0.555085	-0.96038
Q96GQ5	C16orf58	-0.12294	0.239933	-0.46865	0.20279	0.122955	0.105504	0.503012	-0.86726	-0.04518	-0.34886	-0.30496	0.200234	0.478196	-0.10145	-0.1501	0.32157	-0.06889	-0.2604	-0.11521
Q96GQ7	DDX27	0.42774	-0.36895	-0.80564	0.821569	0.090238	0.260569	-0.33789	0.30698	-0.41898	-0.02278	0.37346	-0.18765	0.208534	-0.58871	-0.42491	0.428275	-0.09373	0.125866	-0.50465
Q96GS4	BORCS6	0.03293	1.135687	0.335923	-0.18716	0.114929	-0.61038	0.25807	-0.35512	-0.15839	0.460465	0.20053	0.064971	-0.37806	-0.17983	-0.37054	-0.12344	-0.02854	0.104266	-0.43804
Q96GS6	ABHD17A	-0.21381	0.19042	0.07026	-0.38835	0.082292	-0.00029	0.044147	-0.4393	-0.0894	-0.07005	-0.73437	0.294316	-0.14522	0.263701	-0.23668	-0.38975	-0.30676	0.421754	0.1435
Q96GW9	MARS2	0.241583	-0.12013	0.136627	0.233396	-0.76817	-0.74085	-0.07797	-0.7269	-0.16613	-0.01564	0.594797	-0.23786	-0.15757	0.032783	0.581563	0.665773	0.031043	-0.22747	-0.82656
Q96GX9	APIP	0.13813	-0.49942	1.829782	-0.09843	0.590965	-0.05545	0.475636	0.080244	0.040847	0.06861	0.517423	-0.56467	-0.34768	0.300327	0.586946	1.001883	0.088466	0.86646	-0.12188
Q96GY0	ZC2HC1A	0.201224	-0.33798	-0.51317	-0.41432	-0.94857	-0.79708	-0.18368	-0.55225	-0.0666	-0.51508	0.15719	-0.69847	-0.16918	-1.06712	-0.55168	-0.1213	-0.3608	-0.48652	0.107278
Q96H20	SNF8	-0.3888	0.315853	0.077236	-0.29802	0.026616	-0.20656	-0.03724	-0.19836	0.354836	0.327142	0.025926	-0.21521	-0.18305	0.069741	-0.37203	0.342648	0.158084	-0.05152	0.480004
Q96H79	ZC3HAV1I	0.36226	0.452707	-0.82081	0.531799	-0.92713	-0.87232	1.200735	0.174276	0.034556	-0.11219	0.467207	-1.09051	0.027296	-0.43642	0.998029	0.673734	-0.06705	-0.59711	0.161751
Q96HA7	TONSL	1.217764	-0.02635	-0.02628	-0.58319	0.873644	1.245781	-0.49887	0.101746	-0.36391	-0.03388	-0.00985	-0.27248	0.097841	-0.7751	-0.28983	0.489451	0.037911	-0.0835	-0.35901
Q96HC4	PDLIM5	0.000477	-0.81207	-0.93891	-1.10171	-2.02285	-1.53332	-1.46881	-1.50799	1.096163	-0.40535	-0.12799	-0.789	0.099858	-0.61197	-0.33814	-0.0292	-1.01626	0.522635	1.581812
Q96HD1	CRELD1	0.515523	0.283247	0.16918	-0.57008	-0.45822	0.106373	0.027836	-0.04914	0.399745	0.182171	-0.393	-1.27168	0.125125	-1.01822	-0.74134	-0.34838	-0.55706	0.240658	0.801341

Q96HE7	ERO1A	0.323513	0.540065	-0.83642	0.609895	0.397602	-0.61882	-0.75559	0.154925	-0.09449	0.712588	-0.20787	1.430456	1.469992	1.380422	-1.07208	0.581541	1.716435	-0.18442	-0.15566
Q96HF1	SFRP2	-1.85105	-0.10408	-0.66001	-1.82292	-2.6383	-1.63904	-0.07975	-0.6347	0.597615	-1.99371	-2.93774	-1.31866	-1.05089	-0.95001	0.486797	-2.00754	-1.29456	-1.83991	-0.91833
Q96HH9	GRAMD2E	-1.41934	0.908558	0.124544	-0.59283	-0.64987	-0.25462	0.527921	-1.07199	1.617098	-1.05814	0.386762	-0.71721	-0.00353	0.511561	0.846147	1.806663	-0.16963	-0.66152	0.319996
Q96HN2	AHCYL2	-0.23696	0.185577	-0.11848	-0.16592	-0.61091	-0.01049	0.017566	-0.14938	-0.22545	-0.4321	-0.14313	-0.42085	-0.1603	0.103891	-0.10462	0.069958	-0.28448	-0.02349	-0.06786
Q96HP0	DOCK6	-0.85265	-0.01259	0.493187	-0.57989	-0.39049	-0.60149	-0.18985	0.013629	0.985521	0.562037	0.428621	-0.7022	-0.21144	-0.59139	-0.36282	-0.17849	-0.01466	-0.18353	0.0748
Q96HP4	OXNAD1	-0.39304	-0.06729	-0.06461	-0.20547	0.73081	0.038965	0.095409	-0.54003	0.037166	0.359885	0.679886	-0.30924	0.137535	-0.21105	-0.12634	0.334406	0.150724	-0.05458	-0.17472
Q96HQ2	CDKN2AIF	0.247323	0.157825	-0.10827	0.042308	-0.2842	-0.75351	-0.60695	0.207208	-0.03952	0.638059	0.00223	-0.01905	-0.08774	0.623544	0.210855	0.130496	-0.21155	-0.35722	0.091331
Q96HS1	PGAM5	0.793675	-0.11133	-0.38381	0.962033	-0.07563	0.081035	0.255665	0.516375	-0.19968	-0.34048	0.346867	-0.30111	-0.09225	-0.53801	-0.12467	-0.16109	-0.16145	-0.468	-0.0384
Q96HU1	SGSM3	0.012125	-0.04856	0.011415	-0.01675	-0.37849	0.133638	-0.27863	-0.50961	-0.14666	-0.09402	0.294617	-1.03153	-0.20088	0.068763	-0.34863	-0.07154	0.017733	-0.72469	-0.25047
Q96HV5	TMEM41A	0.590729	0.110756	0.510823	0.698721	-0.23519	-0.32646	0.176765	-0.02899	2.599936	0.029319	-0.24835	-0.79972	0.005588	-0.52736	1.860118	-0.77061	0.499222	-0.48224	0.670437
Q96HW7	INTS4	0.061223	-0.11328	0.001421	-0.05496	-0.28702	-0.1846	0.203826	0.792118	-0.18295	0.159792	0.387463	0.08563	-0.55075	-0.0361	0.274189	-0.08725	0.080441	0.078349	-0.04041
Q96HY6	DDRGK1	-0.29072	0.390859	0.27325	0.151237	0.12603	0.156882	0.165826	-0.81386	-0.31714	0.42536	0.421129	-0.61493	0.001188	-0.23384	0.494613	-0.20379	-0.63098	0.185541	-0.0288
Q96HY7	DHTKD1	0.340362	-1.00843	-0.24394	0.084222	0.368301	0.684985	-0.64899	-0.23881	0.058204	-0.36153	-0.25787	-0.16538	-0.6701	-0.00696	0.772579	1.400743	-0.03547	-0.62255	-0.15012
Q96I15	SCLY	-0.45695	-1.22551	0.121324	-0.32488	-0.30226	-0.48894	-0.34191	0.233091	-0.83811	-0.38184	0.907742	-0.48646	-1.23547	0.283013	0.803797	0.055902	-0.57973	0.350323	0.110092
Q96I24	FUBP3	0.708357	0.110726	0.138361	-0.09232	-0.10428	-0.07465	-0.32349	0.193525	-0.10132	-0.08482	0.309902	-0.1737	-0.06989	-0.26532	0.017435	-0.34423	0.054978	-0.01304	0.071076
Q96I25	RBM17	0.741858	-0.35048	0.241444	0.480725	-0.201	0.536914	-0.36433	0.640685	0.453017	0.153233	0.33374	-0.51469	0.037763	-0.53195	0.122316	-0.30279	0.348773	0.309224	-0.03358
Q96I51	RCC1L	0.210448	0.031271	0.068924	0.100253	0.617936	0.123951	0.14987	-0.47886	0.102049	-0.00427	-0.02659	-0.17724	0.05909	0.116554	0.388812	-0.36252	-0.30462	-0.33718	-0.28323
Q96I59	NARS2	0.322391	-1.22316	-0.00426	0.207065	0.152949	-0.07165	-0.62679	-0.07042	-0.22109	-0.36623	0.309573	0.244598	0.081872	0.32194	0.127195	0.931988	-0.8076	-0.6159	-0.63453
Q96I99	SUCLG2	0.642916	-0.58581	-0.05611	1.314609	1.141585	0.136891	-0.49779	0.439791	-0.22028	0.415254	0.322444	0.213738	-0.0693	0.397776	1.175574	-0.23778	-0.87845	-0.25495	0.025044
Q96IF1	AJUBA	0.911281	-0.09635	0.616828	-0.27916	-0.75243	-0.78841	0.145242	-0.68773	0.294024	-0.44786	0.141974	0.722026	1.71762	-0.17935	-0.55003	0.006036	-0.01744	-0.54524	0.116905
Q96IG2	FBXL20	-0.71637	0.471789	0.031747	0.663016	-0.13172	0.108403	-0.08446	-0.37848	0.264396	-0.03284	0.344393	0.183495	-0.14445	-0.10427	-0.22621	-0.14263	0.126903	0.518531	0.00253
Q96II8	LRCH3	-0.33632	0.359241	-0.55651	-0.12298	0.205278	0.003132	0.353602	-0.62796	-0.00398	0.254076	-0.54172	0.241529	0.45899	0.490514	0.07153	-0.08142	0.431264	0.730308	0.306149
Q96IJ6	GMPPA	0.08038	-0.36471	0.454784	-0.23604	0.255907	0.393568	0.640556	-0.02507	0.685979	-0.56106	0.018346	0.27048	0.556736	0.202277	0.201837	0.140449	-0.00436	0.191376	0.592989
Q96IU4	ABHD14B	-1.39522	-0.31519	-1.74188	-1.44544	1.189079	-0.06247	0.560976	-0.12005	-0.32741	0.501303	0.844329	-0.07074	-0.75056	0.241008	-0.2648	-0.37314	-0.4766	-0.30068	-0.98874
Q96IV0	NGLY1	0.390992	-0.56913	-0.171	-0.54856	0.328397	0.606991	-0.38958	-0.19275	0.130163	-0.16141	0.845144	0.185564	-0.07554	0.021922	0.174503	-0.06061	-0.6497	-0.52264	0.045229
Q96IX5	USMG5	-0.05898	-0.26944	-0.17798	0.42244	0.157038	0.136295	-0.04037	-0.5194	-0.21027	-0.50412	0.15821	0.251143	-0.49817	-0.14681	1.583976	0.845739	-0.03032	-0.8154	-0.65389
Q96IY1	NSL1	0.949654	-0.33637	0.023175	-0.3223	0.070397	-0.11499	-0.22324	0.694205	-0.50748	-0.11318	0.129633	-0.08859	-0.71032	-0.15012	-0.01294	-0.08572	-0.23153	-0.31435	-0.42787
Q96IY4	CPB2	-2.41943	-0.34737	-1.30449	-2.04816	-0.92773	-1.85368	-0.77861	-1.29231	-0.30293	-1.76983	-0.15732	1.664003	-0.70301	1.736041	0.262567	-1.7406	1.546196	-1.10791	-1.39126
Q96IZ0	PAWR	0.907816	-0.73213	-0.61849	-1.18273	-1.00902	-0.19543	-0.14273	0.130534	0.244349	-1.54318	0.106265	-1.17252	0.543643	-1.20278	-0.76787	-0.01937	-1.36122	-0.16345	0.584351
Q96IZ7	RSRC1	0.707448	-0.76603	-0.04066	-0.08522	0.432043	0.547504	-0.40939	0.376676	0.571044	-0.25377	0.258168	-0.45706	-0.19143	-0.70292	1.008893	-0.23205	-0.31256	0.483777	-0.0036
Q96J01	THOC3	0.150868	-0.31025	-0.22027	1.105471	0.831845	1.062408	-0.40365	0.550324	-0.36152	-0.09095	0.473409	0.047527	1.12218	-0.35455	-0.51202	-0.28939	0.887948	0.710615	-0.37691
Q96J02	ITCH	-0.18738	0.462119	0.077593	-0.33239	0.321341	0.442075	0.529255	0.113465	-0.37423	0.199319	-0.10254	0.042948	0.675402	0.165808	0.161626	0.848102	0.279076	0.316551	0.019342
Q96J42	TXNDC15	-0.57561	0.453418	0.274243	0.074067	0.707678	0.530292	0.456032	-0.53807	0.291419	-0.31924	-0.14495	0.032641	0.126268	-0.47546	0.809108	0.051393	-0.19643	0.280573	0.210405
Q96J84	KIRREL1	0.451808	-0.31534	0.432808	-0.47853	-0.4411	-0.5751	0.417674	0.80006	0.065555	0.280894	-0.84275	-0.81561	0.045942	-1.01779	0.508667	0.317106	-0.51491	-0.18947	0.351865
Q96J88	EPSTI1	-0.67315	1.173067	0.269516	-0.73373	-0.01458	1.000105	0.209372	1.520145	0.19735	0.290546	-1.60274	-0.87373	0.55954	-0.47109	-0.47828	-0.29038	-0.69662	1.696941	-0.40821
Q96JA1	LRIG1	-1.04127	-0.59497	-0.39821	1.409545	-0.58372	-0.26711	-0.38516	-0.75761	-0.52311	-1.69747	0.846852	-0.75289	-0.20337	-0.74484	0.374781	-1.49293	-1.03614	-0.81458	-1.0141
Q96JB2	COG3	-0.42119	0.493283	0.078406	-0.17765	0.325644	0.195722	1.227114	-0.13323	-0.5698	0.745137	-0.13249	-0.05295	-0.14811	0.018895	0.841208	0.478155	-0.03112	0.094751	0.41419
Q96JB5	CDK5RAP	-0.24768	0.356963	0.485375	0.203493	0.294151	0.217911	0.397107	-0.72203	-0.14928	0.531363	0.691376	0.016916	-0.12466	0.207434	0.449393	-0.23269	-0.11122	0.230935	-0.14531
Q96JB6	LOXL4	0.95675	0.004009	-0.08346	-0.2393	0.744609	0.42841	-0.16749	-0.05111	-0.30722	0.258873	0.674132	-1.09551	0.259173	-0.99987	-0.29383	-0.4379	-1.18357	-1.24035	0.373796
Q96JC1	VPS39	-0.27298	0.941474	0.3244	0.302297	0.145615	0.152325	0.387847	-0.03205	-0.07443	0.096856	-0.32459	0.688494	0.439153	0.841084	-0.0468	-0.41984	0.189419	-0.21243	-0.21446
Q96JG6	VPS50	0.243722	-0.03084	-0.0813	-0.32756	-0.15525	-0.32715	0.108498	0.202936	-0.67469	0.146715	0.19822	-0.08917	-0.2781	0.192605	0.46028	0.306049	-0.18633	-0.50364	-0.45736
Q96JG8	MAGED4	0.411732	-0.34775	0.000669	-0.18397	-0.35897	-0.42308	-0.42095	0.512998	-0.2108	-0.69053	0.337308	-1.131	0.014264	0.167655	-0.34631	0.428973	-0.49361	-0.00292	0.848451
Q96JH7	VCPIP1	-0.00835	0.127841	-0.01849	-0.902	0.168671	0.440767	-0.15342	-0.43666	-0.27063	0.058794	-0.13382	0.292038	0.545306	0.190601	-0.10714	-0.11988	-0.05448	-0.19299	-0.01142
Q96JI7	SPG11	-0.12886	1.240745	-0.23913	-0.09159	0.07541	0.010433	0.334802	-0.27722	0.040847	0.367338	-0.17871	0.371318	-0.52766	0.49315	-0.00069	-0.55185	-0.26467	-0.20821	0.227481
Q96JJ3	ELMO2	-0.93097	1.14878	0.690256	-0.47933	0.130652	-0.28926	0.325126	-0.59127	0.073399	0.4536	-0.50321	1.012611	0.035518	1.122624	-0.53075	-0.49854	0.082307	0.054006	0.117322
Q96JJ7	TMX3	-0.33812	0.678499	0.584948	0.337594	-0.15482	-0.36026	0.782622	-0.87103	-0.20223	0.061267	0.517208	0.339764	-0.4525	0.216128	-0.40769	0.237104	-0.14798	-0.15453	-0.2286
Q96JK2	DCAF5	-0.48488	0.313796	0.170548	-0.02339	-0.25395	-0.45455	0.202449	0.104941	0.369718	-0.72722	-0.22956	-0.04825	0.074721	-0.15906	-0.27388	-0.40182	-0.31443	0.022592	0.691966
Q96JM2	ZNF462	0.264713	-0.17922	-0.52655	-0.01978	-0.71542	-0.45027	0.079173	1.02556	-0.45561	-0.56512	0.296031	-0.61663	-0.16276	-0.51451	0.807441	-0.89523	-0.14467	0.540201	0.347222
Q96JM3	CHAMP1	0.196881	-0.44758	-0.27191	-0.28988	-0.13401	-0.055	0.00719	0.843889	0.45067	-0.27423	0.137317	-0.6351	-0.75761	-0.5256	0.700723	-0.55986	0.140743	1.064225	0.225133
Q96JP5	ZFP91	0.565972	-0.31917	-0.18543	-0.40702	-0.72459	0.330577	0.030002	0.094074	0.447872	-0.42928	0.363269	0.155493	0.092389	-0.48864	0.295677	-0.05788	-0.19076	0.087518	-0.2024
Q96JQ0	DCHS1	-0.85504	0.501782	0.218237	-1.21673	-0.41387	-1.14321	0.120049	-0.57512	0.265527	-0.09057	-0.75171	-0.13231	-0.01698	-0.17519	-0.7465	-0.14613	-0.24859	0.792364	1.125766
Q96JQ2	CLMN	-0.21402	0.007203	0.166456	0.873831	0.547145	0.726674	-0.00886	0.823567	0.341055	0.439315	-0.84525	-0.03749	-0.41429	0.162506	0.529584	0.189727	-0.14997	0.017214	-0.33991
Q96JY6	PDLIM2	-0.88521	-0.28367	-0.02828	-0.56372	-0.44863	-0.83338	-1.17602	-0.29067	0.921712	-0.00964	-1.21579	-0.5862	1.228479	0.415225	-0.24353	0.609205	-0.42743	0.777957	0.913556
Q96K17	BTF3L4	0.414553	-0.45595	-0.64987	-0.32192	0.696359	-0.08885	-0.4442	0.368589	-0.19087	-0.05341	0.389037	-0.24655	-0.22446	-0.39143	-0.62495	0.464251	-0.15245	0.132347	-0.85908
Q96K19	RNF170	-1.0431	0.835613	1.01166	-0.33282	-0.17802	0.518535	0.603355	-0.77219	-0.0062	-0.03804	-1.32463	-0.05634	0.565306	0.261033	0.564892	1.191989	0.406756	0.257895	-0.18677
Q96K21	ZFYVE19	0.840218	-1.1151	-0.14112	-0.44445	-1.09957	-0.18155	-0.24803	-0.83267	-0.72049	-0.32244	0.650891	-0.62734	-0.45033	-0.66429	-0.14156	2.033388	-0.1173	0.057733	-0.32004
Q96K37	SLC35E1	-1.02773	0.425119	0.797991	-0.38919	-0.57844	-0.67535	0.5194	1.399717	0.05305	-0.46928	-0.00083	-0.35046	0.223841	-0.36985	-0.38014	0.391977	0.034121	0.632104	0.437121
Q96K76	USP47	-0.49653	-0.02072	-0.1183	-0.61164	-0.14523	0.161551	-0.18554	0.052881	0.426199	-0.02329	0.016166	-0.28073	-0.28272	0.070084	0.026926	-0.61418	0.357201	-0.06996	0.451263
Q96KA5	CLPTM1L	0.170954	-0.25117	-0.04439	-0.23272	0.056458	-0.21513	0.865676	-1.03018	0.246041	0.972202	-0.45691	-0.2495	0.179705	-0.27404	-0.02593	-0.34576	0.017518	-0.46752	-0.63677
Q96KB5	PBK	0.944865	-0.05938	0.405209	0.006678	0.72613	1.643754	0.191018	0.378927	0.345921	-0.80674	-1.21396	0.003728	0.034391	0.04274	-0.18996	-0.12153	1.952491	0.318248	-0.01773
Q96KC8	DNAJC1	-0.41945	0.871658	0.139295	-0.27836	0.136403	0.070642	0.094454	-0.29511	0.360072	-0.40397	-0.4474	0.039605	0.762644	-0.15929	-0.91146	0.348911	-0.16666	0.635544	0.501361
Q96KG9	SCYL1	-0.12462	0.255611	0.421212	0.012935	0.590281	0.665479	0.511426	0.208945	0.056721	0.12735	-0.33375	0.073155	0.328265	-0.059	-0.18373	0.512895	0.240268	0.567579	0.448626
Q96KM6	ZNF512B	0.253338	-0.18231	-0.31919	0.863159	-0.34531	0.346019	-0.17244	0.394976	-0.29182	-0.59127	-0.08197	-0.57677	-0.25891	-0.9372	0.86553	-0.69947	-0.34203	0.060131	-0.29938
Q96KN1	FAM84B	0.131228	-0.38686	-0.10167	-0.21866	-0.36634	-0.9873	-0.69395	0.078124	0.984465	0.211879	0.115732	0.068637	0.369591	0.393332	-0.28719	0.633348	-0.09836	0.51394	-0.82333
Q96KP1	EXOC2	-0.33709	0.305679	0.030573	0.179101	-0.11181	-0.36004	0.21567	-0.25558	-0.2801	-0.00593	0.130414	0.134517	-0.37759	0.444551	0.385786	0.078029	-0.34406	-0.46075	0.230382
Q96KP4	CNDP2	-0.22762	-0.83959	0.979189	-0.45741	0.483319	0.231403	-0.66084	-0.0034	0.425962	0.519708	0.840565	-0.33757	0.987426	-0.30756	-0.48079	0.807976	-0.56101	-0.01801	0.229188
Q96KQ7	EHMT2	0.730357	-0.30351	0.102431	0.080103	0.008817	0.588499	0.443831	0.589366	0.163864	-0.47378	-0.12193	-0.52773	0.371747	-0.25709	0.145056	-0.03648	0.009484	0.190009	0.116769
Q96KR1	ZFR	0.300637	-0.31118	-0.22675	-0.10889	-0.10111	-0.00361	-0.38265	0.346222	-0.18683	-0.08126	0.620918	-0.61805	-0.5149	-0.76667	0.200968	0.1013	-0.37948	-0.01816	-0.08993
Q96L58	B3GALT6	0.259524	0.464342	0.382214	-0.67393	0.068485	0.075123	0.339107	-0.89287	0.136179	0.27296	-0.25392	-0.61256	0.141919	-0.43055	0.591258	0.396202	0.37985	1.118792	0.748475
Q96L91	EP400	0.336985	0.452727	-0.17155	-0.24433	0.009585	0.112182	0.343986	0.607762	0.047142	-0.20276	0.012207	-0.17581	0.081614	-0.08624	0.362505	-0.14343	-0.25252	0.042609	0.004598
Q96L92	SNX27	-0.43036	0.69104	0.5741	0.045467	0.0364	-0.18997	-0.48324	0.019146	-0.04293	0.838925	-0.27245	0.941729	-0.14482	0.983594	0.498608	-0.08485	0.799654	0.373456	-0.40015
Q96L93	KIF16B	-0.33363	-0.2927	0.208083	-0.35269	-0.17044	-0.36437	0.205785	0.110572	0.04419	0.003581	-0.07994	0.162103	0.489169	0.461331	0.194035	-0.24354	0.306338	-0.05146	-0.74036
Q96LA8	PRMT6	1.012375	-0.11817	-0.38111	0.19214	-0.6775	-0.65224	-0.06589	0.597791	-0.27553	-0.47741	1.148217	-0.93441	-0.16932	0.101331	-0.97251	0.055258	-0.42354	-0.82981	-0.07929
Q96LB3	IFT74	0.228887	-0.30843	-0.51607	0.045532	-0.38404	-0.16697	-0.11794	0.134668	0.040847	-0.72016	1.568255	-0.76193	-0.3358	-0.75334	-0.1815	-0.36051	-0.47164	-0.45649	-0.06213
Q96LD4	TRIM47	0.374737	-0.4518	0.19415	-0.77833	0.206562	-0.11533	0.122943	0.821659	-0.14527	-0.02188	-0.39504	-0.10761	0.178793	0.002422	0.424731	-0.1526	-0.68153	-0.60024	-0.36294
Q96LI5	CNOT6L	0.294599	0.145308	-0.10369	-0.87865	0.209731	0.496186	-0.02812	-0.51967	0.166293	-0.16621	0.145186	0.021894	-0.26916	-0.32809	-0.69863	0.099202	0.06922	0.349162	-0.26575
Q96LJ7	DHRS1	0.170645	-0.4809	-0.28984	0.80027	-1.78358	-2.03307	-0.29201	0.144617	-0.55665	-0.3008	0.753664	-0.26734	-0.75078	-0.45805	-0.2539	-0.65056	-2.16976	-2.11526	-2.74452
Q96LT7	C9orf72	-0.37492	0.90258	0.215599	-0.54716	-0.00238	-0.74003	0.34479	-1.45435	0.209661	-0.35434	-0.09485	0.75841	-0.09143	0.98601	-0.45872	-0.5102	0.593238	0.07779	-0.07704
Q96LW7	CARD19	-0.61479	0.351011	0.244156	-0.20709	-0.06296	-0.1648	0.325715	-0.03897	0.151505	0.202088	0.315182	-0.14665	0.177113	0.303689	0.73739	0.734994	-0.41702	-0.18485	0.002861
Q96LZ7	RMDN2	-0.68038	0.217815	0.012875	-0.12945	-0.68154	-1.00848	-0.20379	-0.66423	-0.10316	-0.76846	0.440571	-0.34177	-0.46546	-0.31851	0.030817	-0.7851	-0.81213	0.099391	0.032827
Q96M27	PRRC1	-0.7675	0.635374	-0.19013	-0.16228	0.563949	0.362777	0.524056	-0.27447	0.365652	-0.05319	-0.35846	-0.10691	0.37096	-0.27333	1.122387	0.740657	0.529134	0.682952	0.733403
Q96M96	FGD4	0.505081	0.051965	-0.31733	-0.42735	0.485463	0.359075	-0.03056	0.819037	0.095571	0.1018	-0.19491	0.176511	-0.22563	0.908541	0.382264	-0.08899	0.370545	-0.47164	0.36805
Q96ME1	FBXL18	-0.79067	0.117085	0.218163	0.172462	-0.16749	0.130743	0.078097	0.606408	-0.13188	0.558809	0.227732	-1.03622	-0.02102	-0.58227	0.521846	-0.00729	-0.38423	-0.43338	0.356866
Q96ME7	ZNF512	-0.16504	-0.30537	-1.28826	-0.27507	-1.67523	-0.69493	-0.89362	0.562909	0.40007	-0.63311	0.516513	-1.29942	-0.32949	-0.07237	-0.14014	0.023909	-0.67636	-0.07154	-0.24656
Q96MG7	NSMCE3	0.49236	-0.15023	0.147162	0.304753	-0.27164	-0.05931	0.287344	0.42699	-0.26174	-0.45444	-0.03281	-0.32331	0.255463	-0.28612	0.325097	0.391189	-0.45366	-0.38952	-0.00901
Q96MG8	PCMTD1	-0.56686	-0.21955	-0.08584	0.291149	-0.20342	-0.23752	0.130048	-0.98048	-0.30048	-0.84993	0.399991	0.450188	-0.31859	-0.26675	1.506167	0.143209	-0.54662	-0.46436	-0.22495
Q96MH2	HEXIM2	0.935407	-0.32539	0.190624	-0.47105	0.637812	1.323687	-0.1366	-0.11415	-0.19657	-0.14126	0.308831	-0.47658	-0.09387	-0.64525	0.110285	0.166286	-0.12379	-0.72375	-1.12722
Q96MH6	TMEM68	-0.00477	-0.11126	-0.18351	0.135092	-0.16948	-0.18642	-0.2597	-0.09299	-0.15952	-0.13036	1.028206	0.416108	-0.06078	0.003273	0.676798	0.119463	0.282112	-0.07237	-0.39953
Q96MK3	FAM20A	-1.48061	1.006654	0.369357	-0.50802	0.013123	-0.2407	0.234071	-1.24544	0.416728	-0.29669	-1.44322	0.319138	0.447027	0.668238	1.978208	0.854118	0.26968	0.516841	0.664761
Q96MM6	HSPA12B	-1.22876	0.295395	0.216199	-1.18176	-0.33382	-1.28069	0.566757	-0.92813	1.313873	-0.78971	-0.91426	-0.96702	-0.71641	-0.42372	0.165127	-0.72341	-1.2145	0.272504	-0.29038
Q96MU7	YTHDC1	0.695229	-1.08682	0.052394	0.014911	-0.02722	0.316671	-0.7855	0.381634	0.478476	0.115968	0.766087	-0.44038	-0.26665	-0.83146	0.150601	-0.12825	-0.37343	0.533315	-0.11344
Q96MW1	CCDC43	0.56295	-0.24612	-0.50127	-0.50224	-0.06713	0.598728	-0.46134	-0.40859	-0.04216	0.516929	0.411561	0.083804	0.137111	0.066614	0.083595	1.499188	0.055244	0.003606	-0.35973
Q96MW5	COG8	-0.8199	0.574097	0.040517	0.01235	0.061508	0.347122	0.499216	-0.48407	0.284778	0.155758	-0.02826	0.023697	0.337992	-0.17572	0.925221	0.157724	-0.05749	0.544159	0.473996
Q96MX6	WDR92	-0.09588	0.042112	0.036578	-0.36581	-0.09624	-0.01151	0.111966	0.646385	-0.01632	-0.04962	0.563471	-0.47849	-0.17668	-0.1742	0.405988	-0.11555	-0.19757	-0.8772	0.32403
Q96MY1	NOL4L	1.034752	-0.49572	-0.31498	-0.44567	-0.57293	-0.24767	-0.06829	0.567856	0.017324	-0.04388	-0.29921	-0.99543	-0.03399	-0.70085	-0.15654	-0.0488	-0.83872	-1.13389	0.832074
Q96N11	C7orf26	0.103106	-0.09122	-0.00765	-0.12234	-0.02901	0.67326	0.095655	0.902648	0.074027	-0.16147	0.213577	-0.12205	-0.10723	0.158727	0.262302	-0.17384	-0.11462	-0.16838	0.203855
Q96N66	MBOAT7	0.36853	0.506351	-0.54951	-0.44464	-0.13281	-0.38244	0.197792	0.771898	-1.44327	0.567681	0.467455	0.468535	-0.41449	0.613635	0.727458	-0.47711	0.047538	-0.72331	-0.7645
Q96N67	DOCK7	0.17916	0.053946	0.184805	-0.0891	-0.25912	-0.28076	-0.06523	0.202571	0.340797	0.113878	-0.47269	-0.03163	0.514572	-0.25332	-0.19073	-0.3176	0.220368	-0.48569	0.302634
Q96NB2	SFXN2	0.39314	-0.23891	-0.15391	0.073101	2.086215	1.638536	-0.21579	-0.75481	-1.27797	-1.59321	0.218216	0.512815	-0.46881	-0.54117	-1.09387	-0.26159	-0.74118	-1.22012	-1.24494
Q96NB3	ZNF830	0.066861	-0.27638	-0.1396	-0.02117	0.195852	0.008499	-0.37395	0.248202	0.096074	0.081034	0.013066	0.116891	0.0094	-0.3488	-0.26373	0.289219	-0.14283	0.058197	-0.09988
Q96NC0	ZMAT2	0.552617	-0.28777	-0.02579	0.048784	-0.36116	0.637741	-0.97895	0.556762	-0.4134	-0.63883	0.5756	-0.8108	-0.15242	-0.53428	0.551098	-0.03831	-0.21973	0.369339	-0.25938
Q96ND0	FAM210A	0.665101	-0.3855	0.226195	0.2507	0.097177	-0.14574	-0.07891	-0.4314	-0.09439	-0.06465	0.275989	0.131949	0.05252	0.024582	1.524381	0.593224	0.942412	0.316138	-0.75479
Q96NE9	FRMD6	-0.18905	0.004709	-0.37207	-0.58853	-0.19908	-1.02161	0.148466	-0.8183	-0.31096	-0.97342	-0.2001	-0.66172	-0.37576	0.559029	-0.55583	-0.61839	-0.80431	-0.62397	0.207093
Q96NT0	CCDC115	-0.06113	0.240265	0.217431	-0.093	-0.35869	0.084603	0.138371	0.049576	-0.20288	-0.04689	0.282201	0.300269	0.088732	0.191228	0.056399	0.603931	0.55355	0.695685	-0.0355
Q96NW4	ANKRD27	-0.43664	0.155249	0.273899	0.067627	1.416761	1.468969	-0.08627	-0.27396	-0.06456	0.116627	-0.65002	0.651319	0.049014	-0.00691	0.323867	-0.12575	1.129877	0.22702	-0.31657
Q96NY7	CLIC6	-1.1575	-0.66934	-0.04724	-1.68281	-3.50186	-1.75347	-0.76157	-0.66974	1.197203	-0.67025	-1.13358	-1.52057	-1.00162	-0.46631	-0.08849	-1.73275	-0.83863	-2.35132	-0.82704
Q96NY8	NECTIN4	-0.58782	-0.74431	-0.39136	-0.2421	-1.88273	-1.53721	-0.40171	-1.39101	-0.10659	-1.20926	-0.78757	-1.67205	-0.31177	-0.71535	1.865884	0.207479	-0.88111	-1.22989	-1.28191
Q96P11	NSUN5	0.425966	0.25882	0.205408	-0.16965	0.804423	0.849781	0.060264	-0.07868	-0.15841	-0.29674	0.407393	0.237623	-0.35113	-0.18334	1.069213	-0.29371	-0.79537	-0.22837	-0.70885
Q96P16	RPRD1A	0.329071	-0.44921	-0.3152	0.244963	-0.12845	-0.0208	-0.49973	-0.26205	-0.45602	-0.33217	0.492753	-0.46699	-0.17241	-0.37788	0.190466	-0.28828	-0.46977	-0.99207	-0.62093
Q96P44	COL21A1	-3.5067	-1.01133	-1.12654	-2.63582	-1.68311	-3.95993	-0.0684	0.212422	0.652194	-2.87843	-2.79784	-1.53003	-2.33234	-0.85781	-0.61742	-2.98243	-0.47371	-1.41438	-1.83921
Q96P47	AGAP3	-0.50532	-0.10273	-0.10576	-0.18536	-0.33026	-0.05559	-0.12384	0.101614	-0.0743	0.002426	-0.15905	-0.57771	-0.08889	-0.36026	0.467656	-0.32232	-0.80564	-0.21696	-0.66691
Q96P48	ARAP1	-0.46521	0.139987	0.644408	-0.53549	0.264242	0.25874	0.339636	-0.07999	0.306188	0.275717	-0.68366	0.611348	0.567203	0.913563	-0.17758	-0.12833	0.114613	0.164932	0.4846
Q96P63	SERPINB1	-2.00005	1.729989	0.104694	-1.03083	-1.06194	-0.62404	0.792527	-0.34258	0.693883	-1.10119	-1.23494	-0.56305	-0.03075	-0.06784	-1.14005	-0.52245	-0.52102	-1.62143	-0.22284
Q96P70	IPO9	0.687251	-0.05037	-0.20853	-0.2443	-0.1022	0.461177	0.310099	0.366558	0.507827	-0.22197	-0.6804	-0.45985	-0.27794	-0.21785	0.458523	-0.5817	-0.58592	-0.78204	0.577736
Q96PD5	PGLYRP2	-0.8441	-0.01533	-1.19399	-0.66897	-0.1356	-0.91015	0.951695	-0.54332	0.040847	-1.68422	-0.56358	0.852952	-0.03841	0.728211	-0.34115	-1.14229	0.164442	-0.09324	-0.6374
Q96PE1	ADGRA2	-0.2671	0.352296	0.153031	-0.38186	-0.3095	-0.81094	0.434777	-0.93396	0.227049	0.060303	-0.43404	-1.0495	0.017237	-0.38665	0.019682	0.544855	-0.81442	0.211913	0.530559
Q96PE2	ARHGEF1	-0.13962	0.06729	0.229766	-0.58145	0.019879	-0.19275	0.407858	-0.14908	-0.02393	-0.33695	-0.3038	-0.48544	0.313134	-0.28533	0.777814	0.204483	-0.47545	0.430569	0.358768
Q96PE3	INPP4A	-0.3632	0.328741	0.596623	-0.19138	0.207824	0.050362	-0.11235	0.126054	-0.0098	0.066449	-0.92	0.115647	0.10618	0.451092	0.076864	-0.52666	0.748283	-0.00253	-0.18089
Q96PK6	RBM14	0.381318	0.27502	-0.17378	0.316245	-0.30346	0.403639	0.127782	0.428218	-0.51784	-0.12583	0.477178	-0.56774	-0.15899	-0.4806	-0.03944	0.028569	0.208491	0.676187	-0.28093
Q96PP8	GBP5	-2.35187	1.862764	1.486622	-0.87382	0.392946	-0.15051	0.641596	-1.50016	-0.17285	-1.13234	-1.32083	0.936097	1.688308	0.951798	-1.29048	-1.49901	1.685847	0.55684	-1.54672
Q96PP9	GBP4	-1.34166	1.258477	1.697453	-0.40895	-0.08841	0.501877	-0.51547	-1.3869	-0.55631	-0.01639	-0.85715	0.372771	0.131085	0.258796	-1.29122	0.139046	0.450109	0.28437	-1.46258
Q96PQ0	SORCS2	-0.30534	0.439927	0.307044	-0.63118	0.005426	-0.69225	0.731545	-0.24588	0.32317	-0.73032	-1.00285	0.058885	0.365724	-0.22587	0.757958	-0.33429	0.131809	0.551577	1.439076
Q96PU4	UHRF2	-0.00766	-0.13258	-0.05491	-0.66799	-0.05504	0.790565	-0.0519	-0.14313	0.15661	-0.54005	0.163751	-0.17882	0.067158	-0.63957	-0.12926	-0.28074	-0.22329	-0.32936	-0.01153

Q96PU5	NEDD4L	-0.25011	-0.05226	0.524589	0.053243	-0.37167	-0.31662	-0.75906	-0.05467	0.062392	-0.28926	-0.19847	0.323099	-0.12072	0.194616	-1.45548	0.331405	0.516697	0.24638	0.456525
Q96PU8	QKI	-0.39222	0.35688	0.307208	-0.69403	0.527788	0.35882	-1.83038	-0.86256	0.850383	0.746442	0.40247	0.306246	0.040446	0.404115	-0.39361	-0.39574	0.037878	1.120702	0.299942
Q96PV6	LENG8	0.094754	-0.12718	-0.26934	-0.09933	-0.60409	-0.07348	-0.04772	0.029457	0.110994	-0.33348	0.878667	-0.6505	0.189441	-0.48709	-0.00166	0.194629	-0.70064	-0.01415	-0.61632
Q96PX9	PLEKHG4	1.342546	-0.29901	-0.41111	-0.45426	-0.91947	-0.80348	-0.04781	0.548876	0.314695	-0.51066	0.154644	-0.28426	0.124513	0.031887	-0.38843	-0.057	-0.84906	-0.41469	0.36241
Q96PY5	FMNL2	-0.61727	0.926554	0.273694	-0.81535	-0.14036	-0.69668	-0.16923	0.046809	-0.29964	0.056014	-0.22983	0.993415	0.864189	0.22448	-0.50642	-0.95994	-0.09639	-0.30472	-0.84486
Q96PY6	NEK1	-0.92988	0.221305	-0.09308	-1.23285	-0.12095	-0.53341	-0.1737	-0.38446	0.100986	-0.34606	0.606949	-0.40006	0.08279	-0.35315	-0.57311	-0.0687	-0.59692	-0.17504	0.325592
Q96PZ0	PUS7	0.605938	-0.67064	-0.09724	0.277509	0.354388	0.695341	-0.378	0.503138	-0.03791	0.032701	0.276655	-0.22621	0.263984	-0.2294	0.750136	0.512254	-0.39974	-0.38217	0.305219
Q96PZ2	FAM111A	0.782118	-0.08435	0.262855	0.184638	0.344478	0.471618	0.063126	0.25246	-0.35651	-0.67668	0.35284	-0.97383	-0.41058	0.146863	0.431351	-0.47495	-0.32276	0.082068	-0.61766
Q96Q05	TRAPPC9	-0.10616	0.110166	0.402748	-0.33677	-0.58331	-0.91402	0.02828	-0.53563	-0.34378	0.228012	-0.35873	0.43776	0.541656	0.621698	0.395528	0.133184	0.171522	-0.22525	-0.56541
Q96Q06	PLIN4	-4.59516	-0.83201	-1.47629	-2.74362	-4.21063	-4.35427	0.480107	-5.65641	-1.39469	-1.99687	-3.65206	-3.17118	-1.25988	-3.02714	-1.39248	-1.74736	-3.40174	-0.62971	-3.88634
Q96Q11	TRNT1	1.032284	-0.02404	0.196174	-0.29499	-0.25832	0.021705	-0.15874	0.164509	-0.36019	-0.15994	0.597383	-0.46229	0.114687	-0.31264	0.279456	-0.34145	-0.78482	-0.64256	0.201508
Q96Q15	SMG1	0.097217	0.299552	0.446108	-0.37831	-0.08192	-0.21107	0.449003	-0.52869	0.152204	-0.15377	0.153338	0.267431	0.317728	0.121813	0.147413	0.023442	0.271614	0.022428	-0.24561
Q96Q42	ALS2	-0.36035	0.570549	0.256895	-0.47862	-0.53684	-0.55488	0.333031	-0.13994	0.153769	0.562588	-0.50459	0.476628	-0.20268	0.393456	-0.36254	-0.00811	-0.02822	-0.4435	-0.24603
Q96Q89	KIF20B	0.636109	-0.0935	0.125689	1.063007	0.326617	0.711441	-0.06485	0.769596	-0.3461	-0.25657	0.547373	-0.27502	-0.25306	0.127111	-0.16731	-0.10614	0.203604	-0.09987	0.20852
Q96QA5	GSDMA	-0.59881	1.827231	0.164042	-1.24889	-1.37485	-1.17624	2.281994	-0.55575	0.507765	-0.13972	-0.24649	-0.51097	0.526726	-0.55365	-0.55404	-0.58649	-0.74038	-0.44124	0.162623
Q96QC0	PPP1R10	0.616647	-0.38344	-0.33038	-0.10962	-0.34868	0.001126	-0.53005	-0.2857	-0.44506	-0.38569	0.476981	-0.30275	-0.07502	-0.56088	-0.16577	0.063625	-0.1581	0.651666	0.056421
Q96QD8	SLC38A2	0.035407	0.085539	0.082848	1.354228	0.494125	0.925325	-0.31501	-0.63639	0.064559	0.231006	-0.60332	-0.40958	-0.27698	-0.17147	0.400667	0.286714	-0.07519	-0.35149	-0.8108
Q96QD9	FYTTD1	0.340055	-0.22925	0.094278	-0.38846	0.939778	0.64676	-0.28545	0.529615	-0.09198	0.842499	0.052553	-0.54111	-0.78917	-0.50513	0.44118	-0.29251	-0.03857	0.578352	-0.03591
Q96QE5	TEFM	-0.59976	0.20188	0.043781	-0.17052	-0.11433	-0.23769	0.321735	0.163215	0.013152	-0.40186	0.608169	-0.32139	0.125721	0.361952	-0.341	1.229769	1.140044	-0.24903	0.757427
Q96QF0	RAB3IP	0.761553	-0.0675	-0.0233	0.538887	0.231174	0.387518	0.27411	1.022063	-0.19251	-0.08091	-0.44524	-0.49217	-0.38058	-0.33132	-0.3031	0.178084	-0.12404	-0.85547	-0.03529
Q96QG7	MTMR9	0.152749	0.506466	0.161561	-0.23331	-0.01488	-0.59731	-0.43605	-0.6134	0.309217	0.255662	-0.57771	-0.06811	0.048196	0.364444	-0.47137	0.175576	0.045188	-0.1563	0.33078
Q96QK1	VPS35	-0.33093	0.591879	0.153603	0.414461	0.457612	0.393184	0.215978	-0.39291	-0.15146	0.105875	-0.19377	0.100795	-0.04835	0.28931	-0.01172	0.397976	0.049671	-0.10642	-0.10699
Q96QR8	PURB	0.568464	-0.46136	-0.67279	-0.64152	-0.09511	0.040544	-0.64086	0.334821	-0.07735	-1.15681	0.441921	-0.06414	0.024884	-0.57134	-0.23718	0.557036	-0.18763	-0.75354	-0.86164
Q96QT4	TRPM7	0.455	0.608931	0.191313	-0.3401	-0.15433	-0.74942	0.315098	-0.40493	0.336034	-0.16949	0.570912	0.389728	0.014196	-0.46158	-0.90298	-0.02121	0.190526	-0.31573	-0.23342
Q96QU8	XPO6	0.355169	0.639927	0.41699	0.307358	0.077866	0.005394	0.15045	-0.3595	-0.07446	-0.1372	-0.014	-0.02105	0.129181	0.45774	-0.29519	0.038532	0.336399	0.018288	-0.07996
Q96QZ7	MAGI1	-0.1753	-0.08012	-0.22857	-0.39771	-0.83694	-0.90926	0.131186	0.840793	-0.06325	0.737135	0.195923	-0.67427	-0.37944	-0.70596	0.350953	-1.38431	-0.29307	-0.59276	-0.00675
Q96RE7	NACC1	-0.13695	-0.2949	0.574366	0.915325	-0.78658	-0.6181	-0.20758	0.875902	-0.49642	-0.52428	0.364996	-0.44694	-0.81065	-0.48713	0.86918	0.497602	0.201792	0.445049	-0.00056
Q96RF0	SNX18	-0.85115	0.672422	0.532553	-0.32545	-0.37818	-0.39965	-0.46806	-1.3972	0.322155	0.063583	-0.18845	0.133663	0.57744	0.263267	0.297982	-0.30123	-0.26975	0.601656	0.136237
Q96RG2	PASK	-0.17934	0.148627	0.158894	-0.2477	0.249349	0.333318	0.083188	-0.29299	-0.29133	0.23563	-0.21488	0.164879	0.016265	0.384693	-0.64199	-0.07086	0.342748	0.362508	-1.00994
Q96RK0	CIC	0.345567	-0.19585	-0.16618	-0.71457	-0.36764	0.03135	0.022984	0.075083	-0.05293	-0.17152	0.178033	0.054265	0.168628	-0.33284	0.850044	0.008877	0.398069	0.078144	0.656959
Q96RL1	UIMC1	0.236831	-0.1721	-0.23493	-0.3448	0.298147	-0.19972	-0.58007	0.220467	-0.34709	-0.17376	0.494533	-0.92203	-0.14682	-0.70169	0.043868	-0.26067	-0.7913	0.618634	-0.24266
Q96RL7	VPS13A	0.170407	-0.23533	0.054977	0.266512	0.232697	-0.0634	-0.50044	-0.49245	-0.27168	-0.32438	0.875522	-0.48145	-0.24136	-0.13563	-0.52225	0.54287	-0.62741	-0.63057	0.075047
Q96RN5	MED15	0.095211	0.178595	0.072936	-0.22426	0.180774	-0.01449	-0.14306	-0.24307	-0.15958	0.25822	0.586254	-0.17576	0.130802	0.153004	-0.02589	0.274502	0.372772	0.443285	-0.01656
Q96RP7	GAL3ST4	-1.43257	-0.00602	-0.26388	-0.86856	-0.34786	-0.23283	-0.00671	-0.68904	0.521894	-1.43018	-1.79831	-0.4059	-0.61433	-0.56811	0.196192	-0.87422	-0.80569	-0.0266	-1.60678
Q96RP9	GFM1	0.215859	-0.61363	0.325979	1.130127	1.257195	1.145518	-1.13264	0.555808	0.183078	1.034298	0.012887	0.464568	0.339982	0.018425	2.109613	0.799033	0.09057	-0.55508	0.124827
Q96RQ1	ERGIC2	0.094242	0.058956	-0.39158	0.506023	0.383945	0.048875	1.194309	-0.50038	-0.32848	-0.25272	-0.66021	0.305604	0.164799	0.129537	0.37459	0.173642	0.60898	-0.05118	0.213629
Q96RQ3	MCCC1	0.455883	-0.19257	-0.14095	0.7651	-0.07139	0.018484	-0.22632	0.220086	-0.14965	0.140722	0.453931	-0.30212	-0.3474	-0.24727	2.73025	-0.37286	-0.51825	-0.76777	-0.69441
Q96RQ9	IL4I1	-1.51688	2.448466	1.494902	-0.05954	-0.77922	-1.06404	-0.68326	-0.41715	-0.44262	-0.54356	-1.21732	0.870635	0.751144	1.15557	-0.36986	-1.48467	0.422226	-0.36014	-0.15981
Q96RR1	TWNK	0.760491	-0.20464	-0.54281	-0.12372	-0.19535	-1.17647	-0.63708	-0.15812	-0.21146	-0.12058	0.192819	0.104166	-0.16755	0.077456	0.214449	-0.71759	-0.05217	-0.50315	-0.90183
Q96RR4	CAMKK2	0.301829	-0.1521	0.415843	-0.57988	-0.31711	-0.4393	0.226571	0.167326	-0.07563	-0.05867	-0.46058	0.242158	0.111723	1.182138	-0.3764	0.422064	0.795199	-0.24186	0.381907
Q96RS0	TGS1	0.186714	-0.21706	-0.0364	-0.72912	-0.27687	0.250635	0.096806	-0.16142	-0.24302	-0.82332	-0.18991	0.497187	-0.1212	-0.0606	0.76561	0.313317	0.02432	-0.58031	0.141484
Q96RS6	NUDCD1	0.001584	-0.16916	-0.01309	0.284666	0.161332	0.593938	-0.76446	-0.08284	-1.11106	-0.24507	0.005558	0.527429	1.19222	-0.16261	-0.0271	-0.42776	0.576373	-0.15076	-0.31472
Q96RT1	ERBIN	-0.06931	0.069798	-0.32139	0.014733	-0.65848	-0.46376	-0.24888	0.627531	-0.68863	0.407371	0.296173	-0.43593	0.317457	-0.27338	1.092268	-0.62385	0.020082	0.447554	-0.24734
Q96RT7	TUBGCP6	-0.82024	0.385867	-0.03574	-0.32484	-0.39132	0.109977	0.051076	-0.61822	-0.17909	-0.41551	0.663817	-0.34874	0.031402	-0.5044	-0.22935	-0.19689	0.005978	0.294688	-0.16571
Q96RU2	USP28	0.491506	-0.37168	-0.59209	0.964455	-0.06932	-0.04184	0.194137	0.858478	-0.08913	-0.33614	-0.34979	0.485836	0.890679	-0.11625	0.409086	1.451404	0.011361	-0.42029	-0.62997
Q96RU3	FNBP1	-0.29517	0.371527	0.955751	-0.62599	0.651216	0.849647	-0.08189	-0.89123	-0.31815	0.908541	-0.28594	-0.3973	0.036816	0.255905	0.829362	0.129368	-0.07553	0.836577	-0.7259
Q96RW7	HMCN1	-1.11913	0.570846	-0.06223	-0.86356	-1.29584	-1.45601	0.44386	-0.98301	0.243466	0.936975	0.812671	-0.8473	-0.16497	-0.7069	0.025458	-0.68374	-0.79118	-0.25735	0.466999
Q96RY7	IFT140	0.046904	-0.52469	-0.45786	-0.42493	-0.84901	-0.75983	-0.36662	-0.34709	0.472378	-0.34582	1.535899	-0.68776	-0.91188	-0.09042	-0.34683	-0.3006	-0.83735	-0.49173	0.034626
Q96S06	LMF1	-0.38458	0.251672	0.14162	-0.32553	-0.44672	-0.43483	0.479793	-0.25305	0.040847	-1.15742	-0.59087	-0.0845	-0.12755	0.091423	0.492474	-0.2361	-0.37912	-1.22717	0.302053
Q96S15	WDR24	-0.22785	0.529765	-0.28136	0.301784	0.19913	0.135007	-0.08075	-0.1155	-0.17022	-0.06549	0.899762	-0.03259	-0.46047	-0.1766	0.050137	-0.11284	-0.47987	-0.04339	-0.11478
Q96S19	METTL26	0.480819	-0.98775	-0.82404	-0.74954	0.062955	-0.38691	-1.40829	-0.51655	0.499666	0.889203	0.642645	0.685918	0.551266	0.709217	-0.96296	0.83952	0.798105	0.915607	0.601739
Q96S38	RPS6KC1	-1.09243	0.411666	0.307009	-0.39665	-0.08625	-0.18391	0.513618	-0.12651	-0.31523	-0.12719	-0.0974	0.34597	0.131645	0.438547	0.514272	0.308816	0.192034	0.049761	0.126326
Q96S44	TP53RK	0.377449	-0.66293	-0.44909	0.608325	0.258101	0.254097	-0.17052	0.155027	-0.15741	0.038906	0.643655	0.341182	-0.54292	0.340097	0.26365	-0.26598	0.224673	-0.37166	0.066813
Q96S52	PIGS	0.161668	0.053314	0.22928	-0.04093	-0.034	0.21008	0.780421	0.459431	0.1177	0.600655	0.090424	-0.24149	-0.33247	0.013916	0.271336	0.408356	0.256819	0.181696	0.27234
Q96S55	WRNIP1	0.255897	-0.48088	-1.13764	0.417112	-0.23942	-0.03651	-0.69142	0.695636	-0.67957	-0.39752	0.467152	-0.47376	-0.15986	-0.35399	0.410707	1.079065	-0.78519	-1.12988	-0.59868
Q96S59	RANBP9	0.191392	-0.49843	0.262503	0.266418	-0.03299	0.345497	-0.55011	-0.48624	-0.2926	-0.43048	0.245081	0.386182	0.033915	0.606592	0.529325	-0.32933	-0.44866	-0.32809	-0.29435
Q96S66	CLCC1	0.40455	0.119447	0.251006	0.03617	-0.1709	0.271236	0.238932	0.163881	-0.3781	0.411107	0.019857	-0.73873	-0.31755	-0.12941	0.877558	0.172151	-0.02265	-0.10404	-0.37806
Q96S82	UBL7	0.524278	-0.55417	0.094033	-0.43495	0.014626	-0.27661	0.076847	0.091071	-0.17147	0.549823	-0.27764	-0.43556	0.019886	-0.07591	-0.01855	0.261049	0.196032	0.005349	-0.0399
Q96S86	HAPLN3	-0.9164	-0.56997	-0.58141	-1.79724	-1.37085	-1.15999	-0.10249	-0.14924	0.444293	-0.45351	-1.38906	-1.76429	0.745101	-0.26728	-0.31703	-0.92802	-0.03246	1.944478	-0.05517
Q96S94	CCNL2	0.355711	-0.11604	0.050636	0.310569	0.12729	-0.06102	-0.2363	0.175734	-0.22338	-0.18634	0.041112	-0.12621	0.031509	0.374029	1.123025	-0.34297	0.825774	0.59871	0.401305
Q96S97	MYADM	-0.12948	-0.02369	0.064292	-1.55634	-0.66352	-0.45206	0.222734	-0.44512	-0.43161	-0.67559	-0.07981	-0.12937	-0.02779	0.490462	-0.31015	0.428626	0.130172	0.386744	-0.18771
Q96S99	PLEKHF1	-0.48854	0.489578	0.398751	-0.94281	0.817651	1.135593	-0.19299	-0.28812	0.150932	-0.0347	-0.00879	-0.37795	0.084158	0.1208	-0.11196	0.19625	1.004868	0.485377	-0.25422
Q96SB3	PPP1R9B	-0.54223	0.242396	0.126503	-0.52735	-0.26475	-0.70808	-1.01788	-0.53713	-0.0443	0.436065	0.253445	-0.05041	0.006431	0.281518	-0.29092	-0.40769	-0.3426	0.434506	-6.1E-05
Q96SB4	SRPK1	0.284762	-0.23491	-0.00587	0.126863	0.089448	0.325263	0.330745	-0.32648	0.022513	-0.47779	0.351943	-0.12889	0.476089	0.199744	-0.2087	-0.05915	0.684155	0.032161	0.081073
Q96SB8	SMC6	0.214545	0.287607	0.118817	0.138668	-0.26794	-0.08027	0.258328	0.397452	-0.26342	-0.68937	-0.16896	-0.19186	-0.08858	-0.23554	0.426725	0.231077	-0.28411	-0.12512	-0.07647
Q96SI1	KCTD15	1.287645	-0.12196	-0.0864	-0.18505	0.914938	0.206407	-0.07958	0.136269	-0.11879	-0.07877	0.023526	-0.52362	0.324753	-0.59631	-1.35399	-0.77729	-0.07584	0.268942	1.881787
Q96SI9	STRBP	1.06346	-0.28758	-0.12234	0.160457	-0.23481	0.017039	-0.20846	1.214369	-0.34232	-1.01089	0.654568	-0.88592	-0.43789	-0.91904	0.394404	-0.69634	-0.19186	-0.2359	0.225056
Q96SK2	TMEM209	0.088067	-0.07312	-0.10121	0.054231	-0.13125	0.056613	-0.06663	0.087347	-0.10039	-0.23804	0.479075	-0.16088	-0.41189	-0.42597	0.888433	-0.24059	0.095143	-0.59515	0.107613
Q96SL4	GPX7	0.384658	0.383186	0.368917	-0.56866	1.483465	0.521755	0.23737	-0.48896	0.407926	0.737654	-0.66146	-0.3986	0.451245	-0.68924	-1.70212	0.067673	-0.5904	0.669375	1.691985
Q96SM3	CPXM1	-0.82783	0.087438	-0.09173	-1.8749	-0.6882	-0.88283	-0.46627	-0.35971	0.730221	0.092618	0.710394	-0.72611	0.276752	-0.45044	-1.06827	-0.93184	-0.80817	0.906865	1.12417
Q96SN8	CDK5RAP	0.449817	-0.37825	-0.07324	-0.34202	0.732292	0.634286	-0.24299	0.407835	-0.28189	-0.15758	-0.04386	-0.57021	-0.26109	-0.12758	0.399494	-0.20851	0.213358	0.695797	-0.14653
Q96SQ9	CYP2S1	0.384146	-0.2044	1.034861	0.047311	-1.07113	-0.2669	0.82465	1.497199	-0.14498	0.604714	-0.0974	0.955669	0.541707	-0.6747	-0.7705	-0.93315	-1.07452	-1.79213	-1.79625
Q96ST2	IWS1	0.794393	0.023316	0.215677	-0.1617	-0.30891	-0.04262	0.283169	0.555975	0.639275	-0.26716	-0.12661	-0.30017	-0.05356	0.090222	0.397322	0.205148	0.079271	-0.14561	-0.11914
Q96ST3	SIN3A	0.619502	-0.2399	-0.10003	-0.01931	0.024924	0.228871	-0.20089	0.662426	-1.08948	-0.50752	0.409799	-0.33865	-0.3496	-0.15047	0.039686	-0.24499	-0.0734	0.018473	-0.11822
Q96ST8	CEP89	0.028788	-0.05628	-0.17599	0.045204	0.153012	0.164333	-0.07088	0.669201	-0.01396	-0.68372	0.787895	-0.52737	-0.09458	-0.45941	0.446627	-0.10922	-0.25305	0.028876	0.844716
Q96SU4	OSBPL9	0.257901	0.044079	0.501699	0.373874	0.144062	0.398247	-0.18254	-0.02083	0.066625	0.650431	0.078978	-0.29519	0.137554	0.088308	-0.02	0.621938	0.162361	0.172764	-0.19657
Q96SW2	CRBN	-0.07027	-0.05432	-0.17369	-0.49003	-0.40702	-0.58761	-0.80362	-0.26053	1.037484	-0.20708	0.402361	-0.11895	0.559016	-0.01585	0.19875	0.073759	-0.19388	0.214622	-0.04483
Q96SY0	INTS14	0.654657	-0.34085	-0.24434	0.479394	-0.56881	-0.17267	-0.34617	1.052263	-0.17185	0.655644	0.824934	-0.7204	-0.32819	-0.06037	0.465166	0.460005	-0.16341	0.20638	-0.43542
Q96SZ5	ADO	-0.05688	0.219893	-0.81904	-1.30246	0.657282	0.161632	-0.31102	-0.7189	-0.03075	0.425305	-0.09599	-0.14369	-0.11896	-0.3153	-0.15045	0.076209	-0.07883	-0.00972	-0.21131
Q96SZ6	CDK5RAP	-0.05633	-0.07419	-0.02884	0.184714	-0.06662	0.255976	-0.21945	0.239568	-0.15883	-0.04976	-0.14427	0.070231	0.131922	-0.28514	0.435131	0.196413	0.258803	-0.50835	-0.17126
Q96T23	RSF1	0.790385	-0.45919	-0.59615	0.008416	0.542321	1.106451	0.015062	0.792325	-0.12878	-0.14948	0.057368	-0.80238	0.220878	-0.8541	0.282575	0.640052	-0.59905	0.098261	-0.14249
Q96T37	RBM15	0.583273	0.142121	0.355466	0.060797	-0.08992	0.251082	-0.53931	0.62551	-0.0231	0.101281	0.377561	-0.23464	-0.03418	-0.20385	0.466778	-0.09693	0.224476	0.760955	-0.32754
Q96T51	RUFY1	-0.31805	0.580052	0.253955	0.335395	0.689945	0.833708	-0.10866	-0.82307	-0.34501	0.117278	-0.67474	0.622632	1.489445	0.61677	0.390724	0.137503	-0.42587	-0.48028	-0.27552
Q96T58	SPEN	0.401817	0.016219	0.457712	-0.37341	-0.12435	0.098441	-0.1766	0.67097	-0.20741	-0.00981	0.338683	-0.56247	0.135975	-0.50303	0.455437	0.06831	-0.40206	-0.04941	-0.18288
Q96T60	PNKP	1.183874	-0.71758	-0.86289	0.632027	0.176109	0.423328	-0.10873	0.164237	-0.38509	-0.3477	-0.02637	0.037212	-0.31453	0.130702	1.310436	-0.58001	-0.1022	-0.32665	-0.65299
Q96T66	NMNAT3	-0.24645	-0.17858	-0.2607	0.372865	-0.36605	-0.7224	-0.16776	0.206999	-0.084	-0.35733	-0.14821	0.73571	-0.39399	0.235498	2.114018	0.822252	-1.35475	-1.26655	-0.97216
Q96T76	MMS19	0.300957	0.475852	0.191435	-0.47168	-0.30409	0.030266	-0.03562	-0.22776	-0.09282	0.446788	-0.44201	0.385405	0.347051	0.006723	-0.23756	-0.16934	0.469957	-0.26282	0.332576
Q96T83	SLC9A7	-0.43026	-0.13737	-0.04934	0.427456	-0.83322	-0.25923	-0.08528	0.043071	0.048469	2.358028	-0.40412	-0.66233	-0.50478	-0.06716	-0.25344	0.679507	-0.10754	0.159586	0.250324
Q96T88	UHRF1	0.771546	-0.18627	-0.14668	0.094123	1.279499	1.542333	-0.35924	0.236396	-0.45542	-0.38044	-0.48307	-0.43442	0.053794	-0.40742	-1.04692	-0.49986	-0.00594	-0.0311	-0.77983
Q96TA1	FAM129B	0.074255	0.026189	0.183821	-0.12328	-0.09277	0.040009	-0.5924	-0.26998	-0.36691	-0.3271	0.286818	-0.38555	0.132049	-0.35387	-0.5642	-0.06475	0.284592	-0.23446	-0.40657
Q96TA2	YME1L1	0.235421	0.101456	0.052689	0.377551	0.240597	0.406832	0.244746	0.049624	-0.25303	0.194111	-0.12384	-0.35119	-0.21638	-0.2599	0.438399	0.690019	0.127609	0.022275	0.179761
Q96TC7	RMDN3	0.305773	0.704393	-0.05374	0.421389	0.036316	-0.10142	-0.09895	-1.31357	-0.14535	-0.18909	1.212537	-0.29195	-0.09767	-0.38985	0.239464	0.501086	-0.30029	0.15335	-0.22806
Q99417	MYCBP	0.658578	-0.54933	0.458326	0.93638	1.101206	1.567247	-0.15475	0.870212	-0.32066	0.333601	1.037342	-0.14369	0.652487	-0.0761	0.797412	1.125203	0.706201	0.305859	-1.04881
Q99418	CYTH2	0.396319	-0.02983	-0.05399	-0.13778	-0.22031	-0.17672	0.074017	0.277875	-0.83811	-0.05694	0.48849	-0.36659	-0.07561	0.15101	0.723125	-0.33104	0.101419	0.535119	-0.13017
Q99424	ACOX2	1.241893	0.131041	-0.00828	-0.63498	-0.00034	-1.16749	-0.06964	-0.5352	0.040847	1.707159	1.29762	0.216734	0.229202	-0.48081	-0.89679	-1.11448	-0.10314	0.2466	0.213307
Q99426	TBCB	-0.0703	-1.4658	-0.1368	-1.10935	0.883105	-0.21266	-0.71005	1.016539	-0.05149	0.761106	-0.65845	0.274827	0.623654	0.250818	0.259905	-0.0345	1.1027	0.354405	1.877651
Q99436	PSMB7	0.728155	-0.62783	-0.32465	-0.04258	0.142329	0.082029	-0.86452	0.354702	-0.06411	0.065051	0.523509	-0.80057	0.00896	-0.62101	-0.01323	0.21258	-0.29983	-0.13125	0.341714
Q99439	CNN2	-1.15653	-0.89073	-0.62266	-1.32429	-1.60546	-1.10571	-2.06735	-1.12792	0.942996	-0.04962	-0.87774	-1.30545	0.318956	-0.3483	-1.32244	0.332827	-0.76012	0.388129	1.441114
Q99442	SEC62	-0.11333	0.121307	-0.41938	0.769248	0.873951	0.403345	0.294702	-0.48843	-1.12115	-0.59213	-0.83589	0.353867	-0.04337	0.654419	0.581899	-0.38643	0.689413	0.583266	0.206296
Q99447	PCYT2	0.12776	-1.01101	-0.64744	0.017269	0.521066	0.299381	-1.40598	0.102494	0.040847	-0.07447	-0.06912	-0.15299	-0.21951	0.009127	0.404155	0.592255	-0.05974	-0.10356	-0.65097
Q99459	CDC5L	0.469349	-0.13413	0.065211	0.172396	-0.19616	0.278806	-0.13662	0.707509	-0.40381	-0.22881	0.562355	-0.56485	0.064349	-0.55109	0.053499	-0.04333	-0.3517	0.392111	0.021276
Q99460	PSMD1	-0.17053	0.456715	0.471017	-0.06817	-0.10551	-0.05396	0.24128	-0.01826	-0.15894	0.167315	0.044575	0.209974	0.226159	0.260038	-0.19074	0.319925	0.282756	-0.00979	0.356234
Q99470	SDF2	-0.22857	0.088035	0.036323	-0.05594	0.727598	-0.53702	1.81E-05	0.046763	2.115335	-1.09262	-1.32904	-0.05413	0.091726	-0.18488	-0.53097	-0.70445	-0.02142	-1.04604	-0.79066
Q99471	PFDN5	0.536594	-1.02566	0.627215	-0.19179	0.695238	0.837142	0.477197	0.855171	0.040847	0.360247	-0.27216	-0.21337	0.306308	0.046529	0.202512	0.706101	0.125949	-0.412	-0.17739
Q99487	PAFAH2	-0.48776	0.452747	0.193679	0.229732	-0.1458	0.018813	0.547295	-0.47799	-0.10586	-0.07107	-0.09731	0.334428	-0.17989	0.301003	-0.28328	-0.25607	-0.48989	-0.20119	-0.17895
Q99496	RNF2	0.479043	0.401044	0.092432	0.01515	0.127911	0.513717	0.931848	0.885538	-0.93321	-0.1446	-0.04156	-0.27261	-0.21348	-0.25993	0.074733	-1.21443	-0.0145	-0.34019	0.305901
Q99497	PARK7	-0.90585	-0.89899	-2.13961	-1.00713	1.987344	-0.33271	-0.05146	0.400287	1.716711	1.768964	-0.22165	-0.84392	-0.48381	-0.25812	-0.71527	-0.00729	0.214266	0.249666	-1.05692
Q99504	EYA3	-0.04599	-0.19193	0.189185	-0.09699	0.106597	-0.33598	-0.23088	0.776222	-0.17086	-0.0504	-0.29887	-0.34884	-0.09549	-0.35033	0.108818	0.047447	-0.10964	-0.17318	0.241439
Q99519	NEU1	-0.33119	-0.15159	0.332005	-0.51633	0.80011	0.511153	0.061464	0.387994	0.212639	1.348493	-0.25837	0.491131	0.281557	-0.18516	-0.16398	-0.82918	0.799394	1.331809	-0.43334
Q99523	SORT1	0.127027	-0.30919	-0.51541	0.288629	-0.59688	-0.16802	0.049658	0.822677	-0.59624	-1.15631	-0.60698	-0.90226	0.174454	-0.42873	0.203763	-1.17524	-1.30303	-2.85405	-1.36991
Q99536	VAT1	-1.10063	-0.5329	-0.22717	-0.88992	0.274193	-0.60101	0.477889	-0.66271	0.247316	0.035008	-0.977	0.182842	0.485161	0.566368	-0.24715	-0.29022	-0.07967	0.763718	0.300648
Q99538	LGMN	-1.49549	2.324099	1.343972	-0.91054	0.967628	0.048642	1.745214	0.142087	0.527834	0.186919	-0.87272	0.597927	0.804993	-0.02661	-0.86399	-0.70296	-0.15326	0.007559	-0.75063
Q99541	PLIN2	-1.56003	1.615402	-0.86082	-0.42021	-0.2796	-2.07252	-0.40815	-0.37054	-1.41912	-0.65333	-2.89698	3.273952	1.553544	2.255799	-2.31278	-2.30794	1.694231	-0.71512	-1.49696
Q99542	MMP19	-1.78771	0.389532	-0.80187	-1.3012	-0.47886	-0.03309	0.062921	-0.74416	-0.47261	-0.99859	-0.42335	-0.10799	-0.01373	0.288016	-0.44162	-0.49982	-0.08607	-0.09955	-1.1054
Q99543	DNAJC2	0.225319	-0.48297	0.185976	0.229304	0.124673	0.803804	-0.67806	0.266164	0.84854	0.079206	0.22717	-0.50758	-0.57245	-0.07163	0.590897	0.79607	-0.25625	-0.59078	-0.31819
Q99547	MPHOSPI	0.756561	-0.30934	-0.13477	0.042863	0.414902	0.501743	-0.40428	0.360338	-0.3903	0.26281	0.19147	-0.55383	-0.16942	-0.51091	-0.36739	0.458135	-0.24102	0.541604	0.340566
Q99549	MPHOSPI	0.634537	-0.28313	-0.13017	-0.12598	-0.05095	0.609583	-0.43278	0.498044	0.292095	-0.15183	0.401462	-0.7316	-0.02973	-0.24597	0.538454	-0.47039	0.076957	0.216676	0.093872
Q99567	NUP88	0.380898	-0.01481	-0.13177	0.053292	-0.11034	0.078018	0.158021	0.273829	-0.29481	-0.25413	0.464298	-0.05848	-0.28933	-0.02207	0.83071	-0.16646	-0.07304	-0.2986	0.33068
Q99569	PKP4	0.832065	-0.20936	-0.56865	-0.20922	-0.81276	-0.86395	-0.01639	-0.19005	-0.36535	-0.22855	0.692267	-0.6575	-0.30322	-0.63216	1.051691	-0.09109	-0.56702	-0.78762	-1.25908
Q99570	PIK3R4	-0.15895	0.531139	0.496946	-0.00097	0.3502	0.355302	0.559154	-0.55295	0.680563	0.488624	-0.46541	0.516015	0.308418	0.469171	-0.11852	0.259719	-0.06988	-0.13609	-0.45233
Q99571	P2RX4	0.177118	0.687179	0.147817	-0.14017	0.085947	-0.53632	0.317771	0.098815	-0.07642	-0.28738	-0.82029	1.465782	0.133217	1.490457	-0.45459	-0.33327	0.602659	-0.39077	-0.31306
Q99575	POP1	0.053533	-0.72759	-0.05438	0.485734	-0.2523	-0.00237	-0.0446	0.214761	0.107725	0.011295	-0.04499	0.197223	0.342085	-0.46996	0.293681	0.109136	0.146585	0.295723	-0.33048
Q99584	S100A13	-0.99673	-1.04297	-0.51431	1.352123	0.377922	0.709082	-0.14295	0.295456	0.94671	-0.06698	-0.90316	0.66463	-0.33507	-0.30661	1.245514	0.188223	-1.05286	-1.4553	-1.19325

Q99590	SCAF11	0.482192	-0.32671	-0.51951	0.315426	-0.5699	-0.06856	-0.36747	0.11464	0.399264	-0.07437	-0.00184	-0.97829	-0.43047	-0.49652	0.23219	-0.79779	-0.48474	-0.00024	0.292667
Q99594	TEAD3	0.435623	-0.26282	-0.01394	0.669916	-0.08273	-0.11933	-0.29846	0.284859	-0.38709	-0.77987	-0.54524	-0.25084	-0.06694	-1.16382	0.312199	-0.17079	0.261607	0.050378	0.406837
Q99598	TSNAX	0.324697	-0.46885	-0.34277	0.066691	-0.90216	-0.62839	-0.55545	0.272925	-0.45848	-0.34703	0.213607	-0.85291	-0.33614	-0.45064	0.260307	-0.09167	-0.47537	-0.64042	0.144764
Q99611	SEPHS2	-0.27123	0.041058	-0.04093	-0.38655	0.088452	-0.44204	0.190388	0.099602	0.20659	-0.23993	0.064203	-0.00755	-0.19859	-0.1794	0.184087	0.090658	0.022216	-0.18533	-0.42405
Q99614	TTC1	-0.25001	-1.07508	-0.14687	0.930002	1.536901	1.188546	-1.55771	0.038029	-0.07872	0.586395	0.341651	-0.69186	0.426633	-0.24613	0.371808	-0.18923	-0.14598	-0.06888	-0.00982
Q99615	DNAJC7	-0.51847	0.16371	1.493887	0.480855	0.374595	0.97637	-0.57644	-0.32349	-0.22988	0.450989	0.303823	-0.031	0.275346	0.188704	-0.73382	0.515793	0.671507	0.564641	0.079245
Q99623	PHB2	0.14666	0.002481	-0.4326	0.717807	-0.38707	-0.5464	0.112107	0.163424	-0.491	0.163333	-0.10023	0.254455	0.282217	-0.08851	0.577078	1.757069	-0.5152	-0.66334	-0.1617
Q99627	COPS8	-0.07744	0.27297	-0.34002	-0.03965	-0.02151	-0.14438	0.134121	-0.19731	0.664547	-0.07133	0.328876	-0.03142	-0.50713	0.063408	0.095094	-0.0397	0.033008	-0.16675	0.536328
Q99633	PRPF18	-0.0305	-0.18301	0.105618	0.008978	0.061959	0.291605	0.034012	0.432934	-0.24033	0.417914	0.11729	-0.44995	-0.58698	-0.32531	-0.18597	0.0489	-0.16783	0.160927	-0.01402
Q99653	CHP1	-0.00666	-1.17388	0.10675	0.051418	0.996506	0.378108	-0.15652	0.275976	0.825102	0.810129	0.000074	0.619134	0.501608	0.59745	0.007459	-0.05997	0.585291	0.705801	-0.16777
Q99661	KIF2C	0.969682	0.257464	0.691954	-0.17852	1.214193	1.189903	0.336516	1.00269	0.27573	0.094261	-1.22998	-0.0739	-0.12262	-0.75457	-1.64472	0.000304	0.987422	0.119871	-0.63142
Q99683	MAP3K5	-1.03531	0.135547	0.494729	-0.838	-0.3432	-0.40009	0.42107	-1.7645	0.11877	0.087535	-0.08978	-0.44648	-0.14692	0.300003	-0.98685	-0.36126	-0.21638	-0.02725	-0.48435
Q99685	MGLL	-0.36825	0.348962	-0.30314	-0.45315	-0.4002	-1.47982	1.008592	-0.25732	-0.11311	-0.277	-0.53073	-0.31988	-0.14178	0.633298	-0.86542	-1.36	-0.10491	-0.87872	-0.2709
Q99698	LYST	-0.25437	0.314774	0.106789	-0.28235	-0.57138	-0.49715	0.174192	-0.69425	0.456695	0.092631	-0.64926	0.296443	0.001011	1.255904	-0.26397	0.581855	-0.04591	-0.44386	-0.51621
Q99700	ATXN2	-0.50273	-0.31319	0.097024	-0.20508	-0.39715	-0.2779	0.012788	0.000387	-0.58699	-0.11981	0.019478	-0.58894	0.471624	-0.24884	0.445798	0.031657	0.133379	0.625931	0.727861
Q99704	DOK1	0.008211	0.330383	-0.00695	-0.08635	0.16708	-0.47573	0.089872	-0.11903	0.378177	1.187392	-0.05918	0.450366	0.041217	0.769462	0.328869	-0.51293	0.195033	-0.68402	-0.17499
Q99707	MTR	-0.71496	0.191069	-0.51887	-0.55481	-0.05545	-0.0658	0.517355	-0.16423	0.364564	-0.18701	-0.62593	-0.68922	0.014481	-0.23442	0.057036	-0.47453	0.104335	0.432698	0.296628
Q99708	RBBP8	-0.00608	-0.37645	-0.0086	0.944325	2.176436	2.288394	-0.21584	-0.28598	-0.39531	-0.96889	1.430872	-0.86114	-0.1452	-1.18285	-0.1661	-0.22854	-0.75074	-1.72944	-0.90981
Q99714	HSD17B1(	0.071509	-1.02027	-0.27424	0.067641	0.427758	-0.45592	-0.6766	0.25917	0.199721	1.09674	0.125047	0.389822	-0.47744	0.42119	0.272684	0.567519	0.112595	-0.15708	-0.50976
Q99715	COL12A1	-0.68707	-0.84348	-1.40211	-0.48281	-1.47757	-1.52129	-1.19309	-1.59875	0.521722	-0.55335	0.942415	-1.35866	-0.84121	-1.01241	-0.77457	0.773408	-1.17631	-1.75398	-0.5331
Q99717	SMAD5	-0.58058	0.121795	-0.12538	-0.08961	-0.12207	0.203551	0.035973	-0.39601	0.233605	-0.34678	0.776025	-0.01365	-0.02012	0.092716	-0.31533	-0.52047	-0.00354	0.286311	0.27554
Q99719	SEPT5	0.419693	-0.42018	0.178603	-0.76367	-0.56044	-0.55451	0.815126	-0.59701	1.350451	0.600399	-0.12601	-0.77012	-0.38966	1.051566	-0.12788	-0.01594	-0.34347	1.027783	0.391408
Q99720	SIGMAR1	0.460826	0.272759	0.180552	0.158284	-0.2578	0.230107	0.3905	-0.41075	-0.21563	0.212336	-0.10053	-0.07289	-0.67824	-0.23095	-0.23085	-0.57212	-0.39838	-0.93667	-0.29219
Q99729	HNRNPAE	1.029832	-0.26094	-0.05985	0.272082	1.189684	1.041696	-1.11838	0.161201	-0.63622	-0.51642	0.517273	-0.32937	0.66256	-0.61742	-0.26255	0.230822	-0.26569	-0.11208	-0.49474
Q99733	NAP1L4	-0.1681	-0.86641	0.279114	-0.36759	1.15887	1.996054	-0.80992	0.943325	-0.13039	0.383211	-0.00604	-0.37415	0.356416	-0.49229	0.454969	-0.22129	0.08496	0.574253	0.291966
Q99747	NAPG	-0.62606	0.23106	-0.17732	0.036931	0.138727	0.55285	0.428291	0.206174	0.323499	0.280725	-0.23019	0.038111	-0.22424	0.120712	0.464481	0.490222	0.939906	0.009492	-0.15475
Q99755	PIP5K1A	-0.04355	0.47725	0.225343	0.163064	-0.26421	-0.35132	0.53532	-0.28185	0.204748	-0.47879	-0.45455	0.204646	0.171583	-0.02452	0.881717	-0.07819	0.020358	0.116179	0.440196
Q99757	TXN2	0.255426	-0.39406	-0.68411	0.243645	1.746518	-0.46973	-1.22103	0.082212	0.809169	1.88197	-0.13443	0.133621	0.493582	0.345569	0.183255	0.474172	0.352589	0.657277	-0.76537
Q99759	MAP3K3	-0.39408	0.396052	0.275528	-0.09917	0.124431	0.189608	0.044352	-0.79552	0.142175	0.129741	-0.99617	0.480954	0.322116	0.348152	-0.35973	-0.04449	-0.0146	-0.20095	-0.44281
Q99766	ATP5S	0.434971	-0.08687	-0.07618	0.389597	-0.04251	-0.01738	-0.26079	-0.18823	-0.37072	-0.20917	0.847221	-0.21143	-0.38504	0.31236	2.309626	0.319646	-0.188	0.114908	-0.60573
Q99797	MIPEP	-0.2666	-0.18863	-0.74916	0.576804	0.386356	0.547216	-0.45639	0.401327	-0.42504	0.691719	1.039416	-0.34001	-0.34132	-0.10704	0.864215	0.048202	-0.602	-0.51366	0.054678
Q99798	ACO2	-0.3747	-1.15853	0.07699	-0.25418	0.593132	-0.10769	-0.38585	-0.15978	-0.32506	-0.1536	0.679087	-0.56661	0.275603	-0.35085	1.828881	1.261307	-0.79801	0.732194	-0.65023
Q99805	TM9SF2	-0.28908	1.092727	0.512642	-0.29066	0.167357	0.13285	0.042295	0.171901	-0.13451	0.058544	-0.1118	-0.04665	0.545356	-0.15067	0.777835	-0.25415	0.202695	0.341464	0.301198
Q99807	COQ7	0.220643	-0.10282	0.001044	0.005859	-1.00412	-1.00404	-0.45929	-0.6098	-0.33463	-0.51278	0.466099	-0.31388	-0.37833	-0.49183	1.389669	0.185442	-0.5717	0.039231	-0.60284
Q99808	SLC29A1	0.834737	0.850653	0.031986	-0.88745	0.410526	0.735275	1.012008	-0.26873	-0.44523	-0.60074	-1.24681	-0.6632	-0.98807	-0.46121	0.3684	-0.65366	-0.80695	-0.17908	-1.33956
Q99816	TSG101	-0.32826	0.35966	0.438557	-0.28733	-0.31594	-0.217	0.053559	0.221122	-0.25137	-0.32186	0.127463	-0.18593	-0.16549	0.111416	-0.16887	0.024499	0.046561	0.000406	0.146909
Q99828	CIB1	0.364696	-1.0418	-0.33174	1.034663	0.013281	0.372306	0.348067	0.397968	-0.09324	-0.00787	1.183495	-0.48489	-0.45155	0.073275	-0.79477	-0.19523	-0.10642	-0.64818	-1.03946
Q99829	CPNE1	0.174813	-1.08158	0.194236	0.016713	0.769299	0.314136	-0.4878	1.853138	0.515211	1.383299	-0.7155	-0.2066	-0.67086	0.772987	-0.17013	-0.45087	0.378419	0.021357	1.506316
Q99832	CCT7	-0.23271	-0.00149	-0.03134	0.058542	0.053071	0.430414	-0.79918	-0.56281	-0.13571	-0.05772	0.433426	0.119945	0.045462	0.014317	0.010043	0.060802	-0.13789	-0.32982	-0.1277
Q99836	MYD88	0.032365	0.516506	-0.11673	-1.05691	-0.0459	-0.1183	-0.23669	-0.00523	-0.28646	0.074308	0.035346	0.432338	0.292666	0.643091	-0.31188	0.021937	0.230312	-0.05072	0.084536
Q99848	EBNA1BP	0.265055	-0.29761	-0.9674	0.921971	-0.30661	0.208207	-0.49871	-0.15258	-0.57921	-0.36006	0.270021	-0.78185	-0.58593	-0.87195	0.199778	0.383819	-0.11131	-0.27359	-0.35902
Q99856	ARID3A	-1.5612	1.144177	0.738905	-0.53107	-0.02375	-0.37099	0.366393	-0.79355	0.085979	0.411034	-1.37336	0.781395	0.404353	1.068397	-0.57961	-0.15373	2.086538	2.092258	-0.723
Q99873	PRMT1	0.578708	-0.91812	0.171588	0.31045	0.263966	0.111731	-1.08472	0.548529	-0.0542	0.201677	1.260981	-0.34727	0.093154	-0.26496	0.011545	0.147603	0.064228	0.232312	0.801028
Q99933	BAG1	0.467808	0.078121	-1.1668	0.306087	-0.32811	-0.02614	-0.01061	-0.16504	-0.30782	-0.27402	-1.00477	-0.19137	0.064428	-0.1031	0.317294	0.563661	-0.07924	-0.30012	-0.75273
Q99943	AGPAT1	-0.3176	0.391739	-0.33888	-0.20803	-0.23664	-0.25854	-0.13013	-0.65067	0.188765	0.038577	0.128358	0.062763	-0.02801	-0.00581	0.673452	0.307923	0.044552	0.00766	0.135013
Q99959	PKP2	0.845558	-1.18104	-2.50031	1.048075	-0.40881	-0.16692	-0.51233	0.283231	-0.71516	-1.26588	1.196189	-0.64315	-0.06601	-1.33598	-0.73893	-0.92079	-0.86718	-1.20203	-0.87595
Q99961	SH3GL1	-0.37984	0.418007	0.079627	-0.91927	0.101108	-0.21184	-0.40953	-0.67003	-0.14717	-0.01062	-0.43402	-0.2529	0.238319	-0.23527	-0.75428	-0.36555	-0.22652	0.195964	0.644956
Q99973	TEP1	-0.10877	0.259324	-0.29929	-0.19463	-0.13453	0.101876	-0.06893	-0.2264	0.172671	0.011195	-0.25521	0.553658	-0.19539	-0.006	-0.15344	0.25682	-0.28886	-0.73245	-0.3469
Q99983	OMD	-1.93658	-0.20115	-0.60135	-1.53645	-1.00539	-1.90846	-0.01026	-1.41247	0.520997	-0.61431	-1.56528	-0.97364	-0.62399	-1.11232	-1.53396	-0.51143	-0.94137	-1.33658	-1.28923
Q99985	SEMA3C	-0.5744	0.746478	-0.2918	-1.19115	-0.30574	-0.49594	1.077792	-0.28348	0.140543	0.558482	-0.65214	0.52003	0.03013	-0.3424	-0.76056	0.364035	-0.48588	1.405511	0.883392
Q99986	VRK1	-0.28222	-0.3471	0.327628	0.465503	1.068238	1.226545	0.119663	0.785272	0.0931	0.178072	-0.15096	0.148941	-0.38277	0.195944	-0.18236	0.186199	0.689822	0.450235	-0.38811
Q99996	AKAP9	0.043038	0.201014	-0.09214	-0.43711	0.093892	0.152193	-0.00551	-0.0224	0.181667	0.036492	0.289608	-0.53057	-0.20969	-0.20303	0.862919	0.538217	-0.54609	-0.32014	0.138428
Q9BPU6	DPYSL5	0.54458	0.037552	0.203107	0.113086	0.240051	0.40795	0.27768	-0.23373	-0.18118	-0.54733	0.194705	-0.54143	-0.37448	-0.04751	0.245505	-0.44447	-0.41038	0.246581	-0.73225
Q9BPW8	NIPSNAP'	0.243222	-0.04745	-0.23272	0.651034	0.025911	-0.19131	-0.56344	-0.04669	0.082732	0.239274	0.359872	-0.23304	-0.33328	0.135727	0.411851	0.322755	0.003232	0.23927	-0.32787
Q9BPX3	NCAPG	1.183964	-0.23411	-0.03396	0.082214	1.108552	1.175714	0.515861	1.17918	0.056701	-0.07273	-1.10097	-0.17679	-0.35089	-0.01593	-0.74361	0.300596	0.305864	-0.8947	0.321951
Q9BPX5	ARPC5L	0.467106	0.107809	0.214789	-0.67678	0.363404	-0.35233	-0.10542	-0.14342	-0.29154	-0.2401	0.371414	-0.29381	0.005855	-0.27821	-0.708	-0.18928	0.039181	0.444717	-0.01099
Q9BPX6	MICU1	-0.02563	0.347177	0.375227	-0.30614	0.478514	0.302657	0.446329	-0.05808	0.567273	-0.51208	-0.21657	0.052273	0.524628	0.295148	-0.13679	0.270513	0.529391	0.347466	0.098609
Q9BPX7	C7orf25	-0.02354	0.180165	-0.64904	0.195479	0.153296	-0.3128	-0.27255	-0.29487	0.53372	0.537209	0.151212	-0.1709	0.169434	0.390444	-0.15493	0.214591	0.445965	0.219542	0.380808
Q9BPY3	FAM118B	0.069372	0.378042	0.464977	0.677727	0.504049	0.070926	0.372884	0.591368	0.316428	0.285226	-0.65007	-0.0305	0.326005	0.363237	-0.47347	0.378141	0.273585	0.295256	0.589268
Q9BPZ3	PAIP2	0.090764	-0.05006	0.117739	0.077593	0.954257	0.820671	-0.43266	0.326376	-0.4052	0.176217	0.016677	-0.11889	-0.00055	0.471956	0.14083	0.488917	-0.06127	0.829696	-0.24882
Q9BPZ7	MAPKAP1	0.337889	0.401925	-0.07149	-0.40822	-0.23861	-0.18705	-0.01945	-0.10952	0.10134	-0.08173	-0.32568	0.219632	-0.04905	0.275446	0.546908	0.069398	0.294215	-0.7473	0.488631
Q9BQ04	RBM4B	-0.19875	-0.08877	-0.20545	-0.16386	-0.17555	-0.49328	-1.14276	0.051312	1.016744	-0.21913	0.275367	-0.31981	0.139815	0.243824	-0.12146	-0.05769	0.495741	0.297518	-0.0681
Q9BQ13	KCTD14	1.177103	-0.20308	-0.44831	-0.67973	-1.08343	-0.52962	-0.87852	-0.70729	0.364631	-1.31264	0.879891	0.412623	0.241189	-0.2083	0.223462	-0.70941	-0.06408	-0.3123	0.046339
Q9BQ24	ZFYVE21	-0.19478	0.600263	-0.00273	-0.48909	0.384944	0.363929	0.068492	-0.26369	0.179406	0.430267	-0.01358	0.697589	0.111404	0.221692	-0.17943	0.173217	-0.04024	-0.62204	-0.58215
Q9BQ39	DDX50	0.950614	0.039484	-0.35643	0.261628	0.907576	1.160291	0.880239	0.432411	-0.58093	0.003554	0.390755	0.013275	0.091613	-0.46005	0.021029	-0.12878	-0.17934	0.276818	-0.36568
Q9BQ52	ELAC2	0.901645	-0.19799	0.356478	-0.31046	-0.06499	0.365212	-0.33091	0.382957	0.392556	-0.53084	0.555426	-0.3302	-0.37832	-0.27531	0.502147	-0.26254	-0.26777	-0.02304	-0.0397
Q9BQ61	TRIR	-0.43142	0.345462	0.001145	-0.92339	-0.92755	-0.90105	0.081236	0.846476	0.274783	-0.55527	-0.38666	-0.66479	0.356987	-1.22616	-0.52958	0.324067	0.122803	-0.37805	0.546159
Q9BQ67	GRWD1	0.598539	-0.76805	-0.04822	0.255205	-0.16328	0.143719	0.436445	0.063073	0.835883	-0.08888	0.016683	0.060888	0.197211	-0.18916	0.295958	-0.3725	0.087996	0.005794	0.412542
Q9BQ69	MACROD'	-0.02458	-0.88695	-0.25188	0.628524	-0.0258	-0.00483	-0.06743	-0.49815	-0.14585	-0.38404	-0.12482	0.032375	-0.58174	-0.16913	0.841366	0.806503	-0.23311	-0.32675	-0.82605
Q9BQ70	TCF25	-0.11386	0.598114	0.311518	-0.62831	0.290445	0.70949	0.058663	-0.60248	0.635671	-0.03872	-0.26999	0.040448	-0.26934	0.20556	-0.40593	0.767243	-0.35108	0.344935	0.971736
Q9BQ75	CMSS1	1.07918	0.083027	-0.08599	0.197196	0.351351	-0.6356	0.290427	0.063621	0.497715	-0.43356	-0.39099	0.148312	0.157497	-0.60381	0.581863	-1.08104	-0.31001	-0.93121	-0.11903
Q9BQ95	ECSIT	-0.20399	-0.34201	0.425202	-0.13361	-0.41912	-0.24644	0.117941	0.218188	1.836007	-0.58085	0.222909	-0.27377	-0.20433	-0.13008	0.026236	0.245948	-0.3794	-0.2238	0.433647
Q9BQA1	WDR77	0.34656	-0.16479	-0.61018	0.778095	-0.05701	-0.13556	-0.74056	0.885587	-0.17765	-0.16782	0.654007	-0.28081	-0.29599	-0.05535	0.385978	-0.02572	0.010683	-0.1622	-0.22302
Q9BQA9	C17orf62	-0.38299	0.759975	1.452701	-0.03141	0.201594	-0.13787	1.69241	-0.4848	-0.21372	0.029729	-0.34102	1.065717	-0.66296	1.245696	-0.38235	-0.63411	-0.28359	-0.22838	-0.17447
Q9BQB6	VKORC1	-0.05615	0.159665	0.04796	0.01278	0.892405	0.406972	0.537092	-0.34727	0.315496	-1.20738	-0.77157	-0.09724	0.178041	0.116366	-0.43951	-0.43679	0.170177	-0.28977	0.733263
Q9BQC3	DPH2	0.707858	-0.19978	0.121304	0.409207	0.458583	-0.48292	-0.26892	0.191221	-0.24845	0.080871	0.219413	0.080278	-0.37175	-0.5545	-0.6434	-0.13257	0.007598	0.040307	-0.26055
Q9BQE3	TUBA1C	0.421075	0.378792	0.173937	-0.25508	0.186883	0.381181	-0.05833	0.393023	-0.07067	-0.09165	-0.42326	0.05908	0.062801	0.001647	-0.33441	0.072126	0.057382	-0.17143	0.683474
Q9BQE4	SELENOS	-0.34791	-0.19923	0.198934	-0.24075	0.123243	0.301741	-0.45738	-0.44396	0.348084	-0.25181	-0.03688	-0.36152	-0.16559	-0.27415	0.180245	-0.22659	-0.41223	0.297464	-0.19617
Q9BQE5	APOL2	-1.71123	1.062232	0.772372	0.523964	-0.00438	0.206679	1.342051	-0.92115	0.235412	-1.08421	-1.53457	-0.23294	0.270866	0.729559	-0.91391	0.605306	1.744259	1.588166	0.234381
Q9BQE9	BCL7B	0.151803	-0.01649	0.522271	-0.14498	0.494446	0.919093	0.06532	1.368327	0.513148	-0.22656	0.221197	-0.37848	0.019474	0.714402	0.725013	0.217462	-0.07753	-0.30304	-0.27228
Q9BQG0	MYBBP1A	0.9196	-0.1158	-0.36096	0.615629	-0.25965	-0.17858	-0.41217	0.406993	-0.12337	0.220892	1.020828	0.045157	0.090542	-0.5918	-0.33617	-0.42579	-0.25105	-0.33887	-0.00365
Q9BQG2	NUDT12	-0.66233	-0.46835	-0.20735	0.08792	0.118269	-0.0229	-0.22396	-0.08179	-0.30985	-0.92928	1.062447	-0.29591	-0.26712	-0.34926	0.574048	0.798242	0.502296	0.235065	-0.79671
Q9BQI0	AIF1L	1.978949	0.310383	-0.08418	-1.47788	0.767584	-0.24134	-0.58677	0.395334	-0.36402	-0.28213	-0.06323	0.158355	0.280046	0.052423	-0.79084	-0.77459	0.107933	-0.46226	-0.70216
Q9BQK8	LPIN3	-0.35408	0.365959	0.16088	-0.04174	-0.31869	0.451911	0.326261	0.855989	0.155921	-0.38305	0.026515	-0.05323	0.218437	-0.08658	0.088409	0.026771	0.529985	0.553661	0.701185
Q9BQL6	FERMT1	-1.81111	-0.83639	-2.24761	-0.75889	-0.72186	-0.92843	-2.182	-0.93784	-0.7196	-0.90637	1.490275	-0.96624	-0.61566	-1.0009	-0.34752	-0.73763	-0.5649	-0.77267	-0.26888
Q9BQP7	MGME1	0.832999	-0.7769	-0.34778	0.346938	0.124837	-0.32963	-0.42235	0.332343	0.46414	1.138021	-0.07796	0.005966	0.014284	-0.161	-0.27019	0.81427	0.112486	-0.6123	-0.34427
Q9BQQ3	GORASP1	0.097085	0.030152	0.045945	-0.20292	0.001421	-0.05144	0.20744	-0.36977	-0.47732	-1.42779	-0.05437	0.67632	-0.01619	0.197528	-0.23256	0.043145	0.132297	1.043197	0.562764
Q9BQS7	HEPH	-1.03704	0.320995	-0.04777	-1.06656	-1.34861	-1.44988	-0.13905	-1.02533	0.330436	-0.31022	-0.33515	-0.98985	0.018464	-1.15013	-0.87546	0.233626	-0.893	0.881897	0.840132
Q9BQS8	FYCO1	-0.06736	0.04439	-0.1475	-0.32835	-0.24075	-0.15847	-0.13539	-0.78475	-0.2216	-0.71589	-0.04733	-0.18144	0.118392	-0.37562	-0.14185	-0.51251	-0.60686	-0.1308	-0.28288
Q9BQT9	CLSTN3	0.427497	-0.57806	-0.02581	0.194702	0.219056	-0.4656	-0.7437	0.457225	0.090619	1.33109	-1.24948	0.111128	0.287105	0.075263	1.18116	-0.03633	-0.47362	-1.42231	0.701684
Q9BR39	JPH2	-1.38214	0.157855	0.026472	-1.2336	-1.72097	-1.52785	0.226626	-0.52402	0.463723	-1.39996	-0.48564	-1.04586	-0.40515	-0.75672	-0.67967	-1.0344	-1.53359	-0.41477	-0.47069
Q9BR61	ACBD6	-0.25453	0.362324	0.29949	-0.09227	0.084804	0.228555	0.212884	-0.0424	-0.17813	-0.2296	-0.11897	0.202598	-0.04136	0.342291	-0.08688	-0.1241	0.270255	-0.03049	0.523911
Q9BR76	CORO1B	0.037058	0.232775	0.366043	0.078549	0.357434	0.627982	-0.61064	0.70401	-0.35734	0.334631	-0.05406	-0.13981	0.42981	-0.31144	0.135009	0.019566	-0.71843	-0.45929	-0.55234
Q9BR77	CCDC77	-0.11617	0.466975	-0.4879	-0.41801	-0.17012	-0.32886	0.131974	-0.38741	-0.52673	-0.30627	1.074582	-0.5283	0.138332	-0.362	-0.57352	-0.25871	-0.1403	0.384686	-0.31208
Q9BRA2	TXNDC17	-0.77935	0.782981	0.32141	-1.44101	-0.35109	-0.49027	0.232726	0.211298	0.589709	0.290895	-0.01357	-0.30735	0.916282	0.122595	-0.03747	-0.44625	-0.21719	-0.126	-0.49829
Q9BRD0	BUD13	0.550378	-0.0848	0.057503	0.235358	-0.45374	0.193743	-0.23536	0.436584	0.019601	0.189098	-0.03935	-0.6481	-0.04209	-0.33385	-0.16135	0.214271	-0.31417	0.356592	0.212957
Q9BRF8	CPPED1	0.046854	-0.81709	-0.09949	-0.08434	2.061484	-0.44036	-0.89005	0.01467	0.129932	1.036759	-0.51596	0.149808	1.167383	1.116861	-0.10895	0.937829	0.360939	0.167926	0.092956
Q9BRG1	VPS25	-0.69041	0.007542	0.321561	-0.3261	-0.01481	-0.28848	-0.10353	-0.02995	0.079256	0.093581	0.474855	0.013356	-0.21526	0.3031	-0.34433	0.028765	0.289521	0.024396	0.370105
Q9BRJ2	MRPL45	-0.56369	-0.18679	0.545907	1.100374	0.085951	0.19338	-0.21688	-0.31535	0.230366	0.492456	0.488927	0.174616	-0.16077	0.012028	0.714306	-0.19948	0.354319	-0.26525	-0.36147
Q9BRJ6	C7orf50	0.018963	-0.10045	-0.09432	-0.68733	-0.20147	-0.46368	-0.19732	-0.90411	0.536786	-0.52838	0.10297	-0.73322	-1.29204	-1.1379	-0.29185	-0.03939	0.083008	0.405215	0.025425
Q9BRJ7	NUDT16L'	0.395172	-0.03409	0.499027	-1.20553	-0.28144	-0.11746	-0.82295	0.20329	-0.1931	-1.14963	0.425864	-0.46044	-0.02513	-0.14256	-0.34013	-0.00532	0.105591	-0.03732	-0.29709
Q9BRK3	MXRA8	-0.32744	0.499998	0.451504	-1.33484	-0.83761	-1.33505	0.894008	-0.97393	0.366735	-0.23458	-0.90322	-0.9348	-0.35179	-0.3342	-0.16289	-0.31474	-0.95623	1.058462	0.837371
Q9BRK4	LZTS2	0.203822	-0.01654	-0.2998	-0.31483	-0.04929	-0.1865	-0.0188	0.508739	-0.06947	-0.58531	0.326998	-0.35347	0.310831	-0.54918	0.437441	-0.58063	-0.16938	-0.21974	-0.10416
Q9BRK5	SDF4	-0.70468	-0.44838	0.544003	0.149188	0.740384	0.873681	-0.11058	0.745435	0.447459	1.318042	0.040315	-0.58461	0.356707	-0.12608	0.935643	0.694361	0.527435	1.375358	0.319282
Q9BRP4	PAAF1	0.43355	-0.58322	0.138281	0.459782	0.243987	0.677628	-0.28957	-0.09378	-0.43751	-0.21689	0.152263	-0.16345	0.595779	0.143457	-0.12508	-1.00899	0.099666	-0.11332	-0.76096
Q9BRP8	PYM1	-0.06846	0.254651	-0.35136	0.031726	-1.06438	0.198595	-1.10247	-0.61252	0.18393	-0.4575	-0.31855	-0.68919	0.551705	-0.40644	0.740935	0.064187	-0.25968	-0.33628	-0.00039
Q9BRQ6	CHCHD6	-1.18468	0.198341	-0.53139	0.373515	-0.57084	0.178445	-0.21297	0.37821	-0.33381	-1.2377	1.345238	-0.77395	-0.26943	-1.37829	0.330377	0.496963	-1.03476	-0.30929	-0.81357
Q9BRQ8	AIFM2	-0.80974	-0.03382	0.541675	-0.26362	0.449849	0.325622	0.762898	0.672675	0.875098	-0.14934	-0.21873	0.347059	-0.12548	-0.5419	-0.35131	-0.67626	-0.64747	-0.07211	-0.6433
Q9BRR6	ADPGK	-0.09328	0.085768	0.344973	0.129598	-0.05251	0.105134	1.438086	0.16345	-0.14028	0.204517	-0.34576	0.650634	-0.69416	0.483674	-1.13288	-0.50702	0.406232	0.231069	-0.63209
Q9BRR8	GPATCH1	-0.21966	-1.02766	0.118494	0.156111	0.231859	1.399952	-0.47764	0.9745	0.316906	-0.76833	0.027772	-0.26415	0.075002	-0.59962	1.252693	0.314471	-0.08261	0.176651	0.716379
Q9BRR9	ARHGAP9	-1.97614	1.030207	1.150073	-1.0018	-0.00269	-0.44319	0.183834	-1.50186	-0.58436	1.396055	-1.02146	1.156264	-0.89417	1.818915	-0.26683	-0.38216	0.214499	0.489778	-1.26445
Q9BRS2	RIOK1	0.630613	-0.17036	-0.1577	0.559796	-0.36747	0.082285	-0.20904	-0.33687	-0.34069	-0.8009	0.333553	-0.73517	-0.13221	-0.71871	0.92325	0.275475	-0.97062	-0.88631	0.299947
Q9BRT2	UQCC2	0.030464	-0.713	-0.44235	0.158624	0.631726	0.804366	-0.27226	1.398226	0.378312	0.207982	0.474477	-0.20511	0.055739	-0.31977	0.635927	-0.47752	-0.63062	-0.27655	-0.34536
Q9BRT3	MIEN1	0.078176	-0.57812	-1.38181	-0.80332	-0.03736	-0.94783	-1.38895	-0.57464	-0.45299	0.284668	0.428949	0.258687	-0.87211	0.812952	-0.75785	-0.32917	-0.06561	-0.68928	-1.1871
Q9BRV8	SIKE1	0.185256	0.220195	0.194705	-0.11843	-0.49084	0.266578	0.060854	-0.09169	-0.38581	0.293599	0.340979	0.149533	-0.01048	0.015997	-0.51714	0.052062	0.517251	0.821918	-0.86302
Q9BRX2	PELO	0.082051	0.168742	0.405345	-0.00397	-0.19576	-0.14304	0.019544	-0.44773	0.34253	0.488431	-0.35243	0.088475	0.49206	0.254325	0.594385	0.227571	0.62362	0.011026	0.2736
Q9BRX8	FAM213A	0.361399	-0.9632	-0.33499	0.351147	-1.05143	-0.53006	0.276971	0.287212	-0.52775	-0.47199	-0.52933	-0.00748	-0.77093	-0.54133	2.023302	1.001186	1.433523	0.516827	-0.9495
Q9BRX9	WDR83	-0.00543	-0.33779	0.120829	0.117067	0.796296	1.149953	-0.4079	0.860683	0.072428	-0.08263	-0.60776	-0.51054	0.16998	-0.22921	1.247808	-0.0014	-0.30323	-0.47524	0.268319
Q9BRZ2	TRIM56	0.07423	0.464992	-0.3007	-0.49655	0.210537	0.589429	0.314579	0.022725	0.133011	-0.01018	0.102626	0.027468	0.240045	0.077184	0.082121	0.347568	-0.17513	-0.20541	-0.22676
Q9BS26	ERP44	0.088771	0.267127	0.7856	-0.17346	1.071675	0.605261	-0.32411	0.262017	0.230488	0.38788	-0.04166	0.817656	0.576875	0.428806	0.305918	0.295878	0.217426	0.24063	0.168736
Q9BS40	LXN	-1.91328	0.910109	0.613826	-2.14448	0.76782	-0.49496	1.099368	-1.18227	0.60012	0.285274	-1.86091	-1.49686	1.102943	-1.29056	-0.97563	0.065689	-1.05725	1.002279	1.463298
Q9BSB4	ATG101	0.186883	0.178302	0.20245	-0.52834	-0.17203	-0.04029	0.218895	-0.26078	0.19499	0.032353	0.02173	0.158669	-0.09978	0.011173	0.0103	0.105425	0.063689	0.49436	-0.10382
Q9BSC4	NOL10	0.453727	-0.44495	-0.70422	0.663317	-0.47659	-0.25162	-0.55013	0.404475	0.648889	0.164448	0.435772	-0.27917	-1.15496	-0.32305	0.436324	0.274131	0.115937	-0.2205	-0.26988

Q9BSD7	NTPCR	-0.91447	-0.5484	0.056312	-1.41373	0.247738	-0.13972	0.201715	-0.21599	0.670173	-0.65763	0.125762	-0.06745	-0.26609	-0.0531	0.500076	-0.69586	-0.13871	-0.89931	-1.05251
Q9BSF4	TIMM29	0.12911	0.022469	-0.55419	-0.02371	-0.04513	-0.15084	0.300432	0.249044	0.319019	0.189013	0.117901	-0.18936	-0.35185	-0.69434	0.42887	0.560662	-0.68334	-0.15626	-0.27423
Q9BSH4	TACO1	-0.14707	-0.25176	-0.20015	2.230542	-0.08333	-0.11144	-0.95246	-0.29176	-0.04934	0.236879	0.22016	-0.28702	-0.12035	-0.02396	0.344044	1.176461	0.434059	0.49963	-0.42442
Q9BSH5	HDHD3	0.07204	-0.2007	0.161492	0.216025	-0.33227	-0.37867	-0.06754	1.250317	0.492365	-0.10072	0.558753	-0.02268	0.09412	-0.26559	0.534691	0.710597	0.238408	0.419243	-0.32183
Q9BSJ2	TUBGCP2	-0.30301	0.116852	0.224974	-0.41035	0.297977	0.331426	0.578338	-0.31129	-0.21791	0.01826	0.441058	0.130924	0.00754	0.02053	-0.20373	0.423677	0.186845	0.018886	0.036363
Q9BSJ8	ESYT1	0.454456	0.095477	-0.40037	0.14795	0.604065	0.629045	0.174303	0.227779	-0.29188	-0.24639	-0.22894	0.127272	0.0312	-0.01043	0.445463	-0.59841	0.088667	-0.04429	0.350459
Q9BSL1	UBAC1	-0.1689	-0.08015	0.486471	-0.59333	0.118666	-0.32503	0.111389	-0.20328	0.05453	0.3417	0.262887	-0.23467	-0.29894	-0.09849	-0.04285	-0.06275	-0.05198	0.348011	-0.08951
Q9BSQ5	CCM2	-0.06771	0.616579	0.508822	-0.68726	0.076471	0.300634	0.505037	-0.47905	0.111723	0.013551	-0.2291	-0.08221	0.357541	0.517618	-0.19222	0.251952	-0.22164	0.144117	0.195049
Q9BSR8	YIPF4	-0.49294	0.659087	-0.18736	0.544556	-0.0036	0.321003	0.974294	0.014702	0.468354	0.461014	-0.04066	-0.02852	0.448018	-0.43999	0.355802	0.70881	-0.04568	0.144834	0.224506
Q9BST9	RTKN	0.653798	-0.69715	-0.43758	-0.95362	0.031744	0.438234	-0.60587	0.769551	-0.25325	-0.35817	0.393538	0.051108	0.1863	-0.49342	-0.43475	-1.02578	0.369234	-0.79903	-0.10844
Q9BSV6	TSEN34	0.669681	-0.00539	-0.26238	0.081679	-0.33676	0.165648	0.061967	0.229316	-0.0905	-0.30566	0.790125	-0.06074	0.040381	-0.77572	0.016218	0.479128	-0.73527	-0.31563	-0.41534
Q9BT09	CNPY3	-0.31279	-0.3699	0.317997	-0.69389	0.981468	0.06853	-0.17178	0.600678	-0.13749	-0.27374	-0.43683	1.584849	-0.02635	2.406169	-0.96927	-0.28673	1.063667	-0.71066	-1.21149
Q9BT17	MTG1	-0.72141	-0.33607	0.168952	1.045524	0.714589	0.86373	-0.50244	-0.19225	0.261726	0.758776	0.715862	-0.20592	0.073611	-0.25143	0.524276	0.515065	0.036003	0.331371	-0.58264
Q9BT22	ALG1	0.08075	0.459901	0.089983	-0.99657	0.115453	0.183095	0.61502	-0.75156	0.484707	-0.45113	0.629222	0.261129	0.167192	0.306074	0.106067	-0.06929	0.305174	0.298023	-0.18179
Q9BT23	LIMD2	-1.68363	0.895757	0.269061	-0.82435	-0.44543	-0.76582	0.347184	-0.86596	0.241085	0.343126	-3.23114	-0.38518	0.111685	0.738979	-2.31973	-0.41607	0.037616	0.521547	0.447027
Q9BT25	HAUS8	0.313725	0.068254	-0.13615	-0.80229	0.071924	0.501323	0.267535	0.443271	0.438404	-0.52899	0.067488	0.006476	-0.19969	-0.16133	0.065459	-0.26608	0.08061	-0.10061	-0.27142
Q9BT40	INPP5K	0.353806	0.214809	0.400845	-0.13746	0.277407	0.129724	0.698514	-0.19931	-0.01681	-0.22745	0.224615	0.672688	0.132457	0.250738	0.132119	-0.67956	0.00494	0.191696	0.072246
Q9BT78	COPS4	-0.17734	-0.06268	0.04312	-0.06463	-0.38873	-0.11811	0.084675	-0.10146	-0.32991	-0.07274	0.435542	-0.77254	-0.30624	-0.43691	0.191155	0.152248	-0.47999	-0.22639	0.234062
Q9BTA9	WAC	0.441684	-0.45478	-0.11819	0.05156	-1.15138	-0.01728	-0.72143	0.228286	-0.0241	0.325517	0.164104	-1.10254	-0.09138	-0.66042	0.462244	0.297074	-0.92157	-0.61183	-0.40137
Q9BTC0	DIDO1	0.645424	-0.31868	-0.23277	-0.21853	-0.20424	0.043667	0.246783	0.341545	0.05974	-0.28021	0.339868	-0.3239	0.24493	-0.37075	0.094662	-0.26738	-0.33649	0.227879	0.004588
Q9BTC8	MTA3	0.230076	-0.25498	-0.27938	0.064586	0.236572	0.528837	0.295969	0.594955	0.042682	0.429661	-0.27176	-0.40967	-0.06377	-0.14613	0.41902	0.855678	0.34803	0.246916	0.01206
Q9BTD8	RBM42	0.135419	0.190151	-0.26199	0.092232	0.027483	-0.05229	0.523417	1.198024	-0.41899	-0.31014	0.414388	-0.20992	0.104998	-0.40772	0.263877	0.238694	0.209269	0.530436	0.971754
Q9BTE1	DCTN5	0.397925	-0.0718	0.706233	-0.20411	0.078632	-0.20375	-0.60814	-0.12122	0.5989	-0.55896	-0.57086	-0.01575	-0.23035	-0.01836	0.19038	-0.37972	-0.2932	-0.26814	-0.27887
Q9BTE3	MCMBP	0.511523	-0.05122	0.326483	0.140324	0.482102	0.779946	-0.30445	0.339702	-0.17239	0.809501	-0.14662	-0.19917	0.204225	0.302956	-0.1301	0.764499	0.676773	-0.06705	-0.11368
Q9BTE6	AARSD1	-0.70865	-0.04328	-0.38316	0.876922	-0.28529	0.111408	-0.53378	-0.69051	0.095439	-0.40734	0.169996	-0.18877	0.359779	-0.37743	-0.34612	0.746433	0.021856	0.220074	0.212128
Q9BTE7	DCUN1D5	1.148187	-0.10133	-0.38412	0.39155	-0.52346	-0.6616	-0.18789	0.661422	-0.22199	-0.43367	0.541386	0.018135	0.35148	0.057078	-0.19025	-0.24055	-0.77043	-1.02166	0.065099
Q9BTL3	FAM103A1	0.698162	-0.0822	0.242564	1.238669	0.02425	0.368353	0.567743	0.555916	-0.93321	0.087918	0.387669	-0.48535	-0.54478	-0.56556	0.03279	-1.92626	-0.21036	-0.59356	-0.1337
Q9BTT0	ANP32E	0.948237	-0.8057	-0.40334	-0.21212	0.208817	-0.28834	-0.66384	0.57942	0.210736	-0.16573	-0.57268	0.495485	0.031066	0.851589	0.097338	0.023452	0.852928	-0.48664	-0.12134
Q9BTT6	LRRC1	0.896308	-0.6246	-0.14857	0.146029	-0.58645	-0.50517	-0.22805	0.111902	0.3419	-0.18004	0.876487	-0.41722	-0.4228	-0.26573	0.958701	-0.13847	0.244872	0.336025	-0.28956
Q9BTU6	PI4K2A	-0.703	1.378901	0.615781	-0.39738	0.418663	0.066093	0.201648	-0.13324	-0.43886	0.10324	-0.66922	1.333614	0.162217	0.768357	-0.73786	-0.48502	0.28441	-0.09367	-0.192
Q9BTV4	TMEM43	0.185323	0.609013	0.283919	-0.79678	-0.6526	-0.56708	0.667748	-0.35085	-0.34149	-0.60132	0.373533	-0.22979	0.062291	-0.40365	0.037288	-0.57556	-0.56322	-0.35626	0.592208
Q9BTW9	TBCD	0.479066	-0.3805	0.090445	0.555703	-0.25754	0.020413	0.002237	-0.0772	0.061016	-0.00893	-0.4344	0.08551	0.100282	-0.12581	-0.59535	0.627577	0.008583	-0.65799	0.065121
Q9BTX1	NDC1	0.1764	0.616291	0.050014	0.4871	0.404253	0.421634	0.208074	0.433213	-0.18062	-0.27987	-0.08129	0.09836	-0.14653	-0.0017	0.757622	-0.47209	0.279188	-0.09644	-0.20689
Q9BTX3	TMEM208	-0.21877	0.360933	0.080674	0.522156	0.516816	0.087616	0.314892	-0.87156	0.119711	-0.45054	-0.37909	-0.56095	0.168254	-0.14177	0.127696	0.345274	0.580493	0.183506	0.385918
Q9BTY2	FUCA2	1.404807	-0.67165	0.428829	0.393951	0.725773	0.352631	-0.20295	1.470932	-0.17397	0.312906	0.197821	-0.45749	-0.9991	0.325265	0.140322	-0.53063	-0.16117	0.206971	0.140868
Q9BTY7	HGH1	1.072998	-0.36638	-0.06046	-0.00214	-0.25994	-0.09225	-0.25747	-0.17496	0.442816	0.940936	-0.10392	0.708606	0.349437	0.110833	-0.61714	-0.23997	0.38913	0.162216	-0.59914
Q9BTZ2	DHRS4	-0.24062	-0.87026	0.248021	1.446096	1.573333	0.680474	-0.17504	0.566124	0.939156	0.55497	-0.88559	1.685971	-0.37376	0.956891	0.24074	0.752302	0.323875	0.30893	-1.14622
Q9BU02	THTPA	-0.2109	0.22501	0.211636	-0.18875	0.396147	0.007848	-0.02023	0.164693	-0.00431	0.677145	-0.56905	0.551108	0.039283	0.695718	-0.06824	-0.00791	0.337094	-0.63193	-0.44649
Q9BU23	LMF2	-0.53078	1.064426	0.432739	-0.48039	0.551735	0.175391	1.083202	-0.82404	0.161043	-0.18608	-0.51673	0.378194	-0.10719	0.082921	-0.15943	-0.0177	-0.04557	0.723177	0.474773
Q9BU61	NDUFAF3	-0.62789	0.167719	0.1985	-0.20655	0.529473	-0.12361	-0.03778	-0.62264	-0.03943	-0.14482	-0.03762	-0.33023	-0.10502	-0.02575	0.901958	0.146767	-0.19534	-0.55736	-0.64039
Q9BU76	MMTAG2	0.790729	-0.52157	-0.4355	-0.26018	-1.27095	-0.61516	-0.45659	-0.16296	0.067113	-0.19125	-0.02557	-1.07977	0.003306	-1.02779	0.664224	0.280665	-0.23548	-0.15621	-0.45125
Q9BU89	DOHH	-0.32897	1.034623	0.152181	-0.5256	0.593553	-0.4752	0.798927	0.511924	-0.22264	0.298478	0.188355	0.352317	0.847754	0.316121	-0.29777	-0.23505	0.499828	1.011951	-0.30674
Q9BUB7	TMEM70	0.083529	0.233807	0.014511	-0.1357	0.263617	-0.1637	0.05643	-0.63789	-0.19256	-0.41549	-0.01878	0.157172	-0.09252	-0.19017	-0.05329	0.388046	0.19407	0.457801	-0.28449
Q9BUE6	ISCA1	-0.376	-0.0254	0.06019	-0.17385	2.211079	0.874741	-0.27552	0.038726	0.092507	0.360603	0.274165	0.212477	0.068492	0.548233	0.314594	0.506426	0.341305	-0.44931	-0.77344
Q9BUF5	TUBB6	-0.20174	0.789637	0.390754	-0.99507	0.031499	0.00111	0.754511	0.543919	0.637603	0.406537	-0.714	0.040158	0.707586	-0.11061	-0.82526	-0.28911	0.037393	0.005056	1.064329
Q9BUH6	PAXX	-0.09758	-0.64572	-1.43603	-1.51382	-0.06464	-0.40799	-0.99351	0.138195	-0.2823	-0.38208	0.777047	-0.77429	-0.32105	-0.32724	-0.20159	-0.32922	-0.68722	-0.17189	-1.09666
Q9BUI4	POLR3C	0.21913	0.271642	-0.0374	-0.31871	0.16146	0.218627	1.203343	0.229027	-0.20689	-0.24001	-0.02346	-0.11578	0.48282	0.122215	0.584149	0.345536	-0.21538	0.22002	-0.35813
Q9BUJ2	HNRNPUL1	0.961342	-1.04286	0.110451	0.169265	-0.09996	-0.05316	-0.99233	1.549929	-0.23886	0.001685	0.500774	-0.16469	0.254262	-0.02984	0.914836	-0.65293	-0.66838	-0.31205	-0.51227
Q9BUK6	MSTO1	0.080208	0.481438	0.168385	-0.04542	-0.16335	0.040647	0.43818	-0.49894	0.660553	-0.05303	-0.22162	-0.0675	-0.09463	-0.04929	0.638593	0.719605	0.75123	0.075133	0.035299
Q9BUL8	PDCD10	0.110231	-0.20363	-0.08047	-0.42953	0.992015	0.507471	-0.13235	0.236088	-0.44948	0.452681	-0.24309	-0.78962	-0.57928	-0.27131	-0.08908	-0.01216	-0.1068	-0.34701	-0.1997
Q9BUN8	DERL1	-1.37272	0.564443	0.315354	-0.97442	0.229585	0.437839	0.072216	0.033041	0.03229	-0.28877	-0.68684	0.231877	1.284888	-0.41594	-0.20674	0.029426	0.369281	0.134991	-0.29327
Q9BUP0	EFHD1	-0.34641	0.320162	0.141716	-0.63116	-0.21866	-1.50548	0.346701	0.3138	0.268889	-0.9286	-2.41002	0.676823	-0.13647	-0.4126	-0.42774	0.145707	0.593277	0.767037	-0.7842
Q9BUP3	HTATIP2	0.111666	0.374644	1.059751	-0.97528	1.140668	0.770648	-0.03015	-0.40832	-0.20078	0.695509	-0.1443	1.213155	0.017946	1.3414	0.290352	0.238475	0.301979	-0.59999	-1.52835
Q9BUQ8	DDX23	1.055431	-0.19938	0.352052	0.449917	0.255519	0.429384	-0.40309	0.657651	0.056914	0.111204	0.175684	-0.51681	-0.28464	-0.60616	0.118952	-0.3421	-0.05971	0.196022	0.059349
Q9BUR4	WRAP53	0.086992	0.24117	0.134938	-0.32482	0.280951	-0.21969	0.274626	0.206152	1.188406	-0.43199	0.778576	0.294148	-0.21312	0.332016	-0.00681	-0.72039	0.099556	-0.19712	0.06104
Q9BUR5	APOO	-0.01458	-0.00256	-0.41069	0.030066	0.064479	0.096527	-0.06343	-0.25957	-1.22859	-1.08745	0.834115	-0.73837	-0.17669	-0.71652	1.259667	1.441676	-0.90078	-0.7291	-0.96815
Q9BUT1	BDH2	-0.78988	0.598617	-0.03958	-0.83339	-2.65924	-1.76297	0.621513	-0.03906	-0.10873	0.225156	0.812179	-0.0071	-0.20278	-0.06956	-0.25965	-0.01949	-0.48948	-0.61219	-0.13927
Q9BUW7	C9orf16	0.37948	-0.10924	-0.35281	0.058435	-0.55015	-0.29282	-0.31296	0.649679	-0.30444	-0.57805	0.926542	-0.68687	-0.04172	-0.33625	-0.36199	0.127801	-0.31821	0.218222	0.624774
Q9BUZ4	TRAF4	0.152962	-0.07561	0.41775	-0.49836	0.254322	-0.34644	-0.2864	0.064897	0.918392	-0.49071	-0.02471	-0.12078	-0.3492	0.058241	-0.12604	-0.80948	0.268424	-0.03313	-0.29816
Q9BV10	ALG12	-0.62208	0.452571	0.321943	-0.02885	0.352796	-0.41436	0.306022	-0.10683	-0.19377	0.215736	0.041012	0.494616	-0.05995	-0.06093	0.010416	0.383425	0.101973	-0.24525	0.448553
Q9BV20	MRI1	-0.21428	-0.2951	0.370011	-0.15542	-0.01044	-0.51556	1.309745	1.059439	0.330679	0.047789	-0.49141	-0.37678	0.067063	-0.81904	0.224224	0.059818	0.356488	-0.28709	-1.13677
Q9BV35	SLC25A23	-0.19243	-0.01817	-0.33966	-0.01467	-0.97368	-0.50426	0.00907	-0.93716	-0.36253	-0.07038	1.191984	-1.13478	-0.02508	-0.22986	0.46378	-0.61886	-0.00414	0.185417	-0.23055
Q9BV38	WDR18	0.846	-0.39927	-0.26736	-0.09097	0.388949	0.606151	-0.86555	1.183306	-0.07281	0.168751	0.989133	-0.09102	-0.11826	-0.55239	0.236997	0.47604	-0.54994	-0.13022	0.428177
Q9BV40	VAMP8	0.446208	1.003922	1.208195	-0.19638	-0.30924	-0.27575	0.742728	0.627788	0.173935	0.982833	-0.50607	0.854312	1.216932	1.031775	0.177551	0.57712	-0.20769	-0.47317	-0.93074
Q9BV44	THUMPD3	1.071924	-0.35522	-0.69021	-0.22336	0.579809	0.369368	-0.05606	-0.3376	-0.24534	-0.07605	0.71537	0.14935	-0.27853	-0.28667	-0.46973	0.01418	0.588654	0.223296	0.004671
Q9BV57	ADI1	-0.754	-0.02402	-1.49297	-0.3911	-0.15838	-0.83916	-0.59107	1.348328	0.739059	0.271646	-0.73659	-0.43008	0.03851	-0.19084	-0.93529	-0.36118	0.324555	-0.68609	0.168094
Q9BV73	CEP250	-0.63402	-0.05088	0.272056	-0.23815	0.129728	-0.19334	0.046569	0.031235	0.344338	-0.24674	-0.10148	-0.12678	-0.1754	-0.25507	-0.21588	-0.75623	-0.1414	0.004172	0.004733
Q9BV79	MECR	0.0256	-0.22759	-0.38058	-0.57423	0.783147	-0.02946	0.078022	0.648081	0.045185	0.665875	0.673679	0.133003	0.189133	0.316987	0.641151	0.673517	-0.31448	-0.00716	-0.31326
Q9BV86	NTMT1	0.154899	-0.39072	0.086247	-0.55561	0.359766	0.404005	-0.04935	0.485995	0.239032	0.635768	-0.14623	0.649771	0.152734	-0.01176	-1.52822	-0.01191	0.308657	-0.49045	-0.53406
Q9BVA0	KATNB1	-0.02684	0.015917	-0.02291	-0.48588	-0.57145	-0.2855	-0.07486	-0.26112	-0.24016	0.131419	0.184527	-0.12868	0.040879	-0.34048	-0.44048	0.170142	-0.08828	-0.07563	-0.09735
Q9BVC4	MLST8	0.16083	0.994055	-0.07748	-0.02814	-0.44989	-0.31193	-0.12397	-0.13894	0.024882	0.071964	0.104768	-0.224	-0.09553	0.273559	0.391772	0.21939	-0.08168	-0.47057	0.191364
Q9BVC6	TMEM109	0.054034	0.397267	0.273676	-0.20295	-0.57137	-0.71629	0.204159	-0.90118	-0.64525	-0.58178	-0.23257	0.156945	-0.54814	-0.30687	0.451635	-0.31591	-0.29055	-0.10015	-0.43523
Q9BVG4	PBDC1	0.477208	-0.50197	0.045303	0.599498	0.372187	0.045902	-1.49338	0.803967	-0.36058	0.526547	0.390217	-0.67446	-0.22202	-0.15763	-1.11127	0.29622	0.209887	0.11274	-0.34102
Q9BVG9	PTDSS2	-0.36616	-0.29107	-0.01221	-0.51924	0.706174	-0.0027	0.009209	1.115039	0.498812	0.305229	-0.53991	-0.03592	0.042925	0.284137	0.034003	-0.32325	0.27805	-0.5937	-0.02392
Q9BVI4	NOC4L	0.471685	0.036673	0.112828	0.078498	-0.37289	-0.17532	0.390769	-0.01852	-0.3279	0.155479	0.531404	0.152418	-0.10292	-0.19307	0.228404	0.114095	0.071797	-0.41504	0.29451
Q9BVJ6	UTP14A	-0.05293	-0.11377	-0.64031	0.489643	-0.31592	-0.05157	-0.29634	0.376523	-0.24835	-0.05446	0.574252	-0.62697	-0.22904	-0.81793	0.66048	0.031084	-0.34734	-0.23887	0.042947
Q9BVJ7	DUSP23	0.001369	-0.34076	0.652473	-0.07015	0.131833	0.11507	-0.4598	-0.59259	0.264588	0.394416	-0.11914	-0.11896	-0.08412	-0.04118	0.706425	0.408025	-0.58816	-0.71094	0.579828
Q9BVK2	ALG8	0.35199	0.017311	-0.2457	0.201627	0.066504	0.313654	-0.25009	-0.13227	0.001445	-1.32939	-0.37262	0.227289	-0.18749	0.347175	-0.30929	-0.31143	0.563035	-0.57683	0.042019
Q9BVK6	TMED9	-0.8624	0.91304	0.390709	0.228222	0.282875	0.141275	0.561305	-0.34302	-0.28252	-0.3612	-0.06453	0.591025	1.014328	0.220919	0.06459	0.188464	0.621972	0.745154	0.736333
Q9BVL2	NUP58	-0.10616	0.250003	-0.50213	0.030493	0.116852	0.230669	-0.42724	0.213673	-0.20955	-0.186	0.396139	0.069645	-0.00165	-0.0965	0.935054	-0.23407	-0.00527	-0.32889	0.155461
Q9BVM2	DPCD	0.158788	-0.02863	-0.24494	0.778959	-0.32914	0.154555	-0.15001	0.226657	-0.5177	-0.2574	0.745098	-0.51625	-0.31308	-0.80011	-0.51588	-0.43595	-0.07552	-1.40761	-0.43268
Q9BVM4	GGACT	0.003106	1.74103	-0.22469	0.326726	0.717085	0.53884	0.091179	0.696891	0.853569	-1.06928	0.51487	0.04239	-0.15339	-0.09359	-0.52448	-1.89026	-1.48957	0.156939	-0.78566
Q9BVP2	GNL3	0.448935	0.010326	-0.60887	0.494505	-0.46544	-0.47351	-0.85414	-0.65091	-0.34285	-0.51302	0.596499	0.179526	0.248255	-0.34786	-0.08997	-0.15463	0.000956	-0.13645	-0.28691
Q9BVQ7	SPATA5L1	0.21404	0.227916	0.665219	-0.00965	-0.27807	0.212125	0.413819	-0.60362	-0.2346	-0.00476	-0.04468	-0.02633	0.175063	-0.04869	-0.20295	0.17059	0.347804	0.022101	-0.04705
Q9BVS4	RIOK2	-0.7475	0.116472	0.07318	0.348037	-0.03026	-0.15852	-0.44104	-0.15689	-0.07154	1.401631	0.33473	-0.43635	-0.03716	-0.44207	-0.64353	0.307437	-0.0478	0.166493	0.303658
Q9BVS5	TRMT61B	-0.48882	0.174397	0.962809	0.754824	0.303059	-0.34695	-0.35315	-0.08595	-0.11374	0.073571	-0.41015	-0.0027	-0.23955	0.380665	1.973293	-0.2963	-1.03542	-0.32466	
Q9BVT8	TMUB1	0.038415	0.503683	0.12338	0.189774	0.19534	0.480949	0.164504	-0.16019	0.846481	-0.13689	0.002529	-0.7785	-0.40979	-0.73209	0.996899	-0.13421	-0.45132	0.162819	-0.46345
Q9BVV7	TIMM21	-0.06987	-0.0591	-0.38933	0.102606	0.337634	0.231179	-0.57964	-1.07472	-0.29801	-0.19765	0.886915	-0.5855	-0.90285	-0.52929	0.755198	0.761033	-1.05513	-0.54731	-0.56844
Q9BW04	SARG	-1.69074	-1.03748	-0.86781	0.768584	-2.79093	-2.18775	-0.72852	-2.06872	-0.05394	-0.63961	-1.57312	-1.65821	-0.92247	-1.2301	1.593587	-0.49814	-0.59465	-3.46072	-3.21387
Q9BW19	KIFC1	0.840862	-0.04417	0.308048	-0.27642	1.399912	1.665523	0.088628	0.797133	-0.64266	-0.54695	-0.81427	-0.36191	-0.49	0.511988	-0.12543	-0.54692	0.22665	-0.47984	-0.14947
Q9BW27	NUP85	0.225771	0.087466	-0.03702	0.412203	0.145992	0.285021	-0.04642	0.327871	-0.17097	-0.32351	0.251151	0.098465	-0.34238	-0.08579	0.812652	-0.0148	0.218516	-0.00192	0.277224
Q9BW30	TPPP3	-2.73751	-0.18429	-2.85412	-2.48237	-1.98944	-3.22582	-1.81033	-0.15413	0.001896	-2.41662	2.440795	-0.34245	-1.01288	-0.76633	-1.82987	-2.35571	-0.32558	-2.27777	-2.52343
Q9BW62	KATNAL1	-0.19259	0.209275	0.321229	-0.40463	0.239936	-0.22378	0.09381	-0.32624	0.164557	0.009127	-0.15944	-0.24311	0.08742	0.220308	-0.55007	0.022225	-0.1443	0.114247	0.70536
Q9BW71	HIRIP3	0.714986	-0.47493	-0.3437	0.369847	1.768246	1.405658	-0.28637	-0.08481	0.352476	-1.49214	-0.22241	-1.25955	-0.03556	-0.49456	-0.49014	0.872393	-0.09648	0.326167	-0.79289
Q9BW83	IFT27	0.013616	1.808206	0.297852	0.126512	1.277152	-0.59189	-0.33189	-0.61239	0.433417	0.023594	0.609196	0.877115	0.305751	1.007894	-0.60526	0.095303	1.089867	0.330344	-0.02944
Q9BW85	CCDC94	-0.61393	0.032839	0.243078	-0.08461	0.023037	-0.30522	0.635488	0.594629	-0.82659	-0.56229	1.648827	-0.32874	-0.20947	-0.07803	-0.21562	-0.11845	-0.12808	1.242024	0.094901
Q9BW91	NUDT9	1.21696	-0.46991	0.086411	0.192017	0.701953	0.474186	-0.42856	0.397601	0.309287	1.594067	0.512277	-0.30972	0.320983	-0.26706	0.446272	0.307061	0.009675	0.335065	0.114815
Q9BW92	TARS2	0.113398	-0.42292	-0.04983	0.929948	-0.45962	-0.59484	-0.59636	0.359843	-0.36772	-0.05813	0.265625	-0.36865	-0.25783	-0.37326	1.272935	0.419265	-0.5222	-0.63209	0.1368
Q9BWD1	ACAT2	-0.94972	-1.51335	-0.36419	0.16961	2.982431	0.82225	0.196622	-1.09782	0.705419	0.200272	-0.55371	-0.46915	1.008056	-0.5271	-0.45253	0.012038	0.009623	0.459988	-0.74467
Q9BWF3	RBM4	0.62136	-0.39995	0.026154	0.483358	-0.15084	0.345083	0.108303	1.105762	0.01635	-0.06734	0.235897	-0.57065	-0.33612	-0.70698	-0.17964	0.078562	-0.09018	0.317899	0.021076
Q9BWH2	FUNDC2	0.097129	-0.10058	1.091403	0.280883	0.747493	0.699311	0.315576	0.424785	0.099603	0.132035	-0.07332	-0.86462	-0.46959	-0.48858	0.047126	0.970303	-0.28166	-0.14317	-0.06462
Q9BWH6	RPAP1	0.434443	1.448265	-0.20545	0.102957	-0.60948	-0.48769	-1.14276	-0.50154	0.648755	-0.59659	0.639275	-0.23121	0.359304	-0.16799	-0.63735	0.378353	0.35228	-0.02641	0.08471
Q9BWJ5	SF3B5	0.54047	-0.0708	-0.02783	0.567514	-0.3553	0.373359	-0.23409	0.63347	-0.1825	-0.11199	0.189248	-0.5963	-0.15993	-0.24612	0.59772	0.381867	0.141888	-0.14364	0.230126
Q9BWM7	SFXN3	-0.31027	0.942135	0.420174	-0.74259	-0.60282	-0.81533	0.534304	0.092694	0.396603	-0.29518	0.021091	0.15475	0.092086	0.155041	-0.65193	-0.08356	-0.25169	0.223767	1.167263
Q9BWN1	PRR14	0.039378	-0.47394	-0.11853	-0.47508	-0.01063	-0.51186	-0.10408	0.121408	-0.18028	-0.18724	0.163538	-0.2054	0.087013	-0.34798	0.740662	-0.08916	-0.26684	-0.05398	0.300924
Q9BWP8	COLEC11	-2.07796	-0.40371	-0.95567	-2.05165	-2.57762	-2.53979	0.015536	-1.8418	-0.84473	-1.75084	3.22038	-1.63476	-1.04286	-0.30029	-1.72823	-1.62593	-0.25172	-0.78867	-3.04103
Q9BWS9	CHID1	-0.85184	0.511856	-0.12392	-0.50777	0.695106	0.746911	0.698822	0.329321	-0.03813	-0.19282	0.669873	-0.35185	-0.7015	-0.35435	0.48802	0.248542	-0.27774	-0.08397	-0.55586
Q9BWU0	SLC4A1AP	0.654365	-0.00225	0.310188	0.837523	-0.23389	-0.0549	-0.57555	0.394349	0.39222	-0.09671	-0.57048	-0.33176	0.190394	0.005748	-0.43313	0.794614	0.559528	0.827231	-0.14936
Q9BX40	LSM14B	0.660645	0.054869	0.049641	0.017711	-0.42647	-0.50672	-0.34046	-0.03282	-0.27669	-0.02829	-0.23393	-0.21975	-0.29182	0.116522	0.154982	0.070992	-0.03599	-0.18243	0.146266
Q9BX59	TAPBPL	-0.91543	1.063027	-0.21781	0.411224	-0.26652	-0.29439	-0.01913	-1.46324	0.44873	1.08921	0.086046	-0.06336	0.299767	0.005229	-1.33101	0.131792	-0.68946	-0.56902	-1.09883
Q9BX66	SORBS1	-1.64425	-1.30456	-1.16973	-1.54208	-1.64207	-2.59493	-0.40526	-0.93252	-0.46115	-1.8657	-1.05139	-0.7058	-0.20218	-1.18841	-0.48937	-1.2298	-1.34663	-0.64865	-1.71138
Q9BX67	JAM3	-0.29984	0.052231	-0.02062	-1.27657	-1.45565	-1.55802	0.829036	-1.78899	0.345597	-0.48368	-0.50716	-1.91905	-0.62332	-0.39769	-0.28572	-0.34676	-1.55396	0.394949	0.593495
Q9BX68	HINT2	-0.00488	-1.03067	-0.86924	0.527995	0.597117	-0.09462	-0.77641	0.071347	0.001148	-0.92048	0.154496	0.21415	0.199612	-0.31408	-0.06252	1.536176	-0.10638	-0.19208	-1.61433
Q9BX70	BTBD2	-0.1078	0.437895	0.136708	-0.47152	-0.44393	-0.01823	-0.30732	0.006073	-0.28252	-0.19686	0.124276	-0.01724	0.167695	-0.69969	-0.34533	-0.2968	0.089652	-0.26635	-0.19316
Q9BX79	STRA6	2.394874	-0.51397	-0.60135	-1.17554	-0.86597	-1.18025	0.071549	-0.28724	0.201322	-0.22095	0.274853	-0.91831	0.252369	-0.81894	-0.42539	-0.29689	-0.47453	-0.17316	0.820883
Q9BX97	PLVAP	-0.5982	0.467115	0.569169	-0.94578	1.216792	-0.5153	1.241105	-1.29084	0.23422	0.772099	-1.10696	-1.19801	-0.19281	-0.43435	-0.38155	-0.20554	-2.10723	0.465012	-0.2784
Q9BXB4	OSBPL11	0.03687	-0.00278	0.227387	0.501762	0.265585	0.42479	-0.2791	0.336503	0.684858	1.311312	-0.21247	0.241351	0.211214	0.555876	1.003311	-0.09628	0.91218	0.56814	-0.1621
Q9BXB5	OSBPL10	0.30017	-0.09074	0.218219	-0.36973	-0.12396	-0.00316	-0.21585	-0.43971	-0.25964	-0.71204	-0.11107	-0.48266	-0.0577	-0.77065	0.116936	0.249195	-0.66789	-0.7802	0.267362
Q9BXC9	BBS2	-1.20265	-0.21699	-0.35156	-0.45481	-0.55381	0.099537	-0.21476	-1.11993	0.235835	-0.58778	0.915657	-1.00586	-0.14458	-0.78008	-0.46007	-0.48707	-0.98411	-0.24306	0.235424
Q9BXD5	NPL	-3.20716	1.608927	0.651009	-1.77948	0.823323	-0.05965	0.09531	-1.9534	0.325177	0.013777	-1.52626	1.387528	0.908064	1.397275	-1.81656	-1.00813	-0.23837	-0.03267	-2.12132
Q9BXF6	RAB11FIP5	-1.04568	-0.16852	-0.97867	-1.05816	-1.26332	-0.54989	0.315106	-0.27559	0.251302	-0.04855	-0.60977	-0.47317	0.953043	-0.71145	0.781562	-0.05127	-0.11777	-0.28305	0.870248
Q9BXI6	TBC1D10A	-0.60363	0.171249	-0.02942	-0.02905	0.109622	-0.18842	-0.24394	-0.2918	-0.38076	0.131603	-0.14037	0.026651	-0.47397	0.295243	-0.054	0.296131	0.660738	0.732012	-0.12251
Q9BXJ0	C1QTNF5	-1.04575	0.443073	-0.34227	-0.60593	-0.62761	-0.44633	0.177521	-0.80441	0.040847	-0.65911	-1.57658	-0.12485	0.054165	0.144141	-0.37257	-0.77134	0.050533	-0.9622	-0.30925

Q9BXJ4	C1QTNF3	-2.58513	0.853506	-0.86832	-2.49581	-4.29222	-1.62707	0.126621	-1.4121	2.090774	-3.47551	-3.80389	-3.68379	-0.72836	-1.22747	-0.506	-1.7072	-0.85362	-2.39386	-0.31786
Q9BXJ9	NAA15	-0.12396	0.025694	0.285486	0.426115	0.369294	0.519185	-0.19527	0.203302	-0.29049	0.01966	0.340227	-0.36495	-0.18752	-0.29947	-0.76386	0.306535	0.157182	-0.40806	-0.12301
Q9BXK5	BCL2L13	0.237472	0.746016	0.688127	-0.11159	-0.44277	-0.54075	0.17051	-0.4531	0.037801	0.368173	0.348128	-0.4612	0.335651	-0.25126	-0.05351	0.886604	-0.39167	0.618694	0.055895
Q9BXL7	CARD11	-1.62943	0.4444	0.307523	-0.18151	0.141803	0.166975	0.139399	-0.35941	-0.26508	1.037821	-0.03087	-0.41017	-0.23459	0.226246	-0.22194	0.050685	0.134489	0.279293	-1.24278
Q9BXN1	ASPN	-2.8564	-1.98227	-0.77084	-2.38337	-1.03614	-2.32267	0.881719	-1.99346	0.587865	-2.09342	-2.61781	-1.51051	-2.0643	-1.17288	-0.1186	-2.17786	-1.7541	-0.75626	-2.2897
Q9BXP2	SLC12A9	0.034199	0.551074	0.543968	-0.14084	-0.09506	-0.36199	0.801224	0.043045	0.081662	0.877829	-0.55946	0.75241	-0.11639	0.831628	0.896693	0.31097	-0.09115	-0.38631	-0.11577
Q9BXP5	SRRT	0.853326	-0.25613	-0.10842	0.115579	0.119457	0.350894	-0.44417	0.935499	-0.26787	0.220873	0.3726	-0.33249	-0.46581	-0.47564	0.371128	-0.14771	-0.00817	0.30816	-0.20302
Q9BXR0	QTRT1	-0.02912	-0.31166	-0.73585	0.866953	0.051344	-0.24518	-0.62948	0.6895	0.285335	0.781103	0.684377	0.077825	-0.1598	-0.04125	-0.29974	1.018929	-0.18383	0.302309	0.087077
Q9BXS5	AP1M1	-0.59579	0.430494	0.749837	-0.10644	0.041728	-0.14783	0.421445	-0.0333	0.216064	0.391521	0.011234	0.082565	0.059984	0.380476	-0.28532	0.218225	0.048718	-0.00885	0.382122
Q9BXW6	OSBPL1A	-0.71256	0.540878	0.249262	-0.72843	-0.25953	-0.39407	0.228713	-0.00194	0.099608	0.456146	0.540946	0.110063	-0.06015	0.075488	0.295263	-0.43265	-0.69887	-0.185	-0.12182
Q9BXW7	HDHD5	0.904088	0.13042	0.03947	0.993613	-0.24961	-0.81578	0.328681	-0.08445	-0.05733	-0.04759	0.281927	-0.01226	0.130496	0.266382	-0.10222	0.432979	0.119447	0.58376	-0.62998
Q9BXW9	FANCD2	1.955185	-0.54133	-0.07251	-0.23595	2.055694	2.449715	0.035124	1.018259	-0.52685	-0.72855	-0.13355	-1.09428	-0.3456	0.669324	-0.87322	0.438498	-1.07563	-1.30542	-0.52489
Q9BXX0	EMILIN2	-2.4124	1.105927	2.2932	-2.25657	-0.99508	-1.0427	1.291676	-1.01115	0.325871	1.568724	-2.1701	-0.93507	-0.07304	-0.81209	0.192146	-0.81728	-0.78397	1.592601	-0.96372
Q9BXY0	MAK16	0.462892	-0.20631	0.398958	0.249798	0.226495	0.198588	-0.27478	0.07632	-0.14204	-0.35394	0.683468	-0.65742	-0.20743	0.180327	0.859393	0.096265	0.234811	1.152406	-0.20728
Q9BY11	PACSIN1	-1.16795	0.375959	-0.00919	1.425774	-0.39351	0.185919	0.012415	0.156967	-0.21441	0.286528	-1.23424	-0.75618	0.209774	0.295761	-0.05647	-0.10727	0.243445	0.980556	-1.41572
Q9BY12	SCAPER	0.098773	-0.30077	0.178894	0.241677	0.314347	0.916947	-0.20198	0.44016	-0.06074	-0.00676	0.112435	-1.04301	-0.144	0.265703	-0.20788	0.120386	-0.8607	-0.33783	0.320055
Q9BY32	ITPA	0.069965	-0.09974	-0.16501	-1.27499	0.83519	-1.18052	0.523714	1.451247	0.973028	0.426715	-0.23133	0.605321	-0.02036	0.730435	-0.8251	-1.28828	0.794856	-0.43221	0.120757
Q9BY42	RTFDC1	0.341993	-0.20138	0.067444	0.297094	0.165747	0.839871	-0.26385	0.967142	0.073376	0.2527	0.149199	-0.76884	-0.14471	-0.08792	0.540162	-0.08982	0.0613	0.03536	0.524162
Q9BY43	CHMP4A	0.566574	-0.29476	-0.19745	-0.71141	0.326838	0.153116	-0.09599	-0.44508	-0.29121	0.434189	-0.35375	0.287335	-0.26694	0.33102	-0.35849	0.406472	0.23386	0.506164	-0.47046
Q9BY44	EIF2A	-0.41626	0.333226	0.261395	-0.35103	1.023645	0.950172	-0.19198	0.208352	1.073111	0.554987	-0.42666	0.027867	-0.31211	0.281679	1.244073	-0.16842	0.499992	-0.29733	-0.18434
Q9BY49	PECR	-0.67849	-0.01533	0.465464	-0.29182	1.041434	1.070818	0.311634	-0.11671	0.040847	-0.65124	-0.10616	-0.42353	-0.03841	0.70038	0.304995	1.747898	0.99028	1.162069	-0.84484
Q9BY50	SEC11C	-2.02877	-0.75299	-0.02273	-1.52991	-0.24986	-1.22178	3.763402	-1.25158	0.439525	-2.29543	-0.378	-0.76092	-0.45015	-0.78924	-1.36002	-1.23048	-1.14339	1.691325	-1.60761
Q9BY67	CADM1	-0.06154	-0.95495	-1.72667	-0.67139	-1.74793	-0.95262	-0.39955	0.659399	-0.41736	-1.55702	1.848269	-0.86385	-0.54577	-0.28553	0.398927	-0.35611	-1.08753	-1.74632	-0.82863
Q9BY77	POLDIP3	0.155884	-0.01553	0.378163	-0.35025	-0.01779	-0.09759	-0.04429	-0.51839	0.51931	-0.11146	0.485694	-0.39872	-0.40693	-0.36166	-0.8822	-0.24232	0.316386	1.521473	-0.37648
Q9BY89	KIAA1671	0.654746	-0.52966	-0.44466	0.019462	-0.60335	-0.78394	-0.47266	-0.86887	-0.26819	-0.83197	0.303316	-0.72984	-0.54017	-0.95695	-0.33449	-0.17175	-0.24934	-0.44511	-1.02985
Q9BYB4	GNB1L	1.43671	-0.32666	-0.33503	0.479921	-1.10585	-1.4944	-0.39294	-0.60245	-0.65813	0.423884	0.252287	-0.20165	-0.28952	0.464115	0.191395	-0.76694	-0.62209	-0.87799	-1.25855
Q9BYC5	FUT8	0.205353	0.111331	0.170655	-0.20108	0.308822	0.526617	1.214735	-0.14657	-0.27916	-1.09761	-0.03491	-0.44241	-0.21189	-0.24311	-0.85481	0.270566	-0.36376	0.316092	0.547285
Q9BYC9	MRPL20	-0.05919	-0.03656	0.198136	1.185594	0.733809	0.45009	-0.126	-0.34804	-0.39417	0.083365	0.844965	0.305135	-0.3429	0.040931	0.796163	0.10085	-0.13796	-0.24181	-0.6181
Q9BYD1	MRPL13	-0.39135	-0.47181	0.275012	0.33309	0.148803	0.40566	-0.26153	-0.43307	0.206394	0.447466	0.502544	0.085725	0.371666	0.159402	0.783458	-0.06723	-0.02168	-0.02227	-0.43542
Q9BYD2	MRPL9	-0.17949	-0.21513	0.146664	1.169267	0.25403	0.473314	-0.19435	-0.43711	-0.37957	1.147279	0.041881	0.065567	0.086139	-0.02942	0.946189	-0.22755	0.091977	-0.15403	-0.65801
Q9BYD3	MRPL4	-0.41833	0.134259	0.049339	0.882961	-0.01488	0.345514	-0.23065	-0.21574	1.553524	0.138785	0.445316	0.080087	0.375373	-0.26852	0.170074	-0.23622	-0.33284	-0.01516	0.033607
Q9BYD6	MRPL1	-0.41123	-0.113	-0.062	0.568937	0.552732	0.330939	-0.1823	-0.37468	0.447663	0.981956	0.297709	-0.06227	0.058569	-0.38646	0.505076	-0.21244	-0.20408	-0.31181	-0.71697
Q9BYG3	NIFK	0.76768	-0.18284	0.04147	0.406827	-0.60036	-0.24285	-0.07092	0.149864	1.207371	0.112974	0.427127	-0.22076	-0.32461	-0.19654	-0.03881	0.193629	0.197481	0.204749	-0.33187
Q9BYG5	PARD6B	0.719171	0.4235	-0.77227	0.243354	0.005772	0.287646	-0.365	0.151494	-0.29745	0.118892	-0.60633	0.144471	0.005653	-0.15956	0.707899	-0.073	1.21465	-0.62899	0.091725
Q9BYI3	FAM126A	-0.27212	0.380239	0.050126	0.146383	0.244523	0.279567	0.500785	-0.61469	-0.47682	0.211699	0.018565	0.329527	0.125818	0.025251	-0.29418	-0.46789	-0.00924	0.066157	0.498343
Q9BYJ9	YTHDF1	0.254587	-0.40684	-0.34612	-0.8298	0.21267	-0.04793	-0.46126	0.92265	-0.31859	0.029303	0.102393	-0.27792	-0.05023	0.526426	0.561945	0.538462	0.265839	-0.02518	-1.18671
Q9BYK8	HELZ2	0.815061	0.641175	0.589473	-0.45907	0.178245	0.781115	0.086358	0.79127	0.428046	-0.10841	-0.69436	0.040287	0.47422	0.116609	-0.76454	-0.02886	0.546029	0.162767	-1.10084
Q9BYM8	RBCK1	-0.10502	0.271989	0.228037	-0.66926	-0.26933	-0.21158	0.15562	-0.08662	0.136376	0.509174	0.363368	-0.2301	-0.20719	0.380426	0.128699	0.141361	-0.23878	-0.07451	-0.19164
Q9BYN0	SRXN1	0.375938	-0.32199	0.533603	-0.47057	0.105607	-0.23208	-0.0336	0.263708	0.724021	-0.10494	-0.06077	0.263006	-0.61467	0.341134	-0.66652	0.148067	1.213256	0.140379	-0.02441
Q9BYN8	MRPS26	0.09947	-0.43623	-0.13387	0.682131	0.271973	0.672488	0.296646	0.271374	-0.27709	-0.00935	0.475917	-0.3391	-0.14807	-0.52434	1.235704	-0.22014	-0.487	-0.22954	-0.36393
Q9BYT3	STK33	-0.57866	-0.24324	-0.4185	-0.14046	-0.31306	0.938254	0.002935	-0.71658	0.040847	-0.94331	2.471092	-0.44931	-0.20103	-0.41566	0.30554	-1.54993	-0.40193	-0.22835	0.132139
Q9BYT8	NLN	-0.06985	0.298915	0.053941	0.280979	-0.65288	-0.46678	-0.22505	0.778952	-0.52718	-0.46515	0.661784	-0.39866	-0.20681	-0.4262	1.233557	0.172452	-0.28933	-0.49246	0.080585
Q9BYV8	CEP41	0.076272	-0.28671	-0.5908	-0.46422	-0.41293	0.459717	-0.41317	-0.11922	-0.24031	-0.04411	0.506214	-1.18573	-0.01848	-0.52033	0.063708	-0.09694	-0.97176	-1.24376	-1.20964
Q9BYW2	SETD2	0.633682	0.262551	-0.25795	-0.41249	0.203281	-0.04961	-0.42482	0.161921	-0.25431	-0.12626	0.425004	-0.36306	-0.17969	-0.49398	-0.49181	-0.80469	-0.26943	0.279747	0.036254
Q9BYX2	TBC1D2	-1.18572	0.255112	-0.00218	-0.70617	-0.37261	-0.78911	0.73842	-0.16047	0.299193	-0.29741	-0.40599	1.405994	0.140645	1.184054	-1.41622	-0.31055	0.284692	-0.81726	-0.93922
Q9BYX4	IFIH1	0.561837	0.501616	2.241678	-0.56325	-0.45951	-0.09926	-0.36954	0.102322	-0.31349	-0.11197	-0.3147	0.149432	0.022084	0.390463	-0.6978	-0.67249	0.430212	0.109951	-1.53216
Q9BZ23	PANK2	-0.67031	0.656712	0.330138	-0.2272	0.44137	-0.46513	0.127146	0.103712	0.040847	0.117862	-0.20588	0.121096	0.383104	0.389947	-0.17308	0.253709	0.182613	-0.30261	-0.07415
Q9BZ29	DOCK9	-0.19919	-0.68409	-0.26442	-0.98998	-0.19777	-0.32191	-0.27353	-0.18578	0.04477	-0.21791	0.59448	-0.96	-0.78366	-0.58575	-0.17087	-0.60994	-0.98123	-0.58474	-0.57652
Q9BZ67	FRMD8	-0.07346	0.441517	0.057167	-0.23018	0.013304	-0.05215	0.082784	-0.1624	-0.53445	0.220841	0.356741	0.325989	0.579775	0.069314	-0.14369	-0.12022	-0.20713	-0.0683	-0.15984
Q9BZ95	NSD3	0.557873	-0.7597	-0.3996	-0.41513	-0.46425	-0.02396	-0.2498	-0.17548	-0.01126	-0.31547	-0.12531	-0.66778	-0.15522	-0.7276	0.161214	-0.79097	0.251867	0.764621	-0.50292
Q9BZD4	NUF2	0.82458	-0.20117	0.151201	-0.34539	0.84214	1.588693	0.433551	1.477274	0.150049	-0.21779	-1.06932	0.14443	0.098357	-1.2489	-0.71061	0.037581	0.171436	-0.32403	-0.26653
Q9BZE1	MRPL37	-0.18412	-0.21438	0.292359	1.089953	0.42965	0.578879	0.184927	-0.31154	-0.25104	0.616814	0.450916	-0.13956	-0.07921	0.071915	0.721775	-0.01224	0.028676	-0.12948	-0.41578
Q9BZE4	GTPBP4	0.384363	-0.0603	-0.57602	0.573464	0.260801	0.353363	-0.36193	0.071751	-0.01575	-0.07957	0.341984	-0.10893	-0.03297	-0.24077	-0.15007	0.213633	0.37505	0.107511	-0.38346
Q9BZE9	ASPSCR1	-0.38461	0.079131	-0.15227	-0.42861	-0.26966	-0.61048	-0.29313	0.303924	0.43216	-0.05062	-0.19606	-0.31073	0.236135	-0.11894	0.08828	-0.41153	-0.1373	-0.16581	-0.39368
Q9BZF1	OSBPL8	-0.27369	0.394484	0.618359	-0.21548	0.191346	-0.15624	0.109977	-0.88705	-0.07014	0.079344	-0.43089	0.30078	-0.43107	0.404297	0.396917	-0.1211	-0.01149	0.30501	-0.09018
Q9BZF9	UACA	-0.24829	0.296923	0.167399	0.162645	0.367882	-0.37579	-0.20219	-0.33047	-0.04411	-0.35809	0.615936	-0.65425	-0.18848	-0.6485	0.111845	0.018584	0.469822	1.257334	-0.27831
Q9BZG1	RAB34	-0.40123	-0.0704	-0.46851	-0.39814	-0.87482	-0.60195	0.002415	0.400545	0.77896	-0.35345	0.18004	-0.3279	-0.90678	-0.05605	-0.90985	-0.65968	-0.35563	-0.43986	0.281724
Q9BZG8	DPH1	0.729339	-0.18837	0.055628	0.324564	0.496003	0.372186	-0.25182	0.546037	-0.31289	-0.05131	-0.11451	-0.12028	-0.14745	-0.13542	0.127588	0.122312	-0.24343	-0.21876	-0.07709
Q9BZH6	WDR11	-0.27042	0.303802	-0.08353	-0.09641	-0.1136	-0.00886	-0.18115	-0.29587	-0.24712	-0.09221	0.033864	0.44316	0.087099	0.499023	0.318398	0.394108	0.36609	-0.21602	-0.41499
Q9BZI7	UPF3B	0.867481	-0.25204	0.490193	0.037411	-0.12062	0.300585	0.222805	0.595773	0.228985	-0.07005	0.090561	-1.30444	-0.29099	-0.39412	-0.25099	0.815719	-0.4998	-0.21368	-0.29142
Q9BZJ0	CRNKL1	0.138809	-0.05978	0.093255	-0.08468	-0.30579	-9.1E-05	0.051134	1.240065	-0.41415	0.049461	0.457681	-0.43659	-0.32075	-0.12036	0.178023	-0.00573	-0.11716	-0.19759	0.345286
Q9BZK7	TBL1XR1	0.38359	-0.52092	-0.04878	0.768484	1.902703	3.067675	-0.29921	0.357917	-0.06498	-0.61569	0.090556	-0.1211	0.112433	-0.50456	0.886598	-0.14607	0.129041	-0.08901	-0.1575
Q9BZL4	PPP1R12(	-0.53526	0.394186	0.214426	-0.48129	-0.39162	-0.36994	-0.40937	-0.38044	0.298204	0.009209	-0.29282	-0.32449	-0.21384	-0.12849	0.103525	0.113389	-0.28843	0.357247	0.549831
Q9BZL6	PRKD2	0.074384	0.475731	0.263632	-0.14959	-0.79385	0.090353	0.257395	-0.16169	0.349044	-0.10171	-0.22195	-0.40142	0.282931	0.063406	0.478494	-0.45925	-0.20851	0.071138	0.154202
Q9BZQ6	EDEM3	-0.10326	0.651315	0.388387	0.217996	0.176086	0.098573	0.143028	-0.16781	0.038876	0.344396	-0.56737	-0.2242	0.102134	0.285676	-0.03123	0.077335	-0.1234	0.079135	0.363424
Q9BZQ8	FAM129A	-1.68297	0.740982	0.827457	-1.65887	-0.39435	-0.74868	0.145297	-1.76121	0.534333	0.922407	-2.20252	0.124829	-0.66812	1.169935	0.28071	-0.75488	-0.12264	-0.09681	-1.15674
Q9BZV1	UBXN6	-0.58921	0.203759	-0.36562	-1.18888	-0.16994	-0.53787	-0.21639	-0.61151	0.494602	0.490488	0.5013	-0.1467	-0.56643	-0.24422	-0.03113	-0.59962	-0.78159	-0.59116	-0.17886
Q9BZX2	UCK2	0.865513	0.053883	0.163325	0.199912	0.377422	0.228938	-0.11594	0.093573	-0.09504	-0.17487	-0.6639	-0.42514	-0.29695	0.278182	-0.10731	0.794498	0.302278	-0.80325	-0.3
Q9BZZ2	SIGLEC1	-1.58581	2.557786	3.068671	-1.36052	0.524763	-0.51084	1.465001	-1.79325	-0.41138	-1.28214	-1.22177	2.1089	0.104397	1.209039	-0.34991	-0.65225	-0.26277	0.22552	-3.13297
Q9BZZ5	API5	1.014662	-0.33811	0.052686	0.084355	0.238571	0.406157	-0.34042	0.480345	-0.43594	-0.04717	0.719577	-0.81772	-0.50552	-0.73432	-0.25257	-0.03392	-0.54647	0.075315	-0.3656
Q9C002	NMES1	-1.95458	1.72251	1.34218	-0.54262	0.276171	1.247488	-0.6052	-0.84421	-0.33203	1.618385	-2.30778	0.057858	-0.25441	1.112043	-0.99675	-0.70077	0.910449	-0.31746	-1.20939
Q9C005	DPY30	0.476813	-0.00673	0.470636	0.101708	0.376777	0.572656	-1.12748	0.975532	-1.06942	-0.4524	0.043883	0.04413	0.133209	-0.0385	0.338931	1.050694	0.636133	0.396152	-0.61748
Q9C035	TRIM5	-0.2595	0.477781	0.280028	-0.96793	0.784939	0.928993	0.37893	0.143674	0.427944	-0.59582	-0.03576	-0.11199	0.284431	0.295204	-0.69846	-0.18854	0.273366	0.748865	0.530053
Q9C037	TRIM4	-0.31111	0.894378	0.26976	-0.15679	0.153688	-0.03946	0.348104	-0.21238	-0.20858	0.380053	0.009139	0.646727	0.576944	0.241301	0.10402	-0.25859	-0.62374	-0.38364	-0.15403
Q9C040	TRIM2	-0.26382	-0.36119	-0.75609	-0.73611	-1.13725	-0.96533	-1.15567	1.024257	0.123994	-1.09556	0.213204	-1.10911	-1.67893	-0.36374	-0.51777	-0.64444	0.127543	0.421412	0.190969
Q9C075	KRT23	-1.05791	0.0151	-0.31956	-1.07738	-2.74671	-2.46917	-0.10135	0.476342	-1.23982	-0.39759	1.093971	0.147619	1.01782	-1.40016	-1.09183	-1.82388	-1.78719	-3.85876	-1.35785
Q9C0B0	UNK	0.627437	-0.18636	0.156459	-0.04363	-0.47023	-0.01961	-0.58659	0.115719	0.049301	-0.08829	0.073669	-0.54353	-0.06118	-0.28219	-0.77618	0.183276	-0.3685	-0.45121	0.049491
Q9C0B1	FTO	0.059603	-0.29644	-0.47518	0.742526	0.902632	0.214204	-0.55759	0.254767	-0.00335	0.416169	0.131842	-0.33354	0.140476	-0.31268	0.877659	0.656337	-0.17905	0.712566	0.721968
Q9C0B5	ZDHHC5	0.054657	0.401968	-0.082	-0.00214	-0.97228	-0.68713	-0.14607	-0.60426	0.299262	-0.16501	0.116605	-0.03255	0.415233	0.19867	0.88592	0.701662	0.069505	0.04189	0.018807
Q9C0C2	TNKS1BP	-0.38046	-0.06309	0.297423	0.017171	-0.87727	-0.66528	-0.25304	-0.13276	-0.03497	-0.41537	-0.36219	-1.01683	0.706151	-1.01297	0.873547	0.740694	-0.84823	-0.12462	0.724478
Q9C0C4	SEMA4C	0.242688	-0.12941	0.115174	-0.37805	-0.77693	-0.55605	0.013655	0.503531	-0.34403	-0.02073	0.519648	-0.89733	-0.57111	0.046138	-0.72453	0.121878	-0.47087	-0.26854	-0.92701
Q9C0C7	AMBRA1	0.129014	-0.16231	0.11544	-0.15184	-0.02089	0.109778	-0.56688	-0.04662	-0.23701	-0.01392	-0.13846	-0.56516	0.117192	0.047879	-0.08407	-0.113	0.97362	0.589044	-0.13549
Q9C0C9	UBE2O	0.109342	-0.28247	-0.05175	-0.19181	-0.45514	-0.16932	0.015962	0.342159	-0.23574	0.026599	-0.31555	-0.27039	0.024829	-0.41387	-0.47988	0.027628	-0.0796	0.118996	0.075384
Q9C0D3	ZYG11B	0.104248	-0.07302	0.051417	0.171832	0.120198	0.538508	-0.15459	0.180348	0.146791	0.062027	0.061526	-0.20274	0.100811	0.106323	0.537784	0.616135	-0.00845	-0.6948	0.547086
Q9C0D5	TANC1	0.538005	-0.45181	-0.0723	-0.38882	-0.60191	-0.44074	-0.28401	0.232021	0.14707	-0.12094	0.007099	-0.80793	0.040244	-0.40387	0.617578	0.400627	-0.38783	-0.09311	0.060483
Q9C0E2	XPO4	-0.47351	0.558344	-0.14545	0.375075	0.701395	1.008403	0.628321	0.054069	-0.2498	0.005885	-0.00795	-0.31523	0.238085	-0.09822	1.259008	-0.12839	0.02623	-0.17142	0.394123
Q9C0E8	LNPK	0.311903	0.092276	-0.18496	-0.12594	-0.18216	-0.5592	-0.38706	-0.5844	-0.04405	-0.30105	0.066547	0.24016	-0.0582	0.10002	0.282573	0.129559	0.165081	-0.01476	-0.02184
Q9C0F1	CEP44	-0.18216	0.086794	0.379906	-0.2061	-0.11855	0.465523	-0.05451	-1.06478	-0.04054	0.454159	0.225599	-0.32415	-0.0604	-0.11999	-0.81959	0.501845	0.569473	2.044679	-0.16443
Q9C0H2	TTYH3	0.786385	0.458746	0.722025	-0.94113	-0.88326	-0.50876	-0.06088	0.700439	-0.30376	-0.38382	-1.15474	0.056468	0.259065	0.542441	-0.94569	-1.13682	-0.04566	-0.29484	-0.41401
Q9C0I1	MTMR12	0.165591	0.262727	-0.33181	-0.53259	0.105496	0.122289	-0.9306	-0.52866	0.463632	-0.4385	0.249153	-0.21331	-0.0411	-0.23316	0.43264	-0.88845	0.606256	0.428301	-0.25691
Q9C0J8	WDR33	0.313286	0.074097	0.393894	-0.27817	0.196091	0.096323	-0.5938	-0.02166	0.227775	-0.2756	-0.0086	-0.09751	-0.28038	-0.0781	-0.0427	-0.4413	-0.01216	0.229666	-0.04617
Q9GIY3	HLA-DRB'	0.60164	-0.14808	-0.73792	0.235376	0.790424	0.505453	0.009889	0.577461	-0.64266	-0.6237	-0.85511	-0.59076	-0.14434	-0.19396	0.150604	0.026537	0.223347	-0.61708	-1.84782
Q9GZL7	WDR12	0.593229	-0.24597	-0.15438	0.618643	-0.46053	-0.13997	-0.31974	0.258507	-0.05725	-0.01514	0.293667	-0.7112	-0.31931	-0.63099	-0.43464	0.349226	0.517897	0.304746	-0.27413
Q9GZM5	YIPF3	-0.52913	0.384178	0.18269	-0.30904	0.154452	0.292165	0.80228	0.0614	0.622604	0.580842	0.174929	-0.02065	0.063725	-0.39052	0.448135	0.823486	-0.10902	0.400566	0.154512
Q9GZM7	TINAGL1	-0.53062	0.060489	0.994833	0.23797	-0.29188	-1.11219	0.397936	-1.11228	0.091775	0.30783	-0.66252	-0.68063	-0.44686	-0.57916	1.157766	-0.79894	0.807464	1.383427	-1.7971
Q9GZM8	NDEL1	0.111809	0.445646	-0.06035	-0.31607	-0.09816	-0.36288	-0.46813	-0.08772	0.209113	-0.16267	-0.25237	-0.3695	-0.11165	0.051918	-0.1027	-0.20663	-0.23024	-1.05041	-0.22948
Q9GZN1	ACTR6	0.251063	-0.04406	0.054354	-0.02738	0.000954	0.357906	0.115213	0.152341	0.199673	-0.08744	-0.22659	-0.33015	-0.07771	-0.11718	0.063037	-0.3103	-0.0462	-0.46712	-0.17688
Q9GZN7	ROGDI	-0.76348	1.407477	0.63729	-0.92719	0.404956	0.229404	0.226968	-0.87909	0.071364	0.555724	-0.34443	1.027828	0.477347	0.888919	0.097797	-0.13683	0.078796	-0.15602	-0.45916
Q9GZP4	PITHD1	0.748799	-0.21065	0.055385	-0.4661	0.523226	-0.33409	-0.23304	0.605391	0.002127	0.315311	-0.01204	-0.14634	0.110526	-0.25239	0.557579	-0.54254	-0.22142	-0.01792	0.239631
Q9GZQ3	COMMD5	-0.04247	0.427157	0.101798	-0.43943	-0.08431	-0.02905	0.419053	-0.1012	-0.16854	0.410212	-0.02663	0.241515	-0.24173	0.557168	-0.33757	0.177624	-0.10769	-0.5198	-0.02321
Q9GZR1	SENP6	0.521705	-0.54229	-0.02513	0.047279	-0.85111	-0.71129	-0.57116	-0.42922	-0.29717	0.269668	0.198604	-0.91413	0.238211	-1.3989	1.04133	0.462507	-0.43758	0.440652	-0.00175
Q9GZR2	REXO4	0.750155	-0.15472	-0.0405	0.959131	0.106939	0.408056	-0.25935	0.823801	-0.27442	-0.05497	0.015636	-0.39923	-0.08224	-0.97475	-0.45118	0.280915	-0.50616	0.520606	0.40742
Q9GZR7	DDX24	-0.09103	-0.19552	-0.10519	0.397539	0.094125	0.482712	0.241817	0.770855	-0.60148	-0.49881	0.097059	0.098831	0.079475	-0.25023	0.793158	0.808131	0.143112	0.078004	0.194137
Q9GZS1	POLR1E	0.925872	-0.10474	-0.53776	0.292929	-0.2668	0.456334	-0.55688	0.323543	-0.62604	-0.08804	0.417987	-0.63973	0.030132	-0.71039	-0.11495	-0.25172	-0.29571	-0.13979	-0.74764
Q9GZS3	WDR61	0.152598	-0.54393	-0.27949	0.076757	0.398393	-0.01344	-0.39226	0.056554	-0.0064	-0.12159	0.346934	-0.07667	0.003728	0.295432	0.442699	-0.13321	0.239522	-0.01999	0.374711
Q9GZT3	SLIRP	-0.46532	-0.03566	-0.71922	0.330904	-0.09552	-0.11195	-0.61787	0.436682	-0.49062	1.045294	-0.24769	0.178479	0.117234	-1.02701	0.700199	-0.06869	0.002467	-0.74173	-0.90737
Q9GZT6	CCDC90B	-0.32056	-0.14177	0.071159	0.550585	-0.02257	-0.1486	0.10676	-1.18809	-0.76606	-0.25669	0.553339	-0.3953	0.234085	-0.40123	1.372589	1.390604	-0.69254	-0.27271	-0.55515
Q9GZT8	NIF3L1	0.114151	-0.94938	-0.05329	-0.26541	0.064282	-0.22787	-0.80728	0.527668	-0.1457	-0.30869	-0.11832	-0.82652	0.032495	-0.63287	0.039056	0.183181	-0.39177	-0.32943	0.017871
Q9GZT9	EGLN1	0.125853	0.129309	0.321692	-0.07561	-0.06425	-0.36542	-0.09708	-0.08145	-0.05347	0.432713	0.044207	0.370395	0.238238	0.758334	0.132908	-0.29596	1.520319	0.16216	-0.19533
Q9GZU7	CTDSP1	0.233385	0.276482	-0.27714	0.048107	-0.9937	-0.16488	0.014127	0.115365	-0.45879	-0.88502	0.274844	-0.58577	-0.63416	0.367166	-0.0465	-0.26865	0.08111	0.04269	0.399749
Q9GZU8	FAM192A	0.60043	-1.27864	0.51967	0.316565	-0.45485	-0.33933	-0.9221	-0.12623	-0.13033	0.090393	0.624966	-1.01303	-0.03425	-0.60247	0.094491	0.499322	-0.11107	0.705178	0.569418
Q9GZV4	EIF5A2	-1.36154	0.21222	-0.38615	0.114234	0.929719	0.942582	-0.85849	-0.35396	-0.13612	0.824198	-0.14028	-0.36381	-0.33161	0.215735	-1.14367	-0.40962	0.188496	0.044405	-0.52235
Q9GZV5	WWTR1	0.743904	-0.20713	-0.3921	-0.96409	-1.34612	-0.07117	-0.19134	0.848634	-0.42603	-0.31463	-0.11904	-0.39332	0.009561	-0.43476	0.149687	-0.51288	-0.05591	0.854428	0.578248
Q9GZY4	COA1	0.470783	-0.15773	-0.48935	-0.31172	-0.35817	0.219295	-0.18898	0.249782	-0.03461	-0.15945	0.366936	-0.37221	-0.29033	-0.32161	-0.0988	0.60891	-0.37544	-0.07188	-1.23244
Q9GZY8	MFF	-0.10402	0.258852	0.120788	-0.46009	-0.32799	-0.20472	0.237091	-0.23113	1.33911	0.466971	0.612937	-0.07239	0.190035	0.314702	0.13893	0.563795	0.150718	0.770557	-0.03627
Q9GZZ1	NAA50	-0.77054	-0.684	-1.35075	-0.07946	0.713777	-0.00892	-1.28631	0.28276	0.480135	0.569651	0.053241	0.675504	-0.88359	0.648207	-0.92533	-0.47742	0.723347	-0.39822	-0.75144
Q9GZZ9	UBA5	-0.54488	-0.44458	0.384637	0.50979	0.200015	0.497397	-0.60812	0.064268	0.681695	0.307964	-0.38045	0.289724	1.049282	0.635596	0.87485	0.075671	0.475687	0.205125	0.641052
Q9H000	MKRN2	1.034256	-0.29766	0.040986	-1.00883	-0.28197	0.628356	0.060498	-0.45105	0.237528	0.023799	0.360555	-0.25806	0.200878	-0.12333	0.086397	0.816255	-0.53	-0.14055	-0.09335
Q9H008	LHPP	-0.82156	-0.71421	0.829179	0.588717	0.181499	0.170551	0.3507	0.587936	-0.42103	0.56277	0.469428	-0.26056	0.813442	-0.27679	2.227865	0.855225	-0.68606	0.138373	0.358968
Q9H019	MTFR1L	0.345714	1.158743	-0.49552	-0.09943	-0.06485	-0.01155	-0.53069	0.171783	-0.00769	-0.26031	1.280781	-0.0285	0.140465	0.305855	-0.02266	0.46471	-0.48851	0.989283	0.054605
Q9H061	TMEM126.	-0.04109	0.275705	0.195958	0.832142	0.217162	0.019246	-0.62221	-0.59756	-0.30321	-0.3531	0.122535	-0.01756	0.290363	-0.04305	0.922696	1.161729	-0.4921	-0.37289	0.155896
Q9H074	PAIP1	0.461424	-0.65906	0.669826	-0.00586	-0.27323	-0.00729	-1.1713	0.187349	-0.64999	-0.00137	-0.08814	-0.417	-0.27036	-0.46439	-0.01915	-0.10135	-0.49249	-0.43506	0.205664
Q9H078	CLPB	-0.21735	0.549674	0.643543	-0.11513	0.193502	0.227594	0.548462	0.219367	-0.08566	0.674116	0.286075	0.115097	0.433855	-0.09757	0.851329	0.340985	-0.61905	-0.26571	-0.71744

Q9H081	MIS12	0.382384	-0.23576	-0.32763	-0.56839	-0.03903	0.48975	0.130633	0.97369	-0.1562	-1.11046	-0.09992	-0.21562	-0.19003	0.287301	0.032847	-1.04471	-0.28909	-0.44077	-0.23781
Q9H082	RAB33B	-0.06335	0.319438	0.156374	0.014824	0.371846	0.192805	0.338949	0.062872	0.076981	0.023094	-0.24733	0.421039	-0.03693	0.668071	0.112619	0.499352	0.106676	-0.19033	-0.0909
Q9H089	LSG1	0.263623	0.502246	-0.2132	0.38682	0.203363	0.618238	0.218103	0.435125	-0.37667	0.25497	0.352384	-0.4651	-0.27394	-0.36249	1.780502	-0.09162	0.082697	-0.28356	0.009418
Q9H098	FAM107B	-0.98505	0.564264	-0.26703	-0.55491	-0.13307	-0.10557	0.043971	-1.12035	-0.12501	-0.05958	-0.61706	0.714899	0.140926	0.534288	0.124338	-0.22556	0.365973	-0.58228	-0.64323
Q9H0A0	NAT10	0.836014	0.049939	0.143442	1.071573	0.276139	0.525547	-0.18988	0.674975	-0.29045	-0.17397	0.370323	-0.05286	-0.35196	-0.24477	0.06674	0.011768	0.241794	-0.04608	-0.02366
Q9H0A8	COMMD4	-0.25339	0.581568	0.045375	0.242316	0.080163	0.188597	0.036048	-0.05159	0.38112	0.173149	-0.0849	0.258648	-0.03799	0.646185	-0.62512	-0.14279	0.122514	-0.22788	-0.0477
Q9H0B6	KLC2	-0.54987	-0.86158	0.106262	-0.71007	-0.33496	0.295834	0.052023	-0.20342	0.157213	-0.36624	0.200735	-0.32503	-0.13128	-0.1112	-0.29753	0.459099	0.368406	-0.2697	-0.01963
Q9H0B8	CRISPLD2	-2.18287	0.548015	0.47352	-1.12182	0.138335	0.47952	0.576212	-0.55766	0.520441	1.707319	-1.47762	-0.38239	0.629764	-0.32357	-1.77397	-0.04882	-0.7707	0.30405	1.6376
Q9H0C8	ILKAP	0.430223	-0.54978	-0.37583	-0.97445	0.424022	0.185979	-0.43749	0.716624	-0.8187	0.12619	-0.17665	-0.21042	-0.38773	-0.0533	-0.01735	0.068097	0.567136	-0.22991	-0.4314
Q9H0D6	XRN2	0.250312	-0.20018	-0.13967	0.387483	-0.16033	-0.09654	-0.10445	0.5855	-0.08081	-0.0692	0.273987	-0.08197	0.007805	-0.08332	0.036063	0.076523	-0.04322	0.009497	0.103774
Q9H0E2	TOLLIP	-0.60675	0.506964	0.323306	-0.33099	-0.28529	-0.26003	0.482883	-0.15596	-0.31303	0.87596	-0.1404	0.430349	-0.30629	0.827322	-0.15422	0.165762	-0.20427	-0.2876	-0.26633
Q9H0E3	SAP130	0.181635	0.082987	0.14516	-0.72527	-0.26854	-0.27214	-0.20954	0.371142	0.145954	-0.47833	0.086081	-0.10536	-0.05368	-0.45144	-0.00978	-0.25273	0.038499	-0.02579	-0.24885
Q9H0E9	BRD8	0.172788	0.297591	-0.34689	0.194567	-0.33812	-0.03311	-0.23571	0.700133	0.672853	-0.14727	0.404862	-0.82352	0.211598	-0.3771	0.352937	-0.10637	-0.59871	-0.03672	0.22677
Q9H0F6	SHARPIN	0.292438	0.219854	-0.03022	-0.57	-0.2546	-0.12401	0.043466	-0.41667	0.350405	0.61212	-0.10806	0.105135	0.017524	0.385129	0.240666	-0.16203	0.381087	0.042665	-0.04068
Q9H0G5	NSRP1	0.190756	-0.77512	0.494455	0.367392	-0.94234	0.290935	-0.59006	0.548774	-0.26389	-0.26354	0.861202	-1.36174	-0.15752	-1.06945	0.364168	1.102542	-0.70126	0.696074	0.405103
Q9H0H0	INTS2	-0.00406	0.202703	0.574441	-0.01937	-0.2378	-0.40447	1.037655	0.362696	-0.1203	0.233592	0.660004	-0.03847	-0.15048	0.005527	0.373422	0.057439	0.021265	-0.141	0.046887
Q9H0H5	RACGAP1	0.935704	-0.40035	0.179143	0.389667	2.037052	1.894083	0.269183	1.697433	0.113632	-0.67643	-1.05764	-0.61906	-0.04025	-0.31338	-0.6926	-0.30647	0.863534	0.652371	0.191586
Q9H0J9	PARP12	0.263272	0.136715	0.020786	-0.35521	0.293645	0.418481	0.620585	1.006813	0.977452	0.267612	-0.22945	0.050034	0.075286	0.201612	0.183627	-0.15451	-0.46942	-0.47075	-0.13301
Q9H0L4	CSTF2T	0.303787	-0.11714	-0.26767	-0.43974	0.035311	-0.24629	0.208249	0.010599	0.376515	0.070797	0.483687	-0.26434	0.046981	-0.21972	0.069949	-0.1258	-0.41224	0.279044	0.255749
Q9H0M0	WWP1	0.363706	0.03992	-0.11687	0.073802	-0.01949	-0.11313	-0.05873	-0.21663	-0.2546	-0.37046	0.394755	-0.7358	-0.10133	-0.26933	0.013463	0.031288	0.037607	0.468552	-0.21574
Q9H0N0	RAB6C	0.060005	0.093357	0.248391	-0.21338	0.69047	0.220177	1.472989	-0.12962	0.022513	-0.61774	-0.00303	0.233851	0.279551	0.348373	-0.10048	-0.06155	0.574237	0.136639	0.306323
Q9H0P0	NT5C3A	0.654681	0.39024	0.92278	-0.66385	-0.24788	-0.13915	-0.62615	0.598457	-0.55052	-0.45878	0.068696	0.02782	0.775982	0.176319	-0.72376	-0.48054	0.235525	-0.38241	-0.82955
Q9H0Q0	FAM49A	-0.96381	0.13679	0.153775	-0.77718	-0.08584	-0.04854	0.409406	-0.56525	0.255361	0.822632	-1.115	0.220629	-0.55537	0.668364	1.131107	0.1311	-0.20721	-0.21607	-0.34403
Q9H0R1	AP5M1	-0.30564	0.278758	-0.04208	-0.21592	-0.01206	-0.25606	0.102053	-0.04789	-0.25763	-0.2215	0.135853	0.266539	-0.09254	0.434689	-0.72868	-0.08326	-0.33141	-0.0729	-0.09619
Q9H0R4	HDHD2	-0.90634	0.012863	0.418191	-0.44813	0.659723	-0.6879	-0.04408	-0.91309	0.827294	1.514206	0.599859	-0.25833	0.111602	0.134318	0.191007	0.370835	-0.30668	0.541668	0.484273
Q9H0R6	QRSL1	-0.6218	-0.08381	0.129108	1.854714	0.411214	0.657553	-0.34412	-0.32745	-0.30267	0.374412	-0.18805	-0.38696	0.233098	-0.05827	0.848162	0.525443	0.304515	0.288887	0.559615
Q9H0S4	DDX47	0.955677	-0.14673	-0.48844	-0.02243	-0.64024	-0.5699	-0.38322	0.766174	-1.0011	0.298623	-0.25679	-0.21347	-0.54044	-0.06415	-0.2821	-0.01359	0.043698	-0.52463	-0.47565
Q9H0U3	MAGT1	0.378674	0.50943	0.248298	-0.68784	-0.0416	-0.0941	1.02158	-0.50988	0.552549	-0.29979	0.165333	0.358998	0.412132	0.358051	0.289488	0.214576	-0.11296	-0.00798	-0.28794
Q9H0U4	RAB1B	-0.10676	0.277064	0.34574	0.046673	1.125241	0.488898	0.052642	-0.3621	0.148485	-0.36326	-0.44897	1.200206	0.469592	0.702948	0.234236	-0.05493	0.302877	-0.1387	-0.58056
Q9H0U6	MRPL18	-0.50376	0.032616	0.614451	0.266931	-0.31532	0.134898	0.276841	0.052422	-0.0728	0.339255	0.803048	-0.77122	-0.00228	-0.6001	0.788589	-0.21592	-0.06157	0.130619	-0.81474
Q9H0U9	TSPYL1	-0.26211	-0.15844	-0.2119	0.34187	0.207944	-0.37998	0.286558	0.15794	-0.71465	-1.05002	0.179606	0.07005	0.035344	-0.2866	1.400089	-0.24519	-0.05381	0.436084	-0.68281
Q9H0V1	TMEM168	-0.13538	-0.23474	0.018409	0.766022	0.480201	0.169639	-0.20058	-0.18056	-0.31823	0.374712	0.579522	-0.06443	-0.1599	0.092838	1.984462	0.059959	-0.54129	-0.10621	-0.178
Q9H0V9	LMAN2L	0.125988	-0.15906	-0.35138	-0.04399	-0.55262	-0.19498	0.395431	0.371325	0.37116	-0.60292	0.212082	-0.01684	-0.21058	-0.17858	0.1028	-0.0634	0.69252	0.051378	0.681334
Q9H0W5	CCDC8	1.235939	-0.79868	-0.87285	0.238884	-1.43764	-1.30692	-0.31776	-0.77402	-0.56562	-1.22156	-0.38429	-1.37062	0.430273	-0.48263	1.011084	-0.82149	-0.26585	-0.20846	0.228102
Q9H0W8	SMG9	0.077875	-0.21982	-0.05915	-0.74292	-0.55004	0.197018	-0.11791	-0.06194	-0.22039	-0.25596	-0.34133	-0.72202	0.007403	-0.19183	0.26018	-0.20654	-0.08057	-0.49722	-0.07229
Q9H0X4	FAM234A	0.960573	0.092971	-0.17967	-0.01876	-0.36186	-0.19756	0.035993	0.381948	-0.00057	-0.52687	0.114676	-0.95374	0.388469	-0.26683	-0.66607	0.405961	-0.36117	0.233316	0.716225
Q9H0X9	OSBPL5	-1.73546	0.374459	-0.03771	-0.72206	-0.14288	-0.12488	-0.12031	-0.148	0.174075	0.039079	-0.26591	-1.28094	0.063521	-1.01098	-0.90737	-0.56908	0.040007	1.193104	0.263872
Q9H115	NAPB	-0.3678	0.287092	-0.46446	-0.22621	-0.46423	-0.36118	-0.01159	-0.25903	-0.04378	0.072904	-0.67658	-0.34061	0.174411	0.283566	-0.69882	-0.20651	0.151694	-1.01823	-0.59066
Q9H147	DNTTIP1	0.259007	0.029629	-0.09769	0.416076	-0.06215	0.234058	-0.06646	0.497471	-0.05985	-0.18645	-0.15833	-0.60583	-0.21649	-0.05537	0.183089	-0.39821	0.482345	0.82796	-0.30017
Q9H173	SIL1	-0.57595	0.167537	0.220498	0.464696	0.740272	0.924905	-0.43151	0.032617	0.431347	1.306225	0.123732	-0.3598	-0.3422	-0.36292	-0.46268	0.391311	0.276926	0.913052	-0.42129
Q9H1A3	METTL9	0.319622	0.219233	0.338503	-0.10951	-0.67734	-0.502	0.021949	-0.48363	-0.42418	0.294474	-0.48839	0.184396	0.300914	0.511706	-0.04413	0.13148	1.011305	1.043255	-0.18288
Q9H1A4	ANAPC1	0.129378	-0.16374	0.516953	0.264324	-0.01997	0.301078	0.456712	0.26342	0.923104	-0.06208	0.194282	-0.1765	-0.40295	-0.02822	0.20745	0.413218	0.336605	-0.0185	0.502954
Q9H1B7	IRF2BPL	0.210038	-0.3769	-0.47251	0.17626	0.080739	0.562843	-0.08252	1.04083	-0.08769	-0.81307	0.355933	-0.7273	-0.11086	-0.69901	-0.0422	0.864628	-0.50395	-0.29298	0.148544
Q9H1E3	NUCKS1	-0.03567	-2.39478	-0.65013	-0.65617	0.136338	-1.33568	-0.85543	-1.08037	-0.35379	-0.44751	-0.22836	-0.65178	-0.81711	-0.64652	0.611083	-0.77409	-0.52572	-0.24674	-1.07688
Q9H1E5	TMX4	-0.13143	0.264093	-0.01789	-0.50848	-0.18433	-0.0448	0.113133	-0.25884	0.004338	-0.08192	0.24642	-0.03298	-0.08198	0.440233	-0.11283	-0.55743	-0.04005	-0.01747	-0.41227
Q9H1H9	KIF13A	0.204788	-0.34744	-0.17607	0.625904	-0.32453	-0.36173	-0.65143	-0.70944	-0.13736	-0.29339	-0.25782	-0.04102	0.001395	0.282797	0.456056	0.007791	-0.33644	-0.57421	0.130508
Q9H1I8	ASCC2	-0.44442	0.596901	0.154977	0.422005	0.039369	-0.18403	-1.45447	-0.54942	0.944837	0.470467	0.161615	-0.09714	-0.22527	-0.20954	0.355174	-0.0828	0.176985	0.328411	-0.00188
Q9H1K0	RBSN	0.388305	0.578044	0.850761	-0.82551	0.102969	-0.21937	0.432664	-0.5229	0.140326	-0.37074	-0.35441	-0.5098	0.111221	-0.32222	-0.63147	0.051551	-0.48577	0.003232	0.02471
Q9H1K1	ISCU	-0.07217	-0.00236	0.023829	0.132399	0.314325	-0.49714	0.142729	-0.0514	0.083344	-0.25582	0.068856	-0.08907	-0.11931	-0.36438	0.87853	0.009213	-0.7214	-0.24356	-0.32453
Q9H1P3	OSBPL2	-0.09358	0.128835	0.209708	0.073986	0.532146	0.123293	0.006792	0.300898	-0.04995	0.478293	-0.13479	0.395105	0.047701	0.246643	-0.25461	0.180877	0.360964	0.292199	0.55301
Q9H1X3	DNAJC25	-0.3495	0.246766	-0.10266	0.518525	0.989652	0.32112	0.206697	-0.48698	0.027162	-0.32053	0.356184	0.179206	0.054642	0.045764	-0.41543	-0.60433	-0.13232	-0.00038	0.357284
Q9H1Y0	ATG5	-0.06898	0.125838	-0.71582	-0.26837	-0.06239	0.371851	-0.83971	-0.49069	0.60346	-0.0621	0.179729	0.443307	-0.23584	0.206203	0.107763	0.37229	0.095146	-0.26209	-0.36869
Q9H1Z4	WDR13	-0.1289	-0.56663	-0.99363	-0.399	-0.61178	-0.5382	0.024815	-0.6387	-0.05087	0.484259	0.885805	-0.48543	-0.65002	-0.21318	-0.17145	0.457731	-0.43893	-0.41755	-0.54902
Q9H223	EHD4	-0.37214	0.963884	0.887204	-0.75642	0.190006	-0.44252	0.297671	-0.74104	0.072279	0.320135	-0.26316	1.234125	-0.00082	0.618643	-0.47787	0.195314	0.010248	-0.22623	-0.53744
Q9H257	CARD9	-0.12017	0.790939	1.304808	-0.81451	0.013882	0.066585	0.306376	-0.44503	0.002868	-0.02146	0.427407	0.641478	-0.32454	1.009947	-0.11055	-0.04851	0.290285	-0.4294	-1.11669
Q9H267	VPS33B	-0.31758	0.625734	0.137226	0.409936	-0.36082	-0.27691	0.3684	-0.03857	-0.16799	0.284821	0.317355	0.433216	0.230785	0.492863	-0.19678	-0.39568	0.165884	-0.13282	0.13318
Q9H269	VPS16	-0.16109	0.566397	0.358496	-0.05529	0.094731	-0.0585	0.28344	-0.20477	0.030244	0.416652	-0.03164	0.391678	-0.21357	0.667805	-0.17465	-0.20205	-0.15029	-0.29375	-0.01504
Q9H270	VPS11	-0.04483	0.511413	0.356072	0.079097	-0.04594	-0.20214	0.011406	-0.2787	-0.61825	0.215177	-0.05892	0.542431	0.490762	0.512538	-0.2177	-0.00356	-0.00103	-0.16108	0.022448
Q9H299	SH3BGRL	-2.25712	1.357948	-1.28366	-2.73688	1.469412	-0.47909	0.29522	1.121609	0.905942	2.203199	-1.54985	0.323425	0.130569	1.045329	-1.3842	-0.7698	0.392606	0.845033	-0.81478
Q9H2C0	GAN	-0.84679	0.381188	0.138295	-0.06936	-0.51524	-0.38655	0.506122	-0.82777	0.242941	0.025215	-0.49777	-0.35278	0.481275	-0.01502	-0.0306	1.123046	0.66199	0.024182	1.044543
Q9H2D6	TRIOBP	-0.7368	0.648042	0.003804	-0.07141	-0.01393	-0.13219	-0.08636	-0.45164	-0.03154	-0.35437	0.410659	-0.84436	-0.08937	-0.55851	-0.22417	0.303902	-0.13242	1.039822	0.776249
Q9H2G2	SLK	-0.55599	0.062001	0.294811	-0.60007	-0.25344	0.031327	-0.13405	-0.40983	0.056621	0.283257	-0.10041	-0.59065	-0.00733	-0.08939	0.267968	-0.5132	-0.25647	0.008115	0.04938
Q9H2H8	PPIL3	0.136109	0.199144	0.26553	-0.8045	0.238628	-0.30906	0.837061	-0.62676	0.040847	0.140411	-0.11781	0.331414	0.05075	0.939092	0.079413	0.16794	0.849768	0.210316	0.0693
Q9H2I8	LRMDA	-0.13417	0.54153	0.228282	-0.00886	0.392876	0.882427	0.24272	-0.25072	0.492775	0.05026	-0.02174	0.48696	0.300246	0.328846	0.911801	0.063509	-0.01231	-0.13537	0.700104
Q9H2J4	PDCL3	0.10924	-0.34277	0.353739	-0.24772	-0.3447	0.197946	-0.89404	0.377379	0.360825	-0.16531	0.146082	-0.33172	0.563519	-0.34226	-0.36659	0.312477	0.520663	0.609297	0.384168
Q9H2K0	MTIF3	-0.62166	0.193846	-0.31449	-0.25157	0.621157	-0.08428	-0.13304	0.102391	0.564827	0.806099	-0.25225	0.175357	0.266071	0.243398	-0.03477	-0.80072	0.07205	0.108817	-0.68311
Q9H2K8	TAOK3	-0.81463	0.940474	0.636056	0.156409	0.066295	-0.31238	0.242307	-0.83489	-0.23534	0.649661	-0.91138	0.624794	-0.23269	0.681621	-0.01616	0.258977	0.674314	0.934311	-0.41303
Q9H2M9	RAB3GAP	-0.00398	0.098458	-0.09511	-0.16948	-0.19758	-0.4447	-0.0209	0.005101	0.139069	0.374014	-0.25963	0.293565	0.059116	0.43524	0.648481	-0.04723	0.05084	-0.64136	0.137061
Q9H2P0	ADNP	0.819846	-0.62799	-0.90194	0.062341	0.024005	0.774959	-0.01121	1.178765	0.518647	-0.28679	0.017992	-0.93517	-0.04808	-0.53089	0.660839	-0.71811	-0.18162	0.250423	0.256491
Q9H2P9	DPH5	0.03014	-0.33878	-0.09469	0.574172	0.299333	-0.39982	-0.49977	0.480799	-0.18139	0.250907	0.217783	-0.1444	0.041922	0.14685	-0.44551	0.205127	0.444613	-0.77537	-0.01721
Q9H2U1	DHX36	0.268028	-0.00985	0.570621	0.091013	0.875238	1.214252	-0.40195	0.106742	0.166325	0.214476	0.248381	-0.35753	0.179596	-0.37248	0.579711	0.406525	-0.28995	-0.00463	0.796418
Q9H2U2	PPA2	0.038206	-1.04951	-0.38886	0.2708	0.76748	-0.29878	-1.33267	0.414453	0.872957	1.15312	1.552949	0.656224	0.667893	0.234769	1.006629	0.125638	-0.26761	-0.11786	-0.69314
Q9H2V7	SPNS1	0.286931	0.070625	0.235487	-0.27594	-0.17321	-0.21848	0.044008	0.354374	0.006073	0.296981	-0.30863	0.572814	-0.38492	0.557272	-0.80242	-0.19349	-0.21055	0.51833	0.152938
Q9H2W6	MRPL46	0.219422	-0.6483	-0.23267	0.814258	0.175209	0.262705	-0.41173	-0.11384	-1.16225	0.85337	0.730797	-0.03356	-0.28327	-0.11351	-0.08142	-0.01323	0.216224	0.324823	-0.47336
Q9H300	PARL	0.537067	-0.24873	0.157666	0.542641	0.780118	0.75942	-0.69083	0.3965	-0.03853	0.192638	-0.24739	0.276787	0.372206	-0.00222	0.956211	0.543182	-0.17414	0.008426	-0.20285
Q9H307	PNN	0.68862	-0.24636	-0.1715	-0.02295	-0.26173	0.296577	-0.43383	0.475202	-0.15076	-0.17024	0.24161	-0.93774	-0.42662	-0.85915	0.21094	-0.32525	-0.29605	0.35368	-0.07713
Q9H329	EPB41L4E	0.548384	0.087255	-0.33048	-0.07245	-0.44246	-0.55165	-0.22975	-0.71778	0.087097	-0.12448	0.15284	-0.82656	0.185478	-0.04184	-0.34636	0.147338	0.307733	0.23406	-0.80012
Q9H330	TMEM245	0.231451	-0.14179	-0.13908	-0.86988	0.174052	0.034135	-0.08434	-0.2705	-0.18781	-0.726	0.102013	0.025546	-0.5022	-0.41025	0.397534	-0.23255	0.014638	0.255001	-0.21754
Q9H3F6	KCTD10	-0.31901	0.531328	0.340937	0.117199	-0.0663	0.030475	-1.03293	-0.66634	0.094662	-0.18029	-0.2754	-0.13975	0.72555	0.059107	-0.02361	-0.05809	-0.0953	0.159354	0.54454
Q9H3G5	CPVL	-1.78325	1.46753	0.179776	-0.33285	-0.11397	-0.88114	-0.35416	-0.24145	0.309544	1.165254	-1.84066	0.904536	0.971089	0.576117	-0.2344	0.322434	0.250537	1.825853	-0.91296
Q9H3H1	TRIT1	-0.48496	-0.13892	0.308541	-0.55056	-0.24131	0.019987	-0.01784	-0.34519	0.449577	-0.05725	0.064638	0.00701	-0.12017	-0.36939	-0.33556	0.066165	0.087113	-1.06239	-1.20141
Q9H3H3	C11orf68	-0.18579	0.195354	0.074156	-0.10754	0.607003	-0.06791	-0.75384	0.027336	0.185682	0.730936	0.000537	-0.28253	0.212447	-0.34163	-0.26451	0.606724	-0.2138	0.55391	0.170692
Q9H3K2	GHITM	0.356742	-0.12678	-0.1388	-0.09617	-0.02179	-0.30351	-0.28554	-0.25328	-0.11063	0.790252	0.253439	0.050745	-1.1206	-0.15695	0.828478	1.420214	-0.13494	-0.47508	-0.83993
Q9H3K6	BOLA2	-0.32864	-0.60581	0.678241	-0.45451	0.508676	-0.45062	0.25162	-0.32843	0.628823	1.132499	-0.84652	0.842212	-0.18946	1.076501	-1.0487	0.265481	1.271138	0.031544	-0.90211
Q9H3M7	TXNIP	0.439537	0.413417	0.61721	0.775745	0.047577	-0.45738	-0.44707	0.260147	1.135335	1.847638	0.590719	0.834288	0.753532	0.580516	0.45054	-0.45799	0.515806	0.723176	-0.49169
Q9H3N1	TMX1	0.43884	0.569839	0.139499	0.354718	-0.37177	-0.06511	0.588611	-0.28566	-0.62513	0.134679	0.27742	0.486608	-0.16554	0.009944	-0.00847	0.396052	0.572797	0.396461	-0.14106
Q9H3P2	NELFA	0.733062	-0.63416	-0.41117	-0.04762	-0.58373	0.057771	-0.53106	0.187714	0.437308	-0.27979	-0.45627	-0.34464	-0.0111	-0.35952	-0.15189	-0.08188	-0.52397	-0.47629	0.295389
Q9H3P7	ACBD3	-0.38407	-0.64543	-0.00676	0.068568	0.075213	-0.37723	0.173054	-0.09445	0.474255	0.56978	-0.48137	0.018254	0.587308	-0.27054	1.458611	0.325531	0.395963	0.949546	0.140292
Q9H3Q1	CDC42EP	1.164265	-0.91017	-0.7215	-0.67999	-2.03626	-0.62535	-0.38427	0.579438	0.088802	-0.56578	0.530143	-1.13385	-0.25217	-0.5403	-0.19457	0.068784	-0.38445	-0.6916	0.034121
Q9H3S7	PTPN23	0.043424	0.29464	0.139008	-0.74643	-0.18806	-0.07021	-0.01005	-0.04492	-0.03015	-0.26485	0.162701	-0.39313	-0.00889	-0.28228	0.195072	-0.25273	-0.68366	-0.21448	0.053549
Q9H3U1	UNC45A	0.243839	-0.03349	-0.08351	0.82282	0.343129	0.899138	0.225625	0.129663	0.196532	0.185858	-0.15042	-0.04512	-0.08638	0.024001	-0.43921	-0.17785	0.018535	-0.5336	-0.19337
Q9H3U5	MFSD1	-0.92705	1.051056	0.67321	-0.56683	0.873018	0.576997	1.408892	-0.64001	-0.06467	0.775582	-1.60578	1.710056	-0.12693	1.042903	0.341508	-0.48863	-0.63256	-0.62285	-0.05144
Q9H3Z4	DNAJC5	-0.1526	0.134793	-0.31107	-0.16771	-0.13725	-0.32451	0.465703	-0.05492	0.160083	-0.08685	-0.34141	0.82054	0.29813	1.396097	0.174737	-0.30203	0.671402	-0.24715	-0.35333
Q9H410	DSN1	1.3923	-0.03868	0.004761	-0.2675	0.146853	0.190614	-0.07564	0.295514	0.035244	-0.20583	0.139216	-0.06086	-0.32955	0.05626	0.287701	0.476502	0.183144	-0.35041	-0.2499
Q9H425	C1orf198	1.235102	-0.40775	-0.90069	-0.39656	-1.97878	-0.94734	-0.58456	-0.6213	0.335557	-0.63216	-0.51338	-1.00094	0.933114	-1.04349	0.538803	-0.46212	-0.78228	-0.23057	0.114431
Q9H444	CHMP4B	0.745242	-0.136	0.456492	-0.36459	0.491601	0.345692	-0.41327	0.651709	0.083328	0.173696	-0.35407	0.094037	-0.34275	0.710739	0.157141	0.574809	0.139478	-0.1963	-0.12858
Q9H446	RWDD1	-0.78801	0.207502	0.944526	0.392705	0.534936	0.764735	-1.0393	-0.43432	0.319288	0.769251	0.433908	-0.93251	-0.30894	-0.59687	-0.01256	0.049826	-0.41665	0.132593	0.478505
Q9H479	FN3K	-0.0849	-0.97318	-0.50026	0.752775	-1.17603	-0.47738	0.037961	-0.78896	-0.1005	-0.80032	-0.06686	-1.26435	-0.45539	-0.32126	-0.04692	-0.04029	-0.04831	0.004624	-0.76977
Q9H488	POFUT1	1.303899	-0.08535	-0.05556	0.114403	-0.06446	0.227004	-0.42066	0.4614	-0.50945	-0.07738	-0.03125	-0.73888	0.054846	0.029661	1.362518	-0.26298	0.054624	0.008478	0.459297
Q9H490	PIGU	0.584013	-0.4248	-1.11586	0.084148	-0.14003	-0.08476	0.513907	0.825172	0.103639	0.655666	-0.15192	0.164941	0.395436	-0.1658	0.339631	1.049172	-0.07502	-0.42171	0.522791
Q9H497	TOR3A	-0.05392	-0.1787	-0.30384	0.287269	-0.30509	0.184458	-0.00664	0.405074	-0.36059	-0.02175	-0.12703	-1.19177	-0.51287	-0.2821	0.373495	-1.41448	-0.34388	-0.35286	-0.04165
Q9H4A3	WNK1	0.254819	0.030207	-0.88142	-0.17944	-0.61887	-0.52138	-0.34979	-0.06207	0.061775	-0.09272	-0.57948	0.10204	1.146561	-0.11121	-0.46178	1.942667	-0.06245	-0.20921	-0.08968
Q9H4A4	RNPEP	-0.29443	0.163315	0.264277	-0.39302	0.236397	0.722114	-0.02151	0.463273	-0.42532	0.367552	-0.14877	-0.12852	0.18727	0.264786	-0.04564	-0.73866	-0.40816	-1.00519	0.034249
Q9H4A5	GOLPH3L	-0.55022	-0.44756	0.028604	0.282254	0.02555	-0.04881	-0.40475	0.345255	0.163718	0.189587	0.124337	-0.03621	0.733891	-0.54354	0.652825	0.094328	0.157018	-0.58693	-0.27146
Q9H4A6	GOLPH3	-0.21096	0.473073	0.357826	-0.07726	0.367681	0.565735	0.482261	-0.71438	-0.1678	0.10309	-0.42871	-0.15058	0.062915	-0.28997	0.032778	0.340608	1.007058	0.911362	0.089334
Q9H4B0	OSGEPL1	0.43127	0.16302	-0.38852	0.300896	-0.69642	-1.09051	-0.12753	-1.08573	-0.20223	-0.6916	0.671853	-0.82428	-0.05885	0.095854	0.333208	0.442418	0.313371	-0.8566	0.168583
Q9H4B7	TUBB1	-0.79434	0.38635	0.992501	0.597013	1.870173	-1.11083	0.072351	-0.39663	0.813792	0.070164	-0.8599	-0.95095	-0.17663	-0.42734	-0.49684	-2.22858	-0.54321	2.683224	0.710043
Q9H4B8	DPEP3	-0.15844	-1.7591	-0.2269	-0.73478	-0.36557	-0.94792	0.356731	-1.6415	-0.36222	-0.6655	0.211806	-0.41985	-0.04459	-0.29748	-1.49553	-0.30459	-1.02858	-0.55475	-2.20576
Q9H4E7	DEF6	-1.08235	1.317259	-0.25375	-0.53208	0.448452	0.131821	0.069529	-1.37711	0.666267	0.846963	-1.16351	0.98162	0.022282	1.283279	-0.52826	-0.37914	0.267231	0.569435	-0.84009
Q9H4G0	EPB41L1	-0.378	-0.92648	-0.98494	0.287832	-1.09804	-0.46287	-0.79306	0.243396	-0.64932	0.257295	0.492177	-1.07195	-0.92038	-0.53512	1.676316	-1.2274	0.439101	0.715798	-1.48769
Q9H4G4	GLIPR2	0.258762	-0.37437	0.048647	0.213346	-0.25058	-0.71836	-1.08162	-1.28553	0.078133	0.495978	-0.4572	-0.30735	-0.06095	0.572969	-0.16536	-0.23699	-0.16693	-0.04151	0.181258
Q9H4I2	ZHX3	-0.53141	-0.55307	-0.23185	-0.25802	0.025222	0.430233	0.676564	-0.58304	0.21668	-0.36839	-0.09174	-0.28897	-0.05387	-0.04059	-0.20124	-0.66555	0.02951	1.334589	-0.27032
Q9H4I3	TRABD	-0.7763	0.54841	0.237671	0.366471	0.799135	0.322278	0.550217	-0.43517	-0.24292	-0.02453	1.025187	0.041214	-0.44596	-0.02962	-0.34376	-0.23286	-0.09238	0.399847	-0.76999
Q9H4K7	MTG2	-0.0624	-0.0843	-0.25138	-0.03669	0.376969	0.402144	0.214494	0.545469	-0.05869	-0.179	-0.13097	0.362156	-0.22464	0.148191	0.376839	-0.21735	0.067295	-0.28581	0.183405
Q9H4L4	SENP3	0.682967	0.13605	0.08917	-0.19401	0.400999	0.560121	0.107419	0.462985	-0.10612	-0.4151	-0.11461	-0.15846	0.142127	-0.26614	0.07165	-0.18285	0.240139	-0.36151	0.408272
Q9H4L5	OSBPL3	-0.06915	0.007184	-0.25528	-0.48038	-0.3439	0.187615	0.016535	-0.02913	0.149892	-0.44792	-0.25211	-0.33438	-0.71371	-0.06662	1.19377	0.181025	-0.24996	-0.86681	-0.76381
Q9H4L7	SMARCAD	0.977505	-0.10349	-0.93255	-0.30759	0.119728	0.133579	-0.35093	0.095705	-0.07153	0.159328	0.440703	-0.54025	-0.12522	-0.88173	-0.15497	-0.45315	0.000104	0.02515	0.086307
Q9H4M7	PLEKHA4	0.432467	0.294201	-0.20545	0.509659	-0.20258	-0.63672	-1.14276	0.433583	0.040847	0.934552	-0.09332	0.065892	-0.03742	-0.43153	-0.17799	-1.46858	-0.20978	0.379469	-0.45178
Q9H4M9	EHD1	-0.44644	0.209334	0.291481	-0.45677	-0.00122	-0.02433	0.047188	-0.40451	-0.07438	0.266107	-0.47061	-0.12684	-0.31815	0.808278	-0.30017	-0.3968	0.138493	-0.00547	-0.49977
Q9H4Z3	PCIF1	0.2806	-0.80369	-1.01825	0.111607	-0.56152	-0.05756	-0.56554	0.678174	-0.3477	-0.23634	-0.17592	-0.20782	-0.02218	0.137879	-0.00353	-0.36686	-0.02377	0.004069	0.051519
Q9H501	ESF1	1.023835	-0.11892	-0.33663	0.499079	-0.43299	0.324686	-0.57516	0.828762	-0.10621	0.354578	0.087308	-0.14166	-0.07695	-0.1026	-0.24443	-0.21423	-0.14784	-0.40634	-0.19661
Q9H553	ALG2	-0.9437	0.502382	-0.22246	-0.09913	0.400432	0.339251	0.794852	-0.27051	-0.72557	0.044265	0.752962	0.483925	-0.32168	0.65341	-0.58249	0.562838	0.139048	0.125266	0.07823
Q9H582	ZNF644	0.751299	0.072876	0.098447	-0.26169	0.349155	0.396389	-0.06624	0.786251	0.062288	-0.02645	-0.34843	0.417266	0.251274	0.322759	-0.06952	-0.10218	0.670992	0.037446	0.125198

Q9H583	HEATR1	0.302361	0.173873	0.085644	0.818969	-0.06728	0.043219	-0.45878	0.403768	-0.32021	0.215049	0.533491	0.017679	0.069255	-0.30046	0.245086	0.396749	-0.07963	-0.53885	-0.05201
Q9H5H4	ZNF768	0.621504	-0.39077	-0.22575	-0.20092	1.489739	-0.11247	-0.31685	0.250633	0.477448	0.146038	-0.00704	-0.06252	0.070351	-0.58661	0.190383	-0.0365	0.434961	-0.24044	0.430619
Q9H5N1	RABEP2	-0.07007	-0.45933	0.84046	0.183697	-0.23512	-0.09692	-0.3478	-0.11481	0.772833	0.503083	-0.03511	-0.36447	0.161483	-0.16799	0.028736	-0.13854	-0.57298	0.011469	0.520099
Q9H5Q4	TFB2M	-0.21951	0.240585	0.135732	0.307633	0.053026	0.376826	-0.10216	0.392897	0.017043	1.106378	-0.30085	0.286452	0.287217	-0.09034	1.890421	0.488478	-0.25316	-0.24746	-0.19339
Q9H5V8	CDCP1	0.82466	-0.29304	-0.19524	0.227895	0.466614	0.157449	0.835114	-0.3717	-0.20747	-0.89349	0.141724	0.60478	-0.50713	0.177084	-0.18863	0.493763	-0.12906	-0.5093	0.433828
Q9H5V9	CXorf56	0.16773	-0.3613	-0.25775	0.380101	-0.00424	-0.27627	-0.65862	0.140202	-0.28317	0.044457	0.369343	-0.18963	0.692851	0.057014	0.585352	0.217512	0.604156	0.001901	-0.64442
Q9H5X1	FAM96A	-0.13386	0.239747	0.72616	0.292469	-0.24822	-0.50245	-0.49647	-0.66073	0.010769	0.547312	-0.07553	0.819221	0.0655	0.493789	0.23558	0.308979	0.529249	-0.2011	-1.1027
Q9H5Z1	DHX35	-0.28297	0.286586	0.23835	0.153202	0.613131	0.638952	0.266607	0.382903	0.499603	-0.12855	-0.41573	-0.00661	0.318926	0.210054	0.791688	-0.61642	0.225173	0.157955	0.712109
Q9H694	BICC1	-0.65645	0.834103	0.004912	-0.62758	-0.55109	-0.58343	0.100906	-0.94334	1.242356	-0.09843	-1.09866	-1.33897	0.856434	-1.40942	-0.64635	0.101072	1.085776	2.013552	1.557956
Q9H6A0	DENND2D	0.468312	0.73838	-0.30662	0.022691	0.025063	0.122799	-0.01659	0.056537	0.601748	0.392737	0.307249	-0.5399	-0.77913	0.058941	0.254323	0.010604	-0.1671	-0.17644	-0.7651
Q9H6D7	HAUS4	0.390435	0.124878	0.254761	-0.4155	0.465716	0.614057	-0.03986	-0.06359	-0.063	-0.21835	-0.14101	-0.05649	-0.19144	0.127471	-0.88101	0.650325	0.006043	0.168309	0.047456
Q9H6E4	CCDC134	0.146573	-0.41377	-0.37069	-0.44125	0.270569	-0.02193	-0.46677	-0.04137	-0.86536	-0.98427	0.100235	0.071288	0.099816	0.34139	0.018505	0.007128	0.704282	0.677924	-0.33323
Q9H6E5	TUT1	0.530413	-0.14492	0.457211	-0.29626	-0.04593	-0.00058	-0.0897	-0.04555	0.080341	-0.25188	-0.21297	0.039662	-0.01888	-0.27708	-0.83618	-0.13542	0.54994	-0.31221	-0.29103
Q9H6F5	CCDC86	0.077774	-0.36412	-0.04918	-0.10193	-0.74133	0.024152	-0.70565	-0.56333	-0.17977	0.060953	0.453184	-0.96598	0.354107	-1.00765	0.378532	0.839257	0.097987	0.27792	-0.1543
Q9H6H4	REEP4	0.078541	0.157112	0.075995	0.213143	0.097837	-0.26046	1.685921	0.242141	0.040847	0.263967	-0.25835	0.299488	-0.19336	0.780236	0.329182	0.376382	0.482867	-0.20948	0.015584
Q9H6I2	SOX17	1.046186	0.014557	-0.32827	-0.04516	-0.24738	-0.18116	-0.53948	0.934227	0.280118	-1.19827	0.942361	-1.27672	0.667465	-0.81977	0.175796	-0.32637	-0.18085	-0.63098	-0.40218
Q9H6K4	OPA3	0.057656	0.064831	-0.0871	0.003801	-0.55875	-0.21573	0.273014	-0.21081	-0.21633	-0.10137	0.302025	-0.10653	0.140992	0.234642	1.300202	-0.21378	-0.06398	0.068608	-0.55573
Q9H6K5	PRR36	0.326445	-0.04454	-0.55139	-1.29442	-0.40314	-1.38084	-0.23679	-1.0785	-0.08979	-0.38932	0.231999	-0.5319	-0.32818	-0.47565	-0.4545	-0.14386	-0.55404	-1.69452	-0.63797
Q9H6L2	TMEM231	-1.93342	-0.04045	-0.44287	-0.01265	-0.17061	0.216061	-0.47664	-1.09074	-0.33439	0.161717	2.284802	-0.3003	-0.77101	-0.76897	0.303704	0.150399	-0.7985	-1.72707	-0.14974
Q9H6Q4	NARFL	-0.00564	-0.41134	-0.20366	-0.60164	-0.2915	-0.21738	-0.23849	-0.05081	-0.29851	-0.76089	0.095966	-0.1939	0.413102	0.154817	-0.14371	0.470466	0.696773	-0.03306	0.171908
Q9H6R0	DHX33	0.473858	-0.10865	-0.06437	0.33047	0.040716	0.011897	-0.15449	-0.01081	-0.17817	-0.38149	0.357637	-0.06361	-0.08472	-0.21238	0.226459	-0.55991	0.026686	-0.14436	-0.70361
Q9H6R3	ACSS3	-2.10695	-0.09197	-0.04925	0.819848	-1.77164	-1.33103	-0.51071	-0.27449	-0.15228	0.559083	-0.39572	-1.32963	-0.95899	-1.03395	0.300398	0.267703	-0.76384	0.254191	0.285222
Q9H6R4	NOL6	-0.08058	-0.08001	-0.86576	0.569396	-0.41466	-0.15647	-0.82928	0.213932	0.105938	0.264323	0.575517	-0.23778	-0.06281	-0.4804	0.062705	-0.0495	-0.23311	-0.3281	-0.25453
Q9H6R6	ZDHHC6	-0.50227	0.350752	0.302405	-0.16053	0.097874	0.247873	-0.00993	0.449054	0.08937	0.187464	-0.28198	0.929408	0.071239	0.783009	-0.13096	0.121406	0.636855	-0.7503	0.272949
Q9H6S0	YTHDC2	-0.76885	0.129498	0.492163	-0.71733	0.087248	-0.03384	0.050414	-0.25571	-0.66987	-0.02048	0.003232	0.221282	0.784994	0.337949	-0.19053	0.319324	0.240126	-0.00627	-0.35096
Q9H6S1	AZI2	-1.41004	0.236319	0.201298	-0.48536	0.174752	-0.01245	0.10789	-1.00644	0.109378	0.166502	-0.25638	-0.01834	-0.24704	0.274498	-0.51193	-0.14656	-0.58915	0.479851	0.37645
Q9H6S3	EPS8L2	-0.17853	-0.8365	-0.37189	-0.17858	0.545971	0.931185	0.089034	1.846395	0.063131	-1.0482	0.217862	-0.71515	0.226216	-1.02402	-0.13481	1.322596	1.67734	1.183983	-0.03594
Q9H6T0	ESRP2	0.758703	-0.24905	-0.43888	0.821253	-0.37495	0.039005	-0.37287	-0.13268	-0.62367	-1.25891	0.821419	-0.92495	-0.44174	-0.64145	0.524779	0.63148	0.505352	-0.02445	-0.77929
Q9H6T3	RPAP3	0.157141	-0.07648	0.281062	0.010196	0.036355	0.480167	-0.08843	0.678599	-0.96007	0.146006	0.20584	-0.51315	0.021405	-0.71473	0.499457	-0.33008	-0.22171	-0.49024	0.374422
Q9H6U6	BCAS3	-0.58149	0.52896	0.355483	-0.06584	-0.19903	0.27627	0.199975	-0.28395	-0.00149	0.032931	-0.00632	-0.40327	0.070087	-0.03583	0.159102	0.28638	0.07679	0.123948	0.385491
Q9H6U8	ALG9	-0.55096	-0.3222	0.080972	0.184901	-0.51409	-0.70904	0.213814	-0.23696	-0.03152	-0.56894	-0.01143	0.141406	-0.28529	-0.07691	0.273076	-0.06144	0.1903	-0.23949	-0.19393
Q9H6V9	LDAH	-0.15564	0.519868	0.004356	-0.32057	-0.518	-0.90582	0.380781	-1.40868	0.371418	-0.01656	-0.24147	-0.11876	0.145046	0.021105	0.118081	0.938125	-0.13172	-0.16718	0.837112
Q9H6W3	RIOX1	0.500296	-0.31132	-0.53529	0.475634	-0.5048	-0.73603	0.237131	0.174107	-0.16985	-0.30681	-0.26897	0.615459	0.244178	-0.25816	0.051932	0.323667	0.063811	1.458312	0.319849
Q9H6X2	ANTXR1	-1.28609	-0.34437	-0.0395	-1.07924	-0.99924	-1.4335	0.805403	-0.59481	0.062842	-1.09988	0.137296	-1.33567	-0.30823	-1.81351	-1.30105	0.260508	-1.15399	-0.72426	0.961801
Q9H6Y2	WDR55	0.403399	-0.06325	0.184137	0.787064	0.359848	0.090723	-0.24134	0.008967	-0.13521	-0.76845	0.657086	-0.24076	-0.2242	-0.57379	0.475475	-0.44657	0.2508	0.63876	-0.22838
Q9H6Z4	RANBP3	-0.27992	-0.35193	-0.28224	-0.71398	0.243347	-0.29153	-0.3578	-0.07604	0.129993	0.12292	0.081432	-0.73193	-0.41616	-0.36839	-0.14009	-0.66464	-0.65521	-0.11895	0.015061
Q9H773	DCTPP1	1.547027	-1.1083	-0.31595	-0.29858	1.409715	0.999977	-0.27844	0.324002	0.666833	-0.61845	0.959091	-0.63629	-0.09811	-0.97745	0.362293	0.097369	-0.67977	-0.83609	-0.97632
Q9H788	SH2D4A	0.277699	0.123959	-0.09658	-0.22929	-0.40333	0.36997	-0.07721	0.558057	0.280214	-0.61513	0.36652	-0.73894	-0.37224	-0.46695	0.098125	0.529701	0.993732	0.066442	0.621884
Q9H792	PEAK1	-0.21203	0.34727	0.359643	-0.62766	0.241062	-0.49197	0.158354	-0.09859	0.318108	0.737199	-0.85813	-0.72915	0.580719	-0.30349	0.199125	-0.35132	-0.89642	0.185669	0.544416
Q9H7B2	RPF2	-0.18384	-0.0608	0.454058	1.071698	0.010016	-0.32568	-0.34242	-0.5591	0.373084	-0.47528	1.05402	0.704908	0.389819	-0.5527	0.397062	0.122101	0.378076	0.465275	-0.5255
Q9H7B4	SMYD3	0.329611	-0.06183	-1.01048	0.570799	0.206266	1.651514	-0.32743	0.184649	-0.13969	0.141562	0.027258	-0.28239	0.056922	-0.00282	0.352105	-0.90486	-0.02809	-0.36571	0.183706
Q9H7C9	AAMDC	0.825377	-0.24377	-0.04699	0.606199	0.606413	0.566725	-0.46005	-0.67266	-0.17077	-0.46325	-0.75096	-1.30961	-0.05295	-0.53212	0.271468	0.584539	-0.44988	-0.23169	0.257926
Q9H7D0	DOCK5	-0.28208	0.928872	0.678122	-0.47943	-0.17893	-0.21936	0.089865	-1.34423	-0.04367	0.135113	0.13371	0.68384	0.123011	1.271523	-0.78713	-0.02312	0.519322	-0.28359	-0.18067
Q9H7D7	WDR26	-0.32133	-0.01076	-0.12685	0.431988	-0.15406	-0.22344	-0.36247	0.281993	0.048016	0.151925	-0.04047	0.308212	0.452579	0.209399	0.219881	-0.07558	0.188693	-0.2662	-0.06641
Q9H7E2	TDRD3	-0.0384	-0.36686	0.023309	-0.55187	-0.85504	-1.15564	-0.45426	0.202722	-0.11075	0.04793	0.593768	-0.73505	-0.05255	-0.41117	0.062703	0.322108	-0.43477	0.232337	-0.20083
Q9H7E9	C8orf33	1.575831	-0.64183	-0.33192	-0.31567	-0.72293	-0.30048	-0.17687	-0.43239	0.133822	-0.40799	-0.12459	-0.39429	-0.07665	-0.93199	-0.26996	-0.49593	-0.58016	-1.10699	0.239514
Q9H7F0	ATP13A3	-0.34731	0.403348	-0.10227	-0.47357	0.129737	-0.32861	0.672536	-0.59923	0.015281	1.035699	-0.3889	0.609361	-0.20144	0.583161	1.262102	0.000478	0.593198	-0.12701	-0.08579
Q9H7H0	METTL17	-0.0154	-0.31253	0.049737	0.701525	-0.3274	-0.64648	0.168322	0.307451	-0.10733	-0.95645	0.435102	0.103852	-0.09212	-0.16405	0.533649	0.100705	0.34188	0.364667	0.112543
Q9H7L9	SUDS3	0.902344	-0.17248	-0.01066	0.114442	0.114837	0.395938	-0.29969	1.044389	-0.29068	-0.06442	0.391015	-0.40014	-0.32541	0.012651	0.420096	-0.2091	0.049082	0.067751	0.014568
Q9H7M9	VSIR	-1.24537	0.625283	0.66758	-0.73566	0.746326	1.105966	0.123735	-0.77018	-0.42192	1.609788	-0.6573	0.313354	-0.51511	1.137824	1.303632	-0.30781	0.684602	0.273103	-1.0962
Q9H7N4	SCAF1	0.565181	-0.11172	-0.05178	0.188349	-0.47509	0.244117	0.033284	-0.13371	-0.11247	-0.89277	0.321741	-0.33921	-0.12877	-0.59731	0.377676	-0.4851	-0.25671	0.58345	-0.44385
Q9H7P6	MVB12B	-0.66218	0.237628	-0.39555	-0.90869	0.370251	-0.77282	0.118181	-0.04197	0.3123	0.002205	-0.34154	0.358714	-0.2949	0.66878	-0.7622	-0.25541	0.022667	0.67027	0.170669
Q9H7P9	PLEKHG2	0.058629	-0.32157	-0.22297	0.396915	-0.03295	0.60176	0.021716	1.975935	0.180175	-0.1404	-1.16393	-0.26621	0.220462	-0.57852	-0.63139	0.40713	0.11999	0.309222	1.53129
Q9H7X7	IFT22	-0.0955	0.033442	0.334793	0.286402	-0.19357	-0.22967	0.004931	-0.12448	-0.35056	0.081137	0.845133	0.091608	-0.11317	0.19069	-0.52651	-0.0353	0.171163	-0.72887	-0.75575
Q9H7Z3	NRDE2	0.512241	-0.12851	0.126578	0.269656	0.095581	0.34576	-0.14293	0.18073	-0.26306	-0.56057	0.338756	-0.27392	-0.18892	-0.02709	0.038319	-0.10957	0.020951	0.361985	0.682628
Q9H7Z6	KAT8	0.419861	-0.33849	-0.28635	-0.15042	-0.01806	0.282928	-0.30927	0.166958	-0.18726	-0.37349	0.653993	-0.60934	-0.4905	-0.079	0.116161	-0.41217	0.2764	0.130778	-0.73865
Q9H7Z7	PTGES2	0.416942	0.237115	0.157879	0.36937	0.212353	-0.13065	0.075296	0.193088	0.139966	-0.06717	0.094084	-0.00032	-0.31613	-0.08706	0.720378	0.887665	0.717253	0.802383	-0.24795
Q9H814	PHAX	0.007239	-0.1879	0.117701	0.289134	0.613746	0.405221	-1.18999	0.152879	-0.37805	-0.3159	0.401432	-0.85921	0.052078	-1.1464	1.057769	0.455648	-0.14666	0.310523	-0.19961
Q9H832	UBE2Z	-0.53963	0.098984	-0.18647	0.086793	0.121082	0.290504	-0.32417	0.308215	-0.80187	-0.00314	0.125153	-0.44475	0.808289	-0.18087	-0.47202	0.343635	-0.55622	-0.51179	-0.21631
Q9H845	ACAD9	0.553569	-0.036	0.299136	1.246504	1.499495	1.238145	-0.52519	-0.32649	0.029252	1.520322	-0.16191	-0.26451	-0.6736	-0.01311	0.844868	0.339254	-0.63681	-0.80676	-0.44516
Q9H857	NT5DC2	0.851195	-0.10887	-1.04745	0.901423	-1.27303	-1.16643	-0.73449	-0.38833	-0.86509	-0.24779	-0.4131	-0.89652	0.185705	-0.09692	-1.05378	0.39998	-0.78131	-0.69565	0.912492
Q9H871	RMND5A	0.117868	-0.33222	0.2016	0.177774	0.166005	0.382737	0.259954	-0.27962	-0.22559	-0.12452	-1.61087	0.303216	-0.21736	0.024033	0.148613	0.127668	-0.16738	-2.1621	-0.02604
Q9H892	TTC12	0.630757	-0.11011	-0.36971	-0.12395	-0.33534	0.335519	0.063371	-0.94995	0.040847	-0.42623	1.92084	-0.25775	-0.10779	-0.02158	-0.77388	-0.70901	0.050296	-0.80166	0.254087
Q9H8E8	KAT14	0.405409	-0.15819	-0.08018	0.379981	-0.25399	0.170247	-0.21674	0.061962	-0.26483	-0.36773	0.2247	-0.40947	-0.37952	-0.42816	-0.82393	-0.44428	0.239055	-0.35407	0.058073
Q9H8G2	CAAP1	0.1165	-0.02462	0.329053	-0.10719	0.035314	-0.2125	0.0532	-0.01669	0.45771	-0.20149	0.644417	0.122168	-0.54902	-0.07673	-0.52392	-0.15401	-0.05849	0.586873	-0.16413
Q9H8H0	NOL11	0.430108	-0.02116	-0.55074	0.86183	-0.08449	-0.05304	-0.20906	0.341225	0.215806	-0.12618	0.774712	-0.54869	-0.36168	-0.80083	0.229797	0.496919	-0.50155	-0.58185	-0.26466
Q9H8H2	DDX31	0.416442	-1.0767	-0.23003	0.618969	-0.22007	0.080679	-0.2656	0.046722	-0.4116	-0.80739	0.499259	-0.29215	-0.49566	-0.19178	-0.21832	-0.60274	-0.40397	-0.36554	-0.18125
Q9H8H3	METTL7A	0.322139	0.222996	0.69301	-1.46073	1.676599	-0.03838	1.084483	-1.06622	0.182217	-0.29434	-0.96063	0.1732	0.064812	-0.01149	-0.23548	-0.18233	0.109487	0.786369	-0.3777
Q9H8L6	MMRN2	-0.8993	0.817386	0.233483	-1.1727	0.190834	-0.8091	0.232795	-1.07736	-0.24982	0.876884	-0.50574	-1.843	-1.07756	-0.53631	0.582256	-0.1945	-0.60918	0.426248	-1.04897
Q9H8M2	BRD9	0.55721	-0.03204	0.137804	0.200093	0.137188	0.033488	-0.08858	0.262727	0.010939	0.02312	-0.59593	-0.06036	0.123533	-0.31946	0.77373	0.234534	-0.22684	0.418115	-0.06441
Q9H8T0	AKTIP	-0.37823	-0.21068	-0.12726	-0.4293	0.249542	-0.25579	-0.02629	-0.35232	0.084678	-0.5067	0.071696	-0.09073	0.126006	-0.2362	-0.0874	0.512433	0.674906	-0.4243	0.060759
Q9H8V3	ECT2	0.736294	-0.32135	0.99691	-0.22265	1.860629	1.598415	0.427599	0.877915	0.206082	-0.2139	-0.64733	-0.28972	-0.15213	-0.72465	-0.1259	0.401563	0.392741	-0.6017	-0.14961
Q9H8Y5	ANKZF1	0.792041	-0.12888	-0.23693	-0.75468	-0.19846	0.262415	-0.01692	0.598365	0.185869	-0.57029	0.35894	0.218254	0.106403	0.195901	-0.63937	-0.26455	0.534344	-0.13961	-0.12221
Q9H8Y8	GORASP2	-0.03995	-0.45448	-0.28844	-0.31745	0.052597	-0.24801	0.393862	0.190905	0.566762	1.053144	-0.30074	0.045678	0.748946	-0.11947	0.743289	0.712198	0.08281	0.720804	0.640047
Q9H900	ZWILCH	1.058676	-0.2383	0.393408	-0.23533	1.238794	1.929936	0.577959	0.533686	0.093073	-0.89573	-1.12989	-0.33681	-0.17546	-0.26937	-0.46241	0.049449	0.594414	-0.86091	0.22681
Q9H910	JPT2	1.109875	-0.76665	1.04922	-0.48841	-0.74916	-0.76679	-0.72791	-0.78833	-0.04864	-0.43911	-0.0355	-0.58142	0.957742	-0.21837	-0.62773	0.46007	0.079454	1.006262	-0.28042
Q9H920	RNF121	-0.0694	0.124781	0.103122	0.190378	0.152428	0.519085	-0.06624	0.400422	-0.00901	0.130435	-0.71688	0.071093	0.307518	0.627674	0.621007	0.520186	0.329381	0.042442	0.567659
Q9H930	SP140L	-0.04051	0.320283	0.912016	-0.53258	0.611229	0.883336	0.126429	-0.46479	-0.22039	0.361064	0.012016	-0.03031	0.082672	0.227673	0.33427	0.62979	-0.23571	0.154882	-1.29329
Q9H936	SLC25A22	-0.21908	-0.1768	-0.15824	-0.1815	0.887447	0.951471	0.086671	0.229723	0.040847	0.321418	-0.2509	-0.31907	-0.67173	-0.40905	-0.80575	2.28822	0.893132	0.432679	-0.65742
Q9H939	PSTPIP2	-1.53122	0.77277	-0.49298	-1.1531	-0.11794	-0.7879	0.439398	-1.35695	0.618247	1.707325	-1.21065	1.371641	0.878155	1.334867	-1.80579	-0.33146	1.136291	0.648427	-0.7698
Q9H944	MED20	0.410352	-0.15834	0.114637	0.142853	0.535106	0.403241	-0.17421	1.327333	-0.18791	0.127362	0.182977	-0.09528	-0.28236	-0.15713	-0.34148	0.034699	0.789379	0.553815	0.230702
Q9H974	QTRT2	0.002605	-0.1836	-0.24712	0.013144	-0.25014	-0.3945	-0.20585	0.533882	-0.34042	-0.59422	0.327025	0.232144	-0.15258	0.214389	0.232092	-0.0024	0.006153	0.000383	-0.31893
Q9H993	ARMT1	0.66141	-0.18864	-0.42357	-0.02761	-0.26593	-0.11371	-0.68937	-0.54	-0.62515	0.18983	-0.4798	-0.49514	-0.54355	0.03542	0.178664	0.200207	0.292086	-0.1883	0.103631
Q9H9A5	CNOT10	0.422784	-0.10855	0.637014	0.153896	0.406471	0.190863	-0.06538	-0.04112	-0.0018	0.135072	0.595863	-0.12444	0.283493	-0.15045	0.366717	0.610231	0.205384	0.096904	0.231335
Q9H9A6	LRRC40	0.544029	0.392329	-0.07755	-0.19784	-0.1491	0.283989	0.405341	0.328649	0.424471	-0.08753	0.042139	-0.14477	-0.14674	0.194511	-0.06885	-0.2437	0.393931	-0.02803	0.364747
Q9H9B1	EHMT1	0.491103	-0.295	-0.24197	0.035798	-0.13931	-0.21958	0.88663	0.436574	0.071307	-0.40649	0.030723	-0.89939	0.179314	-0.42739	-0.52263	-0.02611	-0.12993	0.574476	0.275168
Q9H9B4	SFXN1	0.361091	0.440611	0.068166	1.070494	0.190792	0.385088	0.150743	-0.59834	0.282481	-0.24483	-0.08151	0.164511	0.43929	0.043923	0.236791	1.717666	0.469718	0.354103	0.154359
Q9H9C1	VIPAS39	-0.28065	0.04154	-0.15938	0.196906	-0.63214	-0.11614	0.124045	0.163731	0.00693	0.411281	0.288748	0.188779	0.549501	0.488164	-0.06716	0.058944	0.267505	-0.4258	0.178599
Q9H9E3	COG4	-0.55313	0.459028	0.358768	0.075865	0.549518	0.293701	0.365199	-0.22312	0.814576	0.435852	-0.02433	-0.00451	0.121587	-0.11719	1.031172	0.161367	-0.08395	0.1482	0.446146
Q9H9F9	ACTR5	-0.02484	0.286682	0.108036	0.071182	0.136708	0.406764	0.050433	0.028927	-0.2491	0.03698	0.250337	0.462994	0.324564	0.230628	0.380761	0.312305	0.312299	0.592495	-0.00952
Q9H9G7	AGO3	-0.0082	-0.09027	-0.62466	-0.3498	0.002941	0.056403	0.364597	0.063707	-0.47859	-0.52737	-0.27627	-0.45176	-0.07282	0.001997	0.612655	-0.12408	-0.10773	-0.56716	0.421335
Q9H9H4	VPS37B	-0.09262	-0.46558	0.530423	0.35569	-0.35037	-0.40355	0.436234	0.402163	0.729844	0.164357	0.509942	-0.17286	-0.52876	0.164504	-0.51689	-0.39986	0.225902	0.286718	0.055493
Q9H9J2	MRPL44	-0.35551	0.092453	0.042648	0.990392	0.259512	0.611522	-0.62456	-0.11863	2.483464	0.409696	0.797012	0.203773	-0.08446	-0.03504	0.491488	0.02836	0.09965	-0.08317	-0.25982
Q9H9L3	ISG20L2	0.500835	-0.16803	-0.27571	0.295474	-0.8549	0.216268	-0.60408	0.476775	-0.58086	-0.53188	-0.17103	-0.41934	-0.12458	-0.3016	0.47353	-0.58163	-0.14533	-1.27502	-0.52771
Q9H9P8	L2HGDH	0.8291	-0.12254	-0.12668	0.58141	-0.29413	-0.38622	-0.09124	-0.24502	-0.46796	-0.14164	0.978425	-0.04917	-0.29403	-0.02579	1.474452	1.890192	0.07336	-0.17416	-0.71865
Q9H9Q2	COPS7B	-0.00814	-0.27331	0.367997	-0.30621	0.028581	0.133496	-0.02732	0.089909	-0.14748	0.137582	0.384017	-1.18278	-0.11796	-0.73969	-0.22915	0.188832	-0.51396	-0.80153	-0.10167
Q9H9Q4	NHEJ1	0.064755	0.176494	0.254959	-0.59337	0.152599	0.057075	-0.10178	-0.49345	-0.16737	0.047341	0.03086	-0.20914	-0.75171	-0.20075	0.030181	0.585354	0.03045	0.397178	-0.39066
Q9H9S4	CAB39L	-0.78545	0.238722	-0.03864	-0.67877	0.003814	0.280313	0.00721	-0.23373	0.066732	8.08E-05	0.074911	-0.17913	-0.12755	-0.08023	0.215802	-0.06599	-0.88304	-0.73765	-0.24015
Q9H9T3	ELP3	0.227816	-0.25668	0.210517	-0.24873	-0.09041	0.026707	-0.01845	-0.08135	-0.17052	-0.26874	-0.10946	-0.03759	-0.41323	-0.13948	-0.65376	-0.27216	0.13696	-0.51233	0.177588
Q9H9Y2	RPF1	0.354053	-0.09735	0.049048	0.141693	-0.06953	0.073196	-0.28976	-0.18703	0.016267	-0.08797	0.519043	-0.14954	0.067815	-0.07518	0.079544	0.16503	0.261498	0.229144	0.113839
Q9H9Y4	GPN2	0.276668	0.067736	0.088587	-0.2428	-0.33952	-0.00709	0.118325	-0.36466	0.37518	-1.1095	-0.09167	0.040931	0.100748	0.049927	0.14983	-0.54931	0.550294	-0.48445	-0.17653
Q9H9Y6	POLR1B	0.51582	0.491551	-0.01408	0.272517	0.451097	0.755699	0.045128	0.704127	-0.33727	0.718425	0.032078	-0.08025	-0.66594	-0.45763	-0.15116	-0.1699	0.043324	-0.34439	-0.01003
Q9HA47	UCK1	0.419555	0.191204	0.118822	-0.22619	0.202694	0.265446	0.168764	-0.01613	0.262018	-0.18519	0.085716	0.365648	0.2774	0.460283	-0.36579	0.390469	0.080105	-0.00086	0.310855
Q9HA64	FN3KRP	0.83412	-0.63197	-0.00764	0.760768	-0.6483	-0.43321	-0.58091	-0.49806	-0.102	0.06501	0.281083	-0.95642	-0.64429	-0.19885	0.206084	0.237595	-0.53335	0.074498	0.076954
Q9HA65	TBC1D17	-0.71373	0.167003	0.540589	-0.44735	0.149594	-0.03876	1.311862	-0.18624	-0.19934	0.136703	-0.28391	-0.41102	-0.82955	-0.27217	0.736317	0.243264	-0.44234	-0.05636	-0.17857
Q9HA72	CALHM2	-0.09247	0.129677	-0.11725	-0.41899	0.283644	-0.36548	0.249919	-0.67454	-0.14074	-0.33459	-0.2548	0.217765	-0.57147	-0.17904	-0.59901	-0.92152	-1.39542	0.164705	-0.16902
Q9HA77	CARS2	-0.16371	0.101039	-0.12448	0.251628	0.034255	-0.01743	-0.94632	-0.41807	0.141166	0.632329	0.821306	-0.32476	-0.43885	-0.28029	0.154878	-1.00461	-0.36024	-0.36133	0.015429
Q9HAB8	PPCS	-0.98851	-0.5605	-0.14304	1.13955	0.372052	-0.50154	-0.3709	0.548262	0.807533	1.305765	0.061494	0.156517	0.716192	0.45764	0.035606	0.623936	-0.01447	0.11338	0.025133
Q9HAD4	WDR41	-0.4019	0.212595	0.274968	-0.30014	0.083584	0.114847	0.034141	0.050177	0.01983	-0.08406	0.131415	0.879317	0.050807	0.771974	-0.23424	0.28134	0.55929	-0.1905	0.353145
Q9HAF1	MEAF6	0.273861	-0.97265	0.018776	-0.16637	0.214732	0.60889	-0.0333	0.88328	-0.4554	-0.79066	0.855465	0.227287	-0.2972	-0.06133	-0.27295	-0.35041	0.023006	0.597737	-0.84693
Q9HAN9	NMNAT1	0.247882	0.073819	-0.25967	1.142663	0.649875	0.564947	-0.89951	0.460187	-0.281	0.257648	1.017348	-0.08984	-0.56386	-0.21832	0.813942	1.223721	-0.52171	-0.17313	-0.77428
Q9HAS0	C17orf75	-0.01254	-0.21885	0.007916	0.102458	-0.0796	0.250158	-0.42003	0.213817	0.052651	-0.20579	0.297983	-0.03838	-0.10976	-0.00496	-0.01239	0.537163	0.021513	-0.1249	-0.7631
Q9HAT2	SIAE	-0.24475	-0.31797	-0.91396	-0.52623	1.361045	-0.86476	-0.59305	-0.67428	0.106076	1.481687	0.356952	0.080713	-0.07304	0.002631	-0.50735	-0.61268	-0.65527	-0.40912	-1.08821
Q9HAU0	PLEKHA5	0.491737	-0.29842	0.557035	-1.0878	-0.97242	-1.07403	0.115424	0.201899	0.264918	-0.03202	0.072307	-0.85578	-0.37579	-0.6851	-0.2146	0.04348	-0.14721	0.36594	0.2189
Q9HAU4	SMURF2	-0.0345	0.21639	0.344774	-0.40953	-0.23843	-0.25815	0.129979	0.107109	0.129179	-0.47256	0.244073	-0.99625	0.41309	-0.06956	-1.14106	0.877428	-0.10874	-0.04185	0.601442
Q9HAU5	UPF2	0.352241	0.204491	0.353452	-0.17896	0.033037	0.339399	-0.55472	0.110628	0.01016	0.338387	0.149724	-0.5225	-0.3114	-0.13154	-0.21969	0.663118	-0.26473	-0.077	-0.15919
Q9HAV0	GNB4	-1.14621	0.558711	0.089481	-0.54206	0.056098	0.237183	0.128448	-0.60309	0.101752	-0.14365	-1.08198	-0.21989	-0.38884	0.192314	0.059808	0.232841	-0.67568	1.24231	-0.0561
Q9HAV4	XPO5	0.796373	-0.30883	-0.15059	-0.42079	-0.05985	0.402987	0.147321	0.933624	-0.63811	-0.45347	-0.22433	-0.09265	0.267399	-0.18072	0.176356	-0.4557	0.080273	-0.78704	0.227713
Q9HAV7	GRPEL1	1.374826	-0.41454	0.027604	-0.19333	0.749993	0.930537	-1.03703	-0.5993	-0.70181	0.924587	0.343542	0.037214	0.131422	-0.24149	-0.82516	1.699217	0.21279	-0.31866	-1.01812
Q9HB07	C12orf10	-0.06868	-1.05158	-0.79152	-0.33816	0.378696	0.170492	-0.041	1.234789	0.439446	0.645952	-0.30506	0.021295	-0.3633	-0.23606	-0.35838	-0.52645	0.022362	-0.23433	0.06441
Q9HB19	PLEKHA2	-0.8721	0.622624	0.511938	-0.67463	0.01299	-0.77614	0.21716	-0.27774	0.267961	0.658346	-0.41908	0.849926	0.069942	0.763136	0.307205	-0.71979	1.704635	0.401858	-0.55965
Q9HB21	PLEKHA1	-0.27171	-0.0696	0.066201	-0.71836	-0.59801	-0.82133	0.064304	0.203478	0.032671	0.71859	-0.49744	0.424124	-0.10798	0.667002	0.28889	-0.12613	1.561863	0.130606	-0.49705
Q9HB40	SCPEP1	-0.06174	0.424601	0.032272	-0.31511	0.848021	0.072634	0.421456	0.451152	-0.61952	-0.37703	-1.25008	1.3865	0.538338	-0.00258	-0.4003	-0.71769	0.30539	0.851057	-1.27338

Q9HB58	SP110	0.493951	-0.20984	1.280766	-0.8041	-0.18215	0.355398	-0.34709	0.115817	-1.06548	-0.13135	-1.08821	0.440689	-0.88366	-0.21849	0.136584	0.295541	-0.39097	0.062505	-0.82502
Q9HB71	CACYBP	0.698664	-0.88713	-0.79881	0.097804	0.846109	0.787483	-1.17058	-0.02621	-0.30375	0.473772	-0.66058	-0.16251	0.013164	-0.01122	-0.10668	0.373028	-0.00399	-0.70311	-0.86452
Q9HB90	RRAGC	-0.13156	0.038916	0.45175	0.19806	0.586739	0.282677	-0.15986	0.306698	0.171298	0.846992	-0.18209	0.878076	0.144399	1.199244	-0.22809	0.152762	0.560571	0.22429	0.353002
Q9HBE1	PATZ1	0.492262	0.156058	-0.189	0.519981	0.014183	-0.05502	-0.45082	0.192443	-0.35412	-0.66878	0.135011	-1.38424	-0.3695	-0.27405	0.244036	-0.33286	-0.1567	0.341341	-0.25372
Q9HBF4	ZFYVE1	-0.31032	0.522458	0.407197	-0.60737	0.116995	-0.06783	0.307197	-0.50216	-0.1845	0.146034	-0.59746	0.248497	0.305749	1.021609	0.210886	0.096069	0.366938	0.043458	0.342098
Q9HBG6	IFT122	0.176669	0.209184	-0.25665	-0.17948	-0.51295	-0.05645	0.430396	0.080664	-0.82262	-0.49735	1.536575	-0.74628	0.166316	-0.26026	-0.40812	-0.96946	-0.52135	-1.00577	0.01188
Q9HBH0	RHOF	-0.22975	0.369143	0.147737	-0.43782	-0.86022	-0.46272	0.021904	-0.63818	0.221177	-0.5429	-0.732	-0.12756	0.104315	1.048529	0.930687	-0.03426	0.013619	-0.8895	-0.25635
Q9HBH5	RDH14	0.081904	0.300787	0.318684	-0.72271	-0.2641	-0.46279	0.194183	-0.32567	-0.06519	-0.00925	0.170829	-0.09396	-0.29213	-0.04559	0.505153	0.950025	0.046887	-0.46679	-0.07428
Q9HBI0	PARVG	-1.60692	0.753919	1.062396	-0.76389	0.898435	0.440914	-0.34169	-0.39696	-0.22897	1.458996	-1.51512	1.184088	0.285723	2.143854	0.428683	-0.36128	0.594679	-0.28586	-1.12387
Q9HBI1	PARVB	-1.07149	0.554258	0.739354	0.287747	0.446368	-0.33209	0.223046	-0.41578	0.128169	0.732257	-0.60622	0.678106	0.024817	0.229575	-0.54393	-0.24471	-0.33824	1.049435	-0.2176
Q9HBI6	CYP4F11	0.214068	-0.62964	-0.83686	-0.25272	-0.74479	-0.52773	-0.1123	1.828576	-0.45436	-0.88609	1.360812	-0.70077	-1.06541	-0.01775	0.348086	-1.08759	0.334926	-1.69529	-2.24993
Q9HBK9	AS3MT	1.079341	0.146395	0.036781	-0.62668	-0.1721	0.043944	-0.23594	-0.08677	0.363355	0.525269	-0.89672	-1.01164	0.493917	-0.75787	-0.9628	-0.56408	-0.05763	0.373048	0.638181
Q9HBL0	TNS1	-0.11276	-0.57106	-0.65049	-1.37703	-0.82898	-1.29406	-0.50279	-0.99485	0.799149	-0.86861	-1.4128	-0.41826	-0.45498	-0.3018	0.317775	-0.67432	-0.86876	-0.583	-0.73407
Q9HBL7	PLGRKT	0.245623	0.327394	0.004978	0.349377	-0.30749	-0.33197	-0.24397	-0.05557	-0.62476	-1.1783	0.492338	-0.46662	-0.45816	-0.16783	0.636553	-0.59844	-1.04322	-0.32751	-0.25521
Q9HBL8	NMRAL1	-0.13936	0.227628	0.150717	-0.73511	0.359835	-0.06146	-0.02101	0.121317	0.542139	-0.06358	0.563965	-0.56038	-0.86663	-0.18953	-0.46307	-0.16044	0.368588	0.053739	-0.24954
Q9HBM0	VEZT	-0.40059	-0.03955	-0.57576	0.159112	-0.7567	-0.66888	-0.6166	0.151046	0.182278	-0.04894	0.855204	-0.45364	0.020202	-0.04868	-0.54748	-0.10093	0.052276	0.348807	0.509146
Q9HBM6	TAF9B	0.114218	-0.10504	-0.5158	-0.94217	-0.00287	-0.30987	0.557543	0.57077	-0.27814	0.21473	-0.02846	-0.24898	-0.147	-0.04313	-0.1128	-0.34083	-0.4335	-0.12948	-0.27188
Q9HBR0	SLC38A1(	-0.34153	0.86826	-0.17809	-0.31507	-0.11145	-0.24021	1.126674	-0.33297	0.292206	-0.1043	-0.31852	-0.35664	0.262656	-0.36837	-0.00915	0.380999	-0.18375	0.742633	0.810722
Q9HBU6	ETNK1	0.36267	0.092326	0.069691	-1.27863	0.141095	-0.41157	0.115233	-0.27578	-0.13225	-0.1735	-0.24361	0.142247	0.34711	0.550134	-0.02129	0.75695	1.031892	0.151082	-0.25855
Q9HC07	TMEM165	-0.39411	1.145611	1.035727	0.053703	0.992336	1.087133	0.436803	-0.31942	0.220953	0.060569	-0.49002	-0.05923	0.236855	0.183081	0.342174	1.214772	0.146846	0.837021	0.073353
Q9HC16	APOBEC3	-1.41269	0.763178	0.75338	-0.07125	0.758558	0.749344	-0.05301	-0.82846	-0.38057	-0.18385	0.279763	-0.16539	-0.50638	-0.32242	-0.9124	1.155546	0.793508	1.566976	-1.57424
Q9HC21	SLC25A1¢	0.660776	1.187887	0.858785	-0.10231	-0.17516	-0.53859	0.65577	-0.01997	0.040559	-0.10055	0.422633	1.866967	0.144428	1.380839	-0.07444	-0.19718	-0.1286	-0.61706	-1.34214
Q9HC35	EML4	-0.36789	-0.22899	0.15928	-0.07585	-0.50404	-0.47979	-0.99768	-0.13017	-0.01266	0.395163	-0.06955	-0.57636	-0.18674	-0.12759	0.883601	-0.25251	-0.61019	-0.12724	0.212838
Q9HC36	MRM3	1.460492	-0.21979	0.085332	-0.55441	0.709246	0.62302	-0.35489	-0.58866	-0.57434	0.104638	0.454896	-0.47166	0.008258	-0.77748	0.282257	0.233881	-1.05375	-0.56576	-0.30754
Q9HC38	GLOD4	0.04812	-0.42372	-1.42184	-1.26615	1.45062	-0.62792	0.035385	1.099526	0.748257	1.589747	-0.30358	-0.39686	0.786678	-0.36952	-0.47922	-0.87449	-0.70713	-0.23302	-0.63359
Q9HC52	CBX8	0.618306	-0.27612	-0.59591	0.447008	-0.36812	0.130107	0.725461	0.958283	-0.40262	-0.91017	0.627011	-0.75242	-0.41248	-0.81237	-0.54379	-0.58249	-0.13352	0.575567	0.152707
Q9HC78	ZBTB20	-0.2285	-0.50719	-0.56827	-1.0566	0.136259	-0.6991	0.216107	-0.89874	-0.15781	-0.56128	0.549052	-0.94603	-0.77299	-1.62742	0.479366	-0.74166	-1.17248	-0.42666	-0.66
Q9HC84	MUC5B	-1.51949	-1.17865	-0.72596	-2.60626	-2.8854	-2.27515	0.069799	1.314015	-0.76615	-0.88739	2.056446	-1.303	-1.0616	-1.50923	-2.58655	-2.20478	-1.48149	-3.06546	-2.02556
Q9HCB6	SPON1	-1.48598	-1.57663	-3.23044	-1.30087	2.106073	1.72032	-2.02538	-0.66855	-0.1878	-2.66073	1.727225	-1.12271	-0.2801	-1.06107	1.283771	-1.81525	-1.36875	-2.35221	-0.92098
Q9HCC0	MCCC2	0.18459	-0.85562	-0.49212	2.663221	-0.26425	-0.08735	-1.0452	0.023836	-0.06612	-0.27597	0.975837	-0.24335	-0.36129	-0.12306	2.359232	1.695186	-0.5389	-0.52697	-0.31101
Q9HCD5	NCOA5	0.093156	-0.4313	-0.24458	0.077993	-0.27955	-0.0466	-0.29456	0.716057	-1.31379	-0.51784	0.408383	-1.12814	-0.43482	-0.79587	0.358837	-0.51565	-0.01569	0.44911	0.09727
Q9HCD6	TANC2	-0.08761	0.311398	0.335426	-0.60775	0.023608	-0.29541	0.121991	-0.1187	0.124493	-0.05349	-0.34889	0.057042	0.112857	0.093554	0.043871	0.191642	0.307266	0.579211	0.484899
Q9HCE0	EPG5	-0.25237	0.462057	0.385544	-0.22732	0.103954	-0.15725	0.206152	-0.79568	0.190363	1.083016	-0.68326	0.379259	0.813062	0.01848	-0.50379	0.244207	-0.42739	-0.23008	0.209066
Q9HCE1	MOV10	0.944188	0.001062	-0.35776	0.42343	0.808495	1.172087	-0.0243	0.046874	-0.07138	-0.11871	0.140469	-0.44169	0.100949	-0.3363	-0.96589	0.560384	-0.07761	0.153043	-0.19519
Q9HCE3	ZNF532	0.506083	-0.1436	-0.53349	-0.37943	-0.61961	-0.51333	-0.36859	-0.37838	-0.15812	-0.33196	0.573441	-1.80927	0.132714	-0.35706	-1.19396	-0.91218	-0.60071	-0.0899	0.985698
Q9HCE5	METTL14	0.096039	-0.14018	-0.70783	0.040441	-0.03917	-0.20506	-0.97995	0.510824	-0.10972	-0.10411	0.885635	-0.36493	-0.04412	-0.27503	0.466365	0.119081	-0.40832	0.036923	0.589225
Q9HCE6	ARHGEF1	-1.23826	0.907798	0.855029	-0.62415	0.180739	-0.32244	0.117939	-0.84235	-0.01205	0.845289	-0.31836	1.719588	0.257974	1.406749	0.166869	-0.62821	0.431297	-1.19899	-0.8831
Q9HCG7	GBA2	0.082696	0.218838	0.300197	-0.03249	-0.0249	0.237484	0.185119	-0.20597	-0.01195	0.42717	0.258385	0.069869	0.008942	0.44681	0.04897	0.307143	0.100721	-0.48287	0.08099
Q9HCG8	CWC22	0.962069	0.620574	-0.39373	0.226008	-0.0286	0.088644	-0.31026	0.232947	-0.20542	-0.15377	0.158199	-0.93608	-0.05941	-0.53732	-0.02635	-0.11929	0.043633	0.025542	0.119315
Q9HCJ6	VAT1L	-0.59393	0.689174	-0.01583	-0.65024	0.20666	-0.20418	-0.04036	0.174193	0.977925	-0.28917	-1.39723	-0.89736	0.624767	-0.43561	-0.44491	0.634483	-0.64463	-0.95268	3.599555
Q9HCK8	CHD8	0.549511	-0.10826	0.022214	-0.0338	-0.37611	-0.3288	-0.08973	0.079893	-0.05586	-0.11137	0.149419	-0.20735	-0.0203	-0.49062	0.066927	0.085529	-0.05927	-0.03269	0.498973
Q9HCL2	GPAM	-0.58834	-0.13519	-0.77606	-0.60065	0.077215	-0.91597	0.006629	-0.22823	0.314498	-0.45089	-0.55756	0.487949	-0.00972	0.347824	-0.10902	-0.00954	0.211828	0.409607	-1.00355
Q9HCM4	EPB41L5	0.452818	-0.56064	-0.75856	-0.86876	-0.76493	-0.81735	0.070811	0.204351	-0.00296	-1.10786	0.31409	-0.00677	-0.20739	-0.09242	0.703589	1.100708	1.684781	1.295832	0.511811
Q9HCN4	GPN1	0.330392	-0.38511	0.04263	-0.37186	-0.03674	0.367851	0.073127	-0.15581	0.252017	-0.82357	-0.76828	-0.34821	-0.01859	-0.19405	-0.53962	-0.10292	0.065133	-0.31791	0.558577
Q9HCP0	CSNK1G1	-0.042	0.214188	0.279697	0.233508	0.272555	0.471404	0.056235	0.021382	-0.16489	0.12202	-0.23655	0.477995	0.134578	0.387816	0.167873	0.372869	0.240178	0.373046	0.006558
Q9HCS7	XAB2	0.285798	0.095418	-0.15491	0.111368	0.128742	0.202992	-0.3339	0.339921	-0.09104	0.034455	0.286345	-0.06652	-0.08246	-0.24299	-0.23593	0.386539	-0.09753	0.047008	-0.17104
Q9HCU0	CD248	-0.48602	0.434884	0.544683	-1.78179	-0.21444	-0.89335	0.879715	-0.39643	0.422185	0.242633	-1.2338	-1.74783	-0.10422	-0.70606	-1.17437	-0.36796	-0.41906	0.623333	0.867544
Q9HCU4	CELSR2	0.236224	-0.57865	-0.53596	0.181124	-0.48607	-0.13783	-0.26767	0.258679	-0.49035	-0.78705	0.131119	-0.47671	-0.47997	-0.3725	0.620553	-0.9853	-1.29354	-1.15352	-1.85185
Q9HCU5	PREB	-0.53452	0.624856	0.500239	0.483767	-0.4109	-0.42183	0.758617	-0.34051	-0.6322	-0.34499	-0.40594	0.316328	0.202754	0.229787	0.485652	0.253118	-0.14039	-0.14485	-0.29753
Q9HCU9	BRMS1	-0.19946	-0.01772	0.226673	-0.39812	0.473132	0.82403	0.218703	0.410458	0.020282	-0.0331	0.036424	-0.26451	-0.14464	0.131458	0.619315	-0.38219	-0.03833	-0.29918	-0.26515
Q9HCY8	S100A14	1.196844	-1.11254	0.02966	2.467471	2.343169	2.14364	-0.75788	-2.05558	-0.06436	0.134109	-0.1298	0.509486	1.469337	-0.0772	-0.42078	1.31228	-0.50495	-2.50954	-1.923
Q9HD20	ATP13A1	-0.10889	0.958716	0.812748	0.079024	-0.05743	-0.62696	0.974746	-1.01431	-0.02493	-0.34516	-0.2273	0.583293	-0.30066	0.433004	-0.98446	0.24365	0.238233	0.114151	-0.19046
Q9HD23	MRS2	-0.33089	0.151477	0.176199	0.484766	-0.57006	-0.40709	-0.16595	-0.8085	0.180944	0.787295	-0.59357	0.223426	-0.01037	0.52086	-0.0351	-0.03525	-0.54807	-0.49303	-0.7503
Q9HD26	GOPC	-0.69363	0.051672	0.736157	0.48416	-0.12389	0.185318	-0.89778	-0.29729	0.306765	-0.0395	-0.01925	-0.73147	0.418336	-0.33746	1.013251	0.164408	0.357859	0.263722	0.608881
Q9HD33	MRPL47	-0.5179	0.129485	-0.04016	0.618326	0.361816	0.606706	-0.18945	-0.40676	-0.23563	0.279696	0.337593	-0.22766	-0.25289	-0.01888	1.263133	-0.17599	-0.09707	-0.1756	-0.29923
Q9HD40	SEPSECS	-0.03987	0.396476	-0.67313	-0.98931	-0.21235	-0.1594	-0.47599	-0.45187	0.171002	-0.18012	0.731648	-0.56943	-0.08917	-0.1538	-0.1263	0.401222	0.298951	-0.13856	-0.57339
Q9HD42	CHMP1A	-0.69761	0.31022	0.216466	-0.95235	0.480576	0.67257	-0.78253	-0.23873	0.288616	-0.33524	-0.1285	-0.08453	-0.30098	0.548734	-0.36176	0.106947	0.259863	-0.06591	-0.09693
Q9HD45	TM9SF3	-0.30857	0.583687	0.442943	0.440707	-0.01101	0.285053	0.06607	-0.24265	-0.62242	0.055995	-0.32056	0.276953	-0.05923	-0.1077	0.948487	0.130325	0.818165	0.574418	0.511734
Q9HD67	MYO10	-0.82038	0.121405	-0.03738	-0.37136	0.042168	-0.26103	0.141837	0.322584	-0.53363	-0.27807	-0.18166	-0.1699	-0.09871	-0.48058	0.591318	0.80773	-0.10347	-1.18472	0.159637
Q9HDC9	APMAP	-0.35834	0.56085	0.225505	-0.38754	-0.61489	-0.80111	0.654923	0.539698	-0.1252	0.687539	-0.05432	0.291398	-0.32387	1.242842	0.688908	-0.50752	0.289204	0.088112	0.150687
Q9NNW5	WDR6	-0.39584	0.458791	0.240965	-0.42263	0.286513	-0.0746	0.155174	0.238249	0.035462	0.201915	-0.15595	0.171651	0.221897	0.456335	-0.23003	0.413291	0.502729	0.142928	0.234729
Q9NNW7	TXNRD2	1.083598	-1.23418	0.911185	0.644977	0.220838	-0.1143	-1.15212	0.061002	0.815252	0.517823	0.105381	-0.11498	-0.70894	0.297	0.878601	1.156143	0.350158	1.018984	-0.61395
Q9NNX1	TUFT1	0.703435	-0.02123	-0.08465	0.433199	-0.14659	0.263488	-0.23596	0.551615	-0.33908	-0.2816	-0.68268	0.082485	-0.11608	-0.31128	-0.02087	0.195878	1.105201	0.110626	-0.65686
Q9NNX6	CD209	-1.75763	-0.0782	0.750619	-1.77389	-1.65627	-2.45221	2.26814	-2.10449	0.083507	-2.11616	0.238511	-1.33351	-0.24898	-0.62194	-0.28322	-0.04252	-0.43824	-0.44615	-1.66168
Q9NP58	ABCB6	0.352493	0.36177	-0.5181	-0.50008	-0.1993	-0.4327	0.442491	0.183357	1.643862	-0.76441	0.424732	-0.02452	0.130336	-0.50651	-0.82458	-0.61264	0.465319	0.258154	-0.17881
Q9NP61	ARFGAP3	-0.8005	0.086191	0.259515	-0.85558	-0.73666	-0.60784	0.020865	-0.26412	0.793574	0.353887	-0.21402	0.052238	0.935938	0.175888	0.004271	1.059567	0.679179	1.277211	1.012901
Q9NP64	ZCCHC17	1.235343	-0.19466	0.042123	0.633844	0.23281	0.122855	-0.2299	0.225798	-0.11356	0.476642	0.465831	-0.43229	-0.13162	-0.40895	0.240537	-0.39295	0.17337	-0.33434	-0.51574
Q9NP66	HMG20A	0.256822	-0.29024	-0.13712	-0.05643	0.067665	0.403519	-0.23304	0.031185	-0.11881	-0.62019	0.691669	-0.67092	-0.15021	-0.84087	0.391239	0.043183	-0.62359	0.266926	-0.46448
Q9NP72	RAB18	-0.531	0.040069	0.021698	-0.12673	0.392207	0.310291	0.449082	-0.20965	0.18425	0.205893	-0.26177	0.690352	-0.04022	0.861033	0.022864	0.874559	0.404096	-0.0328	0.167609
Q9NP74	PALMD	0.930106	-0.90582	-1.05393	0.811149	0.234325	0.449903	-0.53682	-1.32294	-0.20829	-0.92684	0.560492	-1.78856	-0.22104	-1.52728	-0.4273	1.065289	-1.91163	-0.31471	-1.03977
Q9NP77	SSU72	0.288351	-0.32577	0.063699	-0.11778	0.868472	0.728601	-0.2545	0.627077	-0.15755	0.769618	0.50523	-0.31709	0.050213	-0.05433	0.189978	0.794822	0.2276	0.346593	-0.34754
Q9NP79	VTA1	0.019595	-1.28613	0.82268	0.687562	-0.55057	0.177781	-0.65311	-0.68123	-0.52519	0.486809	0.043915	-0.38166	-0.21426	-0.08782	0.12007	0.127815	0.370308	-0.28784	-0.10789
Q9NP80	PNPLA8	-0.38426	0.121731	0.174819	-0.14312	-0.17341	0.068881	0.028365	0.081514	-0.02364	0.169062	0.074849	0.155658	-0.04124	0.314605	0.822676	0.610755	-0.42209	-0.22027	0.098651
Q9NP81	SARS2	0.636253	-0.43298	0.779363	-0.01451	-0.45394	0.241218	-0.05075	2.947635	1.016671	-0.55239	0.287129	0.005196	0.42538	-0.17334	0.782546	-0.02035	-0.19213	-0.4784	3.022635
Q9NP92	MRPS30	-0.41264	0.324997	-0.03982	0.352612	0.06864	-0.00378	0.013196	-0.38387	0.348203	0.714311	-0.03917	0.473645	0.076174	0.282525	0.586985	-0.25178	-0.08036	-0.47933	-0.91919
Q9NPA0	EMC7	0.131129	0.386526	0.873221	0.070824	0.055006	0.224983	0.435377	-0.42505	-0.30181	-0.16975	-0.23675	0.158827	-0.1771	0.11842	0.286894	0.510023	0.220986	0.034395	-0.14196
Q9NPA8	ENY2	0.526011	0.201873	-0.25579	0.311049	0.112694	0.767998	-0.56581	0.052994	-0.14616	-0.9545	0.077485	-0.24833	0.205577	-0.65269	0.42144	-0.47278	0.193523	-0.30557	0.139043
Q9NPB8	GPCPD1	0.692147	0.034614	0.053162	-0.60689	-0.34406	-0.67623	-0.02374	0.10246	-0.10611	0.358235	0.111145	0.666299	-0.33951	0.905587	-0.72393	-1.01078	0.226697	-0.65706	-0.59051
Q9NPD3	EXOSC4	0.553045	0.513429	0.43603	-0.05881	0.590473	0.844131	0.391671	0.1225	0.767069	-0.03362	0.207083	0.379047	0.227762	-0.25788	-0.3686	0.667542	0.093594	0.538214	-0.23709
Q9NPD8	UBE2T	1.30588	-0.43236	0.044485	-0.43675	0.706132	1.380607	0.221661	0.639225	-0.68475	-1.90499	-1.07213	-1.34378	-0.47655	-0.6022	-0.04913	-0.65525	-1.60702	-1.23429	-1.14351
Q9NPF4	OSGEP	0.538801	0.125988	-0.52265	0.038732	1.090932	0.813142	-0.92185	0.201949	-0.51385	0.061295	0.400904	-0.35355	0.590929	-0.18677	0.266862	-0.65906	0.353647	-0.02473	-0.03033
Q9NPF5	DMAP1	0.388657	-0.06606	-0.02144	-0.17428	0.240136	0.653377	0.060471	0.67948	-0.25126	0.234929	-0.06009	-0.33999	0.108714	-0.19749	0.241059	0.292423	-0.02892	-0.03745	-0.15204
Q9NPF8	ADAP2	0.208055	0.073711	0.561578	-0.19875	0.743496	0.280823	0.116785	-0.72452	0.252071	0.100516	-0.13175	0.824808	-0.49652	0.571726	0.313253	-0.42263	-0.64413	0.698325	-1.02963
Q9NPG1	FZD3	0.160992	0.394667	-0.17369	-0.61146	-0.53312	-0.19946	-0.80362	-0.18594	0.040847	-0.10924	0.857432	-0.15879	0.050408	0.080081	-0.01815	-0.02298	-0.16942	0.490839	-0.25012
Q9NPH0	ACP6	0.123457	-0.08295	-0.05963	0.244965	0.685281	-0.27706	-0.31242	0.641116	-0.42428	-0.01581	0.376278	-0.1835	-0.22525	-0.38379	0.222839	1.987029	-0.01526	-0.44356	-0.90234
Q9NPH2	ISYNA1	0.621858	-2.94324	-0.27381	-0.79136	-0.97208	-1.5381	-1.17849	-0.08989	-0.72516	-1.02022	-0.62269	-1.20097	-2.14413	-1.63659	-1.78598	0.509911	-0.34732	-1.01132	-0.2486
Q9NPH3	IL1RAP	0.486974	0.587153	-0.00828	-0.74175	-0.32005	-0.51162	-0.06964	-0.65361	0.294024	-0.50073	0.489771	0.192445	1.142893	0.083234	-0.8661	-0.02682	-0.52009	-0.08459	0.340744
Q9NPI1	BRD7	0.497441	-0.00871	-0.25751	0.089588	-0.00964	-0.06713	0.210797	-0.05988	-0.34987	0.130569	-0.44934	-0.42913	-0.38978	-1.01928	0.211448	-0.71502	-0.28626	0.030835	-0.75
Q9NPI6	DCP1A	0.428791	0.011368	-0.37651	-0.40014	0.010877	0.01307	-0.13751	0.102746	0.318612	-0.63463	0.084628	0.273774	-0.04168	0.245356	-0.64085	-0.67971	0.751031	0.14333	0.111468
Q9NPJ3	ACOT13	-0.05889	-0.30268	-0.2336	0.276394	1.158608	0.077306	-0.32459	-0.49608	0.015902	-0.48037	-0.18207	-0.29159	-0.27832	-0.0944	-0.24545	0.27874	-0.99188	-0.53646	-1.02676
Q9NPJ6	MED4	0.127513	0.30277	0.019336	-0.18337	0.042229	0.21048	-0.03573	0.25853	-0.15852	-0.08536	0.263402	-0.24926	-0.03801	-0.23271	0.43831	0.114984	0.194668	-0.07039	0.128497
Q9NPL8	TIMMDC1	0.10994	0.325453	0.675267	0.877448	0.09082	0.178606	0.544097	-0.05561	0.590899	0.384563	-0.54404	-0.27998	0.172955	-0.33994	1.022632	-0.0198	-0.81313	-0.31663	-0.73999
Q9NPP4	NLRC4	-1.25922	1.290534	1.027709	-0.90884	0.109197	-0.67911	0.09974	-0.98986	-0.11863	1.195384	-1.74352	1.640526	0.110603	2.519305	-0.51613	-0.81089	1.022358	-0.68779	-1.25281
Q9NPQ8	RIC8A	-0.55156	0.500298	-0.26795	-0.69873	0.552405	0.782222	0.481272	0.507236	0.682872	-0.1029	-0.28827	-0.10867	-0.08079	0.160849	-0.05598	0.422288	0.215322	0.042466	0.213079
Q9NPR2	SEMA4B	1.192371	-0.61001	-0.12637	0.034212	0.438306	0.357196	-0.03797	0.047162	0.027616	-0.14002	-0.75776	-0.11637	0.819568	-0.2003	0.617965	0.056581	0.533945	-0.86931	-0.67919
Q9NPY3	CD93	-1.03435	0.304478	-0.01359	-0.40915	0.321137	-0.33689	0.339826	-0.65665	0.162922	-0.0749	0.110836	-0.52521	-0.07907	0.597854	0.354508	-0.44207	-0.88007	1.129647	-1.39821
Q9NQ29	LUC7L	0.134666	0.131398	0.014135	-1.01292	0.146422	0.297017	-0.1485	-0.23603	0.420437	-0.01022	0.55417	-0.87341	0.284033	-0.14627	-2.15904	-0.07018	0.030855	0.767203	0.867756
Q9NQ48	LZTFL1	0.33669	-0.34283	-1.01327	-0.27322	-0.10841	0.351484	-0.3752	-0.13954	-0.0168	-0.69115	1.048478	-1.06342	0.488496	-1.06663	-0.24045	-0.45367	-1.38034	-0.88034	-0.00607
Q9NQ50	MRPL40	-0.31467	0.288431	0.891795	1.222835	0.115855	0.394937	-0.44999	-0.38104	0.013119	-0.12752	0.550332	-0.32645	0.237245	-0.39161	0.710251	0.103671	-0.5374	0.197235	-0.73389
Q9NQ55	PPAN	0.499802	-0.2129	-0.226	0.610512	-0.3048	-0.06186	-0.30743	0.683365	0.159592	0.100061	0.046051	-0.48069	-0.52538	-0.89165	-0.2269	0.737127	0.073894	0.047505	-0.46173
Q9NQ66	PLCB1	1.112327	0.020137	-0.41097	-0.96115	-0.45368	-0.76915	-0.18146	-0.57215	0.135082	0.508869	0.433691	-0.57534	-0.08704	-0.81227	0.82981	-1.14781	-0.36323	-0.19346	-0.19646
Q9NQ79	CRTAC1	-0.50831	-0.50356	-0.54569	-1.12889	-0.78927	-1.69846	-0.26875	0.686739	-0.35925	-2.05838	-1.5528	-0.09764	-0.68388	-0.05177	0.405242	-1.673	-0.48179	-2.43543	-1.31669
Q9NQ84	GPRC5C	-0.36373	-0.4027	-0.91819	0.82936	-1.56322	-0.76983	-0.43317	-0.35111	-0.43686	-2.34682	0.086736	-1.34073	-0.32931	-0.97321	1.457785	-2.18585	0.023645	-1.46193	-0.87107
Q9NQ89	C12orf4	-0.25155	0.365271	-0.19969	-0.23092	-0.20666	-0.44818	0.206164	0.221663	-0.15153	0.142681	0.080995	-0.00671	-0.05698	0.436049	0.373892	0.058103	-0.46201	-0.39733	0.239944
Q9NQC1	JADE2	-0.05889	-0.53949	0.742542	-0.1595	-0.37457	-0.00282	-0.34088	-0.0822	0.538715	-0.56569	-0.48335	-0.07216	0.025222	0.054702	-0.30802	0.324608	0.089576	0.206149	-0.33654
Q9NQC3	RTN4	-0.74912	1.109413	0.481111	-1.03502	-0.47206	-0.67278	0.480206	0.072057	-0.0683	-0.04733	-0.96839	0.377439	-0.07963	0.300754	-0.67643	0.013552	0.005902	-0.15236	0.978626
Q9NQC7	CYLD	-0.1281	0.732117	0.497209	-0.28804	0.107126	-0.24866	0.465667	-0.88619	-0.18614	0.782622	-0.07866	-0.01154	-0.77839	0.294328	-0.25236	0.079407	-0.22332	1.038141	-0.45744
Q9NQC8	IFT46	0.214489	0.072304	-0.38636	0.07862	-0.50717	-0.25164	0.156229	0.256688	0.040847	-0.55885	1.329862	-0.33995	0.499218	-0.36494	-0.0111	-0.36531	-0.77631	-0.87701	-0.2697
Q9NQG5	RPRD1B	0.457311	-0.06651	-0.47247	0.043716	-0.06955	0.368229	-0.10482	1.266558	-0.50908	-0.04952	0.059829	-0.46412	0.320818	-0.67555	0.452312	-0.5101	-0.20026	-0.38045	0.471216
Q9NQG6	MIEF1	-0.7671	0.364642	0.139961	-0.73111	0.371929	-0.01285	0.752878	-0.83996	0.017072	-0.77801	0.486271	-0.25102	-0.0222	0.274676	0.000595	0.806152	-0.13472	0.277295	0.398339
Q9NQH7	XPNPEP3	-0.10353	-0.17772	-0.79274	0.821933	0.848645	1.136214	-0.26505	-0.45688	0.294908	-0.24186	0.553113	-0.37332	-0.04509	-0.59522	-0.282	0.2924	0.320294	-0.46985	-0.0152
Q9NQP4	PFDN4	0.210129	-0.22349	0.319693	0.090663	0.865694	0.767224	-0.04054	0.671551	0.119401	0.120247	-0.02316	-0.26441	0.298199	-0.04856	0.033606	0.919719	-0.12654	-0.04501	-0.09942
Q9NQR4	NIT2	0.038914	-1.14328	-0.66451	0.039106	1.978073	-0.54355	-0.17398	0.078932	1.856707	2.070903	-0.07593	0.285067	0.671269	0.649931	0.715224	-0.22206	0.242209	-0.30306	-0.14009
Q9NQS1	AVEN	0.392561	0.130159	-0.12325	-0.09356	-0.13376	-0.29584	-0.24979	-0.24532	-0.11069	-0.23777	-0.40247	-0.17264	0.269234	-0.88336	-0.14074	0.967873	0.012751	-0.06571	0.053712
Q9NQS3	NECTIN3	1.010177	-0.07958	-0.54999	-0.32064	-1.53744	-1.09135	0.278872	1.018739	-0.27835	-0.68786	-0.09979	-0.5906	0.161793	-0.4011	0.543109	-1.75012	-0.43838	0.387946	0.21815
Q9NQS7	INCENP	1.176997	-0.55805	0.143106	0.289998	1.435456	1.398616	0.336005	0.607573	0.068727	-0.17221	-1.93668	-1.11158	-0.14019	-0.40964	-0.82612	-0.06368	0.242592	-0.38678	0.170506
Q9NQT8	KIF13B	-0.2996	0.235817	0.09824	-0.78725	-0.42659	-0.43893	-0.16264	-0.70801	0.111543	-0.10061	0.0054	0.049329	-0.2925	0.145305	-0.60331	-0.2223	0.52732	0.225271	-0.585
Q9NQW6	ANLN	1.507526	-0.55362	1.323201	-0.26947	0.356695	1.184834	0.666191	1.360143	-0.00965	-0.12819	-1.10706	-0.31085	0.39993	-0.1398	-0.07635	0.08277	0.787928	-0.29752	-0.14205
Q9NQW7	XPNPEP1	-0.62957	0.034187	0.067717	0.009563	0.492385	0.279868	-0.33492	0.660803	0.372109	0.943838	0.110198	0.141247	0.603672	0.316861	-0.54358	-0.14621	0.326111	0.609161	0.558234
Q9NQX3	GPHN	0.906671	-0.25094	-0.28832	-0.25484	-0.87583	-0.77294	-0.72915	-0.60921	0.269107	-0.42135	0.42683	-0.94771	0.073897	-0.59393	0.118397	-0.42808	0.517897	0.315216	0.178934
Q9NQX4	MYO5C	0.407598	-0.18538	-0.98042	1.061553	-1.54504	-1.86006	-0.29677	-0.99268	-0.40433	-1.04048	0.638423	-0.55088	0.191493	-0.52184	-0.39934	0.352686	-0.85659	-1.58642	-1.86632
Q9NQX7	ITM2C	-0.28579	0.367256	-0.12411	-1.56949	-0.17362	0.112301	0.233208	0.006078	0.040847	-1.16317	0.061115	-0.40949	-0.17868	-0.18988	-1.39306	-2.22197	0.542673	-0.13925	0.206142
Q9NQY0	BIN3	-0.16442	-0.01254	0.029859	-0.64011	0.420322	-0.03584	0.062853	-0.12005	-0.05743	0.121072	0.486242	-0.17875	-0.5824	-0.09049	-0.79911	0.407706	-0.10388	0.225246	0.277503

Q9NQZ2	UTP3	0.600905	-0.32134	-0.46948	0.387595	-0.58274	-0.11419	-0.44186	0.552677	-0.13278	0.27135	0.761204	-0.256	0.132823	-0.87246	0.128216	0.161002	-0.1854	-0.62006	-0.72054
Q9NQZ5	STARD7	-0.12102	0.111899	-0.47198	0.364985	0.853303	-0.14867	-0.31618	0.087408	-0.16521	-0.30746	-0.36208	-0.30443	0.104608	-0.65779	-0.29693	-0.13696	0.057142	0.39065	-0.45997
Q9NR09	BIRC6	0.087627	-0.02417	-0.10652	0.073963	-0.1335	-0.00971	0.206161	0.141537	-0.52685	0.167546	-0.26031	-0.15111	0.027702	-0.03856	0.254991	0.704411	-0.40616	-0.61676	-0.15875
Q9NR12	PDLIM7	-0.87674	-0.76754	-0.75469	-1.10013	-1.61484	-1.1801	-1.03701	-0.85488	0.562177	-0.99382	-0.90436	-0.63387	1.112031	-0.54083	-0.68612	-0.42274	-0.51028	-0.0375	1.292156
Q9NR19	ACSS2	0.414485	-0.2439	-0.23979	0.205572	0.444674	0.517971	-0.0288	-0.69323	-0.20143	0.358144	-0.68974	0.268355	-0.29534	0.821189	0.006781	-0.83578	0.522657	-0.09047	-0.2047
Q9NR28	DIABLO	0.257549	-0.63858	0.338739	1.064653	-0.03288	0.029964	-0.51046	0.733692	0.706241	0.42631	0.396249	-0.08205	-0.18562	0.094202	0.210033	0.009123	-0.31222	0.098742	0.95169
Q9NR30	DDX21	1.041388	-0.20359	0.19604	0.257954	0.447172	0.757834	-0.49988	0.312664	-0.35968	-0.30278	0.282431	-0.08943	0.191101	-0.43371	0.184633	-0.18523	0.4246	-0.22847	-0.52364
Q9NR31	SAR1A	0.136356	-0.50769	0.456116	0.436996	1.317775	0.800163	0.504303	0.175647	0.234075	0.715802	-0.22188	0.769838	0.520766	0.64177	0.329606	0.768812	0.945908	0.572871	0.627731
Q9NR45	NANS	-0.82256	-0.5479	-0.16459	0.414392	1.12638	0.310641	-0.21654	0.086789	0.33064	0.48379	0.13024	0.437189	0.5927	0.288688	-0.13025	0.941227	0.223653	0.491584	0.388163
Q9NR46	SH3GLB2	0.088845	0.153085	0.079222	-0.87947	-0.0521	0.461987	0.173548	0.487244	0.216404	-0.44378	0.158004	-0.35638	0.114146	-0.19919	-0.00978	-0.17248	-0.34687	-0.34539	-0.61691
Q9NR50	EIF2B3	-0.31581	-0.01822	0.653315	0.188695	0.174705	0.409364	0.446304	0.016493	-0.0725	-0.25601	0.02549	0.238948	0.038094	-0.12256	0.088409	0.68235	-0.19379	-0.21424	-0.03828
Q9NR81	ARHGEF3	0.147666	-0.42363	0.286427	-0.00354	0.869792	0.596903	-0.00575	0.714822	-0.06999	0.112431	-0.13854	-0.64948	-0.07057	-0.25906	0.555145	0.015586	0.189147	0.100462	-0.64045
Q9NR99	MXRA5	-1.42464	1.190313	-0.03924	-1.6883	-0.70173	0.747709	-0.00674	-0.07179	1.481236	-0.53494	-2.40409	-0.93015	0.742349	-0.81027	-1.16795	1.13838	-0.94874	-1.22544	1.757696
Q9NRA2	SLC17A5	-0.46214	0.309419	0.026989	-0.56946	-0.80566	-1.22609	0.075737	-1.04935	0.163385	0.136692	-0.46829	0.784825	-0.01761	0.48359	-0.24612	-0.07731	0.695658	0.373718	-0.65471
Q9NRA8	EIF4ENIF1	-0.34975	0.427458	0.048707	-0.36744	-0.51618	-0.43129	0.325678	-0.82143	0.19063	0.060228	0.117465	-0.3013	-0.23413	-0.70022	-0.04173	0.052375	0.200226	0.824834	-0.14492
Q9NRB3	CHST12	-1.13195	0.568004	0.056707	-0.60279	0.095527	0.705536	0.539936	-0.24184	0.441199	-0.01397	-0.15472	-0.26658	0.175911	-0.57891	0.152787	0.181446	0.217407	0.457455	0.341374
Q9NRD1	FBXO6	-0.3861	0.418681	0.271183	-0.26191	0.279181	0.357726	0.674042	-0.24061	0.092735	0.052454	-0.61598	-0.41758	0.198524	0.393285	0.22988	0.227943	0.380133	0.508203	0.073488
Q9NRD5	PICK1	0.732201	0.079729	-0.17012	-0.7701	-0.33249	-0.02374	-0.28898	0.081299	-0.20747	-0.36584	0.678012	-0.2642	0.175639	0.125793	0.145871	0.13888	0.038901	-1.18807	-0.32792
Q9NRD9	DUOX1	0.139603	-0.14249	0.102124	-0.2383	-0.17374	-0.3951	-0.53983	-0.19454	-0.20303	-0.00432	0.082186	-0.09673	0.286003	-0.31157	-0.20009	0.186839	-0.02426	0.413112	0.067865
Q9NRF2	SH2B1	-0.01496	0.082494	0.029141	-0.31698	-0.12585	0.118065	-0.52246	-0.58398	0.021534	-0.12356	0.151779	-0.13122	0.142993	-0.61153	-0.2305	-0.16162	-0.35168	-0.79169	-0.07607
Q9NRF8	CTPS2	0.723455	-1.1124	-0.54642	0.243241	-0.06893	0.220121	-0.02544	-0.13397	-0.15544	-0.25856	0.097244	-0.64123	-0.73276	0.113911	-0.38128	0.271815	0.605248	0.336214	-0.24127
Q9NRF9	POLE3	1.698531	-0.45015	0.076367	-0.64069	0.575321	0.291084	-0.97004	1.601099	-0.2693	-0.00744	-0.27902	-0.15448	-0.03364	-0.23654	-1.01893	-0.04828	0.377208	-0.48737	-0.59165
Q9NRG1	PRTFDC1	1.466632	-0.41375	0.289927	0.370441	1.862051	1.410672	0.288768	0.754575	-0.27305	-0.39993	-0.4338	-0.3899	-0.13296	-0.09543	-0.15515	0.440869	-0.03852	-0.15839	-0.25248
Q9NRG4	SMYD2	0.24478	0.06799	-0.35942	0.832211	-0.20342	-0.59891	-0.1494	0.335537	0.124127	-0.36947	1.08076	-0.17977	-0.20721	-0.24167	-0.24561	-0.25144	0.283519	-0.87122	0.513762
Q9NRG7	SDR39U1	0.839034	0.002335	-0.16987	-0.08809	-0.10114	-0.22159	-0.09556	0.247242	-0.24132	-1.6804	0.361724	-0.38196	-0.58105	-0.14711	-0.02433	0.088085	-0.64115	-0.25007	-0.50053
Q9NRG9	AAAS	-0.02402	-0.37901	0.577689	0.332078	0.097395	0.289576	0.745777	0.541191	-0.09339	-0.27807	0.095826	0.137555	-0.2391	0.249922	0.715099	-0.46405	0.242688	-0.372	-0.0306
Q9NRH3	TUBG2	0.049618	0.289338	0.387086	-0.61685	0.408851	0.235491	0.350036	-0.0953	0.365317	0.029336	-0.1291	-0.96294	-0.20419	-0.01461	0.270151	0.038231	0.362457	0.902907	0.814955
Q9NRK6	ABCB10	0.607651	0.27821	-0.14475	0.327538	-0.25239	-0.34514	0.022708	-0.28173	-0.31399	0.503697	0.664662	0.21203	-0.32322	0.063817	0.542416	0.145385	0.158277	-0.41054	-0.76705
Q9NRL2	BAZ1A	0.505635	0.252452	0.205384	0.17347	0.064455	0.213578	-0.23272	0.623241	-0.1926	0.458942	-0.36621	0.04958	-0.10838	0.641038	-0.88176	-0.00124	0.396198	0.295913	0.25492
Q9NRL3	STRN4	-0.20677	0.308313	-0.29808	-0.25404	-0.66022	-0.43984	0.03867	-0.12373	0.810956	-0.56655	-0.29861	-0.40372	0.189029	-0.01627	0.186656	0.324324	-0.0517	-0.03925	0.015105
Q9NRN5	OLFML3	-2.51113	-0.50532	-1.36632	-1.89017	-1.62044	-1.93037	-1.05292	-2.64878	0.125726	-0.32465	-0.88364	-1.98537	-1.23895	-1.23905	-0.54627	-1.01541	-1.53395	0.113798	-0.05324
Q9NRN7	AASDHPPT	0.512697	0.342299	0.230815	0.484223	-0.30755	-0.12615	0.401963	0.215109	0.034164	-0.07081	0.66797	-0.10579	0.610705	-0.00672	0.210839	0.386099	0.411931	0.060034	-0.19835
Q9NRP0	OSTC	0.106307	0.394847	0.557794	-0.02701	1.106981	0.433704	0.78189	-0.12624	0.304994	-0.52224	0.568508	0.488244	0.10626	0.37247	0.265754	0.140055	0.395706	0.331208	0.750768
Q9NRR4	DROSHA	0.15531	0.011084	-0.0353	0.1913	-0.01194	0.373691	-0.14671	0.070751	0.048754	-0.02731	-0.22568	-0.22972	-0.01876	0.132684	-0.20401	0.031503	0.365172	0.552618	0.049068
Q9NRR5	UBQLN4	-0.00346	-0.29304	-0.47356	-0.37855	-0.22443	-0.27772	-0.25603	0.207174	0.232223	-0.10276	-0.10758	-0.38292	0.185113	-0.02689	0.352102	-0.42296	0.096141	0.027786	0.111255
Q9NRS6	SNX15	0.14566	0.282659	-0.08757	0.077159	0.874884	0.775139	-0.27224	0.408572	0.036256	0.291425	-0.0944	0.301338	0.253218	0.448519	-0.27984	0.732993	0.140991	0.024062	-0.35463
Q9NRV9	HEBP1	-0.15745	-0.42712	-1.60667	-1.4031	0.903604	-1.07125	-0.80167	-0.48475	0.361641	0.423013	-0.1004	0.136365	0.293884	-0.84673	-0.99378	-1.07472	0.069173	0.176783	-0.94261
Q9NRW1	RAB6B	0.240452	0.052955	-1.63901	0.381414	2.366171	1.617836	-0.19703	0.082311	0.15278	1.684368	0.546023	-0.16305	0.272965	0.051684	0.857913	-0.28297	0.161306	-1.23226	-0.59825
Q9NRW3	APOBEC3C	-0.2155	1.061671	0.579087	-0.23527	0.189859	0.405308	-0.22298	-0.20232	-0.29498	0.76252	0.825222	0.179855	0.074261	0.405625	-0.45797	0.760123	0.96407	1.07701	-0.08569
Q9NRW7	VPS45	0.225066	0.388411	0.5017	0.293633	0.501861	0.40693	-0.01316	-0.07589	0.140095	0.343539	0.219607	-0.16423	0.243526	-0.38005	-0.08411	-0.20715	-0.28081	0.172042	0.051397
Q9NRX1	PNO1	-0.34291	-0.22613	0.089977	0.658012	-0.04898	-0.09471	-0.16773	0.622936	-0.13305	0.797301	0.543387	-0.24821	-0.06786	-0.5434	0.194617	0.284821	-0.06155	0.15941	-0.19501
Q9NRX2	MRPL17	-0.53713	0.023015	0.516165	1.328743	0.433509	0.522067	0.230725	-0.35263	-0.16552	-0.04137	0.727149	-0.47415	-0.00324	-0.05638	0.638038	-0.06028	-0.28128	-0.27818	-0.41562
Q9NRX4	PHPT1	0.03233	-1.83187	-0.96252	-0.38435	0.694178	-0.94174	0.26376	0.421011	1.312608	0.570774	-0.37319	0.017659	0.418917	0.302962	-0.15571	0.161485	1.132719	0.720867	0.797508
Q9NRX5	SERINC1	0.497856	0.458825	-0.01682	0.315487	0.104189	0.147793	0.141751	-0.97864	-0.22704	-0.61726	-0.99535	0.692934	0.163828	0.402846	0.894314	0.22443	0.439391	-0.48553	-0.77641
Q9NRY4	ARHGAP35	0.203113	-0.23073	-0.71975	0.029347	-0.69077	-0.34517	-1.18862	-0.25604	0.111238	-0.48221	-0.17058	-0.26422	-0.05021	-0.0557	0.423185	-0.40384	0.213034	-0.41961	-0.27106
Q9NRY5	FAM114A2	-0.96105	0.552219	0.299422	0.514141	0.536528	0.684493	-0.00817	-0.74879	-0.05003	0.566512	-0.38647	-0.31949	-0.05556	0.154978	0.40804	0.127426	-0.20289	0.162734	0.078585
Q9NRY6	PLSCR3	-0.58373	-0.06928	-0.09466	-0.26298	-0.43925	-0.589	-0.34987	-0.14329	0.772065	-0.62982	-0.75046	-0.34523	-0.02564	-0.38648	-0.18755	-0.36301	-0.90684	-0.29043	0.50908
Q9NRZ5	AGPAT4	-0.6676	0.121987	-0.41505	-0.59307	-0.88428	-0.83304	0.346338	-0.10356	0.256191	-0.38364	0.21264	-0.12767	-0.40698	0.082345	-0.28657	-0.80209	-0.22296	0.101507	-0.58017
Q9NRZ7	AGPAT3	-0.16477	0.456398	0.452263	-0.15223	0.283064	-0.00792	0.064714	-0.07398	0.023263	0.141699	0.263098	0.943672	0.311571	0.472179	0.234479	0.022575	0.50626	-0.0753	-0.75057
Q9NRZ9	HELLS	1.258042	-0.12994	-0.4268	0.054366	1.355975	1.65139	-0.4273	0.050043	-0.3538	-0.40736	-0.05289	-0.4955	0.381754	-0.58165	-0.92762	-0.22209	0.48589	-0.33542	-0.50176
Q9NS00	C1GALT1	-0.18371	0.811461	0.428498	-0.16666	1.155934	1.055152	-0.02489	-0.14408	-0.00156	0.248649	-0.65566	-0.02866	0.054098	0.501166	0.127947	0.392684	1.787767	-0.31752	-0.22571
Q9NS15	LTBP3	0.328287	-0.41494	-0.18197	-0.98386	-0.74926	-0.67846	-0.00589	0.333228	0.201322	-0.57418	0.243191	-0.8154	-0.15992	-0.7299	0.131383	-1.12634	-0.40427	-0.60429	0.019121
Q9NS69	TOMM22	-0.217	0.052768	-0.45317	0.623526	-0.40047	0.799715	0.410513	0.17519	-0.29823	-0.16413	-0.01839	-0.21393	0.315572	-0.17695	1.233916	0.652268	0.078062	-0.00553	-0.24851
Q9NS73	MBIP	-0.15499	-0.12616	-0.29565	0.122702	0.228608	0.306733	-0.18994	0.273673	-0.31294	-0.99326	0.096726	-0.77867	-0.11709	-0.44161	0.634183	0.429949	-0.23004	0.245467	-0.34302
Q9NS86	LANCL2	0.392818	-0.86858	-0.58288	0.101352	-0.10842	-0.65633	-0.72612	0.714771	-0.1983	-0.23344	0.187273	-0.19248	-0.13413	-0.18088	0.331931	-0.2149	0.638616	-0.23344	0.162845
Q9NS87	KIF15	0.715445	-0.20762	0.456047	0.134939	1.12647	1.38539	0.608615	1.017742	0.118277	-0.5091	-1.37928	-0.23347	-0.062	0.256557	-0.23404	-0.6426	0.240014	-0.44466	0.089781
Q9NSB2	KRT84	-0.17879	0.546438	0.025397	-0.12628	-0.26235	-0.23996	1.10559	-0.30771	-0.52673	-0.08998	0.399737	-0.15216	0.099507	-0.06715	-0.4473	-0.34998	-1.16449	0.2012	-1.4356
Q9NSB8	HOMER2	1.240968	-0.1971	-0.27027	-0.65638	0.089745	-0.13712	-0.04621	1.166714	-0.18268	-1.14297	0.086433	-0.55943	-0.29021	0.087675	0.36468	0.000353	-0.5755	-1.15777	-1.10833
Q9NSC5	HOMER3	-0.53307	0.335326	0.206636	0.31374	-0.5163	-0.49358	-0.10388	-0.02635	-0.59331	0.541849	-0.02505	-0.29078	0.060798	0.06656	0.119145	0.279735	-0.68012	0.086002	0.653989
Q9NSD9	FARSB	0.330216	-0.67724	-0.30546	-0.11681	0.022849	0.099757	-0.72352	-0.14875	-0.79699	-0.47211	0.556038	-0.37703	-0.67797	-0.2465	0.792012	0.698553	0.111859	-0.25203	-0.59864
Q9NSE4	IARS2	0.266522	-0.4828	-0.35651	1.097157	-0.93961	-1.00208	-0.81932	-0.1112	-0.51301	0.669922	0.135247	-0.10368	-0.10551	0.039002	1.40232	0.918695	-0.4881	-0.63475	-0.65025
Q9NSI2	FAM207A	-0.06273	0.198114	-0.31961	0.670816	-0.00551	0.256631	-0.08073	0.499757	0.209951	0.31787	0.062636	-0.01075	0.214044	0.755348	0.370588	0.731721	0.007302	-0.35235	0.254223
Q9NSI6	BRWD1	0.187131	0.266013	0.14439	-1.02739	0.548762	1.949508	-0.21062	-0.17723	0.304653	0.150869	0.066895	0.207053	0.190713	0.248596	1.275649	-0.08278	0.251307	0.490144	-0.08755
Q9NSI8	SAMSN1	-1.06198	0.8471	0.645924	-0.07532	0.338181	0.662379	0.273309	-1.07068	-0.01903	0.916658	-0.42269	1.358367	0.161744	1.203764	-1.0241	-0.23637	0.212513	0.622763	-0.49925
Q9NSK0	KLC4	-0.41238	0.19616	-0.26895	-0.10467	-0.25396	-0.02687	0.303566	-0.10479	0.50059	-0.11493	0.35158	-0.54879	-0.46688	-0.04141	0.710115	-0.32106	-0.50632	-0.74236	0.109918
Q9NSU2	TREX1	-0.59535	-0.05027	0.321793	0.579209	0.836895	1.270966	-0.42247	-0.27544	-0.17845	-0.03143	1.105033	-0.25982	0.082725	0.04248	0.739857	-0.57675	-1.07807	-0.09294	-2.07158
Q9NSV4	DIAPH3	0.566558	-0.05526	0.14515	0.313578	0.657783	0.518328	0.00096	0.973177	0.203731	0.404363	-1.25879	-0.01886	0.1856	0.174396	-0.04539	0.036947	0.291479	0.564605	0.793547
Q9NSY0	NRBP2	0.291925	-0.04403	0.017289	-0.65545	0.452394	0.885342	0.029079	0.063717	0.121581	-0.29415	-0.09662	-0.10034	0.329578	-0.02144	-0.71047	-0.18677	-0.90051	-0.79332	-1.37607
Q9NSY1	BMP2K	-0.3128	1.204802	1.300854	-0.87629	0.22865	0.04714	-0.00778	-0.69168	0.209525	0.162534	-0.33412	0.518429	0.190641	0.335719	0.638633	-0.41822	-0.27481	0.44949	-0.11781
Q9NT22	EMILIN3	-0.93905	1.240898	0.105095	-0.69257	-1.70913	-1.21208	1.017895	-0.08831	0.745276	-2.55306	-1.15061	-0.98442	-0.03576	-2.12266	-2.49501	-1.36496	-0.14706	-2.02673	-0.57573
Q9NT62	ATG3	-0.33074	0.719163	0.572233	-0.22515	0.030306	0.037709	-0.15599	0.057599	-0.46744	0.656844	-0.10414	0.675482	0.512381	0.955872	0.169521	-0.17328	0.29137	-0.30753	-0.01795
Q9NTG7	SIRT3	0.155324	-0.61621	0.085421	0.06857	1.524353	0.684952	-0.13544	1.031971	-0.0191	-0.19547	0.704753	-0.96698	-0.01906	-0.32071	0.765276	0.769048	-0.35189	-0.23823	-0.31856
Q9NTI5	PDS5B	0.387565	-0.20139	-0.14363	-0.1734	0.805885	1.225186	-0.36859	0.204385	-0.23339	0.158137	0.109359	-0.2202	-0.55314	0.226889	0.529298	-0.72334	0.001729	0.134206	-0.10261
Q9NTJ3	SMC4	1.111331	-0.24574	0.683324	0.466493	1.696921	1.960201	0.688221	1.476913	0.162795	-0.54818	-0.69118	-0.61302	0.066984	-0.38338	-0.38928	0.277366	0.003382	-0.57836	0.317277
Q9NTJ4	MAN2C1	0.23596	-0.33772	-0.61428	0.189181	-0.30617	-0.23291	-0.38852	-0.81939	0.769094	-0.23976	0.522789	-0.41402	-0.68231	-0.73039	0.216076	-0.59615	-0.99262	-0.80596	-0.52445
Q9NTJ5	SACM1L	-0.19782	0.189232	-0.24328	-0.6117	0.179095	-0.04736	0.450431	-0.51792	0.054029	-0.07289	0.081019	0.499249	0.106672	0.693282	0.322334	-0.51735	0.105848	-0.04596	-0.40348
Q9NTK5	OLA1	0.795601	-0.88861	0.148201	1.109973	0.218032	-0.23061	-0.72887	0.856827	0.587768	1.51095	0.543799	-0.2309	0.591152	0.061009	0.095329	0.422183	0.799228	0.818597	0.018436
Q9NTM9	CUTC	0.014573	-0.0737	0.439445	0.690735	0.361426	0.694364	-0.61109	-0.72272	0.060352	0.301566	0.285387	-0.29561	0.251105	-0.05862	1.136632	0.108735	-0.10146	0.259876	-0.44531
Q9NTX5	ECHDC1	-0.11547	-0.04403	0.185453	-0.58815	-0.05778	-0.71161	-0.14023	-1.19415	0.139583	0.270919	0.538989	-0.1683	-0.09207	-0.15739	-0.36892	0.121916	-0.19316	0.176987	-0.17038
Q9NTZ6	RBM12	0.593807	-0.34793	-0.79557	-0.03439	-0.02954	0.183032	-0.2936	0.47666	-0.26321	-0.06516	0.462335	-0.17034	-0.35147	-0.53	-0.14248	-0.44281	-0.12132	0.047994	0.01018
Q9NU22	MDN1	1.032016	0.012451	-0.46992	0.20623	-0.39693	-0.14623	-0.28049	-0.59013	0.121842	-0.12456	0.475632	-0.26063	-0.21449	-0.37557	0.467652	-0.52652	-0.11698	-0.24764	-0.53502
Q9NUA8	ZBTB40	0.052377	0.068026	-0.01412	-0.46875	-0.43057	-0.50171	-0.52149	0.186864	-0.30747	0.253509	-0.01608	-0.14474	-0.74984	-0.10741	0.358248	0.233638	-0.08551	0.158581	-0.32346
Q9NUB1	ACSS1	-0.43663	-0.31077	0.303984	-0.20159	-0.42311	-1.24001	0.404386	1.169251	0.676907	1.472706	0.114521	0.008022	0.234774	-0.21929	0.831862	-0.66997	-0.69736	-0.1552	-0.61449
Q9NUD5	ZCCHC3	0.412455	0.486678	-0.07784	-0.96245	-0.76609	-1.33326	0.566425	0.06029	-0.84155	-0.32145	0.446494	-0.59869	-0.16912	0.57901	-1.43873	-2.17503	0.332262	-0.20942	0.248549
Q9NUG6	PDRG1	0.118643	-0.10929	-0.21434	-0.78249	-0.52458	-0.13999	-0.3411	2.076028	0.725048	0.100471	0.336272	-0.87026	0.026618	-0.66675	0.967432	0.005958	-0.52708	-0.76335	0.276062
Q9NUI1	DECR2	0.400932	-0.38271	-0.27467	0.032726	0.417975	0.694141	-0.37972	-0.70643	-0.12775	-0.68785	-0.3118	-0.05059	-0.0705	0.022457	0.083403	0.284033	-0.20055	-0.18689	-1.18335
Q9NUJ1	ABHD10	0.027294	-0.35353	-0.03579	0.096689	-0.36883	-0.65801	-0.60756	0.512928	0.275462	0.857343	0.184618	-0.5632	-0.40859	-0.39294	1.194642	0.506844	-0.34014	0.023658	-0.29215
Q9NUJ3	TCP11L1	-0.59945	-0.13011	-0.17855	-0.59443	-0.15829	0.051511	0.057211	-0.45252	0.233434	0.620961	-0.65745	-0.07235	0.157594	0.341564	-0.12552	0.319242	0.006588	-0.13976	0.694131
Q9NUL3	STAU2	0.119562	-0.31485	-0.18565	-0.58736	-0.09235	0.249374	0.070415	0.170662	0.046734	0.08566	0.228124	-0.48082	0.364381	-0.54043	-0.03694	-0.02863	-0.44044	-0.25181	0.559143
Q9NUL5	RYDEN	-0.21884	-0.38297	-0.06476	-0.73906	-0.48561	-0.43372	-0.16829	0.419565	0.172195	0.00849	0.323521	-0.72439	0.224033	-0.59405	-0.70593	0.182946	-0.38966	0.855005	-0.15057
Q9NUL7	DDX28	-0.74903	-0.09339	0.00455	0.956251	0.129091	0.326006	0.067104	-0.86995	0.627297	-0.09214	0.513698	-0.13603	-0.06912	-0.20862	0.733216	-0.434	-0.4358	-0.16605	-0.40751
Q9NUM4	TMEM106C	0.893363	-0.25414	-0.29143	0.142681	-0.4064	-0.11577	-0.04854	0.09648	-0.28326	0.396856	0.146543	-0.02911	-0.28285	-0.31597	0.262912	0.269146	-0.01726	-0.37841	
Q9NUP1	BLOC1S4	0.295055	0.211405	0.217825	-0.77754	-0.18622	-0.64377	0.180674	-0.43382	0.140461	-0.43475	0.155184	0.28215	-0.22618	-0.08212	-0.37304	-0.44341	-0.3792	-0.36586	-1.08267
Q9NUP9	LIN7C	0.581462	-0.95925	-0.5648	-0.42972	-0.12855	-0.67007	-0.7657	0.353781	-0.20519	-0.49154	0.770898	-0.33455	-0.322	-0.61902	0.474775	-0.37463	-0.48656	0.040547	-0.23649
Q9NUQ2	AGPAT5	0.170748	0.04826	-0.28738	0.032833	0.163794	-0.43496	-0.25603	-0.52019	-0.45078	-0.40454	0.939307	0.313002	-0.47673	0.181501	-0.50889	0.722626	0.56798	-0.29948	-0.17347
Q9NUQ3	TXLNG	0.291028	-0.23947	-0.37754	0.351493	0.370776	0.40905	-0.45482	-0.28223	-0.36051	-0.55619	0.25899	-0.69406	-0.45346	-0.37558	-0.58092	0.047321	0.278423	0.436381	-0.46736
Q9NUQ6	SPATS2L	0.695819	0.210247	0.669546	-0.97655	-0.24063	-0.5258	-0.65157	-0.18777	-0.24552	-0.33313	-0.39887	-0.69494	0.470385	-0.6391	-1.22031	-0.12553	-0.71105	0.71532	0.721016
Q9NUQ7	UFSP2	-0.63172	0.060819	0.144299	0.903893	0.021562	0.134886	0.141326	-0.26709	-0.51312	0.094493	0.253215	-0.02566	-0.72868	-0.01172	-0.55794	0.011282	0.141652	-0.07526	0.208469
Q9NUQ8	ABCF3	0.396348	0.039191	0.313323	0.39835	0.492912	0.911235	-0.21403	-0.21738	-0.09787	0.206116	-0.54444	-0.25663	-0.09066	-0.25121	1.480957	-0.32251	0.173927	-0.21967	0.169094
Q9NUQ9	FAM49B	-0.46075	0.857178	0.486912	-0.1854	0.175698	0.288822	0.201464	-0.57385	-0.04806	1.300121	-0.76658	-0.40079	0.690663	0.276207	0.504552	0.718463	-0.42723	0.008521	-0.6035
Q9NUS5	AP5S1	0.516955	-0.13978	0.111961	0.025416	0.693347	0.631995	-0.21483	-0.02298	-0.12716	0.021005	0.112202	-0.45842	0.067645	0.230425	0.111288	-0.02281	-0.27058	0.055993	0.255334
Q9NUT2	ABCB8	-0.01184	0.876722	0.243557	0.665272	0.638261	0.405078	-0.22424	0.769371	0.522081	0.33951	-0.73025	0.123386	-0.65465	-0.02189	1.307119	0.081977	-0.62322	-0.97575	-0.0265
Q9NUU6	FAM105A	0.218625	0.860798	0.560037	-0.69615	1.096314	0.661424	-0.3739	-0.91849	-0.33291	1.337947	-1.21592	1.579603	0.724391	1.513157	-1.03712	-0.11807	0.633943	-0.13009	-0.9947
Q9NUU7	DDX19A	0.041186	0.25547	-0.0057	0.309928	-0.45507	0.22926	-0.49839	-0.34317	-0.01409	0.014781	0.073994	-1.09674	-0.00704	-0.30569	0.045922	0.280803	-0.11444	-0.19985	0.447167
Q9NUV7	SPTLC3	0.528087	-0.07835	-0.05506	0.713077	-0.67734	-0.65197	0.065967	-0.49173	0.183376	-0.21345	0.083397	-0.56632	0.002419	0.144397	1.672212	0.145556	-0.51947	-0.33918	0.248069
Q9NUV9	GIMAP4	-1.70824	0.995988	1.446818	-1.18923	0.846861	0.371657	0.186966	-1.26472	0.176206	1.205341	-1.39573	0.903836	0.021409	1.159579	-0.0769	-0.33295	-0.07421	0.705733	-0.5634
Q9NUW8	TDP1	0.683078	-0.15762	-0.0666	0.249308	-0.01899	0.521317	-0.08409	0.360319	-0.45984	-0.13952	0.351889	0.032919	-0.16103	0.246104	-0.60099	0.225014	0.158687	-0.41733	0.199478
Q9NUY8	TBC1D23	-0.14097	0.505027	0.38885	-0.07236	0.066519	0.271302	0.170345	-0.15238	1.183773	0.621672	-0.24468	0.528926	0.356159	0.378666	0.889717	0.505204	0.15055	-0.38845	0.264922
Q9NV06	DCAF13	0.243249	-0.08457	-0.23957	0.677586	-0.32378	-0.04093	-0.37452	0.345765	-0.31238	-0.52924	0.975215	-0.03045	-0.00887	-0.69443	0.179879	-0.44776	0.167085	-0.25127	-0.23498
Q9NV31	IMP3	0.381711	0.173554	-0.48325	0.86013	-0.16174	0.064132	0.596938	0.51215	-0.3635	0.399878	1.007024	-0.38976	0.112138	-0.12344	-0.23179	-0.29911	-0.03038	0.458583	0.311467
Q9NV70	EXOC1	-0.29001	0.164485	0.213547	0.21305	-0.13673	-0.31036	0.485785	-0.08883	-0.28725	-0.05184	0.223603	0.056276	-0.21195	0.257864	0.339442	0.122204	-0.30499	-0.36423	0.228655
Q9NV88	INTS9	0.456128	0.203779	0.068065	-0.20018	-0.00439	0.054942	0.006328	0.690284	0.191091	0.21108	0.627712	-0.29987	-0.79344	-0.05296	0.049477	0.182308	0.302155	0.341549	0.45793
Q9NV96	TMEM30A	-0.134	0.414743	0.464972	0.366042	0.202522	0.060504	0.49702	-0.92997	-0.58688	0.185226	-0.24666	0.394011	0.19195	1.095289	0.039068	-0.07099	1.067315	0.637546	-0.45548
Q9NVA1	UQCC1	0.091265	-0.5831	0.113531	0.25035	0.596938	0.662588	-0.37841	1.294326	0.100048	0.166415	0.523712	-0.48865	0.639455	-0.53938	0.696792	0.011611	-0.84145	-0.85702	-0.83158
Q9NVA2	SEPT11	-0.2281	-0.34526	-0.04963	-0.96273	0.109056	-0.27755	-0.64887	-0.15131	0.414547	0.114122	-0.06873	-1.06338	0.146844	-0.59094	-0.3515	0.068452	-0.95872	0.750632	1.356664
Q9NVC6	MED17	0.624621	0.224332	0.03017	0.413169	-0.08724	0.560207	-0.23093	0.53001	-0.16978	-0.37594	0.255676	-0.10703	0.368605	-0.38226	0.275664	0.233427	0.156306	0.107825	-0.04474
Q9NVD7	PARVA	-0.67412	-0.10927	-0.68314	-1.07747	-0.3371	-0.16766	-0.06568	-0.41742	-0.00911	-0.46083	-0.3383	-1.59621	-0.36173	-1.15538	-0.25167	-0.37901	-0.71121	0.308841	0.635157
Q9NVE5	USP40	-0.08638	0.000189	-0.10325	-0.33665	-0.34187	0.236485	-0.34348	-0.50679	-0.14926	-0.21258	0.564264	0.014435	0.023166	0.055673	-0.61104	-0.17533	-0.12456	-0.32891	0.136894
Q9NVE7	PANK4	0.272914	0.287577	0.154988	0.321257	-0.39878	-0.42689	-0.46898	-0.53657	-0.43817	0.484555	-0.15791	0.123674	-0.01279	0.335022	0.018004	-0.07625	1.052607	0.453132	-0.17957
Q9NVF7	FBXO28	0.292429	-0.17603	-0.1706	-0.54826	-0.29937	0.033697	-0.21202	-0.28965	-0.21731	0.058713	-0.32111	-0.91832	-0.17599	-0.00467	0.382416	0.101762	0.072978	-0.15372	-0.1004
Q9NVG8	TBC1D13	-0.09642	0.517749	0.344592	-0.49132	-0.01587	-0.35042	-0.52626	0.057817	0.701306	-0.45023	0.246752	-0.58921	-0.04429	0.130985	-0.56792	-0.74955	-0.25413	-0.42827	0.574541
Q9NVH0	EXD2	-0.07173	-0.09932	0.190988	0.677571	-0.24021	-0.33845	1.2673	0.7902	0.066423	-0.53633	0.618226	-0.27797	-0.34307	-0.39099	0.535255	0.385841	0.046751	-0.20422	0.478675
Q9NVH1	DNAJC11	0.136142	0.000545	-0.03311	0.566566	0.172604	0.182109	0.292069	0.418782	-0.95973	-0.08791	0.577413	-0.53688	-1.02196	-0.61715	0.888129	1.418568	0.081256	-0.21421	-0.06493

Q9NVH2	INTS7	0.21843	-0.21356	-0.24907	-0.00782	-0.25066	-0.27369	-0.02324	0.675526	-0.21869	-0.205	0.538305	-0.17021	-0.26468	-0.09376	0.475196	-0.17819	-0.04609	-0.01309	0.272279
Q9NVH6	TMLHE	0.630738	-0.61792	0.744279	0.975552	-0.38909	-0.31317	-0.93185	-0.28178	-0.55658	-0.82698	0.885478	-0.44417	-0.59566	-0.14314	0.632554	-0.07391	-0.80153	-0.24665	-0.37144
Q9NVI1	FANCI	1.764986	-0.2728	-0.07238	0.361491	1.682785	2.187362	0.090964	0.633309	-0.56952	-0.6689	-0.21868	-0.38461	-0.42999	-0.48609	-0.41977	0.291781	-0.79673	-0.3267	-1.12769
Q9NVI7	ATAD3A	0.259127	0.474718	0.252385	0.581816	1.294547	1.392857	0.498694	0.136419	0.011582	1.211296	0.047824	-0.13512	-0.42578	-0.2752	0.464421	1.439777	0.521196	0.393299	-0.18083
Q9NVJ2	ARL8B	0.142446	-0.0539	0.09068	-0.78924	-0.12509	-0.30548	0.295893	-0.18362	-0.64272	0.057402	-0.63716	0.9947	-0.10993	1.118994	-0.12798	-0.72389	0.17114	-0.66035	-0.18009
Q9NVK5	FGFR1OP	0.106155	0.496675	0.248836	-0.87402	-0.07964	-0.3858	0.160658	-0.50429	0.24908	-0.02821	-0.16535	0.164761	-0.58166	0.293928	-0.46389	-0.49018	0.336547	0.369169	-0.48249
Q9NVM1	EVA1B	-0.10287	-0.08986	-0.27353	-0.6584	-0.24179	0.116808	0.360309	-0.38453	1.044553	-0.34718	-0.3304	-1.15244	-0.70598	0.06707	0.947375	-0.12702	-0.56744	0.624656	-1.01313
Q9NVM4	PRMT7	-0.23171	0.290903	0.014544	0.546983	0.379528	-0.06696	0.285326	-0.08027	0.101868	0.071451	-0.57923	0.036638	-0.10057	-0.05118	0.61755	0.445171	0.934401	-0.26959	0.035714
Q9NVM6	DNAJC17	0.826768	-0.52586	0.340727	0.477455	-0.31899	-0.34508	-0.67878	-0.60156	-0.26928	-0.26345	0.819183	-0.74387	0.003802	-0.54279	-0.58534	0.861999	-0.22639	0.47945	0.00991
Q9NVM9	INTS13	0.591195	0.035702	-0.35324	-0.09965	-0.4415	-0.07317	0.185303	0.894721	-0.27147	0.129436	0.479063	-0.46797	-0.22387	-0.16112	0.300506	0.019136	-0.14748	0.052849	0.037987
Q9NVN3	RIC8B	0.484516	-0.01911	-0.27511	0.09845	0.319966	0.353663	-0.18234	0.068234	-0.00348	-0.02754	0.537567	-0.36373	0.079544	-0.42606	-0.12919	-0.01897	-0.03031	-0.40631	-0.15589
Q9NVP1	DDX18	0.560613	-0.23645	-0.43614	0.940531	-0.69572	-0.48311	-0.59104	0.363763	-0.36774	-0.25307	0.621918	0.189576	-0.11433	-0.42791	-0.32617	0.04215	0.484578	-0.09888	-0.23151
Q9NVQ4	FAIM	0.329753	-0.44341	-0.01885	0.090559	1.773934	0.792855	-0.23647	0.837161	0.213002	0.227785	-0.28317	-0.05673	0.110706	0.415876	0.185164	-0.53965	0.907737	0.336299	0.512529
Q9NVR2	INTS10	0.141332	-0.0717	-0.24979	-0.0155	-0.1435	-0.14596	0.111145	1.014081	0.091865	-0.22277	0.144412	-0.05698	-0.26884	0.043639	0.363082	-0.26214	0.202424	0.084835	0.573663
Q9NVS2	MRPS18A	-0.38	-0.09312	0.123334	1.183631	0.450909	0.631718	-0.27068	-0.55314	-0.04521	0.530366	0.47122	-0.22382	0.172916	-0.49907	0.923667	-0.81831	-0.36564	-0.0904	-0.75901
Q9NVS9	PNPO	-0.39714	-1.28812	0.372004	1.020645	1.142657	0.7056	0.183539	0.917439	0.768782	1.415891	0.522343	0.288831	1.195473	-0.27676	0.253189	0.266339	0.493246	0.352612	-0.57058
Q9NVT9	ARMC1	0.239882	-0.67181	0.275747	-0.00318	0.36507	-0.05166	0.170203	0.522937	-0.30123	-0.22029	0.674616	0.058701	-0.20391	-0.29652	-0.29262	0.338531	0.209956	-0.24194	0.153287
Q9NVU0	POLR3E	0.153174	-0.0765	0.061724	-0.18337	0.042681	-0.20816	-0.30472	0.244737	-0.2034	-0.72534	0.18783	0.18919	0.21651	0.005122	0.056607	0.276016	0.251943	0.155096	-0.16997
Q9NVU7	SDAD1	0.279984	0.147005	0.702464	-0.00089	-0.19135	0.298961	-0.19029	-0.19044	-0.21744	0.053661	0.932441	-0.70119	0.11991	-0.8577	-0.04776	-0.5354	-0.23008	0.343415	-0.46121
Q9NVV4	MTPAP	0.075908	0.214706	0.186364	0.850709	0.38829	0.017372	-0.51138	-0.41501	-0.05775	0.413998	0.719249	-0.27424	-0.52477	-0.33744	1.011277	1.134107	-0.46666	-0.44779	-0.2085
Q9NVX2	NLE1	0.012842	0.853594	0.050469	0.729284	-0.14999	-0.38562	0.025389	-0.07393	-0.41701	0.034073	0.872937	-0.01832	-0.52869	0.028661	-0.31834	0.191084	0.365433	0.488519	0.182111
Q9NVX7	KBTBD4	0.39462	0.094778	-0.1266	-0.28838	-0.40592	-0.17677	-0.349	0.244613	-0.32944	-0.16583	1.078524	-0.65437	0.234465	-0.11249	0.432391	-0.09446	-0.69768	-0.62148	-0.42685
Q9NVZ3	NECAP2	-0.40056	0.061928	0.759808	-0.41404	0.502059	-0.02598	0.32128	-0.46368	0.535836	1.05626	0.097564	0.677843	0.07498	0.545957	0.002309	-0.10802	0.214312	0.108718	0.194277
Q9NW08	POLR3B	0.523216	-0.20908	-0.42597	-0.3777	-0.34499	-0.26587	0.009144	0.352599	-0.12589	-0.42507	0.355252	-0.10095	-0.04076	-0.52276	0.073253	-0.08216	0.222059	-0.09411	-0.11728
Q9NW13	RBM28	-0.01484	-1.28759	-0.2571	1.363439	0.031602	0.209666	-0.38844	0.822703	-0.32334	-0.21603	0.251122	-0.54803	-0.41506	-0.87745	0.48292	-0.31523	-0.38622	-0.03264	-0.36097
Q9NW15	ANO10	-0.52253	0.563306	0.519568	-0.25459	-0.06622	0.036751	1.000904	-0.5865	-0.486	0.242927	-0.34058	0.364738	0.149846	0.625595	-0.39183	0.364959	0.134107	0.229591	0.717186
Q9NW61	PLEKHJ1	-0.03945	-0.23788	0.176082	-0.57071	-0.70524	-0.01237	-0.11031	0.357442	0.381633	1.754394	-0.0685	-0.26505	-0.36738	0.048308	-0.0595	-0.35608	0.383296	0.080237	0.424874
Q9NW64	RBM22	0.165427	0.149839	-0.35587	0.113087	0.364574	0.363793	-1.05739	0.180713	-1.12537	-0.14016	0.50603	-0.39468	-0.46148	-0.15883	0.427966	-0.20921	0.141122	0.125884	-0.2118
Q9NW68	BSDC1	0.05435	0.400696	0.172114	0.052505	0.044676	0.220491	-0.12909	0.269456	0.577465	0.295482	-0.39513	-0.00377	-0.16041	0.309979	0.480134	0.729173	0.204669	0.635584	-0.23555
Q9NW75	GPATCH2	0.53465	-0.02577	0.165667	-0.55137	-0.12758	-0.65231	0.341012	0.054215	-0.3112	0.202598	0.078715	-0.33047	-0.12436	-0.08275	-0.12067	0.095427	-0.28869	-0.09052	-0.20304
Q9NW82	WDR70	0.052686	-0.2871	-0.61389	0.505672	-0.42785	-0.18228	-0.52038	0.10906	0.148265	-0.04538	0.017194	-0.46549	-0.40192	-0.37381	0.599866	0.194972	-0.30772	-0.34396	0.194526
Q9NWB6	ARGLU1	0.582015	-0.41984	0.006223	-0.25274	0.864469	1.224324	-1.28453	0.927731	-0.51382	0.186867	0.865888	-1.18881	-0.42198	-1.20797	0.043183	-0.38557	-0.27774	0.502397	0.2577
Q9NWB7	IFT57	0.072204	0.061146	-0.48277	0.12651	-0.39911	0.098111	-0.30365	-0.29772	0.040847	-0.68209	1.732243	-0.80516	-0.22388	-1.24368	-0.03035	-0.44981	-0.25912	-0.9519	0.022083
Q9NWH9	SLTM	0.399938	-0.15224	0.16486	0.234381	-0.28756	0.601797	0.103713	0.209909	-0.2044	-0.72134	0.440407	-1.0715	-0.14311	-1.00852	0.299978	-0.318	-0.16107	0.74778	-0.37913
Q9NWK9	ZNHIT6	0.964493	-0.41996	-0.13626	0.083793	-0.00887	0.261672	-0.27841	0.452412	-0.09817	-0.11642	-0.36936	-0.92748	-0.20746	-0.64206	1.120257	-0.30633	0.017188	0.157235	0.026924
Q9NWM3	CUEDC1	0.055009	-0.1473	-0.17678	0.191443	-0.40012	-0.14403	0.030874	-0.11034	-0.17472	-0.38331	0.746378	-0.17411	0.073523	-0.47276	0.285587	0.114052	-0.91926	-0.92872	0.617366
Q9NWQ8	PAG1	-0.20701	0.424985	-0.41324	-0.29268	0.09671	-0.82418	0.085941	-0.4808	0.984465	0.452302	0.000772	0.191972	-0.27289	0.209237	-0.8337	-0.11381	-0.04283	0.68382	-0.81806
Q9NWS0	PIH1D1	0.06356	0.330809	-0.01067	-0.35067	-0.0611	0.213876	0.007936	0.184792	0.108744	0.365037	0.241962	-0.34246	0.381545	-0.43388	0.039094	-0.67475	0.062168	-0.39042	0.263431
Q9NWS8	RMND1	0.761518	0.171486	0.01749	0.423715	0.439468	-0.02083	-0.40567	-0.25221	-0.38072	-0.00313	0.517915	-0.79145	-0.3267	-0.06798	0.967672	1.770062	-0.56043	-0.74773	-0.24171
Q9NWT1	PAK1IP1	0.762693	0.677443	-0.52522	1.155551	0.428734	0.321885	-0.99955	0.072638	0.040847	-0.14259	-0.23168	-0.05727	0.518787	-0.09195	0.349388	0.767246	-0.26714	-0.16601	-0.35286
Q9NWT6	HIF1AN	0.053131	-0.47612	-0.32449	0.9582	-0.17518	-0.18629	-0.15846	0.064072	-0.22908	0.812939	0.220238	-0.5509	-0.2542	-0.26725	-0.20075	-0.23707	-0.11645	-0.0588	0.279778
Q9NWU1	OXSM	0.297093	-1.01008	0.025037	-0.12531	0.488656	0.555353	0.08825	-0.58208	-0.22109	-0.94485	0.79096	-0.08171	0.730038	-0.10138	0.087837	0.971306	-0.36106	-0.15561	-0.66731
Q9NWU2	GID8	0.093775	-0.61241	0.33446	0.202769	-0.1199	0.52369	-0.03774	-0.09108	0.213009	0.326781	0.555048	0.356573	0.337942	0.291757	0.20315	0.38026	-0.07224	0.359408	-0.43628
Q9NWU5	MRPL22	-0.197	-0.41951	0.137436	0.661821	0.561421	0.151118	-0.46846	-0.08189	0.341214	0.124015	0.641435	0.227088	0.048588	0.154655	0.549437	-0.60072	-0.08549	0.128023	-0.49255
Q9NWV4	C1orf123	-0.01753	-0.28292	0.277598	-1.00527	1.444285	-0.88567	0.169203	-0.17264	0.728334	2.264129	-0.68118	-0.01871	0.057617	0.382121	-0.92907	-0.44631	-0.38809	-0.56919	-1.26854
Q9NWV8	BABAM1	-0.44582	-0.41544	0.367823	0.070832	0.24605	-0.31345	0.030886	0.373812	-0.19363	-0.03589	-0.25891	0.334542	-0.58375	-0.15343	-0.30289	0.632034	0.006574	-0.43261	-0.20643
Q9NWX6	THG1L	-0.18628	-0.16001	-0.09275	0.478269	-0.26114	-0.11863	-0.21746	-0.03025	-0.39716	-0.06624	0.504968	0.103019	-0.10763	0.379685	0.58865	0.035492	0.279208	0.132138	0.002037
Q9NWY4	HPF1	0.478222	-0.20031	-0.30905	-0.19707	0.307114	0.68403	-0.293	0.170017	-1.48775	-0.26135	0.16567	-0.29829	-0.72024	-0.21363	-0.49385	0.325967	0.086605	0.112173	0.050109
Q9NWZ3	IRAK4	-0.90962	0.653475	0.490235	-0.15471	0.025593	0.003788	0.530204	-0.49979	-0.15141	0.603863	-0.01709	0.495542	-0.2531	0.921975	-0.05008	-0.67438	0.107794	-0.20621	-0.13536
Q9NWZ5	UCKL1	-0.13257	-0.04487	0.345142	0.582941	-0.25717	0.322163	0.260006	0.09894	-0.10065	-0.38206	-0.1664	0.02354	-0.00296	0.260859	0.234209	0.199171	0.316215	-0.24268	-0.35649
Q9NWZ8	GEMIN8	-0.16612	-0.29602	-0.07217	-0.08697	-0.10783	-0.07835	-0.61042	0.040445	-0.70667	-0.18253	1.519835	-1.0251	-0.36679	-0.38418	0.08203	-0.11358	0.171736	0.821412	0.026533
Q9NX00	TMEM160	0.356042	-0.06836	-0.83321	1.150235	-0.57988	-0.15789	0.298197	-0.18112	-0.6022	-0.43686	-0.22457	0.319599	-0.33704	-0.01287	1.445716	-0.48714	-0.18804	-0.04289	-0.56558
Q9NX01	TXNL4B	0.430388	-0.22941	-0.42149	-0.10323	-0.18973	0.217401	0.403759	0.220037	-0.27281	-0.58837	0.71144	-0.21206	-0.20946	-0.31367	0.079325	-0.19068	0.438933	-1.03565	0.240893
Q9NX02	NLRP2	0.896466	0.077976	-1.85449	0.981898	-1.44212	-1.18913	1.79179	2.981839	-0.24564	-1.66745	-1.53649	0.559896	1.550489	-0.81872	-1.78956	-1.72067	1.070207	-0.30367	1.297386
Q9NX05	FAM120C	-0.3249	0.412745	-0.19425	-1.00032	-0.05741	-0.74799	-0.06987	-0.76916	0.00478	-0.99018	-0.10644	-0.29514	-0.26502	-0.25171	-0.45744	-0.43167	-0.41701	-0.48163	-0.87099
Q9NX08	COMMD8	0.236243	-0.02408	0.001053	0.199167	0.392811	0.287656	-0.12741	0.054032	0.049458	1.19956	-0.04799	0.348224	-0.10792	0.745357	-0.39671	0.771014	0.166479	0.118244	0.177405
Q9NX14	NDUFB11	0.344201	0.229157	0.560268	0.012277	0.343748	0.354124	0.197418	-0.34471	-0.54235	0.40558	0.329715	-0.27576	-0.02545	-0.17601	0.914285	-0.01196	-0.5566	0.042629	-0.4183
Q9NX18	SDHAF2	0.394065	-0.09013	0.169996	-0.59632	-0.16081	-0.29585	1.243918	-1.14932	0.246041	2.065436	-1.05657	0.03746	-0.14655	-0.73924	-0.17658	0.631007	-0.47079	-0.56485	-1.23987
Q9NX24	NHP2	0.603827	-0.49819	-0.20815	0.289104	0.238032	-0.38114	-0.53666	-0.40906	-0.36932	-0.27752	0.998087	-0.24377	-0.02057	-0.48807	-0.36242	-0.40471	0.665533	0.791175	-0.70583
Q9NX40	OCIAD1	0.362197	0.223584	0.937406	1.059714	-0.68378	-0.36266	0.11031	-0.6041	-0.07408	0.038178	0.702367	-0.61862	0.519748	-0.89763	0.433214	1.205733	-0.78577	-0.49164	-0.5615
Q9NX46	ADPRHL2	0.047493	-0.92208	0.14527	0.165451	0.553685	0.089057	-0.61741	1.425608	-0.0532	0.945556	-0.21018	-0.10776	0.047846	-0.16916	-0.26941	0.081377	0.160202	-0.33864	-0.13429
Q9NX47	MARCH5	-0.264	-0.06753	0.005649	0.129681	0.065601	0.59647	-0.15207	-0.0323	0.017578	-0.56978	0.294352	-0.22595	0.071994	0.094042	0.040631	0.134054	0.281598	-0.14123	0.412752
Q9NX55	HYPK	0.002873	-0.09338	-0.14846	-0.40573	0.466338	-0.27689	-0.43297	-0.13687	0.212632	1.056875	-0.15479	-0.18432	-0.26319	0.548325	-0.40593	0.418426	0.093332	0.108776	-0.05606
Q9NX57	RAB20	-0.39913	0.378223	-0.41875	-0.17153	-0.11406	-0.59196	0.049969	-0.66607	-0.01867	-0.43026	0.661255	1.044673	-0.15277	1.103428	-0.73965	-0.51866	1.166258	-0.14338	-0.81892
Q9NX58	LYAR	1.974383	0.517102	-0.03821	-0.4333	-0.04564	0.123998	-0.27776	0.057378	-0.23475	-0.26682	0.433997	-0.47705	0.6763	-0.57174	-0.42307	-0.47641	-0.49984	-0.15128	-1.18839
Q9NX62	IMPAD1	-0.43305	0.306915	0.486763	-0.11959	0.101554	0.386588	1.023771	-0.30572	0.206156	-0.93898	-0.35339	0.43097	1.210071	-0.50257	0.217552	0.386447	0.825632	1.804081	0.338879
Q9NX63	CHCHD3	0.528313	0.27225	0.31467	0.621151	-0.13953	-0.11204	0.033855	-0.20742	-0.52188	-0.07386	0.26386	-0.42369	-0.17301	-0.57587	1.306408	0.787816	-0.94799	-0.7866	-0.41672
Q9NX76	CMTM6	-0.01655	0.214137	-0.27687	-0.73309	0.030249	0.160228	0.299164	-0.55131	0.040847	-0.4061	-1.25963	0.67957	-0.09271	0.243198	-0.15211	-0.18946	0.039397	-0.10589	-0.35676
Q9NX78	TMEM260	0.030252	-0.26374	-0.16904	0.033115	-0.07949	-0.51551	0.054248	1.732265	-0.30622	-1.0599	0.513785	-0.21132	-0.21325	0.122668	0.039756	-0.08946	0.299955	-0.1808	0.321466
Q9NXA8	SIRT5	0.663023	0.517023	0.260487	0.255305	0.451426	-0.29965	0.181795	-0.84122	0.053299	0.335662	-0.09947	-0.08158	0.113693	0.191893	0.327469	0.124335	-0.0703	0.41335	-0.07446
Q9NXC2	GFOD1	-0.04217	-0.03399	-0.42918	0.285345	-0.51731	-0.42227	-0.18412	-0.019	-0.41951	-0.6717	0.72981	0.204408	0.054613	0.064184	0.318765	-0.51337	-1.21009	-0.34646	-0.42268
Q9NXC5	MIOS	-0.54758	-0.1902	-0.13393	-0.33247	-0.09471	0.213173	0.539887	-0.08121	0.581691	0.153762	0.676169	-0.29025	-0.68562	0.206574	0.480999	-0.25163	-0.14165	-0.30922	-0.33111
Q9NXE4	SMPD4	0.117285	-0.01765	-0.08577	-0.12136	-0.15293	-0.21837	0.276701	0.405771	-0.8353	-0.31554	0.153729	0.047104	-0.24526	0.064303	0.46945	-0.23735	0.312624	-0.06896	0.464261
Q9NXF1	TEX10	0.416293	-0.19513	0.155712	-0.07181	0.313307	0.398131	0.132248	1.051664	-0.77839	-0.23432	0.709317	-0.04104	0.176844	-0.18452	0.136944	0.128288	-0.46873	-0.06569	0.35023
Q9NXF7	DCAF16	1.40367	0.101565	-0.06393	0.025361	-0.54991	-0.74117	0.319344	-0.2054	0.557034	-1.36067	0.46907	-0.47569	-0.14955	-0.17299	-0.98776	0.503909	-0.35171	-0.20253	0.312187
Q9NXG2	THUMPD1	0.763383	-0.06946	-0.73155	0.066451	-0.60675	-0.5064	-0.67482	-0.41608	-0.65115	-0.62481	0.244864	-0.19952	0.130381	-0.12914	0.016914	-0.03823	0.270479	0.22549	-0.76897
Q9NXG6	P4HTM	0.714778	-0.10894	-1.21531	-0.20888	0.302674	0.414812	-0.352	0.221092	0.362503	0.216772	0.721808	0.097355	-0.17493	-0.06143	-0.14231	-0.04531	-0.40733	-0.17979	-0.32656
Q9NXH8	TOR4A	-0.62206	1.069277	0.175435	-0.02711	0.65901	0.544956	0.076542	-0.69121	0.234622	0.539953	0.297183	-0.14066	0.226635	0.291067	-0.40604	-0.53899	-0.72996	0.210225	0.496663
Q9NXH9	TRMT1	-0.24879	-0.50548	0.148327	-0.25698	-0.09004	-0.13575	-0.24356	0.690097	-0.19029	0.292156	0.175214	-0.36014	-0.20414	-0.05251	1.983182	1.039426	-0.16543	-0.59115	-0.93942
Q9NXJ5	PGPEP1	0.084957	0.280669	0.169709	0.980346	-0.0671	-1.48991	0.034226	-0.39535	0.040847	0.298971	0.02548	0.338151	0.313586	0.988725	0.61174	-0.16752	0.727817	0.342271	-0.08237
Q9NXK8	FBXL12	-0.00638	0.229116	0.162795	-0.13621	0.035233	-0.04556	0.119018	-0.05822	-0.0252	0.547671	0.347485	0.03901	-0.08296	0.210394	-0.34274	0.137354	-0.14976	0.315468	-0.71213
Q9NXN4	GDAP2	-0.50509	0.0889	0.173163	-0.56927	0.070475	-0.23262	0.245583	-0.10303	-0.08269	-0.48913	-0.2514	0.7065	-0.142	1.029808	-0.31032	-0.38669	0.537543	-0.63825	-0.24635
Q9NXR1	NDE1	0.155161	0.517992	0.798487	-0.66022	0.111098	-0.62384	0.264234	-0.20219	0.997536	0.130912	-0.36232	-0.41603	0.141364	-0.16509	-0.25628	0.170118	-0.53739	0.229639	-0.00333
Q9NXR7	BABAM2	-0.11415	0.163586	-0.06468	0.263037	0.253665	0.14217	-0.43107	0.370808	-0.15463	-0.6542	0.013881	0.083074	0.387982	-0.04672	0.242803	0.732253	-0.22451	-0.5759	-0.12056
Q9NXR8	ING3	0.468183	-0.07832	0.131186	-0.93025	-0.21523	0.156075	0.030752	0.084197	0.365743	0.016359	-0.32267	-0.12474	0.09579	-0.47108	-0.02486	-0.14884	-0.09513	-0.75754	0.330825
Q9NXS2	QPCTL	0.017171	0.431224	-0.63535	-0.1732	-0.5063	-0.24989	-0.15911	-0.66178	-0.34256	-0.87858	-0.67085	0.114215	-0.27266	0.21436	0.111777	-0.70516	-0.01805	0.105329	-0.53573
Q9NXU5	ARL15	-1.15608	0.276408	-0.0435	-0.37344	0.313334	-0.91061	0.710089	-0.88109	0.040847	0.038062	0.454766	0.033061	0.266653	0.163229	-0.28784	-0.32883	-0.04236	0.70799	-0.78689
Q9NXV6	CDKN2AIF	-0.1031	-0.34829	0.102151	0.223717	-0.49589	-0.47497	-0.20241	-0.0172	-0.41401	-0.06758	0.270423	-0.5447	-0.1104	-0.25872	-0.45073	0.086296	-0.00187	0.762134	-0.44212
Q9NXW2	DNAJB12	0.618532	0.309394	0.315802	-0.16696	0.342253	1.325468	-0.58559	0.524624	-0.15529	-0.14477	-0.0042	-1.54086	0.072164	-0.81428	-0.08265	-0.10776	0.340119	0.515176	-0.31541
Q9NXW9	ALKBH4	-0.16261	0.46415	0.446868	-0.23568	0.096463	0.114704	-0.13097	-0.11553	0.442999	0.45125	-0.17592	-0.20332	0.03396	0.341716	0.211866	0.178004	0.189851	-0.42588	-0.05429
Q9NXX6	NSMCE4A	0.262707	0.040477	-0.12181	0.010572	-0.16193	-0.31384	0.018935	0.026253	-0.19143	-0.12356	-0.15775	-0.36236	-0.27955	-0.11819	0.325349	0.380567	-0.51807	-0.05771	-0.33603
Q9NY12	GAR1	0.34453	-0.1782	-0.01602	0.04193	0.461097	-0.10831	-0.35865	0.045009	-0.4948	-0.19815	0.909416	0.117105	-0.01352	-0.35734	0.037331	-0.11437	0.427988	0.177045	-0.63646
Q9NY15	STAB1	-1.00093	1.02501	0.220965	-0.63927	0.961136	0.061965	0.664684	-1.23837	0.098308	1.094003	-0.63206	0.671432	0.028047	0.544846	0.014214	0.195642	-0.44749	0.163763	-1.21722
Q9NY27	PPP4R2	0.6773	-0.40532	-0.1285	-0.01448	-0.04927	0.107326	-1.17517	0.891383	-0.39267	-0.33344	0.178657	-1.0749	-0.09405	-0.09528	-0.20598	-0.39385	-0.10997	0.208467	0.064868
Q9NY33	DPP3	-0.26783	-0.03508	-0.05399	-0.09516	0.725864	0.026954	0.033669	0.775544	0.713453	1.431339	-0.63244	-0.15627	0.54379	0.151134	-0.17522	0.221733	0.00391	-0.49137	0.292447
Q9NY35	CLDND1	0.169876	-0.04735	0.077805	-0.15092	-0.04978	0.352694	-0.19929	-0.41498	0.283916	0.184845	0.510038	-0.18008	-0.03467	-0.04811	1.109382	0.669144	0.288006	-0.03371	0.077644
Q9NY47	CACNA2D	0.20952	0.154054	-0.55745	0.172045	-1.49749	-0.28852	1.481151	-0.53306	0.034556	-0.0037	0.486214	-1.05712	0.396566	-0.86728	0.99052	0.281366	-0.48722	-0.06159	-0.65507
Q9NY61	AATF	0.357491	-0.42053	-0.23825	0.663321	-0.34269	-0.1804	-0.23333	0.409286	0.139413	0.20855	0.285654	-0.15354	-0.42188	-0.42958	0.384985	0.391875	0.055708	-0.20731	-0.25011
Q9NY65	TUBA8	-0.69684	0.383759	-0.17074	0.086039	0.016132	0.407649	0.175288	0.334195	-0.05885	0.122651	-0.20162	0.20859	0.030959	0.177212	-0.01024	0.221602	0.095106	-0.12288	0.372492
Q9NY93	DDX56	0.308642	-0.01155	0.407717	0.456512	-0.19103	0.444919	-0.43136	0.881366	0.099192	-0.58439	0.588461	-0.32415	-0.12408	-0.85494	-0.24981	0.289061	-0.12085	0.259667	-0.44309
Q9NYB0	TERF2IP	-0.5578	0.50273	-0.33855	-0.28899	0.432876	0.282738	-0.38569	-0.59606	-0.02691	-0.17393	0.274125	-0.76265	-0.17373	-0.43871	0.129739	0.101769	-0.58536	0.447664	-0.32754
Q9NYB9	ABI2	0.796646	-0.60075	-0.44944	-1.47836	-0.728	-0.74819	-0.65304	0.133547	0.335917	-0.72514	0.111009	-0.678	0.016756	-1.04227	-0.3776	0.062593	-0.06556	-0.40433	0.582218
Q9NYF8	BCLAF1	0.935487	-0.83406	0.046913	0.466724	-0.44449	-0.14463	-0.64622	0.091154	-0.033	-0.23958	0.892339	-0.85403	-0.41757	-1.00171	0.727675	-0.4455	-0.13203	0.862539	-0.13405
Q9NYH9	UTP6	0.17841	-0.04153	-0.09913	0.518966	-0.22507	0.069498	-0.12322	0.30825	-0.21598	0.288713	0.782401	-0.34158	-0.60251	-0.36704	0.082708	-0.10731	-0.17698	-0.61595	0.069041
Q9NYI0	PSD3	-0.67864	0.114	0.226819	-0.45118	-0.43554	-0.83842	-0.08188	-0.12018	-0.05119	0.462248	0.005727	-0.39998	0.402138	0.074713	-0.4106	-0.52464	-0.07842	0.951909	0.69722
Q9NYJ8	TAB2	-0.31289	-0.28624	-0.03692	-0.6266	-0.63409	-0.46103	-0.1702	-0.09001	0.164348	0.164773	0.365785	-0.41413	0.026915	-1.13615	0.71521	-0.07483	-0.0995	-0.04439	-0.00032
Q9NYK5	MRPL39	-0.38012	0.12212	0.365379	1.105414	0.245035	0.455544	-0.11612	-0.4124	0.135316	0.522089	0.550519	0.086185	-0.06425	0.024598	0.847826	-0.36047	-0.03533	-0.26284	-0.59602
Q9NYL2	MAP3K20	-0.26234	-0.05998	0.222326	-0.21978	-0.07747	0.756995	-0.29942	-0.13864	-0.14273	0.065918	0.11723	0.125762	-0.08954	0.277564	1.198169	0.603409	0.430053	0.242636	-0.60888
Q9NYL4	FKBP11	-1.70736	1.27865	0.480749	-1.6581	0.425604	0.153845	2.660318	-0.6777	0.497047	-0.31278	-0.48225	-1.71112	1.14222	-1.79523	-1.4633	-0.14081	-1.56488	0.501067	0.838963
Q9NYL9	TMOD3	0.332711	-0.15248	-0.45332	-0.5646	0.135059	-0.44321	-0.62835	-0.76481	-0.02735	0.752118	-0.16458	0.27287	0.214347	0.344706	-1.09839	-0.27767	0.228605	0.02088	-0.12874
Q9NYM9	BET1L	-0.55392	0.623368	-0.65688	-0.44556	0.49739	0.568533	0.321975	0.130721	0.784779	0.716513	-0.13267	-1.36698	-0.02763	-0.55394	0.777068	0.595327	0.403564	1.205265	1.307174
Q9NYQ6	CELSR1	-0.01484	-1.31575	-0.65325	-0.13709	-0.81733	-0.64449	-0.75456	-0.34106	-0.655	-1.38201	0.784471	-0.3567	-0.19026	-0.21428	0.177996	-1.90423	-0.21438	-0.10741	-1.21094
Q9NYU1	UGGT2	0.40953	0.145306	-0.33812	-0.27544	-0.32865	-0.18723	0.228244	0.404546	0.129105	0.106805	0.170897	-0.39861	-0.21536	-0.56006	-0.26893	-0.50451	-0.48743	0.131925	0.960066
Q9NYU2	UGGT1	0.213844	0.526146	0.176471	-0.41254	-0.28053	-0.33791	0.452815	0.142104	0.060326	0.258417	0.089195	0.367906	0.083386	0.313287	0.08734	0.449341	-0.03915	-0.06495	0.143339
Q9NYV4	CDK12	0.230047	0.159843	-0.05085	0.304097	-0.59673	-0.16723	-0.28952	-0.13396	-0.28688	0.16953	0.050867	-0.82646	0.079329	0.045035	-0.42079	-0.1565	0.206574	0.244868	0.386135
Q9NYY8	FASTKD2	0.006449	0.129056	0.081648	-0.05874	0.353529	0.630262	-0.10649	-0.1673	-0.10007	0.327859	0.474978	0.170399	0.220944	-0.20761	0.828607	-0.00753	0.26228	0.426962	0.386111
Q9NZ01	TECR	-0.47191	-0.41126	0.281264	0.429503	0.182295	0.213733	1.098093	1.377519	-0.71422	-0.9321	0.019709	-0.06956	-0.94006	-0.08068	2.074199	0.712838	0.226347	-0.03651	-0.87056
Q9NZ08	ERAP1	-0.3689	1.345116	0.071	-0.10342	-0.28148	-0.50838	-0.06247	-0.5471	-0.0101	0.363827	-0.90724	-0.38707	-0.48287	-0.04853	-0.35934	0.137404	0.035888	0.818986	0.049074
Q9NZ09	UBAP1	0.024419	0.36426	-0.41673	-0.37382	-0.57586	-0.20901	0.524222	-0.18006	0.015563	0.804217	-0.74314	0.270805	0.205937	0.528013	-0.22653	0.727461	0.678841	0.230313	0.27679
Q9NZ32	ACTR10	0.041945	-0.03626	0.214577	-0.31526	0.202668	0.073673	-1.05374	0.066642	-0.39181	-0.75539	-0.70183	-0.18063	0.157429	0.022441	0.216816	-0.46555	-0.40633	-0.52567	0.029119
Q9NZ43	USE1	-0.84458	0.669549	0.553899	0.187818	-0.47947	-0.21946	1.019081	0.149324	0.801356	0.244178	0.056733	-0.15485	0.032995	-0.16056	0.106412	0.717122	-0.51914	-0.03527	0.324231
Q9NZ45	CISD1	0.689425	-0.73267	-0.11594	-0.50272	-0.72154	-1.02894	-0.56119	-0.80637	-0.59657	-2.37229	1.343331	-0.48529	-0.56859	-0.41167	0.251864	-0.71074	0.236193	-0.05647	-1.17785
Q9NZ52	GGA3	-0.20724	0.068392	0.14817	-0.69583	0.092791	-0.45385	-0.0338	0.610332	0.60828	0.480183	-0.05222	0.023613	0.433515	0.057805	-0.39567	0.378412	0.080604	0.020432	-0.30005

Q9NZ63	C9orf78	1.269336	-0.247	0.141799	-0.40362	-0.47564	-0.39873	-0.80061	0.086778	-0.74631	-0.4639	0.113002	-1.11113	-0.38627	0.276738	-0.34226	-0.22853	-0.67595	-0.10838	-0.20986
Q9NZA1	CLIC5	-0.64053	-2.15504	-2.21477	-0.14946	-1.12891	-1.19302	-2.08013	0.58703	-0.487	-2.40636	1.029155	0.531712	1.550234	-0.07862	-2.34682	0.527422	-1.31795	-2.90314	-0.68498
Q9NZB2	FAM120A	-0.89045	0.21818	0.042345	-0.2499	0.308597	0.401587	-0.44278	-0.28907	-0.52432	-0.35315	0.408719	-0.23051	-0.232	0.038292	0.156395	0.417321	-0.36098	-0.37238	-0.13023
Q9NZB8	MOCS1	0.694213	-0.22716	0.025244	0.312962	1.577007	0.090057	-0.43958	-0.61601	-0.06282	-0.07025	1.075507	0.348234	0.043683	-0.00134	0.467147	0.108458	0.339567	1.442699	-0.6339
Q9NZC3	GDE1	0.119882	0.684159	0.291431	-0.14259	0.146665	-0.25714	0.170982	-0.20131	-0.05434	0.313821	-0.33374	0.788715	0.287762	0.690083	-0.45975	0.284641	-0.21253	-0.39451	-0.1178
Q9NZC7	WWOX	-0.24336	-0.14776	-0.10136	-0.64619	0.788826	1.074564	-0.36955	-0.87426	-0.16427	-0.11263	1.012975	-0.51399	-0.06685	-0.14117	0.333706	0.623493	-0.35097	0.027285	-0.32707
Q9NZC9	SMARCAL	0.090873	-0.60312	0.159812	0.446237	0.359362	0.595956	-0.42954	-0.11607	0.055616	0.026308	0.369406	-0.05779	-0.00369	-0.03025	0.637685	0.468814	0.115378	0.003871	-0.06957
Q9NZD2	GLTP	-0.48675	0.443851	0.131997	-0.16063	-0.14155	-0.29073	0.039926	0.548605	0.086649	0.259273	-0.32755	-0.87682	0.389508	0.105655	-0.34962	0.152546	-0.38405	0.131016	0.042032
Q9NZD4	AHSP	-0.06896	-0.24526	-0.37626	-0.79466	-0.04164	-1.16645	0.078208	-1.02088	0.671165	-0.9233	0.206536	-1.44836	-0.2282	-1.76345	-0.97676	-1.25345	-0.88753	-0.46272	-1.11877
Q9NZD8	SPG21	-0.0529	0.672471	0.52778	-0.13383	0.261207	0.044531	-0.2011	0.17987	0.550108	0.38472	-0.55145	0.657847	-0.12352	1.041191	-0.11469	-0.34021	0.292154	-0.34073	-0.046
Q9NZI7	UBP1	1.023573	0.12767	-0.27919	-0.49109	0.189221	0.544752	-0.9209	-0.19556	-0.06059	-0.08324	-0.47146	0.054408	0.076917	0.157564	-0.07498	0.066773	0.024504	-0.29005	0.668916
Q9NZJ5	EIF2AK3	-0.06596	0.323908	-0.01212	-0.60443	0.17745	-0.23116	-0.1357	-0.26224	-0.30042	-0.27136	0.130421	0.283728	0.122936	0.631282	-0.16222	-0.26177	0.219455	0.024397	0.378528
Q9NZJ6	COQ3	0.499064	-0.27776	-0.58521	0.411387	0.10238	-0.18578	-0.92924	0.087184	-0.17216	0.986211	0.27469	-0.08153	0.10272	0.269237	2.633177	0.29618	0.200709	-0.05935	-0.6204
Q9NZJ7	MTCH1	0.675838	0.014177	-0.32524	-0.10639	0.300177	-0.10511	0.177713	-0.097	0.155553	-0.12134	0.520463	-0.02397	-0.3188	-0.12923	1.082945	0.067085	0.228924	-0.22956	-0.04409
Q9NZJ9	NUDT4	-0.48739	-0.37768	-0.18353	-0.62034	-0.27481	-1.66618	-0.10024	0.652659	0.501906	0.659246	-0.00517	-0.18707	0.073986	0.270662	-1.11725	-0.63854	0.859996	0.261521	0.849456
Q9NZK5	ADA2	-1.30784	1.235411	0.549443	-0.92266	0.677239	-0.13605	0.350946	-1.12336	-0.35224	1.353521	-1.65367	-0.39973	-0.05011	0.871623	-0.1123	-1.05257	-0.285	-0.03102	-1.19148
Q9NZL4	HSPBP1	0.089	0.019166	-0.67293	-0.55088	-0.19572	0.100152	0.588075	0.571389	0.300081	-0.32325	0.23986	-0.07455	-0.3012	-0.93199	-0.33788	-0.51276	0.199048	-0.90993	-0.39478
Q9NZL9	MAT2B	0.38931	-0.94341	0.8227	0.751428	1.435285	0.980749	-0.46824	0.29879	0.320672	0.425264	0.68102	-0.1813	0.438145	-0.08193	0.403179	0.354357	0.028013	-0.10844	0.102514
Q9NZM1	MYOF	-0.8193	0.238572	-1.29816	0.290848	-0.78135	-1.00367	-0.77826	-1.68025	0.243607	-0.53425	0.22891	0.768576	0.79896	0.279654	-0.41502	0.239937	0.421666	0.637734	0.188089
Q9NZM3	ITSN2	-0.88882	0.712659	0.471594	-0.22258	0.107699	0.108756	0.40145	-0.02451	-0.00432	0.702231	-0.36805	0.436521	0.120253	0.642766	0.36764	0.582626	-0.14762	0.105472	-0.60074
Q9NZM4	BICRA	0.377148	-0.2877	-0.17049	-0.04635	-0.53763	-0.18305	-0.07448	0.495553	-0.29119	-0.18679	-0.79028	-0.23967	-0.18695	-0.09408	0.118906	-0.10559	0.149288	-0.24079	-0.04791
Q9NZM5	NOP53	0.329162	-0.18218	-1.07939	0.298059	0.032174	-0.04891	-0.71142	-0.43324	-0.04357	-0.40499	0.831125	0.123873	0.251503	-0.47158	0.010199	0.108824	-1.01458	0.550328	-0.49618
Q9NZN3	EHD3	-0.975	-0.12838	0.034523	-0.4298	-0.50406	-0.89964	-0.44502	-0.53107	-0.36201	-0.42289	-0.09013	-0.38463	-0.8204	-0.05735	-0.33451	-0.55424	-0.22408	0.1642	-0.01052
Q9NZN4	EHD2	-1.95357	-0.5043	-0.68054	-2.53583	-1.15628	-1.87763	0.140865	-2.08297	-0.16341	-1.56464	-1.42129	-1.47464	-0.91772	-0.95756	-0.17335	-1.17298	-1.09709	0.233478	-1.10842
Q9NZN5	ARHGEF1	0.110001	-0.29079	-0.36085	-0.17274	-0.77856	-0.63783	0.077377	0.278145	0.268212	-0.30397	0.229234	-0.84612	0.161895	-0.60103	0.539929	-0.36335	-0.46361	-0.49457	-0.16879
Q9NZN8	CNOT2	0.454793	-0.0431	0.19412	-0.18596	-0.14747	-0.11372	-0.12184	0.005931	0.306986	-0.22313	0.315037	-0.619	-0.07289	-0.68474	0.39128	0.240073	-0.24287	0.177464	-0.02636
Q9NZP8	C1RL	-0.28358	0.005549	-0.57628	-0.47688	-0.22269	-1.39358	0.249153	0.647608	0.483023	0.919547	-0.21855	0.396549	-0.07155	0.33322	-1.30829	-1.0009	-0.42706	-0.48889	-1.06777
Q9NZQ3	NCKIPSD	0.857831	0.129383	-0.37494	-0.38146	0.102746	0.02382	-0.15193	0.1183	-0.39135	-0.39259	0.362404	0.209448	0.159655	0.351391	-0.13873	-0.20818	-0.20803	-0.28404	-0.30229
Q9NZR1	TMOD2	-0.54028	-0.02427	-0.52467	-0.49223	0.316726	-0.88461	0.237826	-1.00046	0.410374	1.518267	0.046552	-0.3363	0.563651	0.109395	-0.34266	-0.80851	-0.52401	-0.15584	-0.74094
Q9NZT1	CALML5	-1.49067	-0.00969	0.360985	-1.26814	2.023025	0.89097	0.896669	-0.40402	1.947386	1.165756	-1.36243	0.098016	0.987066	-0.04501	-1.07802	-0.62028	-0.43337	-1.21364	-0.79238
Q9NZT2	OGFR	-0.00071	0.098997	0.735005	-0.07147	0.642109	0.708627	-0.59618	0.387244	-0.04297	0.424339	-0.06912	0.56617	-0.12728	0.807386	0.57147	0.263269	0.948447	0.020961	-0.63523
Q9NZU0	FLRT3	1.229362	0.379431	0.9052	-0.99295	-0.14657	-0.66838	0.868548	-0.16944	0.040847	-1.83694	0.109444	0.190969	0.493515	-0.17159	-0.83369	0.683382	-0.0517	-0.33697	-0.35488
Q9NZU5	LMCD1	-0.59236	0.108629	-0.19883	-1.03552	-0.02784	-0.45651	-0.57663	-0.24402	0.303728	-0.29333	-1.34179	-0.99219	0.803328	-1.21834	-0.37827	-0.10918	-0.90633	-0.36616	1.233046
Q9NZV5	SELENON	-0.04214	0.415579	0.317172	-1.15611	0.264548	0.031101	0.287946	-0.18097	0.38211	0.527343	-0.11701	-0.21392	0.024842	-0.31418	-0.32102	-0.3968	-0.92811	0.505472	0.353332
Q9NZW5	MPP6	0.790338	-0.33704	-0.56771	-0.76977	-0.75128	-0.7567	-0.82851	0.058009	-0.04705	-0.27364	1.723906	-0.85633	-0.39039	-0.80643	-0.57977	-0.91576	-0.43906	0.623136	0.162795
Q9NZZ3	CHMP5	0.136566	0.013628	0.145788	0.219348	-0.34954	0.090591	-0.1261	-0.30011	-0.06794	-0.22917	-0.11618	-0.21183	-0.03515	0.097693	-0.28285	0.382876	0.154223	-0.20781	-0.16772
Q9P000	COMMD9	0.076238	0.526545	0.96159	-0.52679	-0.20888	-0.40481	0.215859	-0.22331	-0.2009	0.593775	-0.17704	0.10014	-0.01351	0.39078	-0.38697	0.030198	-0.34736	-0.29336	0.042555
Q9P003	CNIH4	-0.2532	0.391947	0.290764	-0.10147	0.233804	-0.0751	0.038931	0.430732	0.191963	0.366043	-0.16693	0.435464	0.123629	0.872153	0.175453	-0.08727	0.34376	-0.71693	-0.02289
Q9P013	CWC15	0.962202	-0.93184	0.543922	0.303469	-0.02701	0.879262	-0.9737	0.934033	-0.15567	-1.07759	0.685813	-0.93708	0.522218	-0.86526	0.270729	0.582051	-0.50441	0.565046	-0.33629
Q9P015	MRPL15	-0.36124	-0.09719	0.162065	0.638156	0.490933	0.399939	-0.32212	-0.5498	0.208734	-0.01121	0.428894	0.309365	0.899458	-0.27372	0.819948	-0.26772	-0.19016	-0.32752	-0.57563
Q9P016	THYN1	0.567728	-1.01747	-0.74724	0.137305	-0.15363	-0.52165	-0.9311	-0.30098	-0.35797	-0.53255	0.797213	0.07944	0.684548	0.13125	0.027497	-0.33531	-0.8945	-0.52303	-0.78407
Q9P032	NDUFAF4	-0.16821	-0.50779	0.126385	0.514379	0.071056	-0.04683	-0.17655	-0.86666	-0.33634	0.292399	0.483816	-0.33276	-0.65143	-0.1769	1.433539	1.013637	-0.41459	-0.10353	-0.82829
Q9P035	HACD3	0.280111	-0.00351	-0.45005	0.365189	-0.00687	0.14367	0.037248	0.19159	-0.54332	-0.60653	0.571635	-0.11526	-0.25531	-0.18518	-0.28141	-0.12873	0.976371	0.297221	0.061645
Q9P086	MED11	0.52284	-0.36145	0.210113	-0.24682	0.300379	-0.04692	-0.00924	0.526706	-0.02324	-0.02138	0.325526	0.171342	-0.24556	-0.37775	-0.19432	-0.04305	-0.00429	0.048838	0.004514
Q9P0B6	CCDC167	0.03738	0.363784	0.449602	-0.44634	-0.11876	0.09875	0.384331	-0.84984	-0.01135	0.012585	-0.70573	0.446873	0.050794	-0.7671	0.202963	0.180469	0.79478	0.95053	-0.23212
Q9P0I2	EMC3	-0.05986	0.624031	0.000211	0.023786	0.142443	0.261853	0.07625	0.126149	0.582114	-0.66696	0.471849	0.177138	0.643481	0.373494	0.0122	-0.10709	0.045975	0.009542	-0.28768
Q9P0J0	NDUFA13	-0.20915	0.272329	0.149129	-0.12202	-0.05004	0.391161	0.41919	-0.40597	-0.43267	-0.8124	0.58917	-0.20017	0.342448	-0.07315	0.807031	-0.45846	-0.62054	-0.03854	-0.53529
Q9P0J1	PDP1	-0.56031	-0.17311	-0.27384	-0.38099	-0.06059	-0.38431	-0.53061	-0.01452	0.729839	0.46435	0.261475	0.333255	0.151932	0.019876	1.407989	1.000322	-0.5436	-0.36366	-0.12808
Q9P0J7	KCMF1	0.160891	0.312147	0.00469	-0.47738	-0.12137	-0.26634	-0.0033	-0.20313	0.469506	0.509178	-0.13489	-0.08341	-0.15569	-0.38354	0.242618	0.631929	-0.41229	-0.48083	-0.02667
Q9P0K7	RAI14	-0.78519	0.427253	-0.1353	-0.54639	-0.72637	-0.55928	-0.19644	-0.28128	0.292654	-0.4455	0.270833	-1.26204	0.074033	-1.25916	-0.66519	0.091126	-0.41019	0.588758	1.052453
Q9P0L0	VAPA	-0.00449	0.43095	0.162907	0.262066	0.516746	0.427105	0.316759	0.054757	-0.17854	0.670745	0.359089	-0.36118	0.02996	-0.39622	0.537637	0.088452	0.15614	0.421632	-0.18769
Q9P0M6	H2AFY2	-0.00287	-0.40877	-0.92341	-0.12479	-0.4217	-0.70829	-0.60197	0.555319	-0.92931	-0.87788	0.816762	-0.46564	-1.06266	-0.5461	0.14529	-0.66273	-0.81712	-1.27145	-0.27303
Q9P0M9	MRPL27	-0.08878	0.251203	0.425892	-0.34062	0.732994	0.579583	0.184536	-0.58787	-0.0552	0.184841	0.19409	0.401454	0.418839	0.006289	0.191612	0.090436	0.052329	-0.23916	-0.8193
Q9P0N9	TBC1D7	0.181918	-0.0241	0.070845	-0.07991	0.1681	0.413515	0.024064	-0.12988	-0.00563	0.028541	0.0783	0.28532	-0.03818	0.334264	-0.11627	0.101558	0.402557	1.108314	-0.05416
Q9P0P0	RNF181	-0.23966	0.199688	-0.50157	0.012399	0.401134	-0.20574	-0.88907	0.018338	-0.13463	0.069134	-0.44869	-0.26419	-0.44052	0.098034	-0.21842	-0.01641	0.815779	0.575479	-0.17971
Q9P0P8	C6orf203	-0.31454	0.20222	0.309285	0.85959	0.668081	-0.17145	0.184718	-1.02418	-0.04685	0.080267	0.887868	0.016471	0.244649	0.737879	0.445064	-0.04405	-0.5423	0.268564	-1.30278
Q9P0T7	TMEM9	0.55877	-0.13841	-0.26641	-0.49178	0.248865	0.246352	0.561896	0.263862	-0.16862	-0.20206	-0.03906	-0.83193	-0.16477	-0.37044	0.142177	-0.22765	-0.7006	-0.05956	0.294327
Q9P0U3	SENP1	0.045157	0.353802	-0.11606	0.379535	0.10354	-0.42464	-0.19739	-0.02911	-0.29729	-0.84964	-0.35302	-0.12962	0.343412	-0.34506	1.001986	-1.06318	-0.17316	-0.35336	0.103964
Q9P0U4	CXXC1	0.572236	-0.04501	0.248447	-0.16146	0.149385	0.229348	-0.01354	-0.06232	-0.33693	-0.46863	-0.17102	-0.01394	-0.3081	-0.38904	0.595535	-0.65853	-0.99057	-0.27687	-0.33221
Q9P0V3	SH3BP4	0.181157	0.120414	-0.38399	-0.26406	-0.73176	-1.1703	-0.00586	0.97125	0.105874	-0.12103	0.085495	-0.44562	0.462438	-0.73323	-0.68114	0.05252	-0.32003	-0.12441	0.482437
Q9P0V9	SEPT10	1.059615	-0.45529	0.10584	-0.96587	-0.22563	-0.46818	-1.04422	0.186631	-0.20747	-0.86286	0.426079	-0.52388	-1.21818	-0.30621	0.246294	0.96353	0.357371	-0.7254	-0.51909
Q9P0W2	HMG20B	0.876084	-0.40472	-0.29331	0.186844	-0.48366	-0.47713	-0.42622	0.662035	-0.02871	-0.27388	0.053359	-1.11148	-0.1999	-0.53071	-0.15783	-0.38509	-0.52766	-0.09385	-0.15835
Q9P107	GMIP	-0.84586	0.603625	0.844118	-0.56263	0.025786	-0.23296	0.325075	-0.43794	0.376968	0.734201	-0.87265	1.000863	-0.50174	1.648776	-0.76318	-0.52103	0.565737	-0.00632	-0.89631
Q9P1U1	ACTR3B	0.428245	0.550248	0.028542	0.557768	-0.62967	-0.73141	0.383662	0.090395	0.265377	0.60274	0.864223	-0.53916	0.569617	-0.31842	0.895894	0.151725	-0.67883	0.041261	-0.69868
Q9P1Y5	CAMSAP3	0.271039	-0.64704	-0.02584	-0.73798	-0.53827	-0.24729	0.04286	0.024847	0.264957	0.19028	0.018302	-0.34347	-0.36239	-0.76864	-0.27503	0.625614	0.303728	-0.45834	-0.9538
Q9P1Y6	PHRF1	-0.1644	-0.20288	0.202041	-0.34654	0.110512	0.507272	-0.17068	1.083274	0.315188	-0.20336	0.052371	-0.21174	0.185114	0.10168	0.348171	0.569929	0.790844	1.148727	-0.08226
Q9P1Z2	CALCOCC	-0.68116	0.080747	0.487165	0.423887	0.162305	-0.15831	-0.0729	0.042135	0.318386	0.337855	-0.26863	-0.15508	0.567077	-0.13904	0.099215	-0.19264	-0.36952	-0.126	-0.12978
Q9P206	KIAA1522	0.265853	-0.66552	-0.39379	0.106635	-0.55875	-0.13208	0.156779	0.671637	-0.09625	-0.19381	0.658041	-1.15283	-0.37497	-1.05043	0.630534	1.049769	-0.02667	-0.09543	-0.09666
Q9P215	POGK	0.643668	-0.0226	-0.24064	0.240636	-0.54954	-0.56182	0.257609	0.82392	-0.14549	0.3989	0.080672	0.027256	-0.12825	0.129786	1.506774	-0.67323	-0.38541	-0.46167	0.540665
Q9P219	CCDC88C	0.09602	0.016385	-0.33519	-0.47171	-0.84173	-0.61843	0.095956	0.529906	-0.3535	-0.43498	1.065265	-0.47858	0.212589	-0.23124	-0.7505	-0.97014	-0.46911	-0.66513	-0.38043
Q9P246	STIM2	-0.25886	0.339982	0.260546	-0.40807	-0.12375	-0.72366	-0.03051	-0.13627	0.189011	0.018566	-0.41499	-0.53719	0.087298	0.026619	-0.37367	-0.44753	-0.27093	0.535723	0.849855
Q9P253	VPS18	-0.12638	0.625667	0.383605	0.352059	-0.084	-0.14171	0.390788	-0.13536	-0.16172	0.288375	-0.1234	0.514512	-0.1075	0.636004	-0.19426	-0.26885	-0.07496	0.012192	0.075363
Q9P258	RCC2	0.968999	-0.5641	0.196557	-0.46978	-0.09014	0.145933	-0.35409	1.222636	-0.29732	0.360515	0.06855	-0.67485	-0.24398	-0.71199	-0.44654	-0.25186	-0.52387	-0.38359	-0.3642
Q9P260	KIAA1468	-0.2876	-0.06302	0.144877	-0.04678	-0.61739	-0.43351	0.04314	-0.53311	0.183865	0.233803	-0.05761	-0.29808	-0.24146	-0.13279	-0.03537	0.685706	-0.24191	-1.00517	-0.22491
Q9P265	DIP2B	0.063591	0.398217	-0.67595	0.034431	-0.02059	-0.12132	0.236843	0.326929	0.268295	-0.02469	-0.469	0.012838	-0.15864	0.182497	0.096842	-0.16851	0.317059	0.419977	0.290177
Q9P266	JCAD	-0.85217	0.40891	0.201102	-0.99221	-0.88187	-0.47512	0.193484	-0.73601	0.472848	1.202216	-0.46113	-1.53163	-0.12554	-0.77627	-0.46696	-0.0161	-0.76525	0.632107	0.527308
Q9P270	SLAIN2	0.100321	-0.80918	-0.19573	0.047797	-0.69366	-0.67538	-0.333	-0.20793	0.20457	-0.06872	-0.58361	-0.57042	0.769692	-0.47062	0.152944	0.484687	-0.3946	0.085727	0.547775
Q9P273	TENM3	-1.37485	0.509305	0.37544	-0.32198	-0.73745	-0.66533	0.267983	0.875326	0.677508	0.573719	-1.16983	-0.6969	0.527863	-0.29524	-0.38998	0.707854	-0.57793	0.758573	2.260106
Q9P275	USP36	0.769051	-0.08444	-0.21921	0.327739	-0.42459	-0.04333	-0.15453	0.627777	-0.42328	-0.34541	0.11095	-0.37821	-0.04575	-1.03751	-0.2958	-0.20851	-0.59444	-0.08995	-0.58485
Q9P287	BCCIP	0.106616	0.317549	0.319724	0.193733	0.599241	0.38665	0.036035	0.208247	0.010279	0.071566	-0.50447	0.573074	0.215768	0.228218	0.129904	-0.26039	0.205184	-0.14274	0.223793
Q9P289	STK26	-0.67581	-0.16846	0.143043	-0.29431	0.404225	1.129875	-0.25818	0.211378	0.06106	0.456597	-0.46815	0.267093	-0.12542	1.389007	-1.52781	-0.15182	0.629127	0.272562	-0.34545
Q9P291	ARMCX1	-0.10722	-0.14494	0.81953	-0.3183	0.514255	0.056552	0.47447	0.724797	0.307638	-0.03548	-0.23803	-1.19743	-0.3788	-1.09577	0.780766	-0.23626	-0.60874	0.00389	0.476637
Q9P299	COPZ2	-1.03853	0.496925	0.088777	-0.97798	-0.07337	0.218109	-0.18189	-0.70582	0.863276	-0.20043	-1.29754	-1.26244	0.635142	-0.77147	-0.8312	0.751207	-1.14308	0.169189	2.015604
Q9P2A4	ABI3	-0.92764	1.406283	-0.20467	-0.41055	0.856433	0.981585	0.017946	-1.07899	-0.02949	0.387294	-0.68877	0.746405	-0.72455	0.297817	-0.35889	-0.1638	-0.49539	0.43647	-0.68973
Q9P2B2	PTGFRN	-0.76723	0.087907	-0.49524	-0.58682	-0.88687	-1.10486	0.509253	-0.87842	-0.13595	-1.20418	0.340724	-1.78079	-0.79986	-1.28156	0.034306	-1.13952	-1.35878	0.182756	0.99219
Q9P2B4	CTTNBP2I	-0.45055	-0.22465	0.123949	-0.35783	-0.35997	-0.2516	-0.38873	0.261817	0.12945	-0.21979	0.262935	-0.43423	0.196369	-0.31914	-0.36787	-0.0201	-0.33638	-0.10922	0.580639
Q9P2C4	TMEM181	-0.51032	0.267349	0.144113	-0.24247	-0.10929	0.046226	0.15552	-0.67049	0.388359	-0.08406	-0.15967	-0.92001	0.233103	-0.22235	0.059978	0.264364	-0.28557	-0.13827	0.616141
Q9P2D0	IBTK	0.429339	0.603317	0.174534	-0.80189	-0.26068	-0.25232	0.468924	-0.55106	0.101773	-0.24139	0.362662	0.391921	0.048661	0.268011	-0.04055	0.461802	-0.04197	1.189287	-0.16655
Q9P2D1	CHD7	0.801752	0.198828	-0.84418	-1.29021	-0.19521	0.701578	-0.15185	0.302119	-0.25975	-0.21304	0.079815	-0.37287	0.076829	-0.32377	-0.73513	-0.98832	0.019066	-0.0777	0.862603
Q9P2D3	HEATR5B	-0.07592	0.220262	0.35621	0.068069	0.039576	-0.04416	0.404178	0.019805	0.374267	0.359676	0.169986	-0.00543	-0.56037	0.470145	0.391023	-0.0373	-0.3558	-0.86184	-0.68177
Q9P2E3	ZNFX1	-0.3227	0.237208	-0.10524	-0.39959	-0.0533	0.206994	0.027266	-0.37108	-0.03756	-0.19561	-0.2668	0.024664	0.053244	0.516848	0.321017	-0.3103	0.152697	0.1423	-0.0257
Q9P2E5	CHPF2	-0.36569	-0.15407	0.032492	1.142215	-0.06523	-0.23165	0.387097	0.498916	0.144657	0.719942	-0.3705	-0.37085	-0.17034	-0.35009	0.876809	0.069604	-0.35315	0.334181	0.714877
Q9P2E9	RRBP1	-0.55679	0.119873	-0.02587	-0.07162	-1.18215	-0.69069	0.457452	-0.21828	0.141556	0.190779	-0.46063	-1.7893	0.803101	-1.43425	0.546591	0.2878	-1.08326	0.900987	1.665633
Q9P2G1	ANKIB1	-0.05703	0.25998	0.30797	-0.27595	-0.05899	0.127477	0.079143	-0.00055	-0.19132	0.314066	-0.41607	0.084936	0.009848	0.324482	-0.18513	0.251509	0.179784	-0.38787	0.394827
Q9P2G3	KLHL14	0.146239	-0.09893	-0.01049	0.324241	0.789908	0.972774	0.070962	0.934344	-0.45801	-0.39744	0.040918	-0.54859	0.049295	-0.14632	0.251554	0.114547	0.034446	-0.85151	-0.77179
Q9P2H3	IFT80	-0.1287	-0.16492	-0.53565	-0.36942	-0.27152	-0.28801	-0.24632	-0.07466	0.040847	-1.11523	2.154847	-0.78122	-0.20041	-0.45904	-0.06957	0.428623	-0.51081	-0.74915	0.217557
Q9P2I0	CPSF2	0.30062	0.221265	0.176631	-0.04642	0.334968	0.283135	-0.03899	0.264954	0.022067	-0.22883	0.588944	-0.13297	0.073146	-0.15176	0.033899	0.217746	-0.03284	0.216902	-0.04255
Q9P2J2	IGSF9	0.509928	-0.79506	-0.41811	-0.72665	-2.88304	-2.02208	-0.15658	1.380337	-0.31773	-0.27625	0.114872	-1.50811	-0.40597	-0.62524	1.112899	-1.72862	-0.685	-2.96043	-0.68674
Q9P2J5	LARS	-0.47191	0.005158	-0.08293	0.466433	0.134653	0.308239	-0.04184	-0.04975	-0.0891	-0.35989	0.296921	-0.04562	-0.1957	0.001508	0.386198	0.912815	-0.39709	-0.41575	0.004827
Q9P2K3	RCOR3	0.046214	-0.25284	-0.53576	-0.55464	-0.79624	-0.46607	0.359153	0.968995	0.511713	-0.19847	0.154474	-1.06159	0.086669	-1.59654	0.92102	-0.33161	-0.65883	-0.41881	-0.58421
Q9P2K5	MYEF2	1.849912	-1.03784	-0.95058	0.187769	-0.43212	-0.24948	-0.12109	-0.08696	0.090981	-0.41878	0.385874	-0.65065	-0.53886	-0.87874	0.249913	-0.10391	-0.59827	-1.0563	0.014535
Q9P2K6	KLHL42	0.341641	-0.03703	0.318831	-0.36716	-0.04812	0.223126	-0.16447	0.552932	-0.24294	-0.35976	-0.31741	-0.29529	-0.06743	0.051722	-0.75328	-0.17469	0.323496	-0.57069	-0.22927
Q9P2K8	EIF2AK4	0.283345	0.403284	-0.27704	0.581302	-0.27178	-0.36959	0.605041	-0.61259	0.843297	-0.20724	0.106334	-0.11374	0.360666	-0.40023	-0.46212	0.870473	0.231502	-0.11391	0.445561
Q9P2L0	WDR35	0.017527	-0.79421	-0.55003	-0.26423	-0.91143	-0.63606	-0.69672	-0.52529	-0.59425	-1.33323	1.478887	-0.68651	-0.6277	-0.00383	-0.27386	-1.22626	-0.54328	-1.11544	0.226421
Q9P2M7	CGN	0.607837	-0.94526	0.233041	0.741181	-0.58195	0.020388	-0.57603	0.327149	-0.49139	-0.59476	-0.43957	-0.60164	0.948853	-1.06137	1.661728	0.674398	0.906907	1.886429	-0.60732
Q9P2N5	RBM27	0.195688	-0.379	-0.07923	0.289229	0.042974	0.727015	-0.73807	0.249515	-0.27852	0.101656	0.334523	-0.31733	-0.4213	-0.51249	0.910384	0.348703	0.076128	0.202302	-0.44482
Q9P2P1	NYNRIN	0.694925	-0.25275	-0.10014	-0.03289	0.261253	0.738472	0.513232	-0.03415	-0.24457	-0.55098	-0.30396	-0.98089	-0.50968	-0.29252	-0.87235	-0.35814	-0.78738	-0.86718	0.555608
Q9P2P6	STARD9	-0.05433	-0.26792	-0.0561	0.088661	0.804772	0.181757	-0.31127	0.004745	0.008975	-0.17266	0.789895	0.06186	0.470488	-0.0764	0.259636	-0.17738	0.060193	0.129455	0.545693
Q9P2R3	ANKFY1	0.899367	0.853471	0.792691	-0.65213	-0.02703	-0.39985	0.442621	-0.42825	-0.08399	0.242105	0.151996	0.415386	0.261967	0.520308	-0.59799	-0.26657	-0.60443	0.116765	0.399166
Q9P2R6	RERE	0.639574	-0.08129	0.056131	0.010296	-0.41178	0.162906	-0.18133	0.368746	-0.06886	-0.07919	0.166593	-0.47233	-0.28499	0.019994	-0.1161	0.157966	0.577882	0.059909	0.616783
Q9P2R7	SUCLA2	-0.23851	-0.60295	-0.26488	-0.17434	-0.01178	-0.32539	-0.8523	0.18907	-0.36643	0.757578	0.660777	0.190693	0.024382	0.057136	1.533148	1.054846	0.472983	0.839685	0.300001
Q9P2T1	GMPR2	-0.07715	-0.46272	0.416854	-0.31868	0.438672	-0.12627	-0.18532	-0.16304	-0.12485	0.22481	0.766902	-0.48514	-0.63643	0.012314	-0.20926	0.766591	-0.55489	-0.37712	-0.39291
Q9P2W9	STX18	-0.21799	0.774879	-0.08759	0.421855	0.012288	-0.10666	0.113401	-0.08861	-0.09167	0.237009	0.349885	-0.28741	-0.58203	-0.37489	0.351454	0.376629	-0.3005	0.551665	0.399956
Q9P2X3	IMPACT	-0.83972	0.020182	-0.43128	0.633144	0.142716	-0.15033	-0.85326	0.033751	-0.32412	0.137491	0.531275	-0.2445	0.042113	-0.01724	0.173362	0.807353	-0.79182	-0.02	0.189719
Q9P2Y4	ZNF219	1.396876	-0.93435	-0.66906	-0.72224	-0.72245	-0.52899	0.936446	1.01572	0.561287	-0.14212	-1.37498	-0.85813	0.057715	-0.22319	0.339965	-0.08852	-1.67249	-2.63836	0.902366
Q9P2Y5	UVRAG	-0.36796	0.685467	0.317007	-0.07698	0.188682	0.413943	0.013427	-0.67732	0.094565	0.321101	-0.43244	0.45804	0.478239	0.314318	-0.41888	1.009202	-0.29426	-0.4135	-0.64888
Q9UBB4	ATXN10	0.332717	-0.29186	0.267183	0.016361	0.231944	0.419146	-0.37814	-0.30722	0.013875	0.275188	0.360362	-0.82163	-0.28799	-0.50027	-0.55665	0.158727	1.11449	0.784662	0.209468
Q9UBB5	MBD2	0.046797	-0.43177	-0.12488	0.184419	-0.107	-0.18279	-0.23241	-0.58416	-0.37819	0.07212	0.457267	-0.3201	-0.39485	-0.42888	0.93065	0.195762	-0.08805	0.794212	-0.44181
Q9UBB6	NCDN	0.028967	0.435336	0.376129	-0.30441	0.011269	0.28395	0.022936	-0.55798	0.49486	0.212928	-0.14538	-0.63628	0.073023	-0.11529	-0.24837	0.077195	0.007902	0.013606	0.554735
Q9UBB9	TFIP11	0.548461	-0.04811	0.433088	-0.1789	-0.05445	0.246894	-0.27081	0.382251	0.10511	-0.14018	0.202434	-0.24298	-0.86945	-0.14952	0.24952	0.066792	0.20331	0.360615	-0.12953
Q9UBC2	EPS15L1	-0.65182	0.294842	0.322897	-0.17546	-0.4466	-0.56929	0.302063	0.501234	0.011391	-0.06437	-0.21811	-0.23787	-0.28233	-0.16823	-0.24724	0.376947	-0.20907	0.346857	-0.03939
Q9UBD5	ORC3	0.685641	-0.0888	-0.03154	-0.34878	0.02468	0.063797	-0.09976	0.206707	-0.53545	-0.18434	0.579279	-0.0188	-0.02875	-0.21305	0.76625	0.358716	-0.16871	-0.42933	-0.28351
Q9UBE0	SAE1	0.872876	-0.81201	-0.54	-0.02635	0.589672	0.598065	-0.7533	1.643732	-0.07247	-0.1177	-0.17025	0.010169	0.496324	-0.03628	0.062901	-0.52525	0.113261	-0.08789	0.074677

Q9UBF2	COPG2	0.008613	0.223775	0.278813	0.76304	-0.25522	-0.11405	0.236006	0.504305	-0.56761	-0.91839	0.225149	-0.15894	-0.14941	0.193837	0.708228	0.570449	-0.35681	-0.63577	-0.06949
Q9UBF8	PI4KB	-0.28072	0.172636	0.225017	-0.35774	0.072677	-0.31663	-0.03813	0.038669	0.180864	0.187874	-0.31014	-0.45243	0.124207	-0.61451	0.47125	-0.25309	0.195523	0.005847	0.085411
Q9UBG0	MRC2	-1.31245	1.262608	0.576864	-1.19795	-0.12221	-0.02297	0.422625	-0.54672	0.836155	0.140531	-0.67459	-0.76583	1.043977	-0.89262	-0.91556	0.148511	-0.55444	0.79461	1.921145
Q9UBH6	XPR1	0.18228	-0.02128	-0.72022	0.617879	-0.966	0.076343	-0.05893	-0.79374	-0.36293	-0.81691	-0.03486	-0.74709	-0.2108	-0.45573	0.205272	0.821474	-1.13884	-1.33492	-0.90173
Q9UBI1	COMMD3	-0.25311	0.682496	0.745879	0.499151	0.118772	0.635098	0.259121	0.477851	0.479457	0.068074	0.079861	0.256328	-0.27013	0.85014	-0.07898	-0.3373	-0.29418	-0.05664	0.610843
Q9UBI6	GNG12	0.331131	0.070935	-0.67814	-0.16977	0.120172	-0.55433	0.140587	-0.73193	0.382625	-0.39769	0.507003	-0.8163	0.058732	-0.93046	0.311728	-0.75623	-0.84738	-0.05153	0.054783
Q9UBK8	MTRR	-0.17662	0.165251	0.274952	-0.17527	-0.1162	0.128312	0.031619	-0.42161	0.047173	1.0296	0.262316	-0.08842	0.040963	0.878422	-0.47186	-1.12112	0.844151	0.242017	0.035831
Q9UBK9	UXT	-0.02516	-0.39744	-0.82371	-0.33416	-0.73148	-0.19712	-1.05	1.170914	0.763404	0.966358	0.183263	-0.76272	-0.35218	-0.7213	1.072905	0.065767	-0.22833	-0.33961	0.2658
Q9UBL3	ASH2L	0.603113	-0.66253	-0.10771	0.11241	0.212128	0.379942	-0.0911	0.583003	-0.0737	-0.17917	0.220278	-0.58042	-0.39105	-0.03524	0.793282	-0.33603	0.192209	0.510552	0.19689
Q9UBM7	DHCR7	0.533393	-0.20598	0.010387	-0.285	1.725028	2.063587	-0.08931	0.698293	-0.25698	-0.74227	-0.05922	-0.34529	-0.03758	-0.42442	3.377047	0.36602	-0.75063	-1.16489	-0.76316
Q9UBN7	HDAC6	-0.21166	-0.61037	-0.99851	-0.5857	-1.06643	-0.59423	-0.93705	-0.52654	-0.08979	0.148189	-0.27819	-0.53173	-0.63679	-0.27434	-0.72632	-0.04191	-0.30875	-1.34578	-0.36869
Q9UBP0	SPAST	-0.20481	0.261995	0.483075	-0.49327	-0.27603	-0.12892	0.288988	0.153138	-0.04741	0.134077	-0.46805	-0.05816	-0.0859	0.293212	-0.27785	0.231667	-0.26399	-0.24559	0.283707
Q9UBP6	METTL1	0.153695	-0.11183	0.007488	0.142291	0.77113	0.536807	-0.11202	0.950456	-0.64438	-0.3027	0.4464	0.346152	-0.21735	0.132674	-0.10139	-0.20059	1.330562	0.0335	-0.96033
Q9UBP9	GULP1	0.645715	-0.75745	-0.56385	-1.15304	-1.33339	-1.35114	-0.37594	-0.96865	-0.28633	-1.6464	-0.17419	-1.33528	-0.47237	-0.53663	0.013729	-0.58845	-0.98453	-0.41778	-0.56757
Q9UBQ0	VPS29	0.656638	-1.83465	0.862555	1.351569	1.786912	0.31231	-0.28019	-0.01955	0.433929	0.925117	-0.16064	0.534128	0.585881	0.937765	0.422108	0.658039	0.974226	0.229558	0.858084
Q9UBQ5	EIF3K	-0.17712	0.20726	-0.20523	-0.25002	0.25241	0.498972	0.246519	0.379536	-0.20549	-0.13762	0.072541	-0.70744	-0.18605	-0.35812	0.846164	0.123479	-0.23069	-0.72646	0.355158
Q9UBQ7	GRHPR	0.43017	-1.4317	-0.40057	0.191081	0.855379	-0.39218	-0.85783	-0.358	0.698292	0.437136	0.32205	0.182178	-0.12435	0.756011	-0.49801	1.715227	-0.38655	-0.20328	-0.32801
Q9UBR2	CTSZ	-0.90861	-0.18205	0.379324	-1.03139	1.274543	-0.74208	2.178449	0.893195	1.073681	1.069501	-1.51555	1.191395	0.277398	0.571275	-0.60736	-0.78974	0.154127	-0.61932	-0.93343
Q9UBS0	RPS6KB2	-0.0864	-0.21547	-0.00979	0.407178	-0.08901	0.032197	0.336028	0.716017	0.108549	0.22748	-0.08614	0.105513	-0.3309	0.038256	-0.94407	0.264771	0.560059	-0.31233	-0.71506
Q9UBS4	DNAJB11	-0.02054	-0.16523	0.246921	0.734969	0.188055	0.250359	0.024092	0.026335	0.367654	0.363757	-0.43031	0.259836	0.160239	0.393643	-0.22786	-0.07375	0.526359	0.153742	-0.0925
Q9UBS8	RNF14	-1.32621	0.780359	-0.1452	0.02575	-0.27124	0.285816	0.487296	-0.45577	0.523155	0.158088	-0.16396	-0.00151	0.800252	0.12994	0.584197	-0.36676	0.476999	-0.11424	0.255039
Q9UBS9	SUCO	-0.11968	-0.17439	-0.16814	-0.87622	-0.84988	-0.32599	0.325829	0.321632	-0.13261	-0.41926	-0.31651	-0.74536	-0.21031	0.091771	0.946625	-0.72825	-0.55885	0.355615	-0.65856
Q9UBT2	UBA2	0.895879	-0.99063	-0.39002	0.149018	0.328769	0.648892	-0.53796	1.480435	-0.21789	-0.22165	-0.13615	-0.20702	0.234059	-0.13728	0.220729	-0.42257	0.122674	0.012515	0.221617
Q9UBT7	CTNNAL1	-0.00105	0.163921	0.510638	-1.08794	0.315755	0.449904	0.660326	0.475246	-0.42367	-0.61263	0.996587	-0.74775	-0.16813	-0.22786	-0.3588	-0.14003	-1.0728	-0.28072	0.606013
Q9UBU6	FAM8A1	-0.2175	0.199754	0.154435	-0.109	0.11061	-0.43483	0.373351	0.19122	-0.7116	-0.63872	-0.38168	-0.29175	-0.27485	-0.62643	0.799058	-0.10032	-0.58496	0.088673	-0.03975
Q9UBU8	MORF4L1	0.262485	-0.09496	-0.01839	0.021125	0.729598	1.23235	0.107672	0.306896	-0.31456	-0.10295	0.245357	-0.46505	-0.4361	-0.31708	0.167732	-0.37698	-0.40794	0.588949	-0.8445
Q9UBU9	NXF1	0.292532	0.021086	-0.16509	0.038069	0.173118	0.206538	-0.45074	0.679592	-0.07081	-0.1584	0.156348	-0.05621	0.186768	-0.15389	0.148894	-0.05036	0.395494	0.266559	-0.12615
Q9UBV2	SEL1L	-0.40388	0.414932	0.268231	0.688673	-0.02168	0.118223	1.219397	-0.06451	-0.32115	-0.20911	0.116266	-0.41594	0.278673	-0.45567	0.929086	-0.06403	-0.45365	0.204432	0.231663
Q9UBV8	PEF1	0.062393	-0.2433	-0.45179	0.528907	0.208068	0.38658	-0.00751	0.561344	0.214174	0.67216	0.733111	-0.52076	-0.8192	0.003739	0.101757	0.196486	-0.37143	-0.1906	-0.07927
Q9UBW5	BIN2	-2.00697	1.458794	1.217887	-0.88885	0.336282	0.247914	0.537592	-1.12095	0.572384	1.749337	-1.29222	0.397632	-0.15636	1.291109	-0.46155	-0.89571	0.282743	0.954207	-0.70413
Q9UBW7	ZMYM2	0.011337	-0.066	-1.42703	-0.21566	0.210917	0.294063	0.412883	0.48205	-0.63426	-0.21053	-0.00513	-0.30985	-0.03408	-0.27659	0.589834	-0.52767	0.099896	0.086313	0.780637
Q9UBW8	COPS7A	-0.58899	0.25118	-0.0431	-0.01772	-0.62164	-0.63862	0.223717	-0.87162	-0.30265	-0.23512	0.188949	-1.00719	-0.00171	-0.75522	0.331015	0.036514	-0.39821	0.153114	0.337695
Q9UBX1	CTSF	-0.45392	0.132952	-0.37261	-0.4942	0.020522	-0.42979	0.400765	0.489008	0.698961	-0.79681	-1.23322	-1	-0.41538	-0.32493	0.131544	-1.29689	0.023286	-0.80532	-1.17566
Q9UBX3	SLC25A1(	0.771165	-0.6828	-0.05319	0.290358	0.210289	0.118968	-0.12834	-0.28564	-0.75424	-1.31997	0.01966	0.220636	0.010479	0.096742	1.299429	0.32559	-0.5774	-0.8891	-1.50106
Q9UBX5	FBLN5	-0.67522	-2.10615	-2.05391	-2.59631	-1.89914	-2.23341	-0.5769	-1.31655	-0.15428	-2.27329	-1.09739	-1.06973	-2.44032	-0.48903	0.849686	-1.21171	-0.80373	-2.4157	-1.25917
Q9UD71	PPP1R1B	-0.8901	0.026726	-0.55516	-1.03968	-1.50886	-0.85756	-0.44846	-1.59152	-0.36451	-1.06934	1.610052	-0.95899	-1.11405	-0.26569	1.427431	-0.28707	0.616799	-2.35324	-0.00701
Q9UDR5	AASS	0.477544	-0.44708	-0.27743	-0.39011	-1.08294	-0.80157	0.059303	-1.77151	-0.53385	-0.20813	0.495539	-1.4935	-0.93198	-0.91207	-1.04424	1.547017	-0.75683	-0.56284	0.111465
Q9UDT6	CLIP2	-0.27894	0.278142	0.4415	-1.05912	0.12395	-0.3666	-0.22307	0.700065	-0.28624	0.402899	-0.72662	-0.88509	-0.0679	-0.61548	-0.38031	-0.26379	-0.5122	0.445695	0.600289
Q9UDW1	UQCR10	0.513305	-0.51152	-0.30593	-0.04996	0.783968	0.928286	-0.29088	0.090456	0.299339	0.003954	0.318238	-0.17079	-0.2374	0.231694	1.666041	-1.0794	-0.62491	-0.82356	-0.95718
Q9UDX5	MTFP1	-0.52562	0.287881	0.062246	0.978914	0.239646	0.897467	0.100149	0.027851	-0.3484	0.166052	0.323277	-0.18471	-0.26856	0.840777	-1.10129	0.138149	0.322725	0.044838	-1.2992
Q9UDY2	TJP2	0.625259	-0.55036	0.811854	-0.29469	0.170229	0.525303	-0.38637	0.319728	-0.55096	-0.4446	-0.08386	-0.75815	-0.5583	-0.45791	0.223156	0.81643	-0.33741	-0.69172	-0.46978
Q9UDY4	DNAJB4	0.881853	0.261236	-0.081	-0.61084	-0.25064	0.539023	-0.3888	-0.28514	0.483976	-0.01176	-0.30532	-0.60725	0.630506	-0.12309	-0.22516	0.018583	0.100572	-0.20761	1.201822
Q9UDY8	MALT1	-0.77931	-1.09032	0.397053	-0.06766	-0.19053	-0.44155	-0.27535	0.708569	0.147804	0.4247	-0.2187	0.064973	-0.03354	-0.38049	-0.62993	-0.19837	1.018299	0.35257	1.001285
Q9UEE9	CFDP1	0.636209	-1.30584	-0.50136	-0.175	-0.15862	0.120327	-1.49606	-0.58988	0.040847	0.143621	0.36467	-0.8046	0.665671	-0.89306	0.627865	0.870671	-0.53454	0.157849	0.406439
Q9UER7	DAXX	1.151988	-0.24489	-0.04033	0.073074	0.401506	0.800094	0.02683	0.249886	-0.77363	-0.04337	-0.43129	-0.1012	-0.43989	0.018075	0.683154	0.102133	0.566496	-0.18241	-0.29502
Q9UEU0	VTI1B	-0.1348	0.619141	0.190368	-0.04434	-0.57803	-0.60313	0.55651	-0.27974	0.696068	-0.08398	-0.32049	0.267811	0.039504	0.416101	0.341283	-0.52791	0.073491	0.133337	0.246291
Q9UEW3	MARCO	-1.75875	0.997637	0.774977	-1.22486	0.117713	-2.65721	-0.40255	-1.11243	-0.10288	1.075778	-1.63965	4.010317	-0.08469	2.53192	-1.269	-1.33459	1.827266	0.466649	-2.40488
Q9UEW8	STK39	-0.06515	0.567025	0.405596	-0.47585	0.061695	0.175194	-0.17998	-0.70063	-0.28855	1.142605	-0.51908	0.199043	0.304411	0.955096	-0.54465	-0.4968	0.60703	-0.31502	-0.08676
Q9UEY8	ADD3	0.032001	-0.57858	0.256832	-0.77737	-0.09801	-0.09109	-0.03351	-0.07127	-0.41649	0.146203	-0.0868	-1.16269	-0.70886	-0.64162	-0.11301	-0.69922	-0.41651	0.401417	-0.13876
Q9UFC0	LRWD1	0.759771	-0.12038	-0.56186	-0.43917	-0.38583	-0.00141	0.34066	0.361085	-0.37494	-0.72282	0.352812	-0.27791	-0.1022	-0.18769	0.426112	-0.35968	-0.18899	-0.31662	-0.14868
Q9UFF9	CNOT8	-1.464	-0.17927	-0.16894	0.516115	0.210173	0.12727	-0.47609	0.360346	0.050501	0.091519	0.201514	0.30926	-0.08549	0.348856	-0.02569	0.28828	0.452252	-0.60271	-0.09343
Q9UFG5	C19orf25	-0.01615	0.544868	0.171369	-0.10224	0.079774	0.25279	0.696034	0.019928	-0.00376	-0.12605	-0.20241	0.446778	-0.29523	0.126255	0.287466	0.321279	0.364742	0.206123	0.240898
Q9UFN0	NIPSNAP:	0.38701	-0.08099	0.561876	-0.88535	1.117228	0.991246	-0.62169	-0.17258	-0.00997	-0.35714	1.257021	-0.2174	0.24998	0.154526	0.910432	1.044201	-0.36227	0.28894	-0.1466
Q9UFW8	CGGBP1	0.865623	-0.49684	-0.12458	-0.23735	-1.24564	-0.24727	-0.60629	0.144875	-0.07971	-0.5905	-0.23654	-0.80105	-0.32652	-0.11684	0.389605	-0.15273	-0.90003	-0.10454	-0.28625
Q9UG01	IFT172	-0.3481	-0.14098	-0.44265	-0.07101	-0.39357	-0.2057	-0.02075	0.011922	0.040847	-0.66899	1.804887	-0.46257	-0.14238	-0.25858	-0.12332	-0.27375	-0.50504	-0.3193	-0.09822
Q9UG22	GIMAP2	-0.8811	0.015795	-0.40183	-1.24149	0.529567	-0.17407	0.703461	-0.10801	-0.7991	-0.43407	-1.01171	0.689411	0.164726	0.419855	-0.38861	-0.35876	0.252186	-0.11522	-0.51616
Q9UG56	PISD	-0.13466	0.054937	0.228104	1.064643	0.060972	0.601758	-0.30028	-0.47432	-0.4173	0.48441	0.269157	-1.3549	0.172636	-0.14378	0.190354	0.691609	-0.47798	1.254945	-0.66333
Q9UG63	ABCF2	-0.14328	-0.00353	0.179101	1.104647	-0.24437	-0.01762	-0.38036	0.518346	-0.92101	0.079352	0.158547	-0.47059	-0.22431	-0.58905	1.051911	0.493894	-0.42065	-0.2217	0.075336
Q9UGI8	TES	0.208389	-0.82232	-0.21307	0.378932	-0.50016	0.091257	-1.0654	1.078288	-0.85277	-0.21011	0.016978	-0.4688	-0.19451	0.067565	0.42006	-0.04794	-0.13354	-0.99256	-0.21782
Q9UGJ0	PRKAG2	-0.07632	0.146425	0.104275	0.536181	0.009788	-0.44402	0.075235	0.431547	-0.33046	0.0564	-0.34645	0.612377	-0.16475	0.38939	1.480439	0.31517	0.628061	-0.99895	-0.65949
Q9UGJ1	TUBGCP4	0.123838	0.295587	0.422998	-0.11659	0.066176	0.172424	0.351568	-0.52284	-0.01832	-0.22424	0.167506	0.250732	0.122183	0.344619	-0.05663	-0.18078	0.262496	0.092183	-0.01298
Q9UGK3	STAP2	0.791349	0.089068	-0.22825	-0.32365	-0.46541	-0.08203	0.923619	0.024481	-0.74099	1.477025	0.283982	-0.76055	-0.49655	-1.09244	-1.15681	-0.13194	-0.68065	-2.23507	-0.55795
Q9UGL1	KDM5B	0.139864	-0.04678	0.006121	-0.19891	-0.85791	-0.5123	-0.14748	0.766535	-0.41858	-0.00951	0.198548	-0.15855	-0.21311	0.891902	1.010971	-0.0394	0.022878	-0.6724	0.244887
Q9UGM5	FETUB	-1.62706	0.780235	-0.60806	-1.82073	-1.05297	-2.37264	0.269669	-1.18316	0.514051	0.189799	-0.91289	0.235907	-0.3708	1.02312	-1.1046	-1.60183	0.888978	0.26439	-1.31969
Q9UGM6	WARS2	0.922382	-0.40914	0.173426	0.675368	-0.05697	-0.63321	-0.13053	-0.08117	0.05979	0.047312	0.50025	0.127006	0.109556	-0.35508	0.402236	0.814018	0.124885	-0.25171	-0.63607
Q9UGN4	CD300A	-1.60022	0.605655	0.447486	-0.54651	-0.2271	-0.65782	0.13435	-0.42574	0.099329	0.344106	-0.194	0.167799	-0.07741	1.180274	-0.5196	-0.04202	0.361293	-0.10203	-0.53892
Q9UGP4	LIMD1	0.626389	0.282068	-0.02298	-0.77822	-0.60148	-0.49378	0.280099	-1.11912	-0.08946	-0.4499	0.129661	0.045461	0.145937	0.279713	0.289388	-0.41673	-0.08986	0.087609	-0.3769
Q9UGP8	SEC63	-0.60366	0.377149	-0.21978	0.557425	-0.01236	0.07112	0.155115	-0.53163	0.081491	0.209774	0.511584	0.627627	0.099556	0.302448	1.200945	-0.11674	0.696736	0.045607	-0.14265
Q9UGQ2	CACFD1	-0.26623	-0.46658	0.601376	-0.5678	-0.84795	-0.06197	1.038242	1.367746	0.676145	-0.43054	0.180719	-1.08156	0.036132	-0.23436	0.160585	-0.31915	-0.1538	-0.02061	0.566158
Q9UGR2	ZC3H7B	-0.16635	-0.18836	0.515836	-0.2547	-0.09528	0.166869	0.424113	-0.6265	0.028101	0.102012	-0.34866	-0.21522	-0.05236	-0.0817	-0.27716	-0.28944	0.37124	0.888394	0.29289
Q9UGT4	SUSD2	0.247149	-0.34064	-0.81947	-1.02322	-0.85255	-1.01357	0.454133	-0.10904	-0.23307	-0.35557	0.366523	-0.87269	-0.22159	-0.66271	1.020942	-0.33122	-0.95792	-0.34492	-1.77981
Q9UGU0	TCF20	0.115073	0.052602	-0.01934	-0.07242	-0.52382	-0.14331	0.072291	-0.49843	-0.1464	-0.3933	0.556316	-0.62336	1.06499	-0.54429	0.777468	0.296177	-0.33622	0.690251	-0.71055
Q9UGU5	HMGXB4	0.255242	-0.22715	-0.12864	0.628834	-0.86788	0.258518	-0.14908	0.144897	-0.38934	-0.83097	0.258432	-1.03826	-0.32582	-0.3255	0.21465	-0.58144	0.116882	1.050523	-0.0295
Q9UGV2	NDRG3	-0.7085	-0.01952	0.207448	-0.53652	0.634673	1.074378	-0.09878	0.399759	-0.36589	0.234946	-1.31671	-0.46294	-0.15245	0.430318	-0.19585	0.072623	0.19545	-0.41986	-0.16166
Q9UH17	APOBEC3	0.195705	-0.36286	0.336036	-0.36479	3.089192	1.489793	-0.35383	0.791841	-0.04252	0.085336	-0.51337	-0.31497	0.455776	0.372988	-0.43582	0.265021	0.532646	-0.60795	0.880012
Q9UH62	ARMCX3	-0.22426	0.437923	0.727404	0.275313	0.75577	0.35621	0.91729	0.683066	0.038198	0.508914	-0.17865	-0.13822	-0.26002	0.055124	0.114986	0.711851	0.544041	0.772306	-0.07358
Q9UH65	SWAP70	-0.81851	0.314214	-0.09919	-0.72975	-0.08492	0.008727	-0.23756	0.070844	-0.23527	0.456088	-0.33248	-0.33164	-0.16099	0.214832	-0.25407	-0.22733	-0.22587	0.025869	-0.62923
Q9UH92	MLX	-0.36481	0.289643	0.1073	-0.35989	-0.61658	0.013519	0.087514	0.228251	0.971428	-1.07239	0.16043	-0.33113	-0.14792	0.01476	-0.19929	-0.79573	-0.07188	0.189081	0.418569
Q9UH99	SUN2	-0.30855	0.379514	0.248529	-0.73846	-0.07578	-0.293	0.420431	-0.62997	0.204655	-0.12451	0.041783	-0.15439	-0.05129	0.354269	-0.20715	-0.10974	-0.1083	0.467389	-0.09635
Q9UHA4	LAMTOR3	-0.34604	-0.28198	0.117419	-0.35837	1.422646	-0.49303	-0.34024	0.620039	0.142812	0.517638	0.208565	0.632982	0.214659	0.96025	-0.46067	0.382318	0.437268	0.248808	-0.24432
Q9UHB6	LIMA1	-0.16198	0.102692	0.513063	0.082833	-0.64421	-0.1528	-0.81695	-0.72928	0.493029	-0.10361	-0.56824	-1.54073	0.28241	-1.26039	-0.72707	-0.00592	0.08187	1.02736	1.583301
Q9UHB7	AFF4	0.277235	-0.0816	-0.08366	-0.49837	-0.04829	0.508723	-0.58258	0.243117	-0.12148	-0.17942	0.379931	-0.64866	-0.10908	-0.13696	0.674683	-0.16058	-0.07273	0.495592	-0.39881
Q9UHB9	SRP68	-0.04735	0.212195	0.422455	0.242159	-0.02555	0.161695	0.508506	-0.40379	-0.01787	0.206418	0.245544	0.029948	0.167338	-0.10119	-0.35323	0.682993	0.527227	0.51215	0.079802
Q9UHD1	CHORDC'	-0.06463	-0.38664	-0.18169	-0.29764	1.081597	0.320251	-0.19368	-0.56137	0.357236	0.636237	0.112951	0.485645	0.994222	0.300184	-0.84644	0.59211	1.252792	0.162393	-0.41415
Q9UHD2	TBK1	0.02287	0.490537	0.612459	-0.50502	0.067575	0.240107	-0.46893	-0.18392	-0.18057	0.301334	-0.46813	0.19901	0.195835	0.645986	-0.40307	-0.26753	0.269933	-0.04926	0.158976
Q9UHD8	SEPT9	0.54751	-0.50532	-0.60245	-0.37953	-0.46531	-0.55849	-0.37387	-0.34011	-0.4728	-0.13494	0.453622	-0.84608	-0.32648	-0.44971	-0.13755	0.212262	-0.19837	0.19618	0.000391
Q9UHD9	UBQLN2	0.287513	-0.08891	-0.16832	-0.34559	-0.33034	-0.05184	0.174415	-0.26604	0.347279	-0.31059	-0.50742	-0.18269	0.096023	0.114741	-0.16124	-0.30636	0.168735	-0.10318	-0.05822
Q9UHE8	STEAP1	-0.5733	0.436669	0.371697	-1.84924	-0.17527	-0.16615	0.210532	-1.43199	0.040847	-0.1538	-0.9231	0.017213	0.118858	0.219827	0.161624	1.86447	-0.11995	-0.05154	0.658241
Q9UHF7	TRPS1	0.037225	-0.08484	0.1574	-0.32352	0.893945	1.523439	-0.03861	-1.1865	-0.36874	-1.28877	-0.97863	0.148044	0.103892	-0.37273	2.318116	0.472088	-0.26926	1.120158	-0.32088
Q9UHG0	DCDC2	1.061129	-0.10392	-0.35424	-0.26459	1.951889	1.765162	-0.2801	0.244934	1.765026	-0.3514	0.32535	-0.90617	-0.2121	-1.3293	-0.36981	-0.17086	0.73073	0.662855	0.023982
Q9UHG3	PCYOX1	-0.18048	-0.1515	-0.55429	0.170899	-0.1276	-0.08006	0.598857	0.444202	0.265695	-0.65735	-0.3317	-0.52322	-0.45242	-0.36677	0.124206	-0.14201	-0.44087	-0.67665	0.188365
Q9UHI6	DDX20	0.040548	0.046195	-0.13842	0.312765	0.161304	0.285794	-0.54862	0.357249	-0.29542	-0.35198	0.840385	-0.38489	-0.30044	-0.13907	0.210235	-0.08663	0.346427	0.154483	0.141665
Q9UHJ6	SHPK	0.733194	0.191952	0.0723	-0.19528	-0.30402	-0.09467	0.466039	-0.57018	0.175935	-0.22132	-0.43184	-0.68222	-0.3184	-0.05121	-0.0517	-0.43366	-0.08079	0.158821	0.114023
Q9UHL4	DPP7	0.683888	0.589377	-1.43809	-0.88635	0.043768	-1.33347	-0.62966	0.62329	-0.02185	0.246671	0.245981	0.521243	-0.34766	0.180837	-0.97289	-0.84068	-1.10166	-0.64185	-0.88396
Q9UHL9	GTF2IRD1	0.243474	0.190549	0.288686	-0.27805	-0.36303	0.126181	0.322228	1.138719	0.040847	-0.35111	-0.93187	-0.27771	-0.17483	0.137872	-0.17997	-0.34633	0.16877	0.155966	-0.079
Q9UHN1	POLG2	0.332767	-0.3688	-0.65649	1.060023	-0.66829	0.049429	-0.23142	0.038336	-0.34569	-0.19923	0.771398	-0.45409	-0.09956	-0.51517	1.306962	0.995704	-0.66863	-1.14961	-0.54796
Q9UHN6	TMEM2	-0.76253	0.593747	0.447754	0.035875	-0.05152	-0.21542	0.046363	-0.37623	0.039459	0.445271	-0.90489	0.484089	0.17616	0.57862	-1.55016	0.234641	0.0021	0.387159	0.41895
Q9UHP3	USP25	-0.34694	1.214723	-0.05857	-0.02448	0.071103	0.097091	-0.09427	-0.48936	0.414227	0.033155	0.116053	0.282928	1.469262	0.108994	-0.21342	-0.33675	0.297641	0.04553	-0.34193
Q9UHQ1	NARF	0.370854	-0.21017	-0.41879	-0.4499	-1.12301	-0.15382	0.029362	0.046448	-0.06329	-1.11959	0.106633	-0.37448	-0.13801	-0.23801	-0.34294	-0.49812	-0.06321	-0.5228	0.060541
Q9UHQ4	BCAP29	-0.08364	0.5857	1.043879	-0.38161	0.255448	0.357149	0.128848	-0.26952	0.040257	-0.29591	-0.23948	-0.28619	0.451786	-0.11415	1.264579	0.16065	-0.45015	0.321151	0.135203
Q9UHQ9	CYB5R1	-0.85475	-0.18118	-0.28633	-0.30085	-0.29685	-0.25587	-0.28184	-1.58273	-0.90249	-0.93172	1.302323	-0.03451	-1.06767	-0.26717	1.223076	-0.69527	-0.6676	-0.87474	-1.47961
Q9UHR4	BAIAP2L1	0.907254	-0.57671	-0.27975	0.003161	-0.06026	0.76081	-0.35305	0.906719	-0.40504	0.276022	0.057483	-0.59162	-0.04194	-0.70334	0.466708	1.321394	-0.91834	-0.92042	-0.87968
Q9UHR5	SAP30BP	0.408178	-0.09283	-0.01387	0.230044	0.349419	0.554857	-0.23184	0.833919	-0.79903	-0.25447	-0.18917	-0.51981	0.123682	-0.62906	0.075827	-0.30708	0.093279	0.421268	0.183811
Q9UHR6	ZNHIT2	0.166061	0.094626	-0.91443	0.169287	0.219742	0.328702	-0.46857	-0.71312	0.061188	-0.56033	0.522979	-0.09057	0.809539	0.104626	0.220477	-0.37033	0.348423	-0.33308	-0.16243
Q9UHV9	PFDN2	0.35233	-1.12221	0.152536	-0.06112	0.502515	0.478639	-0.81119	0.911302	0.105945	-0.19025	-0.15019	-0.35455	0.363237	-0.3493	0.132221	0.313122	-0.18734	-0.22783	-0.21986
Q9UHW5	GPN3	-0.20518	-0.10297	-1.01259	-0.13414	-0.1661	-0.1269	-0.04741	-0.34721	-0.04454	-0.12566	0.085735	-0.29629	0.446293	-0.07931	-0.63158	-0.05924	0.347533	-0.11352	0.221112
Q9UHW9	SLC12A6	-0.30157	1.184201	-0.42464	0.228422	-0.14583	-0.32269	0.010416	0.122756	0.414227	0.812046	-0.97748	0.361557	-0.05878	0.505761	-0.05789	0.788755	0.119223	-0.21324	0.169538
Q9UHX1	PUF60	1.083733	-0.48986	-0.15398	0.069693	-0.00084	0.10907	-0.7858	0.598998	-0.14896	0.154721	0.31636	-0.3983	0.407217	-0.65696	-0.09224	-0.06528	-0.26204	-0.11649	-0.2362
Q9UHY1	NRBP1	-0.30318	0.398895	-0.03098	0.08693	-0.40256	-0.47818	-0.82894	-0.05908	0.320424	0.289572	-0.36234	-0.43698	0.43962	0.015565	-0.34146	-0.38876	0.260993	0.01778	0.5539
Q9UHY7	ENOPH1	0.081527	-0.02389	-1.08099	-0.5567	-0.179	0.257303	-0.79707	-0.1955	0.880879	0.630445	0.002189	-0.75328	-0.08644	-0.27592	-0.34147	-0.1714	-0.1069	0.033637	-0.77058
Q9UI08	EVL	-2.42475	0.123699	1.457385	-1.14351	0.614768	0.856518	-0.29055	-1.73516	0.086799	0.70984	-1.29847	-0.15408	0.439341	-0.21891	-0.21584	-0.45384	-0.59351	1.263299	-0.76496
Q9UI09	NDUFA12	0.003363	0.03648	-0.25637	-0.54803	0.34272	0.028403	-0.10721	-0.39337	-0.05138	-0.55837	0.285918	0.014974	0.007381	-0.17776	0.928262	-0.7897	0.475806	0.152102	-0.01752
Q9UI10	EIF2B4	-0.21604	0.370055	-0.11993	0.377029	-0.14428	0.357761	0.206537	-0.11722	-0.13024	-0.14109	-0.10982	-0.06782	0.11363	-0.55151	0.238131	0.821631	-0.4784	-0.49056	-0.44204
Q9UI12	ATP6V1H	-0.24258	0.772024	-0.22217	-0.51685	-0.01227	-0.05114	-0.01393	-1.0301	-0.30831	-0.0289	-0.29634	0.801494	0.11872	0.591663	0.54152	-0.03484	-0.14261	-0.85264	-0.72708
Q9UI14	RABAC1	-0.47784	0.129774	0.039645	0.605838	0.374555	0.313063	0.255974	0.177706	-0.25628	-0.20346	0.045843	0.316155	0.075918	-0.47449	1.195952	0.357661	0.593559	0.110693	0.175826
Q9UI15	TAGLN3	-0.00783	-1.0186	-0.02794	0.394399	-0.01185	0.377485	-0.4476	0.849277	0.271371	1.434951	-0.52809	-1.2422	0.862188	-0.95503	1.203669	0.730769	-0.32938	-0.56637	-0.15775
Q9UI26	IPO11	0.031449	0.953206	-0.19781	0.633208	-0.0111	0.335608	0.849712	0.042924	-0.10165	-0.2097	-0.1964	-0.06139	-0.32148	-0.12801	0.879405	-0.16839	0.569825	-0.34394	0.406313
Q9UI30	TRMT112	0.984584	-0.42381	0.190484	0.298468	0.04103	0.49489	-0.32854	0.427258	0.135359	-0.20252	0.404066	-0.40377	0.216011	0.120291	0.120291	-0.15111	0.427911	0.485335	0.258812
Q9UI36	DACH1	-1.51649	0.348031	-1.27226	-3.79625	-3.43574	-1.64473	2.637776	3.070936	0.101074	-1.59871	-1.10009	-2.46471	-0.08039	-0.40639	-2.00296	-2.19521	-2.26916	-1.59669	2.056127
Q9UI43	MRM2	-0.17549	0.021441	0.073707	-0.03156	0.547492	0.201195	0.110479	-0.18299	0.484148	0.38313	-0.07876	0.018363	-0.52667	0.26393	-0.01413	0.156151	0.37969	-0.17322	-0.8833
Q9UIA0	CYTH4	-1.33298	1.097212	0.891907	-0.7136	0.543246	0.02799	0.253093	-1.14159	0.016915	0.809514	-0.71765	0.657869	0.206916	1.636243	-0.42838	-0.29066	0.046182	0.436622	-0.98463
Q9UIA9	XPO7	0.381224	-0.04024	-0.05572	-0.06212	-0.40842	-0.21672	0.123619	-0.07382	-0.47181	-0.5362	0.286054	-0.35755	-0.33281	-0.1661	-0.30849	-0.06716	0.377939	-0.14234	0.28295
Q9UIB8	CD84	-2.13478	0.92327	0.927121	-1.06809	0.136027	-0.78594	0.131788	-1.26001	0.041068	0.992051	-0.64843	0.940144	0.016498	1.028583	-0.75866	-0.63153	0.164955	0.398732	-0.99934

Q9UIC8	LCMT1	0.072421	-0.25831	-0.17782	-0.16341	0.315516	-0.54386	0.081005	-0.02019	-1.013	0.33696	-0.3917	0.164232	0.760739	0.280561	-0.12404	-0.00548	0.232311	-0.08074	0.182911
Q9UID3	VPS51	0.228731	0.546394	-0.11767	-0.31132	-0.09224	-0.15199	0.196439	-0.01501	0.131921	0.3884	0.196535	0.076773	0.15456	0.120198	0.592555	0.347616	-0.14086	-0.46952	-0.1703
Q9UIF9	BAZ2A	-0.15505	-0.05886	0.242042	0.232695	0.519318	1.011274	0.003181	0.137146	0.345921	-0.13134	-0.32446	-0.4218	0.061174	0.12818	0.147272	-0.12224	1.077877	0.702153	-0.20221
Q9UIG0	BAZ1B	1.310462	0.199636	0.011541	0.094515	0.566412	1.033165	-0.0024	0.81644	-0.62047	0.011928	-0.09302	-0.59735	-0.48733	-0.25823	0.905092	-0.58093	-0.70266	-0.50725	-0.38078
Q9UII2	ATPIF1	-0.0043	-0.205	0.906234	0.792519	-1.68408	0.776536	-2.43479	-0.88056	-0.88586	-0.46171	-0.03308	-2.21146	-1.23557	-1.50397	0.307245	2.773985	-1.37399	0.134847	-1.46618
Q9UII4	HERC5	0.686001	1.07844	1.163449	-0.62873	1.254254	2.111765	0.14993	0.112044	0.101227	-0.63297	-1.2764	0.076197	-0.23019	0.05467	-1.47034	-0.67988	-0.26158	-0.23882	-1.05086
Q9UIJ7	AK3	-1.30768	0.02627	-1.12443	-0.54647	2.153488	0.293682	-0.07556	-0.23219	0.361935	-0.5356	0.494543	0.227309	-0.2953	-0.0638	-0.69836	-1.16385	-1.21008	-0.11583	-1.48078
Q9UIQ6	LNPEP	-0.4683	-0.08029	-0.31458	0.077604	0.051977	-0.0695	0.224868	-0.3709	-0.33058	-0.62361	-0.13287	0.067867	0.335905	0.117614	0.573705	0.716333	0.186901	-0.01204	-0.03096
Q9UIS9	MBD1	-0.10049	0.017872	0.212907	0.228991	-0.18351	-0.08074	0.551881	-0.1613	0.288921	0.620353	0.479597	0.185377	0.20145	0.194972	0.148555	0.272818	-0.34682	-0.0697	-0.2997
Q9UIU6	SIX4	0.408059	0.055907	0.158964	-0.54235	-0.13446	0.348323	-0.25559	-0.25416	-0.41518	0.090995	0.545292	0.492398	0.280869	0.395957	-0.36633	0.178622	0.244629	0.895967	-0.31175
Q9UIV1	CNOT7	0.301452	-0.4162	-0.8945	-0.69184	0.290755	0.224281	-0.12183	0.14	0.537894	-0.40482	0.279012	0.10687	-0.21347	-0.01962	-0.44469	0.041254	0.364364	0.066917	0.076924
Q9UIW2	PLXNA1	0.039702	0.303992	0.144446	-0.52883	-0.14799	-0.16242	-0.26653	-0.24446	0.125447	-0.00877	-0.72053	-0.28637	0.006584	-0.5732	0.203059	-0.11418	-0.2693	0.156753	0.209139
Q9UJ14	GGT7	0.069967	-0.50932	-0.85681	-0.27546	-0.41243	0.433622	0.788695	0.658309	0.124682	-0.23446	0.19923	-1.26631	-0.95463	-0.50755	0.976855	-1.32034	-0.0377	0.40492	0.250214
Q9UJ41	RABGEF1	0.13176	-0.11176	0.823865	-0.6267	0.315585	0.024472	0.112494	-0.0304	0.36246	0.127174	-0.33646	0.202484	-0.0835	0.11306	-0.16517	-0.39681	-0.28988	-0.05885	0.436544
Q9UJ68	MSRA	-0.17434	0.199269	0.249476	-0.33854	0.994583	-0.6521	0.802517	-0.68198	0.186004	2.370987	0.139679	0.84224	0.031504	1.825728	-0.31752	-0.00183	0.257122	-0.35668	0.048989
Q9UJ70	NAGK	-0.68729	0.225118	1.239692	0.020058	0.217269	0.289968	-0.46092	0.016281	-0.01396	0.86703	-0.91163	0.625484	0.291481	0.748516	0.150526	-0.09774	0.09998	0.367242	0.724361
Q9UJ83	HACL1	0.884725	-0.22036	-0.1651	-0.13218	0.432472	0.816822	-0.48968	0.30947	-0.33496	-0.95766	-0.16689	0.575272	-0.20374	0.174752	0.492523	-0.33224	0.222099	-0.1183	-1.15806
Q9UJA5	TRMT6	0.485947	-0.06587	-0.48039	0.304243	0.344577	0.575429	-0.40169	1.637408	1.616821	0.397798	0.355027	0.057713	0.297039	-0.40903	0.85958	-0.79103	0.115801	-0.36507	0.030695
Q9UJC3	HOOK1	0.832264	-0.58209	-0.17124	0.316341	0.35825	0.69288	0.048933	-0.20958	-0.21884	-0.28756	0.607563	-0.80773	-0.4608	-0.74882	0.626894	1.174359	1.603523	0.846941	-0.98511
Q9UJF2	RASAL2	0.063279	0.484648	-0.08948	-0.96519	-0.57366	-0.55171	-0.03324	0.016569	0.557991	0.259272	-0.23968	-0.42178	0.107855	-0.02393	-0.73921	0.06568	-0.37244	-0.04533	0.198524
Q9UJS0	SLC25A13	0.747618	0.164887	-0.44249	-0.29233	-0.63635	-0.40524	-0.04128	0.857481	0.247814	0.740148	0.019546	-0.41581	-0.71062	-0.26521	1.89585	0.737817	-0.50767	-0.45756	0.299001
Q9UJU6	DBNL	-0.35895	0.165667	0.110089	-0.46653	-0.33606	-0.20824	-0.26487	-0.02053	0.206416	0.697066	-0.39601	0.537993	0.202608	0.632753	-0.14929	-0.13384	0.103045	0.247341	0.04406
Q9UJV9	DDX41	0.812546	-0.01346	0.088752	0.09025	0.587676	0.709153	-0.22784	0.101908	0.088446	-0.04213	0.179788	0.423101	0.227546	0.063681	-0.09506	0.376946	0.61112	0.548177	-0.26055
Q9UJW0	DCTN4	0.162219	0.009421	0.25289	-0.43255	-0.11155	0.196795	0.039682	0.081912	0.84848	-0.70854	0.03848	-0.18039	-0.1342	-0.08347	0.06543	-0.436	-0.272	-0.19438	0.182044
Q9UJX2	CDC23	-0.22174	0.052991	0.724701	0.181244	0.088542	0.452418	-0.17096	0.561011	0.349515	-0.03956	0.501577	0.016635	0.356611	0.124408	0.278514	0.538757	0.337165	0.259164	0.321108
Q9UJX3	ANAPC7	-0.09636	0.761746	-0.05349	0.365471	0.020012	0.409178	-0.11522	0.186487	-0.41089	-0.01618	0.061898	0.011242	0.674992	-0.13374	0.282578	0.551133	0.208797	0.162951	0.194698
Q9UJX4	ANAPC5	-0.01666	-0.01713	0.085352	0.11767	0.020016	0.155627	-0.27929	0.262294	0.009198	-0.34007	0.491363	-0.36092	-0.04791	-0.14406	-0.01228	0.350938	0.252886	0.020869	0.25241
Q9UJX5	ANAPC4	0.244203	-0.01403	0.079656	0.071202	-0.00068	0.343249	-0.20406	0.100693	0.056274	-0.04813	0.39011	-0.09122	0.088372	0.215203	-0.16122	0.707008	0.472607	0.199364	0.244824
Q9UJX6	ANAPC2	-0.09619	-0.1325	0.197491	-0.31034	0.047119	0.397815	0.221986	0.327449	-0.32848	-0.30003	0.019607	-0.45786	-0.36119	-0.10669	-0.2908	0.310779	-0.00291	-0.1383	0.679672
Q9UJY1	HSPB8	-1.83476	0.97162	0.321911	-1.06134	-0.39867	-0.65975	0.344686	-0.42816	0.433555	0.61243	-0.30198	-0.11497	0.385421	-0.61133	-0.15438	-0.91106	0.021314	0.209341	1.040702
Q9UJY4	GGA2	0.307793	0.050673	0.900721	-0.38508	-0.59646	-0.49293	0.187704	-0.40928	0.023312	-0.2875	0.202507	0.14705	0.436136	0.164078	-0.5354	0.148677	0.111578	-0.24985	-0.51899
Q9UJY5	GGA1	-0.53966	0.183299	0.331395	-0.67498	0.221225	-0.04331	-0.01933	-0.40261	-0.08178	-0.36357	0.025466	0.036287	-0.04375	-0.18076	-0.15509	-0.00422	-0.29528	0.023567	-1.24147
Q9UJZ1	STOML2	0.123155	-0.07219	-0.40867	1.022251	0.408259	0.326938	0.077024	-0.31707	-0.00982	-0.38022	0.393259	-0.23492	-0.6032	-0.22074	-0.57885	0.707595	-0.23799	-0.18797	-0.50038
Q9UK22	FBXO2	-0.18769	-0.58577	-0.46076	-0.15777	-2.07031	-1.04031	-0.11233	3.056429	0.616994	0.224751	-1.88107	-0.50637	-0.13251	-0.01954	3.489756	-1.78222	1.285945	-0.8367	-0.0132
Q9UK41	VPS28	-0.0644	-0.36514	0.115019	-0.31273	-0.20137	-0.17739	0.833784	-0.01943	0.410561	0.061098	0.107712	0.026288	-0.39032	0.273623	-0.56295	0.029351	0.058919	-0.09814	0.043449
Q9UK45	LSM7	0.436463	-0.2756	-0.71751	0.175009	0.234553	0.815917	-0.31962	1.017005	0.084122	0.167906	0.735112	0.071264	-0.19531	0.17033	-0.10903	0.465013	-0.06275	0.26933	-0.18952
Q9UK53	ING1	0.416585	-0.22551	-0.5984	-0.4102	-0.70452	-0.33246	-0.32368	-0.18201	-0.38235	-0.21522	0.520531	-0.40869	-0.21425	-0.21559	-0.25625	-0.34769	0.31103	-0.02212	0.08621
Q9UK55	SERPINA1	-1.31293	-0.14004	-1.39984	-0.54648	-0.69259	-1.50717	-0.69722	-0.62242	0.55244	-0.68461	-0.50924	0.711447	-0.41968	0.800322	-0.44173	-1.21882	0.552464	-1.07639	-0.31893
Q9UK59	DBR1	0.623119	-0.43802	0.481422	0.209159	-0.21522	0.518373	-0.74482	0.428569	1.598028	-0.11056	0.026873	-0.2105	0.174423	0.064654	1.353565	-0.04554	-0.05767	-0.07838	0.16846
Q9UK61	FAM208A	0.478401	-0.18009	-0.01209	-0.27972	0.342437	0.671865	-0.14421	0.769985	-0.06501	-0.0302	0.276853	-0.16511	-0.46733	0.022313	0.057087	-0.34016	-0.08016	0.555491	-0.15085
Q9UK73	FEM1B	-0.65845	0.165266	0.051656	0.734265	0.24053	0.275035	-0.11576	-0.21297	0.285269	0.088844	0.575121	-0.60729	0.073604	-0.24918	0.132848	0.029784	-0.40512	0.415259	-0.10576
Q9UK99	FBXO3	-0.48971	-0.38513	0.204244	0.452223	0.13345	0.035875	0.219696	-0.40125	1.155668	-0.38888	0.01677	-0.03231	2.045836	0.241827	0.640423	-0.16906	-0.20866	0.182528	-0.56395
Q9UKA4	AKAP11	-0.07544	0.498359	-0.20611	-0.1642	0.050997	0.112265	-0.17657	-0.12907	0.029248	0.484466	-0.00177	-0.02866	0.441843	-0.13346	0.639713	-0.76775	-0.10899	-0.20002	-0.41902
Q9UKA9	PTBP2	0.583259	-0.61404	-1.41996	-0.82392	-1.39878	-1.95601	0.556496	1.117152	0.220455	-2.25524	-1.36224	-1.09715	-0.5619	-0.37754	-1.23387	-1.32526	-0.64189	-0.59057	0.45502
Q9UKB1	FBXW11	-0.07138	0.2717	0.134198	0.531368	0.444157	-0.23489	0.190048	-0.16834	0.206423	-0.22665	0.032383	-0.0739	0.16151	0.248528	-0.07109	0.093827	0.462764	-0.34001	-0.16967
Q9UKD1	GMEB2	0.494501	-0.19733	-0.03792	-0.20543	0.213842	0.008015	-0.52889	0.746568	0.156186	0.11754	0.263837	0.409026	0.006746	0.233087	0.817836	0.042228	-0.14476	0.290908	-0.23041
Q9UKD2	MRTO4	0.65626	-0.4323	0.0071	0.216614	0.302818	-0.45456	-0.21058	0.558485	-0.0871	0.187949	0.4654	-0.44232	-0.18317	-0.55263	-0.43261	0.294044	0.115483	-0.46361	-0.31188
Q9UKE5	TNIK	0.555004	-0.12967	-0.83467	-0.815	0.008373	0.446998	-0.32988	0.815413	0.41041	1.154354	-1.01615	-0.28644	-0.02979	-1.0774	-1.61103	-1.0185	0.433231	-0.45846	0.665477
Q9UKF6	CPSF3	0.523171	-0.03913	-0.02508	0.070964	0.065782	0.312644	-0.92917	0.317856	0.010645	-0.16776	0.383934	-0.23085	-0.09756	-0.18646	0.389526	0.480006	0.154229	0.029031	0.082222
Q9UKG1	APPL1	0.112968	-0.20452	0.434567	-0.87963	-0.02047	-0.27529	-0.28883	-0.16277	0.192922	0.014213	-0.149	-0.13596	-0.18893	0.049273	-0.01478	-0.52435	0.026643	-0.12126	-0.25535
Q9UKG9	CROT	-0.60408	0.402162	0.857211	0.018807	0.005277	-0.11532	0.360154	0.744342	0.080986	0.640496	0.40785	0.145696	0.085105	-0.3673	-0.27745	0.325618	-0.25991	-0.60047	-0.03873
Q9UKI8	TLK1	0.803312	-0.23918	0.124386	0.161906	0.279191	0.102345	-0.4575	0.367496	0.046454	0.028995	0.356924	-0.32231	-0.36121	-0.28157	-0.02655	0.156315	-0.23456	-0.0758	-0.20096
Q9UKJ3	GPATCH8	0.147898	-0.39284	0.278129	0.361523	-0.93296	-0.07393	-0.77259	0.180546	-0.88548	0.059598	0.051325	-0.83126	0.075485	-1.07104	0.320953	0.163761	-0.25863	0.324683	0.035987
Q9UKK3	PARP4	-0.34879	0.17161	0.273373	0.044875	0.078355	0.235027	-0.21659	-0.77415	-0.14213	-0.11308	0.112048	0.305322	0.133999	0.550875	-0.64307	0.717335	-0.11805	-0.56625	-0.01405
Q9UKK9	NUDT5	0.774352	-1.18546	-0.36935	-0.09817	1.800891	0.141459	0.104607	0.468041	1.129204	0.165262	-0.40854	0.747704	1.321779	-0.07154	0.033056	0.261871	0.303721	-0.57349	-0.30562
Q9UKL0	RCOR1	0.112852	0.431268	0.014773	0.739334	0.675082	1.040773	-0.08807	0.624217	-0.32485	-0.1378	0.338485	-0.17768	-0.04166	-0.34255	0.132697	0.784079	0.129724	0.436014	-0.41158
Q9UKM7	MAN1B1	0.05937	0.270711	1.053385	-0.32782	0.155041	0.120034	0.558072	0.051353	0.462714	-0.06051	0.060165	-0.22429	0.002964	0.030944	0.256207	0.262284	0.307032	0.440009	0.374543
Q9UKM9	RALY	1.52832	-0.54125	0.023667	0.090255	0.231929	0.479637	-0.16893	0.831125	-0.55388	0.037231	-0.28779	0.065008	-0.66631	-0.15572	0.33925	0.051939	-0.14159	-0.04001	0.151529
Q9UKN8	GTF3C4	0.793184	-0.36601	-0.1653	0.154692	0.128643	0.081711	0.004684	0.791815	0.100467	-0.38925	0.224525	-0.30283	-0.38067	-0.25371	0.128163	-0.32387	-0.50276	-0.28355	0.041768
Q9UKR5	ERG28	-0.79078	-0.48316	-0.2923	0.293505	-0.34457	-0.49498	-0.28861	0.455236	-0.22159	-0.44783	0.452821	-0.49216	-0.07157	-0.53091	-0.3323	-0.18989	0.230389	-0.79162	0.313389
Q9UKS6	PACSIN3	0.558159	-0.20458	-0.65589	0.417231	-0.72042	-0.44396	-0.00432	0.094789	-0.48316	-0.66073	-0.54127	-1.10234	0.481603	-0.19443	0.510737	-0.9751	0.238133	0.332343	-0.10907
Q9UKT5	FBXO4	0.406966	0.258713	-0.20529	-0.46672	0.116495	0.10455	0.127173	-0.75269	0.027599	0.089276	0.128062	-0.68187	-0.08496	0.296122	-1.45351	-0.07772	-0.27502	-0.37375	-0.10547
Q9UKU7	ACAD8	0.981482	-0.47152	0.150033	0.35092	-0.33768	-0.71814	0.178943	-0.11444	0.6966	-1.29436	0.505412	0.567804	1.454263	-0.22844	0.802381	-0.69775	-1.31535	-0.6719	-0.46069
Q9UKU9	ANGPTL2	-1.26511	-0.73829	-0.99694	-1.10028	-1.16461	-1.73147	-0.62367	-1.256	0.037276	-0.8003	-0.69466	-0.67592	0.145707	-0.54972	-0.47713	-0.90782	-1.19284	-0.59791	0.424993
Q9UKV3	ACIN1	0.712382	-0.42551	-0.01835	0.124267	-0.42584	0.155427	-0.8934	0.767592	-0.60598	-0.12346	0.478215	-0.92258	-0.12885	-0.66305	0.289817	-0.09684	-0.40805	0.291311	0.026769
Q9UKV5	AMFR	-0.11675	0.440017	0.413042	0.030243	0.188621	0.032874	0.441984	-0.43908	0.857228	-0.41024	-0.09488	-0.14782	0.162561	-0.0164	0.449652	-0.46212	-0.11877	0.581624	-0.16953
Q9UKV8	AGO2	0.386498	-0.65133	-0.07778	-0.46897	-0.12398	-0.22438	-0.61161	-0.32613	0.717329	-0.8578	-0.25369	0.076316	0.333613	0.064020	-0.04696	0.044835	0.18495	0.04779	-0.46709
Q9UKW4	VAV3	-0.43476	0.372985	0.23307	-0.30717	0.220125	-0.51756	0.006462	-0.6197	0.046351	0.15063	0.606733	1.443166	0.001473	1.221434	0.399527	-0.05598	0.196173	-0.69685	-0.23993
Q9UKX2	MYH2	-1.52289	-2.72548	-0.19477	-1.26028	-10.9361	-1.7352	0.15179	-5.85115	-3.10239	-2.47428	-3.66561	-4.96185	-3.53895	-1.63642	-2.73553	-2.64482	-4.81755	-3.20089	-5.84265
Q9UKX5	ITGA11	-1.12616	-0.97791	-0.19718	-0.80646	-1.34088	-0.85784	-0.28953	-1.31152	0.492687	-1.69242	-1.61651	-1.45361	-0.67202	-0.78959	-1.13133	0.981189	-1.52634	-1.19023	-0.71506
Q9UKX7	NUP50	-0.06053	-0.14173	0.145519	-0.77691	0.148128	-0.01749	-0.18689	0.194166	0.274359	0.058744	0.035811	-0.21942	-0.1283	-0.25242	-0.36712	-0.61639	1.841322	3.047441	-0.36679
Q9UKY7	CDV3	-0.36979	0.627366	-0.35438	-0.33922	-0.35004	-0.53809	-0.57134	-0.67688	-0.01804	0.728006	-0.86275	0.107643	0.62872	-0.29099	-0.41056	-0.14418	0.325383	0.044368	-0.32428
Q9UKZ1	CNOT11	0.27855	0.226954	0.304822	-0.02064	0.230355	0.181183	0.300114	-0.05189	-0.11042	-0.28088	0.013776	0.397694	-0.03072	-0.35943	0.059913	0.243625	0.398872	0.043501	0.057975
Q9UL03	INTS6	-0.2784	0.18261	-1.17596	-0.24657	-0.3323	-0.2331	0.474342	0.720418	-0.83811	-0.0732	0.526964	-0.24731	-0.53902	-0.1115	0.841787	-0.54952	-0.03159	0.26973	0.201414
Q9UL12	SARDH	0.851795	-0.01682	-0.3183	1.428816	-0.74866	-0.51789	-0.80168	-0.65602	-0.07842	0.815982	0.167125	-0.8605	-0.2794	-0.01015	0.142258	-0.32031	-0.37245	0.073681	-0.64731
Q9UL15	BAG5	0.47076	-0.04725	-0.47498	0.546528	0.36101	0.678157	-0.25544	0.514278	-0.08016	-0.12199	0.405541	-0.35995	0.025217	-0.12598	-0.02628	0.041572	0.199029	-0.44324	0.285465
Q9UL18	AGO1	0.040237	-0.20914	-1.04889	0.076599	-0.14707	-0.26238	-0.62072	-0.65739	-0.45968	-0.60719	0.173378	-0.61886	-0.56492	-0.01087	-0.04809	-0.25157	-0.47266	-0.69926	-0.12654
Q9UL25	RAB21	-0.00049	0.355137	0.09849	0.223997	-0.03211	-0.28752	0.405056	-0.01442	-0.06421	-0.0086	0.15706	0.352768	-0.00631	0.724526	0.502515	0.534063	0.362127	0.163524	-0.10951
Q9UL26	RAB22A	-0.33758	0.313452	0.125497	0.025085	0.245927	-0.20697	-0.74031	0.223409	-0.16158	0.367281	-0.60049	0.266779	0.009534	0.166982	0.346178	0.542924	0.298972	-0.04461	-0.10435
Q9UL33	TRAPPC2L	-0.25568	0.529336	0.213761	-0.04998	0.424786	-0.11847	-0.202	-0.34435	1.155668	0.17362	-0.0513	0.426311	0.228942	0.664168	0.098633	0.165639	-0.01988	-0.0545	-0.21582
Q9UL42	PNMA2	1.154724	-0.32472	0.214817	0.859723	0.422144	0.561368	-0.58804	-0.97596	0.005519	1.465604	1.707651	-0.29784	0.399616	-0.25916	-1.67679	2.031325	-0.1505	0.536212	1.032
Q9UL46	PSME2	-0.09202	-0.39771	0.119308	-0.0949	1.119599	0.364165	-0.70985	0.279158	0.056826	0.448826	-0.14261	-0.35688	-0.18175	-0.33111	-0.92241	1.130651	-0.36956	-0.1656	-0.73505
Q9UL54	TAOK2	0.048518	0.725822	0.217407	-0.31609	0.160747	-0.02647	0.268197	-0.31791	-0.0607	0.197873	0.257381	-0.05688	0.137271	0.045507	-0.03921	0.267293	0.256191	0.108023	-0.07911
Q9ULA0	DNPEP	-0.30979	-1.14397	-0.59331	-0.32034	0.299497	0.451428	-0.93522	-0.1441	-0.39484	-1.23281	0.554501	-0.36572	-0.57138	-0.57639	0.674135	0.439297	-0.09853	-0.53748	-0.47247
Q9ULC3	RAB23	0.838109	-1.19659	-0.98901	-0.32458	-1.12024	-1.27586	-0.20692	-0.52002	-0.06233	-1.45934	-0.15962	-0.66733	0.647914	-0.203	-0.39271	-0.15653	0.279142	0.41297	1.193808
Q9ULC4	MCTS1	-0.65808	-0.44917	-0.1755	-0.50686	1.085264	0.208256	-0.37423	-0.03495	0.347679	0.796043	-0.1898	0.412161	0.335133	0.635762	-0.4449	-0.20688	0.997063	0.442717	-0.50877
Q9ULC5	ACSL5	-1.13964	0.209505	0.206134	-0.42877	0.134602	0.011857	0.033358	-0.32782	-0.09331	0.22214	1.106055	0.314658	-0.16356	0.278276	0.663225	-0.6036	-0.11448	-0.49912	-1.08746
Q9ULD0	OGDHL	-0.21668	-0.13181	0.12285	0.621954	0.402917	0.221673	-0.5527	1.172768	-0.30133	0.1089	0.592217	-0.90751	-0.08338	-1.02054	0.924166	1.526074	-1.02746	0.051102	-0.13209
Q9ULD2	MTUS1	-0.35478	0.78609	0.44318	0.095532	-0.29074	-0.8149	0.512873	-0.73571	-0.0372	-0.26119	0.393398	-0.23558	-0.29371	0.996157	-1.10106	-0.59263	0.310992	-0.0017	-0.20682
Q9ULD9	ZNF608	0.366223	-0.04707	-0.1986	-0.18683	-0.50445	1.199863	0.46756	-0.5699	0.244621	-0.4331	0.633065	-1.30185	-0.10467	-0.3686	1.565199	-0.79249	-0.33757	-0.12235	-0.2381
Q9ULE0	WWC3	-0.84697	-0.22736	-0.50833	-0.32084	-1.00843	-0.58026	-0.35306	-1.24389	0.046574	-0.30944	-0.50853	-0.81804	-0.47412	0.303223	-0.64516	0.136174	0.012628	-0.68773	-0.65127
Q9ULE6	PALD1	-0.41956	-0.12381	0.209748	-0.51516	0.75962	1.05053	-0.77072	0.764348	0.119372	0.626309	-0.10557	-0.37045	-0.22738	-0.35261	0.778139	0.074054	-0.84429	-0.36999	0.157943
Q9ULF5	SLC39A10	0.596364	-0.43249	-1.33298	-0.0072	-1.51544	-0.76902	-0.78063	-0.57002	0.250167	-0.29437	0.636388	-1.49757	0.087022	-0.58345	0.078003	-0.16626	-0.87616	-0.4734	0.71705
Q9ULG1	INO80	0.178138	0.123358	-0.1086	0.150109	-0.17497	0.159496	0.308599	0.326059	0.08106	0.01914	-0.37261	0.550374	-0.12358	0.019073	-0.13713	-0.09401	0.153056	0.509661	-0.14818
Q9ULG6	CCPG1	-0.35843	0.168379	0.697239	-0.61222	-0.53237	-0.21145	0.529262	-0.58516	0.365487	-0.80129	-0.41409	0.212304	0.130865	0.569467	-0.15604	-0.5402	0.206131	1.274972	0.476951
Q9ULH0	KIDINS220	0.64554	-0.29775	-0.51561	-0.23964	-0.5011	-0.75546	0.497538	0.177845	0.34128	0.155491	-0.16888	-0.2932	-0.28176	-0.52941	1.351508	0.345114	-0.28586	-0.47574	0.151411
Q9ULH1	ASAP1	-0.20715	0.330384	0.009184	-0.55683	-0.53518	-0.638	-0.36113	-0.59047	-0.11336	0.860619	-0.64904	0.071562	-0.01365	0.794946	0.432369	-0.17068	-0.05811	0.041668	-0.31003
Q9ULH7	MKL2	0.698368	-0.29817	0.091596	-0.33866	-0.12634	-0.25556	-0.79662	-0.471	-0.12738	0.13541	0.326605	-0.79279	-0.11243	-0.21123	0.565496	0.261958	-0.39711	0.735024	0.004699
Q9ULI0	ATAD2B	0.222887	0.381129	0.312245	-0.0813	0.805998	0.312366	0.49271	-0.22905	0.293855	-0.19763	-0.25662	-0.1285	0.268121	0.287125	-0.25865	-0.03608	-0.33186	-0.28934	0.107013
Q9ULJ6	ZMIZ1	-0.19795	-0.22586	0.435142	-0.22057	-0.19221	0.177889	-0.01744	0.961287	-0.15343	0.20803	0.251076	-0.52348	-0.20388	-0.54683	0.811639	-0.3551	-0.06715	-0.19836	-0.0091
Q9ULJ8	PPP1R9A	0.6743	-0.39003	-0.76717	-0.93103	0.093433	-0.11008	-0.33682	0.599479	-0.24594	-0.37667	0.361413	-0.8849	-0.37963	-0.49143	1.723319	-0.53842	-0.6895	-1.30022	-0.70627
Q9ULK4	MED23	0.595872	-0.06686	0.07732	0.567965	0.054757	0.278035	-0.01736	0.381541	-0.38324	-0.5884	0.043639	-0.22049	-0.0784	0.162994	0.256796	-0.17868	0.260014	-0.15055	0.011115
Q9ULK5	VANGL2	-0.28574	-0.60582	-0.65351	0.032588	-0.5691	-0.74811	-0.36248	0.50113	-0.44044	-0.19557	0.628697	-1.92458	-0.65643	-0.96154	0.726411	-1.27014	-0.73334	-1.1538	-0.71053
Q9ULL1	PLEKHG1	0.873685	-0.30712	-0.15075	-0.88423	-0.48413	-0.64374	-0.31208	-0.00392	0.025659	-0.38146	0.219194	-0.94962	0.256671	-0.89114	-0.25493	0.951471	-0.84685	0.185996	1.117021
Q9ULL5	PRR12	0.575522	-0.33848	-0.34843	0.024699	-0.62909	-0.20933	-0.04678	0.197201	0.407527	-0.49124	-0.79148	-1.01591	-0.12863	-0.54022	1.238041	-0.59003	-0.33779	0.027184	-0.33632
Q9ULL8	SHROOM4	-0.09986	0.165135	0.195507	-0.16995	0.121044	-0.46308	-0.15311	-0.60935	-0.01877	0.06592	0.015999	-0.56691	-0.15551	-0.38181	-0.77805	-0.04369	1.180571	1.69879	0.389471
Q9ULM3	YEATS2	0.896792	-0.48924	0.152966	0.230502	0.399433	0.05644	-0.15942	0.280152	-0.17417	-0.15376	0.260711	-0.22408	-0.72918	-0.06533	0.351052	0.189616	0.221601	-0.34432	0.075611
Q9ULP9	TBC1D24	1.272202	-0.01806	-0.44095	-0.57867	-0.69802	-0.65211	-0.07365	-0.04045	-0.64205	-0.20139	-0.16237	-0.54778	-0.38812	-7.5E-05	0.578973	-0.61046	0.035322	-0.16423	0.030882
Q9ULQ1	TPCN1	-0.72325	0.369324	0.267724	-0.47264	0.524858	-0.18767	0.361994	-0.59082	-0.47672	0.361068	0.060365	0.978862	0.044632	0.562308	0.846372	0.596152	0.24653	0.511966	-0.09854
Q9ULR0	ISY1	0.309253	-0.41448	-1.35737	-0.13429	0.496704	0.065784	-0.48998	0.325197	-0.90568	-0.17701	0.389833	-0.47311	-1.0251	-0.14657	-0.37559	0.383478	0.055933	0.463251	-0.07479
Q9ULS5	TMCC3	0.716451	0.044647	0.195064	0.066941	-1.18759	-1.21746	-0.27855	0.192715	-0.12654	-1.46172	-1.58246	-0.44982	0.25112	0.07498	2.533532	-1.9546	-0.18123	-1.06517	-0.02817
Q9ULT0	TTC7A	-0.16102	0.287759	0.333807	-0.60527	0.596865	0.13482	0.187999	-0.77943	-0.38199	0.411965	-0.28794	0.620207	0.037978	-0.4133	0.151768	0.529746	0.131768	0.678323	-0.39286
Q9ULT8	HECTD1	-0.69384	0.293618	-0.18239	-0.77059	-0.00161	0.422403	0.375887	0.047897	-0.17285	-0.07971	-0.14492	-0.02337	0.57961	0.047136	0.213512	0.07836	0.619367	0.362895	0.713186
Q9ULU4	ZMYND8	0.967202	-0.05662	0.051853	0.084432	-0.23234	0.055133	0.836454	1.011695	-0.69903	-0.46487	0.034605	-0.50706	-0.60255	-0.74739	0.267519	-0.15578	0.06773	0.204904	-0.08239
Q9ULV0	MYO5B	-0.54764	-0.20173	0.077354	0.265423	-0.56658	-0.54782	0.286661	0.095257	0.013536	0.32772	0.596293	-0.53021	-0.69485	-0.33863	-0.76853	0.069003	-0.29155	-1.25451	-0.47562
Q9ULV3	CIZ1	-0.18054	-0.29085	-0.2671	-0.15687	-0.56512	-0.50047	0.438253	0.091571	0.066571	-0.77454	0.063672	-1.77861	-0.31103	-0.78506	0.051559	-0.67421	-0.13477	-0.22586	-0.38928
Q9ULV4	CORO1C	-1.25757	1.020899	0.889151	-0.14976	0.184329	0.218311	-1.08487	-0.53437	0.376622	0.265501	-1.37374	-0.30164	0.812957	-0.31114	-0.86191	-0.30066	-0.03154	0.696145	0.309385
Q9ULW0	TPX2	1.634164	-0.27314	0.048726	-0.4291	1.286661	1.876879	0.794304	1.652784	0.204781	0.38659	-2.16392	-1.14385	-0.0026	-1.24659	-0.80592	-0.12618	-0.65003	-1.00253	0.78779
Q9ULW3	ABT1	0.799696	-0.29036	-0.06558	0.54903	-0.07563	0.016685	-0.148	0.073102	-1.0737	0.397595	-0.18462	0.229688	-0.74353	0.246361	-0.27199	-0.16349	-0.1266	-0.22106	-0.38418
Q9ULX3	NOB1	-0.19362	-0.3096	-0.07556	0.207871	-0.1027	0.404376	0.441656	-0.36563	0.07795	0.758098	0.217737	-0.39933	-0.00535	-0.03256	-0.21017	0.331432	-0.22994	-0.47553	0.05211
Q9ULX6	AKAP8L	0.161365	0.01462	0.068139	0.159528	-0.36431	-0.13024	-0.23797	0.583337	-0.58324	0.014601	0.329819	-0.49701	-0.19487	-0.5372	1.035233	-0.40612	0.17067	0.603266	-0.30408
Q9ULZ3	PYCARD	-0.1607	0.657749	0.867327	-0.3809	0.991495	0.485994	-0.54085	-0.85019	0.094452	1.121998	-0.47958	0.522827	-0.03068	1.070622	-1.29511	-0.56662	-0.10341	-0.43864	-1.01789
Q9UM00	TMCO1	0.435298	0.121318	0.08973	0.652711	0.103166	0.523184	-0.01155	0.712088	-0.06369	0.062479	-0.48257	0.639665	0.012625	0.073975	1.461362	-0.41556	0.808709	0.264016	-0.46745

Q9UM07	PADI4	-2.65516	-0.24888	1.624527	-3.20201	-0.79672	-2.4524	-0.33718	-3.34988	0.418924	1.408992	-2.35279	1.054594	-0.86831	2.394045	-0.88896	-2.11289	1.646052	-2.26232	-1.38352
Q9UM47	NOTCH3	-0.49769	-0.42507	0.27408	0.216879	-0.71084	-1.52769	-0.17293	0.64352	-0.14709	-0.29899	-0.81731	-1.21975	-0.43159	-0.6741	0.113349	0.640496	-0.69568	-0.32922	0.647037
Q9UM54	MYO6	-0.41297	-0.60767	0.496734	0.650847	-0.79448	-0.18756	-0.44711	-0.06671	-0.29112	-0.36994	0.176984	-0.19782	0.494301	-0.17863	0.475742	-0.06485	1.03146	-0.03819	-0.17938
Q9UMN6	KMT2B	0.288949	-0.02647	-0.26933	-0.45705	-0.48127	-0.14202	0.14754	0.780958	-0.32508	-0.56601	-0.12527	-0.30275	0.042804	-0.14719	1.069039	-0.52897	-0.06245	0.06731	0.276263
Q9UMR2	DDX19B	-0.26302	0.20073	0.403589	0.740242	-0.00188	0.274819	0.061644	-0.72027	0.408607	-0.04239	0.121361	0.13922	0.58374	0.300305	0.397905	-0.37976	0.188914	-0.27811	-0.03424
Q9UMS0	NFU1	-0.64617	0.054669	0.129194	0.17875	0.707014	0.766803	-0.0859	0.015139	0.035661	0.218566	-0.32915	-0.38338	0.048737	-0.27173	0.467098	0.401795	-0.93673	-0.44381	-1.00278
Q9UMS4	PRPF19	0.344045	-0.46854	0.211668	0.141988	0.351611	0.206198	-0.04189	0.813069	-0.43919	-0.36185	0.390421	-0.16408	-0.34612	-0.18399	-0.0714	-0.00729	0.327023	0.195344	-0.0126
Q9UMS6	SYNPO2	-2.7577	-1.10203	-0.47647	-3.27962	-1.17635	-2.70565	-0.60298	-4.33694	-0.3041	-1.01414	-0.42229	-1.33528	-2.35026	-1.04104	-0.41179	-1.7838	-1.05861	-0.54844	-1.14647
Q9UMX0	UBQLN1	-0.15526	0.044389	0.460839	-0.68403	-0.09112	0.61699	-0.16114	-0.1878	0.283945	0.061086	0.152099	-0.18929	0.068757	-0.16659	-0.29848	0.1841	-0.02852	-0.11493	-0.03643
Q9UMX1	SUFU	0.199353	0.415316	0.645751	-0.13375	0.187318	-0.00733	-0.01387	-0.07968	0.091349	0.28612	0.198406	0.180437	0.326973	0.118009	-0.42805	0.119126	0.100801	0.554963	1.143739
Q9UMX5	NENF	-0.79337	0.26718	-0.87023	-0.62363	0.450395	-1.21012	-0.41729	1.343238	-0.10298	0.351954	-0.26032	-0.49655	-0.24919	-0.26558	0.24466	0.193737	-0.00142	0.112562	-0.17437
Q9UMY1	NOL7	0.774684	-0.15331	-0.14407	0.92169	0.151709	0.068993	-0.2892	0.145982	-0.2669	-0.24657	0.361561	-0.23071	-0.1445	-0.73194	0.147692	0.1478	-0.03637	-0.75608	0.030272
Q9UMY4	SNX12	0.198715	0.196407	-0.19244	-0.37552	0.465671	0.11811	-0.97664	0.779749	0.620951	0.890018	-0.2216	0.202372	0.382398	-0.27047	-0.62836	-0.04181	0.280538	0.330938	0.456989
Q9UMZ2	SYNRG	-0.0988	-0.34013	-0.04672	-0.10166	-0.88315	-0.59008	-0.52272	0.17916	0.015632	0.241045	0.215459	-0.35621	0.073917	0.058455	0.421726	0.04424	-0.5014	0.080625	0.280524
Q9UN19	DAPP1	-0.58922	1.199727	1.25136	-0.61362	-0.44063	-0.39117	0.335819	-0.5287	-0.17809	0.254893	0.138936	0.604215	0.26331	1.003614	-0.69099	-0.52699	0.168029	-0.4549	-2.13906
Q9UN36	NDRG2	-0.55376	0.013227	-0.89247	-0.80546	-0.21744	0.034003	-0.75575	-0.93142	-0.32194	0.099803	-1.30314	-2.05744	-0.34384	-0.77956	0.037863	-0.76636	-0.1776	-0.30668	-0.42178
Q9UN37	VPS4A	-0.46136	0.378853	0.392801	-0.04992	-0.21811	0.002402	-0.31676	-0.84077	-0.24029	-0.06362	0.086378	0.34599	-0.31083	0.407285	0.026501	0.140948	0.341626	0.221661	-0.00484
Q9UN70	PCDHGC	-0.58667	0.47227	0.037122	-0.43943	0.132904	-0.20819	0.342825	-0.71923	0.372282	-0.3239	0.283467	-0.15613	0.060267	0.417651	-0.47148	0.080773	-0.58399	0.735916	1.297529
Q9UN79	SOX13	0.109525	-0.35122	-0.28101	0.291736	-0.0565	0.38437	-0.48605	0.58487	-0.32534	-0.39545	0.770311	-1.62734	-0.04483	-0.87783	0.516728	0.132254	-0.81011	-0.11536	-0.97287
Q9UN81	L1RE1	-0.19346	-1.15153	1.057403	-1.58273	0.58657	0.466627	-1.05946	0.541811	-0.34951	-0.86531	-2.10014	-0.25036	1.903422	-0.49928	-0.77661	2.192809	0.724057	0.8691	1.085473
Q9UN86	G3BP2	0.647124	-0.49904	0.607475	-0.46662	-0.66809	0.129687	-0.25654	0.210846	-0.36707	0.454585	0.136337	-0.49025	-0.35757	-0.28422	-0.24053	-0.49513	-0.18936	-0.0683	0.507524
Q9UNA1	ARHGAP2	-1.12343	-0.08099	0.049381	-0.32238	0.865323	1.123717	0.138174	-0.21213	0.147108	0.481752	0.814583	0.57354	-0.34152	0.860671	0.024435	-0.56107	-0.3515	-0.65907	-1.33747
Q9UNA4	POLI	-0.49359	0.521556	-0.00828	1.418435	-0.48582	-0.71727	-0.06964	-0.69921	0.040847	-0.18384	0.490732	0.010083	0.245997	-0.45733	-0.6285	-0.46732	-0.37241	-0.2586	-0.14402
Q9UNE7	STUB1	1.054498	-0.13365	-0.36371	-0.45865	-0.46786	-0.33753	-0.44708	-0.66717	-0.17467	-0.42187	-0.07263	-0.38416	0.1346	-0.25801	-0.05608	0.523044	0.443125	0.292788	-0.49712
Q9UNF0	PACSIN2	-0.32555	0.358219	0.393118	-0.33471	0.003773	0.186454	-0.55661	-0.56561	-0.20731	0.17098	0.466142	-0.09288	0.439612	-0.32946	-0.82653	0.02946	0.647231	1.336313	-0.39306
Q9UNF1	MAGED2	0.669457	0.279178	-0.67882	0.042135	-0.46923	0.064466	-0.21235	0.09712	-0.55806	-0.14418	-0.51501	-0.98491	-0.27832	-0.74102	-0.55173	-0.73699	0.074831	0.047893	0.758968
Q9UNH6	SNX7	0.916852	-0.0839	0.019475	0.274613	0.782312	0.586755	1.048872	-0.36108	-0.64981	-0.02559	0.696624	-0.35769	0.007116	-0.54641	-1.56391	0.608638	0.162277	-0.15142	0.356896
Q9UNH7	SNX6	-0.9164	0.450841	1.122829	0.20298	0.359051	-0.01599	0.081554	-0.31117	-0.01682	0.164557	-0.36598	0.116075	0.068581	0.144014	0.450889	0.413611	-0.04166	0.398222	0.203336
Q9UNI6	DUSP12	-0.22847	0.171151	-0.0726	-0.35063	-0.51615	-0.25519	0.146869	0.258069	-0.32145	-0.3151	0.365812	-0.04511	-0.05347	0.308402	1.309489	0.526719	-0.10461	-0.49363	-0.01846
Q9UNK0	STX8	-0.25024	1.454247	0.488195	0.023439	-0.09828	-0.23564	0.602701	-0.23767	-0.21633	0.753067	-0.12174	0.545282	0.090437	0.586917	-0.34828	-0.47355	-0.18908	0.200316	0.416826
Q9UNL2	SSR3	0.159618	0.639034	0.082486	0.457386	0.336293	0.120013	1.20464	-0.45943	0.10088	-0.28466	-0.01389	0.126534	0.261962	-0.24609	0.959397	0.188999	0.01307	0.404766	0.157154
Q9UNL4	ING4	0.601806	-0.08711	0.028058	-0.52754	0.292954	0.224081	0.066184	1.263623	0.057086	0.429466	-0.24683	-0.77708	-0.20878	-0.11133	0.23701	-0.52646	-0.26157	0.013571	-0.54708
Q9UNM6	PSMD13	-0.16196	0.091796	0.404455	0.049569	-0.02762	0.093761	0.167553	0.07062	-0.23578	0.085535	0.077269	0.246714	-0.23195	0.263661	-0.20407	0.377532	0.264969	-0.20494	0.355832
Q9UNN5	FAF1	0.991234	-0.30924	-0.02838	0.241417	0.687613	1.117484	-0.39089	0.289469	-0.23613	0.305887	0.343734	-1.04985	-0.07252	-0.44439	0.609169	1.223231	-0.19361	-0.19503	-0.52728
Q9UNP9	PPIE	0.261992	-0.08104	-0.14772	-0.07126	0.459503	0.456917	-1.01069	0.261678	-0.00039	-0.11146	0.343235	-0.37561	0.114012	-0.4984	-0.21119	-0.06169	0.13446	0.50059	0.145311
Q9UNQ2	DIMT1	0.229737	-0.15663	-0.15739	0.395994	-0.13474	-0.19294	-0.10575	-0.09066	-0.13476	-0.2639	0.342388	-0.33182	-0.24888	-0.29676	0.105045	-0.07291	-0.06126	0.46784	-0.13398
Q9UNS1	TIMELESS	1.831982	0.095507	-0.49748	0.098104	0.702327	1.309635	-0.11877	-0.38024	-0.07138	-0.41816	-0.59892	-0.33376	-0.03893	-0.37087	-0.72036	-0.13225	0.080371	-0.37532	-0.41761
Q9UNS2	COPS3	-0.16871	-0.1287	0.199	0.028181	-0.30654	-0.10581	0.312908	-0.12244	-0.58704	-0.17674	0.312502	-0.76857	-0.06422	-0.70496	0.262772	0.174205	-0.47791	-0.4278	0.38944
Q9UNW1	MINPP1	-0.06943	0.011814	0.156334	0.539016	0.151326	0.074721	-0.24243	0.297567	-0.25708	0.197661	0.654489	-0.88781	-0.03092	-0.42655	-0.86886	-0.27286	0.173171	0.40461	0.454608
Q9UNX4	WDR3	0.687386	-0.00149	-0.02542	0.29127	-0.22447	0.058107	-0.37149	0.284901	0.152911	-0.19903	1.025862	-0.20935	-0.24362	-0.24767	0.360361	0.133383	0.153425	0.051462	-0.34587
Q9UNY4	TTF2	0.378287	0.388646	0.9723	-0.07894	-0.03271	0.640505	1.152171	0.301881	0.701821	-0.17063	-0.3269	-0.47347	-0.4136	0.06532	0.503976	-0.14259	-0.16748	-0.2284	-0.42968
Q9UNZ2	NSFL1C	-0.03886	-0.52867	-0.18808	-0.6179	-0.02809	-0.53062	-0.54334	0.968784	0.85513	1.838879	-0.24181	-0.57203	0.299704	-0.59446	-0.50209	-1.43267	-0.13688	0.073379	-0.14154
Q9UNZ5	C19orf53	0.773968	-0.26622	0.794352	0.506444	0.198664	0.154759	-0.2856	1.606723	-0.67106	-1.10515	-1.07024	-0.38996	-0.86571	-0.90212	1.418713	1.230686	-1.13686	0.125395	-0.90693
Q9UP38	FZD1	-0.59747	0.15887	-0.16316	-1.29566	-1.29602	-0.71819	-0.10382	-0.5364	0.755051	-0.80927	-0.49107	-1.02618	0.595439	-0.38331	-0.4756	-0.07261	-2.35567	-0.37321	2.085458
Q9UP83	COG5	-0.63987	0.595325	0.044957	-0.02392	0.078092	0.127922	0.42505	-0.46085	1.06375	0.142537	-0.46501	0.038851	-0.35265	-0.1417	0.986945	0.421558	0.040535	0.129663	0.458859
Q9UP95	SLC12A4	-0.08106	1.017945	-0.05709	-0.10378	-0.31256	-0.42044	1.145891	-0.32064	0.329059	-0.02241	-0.38454	-0.41112	-0.00854	-0.05934	0.019766	0.882766	-0.0112	0.399893	0.747214
Q9UPM8	AP4E1	-0.21851	0.362011	-0.20065	-0.18223	0.083924	0.139477	-0.08259	-0.39517	-0.05194	-0.13295	-0.23754	0.533307	0.009095	0.650365	-0.47988	0.795657	0.820249	0.126639	0.003946
Q9UPN3	MACF1	-0.06395	0.251561	0.170807	0.417003	0.116544	0.230314	0.095177	-0.27235	0.042311	0.494827	0.104265	-0.07349	0.512263	-0.19336	-0.23827	0.244611	-0.41834	-0.1207	0.010247
Q9UPN4	CEP131	0.693616	-0.19909	-0.19778	-0.40478	0.015335	0.155627	-0.47441	-0.08177	-0.27504	-0.56195	0.174223	-0.65823	-0.29904	-0.59518	-0.21884	0.593882	-0.21198	0.08343	-0.97038
Q9UPN6	SCAF8	0.024173	-0.29698	-0.05708	-0.16342	-0.55945	-0.00153	-0.0905	0.077184	0.08708	0.191125	0.428964	-0.9356	-0.05548	-0.51847	0.037807	-0.43698	-0.31766	0.43704	0.24224
Q9UPN7	PPP6R1	-0.42873	0.815339	0.287238	-0.18398	-0.17243	0.106601	-0.00879	-0.68913	0.057417	0.605172	-0.33335	0.23218	-0.1285	0.652917	-0.21513	-1.10791	0.327752	0.110623	-0.2194
Q9UPN9	TRIM33	0.693753	-0.19422	-0.70674	-0.01909	0.493059	0.641316	0.211877	0.715786	-0.61679	-0.28174	0.110681	-0.18151	-0.48193	-0.13766	0.126931	-0.40482	0.060659	0.234206	-0.32375
Q9UPP1	PHF8	0.160288	-0.19298	-0.19963	-0.25535	-0.37061	0.080669	0.028011	0.349623	-0.14469	-0.42098	0.31601	-0.33832	-0.51959	-0.12682	-0.06698	-0.16896	0.363336	0.10962	-0.31309
Q9UPQ0	LIMCH1	-0.03562	-0.57973	-0.15536	-0.04563	-0.80141	-0.6259	-0.28667	0.174443	-0.15279	-0.03875	0.126771	-0.63935	-0.92579	-0.20735	1.175656	0.318424	0.894659	0.203196	-0.03958
Q9UPQ3	AGAP1	-0.17237	-0.34206	-0.25046	-0.91336	-0.02976	-0.37656	-0.45919	0.006113	-0.35459	-1.10314	0.554345	-0.29865	-0.4866	-0.88232	1.198026	0.08229	0.535813	-0.04157	-0.24801
Q9UPQ7	PDZRN3	0.037461	0.401259	0.30729	-0.46998	-0.07891	-0.68093	-0.17852	-1.19606	-0.50461	-0.44317	-0.39927	-0.61251	-0.04896	0.027826	0.092758	-0.11533	-0.75947	0.27812	0.928953
Q9UPQ9	TNRC6B	-0.57608	0.473965	0.060229	-0.79214	-0.21351	-0.01248	0.138291	-0.42645	-0.01258	-0.07272	0.51473	-0.38145	0.646024	-0.67976	0.2211	0.525678	-0.34255	0.172963	0.036174
Q9UPR3	SMG5	-0.12377	0.10641	0.28488	0.051697	0.423527	0.259548	0.413231	0.256096	0.656314	0.421069	0.182664	-0.12509	0.419652	0.189832	0.133736	0.014264	0.637637	-0.36594	-0.31723
Q9UPS6	SETD1B	-0.01335	0.174853	-0.01514	-0.512	0.261185	-0.23293	-0.00687	-0.27761	0.777852	-0.87614	0.241243	0.376878	-0.41363	0.213063	-0.02483	-1.09189	0.270571	0.082592	-0.14594
Q9UPT5	EXOC7	-0.07995	0.361458	0.09526	-0.03572	-0.16631	-0.00284	0.02681	-0.11037	-0.66181	-0.02745	0.157166	0.029157	-0.11012	0.116193	0.199667	-0.0079	-0.12619	-0.06445	0.416673
Q9UPT6	MAPK8IP	-0.64036	-0.02669	-0.88392	-0.51604	-0.54325	-1.95813	-0.75574	-1.10202	0.280582	0.36652	-1.53044	-0.01516	0.953921	0.115206	-1.27539	-0.14367	-0.03249	-0.38526	-0.03016
Q9UPT8	ZC3H4	0.760179	-0.70815	1.009807	0.080967	-0.69585	-0.12627	-2.19474	0.042049	-0.08462	-0.09249	0.167944	-0.93798	0.037423	-0.73014	0.664764	0.043112	-1.13964	0.276856	-0.2692
Q9UPT9	USP22	0.677821	-0.02287	-0.12137	-0.0385	0.004608	0.291804	-0.27316	0.828559	-0.03247	-0.04224	0.286319	-0.74643	-0.17546	-0.21387	-0.01065	-0.08209	0.352197	0.191923	0.87302
Q9UPU5	USP24	0.438481	0.274443	0.232578	-0.23518	0.048591	0.304485	0.156056	0.090113	-0.27341	0.15177	0.143086	-0.04237	-0.23277	-0.01246	-0.04631	0.344537	0.09951	-0.47345	-0.45471
Q9UPU7	TBC1D2B	-0.47472	1.184518	0.607055	-0.89181	0.264686	-0.176	0.07683	-0.71762	0.307885	0.58752	-0.38706	0.012586	0.052599	0.28491	-0.34205	-0.06778	0.135018	0.603233	0.273293
Q9UPY3	DICER1	-0.72079	0.197076	0.125146	-0.3613	0.396525	0.549506	-0.33309	-0.14697	0.143474	0.626101	-0.56912	0.451089	-0.368	0.737945	0.437077	0.015558	0.604712	-0.42859	0.376516
Q9UPY8	MAPRE3	-0.44677	-0.40854	-0.36048	0.031518	-1.49044	-0.92262	-0.37046	-1.13355	0.003967	0.230682	0.866132	-1.3003	0.281524	-0.34312	0.320842	0.256734	-0.76755	-0.22004	0.080237
Q9UPZ3	HPS5	-0.23244	1.245796	1.176338	-0.48532	0.30467	-0.19327	1.592008	-0.09442	0.16161	0.208603	-0.62458	0.424787	0.563417	0.844438	-0.06668	0.197694	0.04105	-0.0419	0.029986
Q9UQ03	CORO2B	-0.52069	-0.11944	-0.1427	-1.27445	-0.71957	-1.62651	0.296578	0.765767	0.148886	-0.32192	0.134877	-0.88107	-0.3364	-0.81275	0.453398	-0.91415	-0.7165	0.392582	-0.1046
Q9UQ13	SHOC2	-0.16681	0.185125	0.156275	-0.6408	-0.23545	-0.02773	0.195527	0.106044	-0.16364	0.175714	0.467566	-0.13053	-0.08557	-0.09263	-0.01275	-0.44006	-0.04309	0.110518	-0.01192
Q9UQ16	DNM3	-1.22783	0.534062	1.124662	-0.89571	-0.24285	-0.29414	0.55979	0.449638	0.297846	0.40584	-0.81577	0.329717	0.260377	0.919641	-0.30598	0.178041	0.388572	0.204447	0.187264
Q9UQ35	SRRM2	0.435968	-0.26929	-0.04141	-0.20466	-0.65463	-0.25752	-0.51898	-0.2128	0.143808	-0.06582	0.18642	-0.67707	0.285008	-0.5929	0.12504	0.013701	-0.14072	0.615946	0.189363
Q9UQ53	MGAT4B	0.238451	-0.08492	-0.21867	-0.38095	2.074038	1.124676	-0.57819	-0.21948	1.450902	1.110068	0.029774	0.103175	-0.05399	0.016549	1.193426	0.812165	-0.03729	-0.27118	-0.89901
Q9UQ80	PA2G4	0.188886	-0.69845	0.037936	0.441866	0.718837	0.9959	-0.77497	0.072158	-0.37982	-0.21944	0.153657	-0.22783	0.084207	-0.13675	0.368044	-0.00737	0.755309	-0.11048	-0.13022
Q9UQ88	CDK11A	-0.1189	-0.2954	0.104861	0.343695	0.851076	0.795503	-0.55702	-0.03826	0.151469	0.791097	-0.27107	0.630996	0.277817	0.537403	-0.18472	0.314911	0.683873	0.265063	-0.46949
Q9UQ90	SPG7	-0.11254	0.023152	-1.34669	0.060882	0.006395	-0.12369	-0.89953	-0.05773	-0.36361	0.350943	0.76194	-0.02416	-0.57155	-0.02106	0.697437	0.77466	-0.49437	-0.44742	-0.4655
Q9UQB8	BAIAP2	0.533835	-0.93954	-0.19611	0.305234	-0.12309	0.315526	-0.47563	0.036409	-0.1399	-1.07052	0.690023	-0.65846	0.310146	-0.58865	-1.0661	1.610472	0.137102	-0.39804	-0.86722
Q9UQC2	GAB2	-0.61702	-0.54782	-0.71582	-0.49626	-0.2505	0.184483	-0.83971	-0.76153	0.040847	-0.86938	-0.24339	0.773909	0.939369	0.895052	-0.23871	0.63157	-0.64678	-0.59549	-0.63281
Q9UQE7	SMC3	0.575379	-0.01422	-0.01569	-0.03447	0.722264	1.066466	-0.0472	-0.10096	-0.30619	-0.18269	0.048085	-0.31848	-0.30783	-0.18721	0.473205	-0.27172	-0.20483	0.130221	-0.31946
Q9UQL6	HDAC5	-0.13735	0.414709	0.133118	-0.36705	-0.5747	-0.1029	-0.10739	-1.11054	-0.29024	0.28482	-0.0669	0.545387	0.04111	0.623479	1.122738	0.127786	-0.25422	-0.23784	-0.43029
Q9UQN3	CHMP2B	0.584951	-0.32338	0.285603	-1.22642	-0.39887	0.544559	-0.0837	-0.02832	-0.14449	-1.05116	-0.4298	0.283207	0.204111	0.451932	0.605784	0.19341	0.533236	0.190194	0.3263
Q9UQP3	TNN	-1.04358	0.100273	0.155058	-1.21772	-1.87058	-1.01289	0.297721	-0.58592	0.632954	0.417599	-1.98014	-2.04305	0.439366	-0.60656	-0.45575	-0.50912	-1.62095	0.051546	1.09939
Q9UQR1	ZNF148	0.366533	-0.16735	-0.20294	0.0428	-0.09575	0.48937	-0.09996	0.673446	0.86233	0.143636	-0.18901	-0.28554	0.370466	-0.1111	0.746497	-0.46211	-0.0861	-0.15464	0.254359
Q9Y217	MTMR6	-0.52508	0.340846	0.497865	-0.00533	-0.22101	-0.00947	0.434746	-0.62364	-0.29186	0.917461	-0.52229	-0.05572	0.29591	0.231692	-0.49612	0.602829	-0.24272	0.021345	0.340938
Q9Y221	NIP7	0.169001	-0.32626	0.013795	0.469723	0.086266	-0.21674	-0.42357	0.464714	-0.14477	-0.27032	0.635308	0.684271	-0.12512	0.076879	-0.07075	0.476881	0.774904	-0.37864	0.169312
Q9Y223	GNE	-0.58252	0.675558	0.075446	0.264666	-0.42477	-0.46458	0.43568	-0.0064	-0.10199	0.055715	0.124236	0.204104	0.337269	0.540598	-0.27747	-0.08789	1.626151	1.330043	0.375351
Q9Y224	RTRAF	0.025551	-0.24938	-0.36344	0.118064	-0.36359	-0.47225	-0.5812	-0.00544	-0.25652	-0.03216	0.707175	-0.51664	-0.15408	-0.62696	0.451031	0.229434	-0.26813	0.000901	0.208551
Q9Y228	TRAF3IP3	-1.29752	1.282411	0.867407	-1.84519	-0.09481	-0.61261	0.959311	-2.06251	0.116231	0.666185	-0.90239	-0.06825	-0.37679	0.863467	-1.2593	-0.81386	-0.1254	0.148056	-1.22398
Q9Y230	RUVBL2	0.548652	-0.33379	-0.73182	0.603649	-0.03842	0.307854	-0.67053	0.195558	-0.48899	-0.348	0.711826	0.050809	0.025489	-0.25612	0.466757	0.045534	-0.03873	-0.5643	-0.09777
Q9Y232	CDYL	0.764478	-0.66789	0.180401	0.636577	0.399136	0.201352	-0.5422	0.488495	-0.43693	-0.42709	-0.25263	-0.32016	-0.01054	-0.95939	0.523519	0.58158	-0.36956	-0.57534	-0.52503
Q9Y237	PIN4	-0.34651	-0.00457	-0.64181	0.488249	0.531481	0.172183	0.185969	-0.22239	0.292528	0.119564	-0.25831	-1.34501	-0.00612	-0.96101	-0.56251	0.187379	-1.54567	-0.20412	0.271319
Q9Y240	CLEC11A	-0.58534	-1.52679	-1.39696	-2.04301	-1.53618	-1.29019	-0.88906	-0.662	0.205606	0.228423	-0.81581	-0.79764	-1.6079	-0.54381	-0.83792	-1.07399	-0.52381	-1.07936	-0.12943
Q9Y243	AKT3	-0.28867	0.553167	0.137282	-0.92666	-0.05398	-0.48305	0.046228	-1.55255	0.38983	1.013029	-0.25202	-0.06739	-0.08289	-1.2484	-0.82975	-0.39375	-0.74662	0.205107	0.590986
Q9Y250	LZTS1	-1.16986	0.032708	-0.57456	-0.45984	-0.19665	-0.61404	-0.31545	-0.61008	0.463973	0.034979	-0.80717	-0.55648	-0.08811	-0.46098	-0.19242	-0.2355	1.954149	3.566223	1.39702
Q9Y259	CHKB	-0.5174	0.173234	0.258234	-0.36148	0.443799	-0.77899	0.183306	-0.69706	-0.04225	-0.48299	-0.51348	0.342627	0.079243	0.186508	0.102006	-0.327	-0.20108	0.620437	0.654692
Q9Y262	EIF3L	-0.28254	0.205854	-0.01539	0.056054	0.016592	0.433169	0.203297	0.177765	-0.51516	-0.10377	0.252647	-0.65368	0.009927	-0.43642	0.433685	0.203287	-0.1517	-0.54605	-0.07144
Q9Y263	PLAA	0.041303	0.045421	0.674438	-0.18663	-0.44062	-0.12061	0.177475	0.333401	-0.47805	-0.47403	-0.10541	-0.30682	-0.55271	-0.08386	0.178597	-0.08653	-0.18287	-0.57801	0.203571
Q9Y265	RUVBL1	0.636183	-0.79543	-0.54961	0.85669	0.069736	0.418647	-0.97271	0.386803	-0.20318	-0.43058	0.792613	0.188397	0.178411	-0.06603	0.603362	0.091514	0.147187	-0.53788	-0.19587
Q9Y266	NUDC	0.787068	-1.27258	0.045291	-0.10275	0.963761	1.019534	-1.09146	0.797402	0.100062	0.750424	0.541725	-0.62777	0.139379	-0.57715	-0.48094	-0.26123	-0.75699	-0.4917	-0.19745
Q9Y276	BCS1L	0.18037	-0.36009	-0.35435	-0.29228	-0.38113	0.064088	-0.16345	-0.61268	-0.33454	-0.39884	0.55341	-0.20697	-0.615	-0.02149	0.951203	0.59134	-0.49342	-0.60983	-0.80859
Q9Y277	VDAC3	0.217856	0.056772	0.031556	-0.17672	0.727365	0.627378	-0.10893	-0.39155	-0.61489	-0.665	0.318594	0.706414	0.79947	0.256665	1.682401	1.078838	0.345903	0.297057	-0.26113
Q9Y279	VSIG4	-1.56034	2.640785	1.028613	-0.92366	0.893378	0.143787	0.811719	-1.33282	0.281258	1.08313	-0.8383	2.535542	0.737994	1.854844	-0.3992	-0.56157	0.374674	-0.04368	-0.43514
Q9Y281	CFL2	-0.10446	-1.16816	-0.05459	-0.39404	0.820012	0.155085	-0.63481	0.032451	0.397911	-0.16007	-1.26368	-0.68128	-0.40783	-0.79904	-0.45115	-0.43402	-0.16562	0.593615	0.189537
Q9Y282	ERGIC3	-0.01707	0.279261	-0.03562	0.442627	0.077917	0.273627	1.218341	-0.30657	0.102712	0.368255	-0.39108	-0.16452	0.048359	0.019022	0.393415	0.193215	0.403697	0.310104	0.337162
Q9Y285	FARSA	0.161816	-0.20716	-0.37482	-0.21245	-0.27529	0.021199	-0.12791	-0.19514	-0.07895	-0.53165	0.420485	-0.6143	-0.27962	-0.28535	0.795841	0.62418	0.129375	-0.28975	-0.39238
Q9Y287	ITM2B	-0.16234	-0.17301	0.245568	0.004666	-0.17833	-0.05573	-0.19726	-0.22063	0.488411	-0.17295	0.79093	-0.13795	-0.07919	-0.28689	0.222172	-0.2013	1.126554	3.780618	0.430326
Q9Y294	ASF1A	0.231963	-0.24508	-0.03918	-0.07304	-0.1006	0.343796	-0.17219	0.107105	0.00084	0.095796	-0.0636	0.11651	0.023452	-0.09994	0.121074	0.215153	0.464969	0.153428	-0.84933
Q9Y295	DRG1	0.139903	-0.69313	-0.40329	-0.44209	-0.17029	-0.14823	-0.57106	0.030648	-0.2468	-0.00589	0.573263	-0.29354	-0.04845	-0.19657	0.322047	0.120801	0.098598	-0.28067	-0.29837
Q9Y296	TRAPPC4	-0.08291	-0.16387	0.34454	-0.08801	0.15468	-0.16439	-0.16652	0.115407	0.376543	0.207581	0.295307	0.201018	-0.05421	0.439514	0.034126	-0.09517	0.386303	0.172516	-0.03328
Q9Y2A7	NCKAP1	0.293096	-0.4271	-0.11769	-0.12746	-0.48196	-0.44942	0.020148	0.218178	-0.21221	-0.25744	0.373075	-0.79158	-0.41969	-0.81744	0.389139	0.444523	-0.04951	-0.34174	0.385686
Q9Y2B0	CNPY2	0.036009	-0.56029	-0.39383	0.303025	0.507291	0.322802	-0.30015	0.248923	0.447097	0.275483	0.500873	-0.70134	-0.08535	-0.10488	-0.4028	-0.08563	0.982643	1.729864	-0.18867
Q9Y2C4	EXOG	1.24499	-0.22182	-0.05069	0.279907	0.251209	-0.09709	-0.83624	-0.74605	-0.21791	-0.10856	0.635126	-0.32766	0.227619	-0.16547	-0.02052	0.043448	-0.09295	0.213616	0.410267
Q9Y2D4	EXOC6B	-0.3818	0.295294	0.250075	-0.44993	-0.12286	-0.2428	0.120685	-0.22889	-0.86554	-0.46824	0.078362	-0.23858	-0.08544	-0.15975	-0.02835	-0.04234	-0.12017	0.292157	0.551295
Q9Y2D5	AKAP2	-0.81058	0.306409	0.180265	-1.15296	-0.77305	-0.82996	0.123936	0.214285	0.1331	-0.04251	-0.22959	-0.92943	0.770091	-0.78056	-0.48843	-0.26906	-0.4626	0.445772	0.644556
Q9Y2E4	DIP2C	-0.62895	0.009337	-0.0752	-0.92049	-0.62785	-0.55226	-0.0462	-0.37657	0.494922	-0.07897	-0.80368	-0.52533	0.656514	-1.08568	-0.55354	0.317237	-0.26046	0.491032	0.435729
Q9Y2E5	MAN2B2	1.762423	-0.09037	-0.26701	-0.61026	0.011824	-0.13506	-0.10486	0.805414	0.0084	0.92421	0.153281	0.158502	0.32788	0.804749	-0.30661	0.577815	0.628646	0.51866	0.223866
Q9Y2G3	ATP11B	0.448719	0.045083	0.34524	-0.1675	1.079161	0.405861	-0.09599	-0.41088	-0.20295	0.133416	-0.17952	0.600162	0.207122	1.507772	0.306842	-0.50785	0.432232	-0.30281	-0.18583
Q9Y2G5	POFUT2	0.031755	0.399512	-0.46942	-0.38481	0.664509	0.179868	-0.09962	0.377106	1.029022	0.649235	0.180184	-0.83677	0.200039	-0.4237	0.028886	0.045136	0.843623	1.557661	1.237176
Q9Y2G8	DNAJC16	0.446849	0.155126	0.255553	-0.31045	-0.08389	-0.15709	0.471063	0.755754	-0.08504	0.685191	0.237108	-0.26888	-0.34518	-0.13533	0.702718	0.135348	0.551236	0.508629	0.190575
Q9Y2G9	SBNO2	-0.07712	-0.06906	-0.04604	-0.26716	-0.13843	-0.28426	-0.24393	-0.13114	0.384686	-0.07022	-0.50949	-0.72802	-0.02034	0.054192	-0.12661	0.080926	-0.15311	-0.25644	-0.26873
Q9Y2H0	DLGAP4	-0.30919	-0.15284	-0.01429	-0.67508	0.137849	-0.16734	-0.42597	0.846325	0.425705	-0.04792	0.377723	0.066762	0.621973	0.340186	0.320326	-0.40621	0.830162	0.010713	0.434934
Q9Y2H1	STK38L	0.152603	0.413909	0.152045	-0.4083	-0.14296	-0.16905	0.088195	0.17259	0.349366	0.13596	-0.32144	-0.07573	0.030268	0.101604	-0.05083	0.4184	0.053353	-0.73193	0.314818
Q9Y2H2	INPP5F	-0.42255	0.331624	-0.15412	-0.66067	-0.57714	-0.54955	-0.35113	0.086688	0.298913	0.118885	-0.01847	-0.45591	0.130141	0.066944	-0.18064	0.188616	-0.20132	-0.00117	-0.12481

Q9Y2H5	PLEKHA6	-0.99885	-0.76212	-0.56216	-0.22992	0.277775	0.570211	-0.3211	-1.49003	-0.38793	0.32774	0.070357	-0.07937	0.180086	-0.83954	0.095705	0.519047	-1.33673	-1.80434	-1.78832
Q9Y2H6	FNDC3A	-0.19086	0.39299	0.595759	-0.92369	-0.427	-0.69726	0.790764	-0.21803	-0.03385	-0.01087	0.437148	-0.11452	0.210905	-0.16611	0.148873	-0.40393	0.234986	0.465385	0.555112
Q9Y2I1	NISCH	-0.14701	0.212107	-0.04599	-0.61085	0.193469	-0.19662	0.098894	0.463382	-0.20913	0.082413	-0.47198	0.091945	0.113947	0.189258	-0.46187	-0.49396	-0.31058	0.025994	0.156229
Q9Y2I7	PIKFYVE	-0.45769	0.032357	0.060641	-1.11266	0.241599	0.087189	0.062384	0.302699	0.324229	0.263726	-0.36119	0.265948	0.143278	0.211821	0.039894	0.042673	0.091708	0.12531	0.463189
Q9Y2I8	WDR37	-0.04519	0.298223	-0.10862	0.03285	-0.2425	-0.29968	-0.46659	-0.64093	-0.31412	0.240907	-0.09171	0.22204	-0.11477	0.439706	0.648925	-0.02963	-0.11463	0.144388	0.183309
Q9Y2J2	EPB41L3	-0.78304	1.282187	0.663955	-0.97878	0.100733	-0.54602	0.449887	-1.72347	-0.03964	1.115475	-0.9708	1.17122	0.21735	0.87347	-0.22577	-0.50739	-0.02487	0.647025	-1.2185
Q9Y2J4	AMOTL2	-0.48022	0.402787	0.270248	-0.39456	-0.66018	0.083356	0.248223	-0.30411	0.132227	-0.49575	-0.4438	-0.44819	0.04125	-0.6898	0.16235	0.002746	-0.01087	0.255815	0.47028
Q9Y2J8	PADI2	-1.10869	-0.27083	-0.30279	-0.23841	-0.487	-0.60348	-0.36407	2.41136	-0.71346	0.361621	-1.16614	0.947527	-1.00201	2.992818	-0.29864	-1.24436	-0.23879	-1.5663	-2.12576
Q9Y2K1	ZBTB1	0.157171	-0.09048	-0.05964	0.292396	0.020345	0.215415	0.091231	0.890769	-0.16539	-0.09102	-0.10042	-0.46457	-0.19561	-0.30202	0.163105	-0.29991	0.700662	0.996627	0.455703
Q9Y2K2	SIK3	-0.02901	-0.00179	-0.14853	-0.07103	0.162288	0.005332	0.158362	-0.60209	0.336159	-0.57098	-0.6861	0.349264	0.054453	0.286597	0.126417	-0.04959	0.177963	0.143026	-0.4238
Q9Y2K7	KDM2A	0.4641	-0.38237	0.42845	0.052351	1.093812	0.995602	0.485594	1.170489	-0.49483	-0.6908	-0.26585	-0.3984	-0.03688	0.112529	-0.17196	-0.02436	-0.14134	0.037877	-0.48871
Q9Y2L1	DIS3	0.565337	-0.10002	-0.51702	-0.60913	-0.03324	0.062323	0.045649	0.253812	-0.22029	0.172473	0.119297	-0.20721	-0.14036	-0.04687	0.3706	-0.29998	0.448676	0.041081	0.401289
Q9Y2L5	TRAPPC8	-0.73483	0.724901	0.806336	0.016236	0.265659	0.25816	0.350848	-0.40108	-0.08864	0.511217	-0.0658	0.309146	-0.3141	0.592565	-0.34306	-0.0379	-0.25485	-0.51312	-0.11172
Q9Y2L9	LRCH1	-0.81451	0.221857	0.146949	-0.36588	-0.21534	-0.38325	-0.00092	-0.39034	0.190098	0.710307	-0.09532	0.345458	-0.01088	0.234402	-0.38993	-0.6639	0.061359	0.013364	-0.02171
Q9Y2P4	SLC27A6	0.423101	-0.63692	-0.59901	-1.77852	-0.28559	0.567928	-0.65093	-1.47763	-0.64043	-1.809	2.81692	0.189765	-0.467	-0.48637	0.595451	-1.27779	-0.24161	-1.56978	-1.0634
Q9Y2P8	RCL1	-0.05109	-0.03022	0.118805	0.753096	-0.05159	-0.18726	-0.16898	0.536766	-0.15592	-0.06299	0.276171	-0.08536	-0.45501	-0.40554	0.039548	0.313811	0.056942	-0.45672	0.091656
Q9Y2Q0	ATP8A1	-1.53162	-0.67268	-1.07985	-1.5887	-0.56675	-0.82647	-0.17904	-0.87583	0.290612	-0.14976	-0.22106	-0.2752	-0.36322	2.3856	-0.88442	-2.01891	0.061635	-1.51567	-1.37058
Q9Y2Q3	GSTK1	-0.62177	0.191027	0.394661	2.366512	-0.02255	-0.17902	-0.59596	0.341839	0.071034	0.715338	-0.02201	0.537923	-0.22915	0.540715	2.021499	0.975653	-0.39664	-0.77085	-1.07384
Q9Y2Q5	LAMTOR2	-0.38359	-0.08849	-0.41639	-0.80953	0.908372	0.320046	-0.67626	0.662304	0.160236	0.728321	-0.62002	0.953302	0.05683	0.668658	-1.37659	-0.01236	0.527507	-0.31614	-0.80155
Q9Y2Q9	MRPS28	-0.51334	-0.6417	-0.9155	0.660959	0.784262	0.267089	0.034691	-0.08902	-0.0864	0.761363	0.540803	0.221331	0.084376	0.054735	0.043167	-0.38408	0.368338	0.217473	-0.86242
Q9Y2R0	COA3	-0.41601	0.104745	0.028601	0.637089	0.171219	0.801024	0.005845	-0.9375	-0.4173	-0.24402	-0.10377	-0.37314	-0.02562	-0.61721	0.605227	1.154661	-0.42898	0.654159	-0.14219
Q9Y2R4	DDX52	-0.06178	0.265935	-0.00149	0.996462	-0.50854	-0.10254	-0.07756	0.499882	-0.24108	-0.17525	0.921251	0.08745	0.128144	-0.45969	-0.00521	-0.41887	0.232912	-0.21016	-0.33744
Q9Y2R5	MRPS17	0.167806	-0.38612	-0.11126	0.299417	0.231749	0.554047	-0.52945	0.134531	-0.00506	0.189076	0.005651	0.295762	-0.07446	-0.08924	0.155676	0.643603	-0.18856	0.097941	-0.51087
Q9Y2R9	MRPS7	-0.2593	-0.1497	-0.05614	0.769588	0.15168	0.061302	-0.00732	0.12534	0.196785	0.025969	0.08323	-0.17484	-0.29403	-0.15094	1.443905	-0.15656	-0.24229	-0.24724	-0.75096
Q9Y2S2	CRYL1	-1.66581	0.302203	0.059101	-0.42991	0.160733	-0.12533	0.284139	-0.99234	-0.2027	-0.04528	0.063339	-0.17706	-0.20905	-0.06267	-0.28528	-0.8464	0.035644	0.397052	-0.14479
Q9Y2S7	POLDIP2	0.151654	-0.29228	-0.17398	1.200997	-0.006	0.170756	-0.75216	0.201529	-0.35775	0.181306	0.409538	0.020506	-0.05077	-0.06338	0.691395	0.669874	0.354587	0.591853	-0.02443
Q9Y2T2	AP3M1	-0.41091	0.57607	0.371063	-0.37504	0.128098	0.037483	0.297729	-0.25072	-0.07839	-0.19257	0.065195	0.416736	0.18512	0.585975	0.000985	0.096271	0.059023	-0.0617	0.330558
Q9Y2U5	MAP3K2	-0.00522	0.265253	-0.09427	-0.72875	0.474109	0.324658	-0.13902	-0.22254	0.198854	0.352797	-0.1994	0.535235	0.172635	1.038799	-0.37667	0.701177	0.799049	0.098367	-0.53091
Q9Y2U8	LEMD3	0.358922	-0.02589	-0.3718	-0.13474	0.311054	0.468425	0.208363	-0.46047	-0.11967	-0.7449	-0.07971	-0.09821	-0.25765	-0.06307	0.0557	-0.53033	0.220513	0.304101	0.095756
Q9Y2V2	CARHSP1	-0.16029	0.368219	0.131956	-1.22643	1.013546	-0.20281	-0.18394	-0.19124	0.137348	1.212532	-0.29006	0.518341	0.141576	0.967131	-1.02204	-0.73974	2.469536	-0.13142	-1.09454
Q9Y2V7	COG6	-0.80708	0.312965	-0.14128	-0.13966	0.325815	0.089832	0.163644	-0.4488	0.446355	0.071062	-0.39617	-0.03808	-0.07881	-0.11456	0.835069	0.178096	0.041096	0.068883	0.431276
Q9Y2W1	THRAP3	0.851163	-0.51492	0.237513	0.583232	-0.12078	0.589916	-0.90937	0.556099	-0.58512	0.00762	0.672919	-1.22519	-0.20451	-0.96473	0.24926	0.182426	-0.28981	0.407488	-0.07651
Q9Y2W2	WBP11	0.922747	-0.5344	-0.2943	-0.40553	-0.97265	-0.23521	-0.38996	0.445418	0.057738	-0.80158	0.485803	-0.79495	-0.35979	-0.88574	0.067057	-0.20669	-0.71952	0.303068	-0.17683
Q9Y2W6	TDRKH	-0.68019	0.220498	-0.50721	0.201136	0.21867	0.213178	0.276168	-0.44279	0.602627	-0.41793	-0.21081	-0.10674	0.650096	0.143918	0.689763	-0.25438	0.437058	0.407315	-0.93378
Q9Y2X0	MED16	0.133957	0.24513	0.069241	0.403133	-0.06767	-0.0395	-0.02004	0.662861	0.047966	-0.05493	0.367612	-0.08283	-0.03894	-0.01824	0.438783	0.123426	0.051349	0.196048	-0.13909
Q9Y2X3	NOP58	0.459726	-0.53251	-0.60643	-0.00579	-0.47166	-0.31888	-0.99264	-0.42352	-0.36914	-0.86903	1.259955	-0.59873	-0.44138	-0.56494	0.585546	-0.72586	-0.25629	-0.57768	-0.47031
Q9Y2X7	GIT1	-0.13431	0.134283	-0.15761	-0.35985	-0.39921	-0.4689	-0.27178	-0.02087	-0.39682	0.004824	0.229674	-0.12663	-0.23644	-0.24003	0.062155	-0.08197	-0.20645	-0.01855	-0.37659
Q9Y2Y0	ARL2BP	-0.64608	0.091554	-0.20842	0.014696	0.052335	-0.18773	-1.04943	-0.58163	0.237598	-0.14615	0.185067	-0.75682	-0.47651	-0.24222	-0.19899	-0.01036	0.728528	0.948527	0.289005
Q9Y2Z0	SUGT1	0.006935	0.435718	-0.40372	-0.88646	0.143033	0.14671	-0.07546	0.313007	0.452839	0.801945	0.436254	-0.07534	0.121421	0.006362	0.028784	0.125958	0.1348	-0.09339	0.230754
Q9Y2Z2	MTO1	-0.69834	-0.17914	0.011178	-0.11372	-0.47847	-1.61271	-0.10994	-0.5152	0.346777	-0.03127	0.286673	0.00652	0.108315	0.138488	0.609526	0.625652	0.704917	0.354876	-0.45507
Q9Y2Z4	YARS2	-0.2717	-0.55499	-0.33936	1.2526	-0.38538	-0.26687	-0.23969	0.055537	-0.54236	-0.4112	0.481579	-0.10313	-0.62442	-0.03017	-0.12836	1.22906	0.179662	-0.12717	0.383922
Q9Y2Z9	COQ6	0.291005	0.590376	-0.23024	0.74276	-0.56883	-0.50665	0.272967	-0.22931	-0.3555	-0.05595	0.273996	-0.26029	0.41835	0.233413	1.619816	-0.00243	-0.03707	-0.00934	-0.30179
Q9Y303	AMDHD2	-0.18745	-0.06835	0.06114	-0.46586	0.559276	0.422735	0.764602	-0.367	0.039987	0.102582	-0.90484	0.150343	-0.07411	0.298373	-0.68184	0.890876	-0.22212	0.384535	-0.12559
Q9Y305	ACOT9	-0.02358	0.177043	0.325973	0.237473	0.45772	0.350772	-0.26244	0.464932	-0.04842	0.35581	0.017991	0.389162	-0.1786	0.43703	-0.07026	0.67701	0.061902	0.075821	0.306178
Q9Y312	AAR2	0.454273	0.112758	-0.64046	-0.28914	-0.06917	0.403729	0.0985	0.331863	0.338072	0.327935	-0.44263	-0.1218	-0.11303	-0.09065	-0.20114	-0.24743	1.07418	0.680059	0.550095
Q9Y314	NOSIP	0.302983	-0.36655	-0.14654	0.55977	-0.6602	-0.1131	-0.34283	0.329128	-0.14348	-0.2009	0.035661	-0.80851	-0.15807	-0.24731	0.324037	-0.24002	1.001623	0.821537	-0.10343
Q9Y315	DERA	0.411513	0.726976	-0.18066	-0.27307	0.07176	-0.00667	0.91361	0.161026	0.599683	0.163073	0.208185	-0.2318	0.05753	-0.18946	-0.34283	-0.52586	-0.38761	-0.24345	-0.22626
Q9Y316	MEMO1	0.159071	-0.30694	0.857023	0.122761	0.664379	0.338657	0.227006	0.752512	0.189359	0.78823	-0.26456	-0.09028	0.248655	0.283338	0.357985	0.842224	0.023303	0.225186	0.460672
Q9Y320	TMX2	0.52547	-0.10027	0.308974	0.158889	0.362291	0.196597	0.886567	-0.27663	0.018939	-0.44495	-0.08763	-0.26845	0.160777	-0.51674	1.098679	0.869014	0.604434	0.405549	-0.59025
Q9Y333	LSM2	0.588142	-1.42968	-0.47061	-0.05115	0.952569	0.273112	-0.37779	0.705148	0.186544	-0.30928	0.732156	-0.22486	0.331435	-0.14489	-0.23756	0.507646	0.317968	0.4067	-0.21501
Q9Y366	IFT52	0.498473	-0.5404	-0.53044	0.012054	-0.60834	-0.57856	-0.4062	-0.00054	0.225331	-0.91537	1.811256	-0.68308	-0.02378	-0.87154	-0.4665	-0.72179	-0.08304	-0.26426	0.261898
Q9Y371	SH3GLB1	-0.17449	1.200547	0.728142	-0.677	0.059054	0.239245	0.521554	-0.29209	0.265107	0.685436	-0.41348	0.313874	0.287273	0.204661	-0.45294	-0.05791	-0.03307	0.4628	0.897156
Q9Y375	NDUFAF1	-0.79319	-0.15326	-0.11331	0.672215	-0.44474	-0.34447	0.110841	-0.015	-0.36906	-0.32414	0.812031	-0.56767	0.108149	-0.53458	0.515247	0.094449	-0.58875	0.430326	-0.78615
Q9Y376	CAB39	-0.08085	0.182033	0.112646	-0.58025	0.264813	0.398996	-0.13875	-0.18183	-0.29099	0.513208	0.248853	-0.55815	-0.20989	-0.01217	-0.07584	-0.09677	-0.59995	-0.38621	-0.01352
Q9Y383	LUC7L2	1.131593	-0.43307	0.034152	0.736945	0.131797	0.381322	-0.97383	0.938716	-0.44781	0.207478	0.408229	-0.89632	-0.60365	-0.40343	1.199	0.15799	-0.29608	-0.16465	-0.07002
Q9Y385	UBE2J1	-0.328	0.023414	0.233181	-0.63702	-0.11188	-0.12382	1.447831	-0.94322	0.811503	-0.42093	-0.58217	-1.17677	-0.31651	-0.05537	0.536794	-0.83543	-0.29395	0.338911	-0.54286
Q9Y388	RBMX2	0.879829	-0.30878	0.108763	0.52591	0.172885	0.467651	-0.55897	0.625428	-0.27043	-0.52953	0.2895	-0.43222	-0.20599	-0.46625	0.002323	-0.27735	-0.28133	0.237177	0.088146
Q9Y394	DHRS7	-1.13868	1.028346	0.68474	-0.43531	0.186707	-0.39891	0.03195	-0.68659	-0.05259	-0.09676	-0.34517	0.55137	-0.19414	1.182091	-0.59142	-0.55589	0.521595	0.571192	0.094984
Q9Y399	MRPS2	-0.12113	0.006847	-0.3294	0.418512	0.06115	0.262364	-0.72052	0.26062	-0.78604	0.191718	0.683101	-0.00148	-0.61775	-0.06884	1.115536	-0.07541	-0.17588	-0.5146	-0.30882
Q9Y3A2	UTP11	0.060226	0.064789	0.071872	0.591221	-0.16602	0.022791	-0.24067	0.349729	-0.27139	0.406295	0.919056	-0.0692	-0.13925	-0.40576	0.090225	0.54619	0.120426	-0.09594	0.310276
Q9Y3A3	MOB4	-0.50172	-0.96227	-0.80058	-0.06821	0.265213	-0.57474	-0.78241	0.504511	0.234008	0.595959	-0.00642	-0.07169	-0.26271	0.309326	0.409668	0.749835	0.020091	-0.35776	-0.12033
Q9Y3A4	RRP7A	0.169601	0.204326	-0.25772	0.735868	-0.31108	-0.35146	-0.06185	0.046789	-0.43587	0.004974	0.847874	-0.07502	-0.11761	-0.4389	0.265647	-0.06801	-0.38282	-0.36241	0.238457
Q9Y3A5	SBDS	0.066749	-0.13183	-0.63671	-0.60711	0.58507	0.352215	-0.84398	-0.94936	-0.28893	0.212096	0.439529	-0.2749	-0.08971	-0.12362	0.389994	-0.73874	0.250908	-0.03543	-0.32551
Q9Y3A6	TMED5	0.179861	1.224874	1.260931	-0.49583	0.558408	0.168956	1.481569	-0.24342	0.03162	-0.31338	-0.41422	0.36082	0.194152	0.671493	-0.39119	-0.41998	1.215069	0.888098	0.233347
Q9Y3B2	EXOSC1	0.213934	-0.51508	-0.42631	0.267534	0.672942	0.907602	-0.27134	0.405421	-0.52887	-0.66182	0.554875	-0.08244	-0.20071	0.04383	-0.56935	0.763689	0.174895	0.693357	0.360927
Q9Y3B3	TMED7	-0.75488	0.386831	0.228926	-0.34601	-0.10479	-0.14899	0.180289	-0.58631	0.141405	-0.54591	0.158308	0.329928	0.348802	0.344335	0.920752	0.48769	0.719142	0.520267	0.23905
Q9Y3B4	SF3B6	0.555564	-0.11294	0.258283	0.526212	-0.07242	0.483115	0.138674	0.564867	-0.50682	0.088117	0.232511	0.048233	-0.59585	-0.19305	0.327409	-0.15415	0.083371	0.091769	-0.41431
Q9Y3B7	MRPL11	-0.10856	0.013515	-0.30322	0.521152	0.356442	0.469388	-0.07266	-0.38678	-0.27987	0.61177	0.425304	0.027958	-0.03804	0.079119	0.503988	-0.26343	-0.03704	-0.29058	-0.3582
Q9Y3B8	REXO2	-0.37245	0.405437	0.452606	0.312516	1.729874	-0.65607	0.395745	0.138816	-0.09418	0.981398	-0.21164	0.760712	0.302786	-0.96637	0.13649	1.041812	0.3419	-0.11321	0.568453
Q9Y3B9	RRP15	0.583941	-0.09674	0.079133	0.735245	-0.97562	-0.66835	-0.17781	0.484169	-0.26589	-0.39927	0.373039	-1.27285	0.48902	-0.64991	0.047073	0.549488	-0.75832	-0.43202	-0.34679
Q9Y3C0	WASHC3	-0.36366	0.710894	0.686372	0.024756	-0.19486	0.10404	-0.17505	-0.39532	-0.07158	0.165922	-0.40299	0.085429	0.341265	0.417785	0.121176	0.219973	-0.13178	-0.14679	-0.02884
Q9Y3C1	NOP16	-0.09545	-0.039	-0.10558	1.669045	-0.46214	-0.05197	-0.05566	0.320314	-0.38581	-0.71831	0.798761	-0.84134	0.44224	-1.35983	0.730395	0.783946	-0.89153	-0.32873	-0.38104
Q9Y3C4	TPRKB	0.249183	0.127341	0.031484	0.658227	0.844639	0.604675	-0.1612	0.162378	0.109996	0.382723	0.092571	0.638928	-0.03498	0.513552	-0.18613	0.183622	0.293042	-0.37716	-0.24925
Q9Y3C6	PPIL1	0.620944	-0.3413	-0.05468	0.690498	1.055461	0.192175	-0.34633	-0.14753	0.366407	0.462324	0.337595	-0.18033	0.055815	-0.06429	0.184036	-0.05273	0.909883	0.534743	0.231035
Q9Y3C8	UFC1	0.102891	-1.09121	-0.40757	0.577167	0.586384	0.339017	-0.36918	0.333899	0.454734	0.450253	1.056667	-0.29171	-0.19538	-0.4306	0.388158	-0.05894	-0.82377	-0.12161	-0.09701
Q9Y3D0	FAM96B	0.152005	0.171959	-0.23795	-0.13943	-0.02892	-0.33361	-0.36303	-0.04105	-0.04454	0.528687	-0.44248	-0.203	0.647791	0.083676	-0.1826	-0.44388	0.23979	-0.09877	0.157931
Q9Y3D3	MRPS16	-0.17806	-0.08513	-0.05254	1.109739	0.490213	0.608951	0.029181	0.087167	0.006008	-0.21487	0.339167	0.053623	-0.18827	-0.21299	1.239952	-0.14461	-0.32677	-0.2626	-0.33472
Q9Y3D6	FIS1	0.310655	0.035315	-0.53547	0.261613	0.652104	0.441784	0.607018	-0.2621	0.380695	0.555905	0.652692	-1.03567	-0.3388	-1.45427	1.254607	1.250634	-1.3673	-1.13398	-0.60952
Q9Y3D7	PAM16	0.669051	-0.2563	-0.13238	-0.267	-0.69419	-0.0339	-1.25448	0.041298	-0.72049	-0.12794	0.32003	-0.55785	-0.22682	-0.97821	0.562015	1.87179	-0.33382	-0.22776	-0.86448
Q9Y3D8	AK6	-0.08929	-0.1138	-0.03121	0.516579	0.495732	0.231349	-0.29538	0.372974	0.569544	0.944229	-0.26529	0.219064	-0.07326	0.168453	-0.40867	-0.72221	1.942332	0.883816	0.0378
Q9Y3D9	MRPS23	-0.28104	0.010691	0.057575	0.60465	0.226463	0.484423	-0.08663	0.166201	-0.11793	0.102408	0.560552	-0.29689	-0.29233	-0.32735	0.837427	-0.02573	-0.2304	-0.26943	-0.28903
Q9Y3E1	HDGFL3	-0.46807	0.034689	-0.37098	-0.88832	0.015669	-1.79159	0.045703	-1.51866	0.350805	-1.61168	-0.18896	-0.81537	-0.15603	-0.59027	-0.73714	-1.10937	0.01964	0.827389	0.092814
Q9Y3E5	PTRH2	-0.83262	-0.00054	-0.18614	1.469451	-0.1704	-0.21462	-0.20521	-0.11849	0.959617	-0.92311	-0.07365	0.38215	-0.09421	-0.21024	-0.33055	0.340335	0.075528	-0.10758	-0.16136
Q9Y3E7	CHMP3	-0.03871	1.037245	-0.21269	-0.68221	0.106434	-0.15388	1.137775	0.032974	0.241477	0.001992	-0.29809	0.270096	-0.18797	0.289148	0.042037	-0.13629	-0.13385	-0.55385	-0.2387
Q9Y3F4	STRAP	0.330486	-0.80734	-0.41973	-0.19012	0.090269	0.08933	-0.74557	0.432204	0.35967	0.414643	0.140984	-0.01262	0.447301	0.159958	0.044684	1.007732	0.383146	-0.12676	0.479766
Q9Y3I0	RTCB	0.060938	-0.37651	-0.04907	0.601071	-0.22855	-0.17711	-0.55728	0.09301	-0.3866	-0.01753	0.538175	-0.29305	0.105751	-0.34611	0.601556	0.367004	0.130971	0.104325	0.265004
Q9Y3I1	FBXO7	-0.77736	0.691576	0.216932	0.121445	0.29924	-0.18414	-0.49906	-0.47239	0.352273	-0.15383	-0.06464	0.062592	-0.43381	-0.00269	-0.14542	-0.18754	0.42461	0.440773	-0.42698
Q9Y3L3	SH3BP1	-0.37852	1.206883	0.65914	-0.60458	0.443941	0.459441	0.195464	-1.25373	-0.60317	1.145559	-0.31216	0.51709	0.038809	0.690117	-0.4968	0.007024	0.301488	0.595898	-0.64129
Q9Y3L5	RAP2C	-0.61531	0.492081	0.411597	-1.22518	0.343279	-0.63726	0.233598	-0.78829	0.17251	0.085851	-0.33843	0.269206	0.006687	0.258793	-0.02812	-0.04472	-0.17677	0.742072	0.176887
Q9Y3P9	RABGAP1	0.962758	0.363478	0.087832	-0.46838	-0.39296	-0.37304	-0.01533	0.090158	-0.25975	-0.36695	-0.20964	-0.05441	0.315358	-0.02204	-0.49849	-0.54586	0.166845	-0.53064	0.51973
Q9Y3Q3	TMED3	-0.33285	0.332583	0.015469	0.330933	0.14851	0.293559	0.282055	-0.17196	0.152729	-0.73941	-0.06725	-0.17761	1.066679	-0.7608	0.459506	-0.28916	0.079996	-0.31182	1.707024
Q9Y3Q8	TSC22D4	0.042533	0.164082	-0.26281	-0.56557	-1.10176	-0.57573	-1.14272	0.199155	-0.39682	0.136944	-0.25111	-0.30705	-0.19271	0.098788	0.126358	-0.15312	0.122927	0.35139	-0.53238
Q9Y3R5	DOPEY2	0.036678	1.207374	0.233417	-0.63685	-0.19901	-0.08661	0.728542	-0.26204	-0.19552	-0.14326	-0.76243	0.978868	1.041881	0.714673	0.121189	-0.54619	0.304725	-0.54296	-0.14833
Q9Y3S1	WNK2	0.181331	0.224267	-0.22944	-0.94971	-0.61612	-0.92135	0.131973	1.022727	-0.74099	-0.39947	1.109289	-0.46109	0.104211	0.178638	0.232725	-0.31654	-0.28985	-1.14245	-0.69847
Q9Y3S2	ZNF330	-0.43915	-0.04809	0.042963	-0.78476	-0.35404	0.623485	0.124305	0.209461	0.102783	-0.25586	0.422733	-1.24809	-0.25627	-0.90503	-0.15229	0.038363	0.050622	-0.1095	0.233332
Q9Y3T6	R3HCC1	-0.40482	0.467243	0.152415	-0.0234	-0.06037	0.069367	0.313653	-0.19667	0.144442	0.135784	-0.22116	0.061539	0.341054	-0.1728	0.027161	0.309129	0.247648	0.092457	0.936737
Q9Y3T9	NOC2L	0.714484	-0.46261	-0.24543	0.882658	-0.17064	-0.0861	-0.37053	0.103338	-0.59792	-0.05293	0.429444	0.0582	-0.49087	-0.59828	-0.14543	-0.06582	-0.09406	-0.10232	-0.60908
Q9Y3U8	RPL36	-0.05907	-0.2205	-0.104	0.035687	-0.81904	-0.0698	-0.15471	0.22186	0.499378	-0.89514	0.387718	-0.56679	0.207826	-0.55917	0.593821	0.22165	-0.31398	0.025303	0.122428
Q9Y3X0	CCDC9	-0.08195	0.331906	0.275216	-0.42543	-0.47766	0.508899	-0.22851	-0.23826	0.182475	-0.56974	0.303462	-1.09812	0.801679	-1.07077	0.859479	-0.00586	-0.67797	-0.08519	0.418102
Q9Y3Y2	CHTOP	-0.35982	-0.10281	-0.23066	-0.49609	0.553843	0.066123	-0.24398	-0.11209	-0.0818	-0.38649	-0.19756	-0.44812	0.061213	-0.93409	-0.00961	-0.2253	-0.24872	-0.4829	-0.66813
Q9Y3Z3	SAMHD1	-0.67196	1.22685	1.38679	-0.40762	0.643971	0.568413	-0.29438	-0.55702	-0.33846	0.712635	-0.66783	1.762612	1.16215	1.351282	0.027958	-1.1973	0.676432	0.114593	-1.06828
Q9Y426	C2CD2	-0.22596	-0.01457	0.443483	-0.17552	-0.12316	-0.29371	-0.13187	-0.33249	0.338947	-0.14518	-0.09958	0.078876	-0.22939	0.363207	0.004226	-0.06123	-0.15498	0.557829	-0.19374
Q9Y446	PKP3	-0.39467	-1.07173	-0.797	0.116662	1.513495	1.022807	0.274536	1.424778	0.227396	0.487672	0.639039	-0.63046	-0.27309	-0.97196	1.06794	1.824769	0.173128	-0.59328	-2.61876
Q9Y450	HBS1L	0.027966	-0.19764	0.174079	0.034887	-0.10323	-0.03537	-0.55234	-0.87253	-0.06791	0.203355	0.180179	0.011323	0.247555	-0.06742	0.875947	0.360624	0.18585	0.123657	0.360253
Q9Y478	PRKAB1	0.533892	-0.10975	-0.17054	0.445638	-0.31138	-0.0715	-0.44504	0.08042	-0.11228	-0.17487	0.718252	-0.32073	0.520141	0.677436	0.861797	-0.57528	0.40142	0.174911	0.423863
Q9Y484	WDR45	-0.13532	0.465102	0.090429	0.194234	0.518401	-0.1628	0.090829	-0.1464	0.044901	0.069521	-0.00863	-0.0462	0.310318	0.368682	-0.03193	-0.65275	-0.13211	0.056326	-0.21998
Q9Y485	DMXL1	-0.92241	0.316628	-0.30899	-0.71117	0.065928	-0.05556	-0.37055	-1.11429	-0.07845	-0.08143	-0.26904	0.203655	-0.03872	0.30816	0.563117	-1.00071	0.00751	-0.57062	0.025985
Q9Y487	ATP6V0A2	-0.33202	0.194428	0.126583	-0.29846	-0.12341	0.070861	0.605185	-0.02842	-0.11764	-0.10813	-0.57042	0.52755	0.089788	0.22825	-0.26062	-0.37659	-0.0424	-0.22485	0.026062
Q9Y490	TLN1	-0.59829	0.603667	0.22553	-0.47689	0.368846	0.368897	-0.15601	-0.55127	0.194746	0.217757	-0.67921	-0.05986	0.15728	0.088559	-0.43077	0.291714	-0.55877	0.192693	0.391574
Q9Y496	KIF3A	-0.56863	-0.04456	0.017729	-0.29596	-0.15971	0.305926	0.197028	0.135592	-0.71422	-0.24349	0.029798	-0.07115	-0.12055	-0.60554	0.790135	0.399607	0.086487	-0.22968	0.098214
Q9Y4A5	TRRAP	0.228321	0.027614	-0.02419	-0.14274	0.159994	0.359886	0.703059	0.460442	0.174986	-0.22718	0.063084	-0.1155	-0.11762	0.060623	0.554781	-0.22637	-0.18105	-0.02718	0.067804
Q9Y4B6	DCAF1	0.331046	-0.47015	0.231155	-0.56213	-0.55915	-0.48436	-0.02683	-0.50662	0.065487	-0.90998	0.221715	-0.32061	-0.75722	-0.06321	0.323686	-0.83519	-1.01271	-1.1227	-0.47027
Q9Y4C1	KDM3A	0.655548	-0.18229	-0.21589	0.100876	-0.44523	-0.09635	-0.0173	1.17324	0.045087	-0.04419	-0.26706	0.71103	-0.18654	0.307953	-0.31616	-0.29084	0.304386	-1.00847	0.366936
Q9Y4C2	TCAF1	-0.75852	-0.1384	0.15137	1.993984	0.104103	0.564884	-0.34213	-0.50613	0.422405	-0.11919	0.655904	-0.65031	0.437893	-0.21041	0.124994	0.028145	-0.11223	-0.25193	0.110096
Q9Y4C8	RBM19	0.100586	-0.03476	0.240422	0.04876	-0.49303	-0.30676	0.444058	0.408363	0.487478	-0.23748	0.739224	-0.13978	0.535687	-0.11192	-0.49943	-0.13242	0.113958	-0.13584	0.261908
Q9Y4D1	DAAM1	-0.31701	-0.45351	-0.98498	1.079339	-0.68364	-0.61936	0.561559	-0.05648	0.668889	-0.27337	-0.70316	-0.61397	-0.02507	0.04305	1.350158	-0.19598	-0.54657	0.030139	-0.27594
Q9Y4D7	PLXND1	-0.56558	1.708403	0.555415	-0.76588	0.141098	-0.28996	0.156241	-0.25127	-0.32443	1.097848	-0.82629	0.188348	-0.16267	0.0609	0.342106	-0.57378	0.235017	0.204493	-0.75069
Q9Y4D8	HECTD4	0.024888	0.070647	-0.21097	-0.22562	-0.27633	-0.33299	-0.33561	-0.23669	-0.06656	-0.32797	-0.00158	0.021626	-0.08322	0.003413	-0.20972	1.071726	0.18543	-0.15611	0.171692
Q9Y4E1	WASHC2(	-0.3544	0.287468	-0.0642	-0.20107	-0.19547	-0.40898	-0.54486	-0.14994	0.015871	-0.18436	-0.25627	-0.23084	0.032444	-0.11745	-0.19242	-0.03955	-0.31199	0.00918	0.349072
Q9Y4E6	WDR7	-0.42574	0.537907	0.172068	-0.05196	0.078672	0.21938	-0.33782	-0.83401	0.685714	0.131241	-0.16556	0.678126	0.086492	0.82687	-0.07178	0.068922	-0.0768	-0.23011	-0.09959
Q9Y4E8	USP15	-0.24674	0.161787	0.736833	0.055806	0.54307	0.402428	-0.36688	-0.34014	0.277549	0.838897	-0.59208	0.150554	0.409443	0.649965	0.239908	-0.43087	0.335756	0.309361	0.125112
Q9Y4F1	FARP1	0.087491	-0.77448	-0.64991	-0.90361	-0.66807	-0.82401	-0.32658	0.25427	-0.39992	-0.62484	1.012045	-1.38422	-1.2416	-1.3136	0.073894	-0.34065	-0.22346	-0.03742	0.213936
Q9Y4F5	CEP170B	0.385823	-0.02493	0.316259	-0.60386	-0.10724	-0.21611	0.145246	0.219468	-0.17036	0.485379	0.082838	-0.32352	-0.18917	-0.26961	-0.14514	0.285196	0.169589	0.340764	-0.1522

Q9Y4G6	TLN2	-0.09484	-0.4532	-0.41754	-0.93138	-0.53982	-0.90787	0.050088	-1.30784	-0.29901	-1.24739	-0.10357	-0.94503	-0.5283	-0.72699	0.262252	-0.23359	0.352847	1.17425	0.033567
Q9Y4G8	RAPGEF2	-0.71564	0.250194	0.094117	-0.41584	-0.28997	-0.21049	-0.29536	-0.73827	0.19861	0.290695	-1.11161	-0.03893	0.145286	0.20873	-0.26962	-0.81965	0.224219	-0.06415	0.590802
Q9Y4H2	IRS2	-0.75302	0.032102	-0.17102	-0.74747	-0.69429	-0.67416	-0.1604	-1.05368	0.209118	0.196245	-0.01275	-0.59384	0.159889	0.002466	-0.10683	-0.40623	0.659066	-1.03207	-0.78608
Q9Y4I1	MYO5A	-1.10431	0.79666	0.388016	-0.16368	0.203928	-0.08101	0.384771	-0.8716	0.023366	0.477329	-0.67528	1.11287	0.212542	0.77616	-0.91588	0.141259	0.291612	-0.12814	-0.38789
Q9Y4J8	DTNA	-1.19479	-0.2293	-0.1137	-0.8735	-0.74727	-1.14445	0.375067	-0.31836	0.103399	0.412227	-0.56214	-0.5443	-0.51911	-0.27369	-0.06833	-0.96226	-0.62685	-0.08528	0.583347
Q9Y4K0	LOXL2	-0.689	-0.15751	-1.20258	-1.60728	-0.92396	-0.08404	-0.62757	-0.28376	0.699083	-0.74133	-0.3068	0.307775	1.112913	-0.69851	-1.2214	-0.19207	0.322833	-0.69989	1.578298
Q9Y4K1	CRYBG1	0.092493	0.513458	-0.09021	-0.52931	-0.30797	0.000464	-0.26691	-1.44394	0.137053	0.068854	0.980153	0.192751	-0.65721	0.201174	1.390699	-0.14088	0.779072	0.577127	-0.81647
Q9Y4K3	TRAF6	-0.10432	-0.23188	-0.00603	0.64447	-0.13416	0.033983	-0.37929	-0.07102	-0.15774	-0.06862	0.490741	-0.0365	0.063426	0.326127	0.529223	0.272394	0.214005	-0.16782	-0.05084
Q9Y4K4	MAP4K5	-0.14376	0.093513	0.163554	-0.24926	-0.58843	-0.45856	0.364869	-0.13069	-0.08188	-0.46037	-0.16422	-0.55997	0.198037	-0.63377	-0.25062	0.126967	0.418994	0.317971	0.970261
Q9Y4L1	HYOU1	-0.21256	-0.09676	-0.37474	-0.51216	0.274782	-0.1918	-0.40616	0.292276	0.286459	0.719725	-0.13603	1.201459	1.212798	0.372203	0.648903	-0.12214	0.793977	1.087967	-0.31377
Q9Y4P1	ATG4B	0.116741	0.166646	-0.77024	0.016084	0.242915	0.435258	0.030076	0.708921	0.333682	-0.23612	-1.19308	-0.20061	0.225807	0.299354	-0.27028	1.431541	0.528375	0.247025	0.62512
Q9Y4P3	TBL2	0.073824	0.188514	0.612163	0.153501	-0.17901	0.042575	0.780283	0.010077	0.050633	-0.17434	0.013373	0.406629	-0.08883	0.374625	1.053656	-0.06993	0.041675	-0.30932	-0.1628
Q9Y4P8	WIPI2	-0.65336	0.410512	-0.00109	-0.54214	-0.42322	-0.39817	0.062774	0.796264	0.175491	0.443446	-0.28517	-0.05424	-0.25187	0.392221	0.2149	-0.19826	0.058028	-0.29854	0.166389
Q9Y4R8	TELO2	0.75936	0.187849	0.434787	0.275384	0.044432	-0.13628	0.308368	-0.11699	-0.28412	0.074485	-0.14729	0.009034	0.67162	-0.01585	0.018752	0.385785	0.413935	0.032158	0.489233
Q9Y4W2	LAS1L	0.583787	-0.11684	-0.23057	0.515319	0.134452	0.24835	-0.06036	0.843577	0.072484	-0.1626	0.730214	-0.41775	-0.42155	-0.39499	0.078583	0.116848	-0.44663	0.059143	0.098349
Q9Y4W6	AFG3L2	0.176074	-0.13123	-0.16828	0.755792	0.869425	0.940551	0.11555	0.454487	-0.13706	0.343202	0.474787	-0.19172	-0.41012	-0.59545	0.960518	0.541179	-0.3682	-0.35105	-0.8045
Q9Y4X5	ARIH1	-0.16606	-0.57994	0.228666	0.011459	0.208642	0.084741	0.047476	-0.00804	-0.58888	-0.0401	-0.15829	-0.54296	-0.085	-0.15333	-0.61027	-0.04315	0.874676	0.493213	0.701107
Q9Y4Z0	LSM4	0.668563	-0.81588	-0.30203	0.001939	0.94723	0.738072	0.21563	0.732587	-0.3017	-0.30372	0.995284	0.454972	-0.47919	0.328211	-0.27222	0.457751	0.386566	0.401443	-0.25277
Q9Y508	RNF114	0.573949	-0.44974	0.125381	-0.16188	0.662228	-0.04023	-0.00756	0.783239	-0.67772	-0.06539	-0.53472	-0.47614	-0.36363	0.126918	0.28735	-0.15992	-0.33587	0.474182	-0.62182
Q9Y512	SAMM50	0.315762	0.315972	0.337732	0.384335	0.015373	-0.06713	-0.1428	0.063968	0.106208	0.005093	0.633467	0.042638	0.087536	-0.02194	1.032411	0.824331	-0.66627	-0.26783	-0.24518
Q9Y520	PRRC2C	0.280373	-0.5274	0.088329	-0.41336	-1.107	-0.19654	-0.67842	-0.12986	0.288914	-0.16106	-0.00254	-0.96705	0.058328	-0.7297	0.487951	0.222602	-0.47654	0.126547	0.147228
Q9Y530	OARD1	1.037728	0.088527	0.01039	-0.53493	0.594908	0.890955	-0.23424	-0.05344	0.271456	-0.3632	-0.26276	-0.0398	-0.15007	0.175073	-0.36521	0.010593	0.282817	-0.85079	0.645026
Q9Y535	POLR3H	0.398406	0.54338	-0.35619	-0.69313	0.119273	0.433267	0.187337	0.22986	-0.25243	-0.17158	0.015531	-0.02006	1.344727	0.124938	-0.44457	0.163481	0.196481	-0.00699	0.041948
Q9Y546	LRRC42	1.384648	-0.4922	0.209845	-0.00215	0.194892	1.038879	0.002635	0.419096	-0.33323	0.050435	-0.15111	-0.81253	-0.0844	-0.23667	-0.14694	0.816594	0.331568	-1.00651	0.185204
Q9Y547	HSPB11	-0.96747	-0.48893	0.144524	-0.56109	1.476999	0.18415	0.112177	-0.16009	0.630455	2.962065	0.6774	0.535336	-1.07919	1.340347	-0.6274	0.373721	0.327226	0.284059	-0.74624
Q9Y570	PPME1	0.168105	-0.50575	-0.9406	-0.0015	0.506302	0.507634	-1.01204	0.308336	0.343658	-0.13798	0.26881	-0.31964	0.203496	-0.75602	-0.31707	-0.17607	-0.00644	-0.40432	-0.03014
Q9Y572	RIPK3	-0.94287	0.734741	0.568288	-0.63051	0.347618	0.522387	0.027803	-0.81295	0.133785	0.660756	-0.51771	0.712423	0.152839	1.020129	1.068141	0.352802	0.647421	0.258888	-0.59134
Q9Y580	RBM7	0.494631	-0.18154	0.077091	0.239829	-0.09902	-0.00402	-0.13907	1.064446	-0.14266	-0.04417	0.011327	0.123718	-0.24157	-0.33596	0.60934	-0.17442	0.18772	0.358464	-0.11668
Q9Y597	KCTD3	-0.06291	0.304376	0.25549	-0.29565	-0.06387	-0.22993	0.285919	-0.1198	0.261418	-0.57835	-0.42706	-0.2107	-0.00189	-0.05445	0.114201	0.411097	-0.70278	-0.28835	0.256534
Q9Y5A7	NUB1	-0.43798	0.172486	0.592687	0.267509	0.029684	0.035699	-0.90527	0.180886	0.352154	0.456918	0.052852	-0.26612	-0.13158	0.00469	0.176944	0.576105	0.050342	-0.51858	-0.89514
Q9Y5A9	YTHDF2	0.516406	-0.22585	0.35772	-0.59368	-0.57889	-0.11476	-0.25895	0.216484	0.593359	0.513455	0.023517	-1.16727	0.045316	-0.64625	0.451708	0.111305	0.362206	0.355378	0.042597
Q9Y5B0	CTDP1	-0.34507	0.398385	0.193384	-0.40442	-0.42896	-0.05865	0.250921	-0.21734	-0.15873	0.691816	-0.06912	0.09954	-0.23303	0.221867	0.070161	0.329106	-0.15896	-0.22342	-0.03272
Q9Y5B6	PAXBP1	0.557209	-0.23771	0.078359	-0.41623	-0.14455	0.202485	-0.51834	0.535221	-0.15625	-0.17016	0.083553	-0.27378	-0.27145	-0.37589	0.051046	-0.11223	0.412378	0.385859	-0.23341
Q9Y5B8	NME7	-0.43055	0.08359	-0.16258	-0.00181	-0.10438	0.301916	-0.24183	-0.10641	-0.04941	-0.11747	0.884662	-0.48779	-0.44072	-0.0652	-0.61217	-0.14714	-0.07365	-0.51662	0.399997
Q9Y5B9	SUPT16H	1.777693	-0.56186	-0.02404	0.504237	-0.25604	0.116041	-0.24964	0.597464	-0.41146	-0.2849	0.309262	-0.44274	0.339505	-0.53614	0.481666	0.422897	-0.17357	-0.26255	0.033482
Q9Y5E7	PCDHB2	0.353778	0.73088	0.321145	-0.7414	-0.39433	-1.32097	0.551445	0.452981	0.167117	-0.35532	-1.09725	-0.21798	0.221373	0.12693	-1.02252	-0.23051	0.180343	-0.35064	1.372699
Q9Y5J1	UTP18	0.211086	-0.34033	-0.36663	0.211694	-0.08752	-0.22912	-0.33731	0.164427	0.010619	-0.45064	0.654949	0.014946	0.222069	-0.649	0.06171	-0.15953	-0.22326	-0.35182	-0.54374
Q9Y5J6	TIMM10B	0.153572	-0.43918	-0.22416	0.121242	0.210022	-0.01208	-0.58266	0.459176	0.058031	-0.52501	0.324723	0.217427	0.01642	-0.13161	0.258289	0.433006	-0.36352	-0.57959	-0.60449
Q9Y5J7	TIMM9	0.217646	-0.09689	-0.06483	0.440829	0.413989	0.652318	-0.13089	1.005126	-0.09376	-0.42181	0.729673	-0.33415	0.408638	0.509995	0.368454	0.231476	-0.0915	-1.21039	-0.14838
Q9Y5K5	UCHL5	-0.17068	-0.29483	0.105407	-0.79954	0.221566	0.592109	0.10259	0.119017	-0.53682	0.100104	-0.10262	-0.28857	0.110128	-0.0783	0.154466	-0.03014	-0.35456	-0.38611	-0.52848
Q9Y5K6	CD2AP	0.477638	0.158081	-0.44831	-0.5917	-0.70698	-0.2515	-0.87852	-0.78538	-0.09739	-0.65761	0.717333	-0.29501	0.816812	-0.47848	0.010069	0.000903	0.306094	0.002756	-0.58887
Q9Y5K8	ATP6V1D	-0.37191	0.376676	0.222853	-0.22409	-0.11373	0.021205	-0.18396	-0.72878	-0.76206	-0.08266	-0.01055	0.501385	-0.00696	0.122886	0.594101	-0.16059	-0.26429	-0.6421	-0.37837
Q9Y5L0	TNPO3	0.007354	0.117171	0.162054	0.236827	0.03906	0.261318	-0.01901	0.250111	0.805986	0.306248	-0.08613	-0.01124	-0.23235	0.367911	0.708984	0.115949	-0.19073	-0.40727	0.195043
Q9Y5L4	TIMM13	0.413526	-0.96509	-0.42021	0.632301	0.323369	0.308309	-0.36425	-0.0201	-0.32324	-0.17112	0.441953	-1.25035	-0.44435	-0.59565	0.537479	1.054804	-0.61473	-0.3436	-0.71472
Q9Y5M8	SRPRB	0.145287	0.187021	0.084993	0.444714	0.345262	0.304465	0.225379	-0.34038	-0.26546	0.128573	-0.18601	0.250503	0.082181	-0.19762	1.026213	0.107784	0.785041	0.12876	0.095904
Q9Y5P4	COL4A3BI	-1.25448	-0.81872	0.070871	-0.34202	-0.45234	0.084146	-0.3572	-0.17635	-0.10668	-0.16936	-0.07493	0.445294	0.056978	0.501677	0.909651	0.098972	0.801717	-0.26723	-0.43514
Q9Y5P6	GMPPB	-0.00583	-0.35321	0.195935	-0.54114	0.020952	0.13117	0.1265	-0.29798	-0.1056	-0.34828	-0.35094	0.234108	0.509386	0.043765	-0.15187	-0.28474	-0.09592	-0.10521	0.183698
Q9Y5Q0	FADS3	0.4116	0.226007	0.335077	0.823404	0.864113	-0.84279	0.158501	0.476416	0.627648	0.207371	-0.09599	0.649904	0.439736	0.488608	0.19028	0.025748	0.670955	0.731822	0.457289
Q9Y5Q8	GTF3C5	0.415821	-0.57067	0.325992	0.057855	-0.09131	0.164144	-0.0688	0.557848	-0.33397	-0.22424	0.139836	-0.02403	-0.08511	-0.09649	0.06657	-0.17922	-0.07767	0.069112	-0.00772
Q9Y5Q9	GTF3C3	0.564964	-0.85506	-0.22694	0.379753	-0.05906	0.175678	0.263416	0.681715	0.462657	-0.09474	-0.08598	-0.00584	-0.14031	-0.23154	0.392239	0.251022	-0.20415	-0.60948	0.493149
Q9Y5R8	TRAPPC1	-0.24444	0.157112	0.009457	-0.37158	0.222273	0.320671	0.107589	-0.03813	-0.1331	0.078444	-0.46604	0.230767	-0.19336	0.547669	0.219612	0.238329	0.517624	-0.48134	-0.29376
Q9Y5S1	TRPV2	-1.49315	1.048894	0.453281	-1.42071	0.473608	-0.01915	0.437847	-1.5314	0.064646	0.892011	-0.73839	0.687555	-0.01514	0.519809	-0.16706	-1.43974	-0.20655	-0.09889	-1.29566
Q9Y5S2	CDC42BP	-0.20153	0.202569	0.021266	-0.00039	-0.26729	-0.18018	0.376078	-0.0728	-0.45732	-0.25297	0.27418	-0.52136	0.157315	-0.4442	-0.08882	0.79608	0.210111	0.316433	0.406341
Q9Y5S9	RBM8A	0.92169	-0.55642	0.076186	0.631048	0.48485	0.81276	-1.28794	0.686624	-0.33381	0.036319	0.068242	-0.71232	-0.17159	-0.33536	0.053056	0.030032	-0.15591	0.21425	0.007152
Q9Y5T5	USP16	-0.09535	-0.13287	0.290757	-2.06943	-1.06023	-1.48239	0.086056	-0.32056	-0.5645	-0.19852	0.082657	0.024872	0.657471	-0.11774	0.859641	-0.04244	0.201087	0.308585	-0.30376
Q9Y5U2	TSSC4	-0.01743	-0.54747	-0.77483	0.104226	-0.62855	0.736505	-0.96711	0.619143	-0.23534	0.039474	-0.15614	-1.42445	0.425267	-1.04853	0.162243	1.251882	-0.64113	0.01737	0.03351
Q9Y5U9	IER3IP1	-0.70539	0.543562	0.39199	0.415416	0.163248	-0.17902	0.275585	-0.42198	-0.10429	-0.00109	-0.14293	0.681022	0.283109	0.3701	0.661318	0.882936	0.451617	0.126066	0.716147
Q9Y5V3	MAGED1	0.358541	0.330661	-0.77277	0.419392	-0.76752	-0.8114	0.103548	-0.29488	-0.08137	0.530907	-0.34506	-1.5118	-0.05288	-1.33315	-0.69635	0.538939	-0.34119	0.58646	1.197949
Q9Y5W7	SNX14	0.428251	0.078103	0.184574	-0.29102	0.236931	-0.08772	0.003378	-0.22155	0.054029	-0.58979	0.015484	0.6535	-0.224	0.113609	0.520647	-0.81314	0.200834	-0.11594	-0.31981
Q9Y5W8	SNX13	0.157043	0.157143	0.729729	-0.68084	0.03566	-0.31806	-0.03395	-0.28793	0.75088	0.451894	0.293122	0.374114	-0.31293	0.478568	0.118152	-0.02439	0.083092	-0.03854	-0.55885
Q9Y5X1	SNX9	-1.36396	0.278601	0.611001	-0.05605	0.193363	-0.02552	0.205198	-1.01168	-0.39912	0.022947	-0.21524	0.062103	-0.376	0.140838	0.132671	-0.06554	0.022202	0.726804	0.536087
Q9Y5X2	SNX8	-0.95481	0.85786	0.359009	-0.83873	0.388423	0.089563	-0.60149	-0.05236	0.651253	0.653691	-0.66141	0.390051	-0.04243	0.328264	-0.80565	0.847301	-0.08084	0.402225	0.321059
Q9Y5X3	SNX5	-0.1134	0.088328	0.223365	-0.0546	0.22072	0.146793	-0.26044	-0.34977	-0.79683	0.261995	-0.20389	0.220322	0.218566	0.172214	-0.0264	0.078678	0.765634	0.480283	-0.44488
Q9Y5Y2	NUBP2	-0.0832	0.367712	0.124726	-0.18694	-0.02416	-0.38385	-0.03721	0.070997	0.14237	0.053911	-0.69927	0.893396	0.27611	0.777028	-0.03628	0.08432	1.031132	-0.11149	0.327554
Q9Y5Y5	PEX16	-0.39626	0.777714	0.390159	0.828208	-0.06234	0.469385	0.033139	0.092564	-0.30347	0.717932	-0.19521	0.193354	0.220739	0.360434	0.524271	2.235808	-0.16729	-0.35301	-0.49036
Q9Y5Y6	ST14	1.169731	-0.26067	-0.0568	-0.00622	-0.55193	-0.33412	0.020131	1.019591	-0.38845	0.747743	-0.2685	-0.30653	-0.21903	0.205884	-0.08532	-0.24274	-0.09757	-0.68985	-0.97641
Q9Y5Z4	HEBP2	-1.99798	1.058495	0.701641	0.406695	1.241326	-1.26119	0.104761	-0.6075	-0.31925	1.763708	-0.70521	0.305411	0.125539	1.336782	-0.41542	-0.73981	0.452507	-0.02744	-1.19752
Q9Y605	MRFAP1	0.597142	-0.07626	-0.2599	0.159143	0.365149	1.04571	-0.26865	0.024822	-0.27057	-0.20137	0.180172	-0.31211	0.013811	-0.39898	-0.42236	-0.06001	0.589443	-0.25831	-0.21658
Q9Y606	PUS1	0.398647	-0.30235	-0.13236	0.948929	-0.12538	0.071402	-1.47237	0.296651	0.134942	-0.0831	0.782565	0.044689	0.047682	-0.23934	0.439656	-0.48536	-0.2396	-0.53536	-0.40522
Q9Y608	LRRFIP2	0.91761	-0.37493	0.677804	-0.50386	-0.13042	0.103255	-0.55942	-0.44333	-0.74631	-0.3525	0.282144	-0.74696	0.080762	-0.27569	-0.07308	0.100272	-0.95996	-0.54114	-0.11885
Q9Y613	FHOD1	-0.79412	0.771945	0.224097	-0.27916	-0.04654	-0.26218	0.351726	-1.12289	0.544137	0.679245	-0.23898	0.085561	0.079957	0.016111	-0.41853	0.179704	-0.45549	0.118316	-0.16421
Q9Y617	PSAT1	1.20744	-1.79532	-2.30002	-0.14006	-1.13436	-1.39854	-1.03232	1.307641	-0.45637	-2.19721	-1.10957	0.193916	1.745753	-0.87237	2.538676	2.092876	2.165707	1.250426	-0.99273
Q9Y618	NCOR2	0.312618	-0.14739	-0.25026	-0.08285	-0.94804	-0.57989	-0.91816	0.163642	-0.10554	-0.59854	0.19942	-0.55055	-0.15235	-0.35189	0.151321	-0.42822	-0.03804	0.456161	0.18312
Q9Y623	MYH4	-1.56512	-1.53461	-0.85162	-1.44559	-0.18595	-0.61474	-0.6487	-3.86591	0.168443	-0.82781	-1.47906	1.974024	-1.8976	-3.1157	-5.30465	-1.43742	-0.14321	-0.43491	-0.77851
Q9Y624	F11R	0.799427	-0.23843	-0.02853	-0.15342	0.149474	0.287867	0.274071	0.897461	0.009574	-0.13439	-0.1874	-0.71571	-0.73378	-0.72373	0.525553	-0.63948	-0.47522	0.290473	-1.22503
Q9Y625	GPC6	-0.61812	0.270824	-0.77028	-0.58234	-0.50723	0.123492	0.327105	-0.39462	1.74072	0.390726	-0.8041	-0.76335	0.305514	-0.79147	-1.85211	-0.38814	-1.0818	-0.49071	0.372307
Q9Y639	NPTN	0.218402	-0.07416	0.270294	0.535666	-0.41936	-0.18832	0.83331	0.7203	-0.5096	-0.61909	0.40945	-0.41866	-0.06859	-0.14173	0.262383	-0.4283	-0.74872	-0.22834	0.195102
Q9Y646	CPQ	-0.77528	0.179824	0.379542	-0.06394	0.817552	-0.36656	0.37224	0.362854	-0.2292	-0.19234	0.359319	0.214342	-0.02224	0.409284	-0.22135	0.739362	-0.40098	-0.14766	0.02218
Q9Y653	ADGRG1	0.086229	0.04085	0.309483	-0.09777	0.009152	1.438373	0.285293	0.687752	-0.15316	-0.26434	-0.24768	-0.13594	0.375982	0.158331	0.935983	-0.11963	0.789129	-0.44606	-0.30846
Q9Y657	SPIN1	0.982915	-0.75654	-0.73354	-0.49053	0.617282	0.885191	-0.00435	1.248753	0.002268	-0.35406	1.32291	-0.46665	-0.38238	-0.66896	-0.68812	-0.00101	-0.03581	-0.25786	0.496897
Q9Y666	SLC12A7	0.558233	0.292787	-0.08497	0.020317	-0.51619	-0.52138	0.747209	0.393921	0.442464	1.209141	-0.27471	0.263598	-0.03405	0.152727	0.674216	-0.43012	-0.73465	-0.66396	-0.24864
Q9Y672	ALG6	0.375683	-0.00337	0.007925	0.118987	0.499085	0.463848	-0.37142	0.027616	-0.29564	-0.03657	0.294995	0.087663	-0.8744	-0.18778	0.128152	0.321657	-0.01832	0.046272	-0.07325
Q9Y673	ALG5	-1.1139	0.446066	0.503865	-0.54355	0.548004	0.652417	1.159005	-0.77582	0.926364	-0.16934	-0.03605	0.30786	-0.04367	0.131877	-0.34734	-0.25679	0.254016	0.329222	0.427486
Q9Y676	MRPS18B	-0.54687	0.143454	0.048016	0.241223	-0.16073	0.34705	0.585008	-0.28403	-0.45627	-0.0608	0.498392	-0.34802	-0.03668	-0.35403	1.105777	-0.35559	-0.38831	0.065899	-0.31313
Q9Y678	COPG1	-0.56176	0.695689	0.269906	-0.30023	-0.07246	-0.04146	0.793751	-0.27338	0.283091	0.125675	-0.25627	-0.09219	0.543296	0.164641	0.363063	0.510865	0.642283	0.513812	0.963602
Q9Y679	AUP1	0.314742	0.282806	0.131128	-0.17762	0.169468	0.477981	0.552306	-0.15597	0.020067	0.266763	-0.03744	0.250122	0.159191	-0.19276	0.205978	0.301787	-0.25146	-0.42956	-0.25648
Q9Y680	FKBP7	-0.14884	-1.96509	0.402314	-0.63861	1.996099	-0.91139	-0.35849	-0.84293	1.317276	1.739615	-0.58412	-0.44281	-0.11986	-0.71137	-1.07587	1.023491	-1.56921	0.707485	2.223925
Q9Y692	GMEB1	0.101142	0.12353	-0.16341	-0.29284	0.197255	0.116076	-0.11609	0.242505	-0.7287	-0.19378	0.324315	-0.09299	-0.33764	0.218033	-0.20443	0.035324	0.12892	0.506122	-0.3088
Q9Y696	CLIC4	-0.58007	0.320852	0.468362	-1.11355	0.445743	-0.13937	-0.15939	0.633728	0.649556	0.599972	-1.08867	-0.41526	1.303929	-0.47502	-0.80672	-0.06377	-0.39963	0.217105	1.277085
Q9Y697	NFS1	0.085947	-0.46542	-0.24011	0.674735	-0.20888	-0.16078	-0.75353	0.423428	0.313173	0.134527	0.286466	0.102059	-0.44613	-0.00835	1.540021	-0.4601	-0.44137	-0.39752	-0.04648
Q9Y6A1	POMT1	-0.48271	0.119419	-0.2119	-0.49776	-0.38568	-0.05255	-0.11499	1.467613	-0.07293	0.545072	0.888807	-0.4157	-1.12285	-1.34934	0.040668	0.12858	-0.35074	0.259671	0.224085
Q9Y6A4	CFAP20	-0.16754	0.204206	-0.81094	0.110318	-0.05536	0.134104	-0.99198	0.219245	-0.30518	-0.07941	0.891038	-0.30042	-0.19894	-0.06191	0.685136	0.090199	0.528914	0.043664	-0.17633
Q9Y6A5	TACC3	1.520849	-0.32824	1.588165	-0.61679	0.804631	0.767369	-0.16165	0.347013	0.57426	0.820697	-0.69303	-0.34494	-1.2599	0.53486	-1.10737	-0.14713	-0.4158	-0.52539	-0.32947
Q9Y6A9	SPCS1	-0.41354	0.218047	0.79317	-0.21382	0.121039	-0.0088	0.357266	-0.10129	-0.08929	-1.19106	0.455357	0.264539	0.054148	0.188836	-0.73454	-0.2641	0.146262	0.502802	-0.40863
Q9Y6B6	SAR1B	-0.77376	-0.94942	0.345318	0.668957	1.125548	0.205844	0.143177	-0.3985	0.431325	0.53774	0.045623	1.042042	0.38242	1.009798	1.159651	0.750647	0.935076	0.641552	-0.24682
Q9Y6B7	AP4B1	-0.34509	0.30223	0.141643	-0.41112	0.127849	0.095741	0.168142	-0.40994	0.23338	0.104391	-0.52348	1.134884	0.299891	0.410846	-0.75569	0.39564	0.481071	0.417295	0.296524
Q9Y6C2	EMILIN1	-2.50429	-0.24426	-0.45336	-2.37181	-1.74883	-1.76471	-0.19317	-1.57457	0.312815	-0.58686	-0.20533	-1.65557	-0.32002	-1.23632	-0.5892	-0.82588	-0.75028	-0.58459	0.351659
Q9Y6C9	MTCH2	-0.03815	-0.17049	0.075592	0.776573	-0.25064	-0.46084	0.293986	0.943243	-0.64702	0.014617	0.112972	0.153729	-0.60831	0.119956	0.965705	1.12979	-0.11409	-0.00898	0.06458
Q9Y6D5	ARFGEF2	0.070304	0.147857	0.40556	0.001093	0.392647	0.445125	0.109363	-0.09948	0.905254	0.17535	-0.29177	0.061064	-0.19189	0.184004	0.87819	0.109715	0.414808	0.316842	-0.48574
Q9Y6D6	ARFGEF1	-0.37615	0.303922	0.771626	-0.25995	0.267968	0.338771	0.162179	-0.17971	0.038771	0.060501	-0.32782	0.668122	1.347503	0.288738	0.132752	0.54995	0.84736	0.598497	-0.02489
Q9Y6D9	MAD1L1	0.020287	0.173228	-0.41452	0.247344	0.205214	0.347907	-0.04843	0.251151	-0.37939	0.09525	0.225458	-0.31832	-0.16346	-0.39546	0.748675	-0.06407	-0.35563	-0.06435	0.127773
Q9Y6E0	STK24	0.243323	-0.34818	0.28802	-0.4204	0.472816	0.836276	-0.19239	0.243633	-0.12958	-0.22487	-0.07231	-0.32991	-0.10588	-0.04259	0.804848	-0.05536	0.361549	-0.25742	-0.25713
Q9Y6E2	BZW2	1.624208	0.056517	0.314675	-0.04281	-0.2208	0.169525	-0.18773	0.608995	-0.04744	0.657594	-0.09147	-0.33723	0.365678	-0.63522	-0.01457	0.215838	0.404751	-0.25498	0.191005
Q9Y6F6	MRVI1	-1.70283	0.15119	0.46951	-1.96964	-0.97542	-1.37769	-0.16327	-1.27788	0.749661	-0.4413	-1.10664	-1.23905	-0.78521	-0.97235	-0.36443	-0.95878	-1.27159	1.410778	1.746318
Q9Y6G5	COMMD1(	-0.0401	0.600936	0.241613	-0.28155	0.055033	0.022757	0.297414	-0.21127	0.027216	0.75329	0.102558	0.313125	0.021391	0.486899	-0.2536	-0.01265	-0.11592	-0.25105	-0.24367
Q9Y6G9	DYNC1LI1	1.013141	0.040215	-0.00501	-0.55182	0.30706	0.549891	-0.20302	-0.27049	0.483427	0.222776	-0.1496	0.222981	-0.02543	0.489135	-0.21633	0.485546	0.321377	-0.05801	-0.08571
Q9Y6I3	EPN1	-0.1783	0.402821	0.265828	-0.52697	-0.37252	-0.64323	0.512127	-0.46026	0.236559	0.108075	-0.17691	-0.60978	-0.12154	-0.17485	0.448014	0.139835	-0.13337	0.732697	0.101381
Q9Y6I4	USP3	0.228359	0.41117	-0.07453	0.011713	-0.20658	-0.33554	-0.03471	-0.8439	0.027933	0.449538	-0.3774	0.357085	0.074385	0.54014	-0.20995	0.394704	0.54107	0.137077	0.159764
Q9Y6I9	TEX264	-0.05448	0.513327	0.496036	0.037488	-0.07314	0.465734	0.771765	0.095789	-0.09809	-0.4756	0.409417	-0.52	-0.06076	-0.24367	-0.61609	-0.45901	-0.13529	-0.02856	-0.47662
Q9Y6J0	CABIN1	0.373645	-0.03934	-0.34603	0.237275	-0.42073	-0.57634	-0.18775	0.068942	-0.01924	-0.12003	0.130597	-0.60295	-0.09712	-0.36891	0.04758	0.166176	-0.44438	0.388205	-0.48337
Q9Y6J9	TAF6L	0.256754	-0.03383	0.044214	0.182047	-0.13808	0.520732	0.121128	0.335586	-0.20253	-0.03788	0.148451	-0.31455	-0.1203	-0.16028	0.356376	-0.08513	-0.21446	-0.04219	-0.17169
Q9Y6K1	DNMT3A	1.078375	-0.08738	-0.3923	-0.08736	-0.48918	-0.18864	0.061274	0.955388	-0.18635	-1.02269	-1.15728	-0.62472	-0.1223	-0.15919	-1.34056	-0.96189	-0.49958	-0.51183	0.848602
Q9Y6K5	OAS3	1.314726	1.672042	2.391611	-0.8443	0.32135	0.889409	0.209606	1.258233	-0.43248	-0.78766	-1.04636	0.899674	0.342421	0.281152	-1.09886	-0.63541	0.108406	-0.03811	-1.0427
Q9Y6K9	IKBKG	-0.33085	0.259431	1.319334	-0.41169	0.47625	0.883058	-0.2265	-0.43937	-0.01117	0.279667	-0.12213	0.383917	0.194294	0.559004	-0.20782	-0.06767	0.362064	0.390055	-0.07428
Q9Y6M1	IGF2BP2	-1.11219	-0.61715	-0.12508	1.075776	-1.54707	-1.50844	-0.78826	0.086696	0.132876	0.477262	-0.30447	-0.68232	-0.6226	-0.44872	0.932423	-0.36526	0.604722	-0.30865	0.784625
Q9Y6M5	SLC30A1	0.046639	0.538443	0.033061	-0.15028	-0.78634	-0.79451	0.406479	-0.62429	-0.34264	0.125984	0.598717	0.201855	0.474922	-0.01753	1.596451	-0.53429	-0.32233	-0.39484	-0.03725
Q9Y6M7	SLC4A7	0.076289	0.053147	-0.15279	-0.22099	0.225406	0.155223	0.307078	-0.21542	0.040847	0.074272	-0.06847	0.672798	-0.90845	0.146005	-0.22651	-0.30433	-0.03936	0.066354	-0.43881
Q9Y6M9	NDUFB9	-0.17009	0.235015	0.399937	-0.43196	0.122321	0.438306	0.510137	-0.38279	0.186363	-0.90058	0.433379	-0.00125	-0.03444	0.006141	0.489422	-0.55307	-0.59953	-0.21277	-0.74076
Q9Y6N5	SQOR	-1.16178	1.168792	1.508472	0.472736	0.502068	0.108632	0.382969	-1.73648	-0.22554	0.130365	-0.43621	0.9866	0.281371	0.850685	0.022187	0.768815	-0.10824	-0.39267	-1.26202
Q9Y6N7	ROBO1	-0.44554	0.180273	0.265224	-0.92938	-0.53584	-0.81303	0.083158	-0.41155	0.308123	-0.18741	-0.33133	-1.686	-0.05628	-0.50348	-0.76282	-0.24061	-0.76682	0.171904	0.57106
Q9Y6Q2	STON1	-0.37728	0.3088	-0.15202	-1.0462	0.610701	0.224482	0.117707	0.095153	0.528208	-0.03415	-0.99309	-0.4231	0.658686	-1.0518	-0.6709	0.955185	-0.98212	0.095338	0.502784
Q9Y6Q5	AP1M2	-0.30384	-0.34714	-0.60203	0.241072	-0.81642	-0.32728	-0.08823	-0.9466	-0.25752	-0.28399	1.029366	-0.31935	-0.62367	-1.18682	0.070248	0.495481	-0.67638	-1.03275	-1.31252
Q9Y6Q9	NCOA3	-0.02467	0.144714	0.057974	-0.38201	0.071669	-0.34696	-0.16249	-0.06345	0.597081	-0.04028	-0.42089	-0.17315	-0.3174	-0.13795	0.403933	0.749868	0.06675	0.129903	-0.22372

Q9Y6R0	NUMBL	0.110715	0.270036	0.1871	-0.39782	-0.52336	-0.38741	0.499297	0.179423	0.09373	-0.14645	-0.49234	-0.46031	0.315809	-0.06346	0.897538	-0.34202	0.094855	0.582629	1.131282
Q9Y6R4	MAP3K4	-0.29529	0.32147	0.081184	0.37329	-0.24254	-0.46706	0.323364	-0.35779	0.241421	0.240535	0.127682	0.081616	0.119922	0.186419	0.993784	0.184713	0.527658	0.11609	0.052186
Q9Y6R7	FCGBP	-1.01338	-0.29957	0.05883	0.088865	0.87739	1.875805	-0.44565	-1.10744	0.058026	-0.26333	1.29568	-1.68608	-0.4014	-0.65749	0.508288	1.721073	-0.50032	-1.72621	-1.00807
Q9Y6R9	CCDC61	0.212221	0.47424	0.130242	-0.81856	-0.3314	0.675332	-0.18106	-0.953	-0.19984	-0.59412	-0.49036	-0.49553	-0.01566	0.390815	0.806592	-0.5522	-0.57968	-0.6374	-0.83789
Q9Y6U3	SCIN	-0.72489	0.212764	-0.37341	-0.31322	1.277365	0.10898	-0.20279	-0.69774	0.846197	1.041883	-0.34853	-0.51384	0.139575	0.457997	0.542792	0.893359	1.587478	2.432511	0.891942
Q9Y6V7	DDX49	0.853356	-0.21417	-0.06735	-0.07758	-0.36626	-0.29858	-0.06103	-0.12145	-0.1688	-0.34442	-0.07647	-0.07909	-0.53458	0.010105	-0.29856	0.046876	0.259278	-0.10976	-0.20863
Q9Y6W3	CAPN7	-0.1248	0.528995	-0.13209	-0.71671	-0.48027	-0.23828	0.303976	-0.30336	0.079242	-0.26383	-0.48105	0.163758	0.030462	0.404579	0.572621	0.446143	-0.30865	-0.68099	-0.06543
Q9Y6W5	WASF2	0.104308	0.5444	0.27409	-0.50614	0.221675	0.076721	-0.33957	-0.12085	-0.3689	0.359442	-0.08185	-0.12494	-0.00267	-0.35187	0.209128	0.168947	-0.36905	0.367188	-0.26475
Q9Y6X3	MAU2	0.729095	0.031531	-0.17218	-0.16509	-0.22921	-0.0683	0.105675	-0.15016	-0.18143	-0.28571	0.21509	-0.42588	-0.44559	0.384307	0.215495	-0.11466	-0.19772	0.003592	-0.47679
Q9Y6X4	FAM169A	-1.04861	-1.59121	-0.12748	-0.75838	-1.00922	-0.89739	-0.30377	0.736261	0.077104	-1.3695	0.719501	-0.98959	-0.80996	-1.30424	-0.42851	-0.87263	2.226685	4.905015	-0.53055
Q9Y6X5	ENPP4	0.858651	0.342752	1.151409	-0.5144	-0.19845	0.185096	-0.03289	0.157988	-0.09227	0.024471	-0.26055	0.545205	-0.00658	0.951153	-0.1823	0.229827	0.551206	-0.13528	-0.65381
Q9Y6X8	ZHX2	-0.60893	-0.15411	-0.3249	-0.72789	-0.3944	-0.18251	0.098024	0.617581	0.236254	-0.62983	-0.36256	-0.36952	0.036592	-0.49799	0.097337	-0.81374	-0.63071	-0.11055	-0.40437
Q9Y6X9	MORC2	0.028363	0.00938	-0.42517	0.043873	-0.05622	-0.03647	0.094604	0.384118	-0.23809	-0.69305	0.061889	-0.71544	-0.80929	-0.24044	0.493866	-0.07007	0.125346	0.392137	-0.27718
Q9Y6Y0	IVNS1ABF	-0.30656	-0.77945	-0.31912	0.263079	-0.24414	-0.68672	0.148624	-0.22811	-0.1147	-0.10067	0.264422	0.182965	0.161006	0.645843	0.243591	0.115163	0.718722	-0.46279	-0.42786
Q9Y6Y8	SEC23IP	-0.99717	0.626844	0.25493	0.174832	-0.60279	-0.49376	0.463403	-0.36662	-0.275	0.165844	0.034297	-0.00272	0.423676	-0.09307	0.882697	0.362156	0.536193	0.483076	0.327963

Supplemental Table 13: Global proteomic data for pre-NACT ER and pre-NACT PR tumors.
NACT= Neoadjuvant chemotherapy, PR= poor responder, ER= excellent responder

Accession_Sites_nu	Accession	Gene	PR-PR-2-1	PR-PR-8-3	PR-ER-1-2	PR-PR-3-1	PR-ER-6-1	PR-PR-1-2	PR-PR-7-1	PR-PR-8-1	PR-PR-10-	PR-PR-5-2	PR-PR-6-3	PR-ER-8-3	PR-ER-6-3
A1L390_S488_1_1_	A1L390	PLEKHG3	0.532497	0.177953	0.136154	0.092913	-1.12026	-0.87058	-1.91127	-0.34865	-0.40409	-0.04763	0.940969	0.337835	-0.13056
A1L390_S631_1_1_	A1L390	PLEKHG3	0.29515	0.471697	0.50151	-0.06833	-0.18243	1.036566	-0.37626	-1.08916	-0.79769	-0.57333	-0.04051	0.48166	-0.40322
B2RTY4_S1299_1_	B2RTY4	MYO9A	0.495808	1.004273	-0.27935	0.079036	-0.74396	-0.77103	0.11191	-0.60757	0.873326	-0.06209	0.668394	-0.22292	0.132607
O00159_S408_1_1_	O00159	MYO1C	0.137851	-1.08896	0.222606	0.301226	-0.73423	-0.56032	-0.37164	0.232507	-1.43347	0.113971	-0.37115	-0.00342	-0.24599
O00193_S17_1_1_N	O00193	SMAP	-0.03697	-0.0151	-0.54916	2.129863	0.542888	-0.59796	-0.09627	0.946944	0.215489	0.849356	-0.27332	-0.10248	0.679737
O00203_S276_1_1_	O00203	AP3B1	0.652824	0.048462	-0.54935	-0.85512	-0.25631	0.191345	-0.21853	0.911719	0.185122	-0.54971	0.113349	-0.7033	-1.13616
O00231_S14_1_1_N	O00231	PSMD11	-0.10055	-0.30792	-0.45688	-0.26771	-1.23015	-1.49	-0.58042	-1.64033	-0.20307	-0.85858	-1.78166	-1.35027	-0.99186
O00410_S827_1_1_	O00410	IPO5	-0.24026	-0.09337	-0.88817	0.763133	0.17797	0.198411	0.391268	0.462253	0.09968	0.832635	0.821376	-0.25132	-0.11911
O00418_S18_1_1_N	O00418	EEF2K	1.068943	-0.17989	0.640248	-0.50136	-0.91934	-0.22015	-0.26132	0.595208	-0.36554	-0.46226	0.447706	-0.3573	-0.37555
O00470_S196_1_1_	O00470	MEIS1	0.061614	-0.54715	-0.67607	-0.54228	-0.26809	0.220164	0.453318	-0.27161	0.552021	-0.65908	-0.29439	0.417034	0.020253
O00505_S60_1_1_N	O00505	KPNA3	0.184356	0.330061	-0.37429	0.173117	-1.20785	0.544108	0.60294	-0.0717	0.208301	-0.19414	-0.50559	-0.31985	-0.11863
O00512_S687_1_1_	O00512	BCL9	-0.31412	-0.37682	-0.35365	-0.27104	0.677175	0.253258	-0.45899	-0.20657	0.398164	0.369025	0.087439	0.532206	0.459568
O00567_S520_1_1_	O00567	NOP56	0.603782	0.080665	-0.20214	-0.31854	-0.70903	0.475144	0.060232	-0.44469	0.149895	0.003754	0.30367	-0.39386	-0.10034
O00712_S264_1_1_	O00712	NFIB	0.782411	-0.44664	0.753242	-0.90566	-2.12044	-0.7667	-0.63787	-0.81678	-0.04499	-0.21875	-0.34908	-1.26686	-1.06726
O14497_S1754_1_(	O14497	ARID1A	-0.20625	0.014029	-0.37705	0.754796	-0.15839	-0.05684	-0.40702	0.209965	-0.10042	0.620347	-0.69157	-0.09749	0.573551
O14497_S1755_1_1	O14497	ARID1A	0.002374	-0.09056	-0.18805	0.295705	-0.41059	0.089616	-0.91758	0.099751	0.016593	1.022601	-0.26769	-0.11632	0.268213
O14530_S221;S223	O14530	TXNDC9	0.11391	-0.02636	0.017446	0.298874	0.121398	-0.23661	0.269506	-0.52811	-0.53906	-0.17308	0.274046	0.079368	0.522975
O14545_S415_1_1_	O14545	TRAFD1	0.268185	-0.42203	-0.49709	1.054505	-0.04275	-1.21502	-0.5731	0.700264	-0.79356	0.684707	-0.54233	0.869702	0.276338
O14545_T414_1_0_	O14545	TRAFD1	-1.21737	-0.48133	0.718454	0.637648	0.197577	0.504965	0.061863	0.672992	0.110285	0.952036	0.365096	0.790771	-0.12726
O14558_S16_1_1_N	O14558	HSPB6	-0.83695	-0.18841	0.207151	1.123103	-0.16742	-0.82894	0.665273	0.982814	-0.52394	0.620949	0.076334	0.540087	1.980051
O14639_S431_1_1_	O14639	ABLIM1	0.197703	-0.68815	-0.21581	1.349585	-0.69753	0.316424	-0.87744	0.398796	0.657631	0.562062	-0.33234	0.177602	-0.8535
O14639_S655_1_1_	O14639	ABLIM1	-0.11838	-0.63387	-0.95227	0.712043	0.132078	-0.58681	-0.34587	0.309024	0.84469	0.759543	0.179638	0.86347	-0.51258
O14639_S706_1_1_	O14639	ABLIM1	-0.3768	-0.54839	-0.21471	0.418176	0.163416	-0.39254	-0.60463	0.403938	-0.88007	0.517997	-0.41511	-0.65073	0.019854
O14683_S14_1_1_N	O14683	TP53I11	0.163511	-0.5369	1.295394	0.08198	-1.0629	-0.03352	-0.02632	-0.79248	-1.55183	0.108317	-0.36404	-0.59211	0.17867
O14737_S119_1_1_	O14737	PDCD5	-0.58137	-0.34349	-0.52949	-0.41275	-2.02761	0.227224	0.608225	-0.8212	0.014594	0.170627	0.659244	-0.17385	0.375012
O14974_S507_1_1_	O14974	PPP1R12/	1.095445	-0.3308	0.719261	-0.22968	-2.04254	-0.72064	0.743494	-0.7968	1.056239	0.83532	0.530848	-0.18671	-1.2686
O14974_S668_1_1_	O14974	PPP1R12/	1.057708	1.486268	0.142978	0.425936	-0.25296	1.540046	1.721059	-0.07949	0.155203	1.054375	0.252663	-0.18336	0.529794
O14974_S910_1_1_	O14974	PPP1R12/	0.093461	-1.64038	-0.66021	0.471139	-1.26691	-0.33256	0.071943	-1.47756	-0.90193	0.05091	-1.1837	-1.64146	0.701857
O14974_T443_1_1_	O14974	PPP1R12/	-0.07707	-0.6016	0.645492	0.863074	-1.59368	0.460585	0.287752	1.180289	-0.55388	0.795349	0.132891	-0.00266	-0.37726
O15014_S560_1_1_	O15014	ZNF609	-1.02267	-1.07385	-0.26142	0.591929	0.131217	-0.27235	-0.37723	-0.04232	-0.45614	1.375136	-0.53974	-1.50963	-0.47792
O15173_S104_1_1_	O15173	PGRMC2	-1.1557	-4.03611	-1.04665	3.188082	-0.04627	-0.53636	-2.85981	3.626076	-0.24787	1.335748	-0.8117	0.195921	-0.65602
O15173_T211_1_1_	O15173	PGRMC2	0.692429	-0.11222	0.119614	0.365873	0.699869	0.750033	-0.32815	0.378288	0.109763	0.694118	-0.05378	-0.16624	-0.00348
O15231_S453_1_1_	O15231	ZNF185	0.115136	-0.34004	0.068753	0.823501	0.671343	-0.09805	-1.47977	0.238635	0.015195	0.967493	-0.87004	0.149186	-0.31255
O15400_S129_1_1_	O15400	STX7	-0.78064	0.400699	-0.42266	0.424255	-0.74652	0.016336	-0.50756	-0.51801	0.603842	0.312566	-0.4089	-0.52358	0.028263
O15551_S199_1_1_	O15551	CLDN3	1.233882	-0.70319	-1.0356	1.108851	-2.61647	-1.14428	0.515046	-1.1299	-0.15747	0.101247	0.87429	-0.7679	-2.38825
O15551_S209_1_1_	O15551	CLDN3	-0.10684	0.390327	-1.57716	2.213759	-0.03715	-1.5458	0.244409	-0.31498	-1.50766	0.829861	-1.10984	-0.58786	-1.69173
O43150_S701_1_1_	O43150	ASAP2	0.027024	0.448794	-0.00953	0.234705	0.15652	-1.57598	-1.96229	1.088093	-1.97485	0.783534	0.050711	-1.05132	-0.14129
O43166_S1549_1_1	O43166	SIPA1L1	-0.03012	-0.35324	-0.35354	0.198666	-1.47848	0.033106	-0.46134	-1.0511	0.444469	0.194541	0.068515	-0.37476	-1.01568
O43166_S1585_1_1	O43166	SIPA1L1	-0.08237	-0.34529	-0.31812	-0.18522	-1.11607	0.448345	-0.03767	0.089062	0.095207	0.103797	-1.0301	0.795956	0.23453
O43166_S208_1_1_	O43166	SIPA1L1	-0.12404	-1.17898	-0.23034	-0.2358	-0.51649	-0.18678	-0.42937	-0.91854	0.093024	-1.05042	-1.06649	0.723413	-0.07307
O43290_S448_1_1_	O43290	SART1	0.468478	0.066372	-0.18994	0.023723	-0.50896	-0.09397	-0.5467	-0.64998	0.223851	-0.07219	0.125812	-0.45995	0.185964
O43310_S299_1_1_	O43310	CTIF	-0.43312	-0.20485	-0.72002	0.826719	0.256606	-1.85176	-1.62639	1.320408	0.046132	0.870777	-0.14678	-0.02732	1.398418
O43399_S166_1_1_	O43399	TPD52L2	-0.72772	0.054575	0.215494	-0.24351	-1.58608	-0.64883	0.042111	0.408596	0.469432	0.467158	0.028571	0.679946	0.389663
O43491_S718_1_1_	O43491	EPB41L2	1.797141	-0.40804	0.019538	0.003631	-1.74081	0.298531	-0.71673	-0.27923	-1.5888	0.25781	-0.95285	0.107044	-1.21942
O43491_S87_1_1_N	O43491	EPB41L2	0.306612	-0.4261	0.470601	-0.59354	-2.07816	-0.95286	-0.35209	-1.86198	-0.41556	-0.2749	-0.34697	-0.78681	-0.86382
O43493_S298_1_1_	O43493	TGOLN2	0.219972	0.282078	-2.46855	0.374036	-0.50668	0.394972	0.787303	1.047766	0.380071	0.520478	-0.46925	-0.22313	-0.47205
O43583_S73_1_1_N	O43583	DENR	-0.79406	-0.37776	0.107836	0.254841	1.339443	1.013029	-0.72149	0.038356	-0.76928	0.55583	-0.39063	-0.19238	-0.89499
O43719_S642_1_1_	O43719	HTATSF1	-0.13997	-0.20509	-0.59235	0.611006	-0.1231	0.102193	0.130857	-0.11528	-0.18989	0.050832	-0.27253	0.070563	0.249522
O43719_S676_1_1_	O43719	HTATSF1	0.468958	0.558461	-0.51469	-0.38736	-0.09827	0.43883	0.202268	0.356435	-0.16412	0.136856	0.342986	-0.49793	-0.1526
O43719_S702_1_1_	O43719	HTATSF1	0.604555	0.112244	-0.58282	1.032145	0.551195	0.068778	-0.12879	0.494215	0.301192	-0.02444	-0.97368	-0.89564	-1.71456
O43719_S713_1_1_	O43719	HTATSF1	0.546614	0.702163	-0.11715	0.042385	0.698961	1.127709	-0.23084	2.558332	0.917387	0.725696	-1.57606	-1.31093	-1.40016
O43719_S714_1_1_	O43719	HTATSF1	0.059173	-0.22482	-0.88125	1.042572	0.794195	0.358059	-0.44461	1.014903	-0.90762	0.586374	-0.75508	-1.36515	0.612085
O43719_S721_1_1_	O43719	HTATSF1	0.542994	0.504925	-1.23117	-0.25774	0.730761	0.400625	-0.28335	0.746817	-0.10578	-0.24867	-1.01656	-1.10569	-0.80938
O43719_Y650_1_1_	O43719	HTATSF1	-0.15679	0.499311	-0.24236	0.269659	-0.67052	0.105439	-0.07477	-0.15098	-0.03338	-0.10043	-0.04433	-0.90366	0.08287
O43815_S245_1_1_	O43815	STRN	-0.30166	-0.3846	-0.22703	1.076273	-0.76236	-0.02736	-0.16669	-0.40924	-0.21115	0.329087	0.556718	-0.66428	-0.43883
O43847_S94_1_1_N	O43847	NRDC	0.213204	0.177239	-0.30888	0.728005	0.024075	0.116304	-0.19039	-0.63517	-0.04204	0.213452	-0.62447	-0.36357	0.001641
O60333_S1057_1_1	O60333	KIF1B	0.102945	-0.31992	-1.43811	0.148778	-0.78414	0.374892	0.643643	-0.01987	-0.94224	0.318437	0.088221	-0.74233	-0.09945
O60343_S588_1_1_	O60343	TBC1D4	0.716286	0.379648	-0.45989	0.077563	-1.8198	0.553038	0.382325	-0.91269	0.388401	-0.05324	-0.47949	-0.19456	-0.56552
O60343_S591_1_1_	O60343	TBC1D4	-0.98972	0.077258	-1.18449	0.500359	-0.09192	0.015402	-1.07059	0.161058	-0.2849	0.513157	-0.3021	0.221761	0.452021
O60437_S14_1_1_N	O60437	PPL	0.128998	0.197533	-0.73817	-0.48713	0.584332	1.041523	0.562265	0.63818	0.31243	0.619971	0.149076	-0.01518	-0.68876
O60437_S887_1_1_	O60437	PPL	0.86416	0.83355	-0.57585	-1.0045	-1.40275	0.152309	-1.37778	0.487414	0.735553	-0.05273	1.649276	-0.14693	-0.1421
O60493_S72_1_1_N	O60493	SNX3	1.037387	-0.62064	0.264965	0.060336	0.042555	0.438032	-1.27593	0.058497	0.002612	-0.27835	0.20302	-0.27044	-0.49633
O60504_S545_1_1_	O60504	SORBS3	0.474034	0.821727	-0.1203	0.047151	-0.31847	-0.24114	-0.22346	-0.93135	0.29827	-0.128	-0.25181	-0.07597	-0.00745
O60524_S417_1_1_	O60524	NEMF	-0.09581	0.583566	-0.90507	0.131615	0.100632	0.426001	0.476245	-0.03811	0.387679	0.121107	0.198289	0.286421	0.17345
O60716_S230_1_1_	O60716	CTNND1	0.114168	-1.1302	-0.10016	-0.0156	-1.13847	0.221973	0.63137	0.228967	1.269234	0.14123	-0.95166	-0.6903	-0.16594
O60716_S252_1_1_	O60716	CTNND1	0.026218	-1.82288	0.670088	-0.00772	-1.58629	0.271638	0.745598	-0.87414	1.521683	0.041375	-0.00808	0.349484	-0.25419
O60716_S268_1_1_	O60716	CTNND1	0.652505	-1.15506	0.131172	-0.07526	-3.63678	-0.11822	0.124381	-1.84261	0.558547	0.165362	-0.20494	-1.39339	-0.9478
O60716_S288_1_1_	O60716	CTNND1	0.100903	-0.55676	-0.57768	1.058105	0.777665	0.244011	0.184646	0.752334	0.381109	0.83049	-0.81266	-1.274	0.761008
O60716_S346;S349	O60716	CTNND1	0.327004	-0.63994	-0.75652	-0.75288	-1.41823	0.740574	-1.20021	-0.30274	-1.12539	-1.35158	-0.58301	-1.30569	-1.78156
O60716_S346_1_1_	O60716	CTNND1	-0.64305	-0.8491	-0.72134	0.07587	-0.32701	-0.07974	-0.33305	0.521891	0.076461	-0.21747	0.374892	-0.31205	-0.39113
O60716_S349_1_1_	O60716	CTNND1	0.429207	0.570269	-0.4202	0.102659	-0.51232	0.566595	-0.31124	-0.24823	0.470442	-1.28613	0.477148	0.187783	-0.39056
O60716_S352_1_1_	O60716	CTNND1	-0.12091	-0.48733	-0.44517	0.455144	-0.64128	-1.00571	1.037006	-1.79935	0.373886	-0.17702	0.381652	0.652438	-0.65304
O60716_S861;S864	O60716	CTNND1	0.530065	-0.12091	-0.4772	1.516335	0.121617	-2.13467	0.854223	0.755501	0.908809	0.619546	-0.09601	-0.44107	0.163412
O60716_S864_1_1_	O60716	CTNND1	0.059937	0.333495	-0.19788	-0.1263	-0.39424	-0.017	0.547828	1.448936	0.790414	0.767513	-0.0606	0.251151	0.542606
O60716_S920_1_1_	O60716	CTNND1	1.404295	0.448218	-0.29028	-0.75838	-1.63441	0.519711	0.016077	-0.36015	0.024217	-0.06516	-0.45714	-1.37265	-0.99297
O60716_T869_1_1_	O60716	CTNND1	-0.32812	-0.72556	-0.24966	0.524489	-0.49578	-0.16927	0.385482	0.384498	0.836659	0.214899	-0.59128	-0.4317	-0.94793
O60749_S119_1_1_	O60749	SNX2	0.103408	-0.84518	-0.18602	0.442481	-1.16036	-2.2903	-1.76838	-0.01204	-0.39848	-0.14376	-2.68379	-1.21684	-1.00503
O60784_S355_1_1_	O60784	TOM1	-0.1088	-0.53803	-0.77573	0.976958	-1.21381	-2.48581	-1.46202	0.153479	-0.18448	0.49293	-0.00219	-0.71451	0.896221
O60832_S494_1_1_	O60832	DKC1	-0.54914	-0.58724	-0.79982	-0.09407	0.367139	0.186338	0.047848	0.009556	0.069798	0.607644	0.410053	-0.53166	-0.32456
O60841_S113_1_1_	O60841	EIF5B	-0.1464	-1.25765	-0.53446	0.489498	0.14092	0.520595	-0.23344	-0.32763	-0.22735	0.2972	-0.29358	-0.36359	-0.22387
O60841_S137_1_1_	O60841	EIF5B	-1.44557	-1.75965	-0.10741	1.042846	0.817038	0.291378	-1.91714	1.446301	-0.73725	1.357572	-1.09825	-1.7182	-0.02837
O60841_S164_1_1_	O60841	EIF5B	-0.34856	-0.2365	-0.49188	0.150633	-0.79594	0.016764	0.018229	-0.4421	0.339116	0.291984	0.140949	0.412967	-0.05391
O60841_S214_1_1_	O60841	EIF5B	-0.32871	0.018785	-0.0618	-0.23522	-0.9569	-0.08073	0.431487	-0.72104	-0.35965	-0.07384	0.504546	-0.55333	0.149178
O75122_S368_1_0_	O75122	CLASP2	-0.46387	-1.16004	-0.08265	-0.2954	-1.62516	-0.57043	0.473492	-0.56362	-0.87684	0.237011	-0.67332	-1.00893	-0.1652
O75376_S1472_1_1	O75376	NCOR1	0.141306	-0.47899	-0.16427	-0.05842	-0.58804	-0.58003	-0.48528	-0.15746	-0.63291	0.226843	0.568922	-1.40861	-2.01819
O75376_S2120_1_1	O75376	NCOR1	-0.48772	-0.40167	-0.39616	1.442874	0.051491	-0.28852	-1.14095	1.075809	-0.1936	0.901919	0.229079	-0.13477	-1.31006
O75379_S30_1_1_N	O75379	VAMP4	-0.40936	-0.35951	-0.72254	0.940282	0.246451	0.163029	0.341763	0.522407	0.055981	0.270842	0.482849	-0.20326	0.209576
O75396_S137_1_1_	O75396	SEC22B	0.749543	-0.11271	-0.17853	0.877574	-1.48592	-0.82962	0.979779	-0.62116	-0.31283	0.361178	-2.51657	-0.77083	-0.72733
O75400_T373_1_1_	O75400	PRPF40A	-0.01011	0.484134	-0.28679	-1.03327	0.12628	0.609017	-0.562	0.382116	0.32957	-0.78054	-1.6937	-0.57286	-0.08973
O75420_S638_1_1_	O75420	GIGYF1	0.286454	-0.11691	0.460911	-0.10766	-1.77234	-0.50443	0.388412	-0.70553	-0.30925	-0.74038	-0.74656	-0.44718	-1.18636
O75475_S105_1_0_	O75475	PSIP1	-1.29973	-0.0576	-1.19166	0.470874	-0.45433	-0.62349	-0.14214	-0.168	0.460031	-0.15245	-0.95423	-0.79483	-1.10526
O75475_S106_1_1_	O75475	PSIP1	0.486979	0.351416	-0.76028	1.031985	-0.17488	0.326737	-0.33638	-0.64906	-0.41817	0.376905	-1.1638	-1.02026	-0.87775

O75475_T122_1_1_	O75475	PSIP1	0.392291	0.368251	-1.25065	0.680677	-0.3635	0.62049	0.356059	-0.83126	1.013945	0.385084	0.976664	-0.37337	-0.82653
O75494_S131;S133	O75494	SRSF10	0.952315	0.112514	-0.38075	-0.0352	0.191074	0.250728	-0.74276	-0.39866	0.262389	0.21988	-0.08746	-0.52365	-0.68679
O75533_S129_1_1_	O75533	SF3B1	1.299649	1.331681	-0.11401	-0.44753	-0.04801	1.524769	0.198664	0.548542	2.498619	1.080518	-0.13104	-0.44918	-0.74501
O75569_S18_1_1_N	O75569	PRKRA	-0.63337	-0.75529	-0.39163	-0.42907	-0.65037	-1.82282	-0.21098	-1.45906	-0.54316	-0.48854	-1.74377	-0.60322	-0.47973
O75592_S3440_1_1	O75592	MYCBP2	0.667581	-0.2508	0.498966	1.174156	-0.01755	-1.48502	0.333548	0.030286	0.25955	0.509034	-0.21434	-2.86909	-0.33008
O75909_S340_1_1_	O75909	CCNK	0.097249	0.177778	-0.66602	0.067542	-1.02781	0.578789	0.178731	-0.50616	0.14402	0.282426	0.074088	-0.87246	0.867322
O76021_S361_1_1_	O76021	RSL1D1	-0.43236	-0.87232	-1.06996	1.434306	0.282465	-0.37703	-0.43428	-0.00899	-0.24603	0.951612	-0.4071	-0.15905	-0.18639
O76021_S427_1_1_	O76021	RSL1D1	-1.71904	-0.04376	0.027229	0.073445	0.726799	0.093765	0.199231	0.257111	0.386071	1.2572	-0.68722	-0.32492	0.045072
O76024_S32_1_1_N	O76024	WFS1	0.007394	2.261168	-1.13539	0.483141	-0.99119	0.153952	0.752898	0.336418	1.19586	0.675536	0.486788	1.392967	0.131803
O94874_S458_1_1_	O94874	UFL1	-0.09018	-0.08971	-0.35047	0.76307	0.049978	-0.51761	-0.32124	1.171028	-0.73127	0.820572	-0.34535	-0.23028	0.386454
O94888_S280_1_1_	O94888	UBXN7	0.256682	0.032897	0.606402	0.734027	-2.4081	-1.50678	-0.36455	1.31688	0.075162	0.598981	0.441923	-0.21819	-0.37507
O94923_S73_1_1_N	O94923	GLCE	-0.60798	-0.0618	-1.63262	0.612617	-0.20287	-0.91832	0.544216	0.579813	-0.07355	0.37031	0.737189	-0.27618	-0.3284
O94964_S1017_1_1	O94964	SOGA1	0.177338	-0.25073	-0.08461	0.347579	0.09158	0.335538	0.339462	-0.26208	0.239959	0.323968	-0.23404	0.478958	0.049536
O94979_S527_1_1_	O94979	SEC31A	0.120068	-0.49844	-0.21068	-1.15286	-0.36698	1.428072	0.519586	-0.07485	-0.22653	-0.32735	-0.29628	-0.31181	-0.94752
O94979_S799_1_1_	O94979	SEC31A	0.112467	-0.78906	-0.57749	0.191181	0.017085	0.235189	0.1895	-0.19866	-0.28614	0.150056	-0.534	0.016229	-0.06317
O95071_S1549_1_1	O95071	UBR5	0.503072	-0.05418	-0.34541	-0.23618	-0.59471	-0.2356	0.01647	-0.85032	0.458077	-0.741	-0.44875	-0.44349	0.651982
O95218_S120_1_1_	O95218	ZRANB2	-0.20106	-0.2717	-0.33875	1.494142	-0.24602	0.115452	0.092787	0.526743	-0.06879	0.948894	0.069751	-0.17182	0.224938
O95218_S153_1_1_	O95218	ZRANB2	0.024626	-0.1788	-0.45378	1.069567	-0.42004	-0.0195	-0.22288	0.304001	0.234405	0.558803	-0.04433	-0.29698	0.060539
O95218_S188_1_1_	O95218	ZRANB2	0.187745	0.065384	-0.65657	0.797627	-0.1863	-0.04317	0.222383	0.658029	0.516102	0.692993	0.318814	-0.01328	-0.03642
O95239_S801_1_1_	O95239	KIF4A	0.387083	-0.34441	1.325007	-0.33596	-2.06121	-1.35446	0.560734	-1.18597	0.146791	-0.70663	-1.73619	-2.1412	0.184037
O95251_S57_1_1_N	O95251	KAT7	-1.24661	0.622175	-1.74571	-0.52185	0.567939	0.98652	0.079162	-0.33593	-0.51365	-1.10318	0.044278	-0.50618	0.917679
O95297_S260_1_1_	O95297	MPZL1	0.391463	-1.19901	-0.60466	0.148966	-0.59524	-0.75584	0.391799	-0.27879	-1.0185	0.07791	-1.0571	-0.29594	-0.31893
O95297_Y263_1_1_	O95297	MPZL1	1.133581	-0.55865	-0.83101	0.030778	-1.3946	-1.42039	0.674841	-0.30278	1.139979	0.025098	-0.64976	-0.22555	0.053318
O95391_S215_1_1_	O95391	SLU7	-0.68925	-0.26288	-0.17259	0.933304	-0.05272	-0.06011	-0.10223	0.459185	-0.36085	0.374128	0.362028	0.323316	0.26615
O95425_S245_1_1_	O95425	SVIL	-0.31348	-0.03398	-0.27794	0.14947	-0.22229	0.026662	-0.06684	-1.3301	0.113821	-0.34605	-0.00164	-0.07004	-0.60606
O95466_S624_1_1_	O95466	FMNL1	-0.8368	0.090965	-0.71241	-0.38785	-0.55728	-1.15862	0.393141	-0.09882	-0.4513	0.015023	0.024433	-0.95239	0.616554
O95714_S2928_1_1	O95714	HERC2	1.290157	1.54746	-0.01948	-0.15358	0.557133	0.386712	0.742433	1.11888	-0.59663	0.325072	1.128397	-0.08856	-0.73933
O95747_S339_1_1_	O95747	OXSR1	-0.0741	0.042314	-0.21095	0.274981	0.11095	-0.49827	0.067476	0.192937	-0.29834	1.003626	0.108395	0.270877	0.256395
O95772_S193_1_1_	O95772	STARD3NI	-0.2454	-0.09783	-0.84985	0.096742	0.112169	-0.53832	0.490081	-0.31897	-0.12598	0.178466	-0.38622	-0.46195	-0.27573
O95772_S39_1_1_N	O95772	STARD3NI	0.113812	-0.1674	0.620876	1.066885	-0.77392	-1.55008	-2.14906	0.453319	0.100001	0.880147	-0.34771	-2.00498	0.051476
O95817_S264_1_1_	O95817	BAG3	-0.02099	-0.73756	0.087978	0.951875	-1.49606	-0.21002	0.54434	0.116294	0.268583	0.788217	0.173247	-0.23703	0.213869
O95819_S631_1_1_	O95819	MAP4K4	-2.1005	-0.65324	-0.69092	1.353372	-0.23041	0.54832	-0.21908	-0.79365	0.609798	0.661164	0.547171	0.035961	-0.40917
P00761_S106_1_1_	P00761		-0.12609	0.652335	-0.83578	1.908655	-0.38642	-0.62012	-0.94649	0.929893	-1.26464	0.972178	0.088978	-0.43552	-0.38854
P00761_T103_1_1_	P00761		-0.17274	-0.70887	-0.56484	1.559788	0.840788	-0.45516	-0.28463	1.291664	-0.13715	1.481843	0.320822	-0.28741	-0.28987
P00761_T110_1_1_	P00761		-0.687	-0.52241	-0.58998	1.45135	0.118349	-0.26175	-0.26989	1.162048	-0.5766	0.877025	-0.32651	-0.47291	-0.38607
P02452_S176_1_1_	P02452	COL1A1	-0.70721	0.025763	0.92192	1.368147	1.147491	0.075329	-0.10608	2.226652	1.179507	0.754866	-0.36113	-0.32811	1.06966
P02545_S22_1_1_N	P02545	LMNA	-1.1221	-0.91036	-0.5912	-0.46539	-0.43821	0.1878	-0.84515	0.919025	-0.86302	0.474114	-0.36109	0.367528	-0.34057
P02545_S390_1_1_	P02545	LMNA	-0.07384	-0.2434	-1.16985	0.384773	0.389018	-0.15043	0.275923	0.097778	-0.0651	-0.32231	-1.17533	-1.49973	-0.64024
P02545_S392_1_1_	P02545	LMNA	-0.43364	-0.4671	-0.68286	0.752568	0.397713	-0.05329	0.270882	0.492779	-0.58182	-0.16048	-0.58006	-0.75742	0.363001
P02545_S403_1_0_	P02545	LMNA	0.036811	0.595038	-0.23796	-0.36169	-0.80265	-0.75204	-0.59496	-0.50795	-0.23775	-0.03524	-0.16433	-1.05558	-0.25306
P02545_S404;S406	P02545	LMNA	-1.44662	0.043506	-0.55316	0.836989	-0.56674	-1.00145	0.399293	1.882751	1.033192	0.595242	-1.92577	-0.95928	0.378563
P02545_S429_1_1_	P02545	LMNA	-0.9877	-0.11944	-1.30862	0.063909	-0.26659	0.292282	-0.37951	0.538225	0.688653	0.967224	-0.46896	-0.36877	0.096777
P02671_S274_1_1_	P02671	FGA	0.782374	1.381418	-0.29091	-1.27781	1.42905	0.639623	-0.0528	0.445782	0.850866	0.096726	-0.05028	-0.09716	2.648433
P02671_S594_1_1_	P02671	FGA	0.968055	2.779541	0.244038	-3.6359	1.148956	0.990171	1.074752	1.34639	1.876649	-1.49525	-0.81784	0.55025	2.757587
P04049_S621_1_1_	P04049	RAF1	-0.0776	-0.64305	-1.3835	-0.81276	-0.98925	1.674695	-0.21139	0.057876	0.86413	0.314579	-0.00377	0.584183	-0.36692
P04075_S39_1_1_N	P04075	ALDOA	-1.34345	0.365208	-1.69486	0.773674	-0.12432	-1.01702	-1.14143	0.16919	-0.31467	1.785504	0.287362	-0.91141	-0.94029
P04075_S46_1_1_N	P04075	ALDOA	0.679038	-0.10896	-0.47969	0.328433	-0.80523	-0.17743	-0.78136	0.183379	-1.24491	-0.04562	-0.138	-0.4506	-0.84663
P04114_S4048_1_1	P04114	APOB	-0.00174	-0.41006	-1.33564	1.570044	0.169414	-0.19367	-0.78066	1.354427	-0.75566	0.912113	-0.29837	-0.35828	-0.94355
P04626_S1054_1_1	P04626	ERBB2	-0.93386	1.138939	0.076098	0.943819	1.135015	0.646305	0.251075	-0.27763	-1.02291	0.659855	-0.04453	-0.74726	0.123828
P04792_S82;S86_2	P04792	HSPB1	0.173483	-1.88949	0.298104	-0.58484	-1.75553	-1.58688	-0.86867	-0.75603	-0.14845	-0.50642	-1.49258	-1.58277	-1.34485
P04792_S82_1_1_N	P04792	HSPB1	0.122498	-0.32118	-0.1387	-0.36229	-1.51258	-1.17205	-1.04461	-0.91308	-0.12262	-0.48266	-0.40741	-1.39047	-1.35384
P04792_S86_1_1_N	P04792	HSPB1	-0.18203	-1.41219	-2.37539	-0.02151	-0.34445	0.280509	0.100869	-0.55588	-0.37371	0.263753	-0.02879	-1.30001	1.544403
P05114_S7_1_1_N(	P05114	HMGN1	-0.11413	-1.85611	-0.5671	0.717055	-1.18269	-1.28056	-1.93865	0.008745	-1.98978	0.046453	-1.69269	-1.43586	-1.02132
P05387_S102;S105	P05387	RPLP2	0.008922	-0.33539	-0.33928	0.323435	-0.47839	0.509781	0.072176	-0.26422	0.439799	0.471717	0.441804	-0.22306	0.158697
P05387_S102_1_1_	P05387	RPLP2	-0.70875	-0.82834	-0.97531	0.961595	0.411912	1.573915	-1.21601	1.024319	-0.34608	0.99673	-0.85918	-0.97097	-0.10422
P05387_S105_1_1_	P05387	RPLP2	-0.24956	-0.88079	-0.73314	1.840316	0.419207	2.374807	-0.79509	1.707685	-0.0251	1.032464	-0.23965	0.091741	0.138972
P05388_S304;S307	P05388	RPLP0	-0.62272	-0.50752	-0.686	0.018925	-0.68379	-0.25429	0.246939	-0.83993	0.394198	0.030393	0.364766	-0.26558	-0.07088
P05388_S304_1_1_	P05388	RPLP0	-1.39079	-1.01522	-1.25104	0.588494	-0.00203	1.075301	-0.75	0.504403	-1.161	0.097376	-1.33808	-0.21332	0.047811
P05388_S307_1_1_	P05388	RPLP0	-0.90471	-1.01162	-1.97069	0.156113	0.229777	0.126178	-1.31131	-1.48793	-2.08116	0.52131	0.618411	-0.69956	1.044747
P05455_S366_1_1_	P05455	SSB	-0.33676	0.115543	-1.13703	0.114797	-1.26585	-0.29408	0.468582	-0.30431	0.203072	0.78781	-0.06944	-0.54858	0.106114
P05783_S420_1_1_	P05783	KRT18	-0.4696	-0.49843	0.392744	-0.63115	-0.82821	-0.27481	-0.65252	-0.52633	-0.92293	-0.30046	-0.08468	-1.83282	-1.48179
P05787_S24_1_1_N	P05787	KRT8	-0.60617	-0.83284	0.160317	1.444466	-0.21496	1.184816	0.368916	-0.26166	-0.52898	0.372585	-0.98342	-3.09995	-0.57303
P05787_S274_1_1_	P05787	KRT8	-2.54346	-1.22063	-1.32112	1.581643	0.203076	-0.01797	-0.51501	-0.72701	-0.83653	0.694301	-0.64146	0.881559	0.933894
P05787_S34_1_1_N	P05787	KRT8	0.13538	-1.18746	-0.57186	0.602702	-0.52646	1.272742	-0.19163	-0.17905	-0.79227	1.058359	-0.04788	-1.40888	-0.05005
P06239_T395_1_0_	P06239	LCK	1.154841	0.669264	-0.04224	-0.34731	-2.54049	1.084592	1.060013	0.860607	-0.03457	0.102348	0.346759	-0.01379	0.059619
P06239_Y394_1_1_	P06239	LCK	1.505934	0.851736	0.475609	0.326256	-2.19256	1.371701	2.261043	0.090592	0.335802	-0.18016	1.702092	-0.20285	-0.08637
P06241_T421_1_0_	P06241	FYN	0.274734	-0.25451	-0.33881	0.227532	-2.29015	1.146714	1.382529	0.151653	-0.92672	0.082867	-0.0792	-1.53965	0.081496
P06241_Y420_1_1_	P06241	FYN	0.625828	-0.07204	0.179045	0.9011	-1.94222	1.433823	2.583559	-0.05268	0.07625	-0.75703	0.789169	-0.47635	-0.06449
P06744_T109_1_1_	P06744	GPI	-0.80322	0.32979	-0.46004	1.72592	0.39597	0.483831	-0.52719	1.482013	0.628196	0.61271	-0.12393	-0.19084	0.337791
P06748_S70_1_1_Y	P06748	NPM1	-1.56697	-0.42866	0.32449	2.478948	1.325116	0.36271	0.632391	1.810213	0.142368	1.431347	-0.77902	0.035303	1.367433
P07355_S12_1_1_N	P07355	ANXA2	0.345495	0.876602	-0.24684	-0.94866	-0.22677	-0.00646	0.267106	-0.28582	0.60196	0.026424	0.145928	-0.73388	-1.30044
P07355_S26_1_1_N	P07355	ANXA2	0.894504	-1.10298	-1.45425	0.063118	-0.62267	-0.365	0.446943	-1.51415	-0.53463	-0.01381	-0.29733	-0.11139	0.573346
P07814_S885_1_0_	P07814	EPRS	0.244968	-0.6792	0.027783	1.014126	0.260309	-0.02633	-0.83797	0.444451	-0.41739	0.447774	-0.0834	-1.17068	1.229445
P07900_S231_1_1_	P07900	HSP90AA1	0.938352	0.518205	-0.34835	-0.05958	0.400004	0.678242	-0.3265	0.973697	0.202596	1.03291	-0.57917	-0.42917	-1.34771
P07900_S263_1_1_	P07900	HSP90AA1	0.309114	0.19338	-0.153	-0.51573	0.70898	0.441532	-0.35504	0.092452	0.047612	0.509934	-0.53411	-0.99484	-0.93729
P07910_S233_1_1_	P07910	HNRNPC	-0.3202	-0.13106	-1.34721	0.295818	0.010714	-0.31318	-0.05668	-0.18503	-0.51687	0.37181	0.716501	-0.76933	0.026095
P07910_S260_1_1_	P07910	HNRNPC	0.715646	0.168986	-0.22044	0.061998	0.132908	0.317424	-0.48262	0.19614	0.275928	0.495845	-0.42638	-0.92091	-0.73287
P07947_T427_1_0_	P07947	YES1	0.130166	0.571544	-1.38881	-0.55592	-2.3892	1.638804	0.175651	-0.62477	-0.63261	0.249514	-0.05764	-1.34772	-0.12768
P07947_Y426_1_1_	P07947	YES1	0.48126	0.754016	-0.87096	0.117645	-2.04127	1.925913	1.37668	-0.23184	0.066567	0.075123	-0.38384	-0.74937	-0.27367
P07948_S13_1_1_N	P07948	LYN	0.67636	0.470734	-1.25461	-0.17025	-0.24706	0.356323	-0.53512	0.661547	0.115717	-0.20671	-1.45165	-0.41222	-0.36071
P08238_S226_1_1_	P08238	HSP90AB1	0.400101	0.279008	-0.11958	-0.20994	0.496433	0.685739	-0.15748	1.023647	0.651279	0.591668	-0.67886	-0.59345	-1.43433
P08238_S255_1_1_	P08238	HSP90AB1	-0.08373	-0.35279	-0.03735	0.123308	0.057851	0.202838	-0.01958	0.273897	0.010424	0.377581	0.265441	-0.30213	0.09411
P08559_S293;S300	P08559	PDHA1	-0.51347	-0.13895	0.185337	0.060918	-0.95877	-1.9878	-2.14609	-1.11524	-0.80907	0.406905	1.183607	-1.08245	-0.93457
P08559_S293_1_1_	P08559	PDHA1	-0.12084	-0.14021	-1.46567	-0.3647	-0.44481	-1.29084	-0.95631	0.436955	-1.22616	2.13078	0.135857	-0.73722	-0.46684
P08575_S973_1_1_	P08575	PTPRC	0.481351	0.310318	-1.01074	0.942739	-0.06164	-0.51836	0.006696	0.395443	0.688027	0.845272	0.328774	-0.33799	0.233
P08621_S226_1_1_	P08621	SNRNP70	-0.50479	-1.18987	-0.34546	1.576431	-0.22884	0.017435	0.163119	-0.00249	0.547692	0.654733	-0.00557	-0.27819	0.350856
P08651_S323_1_1_	P08651	NFIC	0.547018	0.876908	-1.10913	-0.63264	-2.05425	0.070837	0.535329	0.393168	1.114156	0.393451	1.50446	-0.42475	0.693826
P08670_S10_1_1_N	P08670	VIM	0.198745	-0.49295	-0.80879	-0.21022	-0.30405	-1.79975	-1.54732	0.13841	-0.57461	-0.07147	-0.69213	-1.65161	2.118224
P08670_S214_1_1_	P08670	VIM	-0.73459	-0.39261	-0.7507	1.247196	-0.00544	-0.44833	-0.63968	0.721922	-0.3769	0.816884	-1.03701	-0.58281	-0.72926
P08670_S29_1_1_N	P08670	VIM	-0.3138	-1.21222	0.465355	0.508239	-1.61996	-1.56551	0.011142	-1.84194	0.202391	0.277711	-0.60988	-2.95664	-0.47611
P08670_S430_1_1_	P08670	VIM	-0.90188	-1.41522	-0.41299	0.747997	-0.4485	-2.0281	-1.16787	0.182073	-0.66703	1.504073	0.144915	-0.92604	-0.45057
P08670_S5_1_1_N(	P08670	VIM	0.63759	-0.94173	1.000269	0.472918	-1.10988	-0.37861	0.145988	0.004586	0.012905	1.192731	-0.60921	-1.39046	0.514351
P08670_S51_1_1_N	P08670	VIM	-0.12349	-0.90394	-0.49901	1.458274	-0.97344	-1.1027	-0.70643	0.348023	0.024612	1.255078	-1.14001	-0.58754	0.398806

P08670_S55_1_1_N	P08670	VIM	-0.29774	-0.66537	-0.64807	0.905099	-0.18497	-0.36443	0.257007	0.346986	-0.94788	-0.20706	-1.00101	-0.4355	0.002064
P08670_S7_1_1_N(	P08670	VIM	0.800607	-0.67058	-0.05535	0.087155	-1.59911	-1.97916	-0.29045	-0.23278	-0.57639	0.485001	-0.96232	-2.15025	0.174781
P08670_T436_1_1_	P08670	VIM	0.311744	0.650274	-1.01477	1.627765	-1.56334	-1.44418	1.892181	-0.05045	1.268805	2.445053	0.888023	-0.21873	-0.13719
P08727_S22_1_1_N	P08727	KRT19	-0.97822	-0.89328	-1.20547	-0.25485	0.323159	0.770765	0.735913	-0.21004	-0.7605	0.133933	-0.15236	-0.21361	0.629066
P08727_S35_1_1_N	P08727	KRT19	-1.14815	-0.89012	-0.03773	0.43319	-0.4207	-0.07189	0.290905	0.020743	-0.86931	0.674093	0.895511	-2.08896	0.590371
P08727_S46_1_1_N	P08727	KRT19	-0.39541	-0.98198	0.723222	-0.58345	-0.82501	-1.22385	-1.07112	-1.314	-0.86713	-0.66661	-1.21364	-2.24346	0.290776
P09496_S105_1_1_	P09496	CLTA	-1.00183	-1.07831	-0.2062	0.749997	-0.11143	-1.08428	-1.02707	0.336701	-0.37937	1.224301	0.461286	-0.3078	-0.33757
P09651_S6_1_1_N(	P09651	HNRNPA1	0.020433	-0.5952	-0.74611	0.137758	-0.01981	0.181125	-0.63176	-0.39144	-0.39751	0.540358	-0.40509	-0.82228	-0.97104
P10398_S582_1_1_	P10398	ARAF	-0.3114	-0.48082	-0.59368	-0.60618	-0.04977	0.60087	0.132358	0.284955	1.058836	-0.02466	0.223277	-0.29555	0.200353
P10451_S303_1_1_	P10451	SPP1	0.380922	0.048843	-3.87963	0.688316	-0.98579	1.259143	-0.00066	1.831333	0.381514	0.797532	-1.41215	0.8318	-0.19092
P10644_S83_1_1_N	P10644	PRKAR1A	-0.94389	0.428973	-0.63236	1.664235	-0.10426	0.379972	-0.70919	-0.10317	-0.34703	0.135973	-0.89499	-0.3555	-0.58768
P11274_S354_1_1_	P11274	BCR	-0.16136	-0.11242	-0.19723	-0.3794	-0.36127	-0.30792	-0.29678	-0.21224	0.116016	-0.08265	-0.05477	-0.24995	0.757915
P11274_S356_1_1_	P11274	BCR	0.02566	-0.19971	0.156821	-0.42283	-1.08756	-0.2477	0.025082	0.10301	-1.39498	-0.0372	-0.26661	-0.33996	-1.01097
P11388_S1106_1_1	P11388	TOP2A	0.271702	-0.38039	-0.4317	1.372529	-0.15527	0.046583	-0.74623	0.641793	0.525281	1.063233	0.265412	-0.29309	0.024265
P11388_S1247_1_1	P11388	TOP2A	0.211762	0.747933	-0.17934	0.776252	0.11704	-0.69006	-1.96076	0.025177	-0.5795	1.52981	0.242522	-1.19006	-0.11699
P11388_S1525_1_1	P11388	TOP2A	-0.34315	-0.55517	-0.71157	-0.13654	-2.70247	-0.00963	0.536743	-0.35356	0.140471	-0.02152	0.593773	0.240239	-0.40317
P12111_S1783_1_1	P12111	COL6A3	-1.78068	-0.87854	-3.05214	0.940713	-0.42353	-1.72335	-2.02897	0.046686	0.084814	0.538407	-0.76069	-0.61396	-1.06562
P12270_S2155_1_1	P12270	TPR	0.862876	-0.92442	0.090778	0.151781	-2.06614	0.133966	1.562586	-0.59973	0.416021	0.483284	-0.88768	-0.28769	-0.71633
P12694_S337_1_1_	P12694	BCKDHA	1.470417	1.147686	-0.60984	1.643374	0.702946	-1.4201	-2.39909	1.520368	-0.128	0.894038	-0.5652	-1.34057	-0.84056
P12694_S347_1_1_	P12694	BCKDHA	1.011808	0.075622	0.524312	-0.98834	0.199324	0.556132	-0.93108	0.108359	0.10522	0.269368	-0.58058	-0.76435	-1.28803
P12931_T420_1_0_	P12931	SRC	0.531177	-0.26488	-1.24506	-0.38528	-1.94289	1.379464	1.19187	-0.80977	-0.62949	0.067375	-0.08846	-1.28056	0.6565
P12931_Y419_1_1_	P12931	SRC	0.88227	-0.08241	-0.72721	0.288289	-1.59496	1.666573	2.3929	-0.12851	-0.04596	-0.85495	1.006203	-0.47542	0.510514
P13498_T147_1_1_	P13498	CYBA	-0.00931	0.434192	0.756563	-0.30789	-0.70839	-2.36387	0.830504	-0.77411	0.981505	0.130366	-0.19844	-0.38429	1.237602
P13611_S2941_1_1	P13611	VCAN	-1.2934	-0.34821	-2.09257	1.179685	0.248704	-1.2942	-0.72133	0.824036	-0.36133	0.673327	1.058202	-0.4282	-1.50264
P13639_T57;T59_2	P13639	EEF2	0.908537	-2.61688	0.541078	-1.56659	-2.58666	-1.84205	0.262253	-0.40915	-2.60775	-0.95556	-1.46646	-1.65508	-0.78109
P13796_S257_1_1_	P13796	LCP1	-0.55879	-0.91033	-1.19399	2.146532	0.305384	-0.13902	-0.0521	0.543647	-0.69416	1.589114	-0.59356	-0.48809	-0.13131
P13984_S248_1_1_	P13984	GTF2F2	-0.16557	-0.23261	-1.0468	0.149741	-0.63098	0.652204	0.690306	-0.0973	-0.07957	0.350099	0.063273	0.278632	-0.00712
P14618_S37_1_1_N	P14618	PKM	1.232211	0.620794	0.127159	1.927891	0.052278	-0.10323	-0.57163	2.125654	-0.04264	1.543333	-0.01932	-0.62939	-0.75682
P14866_S52_1_1_N	P14866	HNRNPL	0.119846	-1.14235	-0.34466	0.040756	-0.35152	-0.25874	-0.85703	-1.73644	-0.52085	0.143809	0.111417	-0.57586	-0.0625
P15056_S446_1_1_	P15056	BRAF	-1.04772	-1.41319	-0.51565	0.164139	-0.23672	0.125455	-1.75902	-0.91937	0.348034	0.303388	-1.32909	-0.24373	-0.08806
P15056_S729_1_1_	P15056	BRAF	-0.35822	-0.29353	-0.59384	-0.2409	0.374829	0.620538	0.192423	-0.58589	-0.03916	0.84231	-0.20304	0.34435	0.900388
P15408_S200_1_1_	P15408	FOSL2	-0.62818	0.633217	0.507041	1.757782	-0.19115	0.059084	0.082958	-0.35544	-0.58909	0.625524	0.18633	-0.40258	0.267198
P15924_S2209_1_1	P15924	DSP	0.372483	-0.18291	-0.19973	-0.15694	-0.67142	-0.07858	0.492449	-0.75783	0.282484	-0.3186	-1.61707	0.390162	0.823682
P15924_S2589_1_1	P15924	DSP	0.797359	-1.59322	-0.12769	-1.2981	-3.19192	-0.97821	-0.91491	-0.76612	-1.82213	-0.49034	-2.00821	-2.43913	-1.35192
P16070_S706_1_1_	P16070	CD44	0.087011	0.431355	-0.95135	0.359774	1.051976	1.202312	0.154584	0.524504	0.388889	1.177024	-0.06396	-0.24417	-0.2084
P16104_S140_1_1_	P16104	H2AFX	-2.13586	1.620555	-1.38909	0.907399	-0.06041	-0.42403	-1.412	3.948616	0.052975	2.169445	-0.89928	-0.43306	0.711518
P16104_T137;S140	P16104	H2AFX	-1.40487	1.969542	-1.41101	2.709964	-1.07381	0.624768	-1.23193	3.661979	0.775397	2.059985	-0.38368	-1.41489	-0.8578
P16144_T1530_1_1	P16144	ITGB4	-0.03279	-0.88448	0.015137	0.5013	-0.02068	1.158219	-0.76754	-0.10247	-1.2351	0.847438	-0.4703	-0.14854	0.292429
P16150_S336_1_1_	P16150	SPN	1.187738	-0.15571	-0.78863	0.040185	-1.8577	-1.65544	0.054218	-1.07364	0.531932	0.197959	-0.05537	0.131751	0.701651
P16150_S355_1_1_	P16150	SPN	-0.01024	-0.37429	-1.8319	-0.01401	-2.14098	0.074143	0.052836	-0.67398	0.507599	0.670213	0.522443	-0.541	0.469105
P16333_S85_1_1_N	P16333	NCK1	0.051018	-0.19312	0.122418	0.408375	-0.56024	-0.51775	0.614316	-1.23628	-0.18317	-0.0047	-0.15605	-0.53691	-1.54132
P16615_S663_1_1_	P16615	ATP2A2	0.518053	-0.23204	-0.34868	-0.29115	-0.36444	-0.32594	0.011648	0.236547	-1.10148	-0.64098	0.240253	-1.04603	-0.36122
P16949_S38_1_1_N	P16949	STMN1	0.316313	-1.04783	1.100297	0.279835	-0.8945	0.119027	-0.40976	1.226194	0.226972	0.043985	-0.29773	-0.84933	-1.07093
P17029_S13_1_1_N	P17029	ZKSCAN1	0.140559	-0.05271	-1.39207	-0.61303	-0.59441	0.372264	0.141136	-0.24054	0.199234	0.317697	1.024289	1.663928	-0.89375
P17152_S17_1_1_N	P17152	TMEM11	-0.13118	-0.16094	-0.41895	0.061887	0.878065	-2.20943	-1.61541	0.874938	0.311666	0.466127	-0.80924	0.41565	1.278282
P17535_S100_1_1_	P17535	JUND	-0.57646	-0.07604	0.151655	1.458951	-0.80392	-0.93209	-0.38495	-0.03391	1.523531	0.823805	0.264408	1.112508	-0.62596
P17612_S339_1_1_	P17612	PRKACA	0.199943	-0.22649	-0.41575	1.027545	0.426628	0.200609	0.103335	0.858407	1.096294	1.089888	0.081754	-0.93931	-0.3665
P17812_S575_1_1_	P17812	CTPS1	0.74995	-0.00915	-1.36485	0.938411	0.000639	0.197916	0.772043	0.506386	0.156888	0.895298	-0.45508	-0.38053	-0.48075
P18031_S378_1_1_	P18031	PTPN1	0.802171	-0.12365	-0.32803	-0.78021	-0.95802	-2.53448	-1.22479	-0.13506	-0.03963	-1.00382	-0.7497	-0.552	1.061808
P18206_S290_1_1_	P18206	VCL	-0.88348	-0.27717	-1.22094	0.732636	-1.05528	-0.52312	-0.2835	0.107519	0.828567	1.059275	0.525142	0.835671	-1.12425
P18206_S346_1_1_	P18206	VCL	-0.23815	-0.15624	-0.41017	0.545218	-0.9782	-0.90013	-0.41391	-0.05713	-0.00167	0.512378	0.101324	0.498477	0.379849
P18206_S721_1_1_	P18206	VCL	-0.44739	0.840072	-0.0568	0.357907	0.37767	0.090922	-0.77035	1.203886	0.681447	-0.56219	-1.49623	-0.65989	-2.37578
P18206_T604_1_1_	P18206	VCL	-0.80923	-0.94178	-0.13312	2.117924	0.187288	0.086983	0.57148	0.687284	-0.13495	0.810596	0.303823	-0.26872	0.4857
P18583_S1697_1_1	P18583	SON	0.380624	-0.23963	-0.33817	0.13743	-3.04328	0.399888	-0.59849	-0.16302	0.29613	0.144707	0.613097	0.672779	-1.56934
P18583_S283_1_1_	P18583	SON	-0.81599	0.119304	-0.69971	0.881519	0.245622	0.13033	-0.81313	1.557091	0.54005	1.069935	-0.68214	-0.41542	0.422206
P18583_T282_1_1_	P18583	SON	-1.85517	-1.97386	-0.49666	2.131826	0.738856	0.414164	-0.39284	1.230573	0.031995	1.830253	-0.19137	0.029825	-0.22158
P18615_S181_1_1_	P18615	NELFE	-0.98639	0.596436	-0.74434	0.522989	0.299611	0.540394	0.699393	0.255322	0.406773	1.260598	-0.08244	-0.65365	0.47425
P18615_S51_1_1_Y	P18615	NELFE	0.150837	-0.46616	-0.03997	1.07095	-1.84252	-2.24062	-1.69523	-0.38758	0.322349	0.403499	0.696714	-2.07632	-1.79269
P18754_S11_1_1_N	P18754	RCC1	0.024392	-0.36088	-0.11739	0.100004	0.004931	-0.19739	-0.24428	1.122087	-0.86568	0.509111	-0.15261	-0.24604	0.080341
P18858_S141_1_1_	P18858	LIG1	-0.78867	-0.86513	-0.61611	0.186926	-1.33176	0.049118	-0.06996	-1.41642	0.205778	0.217914	0.792819	-0.13227	-0.6442
P18858_S66_1_1_N	P18858	LIG1	0.406322	-0.21991	-1.10653	0.461572	-0.68769	1.104788	1.093484	-0.20889	-0.30399	0.800449	1.53475	-0.29917	-1.49997
P18858_S76_1_1_N	P18858	LIG1	-1.18408	-0.40415	0.252987	0.041324	-1.27615	-0.3393	-0.6474	-0.81933	0.424323	0.472037	-0.36253	0.782765	0.545798
P18858_T183_1_1_	P18858	LIG1	-0.03817	0.019847	0.291436	0.806376	-0.08428	0.364029	-0.40101	0.568576	-0.5672	-0.87504	-0.31225	-0.22286	0.264712
P19174_S525_1_0_	P19174	PLCG1	0.147727	-0.36489	0.106221	0.787004	0.638963	0.101496	0.181076	0.640576	0.040842	0.728242	-0.76094	-1.21411	-0.23811
P19338_S580_1_1_	P19338	NCL	1.179387	3.394606	-2.06012	0.855171	0.329782	1.314289	-1.62539	0.909234	-0.38609	0.380025	-1.39522	-0.75768	-1.17473
P19338_S67_1_1_N	P19338	NCL	-2.22425	-0.74276	0.030789	-0.62222	-0.75043	0.26491	0.106332	-0.36991	0.473816	-0.13024	-0.31645	0.101158	0.848069
P19634_S703_1_1_	P19634	SLC9A1	1.466285	0.970424	0.137675	-0.07299	-1.15525	-0.24949	0.222801	-0.20375	0.244199	-0.37532	0.078446	-0.24393	-1.06197
P19823_S60_1_1_Y	P19823	ITIH2	-0.70165	-0.76209	-0.8852	1.466873	0.083454	0.221419	-0.83447	1.1916	-1.04075	1.831118	0.057375	-0.16486	-0.19104
P20020_S1193_1_1	P20020	ATP2B1	0.421921	0.00411	-0.42516	-0.42053	-1.43583	-0.1242	-0.06831	-0.36326	-1.5469	-0.50484	0.13603	-0.85666	-1.01169
P20700_S23_1_1_N	P20700	LMNB1	-0.38691	-0.1752	-1.29852	-1.32356	0.686312	-0.4119	-0.7335	0.184359	-0.55297	0.34004	-0.07743	-0.74964	0.372005
P20700_S375_1_1_	P20700	LMNB1	0.103034	-0.81608	-0.07946	0.561663	-0.50208	0.010237	-0.20735	1.133431	0.495046	0.151621	-0.88548	-0.13463	-0.03107
P20700_S393_1_1_	P20700	LMNB1	-0.42065	0.197794	-0.38615	-0.03476	-0.02896	-0.44648	0.799923	-0.19462	-0.57319	-0.52704	-1.38121	-0.71712	0.350865
P21127_S115_1_1_	P21127	CDK11B	0.026211	0.00825	-0.42881	-0.6002	-0.34507	-2.24344	-0.68202	-0.954	-1.7418	-0.02605	0.02868	-0.87016	-0.10876
P21127_S283_1_1_	P21127	CDK11B	0.380908	0.340285	-0.62131	0.491813	0.363046	1.342832	-0.07536	1.238694	0.039211	0.818061	-0.59948	-0.30678	-0.16585
P21127_S589_1_1_	P21127	CDK11B	-0.35309	-0.21628	-0.78322	1.367996	0.041632	-0.01921	0.05422	1.185177	0.136818	0.813889	0.191487	-0.30577	-0.36292
P21127_S752_1_1_	P21127	CDK11B	-0.05819	-0.3579	-0.7195	-0.13424	-0.15928	0.427688	0.330241	-0.2186	-0.2705	-0.18488	-0.1666	-0.16248	-0.96484
P21127_T751_1_0_	P21127	CDK11B	-0.11377	-0.76917	-0.14235	0.236531	0.28127	0.490041	0.12683	-0.1301	0.648616	0.097384	-0.95861	-0.41758	-0.78347
P21333_S1630_1_1	P21333	FLNA	-1.46034	-1.77377	-2.41419	0.502784	-0.30472	-0.655	-0.35341	0.304191	-0.09838	0.485683	0.690793	-0.39348	-0.55581
P21730_S327_1_1_	P21730	C5AR1	-0.53969	-0.94301	-0.40778	2.957276	-0.15668	-1.1995	-0.83221	2.875941	-0.50715	2.73523	0.563415	0.279784	-1.08392
P22087_S124_1_1_	P22087	FBL	-0.53966	-0.08472	1.309167	0.263872	-0.61486	2.072336	-0.74436	1.276479	-0.65212	0.684607	-2.28376	0.544608	0.259101
P22626_S212_1_1_	P22626	HNRNPA2	-0.23177	0.411413	-0.67036	1.252666	-0.86304	-0.89837	-0.52024	-0.83189	1.508467	-0.28488	0.551909	-0.30923	-0.7635
P22694_S339_1_1_	P22694	PRKACB	-0.91693	-0.02384	-0.25343	0.849861	0.390717	0.273037	-0.06904	0.378675	0.029722	0.781041	-0.69562	-0.68127	-0.54426
P23142_S147_1_1_	P23142	FBLN1	-0.61765	0.632703	-1.51833	1.134542	0.869005	-0.78913	-0.87664	1.30226	1.035039	0.814364	-0.00983	-0.27109	0.659118
P23193_S100_1_1_	P23193	TCEA1	-0.5216	-0.26628	-0.80444	0.413072	-0.58648	-0.03974	0.036681	0.853545	0.82056	0.837864	0.38572	-0.90918	-0.34814
P23396_T221_1_1_	P23396	RPS3	-0.47963	-0.49248	0.142528	-0.18236	-0.16355	-0.05328	-0.4117	0.703359	-0.02985	-0.3778	-0.8073	-0.38323	-0.4662
P23443_S447_1_1_	P23443	RPS6KB1	0.329593	-0.18555	-0.69996	-0.38816	-1.21533	-0.17833	0.45994	-0.98389	-0.34191	0.054176	-0.13224	0.1536	0.181396
P23497_S157_1_1_	P23497	SP100	0.126584	-0.19565	-1.05823	1.25094	-0.3785	0.296447	0.110793	0.562888	0.093011	0.501756	0.056173	-0.22031	-0.19673
P23588_S497_1_1_	P23588	EIF4B	0.494975	1.57085	0.026267	0.297329	-0.16941	-0.05101	-0.83081	0.107072	0.323572	1.022715	-0.29702	-0.52606	-0.36517
P23588_S504_1_1_	P23588	EIF4B	-1.18202	-1.39332	1.205401	1.208257	0.000552	0.151112	-0.46593	-0.23575	0.304456	1.016698	-1.24619	-0.08068	-0.51283
P24534_S106_1_1_	P24534	EEF1B2	0.212454	-0.28418	-0.42571	0.650509	0.193389	-0.41917	-0.34905	-0.17412	-0.01803	0.731438	-0.01795	-0.45491	0.037067
P24534_T93_1_0_N	P24534	EEF1B2	-0.34275	-0.14212	-0.24517	0.824177	0.148276	-0.16908	-0.27042	0.059358	-0.48278	0.982999	0.132855	-0.55045	0.026844
P24928_S1861_1_1	P24928	POLR2A	-0.17146	-0.57152	-0.18661	-0.16044	0.479207	-1.10228	-0.87171	0.207608	-0.59654	-0.09566	-0.4631	-1.84927	-0.13997
P25440_T629_1_0_	P25440	BRD2	-0.23809	-0.51331	0.216001	0.342063	-1.18661	-0.4288	-0.48069	0.560565	-0.50095	0.86848	0.85445	-0.33445	0.790884

P25788_S250_1_1_	P25788	PSMA3	0.281631	0.71161	-1.55513	0.450353	-0.57514	0.076196	0.035219	-0.07814	0.321959	0.336789	0.394298	0.129151	0.942763
P25788_S250_1_1_	P25788	PSMA3	-0.26282	-0.03842	-0.65385	1.109518	-0.13725	0.069763	-0.34782	0.172438	0.091948	0.867755	-0.12847	-0.38471	-0.31619
P26232_S654_1_1_	P26232	CTNNA2	1.995452	1.595762	0.444696	-0.22725	-1.4404	0.541798	2.129522	0.932453	-0.15055	-0.18016	2.1592	-0.30003	-1.69476
P27708_S1859_1_1	P27708	CAD	-0.9042	-0.26982	-0.92818	0.573426	-0.29864	-1.23568	-0.04803	-0.09773	-0.07607	-0.28284	-2.002	-1.76121	-0.58103
P27816_S280_1_1_	P27816	MAP4	-0.84811	-0.54908	-0.31711	1.247626	0.550609	-0.11754	-0.11086	-0.70998	0.309982	0.706897	0.125994	-0.62182	0.547258
P27816_S507_1_1_	P27816	MAP4	0.009181	-0.14561	-0.49835	1.08958	0.348026	-0.44871	-0.85371	0.478188	0.535612	0.874449	-1.02282	-0.64582	-0.21673
P27816_S941_1_1_	P27816	MAP4	-0.01248	0.259379	-0.19408	1.024315	-0.50626	0.510436	0.957591	-0.45348	0.169068	0.664474	0.071476	0.134577	0.075625
P27816_T521_1_1_	P27816	MAP4	0.231592	0.138194	-0.07099	0.802383	0.974399	0.265197	-0.86753	1.037605	0.589651	1.005446	0.196618	-0.42114	-0.63102
P27824_S564_1_1_	P27824	CANX	-2.8204	-1.09228	-1.79443	0.829095	-1.56647	-0.41303	-0.18847	-1.31499	-1.53767	0.080686	-0.7344	-0.04577	0.9206
P27824_S583_1_1_	P27824	CANX	-0.18601	-0.38697	-0.74763	-0.7032	-0.3654	0.321283	0.223813	-1.68983	-0.41827	-1.25785	0.235091	-0.43679	0.137294
P28066_S56_1_1_Y	P28066	PSMA5	-0.78958	0.337827	1.18685	2.384082	-0.94492	-0.35157	-0.03117	2.915847	-0.67525	1.592771	-0.10934	-1.1301	-0.30771
P28290_S668_1_1_	P28290	SSFA2	-0.13731	-0.40309	0.050445	-0.29691	-0.18262	-1.31672	-0.23827	0.417865	-0.5021	0.083565	-0.38033	0.09313	-0.85664
P28290_S92_1_1_N	P28290	SSFA2	-0.38552	0.078044	-0.52348	1.26527	-0.76986	-0.60045	-1.74377	-0.15197	0.102168	0.846081	-0.38561	-0.8078	-0.72306
P29323_S575_1_1_	P29323	EPHB2	0.12685	-0.36065	0.272272	-0.50405	0.44826	-0.83585	-0.57975	0.369864	-0.3622	-1.11347	-1.54443	-0.2498	-0.28399
P29590_S36_1_1_N	P29590	PML	-1.60168	0.465558	-0.15057	1.110182	0.713366	0.430961	0.134648	0.259684	-0.09467	-0.2141	0.219397	-0.57699	0.424977
P29590_S403_1_1_	P29590	PML	-0.38773	-0.4549	-0.76493	2.075858	0.445173	0.218385	-0.55403	0.890706	0.059742	1.093396	-0.36473	-0.28686	-0.471
P29590_S505_1_1_	P29590	PML	-1.27499	-0.78346	-0.91296	1.015305	0.002	0.315213	-0.29808	0.009935	-0.19246	0.327694	-0.16141	0.113651	0.061301
P29590_T867_1_1_	P29590	PML	1.157765	-1.3675	0.080186	-0.38099	-2.09616	-0.93918	0.17983	-0.76962	0.294358	0.214861	-0.28413	-1.36864	-0.932
P29692_S133_1_1_	P29692	EEF1D	-0.69769	-1.06316	-1.22832	0.499987	-0.35466	0.566953	0.589855	0.12365	-0.17757	0.345538	1.23088	-0.12031	0.029409
P29692_S162_1_1_	P29692	EEF1D	-0.13914	-0.35343	-0.38428	0.880842	0.174372	-0.02039	0.056297	0.671335	-0.13574	0.514611	0.344735	-0.35787	0.043823
P30273_S69_1_1_N	P30273	FCER1G	1.629962	0.065066	-0.08656	0.089658	-1.03439	-1.90035	0.018939	-1.28936	0.254342	0.864782	-0.64711	0.562413	1.496956
P30273_T75_1_1_N	P30273	FCER1G	1.138147	-1.28389	0.306751	-0.41826	-0.732	-1.19081	0.704207	-2.83255	0.016063	-0.051	-0.34931	0.171567	0.703643
P30273_T78_1_1_N	P30273	FCER1G	1.558853	0.048698	0.880077	1.367253	-1.19148	-0.72916	0.537273	-0.3876	1.236215	0.071151	0.127604	0.721144	-0.2932
P30419_S47_1_1_N	P30419	NMT1	0.673294	-0.3098	0.100007	0.104442	-1.70557	-0.29502	-0.31133	-0.2702	0.169296	0.434687	0.183753	-0.23698	-0.32871
P31751_T451_1_1_	P31751	AKT2	0.536061	0.630087	-0.41689	-0.00204	-0.16688	0.436073	-0.44505	1.02644	-0.60316	0.293159	-0.47488	-1.59144	-0.48133
P31943_S104_1_1_	P31943	HNRNPH1	-0.16947	-0.15527	-0.53314	1.33401	-0.15074	-0.46982	-0.30595	0.552626	-0.3096	1.26834	-1.16271	-0.51264	-0.31822
P33241_S252_1_1_	P33241	LSP1	-0.50328	-0.09263	-0.94264	0.628354	0.102956	0.000131	-0.73835	0.424286	0.259976	0.758854	-0.20108	-0.26946	0.131343
P35221_S641_1_1_	P35221	CTNNA1	-0.16263	0.098637	-0.92632	0.910815	0.377815	0.110055	0.145682	0.966157	0.208444	-0.11975	0.00047	0.078267	0.119577
P35221_S655_1_1_	P35221	CTNNA1	1.026795	1.430753	0.168603	-0.31765	-1.73701	0.111378	0.561783	1.18531	0.808017	0.115849	0.262948	-0.57436	-1.56996
P35222_S191_1_1_	P35222	CTNNB1	0.529964	-0.38633	0.009858	0.523304	0.074452	0.034662	-1.641	0.684402	-0.36132	0.836731	-0.45456	0.023583	-0.48394
P35579_S1943_1_1	P35579	MYH9	-0.41081	-0.11016	-0.41566	0.256311	0.181439	0.198501	-0.08303	-0.29094	-0.00648	0.164643	-0.45372	-0.30168	0.0731
P35580_S1956_1_1	P35580	MYH10	0.220761	0.492586	0.027182	0.889184	0.519306	-1.30992	-1.23294	1.046426	-1.5439	-0.21061	-0.25137	-0.61507	-1.31352
P35611_S358_1_1_	P35611	ADD1	-0.66987	-0.58334	-0.37894	2.365161	0.345204	-0.49368	-0.83641	1.535841	-0.05916	1.241104	-0.56729	-0.32663	-0.49683
P35611_S586_1_1_	P35611	ADD1	0.923433	0.488413	-0.51296	0.78214	0.846872	-0.61389	-2.47055	0.582478	-1.64273	1.24809	-0.2496	-0.31925	-0.64675
P35749_S8_1_1_NO	P35749	MYH11	-2.28431	0.056046	-2.52865	-0.37194	-2.5861	-0.75548	0.355885	-0.41968	-0.82848	-1.26816	-0.41039	0.134035	0.838457
P35908_S62_1_1_N	P35908	KRT2	-1.53034	-0.51221	-1.50087	1.360126	-0.17305	0.179172	-0.74962	0.286604	-1.30021	0.776238	-0.38241	-1.25356	0.217908
P36507_T394_1_1_	P36507	MAP2K2	0.146941	-0.09354	-0.49569	-0.73565	-0.77372	-0.43548	-0.27401	-0.44881	0.262085	0.498693	0.398401	-0.74709	-0.58516
P37837_S237_1_1_	P37837	TALDO1	-0.29221	-1.60348	-1.35845	1.30768	-1.0532	-1.17497	-1.38806	0.671027	-0.73255	0.791219	-0.92576	-1.87985	-1.28469
P38159_S208_1_1_	P38159	RBMX	0.092046	0.436979	-1.71668	0.935158	-0.30823	0.565721	0.062821	-0.46679	0.261186	0.182211	0.167887	-0.08079	-0.74092
P38159_S352_1_1_	P38159	RBMX	-0.43507	-0.71717	-0.7972	1.544571	-1.42665	-0.2614	0.006125	-0.18524	0.738364	2.042297	0.638916	0.467392	-0.62733
P39687_T15_1_1_N	P39687	ANP32A	0.634676	-0.18978	-0.36879	0.837493	0.688855	-0.41159	-0.59902	-0.22277	-0.19315	0.978881	0.347269	-0.39283	0.414216
P39880_S1496_1_1	P39880	CUX1	0.04675	0.658146	-0.70626	0.288517	-2.01046	-0.33371	0.348709	-0.23075	0.312063	0.931041	0.590963	-0.54697	1.003037
P40818_S718_1_1_	P40818	USP8	-0.37687	-1.38867	-0.41566	-0.25634	-0.55316	0.218087	-0.37852	-0.22986	-0.12854	-0.1017	-0.12814	0.209447	-0.16807
P41227_S205_1_1_	P41227	NAA10	-0.3666	-1.01796	-0.37682	0.632269	0.181282	0.141728	-0.20527	-0.5704	-0.76952	0.06802	-0.93493	-0.38852	-0.93547
P42166_S67_1_1_N	P42166	TMPO	-0.5761	-0.3181	-0.55442	0.90198	0.296558	0.341488	-0.49554	-1.19024	0.73336	0.505958	2.026902	-0.3911	-0.78505
P42166_T160_1_1_	P42166	TMPO	-0.18032	-1.19333	-0.86461	-0.11937	-1.22184	0.043447	0.58864	-1.6847	0.215848	1.025678	0.322496	-1.47623	-1.20604
P42166_T74_1_1_N	P42166	TMPO	-0.36211	-1.3831	-1.01759	-0.5392	-1.09905	0.363222	0.197953	-1.47711	-0.24212	-0.3753	0.507051	-1.78724	-0.95065
P42167_S385_1_1_	P42167	TMPO	-0.42672	0.711377	-0.26987	1.047566	-0.39148	-1.62747	-0.17451	-0.0885	1.101702	0.37414	1.50666	-1.35715	-0.47032
P42167_S67_1_1_N	P42167	TMPO	-0.1834	0.212724	-0.56855	1.638847	1.265513	0.314059	-0.50043	-0.24683	0.308671	0.646374	1.044977	-0.27078	-0.00181
P42167_T160_1_1_	P42167	TMPO	0.212387	-0.66251	-0.6935	0.617498	-0.25289	0.016018	0.58375	-0.7413	-0.20884	1.166094	-0.65943	-0.15511	-0.4228
P42167_T74_1_1_N	P42167	TMPO	0.030602	-0.85228	-0.84647	0.197668	-0.13009	0.335793	0.193063	-0.53371	-0.66681	-0.23489	-0.47487	-0.46612	-0.16741
P42224_S727_1_1_	P42224	STAT1	0.600136	-0.31426	1.374341	0.305024	-0.63957	-0.63329	0.060894	0.051915	0.966764	-0.35858	-0.00753	-0.53306	-1.01691
P42331_S379_1_1_	P42331	ARHGAP25	0.589289	-0.27302	-0.31909	0.545927	-0.21815	-0.78831	0.270307	0.915551	0.133804	0.19406	-1.57781	0.030959	1.347565
P43243_S188_1_1_	P43243	MATR3	-0.81174	-0.98382	-0.56535	0.454114	-0.75962	-0.59067	0.086207	0.083854	-0.82092	0.552029	-0.27875	-0.06771	0.163001
P43243_S598_1_1_	P43243	MATR3	0.208004	0.023491	-0.25227	1.639015	0.389706	0.206141	-0.2625	1.43716	0.146361	0.841833	-0.24645	-0.33544	-0.16549
P45974_T623_1_1_	P45974	USP5	-0.29942	-0.30442	0.950549	0.154853	-0.4326	-0.96676	-0.60779	0.811528	0.697766	0.440142	-0.54003	-1.22474	-1.43192
P46100_S1348;S13	P46100	ATRX	-0.29191	0.71687	-0.65327	0.420433	-0.69301	-0.03305	0.318486	-0.41062	0.268631	-0.6888	0.55178	0.162747	0.02394
P46100_S1348_1_1	P46100	ATRX	-0.44091	0.053342	-0.83463	-0.04414	1.80011	-1.30951	-1.43041	0.550613	-1.16711	0.364893	-0.47166	-0.05467	0.181731
P46821_S1427_1_1	P46821	MAP1B	-0.18552	-0.39064	-0.25588	1.397404	-0.40518	0.585019	-0.46615	0.203415	0.351368	0.514746	0.609753	1.787745	-0.02591
P46821_S1852_1_1	P46821	MAP1B	0.009172	-0.26871	0.289662	-1.40091	-0.92074	-0.90404	0.430825	1.666237	-1.14575	0.580769	-1.84602	-2.87907	-0.15256
P46821_S832_1_1_	P46821	MAP1B	0.146612	-0.91345	-0.27922	0.926016	1.458223	-0.8542	-1.42192	-1.18162	-1.54743	0.372897	-0.2677	-1.64086	-1.24041
P46821_T1788_1_1	P46821	MAP1B	0.356892	0.052967	0.556219	-0.03484	-1.61532	-0.85073	-0.14803	-0.47991	-0.50591	-0.38641	-0.04953	-0.53171	-0.23492
P47712_S437_1_1_	P47712	PLA2G4A	-0.10383	0.007035	0.241765	1.120766	0.541482	0.347928	0.335294	0.414306	0.164882	-0.13071	-0.03248	-0.06022	-0.09513
P48681_S1016_1_1	P48681	NES	0.411261	0.219761	-0.23841	1.044742	-0.65099	0.521291	0.239011	0.536712	0.512133	0.928601	0.157851	-0.19886	0.040998
P48681_S471_1_1_	P48681	NES	0.233155	0.14331	-0.75057	0.947983	-0.6573	-0.09735	0.007698	-0.44205	0.35418	0.225704	0.900113	-0.11828	-0.15082
P48681_S680_1_1_	P48681	NES	0.233924	-0.11665	0.541959	1.096032	-0.77853	-0.86569	-0.05421	-0.28294	-0.85949	0.267343	-0.68252	-0.69045	0.02203
P48681_S768_1_1_	P48681	NES	-0.43161	-0.3692	-0.28623	0.505477	-0.29443	-0.04115	-0.36818	-0.6814	-0.00039	-0.27352	0.430841	-0.0862	0.276873
P49321_S421_1_1_	P49321	NASP	-1.3872	-0.8953	-1.30638	1.245038	0.385533	-0.24416	-1.46948	1.59745	-0.97398	0.9883	-0.17937	0.033881	1.69768
P49585_S315_1_1_	P49585	PCYT1A	0.017239	0.387869	-0.84066	-0.04853	-0.22005	1.0065	0.22105	0.509285	0.428007	0.833412	-0.04425	0.62886	-0.4837
P49736_S139_1_1_	P49736	MCM2	-0.12444	-0.39683	-0.16643	0.501181	-0.30033	0.310156	0.136181	-0.95751	-0.29106	0.014054	0.005141	0.071498	0.724065
P49736_S27_1_1_N	P49736	MCM2	-0.18476	0.217109	-0.19708	-0.54631	-0.70518	0.083268	-0.25253	-1.1313	-0.19118	0.000745	-0.0902	-0.31805	0.506578
P49736_S41_1_1_N	P49736	MCM2	0.879165	-0.13814	0.353001	-0.47987	0.171198	0.921151	-0.71288	-0.1196	0.010555	-0.3211	-0.36461	-0.40872	-0.43623
P49750_S561_1_1_	P49750	YLPM1	1.150582	-1.59304	0.482114	-0.49046	-2.20678	-1.38147	-1.19246	0.679537	-1.30324	-0.3464	-0.88613	-2.63568	-1.1124
P49756_S677_1_1_	P49756	RBM25	-0.17699	-0.46411	-0.68683	-0.16855	-1.57218	0.526998	0.741916	-0.75816	-0.25134	0.252221	0.863323	-2.09711	-1.55817
P49792_S2900_1_1	P49792	RANBP2	0.333404	0.561667	-0.04847	0.756599	-0.15242	-0.50805	0.421872	-0.26612	0.467773	0.373402	-0.70323	-0.35947	0.252661
P49792_T2450;S24	P49792	RANBP2	-0.75052	-1.49618	0.091934	-0.62027	-0.08877	0.455609	0.031849	-0.42844	1.104371	-0.19405	-0.26844	0.057036	0.159979
P49959_S649_1_1_	P49959	MRE11	-0.32741	-0.11597	0.889497	0.593986	-1.93308	-0.40829	-0.20115	0.499034	0.626963	1.547092	0.871675	-0.22049	-0.587
P50479_S112_1_1_	P50479	PDLIM4	0.09502	0.263524	0.510501	0.266194	0.46725	-0.83434	-0.53492	2.653356	-1.7324	0.13372	-0.30482	-0.07873	-0.27011
P50552_S305_1_1_	P50552	VASP	0.16441	-0.13317	-0.98462	-0.09287	-2.13169	-0.57334	1.09336	-1.06325	-0.43438	1.05547	-0.74955	-0.94635	1.567748
P51532_S1382_1_1	P51532	SMARCA4	0.450232	-0.01727	0.304931	-0.19222	-1.23575	-0.88161	-0.13494	-0.46543	-0.17976	-0.78429	0.617408	-0.85931	-0.74926
P51580_S14_1_1_N	P51580	TPMT	0.094718	-0.33283	-0.66307	-0.10562	-0.12402	-0.09949	-0.2941	0.361859	0.629739	-0.50703	-0.26118	-0.26981	0.328066
P51858_S165_1_1_	P51858	HDGF	0.066642	-0.23684	-0.65662	0.706988	-0.51407	0.300354	0.044061	0.326966	0.24988	0.805325	-0.04434	-0.4267	-0.18237
P51991_S358_1_1_	P51991	HNRNPA3	-0.099	-0.44489	-0.54618	-0.19804	-0.23768	0.689224	0.400433	-0.38078	0.090267	-0.23467	-0.46659	-0.07376	-0.13183
P52701_S65_1_1_N	P52701	MSH6	-0.95866	-0.79516	-0.84392	0.197248	-0.65326	0.079662	0.051747	0.191397	-0.3027	0.088679	0.035064	-0.61762	-0.15807
P52756_S621_1_0_	P52756	RBM5	0.80048	0.028036	-0.72876	1.401282	-0.0932	-0.4069	-0.19461	1.734327	0.464235	0.527238	0.203192	-0.63204	-0.21713
P52756_S624_1_1_	P52756	RBM5	-0.22253	-0.28188	-0.40039	1.511206	-0.55025	-0.22698	-0.06427	0.4578	0.279319	0.966486	1.450997	0.241588	-0.48788
P52948_S888_1_1_	P52948	NUP98	0.308151	0.378327	-0.40497	0.36577	-0.72248	0.323873	0.229139	0.214994	0.449338	0.634979	0.678276	-0.08942	-0.18198
P53396_S455_1_1_	P53396	ACLY	-0.06787	0.450618	-0.73535	0.232792	-0.6499	-0.10802	0.120863	-0.3616	0.121585	-0.04769	-0.51723	-0.7075	-0.01118
P53396_S481_1_1_	P53396	ACLY	-0.10688	-0.83789	0.28864	-0.62154	-0.08776	-0.09696	-0.9311	-0.13398	-0.2881	0.452021	1.626863	-0.26452	0.001327
P53621_S173_1_1_	P53621	COPA	-0.45972	-0.6444	-0.44953	1.19458	0.166472	-1.24022	-0.84873	2.135498	-0.626	1.2448	-0.3316	0.547869	0.402454
P54105_S102_1_1_	P54105	CLNS1A	0.357666	-0.18216	-0.24232	1.093888	0.641532	0.804071	0.397955	-0.0883	0.229536	-0.22876	0.227748	-0.17688	0.908068
P54259_S645_1_1_	P54259	ATN1	0.786944	-0.20077	-0.48031	0.181253	-0.01676	-0.1143	-0.28199	-0.67826	0.188665	0.302585	-0.07828	-0.4197	0.325142
P54727_S160_1_0_	P54727	RAD23B	-0.34163	-0.86775	0.461033	0.214824	-1.7185	-1.27694	-2.29636	-0.28154	-2.18895	-0.36567	-0.84682	-1.78953	-1.30688

P55081_S116_1_1_	P55081	MFAP1	-0.74844	-1.23378	-0.99124	0.968023	0.921096	-0.49058	-2.5805	0.904116	-1.21668	1.242563	-1.28411	-0.82856	-0.25658
P55196_S1083_1_1	P55196	AFDN	-0.66405	0.023026	-0.14058	0.201653	-0.41123	-1.19805	-0.48959	0.715241	0.165167	0.222275	0.167619	0.175477	0.494563
P55265_T601_1_1_	P55265	ADAR	0.215454	0.134805	0.964004	0.170632	-1.5238	-1.14715	-0.96922	-0.94045	-1.41295	-0.20748	0.446569	-1.26003	-1.10683
P55285_S786_1_1_	P55285	CDH6	0.083954	1.81614	-0.3262	1.138316	-0.1719	-0.24475	0.356991	1.314533	-0.41685	0.65353	0.335959	0.121911	0.15895
P55795_S104_1_1_	P55795	HNRNPH2	-0.24227	-0.03165	-0.56862	1.422647	0.048489	-0.39919	-0.82716	0.50234	-0.37838	0.957817	-0.35018	-0.22231	-0.16785
P58107_S2716_1_1	P58107	EPPK1	-0.17861	0.39554	-0.52643	-0.02282	-0.07626	0.017709	0.111982	0.476134	1.160943	0.272374	0.196545	-0.04547	-0.51574
P62805_S48_1_1_N	P62805	HIST1H4A	-1.34438	-0.28926	-1.38831	0.453583	-0.64591	-0.75657	-1.50175	0.176295	-0.9239	-0.1613	-0.53666	-3.04797	-1.88294
P62805_T72_1_1_N	P62805	HIST1H4A	-1.85787	-1.03229	-0.8495	0.969354	-0.33643	-0.52465	-1.86676	0.4454	-2.67106	0.829918	-0.5356	-1.78559	-2.14594
P62995_S22_1_1_N	P62995	TRA2B	0.742429	0.65649	-0.32014	-0.67494	0.210431	0.747086	0.244428	0.714354	0.331182	0.220523	-0.04737	-0.40516	-0.93583
P62995_S264;S266_	P62995	TRA2B	0.156421	-0.02122	-0.36177	-0.22276	-0.05698	0.276726	0.243049	-0.16127	0.022225	0.294937	0.113711	-0.18658	-0.08883
P62995_S266_1_1_	P62995	TRA2B	-0.81411	0.009509	-1.70002	0.946297	0.01976	0.735266	-1.29861	2.755342	-1.77336	1.318501	-0.90199	-0.83027	-0.84744
P67809_S165_1_1_	P67809	YBX1	0.78531	-0.37993	-0.85623	0.385547	-0.93055	0.134583	-0.03522	-1.6313	-0.34471	1.316192	-0.52583	-0.72614	-0.35832
P68366_S158_1_1_	P68366	TUBA4A	-2.10524	-0.73315	-1.32471	1.397663	0.341369	-1.74226	-0.99182	0.885934	-0.1402	1.364655	-1.36426	-2.17122	1.383593
P69905_S4_1_1_NC	P69905	HBA1	-0.03133	-0.22276	-0.76585	0.661562	-0.20826	0.248502	-1.51033	-0.04717	-0.45923	0.353901	-1.00502	-2.23799	-0.32858
P78332_S1025_1_1	P78332	RBM6	0.15105	-0.27771	-0.46541	0.779925	-0.50516	0.515665	0.150575	-0.10475	-0.75496	0.819607	0.146902	-0.48447	-0.58682
P78347_S103_1_1_	P78347	GTF2I	0.437079	-0.82894	0.50615	-0.90114	-2.45775	-2.4457	-1.31493	0.189524	-2.10583	-0.50131	-0.55006	-1.72533	-1.96792
P78347_S674_1_1_	P78347	GTF2I	0.475725	-0.27102	-0.25123	-0.25539	-1.34767	0.376873	-0.60002	-1.00757	-0.47403	-0.1208	-0.00336	-1.05302	-0.35672
P78527_S3205_1_1	P78527	PRKDC	-0.47232	-0.38895	-0.75634	1.141769	-0.78825	-0.61957	-1.01134	1.607726	-0.6555	1.530518	-0.58459	-0.21549	-0.13114
P78549_S71_1_1_N	P78549	NTHL1	1.008939	-0.38855	-1.45277	0.456897	-0.32094	0.340909	-0.09422	0.24771	-0.07268	0.462157	-0.19128	0.197879	-0.91014
P78559_S2022_1_1	P78559	MAP1A	-0.17238	-1.08931	-0.98408	0.430254	-0.67791	0.232411	0.226099	0.325369	0.481322	0.594172	0.21332	1.263795	-1.06817
P78559_S612_1_1_	P78559	MAP1A	0.048147	-1.57114	-0.67932	0.035296	0.044332	-0.14564	-0.64534	-1.11679	-0.22453	-1.28028	-1.9172	-0.18592	-0.18573
P82094_S344_1_1_	P82094	TMF1	-0.48809	-0.81105	-0.56599	0.885605	0.032151	0.932958	0.347805	0.75529	0.883656	1.457491	-0.48817	-0.31829	-0.12887
P84157_S127_1_1_	P84157	MXRA7	0.517359	0.610326	-0.07915	-0.0983	-0.20422	0.419644	0.020304	0.123097	-0.58593	0.149556	-0.42369	-1.46816	-0.10485
P84157_S140_1_1_	P84157	MXRA7	0.674011	-0.82109	-0.3709	0.077504	-1.08235	-1.25044	-0.88344	-0.09757	0.663272	0.0217	-0.68497	-1.17402	1.112592
P85037_S445_1_1_	P85037	FOXK1	-0.53704	-0.15358	-0.54362	-0.97507	-0.05322	0.018817	-0.44238	-0.2445	-0.25089	-0.20869	-0.77243	0.383998	-0.08049
P98175_S723_1_1_	P98175	RBM10	-0.19346	-0.35894	-0.17545	1.339756	0.282089	0.078778	-0.38802	0.408954	0.091794	0.682423	0.026206	-0.30956	0.10413
Q00059_S193_1_1_	Q00059	TFAM	-0.63858	0.359268	-1.02418	0.297173	1.209701	-0.11303	0.163017	1.089445	0.743339	0.581499	0.085337	-0.23715	-0.01812
Q00587_S192_1_1_	Q00587	CDC42EP1	-0.09431	-0.63611	-0.83369	0.687512	-0.91268	0.219429	-0.64284	-0.35786	-0.26068	0.514195	-0.99717	-0.05724	0.461189
Q00587_S350_1_1_	Q00587	CDC42EP1	-0.93569	-0.49222	-1.93383	0.192333	-0.99668	0.351915	-0.84372	0.357466	-0.12114	0.707685	-1.05289	-0.03412	-0.14929
Q00610_T394_1_1_	Q00610	CLTC	0.167296	0.345436	0.507975	0.129156	0.07922	-0.04319	0.573537	-0.0272	-0.21914	-0.22668	0.325755	-0.35895	-0.35046
Q01082_S2102_1_1	Q01082	SPTBN1	-0.48048	-0.2132	-0.45448	0.293652	0.33851	0.358563	-0.063	0.03045	-0.01249	0.382189	0.180753	-0.40118	-0.2844
Q01082_S2160_1_0	Q01082	SPTBN1	-1.47237	-1.43022	-0.64006	0.409608	-0.81574	-2.34071	-0.9793	0.981658	-1.86891	0.836886	-1.05087	-2.61604	-1.68226
Q01130_S191_1_1_	Q01130	SRSF2	-0.05912	-0.12692	-1.51926	3.03034	0.658282	0.006443	-2.3392	3.69261	-1.2211	1.356	-1.62381	-0.89918	-1.34875
Q01433_S168_1_1_	Q01433	AMPD2	0.210285	0.515131	-0.40338	1.205336	-1.03221	-0.11583	-0.7978	0.533345	0.749352	1.138368	-0.00112	-0.22507	-0.20352
Q01433_S190_1_1_	Q01433	AMPD2	-0.75003	-2.22812	-0.66833	1.682505	-0.82524	-1.19603	-2.19647	1.752844	-1.62647	1.679091	-1.3197	-1.79084	-0.67689
Q01484_S3909_1_1	Q01484	ANK2	0.476342	1.594034	-1.506	0.723516	-0.22256	-0.96987	-0.91493	0.184144	0.147773	-0.01775	0.300802	-0.37288	0.090994
Q01518_S34_1_1_N	Q01518	CAP1	-1.76441	-1.92206	0.136358	-0.26435	0.681356	0.536237	-0.13655	-0.96849	-0.05895	-1.06113	-0.25045	0.417681	1.698971
Q01804_S443_1_1_	Q01804	OTUD4	-0.22676	-0.36235	-0.09695	0.678033	-1.08713	0.285555	0.583362	0.37372	0.148228	0.683654	0.284636	1.412172	-0.21441
Q02040_S537_1_1_	Q02040	AKAP17A	-0.53386	0.032819	-0.44102	0.960918	0.175533	0.002998	0.063341	0.722772	0.603832	0.208986	0.029938	-0.72821	-0.70219
Q02818_S369_1_1_	Q02818	NUCB1	-0.21293	0.993636	-4.2676	1.362409	-0.15164	-0.54964	0.360548	1.819718	0.537718	1.834443	0.108612	-1.70366	-1.6677
Q02880_S1375_1_1	Q02880	TOP2B	0.09834	-0.19345	-0.58292	0.033219	-0.7528	0.398928	-0.06223	-0.86141	0.545331	0.099744	0.229608	0.150179	-0.02205
Q02880_S1466_1_1	Q02880	TOP2B	-0.39818	-0.02071	-1.87132	-0.41581	-0.33004	-0.36235	-0.43493	-1.23582	0.172152	0.143607	-0.11288	-0.16917	0.663252
Q02880_S1550;S15	Q02880	TOP2B	0.645541	0.280557	-0.46918	0.014679	-0.88933	-1.50605	-0.56916	-0.391	-0.37144	0.8198	0.100208	-0.9839	-0.85708
Q02880_S1550_1_1	Q02880	TOP2B	0.908188	-0.16343	0.800075	-0.4067	-1.13346	-1.24324	-0.98658	-0.32915	-0.28426	0.094556	-0.56578	-0.87775	-0.50891
Q02880_S1613_1_1	Q02880	TOP2B	-0.14508	-0.46903	-0.01846	-0.45887	-0.46069	-0.05013	-0.01138	-0.72366	-0.24028	0.605985	0.493554	-0.45947	0.116757
Q02952_S1331_1_1	Q02952	AKAP12	-0.53759	-0.68504	-0.40362	0.097023	0.437179	0.000676	-1.19946	0.42346	-0.75789	0.580252	-0.19349	-0.90742	-0.49587
Q02952_S371_1_1_	Q02952	AKAP12	-0.01243	0.20326	-0.67553	-0.20546	-0.15761	0.136871	-1.66233	-0.46219	0.138922	-0.868	-1.37778	-1.66327	-1.82949
Q03135_S37_1_1_Y	Q03135	CAV1	0.154965	0.566272	-0.59713	-0.41661	-0.01794	0.486285	-0.58418	-0.28312	-0.54702	-0.30911	0.178618	-0.55031	0.112808
Q03252_S37_1_1_N	Q03252	LMNB2	-1.21978	-0.46022	-1.05402	-0.35181	0.173184	-0.23395	0.001243	-0.22287	-0.49354	-0.62157	-0.97432	-1.21369	-0.11773
Q03252_S407_1_1_	Q03252	LMNB2	-0.77884	0.553995	0.935387	0.710663	1.152986	0.086557	1.759733	0.561069	-0.08764	0.620381	-0.28885	0.562835	1.524187
Q03252_T34_1_1_N	Q03252	LMNB2	-0.78542	-0.72733	-0.36153	-0.30723	-0.02666	-1.57265	-0.97141	-0.18752	-0.54847	0.206128	-0.83143	-1.71879	-0.9527
Q04637_S1187_1_1	Q04637	EIF4G1	-0.32858	0.031127	0.043635	0.596292	-1.59949	-0.5574	0.110704	-1.40915	1.468438	0.4348	-0.0602	-0.12498	-0.02449
Q04637_S1209_1_1	Q04637	EIF4G1	0.033095	0.041818	-0.35152	-0.25831	-0.43831	0.447958	-0.41985	0.361258	-1.01042	-0.12198	-0.33372	-0.95303	-0.82103
Q04637_S1231_1_1	Q04637	EIF4G1	0.219507	0.434694	0.510501	-0.81417	-0.93944	-0.57129	-1.06655	-0.86335	-0.95538	0.031483	-0.45769	-1.49818	-0.34284
Q04637_S1238_1_1	Q04637	EIF4G1	0.439456	-1.18377	-0.74095	-0.33966	-0.52985	1.933866	-0.38877	1.81029	-2.02032	-0.32771	-0.17669	-0.55746	-0.86684
Q05209_S435_1_1_	Q05209	PTPN12	0.495652	-0.13742	-0.10842	-0.14437	-2.12977	-0.43722	0.001366	-0.97268	0.271022	0.530791	0.323253	0.392266	0.268539
Q05519_S434_1_1_	Q05519	SRSF11	-0.06551	-0.29142	-0.37613	0.368454	0.333362	0.159848	-0.43862	0.601955	0.325325	-0.04694	0.142302	-0.21506	-0.0914
Q05519_S449_1_1_	Q05519	SRSF11	-0.36881	-0.63115	-0.42302	0.46486	0.790941	-0.55567	-1.83664	0.334376	-0.18313	0.578033	-0.25952	-0.31532	-0.18897
Q05655_S304_1_1_	Q05655	PRKCD	0.251889	0.687439	0.342226	-0.00558	-0.11087	-1.03271	-0.12765	-0.69108	-0.07555	0.327778	1.517583	-0.31653	0.301914
Q05682_S202_1_1_	Q05682	CALD1	-0.2765	-1.1922	-3.72201	1.124555	-0.25892	-1.64974	-1.78555	0.627489	-4.57004	0.739329	0.095746	-0.84747	-0.41284
Q06265_S306_1_1_	Q06265	EXOSC9	-0.1454	-0.04238	-0.75541	-0.27687	-0.34405	1.024491	0.169933	0.091415	0.878404	0.538273	-0.83611	-0.29118	-1.38765
Q06265_T305_1_0_	Q06265	EXOSC9	-0.23559	0.627322	-1.18509	-0.242	-0.34013	0.838423	0.475238	0.466702	1.222499	0.423337	-0.64177	-0.35325	-1.0349
Q06481_S213_1_1_	Q06481	APLP2	-1.75329	-4.57126	-0.32044	-1.91532	-1.85566	-2.42369	-1.91631	-0.73364	0.152149	0.006965	-0.24281	-1.58223	-0.60023
Q07157_S1278_1_1	Q07157	TJP1	-1.19903	0.02838	-0.30757	-0.22306	-0.5062	0.863919	0.132504	-1.06796	-0.45012	-0.49462	-0.81375	-0.16662	-2.24648
Q07954_S4520_1_1	Q07954	LRP1	0.870179	-0.1347	-0.4112	0.25499	-1.53743	0.208304	0.711593	0.074159	0.007699	0.805919	-0.87038	-0.68106	0.391977
Q07955_S199;S201	Q07955	SRSF1	0.602653	0.145365	0.375308	0.137671	0.953568	0.678813	-0.65367	0.293452	-0.6648	0.389436	-0.29339	-0.45794	-0.47172
Q07955_S199_1_1_	Q07955	SRSF1	-0.63566	-0.38137	-1.81743	1.328504	0.365344	1.270134	0.354169	1.072372	0.451807	0.618075	0.710136	-0.06368	0.273753
Q08357_S256_1_1_	Q08357	SLC20A2	0.109652	-1.39147	-1.16482	2.32518	0.247676	-0.92947	-2.20487	3.064763	-0.8155	1.276799	-0.93391	-1.51319	-0.98127
Q08499_S202_1_1_	Q08499	PDE4D	-0.69702	-1.26052	-2.12257	0.192032	-1.15931	-0.51656	-0.73165	0.080783	-0.09389	1.718022	0.737863	-0.08903	-1.15987
Q08945_S659_1_1_	Q08945	SSRP1	0.098881	0.317284	-1.41105	0.49984	-1.46731	-1.19137	0.777159	-0.23594	0.139755	0.460272	1.446021	0.175832	0.446854
Q09666_S1068_1_1	Q09666	AHNAK	-0.59799	0.640765	-1.34472	0.237607	-1.13624	-0.20907	0.318741	-1.294	1.09954	1.706674	-2.56019	-0.73121	-0.2405
Q09666_S1286_1_1	Q09666	AHNAK	-0.34687	0.103341	0.643262	-1.06537	-1.52524	-0.58988	-0.07521	-0.80579	0.819346	-0.16627	-0.07871	-0.43541	0.628162
Q09666_S135_1_1_	Q09666	AHNAK	0.225495	-0.61381	-0.44049	0.185354	-0.79001	-0.71731	-0.95186	-0.58751	-0.24651	-0.06635	-0.65667	-1.63691	-0.50102
Q09666_S177_1_1_	Q09666	AHNAK	1.125929	-0.22596	-0.69243	0.594894	-0.93162	0.733173	0.08555	-0.48481	-0.99404	0.621523	-0.79056	1.275242	-0.88349
Q09666_S210_1_1_	Q09666	AHNAK	-0.01328	-0.34455	-0.724	0.669673	-0.80272	-1.27398	-1.04753	-0.55956	0.041413	0.455064	-0.16249	-0.72709	-0.3074
Q09666_S216_1_1_	Q09666	AHNAK	-0.16873	-0.36825	-0.84127	0.959515	0.317981	1.094983	0.416492	0.054115	-0.54764	0.483093	-0.68449	-0.00302	0.571357
Q09666_S3054_1_1	Q09666	AHNAK	-0.34687	0.103341	0.643262	-1.06537	-1.52524	-0.58988	-0.07521	-0.80579	0.819346	-0.16627	-0.07871	-0.43541	0.628162
Q09666_S5110_1_1	Q09666	AHNAK	0.939933	-0.26061	0.135475	-1.07138	-1.36101	-0.703	-0.07401	-0.8326	-0.05028	0.015277	-0.15375	-0.91242	-0.66373
Q09666_S5448_1_1	Q09666	AHNAK	-0.57381	0.313449	-1.63893	0.245484	-1.7236	-0.41524	-0.94831	-0.56203	0.642169	0.251479	0.970771	0.50778	0.44434
Q09666_S5731_1_1	Q09666	AHNAK	0.867102	0.037766	-0.4816	0.408905	0.428561	0.264069	0.559912	0.164214	-0.58898	0.448663	1.019298	0.167226	0.27633
Q09666_S5752_1_1	Q09666	AHNAK	0.34202	0.110488	-0.33226	0.374793	-0.83724	0.682993	0.479338	0.213913	-0.19365	0.932325	0.485983	-0.87383	-0.36895
Q09666_S5763_1_1	Q09666	AHNAK	0.813298	0.290967	-0.51401	1.480813	-0.32965	-0.44567	0.068351	0.05859	-0.88018	0.892037	0.021101	0.595362	0.22323
Q09666_S5841_1_1	Q09666	AHNAK	0.633516	0.333635	-0.8605	0.837382	-0.90902	0.761932	0.81227	0.278324	0.706147	1.033695	1.031295	0.261835	-0.09173
Q09666_S93_1_1_N	Q09666	AHNAK	0.024228	-0.60292	-0.28597	1.55421	0.262491	-0.80796	-0.0887	0.46773	-0.19043	1.204933	-1.18897	-1.14179	0.11517
Q09666_T5794_1_1	Q09666	AHNAK	0.790428	0.112594	0.652636	-0.33775	-1.80354	-0.40549	0.657186	-0.40633	-0.41415	0.714738	-0.37826	-0.44499	-0.57828
Q0JRZ9_S403_1_1_	Q0JRZ9	FCHO2	0.19803	-0.88082	0.041185	0.492298	-0.58418	-0.16397	-0.59876	-0.41663	-0.23933	0.914221	0.786208	-1.02738	-0.52017
Q0ZGT2_S80_1_1_	Q0ZGT2	NEXN	-0.63151	-1.67382	-1.35366	0.809141	-0.16626	0.241879	-0.06202	0.037261	1.385038	1.276512	-0.48385	1.054424	-0.18115
Q12802_S2498_1_1	Q12802	AKAP13	-0.92556	-0.96055	-1.26691	0.718681	-0.53112	-1.82978	-1.57073	0.847825	-0.25615	0.674285	-0.15251	-0.93544	-0.16473
Q12802_T2411_1_1	Q12802	AKAP13	-0.12576	-0.05826	-0.45379	0.019278	-0.1691	-0.65432	0.155048	0.238885	0.47555	-0.43206	-2.17671	-0.11532	0.251498
Q12830_S216_1_1_	Q12830	BPTF	0.144277	0.475394	-0.38214	-0.20347	-0.02791	0.233733	-0.94973	0.862545	-0.48736	-0.11076	-0.14989	-0.46005	-0.22502
Q12857_S280_1_1_	Q12857	NFIA	0.101415	-0.49638	0.636291	-0.26911	-1.94311	-0.74236	-1.79811	-0.70841	-0.64812	0.171962	0.616321	-1.08965	-1.25505
Q12888_S1113_1_0	Q12888	TP53BP1	0.023207	-0.54328	-0.4726	0.76262	1.005094	0.033982	0.261577	0.412261	-0.24317	0.493485	-0.48849	0.173054	0.292484
Q12888_S265_1_1_	Q12888	TP53BP1	0.561432	-0.07232	-0.61459	-0.44451	-1.13831	0.994337	-0.70609	-0.3926	0.24631	0.645547	0.470685	1.04966	-0.66332

Q12888_S294_1_1_	Q12888	TP53BP1	0.628194	-0.4169	0.365128	0.567063	-0.49077	0.493533	-0.8833	0.435958	0.101781	2.329714	-0.36631	0.870597	-0.17317
Q12888_S380_1_1_	Q12888	TP53BP1	-0.73636	-0.40867	-0.79925	0.306376	-0.0678	-0.42835	0.330131	0.305312	-0.42446	0.277249	-0.04579	0.235502	-0.13149
Q12888_S552_1_1_	Q12888	TP53BP1	0.033935	-0.39133	-0.12579	-0.27895	-2.3625	0.103321	0.083579	0.900167	0.108512	0.349579	-0.14998	0.313327	0.162086
Q12893_S320_1_1_	Q12893	TMEM115	0.895292	-0.03962	1.019768	-0.96685	-1.26744	-0.80312	0.031964	0.058946	-0.38575	-0.25324	-0.35523	-1.74087	-0.1719
Q12955_S4342_1_1	Q12955	ANK3	0.168332	-0.6925	0.128134	0.036245	-2.87368	-0.38723	0.168572	-0.89026	-0.50779	-0.06153	0.478285	-0.94689	-0.83138
Q13045_S436_1_1_	Q13045	FLII	-0.02859	0.237885	-0.43166	-0.10902	-0.68007	0.113022	0.556741	1.156078	-0.34087	-0.02288	-0.1498	-0.2792	-0.19522
Q13131_S496_1_1_	Q13131	PRKAA1	0.041368	-0.35225	-0.15607	-0.51446	-2.02633	-0.76345	0.145414	-1.00609	-0.09829	-0.14722	-0.02948	-0.57854	-0.90593
Q13144_S544_1_1_	Q13144	EIF2B5	0.128897	-0.67253	-1.08014	1.650165	1.262195	0.159586	-0.27747	1.540158	-0.3443	1.046671	-0.28537	-1.65072	-0.25013
Q13153_S144_1_1_	Q13153	PAK1	0.433515	0.038819	-0.34505	-0.25082	-0.8009	-0.59786	-0.08201	0.346665	0.701012	0.250451	0.009121	0.588182	-0.89491
Q13153_T212_1_1_	Q13153	PAK1	1.301322	-0.83383	0.927794	-0.06491	-1.41101	-0.87615	0.049245	0.038887	-0.40571	-0.09585	-0.10561	-0.78854	-0.49973
Q13177_S141_1_1_	Q13177	PAK2	0.329497	0.442386	-0.47317	-0.21013	-0.21124	-0.70381	0.074871	0.664573	0.840342	-0.10328	0.049436	-0.23237	-0.68129
Q13185_S93_1_1_N	Q13185	CBX3	0.478596	-0.08462	0.614597	-0.02515	-0.56114	-0.83496	-0.80924	-0.24867	-0.70089	-0.03533	-0.32472	-1.17287	-1.87194
Q13185_S95_1_1_N	Q13185	CBX3	-0.29628	0.278653	-1.89698	0.179599	0.079841	0.317632	0.014945	0.089893	-0.14094	-0.65712	0.330068	-0.27968	-0.34551
Q13185_S97_1_1_N	Q13185	CBX3	-0.49164	0.627409	-0.81963	0.672566	-0.21383	-0.34107	-0.14369	0.151872	-0.6552	0.846293	-0.25344	-0.31399	-1.76384
Q13185_S99_1_1_N	Q13185	CBX3	-1.28813	-0.37272	-0.96743	0.690489	-0.5941	-0.13284	-0.35701	1.077543	-1.31258	0.589486	-0.40497	-1.60115	-0.70073
Q13200_S361_1_1_	Q13200	PSMD2	-1.19593	1.009968	-0.61848	0.068942	0.241451	1.265506	-0.16492	-0.01363	-0.17598	-0.93595	-0.35178	-0.34115	-0.99517
Q13242_S189_1_1_	Q13242	SRSF9	0.283381	-0.53386	-0.30712	0.826121	0.111758	0.219629	-0.61482	0.022286	0.073685	0.374567	-0.34888	-0.01179	-0.08157
Q13242_S204_1_1_	Q13242	SRSF9	0.938694	0.035972	-0.33644	-0.14143	0.52045	0.696047	-0.53634	-0.12951	0.335227	0.723231	-0.17412	-0.76187	-0.53537
Q13247_S303_1_1_	Q13247	SRSF6	-0.62319	-0.16573	-1.12832	1.018454	0.360932	0.460906	0.26005	0.294014	-0.28259	0.583394	0.013767	-0.86078	-0.67515
Q13247_S316_1_1_	Q13247	SRSF6	-0.50727	-0.3541	-1.58276	1.744107	1.271635	0.652027	-1.42713	2.453776	-0.8372	2.084899	-2.43171	-1.97663	-0.90279
Q13263_S473_1_1_	Q13263	TRIM28	-0.74484	-0.73686	0.576687	0.196643	-1.6487	-0.91953	-0.18802	-0.93857	-1.60369	0.197566	-1.31208	-1.78183	-0.69612
Q13283_S149_1_1_	Q13283	G3BP1	-0.26509	-0.39791	-0.66524	0.965644	0.117565	0.359912	-0.21984	-0.01918	0.077158	0.563799	0.323022	-0.51914	0.045714
Q13283_S231_1_0_	Q13283	G3BP1	0.138028	0.338971	-1.03712	1.980248	-0.67683	0.145062	-0.38062	-0.88164	0.202603	-1.6336	0.143753	-0.83831	0.387172
Q13283_S232_1_1_	Q13283	G3BP1	0.300967	0.210925	-0.51307	0.959695	-0.39591	0.504114	-0.13554	-0.94961	0.752036	-1.35873	0.515482	0.371644	0.307612
Q13308_S784_1_1_	Q13308	PTK7	-0.23361	-0.17986	-0.23583	-1.45578	-2.34864	-0.75181	-0.04639	-0.7509	0.456968	-0.2026	0.031428	-0.30593	-0.34177
Q13367_S272_1_1_	Q13367	AP3B2	-0.52502	-1.10085	-1.87343	-0.59642	-1.38723	0.700455	-2.35084	-1.72701	-2.18494	-1.5744	-3.23204	-1.26143	-0.61726
Q13425_S110_1_1_	Q13425	SNTB2	-0.01039	-0.15427	-0.54466	0.789902	0.293513	-0.03009	-0.18452	-0.54385	0.166865	-0.40885	0.125225	-0.12481	-0.32873
Q13425_S95_1_1_N	Q13425	SNTB2	0.627006	0.28331	-0.00233	0.117284	-0.02664	0.13259	0.124527	0.240836	0.030156	0.514014	-1.27281	0.747026	0.14196
Q13427_S687_1_1_	Q13427	PPIG	-0.20292	-0.39604	-0.2517	-0.94853	-0.45466	-0.18547	-0.08714	-0.49413	-0.06664	0.309268	-0.21285	-0.25927	0.276696
Q13427_T358_1_1_	Q13427	PPIG	0.043941	-0.2744	-0.56723	0.21021	-0.22409	-0.87829	-1.06854	0.315225	0.286241	0.502506	-0.25314	-0.38135	-0.30436
Q13428_S1350_1_1	Q13428	TCOF1	-0.44369	0.23543	-0.08261	0.357557	0.561369	-0.58771	-0.51724	-0.59687	0.220301	-0.02518	-0.50945	-0.34582	0.660698
Q13428_S1378_1_1	Q13428	TCOF1	-1.13956	0.260692	0.13929	1.250951	0.44359	-0.59527	-0.01121	1.281588	-0.66046	0.819573	0.747532	0.764609	1.559159
Q13428_S233_1_1_	Q13428	TCOF1	-0.63026	-0.3514	-0.44098	-0.34818	0.687389	0.185451	0.355079	-0.92553	0.226264	0.609347	-0.09844	-0.35543	0.830562
Q13428_S381_1_1_	Q13428	TCOF1	-1.31601	-0.38063	-0.77793	0.193168	0.429885	-0.0006	-0.40703	-0.13018	0.364504	0.602608	0.138497	0.630063	1.199636
Q13428_S906_1_1_	Q13428	TCOF1	-0.00105	-0.17554	-0.82631	-0.47827	0.742369	0.117904	-0.22624	-0.49971	0.518894	0.350098	0.553562	1.263912	0.947333
Q13435_S309_1_1_	Q13435	SF3B2	0.549194	-0.48256	-1.33559	1.410073	0.594488	0.506369	0.158009	0.750262	-0.16075	0.728915	0.003495	-0.29038	0.21398
Q13442_S60;S63_2	Q13442	PDAP1	-1.35077	-0.1069	-0.63253	1.599621	0.437951	-0.45025	-0.21768	0.600772	0.280989	0.466527	0.036439	0.975545	0.222164
Q13459_S1354_1_1	Q13459	MYO9B	-0.30968	0.04215	-0.48416	-0.39205	-0.55462	0.294049	0.27149	-1.03647	-0.00227	-0.04817	-0.24077	-0.21062	0.794523
Q13459_T1346_1_1	Q13459	MYO9B	-0.87403	-0.88042	-0.36426	0.212566	-1.15016	-0.82758	0.350738	-0.53754	1.687904	-0.12142	0.217206	-0.28552	-0.69265
Q13501_S272_1_1_	Q13501	SQSTM1	0.102661	-0.836	-0.03659	0.590646	-0.6733	0.041934	-1.04176	0.233048	-0.93366	0.363001	-1.09284	-0.29353	-0.03562
Q13501_T269;S272	Q13501	SQSTM1	-0.42518	-0.43476	-0.10464	0.388053	1.147322	0.664283	-0.58516	-0.16399	-0.55978	0.243865	-0.83906	0.342385	1.431542
Q13501_T269_1_1_	Q13501	SQSTM1	-0.45766	-0.25183	-0.85989	-0.19181	0.563373	0.964789	0.732617	0.010316	-0.51969	0.121425	0.82001	1.033325	1.408646
Q13523_S257_1_1_	Q13523	PRPF4B	0.582178	0.646716	-0.19202	0.063208	0.454896	-0.08471	-0.602	0.51276	0.109309	0.520313	0.128259	-0.50908	-0.82984
Q13523_S294_1_1_	Q13523	PRPF4B	-1.43406	0.127787	0.029451	1.149411	0.889987	-0.98111	-0.62516	1.825013	-1.29054	0.353033	0.057698	0.072968	-0.00098
Q13547_S393_1_1_	Q13547	HDAC1	-0.36037	-0.4869	-0.42698	-0.66589	-0.13366	0.444352	0.936111	0.418862	0.509251	0.258841	0.477394	-0.07407	-0.20578
Q13576_S16_1_1_N	Q13576	IQGAP2	0.42623	0.729474	-1.37917	0.021829	-1.54941	0.819424	0.963476	-0.56019	-0.68392	0.401675	-0.04539	-1.07578	0.480752
Q13586_S257_1_1_	Q13586	STIM1	0.28373	-2.8101	-0.17658	1.052361	-2.77174	-1.81814	-1.76757	0.494187	-2.24407	0.888558	-2.6554	-2.186	-2.04742
Q13595_S260;S262	Q13595	TRA2A	-0.17925	-0.38812	-0.44651	-0.29241	0.395372	0.480164	-0.45079	-0.18884	0.649993	0.372788	0.907461	0.048507	0.416699
Q13595_S262_1_1_	Q13595	TRA2A	-1.14978	-0.35739	-1.78476	0.876648	0.472111	0.938703	-1.99245	2.727764	-1.14559	1.396352	-0.10824	-0.67665	-0.34191
Q13610_S50_1_1_\	Q13610	PWP1	-2.09235	-0.71024	-0.44474	1.918461	-0.20883	0.060732	0.212034	-0.09508	-1.48007	0.347127	-0.70143	-0.75136	1.100628
Q13627_Y321_1_1_	Q13627	DYRK1A	-0.55992	-0.13952	-1.1081	1.099717	0.121111	-0.05675	-0.19604	-0.0992	0.129524	0.446162	0.025972	0.676642	-0.24058
Q13813_S1217_1_1	Q13813	SPTAN1	0.423033	-0.62251	0.457157	0.157052	0.208556	-0.47067	-0.2736	0.428106	-0.24331	0.145994	-0.32192	-1.04605	-1.07923
Q13895_S98_1_1_\	Q13895	BYSL	-1.19308	0.639708	-1.4269	1.001606	-0.25936	0.348284	-0.71416	0.521604	0.244789	0.934821	-0.11568	-0.23861	0.100344
Q14126_S703_1_1_	Q14126	DSG2	0.527446	-1.3234	-0.66305	-1.01778	-1.82373	-1.16495	0.163906	-0.78002	-0.26411	-0.08206	-0.87595	-0.43889	-0.33354
Q14152_S881_1_1_	Q14152	EIF3A	-0.30933	0.337309	-1.69675	1.09112	-1.11818	-0.85503	0.611049	-0.64316	2.28213	0.444445	-0.18567	-0.72665	-1.58255
Q14155_S518_1_1_	Q14155	ARHGEF7	-0.22716	-0.00481	-0.60147	1.089788	0.237709	0.292353	0.134656	0.553624	0.168067	1.905919	0.046478	-0.68001	0.110029
Q14155_S694_1_1_	Q14155	ARHGEF7	0.466755	0.890842	0.050384	0.117436	-0.59704	-0.78272	-0.24307	-0.09781	-0.24793	-0.42507	-0.79554	-1.55606	-0.01352
Q14160_S1220_1_1	Q14160	SCRIB	0.267295	0.324849	0.194919	-0.12798	0.09699	0.053567	-0.21533	-0.62453	-0.31242	-0.08289	-0.51351	-0.91575	0.01324
Q14160_S1486_1_1	Q14160	SCRIB	0.45297	-1.09672	0.382749	-0.56618	-1.76886	0.662829	-1.10771	0.365503	-0.13106	0.880439	0.521423	1.85933	0.036826
Q14160_S1566_1_1	Q14160	SCRIB	0.02634	0.767904	0.888496	-0.80818	-0.16383	0.774449	0.199969	0.403625	0.527617	0.068228	-0.25074	-0.67898	-0.71418
Q14160_S835_1_1_	Q14160	SCRIB	-0.05108	-0.18282	0.580487	0.323893	-0.01182	0.931	0.178702	0.346381	0.170535	0.633031	-0.46014	1.345465	-0.23126
Q14195_S522_1_1_	Q14195	DPYSL3	-0.56408	-0.49052	-0.66875	-0.10697	-0.35845	-0.00605	-0.258	-1.579	0.075396	-0.78523	-1.36721	-1.05636	-0.23289
Q14195_T509_1_1_	Q14195	DPYSL3	-1.18813	-0.81078	0.070827	0.213038	0.396175	1.42833	0.029462	0.131682	0.764104	0.552858	0.058948	-0.00551	-0.14153
Q14247_S417_1_0_	Q14247	CTTN	0.919602	-0.01066	-0.06126	0.908271	-0.88093	-0.24826	-0.76284	-0.68921	-0.10047	-0.17894	-1.41344	-0.99392	-1.27598
Q14247_S418_1_1_	Q14247	CTTN	0.850677	0.040074	0.152786	1.142847	-0.74873	-0.64556	-1.01762	-0.10193	-0.15404	-0.23375	-1.51487	-1.45893	-1.36835
Q14315_S2233_1_1	Q14315	FLNC	-0.24156	0.145566	-0.77255	-0.5257	-0.73855	0.048736	-0.50144	-0.5227	0.169439	-0.22979	-1.09384	-0.00461	0.058932
Q14498_S136_1_1_	Q14498	RBM39	-0.39764	-0.3503	-0.36263	0.181952	-0.05875	-0.29442	-0.26279	0.194995	0.282674	0.405236	0.292067	-0.40893	-0.03889
Q14498_S97_1_1_N	Q14498	RBM39	0.051152	-0.10286	-0.7768	-0.44771	-0.82549	-0.27052	0.028712	-1.23674	0.033122	0.096984	0.111909	0.288202	-0.09618
Q14573_S1832_1_1	Q14573	ITPR3	0.429376	-0.5682	-0.0114	-0.33937	-1.07796	-1.04444	1.198156	-0.36628	0.200052	0.124306	-0.76931	-1.08757	0.074388
Q14669_S1376_1_(	Q14669	TRIP12	-0.23608	-0.40382	-0.98501	1.885428	1.078408	0.199741	0.65411	0.440905	-0.04366	0.505682	-0.10061	-0.41225	0.646585
Q14669_S312_1_1_	Q14669	TRIP12	-0.0944	-0.05965	-0.42656	0.246962	-0.8011	0.193093	-0.00973	0.115473	-0.09723	0.347065	0.032908	-0.22699	0.18079
Q14671_S709_1_1_	Q14671	PUM1	-0.21692	-0.15564	0.184398	0.445288	-1.15854	0.368531	0.223932	-0.31409	0.008561	-0.06622	-0.23503	-0.31688	0.217454
Q14676_S168_1_1_	Q14676	MDC1	-0.22963	0.388115	-0.19386	1.470048	-0.42	0.508104	1.225746	0.224135	0.447281	0.66174	0.32774	0.314353	0.348541
Q14739_S99_1_1_N	Q14739	LBR	0.008687	-0.52429	0.241181	-0.12962	-0.81872	-0.28284	0.462255	-0.35256	0.273976	-0.35832	0.244054	0.11415	-0.37048
Q14767_S250_1_1_	Q14767	LTBP2	1.100307	1.347095	-0.22222	0.121984	0.525327	0.150613	0.106152	0.020742	-0.09267	0.140844	-0.64241	-0.85913	1.52647
Q14839_S1308_1_1	Q14839	CHD4	0.054184	-0.57669	-0.81099	0.259656	-1.53784	-1.40029	-0.2407	-0.28001	0.819205	-0.04511	-0.28475	0.028623	-1.38648
Q14839_S1535_1_1	Q14839	CHD4	-0.55364	-0.26048	-1.03296	0.244011	0.732417	-0.36515	-0.19116	0.009944	0.089163	0.284627	-0.95782	-0.3923	-0.17913
Q14847_S146_1_1_	Q14847	LASP1	-0.58753	-0.21324	-0.53854	0.295156	-0.52735	-0.53959	0.442621	1.28433	0.445257	2.976612	0.067879	-0.28496	0.065293
Q14938_S265_1_1_	Q14938	NFIX	-0.22998	-0.47214	0.101958	0.212856	-2.50817	-1.07624	-0.92924	0.949913	0.163455	0.347622	-0.92063	-1.0232	-0.37147
Q14938_S280_1_1_	Q14938	NFIX	-0.86674	-0.18178	-0.17301	1.026333	-0.31249	0.120986	-1.81688	1.199503	-0.44008	1.218036	-0.46125	-0.82985	0.817372
Q14978_S563_1_1_	Q14978	NOLC1	-0.8268	-0.58512	-0.78043	1.100355	0.626832	0.14855	-1.19525	0.690116	0.271998	1.340899	-0.03488	0.074508	-0.24555
Q14980_S1757_1_1	Q14980	NUMA1	-0.16627	-0.13703	0.041733	1.689348	-0.78692	0.004566	-0.81422	1.789891	0.393553	1.97017	-0.54283	0.334683	-1.38971
Q14980_S1853_1_1	Q14980	NUMA1	-0.19002	-2.4943	-2.0267	-0.5633	-2.82964	0.161024	-1.88373	-1.32346	-1.47082	-0.38254	-1.07686	-1.41765	0.792667
Q14980_S271_1_1_	Q14980	NUMA1	-0.49526	-0.51395	-0.16213	0.36554	-0.79218	0.13417	-1.12808	-0.06534	-0.59029	0.519351	-0.12705	-1.18123	-0.94862
Q15019_S218_1_1_	Q15019	2-Sep	0.507806	0.726317	0.100812	1.64497	0.874237	-1.54519	-2.43458	1.201841	-3.56852	0.497485	-0.22576	-0.78472	-1.2515
Q15036_S409_1_1_	Q15036	SNX17	-0.02692	0.128062	-0.28728	0.376338	-2.1329	-0.14168	0.243275	0.322066	-0.05662	0.314151	-0.16129	-1.45633	0.271842
Q15052_S488_1_1_	Q15052	ARHGEF6	0.468565	-0.18974	0.476664	-0.0694	-0.18329	-0.92529	0.093844	0.270049	-0.23815	0.522154	0.130923	0.900389	-0.26545
Q15052_S640_1_1_	Q15052	ARHGEF6	0.305416	0.721134	-0.0828	0.020988	-0.11306	-0.53557	0.09768	-0.88686	-0.13135	0.093457	0.848731	-0.553	0.142249
Q15052_S684_1_1_	Q15052	ARHGEF6	0.119273	0.669144	-1.6567	-0.14626	-0.68246	0.385716	0.327009	-0.63412	0.394274	0.715599	0.360895	0.081631	0.040805
Q15054_S307_1_1_	Q15054	POLD3	0.205031	0.712234	-0.4808	-0.36017	-0.96758	0.583932	0.342476	0.084753	0.559854	0.430152	-0.1197	-0.60068	-0.66938
Q15054_S307_1_1_	Q15054	POLD3	-1.13415	-1.02139	-0.32441	0.657946	-1.35591	-0.17408	-0.19612	-0.4143	-0.28065	0.696204	-0.13785	0.050276	0.584393
Q15059_S263_1_1_	Q15059	BRD3	-0.27254	-0.68599	-0.60334	0.416061	-0.55038	-0.22145	-0.68378	0.522336	0.417884	0.418032	-0.15741	-0.6087	0.599954
Q15059_S563_1_1_	Q15059	BRD3	-0.43853	-0.55001	0.17824	1.289105	0.561206	-0.47798	-0.33151	0.892662	-0.361	1.171736	-0.10546	-0.80032	-0.06402

Q15084_S428_1_1_	Q15084	PDIA6	0.193751	-0.00665	-0.88776	1.121922	-0.07322	-1.36752	-0.96427	1.639543	0.364045	1.579007	0.88215	0.382897	0.691866
Q15121_S116_1_1_	Q15121	PEA15	0.250703	0.461504	-0.06023	-0.55666	0.028397	-0.64562	0.075875	-0.35425	0.396202	-0.83425	-0.68503	-0.10753	0.114879
Q15149_S1435_1_1	Q15149	PLEC	-1.61642	0.205215	0.25067	-0.02522	0.187245	-0.07844	-0.22986	0.600746	-0.36562	0.214847	-0.15655	0.088404	0.59372
Q15149_S1721_1_1	Q15149	PLEC	1.434429	0.221842	-0.32518	-0.52269	0.185825	0.624893	-0.46851	-0.05842	0.099565	-0.05665	0.289878	1.027755	0.14775
Q15149_S4386_1_1	Q15149	PLEC	-0.18657	0.319054	-0.17768	0.069687	0.109846	-0.34397	0.40076	-0.30821	0.442535	-0.19948	-0.72063	-0.91869	0.904521
Q15149_T4030_1_1	Q15149	PLEC	-0.07692	-0.07525	-0.24775	-0.49914	-0.4027	-0.04243	-0.46761	-0.81312	0.266793	-0.14023	-1.44888	-0.73065	-0.05344
Q15311_S29_1_1_N	Q15311	RALBP1	0.340681	0.720218	-0.408	0.156066	-2.03065	0.571976	0.363128	0.103924	0.182759	0.04157	0.238794	-0.40348	-2.31334
Q15366_S364_1_0_	Q15366	PCBP2	-0.12643	-0.28989	-0.32104	1.122048	0.462968	0.536658	0.42581	-0.00879	-0.24937	1.545671	-0.41845	-1.55968	-0.53042
Q15424_S601_1_1_	Q15424	SAFB	0.066929	-1.96736	-0.58322	1.036543	-0.68133	-2.33945	-0.70216	0.52401	-0.41047	0.62369	-0.73333	0.299955	-0.09377
Q15424_S604_1_1_	Q15424	SAFB	0.118688	-0.87273	-0.40081	0.173252	-0.49282	-2.12378	-1.28093	0.598233	0.61612	-0.24112	-1.41781	0.128685	-1.15895
Q15435_S27_1_1_N	Q15435	PPP1R7	0.031847	-0.02404	-0.16002	1.104345	-1.4186	-0.19477	-0.47399	1.500049	0.150637	0.869999	-0.12991	-0.56607	-0.63975
Q15459_S329_1_1_	Q15459	SF3A1	0.053707	-0.0179	-0.63322	0.872647	0.106811	-0.1835	0.081772	0.970391	0.868277	1.228487	0.065109	-0.27224	0.097504
Q15459_S329_1_1_	Q15459	SF3A1	-0.26743	-0.42301	-0.5104	0.958058	0.090704	0.333838	0.089321	0.805886	0.144	0.843839	-0.17266	-0.40352	-0.15768
Q15477_S255_1_0_	Q15477	SKIV2L	-2.1759	0.170243	-0.67491	-0.0796	-0.29187	0.045935	-0.71366	0.312218	0.269294	0.208345	-0.79913	-0.90424	-0.3563
Q15477_S256_1_1_	Q15477	SKIV2L	-0.24288	-0.65614	-0.71881	0.029554	-0.038	-0.15648	-0.04218	0.181734	-0.0461	-0.35598	-0.49408	-0.45775	-0.26984
Q15555_S228_1_1_	Q15555	MAPRE2	0.363058	0.211167	-0.59848	0.395394	-0.60332	-0.58106	0.582977	0.537433	-0.17512	0.391518	-1.03978	0.04732	0.141148
Q15596_S736_1_1_	Q15596	NCOA2	0.218347	0.516258	-0.12187	0.182257	0.199307	0.129149	-0.11361	0.250532	-0.06978	-0.01276	0.068528	0.057732	-0.01294
Q15773_S238_1_1_	Q15773	MLF2	-1.72301	1.900661	-0.63675	0.701437	-0.07693	1.255372	-0.454	0.956573	0.071615	0.710934	-0.66534	-0.07459	0.546717
Q15942_S267_1_1_	Q15942	ZYX	-0.85386	-0.36743	0.581448	-0.8653	-0.39646	0.508209	0.394018	-0.92323	-0.67494	0.247399	-0.20996	0.616908	-0.42123
Q15942_S344_1_1_	Q15942	ZYX	0.213978	-0.1466	-0.12946	-0.39968	-1.35528	-1.34396	-0.87313	-0.06016	-0.47542	0.269898	-1.15004	-1.41557	-0.52052
Q16204_S240;S244	Q16204	CCDC6	0.16878	-1.11589	-0.01371	0.383116	-0.54838	-1.21334	-0.92117	-1.32871	-0.29211	0.288715	-0.01514	-0.58262	-0.63049
Q16204_S244_1_1_	Q16204	CCDC6	-0.00576	-0.24519	-0.19853	0.369191	0.027095	0.177893	-0.60792	-0.90775	-0.70377	0.184089	-0.16974	-1.61134	-0.38321
Q16512_S374_1_1_	Q16512	PKN1	0.498768	-0.2163	-0.1304	0.274378	-1.62504	0.016798	0.120153	-0.31861	0.725731	0.239343	0.0127	-0.77057	0.641762
Q16512_S916_1_1_	Q16512	PKN1	-0.10551	-0.0923	-0.5244	0.875866	-0.99827	1.090643	0.821297	0.608355	0.319688	1.581487	0.300788	-0.2037	-0.49566
Q16513_S583_1_1_	Q16513	PKN2	0.293575	-0.49535	0.040346	0.500922	-0.77157	-0.90476	-0.03389	-0.36653	-0.988	0.027815	-0.89225	-1.4799	-0.42833
Q16513_T958_1_1_	Q16513	PKN2	-0.50366	-0.40925	0.097468	0.52194	-0.014	0.112223	0.207619	0.438122	0.576272	1.800211	0.233115	-0.2586	-0.73905
Q16555_T514_1_1_	Q16555	DPYSL2	-1.14393	-0.95422	0.270428	-0.26353	-1.62971	0.491334	0.431605	-1.00658	1.505825	-0.72878	-0.35681	2.327507	-0.00784
Q16643_S141_1_1_	Q16643	DBN1	-0.07389	-0.3036	-0.72864	-0.38019	0.079882	-0.41335	-0.60186	0.319505	-0.65569	0.735143	-0.56746	0.473126	0.23411
Q16643_S142_1_1_	Q16643	DBN1	-0.30456	-0.59405	-0.54311	0.852014	-0.25793	0.251544	0.922313	-0.15455	0.594392	0.588863	-0.83622	0.662324	0.459126
Q16643_S337_1_0_	Q16643	DBN1	-1.19133	-0.26713	-0.27637	0.758783	0.342786	-0.34138	-1.02924	0.48538	-1.61967	0.995175	0.491224	-0.6901	-0.09938
Q1KMD3_S161_1_1	Q1KMD3	HNRNPUL	0.066527	-1.22347	-1.28396	0.504532	-0.68596	0.035363	-0.25954	0.331843	-0.30546	0.153405	-0.30915	-1.17913	-0.2294
Q1KMD3_T165_1_1	Q1KMD3	HNRNPUL	-0.96241	-1.46593	-0.26698	0.08855	-0.07895	1.174763	0.535966	-0.1145	-0.71455	-0.15192	0.029852	0.219013	-0.48947
Q2M2I8_S678_1_1_	Q2M2I8	AAK1	-0.50553	0.081302	-1.19517	-0.58733	0.142053	1.367545	0.311723	-0.17584	-0.37667	-0.14157	0.030662	0.016406	-0.17449
Q2TBE0_S479_1_1	Q2TBE0	CWF19L2	0.973144	-0.70493	-0.61449	0.058493	-0.38225	-0.28741	-0.57707	-0.83103	0.098021	0.012191	0.394646	-0.06548	-0.07956
Q32MZ4_S120_1_1	Q32MZ4	LRRFIP1	-0.60316	-1.1196	-0.4171	2.17747	-0.07911	-0.74524	-0.54452	0.944554	0.194947	1.021561	-0.07774	-0.28477	-0.18435
Q32P44_S176_1_1_	Q32P44	EML3	0.247945	0.013811	0.247759	0.338102	-0.05261	-1.25245	-0.02502	-0.93807	-0.53513	-0.1008	-0.17555	-1.07298	0.206935
Q4KMP7_S132_1_1	Q4KMP7	TBC1D10E	0.053078	0.036462	0.009944	-0.81509	-0.19052	0.879285	0.265413	-0.1854	-0.48261	-1.01216	-1.14233	0.695975	-0.48229
Q53EL6_S457_1_1_	Q53EL6	PDCD4	0.795238	-0.12137	0.228692	-0.60674	-1.3123	-1.6341	-0.78001	-1.85085	-0.02522	-0.89513	-0.35502	-0.97351	-1.21373
Q53EL6_S94_1_1_N	Q53EL6	PDCD4	-0.37443	-0.05168	-0.02376	-0.52533	0.116986	-1.19541	-0.09536	0.251227	-0.32168	0.268541	0.446766	0.008129	0.129622
Q5H9R7_S617_1_1_	Q5H9R7	PPP6R3	-0.04425	-0.54974	0.177131	1.139298	-0.24438	0.205791	0.308864	0.359495	0.5514	0.255053	0.35637	-0.01956	-0.04316
Q5JRA6_S727_1_1_	Q5JRA6	MIA3	-1.34928	-0.76023	-2.3392	1.991136	-0.00641	0.24147	1.194369	1.351403	-0.20171	1.297893	1.56608	-0.69145	-0.51561
Q5JSH3_S403_1_1_	Q5JSH3	WDR44	0.928231	1.005405	-0.22293	-0.14953	-0.95859	0.19319	-0.07235	0.095813	-0.17675	0.117517	0.156234	-0.48115	-0.45885
Q5JSH3_S50_1_1_N	Q5JSH3	WDR44	-0.13845	0.163467	0.218972	0.078731	-0.68794	0.92982	-0.46566	-0.06136	0.061762	0.320712	-0.63339	-0.24479	-0.09529
Q5JSH3_S561_1_1_	Q5JSH3	WDR44	-0.42091	-0.54282	0.151708	-0.24807	1.014291	0.465734	-1.57394	-0.173	-0.42993	1.41009	0.040233	0.003381	0.044957
Q5JSZ5_S388_1_1_	Q5JSZ5	PRRC2B	0.078794	0.49223	-0.30728	0.096468	0.211329	0.562833	0.035573	-0.46703	0.512172	0.393717	-0.62639	-0.32578	-0.22275
Q5JTD0_S300_1_1_	Q5JTD0	TJAP1	0.119901	0.002414	-0.20521	1.071573	0.185122	-0.42149	-0.64741	-0.2373	0.05034	0.556971	-0.98635	0.161361	-0.08769
Q5JTV8_S156;S157	Q5JTV8	TOR1AIP1	1.647996	2.864638	-0.43174	2.236	1.443798	-1.20073	-1.30443	1.307319	-0.49315	0.211017	-0.62142	-0.46327	0.061348
Q5SNT2_S441_1_1	Q5SNT2	TMEM201	0.152202	-0.03954	-0.26669	0.546637	0.266432	-0.03943	0.332318	-0.06029	-0.83616	0.283004	0.75512	1.346931	1.286776
Q5SSJ5_T51_1_1_N	Q5SSJ5	HP1BP3	-0.88902	-0.10427	-0.59219	-0.14143	0.731849	-0.31219	0.76129	-0.79361	-0.29326	0.331703	-0.17096	-0.30022	0.623604
Q5SW79_S1112_1_	Q5SW79	CEP170	-0.76567	-1.07198	-0.82647	0.88153	-0.80407	1.3344	0.511126	0.113856	0.461561	0.075142	0.405834	-0.63031	-0.48191
Q5SW79_S126_1_1	Q5SW79	CEP170	-1.64041	-2.22496	-1.00589	1.071843	-0.49196	-1.57916	-1.80629	0.672483	-1.14696	0.835019	-2.03834	-1.96973	-1.6813
Q5T0N5_S295_1_1_	Q5T0N5	FNBP1L	-0.00838	-0.5292	0.08016	0.046175	-0.57752	-0.38328	0.319458	-0.50483	0.043008	-0.6417	0.085633	-0.21528	-1.20845
Q5T1M5_S1162_1_	Q5T1M5	FKBP15	-0.08677	-0.46395	0.027516	-0.38692	-0.33594	1.485742	-0.40916	-0.2275	-0.16762	-0.12603	0.126345	0.126127	-0.69615
Q5T1M5_S1164_1_	Q5T1M5	FKBP15	-0.29155	-0.16293	-0.6836	-0.21131	-0.02821	1.293829	0.673188	-0.40499	0.705301	-0.60491	0.675187	0.90721	0.415872
Q5T200_S1364_1_1	Q5T200	ZC3H13	0.522958	-1.9945	0.229788	-0.47886	-2.56437	-1.38154	-1.40431	-0.67184	-0.2231	-0.35771	-0.42284	-2.10309	-0.56927
Q5T200_S1366_1_1	Q5T200	ZC3H13	0.507392	-0.55872	-0.28847	0.20081	-1.79497	-0.02423	0.574844	-0.49483	0.301062	0.893864	0.612843	0.034249	-0.82123
Q5T200_S198_1_1_	Q5T200	ZC3H13	0.210187	-0.06911	-0.83596	1.417271	0.096897	-0.12473	0.170843	0.656917	0.205562	1.036987	1.366247	-0.13629	0.203899
Q5T200_S877_1_1_	Q5T200	ZC3H13	0.121255	-0.22732	-0.66512	0.556883	-0.2633	-0.02962	0.158404	0.207143	0.000151	0.408511	0.03228	0.547432	0.764164
Q5T200_T1033_1_1	Q5T200	ZC3H13	0.376906	-0.46164	-0.37243	0.103713	0.311013	-0.40985	-0.26884	1.114083	1.534519	0.554974	-0.38777	-0.59139	-0.54794
Q5T4S7_S181_1_1_	Q5T4S7	UBR4	0.259676	0.017074	-0.28288	0.063653	-0.19224	-0.88866	-1.82928	1.25021	-0.95029	1.0018	-0.28666	-0.6785	-0.45417
Q5T7W0_S429_1_1	Q5T7W0	ZNF618	-0.36143	-0.10977	-1.09568	0.546544	0.013002	0.855718	-0.25697	0.393227	0.340344	0.299924	0.281621	-1.35232	-0.52076
Q5TGY3_S1476_1_	Q5TGY3	AHDC1	0.344368	0.44455	-0.11895	-1.34027	-0.4786	0.020323	0.364316	-0.66123	0.267845	0.154702	0.318692	0.406909	-0.45147
Q5TKA1_S65_1_1_	Q5TKA1	LIN9	2.462727	-0.99275	-0.04626	-0.79004	-2.83254	-0.58103	-0.62399	-0.42125	0.540771	0.370214	0.139897	0.062457	-1.59906
Q5U651_S328;S331	Q5U651	RASIP1	-0.39867	-0.29325	-0.69329	-0.75845	-0.22453	1.059983	0.451558	0.239577	-1.40575	-0.37563	0.178555	-0.29751	-1.42405
Q5VSL9_S335_1_1_	Q5VSL9	STRIP1	0.079524	0.058729	-0.28007	0.981259	0.474807	-1.16356	-0.52698	0.025877	-0.3044	1.353593	0.101512	-0.76049	-0.43492
Q5VSL9_S59_1_1_N	Q5VSL9	STRIP1	0.270356	0.031445	-0.42769	-0.86441	-0.72825	-1.06038	0.347603	-0.48545	0.243092	-0.51679	-0.37398	-0.74776	-0.55784
Q5VT25_S1721_1_1	Q5VT25	CDC42BPA	0.205006	-0.14749	-0.72515	0.52681	0.190508	0.037037	0.058848	0.104673	0.032423	0.564903	0.105193	-0.7127	-0.07026
Q5VT52_T664_1_1_	Q5VT52	RPRD2	-0.4248	1.06988	0.286569	1.189417	-0.91151	0.361952	0.596571	-0.66114	-0.15973	0.419841	-0.15179	-0.09368	-0.42879
Q5VTL8_S491_1_1_	Q5VTL8	PRPF38B	-0.22036	0.751321	-0.36448	-1.51308	-0.13267	-0.01622	-1.019	-0.04112	-0.32187	0.324779	0.057437	-0.34007	0.113505
Q5VTR2_S138_1_1	Q5VTR2	RNF20	0.16851	-1.27302	-1.01245	0.649855	-0.89602	-0.04699	-0.19206	-0.14612	0.046254	0.728351	-0.48856	-0.76974	-0.55118
Q5VUA4_S527_1_1	Q5VUA4	ZNF318	-0.00023	0.110568	0.384917	-0.14995	0.146064	-0.24372	-0.11216	-0.64699	0.354719	0.078412	0.020142	-0.84009	-0.30513
Q5VWG9_S183_1_1	Q5VWG9	TAF3	0.393478	-0.36094	-0.23304	1.24008	0.453274	0.804297	-0.04529	1.175252	-0.2992	0.920562	0.407648	-0.41194	-0.0413
Q63HR2_S120_1_1	Q63HR2	TNS2	-0.28221	0.137134	-0.93506	-1.28922	-1.76404	-0.83764	0.060508	-0.52197	-1.06344	-0.19224	-0.4383	-0.55391	-0.21582
Q63HR2_S845_1_1	Q63HR2	TNS2	0.430694	0.29126	-0.10972	-1.45912	-1.30519	-0.32483	-0.00243	-0.15785	0.216352	0.158558	-0.00974	-1.18621	-0.04887
Q63ZY3_S356_1_1_	Q63ZY3	KANK2	-0.50099	-0.92224	-0.84115	0.249876	-1.4338	-1.2043	-0.52903	0.002949	-0.45387	0.040208	-0.98798	-0.75092	-0.16046
Q63ZY3_S375_1_1_	Q63ZY3	KANK2	-0.12479	-0.14988	-0.71742	0.362976	-0.35922	0.644215	-0.11526	-0.23858	0.043183	0.390388	0.063982	-0.68315	-0.31489
Q66K74_S657_1_1_	Q66K74	MAP1S	-0.39147	-0.30465	0.154856	-1.60741	-1.22749	0.615736	0.383258	-0.34634	1.0252	-0.35286	-0.19262	0.749943	0.284148
Q68DK2_S297_1_1_	Q68DK2	ZFYVE26	-0.10149	0.604167	-0.3527	2.21755	-0.35769	0.486679	-0.01636	0.853528	-0.53957	0.844528	0.010838	-0.50054	0.009112
Q69YN4_S1579_1_	Q69YN4	VIRMA	0.158381	-0.02993	0.055665	0.533111	0.064594	-0.54211	-1.03761	1.403053	-0.26975	0.946904	-0.21989	-0.80228	-0.59296
Q69YQ0_S220_1_1	Q69YQ0	SPECC1L	-0.17688	0.015103	-0.7886	0.328605	-0.18897	0.481242	0.420239	-0.43858	0.549617	-0.27951	0.406337	-0.25169	-0.77574
Q69YQ0_S868_1_1	Q69YQ0	SPECC1L	0.73117	0.146077	0.824163	0.540326	-1.71593	-0.57398	-0.56819	0.18389	0.352756	0.583412	-0.06192	0.181815	-0.84183
Q6IQ23_S612_1_1_	Q6IQ23	PLEKHA7	-0.57557	0.233398	-0.88951	-0.54927	-0.03645	1.302955	0.840479	0.414116	0.959359	0.262309	-0.46201	0.198693	0.274716
Q6KB66_S451_1_1	Q6KB66	KRT80	0.247367	0.515921	-0.80569	0.258733	-0.35604	-2.08421	-0.72387	1.228768	-0.05873	0.155169	-0.62586	-0.64322	1.048479
Q6NYC8_S224_1_1	Q6NYC8	PPP1R18	-0.17553	-1.02662	0.298242	0.39779	-0.16605	-1.44084	-0.56053	-0.00496	0.653857	0.431502	-0.56929	-1.15694	0.071191
Q6P3W7_S677_1_1	Q6P3W7	SCYL2	0.364076	0.707335	-0.48381	0.487497	-0.83146	0.197618	0.554439	-0.85602	-0.70962	0.082281	0.040258	-1.53269	-1.07319
Q6P4Q7_S664_1_1	Q6P4Q7	CNNM4	0.740826	-0.25569	-0.32099	-0.06092	-1.27579	-0.30228	0.503596	-0.51627	0.137505	-0.44549	0.015862	0.589503	0.332696
Q6P4R8_S228_1_1	Q6P4R8	NFRKB	-0.29081	-0.28355	-0.36997	-0.30269	0.200846	-0.11503	0.43771	0.751103	0.293839	0.409134	-0.37023	-0.66458	-0.17395
Q6P6C2_S64_1_1_	Q6P6C2	ALKBH5	0.085024	-0.63991	0.374713	0.166959	0.824348	-0.54854	-1.0882	0.404688	-0.49261	-0.02524	-0.6981	-0.42236	0.328375
Q6PID6_S197_1_1_	Q6PID6	TTC33	0.85046	-0.00589	-0.68546	0.905523	-0.79096	0.070838	-0.4145	0.274914	-0.07022	1.356788	0.415299	1.936398	-0.0222
Q6PJF5_S90_1_1_N	Q6PJF5	RHBDF2	0.77603	0.935446	0.067737	0.658184	0.135395	-1.0941	0.072382	0.448867	-0.13886	0.321759	-0.59004	0.127741	0.182035
Q6PJT7_S515_1_1_	Q6PJT7	ZC3H14	-1.0867	-1.13585	0.215504	0.888728	0.277801	0.567385	-0.44684	0.16684	-0.10265	0.418903	-0.12102	-0.17461	0.102215
Q6PKG0_T526_1_1	Q6PKG0	LARP1	-0.98077	-0.80676	-0.09762	-0.20548	0.042806	-0.05777	0.153986	-0.57128	0.458937	-0.07872	-0.04753	0.540995	0.153044
Q6R327_S21_1_1_N	Q6R327	RICTOR	0.089062	1.06743	-1.10486	1.005963	-0.04546	0.33176	-1.03706	-0.11624	-0.67729	0.628438	-0.42618	-0.64338	-0.67513

Q6UX71_S506_1_1	Q6UX71	PLXDC2	-0.05771	0.485759	-0.55229	1.115973	-1.09208	0.202175	0.546701	0.854508	0.541288	0.754088	-0.07063	0.479727	0.231288
Q6WKZ4_S280_1_1	Q6WKZ4	RAB11FIP	-0.21918	-0.17473	-0.45045	0.715719	-2.81812	0.02649	0.983387	-0.4025	-0.00062	0.872199	-1.02767	-0.58799	-1.14383
Q6ZRV2_S1003_1_	Q6ZRV2	FAM83H	-0.10072	0.350463	-0.04869	0.022427	-0.3001	-0.02268	0.831113	-1.039	0.233305	-0.24107	-0.32135	-1.02545	0.220336
Q6ZRV2_S870_1_1	Q6ZRV2	FAM83H	-0.401	-0.44073	1.152956	-0.21052	-0.04905	-0.77923	-0.25502	0.087629	-0.86946	-0.04396	-0.13264	-0.51385	-0.46136
Q6ZRV2_S892_1_1	Q6ZRV2	FAM83H	-1.80274	-1.67614	0.404075	-0.3981	0.144683	3.05E-05	0.750086	0.152138	-0.52831	-0.15339	-0.24956	-0.30683	0.233107
Q6ZSS7_T10_1_1_	Q6ZSS7	MFSD6	0.234506	-0.06388	-0.17928	-0.46925	-0.49897	0.865174	1.035836	-0.38721	-0.12442	0.530723	0.862693	0.331093	-0.43438
Q6ZUM4_S632_1_1	Q6ZUM4	ARHGAP2	-0.88325	0.371444	-1.41772	0.752227	0.128442	0.879281	0.243336	0.285261	0.473397	0.996389	1.064905	0.460032	0.378537
Q76FK4_S890_1_1	Q76FK4	NOL8	-0.81091	-1.35383	-0.77108	1.41561	0.509021	-0.98556	-1.64166	0.909181	-0.73721	0.850548	-1.52443	-1.04802	-0.64042
Q76M96_S503_1_1	Q76M96	CCDC80	-0.80382	0.612157	-1.93312	0.648745	-2.15262	-1.17119	-1.22232	-0.16835	0.862872	0.675358	0.630245	-1.16173	-0.44862
Q7KZI7_T596_1_1	Q7KZI7	MARK2	0.923002	-0.09047	0.729072	0.129835	-3.59829	-1.14194	1.813734	-0.43643	0.357055	0.433615	-0.30018	-1.08869	-0.3093
Q7L804_S277_1_1	Q7L804	RAB11FIP	1.139379	-0.48912	-0.02466	-0.13694	-1.10045	-1.74915	-0.12893	-0.46402	0.239989	-0.07671	0.258078	0.272103	-1.18247
Q7LBC6_S798_1_1	Q7LBC6	KDM3B	0.905457	-0.51722	0.494477	-0.41189	-1.97488	-1.49264	-0.85173	-0.74017	-0.09864	-0.10567	-0.37641	-1.0648	-0.33398
Q7RTP6_S1310_1_	Q7RTP6	MICAL3	0.428001	0.487378	0.155884	-0.47605	-0.23834	-0.41013	-1.50276	-0.61332	0.022866	-0.07272	0.8026	-0.68399	0.073812
Q7RTP6_S685_1_1	Q7RTP6	MICAL3	-0.15367	-0.64433	0.52639	0.165896	-1.63823	-0.34658	0.501338	-1.20235	-0.52561	-1.02885	-0.06191	-0.5782	-0.26038
Q7RTP6_S977_1_1	Q7RTP6	MICAL3	-0.06201	0.929067	-0.58155	1.088851	0.386086	0.166195	-0.5359	0.011416	1.016404	-0.57235	1.114845	-0.29203	0.79435
Q7Z2W4_S284_1_1	Q7Z2W4	ZC3HAV1	-0.86146	-0.09882	-0.30035	0.334904	-0.28986	-0.44236	-0.07461	-0.33162	0.224315	-0.20575	-0.42686	-0.82109	-0.19606
Q7Z3K3_S425_1_1	Q7Z3K3	POGZ	0.267465	-0.49869	-0.45136	1.112584	0.19078	0.082805	-0.66816	1.397207	-0.04776	0.653926	0.195247	-0.18203	0.038369
Q7Z417_S652_1_1	Q7Z417	NUFIP2	-0.99146	-1.59992	-0.25813	0.991211	-0.05122	-0.4216	-0.423	0.518655	0.742389	0.003817	-0.79644	-0.62001	-0.36883
Q7Z5K2_S221_1_1	Q7Z5K2	WAPL	-0.07662	-0.35043	-0.35953	0.595594	-0.61144	0.250885	0.124481	-0.66139	-0.54113	-0.15881	0.350437	-2.03615	-1.00853
Q7Z5K2_S81_1_0_	Q7Z5K2	WAPL	-0.14679	-0.70658	-0.43675	1.307916	-0.37599	0.530656	0.241493	0.998149	0.355064	0.711043	0.045635	-0.2732	-0.75897
Q7Z5L9_S175_1_1	Q7Z5L9	IRF2BP2	0.005133	-0.8728	-0.78414	1.69921	-0.22183	-0.00123	0.016657	-0.7496	0.285744	-0.46937	-0.08143	-0.17697	-0.41716
Q7Z5L9_S360_1_1	Q7Z5L9	IRF2BP2	0.191086	-0.36709	-0.17587	1.44149	0.223608	0.385495	0.074786	0.179775	0.036176	0.768067	0.162492	-0.31027	0.434812
Q7Z5R6_S525_1_0	Q7Z5R6	APBB1IP	0.509613	1.220771	-0.50562	0.684944	-1.50483	-0.49773	1.123276	-0.26789	0.44341	0.028838	0.170178	-0.58952	0.031972
Q7Z6E9_T984_1_1	Q7Z6E9	RBBP6	0.423917	-0.23154	0.009815	0.312474	-0.64513	0.653189	0.090001	-1.11453	-0.22952	0.770067	0.376922	-0.22753	-0.33117
Q7Z6Z7_S1907_1_	Q7Z6Z7	HUWE1	-0.28651	-0.35234	-0.4071	0.134187	-0.32678	-0.20349	-0.16195	0.032471	-0.08434	0.26659	-0.00312	-0.15135	0.16625
Q86T65_S656_1_1	Q86T65	DAAM2	0.497883	0.032289	-1.62186	0.084925	-0.19582	0.378513	-0.208	-0.06364	-0.83364	0.283505	0.6534	0.112192	-0.39506
Q86TB9_S179_1_1	Q86TB9	PATL1	-0.2735	0.253896	-0.53355	0.533113	-0.11808	0.025197	-0.24604	0.055291	-0.38163	0.289321	-0.05181	-0.73591	-1.14158
Q86UE4_S298_1_1	Q86UE4	MTDH	0.394261	-1.08871	0.042952	0.23321	-2.38272	-2.19512	-0.8993	-1.10703	0.195151	0.124757	-1.47626	-1.50605	-1.36627
Q86UE4_S426_1_1	Q86UE4	MTDH	-0.18035	-0.11805	-0.37453	0.376473	0.150651	0.181142	-0.09524	-0.04281	0.145927	0.169447	0.033066	-0.53261	-0.28127
Q86UE4_S568_1_1	Q86UE4	MTDH	-1.04928	-1.83407	-1.17083	-0.00704	-0.84126	0.835298	2.387721	-0.88791	-0.96808	-0.04958	2.060229	-0.02534	-0.41652
Q86UU1_S430_1_1	Q86UU1	PHLDB1	0.32983	-1.05344	-0.03462	-0.20377	-2.7268	-0.1723	0.815454	1.692671	0.385794	0.278031	1.093219	-0.43252	-0.8384
Q86UU1_S461_1_1	Q86UU1	PHLDB1	-0.83004	-1.5612	0.906808	0.991296	-1.10503	0.318925	1.013217	0.214051	0.488874	0.60953	0.417925	0.053497	-0.13225
Q86UW7_S58_1_1_	Q86UW7	CADPS2	0.132728	0.006245	0.11239	-0.00167	0.058206	0.717366	-0.43404	-0.25146	0.445372	0.312832	-0.34844	-0.66362	-1.53197
Q86V48_S394_1_1	Q86V48	LUZP1	0.067686	-0.16288	-0.64532	-0.11288	-0.29613	-0.04749	-0.15849	-0.25872	-0.12875	0.051612	0.560781	0.365516	0.211923
Q86VM9_S534_1_1	Q86VM9	ZC3H18	0.220621	-0.0333	-0.53083	0.638264	0.029022	-0.39465	0.63252	0.202858	0.226349	0.668959	1.062867	-0.23303	1.096751
Q86VM9_S893_1_1	Q86VM9	ZC3H18	-0.51513	-0.43367	-0.25784	1.024422	-1.56328	-0.05693	0.077766	0.766571	-0.44769	1.393538	0.196936	-1.46646	-0.07769
Q86VR2_S320_1_1	Q86VR2	RETREG3	-0.50422	0.336757	-0.10763	0.710459	-0.01355	0.398504	-0.21482	-0.04304	-0.46763	0.346515	0.796384	-0.32322	0.127047
Q86W92_S601_1_1	Q86W92	PPFIBP1	0.410611	-1.06556	-0.08209	-0.09498	-1.54391	-0.79114	0.227933	0.134536	-0.24846	0.125502	-0.55062	-0.70502	-0.21323
Q86WB0_S321_1_1	Q86WB0	ZC3HC1	0.70118	0.699838	0.006791	0.182831	-0.19474	-0.55765	-1.2346	0.091877	-0.29257	0.56073	1.25287	-0.50019	-0.05648
Q86WB0_S344_1_1	Q86WB0	ZC3HC1	0.563737	0.152865	0.063604	-0.25958	-0.7541	-0.54064	-0.51935	-0.90231	-1.33091	0.342494	-0.75269	-0.77936	-0.72856
Q86YS7_S659_1_1	Q86YS7	C2CD5	-0.16199	-0.50697	0.34828	0.274083	-0.19071	0.730727	1.181169	-0.17661	0.610875	0.237066	0.440345	-0.1414	0.900998
Q86YZ3_S903_1_1	Q86YZ3	HRNR	-0.06693	-0.34005	-0.88796	1.577875	0.066452	-0.05012	0.056472	0.190317	-0.45118	1.050416	-0.14053	-0.26098	-0.07303
Q8IU81_S384_1_1	Q8IU81	IRF2BP1	-0.50573	-0.56462	0.336018	0.4578	-0.12332	0.286736	-0.27209	-0.35533	-0.53625	0.315304	0.409973	-0.46123	-0.28839
Q8IU81_S66_1_1_	Q8IU81	IRF2BP1	0.00773	-0.58123	-0.59475	-0.05667	-1.75862	0.969691	0.920014	-0.93475	0.30898	0.175016	0.388857	0.392212	-1.5392
Q8IUC4_T606_1_1	Q8IUC4	RHPN2	-0.34923	-1.77097	0.053575	-0.99652	-1.43297	-0.51745	0.282866	-1.02853	-0.93231	-0.25993	-0.66079	-0.49631	-0.31666
Q8IVT2_S394_1_1	Q8IVT2	MISP	-0.86382	-1.30043	-0.91344	-0.73331	-0.60942	0.139617	-0.34119	-0.39817	-0.81931	-0.4541	-1.51195	-0.11935	0.435893
Q8IXM2_S96_1_1_	Q8IXM2	BAP18	-0.55208	0.612065	0.164714	0.383686	0.328549	0.216961	-0.49558	0.446306	0.428371	0.188091	0.586613	-0.12392	0.855816
Q8IXT5_S638_1_1	Q8IXT5	RBM12B	-0.8467	-0.48109	-0.40614	1.675725	-0.3504	-0.23704	-0.05758	1.229304	-0.07789	0.827596	0.549434	0.045577	0.100365
Q8IYB3_S389;S393	Q8IYB3	SRRM1	-0.9873	-0.60296	-1.33911	1.750223	0.60507	-0.57302	-0.19369	1.582169	0.339268	0.748489	-0.05665	-0.78724	-0.13863
Q8IYB3_S463_1_1	Q8IYB3	SRRM1	-0.41552	0.021835	-0.1273	0.4485	0.612422	0.045958	0.662225	0.589863	-0.04011	0.666898	-0.14782	-0.64436	-0.85652
Q8IYB3_S597_1_1	Q8IYB3	SRRM1	0.117773	-0.38408	-0.44825	0.761156	-0.18636	-0.1497	-0.36321	0.85474	0.05508	0.417935	0.224382	-0.11003	0.035835
Q8IYB3_S616_1_1	Q8IYB3	SRRM1	-0.10258	-0.58735	-0.57446	1.18289	0.056773	-0.37204	-0.61431	0.956028	-0.5607	0.498771	-0.1674	-0.05634	-0.28859
Q8IYB3_S653_1_1	Q8IYB3	SRRM1	-0.26033	0.079305	-0.30682	0.796097	0.394038	-0.05291	0.268518	0.822662	-0.26712	1.155988	0.354414	-0.17818	0.051897
Q8IYB3_S675_1_1	Q8IYB3	SRRM1	-0.41268	-0.25804	0.283436	0.118169	-0.48726	-0.07365	0.057019	-0.88628	-0.55911	0.520283	-1.83808	-0.6714	-1.37667
Q8IYB3_S696_1_1	Q8IYB3	SRRM1	0.577327	-0.155	-0.61711	0.347271	-0.61574	0.493754	-0.32101	-0.27585	-0.01967	2.470455	0.798391	-0.48531	-0.32992
Q8IYB3_S754;S756	Q8IYB3	SRRM1	-0.21318	-0.30118	-1.33004	1.454569	1.249034	1.466189	-0.50131	1.982617	-0.23441	0.123825	-0.31315	-0.8091	-0.32326
Q8IYB3_S756_1_1	Q8IYB3	SRRM1	-1.05801	-0.38274	-1.78626	1.868562	1.301365	1.316685	-1.50772	1.692259	-1.08368	1.029508	-0.96901	-1.94774	-0.76292
Q8IYB3_S874_1_1	Q8IYB3	SRRM1	0.160533	-0.56162	0.344027	0.695864	-1.87024	0.063399	0.195556	0.439265	0.026182	0.257203	0.674972	-0.05078	-0.02742
Q8IYB3_T220_1_1	Q8IYB3	SRRM1	0.242622	-0.73192	-0.55615	1.0912	0.27671	-0.00085	0.12692	0.338482	0.39767	1.116533	0.470846	-0.09935	-0.0216
Q8IYB3_T872;S874	Q8IYB3	SRRM1	0.072523	0.094548	-0.40106	0.942767	-1.62246	-0.44283	0.348576	-0.75402	0.568522	-0.12786	0.808522	-0.14176	-0.00109
Q8IYB3_T872_1_1	Q8IYB3	SRRM1	-0.04445	-0.12651	-0.59231	0.948224	-0.8179	0.391343	-0.26888	0.732182	-0.03926	0.708497	-0.41567	-0.25806	0.059222
Q8IYI6_S19_1_1_N	Q8IYI6	EXOC8	-0.99565	1.108251	-1.47112	-0.27583	0.101668	-0.59502	-0.65714	0.538833	1.023064	0.210792	0.988155	-0.35254	-0.75714
Q8IZD4_S275_1_1	Q8IZD4	DCP1B	0.334618	-0.22922	-0.10567	-0.5668	-2.09086	-0.54602	0.273121	-1.08236	0.075973	-0.09489	-0.20463	-0.27167	-1.01781
Q8IZL8_S743_1_1	Q8IZL8	PELP1	-0.37704	0.765024	0.1969	0.285	-0.28249	0.155567	0.273979	-0.11862	-0.09035	1.05E-05	0.326388	0.09081	0.4403
Q8IZP0_S222_1_1	Q8IZP0	ABI1	1.35974	-0.99728	-1.32687	0.569269	-1.97064	-2.0435	0.458103	-0.47453	-0.61288	0.242622	0.507096	-0.59489	-0.37548
Q8IZP0_S225_1_1	Q8IZP0	ABI1	0.440085	0.368754	-0.65595	-1.12692	-1.10427	0.875926	0.843322	-0.65271	0.336425	-0.50053	0.264465	0.586873	0.324685
Q8N108_S369_1_1	Q8N108	MIER1	0.122794	-0.22901	-0.99757	-0.29567	-0.51548	0.908207	-0.34498	-0.48658	-0.42354	0.001579	-0.50839	0.03897	0.253725
Q8N1F7_S52_1_1_	Q8N1F7	NUP93	-0.72402	-0.40992	-0.18785	1.315547	0.07591	0.047622	-0.66325	0.21389	-0.10747	0.943316	-1.37844	-0.24952	0.258377
Q8N1G4_S520_1_1	Q8N1G4	LRRC47	0.233952	-0.27004	-0.49364	0.797777	0.884809	0.642899	-0.3185	-0.10677	0.418878	0.253679	-0.36449	-0.35738	-0.18542
Q8N2U9_S110_1_1	Q8N2U9	PQLC1	0.398133	0.794703	-1.55184	-0.94096	-0.717	-0.39196	0.459651	1.344497	0.857817	0.035182	-0.76925	-2.38862	-0.49496
Q8N3V7_T461_1_1	Q8N3V7	SYNPO	0.349187	-0.18683	0.436042	-0.58281	-0.81563	-1.16072	-1.65202	-0.38425	-0.43957	-0.23547	-0.85175	-1.01757	-0.39632
Q8N3X1_S499_1_1	Q8N3X1	FNBP4	0.476784	0.2605	-0.8742	1.483072	-0.11396	0.660668	0.75019	0.421024	0.062725	-0.67408	-0.77513	-0.48619	-1.66335
Q8N556_S668_1_1	Q8N556	AFAP1	-0.16816	-0.88428	-0.2109	-0.06854	-1.27848	1.368407	0.407746	-0.89066	0.524326	0.151259	-0.06924	0.123859	0.689789
Q8N573_S201_1_1	Q8N573	OXR1	-0.28414	-0.02172	0.684607	0.081471	-1.20233	-1.2828	-0.85048	0.231402	0.545865	0.345938	0.12977	-0.10639	-0.35229
Q8N9B5_S115_1_1	Q8N9B5	JMY	-0.01813	-0.82086	-0.13784	0.916344	-0.90595	0.220531	1.030684	0.292938	-1.214	0.276139	0.949731	1.780934	0.687813
Q8N9T8_S171_1_1	Q8N9T8	KRI1	-0.35379	-0.50527	-0.31359	0.915477	-0.46339	0.105536	0.354171	0.715846	0.325976	1.222418	-0.12397	0.038975	0.308986
Q8NBJ4_T255_1_1	Q8NBJ4	GOLM1	-0.57851	-0.32856	-0.21882	1.364839	-0.688	-2.00553	0.047933	1.28296	-0.23817	0.73799	2.490905	-0.55658	-1.23971
Q8ND76_S25_1_1_	Q8ND76	CCNY	1.09456	0.784761	-0.68987	-0.22121	-2.18725	-0.42861	0.951621	0.13223	0.851452	-0.15001	0.19829	-0.76037	-0.37112
Q8ND76_S326_1_1	Q8ND76	CCNY	0.133151	-0.33301	-0.37993	0.759382	0.179414	0.061615	-0.15692	0.235779	-0.18823	-0.58464	-0.32802	-0.14435	-0.19527
Q8NDI1_S1035_1_	Q8NDI1	EHBP1	1.044741	0.347009	-0.17685	-0.15635	-0.78562	-0.56692	-0.07939	-1.02945	0.152975	-0.31446	0.133901	-0.09354	-0.49149
Q8NDI1_S1058_1_	Q8NDI1	EHBP1	-0.27042	-1.06075	-0.6409	0.046057	-0.94308	-0.94137	-1.48239	0.182268	-0.49214	-0.91112	-0.86613	-1.24703	-0.55923
Q8NDT2_S267_1_1	Q8NDT2	RBM15B	-0.80152	-0.68044	-0.14955	0.873333	0.615636	0.156671	-0.80924	0.177468	-0.38933	0.8801	-1.83876	-0.50606	-0.23726
Q8NDT2_S562_1_1	Q8NDT2	RBM15B	0.138864	-0.407	-0.06842	0.544505	-0.07417	-0.31004	-0.13478	-0.23519	0.070516	0.183146	-0.9191	-0.13149	-0.63668
Q8NDT2_T532_1_1	Q8NDT2	RBM15B	0.329412	-0.2083	-0.32996	-0.26685	-0.00764	-0.07631	0.532707	-0.69159	0.232671	1.218589	0.301593	-0.18781	-0.03851
Q8NE71_S228_1_1	Q8NE71	ABCF1	-0.6715	-0.25243	-0.18274	0.301852	-0.21706	0.383773	-0.34058	-0.33744	0.510966	-0.54975	-0.71216	0.39707	-0.00778
Q8NEY8_S121_1_1	Q8NEY8	PPHLN1	0.130524	-0.43185	-0.17677	-0.11475	0.050327	-0.12511	0.234018	-0.59206	-0.51927	0.30061	-0.03268	0.21888	-0.27751
Q8NEY8_S133_1_1	Q8NEY8	PPHLN1	-0.75241	-0.86572	-0.15735	0.861942	-0.33278	-0.14528	-0.21492	-0.50448	0.255694	0.607479	0.686249	-0.00313	0.326003
Q8NFC6_S538_1_1	Q8NFC6	BOD1L1	-0.1842	-0.49171	-0.28933	1.875646	-0.06834	-1.13566	-0.56558	0.778056	-0.23312	1.049102	-0.95661	-0.17929	-0.2972
Q8NHM5_S474_1_1	Q8NHM5	KDM2B	-0.07468	-0.12409	0.307739	-0.73635	0.468875	-0.65512	-2.00468	-0.56787	-0.09137	0.775134	0.663335	-0.36181	0.110127
Q8NI35_S645_1_1	Q8NI35	PATJ	0.240282	-0.35805	-0.1766	0.049462	-1.5967	-0.34815	-0.23623	1.305128	-0.33141	-0.01742	0.209158	0.115849	0.122262
Q8TAQ2_S347_1_1	Q8TAQ2	SMARCC2	-0.85572	0.01653	-0.82445	0.233833	-1.02287	-0.20084	-0.14282	-0.0394	0.025611	0.089667	-1.61179	-1.03798	-0.72339
Q8TB72_S136_1_1	Q8TB72	PUM2	0.565224	0.316241	-0.17119	-0.48806	-0.51737	0.204274	0.071391	0.010273	0.086088	-0.22776	-0.0138	-0.69737	-0.86229
Q8TB72_S182_1_1	Q8TB72	PUM2	0.142208	0.979996	-0.24119	-0.77351	-0.11138	0.797127	0.33939	-0.10615	-0.14059	-0.12219	-0.43382	-1.04527	-0.3811

Q8TB72_S587_1_1	Q8TB72	PUM2	1.05678	0.529688	0.333932	-0.95008	-1.52857	-0.05952	0.327867	-0.65269	0.616581	-0.36086	-0.53467	-2.14173	0.852979
Q8TDB6_S202_1_1	Q8TDB6	DTX3L	0.140205	-0.45469	-0.05551	-0.05741	-1.99067	-0.21775	-0.63814	-0.60661	-0.23559	1.214482	-0.69912	-0.51214	-0.64309
Q8TDY2_S222_1_1	Q8TDY2	RB1CC1	1.106709	0.468126	0.252888	0.200135	-0.96686	-0.50257	-1.10483	-0.08487	0.302111	-0.04763	-0.16742	-0.4166	-0.64494
Q8TF01_S290_1_1	Q8TF01	PNISR	-0.59531	-0.31874	0.326796	2.294334	0.521152	-0.28505	-1.19654	0.632771	-0.28176	2.344039	-1.11881	-0.55459	-0.12522
Q8WV41_S169_1_1	Q8WV41	SNX33	-0.61932	0.543777	-0.40065	0.122917	-1.00863	-0.24222	0.209769	-0.56869	0.193994	0.14468	-0.70673	-0.07477	0.900724
Q8WVC0_S205_1_	Q8WVC0	LEO1	0.064458	0.120198	-0.57758	0.248328	-0.08122	0.085407	-0.12621	1.198194	0.937377	-0.30765	-0.02058	-0.46316	-1.48329
Q8WVC0_S630_1_	Q8WVC0	LEO1	0.384206	0.363118	-0.50312	0.540947	-0.40304	-0.06209	-0.35651	0.439726	0.161169	-0.1754	0.777482	-0.56497	-0.20218
Q8WVC0_S658_1_	Q8WVC0	LEO1	-0.54524	-0.5725	-0.61365	0.413678	-0.70169	-0.31982	0.62476	0.767193	0.154929	-0.03222	0.544172	0.188917	0.182095
Q8WVC0_T629_1_(	Q8WVC0	LEO1	-0.05234	-0.17376	-0.47776	0.497428	-0.38205	0.381869	-0.19048	-0.24505	-0.28433	0.627926	0.123394	-0.38871	-0.30687
Q8WVT3_S184_1_	Q8WVT3	TRAPPC1:	0.383428	0.053953	0.227125	0.176054	-2.04315	-1.56991	-1.95783	-0.76705	-0.39142	-1.02294	-1.51014	-1.69635	-0.73188
Q8WWI1_S1510_1	Q8WWI1	LMO7	0.42844	0.031272	-0.6536	1.704661	-0.642	-0.84592	0.14622	0.233474	0.09273	0.854655	-1.02585	-1.70318	0.161397
Q8WWI1_S246_1_1	Q8WWI1	LMO7	-0.57412	0.553419	-0.3821	1.352117	0.035705	0.261676	1.343512	-0.22434	-0.70462	1.010457	-0.25242	-0.2157	0.677917
Q8WWI1_S276_1_1	Q8WWI1	LMO7	-1.00201	-0.69041	-0.67655	0.926471	0.448049	0.468467	0.371183	-1.46299	0.787478	0.465947	0.362299	0.92442	0.652268
Q8WWI1_S805_1_1	Q8WWI1	LMO7	0.515428	-1.28067	-1.58926	0.342745	-1.72103	-0.72415	0.191	-0.11972	0.147155	0.493895	-0.37715	-1.38163	0.806512
Q8WWQ0_S1783_1	Q8WWQ0	PHIP	-0.48601	0.188874	-0.10013	-0.44119	-0.06978	0.077051	-0.18067	-0.04721	-0.20626	-0.6565	0.245878	-0.12148	-0.23201
Q8WX93_S893_1_1	Q8WX93	PALLD	-0.86009	0.098982	-1.33011	-0.5252	-1.04931	-1.69845	-0.86891	-1.57484	-0.74464	-0.076	-1.24993	-2.07273	-1.02008
Q8WXE1_S518_1_	Q8WXE1	ATRIP	0.423008	-0.33953	-0.40788	-0.19815	1.518178	-0.37706	-0.05199	-0.12084	0.214729	0.402934	-0.45426	-1.07205	1.808643
Q8WXH0_S4110_1	Q8WXH0	SYNE2	0.05415	-0.66192	-0.48875	1.170802	-0.79359	0.537564	-0.65399	-0.12639	0.410152	0.635104	0.03263	-0.01699	0.004387
Q8WYA6_S545_1_	Q8WYA6	CTNNBL1	0.349984	-0.22388	-0.28929	1.301732	0.641726	0.039433	0.294848	0.83293	-0.34075	1.229477	-0.04435	-0.31944	0.241761
Q92466_S26_1_1_N	Q92466	DDB2	-0.83847	-1.60337	-0.49589	0.001071	-0.60272	0.262565	-0.21729	-0.13132	-0.91246	0.228456	-0.00294	0.080351	-0.08617
Q92508_S1646_1_1	Q92508	PIEZO1	-0.13051	0.462948	-0.73868	0.333959	-1.08875	0.080563	0.287432	-0.39484	0.25943	0.097151	-0.15176	-0.7346	-0.9044
Q92597_S330_1_1	Q92597	NDRG1	0.707478	-0.81171	-0.17047	-0.09404	-0.67611	0.053975	0.431524	-0.07663	-0.806	0.952483	1.135272	0.002402	-0.16302
Q92597_T328_1_1	Q92597	NDRG1	0.62957	0.775223	-0.699	-0.68782	-0.68719	0.163983	0.241616	0.196665	1.370917	2.132464	-0.26941	-0.14618	0.12347
Q92608_S1685_1_1	Q92608	DOCK2	0.616279	0.15772	0.419758	1.07958	-0.3772	-1.32486	0.684883	-0.05064	0.156212	0.363905	-0.33162	1.020058	0.77591
Q92608_S1706_1_1	Q92608	DOCK2	-0.08615	0.922253	-0.33394	-0.02613	-0.34689	0.797284	0.401944	-1.30478	0.374305	0.198929	0.073188	-0.20255	0.630646
Q92614_S1998_1_1	Q92614	MYO18A	-0.57927	0.230003	-1.24827	-0.06083	0.603866	0.850015	-1.07508	0.059023	-0.43517	-0.38593	-0.24011	-0.17535	0.312718
Q92614_S2002_1_1	Q92614	MYO18A	-0.8077	-0.5511	-0.72125	-0.80434	0.931971	0.504603	-0.92189	-0.33222	-1.22036	0.637157	-0.10209	-0.31328	-0.32495
Q92615_S601_1_1	Q92615	LARP4B	0.520848	0.650209	-0.44363	0.485344	-0.58779	-0.01131	-0.30403	-0.08853	0.046388	-0.06386	0.258113	-0.23374	-0.29614
Q92625_S663_1_1	Q92625	ANKS1A	0.330487	0.136074	0.228174	-0.54297	-0.61783	0.117169	-0.1804	-0.47014	0.395834	-0.20964	0.204902	-0.82763	0.583969
Q92688_T244_1_1	Q92688	ANP32B	-0.42212	-0.03999	-0.8105	0.570022	-1.54581	0.528174	0.104754	-0.30468	0.497036	-0.00364	0.831537	-0.05767	-0.194
Q92733_S267_1_1	Q92733	PRCC	-0.76352	-0.6468	-1.09989	1.376806	0.245255	0.477323	-0.09767	0.372322	0.241199	0.329549	0.448989	-0.45345	-0.82108
Q92785_S142_1_1	Q92785	DPF2	0.360801	-2.14685	-0.76394	0.645387	-1.46212	-0.6848	0.189784	-0.41196	0.551284	-0.91498	-1.57321	-0.6631	-1.36936
Q92817_S2025_1_1	Q92817	EVPL	-1.02749	-0.55283	-0.53023	0.993174	0.148636	0.131878	-0.02969	-0.55637	-0.06205	0.764128	-0.08502	-1.44367	0.214294
Q92841_S599_1_1	Q92841	DDX17	0.989992	-0.59288	-0.05764	-0.28737	-0.81149	-0.89184	-0.22141	-0.39388	0.093467	-0.19017	-0.43607	-2.59485	0.467326
Q92882_S213_1_1	Q92882	OSTF1	-1.1717	-1.06926	-1.52449	0.206222	0.563347	0.693458	-0.56449	0.036153	0.076985	-0.44167	0.018934	-0.12963	0.152867
Q92934_S118_1_1	Q92934	BAD	0.196631	-0.1298	-0.14551	1.278535	0.178773	-0.54557	-1.97703	1.217591	-1.45485	0.721721	-1.56276	-0.3025	-0.29566
Q92945_S181_1_1	Q92945	KHSRP	-0.86916	-0.5202	-0.62786	0.461947	-0.16184	0.051531	1.174102	0.454847	0.15013	0.706842	0.008183	-0.39059	-0.73171
Q92974_S956;S960	Q92974	ARHGEF2	0.406715	0.076549	0.096229	-0.3613	-0.71844	0.021672	0.332956	-0.42867	0.525787	-0.05463	0.394386	-0.32409	-0.47471
Q92974_S956_1_1	Q92974	ARHGEF2	-1.57258	-0.27403	-1.43482	0.26621	-0.18728	0.700097	-0.0573	0.766681	1.606248	1.017349	-0.16401	-0.20125	-0.01925
Q969X1_S81_1_1_N	Q969X1	TMBIM1	0.619335	-0.32633	-0.92979	-0.71014	-0.53147	-0.19726	-0.26278	0.269034	-1.1992	-0.85342	0.523296	-0.43712	-0.57671
Q96A49_T248_1_1	Q96A49	SYAP1	-0.21654	-0.35207	-0.31281	0.103009	0.156117	-0.24988	-0.60261	0.240918	-0.34662	0.65726	0.167152	-0.16757	0.348763
Q96AE4_S99_1_1_	Q96AE4	FUBP1	0.792227	-1.61496	0.145379	-0.32895	-0.42503	-0.87306	-1.20612	-0.10855	-1.73697	-0.67799	-2.40619	-2.85116	-0.7527
Q96B36_T246_1_1	Q96B36	AKT1S1	-0.19796	0.812366	1.044499	0.549286	-1.19162	0.78899	0.763076	-0.05171	1.552723	0.723487	0.214585	-0.2283	0.383548
Q96CV9_S526_1_1	Q96CV9	OPTN	0.339226	0.2449	0.20196	0.203458	-2.20278	-1.12689	-0.75746	-0.83875	-0.12259	-0.06622	-0.34543	-1.17049	-1.00456
Q96CW1_T156_1_1	Q96CW1	AP2M1	-0.93499	-0.73901	0.136962	0.533691	-1.20449	0.043993	-0.20922	-0.81348	0.036846	0.257128	0.302735	0.1469	-0.05942
Q96CX2_S176_1_1	Q96CX2	KCTD12	-0.0905	-0.10152	0.139085	1.317946	-0.32583	-0.54848	-0.14959	2.152569	-0.69009	1.154567	-0.26191	-0.48302	-0.17941
Q96CX2_S185_1_1	Q96CX2	KCTD12	-0.40146	0.571525	-0.13926	1.044353	-1.16345	0.025473	0.502065	1.691608	0.244932	1.227104	0.069046	0.390916	0.15195
Q96DF8_S292_1_1	Q96DF8	ESS2	-0.29181	-1.50666	-0.96942	0.297698	0.615004	-2.73424	0.002615	0.994357	0.742847	-0.08813	0.006618	0.558971	-0.66116
Q96E39_S208_1_1	Q96E39	RBMXL1	0.49353	0.888811	-2.94812	1.05343	0.357692	1.720199	-0.18926	-0.18667	0.917426	0.053609	0.271356	-0.33768	0.252457
Q96GP6_S653_1_1	Q96GP6	SCARF2	-0.52375	1.616375	-0.26376	0.352292	-0.63437	-0.06923	0.06432	-0.59295	-0.0933	-0.76301	-1.24389	0.459827	-0.57002
Q96HC4_S228_1_1	Q96HC4	PDLIM5	-0.23058	-0.27311	-0.65486	-0.17393	-0.75487	-0.74823	0.470882	-0.75782	-0.33596	-0.10001	-0.90552	-0.68388	-0.23963
Q96HP0_S159_1_1	Q96HP0	DOCK6	-0.0646	0.297688	-0.68241	0.579695	-1.02278	0.526939	0.407236	0.618613	0.49548	0.421731	0.363111	0.42294	-0.62782
Q96I24_T76_1_1_N	Q96I24	FUBP3	1.250834	1.24767	-2.0284	-0.57594	0.428927	-0.98607	0.337041	0.35837	0.308987	-0.80116	-1.67675	-0.65163	0.204169
Q96JG6_S561_1_0	Q96JG6	VPS50	-0.27431	-0.21887	-0.43541	0.113385	0.170266	0.748248	0.037073	0.04157	-0.51132	-0.33818	0.002398	-0.48806	-0.94087
Q96JH7_S747_1_1	Q96JH7	VCPIP1	0.007557	-0.23215	-0.01507	-0.44393	0.696521	0.440251	0.146042	-0.31745	0.774616	0.42722	0.198029	0.436948	0.886359
Q96JM3_S416_1_1	Q96JM3	CHAMP1	-0.32743	-1.12645	-0.19819	-0.46623	-0.2023	0.152107	-0.64005	0.081173	0.68324	0.033109	0.427366	-0.28487	0.439108
Q96JM3_S436_1_1	Q96JM3	CHAMP1	-1.00981	0.40324	-1.96928	0.357447	1.520144	-0.09107	-1.08269	0.211456	-0.55936	0.394358	-0.11874	-1.86284	1.329081
Q96JM3_S476_1_1	Q96JM3	CHAMP1	-0.32432	-0.57644	-0.53697	0.224633	-0.89828	-0.06691	-0.56623	0.076784	0.254021	0.336985	-0.21053	0.33082	0.399239
Q96JM3_S542_1_1	Q96JM3	CHAMP1	0.033994	-0.44875	-0.24582	0.268684	-1.05114	-0.11321	-0.32324	-0.11466	-1.15394	0.142996	-0.44958	-0.74838	-0.19641
Q96JY6_S129_1_1	Q96JY6	PDLIM2	0.471902	-0.51459	0.020207	0.038531	-0.10638	-0.22298	-1.22977	0.128058	-0.68909	0.244035	-0.21486	0.253308	0.722929
Q96KR1_S1054_1_	Q96KR1	ZFR	-0.29036	-0.28906	0.139207	0.568016	-0.16368	-0.73756	-0.74891	0.454312	-0.6987	0.383304	-0.45249	-0.31149	0.122717
Q96MU7_S146;T14	Q96MU7	YTHDC1	-0.39596	-0.4944	-0.53244	0.010824	-0.46759	0.006838	-0.13967	0.51073	-0.07626	0.437575	-0.07236	-0.46333	-0.36358
Q96MU7_S308_1_1	Q96MU7	YTHDC1	-0.1444	0.166934	-0.49833	-0.31759	0.70387	0.314179	-0.34547	0.8131	-0.85977	0.023559	-0.3557	-0.86138	-0.73957
Q96MY1_S130_1_1	Q96MY1	NOL4L	0.284756	0.167887	-0.19682	1.995812	-0.14207	0.68374	-0.54058	-0.0538	-1.06226	0.515447	-0.05854	-0.17692	0.010061
Q96PE2_S420_1_1	Q96PE2	ARHGEF1	0.250966	-0.1459	-0.13763	0.312573	-1.12006	-0.52918	0.780706	-1.4673	-0.08258	0.35727	-0.86782	-0.77697	-0.03872
Q96PK6_S618_1_1	Q96PK6	RBM14	0.618953	-0.66469	-0.22166	0.728195	-1.45838	-0.37846	-0.17087	-0.67741	-0.66926	0.887322	-0.79062	-0.59361	-0.36723
Q96PK6_T206_1_1	Q96PK6	RBM14	0.012168	-0.08217	-0.32963	0.797515	0.402344	0.367148	-0.07668	0.139277	-0.44518	0.825023	-0.22265	-0.20079	0.004969
Q96PV6_S314_1_1	Q96PV6	LENG8	-1.05902	0.113578	-0.01237	0.479287	0.815096	-0.1546	-1.17759	0.208951	-1.07094	0.578087	-0.18189	-0.04774	1.231161
Q96RL1_S677_1_1	Q96RL1	UIMC1	-1.16674	-1.39324	-0.23191	0.891287	-0.05693	-0.22101	0.821397	0.08863	0.310876	-0.57759	0.596133	-0.30129	1.305959
Q96RU3_S296_1_1	Q96RU3	FNBP1	0.109094	-0.08881	-0.70728	0.522407	0.003642	-0.59023	-0.42303	-0.29569	0.107274	0.588758	-0.08753	-0.52421	0.101896
Q96S55_S75_1_1_I	Q96S55	WRNIP1	0.23037	-0.59423	-0.07	-0.64803	-0.9312	-0.22656	0.345558	-0.71392	-0.00037	-0.53032	-0.2714	-0.95516	0.330401
Q96ST2_S300_1_1	Q96ST2	IWS1	-0.30235	0.070183	-0.84679	0.784242	1.951561	0.378108	-1.20933	1.250143	-1.04728	0.035514	-2.30548	0.557864	-0.48607
Q96ST2_S400_1_1	Q96ST2	IWS1	-0.8244	-1.64259	-0.29758	-0.65788	1.111903	-1.89554	-0.18131	-0.5073	-1.5447	-0.83242	-1.55968	-1.98038	-0.67138
Q96ST2_S440_1_1	Q96ST2	IWS1	-0.8447	-2.05709	-0.7995	1.427568	1.269511	-1.15645	-2.98911	1.530373	-1.65832	0.680959	-1.08552	-1.20018	-1.64684
Q96ST3_T1111_1_(	Q96ST3	SIN3A	-0.11276	-1.3918	-0.11349	0.239624	-0.13514	-0.56641	-0.93904	-0.01492	-0.04828	-0.16414	0.356515	-0.48416	0.025512
Q96T23_S1345_1_	Q96T23	RSF1	-0.20744	-0.52939	-0.4429	0.539235	-1.16847	0.295403	0.231693	0.134515	-0.00868	0.459478	0.052132	-0.63934	0.059024
Q96T23_S604_1_1	Q96T23	RSF1	-0.2591	-0.48115	-0.56	0.330248	0.057232	0.018697	0.204689	0.379901	0.104849	0.317836	-0.20173	-0.48137	0.016949
Q96T37_S294_1_1	Q96T37	RBM15	-0.14916	-0.89508	-0.62169	0.066558	-0.00838	0.458518	0.685316	0.061514	0.188732	0.014603	-0.12686	-0.15933	-0.26606
Q96T37_T568_1_1	Q96T37	RBM15	-0.5885	-0.68428	-0.34932	0.354194	-0.01275	0.140667	-0.04661	-0.17403	0.530461	-0.04408	-0.33557	-0.19051	-0.13649
Q96T60_T118_1_1	Q96T60	PNKP	-0.1285	-0.09085	-0.85858	0.847106	-0.18026	0.482244	0.277471	0.595366	-0.47079	0.581329	-0.12573	-1.40433	-0.80074
Q96TC7_S46_1_1_I	Q96TC7	RMDN3	-0.54929	-0.20154	-0.64835	0.770542	-0.77243	-0.66454	0.696908	0.47975	-1.11162	0.39781	-0.30513	-0.09388	0.18807
Q99442_S335_1_1	Q99442	SEC62	0.013266	-0.11235	-0.5331	0.234535	-0.49826	1.816772	-0.73357	-0.01716	-0.86481	-0.06098	-0.74268	-0.46444	-0.18407
Q99442_T158_1_1	Q99442	SEC62	0.118297	-0.56511	-0.55261	-1.40878	0.337291	1.097057	0.608794	-1.37178	-0.44183	-0.8486	0.490019	-0.05334	0.232091
Q99460_T273_1_1	Q99460	PSMD1	-0.73772	-0.74184	-0.68685	0.010412	-0.40769	-0.88315	-1.01953	1.780976	-0.07496	0.250653	-1.5583	-0.57539	-0.46079
Q99543_S47_1_1_N	Q99543	DNAJC2	0.241057	-1.63365	0.123985	0.901549	0.758822	0.549916	-0.47788	-0.93442	0.340987	0.494976	-0.10911	-1.03782	-1.89292
Q99549_S51_1_1_N	Q99549	MPHOSPH	-0.34457	0.216063	-1.02461	0.653929	0.260999	0.590679	0.360978	0.182201	-0.10271	0.037878	0.296173	-0.48626	-0.42822
Q99569_S314_1_1	Q99569	PKP4	0.954111	-0.31409	-0.02972	0.406685	-2.1241	0.566182	-0.64017	0.243848	0.158339	0.72375	0.389916	0.959818	-0.53035
Q99590_S608_1_1	Q99590	SCAF11	0.172782	-0.25293	-0.65634	1.185905	1.130318	0.046105	0.071369	0.016766	-0.08116	0.648159	-0.37626	-0.43628	0.302078
Q99700_T741_1_1	Q99700	ATXN2	0.035503	-0.30973	0.565656	-0.6003	-1.14188	0.651267	0.55974	-1.15941	1.134214	-0.29137	-0.56769	0.098713	-0.51234
Q99733_S125_1_1	Q99733	NAP1L4	-0.03018	-0.65415	-0.72802	0.976038	-0.08487	0.107029	0.233639	0.949451	0.592578	1.088213	0.232354	-0.19461	-0.09556
Q99759_S176_1_1	Q99759	MAP3K3	0.260854	0.345286	-0.48218	-0.13207	-0.51969	0.929795	0.419772	-0.39422	0.259987	1.324147	0.852896	-0.13076	-0.23448
Q99959_S135_1_1	Q99959	PKP2	-0.78368	-2.37362	-1.02192	0.016503	-2.22099	-1.85877	-1.66541	-0.15673	0.670092	0.177114	-0.11295	-2.58231	-0.71745
Q99959_S151;S155	Q99959	PKP2	0.607505	0.93134	-0.43829	0.946006	0.086627	1.204331	0.239791	-0.5645	-0.17729	1.832147	0.431178	-0.59269	-0.13221

Q99959_S151_1_1_	Q99959	PKP2	0.212137	-0.49723	-0.75846	0.654967	-0.27619	0.753444	-1.27266	-0.54044	0.297882	0.824053	-0.81928	0.664117	-0.59243
Q99959_S155_1_1_	Q99959	PKP2	-1.18586	0.631135	-0.63785	2.308502	0.56441	-0.9527	-1.21806	1.640531	0.205064	1.279094	-1.2124	-1.80206	0.463413
Q99959_S197_1_1_	Q99959	PKP2	-0.32986	-1.16464	0.292935	1.838642	-0.16577	-0.86941	-0.504	-0.19861	0.568886	0.645082	-0.42822	-1.40252	-0.6185
Q99959_S251_1_1_	Q99959	PKP2	-0.33223	-2.18064	0.178297	-0.55319	-1.82671	0.168336	-0.93949	-1.35997	-0.02882	-0.88086	-1.56028	-1.26286	-2.0654
Q9BQE3_S158_1_1	Q9BQE3	TUBA1C	-1.90558	-0.85827	-1.4647	0.946121	0.302548	-1.89539	-0.9022	1.101705	-0.63682	1.464375	-0.8373	-2.03828	0.777139
Q9BRD0_S271_1_1	Q9BRD0	BUD13	-0.68548	-0.64837	-0.45213	1.270802	0.424055	-0.47712	-1.45586	0.959874	-0.55912	1.157791	0.741908	-0.19941	0.061178
Q9BRK4_S570_1_1	Q9BRK4	LZTS2	0.767125	-0.81442	-0.74301	0.487984	-0.13774	0.372496	-0.2204	0.094082	-0.00754	0.649005	0.190707	0.564043	-0.13268
Q9BRQ6_S13_1_1_	Q9BRQ6	CHCHD6	-2.19138	0.042343	-0.55623	2.927846	-0.02785	1.213259	0.6212	1.606903	0.255317	1.03593	-1.19122	-0.50511	-0.01974
Q9BSJ8_S1034_1_	Q9BSJ8	ESYT1	-1.02261	-0.50456	-0.70952	1.621825	0.395163	-0.1527	-1.07376	2.091929	0.002773	1.274499	0.023032	-0.03079	-0.18754
Q9BSQ5_S384_1_1	Q9BSQ5	CCM2	-0.39242	-0.03331	-0.05556	-0.36052	-2.33956	-0.32869	-0.29383	-0.13594	0.488812	-0.50348	-0.08438	0.369852	-1.76837
Q9BTA9_S64_1_1_	Q9BTA9	WAC	-0.16364	0.645532	0.024468	1.280149	0.124062	-0.03093	0.046522	0.883876	0.930977	-0.28547	-0.33654	-0.10746	-0.11478
Q9BTU6_S462_1_1	Q9BTU6	PI4K2A	-0.82668	-0.43166	-0.515	0.165868	-0.8979	-1.50345	-0.9926	-0.40112	-1.19037	0.127373	-0.14055	-1.50333	-0.27035
Q9BUH6_S148_1_1	Q9BUH6	PAXX	0.15003	-0.81254	0.51099	-0.32858	-0.1813	-0.33272	-0.31357	0.412193	-0.32256	-0.52349	-0.87749	0.31453	0.368045
Q9BUR4_S54_1_1_	Q9BUR4	WRAP53	-0.05241	-1.13548	0.087233	0.354852	-1.15877	0.348616	-1.12427	-0.45478	-0.13594	0.173449	-0.32252	0.023796	0.287535
Q9BW71_S227_1_1	Q9BW71	HIRIP3	-0.19844	-1.35375	0.407568	1.552012	-0.12052	0.413102	-0.50402	0.077795	-0.79347	0.902499	-0.31665	-0.46946	0.474666
Q9BX66_S465_1_1	Q9BX66	SORBS1	-0.39956	-0.36816	-0.9523	-0.89307	-1.74315	0.396245	0.393294	-0.15708	0.619383	0.271943	-0.80501	-0.35642	-1.41961
Q9BX66_S556_1_1	Q9BX66	SORBS1	-2.34094	-0.65482	-1.53311	0.848171	-0.61193	-1.13076	-1.30117	-1.22472	0.43903	0.739331	0.252605	-0.45125	-1.36497
Q9BXK5_S370_1_0	Q9BXK5	BCL2L13	-0.40549	-0.40861	0.723008	0.877705	-0.61827	0.294414	-0.93352	1.739262	-1.62147	0.903302	0.223146	-0.47854	0.749744
Q9BXK5_S371_1_1	Q9BXK5	BCL2L13	-1.45662	-0.89062	0.238524	0.901095	-0.64762	-0.00291	-0.29544	0.578465	-0.77084	0.642736	0.482583	-0.43371	-0.06409
Q9BXK5_S420_1_1	Q9BXK5	BCL2L13	-0.01735	0.37224	0.689134	0.620331	-0.23614	-1.00465	-0.00238	0.40593	-0.77917	0.196757	-0.37202	-0.62326	-0.66281
Q9BXK5_S426_1_1	Q9BXK5	BCL2L13	-0.74044	-1.44823	-0.65077	1.048029	0.461575	-0.7681	-2.07687	0.982653	0.375101	0.775319	0.034801	0.248224	0.099826
Q9BXP5_S74_1_1_	Q9BXP5	SRRT	0.616464	0.727779	-0.50864	-0.23024	0.012106	0.030108	0.162971	0.001214	0.594639	0.068927	0.099275	-0.25795	-0.50125
Q9BY44_S506_1_1	Q9BY44	EIF2A	0.085634	0.103821	-0.04952	-1.47937	-1.36894	0.148089	0.064427	-0.16225	0.366863	-0.14324	0.160526	0.400288	0.275743
Q9BY89_S1701_1_	Q9BY89	KIAA1671	-0.87453	-0.5516	0.047647	-0.02094	0.250869	-0.38972	1.898586	-0.38266	0.824077	-0.51501	-0.357	-1.26365	0.24726
Q9BYX4_S301_1_1	Q9BYX4	IFIH1	-1.76094	-1.82146	-0.24129	1.939569	-0.28636	-0.54844	-0.18538	-0.62732	-0.84193	0.270609	0.18441	-0.18859	1.072416
Q9BZ29_S927_1_1	Q9BZ29	DOCK9	-0.13359	-1.78373	-0.78704	0.756711	-0.1048	-0.70232	-0.57328	0.28777	-0.18395	0.105228	-0.09818	0.27597	-0.39583
Q9BZ67_S408_1_1	Q9BZ67	FRMD8	0.451108	0.893689	-0.17966	-0.31206	-0.11852	-1.08244	-0.50532	0.203734	0.666959	0.336399	0.297039	0.222832	0.251178
Q9BZZ5_S464_1_1	Q9BZZ5	API5	-0.8976	0.232582	-0.28668	1.05204	0.829691	0.032664	-0.78007	0.733319	-0.23913	0.690002	-0.96682	0.130245	0.621441
Q9C0B5_S380_1_1	Q9C0B5	ZDHHC5	0.253941	-0.00174	-0.34432	-0.22744	-1.16585	0.138905	0.568281	-0.26979	0.057599	-0.53416	0.894728	-0.1247	-0.3488
Q9C0C2_S1138_1_	Q9C0C2	TNKS1BP	-0.54737	0.68965	-0.09392	-0.38332	-0.34291	-1.06081	-1.42108	-0.0013	-0.4984	-0.21018	-0.23006	-0.43009	-0.45065
Q9C0C2_S1545_1_	Q9C0C2	TNKS1BP	0.097932	0.217822	-0.3866	0.227448	-0.51742	-0.06672	0.798424	0.059666	0.092556	2.418113	-0.25704	0.096768	-0.02295
Q9C0C2_S1554_1_	Q9C0C2	TNKS1BP	0.436652	0.514549	-1.05784	0.333148	0.010243	-0.01638	-0.28862	0.291613	-0.19255	0.159951	-1.22225	0.289502	-0.14391
Q9C0C2_S1666_1_	Q9C0C2	TNKS1BP	-0.59223	-1.08602	0.549672	0.799651	-1.04691	-1.19108	-0.61548	0.555122	-1.1172	-0.41041	-0.02108	-0.65063	-0.09029
Q9C0C2_S221_1_1	Q9C0C2	TNKS1BP	0.394986	0.329366	-0.0915	0.973486	-1.4111	-0.94947	-0.33385	-0.31089	-0.16608	0.512704	-0.21662	-1.66772	-0.24344
Q9C0C2_S429_1_1	Q9C0C2	TNKS1BP	0.933984	-0.83323	0.467284	1.258792	-0.79516	0.483678	0.707236	-0.57889	0.309122	-0.70144	-0.51747	-0.15592	0.557749
Q9C0C2_S494_1_1	Q9C0C2	TNKS1BP	-0.45013	-0.62313	-0.73011	1.650143	0.926306	0.921666	-0.28358	2.477118	-0.57482	1.093198	-0.28564	-0.84389	-0.42351
Q9C0C2_S601_1_1	Q9C0C2	TNKS1BP	0.805118	-0.15178	-0.72909	1.423099	0.81078	-0.13447	-0.93681	0.711135	-0.44795	0.799476	-0.42321	-0.56556	-0.01879
Q9C0C2_S836_1_1	Q9C0C2	TNKS1BP	0.302447	0.212814	-0.10602	-0.09639	-1.42601	-0.96714	-0.61429	0.145912	0.343328	0.408218	-0.14406	-1.10475	-1.1638
Q9C0C2_S872_1_1	Q9C0C2	TNKS1BP	-0.03776	0.595856	-0.593	1.098449	-0.1237	1.022493	0.652905	0.713193	0.643828	0.281794	0.685778	0.06312	-0.15389
Q9C0C2_S893_1_1	Q9C0C2	TNKS1BP	-0.28078	-0.25988	-0.7572	0.930006	-0.41637	0.898757	0.963429	-0.12424	0.901031	-0.02154	0.936573	0.040515	-0.16104
Q9C0C2_S936_1_1	Q9C0C2	TNKS1BP	-1.68157	-2.15046	-0.08324	0.707654	-1.57417	-3.64631	-2.43113	0.567997	-0.42849	0.813488	-0.84096	-1.17126	-0.94233
Q9H1B7_S547_1_1	Q9H1B7	IRF2BPL	-0.11617	-0.27729	-0.14324	0.399622	0.047269	-0.13962	-0.42405	0.213439	0.074641	0.584995	0.218311	-0.43208	-0.01546
Q9H1E3_S181_1_1	Q9H1E3	NUCKS1	-1.07511	-0.37896	-0.16988	-1.03398	0.609828	0.158783	-0.77064	-0.25921	0.745616	0.189114	0.871018	1.690746	1.582126
Q9H1E3_S19_1_1_	Q9H1E3	NUCKS1	0.365651	-0.03115	-0.4125	0.772582	-1.4958	0.45981	0.852846	0.256372	1.013811	0.676598	-0.31842	1.0035	0.689582
Q9H1E3_S214_1_1	Q9H1E3	NUCKS1	-0.66508	-0.28805	-0.41644	1.191	-0.72778	0.325319	-0.67736	0.203678	0.465138	1.222918	0.73841	-0.15165	-0.10588
Q9H1E3_S223_1_1	Q9H1E3	NUCKS1	-0.91034	-0.73066	-0.73027	2.451073	0.934706	0.438685	-1.86022	1.450731	-0.11849	1.225269	-0.80053	-1.40002	-1.16926
Q9H1E3_T222_1_1	Q9H1E3	NUCKS1	-0.0666	0.108922	-0.99775	2.372804	0.955639	0.448141	-1.58095	0.864084	-0.9305	0.374135	-1.87044	-1.00446	-0.34781
Q9H1Z4_S70_1_1_l	Q9H1Z4	WDR13	-0.16198	-1.33308	-1.66283	0.744048	-0.24587	-1.0858	-1.01053	-0.50457	-0.66499	-0.30381	-0.65644	-0.76326	-0.13597
Q9H2P0_S921_1_1	Q9H2P0	ADNP	-0.06396	-0.34451	-0.98024	-0.09074	-0.63257	0.543564	0.20846	0.255033	0.42049	-0.42303	-0.48374	-0.41282	-0.93844
Q9H307_S347_1_1	Q9H307	PNN	1.253781	1.075633	-0.3863	0.43559	0.646847	1.006093	-0.24652	1.039851	1.307879	0.623584	-0.40789	-1.41203	-1.32962
Q9H307_S443_1_1	Q9H307	PNN	0.710061	-0.59688	0.18776	1.837082	0.101781	0.461565	0.132371	1.622615	-1.14147	-0.13624	0.446999	-0.22984	-0.86876
Q9H307_S66_1_1_l	Q9H307	PNN	0.698424	-1.12618	0.162383	0.314824	-1.52982	-1.24289	-1.5623	-0.90701	-1.05234	-0.13831	-0.4388	-2.30244	-1.69312
Q9H3N1_S247_1_1	Q9H3N1	TMX1	0.150119	0.370618	-0.39948	0.246221	-0.05174	0.322923	-0.2197	-0.31138	0.101758	0.586508	-0.03577	-0.23267	0.017503
Q9H3N1_S270_1_1	Q9H3N1	TMX1	-0.00471	-0.73863	-0.2762	-0.17265	-1.87129	-1.13126	0.82806	-1.64259	-0.36959	0.582007	-1.07383	-2.11038	0.016299
Q9H4A3_S185_1_1	Q9H4A3	WNK1	-0.20494	-0.29842	-0.21161	0.024109	0.31119	0.220912	-0.5479	0.254216	-0.05187	-0.00792	-0.41758	-0.19431	-0.04063
Q9H5H4_S125_1_1	Q9H5H4	ZNF768	0.38571	-0.03366	-0.08719	-0.30724	-2.344	-0.13946	0.556326	-1.04129	-0.05512	0.175938	0.115462	0.251366	0.021855
Q9H5H4_S83_1_1_	Q9H5H4	ZNF768	0.537873	-0.40773	-0.20858	-0.62738	-3.16059	0.165797	0.254129	-2.172	-0.65536	-0.68374	0.732164	0.039971	-0.14969
Q9H6F5_S113_1_1	Q9H6F5	CCDC86	0.893274	-0.47317	-0.64169	0.919505	0.687784	0.670813	0.0344	0.251363	-0.50081	0.289595	0.015438	0.162896	-0.52439
Q9H6S3_S570_1_1	Q9H6S3	EPS8L2	1.177721	0.321169	0.472183	-0.58844	-2.14111	-0.50918	1.024494	0.411339	-0.07242	0.133475	0.464974	-0.49368	-0.07287
Q9H788_S315_1_1	Q9H788	SH2D4A	0.31875	-0.37418	0.143783	0.89262	-1.35355	-1.94024	0.090046	-0.93655	0.504488	-0.63661	0.129293	0.05501	-1.24245
Q9H792_S281_1_1	Q9H792	PEAK1	0.560611	0.144762	-0.22754	0.346127	0.335189	-0.20715	-0.33391	-0.277	0.088948	0.469696	-0.15388	0.057894	0.328007
Q9H7D7_S121_1_1	Q9H7D7	WDR26	0.176044	0.16339	-1.00397	1.212562	-0.18232	-0.16138	0.166461	1.192996	0.25417	1.161793	0.332457	-0.62002	-0.06558
Q9H7N4_S725_1_1	Q9H7N4	SCAF1	-0.90593	-1.79961	-0.61389	0.395163	1.414274	0.860872	-0.94543	1.346889	-0.16255	0.642425	-0.33036	-2.21048	-0.03072
Q9H7N4_S975_1_1	Q9H7N4	SCAF1	-0.28054	-0.48732	-0.36251	-0.71355	0.13202	0.640706	0.123995	-0.25591	0.246691	-0.19242	1.158753	0.495032	-0.104
Q9H7N4_T976_1_1	Q9H7N4	SCAF1	-0.86555	-1.07169	0.125177	0.046104	-0.23797	0.629835	0.128615	-0.07709	0.111748	0.358804	0.427719	0.058112	-0.33973
Q9HB58_S244_1_1	Q9HB58	SP110	0.558732	0.3545	-0.49063	-0.68901	0.226693	0.273849	-0.21852	0.404402	0.013618	0.147918	-0.38428	-0.52631	0.14946
Q9HBL0_S1124_1_	Q9HBL0	TNS1	0.102036	-0.03401	0.224085	0.237689	-0.68717	-0.00686	0.011729	1.843693	-0.05155	0.591342	-0.54575	0.460881	0.077771
Q9HBL0_S1269_1_	Q9HBL0	TNS1	-0.94785	-0.31658	-0.65805	0.354693	-0.35819	0.931334	1.059633	0.263977	0.461016	0.302401	0.243507	-0.19814	-0.40404
Q9HBL0_S1381_1_	Q9HBL0	TNS1	-0.2602	0.663792	-0.36274	0.218327	-0.43811	0.861467	0.675561	-0.38695	0.543472	0.165459	0.365087	0.479001	-0.52539
Q9HBL0_S338_1_1	Q9HBL0	TNS1	-0.18055	-0.10533	-0.47891	1.108033	0.242495	-0.30161	0.111203	0.517122	0.024914	0.274519	0.253147	1.166376	-0.01907
Q9HBL0_T860_1_1	Q9HBL0	TNS1	-1.62815	-1.5893	-0.70463	1.963413	-0.28214	-0.187	0.142074	-0.5494	0.549735	0.872701	0.072193	-0.07491	-1.03015
Q9HCK8_S2046_1_	Q9HCK8	CHD8	-0.20734	0.165855	0.189612	0.041079	-0.49895	-0.51951	-0.38156	0.214488	-0.48316	0.468414	0.428525	0.303638	0.564624
Q9NPQ8_S436;T44	Q9NPQ8	RIC8A	-0.08455	-0.22035	-0.21819	-0.37506	-0.48911	0.765023	0.406999	0.056489	1.309061	0.475281	0.181803	0.084785	0.640413
Q9NQ55_S359_1_1	Q9NQ55	PPAN	-0.31465	-0.04799	-0.75537	0.376235	0.001073	0.236985	0.009925	-0.55248	-0.20436	0.989346	0.071792	-0.05802	-0.70754
Q9NQS7_S263_1_1	Q9NQS7	INCENP	1.912569	1.401269	-0.19291	-0.21452	-1.38708	0.416579	-0.59209	-0.08206	0.231796	-0.31117	0.018935	0.305914	0.192057
Q9NR19_S30_1_1_	Q9NR19	ACSS2	-0.14239	0.217593	-0.78923	0.451731	-0.26428	-0.14108	-0.37983	0.102553	0.429675	0.041314	0.194649	-0.94007	0.138352
Q9NRA8_S78_1_1_	Q9NRA8	EIF4ENIF1	1.052726	0.669667	0.143435	0.076402	0.00533	0.453279	-0.34051	0.866057	0.058772	0.255076	-0.31113	-0.19658	-0.12881
Q9NRA8_S951_1_1	Q9NRA8	EIF4ENIF1	-0.78772	-0.53218	-0.24953	0.892216	-0.11451	0.247241	-0.67848	0.312332	-0.65883	0.331656	-0.34211	-0.27387	0.106019
Q9NRG9_S495_1_1	Q9NRG9	AAAS	-0.19329	-0.2887	-0.22711	1.754256	0.328272	0.592668	-0.25901	-0.00736	0.091251	0.895923	-0.1577	-0.29298	0.031456
Q9NSK0_S566_1_1	Q9NSK0	KLC4	0.47998	0.89894	0.193975	0.875158	-0.08804	-1.34384	0.944621	0.117797	0.362259	0.328251	-2.16527	-0.42085	0.06017
Q9NSK0_S611_1_1	Q9NSK0	KLC4	0.04343	-0.494	0.076804	-0.07116	-1.09988	-1.14125	0.547779	-0.31844	0.287837	0.406836	0.220766	-1.42085	0.160545
Q9NTI5_S1257_1_1	Q9NTI5	PDS5B	-0.15631	-0.33593	-0.06632	-0.28529	-1.81638	-1.55077	-0.80252	-0.70459	-0.54189	-0.00536	0.614045	-0.92426	-1.07566
Q9NTI5_S1358_1_1	Q9NTI5	PDS5B	-0.05219	0.029503	-0.3532	-0.50011	-0.80292	-0.15903	0.158463	-0.41591	0.509179	-0.82521	-0.10071	-0.3147	-0.53398
Q9NVU7_S585_1_1	Q9NVU7	SDAD1	-0.47145	-0.20257	-0.7295	1.100384	0.289658	0.395667	-0.06634	0.3775	0.23081	0.483843	-0.60208	-0.55803	-0.46755
Q9NW75_S284_1_1	Q9NW75	GPATCH2	-1.03596	-2.12533	0.833368	0.42054	-2.17715	-0.68022	1.051497	0.549255	1.553787	-0.0151	0.030566	-0.05176	-0.7368
Q9NWH9_S289_1_	Q9NWH9	SLTM	-0.55349	-0.46051	-0.58379	1.75077	0.427992	0.428918	-0.42956	1.007975	0.266489	0.80781	-0.36484	-0.37825	-0.26876
Q9NWH9_S553_1_	Q9NWH9	SLTM	0.957924	1.626154	-0.00617	1.193975	-0.40218	-0.35979	-0.14962	-0.95897	-0.37514	0.223021	-0.31197	-0.40171	-0.17073
Q9NX63_S50_1_1_l	Q9NX63	CHCHD3	-1.4531	0.707205	-0.80767	1.333425	0.068707	1.278583	-0.00397	0.552946	1.563199	1.376131	0.50321	0.096106	0.539717
Q9NYF8_S177_1_1	Q9NYF8	BCLAF1	-1.02975	-0.25704	-0.43046	0.955396	0.722901	0.370996	-0.12088	0.655001	0.548383	0.85592	-0.15947	0.160162	-0.1196
Q9NYF8_S385_1_1	Q9NYF8	BCLAF1	-0.09474	-0.61262	-0.47931	0.609793	-1.34679	0.296312	-0.17603	0.214951	0.481317	0.170151	-0.03672	-0.01071	-0.44014
Q9NYF8_S397_1_1	Q9NYF8	BCLAF1	-0.66713	-0.93822	-1.8428	2.120934	-0.59421	-0.6654	-0.79461	0.622403	0.233351	1.103725	0.107645	0.875292	2.071512
Q9NYF8_S496_1_1	Q9NYF8	BCLAF1	-0.5665	-0.72878	-0.43428	0.817115	0.416018	0.084735	-0.40935	0.7069	0.152675	0.518992	-0.4158	0.077128	0.173787
Q9NYF8_S531_1_1	Q9NYF8	BCLAF1	-1.07479	-1.07541	-0.84767	1.086351	-0.14914	-0.38829	-0.27242	-0.22517	-0.85104	0.297912	-0.06561	0.217072	0.592682

Q9NYF8_S648_1_1	Q9NYF8	BCLAF1	-1.07955	-0.33544	-0.36366	1.207314	-0.17926	0.112253	0.003553	1.373616	1.132592	1.119831	-0.42836	-0.0224	0.253968
Q9NYF8_S658_1_1	Q9NYF8	BCLAF1	-1.03043	-0.91815	-0.42959	1.060971	0.04702	0.171452	-0.34299	0.572796	-0.38014	0.407038	-0.38624	-0.35136	0.272402
Q9NYM9_S9_1_1_N	Q9NYM9	BET1L	0.57386	-0.35783	-1.2589	0.507041	-0.5195	-0.2993	0.034966	-0.58853	-0.3909	0.757615	0.957795	1.56445	-0.55824
Q9NZM1_S174_1_1	Q9NZM1	MYOF	-0.22202	-0.04356	-1.57208	0.68966	-0.29641	-0.50672	-0.51295	-0.14941	-0.56452	0.183626	-0.56443	-0.20872	0.030176
Q9NZN4_S438_1_1	Q9NZN4	EHD2	0.501822	0.629751	-0.52376	0.427723	-0.39919	0.558133	0.257405	0.635823	0.67449	0.356298	0.332781	-0.25547	-0.25184
Q9NZN4_S438_1_1	Q9NZN4	EHD2	-0.45386	-0.15137	-0.72756	0.740043	-0.04497	0.278515	-0.3715	-0.14362	-0.03318	-0.53933	0.188392	-0.4867	-0.44546
Q9NZT2_S315_1_1	Q9NZT2	OGFR	0.317721	-0.57169	-0.85467	1.115591	0.062772	0.459472	0.389834	-0.80147	-0.38223	0.622735	0.527107	-0.03538	0.212462
Q9P035_S114_1_1	Q9P035	HACD3	-0.11567	0.470892	-1.81889	0.55099	0.008035	0.232941	0.585482	0.819662	0.942441	-0.41014	0.607806	0.590824	0.53659
Q9P035_S114_1_1	Q9P035	HACD3	-0.20971	0.207339	-0.77123	0.009774	0.022983	0.093658	-0.19023	0.783527	0.203342	-0.32653	0.291113	-0.10702	-0.02528
Q9P0U4_S138_1_1	Q9P0U4	CXXC1	-0.10883	0.219346	-0.37676	-1.21481	-0.70852	0.0573	0.409565	-0.4456	0.519283	-0.39352	0.020846	-0.17724	-0.31571
Q9P107_S437_1_1	Q9P107	GMIP	1.043423	0.510719	-0.35516	-0.51178	-0.1147	0.142934	-0.1389	-0.28417	1.267407	0.23718	-0.70378	-0.28761	0.636624
Q9P1Y6_S1229_1_	Q9P1Y6	PHRF1	-0.62734	-0.5181	-0.42398	0.691347	0.297738	0.563409	0.472425	0.50341	0.687133	1.045302	0.023165	-0.19144	0.285606
Q9P260_S180_1_1	Q9P260	KIAA1468	0.236296	-0.12064	1.059879	-0.46825	-1.40565	-0.39756	-0.85819	-0.29272	-0.57741	-0.03943	0.061214	-0.56298	0.761533
Q9P266_S1225_1_	Q9P266	JCAD	0.479394	0.086587	-0.90168	0.442902	-0.03389	-0.59491	-0.61	0.002917	-0.05488	0.731131	-0.00511	-0.44262	1.050134
Q9P2E9_S1277_1_	Q9P2E9	RRBP1	-0.46287	-0.46963	-0.82622	1.603123	0.332941	-1.55173	-1.08153	0.086538	-1.3792	0.715989	-1.04992	0.0061	0.908526
Q9P2E9_S573_1_1	Q9P2E9	RRBP1	0.09188	-0.63899	-0.7061	0.859988	-0.51113	0.51054	0.001487	1.314358	-0.21991	1.238961	0.371603	-0.38388	0.057085
Q9P2E9_S583_1_1	Q9P2E9	RRBP1	-0.41593	-0.99661	0.040862	1.067318	-0.60757	0.512584	0.608953	0.547076	-0.79996	1.433263	1.024779	-0.0494	0.37957
Q9P2G1_S737_1_1	Q9P2G1	ANKIB1	-0.4641	0.35205	-0.83502	2.212946	0.986714	-0.28348	-0.18096	-0.37458	0.310748	0.787025	0.32851	-0.85061	0.137278
Q9P2K5_S17_1_1_	Q9P2K5	MYEF2	0.052706	0.1564	-0.69526	-0.03442	-0.68821	-0.00747	0.791825	-0.1705	0.169745	0.362743	0.413915	-0.2718	-0.0199
Q9UBB9_S210_1_1	Q9UBB9	TFIP11	0.189591	0.641424	-0.17333	0.676261	0.048886	-0.0503	-0.13422	0.671355	0.967144	0.428006	0.21928	-0.21168	-0.03103
Q9UBC2_T366_1_1	Q9UBC2	EPS15L1	-0.25377	-0.89252	0.354174	1.346959	-0.42294	-1.17719	-1.2486	1.180488	1.668109	0.928699	2.693676	1.626298	-0.40062
Q9UBG0_S1457_1_	Q9UBG0	MRC2	-0.85281	-0.74604	-1.0024	-0.47508	-1.03189	-1.49974	-0.40007	-0.27368	-0.5702	-0.05801	-0.93804	-0.85121	0.232519
Q9UBG0_S1458_1_	Q9UBG0	MRC2	-0.22807	-0.35172	-0.67821	-0.20222	-0.79866	-0.70875	-0.57734	-0.86715	-0.821	-1.32426	-1.5395	-0.05558	-0.09877
Q9UBW5_S395_1_	Q9UBW5	BIN2	-0.32178	0.536866	-1.01956	0.572065	0.495075	-0.37154	0.618656	1.221731	-0.6494	1.322357	0.223401	-0.33396	0.593947
Q9UBW5_S458_1_	Q9UBW5	BIN2	-0.55826	-0.2285	-1.39738	-0.33618	-0.80058	0.404623	0.691049	-0.00086	0.16906	0.656981	-0.49855	-0.24339	0.011385
Q9UDY2_S174_1_1	Q9UDY2	TJP2	-0.00394	-0.3553	-0.26265	0.921426	-0.1619	-0.00648	-0.08155	0.408841	0.156687	0.517518	0.760343	-0.06088	0.171146
Q9UDY2_S266_1_1	Q9UDY2	TJP2	0.256534	-0.67057	-0.25466	0.243199	-0.0618	0.29403	0.473165	-0.02644	0.086818	-0.27291	-0.39346	0.36668	0.264247
Q9UER7_S495_1_1	Q9UER7	DAXX	0.313821	0.164997	-0.72157	-0.86587	-0.56459	0.456546	-0.03773	-1.39384	0.013874	0.023883	-0.37851	-0.24358	0.353051
Q9UEW8_S385_1_	Q9UEW8	STK39	0.199795	0.764658	-0.03339	1.154234	-0.95638	-0.23915	0.234579	-0.23167	-0.13257	0.658065	-0.01335	0.588189	-0.69467
Q9UGU0_S574_1_1	Q9UGU0	TCF20	-0.13821	0.351075	-0.83238	0.552778	-0.12835	0.744123	0.466564	0.203049	0.08414	0.343413	0.375989	-0.22762	-0.37404
Q9UGU0_T1671_1_	Q9UGU0	TCF20	0.249472	0.14628	-0.79568	0.810003	0.803968	-0.12747	-0.33436	0.852599	0.600239	0.484355	0.27118	-0.01326	0.552462
Q9UH62_S61_1_1_	Q9UH62	ARMCX3	-0.46936	0.546954	-0.66695	0.89683	-0.20057	-0.46461	0.105805	-0.09566	0.491703	0.303583	0.153535	-0.78074	0.188163
Q9UH99_S116_1_1	Q9UH99	SUN2	0.422195	0.336043	-0.66489	-0.58231	0.835552	0.264829	-0.32886	0.293145	0.086403	-1.13954	-2.25016	-0.22139	-0.24317
Q9UHB6_S15_1_1_	Q9UHB6	LIMA1	1.164406	-1.45865	0.609776	0.402801	-0.01486	-2.05761	0.004135	0.30591	-1.70254	0.068378	-0.7174	-0.32412	-0.79084
Q9UHB6_S369_1_1	Q9UHB6	LIMA1	-0.14564	0.372637	-0.51628	0.066351	-0.41839	0.515816	1.901597	0.646293	-0.58879	1.692602	0.410246	-0.33813	0.750306
Q9UHB6_S373_1_0	Q9UHB6	LIMA1	-0.20932	0.148897	0.105855	1.869945	1.204971	0.828184	-1.17478	1.833262	-0.52318	0.813558	-0.50177	-0.82416	-0.07855
Q9UHB6_S490_1_1	Q9UHB6	LIMA1	0.263211	-0.39074	-0.52986	0.508817	-0.14037	-0.30991	-0.30896	-0.24812	-0.15539	0.853803	-0.11287	-0.35271	0.144668
Q9UHB6_S609_1_1	Q9UHB6	LIMA1	-0.54195	0.359723	-0.41611	0.812359	0.699766	0.340698	-0.19546	0.224988	-0.58537	0.575093	0.041566	-0.63408	0.050212
Q9UHB6_S698_1_1	Q9UHB6	LIMA1	-0.57475	-0.42904	-0.32445	-0.17461	-0.27871	-0.23825	-0.00724	-0.65505	-0.21619	0.529686	-0.19122	0.000726	-0.1551
Q9UHW9_S1032_1_	Q9UHW9	SLC12A6	0.269813	-0.09343	-0.27838	1.309737	-1.02603	-1.18143	-0.62799	1.19233	-1.6727	1.84822	0.355367	-0.36884	0.402022
Q9UJ70_S76_1_1_	Q9UJ70	NAGK	0.345206	0.707537	-0.58137	0.996406	-0.0269	-0.33974	-0.21358	0.387784	-0.5371	1.120177	-0.25012	0.23038	-0.21424
Q9UJU6_S269_1_1	Q9UJU6	DBNL	0.921279	1.054964	-0.42699	-0.13345	-1.04723	-0.1562	0.963617	-0.17698	-0.27032	0.875165	0.3003	-2.28315	0.702262
Q9UJU6_T291_1_1	Q9UJU6	DBNL	1.090823	-0.00827	-0.29199	-0.35105	-1.0494	-1.14909	0.536634	-0.13612	-0.05777	0.061782	-0.94209	0.366428	0.561778
Q9UJZ1_T327_1_1	Q9UJZ1	STOML2	0.01937	0.626999	-1.00625	2.061381	1.062329	-0.06132	-2.32051	0.213572	-0.46592	0.995939	-0.38881	-0.49319	0.124352
Q9UK61_S927_1_1	Q9UK61	FAM208A	0.604333	1.033565	-0.24855	0.489275	0.025741	0.329525	-0.29311	0.481095	-0.03173	0.771488	-0.18039	-0.58238	-1.36793
Q9UKA4_S1242_1_	Q9UKA4	AKAP11	-0.16954	-0.30671	-1.73886	0.802961	0.013034	0.41587	0.919215	-0.98088	-0.82339	0.628175	0.448008	-0.37718	0.728415
Q9UKA4_S448_1_1	Q9UKA4	AKAP11	1.067077	-0.61973	-0.11827	0.07642	-1.98632	0.434063	1.256719	-0.8202	0.823835	0.584816	0.948823	0.377484	-0.84234
Q9UKJ3_S1014_1_	Q9UKJ3	GPATCH8	0.039604	-0.05365	-0.54433	2.03857	1.352234	0.604699	1.302416	0.2027	0.220521	1.063608	-0.07504	-0.4353	0.899438
Q9UKM9_S135_1_1	Q9UKM9	RALY	0.220522	-0.46877	-0.80971	-0.00667	-0.25325	0.115344	-0.17375	-0.46621	0.241757	0.287987	-0.00548	-0.50854	-0.21742
Q9UKN8_S611_1_1	Q9UKN8	GTF3C4	0.809032	0.017333	-0.53503	0.398535	-0.17173	0.635135	0.30153	0.054292	0.289254	0.434086	0.049353	-1.29196	0.180362
Q9UKT5_T47_1_0_	Q9UKT5	FBXO4	0.541271	-0.53315	-0.29722	1.808121	-0.52471	-0.60291	0.277666	-1.94487	-0.55566	0.589198	-0.82573	-0.96931	-1.19152
Q9UKV3_S1004_1_	Q9UKV3	ACIN1	-0.23195	-0.14391	-0.46883	2.053147	0.682191	-0.15727	-0.45464	1.376331	-0.25407	1.471922	-0.21839	-0.67968	-0.08021
Q9UKV3_S490_1_1	Q9UKV3	ACIN1	-0.30771	-0.8622	-0.40017	1.736771	-0.81613	0.111101	0.087863	0.288692	-0.04863	-0.18081	-0.79388	-1.59182	-0.20107
Q9UKV3_S657_1_1	Q9UKV3	ACIN1	0.135671	-0.24523	-0.59331	1.323568	0.71987	0.394369	-0.0552	0.476775	-0.69245	1.01153	-0.20315	-0.97825	-0.49131
Q9UKV3_S710_1_1	Q9UKV3	ACIN1	0.288009	-0.02966	-0.35948	0.217465	-0.61205	-0.62374	-0.2605	-0.50744	-0.23187	0.35843	0.072902	-1.56437	0.032674
Q9ULJ8_S840_1_1	Q9ULJ8	PPP1R9A	1.282442	-0.51356	-0.09064	0.193262	-0.53239	-0.25318	-0.21146	-1.25517	0.417639	-1.43431	-0.40584	-0.49891	-0.20345
Q9ULT8_S357_1_1	Q9ULT8	HECTD1	-0.81074	-0.18555	-0.67703	0.55813	-1.05692	-2.19827	-0.24375	-1.17422	0.175564	0.294711	-0.69518	-0.68351	-0.31318
Q9ULU4_S425_1_1	Q9ULU4	ZMYND8	0.568317	0.503104	-0.58243	0.749677	-0.87882	0.350662	-0.2916	-0.81514	0.32553	0.278147	0.018674	-1.38956	0.355806
Q9ULU4_S475_1_1	Q9ULU4	ZMYND8	-0.04731	-0.14137	-0.61664	-0.32302	-1.23495	0.459124	0.629723	-0.91874	0.488365	-0.34139	-0.07266	0.428629	-0.23015
Q9UMS6_S363_1_1	Q9UMS6	SYNPO2	-0.89978	-0.27193	-0.9034	-0.07286	-0.94793	-1.34328	-2.15278	0.530636	-1.38084	0.920929	0.291709	-1.0993	-0.2535
Q9UMZ2_S752_1_1	Q9UMZ2	SYNRG	-0.21042	0.024449	0.402289	0.209146	-0.78069	0.28726	-0.02139	0.768183	0.366856	0.367271	-0.17859	0.319617	-0.3219
Q9UN36_S332_1_1	Q9UN36	NDRG2	0.172467	-0.7125	0.182955	0.849151	-1.03424	-0.64204	1.203904	0.308972	-0.11442	0.237375	0.046544	-0.14114	-0.73529
Q9UN81_S18_1_1_	Q9UN81	L1RE1	-0.85637	0.56971	0.195894	-0.18249	-0.4072	0.269039	0.3231	0.174635	-0.02052	0.114984	0.030994	0.048892	-0.45677
Q9UN86_S225_1_1	Q9UN86	G3BP2	0.01667	0.066782	-0.6454	0.139462	-0.29626	0.318105	-0.52133	-0.53799	0.214291	0.378215	-0.17934	-0.9218	-0.09175
Q9UNE7_S19_1_1_	Q9UNE7	STUB1	0.101062	0.740355	-0.64791	0.951084	-0.43904	0.050235	-0.50864	1.965422	-0.98049	-0.42659	-0.09323	-0.93035	-0.7202
Q9UNL2_S105_1_1	Q9UNL2	SSR3	-0.54139	0.583562	-0.6724	-0.1211	-1.20074	-0.83942	-0.70359	-0.9134	-1.31799	-0.36293	-1.96956	-0.92555	-0.50253
Q9UP95_S967_1_1	Q9UP95	SLC12A4	0.162423	0.017678	-0.44172	1.200528	-0.87733	-0.36865	-0.44446	0.276443	-0.28696	0.68314	0.277131	0.472753	0.722793
Q9UPN3_S280_1_1	Q9UPN3	MACF1	-1.03835	-0.74809	0.488492	1.665825	0.584108	-0.82201	-0.05297	1.132413	-0.17713	1.095213	-0.09888	-0.45327	-0.00901
Q9UPN3_S3927_1_	Q9UPN3	MACF1	-0.62656	0.117813	0.157367	0.38349	-0.32663	-0.3571	0.647664	0.515114	0.326245	0.940284	-0.29881	-0.15211	0.088721
Q9UPN6_S617_1_1	Q9UPN6	SCAF8	-0.19094	0.436201	0.601613	0.554688	0.274155	-0.0135	0.746815	0.098472	-0.18504	0.605414	-0.2859	-1.36664	-0.12379
Q9UPQ0_S217_1_1	Q9UPQ0	LIMCH1	0.155283	-0.82286	-0.09449	0.212816	0.227971	-0.58792	-1.35586	-0.73481	-0.55286	0.263165	0.29256	-0.65427	0.973519
Q9UPT8_S1269;S1:	Q9UPT8	ZC3H4	0.256959	-1.25664	-0.74742	0.007747	-2.24641	0.212694	0.886584	-0.44031	0.438336	0.609042	0.362282	-0.19934	-1.14361
Q9UPU5_S1141_1_	Q9UPU5	USP24	-0.07935	1.010457	-0.04005	-0.18122	0.417495	0.128904	-0.41067	0.071051	-0.08686	0.423911	0.364507	0.174759	-0.11058
Q9UPU5_S1299_1_	Q9UPU5	USP24	0.250942	0.459292	-0.12811	0.107343	-1.40117	-0.36737	-0.37054	-1.54798	0.0863	0.055146	-0.2386	-0.88029	-0.19146
Q9UPU5_S1943_1_	Q9UPU5	USP24	-0.53995	-0.01275	-0.53308	0.718722	0.356574	-0.06713	-0.04676	0.969536	0.153446	0.145171	0.712119	-0.67084	0.538318
Q9UPU5_S2047_1_	Q9UPU5	USP24	-0.42631	-1.30072	0.243985	1.902326	0.185855	-0.00707	-0.47059	0.882235	-0.44358	1.885366	-0.06451	-0.99885	-0.0698
Q9UPU9_S420_1_1	Q9UPU9	SAMD4A	-1.43644	-1.31684	-0.07765	1.605438	-0.71534	-0.73522	0.275111	0.795806	-0.1322	0.486416	0.660875	-0.43505	0.648508
Q9UPW0_S223_1_	Q9UPW0	FOXJ3	0.896423	0.90345	-0.32372	0.639978	0.219304	0.696548	0.061366	-0.05105	0.491627	0.407019	0.01454	0.950192	0.117923
Q9UQ35_S1103_1_	Q9UQ35	SRRM2	0.49217	0.14008	-0.50591	1.801036	1.05085	0.588367	-0.7093	1.363978	0.117351	0.947441	-0.09114	-1.11579	-0.48362
[truncated: 173,418 more chars]
