# Supplementary material for: In situ profiling reveals metabolic alterations in the tumor microenvironment of ovarian cancer after chemotherapy
Source: NPJ Precis Oncol. 2023 Nov 3;7:115. doi: 10.1038/s41698-023-00454-0 (PMC10624842; doi:10.1038/s41698-023-00454-0)
Supplement: Supplementary file 2 — Reporting Summary [file 41698_2023_454_MOESM2_ESM.pdf]

nature portfolio

Corresponding author(s): *Double-blind peer review submissions: write DBPR and your manuscript number here instead of author names.*

Last updated by author(s): *YYYY-MM-DD*

Reporting Summary

Nature Portfolio wishes to improve the reproducibility of the work that we publish. This form provides structure for consistency and transparency in reporting. For further information on Nature Portfolio policies, see our Editorial Policies and the Editorial Policy Checklist.

Statistics

For all statistical analyses, confirm that the following items are present in the figure legend, table legend, main text, or Methods section.

n/a	Confirmed	
☐	☒	The exact sample size (n) for each experimental group/condition, given as a discrete number and unit of measurement
☐	☒	A statement on whether measurements were taken from distinct samples or whether the same sample was measured repeatedly
☐	☒	The statistical test(s) used AND whether they are one- or two-sided<br>*Only common tests should be described solely by name; describe more complex techniques in the Methods section.*
☐	☒	A description of all covariates tested
☐	☒	A description of any assumptions or corrections, such as tests of normality and adjustment for multiple comparisons
☐	☒	A full description of the statistical parameters including central tendency (e.g. means) or other basic estimates (e.g. regression coefficient) AND variation (e.g. standard deviation) or associated estimates of uncertainty (e.g. confidence intervals)
☐	☒	For null hypothesis testing, the test statistic (e.g. F, t, r) with confidence intervals, effect sizes, degrees of freedom and P value noted<br>*Give P values as exact values whenever suitable.*
☒	☐	For Bayesian analysis, information on the choice of priors and Markov chain Monte Carlo settings
☒	☐	For hierarchical and complex designs, identification of the appropriate level for tests and full reporting of outcomes
☒	☐	Estimates of effect sizes (e.g. Cohen's d, Pearson's r), indicating how they were calculated

Our web collection on statistics for biologists contains articles on many of the points above.

Software and code

Policy information about availability of computer code

Data collection	The mass spectrometry proteomics data are available at the ProteomeXchange Consortium via the PRIDE (https://doi.org/10.1093/nar/gky1106) partner repository with the dataset identifier PXD014980.<br>DESI-MS data were deposited at https://data.mendeley.com/datasets/zzr5rk7vj5/1
Data analysis	https://reactome.org/ and https://www.pathwaystudio.com/ for pathway analysis.<br>glmnet package in R(34) was used to create a ridge regression model for the classification of treatment response<br>Metaboanalyst 5.0 was used for sparse partial least squares discriminant analysis

For manuscripts utilizing custom algorithms or software that are central to the research but not yet described in published literature, software must be made available to editors and reviewers. We strongly encourage code deposition in a community repository (e.g. GitHub). See the Nature Portfolio guidelines for submitting code & software for further information.

Data

Policy information about availability of data

All manuscripts must include a data availability statement. This statement should provide the following information, where applicable:

- Accession codes, unique identifiers, or web links for publicly available datasets
- A description of any restrictions on data availability
- For clinical datasets or third party data, please ensure that the statement adheres to our policy

The mass spectrometry proteomics data are available at the ProteomeXchange Consortium via the PRIDE (https://doi.org/10.1093/nar/gky1106) partner repository with the dataset identifier PXD014980.
DESI-MS data were deposited at https://data.mendeley.com/datasets/zzr5rk7vj5/1

Human research participants

Policy information about studies involving human research participants and Sex and Gender in Research.

Reporting on sex and gender	Patients were all female.
Population characteristics	population characteristics are reported in ST1
Recruitment	All patients available were selected according to the inclusion criteria
Ethics oversight	The study was approved by the Institutional Review Board of The University of Texas MD Anderson Cancer Center, and all samples were collected after obtaining written informed consent from patients (PA11-1015_CR003).For the collection of tissues from GYN-COE, patients provided broad consent for their tissues to be used in future research under WCG IRB Protocol #20110222, Tissue and Data Acquisition Activity for the Study of Gynecologic Disease; The paired tumor specimens and clinical data were collaboratively evaluated under WCG IRB Protocol #14-1679, an Integrated Molecular Analysis of Endometrial Cancer, Ovarian Cancer, and Other Medical Conditions to Identify and Validate Clinically Informative Biomarkers and Factors, and the fully executed Material Transfer Agreement #205-20. For the collection from Washington University the study was approved by the Institutional Review Board of the University of Iowa (protocol #201507805).

Note that full information on the approval of the study protocol must also be provided in the manuscript.

Field-specific reporting

Please select the one below that is the best fit for your research. If you are not sure, read the appropriate sections before making your selection.

☒ Life sciences ☐ Behavioural & social sciences ☐ Ecological, evolutionary & environmental sciences

For a reference copy of the document with all sections, see nature.com/documents/nr-reporting-summary-flat.pdf

Life sciences study design

All studies must disclose on these points even when the disclosure is negative.

Sample size	Sample size was sufficient to detect statistically significant SAM differences between the groups
Data exclusions	After passing quality check on tumor sections, no data were excluded from the analysis
Replication	DESI MS was not performed in duplicate since prior studies have evaluated its reproducibility on serial sections. Cell viability experiment (MTT) after GLDC knock down was performed in three biological replicates, each replicate had at east three technical replicates per condition. RT pcr was perfomed in three technical replicates
Randomization	patients were allocated in the ER and PR groups according to their response to NACT
Blinding	It was not possible to conduct the analysis blindly due to the explanatory codes provided for each sample. The allocation of each sample in the ER and PR group did not bias the analysis at any step due to the exploratory nature of the experimental design.

Reporting for specific materials, systems and methods

We require information from authors about some types of materials, experimental systems and methods used in many studies. Here, indicate whether each material, system or method listed is relevant to your study. If you are not sure if a list item applies to your research, read the appropriate section before selecting a response.

Materials & experimental systems

n/a	Involved in the study
☒	☐ Antibodies
☐	☒ Eukaryotic cell lines
☒	☐ Palaeontology and archaeology
☒	☐ Animals and other organisms
☐	☒ Clinical data
☒	☐ Dual use research of concern

Methods

n/a	Involved in the study
☒	☐ ChIP-seq
☒	☐ Flow cytometry
☒	☐ MRI-based neuroimaging

Eukaryotic cell lines

Policy information about cell lines and Sex and Gender in Research

Cell line source(s)	All ovarian cancer cell lines were obtained from the American Type Culture Collection or the MD Anderson Characterized Cell Line Core Facility, which supplies authenticated cell lines. OVCAR8ip2 was derived from 2 consecutive intraperitoneal injections in nude mice after first injection and tumor formation, while SKOV3ip1 was derived from 1 consecutive intraperitoneal injection in nude mice after first injection and tumor formation. The immortalized non-transformed human ovarian surface epithelial cell line HIO-180 was a kind gift from Dr. Andrew Godwin at the Fox Chase Cancer Center (Philadelphia, PA).
Authentication	All ovarian cancer cell lines were obtained from the American Type Culture Collection and the MD Anderson Characterized Cell Line Core Facility, which supplies authenticated cell lines via Short Tandem Repeat (STR) DNA profiling
Mycoplasma contamination	cell lines tested negative for mycoplasma
Commonly misidentified lines (See ICLAC register)	*Name any commonly misidentified cell lines used in the study and provide a rationale for their use.*

Clinical data

Policy information about clinical studies

All manuscripts should comply with the ICMJE guidelines for publication of clinical research and a completed CONSORT checklist must be included with all submissions.

Clinical trial registration	*Provide the trial registration number from ClinicalTrials.gov or an equivalent agency.*
Study protocol	Samples were collected under the study protocol # PA11-1015_CR003
Data collection	Data were collected at the university of Texas MD Anderson Cancer Center, dept of Gynecologic Oncology and Reproductive Medicine, at the Gynecologic Cancer Translational Research Center of Excellence (GYN-COE) and at Washington University, St. Louis, as part of a collaborative study with the University of Iowa and MD Anderson Cancer Center
Outcomes	Outcome measures were defined based on RECIST criteria defining response to 3-4 cycles of NACT
